# *MOXD2*, a Gene Possibly Associated with Olfaction, Is Frequently Inactivated in Birds

**DOI:** 10.1371/journal.pone.0152431

**Published:** 2016-04-13

**Authors:** Chul Jun Goh, Dongjin Choi, Dong-Bin Park, Hyein Kim, Yoonsoo Hahn

**Affiliations:** Department of Life Science, Research Center for Biomolecules and Biosystems, Chung-Ang University, Seoul, Republic of Korea; CSIRO, AUSTRALIA

## Abstract

Vertebrate *MOXD2* encodes a monooxygenase DBH-like 2 protein that could be involved in neurotransmitter metabolism, potentially during olfactory transduction. Loss of *MOXD2* in apes and whales has been proposed to be associated with evolution of olfaction in these clades. We analyzed 57 bird genomes to identify *MOXD2* sequences and found frequent loss of *MOXD2* in 38 birds. Among the 57 birds, 19 species appeared to have an intact *MOXD2* that encoded a full-length protein; 32 birds had a gene with open reading frame-disrupting point mutations and/or exon deletions; and the remaining 6 species did not show any *MOXD2* sequence, suggesting a whole-gene deletion. Notably, among 10 passerine birds examined, 9 species shared a common genomic deletion that spanned several exons, implying the gene loss occurred in a common ancestor of these birds. However, 2 closely related penguin species, each of which had an inactive *MOXD2*, did not share any mutation, suggesting an independent loss after their divergence. Distribution of the 38 birds without an intact *MOXD2* in the bird phylogenetic tree clearly indicates that *MOXD2* loss is widespread and independent in bird lineages. We propose that widespread *MOXD2* loss in some bird lineages may be implicated in the evolution of olfactory perception in these birds.

## Introduction

*MOXD2* encodes a monooxygenase dopamine β-hydroxylase (DBH)-like 2 protein, and highly orthologous proteins are found in vertebrates [1, 2]. MOXD2 and its paralogs, MOXD1 and DBH, are members of a copper type II, ascorbate-dependent monooxygenase family, which was formed by sequential duplication during bilaterian evolution [1]. DBH is involved in the conversion of dopamine to norepinephrine (noradrenaline) in postganglionic sympathetic neurons, and its malfunction is implicated in a wide range of neuropsychiatric disorders [3–5]. It is likely that vertebrate MOXD2 is also involved in neurotransmitter metabolism, potentially during olfactory transduction, because mouse ortholog *Moxd2* is highly expressed in the medial olfactory epithelium [6].

Human *MOXD2* has a genomic deletion that spans 2 exons, which occurred after humans and chimpanzees diverged [1]. Orangutan *MOXD2* has multiple nonsense mutations, and the gene has been completely deleted in gibbons [2]. Primates, especially Old World monkeys and apes, have enhanced visual perception, and they are less dependent on olfactory perception, which might have resulted in diminished olfaction and inactivation of olfaction-related genes [7, 8].

Interestingly, *MOXD2* inactivation has also occurred in whales, as evidenced by many disruptive mutations such as small frameshift insertions/deletions, nonsense mutations, and whole-gene deletions [2]. The aquatic lifestyle of whales with vocal communication and sophisticate echolocation may have reduced the dependence on olfaction and led to the reduction in olfactory apparatus and inactivation of olfaction-related genes [9–12]. Therefore, convergent inactivation of *MOXD2* in apes and whales could be an outstanding molecular signature of adaptive evolution for ecological and/or behavioral adaptation.

In this study, we examined 57 bird genomes and found widespread and independent loss of *MOXD2* in 38 birds. Loss of functional *MOXD2* may be associated with the evolution of olfaction in birds.

## Materials and Methods

### Identification of bird *MOXD2* sequences

Bird *MOXD2* sequences were identified by BLASTN searches (http://blast.ncbi.nlm.nih.gov/Blast.cgi) of the database for whole genome shotgun (WGS) contigs in the National Center for Biotechnology Information (NCBI). Initially, the chimpanzee *MOXD2* cDNA sequence was used as a query to identify bird *MOXD2* genomic sequences. Rifleman *MOXD2* was chosen as the reference sequence for subsequent bird gene analyses simply because it was the first gene identified to have an intact coding sequence in this study. Pairwise sequence comparison was performed using FASTA (version 36.3.6f) (http://fasta.bioch.virginia.edu/fasta_www2/fasta_down.shtml) [13]. Exonic sequences that matched the corresponding rifleman *MOXD2* exons from each bird genome were extracted and concatenated to generate virtual cDNA sequences. When a genomic contig contained only a partial region of an exon, raw WGS data, if available, were detected and retrieved from the NCBI Sequence Read Archive (http://www.ncbi.nlm.nih.gov/sra). CAP3 (version date 12/21/07) was used to align and assemble WGS data (http://seq.cs.iastate.edu) [14]. The resulting cDNA sequences were virtually translated into protein sequences. In October 2014, 57 bird genomes were available for analysis.

### Comparative sequence analyses

Multiple sequence alignments of exon, cDNA, or protein sequences were performed using MUSCLE (v3.8.31) (http://www.drive5.com/muscle) [15]. Presence of a signal peptide at the N-terminal end of proteins was predicted using the SignalP 4.1 web server (http://www.cbs.dtu.dk/services/SignalP) [16]. Presence of a glycosylphosphatidylinositol (GPI) anchor at the C-terminal end of proteins was predicted using the PredGPI web server (http://gpcr.biocomp.unibo.it/predgpi) [17].

Dotplots were created to identify and visually inspect exon deletions. *MOXD2* genomic sequences of the rifleman and other birds were aligned using blastz (version 2003-05-14) (http://www.bx.psu.edu/miller_lab) with default options [18]. The blastz outputs were parsed using an ad hoc perl script to extract matched coordinates that were plotted using gnuplot (version 4.6 patchlevel 4) (http://www.gnuplot.info).

## Results

### Identification of *MOXD2* from 57 bird genomes

**Fig 1:**
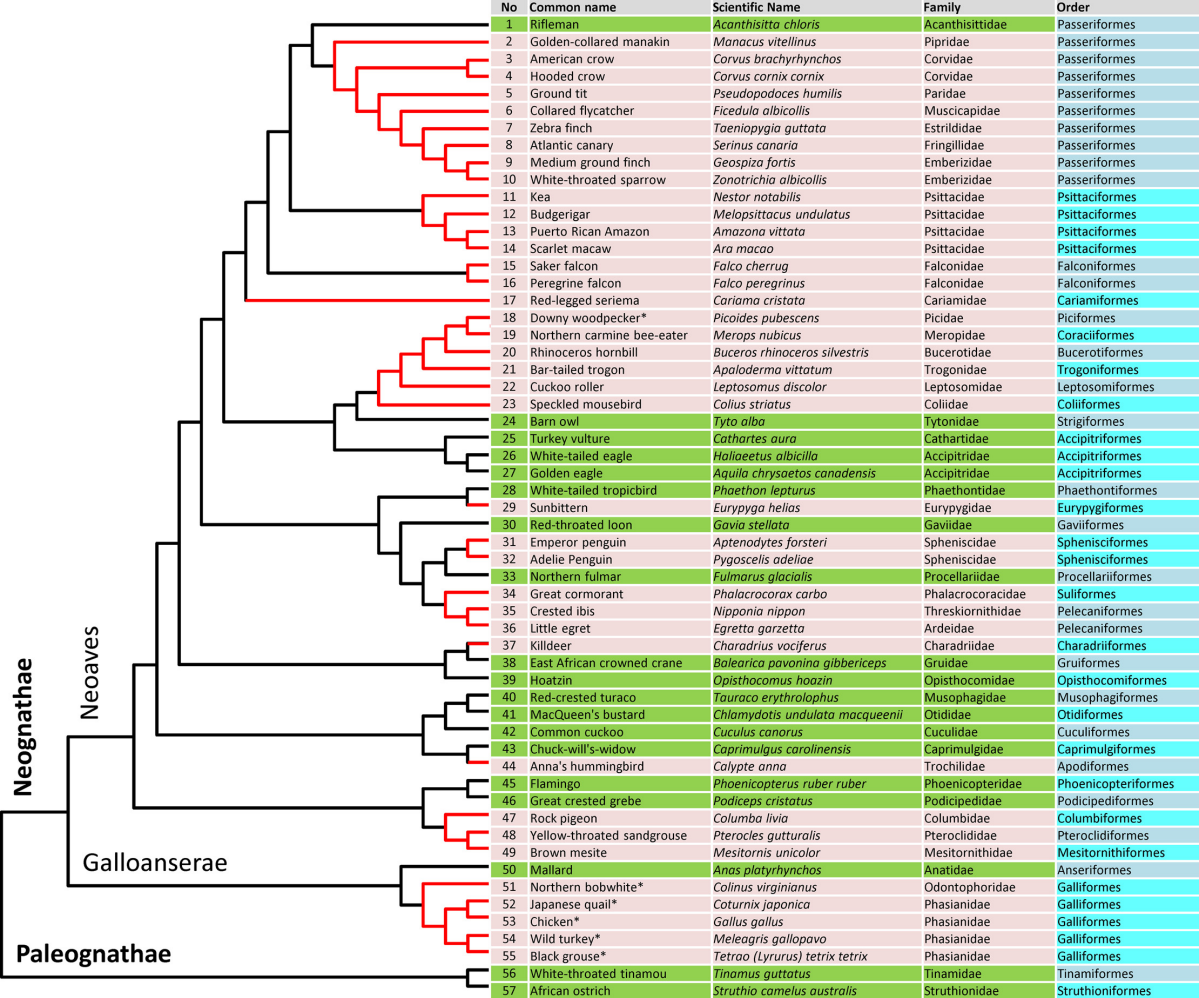
Phylogenetic tree for the birds. A phylogenetic tree for the 57 birds analyzed in this study is presented. Species with intact *MOXD2* are highlighted using a green background. Other species with *MOXD2* with disruptive mutations are highlighted using a reddish background, and their branches are in red. Asterisks (*) indicate species that probably underwent complete-gene deletion. The orders of the birds are alternately colored (the last column). Major bird clades are mentioned above the corresponding branches. See S1 Fig for detailed sequence information on bird *MOXD2* genes.

We analyzed 57 bird genomes to identify *MOXD2*. The list and phylogenetic tree for the 57 birds examined in this study are shown in Fig 1. The phylogenetic tree is based on recently published genome data [19]. Among the 57 bird species, 19 appeared to have intact *MOXD2* that encoded a full-length protein; 32 had a gene with deleterious mutations and/or exon deletions (21, both point mutations and exon deletions; 10, only point mutations; and 1, only an exon deletion); and 6 species did not yield any *MOXD2* sequence, suggesting a complete gene deletion. Mutations identified in bird *MOXD2* genes are listed in Table 1. Detailed information on bird *MOXD2* genes, including accession numbers of genomic sequences, coordinates of exons, and cDNA and protein sequences (if available), is provided in S1 Fig.

**Table 1:** Summary of mutations in *MOXD2* in birds.

No^a^	Species	e1^b^	e2	e3	e4	e5	e6	e7	e8	e9	e10	e11	e12	e13
2	Golden-collared manakin	d17, sd	ed	ed	ed	ed	ed	ed	ed	ed	ed	ed	ed	
3	American crow	d1, ns	tl, d1, d2	5d69	ed	ed	ed	ed	ed	ed	ed	ed	tl, 5d27, d10, sd	5d54, d1
4	Hooded crow	d1, ns	tl, d1, d2	5d69	ed	ed	ed	ed	ed	ed	ed	ed	tl, 5d27, d10, sd	5d54, d1
5	Ground tit	d1, d2	tl, 5d25, ns, d2	5d69	ed	ed	ed	ed	ed	ed	ed	ed	ed	5d55
6	Collared flycatcher	ns, d1	tl, ns, ns	5d64, ns	ed	ed	ed	ed	ed	ed	ed	ed	ed	5d117
7	Zebra finch	d1, i1, ns	tl, ns, d1	5d64	ed	ed	ed	ed	ed	ed	ed	ed	ed	5d32, 3d20
8	Atlantic canary	d1, ns	tl, d1, d1	5d64, ns	ed	ed	ed	ed	ed	ed	ed	ed	ed	5d105, 3d35
9	Medium ground finch	d1, ns	tl, ns, d1	5d64, ns	ed	ed	ed	ed	ed	ed	ed	ed	ed	5d105
10	White-throated sparrow	d11, d1, ns	tl, ns, d1	5d64, d2	ed	ed	ed	ed	ed	ed	ed	ed	ed	5d105
11	Kea	d5, d5, d1	ed	5d37, d8	sa, i4, i1	ed	ed	ed		ed	ed	ed	ed	ed
12	Budgerigar	d5	ed	sa, i1	sa, d7, i1	ed	ed	ed	sd	ed	ed	ed	ed	ed
13	Puerto Rican Amazon	d5, ns, ns, d14	ed	5d19	sa, d7, i1	ed	ed	ed		ed	ed	ed	ed	ed
14	Scarlet macaw	d5	ed	sa, i1	sa, d7, i1	ed	ed	ed	ns	ed	ed	ed	ed	ed
15	Saker falcon	d5, ns, sd	sa	sd	d1			ns, d1			ns	sa		
16	Peregrine falcon	d5, ns, sd	sa	sd	d1			ns, d1		ns	sa			
17	Red-legged seriema					ns	ed	ed			sa, sd			
18	Downy woodpecker	gd	gd	gd	gd	gd	gd	gd	gd	gd	gd	gd	gd	gd
19	Northern carmine bee-eater	d181	ed		ed	ed	ed	ed	ed	d7		ns, sd		sa, ns
20	Rhinoceros hornbill							d1						
21	Bar-tailed trogon		sa, sd	5d58, ns, d2		i7	5d4, ns, ns	d10			sa			i1, ns, 3d21
22	Cuckoo roller		sa, ns		d1	sd	sa, ns		d2, sd		sa	d2		d76
23	Speckled mousebird	5d12, d1, sd	sd	d8	sa, ns, d2	sa, sd		sa, ns, ns		ed	ed	ns, ns	sa, sd	
29	Sunbittern	ed	ed	ns, d7	i1	sa, i1	sa, ns, ns		ed	ed	ed	ed	ed	ed
31	Emperor penguin			sa					i7		sd	ed	ed	
32	Adelie Penguin	ns							i2					
34	Great cormorant	d5, d1, ns	d1		5d7, d7	ns	ns	ns		sd	ns, sd		ns, 3d4	i1, d11
35	Crested ibis	d5	ns, d1	i1										d4
36	Little egret	d5, ns	ns	sa, ns, sd	sa			ns		d7		i13, d1, ns, sd	ns	d7, d1
37	Killdeer							ed						
44	Anna's hummingbird		ed	ed				d8					3d59	ed
47	Rock pigeon	ns	d1, sd	sd	sd				d20		sa			
48	Yellow-throated sandgrouse	d1, ns		ed		i1			ed	ed	ed			d2
49	Brown mesite	d5	ed	ed	ns		d1, d8	ns	ed	ns	sa	sd		ns
51	Northern bobwhite	gd	gd	gd	gd	gd	gd	gd	gd	gd	gd	gd	gd	gd
52	Japanese quail	gd	gd	gd	gd	gd	gd	gd	gd	gd	gd	gd	gd	gd
53	Chicken	gd	gd	gd	gd	gd	gd	gd	gd	gd	gd	gd	gd	gd
54	Wild turkey	gd	gd	gd	gd	gd	gd	gd	gd	gd	gd	gd	gd	gd
55	Black grouse	gd	gd	gd	gd	gd	gd	gd	gd	gd	gd	gd	gd	gd

^a^ Species number used in this study. Species without mutations include: 1, rifleman; 24, barn owl; 25, turkey vulture; 26, white-tailed eagle; 27, golden eagle; 28, white-tailed tropicbird; 30, red-throated loon; 33, Northern fulmar; 38, East African crowned crane; 39, hoatzin; 40, red-crested turaco; 41, MacQueen’s bustard; 42, common cuckoo; 43, Chuck-will’s-widow; 45, flamingo; 46, great crested grebe; 50, mallard; 56, white-throated tinamou; and 57, African ostrich.

^b^ Blank indicates no mutation in the given exon; sa, splice acceptor mutation; 5d#, #-nt deletion at the 5′-end; d#, internal deletion of # nt; i#, insertion of # nt; ns, nonsense codon; 3d#, #-nt deletion at the 3′-end; sd, splice donor mutation; ed, exon deletion; tl, translocation; gd, gene deletion.

*MOXD2* genes that encoded a full-length protein were identified in 19 bird genomes: rifleman, barn owl, turkey vulture, white-tailed eagle, golden eagle, white-tailed tropicbird, red-throated loon, northern fulmar, East African crowned crane, hoatzin, red-crested turaco, MacQueen’s bustard, common cuckoo, Chuck-will’s-widow, flamingo, great crested grebe, mallard, white-throated tinamou, and African ostrich. Multiple sequence alignment of full-length MOXD2 proteins showed sequence conservation (S2 Fig). These bird MOXD2 proteins were predicted to have a signal peptide and a GPI anchor signal as other previously reported MOXD2 proteins do, suggesting that they are functional [1, 2]. Rifleman (order Passeriformes; species No. 1) *MOXD2* was used as the reference sequence in subsequent analyses.

### ORF-disrupting point mutations in *MOXD2* in 31 bird genomes

Among the 57 bird species, 31 species were identified to have *MOXD2* with ORF-disrupting point mutations (Table 1). These species include golden-collared manakin, American crow, hooded crow, ground tit, collared flycatcher, zebra finch, Atlantic canary, medium ground finch, white-throated sparrow, kea, budgerigar, Puerto Rican Amazon, scarlet macaw, saker falcon, peregrine falcon, red-legged seriema, northern carmine bee-eater, rhinoceros hornbill, bar-tailed trogon, cuckoo roller, speckled mousebird, sunbittern, emperor penguin, Adelie penguin, great cormorant, crested ibis, little egret, killdeer, Anna’s hummingbird, rock pigeon, yellow-throated sandgrouse, and brown mesite.

**Fig 2:**
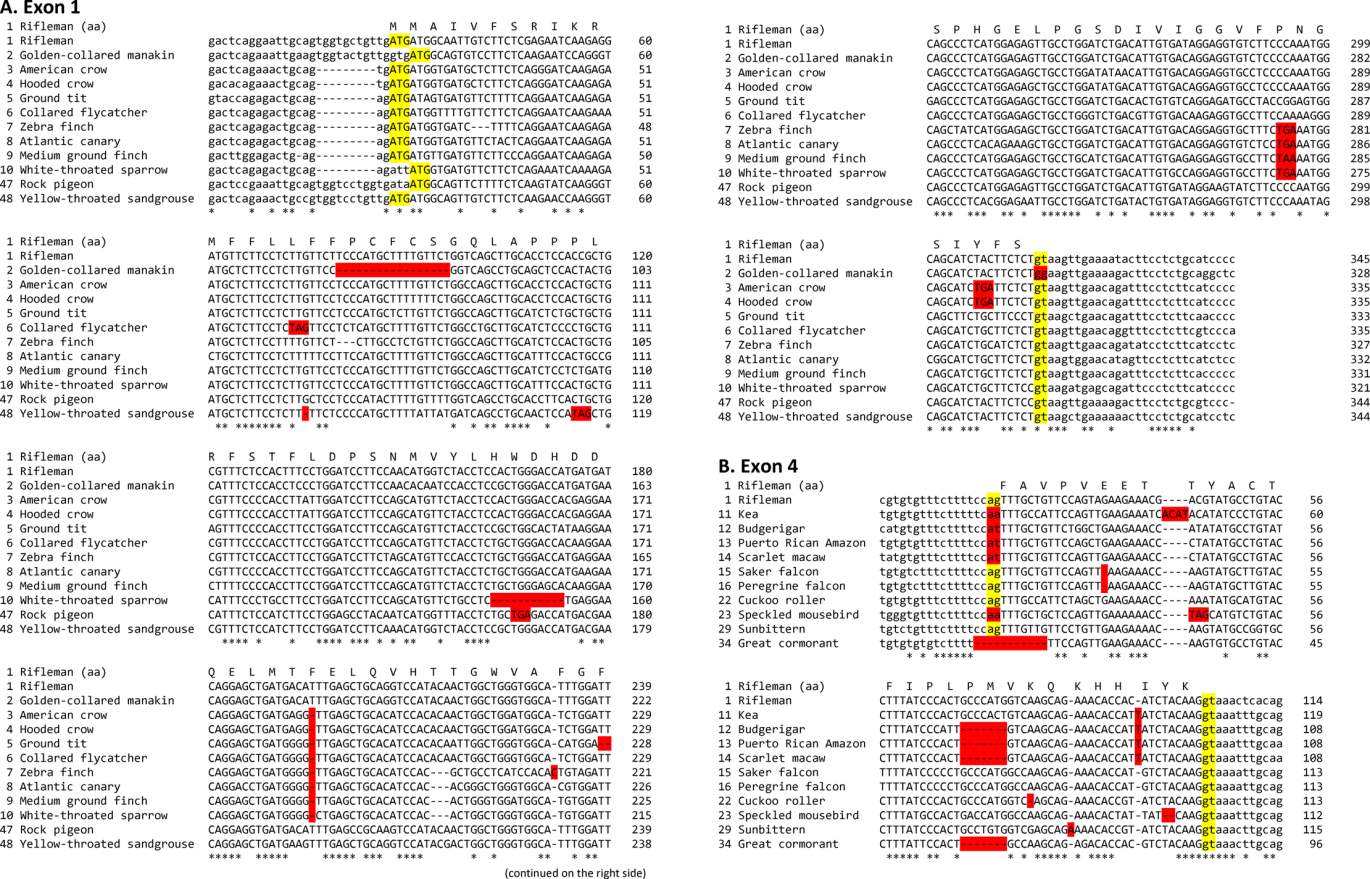
Disruptive mutations in bird *MOXD2*. ORF-disrupting mutations in exon 1 (A) and exon 4 (B) of representative birds are presented. Deleterious mutations, including nonsense codons, insertions, deletions, and splice-site mutations, are highlighted using a red background. Start codons and splice donor and acceptor sequences are highlighted using a yellow background. Amino acid sequences of the rifleman are shown above the DNA sequence alignments. Exonic and intronic sequences are in uppercase and lowercase letters, respectively. See S3 Fig for more disruptive mutations in other exons.

The ORF-disrupting point mutations included splice site mutations, frameshifting small insertions/deletions, and nonsense mutations. Two representative exons (exons 1 and 4) of selected species with such point mutations are shown in Fig 2. Other selected exons with point mutations are presented in S3 Fig. These point mutations are not attributable to sequencing errors; alignments and assemblies of WGS sequences derived from these exons confirmed that the exon sequences were assembled from a large amount of raw sequence data and therefore the mutations were genuine. Partial genomic assemblies that span the selected exons in S3 Fig are presented in S4 Fig.

As a representative case, the exon 1 sequences of 12 species (11 selected species with point mutations and the rifleman) are shown in Fig 2A. The rifleman (order Passeriformes) *MOXD2* gene, which may encode an intact full-length protein, was used as the reference sequence. Exon 1 of golden-collared manakin (order Passeriformes; species No. 2) *MOXD2* showed a 17-nt deletion and a splice donor mutation (gt to gg). The other 8 passerine birds (American crow, hooded crow, ground tit, collared flycatcher, zebra finch, Atlantic canary, medium ground finch, and white-throated sparrow; species Nos. 3 to 10), rock pigeon, and yellow-throated sandgrouse had diverse ORF-disrupting point mutations, including small insertions/deletions, nonsense mutations, and a splice site mutation. Some mutations were shared by closely related species, for example, a 1-nt deletion was common in 8 passerine birds (see species Nos. 3 to 10 in Fig 2A), indicating this mutation occurred in a common ancestor of these birds.

As another representative case, exon 4 sequences of 11 species (10 selected species with point mutations and the rifleman) are shown in Fig 2B. These include 4 parrots (order Psittaciformes; kea, budgerigar, Puerto Rican Amazon, and scarlet macaw), 2 falcons (order Falconiformes; saker falcon and peregrine falcon), cuckoo roller, speckled mousebird, sunbittern, and great cormorant. As in exon 1, a variety of point mutations, including splice site mutations, small insertions/deletions, and nonsense mutations, were observed. Some mutations were shared by closely related species, for example, a 1-nt insertion was common in parrots (species Nos. 11 to 14 in Fig 2B) and a 1-nt deletion was common in falcons (Nos. 15 and 16).

### Exon deletions and translocations in *MOXD2* in 22 bird genomes

Among the 32 birds with a disrupted *MOXD2* gene, 22 were identified to have an exon deletion. When an exon was not present in any genomic contig and its 5′- and 3′-flanking regions were found in a single genomic contig, the missing exon was regarded to be deleted in the bird genome. For example, exons 1 to 10 and 13 of the emperor penguin *MOXD2* gene were found in the contig “JMFQ01072246.1” and exons 11 and 12 were not present in this contig or any other contigs, suggesting that a genomic deletion that spanned exons 11 and 12 occurred in this species (S1 Fig, species No. 31). A genomic deletion that removed at least 1 exon was observed in 22 birds (marked as “ed” in Table 1): 21 of them also had at least 1 point mutation in other exons; the killdeer (No. 37) was the only species that had an exon deletion with no point mutation in other exons.

Some exons were identified to be translocated: an exon was considered to be translocated when its 5′- and the 3′-flanking regions were present in a genomic contig with other exons and the given exon itself was found in a different genomic contig. Exon translocation events were identified in *MOXD2* in 8 bird genomes (marked as “tl” in Table 1). For example, exons 1, 3, and 13 of the American crow *MOXD2* gene were present in the contig “JMFN01085921.1,” while exons 2 and 12 were found in different contigs, “JMFN01029801.1” and “JMFN01085927.1,” respectively (S1 Fig, species No. 3).

**Fig 3:**
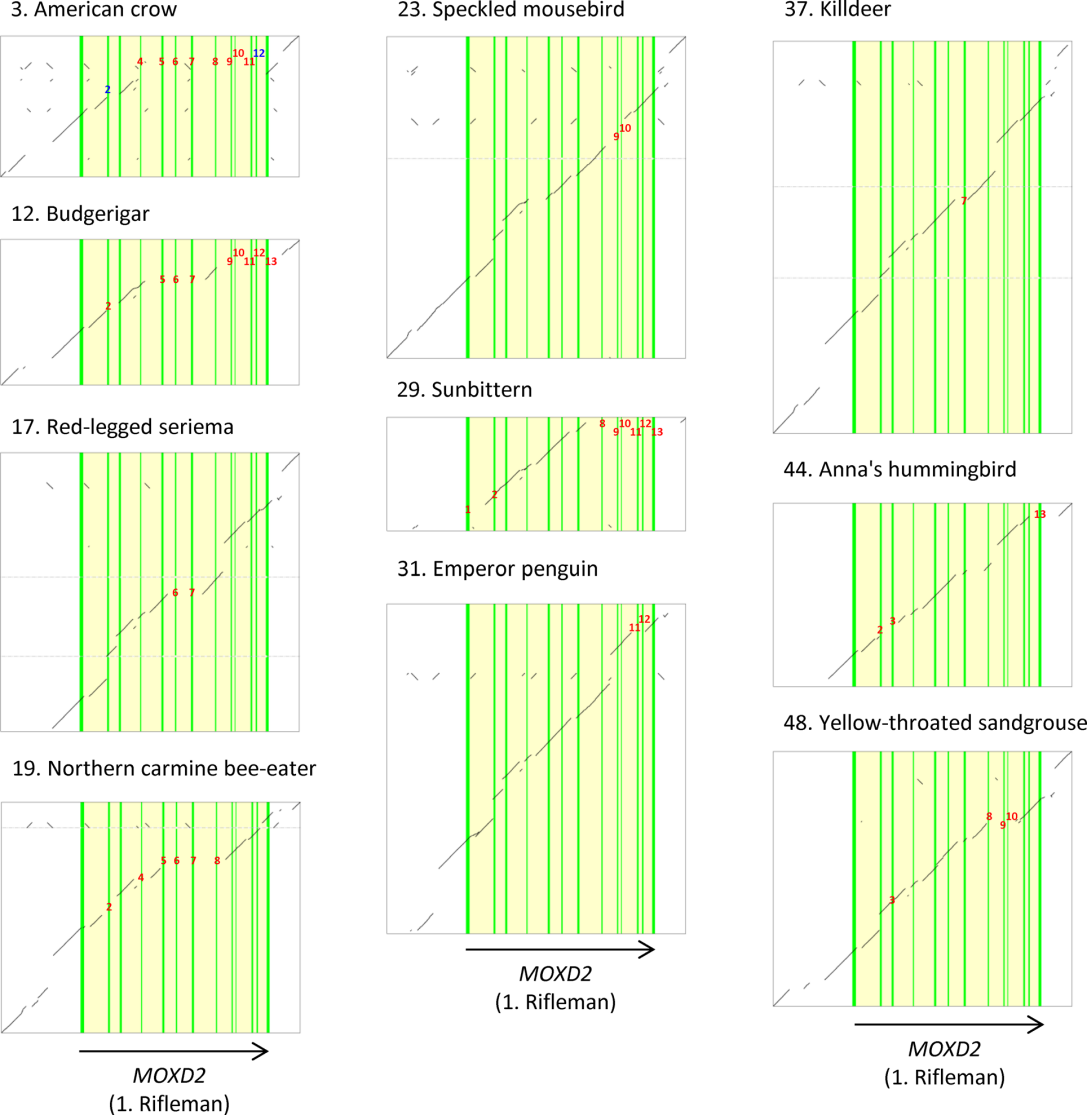
Examples of exon deletions and translocations in bird *MOXD2*. Dotplots between *MOXD2* genomic sequences of selected birds (vertical) and that of the rifleman *MOXD2* gene (horizontal) are shown. Exonic and intronic segments of rifleman *MOXD2* are marked in green and yellow, respectively. Diagonal lines indicate an aligned segment and hence the presence of corresponding genomic segments. Note that some exons are missing in these birds, as evidenced by the lack of a segment aligned with the rifleman *MOXD2* exons. Red and blue numbers indicate deleted and translocated exons, respectively. See S5 Fig for dotplots of *MOXD2* in other birds.

Dotplots between the rifleman *MOXD2* genomic sequence and those of each of the 57 birds were produced to confirm and visualize genomic rearrangements that resulted in exon deletions and/or translocations (S5 Fig). Representative dotplots of 10 birds are shown in Fig 3. For example, American crow *MOXD2* exhibited a genomic deletion that spanned exons 4 to 11 and two translocations involving exons 2 and 12 (Fig 3, species No. 3). The deletion spanning exons 4 to 11 was common in the 9 passerine birds (species Nos. 2 to 10), suggesting that this deletion occurred in a common ancestor of these birds after the rifleman diverged. The translocation event involving exon 2 was shared with the other 7 passerine birds (species Nos. 4 to 10). Exon 12 translocation was also found in the hooded crow (species No. 4), the closest relative of the American crow.

### Possible complete deletion of *MOXD2* in 6 bird genomes

In 6 bird genomes, no *MOXD2* sequence was detected, raising the possibility of whole-gene deletion (marked as “gd” in Table 1 and with an asterisk in Fig 1). These birds include downy woodpecker, northern bobwhite, Japanese quail, chicken, wild turkey, and black grouse.

The downy woodpecker (species No. 18), which belongs to the order Piciformes, did not show any *MOXD2* sequence. It is possible that the lack of the *MOXD2* sequence is because of incomplete coverage of the WGS data. However, a sequence similarity search of the downy woodpecker WGS sequences using the *MOXD2* genomic contigs of Northern carmine bee-eater, which is the closest species of downy woodpecker in our dataset, as queries, yielded 5 WGS contigs. Dotplot comparisons of downy woodpecker WGS contigs with Northern carmine bee-eater (S6 Fig) or rifleman genomic sequences (S5 Fig, species No. 18) suggested that the whole *MOXD2* genomic segment is missing in the downy woodpecker. It is also noteworthy that an almost complete sequence of downy woodpecker *MOXD1*, a paralog of *MOXD2*, can be recovered from current genomic sequence data. Therefore, it is likely that the *MOXD2* deletion is genuine.

All the other 5 species belong to the order Galliformes, suggesting that the *MOXD2* deletion may be the ancestral state. It is also possible that the genomic segment containing the *MOXD2* fragment needs to be sequenced. However, the gene is absent even in the chicken, the genome of which has been extensively studied. All these 5 Galliformes bird genomes yielded complete or partial sequences of *MOXD1*, a paralog of *MOXD2*, suggesting that the lack of *MOXD2* sequences in these genomes may not be because of incomplete sequencing. A region from the chicken chromosome 1 was identified to be orthologous to the mallard genomic contig NW_004676532.1. A dotplot comparison of a 6,000,000-bp-long segment from the chicken chromosome 1 and the 1,916,416-bp-long mallard genomic contig confirmed the complete deletion of *MOXD2* gene in the chicken (S7 Fig). Interestingly, the deleted segment was in an inversion boundary, suggesting that *MOXD2* gene deletion might have been accompanied by a genomic rearrangement. Therefore, it is highly probable that the *MOXD2* deletion is genuine in these 5 Galliformes birds, and it might have occurred in a common ancestor of these birds.

## Discussion

Analysis of 57 bird genomes revealed that *MOXD2* has been inactivated in 38 birds, as evidenced by ORF-disrupting point mutations, genomic rearrangements that cause exon deletions and/or translocations, or whole-gene deletions. Although in some cases ORF-disrupting mutations might lead to functional modifications which may be neutral or even result in evolution of advantageous phenotypes [20, 21], it is not likely that mutant bird *MOXD2* genes produce functional proteins because they have multiple and/or highly disruptive mutations. As shown in Fig 1, 19 birdsn with an intact *MOXD2* gene and 38 birds with a disrupted gene were distributed throughout the bird phylogenetic tree, indicating that *MOXD2* inactivation is widespread and independent in bird lineages.

In some lineages, mutations were shared by closely related species, implying that the gene-disrupting mutation occurred in a common ancestor of those birds. For example, a genomic deletion that spanned exons 4 to 11 was commonly found in 9 passerine birds (see Table 1, species Nos. 2 to 10), suggesting that the deletion occurred in a common ancestor of these birds after the rifleman diverged. The 4 parrots (see Table 1, Nos. 11 to 14) shared many mutations in *MOXD2*, including a 5-nt deletion in exon 1, a 1-nt insertion in exon 4 (see Fig 2B), and 3 genomic deletions that spanned exons 2, 5 to 7, and 9 to 13, respectively.

The 6 birds, downy woodpecker, northern carmine bee-eater, rhinoceros hornbill, bar-tailed trogon, cuckoo roller, and speckled mousebird, in which *MOXD2* was identified to be inactive, form a single clade, although they belong to different orders (Fig 1, species Nos. 18 to 23). Mutations in *MOXD2* in these birds were not common, suggesting that the gene inactivation was of independent origin. The rhinoceros hornbill had only 1 mutation, a 1-nt deletion in exon 7 (S3E Fig), implying that the gene inactivation might have occurred quite recently in this bird. Even in the 2 closely related penguins, the emperor penguin and Adelie penguin, there was no common mutation (Table 1, species Nos. 31 and 32). This suggests that the gene might have become inactivated independently in each penguin lineage. Another possibility is that the gene might become of less or no use in an ancestral penguin species and accumulated different mutations after they diverged around 23 million years ago [22].

Widespread and independent inactivation of *MOXD2* in bird lineages implies that this gene might have become generally dispensable during bird evolution. The phenotype associated with the inactivation of *MOXD2* in birds has not been identified. *MOXD2* was suggested to be involved in olfactory perception in mammals based on its strong expression in the mouse olfactory epithelium, although its molecular function has not yet been determined [6]. Inactivation of *MOXD2* was proposed to be associated with diminished olfaction in apes and whales [1, 2]. As in birds, *MOXD2* inactivation in apes and whales seemed to have occurred independently in lineages of each clade [1, 2]. The human *MOXD2* gene has a genomic deletion that spanned exons 12 and 13, while chimpanzees, bonobos, and gorillas have a gene with intact ORF. Orangutans have a couple of nonsense mutations, while gibbons lost the whole gene by a genomic deletion. Both toothed and baleen whales have *MOXD2* with disruptive mutations. However, no common mutation was found between these 2 whale clades [2].

Similar inactivation patterns for sensory perception genes occurred along with ecological habitat shift and/or changes in feeding or communication behaviors. For examples, a large portion of olfactory receptor (OR) genes are inactive in catarrhine primates, possibly because of reduced reliance on olfaction [7, 11]. On the basis of the same reason, *TRPC2*, which encodes the transient receptor potential cation channel, subfamily C, member 2 protein, a crucial component of pheromone transduction, is inactive in catarrhine primates and whales, and it is frequently inactivated in bats and other aquatic mammals [23–26]. *Tas1r2*, which encodes a component of the sweet receptor, is inactive in many carnivorous mammals such as cats, spotted hyenas, and seals [27]. It is probable that the sweet receptor became dispensable in these exclusive meat-eaters and accumulated disruptive mutations under absence of selection.

Interestingly, *Tas1r2* was found to be deleted in 16 bird genomes [28]. In addition, penguins do not have genes for the umami and bitter taste receptors, probably because they swallow food whole and have no dependence on the taste perception, which might have allowed the loss of these taste receptor genes. Bird genome analysis also revealed that two diet-related genes, *AGT* and *GULO*, that encode alanine/glyoxylate aminotransferase and l-gulonolactone oxidase, respectively, had been inactivated independently in some bird lineages: *AGT* is inactive in the cuckoo roller, American crow, zebra finch, medium ground-finch, and Anna’s hummingbird, while *GULO* is a pseudogene in the golden-collared manakin, zebra finch, and medium ground finch [29]. *MOXD2* seems to be another example of a gene that was independently inactivated during bird evolution.

Recent studies have shown that the olfactory bulb (OB) size and OR gene repertoires in birds are correlated with their ecological adaptations and behavioral characteristics [30, 31]. For example, semi-aquatic birds have relatively larger OBs than terrestrial birds, suggesting that the former rely on olfaction more than the latter. Interestingly, the mallard, an Anseriformes, which inhabits a semi-aquatic environment, has an intact *MOXD2* gene, while its close relatives, Galliformes including chicken and turkey that are terrestrial, lost the gene by complete gene deletion. Songbirds (Passeriformes), or vocal-learning species, which more rely on cognitive ability than olfaction, have the smallest OBs and least number of OR genes [30, 31]. As expected, the *MOXD2* gene is inactive in all passerine birds. This observation strengthens our notion that loss of *MOXD2* gene is associated with evolution of olfactory function in birds although detailed further study is required for a conclusive answer.

In summary, 57 bird genomes were analyzed and widespread and independent losses of *MOXD2* were found in 38 birds. Frequent *MOXD2* inactivation in some birds may be associated with the evolution of olfaction in these birds depending on their ecological and/or behavioral adaptations.

## Supporting Information

S1 Fig Summary of bird *MOXD2* genes. (PDF)

S2 Fig Alignment of full-length bird MOXD2 protein sequences. (PDF)

S3 Fig Alignment of selected exons with point mutations. (PDF)

S4 Fig WGS sequence assemblies of selected exons. (PDF)

S5 Fig Dotplot analysis of *MOXD2* genomic sequences. (PDF)

S6 Fig Dotplot comparison of the downy woodpecker and Northern carmine bee-eater *MOXD2* loci. (PDF)

S7 Fig Dotplot comparison of the chicken and mallard *MOXD2* loci. (PDF)
