# Supplementary material for: MOXD2, a Gene Possibly Associated with Olfaction, Is Frequently Inactivated in Birds
Source: PLoS One. 2016 Apr 13;11(4):e0152431. doi: 10.1371/journal.pone.0152431 (PMC4830563; doi:10.1371/journal.pone.0152431)
Supplement: S1 Fig — (PDF) [file pone.0152431.s001.pdf]

S1 Fig. Summary of bird *MOXD2* genes

1. Rifleman (*Acanthisitta chloris*)

```
>Acanthisitta_chloris No=1 length=1836 name="Rifleman"
ATGATGGCAATTGTCTTCTCGAGAATCAAGAGGATGTTCTTCCTCTTGTTCTTCCCATGCTTTTGTTCTG
GTCAGCTTGCACCTCCACCGCTGCGTTTCTCCACTTTCCTGGATCCTTCCAACATGGTCTACCTCCACTG
GGACCATGATGATCAGGAGCTGATGACATTTGAGCTGCAGGTCCATACAACTGGCTGGGTGGCATTTGGA
TTCAGCCCTCATGGAGAGTTGCCTGGATCTGACATTGTGATAGGAGGTGTCTTCCCAAATGGCAGCATCT
ACTTCTCTGATTGTCACATGGAAGGTGAGGCAACCCTTGAGGAAGATGAGAGCCAGGACTACCAACTGCT
GTCAGTGACAGAGAACAAAACCTCCACCACCATGTCGTTCAAACGTCACCTCCGGACGTGTGACACAAAT
GACCTGGATATCACAATGGACACAGCCCATCTTGTTGCTGCATTTGGCACTGATGACACAGTCCAATTCT
TTAAAAGCCAAAGATTTTCCAGATCTCTTTTCCTGATGAGGTACAAAAGCCCATCTGACTCAACTGACCC
CAAAGTATTCTTCACCTATGACCTGAGGCTGGACAACTTTGCTGTTCCAGTAGAAGAAACGACGTATGCC
TGTACCTTTATCCCACTGCCCATGGTCAAGCAGAAACACCACATCTACAAGTTTGAACCTGTAATAACAT
CCCACAATATAACCTTGGTTCATCATATTCTTGTTTATGCCTGTGGCAACTCCAGCATCCTACCCAGTGG
CATAGGTGATTGCTATGGAGCCAATCCAGATTTTTCCCTGTGCTCTCAGGTCCTTGTGGGCTGGGCTGTT
GGAGGAGAGTCCTATCAATTTCCAGATGAAGCTGCAGTTTCCATAGGGACACCTTGGGACCCTCAGTACA
TACGACTGGAAATCCATTACAGCAATCTTGACTTGTTACCAGGCTTGATTGACAGCTCAGGGATACGAAT
CTACTACACACCAGAGCTACGGAAATATGATGTGGGGGTTCTGCAAACAGGTGTCTTCATTTTCCCTGCA
CATTTCATTCCTCCTGGAGCAGAATCCTACAGATCTTACGGTCTTTGCAATTCCAGCCAGTTCGACGAAA
TGAATGGGATGCTGGTTCCAGATCTCCATGTCTTTGCCTATCTGCTTCACACTCACCTGTCTGGCAGAGG
AGTGAAAGTCGTTCAATACCGGAACGGTGAGCAGCTGAGGATCATCTGTGAGGACAATAAGTATGACTTC
AGGCTGCAGGAAATTCGGGACACGAAGGAAATCCTCACAATTAAACCAGGGGATGAAATCCTGACTGAAT
GCAACTTTCAAACACTGGATCGGTCAGGGGTTACTTTTGGTGGGCTAAGCACCATGAATGAGATGTGCCT
TGCATTCCTCTTCTACTACCCTCGTAACAACATCTCCAGTTGCATGGGCTACCCAGACATTTTGTATGTT
GCACACACAGTCAAGCAGGAGGCCTCAGATGCATTGGAAGGAATGATGGCCATGAACTTTGTTGACTGGG
ATGATGATACTGTCAAAATTGCAGAGAAAGCTGCCAAGGAGGCAAACCAAGTGGTCGTGATTAAAACCAT
CAATGAGCTCCAGAGAAATGAGAGTGGTCTCATCAGAGATATCAGTATTCCAGAGCAGGCTGCCTGTCAC
AATATTTCTGGACATGTCTCCCTATCAGATCTGAGGGTCACTGCAATAGACACTTCAGAGTCATCAACCA
CCAAAGAAACTGCTTCACTTCCTCTTCTTTCTCTCACACAGCTGGTGTTTGCTTGGCTTATCTTGGCCTC
TGAGTACGAAAAGTGA
```

```
>Acanthisitta_chloris No=1 length=611 name="Rifleman"
MMAIVFSRIKRMFFLLFFPCFCSGQLAPPPLRFSTFLDPSNMVYLHWDHDDQELMTFELQVHTTGWVAFG
FSPHGELPGSDIVIGGVFPNGSIYFSDCHMEGEATLEEDESQDYQLLSVTENKTSTTMSFKRHLRTCDTN
DLDITMDTAHLVAAFGTDDTVQFFKSQRFSRSLFLMRYKSPSDSTDPKVFFTYDLRLDNFAVPVEETTYA
CTFIPLPMVKQKHHIYKFEPVITSHNITLVHHILVYACGNSSILPSGIGDCYGANPDFSLCSQVLVGWAV
GGESYQFPDEAAVSIGTPWDPQYIRLEIHYSNLDLLPGLIDSSGIRIYYTPELRKYDVGVLQTGVFIFPA
HFIPPGAESYRSYGLCNSSQFDEMNGMLVPDLHVFAYLLHTHLSGRGVKVVQYRNGEQLRIICEDNKYDF
RLQEIRDTKEILTIKPGDEILTECNFQTLDRSGVTFGGLSTMNEMCLAFLFYYPRNNISSCMGYPDILYV
AHTVKQEASDALEGMMAMNFVDWDDDTVKIAEKAAKEANQVVVIKTINELQRNESGLIRDISIPEQAACH
NISGHVSLSDLRVTAIDTSESSTTKETASLPLLSLTQLVFAWLILASEYEK
```

Exon	NCBI Accession	Strand	Start	End
1	JJRS01053877.1	-	17154	17441
2	JJRS01053877.1	-	15105	15251
3	JJRS01053877.1	-	14161	14322
4	JJRS01053877.1	-	12553	12636
5	JJRS01053877.1	-	10782	10949
6	JJRS01053877.1	-	9766	9868
7	JJRS01053877.1	-	8436	8602

8	JJRS01053877.1	-	6597	6688
9	JJRS01053877.1	-	5352	5448
10	JJRS01053877.1	-	5075	5134
11	JJRS01053877.1	-	3740	3870
12	JJRS01053877.1	-	3352	3466
13	JJRS01053877.1	-	2439	2660

---

2. Golden-collared manakin (*Manacus vitellinus*)

Exon	NCBI Accession	Strand	Start	End	
1	JMFM02043338.1	-	8540	8807	17-nt deletion, splice donor GG
2					exon deletion
3					exon deletion
4					exon deletion
5					exon deletion
6					exon deletion
7					exon deletion
8					exon deletion
9					exon deletion
10					exon deletion
11					exon deletion
12					exon deletion
13	JMFM02004428.1	+	1320	1559	

---

3. American crow (*Corvus brachyrhynchos*)

Exon	NCBI Accession	Strand	Start	End	
1	JMFN01085921.1	+	35028	35314	1-nt deletion, 1 nonsense codon
2	JMFN01029801.1	-	30613	30750	translocation, 1-nt deletion, 2-nt deletion
3	JMFN01085921.1	+	36856	36948	5' deletion (69 nt)
4					exon deletion
5					exon deletion
6					exon deletion
7					exon deletion
8					exon deletion
9					exon deletion
10					exon deletion
11					exon deletion
12	JMFN01085927.1	-	1788	1865	translocation, 5' deletion (27-nt), 10-nt deletion, splice donor GG
13	JMFN01085921.1	+	38482	38660	5' deletion (54 nt), 1-nt deletion

---

4. Hooded crow (*Corvus cornix cornix*)

Exon	NCBI Accession	Strand	Start	End	
1	JPSR01026093.1	-	67948	68234	1-nt deletion, 1 nonsense codon
2	JPSR01014826.1	+	113810	113947	translocation, 1-nt deletion, 2-nt deletion
3	JPSR01026093.1	-	66314	66406	5' deletion (69 nt)
4					exon deletion
5					exon deletion
6					exon deletion
7					exon deletion
8					exon deletion
9					exon deletion
10					exon deletion
11					exon deletion
12	JPSR01026093.1	+	42743	42820	translocation, 5' deletion (27 nt), 10-nt deletion, splice donor GG
13	JPSR01026093.1	-	64596	64774	5' deletion (54 nt), 1-nt deletion

5. Ground tit (*Pseudopodoces humilis*)

Exon	NCBI Accession	Strand	Start	End	
1	ANZD01002549.1	-	90630	90914	1-nt deletion, 2-nt deletion
2	ANZD01025988.1	+	41973	42085	translocation, 5' deletion (25 nt), 1 nonsense codon, 2-nt deletion
3	ANZD01002549.1	-	88982	89074	5' deletion (69 nt)
4					exon deletion
5					exon deletion
6					exon deletion
7					exon deletion
8					exon deletion
9					exon deletion
10					exon deletion
11					exon deletion
12					exon deletion
13	ANZD01002549.1	-	87243	87427	5' deletion (55 nt)

6. Collared flycatcher (*Ficedula albicollis*)

Exon	NCBI Accession	Strand	Start	End	
1	AGTO02001188.1	-	9186	9472	1 nonsense codon, 1-nt deletion
2	AGTO02000237.1	-	317365	317511	translocation, 2 nonsense codons
3	AGTO02001188.1	-	7512	7609	5' deletion (64 nt), 1 nonsense codon
4					exon deletion

5					exon deletion
6					exon deletion
7					exon deletion
8					exon deletion
9					exon deletion
10					exon deletion
11					exon deletion
12					exon deletion
13	AGTO02001188.1	-	5835	5939	5' deletion (117 nt)

7. Zebra finch (*Taeniopygia guttata*)

Exon	NCBI Accession	Strand	Start	End	
1	ABQF01036336.1	-	16726	17004	1-nt deletion, 1-nt insertion, 1 nonsense codon
2	ABQF01007442.1	-	13688	13833	translocation, 1 nonsense codon, 1-nt deletion
3	ABQF01036336.1	-	15058	15155	5' deletion (64 nt)
4					exon deletion
5					exon deletion
6					exon deletion
7					exon deletion
8					exon deletion
9					exon deletion
10					exon deletion
11					exon deletion
12					exon deletion
13	ABQF01036336.1	-	13416	13594	5' deletion (32 nt), 3' deletion (20 nt)

8. Atlantic canary (*Serinus canaria*)

Exon	NCBI Accession	Strand	Start	End	
1	CAVT010026339.1	-	3022	3305	1-nt deletion, 1 nonsense codon
2	CAVT010007653.1	+	13641	13785	translocation, 1-nt deletion, 1-nt deletion
3	CAVT010026339.1	-	1398	1495	5' deletion (64 nt), 1 nonsense codon
4					exon deletion
5					exon deletion
6					exon deletion
7					exon deletion
8					exon deletion
9					exon deletion
10					exon deletion
11					exon deletion

12					exon deletion
13	CAVT010026338.1	-	11275	11356	5' deletion (105 nt), 3' deletion (35 nt)

9. Medium ground finch (*Geospiza fortis*)

Exon	NCBI Accession	Strand	Start	End	
1	AKZB01056505.1	+	41098	41381	1-nt deletion, 1 nonsense codon
2	AKZB01004402.1	-	8834	8979	translocation, 1 nonsense codon, 1-nt deletion
3	AKZB01056505.1	+	42908	43005	5' deletion (64 nt), 1 nonsense codon
4					exon deletion
5					exon deletion
6					exon deletion
7					exon deletion
8					exon deletion
9					exon deletion
10					exon deletion
11					exon deletion
12					exon deletion
13	AKZB01056505.1	+	44552	44668	5' deletion (105 nt)

10. White-throated sparrow (*Zonotrichia albicollis*)

Exon	NCBI Accession	Strand	Start	End	
1	ARWJ01002597.1	+	41176	41445	11-nt deletion, 1-nt deletion, 1 nonsense codon
2	ARWJ01002366.1	-	303048	303193	translocation, 1 nonsense codon, 1-nt deletion
3	ARWJ01002597.1	+	42965	43060	5' deletion (64 nt), 2-nt deletion
4					exon deletion
5					exon deletion
6					exon deletion
7					exon deletion
8					exon deletion
9					exon deletion
10					exon deletion
11					exon deletion
12					exon deletion
13	ARWJ01002597.1	+	44680	44796	5' deletion (105 nt)

11. Kea (*Nestor notabilis*)

Exon	NCBI Accession	Strand	Start	End	
1	JJRH01053300.1	-	4293	4569	5-nt deletion, 5-nt deletion, 1-nt deletion

2					exon deletion
3	JJRH01053300.1	-	2058	2174	5' deletion (37 nt), 8-nt deletion
4	JJRH01053300.1	-	614	702	splice acceptor AA, 4-nt insertion, 1-nt insertion
5					exon deletion
6					exon deletion
7					exon deletion
8	JJRH01053299.1	-	23293	23384	
9					exon deletion
10					exon deletion
11					exon deletion
12					exon deletion
13					exon deletion

12. Budgerigar (*Melopsittacus undulatus*)

Exon	NCBI Accession	Strand	Start	End	
1	AGAI01057471.1	-	11394	11673	5-nt deletion
2					exon deletion
3	AGAI01057471.1	-	9187	9349	splice acceptor GT, 1-nt insertion
4	AGAI01057471.1	-	7849	7926	splice acceptor AT, 7-nt deletion, 1-nt insertion
5					exon deletion
6					exon deletion
7					exon deletion
8	AGAI01057471.1	-	6514	6605	splice donor AT
9					exon deletion
10					exon deletion
11					exon deletion
12					exon deletion
13					exon deletion

13. Puerto Rican Amazon (*Amazona vittata*)

Exon	NCBI Accession	Strand	Start	End	
1	AOCU01260777.1	-	1784	2049	5-nt deletion, 2 nonsense codons, 14-nt deletion
2					exon deletion
3	AOCU01289053.1	-	6271	6413	5' deletion (19 nt)
4	AOCU01289053.1	-	4927	5004	splice acceptor AT, 7-nt deletion, 1-nt insertion
5					exon deletion
6					exon deletion
7					exon deletion
8	AOCU01289053.1	-	3587	3678	

9					exon deletion
10					exon deletion
11					exon deletion
12					exon deletion
13					exon deletion

14. Scarlet macaw (*Ara macao*)

Exon	NCBI Accession	Strand	Start	End	
1	AOUJ01293014.1	-	2895	3168	5-nt deletion
2					exon deletion
3	AOUJ01073286.1	-	5260	5422	splice acceptor GT, 1-nt insertion
4	AOUJ01073286.1	-	3933	4010	splice acceptor AT, 7-nt deletion, 1-nt insertion
5					exon deletion
6					exon deletion
7					exon deletion
8	AOUJ01073286.1	-	2613	2704	1 nonsense codon
9					exon deletion
10					exon deletion
11					exon deletion
12					exon deletion
13					exon deletion

15. Saker falcon (*Falco cherrug*)

Exon	NCBI Accession	Strand	Start	End	
1	AKMU01022476.1	+	4606	4885	5-nt deletion, 1 nonsense codon, splice donor GG
2	AKMU01022476.1	+	7959	8105	splice acceptor AA
3	AKMU01022476.1	+	9661	9822	splice donor GC
4	AKMU01022476.1	+	11065	11147	1-nt deletion
5	AKMU01022476.1	+	12346	12513	
6	AKMU01022476.1	+	13470	13575	
7	AKMU01022476.1	+	14426	14591	1 nonsense codon, 1-nt deletion
8	AKMU01022477.1	+	641	732	
9	AKMU01022477.1	+	3900	3996	
10	AKMU01022477.1	+	4213	4272	1 nonsense codon
11	AKMU01022477.1	+	5292	5422	splice acceptor TG
12	AKMU01022477.1	+	5712	5826	
13	AKMU01022477.1	+	6579	6818	

16. Peregrine falcon (*Falco peregrinus*)

Exon	NCBI Accession	Strand	Start	End	
1	AKMT01062038.1	-	2847	3126	5-nt deletion, 1 nonsense codon, splice donor GG
2	AKMT01062037.1	-	7069	7215	splice acceptor AA
3	AKMT01062037.1	-	5352	5513	splice donor GC
4	AKMT01062037.1	-	4029	4111	1-nt deletion
5	AKMT01062037.1	-	2659	2826	
6	AKMT01062037.1	-	1597	1702	
7	AKMT01062037.1	-	581	746	1 nonsense codon, 1-nt deletion
8	AKMT01062036.1	-	160	251	
9	AKMT01062035.1	-	16968	17064	1 nonsense codon
10	AKMT01062035.1	-	16692	16751	splice acceptor TG
11	AKMT01062035.1	-	15542	15672	
12	AKMT01062035.1	-	15138	15252	
13	AKMT01062035.1	-	14146	14385	

---

17. Red-legged seriema (*Cariama cristata*)

Exon	NCBI Accession	Strand	Start	End	
1	JJRQ01067943.1	-	3196	3483	
2	JJRQ01089398.1	-	6026	6172	
3	JJRQ01089398.1	-	4295	4456	
4	JJRQ01089398.1	-	3008	3091	
5	JJRQ01089398.1	-	1668	1835	1 nonsense codon
6					exon deletion
7					exon deletion
8	JJRQ01089398.1	-	157	248	
9	JJRQ01089397.1	-	9251	9347	
10	JJRQ01089397.1	-	8975	9034	splice acceptor AC, splice donor CT
11	JJRQ01089397.1	-	7870	8000	
12	JJRQ01089397.1	-	7477	7591	
13	JJRQ01089397.1	-	6778	7017	

---

18. Downy woodpecker (*Picoides pubescens*)

gene deletion

---

19. Northern carmine bee-eater (*Merops nubicus*)

Exon	NCBI Accession	Strand	Start	End	
1	JJRJ01070578.1	-	7864	7967	181-nt deletion
2					exon deletion

3	JJRJ01070578.1	-	5056	5217	
4					exon deletion
5					exon deletion
6					exon deletion
7					exon deletion
8					exon deletion
9	JJRJ01070578.1	-	1946	2035	7-nt deletion
10	JJRJ01070578.1	-	1669	1728	
11	JJRJ01070578.1	-	594	724	1 nonsense codon, splice donor TT
12	JJRJ01070578.1	-	191	305	
13	JJRJ01102545.1	-	12533	12766	splice acceptor AA, 1 nonsense codon

20. Rhinoceros hornbill (*Buceros rhinoceros silvestris*)

Exon	NCBI Accession	Strand	Start	End	
1	JMFK01029556.1	+	2267	2539	
2	JMFK01029556.1	+	5429	5575	
3	JMFK01029556.1	+	7116	7277	
4	JMFK01029556.1	+	8453	8536	
5	JMFK01029556.1	+	9694	9861	
6	JMFK01029556.1	+	10793	10895	
7	JMFK01029556.1	+	11673	11838	1-nt deletion
8	JMFK01029557.1	+	513	604	
9	JMFK01029557.1	+	3195	3291	
10	JMFK01029557.1	+	3509	3568	
11	JMFK01029557.1	+	4586	4716	
12	JMFK01029558.1	+	<1	82	5' partial (33-nt; recovered from WGS data)
13	JMFK01029559.1	+	857	1096	

21. Bar-tailed trogon (*Apaloderma vittatum*)

Exon	NCBI Accession	Strand	Start	End	
1	JMFV01079656.1	-	34047	34331	
2	JMFV01079656.1	-	30744	30878	splice acceptor AT, splice donor TT
3	JMFV01079656.1	-	28613	28714	5' deletion (58 nt), 1 nonsense codon, 2-nt deletion
4	JMFV01079656.1	-	27329	27412	
5	JMFV01079656.1	-	25990	26164	7-nt insertion
6	JMFV01079656.1	-	24948	25046	5' deletion (4 nt), 2 nonsense codons
7	JMFV01079656.1	-	24055	24211	10-nt deletion
8	JMFV01079656.1	-	22011	22102	
9	JMFV01079656.1	-	20694	20790	

10	JMFV01079656.1	-	20411	20470	splice acceptor GG
11	JMFV01079656.1	-	19249	19379	
12	JMFV01079656.1	-	18874	18988	
13	JMFV01079656.1	-	17962	18181	1-nt insertion, 1 nonsense codon, 3' deletion (21 nt)

22. Cuckoo roller (*Leptosomus discolor*)

Exon	NCBI Accession	Strand	Start	End	
1	JJRK01070547.1	-	16649	16933	
2	JJRK01070547.1	-	13491	13637	splice acceptor GG, 1 nonsense codon
3	JJRK01070547.1	-	12909	13061	
4	JJRK01070547.1	-	11587	11669	1-nt deletion
5	JJRK01070547.1	-	11038	11205	splice donor AG
6	JJRK01070547.1	-	10411	10513	splice acceptor TG, 1 nonsense codon
7	JJRK01070547.1	-	9447	9613	
8	JJRK01070547.1	-	7696	7785	2-nt deletion, splice donor AT
9	JJRK01070547.1	-	3835	3925	
10	JJRK01070547.1	-	3573	3632	splice acceptor TG
11	JJRK01070547.1	-	2432	2560	2-nt deletion
12	JJRK01070547.1	-	2032	2146	
13	JJRK01070547.1	-	1126	1289	76-nt deletion

23. Speckled mousebird (*Colius striatus*)

Exon	NCBI Accession	Strand	Start	End	
1	JJRP01021333.1	-	9248	9516	5' deletion (12 nt), 1-nt deletion, splice donor TC
2	JJRP01021333.1	-	6158	6304	splice donor GC
3	JJRP01021333.1	-	4520	4673	8-nt deletion
4	JJRP01021333.1	-	3242	3323	splice acceptor AA, 1 nonsense codon, 2-nt deletion
5	JJRP01021333.1	-	2869	3036	splice acceptor GA, splice donor AT
6	JJRP01021333.1	-	2046	2148	
7	JJRP01021333.1	-	1104	1270	splice acceptor AT, 2 nonsense codons
8	JJRP01021332.1	-	9970	10061	
9					exon deletion
10					exon deletion
11	JJRP01021332.1	-	6727	6857	2 nonsense codons
12	JJRP01021332.1	-	6328	6442	splice acceptor GG, splice donor CT
13	JJRP01021332.1	-	5381	5620	

24. Barn owl (*Tyto alba*)

>Tyto_alba No=24 length=1854 name="Barn owl"
ATGATGGCAGTTCTCCTCTCAAGAATCAAGGGTATGCTCTTCCTCTTGTTTCTCCCATTCTTTTGTTCTG
GTCAGCCCGCACCTCCACTGCTGCGTTTCTCCATCTTCCTGGATCCTTCAAACACGGTCTACCTCCGCTG
GGACCATGACGAACAGGAGCTGATGACGTTTGAGCTGCAGGTCCCTACAACGGGCTGGGTGGCATTTGGA
TTCAGCCCTCGCGGAGAATTGCCTGGATCTGACATTGTGATAGGAGGTACCTTCCCAAATGGCAGTATCT
ACTTCTCTGATTGTCACATGGTAGATGAGGCAACGCTTGAGGAAGATGAGAGCCAGGACTACCAACTGCT
GTCAGTGACAGAGAATGAGACCTCCACTACCATGCTGTTCAAACGCCACCTCCGGACGTGTGACCCAAAT
GACCTTGATATCACGACGGACACAGCACGCCTCATTGCTGCATTTGGCACTGATGACACAGTCCAATTCT
TTAAAGGCCAAAGATTTTCCAAATCTCTTTTCTTGATGAGGTACAGAGGCCCATCTGACTTAAATGACCC
CAAAATATTCTTCACCTATGACCTGAGGCTGGACAATTTTGCTGTTCCCATTGAAGAAACCAAGTATGCC
TGTACATTTCTCCCACTGCCCGTGGTCAAGCAGAAGCACCATATTTACAAGTTTGAACCCATAATAACAC
CCCACAACATAACCTTGGTTCATCATATTCTTGTTTATGCTTGTGGCAACGTCAGCGTGTTACCCCGTGG
CATAGATGATTGCTATGGAGCCAATCCAGATTTTGCCTTGTGCTCTCAGGTGCTGGTGGGCTGGGCTGTT
GGAGGAGAGTCTTATCAACTTCCAGATGAAGCTGCATTTTCCATAGGGACACCCTGGGACCCTTGGTACA
TCCGACTAGAAATCCATTACAGCAATTTTGACTTATTACCAGGCTTGATTGACAGCTCAGGGGTACGAAT
CTACTATACTCCAGAGCTACGGAATTATGATGTGGGCGTTCTGCAAACAGGCATCTTCACTTTCCCTGTG
CATTTCATTCCTCCTGGAGCAGAATCCTACAGATCTTATGGCCTTTGCAATTCGAGCCAGTTTGATGAAA
TGAATGGGATGCTGGTTCCAGATCTGCATGTCTTTGCCTACTTGCTTCACACCCACTTGTCTGGCAGAGG
AGTGAAAGCTGCTCAATACCGGAACGGCGAGCAGCTAAGGATCATCTGTGAGGACAATAACTATGACTTC
AGACTGCAGGAGATACGGGACATGAAGGAAATCCTCATAATCAAACCAGGGGATGAGATCCTGGTCGAAT
GCAACTTTCAGACACTGGATCGGTCAGGGGTTACTTTTGGTGGGCCAAGCACCATGAATGAGATGTGTCT
CACTTTCCTCTTCTATTACCCTCGTAACAACATCTCCAGTTGTATGGGCTACCCTGACATTTTGTACATT
GCGCATGTACTCAAGCAGGAGGCCTCAGATGCAGTGGAAGGAATGATGGCCATGGACTTCGTTGACTGGG
ACAACGATACTGTCAAAATTGCAGAGAAAGCAGCCAAGGAGGCAAATCAAGTAGTCATGATTAAAAACAT
TAATGAACTCCAAAGAAGTGAGAGTGGTCTAATCAGAGACATTAGTATTCCAGAGCAGGCTGCCTGCCAC
AATATTTCTGGACAACTCTCACTGTCGGGTCTGAGGGCCACTGCAAACCTTCATTTGACCTCAGTGTGCA
CTTCTGAGTCATCAGCTACCACAGAAATTGCTTCACTTCCTCTTATTTCTCTCACACATCTGGTGTTTGC
TTGCCTTATTTTGGCCTCTGACTATGGGAAGTAA

>Tyto_alba No=24 length=617 name="Barn owl"
MMAVLLSRIKGMLFLLFLPFFCSGQPAPPLLRFSIFLDPSNTVYLRWDHDEQELMTFELQVPTTGWVAFG
FSPRGELPGSDIVIGGTFPNGSIYFSDCHMVDEATLEEDESQDYQLLSVTENETSTTMLFKRHLRTCDPN
DLDITTDTARLIAAFGTDDTVQFFKGQRFSKSLFLMRYRGPSDLNDPKIFFTYDLRLDNFAVPIEETKYA
CTFLPLPVVKQKHHIYKFEPIITPHNITLVHHILVYACGNVSVLPRGIDDCYGANPDFALCSQVLVGWAV
GGESYQLPDEAAFSIGTPWDPWYIRLEIHYSNFDLLPGLIDSSGVRIYYTPELRNYDVGVLQTGIFTFPV
HFIPPGAESYRSYGLCNSSQFDEMNGMLVPDLHVFAYLLHTHLSGRGVKAAQYRNGEQLRIICEDNNYDF
RLQEIRDMKEILIIKPGDEILVECNFQTLDRSGVTFGGPSTMNEMCLTFLFYYPRNNISSCMGYPDILYI
AHVLKQEASDAVEGMMAMDFVDWDNDTVKIAEKAAKEANQVVMIKNINELQRSESGLIRDISIPEQAACH
NISGQLSLSGLRATANLHLTSVCTSESSATTEIASLPLISLTHLVFACLILASDYGK

Exon	NCBI Accession	Strand	Start	End
1	JJRD01156581.1	-	19802	20089
2	JJRD01156581.1	-	16910	17056
3	JJRD01156581.1	-	15196	15357
4	JJRD01156581.1	-	13897	13980
5	JJRD01156581.1	-	12547	12714
6	JJRD01156581.1	-	11498	11600
7	JJRD01156581.1	-	10482	10648
8	JJRD01156581.1	-	8577	8668
9	JJRD01156581.1	-	4288	4384
10	JJRD01156581.1	-	4025	4084
11	JJRD01156581.1	-	2883	3013
12	JJRD01156581.1	-	2470	2584

13	JJRD01156581.1	-	1516	1755

25. Turkey vulture (*Cathartes aura*)

```
>Cathartes_aura No=25 length=1854 name="Turkey vulture"
ATGATGGCAGTTCTCTTCTCAAGAATCAAGGATATGCTCTTTCTCTTGTTCCTCCCATGCTTTTGTTCTG
GTCAGCCTGCATGTCCACTTCTGCGTTTCTCCATCTTCCTGGATCCTTCAAACATGGTCTACCTCCGCTG
GGACCATGATGAACAGGAGCTGATGACATTTGAGCTGCAGGTCCATACAACTGGCTGGGTGGCATTTGGA
TTCAGCCCTCATGGAGAGTTGCCTGGATCTGACATTGTGATAGGAGGTGTCTTCCCAAATGGCAGCATCT
ACTTCTCTGATTGTCATGTGGTAGATGAGGCAACCCTTGAGGAAGATGAGAGCCAAGACTACCAACTGCT
GTCAGTGACAGAGAATGAGACCTCCACCACCATGCTGTTCAAACGCCACCTCCGGACATGTGACCCAAAT
GACCTGGATATCACAGTGGACACAGCACGCGTCGTTACTGCATTTGGCACTGATGACACAGTCCAATTCT
TTAAAGGCCAAAGATTCTCCAAATCTCTCTTCTTGATGAGGTACAGAGGCCCATGTGACTCCACTGACCC
CAAAATATTCTTCAGCTATGACCTGAGGCTGGACAATTTTGCTGTTCCAGTTGAAGAAACCAAGTATGCC
TGTACCTTTATCCCACTGCCCATGGTCAAGCAGAAACATCATGTCTACAAGTTCGAACCTGTAATAACAC
CCCGCAACATAACCTTGATTCATCATATTCTTATTTATGCCTGTGGCAACGCTAGTGTGTTACCCAGTGG
CATAGATGATTGCTATGGAGCCAATTCAGATTTCTCCCTGTGCTCTCAAGTGCTTTTTGGCTGGGCTGTT
GGAGGAAAGTCTTATCAATTTCCAGATGAAGCTGCAGTTTCCATAGGGACACCTTGGGACCCTCAGTACG
TCCGACTAGAAATCCATTACAGCAATTTTGACTTGTTACCAGGCTTGATCGACAGCTCAGGGGTACGAAT
CTACTATACTCCAGAACTACGGAAATATGATGTGGGGGTTCTGCAAACAGGCATCTTCATTTTCCCTATG
CATTTCATTCCTCCTGGAGCAGAATCCTACAGATCTTATGGCCTTTGCAATTCCAGCCAGTTTGATGAAA
TGAATGGGATGCTGGTTCCAGATCTGCATGTCTTTGCCTACTTGCTTCACACCCACCTGTCTGGCAGAGG
AGTGAAAGCTGCTCAATACCGGAACGGTGAGCAGCTGAGGATCATCTGTGAGGACAATAAGTATGACTTC
AGACTGCAGGAGATTCGGGACACGAAGGAAATCCTCATAATCAAACCAGGGGATGAGATCCTGGTCGAAT
GCAACTTTCAGACACTGGATCGGTCAGGGATTACTTTTGGTGGACCAAGCACCATGAATGAGATGTGTCT
CACGTTCCTCTTCTACTACCCTCGTAACAACATCTCCAGTTGTATGGGCTACGCCGACATTTTGTACATT
GCGCACGTACTCAAGCAGGAGGCCTCAGATGCAGTGGAAGGAATGATGGCCATGGACTTTGTTGACTGGG
ACAATGATACTATCAAAATTGCAGAGAAAGCAGCCAAGGAGGCAGATCAAGTAGTCATGATTAAAACTAT
TAATGAACTCCAGAGAAATGAGAGTGGTCTAATCCGAGACATGAGTATTCCAGAGCGGGCTGCCTGTCAC
AACATTTCTGGACACCTCTCACTGTCACATCTGAGGGCCACTGCAAACCTTCGTTTGACCGCAGTATGCA
CTTCTGAGTCATCAACCACCAAAGAAACTGCTTCACTTCCTCTTCTTTCTTTCACACAGCTGGTGTTTCC
TTGGCTTATCTTGGCCTCTGAGTATGGGAAGTGA
```

```
>Cathartes_aura No=25 length=617 name="Turkey vulture"
MMAVLFSRIKDMLFLLFLPCFCSGQPACPLLRFSIFLDPSNMVYLRWDHDEQELMTFELQVHTTGWVAFG
FSPHGELPGSDIVIGGVFPNGSIYFSDCHVVDEATLEEDESQDYQLLSVTENETSTTMLFKRHLRTCDPN
DLDITVDTARVVTAFGTDDTVQFFKGQRFSKSLFLMRYRGPCDSTDPKIFFSYDLRLDNFAVPVEETKYA
CTFIPLPMVKQKHHVYKFEPVITPRNITLIHHILIYACGNASVLPSGIDDCYGANSDFSLCSQVLFGWAV
GGKSYQFPDEAAVSIGTPWDPQYVRLEIHYSNFDLLPGLIDSSGVRIYYTPELRKYDVGVLQTGIFIFPM
HFIPPGAESYRSYGLCNSSQFDEMNGMLVPDLHVFAYLLHTHLSGRGVKAAQYRNGEQLRIICEDNKYDF
RLQEIRDTKEILIIKPGDEILVECNFQTLDRSGITFGGPSTMNEMCLTFLFYYPRNNISSCMGYADILYI
AHVLKQEASDAVEGMMAMDFVDWDNDTIKIAEKAAKEADQVVMIKTINELQRNESGLIRDMSIPERAACH
NISGHLSLSHLRATANLRLTAVCTSESSTTKETASLPLLSFTQLVFPWLILASEYGK
```

Exon	NCBI Accession	Strand	Start	End
1	JMFT01000581.1	+	49174	49461
2	JMFT01000581.1	+	52508	52654
3	JMFT01000581.1	+	54229	54390
4	JMFT01000581.1	+	55611	55694
5	JMFT01000581.1	+	56857	57024
6	JMFT01000581.1	+	57966	58068
7	JMFT01000581.1	+	58858	59024
8	JMFT01000581.1	+	60842	60933

9	JMFT01000582.1	+	3081	3177	
10	JMFT01000582.1	+	3394	3453	
11	JMFT01000582.1	+	4483	4613	
12	JMFT01000582.1	+	<4895	5000	5' partial (9 nt; recovered from WGS data)
13	JMFT01000582.1	+	5757	5996	

---

26. White-tailed eagle (*Haliaeetus albicilla*)

```
>Haliaeetus_albicilla No=26 length=1854 name="White-tailed eagle"
ATGATGGCAGTTCTCTTCTCAGGAATCAAGGGGATGCTCTTCTTCTTGTTCCTCTCATGCTTTTGTTCTT
GTCAGCCTGCACCTCCACTGCTGCGTTTCTCCATCTTCCTGGATCCTTCAAACATGGTCTACCTCCGCTG
GGACCATGACGAACAGGAGCTGATGACATTTGAGCTGCAGGTCCGTACAACTGGCTGGGTGGCATTTGGA
TTCAGCCCTCACGGAGAGTTGCCTGGATCTGACATTGTGATAGGAGGTGTCTTCCCAAATGGCAGCATCT
ACTTCTCCGATTGTCACATAGTAGATGAGGCAACCCTTGAGGAAGATGGGAGCCAGGACTACCAACTGCT
GTCAATGACAGAGAATGAGACCTCCACCACCGTGCTGTTCAAACGCCACCTAAGGACGTGTGACCCCAAT
GACCAGGATATCACGATGGACACAGCACGCCTCATTACTGCATTTGGCACTGATGACACAGTCCAATTCT
TTGAAGGCCAAAGATTTTCCAAATCTGTTTTCTTGATGAGGTACAGAGGCCCATCTGACTTAACTGACCC
CAAAATATTCTTCACCTATGACCTGAGGCTGGACAATTTTGCTGTCCCAGTTGAAGAAACCATGTATGCC
TGTACCTTTATCCCACTGCCCATGGTCAAGCAGAAACACCATATCTACAAGTTCGAACCTGTAATAACAC
CCCACAACATAACCTTGGTTCATCATATTCTTGTTTATGCCTGTGGCAACGCCAGCGTGTTACCCAGTGG
CATAGACGATTGCTATGGAGCCAATCCAGATTTTGCCCTGTGCTCTCAGGTGCTTGTGGGCTGGGCTGTC
GGAGGGGAGTCTTATCAATTTCCAGATGAAGCTGCGGTTTCCATAGGGACACCTTGGGACCCTCAGTACG
TCCGACTAGAAATCCATTACAGCAATTTTGACTTGTTACCAGGCTTGATCGACAGCTCAGGGGTACGAAT
CTACTATACTCCGGAGCTACGGCAGTATGATGTGGGGGTTCTGCAAACAGGCGTCTTCATTTTCCCTGTG
CATTTCATTCCTCCCGGGGCAGAATCCTACAGATCTTATGGCCTTTGCAATTCCAGCCAGTTTGATGAAA
TGAATGGGATGTTGGTTCCAGATTTGCATGTATTTGCCTACTTGCTTCACACCCACCTGTCTGGCAGAGG
AGTGAAAGCTGCTCAATACCGGAATGGTGAGCAGCTGAGGATCATCTGTGAGGACAATAAGTACGACTTC
AGACTGCAGGAGATTCGGGACATGAAAGAAATCCTCATAATCAAACCAGGCGATGAGATCCTGGTCGAAT
GCAATTTTCAGACGCTGGATCGATCAGGGATTACTTTTGGTGGGCCAAGCACCATGGATGAGATGTGTCT
GACATTCCTCTTCTACTACCCTCGTAACAACATCTCCAGTTGTATGGGCTTCCCTGACATACTGTACGTT
GCTCATGCACTCAAACAGGAGGCTTCAGATGCAGTGGAAGGAATGATGGCCATGGAGTTTGTTGACTGGG
ACAATGATACTGTCAAAATTGCAGAGAAGGCAGCCAAGGAGGCAGATCAAGTAGTCATGATTAAAACCAT
TGATGAACTCCAGAGAAATGAGAGTGGTCTAATCAGAGACATTAGTATGCCAGAGCGGGCTGCCTGTCAC
AATATTTCTGGACACCTCTCACTGTCAGGTCGGAGGGCCACTGCAAACCTCCGTTTGACCGCAGTATGCA
CTTCTGAGTCACCAACCACCAAAGAAACTGCTATACTTCCTCTTCTTTCTCTCACACAGCTGGTGTTTTC
TTGGCTGATCTTGGCCTCCGAGTATGGGAAATGA

>Haliaeetus_albicilla No=26 length=617 name="White-tailed eagle"
MMAVLFSGIKGMLFFLFLSCFCSCQPAPPLLRFSIFLDPSNMVYLRWDHDEQELMTFELQVRTTGWVAFG
FSPHGELPGSDIVIGGVFPNGSIYFSDCHIVDEATLEEDGSQDYQLLSMTENETSTTVLFKRHLRTCDPN
DQDITMDTARLITAFGTDDTVQFFEGQRFSKSVFLMRYRGPSDLTDPKIFFTYDLRLDNFAVPVEETMYA
CTFIPLPMVKQKHHIYKFEPVITPHNITLVHHILVYACGNASVLPSGIDDCYGANPDFALCSQVLVGWAV
GGESYQFPDEAAVSIGTPWDPQYVRLEIHYSNFDLLPGLIDSSGVRIYYTPELRQYDVGVLQTGVFIFPV
HFIPPGAESYRSYGLCNSSQFDEMNGMLVPDLHVFAYLLHTHLSGRGVKAAQYRNGEQLRIICEDNKYDF
RLQEIRDMKEILIIKPGDEILVECNFQTLDRSGITFGGPSTMDEMCLTFLFYYPRNNISSCMGFPDILYV
AHALKQEASDAVEGMMAMEFVDWDNDTVKIAEKAAKEADQVVMIKTIDELQRNESGLIRDISMPERAACH
NISGHLSLSGRRATANLRLTAVCTSESPTTKETAILPLLSLTQLVFSWLILASEYGK
```

Exon	NCBI Accession	Strand	Start	End
1	JPRR01021096.1	+	26194	26481
2	JPRR01021096.1	+	29471	29617
3	JPRR01021096.1	+	31199	31360
4	JPRR01021096.1	+	32581	32664

5	JPRR01021096.1	+	34999	35166
6	JPRR01021096.1	+	36110	36212
7	JPRR01021096.1	+	37000	37166
8	JPRR01021096.1	+	38969	39060
9	JPRR01021096.1	+	41183	41279
10	JPRR01021096.1	+	41496	41555
11	JPRR01021096.1	+	42570	42700
12	JPRR01021096.1	+	42991	43105
13	JPRR01021096.1	+	43860	44099

---

27. Golden eagle (*Aquila chrysaetos canadensis*)

```
>Aquila_chrysaetos_canadensis No=27 length=1851 name="Golden eagle"
ATGGCAGTTCTCTTCTCAGGAATCAAGGGTATGCTCTTCTTCTTGTCCCTCCCATGCTTTTGTTCTTGTC
AGCCTGCACCTCCACTGCTGCGTTTCTCCATCTTCCTGGATCCTTCAAACATGGTCTACCTCCGCTGGGA
CCATGACGAACAGGAGCTGATGACATTTGAGCTGCAGGTCCATACAACTGGCTGGGTGGCATTTGGATTC
AGCCCTCACGGAGAGTTGCCTGGATCTGACATTGTGATAGGAGGTGTCTCCCCAAATGGCAGCATCTACT
TCTCCGATTGTCACGTGGTAGATGAGGCAACCCTTGAGGAAGATGGGAGCCAGGACTACCAACTGCTGTC
AGTGACAGAGAATGAGACCTCCACCACCGTGCTGTTCAAACGCCACCTAAGGACATGTGACCCCAATGAC
CAGGATATCACAATGGACACAGCACGCCTCATAACTGCATTTGGCACTGATGACACAGTCCAATTCTTTG
AAGGCCAAAGATTTTCCAAATCCCTTTTCTTGATGAGGTACAGAGGCCCATCTGACTTAACTGACCCCAA
AATATTCTTCACCTATGACCTGAGGCTGGACAATTTTGCTGTTCCAGTTGAAGAAACCACGTATGCCTGT
ACCTTTATCCCACTGCCCGTGGTCAAGCAGAAACACCATATCTACAAGTTCGAACCTGTAGTAACACCCC
ACAACATAACCTTGGTTCATCATATTCTTGTTTATGCCTGCGGCAACGCCAGCGTGTTACCCGGTGGCAT
AGATGATTGCTATGGAGCCAATCCAGATTTTGCCCTGTGCTCTCAGGTGCTTGTGGGCTGGGCTGTTGGA
GGGGAGTCTTATCAATTTCCAGATGAAGCTGCCGTTTCCATAGGGACACCTTGGGACCCTCAGTACGTCC
GACTAGAAATCCATTACAGCAATTTTGACTTGTTACCAGGCTTGATCGACAGCTCAGGGGTACGAATCTA
CTATACTCCGGAGCTACGGAAGTATGACGTGGGGGTTCTGCAAACAGGCATCTTCACTTTCCCTGTGCAT
TTCATTCCTCCTGGGGCAGAATCCTACAGATCTTATGGCCTTTGCAATTCCAGCCGGTTTGATGAAATGA
ATGGGATGTTGGTTCCAGATTTGCATGTATTTGCCTACTTGCTTCACACCCACCTGTCTGGCAGAGGAGT
GAAAGCTGCTCAATACCGGAATGGTGAGCAGCTGAGGATCATCTGTGAGGACAATCAGTACGACTTCAGA
CTGCAGGAGATTCGGGACATGAAAGAAATCCTCATAATCAAACCAGGGGATGAGATCCTGGTCGAATGCA
ATTTTCAGACGCTGGATCGATCAGGGATTACTTTTGGTGGGCCAAGCACCATGGATGAGATGTGTCTGAC
ATTCCTCTTCTACTACCCTCGTAACAACATCTCCAGTTGTATGGGCTTCCCTGACATACTGTACATTGCG
CATGCACTCAAACAGGAGGCTTCAGATGCAGTGGAAGGAATGATGGCCATGGAGTTTGTTGACTGGGACA
ATGATACTGTCAAAATTGCAGAGAAGGCAGCCAAGGAGGCAGATCAAGTAGTCATGATTAAAACCATTGA
CGAACTCCAGAGAAATGAGAGTGGTTTAATCAGAGACATTAGTATTCCAGAGCGGCCTGCCTGTCACAAT
ATTTCTGGACACCTCTCACTGTCAGGTCTGAGGGCCACTGCAAACCTCCGTTTGACCGCAGTATGCACTT
CTGAGTCACCAACCACCAAAGAAACTGCTATACGTCCTCTTCTTTCTCTCACACAGCTGGTGTTTTCTTG
GTTGATCTTGGCCTCCGAGTATGGGAAGTGA
```

```
>Aquila_chrysaetos_canadensis No=27 length=616 name="Golden eagle"
MAVLFSGIKGMLFFLSLPCFCSCQPAPPLLRFSIFLDPSNMVYLRWDHDEQELMTFELQVHTTGWVAFGF
SPHGELPGSDIVIGGVSPNGSIYFSDCHVVDEATLEEDGSQDYQLLSVTENETSTTVLFKRHLRTCDPND
QDITMDTARLITAFGTDDTVQFFEGQRFSKSLFLMRYRGPSDLTDPKIFFTYDLRLDNFAVPVEETTYAC
TFIPLPVVKQKHHIYKFEPVVTPHNITLVHHILVYACGNASVLPGGIDDCYGANPDFALCSQVLVGWAVG
GESYQFPDEAAVSIGTPWDPQYVRLEIHYSNFDLLPGLIDSSGVRIYYTPELRKYDVGVLQTGIFTFPVH
FIPPGAESYRSYGLCNSSRFDEMNGMLVPDLHVFAYLLHTHLSGRGVKAAQYRNGEQLRIICEDNQYDFR
LQEIRDMKEILIIKPGDEILVECNFQTLDRSGITFGGPSTMDEMCLTFLFYYPRNNISSCMGFPDILYIA
HALKQEASDAVEGMMAMEFVDWDNDTVKIAEKAAKEADQVVMIKTIDELQRNESGLIRDISIPERPACHN
ISGHLSLSGLRATANLRLTAVCTSESPTTKETAIRPLLSLTQLVFSWLILASEYGK
```

Exon	NCBI Accession	Strand	Start	End

1	JDSB01102625.1	-	11004	11288
2	JDSB01102625.1	-	8087	8233
3	JDSB01102625.1	-	6347	6508
4	JDSB01102625.1	-	5036	5119
5	JDSB01102625.1	-	3658	3825
6	JDSB01102625.1	-	2615	2717
7	JDSB01102625.1	-	1653	1819
8	JDSB01102624.1	-	2908	2999
9	JDSB01102623.1	-	4253	4349
10	JDSB01102623.1	-	3976	4035
11	JDSB01102623.1	-	2834	2964
12	JDSB01102623.1	-	2430	2544
13	JDSB01102623.1	-	1435	1674

---

28. White-tailed tropicbird (*Phaethon lepturus*)

```
>Phaethon_lepturus No=28 length=1854 name="White-tailed tropicbird"
ATGATGGCAGTTCTCTTCACAAGAATCAAGGGTATGCCCTTCCTCTTGTTCCTCCCATGCTTTTGTTCTG
GTCATCCTGCAGCTCCACTGCTGCGTTTCTCCATCTTTCTGGAGCCTTCAAACATGGTCTACCTCCGCTG
GGACCATGACGAACAGGAGATGATAACATTTGAGCTGCAGGTCCATACAACTGGCTGGGTGGCATTTGGA
TTCAGCCCTCATGGAGAGTTGCCTGGATCTGATATTGTGATAGGAGGTGTCTTCCCGAATGGCAGCATCT
ACTTCTCTGACTGTCATGTGGTCGATGAGGCAACCCTGGAGGAAGATGAGAGCCAGGACTACCAACTGCT
GTCAGTAACAGAGAATGAGACCTCCACCACCATGATGTTCAAACGCCACCTCCGGACATGTGACCCAAAT
GACCTGGATATCACAGTGGATACAGCACGCCTTGTTACTTCATTTGGCACTGATGACACAGTCCAATTCT
TTAAAGGCCAAAGATTTTCCAAATCTCTTTTCTTGATGAGGTCCAGAGGCCCATCTGACTCAACTGGCCC
CAAAACATTCTTCACCTATGACCTGAGGCTGGACAATTTTGCTGTTCCAGCTGAAGAAACCAAGTATGCC
TGTACTTTTATGCCACTGCCCATGGTCAAGCAGAAACACCATATCTACAAGTTCGAACCTGTAATAACAC
CCCACAACATAACCTTGGTTCATCATATTCTTGTTTATGCCTGTGGCAACGCCAGCGTGTTACCCAGTGG
CATAGACGATTGCTATGGAGCCAATCCAGATTTTGCCCTGTGCTCACAGGTGCTTGTGGGCTGGGCTGTT
GGAGGAGAGTCTTACCAATTTCCAGATGAAGCTGCCATTTCCATAGGCACACCTCGGGACCCAGAGTACA
TCCGACTAGAAATCCATTACAGCAATTTTGACTTGTTACCAGGCCTGATTGACAGCTCAGGGGTACGAAT
CTATTATACGCCAGAGCTACGGAAATATGATGTGGGAGTTCTGCAAACAGGCATCTTCACTTTCCCTGTG
CATTTCATTCCTCCTGGAGCAGAATCCTACAGATCCTACGGTCTTTGCAATTCCAGCCAGTTTGATGAAA
TGAATGGGATGCTGGTTCCAGATCTGCATGTCTTTGCCTACTTGCTTCACACCCACTTATCTGGCAGAGG
AGTGAGAGCTGCTCAATACAGGAATGGTGAGCCGCTGGGGATCATCTGTGAGGACAATAAGTATGACTTC
AGACTGCAGGAGATTCGGGACATGAAGGAAATCCTCATAATCAAACCAGGGGACGAGATCCTGGTTGAAT
GCAACTTTCAGACACTGGATCGGTCAGAGATTACTTTTGGTGGGCCAGGCACCATGAATGAAATGTGTCT
CACATTCCTCTTCTACTATCCTCGTAACAACATGTCCAGTTGTATGGGCTACCCTGACATTTTGTACATT
GCACATGTACTCAAGCAGAAGGCCTCAGATGCACTGGAAGGAATGATGGCCATGGGCTTTGTTGACTGGG
ACAATGAGACTGTCAAAATTGCAGAGAAAGCAGCCAAGGAGGCAGATCAAGTAGTCATGATTAAAACCAT
TAATGAACTCCAGAGAAATGAGACTGGTCTAATCAGAGACATTAGTATTCCAGAGCGGGCTGCCTGTCAC
AATATTTCTGGACACCTCTCACTGTCAGGTCTGAGGGCCACTGCAAACCTTCGTCTGACCGCAGTATGCA
CTTCTGAGTCATCAACCACCAAAGAAACTGCTTCACTTCCTCTTCTTTCTCTCACACAGCTGGTGTTTGC
TTGGCTTATCTTGGCCTCTGAGTATGGGAAGTGA

>Phaethon_lepturus No=28 length=617 name="White-tailed tropicbird"
MMAVLFTRIKGMPFLLFLPCFCSGHPAAPLLRFSIFLEPSNMVYLRWDHDEQEMITFELQVHTTGWVAFG
FSPHGELPGSDIVIGGVFPNGSIYFSDCHVVDEATLEEDESQDYQLLSVTENETSTTMMFKRHLRTCDPN
DLDITVDTARLVTSFGTDDTVQFFKGQRFSKSLFLMRSRGPSDSTGPKTFFTYDLRLDNFAVPAEETKYA
CTFMPLPMVKQKHHIYKFEPVITPHNITLVHHILVYACGNASVLPSGIDDCYGANPDFALCSQVLVGWAV
GGESYQFPDEAAISIGTPRDPEYIRLEIHYSNFDLLPGLIDSSGVRIYYTPELRKYDVGVLQTGIFTFPV
HFIPPGAESYRSYGLCNSSQFDEMNGMLVPDLHVFAYLLHTHLSGRGVRAAQYRNGEPLGIICEDNKYDF
```

RLQEIRDMKEILIIKPGDEILVECNFQTLDRSEITFGGPGTMNEMCLTFLFYYPRNNMSSCMGYPDILYI
AHVLKQKASDALEGMMAMGFVDWDNETVKIAEKAAKEADQVVMIKTINELQRNETGLIRDISIPERAACH
NISGHLSLSGLRATANLRLTAVCTSESSTTKETASLPLLSLTQLVFAWLILASEYGK

Exon	NCBI Accession	Strand	Start	End
1	JJRF01073064.1	+	2912	3199
2	JJRF01073064.1	+	6164	6310
3	JJRF01073064.1	+	8384	8545
4	JJRF01073064.1	+	9753	9836
5	JJRF01073064.1	+	11029	11196
6	JJRF01073064.1	+	12608	12710
7	JJRF01073064.1	+	13374	13540
8	JJRF01073064.1	+	15359	15450
9	JJRF01073065.1	+	1476	1572
10	JJRF01073066.1	+	191	250
11	JJRF01073066.1	+	1255	1385
12	JJRF01073066.1	+	1671	1785
13	JJRF01073066.1	+	2537	2776

29. Sunbittern (*Eurypyga helias*)

Exon	NCBI Accession	Strand	Start	End	
1					exon deletion
2					exon deletion
3	JJRO01041941.1	+	3849	4003	1 nonsense codon, 7-nt deletion
4	JJRO01041941.1	+	5215	5299	1-nt insertion
5	JJRO01041941.1	+	6479	6647	splice acceptor AT, 1-nt insertion
6	JJRO01041941.1	+	7576	7678	splice acceptor TT, 2 nonsense codons
7	JJRO01041941.1	+	8312	8475	
8					exon deletion
9					exon deletion
10					exon deletion
11					exon deletion
12					exon deletion
13					exon deletion

30. Red-throated loon (*Gavia stellata*)

```
>Gavia_stellata No=30 length=1854 name="Red-throated loon"
ATGATGGCAGTGCTCTTCTCAAGAATCAAGGGTATGCTCTTCCTCTTGTTCCTCCCATGCTTTTGTTCTG
GTCAGCCTGCACCTCCGCTGCTGCGTTTCTCCATCTTCCTGGATCCTTCAAACACGGTCTACCTCCGCTG
GGACCATGACGAACAGGAGCTGATGACGTTTGAGCTGCAGGTCCCTACAACTGGCTGGGTGGCATTTGGA
TTCAGCCCTCACGGAGAGTTGCCTGGATCTGACATTGTGATAGGAGGTGTCTTCCCAAATGGCAGCATCT
```

```
ACTTCTCCGATTGTCATGTGGTAGATGAGGCAACCCTTGAGGAAGATGAGAGCCAGGACTACCAACTGCT
GTCAGTAACAGAGAATGAGACCTCCACCACCATGCTGTTCAAACGCCATCTCCGGACATGTGACCCAAAT
GACCTGGATATCACAATGGACACAGCACGCCTCGTTACTGCATTTGGCATGGATGACACAGTCCAATTCT
TTAAAGGCCAAAGATTTTCCAAATCTCTTTTCTTGATGAGGTACAGAGGCCCATCTGACTCAACTGACCC
CAAAATATTCTTCACCTATGACCTGAGGCTGGACAATTTTGCCGTTCCAGCTCAAGAGACCAAGTATGCC
TGTACCTTTATCCCACTGCCCATGGTCAAGCAGAAACACCATATTTACAAGTTCGAACCTGTAATAACAC
CCCACAAAATAACCTTGATTCATCATATTCTTGTTTACGCCTGTGGCAACGCCAGCCCGTTACCCAGTGG
CATAGATGATTGCTATGGGGCCAATCCAGATTTTGCCCTGTGCTCTCAGGTGCTTGTGGGCTGGGCTGTT
GGAGGAGAGTCTTACCAATTTCCAGATGAAGCTGCAGTTTCCATAGGGACACCTTGGGACCCTCAGTACG
TCCGACTAGAAATCCATTACAGCAATTTTGACTTGTTACCAGGCTTGATAGACAGTTCAGGGCTACGAAT
CTACTATACGCCGGAGCTACGGAAATATGATGTGGGGGTTCTGCAAACAGGCATCTTCACTTTCCCTGTG
CATTTCATTCCTCCTGGAGCAGAATCCTACAGATCCTACGGCCTTTGCAATTCCAGCCAGTTTGATGAAA
TGAATGGGATGCTGGTTCCAGATCTGCATGTCTTTGCCTACTTGCTTCACACCCACCTGTCTGGCAGAGG
AGTGAAAGCTGCTCAATACCGCAATGGTGAGCAGCTGGGGATCATCTGTGAGGACAATAAGTACGACTTC
AGACTGCAGGAGATTCGGGACATGAAGGAAAACCTCATAATCAAACCAGGTGATGAGATCCTGGTCGAAT
GCAACTTTCAGACACTGGATCGGTCAGGGATTACTTTTGGTGGGCCAAGCACCATGAATGAGATGTGTCT
CACATTCCTCTTCTACTACCCTCGTAACAACATCTCCAGTTGTATGGGCTACCCTGACATTTTGTATGTT
GCGCATGTACTCAAGCAGGAGGCCTCAGATGTGGTGGAAGGAATGATGGCCATGGACTTTGTTGACTGGG
ACAATGAGACTGTCAAAATTGCAGAAAAAGCAGCCAAGGAGGCAGATCAAGTAATCATGATTAAAACCAT
TAATGAACTCCAGAGAAATGAGAGTGGTCTCATCAGAGACATCAGTATTCCAGAGCGGGCTGCCTGTCAC
AATATTTCTGGATACCTCTCGCTGTCAGGCCTGAAGGCCACTGCAAACCTTCGTTTGACCGCAGTATGCA
CTTCTGAGTCACCAGCCACCAAAGAAACCGCTTCACATCCTCTTCTCTCTCTGACACAGCTGGTGTTTGC
TTGGCTTATCTTGTCCTCTGAGCATGGGAAATGA

>Gavia_stellata No=30 length=617 name="Red-throated loon"
MMAVLFSRIKGMLFLLFLPCFCSGQPAPPLLRFSIFLDPSNTVYLRWDHDEQELMTFELQVPTTGWVAFG
FSPHGELPGSDIVIGGVFPNGSIYFSDCHVVDEATLEEDESQDYQLLSVTENETSTTMLFKRHLRTCDPN
DLDITMDTARLVTAFGMDDTVQFFKGQRFSKSLFLMRYRGPSDSTDPKIFFTYDLRLDNFAVPAQETKYA
CTFIPLPMVKQKHHIYKFEPVITPHKITLIHHILVYACGNASPLPSGIDDCYGANPDFALCSQVLVGWAV
GGESYQFPDEAAVSIGTPWDPQYVRLEIHYSNFDLLPGLIDSSGLRIYYTPELRKYDVGVLQTGIFTFPV
HFIPPGAESYRSYGLCNSSQFDEMNGMLVPDLHVFAYLLHTHLSGRGVKAAQYRNGEQLGIICEDNKYDF
RLQEIRDMKENLIIKPGDEILVECNFQTLDRSGITFGGPSTMNEMCLTFLFYYPRNNISSCMGYPDILYV
AHVLKQEASDVVEGMMAMDFVDWDNETVKIAEKAAKEADQVIMIKTINELQRNESGLIRDISIPERAACH
NISGYLSLSGLKATANLRLTAVCTSESPATKETASHPLLSLTQLVFAWLILSSEHGK
```

Exon	NCBI Accession	Strand	Start	End
1	JJRM01006527.1	+	21615	21902
2	JJRM01006527.1	+	25043	25189
3	JJRM01006527.1	+	26766	26927
4	JJRM01006527.1	+	28266	28349
5	JJRM01006527.1	+	29496	29663
6	JJRM01006527.1	+	30603	30705
7	JJRM01006527.1	+	31385	31551
8	JJRM01006527.1	+	33436	33527
9	JJRM01006527.1	+	36869	36965
10	JJRM01006527.1	+	37182	37241
11	JJRM01006527.1	+	38594	38724
12	JJRM01006527.1	+	39015	39129
13	JJRM01006527.1	+	39888	40127

31. Emperor penguin (*Aptenodytes forsteri*)

Exon	NCBI Accession	Strand	Start	End	
1	JMFQ01072246.1	+	7243	7530	
2	JMFQ01072246.1	+	10558	10704	
3	JMFQ01072246.1	+	12266	12427	splice acceptor AT
4	JMFQ01072246.1	+	13645	13728	
5	JMFQ01072246.1	+	14914	15081	
6	JMFQ01072246.1	+	16031	16133	
7	JMFQ01072246.1	+	16929	17095	
8	JMFQ01072246.1	+	18865	18963	7-nt insertion
9	JMFQ01072246.1	+	22335	22431	
10	JMFQ01072246.1	+	22648	22707	splice donor GA
11					exon deletion
12					exon deletion
13	JMFQ01072246.1	+	24182	24424	

32. Adelie Penguin (*Pygoscelis adeliae*)

Exon	NCBI Accession	Strand	Start	End	
1	JMFP01065789.1	-	914	1201	1 nonsense codon
2	JMFP01065788.1	-	7639	7785	
3	JMFP01065788.1	-	5913	6074	
4	JMFP01065788.1	-	4617	4700	
5	JMFP01065788.1	-	3113	3280	
6	JMFP01065788.1	-	2071	2173	
7	JMFP01065788.1	-	1125	1291	
8	JMFP01065787.1	-	823	916	2-nt insertion
9	JMFP01065786.1	-	4183	4279	
10	JMFP01065786.1	-	3907	3966	
11	JMFP01065786.1	-	2761	2891	
12	JMFP01065786.1	-	2362	2476	
13	JMFP01065786.1	-	1356	1598	

33. Northern fulmar (*Fulmarus glacialis*)

```
>Fulmarus_glacialis No=33 length=1809 name="Northern fulmar"
ATGATGGCAGTTCTCTTATCAAGAATCAAGGGTATGCTCTTCCTCTTGTTCCTCCCATGCTTTTGTTCTG
GTCAGCCTGCACCTCCACCTCTGCGTTTCTCCATCTTCCTGGATCCTTCAAAGATGGTCTACCTCCGCTG
GGACCATGACGAACAGGAGCTGATGACGTTTGAGCTGCAGGTCCATACAACTGGCTGGGTGGCATTTGGA
TTCAGCTCTCATGGAGAGTTGCCTGGATCTGACATTGTGATAGGAGGTGTCTTCCCAAATGGCAGCATCT
ACTTCTCTGATTGTCATGTGGTAGATGAGGCAACCATTGAGGAAGACGAGAGCCAGGACTACCAACTGCT
GTCAGTGACAGAGAATGAGACCTCCACCACCATGCTGTTCAAACGCCACCTCCGGACATGTGACCCAAAC
GACCTGGATATCACAGTAGACACAGCACGCCTCGTTGCTGCCTTTGGTACTGATGACACAGTCCCATTCT
TTAAAGGCCAAATATTTTCCAAGTCCCTTTTCTTGTTGAGGTTCAGAAGCCCATCAACTGACCCTAAAAT
```

```
ATTCTTCACCTATGACCTGAGGCTGGACAATTTTGCTGTTCCAGTTGAAGAAACCAAGTATGCCTGTAAA
TTTATCCCACTGCCCATGGTCAAGCAGAAACACCATATCTACAAGTTCGATCCAGTAATAACACCCCACA
ACATAACCTTGGTTCATCATATTCTTATTTATGGCTGTGGCAATGCCAGCGTGTTACCCAGTGGCATAGA
TGATTGCTATGGAGCCAATCCAGATTTTTCCCTGTGCTCTCAGGTGCTTATGGGCTGGGCTATTGGAGGA
CAGACTTATCAATTTCCAGATGAAGCTGCAGTTTCCATAGGGACACCTTTGGATCCTCAGTACATCCGGC
TAGAAATCCATTACAGCAATTTTGACTTGTTACCAGGTTTGATCGACAGCTCAGGGGTACGAATCTACTA
TACTCCAGAGCTACGGAAACATGATGTGGGGATTCTGCAAACAGGCATCTTCTCTTTCCCTTACGGCTTT
TGCAATTCCAGCCAGTTTGATGAAATGAACGGGATGGCGGTTCCAGATATGCATGTCTTTGCCTACTTGC
TTCACACCCACCTGGCTGGAAGAGGACTAAAAGCTGTTCAATACCGGAATGGTGAGCAGCTGGTGATCAT
CTGTGAGGACAATAAGTATGACTTCGGTCTGCAGGAGATACGGAACATGAAGGAAATCGTCATAGTCAAA
TCAGGGGATGAGGTCCTGGTCGAATGCAACTTTCAGACACTGGATCGGTCACAGAGTACTTTTGGTGGGC
CAAGCACCATGAATGAGATGTGTCTCACATTCCTCTTCTACTACCCTCGTAACAACATCTCCAGTTGTAT
GGGCTACCCTGATATTTTGCATGTTGTGCATGCACTCAAGCAGGAGGCCTCAGATGCATTGGAAGGAATG
ATGGCCATGGACTTTGTTGACTGGAACAATGAGACTGTCAAAATTGCAGAGCATGCAGCCAAGGAGGCAG
ATCAATTCGTCATCATTAAAACCATTGATGAACTCCAGAGAAACGAGACTGGTCTAGTCAGAGACATTAT
TATTCCAGAGCGGGCTGCCTGTCACAATCTTTCTGGACACCTCTCACTGCCGGGTCTGAAGGCTGCTGCA
AACCTTCGTTTGACTGCAGTACGCACTTCTGAGTCATCAACCACCAAAGAAACTGCTTCACTTCCTCTAC
TTTCTCTCACACAGCTGGTGTTTGCTTGGCTTATCTTGGCCTCTGAGTATGGGAAGTGA

>Fulmarus_glacialis No=33 length=602 name="Northern fulmar"
MMAVLLSRIKGMLFLLFLPCFCSGQPAPPPLRFSIFLDPSKMVYLRWDHDEQELMTFELQVHTTGWVAFG
FSSHGELPGSDIVIGGVFPNGSIYFSDCHVVDEATIEEDESQDYQLLSVTENETSTTMLFKRHLRTCDPN
DLDITVDTARLVAAFGTDDTVPFFKGQIFSKSLFLLRFRSPSTDPKIFFTYDLRLDNFAVPVEETKYACK
FIPLPMVKQKHHIYKFDPVITPHNITLVHHILIYGCGNASVLPSGIDDCYGANPDFSLCSQVLMGWAIGG
QTYQFPDEAAVSIGTPLDPQYIRLEIHYSNFDLLPGLIDSSGVRIYYTPELRKHDVGILQTGIFSFPYGF
CNSSQFDEMNGMAVPDMHVFAYLLHTHLAGRGLKAVQYRNGEQLVIICEDNKYDFGLQEIRNMKEIVIVK
SGDEVLVECNFQTLDRSQSTFGGPSTMNEMCLTFLFYYPRNNISSCMGYPDILHVVHALKQEASDALEGM
MAMDFVDWNNETVKIAEHAAKEADQFVIIKTIDELQRNETGLVRDIIIPERAACHNLSGHLSLPGLKAAA
NLRLTAVRTSESSTTKETASLPLLSLTQLVFAWLILASEYGK
```

Exon	NCBI Accession	Strand	Start	End
1	JJRN01045362.1	+	15184	15471
2	JJRN01045362.1	+	18496	18642
3	JJRN01045362.1	+	20207	20362
4	JJRN01045362.1	+	21579	21662
5	JJRN01045362.1	+	22851	23018
6	JJRN01045362.1	+	23969	24071
7	JJRN01045362.1	+	24860	24987
8	JJRN01045362.1	+	26800	26891
9	JJRN01045362.1	+	30094	30190
10	JJRN01045362.1	+	30407	30466
11	JJRN01045362.1	+	31438	31568
12	JJRN01045362.1	+	31843	31957
13	JJRN01045362.1	+	32686	32925

---

34. Great cormorant (*Phalacrocorax carbo*)

Exon	NCBI Accession	Strand	Start	End	
1	JMFI01047762.1	-	15503	15784	5-nt deletion, 1-nt deletion, 1 nonsense codon
2	JMFI01047762.1	-	11849	11994	1-nt deletion

3	JMFI01047762.1	-	10180	10341	
4	JMFI01047762.1	-	8900	8968	5' deletion (7 nt), 7-nt deletion
5	JMFI01047762.1	-	7543	7710	1 nonsense codon
6	JMFI01047762.1	-	6499	6601	1 nonsense codon
7	JMFI01047762.1	-	5561	5715	1 nonsense codon
8	JMFI01047762.1	-	3663	3754	
9	JMFI01047762.1	-	292	388	splice donor TC
10	JMFI01138480.1	-	3788	3847	1 nonsense codon, splice donor CT
11	JMFI01138480.1	-	2641	2771	
12	JMFI01138480.1	-	2247	2357	1 nonsense codon, 3' deletion (4 nt)
13	JMFI01138480.1	-	1258	1487	1-nt insertion, 11-nt deletion

35. Crested ibis (*Nipponia nippon*)

Exon	NCBI Accession	Strand	Start	End	
1	JMFH01007490.1	+	55645	55927	5-nt deletion
2	JMFH01007490.1	+	58934	59079	1 nonsense codon, 1-nt deletion
3	JMFH01007490.1	+	60657	60819	1-nt insertion
4	JMFH01007490.1	+	62038	62121	
5	JMFH01007490.1	+	63335	63502	
6	JMFH01007490.1	+	64450	64552	
7	JMFH01007490.1	+	65335	65501	
8	JMFH01007490.1	+	67310	67401	
9	JMFH01007490.1	+	70605	70701	
10	JMFH01007490.1	+	70918	70977	
11	JMFH01007490.1	+	71978	72108	
12	JMFH01007490.1	+	72396	72510	
13	JMFH01007490.1	+	73260	73495	4-nt deletion

36. Little egret (*Egretta garzetta*)

Exon	NCBI Accession	Strand	Start	End	
1	JJRC01034364.1	-	20167	20446	5-nt deletion, 1 nonsense codon
2	JJRC01034364.1	-	16999	17133	1 nonsense codon
3	JJRC01034364.1	-	15236	15397	splice acceptor TG, 1 nonsense codon, splice donor AT
4	JJRC01034364.1	-	13929	14012	splice acceptor CT
5	JJRC01034364.1	-	12629	12796	
6	JJRC01034364.1	-	11573	11675	
7	JJRC01034364.1	-	10628	10794	1 nonsense codon
8	JJRC01034364.1	-	8863	8954	
9	JJRC01034364.1	-	5585	5674	7-nt deletion

10	JJRC01034364.1	-	5309	5368	
11	JJRC01034364.1	-	4193	4335	13-nt insertion, 1-nt deletion, 1 nonsense codon, splice donor AT
12	JJRC01034364.1	-	3816	3930	1 nonsense codon
13	JJRC01034364.1	-	2845	3076	7-nt deletion, 1-nt deletion

---

37. Killdeer (*Charadrius vociferus*)

Exon	NCBI Accession	Strand	Start	End	
1	JMFX02000767.1	-	2694	2981	
2	JMFX02000805.1	+	70	216	
3	JMFX02000805.1	+	1779	1940	
4	JMFX02000805.1	+	3118	3201	
5	JMFX02000805.1	+	4394	4561	
6	JMFX02000805.1	+	5496	5598	
7					exon deletion
8	JMFX02000806.1	+	586	677	
9	JMFX02000806.1	+	3973	4069	
10	JMFX02000806.1	+	4284	4343	
11	JMFX02000806.1	+	5365	5495	
12	JMFX02000806.1	+	5771	5885	
13	JMFX02000806.1	+	6616	6855	

---

38. East African crowned crane (*Balearica pavonina gibbericeps*)

```
>Balearica_pavonina_gibbericeps No=38 length=1851 name="East African
crowned crane"
ATGATGGCAGTTCTCTTAAGAATCAAGGCTGTGCTCTTCCTCTTGTTCCTCCCATCCTTTTGTTCTGGTC
AGCCTGCACCTCCACTGCTGCATTTCTCCATCTTGCTGGAGCCTTCAAACATGGTCTACCTCCACTGGGA
CCATGACGAACAGGAGATGATGACATTTGAGCTGCAGGTCCATACAACTGGCTGGGTGGCATTTGGATTC
AGCCCTCATGGAGAGTTGCCTGGCTCTGACATTGTGATAGGAGGTGTCTTCCCAAATGGCAGCATCTACT
TCTCTGATTTTCATGTGGTAGATGAGGCAACCCTTGAGGAAGATGAGAGCCAGGACTACCAACTGCTGTC
AGTAACGGAAAATGAGACCTCCACCACCATGCTGTTCAAGCGCCACTTCCGGACATGTGACGTGAATGAC
CTGGATATCACAATGGACACAGCACGTCTTGTTACTGCTTTTGGCACTGATTACACAGTCCAATTCTTTA
AAGGCCAAAGATTTTCCAACTCTCTTTTCTTGATGAGGTACAGAGGCCCATCTGACCCAACTGATCCCAA
AATATTCTTCACCTATGACCTGAGGCTGGACAATTTTGCTGTTCCAGTTGAAGAAACCAAGTATGCCTGT
ACCTTTATCCCACTGCCCATGGTCAAGCAGAAACACCATATCTACAAGTTCGAACCTACAATAACACCCC
ACAACATAACGTTGGTTCATCATATTCTTGTTTACGCCTGTGGCAACGCCAGCATGTTACCCAGTGGCAT
AGATGATTGCTACGGAGCCAATCCAGATTTTTCCCTGTGCTCTCAGGTGCTCATGGGCTGGGCTGTTGGA
GGACAGTCTTACCAATTTCCAGATGAAGCTGCAGTTTCCATAGGGACACCTTGGGACCCTCAGTACATCC
GACTAGAAATCCATTACAGCAATTTTGATTTGTTACCAGGCTGGATCGACAGCTCAGGGATACGAATCTA
CTATACACCTGAGCTACGGAAATATGACGTGGGGGTTCTGCAAACAGGCATCTTCACTTTCCCCGTGCAT
TTCATTCCCCCTGGAGCAGAATCCTACAGATCTTATGGTCTTTGCAATTCCAGTCAGTTTGATGAAATGA
ATGGGATGCTGGTTCCAGATCTGCATGTCTTTGCCTACTTGCTTCACACTCATCTGTCTGGCAGAGGAGT
GAAAGCTGCTCAATACCGGAATGGTGAGCAGCTGAGGATCATCTGTGAGGACAATAAGTACGACTTCAGA
CTGCAAGAGATTCGAGACATTAAGGAAATCCTCATAATCAAGCCAGGGGATGAGATCCTGGTTGAATGCA
ACTTCCAGACATTGGATCGGTCAGGGATTACTTTTGGTGGGCCAAGCACCATGAATGAGATGTGTCTCAC
ATTCCTCTTGTACTACCCTCATAACAACATCTCCAGTTGTATGGGCTACCCTGACATTTTGTACATTGCA
```

CACATACTCAAGCAGGAGGCCTCAGATACAGTGGAAGGAATGATGGCCATGGACTATGTTGACTGGGACC
ATGATACTGTCAAAATTGCAGAGAAAGCAGCCAAGGAGGCAGATCAAGTAGTCATGATTAAAACCATTAC
TGAGCTCCAGAGAAATGAGAGTGGTCTAATCAGAGACATTAGCATTCCAGAGCAGGCTGCCTGTCACAAT
ATTTCTGGACACCTCTCACTGTCGGGTCTGAGATCCACTGCAAATGTTCGTTTGACCGCACTATGCACTT
CTGAGTCATCAACCACCAAAGAAACTGCTTCACTTCCTCTTCTTTCTCTCACACAGCTAGTGTTTGCTTG
GCTTACCTTGGCCTCTGAGTATAAGAAGTGA

>Balearica_pavonina_gibbericeps No=38 length=616 name="East African crowned crane"
MMAVLLRIKAVLFLLFLPSFCSGQPAPPLLHFSILLEPSNMVYLHWDHDEQEMMTFELQVHTTGWVAFGF
SPHGELPGSDIVIGGVFPNGSIYFSDFHVVDEATLEEDESQDYQLLSVTENETSTTMLFKRHFRTCDVND
LDITMDTARLVTAFGTDYTVQFFKGQRFSNSLFLMRYRGPSDPTDPKIFFTYDLRLDNFAVPVEETKYAC
TFIPLPMVKQKHHIYKFEPTITPHNITLVHHILVYACGNASMLPSGIDDCYGANPDFSLCSQVLMGWAVG
GQSYQFPDEAAVSIGTPWDPQYIRLEIHYSNFDLLPGWIDSSGIRIYYTPELRKYDVGVLQTGIFTFPVH
FIPPGAESYRSYGLCNSSQFDEMNGMLVPDLHVFAYLLHTHLSGRGVKAAQYRNGEQLRIICEDNKYDFR
LQEIRDIKEILIIKPGDEILVECNFQTLDRSGITFGGPSTMNEMCLTFLLYYPHNNISSCMGYPDILYIA
HILKQEASDTVEGMMAMDYVDWDHDTVKIAEKAAKEADQVVMIKTITELQRNESGLIRDISIPEQAACHN
ISGHLSLSGLRSTANVRLTALCTSESSTTKETASLPLLSLTQLVFAWLTLASEYKK

Exon	NCBI Accession	Strand	Start	End
1	JJRR01082268.1	+	2012	2296
2	JJRR01082270.1	+	246	392
3	JJRR01082270.1	+	1031	1192
4	JJRR01082270.1	+	2395	2478
5	JJRR01082270.1	+	3645	3812
6	JJRR01082270.1	+	4731	4833
7	JJRR01082270.1	+	5508	5674
8	JJRR01082270.1	+	7478	7569
9	JJRR01082270.1	+	10001	10097
10	JJRR01082270.1	+	10311	10370
11	JJRR01082270.1	+	11277	11407
12	JJRR01082271.1	+	7	121
13	JJRR01082271.1	+	859	1098

---

39. Hoatzin (*Opisthocomus hoazin*)

>Opisthocomus_hoazin No=39 length=1869 name="Hoatzin"
ATGGCAGTTCTCCTCTCAACAATGAAGGGTATGCTCTTTCTCTTGCTCCTCCCATGCTTTTGTTTTGGTC
AGCCTGCACCTCCACTGCTGCGTTTCTCCATCTTCCTGGAGCCTGCAAACAGGGTCTACCTCCACTGGGA
CCATGACGAAGAGGAGATGATGACATTTGAGCTGCAGGTCCATACAACTGGCTGGGTGGCATTTGGATTC
AGCCCTGATGGAGAGTTACCTGGATCTGACATTGTGATAGGAGGTGTCTTCCCAAATGGCAGCATCTACT
TCTCTGATTGTCACGTGGTAGATCAAGCAACCCTTGAGGAAGATGAGAGCCAGGACTACCAACTGCTGTC
AGTGACAGAGAACGAGACCTCCACCACCATGCTGTTCAAACGCCACCTCCAAACATGTGACCCTAATGAC
CTGGATATCACAACGGACACAGCACGCCTTGTTACTGCATTTGGCACTGACGACACAGTCCAATTCTTTA
AAGGCCAAAGATTTTCCAGATCTCTTCTCCTGATGAGGTACAGAGGCCCATCAGACTCAACTGACCCCAA
AATATTCTTCACCTATGACCTGAGGCTGGACAATTTTGCTGTCCCAGCTGAAGAAACCAAGTATGCCTGC
ACCTTTATTCCACTGCCCAGGGTCAAGCAGAAACACCATATCTACAAGTTTGAACCTGTAATAACACCCC
ACAACATAACTTTGGTTCATCATATTCTTGTTTATGCCTGTGGAAACGCCAGCGTGTTACCCAGCAGCAT
AGACGATTGCTATGGACCCAATCCGGATTTTGCCCTGTGCTCTCAGGTGCTCGTGGGCTGGGCTGTGGGA
GGAGAGTCTTATCAATATCCAGAGGAAGCTGCAGTTTCCATAGGGACACCTTGGGACCCTCAGTATGTCC
GACTAGAAATCCACTACAGCAATTTTGACTTGTTACCAGGCTTGATCGACAGCTCAGGGATACGACTCTA

```
CTATACGCCAGAGCTGCGGAAATATGATGTGGGTGTTCTGCAAACAGGCATCTTCACTTTCCCTGTGCAT
TTCATTCCTCCTGGTGCAGAATCCTACAGATCTTACGGCCTTTGCAACTCCAGCCAGTTTGATGAAATGA
ATGGGATGCTGGTTCCAGATCTGCATGTCTTTGCCTACTTGCTTCACACCCACCTATCTGGCAGAGGAGT
GAAAGCTGCTCAATACCGGAATGGTGAGCAGCTGGGGATCATCTGCGAGGACAATAAGTACGACTTCAGC
CTGCAGGAGGTTCGGGACATGAAGGAAACCCTCACAGTCAGACCGGGGGATGAGATCCTGGTTGAATGCA
ACTTTCAGACACTGGATCGGTCAGGGATTACTTATGGTGGGCCAAGCACCATGAATGAGATGTGTCTCAC
ATTCCTTTTCTACTACCCTCGTAACAACATCTCCAGTTGTATGGGCTACCCTGATATTTTGTACATTGCG
CATGCACTCAAGCAGGAGGCCTCAGATGCAGTGGAAGCAATCATGGCCTTGGACTTTGTTGACTGGGATA
ACGAGACTGTCAAAATTGCAGAGAAAGTAGCCAAGGAGGCAGACCAAGTAGTCATGATTAAAACCATTAA
TGAACTCCAGAGAAATGAGACTGGTCTAATCAGAGACATCAGTATTCCAGAGTGGTCTCCCTGTCACAAT
ATTTCTGGACACCTCTCAATTTCTGGGCACCTCTCACCGTCAGGACTGAGGGCTGCTACAAACCCTTGTT
TGACTGCAGTATGCACTTCTGAGTCATCAACCCTCAGAGAAACTGCTTCACCTCCTCTGCTTCCTCTCAC
TCAGCTCGTGTTTTCTTGGCTTGTCTTGGCCTCTGAGTATGGGAAGTGA
```

```
>Opisthocomus_hoazin No=39 length=622 name="Hoatzin"
MAVLLSTMKGMLFLLLLPCFCFGQPAPPLLRFSIFLEPANRVYLHWDHDEEEMMTFELQVHTTGWVAFGF
SPDGELPGSDIVIGGVFPNGSIYFSDCHVVDQATLEEDESQDYQLLSVTENETSTTMLFKRHLQTCDPND
LDITTDTARLVTAFGTDDTVQFFKGQRFSRSLLLMRYRGPSDSTDPKIFFTYDLRLDNFAVPAEETKYAC
TFIPLPRVKQKHHIYKFEPVITPHNITLVHHILVYACGNASVLPSSIDDCYGPNPDFALCSQVLVGWAVG
GESYQYPEEAAVSIGTPWDPQYVRLEIHYSNFDLLPGLIDSSGIRLYYTPELRKYDVGVLQTGIFTFPVH
FIPPGAESYRSYGLCNSSQFDEMNGMLVPDLHVFAYLLHTHLSGRGVKAAQYRNGEQLGIICEDNKYDFS
LQEVRDMKETLTVRPGDEILVECNFQTLDRSGITYGGPSTMNEMCLTFLFYYPRNNISSCMGYPDILYIA
HALKQEASDAVEAIMALDFVDWDNETVKIAEKVAKEADQVVMIKTINELQRNETGLIRDISIPEWSPCHN
ISGHLSISGHLSPSGLRAATNPCLTAVCTSESSTLRETASPPLLPLTQLVFSWLVLASEYGK
```

Exon	NCBI Accession	Strand	Start	End
1	JMFL01069564.1	-	4958	5242
2	JMFL01069564.1	-	1971	2117
3	JMFL01038297.1	+	401	562
4	JMFL01038297.1	+	1769	1852
5	JMFL01038297.1	+	2527	2694
6	JMFL01069562.1	-	40864	40966
7	JMFL01069562.1	-	39928	40094
8	JMFL01069562.1	-	39052	39143
9	JMFL01069562.1	-	35673	35769
10	JMFL01069562.1	-	35397	35456
11	JMFL01069562.1	-	34147	34277
12	JMFL01069562.1	-	33744	33858
13	JMFL01069562.1	-	32732	32989

---

40. Red-crested turaco (*Tauraco erythrolophus*)

```
>Tauraco_erythrolophus No=40 length=1842 name="Red-crested turaco"
ATGATGGCAGTTCTCTTCTCAAGAATCAAGGGTATGCTCTTACTCTTGTTCTTCTTATGCTTTTGTTCTA
GTCAGCCTGCACCTCCACTGCTGCGTTTCTCCATCTTCCTGGATCCTTCAAACATGGTCTATCTCCGCTG
GGACCATGACGAACAGGAGCTGATGACGTTTGAGCTTCAGGTCCACACAACTGGCTGGGTGGCATTTGGA
TTCAGCCCTCATGGAGAGTTGCCTGGGTCTGACATTGTGATAGGAGGTGTCTTCCCAAATGGCAGTATCT
ACTTCTCTGATTGTCATGTGGCAGATGAGGCAACCCTTGAGGAAGATGAGAGCCAGGACTACCAACTGCT
GTCAGTGACAGAGAATGAGACCTCTACCACAATGCTATTCAAACGCCACCTCCGGACATGTGACCCAAAT
GACCTGGATATCACAGTGGACACAGCACGCCTCGTTACTGCGTTTGGCACTGATGACACAACCCAATTCT
TTAAAGGCCAAAGATTTTCCAAATCTCTTTTCTTGATGAGGTACAGAGGCCCATCTGACTCAACTAACCC
```

CAAAATATTCTTCACCTATGACCTGAGGCTGGACAATTTTGCTGTTCCAGTTGAAGAAACAAAGTATGCC
TGTACCTTTATCCCATTGCCCATGGTCAAGCAGAAACACCATATCTACAAGTTTGAGCCTGTAATAACAC
CCCACAACATAACCTTAGTTCATCATATTCTTGTTTATGCCTGTGGCAACGCCAGTGTGTTACCCAGTGG
CGTAGACGATTGTTATGGAGCCAATCCAGATTTTGCCCTGTGCTCGCAGGTGCTTGCGGGCTGGGCTGTT
GGAGGAGAGTCTTACCAGTTTCCAGATGAAGCTGCAATTTCCATAGGGACACCTTTGGATCCTCAGTATG
TCCGACTAGAAATCCATTACAGCAATTTTGACTTGTTACCAGGCTTGATTGACAGCTCGGGGGTACGAAT
CTACTATACGCCGGAGCTACGGAAATATGATGCGGGGGTCCTGCAAACAGGAGTCTTCACTTTCCCTGTG
CATTTCATTCCTCCTGGAGCAGAATCCTACAGATCTTATGGCCTTTGCAATTCCAGCCAGTTTGATGAAA
TTAATGGGATGCTGGTTCCAGATCTGCATGTCTTTGCCTACATGCTTCACACCCACCTGTCTGGCAGAGG
ATTGAAAGCTGCTCAATACCGGAATGGTGAGCAGTTGAGGATCATCTGTGAGGACAATAAGTATGACTTC
AAACTGCAGGAGATTCGGGATATGAAGGAAATCATCATAATCAAACCAGGGGATGAGATCCTGGTTGAAT
GCAACTTTCAGACGCTGGATCGGTCAGGGATTACTTTTGGTGGGCCAAGCACCATGAACGAGATGTGTCT
CACATTCCTCTTCTACTACCCTCGTAACAACATCTCCAGTTGTATGGGCTACCCTGACATTTTGTACATT
GCTCAGGCACTCAAGCAGGAGGCCTCAGATGCAGTGGAAGGAATGATGGCCATGGACTTTGTTGACTGGG
ACAATGAGACTGTCAAAATTGCAGAGAGAGCAGCCAAAGAGGCAGATCAAGTAGTCATGATTAAAACCAT
TAATGAACTCCAGAGAAATGAGAGTGGTCTAATCAGAGACATTAGTATTCCAGAGCGGGCTACCTGTCAC
AATATTTCTGGACACCTCTCACTGTCAGGTCTGAGGGCGACTGCAAACCTTTGTTTGACTGCTGTACACA
CTCCTGAGTCATCAACCACCAAAGAAACTGCTTCGTTTCCTCTTCTTTCCCTCACACAGCTGGTGTTTGC
TTGGCTTTTATTGGCCTCTTGA

>Tauraco_erythrolophus No=40 length=613 name="Red-crested turaco"
MMAVLFSRIKGMLLLLFFLCFCSSQPAPPLLRFSIFLDPSNMVYLRWDHDEQELMTFELQVHTTGWVAFG
FSPHGELPGSDIVIGGVFPNGSIYFSDCHVADEATLEEDESQDYQLLSVTENETSTTMLFKRHLRTCDPN
DLDITVDTARLVTAFGTDDTTQFFKGQRFSKSLFLMRYRGPSDSTNPKIFFTYDLRLDNFAVPVEETKYA
CTFIPLPMVKQKHHIYKFEPVITPHNITLVHHILVYACGNASVLPSGVDDCYGANPDFALCSQVLAGWAV
GGESYQFPDEAAISIGTPLDPQYVRLEIHYSNFDLLPGLIDSSGVRIYYTPELRKYDAGVLQTGVFTFPV
HFIPPGAESYRSYGLCNSSQFDEINGMLVPDLHVFAYMLHTHLSGRGLKAAQYRNGEQLRIICEDNKYDF
KLQEIRDMKEIIIIKPGDEILVECNFQTLDRSGITFGGPSTMNEMCLTFLFYYPRNNISSCMGYPDILYI
AQALKQEASDAVEGMMAMDFVDWDNETVKIAERAAKEADQVVMIKTINELQRNESGLIRDISIPERATCH
NISGHLSLSGLRATANLCLTAVHTPESSTTKETASFPLLSLTQLVFAWLLLAS

Exon	NCBI Accession	Strand	Start	End
1	JNOY01030931.1	-	891	1178
2	JNOY01087376.1	-	1072	1218
3	JNOY01132768.1	+	567	728
4	JNOY01022207.1	+	238	321
5	JNOY01022207.1	+	1515	1682
6	JNOY01022207.1	+	2221	2323
7	JNOY01022207.1	+	2987	3153
8	JNOY01022207.1	+	4230	4321
9	JNOY01022207.1	+	7279	7375
10	JNOY01022207.1	+	7592	7651
11	JNOY01022207.1	+	8665	8795
12	JNOY01022207.1	+	9080	9194
13	JNOY01022207.1	+	9952	10179

41. MacQueen's bustard (*Chlamydotis undulata macqueenii*)

>Chlamydotis_undulata_macqueenii No=41 length=1854 name="MacQueen's bustard"
ATGAAGGCAGTTGTCTTCTCAAGAATTAAGAGTATGCTCTTCCTCTTGTTTCTCCCATGGTTTTGTTCTG

GTCAGACTGCACATCCACTGCTGCGTCACTCCATCTTCCTGGATCCTTCAAACATGGTCTACTTCCGCTG
GGACCATGATGAACAGGAGCTGATGACGTTTGAGCTGCAGGTCCATACAGCTGGCTGGGTGGCTTTTGGA
TTCAGCCCTTATGGAGAGTTGCCTGGATCTGACATTGTGATAGGAGGTGCCTTCCCAAATGGCAGCATCT
ACTTCTCTGATTGCCATGTGGTAGACGAGTCAACCCTTGAGGAAGATGAGAGCCAGGACTACCAGTTGCT
GTCACTGACAGAGAATGAGACCTCCACCACCATGCTGTTCAAACGCTACTTCCAGACATGTGACCCAAAT
GACTTAGATATCACAATGGACACAGCACGCCTCATAACTGCCTTTGGTACTGATGACACAGTCCAATTCT
TTAAAGGCCAAAGATTTTCCAAGTCTCTTTTCTTGATGAGGTACAGAGGCCCATCTGACTCAACTGATCC
AAAAATATTCCTCACCTATGACCTGACGCTGGACAATTTTGCTGTTCCAGTTGAAGAAACCAAGTATGCT
TGTACCTTTATCCCACTGCCCATGGTCAAGCAGAAACACCATATCTACAAGTTTGAACCTGTAATAACAC
CCCACAACATAACCTTGGTTCATCACATTCTTGTTTATGCCTGTGGCAATGCCAGCATGTTACCCAGTGG
CATAGATGACTGCTATGGAGCCAATCCAGATTTTGCCCTGTGCTCTCAGGTGATTGTAGGCTGGGCTGTT
GGAGGAAAGTCTTATCAATTTCCAGATGAAGCTGCAATTTCCATAGGGGCACCTCTGGATCCTCAGTATG
TCCGACTAGAAATCCATTACAGCAATTTTGACTTGTTACCAGGTTTGATCGACAGCTCAGGGATACGAAT
CTACTATACTCCAGAGCTACGGAAATATGATGTGGGGATTCTGCAAACAGGCATCTTCACTTTTCCTGTG
CATTTCATTCCTCCTGGAGCAGAATCCTACAGATCTTACGGCCTTTGCAATTCCAGCCAGTTTGACGAAA
TGAATGGGATGCTGGTTCCAGATCTGCATGTCTTTGCCTACTTGCTTCACACCCACCTGTCTGGCAGAGG
AGTGAAAGCTGTTCAATACCGGAATGGTGAACAGCTGAGGATCATCTGTGAGGATAATAAGTATGACTTC
AGACTACAGGAGATTCGGGATATGAAGGAAATCCTCATAATCAAACCAGGGGATGAGATCCTGGTCGAAT
GCAACTTTCAGACACTGGATCGCTTAAAGATTACTTTTGGTGGGCCAAGCACCATGAACGAGATGTGTCT
CACATTTCTCTTCTACTACCCTCGTAACAACATCTCCAGTTGTATGGGCTACCCTGACATTTTGTACATT
GCACACGTACTCAAGCAGGAGGCGTCAGATGCAGTGGAAGGAATGATGGCCATGGACTTTGTTGACTGGG
ACAACGATACTGTCAAAATTGCAGAGAAAGCAGCCAAGGAGGCAGATCAAGTAGTCATGATTAAAACCAT
TAATGAACTCCAGAGAAATGAGAGTGGTCTAATCAGAGACATTAGTATGCCAGAGCGGGCTGCCTGTCAC
AATATTTCTGGACACTTCCCACTGTCAGGTCTGAGGGCCACTGCAAACCTTCGTTTGACTGCAGTATGCA
CTTCTGAATCATCAACCAACAAAAGAACTACTTCACTGCCTCTTCTTTCTCTCACACAGCTGGTGTTTGC
TTGGCTTATCTTGGCCTCTGTGTATGGGAAATGA

>Chlamydotis_undulata_macqueenii No=41 length=617 name="MacQueen's bustard"
MKAVVFSRIKSMLFLLFLPWFCSGQTAHPLLRHSIFLDPSNMVYFRWDHDEQELMTFELQVHTAGWVAFG
FSPYGELPGSDIVIGGAFPNGSIYFSDCHVVDESTLEEDESQDYQLLSLTENETSTTMLFKRYFQTCDPN
DLDITMDTARLITAFGTDDTVQFFKGQRFSKSLFLMRYRGPSDSTDPKIFLTYDLTLDNFAVPVEETKYA
CTFIPLPMVKQKHHIYKFEPVITPHNITLVHHILVYACGNASMLPSGIDDCYGANPDFALCSQVIVGWAV
GGKSYQFPDEAAISIGAPLDPQYVRLEIHYSNFDLLPGLIDSSGIRIYYTPELRKYDVGILQTGIFTFPV
HFIPPGAESYRSYGLCNSSQFDEMNGMLVPDLHVFAYLLHTHLSGRGVKAVQYRNGEQLRIICEDNKYDF
RLQEIRDMKEILIIKPGDEILVECNFQTLDRLKITFGGPSTMNEMCLTFLFYYPRNNISSCMGYPDILYI
AHVLKQEASDAVEGMMAMDFVDWDNDTVKIAEKAAKEADQVVMIKTINELQRNESGLIRDISMPERAACH
NISGHFPLSGLRATANLRLTAVCTSESSTNKRTTSLPLLSLTQLVFAWLILASVYGK

Exon	NCBI Accession	Strand	Start	End
1	JMFJ01015205.1	-	15272	15559
2	JMFJ01015205.1	-	11272	11418
3	JMFJ01015205.1	-	9555	9716
4	JMFJ01015205.1	-	8295	8378
5	JMFJ01015205.1	-	6921	7088
6	JMFJ01015205.1	-	6105	6207
7	JMFJ01015205.1	-	5170	5336
8	JMFJ01015205.1	-	3299	3390
9	JMFJ01015205.1	-	455	551
10	JMFJ01015205.1	-	165	224
11	JMFJ01097322.1	+	671	801
12	JMFJ01097322.1	+	1090	1204
13	JMFJ01097322.1	+	1937	2176

42. Common cuckoo (*Cuculus canorus*)

```
>Cuculus_canorus No=42 length=1854 name="Common cuckoo"
ATGATGGCAGTTCTCTTCTCAAGAATCAAAGGTATGCTCTTCCTTTTATTCCTCCCACGCTTTTGTTCTG
GTCAGCCTGCACTGCCACTGCTGCGTTTCTCCACCTTCCTGGATCCTTCAAACATGGTCTATCTCCGCTG
GGACCATGATGAACAGGAGCTGATGACGTTTGAGCTGCATGTCCACACAACAGGCTGGGTGGCATTTGGA
CTCAGCCTTCACGGAGAGTGGCCTGGATCAGACGTTGTGATAGGAGGTGTCTTCCCAAATGGCAGCATCT
ACTTCTCTGACTGTCATATGGTAGATGAAGCAATCCTTGAGGAAGATGACAGCCAGGACTACCAATTGCT
GTCAGTGACAGAGAATGAGACATGCACCACCATGCTGTTCAGACGCCACTTCAGGACATGTGACCCAAAT
GACCTTGATATTACAGTGGACACAGCACGTCTCATTACTGCTTTTGGTACAGATGACACCATCCAGTTTG
TTAAAGGCCAAAGATTTTCCAAATCTCTTTCCTTGATGAGGTACAGAGGCCCATCTGACTTAACTGACCC
CAAAATATTCTTCACTTATGACCTGAGGCTGGACAATTTCCCTGTTCCAGTTGAAGAAACCAAATATGCC
TGTACCTTTATCCCACTGCCCATAGTCAAGCAGAAGCACCATATCTACAAGTTTGAACCTGTAATAACAC
CACACAACACAGCCTTGGTTCACCATATTCTTGTTTATGCCTGTGGCAATGCCAGTGTGTTGCCCAGTGG
CATTGATGATTGCTATGGAGCCAACCCAGATTTTGCCCTGTGCTCTCAGGTGATTGCAGGCTGGGCAGTT
GGAGGAGAGTCTTATCAATTTCCAGATGAAGCTGCAATTTCCATAGGGACACCCTGGGACCCTCAGTATG
TCCGACTAGAAATCCATTACAGCAATTTTGACTTGTTACCAGGTTTGATCGACAGCTCGGGGCTACAAAT
CTACTACACTCCAGAGCTACGGAAATATGATGTGGGGGTTCTGCAAACAGGCATCTTCACTTTCCCTGTG
CATTTCATTCCTCCTGGAGCAGAATCCTACAGATCTTATGGCCTTTGCAATTCCAGCCAGTTTGACGAAA
TGAATGGGATGCTTGTTCCTGATATGCATGTCTTTGCCTACTTGCTTCACACCCACCTGTCTGGCAGAGG
AGTGAAAACTGTTCAATACCGGAATGGTAAGCAGCTGAACATCATCTGTGAAGACAATAAATATGACTTT
GGACTTCAGGAATTTCGAGACATGAAGGAAATCCTCATAATCAAACCAGGGGATGAAATCCTGGTTGAAT
GCAATTTTCGAACACTGGATCGGTCAGAGATTACTTTTGGTGGGCTAAGCACAATGAATGAGATGTGTCT
CACATTCCTCTTCTACTATCCTCGTAACAACATCTCCAGTTGTATGAGCTACCCTGACATTTTGTACATT
GCACATGAACTCAAGCAAGAGGCCTCAGATGCAGTAGAAGGAATGATGGCCATGTACCTTGTTGACTGGG
ACAATGATACTATCAAAACTGCAGAGAAAGCAGCCAAGGAAGCAGATCAAGTAGTCACGATTAAGACCAT
TAATGAACTCCACAGAAATGAGAGTGGTCTAATCAGAGACATAAGTATTCCAGAGAGGGCTGCCTGTCAC
AATATTTCTGGACACCTCTCACTATCAGGTCCAAGGGCCTCTGCAAACCTTCGTTTGACCGCAGCATGCA
CTTTGGAGTCACCAGCCACCAAAGGAACTGCTTCACTTCCTCTTCTTTCTCTCACACAGCTGGTGTTTGT
TTGGCTTATCTTGACTTCTGAGTATGGACAGTGA

>Cuculus_canorus No=42 length=617 name="Common cuckoo"
MMAVLFSRIKGMLFLLFLPRFCSGQPALPLLRFSTFLDPSNMVYLRWDHDEQELMTFELHVHTTGWVAFG
LSLHGEWPGSDVVIGGVFPNGSIYFSDCHMVDEAILEEDDSQDYQLLSVTENETCTTMLFRRHFRTCDPN
DLDITVDTARLITAFGTDDTIQFVKGQRFSKSLSLMRYRGPSDLTDPKIFFTYDLRLDNFPVPVEETKYA
CTFIPLPIVKQKHHIYKFEPVITPHNTALVHHILVYACGNASVLPSGIDDCYGANPDFALCSQVIAGWAV
GGESYQFPDEAAISIGTPWDPQYVRLEIHYSNFDLLPGLIDSSGLQIYYTPELRKYDVGVLQTGIFTFPV
HFIPPGAESYRSYGLCNSSQFDEMNGMLVPDMHVFAYLLHTHLSGRGVKTVQYRNGKQLNIICEDNKYDF
GLQEFRDMKEILIIKPGDEILVECNFRTLDRSEITFGGLSTMNEMCLTFLFYYPRNNISSCMSYPDILYI
AHELKQEASDAVEGMMAMYLVDWDNDTIKTAEKAAKEADQVVTIKTINELHRNESGLIRDISIPERAACH
NISGHLSLSGPRASANLRLTAACTLESPATKGTASLPLLSLTQLVFVWLILTSEYGQ
```

Exon	NCBI Accession	Strand	Start	End
1	JNOX01069217.1	+	39724	40011
2	JNOX01069217.1	+	43187	43333
3	JNOX01069217.1	+	44863	45024
4	JNOX01069217.1	+	45938	46021
5	JNOX01069217.1	+	47136	47303
6	JNOX01069217.1	+	48243	48345
7	JNOX01069217.1	+	49962	50128
8	JNOX01069217.1	+	51900	51991
9	JNOX01069217.1	+	56602	56698
10	JNOX01069217.1	+	56912	56971

11	JNOX01069217.1	+	57730	57860	
12	JNOX01069218.1	+	<1	83	5' partial (32-nt; recovered from WGS data)
13	JNOX01069218.1	+	832	1071	

---

43. Chuck-will's-widow (*Caprimulgus carolinensis*)

```
>Caprimulgus_carolinensis No=43 length=1854 name="Chuck-will's-widow"
ATGATGGCAGTTGTCTTCTCAAGGATCAAGGGTATTCTCTTCCTCTTGTTTCTCCCATGCTTTTGTTCTG
GTCAGCCTGCACCTCCACTTCTGCGTTTCTCCATCTTCCTGGATCCTTCAAACATGGTCTACCTCCGCTG
GGACCATGACGAACAGGAGCTGATGACGTTTGAGCTGCAGGTCCATACAACTGGCTGGGTGGCATTTGGA
TTCAGCCCTCATGGAGAGTTGCCTGGATCTGACATTGTGATAGGAGGCATCTTCCCAAATGGCAGCATCT
ACTTCTCTGACTGTCATGTGGTAGATGAGGCAACCCTTGAAGAAGATGAGAGTCAGGACTACCAACTGCT
GTCAGTAACAGAGAATGAGACCTCCACTACGATGCTGTTCAAACGCCACCTCCGGACATGTGACCCAGAT
GACCAGGACATCACAATGGACACAGCACGCCTCATTACTGCACTTGGCACTGATGACACAGTCCAATTCT
TTCAAGGCCAAAGATTTTCCAAATCTCTTTTCTTGATGAGGTACAGAGGCCCATCTGACTCAACTGATCC
CAAAATATTCTTCACCTATGACTTGAGGCTAGACAATTTTGCTGTTCCAGCTGAAGAAACCAAGTATGCC
TGTACCTTTATCCCACTGCCCATGGTCAAGCAGAAACACCATATCTACAAGTTCGAACCTGTAATAACAC
CGCACAACATAACCTTGGTTCATCACATTCTCGTTTATGCCTGTGGCAACGCCAGTGTGCTGCCCACTGG
CATAGATGATTGCTATGGAGCCAATCCAGACTTTGCCCTCTGCTCTCAGGTGTTTGTGGGCTGGGCTGTT
GGAGGAGAGTCTTACCAATTTCCAGATGAAGCTGCAATTTCCATAGGGACACCTTGGGATCCTCAGTACG
TCCGACTAGAAATCCATTACAGCAATTTTGACTTGTTACCAGGTTTGATCGACAGCTCAGGAGTACGAAT
TTACTATACACCAGAGCTAAGGAAACATGATGTGGGGGTACTGCAAACAGGCATCTTCACTTTCCCTGTG
CATTTCATTCCTCCTGGAGCAGAATCCTACAGATCTTATGGCTTCTGCAATTCCAGCCAGTTTGATGAAA
TGAATGGGATGCTGGTTCCGGATCTACATGTCTTTGCCTACTTGCTTCACACCCACCTTTCTGGCAGAGG
AGTGAAAGCTGCTCAGTACCGGAATGGTAAGCAGCTGGGTATACTCTGTGAGGACAATAAGTATGACTTC
AGACTGCAGGAGATTCGGGACATGAAGGAAATCCTCATAATCAAACCGGGGGATGAGATCCTGGTTGAAT
GCAACTTTCAGACACTGGATCGGTCAGAGATTACTTTTGGTGGGCCAAGCACCATGAATGAAATGTGTCT
CACATTCCTCTTCTACTACCCTCGTAACAACATCTCCAGTTGTATGGGCTACCCTGACATTTTGTATGTT
GCGCATGTACTGAAGCAGGAGGCCTCAGATGCAGCGGAAGGAATGATGGCCATGGAATTTGTTGACTGGG
ACAATGAGACTGTCAGAATTGCAGAGAAAGCAGCCAAGGAGGCAGATCAAGTAGTAATGATTAAAACCAT
TAACGAACTCCAGAGAAATGAGACTGGACTAATCAGAGACATCAGTATTCCAGAGCGGGCTGCTTGTCAC
AATATTTCTGGATATCTCTCACTGTCACATCTGAGGGCCACTGCAAATCTCCGTTTGACCGCAGTATGTG
CTTCTGAGTCATCAACCACTAAAGAAACTGCTTCACTTCCCCTTCTTTATCTCACACAGCTGGTGTTTGC
TTGGCTTATCTTGGCCCCTGAGCATGGGAAATGA
```

```
>Caprimulgus_carolinensis No=43 length=617 name="Chuck-will's-widow"
MMAVVFSRIKGILFLLFLPCFCSGQPAPPLLRFSIFLDPSNMVYLRWDHDEQELMTFELQVHTTGWVAFG
FSPHGELPGSDIVIGGIFPNGSIYFSDCHVVDEATLEEDESQDYQLLSVTENETSTTMLFKRHLRTCDPD
DQDITMDTARLITALGTDDTVQFFQGQRFSKSLFLMRYRGPSDSTDPKIFFTYDLRLDNFAVPAEETKYA
CTFIPLPMVKQKHHIYKFEPVITPHNITLVHHILVYACGNASVLPTGIDDCYGANPDFALCSQVFVGWAV
GGESYQFPDEAAISIGTPWDPQYVRLEIHYSNFDLLPGLIDSSGVRIYYTPELRKHDVGVLQTGIFTFPV
HFIPPGAESYRSYGFCNSSQFDEMNGMLVPDLHVFAYLLHTHLSGRGVKAAQYRNGKQLGILCEDNKYDF
RLQEIRDMKEILIIKPGDEILVECNFQTLDRSEITFGGPSTMNEMCLTFLFYYPRNNISSCMGYPDILYV
AHVLKQEASDAAEGMMAMEFVDWDNETVRIAEKAAKEADQVVMIKTINELQRNETGLIRDISIPERAACH
NISGYLSLSHLRATANLRLTAVCASESSTTKETASLPLLYLTQLVFAWLILAPEHGK
```

Exon	NCBI Accession	Strand	Start	End
1	JMFU01059780.1	-	10639	10926
2	JMFU01059780.1	-	7454	7600
3	JMFU01059780.1	-	5758	5919
4	JMFU01059780.1	-	4496	4579
5	JMFU01059780.1	-	3156	3323
6	JMFU01059780.1	-	2092	2194

7	JMFU01059780.1	-	1136	1302
8	JMFU01059779.1	-	16547	16638
9	JMFU01059779.1	-	15600	15696
10	JMFU01059779.1	-	15320	15379
11	JMFU01059779.1	-	14194	14324
12	JMFU01059779.1	-	13809	13923
13	JMFU01059779.1	-	12822	13061

---

44. Anna's hummingbird (*Calypte anna*)

Exon	NCBI Accession	Strand	Start	End	
1	JJRV01124598.1	-	37141	37431	
2					exon deletion
3					exon deletion
4	JJRV01124598.1	-	33905	33988	
5	JJRV01124598.1	-	32551	32718	
6	JJRV01124598.1	-	31520	31622	
7	JJRV01124598.1	-	30696	30854	8-nt deletion
8	JJRV01124598.1	-	30252	30343	
9	JJRV01124598.1	-	28000	28096	
10	JJRV01124598.1	-	27739	27798	
11	JJRV01124598.1	-	26641	26771	
12	JJRV01124598.1	-	26307	26362	3' deletion (59 nt)
13					exon deletion

---

45. Flamingo (*Phoenicopterus ruber ruber*)

```
>Phoenicopterus_ruber_ruber No=45 length=1854 name="Flamingo"
ATGATGGCAGTTCTCTTCTCGAGAATCAAGGGTACGCTCTTCCTCTTGTTCCTCCCATGCTTTTGTTCTG
GTCAGCCTGCACCTCCACCGCTGCGTTTCTCCATCTTCCTGGATCCTTCAAACATGGTCTACCTCCGCTG
GGACCACGATGAACAGGAGCTGATGACGTTTGAGCTGCAGGTCCATACAACTGGCTGGGTGGCATTTGGA
TTCAGCCCTCATGGAGAGTTGCCTGGATCTGACATTGTGATAGGAGGTGTCTTCCCAAATGGCAGCATCT
ACTTCTCTGATTGTCATGTGGTAGATGAGGCAACCCTTGAGGAAGACGAGAGCCAGGACTACCAACTGCT
GTCAGTGACGGAGAATGAGACCTCCACCACTATGCTGTTCAAACGCCACCTCCGGACGTGCGACCCAAAT
GACCTGGATATCTCAATGGACACAGCACGCCTCGTTACTGCATTTGGCACTGATGACACAGTCCAATTCT
TTAAAGGCCACAGATTTTACAAATCTCTTTTCTTGATGAACTACAGAGGCCCATATGACTCAACTGACCC
CAAAATATTCTCTACCTATGACCTGAGGCTGGACAATTTTGCTGTTCCAGCTGAAGAAACCAAGTATGCC
TGTACCTTTATCCCACTGCCCATGGTCAAGCAGAAACACCATATCTACAAGTTTGAACCTGTAATAACAC
CCCACAACATAACCTTGGTTCATCATATTCTTGTTTACGCCTGTGGCAACGCCAGCGTGTTACCCAGTGG
CATAGATGATTGCTATGGAGCCGATCCAGATTTTGCCCTGTGCTCTCAGGTGCTTGTGGGCTGGGCTGTT
GGAGGAGAGTCTTACCAATTTCCAGATGAAGCTGCAGTTTCCATAGGGACACCTTGGGACCCTCAGTATG
TCCGACTAGAAATCCATTACAGCAATTTTGACTTGTTAGCAGGCTTGATCGACAGCTCGGGGGTACGAAT
CTACTATACGCCTGAGCTACGGAAATATGATGTGGGGGTTCTGCAAACAGGCATCTTCACTTTCCCTGTG
CATTTCATTCCTCCTGGAGCAGAATCCTACAGATCTTACGGCCTGTGCAATTCCAGCCAGTTTGATGAAA
TGAATGGGATGCCAGTTCCAGATCTGCATGTCTTTGCCTACTTGCTTCACACCCACCTGTCTGGCAGAGG
AGTGAAAGCTGCTCAATACCGGAATGGTGAGCAGCTAGGGATCATCTGTGAGGACAATAAGTATGATTTC
```

```
AGACTGCAGGAGATTCGGGACATGAAGGAAATCCTCACAATCAAATCAGGGGATGAGATCCTGGTCGAAT
GCAACTTTCAGACACTGGATCGGTCAGAGGTTACTTTTGGTGGGCCAAGCACCATGAATGAGATGTGTCT
CACATTCCTCTTCTACTACCCTCGTAACAACATCTCCAGTTGTATGAGCTACCCTGACATTTTGTACATT
GCACATGTACTCAAGCAGGAGGCCTCAGATGCAGCGGAAGGAATGATGGCCATGGAATTTGTTGACTGGG
ACAATGAGACTGTCAGAATTGCAGAGAAAGCAGCCAAGGAGGCAGATCAAGTAGTCATCATTAAAACCAT
TAATGAACTCCAGAGAAATGAAAGTGGTCTAATCAGAGACATTAGTATTCCAGAGCGGGCTGCCTGTCAC
AATATTTCTGGACACCGCTCACGGTCAGGTCTGAGGGCCACTGCAAACCTTCGTTTGACCGCAGCATGCA
GTTCCGAGTCATCAACTACCAAAGAAAGTTCTTCACTTCCTCTTCTTTCTCTCACACAGCTGGTGTTTGC
TTGGCTTGTCTTGGCCTCTGAGTACGGGAAGTGA

>Phoenicopterus_ruber_ruber No=45 length=617 name="Flamingo"
MMAVLFSRIKGTLFLLFLPCFCSGQPAPPPLRFSIFLDPSNMVYLRWDHDEQELMTFELQVHTTGWVAFG
FSPHGELPGSDIVIGGVFPNGSIYFSDCHVVDEATLEEDESQDYQLLSVTENETSTTMLFKRHLRTCDPN
DLDISMDTARLVTAFGTDDTVQFFKGHRFYKSLFLMNYRGPYDSTDPKIFSTYDLRLDNFAVPAEETKYA
CTFIPLPMVKQKHHIYKFEPVITPHNITLVHHILVYACGNASVLPSGIDDCYGADPDFALCSQVLVGWAV
GGESYQFPDEAAVSIGTPWDPQYVRLEIHYSNFDLLAGLIDSSGVRIYYTPELRKYDVGVLQTGIFTFPV
HFIPPGAESYRSYGLCNSSQFDEMNGMPVPDLHVFAYLLHTHLSGRGVKAAQYRNGEQLGIICEDNKYDF
RLQEIRDMKEILTIKSGDEILVECNFQTLDRSEVTFGGPSTMNEMCLTFLFYYPRNNISSCMSYPDILYI
AHVLKQEASDAAEGMMAMEFVDWDNETVRIAEKAAKEADQVVIIKTINELQRNESGLIRDISIPERAACH
NISGHRSRSGLRATANLRLTAACSSESSTTKESSSLPLLSLTQLVFAWLVLASEYGK
```

Exon	NCBI Accession	Strand	Start	End	
1	JJRE01063800.1	-	944	>1204	5' partial (27 nt; recovered from WGS data)
2	JJRE01063799.1	-	7402	7548	
3	JJRE01063799.1	-	5758	5919	
4	JJRE01063799.1	-	4300	4383	
5	JJRE01063799.1	-	2936	3103	
6	JJRE01063799.1	-	1904	2006	
7	JJRE01063799.1	-	1056	1222	
8	JJRE01038075.1	+	185	276	
9	JJRE01038075.1	+	3451	3547	
10	JJRE01038075.1	+	3764	3823	
11	JJRE01038075.1	+	4844	4974	
12	JJRE01038075.1	+	5828	5942	
13	JJRE01038075.1	+	6609	6848	

---

46. Great crested grebe (*Podiceps cristatus*)

```
>Podiceps_cristatus No=46 length=1863 name="Great crested grebe"
ATGATGATGGCAGTGTCTGTTCTTTTCTTGAGAATCAAGGGTATGCTCTTCCTCTTGTTCCTCCCATGCT
TCTGTTCTGGTCAGCCTGCACTTCCACAGCTGCGTTTCTCCGTCTTCCTGGATCCTTCAAACATGGTCTA
CCTCCGCTGGGACCATGACGAACAGGAGCTGATGATGTTTGAGCTGCAGGTCCATACAACTGGCTGGGTG
GCATTTGGATTCAGCCCTCACGGAGGGTTGCCTGGATCTGACATTGTGATAGGAGGTGTCTTCCCAAATG
GCACTATCTACTTCTCTGATTGTCATGTGGTAGATGAGGCAACCCTTGAAGAAGATGAGAGCCAGGACTA
CCAACTGCTGTCAGTAACAGAGAATGAGACCTCCACCACCGTGCTGTTCAAACGCCACCTCCGGACATGT
GACCCGAATGACCTGGATATCACAATGGACACAGCACGACTCGTTACTGCATTTGGCACTGATGACACAG
TCCAATTCTTTAAAGGCCAAAGACTTTACAAATCTCTTTTCTTGATGAGCTACAGAGGCCCATCTGACTC
AACTGACCCCAAAATATTCTTCACCTATGACCTGAGGCTGGACAACTTTGCTGTTCCCGTTGAAGAAACC
AAGTATGCCTGTACTTTTATCCCACTGCCCATGGTCAAGCAGAAACACCATATCTACAAGTTCGAACCTG
TAATGACACCCCACAACATAACCTTGGTTCATCATATCCTTGTTTATGCCTGTGGCAATGCCAGCGTGTT
ACCCAGTGGCATAGATGATTGCTACGGAGCCAATCCAGATTTTGCCCTGTGCTCTCAGGTTATTGTGGGC
```

```
TGGGCTGTTGGAGGAGAGTCTTACCAATTTCCAGATGAAGCTGCATTTTCTATTGGGACACCTTGGGACC
CTCAGTACGTCCGACTAGAAATCCATTACAGCAATCTCGACTTGTTACCAGGCTTGACCGACAGCTCAGG
GGTACGAATCTACTATACGCCTGAGCTACGGAAATATGATGTAGGGGTTCTGCAAACAGGCATCTTCACT
TCCCCTGTGCATTTCATTCCTCCTGGAGCAGAATCCTACAGATCTTATGGCCTTTGCAATTCCAGCCAGT
TTGATGAAATGAATGGGATGCTGGTTCCAGATCTGCATGTCTTTGCCTACTTGCTTCACACTCACCTGTC
TGGCAGAGGAGTGAAAGCTGCTCAATACCGGAATGGTGAGCAGCTGGGCATCATCTGCGAGGACAATAAG
TACGACTTCAGACTGCAGGAGATTCGGGACATGAAGGAAATCCTCATAATCAAACCAGGCGATGAGATCC
TGGTCGAATGCAACTTTCAGACACTGGATCGGGCAGAGATTACTTTTGGTGGGCCAAGCACCACGAATGA
GATGTGTTTCACATTCCTCTTCTACTACCCTCGTAACAACATCTCCAGTTGTATGAGCTACCCTGACATC
TTGTATGTTGCACATGTACTCAAGCAGGAGGCTTCAGATGTAGTGGAAGGAATGATGGTAATGGACTTTG
TTGACTGGGACAATGAGACTGTCAAAATTGCAGAGAAAGCAGCCAAGGAGGCAGATCAAGTAGTCATGAT
TAAAACCATTAATGAACTCCAGAGAAATGAAAGTGGTCTAATCAGAGACATTAGTATTCCAGAGAGGGCT
GCCTGTCACAGTATTTCTGGACACCTCTCACTGTCAGGTCTGAGGGCCACTGCAAACCTTCGTTTGACCG
GAGTATGGACTTCTGAGTCATCAGCTACCAAAGGAAATCCTTCACTTCCTCTCCTTTCTCTCACACAGCT
GGTGTTTGCTTGGCTTGTCTTGGCCTCTGAGTATGGGAAGTGA
```

```
>Podiceps_cristatus No=46 length=620 name="Great crested grebe"
MMMAVSVLFLRIKGMLFLLFLPCFCSGQPALPQLRFSVFLDPSNMVYLRWDHDEQELMMFELQVHTTGWV
AFGFSPHGGLPGSDIVIGGVFPNGTIYFSDCHVVDEATLEEDESQDYQLLSVTENETSTTVLFKRHLRTC
DPNDLDITMDTARLVTAFGTDDTVQFFKGQRLYKSLFLMSYRGPSDSTDPKIFFTYDLRLDNFAVPVEET
KYACTFIPLPMVKQKHHIYKFEPVMTPHNITLVHHILVYACGNASVLPSGIDDCYGANPDFALCSQVIVG
WAVGGESYQFPDEAAFSIGTPWDPQYVRLEIHYSNLDLLPGLTDSSGVRIYYTPELRKYDVGVLQTGIFT
SPVHFIPPGAESYRSYGLCNSSQFDEMNGMLVPDLHVFAYLLHTHLSGRGVKAAQYRNGEQLGIICEDNK
YDFRLQEIRDMKEILIIKPGDEILVECNFQTLDRAEITFGGPSTTNEMCFTFLFYYPRNNISSCMSYPDI
LYVAHVLKQEASDVVEGMMVMDFVDWDNETVKIAEKAAKEADQVVMIKTINELQRNESGLIRDISIPERA
ACHSISGHLSLSGLRATANLRLTGVWTSESSATKGNPSLPLLSLTQLVFAWLVLASEYGK
```

Exon	NCBI Accession	Strand	Start	End
1	JMFS01078962.1	+	5051	5347
2	JMFS01078962.1	+	8162	8308
3	JMFS01078962.1	+	10875	11036
4	JMFS01078962.1	+	12253	12336
5	JMFS01078962.1	+	13560	13727
6	JMFS01078962.1	+	14639	14741
7	JMFS01078962.1	+	15411	15577
8	JMFS01057080.1	-	9054	9145
9	JMFS01057080.1	-	5754	5850
10	JMFS01057080.1	-	5478	5537
11	JMFS01057080.1	-	4258	4388
12	JMFS01057080.1	-	3304	3418
13	JMFS01057080.1	-	2314	2553

---

47. Rock pigeon (*Columba livia*)

Exon	NCBI Accession	Strand	Start	End	
1	AKCR01010579.1	+	20544	20828	1 nonsense codon
2	AKCR01010579.1	+	24783	24928	1-nt deletion, splice donor CA
3	AKCR01010579.1	+	26483	26644	splice donor AT
4	AKCR01010579.1	+	27845	27928	splice donor AT
5	AKCR01010580.1	+	189	356	

6	AKCR01010580.1	+	1298	1400	
7	AKCR01010580.1	+	2072	2238	
8	AKCR01010581.1	+	611	682	20-nt deletion
9	AKCR01010581.1	+	3030	3126	
10	AKCR01010581.1	+	3342	3401	splice acceptor TA
11	AKCR01010581.1	+	4491	4621	
12	AKCR01010581.1	+	4910	5024	
13	AKCR01010581.1	+	5760	5999	

48. Yellow-throated sandgrouse (*Pterocles gutturalis*)

Exon	NCBI Accession	Strand	Start	End	
1	JMFR01016479.1	-	17680	17966	1-nt deletion, 1 nonsense codon
2	JMFR01016479.1	-	14766	14912	
3					exon deletion
4	JMFR01016479.1	-	12587	12670	
5	JMFR01016479.1	-	11256	11424	1-nt insertion
6	JMFR01016479.1	-	9948	10050	
7	JMFR01016479.1	-	9058	9224	
8					exon deletion
9					exon deletion
10					exon deletion
11	JMFR01016479.1	-	7185	7318	
12	JMFR01016479.1	-	6779	6893	
13	JMFR01016479.1	-	5843	6068	2-nt deletion

49. Brown mesite (*Mesitornis unicolor*)

Exon	NCBI Accession	Strand	Start	End	
1	JJRI01040398.1	-	16359	16611	5-nt deletion
2					exon deletion
3					exon deletion
4	JJRI01040398.1	-	12700	12783	1 nonsense codon
5	JJRI01040398.1	-	11358	11525	
6	JJRI01040398.1	-	10302	10395	1-nt deletion, 8-nt deletion
7	JJRI01040398.1	-	9371	9537	1 nonsense codon
8					exon deletion
9	JJRI01040398.1	-	6954	7050	1 nonsense codon
10	JJRI01040398.1	-	6679	6738	splice acceptor CA
11	JJRI01040398.1	-	5529	5659	splice donor AA
12	JJRI01040398.1	-	5156	5270	

13	JJRI01040398.1	-	4175	4411	1 nonsense codon

---

50. Mallard (*Anas platyrhynchos*)

```
>Anas_platyrhynchos No=50 length=1851 name="Mallard"
ATGTCTGTTCTCTTCTCAAGAATAAGGGGCATGTTCTTCCTCTTGTTCCTCACATGGTTTTGTTCTGGTC
AGCCTGCACCTCCACTCTTACGTTTCTCTGTCTACCTGGATCCTTTAAACATGGTATACCTTCGTTGGGA
CCATGATGAACAGGAGATGATGACTTTTGAGCTGCAGGTTCACACAACAGGCTGGGTGGCGTTTGGATTC
AGCCCTCATGGAGAGTTGCCTGGATCTGACATTGTGATTGGAGGTGTCTTCTCTAATGGCAGCATCTACT
TCTCTGATTTTCATGTGGTAGATGATGCAACCCTTGAGGAAGATGATAGCCAGGACTACCAACTGCTGTC
AGTGACAGAGAATGAAACCTCCACCACCATGCTGTTCAAGCGCCACCTCCGGACATGTGACTCAAATGAC
CTGGATATCACAATGGACACAGCACGTCTCATTACTGCATTTGGCACTGATGACACAGTACAACTCTTTA
AGGGCCAAAAATTTTCTAAGTCTCTTTTCTTGATGAGATACAGAAGTCCATCCAACTTAACTGACCCTAA
AATATTCTTCACCTATGACCTGAGGCTGGACAACTTTGCTGTTCCTGTTGAAGAAACCAAATATGCCTGT
ACCTTTCTTCCACTGCCCAGGGTTAAGCAGAAACACCACATCTACAAGTTTGAGCCTGTAATAACTCCCC
ACAACATAACTTTAGTTCATCATATTCTGGTTTACGCCTGTGGCAATGCCAGTGTGTTACCAAGTGGCAT
AGATGACTGCTATGGAGCCAATCCAGATTTTGCCCTGTGCTCTCAGGTGGTTGTGGGCTGGGCTGTTGGA
GGAGAGTCTTATCAATTTCCAGATGATGCTGCATATTCCATCGGAACACCTTGGGACCCTCAGTATGTCC
GACTGGAAATCCATTACAGCAATTTTGACTTGGTATCAGGTTTGATCGACAGCTCAGGGGTACGAATATA
CTATACACCAGAGGTACGAAAGTATGATGTGGGGATACTGCAAACAGGCATCTTCACCTTCCCTGTGCAT
TTCATTCCTCCTGGAGCAGAATCCTACAGATCTTATGGCCTTTGCAATTCAAGCCAGTTTGATGAAATGA
ATGGGACACCAGTTCCAGATCTGCACGTCTTTGCCTACTTGCTTCACACTCATCTGGCTGGCAGAGGAGT
GAAGGTTGCCCAGTACAGGAATGAAAAACAGCTGGGGATCATCTGTGAGGACAACAAGTATGACTTCACC
TTGCAGGAGATTCGGGACATGAAGGAAATCCTTGTAATCAAACCAGGGGATGAGATCCTGGTCGAATGTA
ACTTTCAGACACTGGATCGGTCAGGGATTACTTTTGCTGGGCCAAGCACCATGAATGAGATGTGTCTCTC
ATTCCTCTTCTACTACCCTCGTAACAACATCTCCAGCTGTATGGGCTACCCTGACATTTTGTATGTTGCG
CATGTACTCAAGCAGGAGGCCTCAGATGCAGTGGAAGGAATGATGGCCATGGACTTTGTTGACTGGAACA
ACGAAACTGTCAAAACTGCAGAGAAAGCAGCCAAGGAGGCAGATCAAATAGTTGTGATTAAAACCATTAA
TGAAATCCAGAAAAATGAGAGTGGTCTCATCAGAGACATTCATATTCCAGAGCGGGCTGTCTGTCACAAT
GTTTCTGTACAACAGACACTGTCAGATCTGAGAGCTACTGCAAACCTTCGTTTGACCGCAGTACACAATT
TTGAATCCTCAACCACCAAAGAATTTGTTTCACTTCCTCTTCTTTTTCTCACACAGCTGTTGTTTGCTTG
GATTATTTTTTCCTCTGAGTATAGGAAGTGA

>Anas_platyrhynchos No=50 length=616 name="Mallard"
MSVLFSRIRGMFFLLFLTWFCSGQPAPPLLRFSVYLDPLNMVYLRWDHDEQEMMTFELQVHTTGWVAFGF
SPHGELPGSDIVIGGVFSNGSIYFSDFHVVDDATLEEDDSQDYQLLSVTENETSTTMLFKRHLRTCDSND
LDITMDTARLITAFGTDDTVQLFKGQKFSKSLFLMRYRSPSNLTDPKIFFTYDLRLDNFAVPVEETKYAC
TFLPLPRVKQKHHIYKFEPVITPHNITLVHHILVYACGNASVLPSGIDDCYGANPDFALCSQVVVGWAVG
GESYQFPDDAAYSIGTPWDPQYVRLEIHYSNFDLVSGLIDSSGVRIYYTPEVRKYDVGILQTGIFTFPVH
FIPPGAESYRSYGLCNSSQFDEMNGTPVPDLHVFAYLLHTHLAGRGVKVAQYRNEKQLGIICEDNKYDFT
LQEIRDMKEILVIKPGDEILVECNFQTLDRSGITFAGPSTMNEMCLSFLFYYPRNNISSCMGYPDILYVA
HVLKQEASDAVEGMMAMDFVDWNNETVKTAEKAAKEADQIVVIKTINEIQKNESGLIRDIHIPERAVCHN
VSVQQTLSDLRATANLRLTAVHNFESSTTKEFVSLPLLFLTQLLFAWIIFSSEYRK
```

Exon	NCBI Accession	Strand	Start	End
1	NW_004676532.1	+	1591828	1592112
2	NW_004676532.1	+	1594523	1594669
3	NW_004676532.1	+	1595616	1595777
4	NW_004676532.1	+	1596280	1596363
5	NW_004676532.1	+	1597559	1597726
6	NW_004676532.1	+	1598701	1598803
7	NW_004676532.1	+	1599136	1599302
8	NW_004676532.1	+	1601044	1601135

9	NW_004676532.1	+	1603475	1603571
10	NW_004676532.1	+	1603765	1603824
11	NW_004676532.1	+	1604680	1604810
12	NW_004676532.1	+	1605060	1605174
13	NW_004676532.1	+	1605889	1606128

---

51. Northern bobwhite (*Colinus virginianus*)

gene deletion

---

52. Japanese quail (*Coturnix japonica*)

gene deletion

---

53. Chicken (*Gallus gallus*)

gene deletion

---

54. Wild turkey (*Meleagris gallopavo*)

gene deletion

---

55. Black grouse (*Tetrao (Lyrurus) tetrix tetrix*)

gene deletion

---

56. White-throated tinamou (*Tinamus guttatus*)

```
>Tinamus_guttatus No=56 length=1851 name="White-throated tinamou"
ATGTTAGTAGATCTTTTCACAAGAATCAAGGGTATGCTCCTCTTCTTGTTCATTCCATGCCTTTGTTCTG
GTCAGGCCACACCTGCATTGTTGCGTTTTTCCAGCTTTTTGGATCCTTCCAACATAGTCCACCTCCGCTG
GGACCACAATGAACAGGAGATGATAACTTTTGAGCTGCAGGTCCGTGCACCTGGCTGGGTGGCATTTGGG
TTCAGCCCGCATGGAGAGTTACCTGGATCTGACATTGTAATCGGAGGTGTCTTCCCAAATGGCAGCATAT
ATTTCTCTGATTGTCATGTTGTAGATGAGGCAACTGTTGACGCAGATGAGAGCCAGGACTACCAACTGCT
GTCACTGGCGCAGGATGAGACTTTTACTACCATGATGTTCAAACGCCACCTCCTTACATGTGACCTGAAT
GACCTAGATATCACAATGGATACAGCACACCTCATTACAGCATTTGGCACTGATGACACGGTCCAATTCT
CTAAAGGCCAAAAATTTTCCAAATCTCTTTTCTTGATGAAGTACAGAGGGTCATCTGACTCAGCTGACCC
CAAAATATTCTTCACATATGACCTGAGACTGGACAATTTCGCTGTTCCAGCTGAAGAAACTAAGTATGCC
TGTACCTTTATACCACTGCCTATGGTCAAGCAGAAACATCACATCTATAAGTTTGAACCTGTATTAACAC
CCCACAACATAACTTTGGTTCATCACATCCTGGTTTATGCCTGTGGCAGTGACAGTGTAATACCCAGTGG
CATAGATGATTGCTATGGAGTTGATCCAGATTTTGCCCTGTGCTCTCAGGTGCTTGTGGGCTGGGCTGTT
GGGGGAGAGTCCTATCGATTTCCAGATGAAACTGCACTCTCCATAGGGACACCGTTGGATCCTCAGTACA
TCCGACTGGAAATCCACTACAGCAATTTCGACTTGTTACCAGGGTTGATCGACAGCTCAGGGGTGCGAAT
```

```
CTACTACACATCGGAGCTGCGGAAATATGATGCGGGGGTTCTGCAGACAGGCATCTTCACTTTTCCTGTA
CATTTCATCCCTCCTGGAGCAGAATCCTATAGATCTTATGGCCTTTGCAATACTAGCCAGTTTGATGAAG
TGAATGGGACACCAGTTCCAGAGTTGCACGTATTTGCATACCTTCTTCACACACACCTGTCTGGTAGAGG
AGTGAAAGTAGCCCAGTACCGGAATGGTGAGCAGCTGCGCATCATCTGTGAGGACAATAAGTATGATTTC
ACACTGCAGGAGGTTCGGGATATGAAGAAAATCCTCACAATCAAACCAGGGGATGAGATCCTGGTGGAAT
GTAGCTTTCAGACACTGGATCGGTCAGTGATTACTTTTGGTGGGCTAAGTACCATGAATGAGATGTGCCT
CACGTTCCTCTTCTACTATCCTCGTAACAACATCTCCAGTTGTATGGGCTACCCTGACATTCTATATATT
GCCCACATGCTCAAGCAGGAGGCCTCGGATACAGTAGAAGGAATGATGGCCATGGACTTTATTGACTGGG
ACAATGAGACCATCAAAACTGCAGAGAAAGCAGCCAAGGAGGCAAATCAAGTAGTTGTGATTAAAACAAT
TAATGAACTGCAGAAAATCGGGAGTGGCCTAATCAGAGACATGATTAATCCAGAGCGGGCTGTCTGTCAC
AGTATTTCAGAAAATATCTCACTGTCACTGAAGGCTGCCACGAACCTTAATTTTGCTGCAGTGCTCAGGT
CTGAGTCATCGACTACCAAAGAGATGGCTTCACTTCCTCTTCTTTCTTTCACACAGATACTATTTGCTTG
TCTTCTTTTGTCATCTGAGCTTAGAATGTAA

>Tinamus_guttatus No=56 length=616 name="White-throated tinamou"
MLVDLFTRIKGMLLFLFIPCLCSGQATPALLRFSSFLDPSNIVHLRWDHNEQEMITFELQVRAPGWVAFG
FSPHGELPGSDIVIGGVFPNGSIYFSDCHVVDEATVDADESQDYQLLSLAQDETFTTMMFKRHLLTCDLN
DLDITMDTAHLITAFGTDDTVQFSKGQKFSKSLFLMKYRGSSDSADPKIFFTYDLRLDNFAVPAEETKYA
CTFIPLPMVKQKHHIYKFEPVLTPHNITLVHHILVYACGSDSVIPSGIDDCYGVDPDFALCSQVLVGWAV
GGESYRFPDETALSIGTPLDPQYIRLEIHYSNFDLLPGLIDSSGVRIYYTSELRKYDAGVLQTGIFTFPV
HFIPPGAESYRSYGLCNTSQFDEVNGTPVPELHVFAYLLHTHLSGRGVKVAQYRNGEQLRIICEDNKYDF
TLQEVRDMKKILTIKPGDEILVECSFQTLDRSVITFGGLSTMNEMCLTFLFYYPRNNISSCMGYPDILYI
AHMLKQEASDTVEGMMAMDFIDWDNETIKTAEKAAKEANQVVVIKTINELQKIGSGLIRDMINPERAVCH
SISENISLSLKAATNLNFAAVLRSESSTTKEMASLPLLSFTQILFACLLLSSELRM
```

Exon	NCBI Accession	Strand	Start	End
1	JMFW02052843.1	+	44990	45277
2	JMFW02052843.1	+	48249	48395
3	JMFW02052843.1	+	49878	50039
4	JMFW02052843.1	+	51353	51436
5	JMFW02052843.1	+	52568	52735
6	JMFW02052843.1	+	53259	53361
7	JMFW02052843.1	+	54442	54608
8	JMFW02052843.1	+	55880	55971
9	JMFW02052843.1	+	59767	59863
10	JMFW02052843.1	+	60072	60131
11	JMFW02052843.1	+	60990	61120
12	JMFW02052843.1	+	61409	61523
13	JMFW02052843.1	+	62171	62407

---

57. African ostrich (*Struthio camelus australis*)

```
>Struthio_camelus_australis No=57 length=1833 name="African ostrich"
ATGGTGGTGATTCTGTTCTCAAGGATCAAAGATATGCTCCTACTCTTGTTCATCCCATGCCTTTGTTCTG
GTCAGTCTGCACCTCCATTGCTGCGTTTCTCCAGCTTCCTGGATCCTTCTAACATGGTCTACCTCCGCTG
GGACCACAATGAACAGGAGTTGATGACCTTTGAGCTGCAGGTCCTCACGACTGGCTGGGTGGCATTTGGG
TTCAGCCCTCATGGAAAGTTGCCTGGATCTGACATTGTGATGGGAGGCATATTTCCTAATGGCAGCATCT
ATTTCTCTGATTGTCATGTTGTAGATGAGGCAACCCTTGAGGAAGACGAGAGCCAAGACTACCAACTGCT
GTCACTGACAGAGGATGAGACCTTCACCACTATGCTGTTCAAACGCCACCTCCGGACATGTGACCCAAAT
GACCTGGATATCACAATGGACACAGCACACCTCATTACTGCATTTGGCACTGCTGACACAGTCCAATTCT
TTAAAGGCCAAAGGTTTTCCAAATCTCTTTTCTTGATGAGGTACAGAGGCACATCTGACTCAACTGACCC
```

CAAAATATTCTTCACTTATGACATGGGGCTGTACAATTTTGCTGTTCCAGCTGAAGAAACGAAGTATGCC
TGTACCTTTATCCCACTGCCCATGGTCAAACAGAAACACCCACATCTACAAGTTTGAACCTGTAATAACAC
CCCACAACATAACTTTGGTTCATCACATTCTGGTTTATGCCTGTGGCAATGCCAATGTGTTACCCAGTGG
CATAAATGACTGCTACGGAGCTGATCCAGATTTTGCCCTGTGCTCTCAGGTGCTTATGGGCTGGGCTGTT
GGGGGAGAGTCCTATCAATTTCCAGATGAAGCTGCGATCTCCATAGGGACATCTTTGGACCCTCAGTACA
TCCGACTGGAAATCCACTACAGCAATTTTGACTTGTTACCAGGTTTGATCGACAGCTCAGGGGTACGAAT
CTACTACACACCAGAGCTACGAAAATATGATATGGGGATTCTGCAAACAGGCATCCTCACTTTCCCTGTA
CATTTCATCCCTCCTGGAGCAGAAACCTACAGATCTTACGGCCTTTGCAATACTAGCCAGTTTGATGAAA
TGAACGGGACACCAGTTACAGAGCTGCATGTGTTTGCCTACCTGCTTCACACCCACCTGTCTGGCAGAGG
AGTGAAAGTTGCCCAATACCGGAATGGTGAGCAGCTGGGGATCATCTGTGAGGACAATAAGTATGATTTC
ACATTGCAGGAGATTCGGGACACGAAGGAAATCGTCACAATCAAACCAGGGGATGAGATCCTGGTGGAAT
GTAGCTTTCAGACACTGGATCGGTCAGGGATTACTTTCGGTGGGCCAAGCACCATGAATGAGATGTGCCT
CACATTCCTCTTCTACTACCCTCGTAACAACATCTCCAGTTGCATGGGCTACCCTGACATTTTATATATT
GCACACATACTGAAGCAGGAGGCCTCAGATACAGTGGAAGGAATGATGGCCATGGACTTTGTTGACTGGG
ACAACGAGACCATCAAAATTGCAGAGAAAGCAACCAAGGAGGCAGATCAAGTAGTCATGATTAAAACAAT
TAATGAACAGCAGAAAAATGAGACAGGTCTAATCAGAGACATAATTATTCCAGAGCGGGCTGCCTGTCAC
AATATTTCTGAAAACCTCTCATTGTCAGGTCTGAGGGCTGCCACAAATCTTCGTTTGGCTGCAGTGTGTA
GGTCTGAGTCATCAACCACCAAAGAAATGTCTTCACTTCCTCTTCTTTCTCTCACACAGCTGGTATTTGC
TTGGCTTATCTAG

>Struthio_camelus_australis No=57 length=610 name="African ostrich"
MVVILFSRIKDMLLLLFIPCLCSGQSAPPLLRFSSFLDPSNMVYLRWDHNEQELMTFELQVLTTGWVAFG
FSPHGKLPGSDIVMGGIFPNGSIYFSDCHVVDEATLEEDESQDYQLLSLTEDETFTTMLFKRHLRTCDPN
DLDITMDTAHLITAFGTADTVQFFKGQRFSKSLFLMRYRGTSDSTDPKIFFTYDMGLYNFAVPAEETKYA
CTFIPLPMVKQKHHIYKFEPVITPHNITLVHHILVYACGNANVLPSGINDCYGADPDFALCSQVLMGWAV
GGESYQFPDEAAISIGTSLDPQYIRLEIHYSNFDLLPGLIDSSGVRIYYTPELRKYDMGILQTGILTFPV
HFIPPGAETYRSYGLCNTSQFDEMNGTPVTELHVFAYLLHTHLSGRGVKVAQYRNGEQLGIICEDNKYDF
TLQEIRDTKEIVTIKPGDEILVECSFQTLDRSGITFGGPSTMNEMCLTFLFYYPRNNISSCMGYPDILYI
AHILKQEASDTVEGMMAMDFVDWDNETIKIAEKATKEADQVVMIKTINEQQKNETGLIRDIIIPERAACH
NISENLSLSGLRAATNLRLAAVCRSESSTTKEMSSLPLLSLTQLVFAWLI

Exon	NCBI Accession	Strand	Start	End
1	JJRT01038259.1	-	1996	2283
2	JJRT01038258.1	-	4444	4590
3	JJRT01038258.1	-	2725	2886
4	JJRT01038258.1	-	1291	1374
5	JJRT01038257.1	-	39243	39410
6	JJRT01038257.1	-	38417	38519
7	JJRT01038257.1	-	37163	37329
8	JJRT01038257.1	-	35268	35359
9	JJRT01038257.1	-	30763	30859
10	JJRT01038257.1	-	30487	30546
11	JJRT01038257.1	-	29354	29484
12	JJRT01038257.1	-	28956	29070
13	JJRT01038257.1	-	27998	28216
