# Supplementary material for: MOXD2, a Gene Possibly Associated with Olfaction, Is Frequently Inactivated in Birds
Source: PLoS One. 2016 Apr 13;11(4):e0152431. doi: 10.1371/journal.pone.0152431 (PMC4830563; doi:10.1371/journal.pone.0152431)

S2 Fig. Alignment of full-length bird MOXD2 sequences

```
                                   ::  ::   ::* :  :*  : : . *:.*  *:* : *:::***:::*:: ***:* :.******:*  *
 1 Rifleman                     ---MMAIVFSRIKRMFFLLFFPCFCSGQLAPPPLRFSTFLDPSNMVYLHWDHDDQELMTFELQVHTTGWVAFGFSPHGEL   77
24 Barn owl                     ---MMAVLLSRIKGMLFLLFLPFFCSGQPAPPLLRFSIFLDPSNTVYLRWDHDEQELMTFELQVPTTGWVAFGFSPRGEL   77
25 Turkey vulture               ---MMAVLFSRIKDMLFLLFLPCFCSGQPACPLLRFSIFLDPSNMVYLRWDHDEQELMTFELQVHTTGWVAFGFSPHGEL   77
26 White-tailed eagle           ---MMAVLFSGIKGMLFFLFLSCFCSCQPAPPLLRFSIFLDPSNMVYLRWDHDEQELMTFELQVRTTGWVAFGFSPHGEL   77
27 Golden eagle                 ----MAVLFSGIKGMLFFLSLPCFCSCQPAPPLLRFSIFLDPSNMVYLRWDHDEQELMTFELQVHTTGWVAFGFSPHGEL   76
28 White-tailed tropicbird      ---MMAVLFTRIKGMPFLLFLPCFCSGHPAAPLLRFSIFLEPSNMVYLRWDHDEQEMITFELQVHTTGWVAFGFSPHGEL   77
30 Red-throated loon            ---MMAVLFSRIKGMLFLLFLPCFCSGQPAPPLLRFSIFLDPSNTVYLRWDHDEQELMTFELQVPTTGWVAFGFSPHGEL   77
33 Northern fulmar              ---MMAVLLSRIKGMLFLLFLPCFCSGQPAPPPLRFSIFLDPSKMVYLRWDHDEQELMTFELQVHTTGWVAFGFSSHGEL   77
38 East African crowned crane   ---MMAVLL-RIKAVLFLLFLPSFCSGQPAPPLLHFSILLEPSNMVYLHWDHDEQEMMTFELQVHTTGWVAFGFSPHGEL   76
39 Hoatzin                      ----MAVLLSTMKGMLFLLLLPCFCFGQPAPPLLRFSIFLEPANRVYLHWDHDEEEMMTFELQVHTTGWVAFGFSPDGEL   76
40 Red-crested turaco           ---MMAVLFSRIKGMLLLLFFLCFCSSQPAPPLLRFSIFLDPSNMVYLRWDHDEQELMTFELQVHTTGWVAFGFSPHGEL   77
41 MacQueen's bustard           ---MKAVVFSRIKSMLFLLFLPWFCSGQTAHPLLRHSIFLDPSNMVYFRWDHDEQELMTFELQVHTAGWVAFGFSPYGEL   77
42 Common cuckoo                ---MMAVLFSRIKGMLFLLFLPRFCSGQPALPLLRFSTFLDPSNMVYLRWDHDEQELMTFELHVHTTGWVAFGLSLHGEW   77
43 Chuck-will's-widow           ---MMAVVFSRIKGILFLLFLPCFCSGQPAPPLLRFSIFLDPSNMVYLRWDHDEQELMTFELQVHTTGWVAFGFSPHGEL   77
45 Flamingo                     ---MMAVLFSRIKGTLFLLFLPCFCSGQPAPPPLRFSIFLDPSNMVYLRWDHDEQELMTFELQVHTTGWVAFGFSPHGEL   77
46 Great crested grebe          MMMAVSVLFLRIKGMLFLLFLPCFCSGQPALPQLRFSVFLDPSNMVYLRWDHDEQELMMFELQVHTTGWVAFGFSPHGGL   80
50 Mallard                      ----MSVLFSRIRGMFFLLFLTWFCSGQPAPPLLRFSVYLDPLNMVYLRWDHDEQEMMTFELQVHTTGWVAFGFSPHGEL   76
56 White-throated tinamou       ---MLVDLFTRIKGMLLFLFIPCLCSGQATPALLRFSSFLDPSNIVHLRWDHNEQEMITFELQVRAPGWVAFGFSPHGEL   77
57 African ostrich              ---MVVILFSRIKDMLLLLFIPCLCSGQSAPPLLRFSSFLDPSNMVYLRWDHNEQELMTFELQVLTTGWVAFGFSPHGKL   77
                                1.......10........20........30........40........50........60........70........80
```

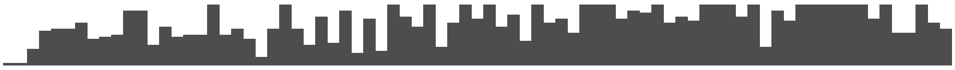

```
                                ****:*:**  .**:***** *:  .:: :: * ********:::::* **: *:*:: *** :* **: ***:::::*
 1 Rifleman                     PGSDIVIGGVFPNGSIYFSDCHMEGEATLEEDESQDYQLLSVTENKTSTTMSFKRHLRTCDTNDLDITMDTAHLVAAFGT   157
24 Barn owl                     PGSDIVIGGTFPNGSIYFSDCHMVDEATLEEDESQDYQLLSVTENETSTTMLFKRHLRTCDPNDLDITTDTARLIAAFGT   157
25 Turkey vulture               PGSDIVIGGVFPNGSIYFSDCHVVDEATLEEDESQDYQLLSVTENETSTTMLFKRHLRTCDPNDLDITVDTARVVTAFGT   157
26 White-tailed eagle           PGSDIVIGGVFPNGSIYFSDCHIVDEATLEEDGSQDYQLLSMTENETSTTVLFKRHLRTCDPNDQDITMDTARLITAFGT   157
27 Golden eagle                 PGSDIVIGGVSPNGSIYFSDCHVVDEATLEEDGSQDYQLLSVTENETSTTVLFKRHLRTCDPNDQDITMDTARLITAFGT   156
28 White-tailed tropicbird      PGSDIVIGGVFPNGSIYFSDCHVVDEATLEEDESQDYQLLSVTENETSTTMMFKRHLRTCDPNDLDITVDTARLVTSFGT   157
30 Red-throated loon            PGSDIVIGGVFPNGSIYFSDCHVVDEATLEEDESQDYQLLSVTENETSTTMLFKRHLRTCDPNDLDITMDTARLVTAFGM   157
33 Northern fulmar              PGSDIVIGGVFPNGSIYFSDCHVVDEATIEEDESQDYQLLSVTENETSTTMLFKRHLRTCDPNDLDITVDTARLVAAFGT   157
38 East African crowned crane   PGSDIVIGGVFPNGSIYFSDFHVVDEATLEEDESQDYQLLSVTENETSTTMLFKRHFRTCDVNDLDITMDTARLVTAFGT   156
39 Hoatzin                      PGSDIVIGGVFPNGSIYFSDCHVVDQATLEEDESQDYQLLSVTENETSTTMLFKRHLQTCDPNDLDITTDTARLVTAFGT   156
40 Red-crested turaco           PGSDIVIGGVFPNGSIYFSDCHVADEATLEEDESQDYQLLSVTENETSTTMLFKRHLRTCDPNDLDITVDTARLVTAFGT   157
41 MacQueen's bustard           PGSDIVIGGAFPNGSIYFSDCHVVDESTLEEDESQDYQLLSLTENETSTTMLFKRYFQTCDPNDLDITMDTARLITAFGT   157
42 Common cuckoo                PGSDVVIGGVFPNGSIYFSDCHMVDEAILEEDDSQDYQLLSVTENETCTTMLFRRHFRTCDPNDLDITVDTARLITAFGT   157
43 Chuck-will's-widow           PGSDIVIGGIFPNGSIYFSDCHVVDEATLEEDESQDYQLLSVTENETSTTMLFKRHLRTCDPDDQDITMDTARLITALGT   157
45 Flamingo                     PGSDIVIGGVFPNGSIYFSDCHVVDEATLEEDESQDYQLLSVTENETSTTMLFKRHLRTCDPNDLDISMDTARLVTAFGT   157
46 Great crested grebe          PGSDIVIGGVFPNGTIYFSDCHVVDEATLEEDESQDYQLLSVTENETSTTVLFKRHLRTCDPNDLDITMDTARLVTAFGT   160
50 Mallard                      PGSDIVIGGVFSNGSIYFSDFHVVDDATLEEDDSQDYQLLSVTENETSTTMLFKRHLRTCDSNDLDITMDTARLITAFGT   156
56 White-throated tinamou       PGSDIVIGGVFPNGSIYFSDCHVVDEATVDADESQDYQLLSLAQDETFTTMMFKRHLLTCDLNDLDITMDTAHLITAFGT   157
57 African ostrich              PGSDIVMGGIFPNGSIYFSDCHVVDEATLEEDESQDYQLLSLTEDETFTTMLFKRHLRTCDPNDLDITMDTAHLITAFGT   157
                                ........90.......100.......110.......120.......130.......140.......150.......160
```

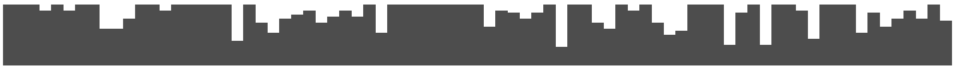

```
                                  *  : :.: : .*: *:  :..     .** * :**: * **.** :** ***.*:*** ******:***:* :*.::
 1 Rifleman                     DDTVQFFKSQRFSRSLFLMRYKSPSDSTDPKVFFTYDLRLDNFAVPVEETTYACTFIPLPMVKQKHHIYKFEPVITSHNI   237
24 Barn owl                     DDTVQFFKGQRFSKSLFLMRYRGPSDLNDPKIFFTYDLRLDNFAVPIEETKYACTFLPLPVVKQKHHIYKFEPIITPHNI   237
25 Turkey vulture               DDTVQFFKGQRFSKSLFLMRYRGPCDSTDPKIFFSYDLRLDNFAVPVEETKYACTFIPLPMVKQKHHVYKFEPVITPRNI   237
26 White-tailed eagle           DDTVQFFEGQRFSKSVFLMRYRGPSDLTDPKIFFTYDLRLDNFAVPVEETMYACTFIPLPMVKQKHHIYKFEPVITPHNI   237
27 Golden eagle                 DDTVQFFEGQRFSKSLFLMRYRGPSDLTDPKIFFTYDLRLDNFAVPVEETTYACTFIPLPVVKQKHHIYKFEPVVTPHNI   236
28 White-tailed tropicbird      DDTVQFFKGQRFSKSLFLMRSRGPSDSTGPKTFFTYDLRLDNFAVPAEETKYACTFMPLPMVKQKHHIYKFEPVITPHNI   237
30 Red-throated loon            DDTVQFFKGQRFSKSLFLMRYRGPSDSTDPKIFFTYDLRLDNFAVPAQETKYACTFIPLPMVKQKHHIYKFEPVITPHKI   237
33 Northern fulmar              DDTVPFFKGQIFSKSLFLLRFRSPS--TDPKIFFTYDLRLDNFAVPVEETKYACKFIPLPMVKQKHHIYKFDPVITPHNI   235
38 East African crowned crane   DYTVQFFKGQRFSNSLFLMRYRGPSDPTDPKIFFTYDLRLDNFAVPVEETKYACTFIPLPMVKQKHHIYKFEPTITPHNI   236
39 Hoatzin                      DDTVQFFKGQRFSRSLLLMRYRGPSDSTDPKIFFTYDLRLDNFAVPAEETKYACTFIPLPRVKQKHHIYKFEPVITPHNI   236
40 Red-crested turaco           DDTTQFFKGQRFSKSLFLMRYRGPSDSTNPKIFFTYDLRLDNFAVPVEETKYACTFIPLPMVKQKHHIYKFEPVITPHNI   237
41 MacQueen's bustard           DDTVQFFKGQRFSKSLFLMRYRGPSDSTDPKIFLTYDLTLDNFAVPVEETKYACTFIPLPMVKQKHHIYKFEPVITPHNI   237
42 Common cuckoo                DDTIQFVKGQRFSKSLSLMRYRGPSDLTDPKIFFTYDLRLDNFPVPVEETKYACTFIPLPIVKQKHHIYKFEPVITPHNT   237
43 Chuck-will's-widow           DDTVQFFQGQRFSKSLFLMRYRGPSDSTDPKIFFTYDLRLDNFAVPAEETKYACTFIPLPMVKQKHHIYKFEPVITPHNI   237
45 Flamingo                     DDTVQFFKGHRFYKSLFLMNYRGPYDSTDPKIFSTYDLRLDNFAVPAEETKYACTFIPLPMVKQKHHIYKFEPVITPHNI   237
46 Great crested grebe          DDTVQFFKGQRLYKSLFLMSYRGPSDSTDPKIFFTYDLRLDNFAVPVEETKYACTFIPLPMVKQKHHIYKFEPVMTPHNI   240
50 Mallard                      DDTVQLFKGQKFSKSLFLMRYRSPSNLTDPKIFFTYDLRLDNFAVPVEETKYACTFLPLPRVKQKHHIYKFEPVITPHNI   236
56 White-throated tinamou       DDTVQFSKGQKFSKSLFLMKYRGSSDSADPKIFFTYDLRLDNFAVPAEETKYACTFIPLPMVKQKHHIYKFEPVLTPHNI   237
57 African ostrich              ADTVQFFKGQRFSKSLFLMRYRGTSDSTDPKIFFTYDMGLYNFAVPAEETKYACTFIPLPMVKQKHHIYKFEPVITPHNI   237
                                .......170.......180.......190.......200.......210.......220.......230.......240
```

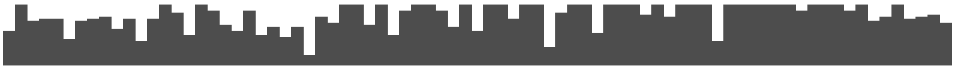

```
                                :*:****:*.**. . :* .:.**** :.**:*****. ***:**::*: *:::* ***:. ** *:********:**:.
 1 Rifleman                     TLVHHILVYACGNSSILPSGIGDCYGANPDFSLCSQVLVGWAVGGESYQFPDEAAVSIGTPWDPQYIRLEIHYSNLDLLP   317
24 Barn owl                     TLVHHILVYACGNVSVLPRGIDDCYGANPDFALCSQVLVGWAVGGESYQLPDEAAFSIGTPWDPWYIRLEIHYSNFDLLP   317
25 Turkey vulture               TLIHHILIYACGNASVLPSGIDDCYGANSDFSLCSQVLFGWAVGGKSYQFPDEAAVSIGTPWDPQYVRLEIHYSNFDLLP   317
26 White-tailed eagle           TLVHHILVYACGNASVLPSGIDDCYGANPDFALCSQVLVGWAVGGESYQFPDEAAVSIGTPWDPQYVRLEIHYSNFDLLP   317
27 Golden eagle                 TLVHHILVYACGNASVLPGGIDDCYGANPDFALCSQVLVGWAVGGESYQFPDEAAVSIGTPWDPQYVRLEIHYSNFDLLP   316
28 White-tailed tropicbird      TLVHHILVYACGNASVLPSGIDDCYGANPDFALCSQVLVGWAVGGESYQFPDEAAISIGTPRDPEYIRLEIHYSNFDLLP   317
30 Red-throated loon            TLIHHILVYACGNASPLPSGIDDCYGANPDFALCSQVLVGWAVGGESYQFPDEAAVSIGTPWDPQYVRLEIHYSNFDLLP   317
33 Northern fulmar              TLVHHILIYGCGNASVLPSGIDDCYGANPDFSLCSQVLMGWAIGGQTYQFPDEAAVSIGTPLDPQYIRLEIHYSNFDLLP   315
38 East African crowned crane   TLVHHILVYACGNASMLPSGIDDCYGANPDFSLCSQVLMGWAVGGQSYQFPDEAAVSIGTPWDPQYIRLEIHYSNFDLLP   316
39 Hoatzin                      TLVHHILVYACGNASVLPSSIDDCYGPNPDFALCSQVLVGWAVGGESYQYPEEAAVSIGTPWDPQYVRLEIHYSNFDLLP   316
40 Red-crested turaco           TLVHHILVYACGNASVLPSGVDDCYGANPDFALCSQVLAGWAVGGESYQFPDEAAISIGTPLDPQYVRLEIHYSNFDLLP   317
41 MacQueen's bustard           TLVHHILVYACGNASMLPSGIDDCYGANPDFALCSQVIVGWAVGGKSYQFPDEAAISIGAPLDPQYVRLEIHYSNFDLLP   317
42 Common cuckoo                ALVHHILVYACGNASVLPSGIDDCYGANPDFALCSQVIAGWAVGGESYQFPDEAAISIGTPWDPQYVRLEIHYSNFDLLP   317
43 Chuck-will's-widow           TLVHHILVYACGNASVLPTGIDDCYGANPDFALCSQVFVGWAVGGESYQFPDEAAISIGTPWDPQYVRLEIHYSNFDLLP   317
45 Flamingo                     TLVHHILVYACGNASVLPSGIDDCYGADPDFALCSQVLVGWAVGGESYQFPDEAAVSIGTPWDPQYVRLEIHYSNFDLLA   317
46 Great crested grebe          TLVHHILVYACGNASVLPSGIDDCYGANPDFALCSQVIVGWAVGGESYQFPDEAAFSIGTPWDPQYVRLEIHYSNLDLLP   320
50 Mallard                      TLVHHILVYACGNASVLPSGIDDCYGANPDFALCSQVVVGWAVGGESYQFPDDAAYSIGTPWDPQYVRLEIHYSNFDLVS   316
56 White-throated tinamou       TLVHHILVYACGSDSVIPSGIDDCYGVDPDFALCSQVLVGWAVGGESYRFPDETALSIGTPLDPQYIRLEIHYSNFDLLP   317
57 African ostrich              TLVHHILVYACGNANVLPSGINDCYGADPDFALCSQVLMGWAVGGESYQFPDEAAISIGTSLDPQYIRLEIHYSNFDLLP   317
                                .......250.......260.......270.......280.......290.......300.......310.......320
```

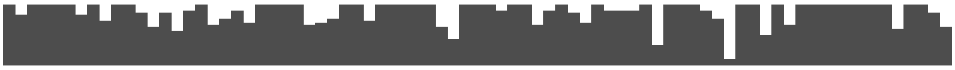

```
                               *  ****:::***.*:*::* *:****::  *            **:**:*:***:**  *.::*****:*****:***
 1 Rifleman                    GLIDSSGIRIYYTPELRKYDVGVLQTGVFIFPAHFIPPGAESYRSYGLCNSSQFDEMNGMLVPDLHVFAYLLHTHLSGRG   397
24 Barn owl                    GLIDSSGVRIYYTPELRNYDVGVLQTGIFTFPVHFIPPGAESYRSYGLCNSSQFDEMNGMLVPDLHVFAYLLHTHLSGRG   397
25 Turkey vulture              GLIDSSGVRIYYTPELRKYDVGVLQTGIFIFPMHFIPPGAESYRSYGLCNSSQFDEMNGMLVPDLHVFAYLLHTHLSGRG   397
26 White-tailed eagle          GLIDSSGVRIYYTPELRQYDVGVLQTGVFIFPVHFIPPGAESYRSYGLCNSSQFDEMNGMLVPDLHVFAYLLHTHLSGRG   397
27 Golden eagle                GLIDSSGVRIYYTPELRKYDVGVLQTGIFTFPVHFIPPGAESYRSYGLCNSSRFDEMNGMLVPDLHVFAYLLHTHLSGRG   396
28 White-tailed tropicbird     GLIDSSGVRIYYTPELRKYDVGVLQTGIFTFPVHFIPPGAESYRSYGLCNSSQFDEMNGMLVPDLHVFAYLLHTHLSGRG   397
30 Red-throated loon           GLIDSSGLRIYYTPELRKYDVGVLQTGIFTFPVHFIPPGAESYRSYGLCNSSQFDEMNGMLVPDLHVFAYLLHTHLSGRG   397
33 Northern fulmar             GLIDSSGVRIYYTPELRKHDVGILQTGIFSFP-------------YGFCNSSQFDEMNGMAVPDMHVFAYLLHTHLAGRG   382
38 East African crowned crane  GWIDSSGIRIYYTPELRKYDVGVLQTGIFTFPVHFIPPGAESYRSYGLCNSSQFDEMNGMLVPDLHVFAYLLHTHLSGRG   396
39 Hoatzin                     GLIDSSGIRLYYTPELRKYDVGVLQTGIFTFPVHFIPPGAESYRSYGLCNSSQFDEMNGMLVPDLHVFAYLLHTHLSGRG   396
40 Red-crested turaco          GLIDSSGVRIYYTPELRKYDAGVLQTGVFTFPVHFIPPGAESYRSYGLCNSSQFDEINGMLVPDLHVFAYMLHTHLSGRG   397
41 MacQueen's bustard          GLIDSSGIRIYYTPELRKYDVGILQTGIFTFPVHFIPPGAESYRSYGLCNSSQFDEMNGMLVPDLHVFAYLLHTHLSGRG   397
42 Common cuckoo               GLIDSSGLQIYYTPELRKYDVGVLQTGIFTFPVHFIPPGAESYRSYGLCNSSQFDEMNGMLVPDMHVFAYLLHTHLSGRG   397
43 Chuck-will's-widow          GLIDSSGVRIYYTPELRKHDVGVLQTGIFTFPVHFIPPGAESYRSYGFCNSSQFDEMNGMLVPDLHVFAYLLHTHLSGRG   397
45 Flamingo                    GLIDSSGVRIYYTPELRKYDVGVLQTGIFTFPVHFIPPGAESYRSYGLCNSSQFDEMNGMPVPDLHVFAYLLHTHLSGRG   397
46 Great crested grebe         GLTDSSGVRIYYTPELRKYDVGVLQTGIFTSPVHFIPPGAESYRSYGLCNSSQFDEMNGMLVPDLHVFAYLLHTHLSGRG   400
50 Mallard                     GLIDSSGVRIYYTPEVRKYDVGILQTGIFTFPVHFIPPGAESYRSYGLCNSSQFDEMNGTPVPDLHVFAYLLHTHLAGRG   396
56 White-throated tinamou      GLIDSSGVRIYYTSELRKYDAGVLQTGIFTFPVHFIPPGAESYRSYGLCNTSQFDEVNGTPVPELHVFAYLLHTHLSGRG   397
57 African ostrich             GLIDSSGVRIYYTPELRKYDMGILQTGILTFPVHFIPPGAETYRSYGLCNTSQFDEMNGTPVTELHVFAYLLHTHLSGRG   397
                               .......330.......340.......350.......360.......370.......380.......390.......400
```

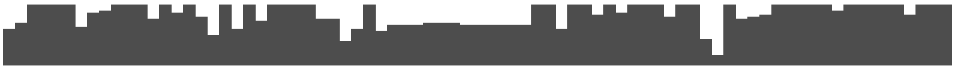

```
                               ::..**** : * *:****:*** ***.*: *: : ::.***:*.**.*:****   *:.* .* :***::**:***:**
 1 Rifleman                    VKVVQYRNGEQLRIICEDNKYDFRLQEIRDTKEILTIKPGDEILTECNFQTLDRSGVTFGGLSTMNEMCLAFLFYYPRNN   477
24 Barn owl                    VKAAQYRNGEQLRIICEDNNYDFRLQEIRDMKEILIIKPGDEILVECNFQTLDRSGVTFGGPSTMNEMCLTFLFYYPRNN   477
25 Turkey vulture              VKAAQYRNGEQLRIICEDNKYDFRLQEIRDTKEILIIKPGDEILVECNFQTLDRSGITFGGPSTMNEMCLTFLFYYPRNN   477
26 White-tailed eagle          VKAAQYRNGEQLRIICEDNKYDFRLQEIRDMKEILIIKPGDEILVECNFQTLDRSGITFGGPSTMDEMCLTFLFYYPRNN   477
27 Golden eagle                VKAAQYRNGEQLRIICEDNQYDFRLQEIRDMKEILIIKPGDEILVECNFQTLDRSGITFGGPSTMDEMCLTFLFYYPRNN   476
28 White-tailed tropicbird     VRAAQYRNGEPLGIICEDNKYDFRLQEIRDMKEILIIKPGDEILVECNFQTLDRSEITFGGPGTMNEMCLTFLFYYPRNN   477
30 Red-throated loon           VKAAQYRNGEQLGIICEDNKYDFRLQEIRDMKENLIIKPGDEILVECNFQTLDRSGITFGGPSTMNEMCLTFLFYYPRNN   477
33 Northern fulmar             LKAVQYRNGEQLVIICEDNKYDFGLQEIRNMKEIVIVKSGDEVLVECNFQTLDRSQSTFGGPSTMNEMCLTFLFYYPRNN   462
38 East African crowned crane  VKAAQYRNGEQLRIICEDNKYDFRLQEIRDIKEILIIKPGDEILVECNFQTLDRSGITFGGPSTMNEMCLTFLLYYPHNN   476
39 Hoatzin                     VKAAQYRNGEQLGIICEDNKYDFSLQEVRDMKETLTVRPGDEILVECNFQTLDRSGITYGGPSTMNEMCLTFLFYYPRNN   476
40 Red-crested turaco          LKAAQYRNGEQLRIICEDNKYDFKLQEIRDMKEIIIIKPGDEILVECNFQTLDRSGITFGGPSTMNEMCLTFLFYYPRNN   477
41 MacQueen's bustard          VKAVQYRNGEQLRIICEDNKYDFRLQEIRDMKEILIIKPGDEILVECNFQTLDRLKITFGGPSTMNEMCLTFLFYYPRNN   477
42 Common cuckoo               VKTVQYRNGKQLNIICEDNKYDFGLQEFRDMKEILIIKPGDEILVECNFRTLDRSEITFGGLSTMNEMCLTFLFYYPRNN   477
43 Chuck-will's-widow          VKAAQYRNGKQLGILCEDNKYDFRLQEIRDMKEILIIKPGDEILVECNFQTLDRSEITFGGPSTMNEMCLTFLFYYPRNN   477
45 Flamingo                    VKAAQYRNGEQLGIICEDNKYDFRLQEIRDMKEILTIKSGDEILVECNFQTLDRSEVTFGGPSTMNEMCLTFLFYYPRNN   477
46 Great crested grebe         VKAAQYRNGEQLGIICEDNKYDFRLQEIRDMKEILIIKPGDEILVECNFQTLDRAEITFGGPSTTNEMCFTFLFYYPRNN   480
50 Mallard                     VKVAQYRNEKQLGIICEDNKYDFTLQEIRDMKEILVIKPGDEILVECNFQTLDRSGITFAGPSTMNEMCLSFLFYYPRNN   476
56 White-throated tinamou      VKVAQYRNGEQLRIICEDNKYDFTLQEVRDMKKILTIKPGDEILVECSFQTLDRSVITFGGLSTMNEMCLTFLFYYPRNN   477
57 African ostrich             VKVAQYRNGEQLGIICEDNKYDFTLQEIRDTKEIVTIKPGDEILVECSFQTLDRSGITFGGPSTMNEMCLTFLFYYPRNN   477
                               .......410.......420.......430.......440.......450.......460.......470.......480
```

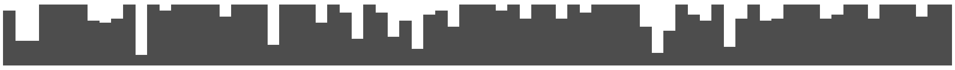

```
                               :****.:.***::.: :**:***. *.:*.:  :**:.:*:: **:.:***:*.: **.* * ::  :**:**:  ** .
 1 Rifleman                    ISSCMGYPDILYVAHTVKQEASDALEGMMAMNFVDWDDDTVKIAEKAAKEANQVVVIKTINELQRNESGLIRDISIPEQA   557
24 Barn owl                    ISSCMGYPDILYIAHVLKQEASDAVEGMMAMDFVDWDNDTVKIAEKAAKEANQVVMIKNINELQRSESGLIRDISIPEQA   557
25 Turkey vulture              ISSCMGYADILYIAHVLKQEASDAVEGMMAMDFVDWDNDTIKIAEKAAKEADQVVMIKTINELQRNESGLIRDMSIPERA   557
26 White-tailed eagle          ISSCMGFPDILYVAHALKQEASDAVEGMMAMEFVDWDNDTVKIAEKAAKEADQVVMIKTIDELQRNESGLIRDISMPERA   557
27 Golden eagle                ISSCMGFPDILYIAHALKQEASDAVEGMMAMEFVDWDNDTVKIAEKAAKEADQVVMIKTIDELQRNESGLIRDISIPERP   556
28 White-tailed tropicbird     MSSCMGYPDILYIAHVLKQKASDALEGMMAMGFVDWDNETVKIAEKAAKEADQVVMIKTINELQRNETGLIRDISIPERA   557
30 Red-throated loon           ISSCMGYPDILYVAHVLKQEASDVVEGMMAMDFVDWDNETVKIAEKAAKEADQVIMIKTINELQRNESGLIRDISIPERA   557
33 Northern fulmar             ISSCMGYPDILHVVHALKQEASDALEGMMAMDFVDWNNETVKIAEHAAKEADQFVIIKTIDELQRNETGLVRDIIIPERA   542
38 East African crowned crane  ISSCMGYPDILYIAHILKQEASDTVEGMMAMDYVDWHDTVKIAEKAAKEADQVVMIKTITELQRNESGLIRDISIPEQA   556
39 Hoatzin                     ISSCMGYPDILYIAHALKQEASDAVEAIMALDFVDWDNETVKIAEKVAKEADQVVMIKTINELQRNETGLIRDISIPEWS   556
40 Red-crested turaco          ISSCMGYPDILYIAQALKQEASDAVEGMMAMDFVDWDNETVKIAERAAKEADQVVMIKTINELQRNESGLIRDISIPERA   557
41 MacQueen's bustard          ISSCMGYPDILYIAHVLKQEASDAVEGMMAMDFVDWDNDTVKIAEKAAKEADQVVMIKTINELQRNESGLIRDISMPERA   557
42 Common cuckoo               ISSCMSYPDILYIAHELKQEASDAVEGMMAMYLVDWDNDTIKTAEKAAKEADQVVTIKTINELHRNESGLIRDISIPERA   557
43 Chuck-will's-widow          ISSCMGYPDILYVAHVLKQEASDAAEGMMAMEFVDWDNETVRIAEKAAKEADQVVMIKTINELQRNETGLIRDISIPERA   557
45 Flamingo                    ISSCMSYPDILYIAHVLKQEASDAAEGMMAMEFVDWDNETVRIAEKAAKEADQVVIIKTINELQRNESGLIRDISIPERA   557
46 Great crested grebe         ISSCMSYPDILYVAHVLKQEASDVVEGMMVMDFVDWDNETVKIAEKAAKEADQVVMIKTINELQRNESGLIRDISIPERA   560
50 Mallard                     ISSCMGYPDILYVAHVLKQEASDAVEGMMAMDFVDWNNETVKTAEKAAKEADQIVVIKTINEIQKNESGLIRDIHIPERA   556
56 White-throated tinamou      ISSCMGYPDILYIAHMLKQEASDTVEGMMAMDFIDWDNETIKTAEKAAKEANQVVVIKTINELQKIGSGLIRDMINPERA   557
57 African ostrich             ISSCMGYPDILYIAHILKQEASDTVEGMMAMDFVDWDNETIKIAEKATKEADQVVMIKTINEQQKNETGLIRDIIIPERA   557
                               .......490.......500.......510.......520.......530.......540.......550.......560
```

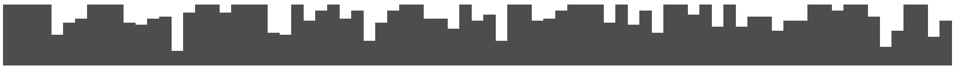

```
                                **.:*   . .                  .:.    **.:         **: :*:::*  :
 1 Rifleman                    ACHNISGHVSLSD------------LRVTAIDTSESSTTKETASLPLLSLTQLVFAWLILASEYEK   611
24 Barn owl                    ACHNISGQLSLSG------LRATANLHLTSVCTSESSATTEIASLPLISLTHLVFACLILASDYGK   617
25 Turkey vulture              ACHNISGHLSLSH------LRATANLRLTAVCTSESSTTKETASLPLLSFTQLVFPWLILASEYGK   617
26 White-tailed eagle          ACHNISGHLSLSG------RRATANLRLTAVCTSESPTTKETAILPLLSLTQLVFSWLILASEYGK   617
27 Golden eagle                ACHNISGHLSLSG------LRATANLRLTAVCTSESPTTKETAIRPLLSLTQLVFSWLILASEYGK   616
28 White-tailed tropicbird     ACHNISGHLSLSG------LRATANLRLTAVCTSESSTTKETASLPLLSLTQLVFAWLILASEYGK   617
30 Red-throated loon           ACHNISGYLSLSG------LKATANLRLTAVCTSESPATKETASHPLLSLTQLVFAWLILSSEHGK   617
33 Northern fulmar             ACHNLSGHLSLPG------LKAAANLRLTAVRTSESSTTKETASLPLLSLTQLVFAWLILASEYGK   602
38 East African crowned crane  ACHNISGHLSLSG------LRSTANVRLTALCTSESSTTKETASLPLLSLTQLVFAWLTLASEYKK   616
39 Hoatzin                     PCHNISGHLSISGHLSPSGLRAATNPCLTAVCTSESSTLRETASPPLLPLTQLVFSWLVLASEYGK   622
40 Red-crested turaco          TCHNISGHLSLSG------LRATANLCLTAVHTPESSTTKETASFPLLSLTQLVFAWLLLAS----   613
41 MacQueen's bustard          ACHNISGHFPLSG------LRATANLRLTAVCTSESSTNKRTTSLPLLSLTQLVFAWLILASVYGK   617
42 Common cuckoo               ACHNISGHLSLSG------PRASANLRLTAACTLESPATKGTASLPLLSLTQLVFVWLILTSEYGQ   617
43 Chuck-will's-widow          ACHNISGYLSLSH------LRATANLRLTAVCASESSTTKETASLPLLYLTQLVFAWLILAPEHGK   617
45 Flamingo                    ACHNISGHRSRSG------LRATANLRLTAACSSESSTTKESSSLPLLSLTQLVFAWLVLASEYGK   617
46 Great crested grebe         ACHSISGHLSLSG------LRATANLRLTGVWTSESSATKGNPSLPLLSLTQLVFAWLVLASEYGK   620
50 Mallard                     VCHNVSVQQTLSD------LRATANLRLTAVHNFESSTTKEFVSLPLLFLTQLLFAWIIFSSEYRK   616
56 White-throated tinamou      VCHSISENISLS-------LKAATNLNFAAVLRSESSTTKEMASLPLLSFTQILFACLLLSSELRM   616
57 African ostrich             ACHNISENLSLSG------LRAATNLRLAAVCRSESSTTKEMSSLPLLSLTQLVFAWLI-------   610
                               .......570.......580.......590.......600.......610.......620......
```

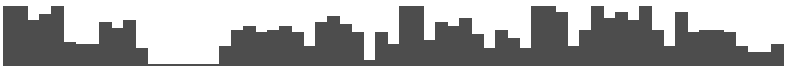

Supplement: S2 Fig — (PDF) [file pone.0152431.s002.pdf]
