# Supplementary material for: MOXD2, a Gene Possibly Associated with Olfaction, Is Frequently Inactivated in Birds
Source: PLoS One. 2016 Apr 13;11(4):e0152431. doi: 10.1371/journal.pone.0152431 (PMC4830563; doi:10.1371/journal.pone.0152431)
Supplement: S3 Fig — (PDF) [file pone.0152431.s003.pdf]

S3 Fig. Alignment of selected exons with point mutations

- Open reading frame disrupting mutations are marked on red background
- Start codon (ATG), splice donor (gt), and splice acceptor (ag) sequences are on yellow background
- Exon sequences are in uppercase letters
- Amino acid sequence of the rifleman MOXD2 protein is shown at the top

---

A. Multiple alignment of exon 1 sequences from 11 birds

```
 1 Rifleman (aa)                                           M  M  A  I  V  F  S  R  I  K  R
 1 Rifleman                    gactcaggaattgcagtggtgctgttgATGATGGCAATTGTCTTCTCGAGAATCAAGAGG    60
 2 Golden-collared manakin     gactcagaaattgaagtggtactgttggtgATGGCAGTGTCCTTCTCAAGAATCCAGGGT    60
 3 American crow               gactcagaaactgcag---------tgATGATGGTGATGCTCTTCTCAGGGATCAAGAGA    51
 4 Hooded crow                 gacacagaaactgcag---------tgATGATGGTGATGCTCTTCTCAGGGATCAAGAGA    51
 5 Ground tit                  gtaccagagactgcag---------agATGATAGTGATGTTCTTTTCAGGAATCAAGAGA    51
 6 Collared flycatcher         gactcagagactgcag---------agATGATGGTTTTGTTCTTCTCAGGAATCAAGAAA    51
 7 Zebra finch                 gactcagagactgcag---------agATGATGGTGATC---TTTTCAGGAATCAAGAGA    48
 8 Atlantic canary             gactcagagactgcag---------agATGATGGTGATGTTCTACTCAGGAATCAAGAGA    51
 9 Medium ground finch         gacttggagactg-ag---------agATGATGTTGATGTTCTTCCCAGGAATCAAGAGA    50
10 White-throated sparrow      gactcagagactgcag---------agattATGGTGATGTTCTTCTCAGAAATCAAAAGA    51
47 Rock pigeon                 gactccgaaattgcagtggtcctggtgataATGGCAGTTCTTTTCTCAAGTATCAAGGGT    60
48 Yellow-throated sandgrouse  gactcagaaactgccgtggtcctgttgATGATGGCAGTTGTCTTCTCAAGAACCAAGGGT    60
                               *     *  * **  *          * * **     *    *   *    * * *

 1 Rifleman (aa)               M  F  F  L  L  F  F  P  C  F  C  S  G  Q  L  A  P  P  P  L
 1 Rifleman                    ATGTTCTTCCTCTTGTTCTTCCCATGCTTTTGTTCTGGTCAGCTTGCACCTCCACCGCTG   120
 2 Golden-collared manakin     ATGCTCTTCCTCTTGTTCC-----------------GGTCAGCCTGCAGCTCCACTACTG   103
 3 American crow               ATGCTCTTCCTCTTGTTCCTCCCATGCTTTTGTTCTGGCCAGCTTGCACCTCCACTGCTG   111
 4 Hooded crow                 ATGCTCTTCCTCTTGTTCCTCCCATGCTTTTTTTCTGGCCAGCTTGCACCTCCACTGCTG   111
 5 Ground tit                  ATGCTCTTCCTCTTGTTCCTCCCATGCTCTTGTTCTGGCCAGCTTGCATCTCTGCTGCTG   111
 6 Collared flycatcher         ATGCTCTTCCTCTAGTTCCTCTCATGCTTTTGTTCTGGCCTGCTTGCATCTCCCCTGCTG   111
 7 Zebra finch                 ATGCTCTTCCTTTTGTTCT---CTTGCCTCTGTTCTGGCCAGCTTGCATCTCCACTGCTG   105
 8 Atlantic canary             CTGCTCTTCCTCTTTTTCCTTCCATGCTTTTGTTCTGGCCAGCTTGCATTTCCACTGCCG   111
 9 Medium ground finch         ATGCTCTTCCTCTTGTTCCTCCCATGCTTTTGTTCTGGCCAGCTTGCATCTCCTCTGATG   110
10 White-throated sparrow      ATGCTCTTCCTCTTGTTCCTCCCATGCTTTTGTTCTGGCCAGCTTGCATTTCCACTGCTG   111
47 Rock pigeon                 ATGCTCTTCCTCTTGCTCCTCCCATACTTTTGTTTTGGTCAGCCTGCACCTTCACTGCTG   120
48 Yellow-throated sandgrouse  ATGCTCTTCCTCTT-TTCTCCCCATGCTTTTATTATGATCAGCCTGCAACTCCATAGCTG   119
                                ** ******* *   **                  *  * ** ****  *        *

 1 Rifleman (aa)               R  F  S  T  F  L  D  P  S  N  M  V  Y  L  H  W  D  H  D  D
 1 Rifleman                    CGTTTCTCCACTTTCCTGGATCCTTCCAACATGGTCTACCTCCACTGGGACCATGATGAT   180
 2 Golden-collared manakin     CATTTCTCCACCTCCCTGGATCCTTCCAACACAATCCACCTCCGCTGGGACCATGATGAA   163
 3 American crow               CGTTTCCCCACCTTATTGGATCCTTCCAGCATGTTCTACCTCCACTGGGACCACGAGGAA   171
 4 Hooded crow                 CGTTTCCCCACCTTATTGGATCCTTCCAGCATGTTCTACCTCCACTGGGACCACGAGGAA   171
 5 Ground tit                  AGTTTCCCCACCTTCCTGGATCCTTCCAGCATGTTCTACCTCCGCTGGCACTATAAGGAA   171
 6 Collared flycatcher         CGTTTCCCCACCTTCCTGGATCCTTCCAGCATGTTCTACCTCTGCTGGGACCACAAGGAA   171
 7 Zebra finch                 CGTTTCTCCACCTTCCTGGATCCTTCTAGCATGTTCCACCTCTGCTGGGACCATGAGGAA   165
 8 Atlantic canary             CGTTTCCCCACCTTCCTGGATCCTTCCAGCATGTTCTACCTCTGCTGGGACCATGAAGAA   171
 9 Medium ground finch         CTTTTCCCCACCTTCCTGGATCCTTCCAGCATGTTCTACCTCTGCTGGGAGCACAAGGAA   170
10 White-throated sparrow      CATTTCCCTGCCTTCCTGGATCCTTCCAGCATGTTCTGCCTC-----------TGAGGAA   160
47 Rock pigeon                 CATTTCTCCATCTTCCTGGAGCCTACAATCATGGTTTACCTCTGCTGAGACCATGACGAA   180
48 Yellow-throated sandgrouse  CGTTTCTCCATCTTCCTGGATCCTTCAAACATGGTCTACCTCCGCTGGGACCATGACGAA   179
                                 **** *    *   **** *** * * **   *   ****             * **

 1 Rifleman (aa)               Q  E  L  M  T  F  E  L  Q  V  H  T  T  G  W  V  A   F  G  F
 1 Rifleman                    CAGGAGCTGATGACATTTGAGCTGCAGGTCCATACAACTGGCTGGGTGGCA-TTTGGATT   239
 2 Golden-collared manakin     CAGGAGCTGATGACATTTGAGCTGCAGGTCCATACAACTGGCTGGGTGGCA-TTTGGATT   222
 3 American crow               CAGGAGCTGATGAGG-TTGAGCTGCACATCCACACAACTGGCTGGATGGCA-TCTGGATT   229
 4 Hooded crow                 CAGGAGCTGATGAGG-TTGAGCTGCACATCCACACAACTGGCTGGATGGCA-TCTGGATT   229
 5 Ground tit                  CAGGAGCTGATGGGG-TTGAGCTGCACATCCACACAATTGGCTGGGTGGCA-CATGGA--   228
 6 Collared flycatcher         CAGGAGCTGATGGGG-TTGAGCTGCACATCCACACAACTGGCTGGGTGGCA-TCTGGATT   229
 7 Zebra finch                 CAGGAGCTGATGGGG-TTGAGCTGCACATCCAC---GCTGCCTCATCCACACTGTAGATT   221
 8 Atlantic canary             CAGGACCTGATGGGG-TTGAGCTGCACATCCAC---ACGGGCTGGGTGGCA-CGTGGATT   226
 9 Medium ground finch         CAGGAGCTGATGGGG-TTGAGCTGCACATCCAC---ACTGGGTGGATGGCA-TGTGGATT   225
10 White-throated sparrow      CAGGAGCTGATGGGG-CTGAGCTGCACATCCAC---ACTGGCTGGGTGGCA-TGTGGATT   215
47 Rock pigeon                 CAGGAGGTGATGACATTTGAGCCGCAAGTCCATACAACTGGCTGGGTGGCA-TTTGGATT   239
48 Yellow-throated sandgrouse  CAGGAGCTGATGAAGTTTGAGCTGCAGGTCCATACGACTGGCTGGGTGGCA-TTTGGATT   238
                               *****  *****     ***** ***  ****       *  *      **   * **
```

```
 1 Rifleman (aa)                S  P  H  G  E  L  P  G  S  D  I  V  I  G  G  V  F  P  N  G
 1 Rifleman                    CAGCCCTCATGGAGAGTTGCCTGGATCTGACATTGTGATAGGAGGTGTCTTCCCAAATGG   299
 2 Golden-collared manakin     CAGCCCTCATGGAGAGTTGCCTGGATCTGACATTGTGACAGGAGGTGTCTCCCCAAATGG   282
 3 American crow               CAGCCCTCATGGAGAGCTGCCTGGATATAACATTGTGACAGGAGGTGCCTCCCCAAATGG   289
 4 Hooded crow                 CAGCCCTCATGGAGAGCTGCCTGGATATGACATTGTGACAGGAGGTGCCTCCCCAAATGG   289
 5 Ground tit                  GAGCCCTCATGGAGAGCTGCCTGGATCTGACACTGTGTCAGGAGATGCCTACCGGAGTGG   287
 6 Collared flycatcher         CAGCCCTCATGGAGAGCTGCCTGGGTCTGACGTTGTGACAAGAGGTGCCTTCCAAAAGGG   289
 7 Zebra finch                 CAGCTATCATGGAGAGCTGCCTGGATCTGACATTGTGACAGGAGGTGCTTTCTGAAATGG   281
 8 Atlantic canary             CAGCCCTCACAGAAAGCTGCCTGGATCTGACATTGTGACAGGAGGTGTCTCCTGAAATGG   286
 9 Medium ground finch         CAGCCCTCATGGAGAGCTGCCTGCATCTGACATTGTGAGAGGAGGTGCCTTCTAAAATGG   285
10 White-throated sparrow      CAGCCCTCATGGAGAGCTGCCTGGATCTGACATTGTGCCAGGAGGTGCCTTCTGAAATGG   275
47 Rock pigeon                 CAGCCCTCATGGAGAGTTGCCTGGATCTGACATTGTGATAGGAAGTATCTTCCCCAATGG   299
48 Yellow-throated sandgrouse  CAGCCCTCACGGAGAATTGCCTGGATCTGATACTGTGATAGGAGGTGTCTTCCCAAATAG   298
                                ***  ***  ** *  ******  * * *   ****  * **  *   * *   *   *

 1 Rifleman (aa)                S  I  Y  F  S
 1 Rifleman                    CAGCATCTACTTCTCTgtaagttgaaaatacttcctctgcatcccc                 345
 2 Golden-collared manakin     CAGCATCTACTTCTCTggaagttgaaaagacttcctctgcaggctc                 328
 3 American crow               CAGCATCTGATTCTCTgtaagttgaacagatttcctcttcatcccc                 335
 4 Hooded crow                 CAGCATCTGATTCTCTgtaagttgaacagatttcctcttcatcccc                 335
 5 Ground tit                  CAGCTTCTGCTTCCCTgtaagctgaacagatttcctcttcaacccc                 333
 6 Collared flycatcher         CAGCATCTGCTTCTCTgtaagttgaacaggtttcctcttcgtccca                 335
 7 Zebra finch                 CAGCATCTGCATCTCTgtaagttgaacagatatcctcttcatcctc                 327
 8 Atlantic canary             CGGCATCTGCTTCTCTgtaagtggaacatagttcctcttcatctcc                 332
 9 Medium ground finch         CAGCATCTGCTTCTCTgtaagttgaacagatttcctcttcaccccc                 331
10 White-throated sparrow      CAGCATCTGCTTCTCCgtaagatgagcagattccctcttcatcccc                 321
47 Rock pigeon                 CAGCATCTACTTCTCCgtaagttgaaaagacttcctctgcgtccct                 345
48 Yellow-throated sandgrouse  CAGCATCTACTTCTCTgtaagctgaaaaacttcctctgcatcctc                 344
                               * ** ***   ** * * ***  **  *    ***** *
```

B. Multiple alignment of exon 3 sequences from 3 birds

```
 1 Rifleman (aa)                                M  D  T  A  H  L  V  A  A  F
 1 Rifleman       agatttgccagtgaaacacttccatttcagATGGACACAGCCCATCTTGTTGCTGCATTT    60
35 Crested ibis   agactttccaatgaaacactcccattacagATGGACACAGCATGCCTTGTTACTGCCTTT    60
36 Little egret   agagtttccagtg-aatgttcccattactgATGGATACAGCACACTTCATTACTGCCTTT    59
                  *** ** *** ** **   * ***** * ****** *****     *  ** **** ***

 1 Rifleman (aa)  G  T  D  D  T  V  Q  F  F  K  S  Q  R  F  S  R  S   L  F  L
 1 Rifleman       GGCACTGATGACACAGTCCAATTCTTTAAAAGCCAAAGATTTTCCAGATCT-CTTTTCCT   119
35 Crested ibis   TGTGCTGATGACACAGTCCAATTCTTTAAAGGCCAAAGATTTTCCAAGTCTTTTTTTCTT   120
36 Little egret   GGTACTTATGACACAGTCTAATTCTTTAAAGGCCAAAGATTTTCTAAATGT-CTTTTCTT   118
                   *  ** *********** *********** ************* *  * *  ***** *

 1 Rifleman (aa)   M  R  Y  K  S  P  S  D  S  T  D  P  K  V  F  F  T  Y  D  L
 1 Rifleman       GATGAGGTACAAAAGCCCATCTGACTCAACTGACCCCAAAGTATTCTTCACCTATGACCT   179
35 Crested ibis   GATGAGGTACAGAGGCCCATCTGACTCAACTGACCCCAAAATATTCTTCACCTATGACCT   180
36 Little egret   GATGAGGTACAGAGGCCCATCTGACCCAACTGACCCCAAAATAATCTTCACCTATGACCT   178
                  *********** * *********** ************** ** ****************

 1 Rifleman (aa)   R  L  D  N
 1 Rifleman       GAGGCTGGACAACgtaagtattggacaagaactttgtttagta                    222
35 Crested ibis   GAGGCTGGACAATgtaagtactggacaagagctttacttggca                    223
36 Little egret   GAGGCTGGACAACataagtattggacaagagcattacttggca                    221
                  ************  ****** ********* * **  ** * *
```

C. Multiple alignment of exon 4 sequences from 11 birds

```
 1 Rifleman (aa)                          F  A  V  P  V  E  E  T      T  Y  A  C  T
 1 Rifleman             cgtgtgtttcttttccagTTTGCTGTTCCAGTAGAAGAAACG----ACGTATGCCTGTAC    56
11 Kea                  tgtgtgtttctttttcaaTTTGCCATTCCAGTTGAAGAAATCACATACATATCCCTGTAC    60
12 Budgerigar           catgtgtttcttttccatTTTGCTGTTCTGGCTGAAGAAACC----ATATATGCCTGTAT    56
13 Puerto Rican Amazon  tgtgtgtttcttttccatTTTGCTGTTCCAGCTGAAGAAACC----CTATATGCCTGTAC    56
14 Scarlet macaw        tatgtgtttcttttccatTTTGCTGTTCCAGTTGAAGAAACC----CTATATGCCTGTAC    56
15 Saker falcon         tgtgtctttcttttccagTTTGCTGTTCCAGTT-AAGAAACC----AAGTATGCTTGTAC    55
16 Peregrine falcon     tgtgtctttcttttccagTTTGCTGTTCCAGTT-AAGAAACC----AAGTATGCTTGTAC    55
22 Cuckoo roller        tgtgtgtttcttttccagTTTGCCATTCTAGCTGAAGAAACC----AAATATGCATGTAC    56
23 Speckled mousebird   tgggtgtttcttttccaaTTTGCTGCTCCAGTTGAAAAAACC----TAGCATGTCTGTAC    56
29 Sunbittern           tgtctgtttcttttccagTTTGTTGTTCCTGTTGAAGAAAAC----AAGTATGCCGGTGC    56
34 Great cormorant      tgtgtgtgtctttt-----------TTCCAGTTGAAGAAACC----AAGTGTGCCTGTAC    45
                            * * ******            **  *   ** ***           *    **

 1 Rifleman (aa)         F  I  P  L  P  M  V  K  Q   K  H  H   I  Y  K
 1 Rifleman             CTTTATCCCACTGCCCATGGTCAAGCAG-AAACACCAC-ATCTACAAGgtaaactcacag   114
11 Kea                  CTTTATCCCACTGCCCACTGTCAAGCAG-AAACACCATTATCTACAAGgtaaatttgcag   119
12 Budgerigar           CTTTATCCCATT-------GTCAAGCAG-AAACACCATTATCTACAAGgtaaatttgcag   108
13 Puerto Rican Amazon  CTTTATCCCACT-------GTCAAGCAG-AAACACCATTATCTACAAGgtaaatttgcaa   108
14 Scarlet macaw        CTTTATCCCACT-------GTCAAGCAG-AAACACCATTATCTACAAGgtaaatttgcaa   108
15 Saker falcon         TTTTATCCCCCTGCCCATGGCCAAGCAG-AAACACCAT-GTCTACAAGgtaaaattgcag   113
16 Peregrine falcon     TTTTATCCCCCTGCCCATGGCCAAGCAG-AAACACCAT-GTCTACAAGgtaaaattgcag   113
22 Cuckoo roller        CTTTATCCCACTGCCCATGGTC-AGCAG-AAACACCGT-ATCTACAAGgtaaacttgcag   113
23 Speckled mousebird   CTTTATGCCACTGACCATGGCCAAGCAG-AAACACTAT-TAT--CAAGgtaaatttgcag   112
29 Sunbittern           CTTTATCCCACTGCCTGTGGTCGAGCAGAAAACACCGT-ATCTACAAGgtaaacttgcag   115
34 Great cormorant      CTTTATTCCACT-------GCCAAGCAG-AGACACCAC-GTCTACAAGgtaaacttgcag    96
                         ***** **  *       * * ***** * ****         ********* *  **
```

D. Multiple alignment of exon 5 sequences from 3 birds

```
 1 Rifleman (aa)                                      F  E  P  V  I  T  S  H  N  I
 1 Rifleman            aatatttttgactctttttcttggttacagTTTGAACCTGTAATAACATCCCACAATATA    60
17 Red-legged_seriema  ataattttttcctctttctctttgttacagTTTGAACCTGTAATAACATGCCACAACATC    60
21 Bar-tailed_trogon   actttctctgactctttctcttggtttcagTTCGAACCTGTACTAACACCCCACAACATC    60
                       *   * * *  ****** **** *** ***** ********* *****  ****** **

 1 Rifleman (aa)       T  L  V  H  H  I  L  V  Y  A  C  G  N  S  S  I  L  P  S  G
 1 Rifleman            ACCTTGGTTCATCATATTCTTGTTTATGCCTGTGGCAACTCCAGCATCCTACCCAGTGGC   120
17 Red-legged_seriema  ACCTTGGGTCATCATATTCTTGTTTATGCCTGTGGCAACGCCAGCGTGTTACCCAGTGGC   120
21 Bar-tailed_trogon   ACCTTGGTTCATCACATTCTTGTTTATGCCTGCGGCAGCTCTGGCGTGTTGCCCAGTGGC   120
                       ******* ****** ***************** **** * *  ** *  * *********

 1 Rifleman (aa)       I  G  D  C  Y  G  A  N  P  D  F  S  L         C  S  Q  V  L
 1 Rifleman            ATAGGTGATTGCTATGGAGCCAATCCAGATTTTTCCCT-------GTGCTCTCAGGTCCT   173
17 Red-legged_seriema  ATAGACGATTGCTATGGAGCCAAGCCAGATTTTGCCCT-------GTGCTGTCAGGTGCT   173
21 Bar-tailed_trogon   ACAGATGATTGCTATGGAGCCAACTCAGATTTTACCCTCAGATTAGTGCTCTCAGGTTCT   180
                       * **  *****************  ******** ****       ***** ****** **

 1 Rifleman (aa)        V  G  W  A  V  G  G  E
 1 Rifleman            TGTGGGCTGGGCTGTTGGAGGAGAGgtgagtctgcagggcccattcccttcccta        228
17 Red-legged_seriema  TGTGGGCTGAGGGACTGGAGGAGAGgtgagtctgcagaacccattcccttcccca        228
21 Bar-tailed_trogon   TGTGGGCTGGGCTGTTGGGAGAGAGgtgaatctgtggggcccattcctttctcca        235
                       ********* *    *** *********  ****  *  ******** *** * *
```

E. Multiple alignment of exon 7 sequences from 4 birds

```
 1 Rifleman (aa)                                         L  I  D  S  S  G  I  R  I
 1 Rifleman             ttgctaaaggagaggtcatttttctttttacagGCTTGATTGACAGCTCAGGGATACGAAT    60
20 Rhinoceros hornbill  tggtcattgctgaggtaatatttcttttacagCCTTGATTGACAGCTCAGGGGTACGAAT    60
44 Anna's hummingbird   atgatgaagaagtggcaatttttgtttttacagATTTGATTGACAGTTCAGGGGTATGAAT    60
49 Brown mesite         cagctgaaggagtaggcatttttctttttacagGTTTGATCGACAGCTCAGGGGTACAAAT    60
                          *     *  *  *  ** *** ********  ***** ***** ****** **  ***

 1 Rifleman (aa)         Y  Y  T  P  E  L  R  K  Y  D  V  G  V  L  Q  T  G  V  F  I
 1 Rifleman             CTACTACACACCAGAGCTACGGAAATATGATGTGGGGGTTCTGCAAACAGGTGTCTTCAT   120
20 Rhinoceros hornbill  CTACTATACTCCGGAGATACGAAAATATGATGTTGGGATTCTGCAAACAGGCATCTTCAT   120
44 Anna's hummingbird   --------CACCAGAGCTACGGAAATATGATGTGGGGATTCTGCAAACAGGTGTCTTCAT   112
49 Brown mesite         CTAAAACACTTCAGAGCTACGGAAATATGATGTGGGGGTTCTGCAAACAGGCATCTTCAC   120
                                *  * *** ****  *********** ***  *************  ******

 1 Rifleman (aa)         F  P  A  H  F  I  P  P  G  A  E  S  Y  R  S  Y  G  L  C  N
 1 Rifleman             TTTCCCTGCACATTTCATTCCTCCTGGAGCAGAATCCTACAGATCTTACGGTCTTTGCAA   180
20 Rhinoceros hornbill  TTT-CCTGTGCATTTCATTCCTCCTGGGGCACAATCCTACAAATCTTACGGCCTTTGCAA   179
44 Anna's hummingbird   TTTCCCTGTGCATTTCATTCCCCCTGGAGCAGAATCCTACAGATCTTATGGACTTTGCAA   172
49 Brown mesite         TCTCCCTGTGCATTTCATTCCTCTTGGAGCAGTATCCTACAGATCTCATGGTCTTTACAA   180
                        * * ****  *********** * *** ***  ******** **** * ** **** ***

 1 Rifleman (aa)         S  S  Q  F  D  E
 1 Rifleman             TTCCAGCCAGTTCGACGAAgtgagtgtggggaaagccatacaaaattct             229
20 Rhinoceros hornbill  TTCCAGCCAGTTTGATGAAgtgagtgtggggaaagccatac-aaattct             227
44 Anna's hummingbird   TTCCAGCCAGTTTGATGAAgtgagtgtggggacagccatacgagatcct             221
49 Brown mesite         TTCCAGCCAGTTTGATGAAgtgagtgtggggaaagacatgcaaaatcct             229
                        ************ ** ************** ** *** * * ** **
```

F. Multiple alignment of exon 8 sequences from 3 birds

```
 1 Rifleman (aa)                                   M  N  G           M  L  V  P  D
 1 Rifleman         ggctctgatgttctctgttttgaactgcagATGAATGGG-------ATGCTGGTTCCAGA    53
31 Emperor penguin  gactctgacctacttcatttgaactgcagATGAATGGTTCCAGATCTGCTGGTTCCAGA    60
32 Adelie penguin   gactctgacctacttcatttgaactgcagATGAATGGG-------ATGCTGGTTCCAGA    53
                    * ******  * **   *********************         *************

 1 Rifleman (aa)      L  H  V     F  A  Y  L  L  H  T  H  L  S  G  R  G  V  K  V
 1 Rifleman         TCTCCATGTC--TTTGCCTATCTGCTTCACACTCACCTGTCTGGCAGAGGAGTGAAAGTC   111
31 Emperor penguin  TCTGCATGTC--TTTGCCTACTTGCTTCATACCCACCTGTCTGGCAGAGGAGTGAAAGCT   118
32 Adelie penguin   TCTGCATGTCTTTTTGCCTACTTGCTTCATACCCACCTGTCTGGCAGAGGAGTGAAAGCT   113
                    *** ******  ********  ******* ** *************************

 1 Rifleman (aa)    V  Q  Y  R
 1 Rifleman         GTTCAATACCGgtaagagaaggaaacac--agtattgctggac                     152
31 Emperor penguin  GCTCAATACCGgtaagagaagcaaactc--ggtattgctgcct                     159
32 Adelie penguin   GCTCAATACCGgtaagagaagcaaactcatagtgttgctgcct                     156
                    * ******************* **** *   ** ******
```

G. Multiple alignment of exon 9 sequences from 2 birds

```
 1 Rifleman (aa)                                          N  G  E  Q  L  R  I  I  C  E
 1 Rifleman                    cacacttctccctttgtttgtcctccccagGAACGGTGAGCAGCTGAGGATCATCTGTGA    60
19 Northern carmine bee-eater  taactttcttcccttctctgtctttcccagGAATGGTGAGCAGCTAAGGATCATCTGTGA    60
                                 *    **** ** ** * **** * ******** *********** **************

 1 Rifleman (aa)                D  N  K  Y  D  F  R  L  Q  E  I  R  D  T  K  E  I  L  T  I
 1 Rifleman                    GGACAATAAGTATGACTTCAGGCTGCAGGAAATTCGGGACACGAAGGAAATCCTCACAAT   120
19 Northern carmine bee-eater  GGACAATAAGTATGACTTCAGG-------ACATTGAGGACATGAAGGAAATTGTGATAAC   113
                               **********************       * ***   ***** ********  * * **

 1 Rifleman (aa)                 K  P
 1 Rifleman                    TAAACCAgtatgatcctctcctgggatgtagcactgg                          157
19 Northern carmine bee-eater  CAAACCAgtaagatccttccctgtgacacagccacgg                          150
                                ********* ******  **** **   ***   **
```
