# Supplementary material for: MOXD2, a Gene Possibly Associated with Olfaction, Is Frequently Inactivated in Birds
Source: PLoS One. 2016 Apr 13;11(4):e0152431. doi: 10.1371/journal.pone.0152431 (PMC4830563; doi:10.1371/journal.pone.0152431)
Supplement: S4 Fig — (PDF) [file pone.0152431.s004.pdf]

S4 Fig. WGS sequence assemblies of selected exons

A. Golden-collared manakin (No. 2), exon 1, WGS data

- CAP3 alignment of WGS data spanning exon 1 (uppercase letters)

```
                             .    :    .    :    .    :    .    :    .    :    .    :
SRR946955.63931851.2+                        tgaagtggtactgttggtgATGGCAGTGTCCTTCTCAAGA
SRR946955.104117134.2+                                          ATGGCAGTGTCCTTCTCAAGA
SRR946955.28016599.1+                                           ATGGCAGTGTCCTTCTCAAGA
SRR946955.72129725.1+                                                           CAAGA
                         ____________________________________________________________
consensus                                    tgaagtggtactgttggtgATGGCAGTGTCCTTCTCAAGA

                             .    :    .    :    .    :    .    :    .    :    .    :
SRR946955.63931851.2+    ATCCAGGGTATGCTCTTCCTCTTGTTCCGGTCAGCCTGCAG
SRR946955.104117134.2+   ATCCAGGGTATGCTCTTCCTCTTGTTCCGGTCAGCCTGCAGCTCCACTACTGCA
SRR946955.28016599.1+    ATCCAGGGTATGCTCTTCCTCTTGTTCCGGTCAGCCTGCAGCTCCACTACTGCATTTCTC
SRR946955.72129725.1+    ATCCAGGGTATGCTCTTCCTCTTGTTCCGGTCAGCCTGCAGCTCCACTACTGCATTTCTC
SRR946955.44534172.2+     TCCAGGGTATGCTCTTCCTCTTGTTCCGGTCAGCCTGCAGCTCCACTACTGCATTTCTC
SRR946955.58346422.1+        AGGGTATGCTCTTCCTCTTGTTCCGGTCAGCCTGCAGCTCCACTACTGCATTTCTC
SRR946955.1407940.2+            GTATGCTCTTCCTCTTGTTCCGGTCAGCCTGCAGCTCCACTACTGCATTTCTC
SRR946955.112874748.2+           TATGCTCTTCCTCTTGTTCCGGTCAGCCTGCAGCTCCACTACTGCATTTCTC
SRR946957.52145586.1+                                                      TCCACTACTGCATTTCTC
SRR946957.74253506.1+                                                       CCACTACTGCATTTCTC
SRR946957.71782474.2-                                                        CACTACTGCATTTCTC
SRR946955.104117134.1-                                                         CTACTGCATTTCTC
SRR946957.84913099.2-                                                          CTACTGCATTTCAC
SRR946957.103208127.1-                                                          TACTGCTTTTCTC
SRR946958.69296965.2+                                                            ACTGCATTTCTC
SRR946955.63931851.1-                                                              TGCATTTCTC
SRR946960.106834680.1-                                                             TGCATTTCTC
SRR946957.59413978.1-                                                               GCATTTCCC
SRR946958.16186972.2-                                                               GCATTTCTC
SRR946957.117350774.1+                                                               CATTTCTC
SRR946957.94635362.2-                                                                CATTTCTC
SRR946960.37232613.2+                                                                CATTTCTC
SRR946958.1915339.2+                                                                  ATTTCTC
SRR946960.2459542.2+                                                                  ATTTCTC
SRR946960.29865046.1-                                                                  TTTCCC
SRR946960.3126220.2+                                                                    TTCTC
SRR946957.73860379.2-                                                                     CTC
SRR946957.94172165.2-                                                                     CTC
SRR946958.69981891.1+                                                                     CTC
SRR946958.83070100.1-                                                                     CTC
                         ____________________________________________________________
consensus                ATCCAGGGTATGCTCTTCCTCTTGTTCCGGTCAGCCTGCAGCTCCACTACTGCATTTCTC

                             .    :    .    :    .    :    .    :    .    :    .    :
SRR946955.28016599.1+    CACCTCCCTGGATC
SRR946955.44534172.2+    CACCTCCCTGGATCCTTCCAA
SRR946955.1407940.2+     CACCTCCCTGGATCCTTCCAACACAAT
SRR946955.112874748.2+   CACCTCCCTGGATCCTTCCAACACAATC
SRR946955.72129725.1+    CACCTCCCTGGATCCTTCCAACACAATCCACCTCC
SRR946955.58346422.1+    CACCTCCCTGGATCCTTCCAACACAATCCACCTCCGCTGGGACC
SRR946955.104117134.1-   CACCTCCCTGGATCCTTCCAACACAATCCACCTCCGCTGGGACCATGATGAACAGGAGCT
SRR946955.63931851.1-    CACCTCCCTGGATCCTTCCAACACAATCCACCTCCGCTGGGACCATGATGAACAGGAGCT
SRR946957.103208127.1-   CCCCTCCCTGGATCCTCCCAACACAATCCACCTCCGCTGGGACCATGATGAACAGGAGCT
SRR946957.117350774.1+   CACCTCCCTGGATCCTTCCAACACAATCCACCTCCGCTGGGACCATGATGAACAGGAGCT
SRR946957.52145586.1+    CACCTCCCTGGATCCTTCCAACACAATCCACCTCCGCTGGGACCATGATGAACAGGAGCT
SRR946957.59413978.1-    CCCCCCCCTGGATCCTTCCAACACAACCCCCCTCCGCTGGGACCATGATGAACAGGAGCT
SRR946957.71782474.2-    CCCCTCCCTGGATCCTTCCAACACAATCCACCTCCGCTGGGACCATGATGAACAGGAGCT
SRR946957.73860379.2-    CCCCTCCCTGGATCCTTCCAACACAATCCACCTCCGCTGGGACCATGATGAACAGGAGCT
SRR946957.74253506.1+    CACCTCCCTGGATCCTTCCAACACAATCCACCTCCGCTGGGACCATGATGAACAGGAGCT
SRR946957.84913099.2-    CACCTCCCTGGATCCTTCCAACACAATCCACCTCCGCTGGGACCATGATGAACAGGAGCT
SRR946957.94172165.2-    CCCCTCCCTGGATCCTTCCAACACAATCCACCTCCGCTGGGACCATGATGAACAGGAGCT
SRR946957.94635362.2-    CACCTCCCTGGATCCTTCCAACCCAATCCACCTCCGCTGGGACCATGATGAACAGGAGCT
SRR946958.16186972.2-    CACCTCCCTGGATCCTTCCAACACAATCCACCTCCGCTGGGACCATGATGAACAGGAGCT
SRR946958.1915339.2+     CACCTCCCTGGATCCTTCCAACACAATCCACCTCCGCTGGGACCATGATGAACAGGAGCT
SRR946958.69296965.2+    CACCTCCCTGGATCCTTCCAACACAATCCACCTCCGCTGGGACCATGATGAACAGGAGCT
SRR946958.69981891.1+    CACCTCCCTGGATCCTTCCAACACAATCCACCTCCGCTGGGACCATGATGAACAGGAGCT
SRR946958.83070100.1-    CCCCTCCCTGGATCCTTCCAACACAATCCACCTCCGCTGGGACCATGATGAACAGGAGCT
SRR946960.106834680.1-   CCCCTCCCTGGATCCTTCCAACACAATCCACCTCCGCTGGGACCATGATGAACAGGAGCT
SRR946960.2459542.2+     CACCTCCCTGGTTCCTTCCAACACAATCCACCTCCGCTGGGACCATGATGAACAGGAGCT
SRR946960.29865046.1-    CCCCTCCCTGGATCCTTCCAACACAATCCACCTCCGCTGGGACCATGATGAACAGGAGCT
SRR946960.3126220.2+     CACCTCCCTGGATCCTTCCAACACAATCCACCTCCGCTGGGACCATGATGAACAGGAGCT
SRR946960.37232613.2+    CACCTCCCTGGATCCTTCCAACACAATCCACCTCCGCTGGGACCATGATGAACAGGAGCT
SRR946955.99198518.2+       CTCCCTGGATCCTTCCAACACAATCCACCTCCGCTGGGACCATGATGAACAGGAGCT
SRR946958.37554905.1+       CTCCCTGGATCCTTCCAACACAATCCACCTCCGCTGGGACCATGATGAACAGGAGCT
```

```
SRR946957.15624193.2-        TCCCTGGATCCTTCCAACACAATCCACCTCCGCTGGGACCATGATGAACAGGAGCT
SRR946955.28016599.2-          CCTGGATCCTTCCAACACAATCCACCTCCGCTGGGACCATGATGAACAGGAGCT
SRR946957.47194416.1-              GATCCTTCCAACACAATCCACCTCCGCTGGGACCATGATGAACAGGAGCT
SRR946960.1628156.2-                ATCCTTCCAACACAATCCACCTCCGCTGGGACCATGATGAACAGGAGCT
SRR946957.983545.1+                  TCCTTCCAACACAATCCACCTCCGCTGGGACCATGATGAACAGGAGCT
SRR946960.11892854.2+                TCCTTCCAACACAATCCACCTCCGCTGGGACCATGATGAACAGGAGCT
SRR946957.60371889.2-                     CCAACACAATCCACCTCCGCTGGGACCATGATGAACAGGAGCT
SRR946957.62700518.2-                     CCAACACAATCCACCGCCGCTGGAACCATGATGAACAGGAGCT
SRR946960.97828224.1-                         CACAATCCCCCTCCGCTGGGACCATGATGAACAGGAGCT
SRR946957.31666787.1-                           CAATCCACCTCCGCTGGGACCATGATAAACAGGAGCT
SRR946960.15295326.1-                           CAATCCTCCTCCGCTGGGACCATGATGAACAGGAGCT
SRR946955.73984303.1+                            AATCCACCTCCGCTGGGACCATGATGAACAGGAGCT
SRR946955.1407940.1-                                  ACCTCCGCTGGGACCATGATGAACAGGAGCT
SRR946960.77112329.1+                                 ACCTCCGCTGGGACCATGATGAACAGGAGCT
SRR946955.58346422.2-                                  CCCCCGCTGGGACCATGATGAACAGGAGCT
SRR946960.28393688.2-                                  CCCCCGCTGGGACCATGATGAACAGGAGCT
SRR946955.112874748.1-                                   TCCGCTGGGACCATGATGAACAGGAGCT
SRR946958.97045906.2+                                    TCCGCTGGGACCATGATGAACAGGAGCT
SRR946955.44534172.1-                                     CCGCTGGGACCATGATGAACAGGAGCT
SRR946963.367693.1-                                       CCGCTGGGACCATGATGAACAGGAGCT
SRR946961.32098347.2+                                      CGCTGGGACCATGATGAACAGGAGCT
SRR946955.25344625.2+                                         TGGGACCATGATGAACAGGAGCT
SRR946960.26708667.2-                                         TGGGACAATGATGAAAAGGAGCT
SRR946960.5920316.1-                                          TGGGACCATGATGAACAGGAGCT
SRR946957.38019584.2+                                          GGGACCATGATGAACAGGAGCT
SRR946959.36321848.2+                                           GGACCATGATGAACAGGAGCT
SRR946959.55916250.2-                                           GGACCATGATGAACAGGAGCT
SRR946960.39066531.1-                                           GGACCATGAGGAACAGGAGCT
SRR946955.72129725.2-                                              CCATGATGAACAGGAGCT
SRR946959.109004496.2+                                             CCATGATGAACAGGAGCT
SRR946959.48788881.2+                                              CCATGATGAACAGGAGCT
SRR946959.50720170.1+                                              CCATGATGAACAGGAGCT
SRR946959.76768301.1+                                              CCATGATGAACAGGAGCT
SRR946959.3069236.2-                                                CATGATGAACAGGAGCT
SRR946959.52109877.2-                                               CATGATGAACAGGAGCT
SRR946960.81123918.1-                                               CATGATGAACAGGAGCT
SRR946957.45164104.2-                                                ATGATGAACAGGAGCT
SRR946964.63159785.2+                                                ATGATGAACAGGAGCT
SRR946963.62990066.2-                                                 TGATGAACAGGAGCT
SRR946960.1810227.2+                                                   GATGAACAGGAGCT
SRR946957.110600614.2+                                                  ATGAACAGGAGCT
SRR946957.46436543.1+                                                   ATGAACAGGAGCT
SRR946964.24045114.1+                                                   ATGAACAGGAGCT
SRR946957.9360394.2+                                                       AACAGGAGCT
SRR946959.104735650.2+                                                      ACAGGAGCT
SRR946963.71360651.2+                                                       ACAGGAGCT
SRR946955.112096017.1+                                                         GGAGCT
SRR946963.8610550.2+                                                            GAGCT
SRR946964.47953399.2+                                                           GAGCT
SRR946963.27476630.1-                                                            AGCT
SRR946964.4978041.2-                                                             AGCT
SRR946964.68540145.2-                                                            AGCT
SRR946961.34249143.1-                                                              CT
SRR946959.109831930.1+                                                              T
SRR946960.105947877.2-                                                              T
SRR946960.98815412.1-                                                               T

consensus                CACCTCCCTGGATCCTTCCAACACAATCCACCTCCGCTGGGACCATGATGAACAGGAGCT

                             .    :    .    :    .    :    .    :    .    :    .    :
SRR946960.2459542.2+     GATGACATTTGAGCTGCAGGT
SRR946957.52145586.1+    GATGACATTTGAGCTGCAGGTC
SRR946963.367693.1-      GATGACATTTGAGCTGCAGGTC
SRR946957.59413978.1-    GATGACATTTGAGCTGCAGGTCC
SRR946957.74253506.1+    GATGACATTTGAGCTGCAGGTCC
SRR946961.32098347.2+    GATGACATTTGAGCTGCAGGTCC
SRR946957.71782474.2-    GATGACATTTGAGCTGCAGGTCCA
SRR946955.104117134.1-   GATGACATTTGAGCTGCAGGTCCATA
SRR946955.28016599.2-    GATGACATTTGAGCTGCAGGTCCATA
SRR946955.99198518.2+    GATGACATTTGAGCTGCAGGTCCATA
SRR946957.84913099.2-    GATGACATTTGAGCTGCAGGTCCATA
SRR946957.103208127.1-   GATGACATTTGAGCTGCAGGTCCATAC
SRR946958.69296965.2+    GATGACATTTGAGCTGCAGGTCCATACA
SRR946959.36321848.2+    GATGACATTTGAGCTGCAGGTCCATACA
SRR946959.55916250.2-    GATGACATTTGAGCTGCAGGTCCATACA
SRR946955.63931851.1-    GATGACATTTGAGCTGCAGGTCCATACAAC
SRR946960.106834680.1-   GATGACATTTGAGCTGCAGGTCCATACAAC
SRR946960.37232613.2+    GATGACATTTGAGCTGCAGGTCCATACAAC
SRR946958.16186972.2-    GATGACATTTGAGCTGCAGGTCCATACAACT
SRR946959.109004496.2+   GATGACATTTGAGCTGCAGGTCCATACAACT
SRR946959.48788881.2+    GATGACATTTGAGCTGCAGGTCCATACAACT
SRR946959.50720170.1+    GATGACATTTGAGCTGCAGGTCCATACAACT
SRR946959.76768301.1+    GATGACATTTGAGCTGCAGGTCCATACAACT
SRR946957.117350774.1+   GATGACATTTGAGCTGCAGGTCCATACAACTG
```

```
SRR946957.73860379.2-     GATGACATTTGAGCTGCAGGTCCATACAACTG
SRR946957.94635362.2-     GATGACATTTGAGCTGCAGGTCCATACAACTG
SRR946959.3069236.2-      GATGACATTTGAGCTGCAGGTCCATACAACTG
SRR946959.52109877.2-     GATGACATTTGAGCTGCAGGTCCATACAACTG
SRR946958.1915339.2+      GATGACATTTGAGCTGCAGGCCCCTACAACTGG
SRR946964.63159785.2+     GATGACATTTGAGCTGCAGGTCCATACAACTGG
SRR946960.29865046.1-     GATGACATTTGAGCTGCAGGTCCATACAACTGGC
SRR946963.62990066.2-     GATGACATTTGAGCTGCAGGTCCATACAACTGGC
SRR946957.94172165.2-     GATGACATTTGAGCTGCAGGTCCATACAACTGGCT
SRR946960.3126220.2+      GATGACATTTGAGCTGCAGGTCCATACAACTGGCT
SRR946964.24045114.1+     GATGACATTTGAGCTGCAGGTCCATACAACTGGCTG
SRR946958.69981891.1+     GATGACATTTGAGCTGCAGGTCCATACAACTGGCTGG
SRR946958.83070100.1-     GATGACATTTGAGCTGCAGGTCCATACAACTGGCTGG
SRR946959.104735650.2+    GATGACATTTGAGCTGCAGGTCCATACAACTGGCTGGGTG
SRR946963.71360651.2+     GATGACATTTGAGCTGCAGGTCCATACAACTGGCTGGGGG
SRR946957.983545.1+       GATGACATTTGAGCTGCAGGTCCATACAACTGGCTGGGGGGCA
SRR946958.37554905.1+     GATGACATTTGAGCTGCAGGTCCATACAACTGGCTGGGGGGGA
SRR946963.8610550.2+      GATGACATTTGAGCTGCAGGTCCATACAACTGGCTGGGGGGCA
SRR946957.15624193.2-     GATGACATTTGAGCTGCAGGTCCATACAACTGGCTGGGTGGCAT
SRR946964.47953399.2+     GATGACATTTGAGCTGCAGGTCCATACAACTGGCTGGGGGGCAT
SRR946963.27476630.1-     GATGACATTTGAGCTGCAGGTCCATACAACTGGCTGGGTGGCATT
SRR946964.4978041.2-      GATGACATTTGAGCTGCAGGTCCATACAACTGGCTGGGTGGCATT
SRR946964.68540145.2-     GATGACATTTGAGCTGCAGGTCCATACAACTGGCTGGGTGGCATT
SRR946961.34249143.1-     GATGACATTTGAGCTGCAGGTCCATACAACTGGCTGGGTGGCATTTG
SRR946959.109831930.1+    GATGACATTTGAGCTGCAGGTCCATACAACTGGCTGGGTGGCATTTGG
SRR946957.47194416.1-     GATGACATTTGAGCTGCAGGTCCATACAACTGGCTGGGTGGCATTTGGAT
SRR946960.11892854.2+     GATGACATTTGAGCTGCAGGTCCATACAACTGGCTGGGGGGCCTTTGGATT
SRR946960.1628156.2-      GATGACATTTGAGCTGCAGGTCCATACAACTGGCTGGGTGGCATTTGGATT
SRR946955.58346422.2-     GATGACATTTGAGCTGCAGGTCCATACAACTGGCTGGGTGGCATTTGGATTCAGC
SRR946957.60371889.2-     GATGACATTTGAGCTGCAGGTCCATACAACTGGCTGGGTGGCATTTGGATTCAGCC
SRR946955.25344625.2+     GATGACATTTGAGCTGCAGGTCCATACAACTGGCTGGGTGGCATTTGGATTCAGCCC
SRR946955.73984303.1+     GATGACATTTGAGCTGCAGGTCCATACAACTGGCTGGGGGGCATTTGGATTCAGCCC
SRR946957.62700518.2-     GATGACATTTGAGCTGCAGGTCCATACAACTGGCTGGGTGGCATTTGGATTCAGCCC
SRR946960.97828224.1-     GATGACATTTGAGCTGCAGGTCCATACAACTGGCTGGGTGGCATTTGGATTCAGCCCT
SRR946957.31666787.1-     GATGACATTTGGGCTGCAGGTCCATACAACTGGCTGGGTGGCATTTGGATTCAGCCCTC
SRR946960.28393688.2-     GATGACATTTGAGCTGCAGGTCCATACAACTGGCTGGGTGGCATTTGGATTCAGCCCTC
SRR946960.77112329.1+     GATGACATTTGAGCTGCAGGTCCATACAACTGGCTGGGGGGCAATTGGATTCAGCCCTC
SRR946955.112096017.1+    GATGACATTTGAGCTGCAGGTCCATACAACTGGCTGGGTGGCATTTGGATTCAGCCCTCA
SRR946955.112874748.1-    GATGACATTTGAGCTGCAGGTCCATACAACTGGCTGGGTGGCATTTGGATTCAGCCCTCA
SRR946955.1407940.1-      GATGACATTTGAGCTGCAGGTCCATACAACTGGCTGGGTGGCATTTGGATTCAGCCCTCA
SRR946955.44534172.1-     GATGACATTTGAGCTGCAGGTCCATACAACTGGCTGGGTGGCATTTGGATTCAGCCCTCA
SRR946955.72129725.2-     GATGACATTTGAGCTGCAGGTCCATACAACTGGCTGGGTGGCATTTGGATTCAGCCCTCA
SRR946957.110600614.2+    GATGACATTTGAGCTGCAGGTCCATACAACTGGCTGGGTGGGATTTGGGTTCAGGCCTCA
SRR946957.38019584.2+     GATGACATTTGAGCTGCAGGTCCATACAACTGGCTGGGTGGCATTTGGATTCAGCCCTCA
SRR946957.45164104.2-     GATGACATTTGAGCTGCAGGTCCATACAACTGGCTGGGTGGCATTTGGATTCAGCCCTCA
SRR946957.46436543.1+     GATGACATTTGAGCTGCAGGTCCATACAACTGGCTGGGGGGCATTTGGATTTCGCCCTCA
SRR946957.9360394.2+      GATGACATTTGAGCTGCAGGTCCATACAACTGGCTGGGTGGCATTTGGATTCAGGCCTCA
SRR946958.97045906.2+     GATGACATTTGAGCTGCAGGTCCATACAACTGGCTGGGGGGCATTTGGATTCAGCCCTCA
SRR946960.105947877.2-    GATGACATTTGAGCTGCAGGTCCATACAACTGGCTGGGTGGCATTTGGATTCAGCCCTCA
SRR946960.15295326.1-     GATGACATTTGAGCTGCAGGGCCATACAACTGGCTGGGTGGCATTTGGATTCAGCCCTCA
SRR946960.1810227.2+      GATGACATTTGAGCTGCAGGTCCATACAACTGGCTGGGGGGCATTTGGATTCAGCCCTCA
SRR946960.26708667.2-     GATGACATTTGAGCTGCAGGTCCATACAACTGGCTGGGTGGCATTTGGATTCAGCCCTCA
SRR946960.39066531.1-     GATGACATTTGAGCTGCAGGTCCATACAACTGGCTGGGTGGCATTTGGATTCAGCCCTCA
SRR946960.5920316.1-      GATGACATTTGAGCTGCAGGTCCATACAACTGGCTGGGTGGCATTTGGATTCAGCCCTCA
SRR946960.81123918.1-     GATGACATTTGAGCTGCAGGTCCATACAACTGGCTGGGTGGCATTTGGATTCAGCCCTCA
SRR946960.98815412.1-     GATGACATTTGAGCTGCAGGTCCATACAACTGGCTGGGTGGCATTTGGATTCAGCCCTCA
SRR946958.109621346.1-       GACATTTGAGCTGCAGGTCCATACAACTGGCTGGGGGGCTTTTGGATTCAGCCCTCA
SRR946958.9738316.2-          ACATTTGAGCTGCAGGTCCATACAACTGGCTGGGTGGCATTTGGATTCAGCCCTCA
SRR946960.90974710.1-            TTTGAGCTGCAGATCCATACAACTGGCGGGGTGGCATTTGGATTCAGCCCTCA
SRR946960.79233224.1-             TTGAGCTGCAGGTCCATACAACTGGCTGGGTGGCATTTGGATTCAGCCCTCA
SRR946963.89925099.1+              TGAGCTGCAGGTCCATACAACTGGCTGGGGGGCATTTGGATTCAGCCCT
SRR946964.59460155.1-              TGAGCTGCAGGTCCATACAACTGGCTGGGTGGCATTTGGATTCAGCCCT
SRR946957.69847799.2+              TGAGCTGCAGGTCCATACAACTGGCTGGGGGGCATTTGGATTCAGCCCTCA
SRR946963.20401976.2-               GAGCTGCAGGTCCATACAACTGGCTGGGTGGCATTTGGATTCAGCCCTC
SRR946958.35361805.2-               GAGCTGCAGGTCCATCCAACTGGCTGGGTGGCATTTGGATTCAGCCCTCA
SRR946960.25709152.2-                AGCTGCAGGTCCATACAACTGGCTGGGTGGCATTTGGATTCAGCCCTCA
SRR946961.4459041.1-                  GCTGCAGGTCCATACAACTGGCTGGGTGGCATTTGGATTCAGCCCTCA
SRR946960.91024589.1-                   TGCAGGTCCATACAACTGGCTGGGTGGCATTTGGATTCAGCCCTCA
SRR946957.105630903.1+                    CAGGTCCATACAACTGGCTGGGTGGCATTTGGACTCAGCCCCCA
SRR946957.117309917.2+                     AGGTCCATACAACTGGCTGGGTGGCATTTGGATTCAGCCCTCA
SRR946957.90080588.2-                      AGGTCCATACAACTGGCTGGGTGGCATTTGGATTCAGCCCTCA
SRR946963.50779704.1+                      AGGTCCATACAACTGGCTGGGTGGCATTTGGATTCAGCCCTCA
SRR946955.109130572.1+                       GTCCATACAACTGGCTGGGTGGCATTTGGATTCAGCCCTCA
SRR946958.66642230.1-                         TCCATACAACTGGCTGGGTGGCATTTGGATTCAGCCCTCA
SRR946960.57097995.1-                         TCCATACAACTGGCTGGGTGGCATTTGGATTCAGCCCTCA
SRR946959.43540423.2+                          CCATACAACTGGCTGGGTGGCATTTGGATTCAGCCCTCA
SRR946959.6283813.2+                           CCATACAACTGGCTGGGTGGCATTTGGATTCAGCCCTCA
SRR946957.12408494.1+                            ATACAACTGGCTGGGTGGCATTTGGATTCAGCCCTCA
SRR946960.4354927.2-                             ATACAACTGTCTGGGTGGCATTTGGATTCAGCCCTCA
SRR946955.99198518.1-                             TACAACTGGCTGGGTGGCATTTGGATTCAGCCCTCA
SRR946957.89864013.2-                              ACAACTGGCTGGGTGGCATTTGGATTCAGCCCTCA
SRR946959.45818324.2-                              ACAACTGGCTGGGTGGCATTTGGATTCAGCCCTCA
SRR946957.15683146.2-                               CAACTGGCTGGGTGGCATTTGGATTCAGCCCTCA
SRR946964.65578922.2-                               CAACTGGCTGGGTGGCATTTGGATTCAGCCCTCA
```

```
SRR946958.21115458.2-                               AACTGGCTGGGTGGCATTTGGATTCAGCCCTCA
SRR946955.72441576.2+                                 CTGGCTGGATGGCATTTGGATTCAGCCCTCA
SRR946957.65045800.1-                                  TGGCTGGGTGGCATTTGGATTCAGCCCTCA
SRR946958.100558939.2+                                 TGGCTGGGTGGCATTTGGATTCAGCCCTCA
SRR946957.46227545.1+                                    GCTGGGTGGCATTTGGATTCAGCCCTCA
SRR946959.47282992.1+                                      TGGGTGGCATTTGGATTCAGCCCTCA
SRR946959.13433807.1-                                       GGGTGGCATTTGGATTCAGCCCTCA
SRR946959.21075074.1-                                       GGGTGGCATTTGGATTCAGCCCTCA
SRR946959.76557600.1-                                       GGGTGGCATTTGGATTCAGCCCTCA
SRR946963.20403013.2+                                       GGGTGGCATTTGGATTCAGCCCTCA
SRR946957.12900251.1-                                        GGTGGCATTTGGATTCAGCCCTCA
SRR946960.102188235.1+                                       GGTGGCATTTGGATTCAGCCCTCA
SRR946960.56373046.2+                                        GGTGGCATTTGGATTCAGCCCTCA
SRR946963.106067756.2+                                       GGTGGCATTTGGATTCAGCCCTCA
SRR946963.99457526.2-                                        GGTGGCATTTGGATTCAGCCCTCA
SRR946964.8249685.2+                                         GGTGGCATTTGGATTCAGCCCTCA
SRR946962.72246860.1+                                         GTGGCATTTGGATTCAGCCCTCA
SRR946963.48241.1-                                            GTGGCATTTGGATTCAGCCCTCA
SRR946958.58370706.2+                                          TGGCATTTGGATTCAGCCCTCA
SRR946955.5939405.1+                                            GGCATTTGGATTCAGCCCTCA
SRR946964.43257920.1-                                            GCATTTGGATTCAGCCCTCA
SRR946961.28750677.1+                                             CATTTGGATTCAGCCCTCA
SRR946959.65056747.2+                                              ATTTGGATTCAGCCCTCA
SRR946959.73130109.2+                                              ATTTGGATTCAGCCCTCA
SRR946959.77995769.2+                                              ATTTGGATTCAGCCCTCA
SRR946959.81666768.1-                                              ATTTGGATTCAGCCCTCA
SRR946960.40330220.1-                                              ATTTGGATTCAGCCCTCA
SRR946955.69121824.2+                                                 TGGATTCAGCCCTCA
SRR946958.47746428.1-                                                   GATTCAGCCCTCA
SRR946958.49561749.1-                                                   GATTCAGCCCTCA
SRR946960.2414663.2+                                                    GATTCAGCCCTCA
SRR946960.56936711.2+                                                   GATTCAGCCCTCA
SRR946962.11807903.1+                                                   GATTCAGCCCTCA
SRR946962.23989330.1+                                                   GATTCAGCCCTCA
SRR946962.4256903.1+                                                    GATTCAGCCCTCA
SRR946962.57337807.1+                                                   GATTCAGCCCTCA
SRR946962.70463522.1+                                                   GATTCAGCCCTCA
SRR946962.72210814.1+                                                   GATTCAGCCCTCA
SRR946962.72476209.1+                                                   GATTCAGCCCTCA
SRR946955.25344625.1-                                                     TTCAGCCCTCA
SRR946957.39160918.1-                                                     TTCAGCCCTCA
SRR946959.95583833.1+                                                     TTCAGCCCTCA
SRR946961.19671190.1-                                                        AGCCCTCA
SRR946961.37626057.1-                                                        AGCCCTCA
SRR946961.45962118.1-                                                        AGCCCTCA
SRR946961.85085209.1-                                                        AGCCCTCA
SRR946959.12813377.1-                                                            CTCA
SRR946959.32698026.1-                                                            CTCA
SRR946959.91786797.1-                                                            CTCA
SRR946960.104539216.2-                                                           CTCA
SRR946960.7002808.2+                                                             CTCA
SRR946963.93499107.1-                                                             TCA
SRR946964.26443770.1-                                                             TCA
                         ------------------------------------------------------------
consensus                GATGACATTTGAGCTGCAGGTCCATACAACTGGCTGGGTGGCATTTGGATTCAGCCCTCA

                             .    :    .    :    .    :    .    :    .    :    .    :
SRR946960.15295326.1-    T
SRR946961.4459041.1-     T
SRR946955.72129725.2-    TG
SRR946963.50779704.1+    TGGAGA
SRR946955.1407940.1-     TGGAGAGTT
SRR946959.43540423.2+    TGGAGAGTTG
SRR946959.6283813.2+     TGGAGAGTTG
SRR946955.112874748.1-   TGGAGAGTTGCC
SRR946958.97045906.2+    TGGGGAGTTGCC
SRR946955.44534172.1-    TGGAGAGTTGCCT
SRR946957.9360394.2+     TGGGGAGGTGCCT
SRR946959.45818324.2-    TGGAGAGTTGCCTG
SRR946964.65578922.2-    TGGAGAGTTGCCTGG
SRR946960.26708667.2-    TGGAGAGTTGCCTGGAT
SRR946960.5920316.1-     TGGAGAGTTGCCTGGAT
SRR946957.38019584.2+    TGGAGGGTTGGCTGGATC
SRR946960.39066531.1-    TGGAGAGTTGCCTGGATCT
SRR946957.110600614.2+   TGGAGAGTTGCCCGGATCTGACA
SRR946959.47282992.1+    TGGAGAGTTGCCTGGATCTGACA
SRR946960.81123918.1-    TGGAGAGTTGCCTGGATCTGACA
SRR946957.45164104.2-    TGGAGAGTTGCCTGGATCTGACAT
SRR946959.13433807.1-    TGGAGAGTTGCCTGGATCTGACAT
SRR946959.21075074.1-    TGGAGAGTTGCCTGGATCTGACAT
SRR946959.76557600.1-    TGGAGAGTTGCCTGGATCTGACAT
SRR946963.106067756.2+   TGGAGAGTTGCCTGGATCTGACAT
SRR946963.20403013.2+    TGGAGAGTTGCCTGGATCTGACAT
SRR946964.8249685.2+     TGGAGAGTTGCCTGGATCTGACAT
```

```
SRR946963.99457526.2-    TGGAGAGTTGCCTGGATCTGACATT
SRR946960.1810227.2+     TGGAGAGTTGCCTGGGTCTGGCATTG
SRR946962.72246860.1+    TGGAGAGTTGCCTGGATCTGACATTG
SRR946963.48241.1-       TGGAGAGTTGCCTGGATCTGACATTG
SRR946957.46436543.1+    TGGAGAGTTGCCCGGATCTGACATTGT
SRR946964.43257920.1-    TGGAGAGTTGCCTGGATCTGACATTGTGA
SRR946961.28750677.1+    TGGAGAGTTGCCTGGATCTGACATTGTGAC
SRR946959.65056747.2+    TGGAGAGTTGCCTGGATCTGACATTGTGACA
SRR946959.73130109.2+    TGGAGAGTTGCCTGGATCTGACATTGTGACA
SRR946959.77995769.2+    TGGAGAGTTGCCTGGATCTGACATTGTGACA
SRR946959.81666768.1-    TGGAGAGTTGCCTGGATCTGACATTGTGACA
SRR946955.112096017.1+   TGGAGAGTTGCCTGGATCTGACATTGTGACAGGA
SRR946962.11807903.1+    TGGAGAGTTGCCTGGATCTGACATTGTGACAGGAGG
SRR946962.23989330.1+    TGGAGAGTTGCCTGGATCTGACATTGTGACAGGAGG
SRR946962.4256903.1+     TGGAGAGTTGCCTGGATCTGACATTGTGACAGGAGG
SRR946962.57337807.1+    TGGAGAGTTGCCTGGATCTGACATTGTGACAGGAGG
SRR946962.70463522.1+    TGGAGAGTTGCCTGGATCTGACATTGTGACAGGAGG
SRR946962.72210814.1+    TGGAGAGTTGCCTGGATCTGACATTGTGACAGGAGG
SRR946962.72476209.1+    TGGAGAGTTGCCTGGATCTGACATTGTGACAGGAGG
SRR946957.105630903.1+   TGGAGAGTTGCCTGGATCTGACATTGTGCCAGGGGGGG
SRR946959.95583833.1+    TGGAGAGTTGCCTGGATCTGACATTGTGACAGGAGGTG
SRR946960.105947877.2-   TGGAGAGTTGCCTGGATCTGACATTGTGACAGGAGGTGT
SRR946960.98815412.1-    TGGAGAGTTGCCTGGATCTGACATTGTGACAGGAGGTGT
SRR946961.19671190.1-    TGGAGAGTTGCCTGGATCTGACATTGTGACAGGAGGTGTCT
SRR946961.37626057.1-    TGGAGAGTTGCCTGGATCTGACATTGTGACAGGAGGTGTCT
SRR946961.45962118.1-    TGGAGAGTTGCCTGGATCTGACATTGTGACAGGAGGTGTCT
SRR946961.85085209.1-    TGGAGAGTTGCCTGGATCTGACATTGTGACAGGAGGTGTCT
SRR946958.109621346.1-   TGGAGAATTGCCTGGATCTGACATTGTGACAGGAGGTGTCTCC
SRR946958.9738316.2-     TGGAGAGTTGCCTGGATCTGACATTGTGACAGGAGGTGTCTCCC
SRR946957.69847799.2+    TGGAGAGTTGCCTGGATCTGACATTGTGACAGGGGGGGGCTCCCC
SRR946959.12813377.1-    TGGAGAGTTGCCTGGATCTGACATTGTGACAGGAGGTGTCTCCCC
SRR946959.32698026.1-    TGGAGAGTTGCCTGGATCTGACATTGTGACAGGAGGTGTCTCCCC
SRR946959.91786797.1-    TGGAGAGTTGCCTGGATCTGACATTGTGACAGGAGGTGTCTCCCC
SRR946963.93499107.1-    TGGAGAGTTGCCTGGATCTGACATTGTGACAGGAGGTGTCTCCCCA
SRR946964.26443770.1-    TGGAGAGTTGCCTGGATCTGACATTGTGACAGGAGGTGTCTCCCCA
SRR946960.90974710.1-    TGGAGAGTTGCCTGGATCTGACATTGTGACAGGAGGTGTCTCCCCAA
SRR946960.79233224.1-    TGGAGAGTTGCCTGGATCTGACATTGTGACAGGAGGTGTCTCCCCAAA
SRR946960.25709152.2-    TGGAGAGTTGCCTGGATCTGACATTGTGACAGGAGGTGTCTCTCCAAAT
SRR946955.72441576.2+    TGGAGAGTTGCCTGGATCTGACATTGTGACAGGAGGTGTCTCCCCAAATG
SRR946958.35361805.2-    TGGAGAGTTGCCTGGATCTGACATTGTGACAGGAGGTGTCTCCCCAAATG
SRR946957.117309917.2+   TGGAGAGTTGCCTGGATCTGACATTGTGACAGGAGGTGTCTCCCCAAATGGCAG
SRR946960.91024589.1-    TGGAGAGTTGCCTGGATCTGACATTGTGACAGGAGGTGTCTCCCCAAATGGCAG
SRR946957.90080588.2-    TGGAGAGTTGCCTGGATCTGACATTGTGACAGGAGGTGTCTCCCCAAATGGCAGCAT
SRR946955.109130572.1+   TGGAGAGTTGCCTGGATCTGACATTGTGACAGGAGGTGGCTCCCCAAATGGCAGCATCT
SRR946958.100558939.2+   TGGAGAGTTGCCTGGATCTGACATTGTGACAGGAGGTGTCTCCCCAAATCGCAGCATCT
SRR946955.20023076.2+    TGGAGAGTTGCCTGGATCTGACATTGTGACAGGAGGTGTCTCCCCAAATGGCAGCATCTA
SRR946955.22777120.1+    TGGAGAGTTGCCTGGATCTGACATTGTGACAGGAGGTGTCTCCCCAAATGGCAGCATCTA
SRR946955.25344625.1-    TGGAGAGTTGCCTGGATCTGACATTGTGACAGGAGGTGTCTCCCCAAATGGCAGCATCTA
SRR946955.5939405.1+     TGGAGAGTTGCCTGGATCTGACATTGTGACAGGAGGGGTCTCCCTAAATGGCAGCATCTA
SRR946955.69121824.2+    TGGAGAGTTGCCTGGATCTGACATTGTGACAGGAGGTGTCTCCCCAAATGGCAGCATCTA
SRR946955.99198518.1-    TGGAGAGTTGCCTGGATCTGACATTGTGACAGGAGGTGTCTCCCCAAATGGCAGCATCTA
SRR946957.12408494.1+    TGGAGAGTTGCCTGGATCTGACATTGTGACAGGGGGGGGTCTCCCCAAATGGCAGGATCTA
SRR946957.12900251.1-    TGGAGAGTTGCCTGGATCTGACATTGTGACAGGAGGTGTCTCCCCAAATGGCAGCATCTA
SRR946957.15683146.2-    TGGAGAGTTGCCTGGATCTGACATTGTGACAGGAGGTGTCTCCCCAAATGGCAGCATCTA
SRR946957.39160918.1-    TGGAGAGTTGCCTGGATCTGACATTGTGACAGGAGGTGTCTCCCCAAATGGCAGCATCTA
SRR946957.46227545.1+    TGGAGAGTTGCCTGGATCTGACATTGTGACAGGAGGGGGTCTCCCCAAATGGCAGGATCTA
SRR946957.65045800.1-    TGGAGAGTTGCCTGGATCTGACATTGTGACAGGAGGTGTCTCCCCAAATGGCAGCATCTA
SRR946957.89864013.2-    TGGAGAGTTGCCTGGATCTGACATTGTGACAGGAGGTGTCTCCCCAAATGGCAGCATCTA
SRR946958.21115458.2-    TGGAGAGTTGCCTGGATCTGACATTGTGACAGGAGGTGTCTCCCCAAATGGCAGCATCTA
SRR946958.47746428.1-    TGGAGAGTTGCCTGGATCTGACATTGTGACAGGAGGTGTCTCCCCAAATGGCAGCATCTA
SRR946958.49561749.1-    TGGAGAGTTGCCTGGATCTGACATTGTGGCAGGAGGTGTCTCCCCAAATGGCAGCATCTA
SRR946958.58370706.2+    TGGAGAGTTGCCTGGATCTGACATTGTGACAGGAGGTGTCTCCCCAAATGGCAGCAACTA
SRR946958.66642230.1-    TGGAGAGTTGCCTGGATCTGACATTGTGACAGGAGGTGTCTCCCCAAATGGCAGCATCTA
SRR946960.102188235.1+   TGGAGAGTTGCCTGGATCTGACATTGTGACAGGAGGGGTCTCCCCCAATGGCAGCATCTA
SRR946960.104539216.2-   TGGAGAGTTGCCTGGATCTGACATTGTGACAGGAGGTGTCTCCCCAAATGGCAGCATCTA
SRR946960.2414663.2+     TGGAGAGTTGCCTGGATCTGACATTGTGACAGGAGGTGTCTCCCCAAATGGCAGCATCTA
SRR946960.40330220.1-    TGGAGAGTTGCCTGGATCTGACATTGTGACAGGAGGTGTCTCCCCAAATGGCAGCATCTA
SRR946960.4354927.2-     TGGAGAGTTGCCTGGCTCTGACATTGTGACAGGAGGGGTCCCCCCAAATGGCAGCATCTA
SRR946960.56373046.2+    TGGAGAGTTGCCTGGATCTGACATTGTGACAGGAGGTGTCTCCCCAAATGGCAGCATCTA
SRR946960.56936711.2+    TGGAGAGTTGCCTGGATCTGACATTGTGACAGGAGGTGTCTCCCCAAATGGCAGCATCTA
SRR946960.57097995.1-    TGGAGAGTTGCCTGGATCTGACATTGTGACAGGAGGTGTCTCCCCAAATGGCAGCATCTA
SRR946960.7002808.2+     TGGAGAGTTGCCTGGATCTGACATTGTGACAGGAGGTGTCTCCCCAAATGGCAGCATCTA
SRR946963.65512757.1-     GGAGAGTTGCCTGGATCTGACATTGTGACAGGAGGTGTCTCCCCAAATG
SRR946955.28401826.2+     GGAGAGTTGCCTGGATCTGACATTGTGACAGGAGGTGTCTCCCCAAATGGCAGCATCTA
SRR946955.73984303.2-      GAGAGTTGCCTGGATCTGACATTGTGACAGGAGGTGTCTCCCCAAATGGCAGCATCTA
SRR946955.112096017.2-                        CATTGTGACAGGAGGTGTCTCCCCAAATGGCAGCATCTA
SRR946959.43540423.1-                                                  AATGGCAGCATCTA
SRR946959.6283813.1-                                                   AATGGCAGCATCTA
SRR946955.72441576.1-                                                     GGCAGCATCTA
SRR946955.109130572.2-                                                         CATCTA
                         ____________________________________________________________
consensus                TGGAGAGTTGCCTGGATCTGACATTGTGACAGGAGGTGTCTCCCCAAATGGCAGCATCTA

                             .    :    .    :    .    :    .    :    .    :    .    :
```

```
SRR946955.69121824.2+    CT
SRR946960.4354927.2-     CT
SRR946957.12408494.1+    CTT
SRR946955.99198518.1-    CTTC
SRR946957.15683146.2-    CTTC
SRR946957.89864013.2-    CTTCT
SRR946957.46227545.1+    CTTCTC
SRR946958.21115458.2-    CTTCTCT
SRR946957.65045800.1-    CTTCTCTgga
SRR946960.102188235.1+   CTTTTCTggaagtt
SRR946957.12900251.1-    CTTCTCTggaagttga
SRR946960.56373046.2+    CTTCTCTggaagttga
SRR946958.58370706.2+    CTTCTCTtgaaggtgaaa
SRR946955.5939405.1+     CTTCTCTggcagttgaaaa
SRR946960.2414663.2+     CTTCTCTtgaagttgaaaa
SRR946955.20023076.2+    CTTCTCTggaagttgaaaaga
SRR946955.73984303.2-    CTTCTCTggaagttgaaaaga
SRR946960.40330220.1-    CTTCTCTggaagttgaaaagac
SRR946955.28401826.2+    CTTCTCTggaagttgaaaagactt
SRR946958.47746428.1-    CTTCTCTggaagttgaaaagacttcct
SRR946958.49561749.1-    CTTCTCTggaagttgaaaagacttcct
SRR946960.56936711.2+    CTTCTCTggaagttgaaaagacttcct
SRR946955.25344625.1-    CTTCTCTggaagttgaaaagacttcctct
SRR946957.39160918.1-    CTTCTCTggaagttgaaaagacttcctct
SRR946959.43540423.1-    CTTCTCTggaagttgaaaagacttcctctgcaggc
SRR946959.6283813.1-     CTTCTCTggaagttgaaaagacttcctctgcaggc
SRR946960.104539216.2-   CTTCTCTggaagttgaaaagacttcctctgcaggct
SRR946960.7002808.2+     CTTCTCTggaagttgaaaagaattcctccgcaggct
SRR946955.112096017.2-   CTTCTCTggaagttgaaaagacttcctctgcaggctcctt
SRR946955.22777120.1+    CTTCTCTggaagttgaaaagacttcctctgcaggctcctt
SRR946955.109130572.2-   CTTCTCTggaagttgaaaagacttcctctgcaggctcctttgtcaggggtcatgctccaa
SRR946955.72441576.1-    CTTCTCTggaagttgaaaagacttcctctgcaggctcctttgtcaggggtcatgctccaa
SRR946955.28401826.1-                ttgaaaagacttcctctgcaggctcctttgtcagtggtcatgctccaa
SRR946955.5939405.2-                        gacttcctctgcaggctcctttgtcaggggtcatgctccaa
SRR946955.69121824.1-                       gacttcctctgcaggctcctttgtcaggggtcatgctccaa
SRR946955.20023076.1-                        acttcctctgcaggctcctttgtcaggggtcatgctccaa
SRR946955.22777120.2-                            cctctgcaggctcctttgtcaggggtcatgctccaa
                         ____________________________________________________________
consensus                CTTCTCTggaagttgaaaagacttcctctgcaggctcctttgtcaggggtcatgctccaa
```

B. American crow (No. 3), exon 1, WGS data

- CAP3 alignment of WGS data spanning exon 1 (uppercase letters)

```
                             .    :    .    :    .    :    .    :    .    :    .    :
SRR944633.99564964.1+    gtagctgcccgaggtgtcagcaagactcagaaactgcagtgA
SRR944636.20310598.1-    gtagctgcccgaggtgtcagcaagactcagaaactgcagtgA
SRR944632.64464026.2+    gtagctgcccgaggtgtcagcaagactcagaaactgcagtgAT
SRR944639.30631809.1+    gtagctgcccgaggtgtcagcaagactcagaaactgcagtgAT
SRR944636.15853340.1+    gtagctgcccgaggtgtcagcaagactcagaaactgcagtgATGA
SRR944633.73901834.2-    gtagctgcccgaggtgtcagcaagactcagaaactgcagtgATGAT
SRR944634.81565879.1+    gtagctgcccgaggtgtcagcaagactcagaaactgcagtgATGATGG
SRR944638.33872132.1+    gtagctgcccgaggtgtcagcaagactcagaaactgcagtgATGATGG
SRR944638.46339275.1+    gtagctgcccgaggtgtcagcaagactcagaaactgcagtgATGATGG
SRR944637.65354816.1-    gtagctgcccgaggtgtcagcaagactcagaaactgcagtgATGATGGTGATGCT
SRR944634.74122007.1+    gtagctgcccgaggtgtcagcaagactcagaaactgcagtgATGATGGTGATGCTC
SRR944633.25313478.2+    gtagctgcccgaggtgtcagcaagactcagaaactgcagtgATGATGGTGATGCTCTTCT
SRR944633.26564911.1+    gtagctgcccgaggtgtcagcaagactcagaaactgcagtgATGATGGTGATGCTCTTCT
SRR944633.30133295.2-    gtagctgcccgaggtgtcagcaagactcagaaactgcagtgATGATGGTGATGCTCTTCT
SRR944633.34300351.2-    gtagctgcccgaggtgtcagcaagactcagaaactgcagtgATGATGGTGATGCTCTTCT
SRR944633.4272160.2-     gtagctgcccgaggtgtcagcaagactcagaaactgcagtgATGATGGTGATGCTCTTCT
SRR944633.57914021.2-    gtagctgcccgaggtgtcagcaagactcagaaactgcagtgATGATGGTGATGCTCTTCT
SRR944633.60527143.1-    gtagctgcccgaggtgtcagcaagactcagaaactgcagtgATGATGGTGATGCTCTTCT
SRR944633.67034951.2-    gtagctgcccgaggtgtcagcaagactcagaaactgcagtgATGATGGTGATGCTCTTCT
SRR944633.7837185.2-     gtagctgcccgaggtgtcagcaagactcagaaactgcagtgATGATGGTGATGCTCTTCT
SRR944634.24301483.2+    gtagctgcccgaggtgtcagcaagactcagaaactgcagtgATGATGGTGATGCTCTTCT
SRR944634.29623047.2+    gtagctgcccgaggtgtcagcaagactcagaaactgcagtgATGATGGTGATGCTCTTCT
SRR944634.34802299.1+    gtagctgcccgaggtgtcagcaagactcagaaactgcagtgATGATGGTGATGCTCTTCT
SRR944634.41644313.1-    gtagctgcccgaggtgtcagcaagactcagaaactgcagtgATGATGGTGATGCTCTTCT
SRR944634.46910015.1+    gtagctgcccgaggtgtcagcaagactcagaaactgcagtgATGATGGTGATGCTCTTCT
SRR944634.66207389.2+    gtagctgcccgaggtgtcagcaagactcagaaactgcagtgATGATGGTGATGCTCTTCT
SRR944634.77968149.2+    gtagctgcccgaggtgtcagcaagactcagaaactgcagtgATGATGGTGATGCTCTTCT
SRR944634.84693929.1+    gtagctgcccgaggtgtcagcaagactcagaaactgcagtgATGATGGTGATGCTCTTCT
SRR944635.2300460.1-     gtagctgcccgaggtgtcagcaagactcagaaactgcagtgATGATGGTGATGCTCTTCT
SRR944635.40788816.2-    gtagctgcccgaggtgtcagcaagactcagaaactgcagtgATGATGGTGATGCTCTTCT
SRR944635.71478536.1-    gtagctgcccgaggtgtcagcaagactcagaaactgcagtgATGATGGTGATGCTCTTCT
SRR944635.81760264.2-    gtagctgcccgaggtgtcagcaagactcagaaactgcagtgATGATGGTGATGCTCTTCT
SRR944635.81781524.1-    gtagctgcccgaggtgtcagcaagactcagaaactgcagtgATGATGGTGATGCTCTTCT
SRR944635.93226745.1-    gtagctgcccgaggtgtcagcaagactcagaaactgcagtgATGATGGTGATGCTCTTCT
SRR944637.21642352.1-    gtagctgcccgaggtgtcagcaagactcagaaactgcagtgATGATGGTGATGCTCTTCT
SRR944637.26459078.2-    gtagctgcccgaggtgtcagcaagactcagaaactgcagtgATGATGGTGATGCTCTTCT
SRR944637.34494868.2-    gtagctgcccgaggtgtcagcaagactcagaaactgcagtgATGATGGTGATGCTCTTCT
SRR944637.37249548.2-    gtagctgcccgaggtgtcagcaagactcagaaactgcagtgATGATGGTGATGCTCTTCT
SRR944637.47526072.2-    gtagctgcccgaggtgtcagcaagcctcagaaactgcagtgATGATGGTGATGCTCTTCT
SRR944637.64118957.1-    gtagctgcccgaggtgtcagcaagactcagaaactgcagtgATGATGGTGATGCTCTTCT
SRR944637.69311163.1+    gtagctgcccgaggtgtcagcaagactcagaaactgcagtgATGATGGTGATGCTCTTCT
SRR944632.99118942.1+     tagctgcccgaggtgtcagcaagactcagaaactgcagtgATGATGGTG
SRR944634.13125669.1+     tagctgcccgaggtgtcagcaagactcagaaactgcagtgATGATGGTGATGCTCTTCT
SRR944637.1220408.2+      tagctgcccgaggtgtcagcaagactcagaaactgcagtgATGATGGTGATGCTCTTCT
SRR944637.25240257.2+     tagctgcccgaggtgtcagcaagactcagaaactgcagtgATGATGGTGATGCTCTTCT
SRR944638.77644039.2-      agctgcccgaggtgtcagcaagactcagaaactgcagtgATGATGGTGA
SRR944633.31142104.1-         tgcccgaggtgtcagcaagactcagaaactgcagtgATGATGGTGATGCTCTTCT
SRR944633.99564964.2-         tgcccgaggtgtcagcaagactcagaaactgcagtgATGATGGTGATGCTCTTCT
SRR944634.12799848.1+         tgcccgaggtgtcagcaagactcagaaactgcagtgATGATGGTGATGCTCTTCT
SRR944634.51759179.2-         tgcccgaggtgtcagcaagactcagaaactgcagtgATGATGGTGATGCTCTTCT
SRR944634.80853444.1+         tgcccgaggtgtcagcaagactcagaaactgcagtgATGATGGTGATGCTCTTCT
SRR944636.59987069.2+          gcccgaggtgtcagcaagactcagaaactgcagtgATGATGGTGATGCT
SRR944636.91570116.2+          gcccgaggtgtcagcaagactcagaaactgcagtgATGATGGTGATGCT
SRR944632.115399270.2-          cccgaggtgtcagcaagactcagaaactgcagtgATGATGGTGATGCTC
SRR944634.77387431.1+           cccgaggtgtcagcaagactcagaaactgcagtgATGATGGTGATGCTCTTCT
SRR944630.38900498.2-            ccgaggtgtcagcaagactcagaaactgcagtgATGATGGTGATGCTCT
SRR944636.19312528.1+            ccgaggtgtcagcaagactcagaaactgcagtgATGATGGTGATGCTCT
SRR944636.64995817.1+            ccgaggtgtcagcaagactcagaaactgcagtgATGATGGTGATGCTCT
SRR944636.90108431.1+            ccgaggtgtcagcaagactcagaaactgcagtgATGATGGTGATGCTCT
SRR944632.41752938.1+              gaggtgtcagcaagactcagaaactgcagtgATGATGGTGATGCTCTTC
SRR944638.56650498.2+              gaggtgtcagcaagactcagaaactgcagtgATGATGGTGATGCTCTTC
SRR944638.85031089.2+              gaggtgtcagcaagactcagaaactgcagtgATGATGGTGATGCTCTTC
SRR944638.86199803.1-              gaggtgtcagcaagactcagaaactgcagtgATGATGGTGATGCTCTTC
SRR944633.18236212.1+              gaggtgtcagcaagactcagaaactgcagtgATGATGGTGATGCTCTTCT
SRR944634.20628373.2+              gaggtgtcagcaagactcagaaactgcagtgATGATGGTGATGCTCTTCT
SRR944634.70964299.1+               aggtgtcagcaagactcagaaactgcagtgATGATGGTGATGCTCTTCT
SRR944635.7872034.1-                  gtgtcagcaagactcagaaactgcagtgATGATGGTGATGCTCTTCT
SRR944633.98128369.1+                  tgtcagcaagactcagaaactgcagtgATGATGGTGATGCTCTTCT
SRR944632.81135265.1+                   gtcagcaagactcagaaactgcagtgATGATGGTGATGCTCTTCT
SRR944635.49513625.1-                   gtcagcaagactcagaaactgcagtgATGATGGTGATGCTCTTCT
SRR944633.99195671.2+                    tcagcaagactcagaaactgcagtgATGATGGTGATGCTCTTCT
SRR944635.61730673.1-                    tcagcaagactcagaaactgcagtgATGATGGTGATGCTCTTCT
SRR944634.74122007.2-                      agcaagactcagaaactgcagtgATGATGGTGATGCTCTTCT
SRR944634.66207389.1-                       gcaagactcagaaactgcagtgATGATGGTGATGCTCTTCT
SRR944634.81565879.2-                       gcaagactcagaaactgcagtgATGATGGTGATGCTCTTCT
```

```
SRR944630.84608148.1-                        caagactcagaaactgcagtgATGATGGTGATGCTCTTCT
SRR944638.25047412.1-                        caagactcagaaactgcagtgATGATGGTGATGCTCTTCT
SRR944639.64659294.1+                        caagactcagaaactgcagtgATGATGGTGATGCTCTTCT
SRR944635.40200896.1-                           gactcagaaactgcagtgATGATGGTGATGCTCTTCT
SRR944636.16964149.1+                             ctcagaaactgcagtgATGATGGTGATGCTCTTCT
SRR944636.4351696.1+                              ctcagaaactgcagtgATGATGGTGATGCTCTTCT
SRR944636.64049083.1+                             ctcagaaactgcagtgATGATGGTGATGCTCTTCT
SRR944632.111782190.2+                             tcagaaactgcagtgATGATGGTGATGCTCTTCT
SRR944632.74211923.2+                              tcagaaactgcagtgATGATGGTGATGCTCTTCT
SRR944632.81005069.1+                              tcagaaactgcagtgATGATGGTGATGCTCTTCT
SRR944632.99936685.2+                              tcagaaactgcagtgATGATGGTGATGCTCTTCT
SRR944637.11188729.2+                              tcagaaactgcagtgATGATGGTGATGCTCTTCT
SRR944634.54009543.2+                               cagaaactgcagtgATGATGGTGATGCTCTTCT
SRR944636.96397436.2-                               cagaaactgcagtgATGATGGTGATGCTCTTCT
SRR944630.46900203.2-                                 gaaactgcagtgATGATGGTGATGCTCTTCT
SRR944639.12060957.1+                                 gaaactgcagtgATGATGGTGATGCTCTTCT
SRR944632.47334264.2+                                  aaactgcagtgATGATGGTGATGCTCTTCT
SRR944637.16414866.2+                                  aaactgcagtgATGATGGTGATGCTCTTCT
SRR944637.4122469.2+                                    aactgcagtgATGATGGTGATGCTCTTCT
SRR944639.65025889.2+                                     ctgcagtgATGATGGTGATGCTCTTCT
SRR944635.52001870.1-                                       gcagtgATGAGGGTGATGCTCTTCT
SRR944633.40489199.2+                                        cagtgATGATGGTGATGCTCTTCT
SRR944636.53558935.1+                                          gtgATGATGGTGATGCTCTTCT
SRR944632.2401542.1+                                            tgATGATGGTGATGCTCTTCT
SRR944638.2779174.1+                                             gATGATGGTGATGCTCTTCT
SRR944638.63373416.1+                                            gATGATGGTGATGCTCTTCT
SRR944638.7686562.1+                                             gATGATGGTGATGCTCTTCT
SRR944631.21892125.2-                                              TGATGGTGATGCTCTTCT
SRR944631.31991311.2-                                              TGATGGTGATGCTCTTCT
SRR944631.38205968.2-                                              TGATGGTGATGCTCTTCT
SRR944631.40005968.2-                                              TGATGGTGATGCTCTTCT
SRR944631.49279245.2-                                              TGATGGTGATGCTCTTCT
SRR944634.94919320.2+                                               GATGGTGATGCTCTTCT
SRR944632.72360615.1+                                                ATGGTGATGCTCTTCT
SRR944634.61033746.1+                                                ATGGTGATGCTCTTCT
SRR944639.90324502.1+                                                ATGGTGATGCTCTTCT
SRR944633.82191784.1+                                                 TGGTGATGCTCTTCT
SRR944634.46910015.2-                                                 TGGTGATGCTCTTCT
SRR944632.28355724.1+                                                  GGTGATGCTCTTCT
SRR944638.51612977.1+                                                    TGATGCTCTTCT
SRR944638.67869952.1+                                                    TGATGCTCTTCT
SRR944634.63040457.2+                                                     GATGCTCTTCT
SRR944634.77968149.1-                                                     GATGCTCTTCT
SRR944638.16884164.2+                                                      ATGCTCTTCT
SRR944633.25313478.1-                                                       TGCTCTTCT
SRR944634.34545057.2+                                                        GCTCTTCT
SRR944638.13663328.2+                                                        GCTCTTCT
SRR944632.13727313.2+                                                          TCTTCT
SRR944633.90674241.2+                                                          TCTTCT
SRR944634.29623047.1-                                                           CTTCT
SRR944634.3508483.2+                                                            CTTCT
SRR944635.49727187.1+                                                               T
                         ____________________________________________________________
consensus                gtagctgcccgaggtgtcagcaagactcagaaactgcagtgATGATGGTGATGCTCTTCT

                             .    :    .    :    .    :    .    :    .    :    .    :
SRR944633.7837185.2-     CA
SRR944632.81135265.1+    CAGG
SRR944633.60527143.1-    CAGGGAT
SRR944634.84693929.1+    CAGGGATC
SRR944630.84608148.1-    CAGGGATCA
SRR944638.25047412.1-    CAGGGATCA
SRR944639.64659294.1+    CAGGGATCA
SRR944633.26564911.1+    CAGGGATCAA
SRR944633.67034951.2-    CAGGGATCAAGA
SRR944635.40788816.2-    CAGGGATCAAGAG
SRR944635.71478536.1-    CAGGGATCAAGAG
SRR944635.93226745.1-    CAGGGATCAAGAG
SRR944636.16964149.1+    CAGGGATCAAGAGA
SRR944636.4351696.1+     CAGGGATCAAGAGA
SRR944636.64049083.1+    CAGGGATCAAGAGA
SRR944632.111782190.2+   CAGGGATCAAGAGAA
SRR944632.74211923.2+    CAGGGATCAAGAGAA
SRR944632.81005069.1+    CAGGGATCAAGAGAA
SRR944632.99936685.2+    CAGGGATCAAGAGAA
SRR944634.34802299.1+    CAGGGATCAAGAGAAT
SRR944636.96397436.2-    CAGGGATCAAGAGAAT
SRR944637.21642352.1-    CAGGGATCAAGAGAAT
SRR944634.41644313.1-    CAGGGATCAAGAGAATG
SRR944630.46900203.2-    CAGGGATCAAGAGAATGC
SRR944637.34494868.2-    CAGGGATCAAGAGAATGC
SRR944639.12060957.1+    CAGGGATCAAGAGAATGC
SRR944632.47334264.2+    CAGGGATCAAGAGAATGCT
SRR944633.4272160.2-     CAGGGATCAAGAGAATGCT
```

SRR944634.66207389.2+	CAGGGATCAAGAGAATGCT
SRR944634.77968149.2+	CAGGGATCAAGAGAATGCT
SRR944634.46910015.1+	CAGGGATCAAGAGAATGCTC
SRR944635.81781524.1-	CAGGGATCAAGAGAATGCTC
SRR944637.26459078.2-	CAGGGATCAAGAGAATGCTCT
SRR944639.65025889.2+	CAGGGATCAAGAGAATGCTCTT
SRR944637.37249548.2-	CAGGGATCAAGAGAATGCTCTTCC
SRR944634.29623047.2+	CAGGGATCAAGAGAATGCTCTTCCT
SRR944633.34300351.2-	CAGGGATCAAGAGAATGCTCTTCCTCT
SRR944636.53558935.1+	CAGGGATCAAGAGAATGCTCTTCCTCT
SRR944637.64118957.1-	CAGGGATCAAGAGAATGCTCTTCCTCT
SRR944632.2401542.1+	CAGGGATCAAGAGAATGCTCTTCCTCTT
SRR944633.30133295.2-	CAGGGATCAAGAGAATGCTCTTCCTCTT
SRR944633.57914021.2-	CAGGGATCAAGAGAATGCTCTTCCTCTT
SRR944635.2300460.1-	CAGGGATCAAGAGAATGCTCTTCCTCTT
SRR944635.81760264.2-	CAGGGATCAAGAGAATGCTCTTCCTCTT
SRR944638.2779174.1+	CAGGGATCAAGAGAATGCTCTTCCTCTTG
SRR944638.63373416.1+	CAGGGATCAAGAGAATGCTCTTCCTCTTG
SRR944638.7686562.1+	CAGGGATCAAGAGAATGCTCTTCCTCTTG
SRR944639.90324502.1+	CAGGGATCAAGAGAATGCTCTTCCTCTTGT
SRR944631.21892125.2-	CAGGGATCAAGAGAATGCTCTTCCTCTTGTT
SRR944631.31991311.2-	CAGGGATCAAGAGAATGCTCTTCCTCTTGTT
SRR944631.38205968.2-	CAGGGATCAAGAGAATGCTCTTCCTCTTGTT
SRR944631.40005968.2-	CAGGGATCAAGAGAATGCTCTTCCTCTTGTT
SRR944631.49279245.2-	CAGGGATCAAGAGAATGCTCTTCCTCTTGTT
SRR944632.72360615.1+	CAGGGATCAAGAGAATGCTCTTCCTCTTGTTCC
SRR944632.28355724.1+	CAGGGATCAAGAGAATGCTCTTCCTCTTGTTCCTC
SRR944633.25313478.2+	CAGGGATCAAGAGAATGCTCTTCCTCTTGTTCCTC
SRR944634.24301483.2+	CAGGGATCAAGAGAATGCTCTTCCTCTTGTTCCTCCC
SRR944637.25240257.2+	CAGGGATCAAGAGAATGCTCTTCCTCTTGTTACTCCC
SRR944638.51612977.1+	CAGGGATCAAGAGAATGCTCTTCCTCTTGTTCCTCCC
SRR944638.67869952.1+	CAGGGATCAAGAGAATGCTCTTCCTCTTGTTCCTCCC
SRR944637.47526072.2-	CAGTGATCAAGAGAATGCTCTTCCTCTTGTTCCTCCCA
SRR944637.69311163.1+	CAGGGATCAAGAGAATGCTCTTCCTCTTGTTCCTCCCA
SRR944638.16884164.2+	CAGGGATCAAGAGAATGCTCTTCCTCTTGTTCCTCCCAT
SRR944634.13125669.1+	CAGGGATCAAGAGAATGCTCTTCCTCTTGTTCCTCCCATGC
SRR944637.1220408.2+	CAGGGATCAAGAGAATGCTCTTCCTCTTGTTCCTCCCATGC
SRR944638.13663328.2+	CAGGGATCAAGAGAATGCTCTTCCTCTTGTTCCTCCCATGC
SRR944632.13727313.2+	CAGGGATCAAGAGAATGCTCTTCCTCTTGTTCCTCCCATGCTT
SRR944633.31142104.1-	CAGGGATCAAGAGAATGCTCTTCCTCTTGTTCCTCCCATGCTTTT
SRR944633.99564964.2-	CAGGGATCAAGAGAATGCTCTTCCTCTTGTTCCTCCCATGCTTTT
SRR944634.12799848.1+	CAGGGATCAAGAGAATGCTCTTCCTCTTGTTCCTCCCATGCTTTT
SRR944634.51759179.2-	CAGGGATCAAGAGAATGCTCTTCCTCTTGTTCCTCCCATGCTTTT
SRR944634.80853444.1+	CAGGGATCAAGAGAATGCTCTTCCTCTTGTTCCTCCCATGCTTTT
SRR944634.77387431.1+	CAGGGATCAAGAGAATGCTCTTCCTCTTGTTCCTCCCATGCTTTTGT
SRR944631.31144036.2+	CAGGGATCAAGAGAATGCTCTTCCTCTTGTTCCTCCCATGCTTTTGTTC
SRR944631.6910189.1+	CAGGGATCAAGAGAATGCTCTTCCTCTTGTTCCTCCCATGCTTTTGTTC
SRR944636.11342958.1+	CAGGGATCAAGAGAATGCTCTTCCTCTTGTTCCTCCCATGCTTTTGTTC
SRR944636.75478208.1+	CAGGGATCAAGAGAATGCTCTTCCTCTTGTTCCTCCCATGCTTTTGTTC
SRR944636.84196590.2+	CAGGGATCAAGAGAATGCTCTTCCTCTTGTTCCTCCCATGCTTTTGTTC
SRR944636.95597625.1+	CAGGGATCAAGAGAATGCTCTTCCTCTTGTTCCTCCCATGCTTTTGTTC
SRR944633.18236212.1+	CAGGGATCAAGAGAATGCTCTTCCTCTTGTTCCTCCCATGCTTTTGTTCT
SRR944634.20628373.2+	CAGGGATCAAGAGAATGCTCTTCCTCTTGTTCCTCCCATGCTTTTGTTCT
SRR944634.70964299.1+	CAGGGATCAAGAGAATGCTCTTCCTCTTGTTCCTCCCATGCTTTTGTTCTG
SRR944635.7872034.1-	CAGGGATCAAGAGAATGCTCTTCCTCTTGTTCCTCCCATGCTTTTGTTCTGGC
SRR944633.98128369.1+	CAGGGATCAAGAGAATGCTCTTCCTCTTGTTCCTCCCCTGCTTTTGTTTTGGCC
SRR944635.49513625.1-	CAGGGATCAAGAGAATGCTCTTCCTCTTGTTCCTCCCATGCTTTTGTTCTGGCCA
SRR944633.99195671.2+	CAGGGATCAAGAGAATGCTCTTCCTCTTGTTCCTCCCATGCTTTTGTTCTGGCCAG
SRR944635.61730673.1-	CAGGGATCAAGAGAATGCTCTTCCTCTTGTTCCTCCCATGCTTTTGTTCTGGCCAG
SRR944634.74122007.2-	CAGGGATCAAGAGAATGCTCTTCCTCTTGTTCCTCCCATGCTTTTGTTCTGGCCAGCT
SRR944634.66207389.1-	CAGGGATCAAGAGAATGCTCTTCCTCTTGTTCCTCCCATGCTTTTGTTCTGGCCAGCTT
SRR944634.81565879.2-	CAGGGATCAAGAGAATGCCCTTCCTCTTGTTCCTCCCATGCTTTTGTTCTGGCCAGCTT
SRR944633.25313478.1-	CAGGGATCAAGAGAATCCTCTTCCTCTTGTTCCTCCCATGCTTTTGTTCTGGCCAGCTTG
SRR944633.40489199.2+	CAGGGATCAAGAGAATGCTCTTCCTCTTGTTCCTCCCATGCTTTTGTTCTGGCCAGCTTG
SRR944633.82191784.1+	CAGGGATCAAGAGAATGCTCTTCCTCTTGTTCCTCCCATGCTTTTGTTCTGGCCAGCTTG
SRR944633.90674241.2+	CAGGGATCAAGAGAATGCTCTTCCTCTTGTTCCTCCCATGCTTTTGTTCTGGCCAGCTTG
SRR944634.29623047.1-	CGGGGATCAAGAGAATGCTCTTCCTCTTGTTCCTCCCATGCTTTTGTTCGGGCCAGCTTG
SRR944634.34545057.2+	CAGGGATCAAGAGAATGCTCTTCCTCTTGTTCCTCCCATGCTTTTGTTCTGGCCAGCTTG
SRR944634.3508483.2+	CAGGGATCAAGAGAATGCTCTTCCTCTTGTTCCTCCCATGCTTTTGTTCTGGCCAGCTTG
SRR944634.46910015.2-	CAGGGATCAAGAGAATGCTCTTCCTCTTGTTCCCCCCATGCTTTTGTTCTGGCCAGCTTG
SRR944634.54009543.2+	CAGGGATCAAGAGAATGCTCTTCCTCTTGTTCCTCCCATGCTTTTGTTCTAGCCAGCTTG
SRR944634.61033746.1+	CAGGGATCAAGAGAATGCTCTTCCTCTTGTTCCTCCCATGCTTTTGTTCTGGCCAGCTTG
SRR944634.63040457.2+	CAGGGATCAAGAGAATGCTCTTCCTCTTGTTCCTCCCATGCTTTTGTTCTGGCCAGCTTG
SRR944634.77968149.1-	CAGGGATCAAGAGAATGCTCTTCCTCTTGTTCCTCCCATGCTTTTGTTCTGGCCAGCTTG
SRR944634.94919320.2+	CAGGGATCAAGAGAATGCTCTTCCTCTTGTTCCTCCCATGCTTTTGTTCTGGCCAGCTTG
SRR944635.40200896.1-	CAGGGATCAAGAGAATGCTCTTCCTCTTGTTCCTCCCATGCTTTTGTTCTGGCCAGCTTG
SRR944635.49727187.1+	CAGGGATCAAGAGAATGCTCTTCCTCTTGTTCCTCCCATGCTTTTGTTCTGGCCAGCTTG
SRR944635.52001870.1-	CAGGGATCAAGAGAATGCTCTTCCTCTTGTTCCTCCCATGCTTTTGTTCTGGCCAGCTTG
SRR944637.11188729.2+	CAGGGATCAAGAGAATGCTCTTCCTCTTGTTCCTCCCATGCTTTTGTTCTGGCCAGCTTG
SRR944637.16414866.2+	CAGGGATCAAGAGAATGCTCTTCCTCTTGTTCCTCCCATGCTTTTGTTCTGGCCAGCTTG
SRR944637.4122469.2+	CAGGGATCAAGAGAATGCTCTTCCTCTTGTTCCTCCCATGCTTTTGTTCTGGCCAGCCTG
SRR944636.31304490.2-	GGGATCAAGAGAATGCTCTTCCTCTTGTTCCTCCCATGCTTTTGTTCTG
SRR944638.3016663.1-	GGGATCAAGAGAATGCTCTTCCTCTTGTTCCTCCCATGCTTTTGTTCTG
SRR944638.52827038.2-	GGGATCAAGAGAATGCTCTTCCTCTTGTTCCTCCCATGCTTTTGTTCTG

```
SRR944638.60189056.2-      GGGATCAAGAGAATGCTCTTCCTCTTGTTCCTCCCATGCTTTTGTTCTG
SRR944634.69071785.1+      GGGATCAAGAGAATGCTCTTCCTCTTGTTCCTCCCATGCTTTTGTTCTGGCCAGCTTG
SRR944635.30275578.1-      GGGATCAAGAGAATGCTCTTCCTCTTGTTCCTCCCATGCTTTTGTTCTGGCCAGCTTG
SRR944634.24301483.1-       GGATCAAGAGAATGCTCTTCCTCTTCTTNCTCCCATGCTTTTGTTCTGGCCAGCTTG
SRR944637.20928485.2+       GGATCAAGAGAATGCTCTTCCTCTTGTTCCTCCCATGCTTTTGTTCTGGCCAGCTTG
SRR944639.78391931.1-          TCAAGAGAATGCTCTTCCTCTTGTTCCTCCCATGCTTTTGTTCTGGCCA
SRR944630.42215617.1+           CAAGAGAATGCTCTTCCTCTTGTTCCTCCCATGCTTTTGTTCTGGCCAG
SRR944630.55209506.2+           CAAGAGAATGCTCTTCCTCTTGTTCCTCCCATGCTTTTGTTCTGGCCAG
SRR944636.34194314.1-           CAAGAGAATGCTCTTCCTCTTGTTCCTCCCATGCTTTTGTTCTGGCCAG
SRR944638.40971872.2+           CAAGAGAATGCTCTTCCTCTTGTTCCTCCCATGCTTTTGTTCTGGCCAG
SRR944638.50549606.2+           CAAGAGAATGCTCTTCCTCTTGTTCCTCCCATGCTTTTGTTCTGGCCAG
SRR944639.57379420.1+           CAAGAGAATGCTCTTCCTCTTGTTCCTCCCATGCTTTTGTTCTGGCCAG
SRR944634.71474468.2+           CAAGAGAATGCTCTTCCTCTTGTTCCTCCCATGCTTTTGTTCTGGCCAGCTTG
SRR944639.85904287.1-            AAGAGAATGCTCTTCCTCTTGTTCCTCCCATGCTTTTGTTCTGGCCAGC
SRR944632.105719955.1-            AGAGAATGCTCTTCCTCTTGTTCCTCCCATGCTTTTGTTCTGGCCAGCT
SRR944633.98416869.1-               AGAATGCTCTTCCTCTTGTTCCTCCCATGCTTTTGTTCTGGCCAGCTTG
SRR944633.98416869.2+               AGAATGCTCTTCCTCTTGTTCCTCCCATGCTTTTGTTCTGGCCAGCTTG
SRR944633.22219140.2+                GAATGCTCTTCCTCTTGTTCCTCCCATGCTTTTGTTCTGGCCAGCTTG
SRR944636.85657916.2+                 AATGCTCTTCCTCTTGTTCCTCCCATGCTTTTGTTCTGGCCAGCTTG
SRR944633.54109791.1+                  ATGCTCTTCCTCTTGTTCCTCCCATGCTTTTGTTCTGGCCAGCTTG
SRR944634.20628373.1-                  ATGCTCTTCCTCTTGTTCCTCCCAGGCTTTTGTTCTGGCCAGCTTG
SRR944633.99195671.1-                   TGCTCTTCCTCTTGTTCCTCCCATGCTTTTGTTCTGGCCAGCTTG
SRR944636.85623294.1+                   TGCTCTTCCTCTTGTTCCTCCCATGCTTTTGTTCTGGCCAGCTTG
SRR944637.36954774.1+                   TGCTCTTCCTCTTGTTCCTCCCATGCTTTTGTTCTGGCCAGCTTG
SRR944637.48405569.1+                   TGCTCTTCCTCTTGTTCCTCCCATGCTTTTGTTCTGGCCAGCTTG
SRR944637.67596548.1+                   TGCTCTTCCTCTTGTTCCTCCCATGCTTTTGTTCTGGCCAGCTTG
SRR944639.70391911.2+                    GCTCTTCCTCTTGTTCCTCCCATGCTTTTGTTCTGGCCAGCTTG
SRR944631.8853804.1+                        CTTCCTCTTGTTCCTCCCATGCTTTTGTTCTGGCCAGCTTG
SRR944637.20349171.2+                       CTTCCTCTTGTTCCTCCCATGCTTTTGTTCTGGCCAGCTTG
SRR944633.94174440.1+                        TTCCTCTTGTTCCTCCCATGCTTTTGTTCTGGCCAGCTTG
SRR944639.795176.2+                          TTCCTCTTGTTCCTCCCATGCTTTTGTTCTGGCCAGCTTG
SRR944634.36413891.2+                            TCTTGTTCCTCCCATGCTTTTGTTCTGGCCAGCTTG
SRR944636.80493380.2-                            TCTTGTTCCTCCCATGCTTTTGTTCTGGCCAGCTTG
SRR944639.1190717.2+                                TGTTCCTCCCATGCTTTTGTTCTGGCCAGCTTG
SRR944632.124814170.1-                             GTTCCTCCCATGCTTTTGTTCTGGCCAGCTTG
SRR944636.12772580.1+                                 TTCCTCCCATGCTTTTGTTCTGGCCAGCTTG
SRR944636.68422628.1+                                 TTCCTCCCATGCTTTTGTTCTGGCCAGCTTG
SRR944637.42475624.1+                                  TCCTCCCATGCTTTTGTTCTGGCCAGCTTG
SRR944632.103969568.1+                                     CCCATGCTTTTGTTCTGGCCAGCTTG
SRR944633.37543341.1+                                       CCATGCTTTTGTTCTGGCCAGCTTG
SRR944639.51517071.1+                                       CCATGCTTTTGTTCTGGCCAGCTTG
SRR944637.29882792.1-                                           GCTTTTGTCCTGGCCAGCTTG
SRR944639.4540333.1+                                            GCTTTTGTTCTGGCCAGCTTG
SRR944634.95930937.1+                                            CTTTTGTTCTGGCCAGCTTG
SRR944634.96353591.2+                                            CTTTTGTTCTGGCCAGCTTG
SRR944631.20498242.2+                                             TTTTGTTCTGGCCAGCTTG
SRR944631.36807333.2+                                             TTTTGTTCTGGCCAGCTTG
SRR944631.50529730.2+                                             TTTTGTTCTGGCCAGCTTG
SRR944637.48110385.1-                                             TTTTGTTCTGGCCAGCTTG
SRR944639.46024088.2+                                              TTTGTTCTGGCCAGCTTG
SRR944639.11696087.2+                                               TTGTTCTGGCCAGCTTG
SRR944631.10860893.1+                                                  TTCTGGCCAGCTTG
SRR944631.50426033.1+                                                  TTCTGGCCAGCTTG
SRR944635.19297688.2+                                                   TCTGGCCAGCTTG
SRR944632.81552681.2-                                                    CTGGCCAGCTTG
SRR944633.33685071.2+                                                     TGGCCAGCTTG
SRR944636.87173253.2-                                                         CAGCTTG
SRR944639.22522669.2+                                                         CAGCTTG
SRR944639.60415629.2+                                                         CAGCTTG
SRR944632.98185584.2+                                                          AGCTTG
SRR944634.56993620.1+                                                          AGCTTG
SRR944636.92678193.1-                                                          AGCTTG
SRR944639.56680004.1+                                                           GCTTG
SRR944633.49669968.2+                                                            CTTG
SRR944633.82191784.2-                                                             TCC
SRR944634.54009543.1-                                                             TTC
SRR944634.61033746.2-                                                             TCC
SRR944638.25394220.1+                                                             TTG
SRR944638.83126803.1+                                                             TTG
SRR944635.75573623.2-                                                              CG
SRR944634.94919320.1-                                                               C
SRR944635.8277624.2-                                                                G

consensus                CAGGGATCAAGAGAATGCTCTTCCTCTTGTTCCTCCCATGCTTTTGTTCTGGCCAGCTTG

                             .    :    .    :    .    :    .    :    .    :    .    :
SRR944636.85657916.2+    CA
SRR944635.40200896.1-    CAC
SRR944636.85623294.1+    CACC
SRR944639.70391911.2+    CACCT
SRR944637.11188729.2+    CACCTC
SRR944634.54009543.2+    CACCTCC
SRR944631.8853804.1+     CACCTCCA
SRR944633.98416869.1-    CACCTCCA
```

SRR944633.98416869.2+ CACCTCCA
SRR944639.795176.2+ CACCTCCAC
SRR944637.16414866.2+ CACCTCCACT
SRR944635.52001870.1- CCCCTCCACTG
SRR944637.4122469.2+ CACCTCCACTG
SRR944633.40489199.2+ CACCTCCACTGCT
SRR944636.80493380.2- CACCTCCACTGCT
SRR944639.1190717.2+ CACCTCCACTGCTGCG
SRR944632.124814170.1- CACCTCCACTGCTGCGT
SRR944636.12772580.1+ CACCTCCACTGCTGCGTT
SRR944636.68422628.1+ CACCTCCACTGCTGCGTT
SRR944634.94919320.2+ CACCTCCACTGCTGCGTTTC
SRR944632.103969568.1+ CACCTCCACTGCTGCGTTTCCCC
SRR944634.46910015.2- CCCCTCCACTGCTGCGTTTCCCC
SRR944634.61033746.1+ CACCTCCACTGCTGCGTTTCCCCA
SRR944639.51517071.1+ CACCTCCACTGCTGCGTTTCCCCA
SRR944633.82191784.1+ CACCTCCACTGCTGCGTTTCCCCAC
SRR944634.77968149.1- CCCCTCCACTGCTGCGTTTCCCCACCT
SRR944639.4540333.1+ CACCTCCACTGCTGCGTTTCCCCACCTT
SRR944634.63040457.2+ CACCTCCACTGCTGCGTTTCCCCACCTTA
SRR944631.20498242.2+ CACCTCCACTGCTGCGTTTCCCCACCTTAT
SRR944631.36807333.2+ CACCTCCACTGCTGCGTTTCCCCACCTTAT
SRR944631.50529730.2+ CACCTCCACTGCTGCGTTTCCCCACCTTAT
SRR944633.25313478.1- CACCTCCACTGCTGCGTTTCCCCACCTTATT
SRR944639.46024088.2+ CACCTCCACTGCTGCGTTTCCCCACCTTATT
SRR944634.34545057.2+ CACCTCCACTGCTGCGTTTCCCCCCCTTATTG
SRR944639.11696087.2+ CACCTCCACTGCTGCGTTTCCCCACCTTATTG
SRR944633.90674241.2+ CACCTCCACTGCTGCGTTTCCCCACCTTATTGGA
SRR944637.20928485.2+ CACCTCCACTGCTGCGTTTCCCCACCTTATTGGA
SRR944631.10860893.1+ CACCTCCACTGCTGCGTTTCCCCACCTTATTGGAT
SRR944631.50426033.1+ CACCTCCACTGCTGCGTTTCCCCACCTTATTGGAT
SRR944634.29623047.1- CACCTCCACTGCTGCGTTTCCCCACCTTATTGGAT
SRR944634.3508483.2+ CACCTCCACTGCTGCGTTTCCCCACCTTATTGGAT
SRR944632.81552681.2- CCCCTCCACTGCTGCGTTTCCCCACCTTATTGGATCC
SRR944635.49727187.1+ CACCTCCACTGCTGCGTTTCCCCACCTTATTGGATCCTT
SRR944635.30275578.1- CCCCTCCACTGCTGCGTTTCCCCACCTTATTGGATCCTTCC
SRR944634.69071785.1+ CACCTCCACTGCTGCGTTTCCCCACCTTATTGGATCCTTCCA
SRR944636.87173253.2- CCCCTCCACTGCTGCGTTTCCCCACCTTATTGGATCCTTCCA
SRR944639.22522669.2+ CACCTCCACTGCTGCGTTTCCCCACCTTATTGGATCCTTCCA
SRR944639.60415629.2+ CACCTCCACTGCTGCGTTTCCCCACCTTATTGGATCCTTCCA
SRR944632.98185584.2+ CACCTCCACTGCTGCGTTTCCCCACCTTATTGGATCCTTCCAG
SRR944634.24301483.1- CCCCTCCACTGCTGCGTTTCCCCACCTTATTGGATCCTTCCAG
SRR944636.92678193.1- CACCTCCACTGCTGCGTTTCCCCACCTTATTGGATCCTTCCAG
SRR944639.56680004.1+ CACCTCCACTGCTGCGTTTCCCCACCTTATTGGATCCTTCCAGC
SRR944638.25394220.1+ CACCTCCACTGCTGCGTTTCCCCACCTTATTGGATCCTTCCAGCAT
SRR944638.83126803.1+ CACCTCCACTGCTGCGTTTCCCCACCTTATTGGATCCTTCCAGCAT
SRR944634.71474468.2+ CACCTCCACTGCTGCGTTTCCCCACCTTATTGGATCCTTCCAGCATG
SRR944633.22219140.2+ CACCTCCACTGCTGCGTTTCCCCACCTTATTGGATCCTTCCAGCATGTTCTA
SRR944633.54109791.1+ CACCTCCACTGCTGCGTTTCCCCACCTTATTGGATCCTTCCAGCATGTTCTACC
SRR944634.20628373.1- CCCCTCCACTGCTGCGTTTCCCCACCTTATTGGATCCTTCCAGCATGTTCTACC
SRR944633.98128369.2- CACCTCCACTGCTGCGTTTCCACACCTTATTGGATCCTTCCAGCATGTTCTACCT
SRR944633.99195671.1- CACCTCCACTGCTGCGTTTCCCCACCTTATTGGATCCTTCCAGCATGTTCTACCT
SRR944637.36954774.1+ CACCTCCACTGCTGCGTTTCCCCACCTTATTGGATCCTTCCCGCATGTTCTACCT
SRR944637.48405569.1+ CACCTCCACTGCTGCGTTTCCCCACCTTATTGGATCCTTCCAGCATGTTCTACCT
SRR944637.67596548.1+ CACCTCCACTGCTGCGTTTCCCCACCTTATTGGATCCTTCCAGCATGTTCTACCT
SRR944637.20349171.2+ CACCTCCACTGCTGCGTTTCCCCACCTTATTGGATCCTTCCAGCATGTTCTACCTCCAC
SRR944633.33685071.2+ CACCTCCACTGCTGCGTTTCCCCACCTTATTGGATCCTTCCAGCATGTTCTACCTCCACT
SRR944633.37543341.1+ CACCTCCACTGCTGCGTTTCCCCACCTTATTGGATCCTTCCAGCATGTTCTACCTCCACT
SRR944633.49669968.2+ CACCTCCACTGCTGCGTTTCCCCACCTTATTGGATCCTTCCAGCATGTTCTACCTCCACT
SRR944633.82191784.2- CCCCTCCACTGCTGCGTTTCCCCACCTTATTGGATCCTTCCAGCATGTTCTACCTCCACT
SRR944633.94174440.1+ CACCTCCACTGCTGCGTTTCCCCACCTTATTGGATCCTTCCAGCATGTTCTACCTCCACT
SRR944634.34545057.1- CCCCTCCACTGCTGCGTTTCCCCACCTTATTGGATCCTTCCAGCATGTTCTACCTCCACT
SRR944634.36413891.2+ CACCTCCACTGCTGCGTTTCCCCACCTTATTGGATCCTTCCAGCATGTTCTACCTCCACT
SRR944634.54009543.1- CCCCTCCACTGCTGCGTTTCCCCACCTTATTGGATCCTTCCAGCATGTTCTACCTCCACT
SRR944634.56993620.1+ CACCTCCACTGCTGCGTTTCCCCACCTTATTGGATCCTTCCAGCATGTTCTACCTCCACT
SRR944634.61033746.2- CCCCTCCACTGCTGCGTTTCCCCACCTTATTGGATCCTTCCAGCATGTTCTACCTCCACT
SRR944634.94919320.1- CCCCTCCGCTGCTGCGTTTCCCCACCTTATTGGATCCTTCCAGCATGTTCTACCTCCACT
SRR944634.95930937.1+ CACCTCCACTGCTGCGTTTCCCCACCTTATTGGATCCTTCCAGCATGTTCTACCTCCACT
SRR944634.96353591.2+ CACCTCCACTGCTGCGTTTCCCCACCTTATTGGATCCTTCCAGCATGTTCTACCTCCACT
SRR944635.19297688.2+ CACCTCCACTGCTGCGTTTCCCCACCTTATTGGATCCTTCCAGCATGTTCTACCTCCACT
SRR944635.75573623.2- CCCCTCCACTGCTCCGTTTCCCCACCTTATTGGATCCTCCCAGCATGTTCTCCCTCCACT
SRR944635.8277624.2- CCCCCCCACTGCTGCGTTTCCCCACCTTATTGGATCCTTCCAGCATGTTCTACCTCCACT
SRR944637.29882792.1- CCCCTCCACTGCTGCGTTTCCCCACCTTATTGGATCCTTCCAGCATGTTCTACCTCCACT
SRR944637.42475624.1+ CACCTCCACTGCTGCGTTTCCCCACCTTATTGGATCCTTCCAGCATGTTCTACTTCCACT
SRR944637.48110385.1- CCCCTCCACTGCTGCGTTTCCCCACCTTATTGGATCCTTCCAGCATGTTCTACCTCCACT
SRR944631.16048198.1- CCTCCACTGCTGCGTTTCCCCACCTTATTGGATCCTTCCAGCATGTTCT
SRR944631.20551822.1- CCTCCACTGCTGCGTTTCCCCACCTTATTGGATCCTTCCAGCATGTTCT
SRR944631.25176894.1- CCTCCACTGCTGCGTTTCCCCACCTTATTGGATCCTTCCAGCATGTTCT
SRR944631.52096585.1- CCTCCACTGCTGCGTTTCCCCACCTTATTGGATCCTTCCAGCATGTTCT
SRR944636.55339904.1- CCTCCACTGCTGCGTTTCCCCACCTTATTGGATCCTTCCAGCATGTTCT
SRR944634.63040457.1- CCTCCACTGCGGCGTTTCCCCACCTTATTGGATCCTTCCAGCATGTTCTACCTCCACT
SRR944630.24118277.2- CTCCACTGCTGCGTTTCCCCACCTTATTGGATCCTTCCAGCATGTTCTA
SRR944630.25715866.2- CTCCACTGCTGCGTTTCCCCACCTTATTGGATCCTTCCAGCATGTTCTA
SRR944630.56020051.2- CTCCACTGCTGCGTTTCCCCACCTTATTGGATCCTTCCAGCATGTTCTA

```
SRR944630.84361880.2-       CTCCACTGCTGCGTTTCCCCACCTTATTGGATCCTTCCAGCATGTTCTA
SRR944630.97039812.2-       CTCCACTGCTGCGTTTCCCCACCTTATTGGATCCTTCCAGCATGTTCTA
SRR944635.49884631.1-         CCACTGCTGCGTTTCCCCACCTTATTGGATCCTTCCAGCATGTTCTACCTCCACT
SRR944630.25453168.2+          CACTGCTGCGTTTCCCCACCTTATTGGATCCTTCCAGCATGTTCTACCT
SRR944630.53809540.1+          CACTGCTGCGTTTCCCCACCTTATTGGATCCTTCCAGCATGTTCTACCT
SRR944637.27932713.2+          CACTGCTGCGTTTCCCCACCTTATTGGATCCTTCCAGCATGTTCTACCTCCACT
SRR944634.27747891.1+           ACTGCTGCGTTTCCCCACCTTATTGGATCCTTCCAGCATGTTCTACCTCCACT
SRR944639.42258251.1+            CTGCTGCGTTTCCCCACCTTATTGGATCCTTCCAGCATGTTCTACCTCC
SRR944639.90379582.1+            CTGCTGCGTTTCCCCACCTTATTGGATCCTTCCAGCATGTTCTACCTCC
SRR944633.22219140.1-            CTGCTGCGTTTCCCCACCTTATTGGATCCTTCCAGCATGTTCTACCTCCACT
SRR944633.6245784.1+             CTGCTGCGTTTCCCCACCTTATTGGATCCTTCCAGCATGTTCTACCTCCACT
SRR944638.10407365.2-             TGCTGCGTTTCCCCACCTTATTGGATCCTTCCAGCATGTTCTACCTCCA
SRR944633.90674241.1-              GCTGCGTTTCCCCACCTTATTGGATCCTTCCAGCATGTTCTACCTCCACT
SRR944634.3508483.1-               GCTGCTTTTCCCCCCCTTATTGGATCCTTCCAGCATGTTCTACCTCCACT
SRR944636.39659613.2+                  CGTTTCCCCACCTTATTGGATCCTTCCAGCATGTTCTACCTCCACT
SRR944632.54147278.1+                   GTTTCCCCACCTTATTGGATCCTTCCAGCATGTTCTACCTCCACT
SRR944632.59154265.1-                   GTTTCCCCACCTTATTGGATCCTTCCAGCATGTTCTACCTCCACT
SRR944634.10483507.1+                   GTTTCCCCACCTTATTGGATCCTTCCAGCATGTTCTACCTCCACT
SRR944635.36734830.2+                   GTTTCCCCACCTTATTGGATCCTTCCAGCATGTTCTACCTCCACT
SRR944639.40454281.2+                   GTTTCCCCACCTTATTGGATCCTTCCAGCATGTTCTACCTCCACT
SRR944634.18725566.1+                    TTTCCCCACCTTATTGGATCCTTCCAGCATGTTCTACCTCCACT
SRR944637.66585051.1-                    TTTCCCCACCTTATTGGATCCTTCCAGCATGTTCTACCTCCACT
SRR944638.19970532.1+                    TTTCCCCACCTTATTGGATCCTTCCAGCATGTTCTACCTCCACT
SRR944638.4524910.1+                     TTTCCCCACCTTATTGGATCCTTCCAGCATGTTCTACCTCCACT
SRR944639.27712427.2+                    TTTCCCCACCTTATTGGATCCTTCCAGCATGTTCTACCTCCACT
SRR944639.8021464.2+                     TTTCCCCACCTTATTGGATCCTTCCAGCATGTTCTACCTCCACT
SRR944631.38021789.1+                     TTCCCCACCTTATTGGATCCTTCCAGCATGTTCTACCTCCACT
SRR944631.45456746.1+                     TTCCCCACCTTATTGGATCCTTCCAGCATGTTCTACCTCCACT
SRR944631.46791067.1+                     TTCCCCACCTTATTGGATCCTTCCAGCATGTTCTACCTCCACT
SRR944634.44547124.1+                     TTCCCCACCTTATTGGATCCTTCCAGCATGTTCTACCTCCACT
SRR944639.40231068.1+                     TTCCCCACCTTATTGGATCCTTCCAGCATGTTCTACCTCCACT
SRR944632.58349281.2-                      TCCCCACCTTATTGGATCCTTCCAGCATGTTCTACCTCCACT
SRR944632.68184668.2-                      TCCCCACCTTATTGGATCCTTCCAGCATGTTCTACCTCCACT
SRR944634.69071785.2-                       CCCCCCCTTATTGGATCCTCCCAGCATGTTCTCCCTCCACT
SRR944638.26820928.1+                       CCCCACCTTATTGGATCCTTCCAGCATGTTCTACCTCCACT
SRR944630.121406811.1-                       CCCACCTTATTGGATCCTTCCAGCATGTTCTACCTCCACT
SRR944630.22625207.1-                        CCCACCTTATTGGATCCTTCCAGCATGTTCTACCTCCACT
SRR944630.63102084.1-                        CCCACCTTATTGGATCCTTCCAGCATGTTCTACCTCCACT
SRR944630.89008962.1-                        CCCACCTTATTGGATCCTTCCAGCATGTTCTACCTCCACT
SRR944633.5304783.2+                         CCCACCTTATTGGATCCTTCCAGCATGTTCTACCTCCACT
SRR944637.60761079.2+                          CACCTTATTGGATCCTTCCAGCATGTTCTACCTCCACT
SRR944632.44981717.1+                            CCTTATTGGATCCTTCCAGCATGTTCTACCTCCACT
SRR944634.71474468.1-                            CCTTATTGGATCCTTCCAGCATGTTCTACCTCCACT
SRR944635.81118867.1-                             CTTATTGGATCCTTCCAGCATGTTCTCCCTCCACT
SRR944636.41076715.1+                              TTATTGGATCCTTCCAGCATGTTCTACCTCCACT
SRR944636.4281731.2+                               TTATTGGATCCTTCCAGCATGTTCTACCTCCACT
SRR944636.608439.1+                                TTATTGGATCCTTCCAGCATGTTCTACCTCCACT
SRR944639.25479476.1+                               TATTGGATCCTTCCAGCATGTTCTACCTCCACT
SRR944636.64757170.2+                                 TTGGATCCTTCCAGCATGTTCTACCTCCACT
SRR944636.73199438.1+                                 TTGGATCCTTCCAGCATGTTCTACCTCCACT
SRR944636.85911281.1+                                 TTGGATCCTTCCAGCATGTTCTACCTCCACT
SRR944632.18161690.2+                                  TGGATCCTTCCAGCATGTTCTACCTCCACT
SRR944632.20945602.2+                                  TGGATCCTTCCAGCATGTTCTACCTCCACT
SRR944632.113114427.2+                                   GATCCTTCCAGCATGTTCTACCTCCACT
SRR944635.6426379.1-                                     GATCCTTCCAGCATGTTCTACCTCCACT
SRR944636.66276717.1+                                    GATCCTTCCAGCATGTTCTACCTCCACT
SRR944635.90005215.1-                                      TCCTCCCAGCATGTTCTCCCTCCACT
SRR944639.91169517.1-                                      TCCTTCCAGCATGTTCTACCTCCACT
SRR944636.82506820.2+                                       CCTTCCAGCATGTTCTACCTCCACT
SRR944633.6245784.2-                                         CTCCCAGCATTTTCCACCTCCACT
SRR944635.57178392.1+                                        CTTCCAGCATGTTCTACCTCCACT
SRR944637.2949431.2-                                         CTCCCAGCATGTTCTACCTCCACT
SRR944638.23438986.2+                                        CTTCCAGCATGTTCTACCTCCACT
SRR944638.61445856.2+                                        CTTCCAGCATGTTCTACCTCCACT
SRR944638.80030283.2+                                        CTTCCAGCATGTTCTACCTCCACT
SRR944636.31398530.1-                                          TCCAGCATGTTCTACCTCCACT
SRR944632.66509157.2-                                            CAGCATGTTCTCCCTCCACT
SRR944634.90107683.2+                                            CAGCATGTTCTACCTCCACT
SRR944636.1766768.1-                                              AGCATGTTCTACCTCCACT
SRR944636.61934370.1-                                             AGCATGTTCTACCTCCACT
SRR944637.21269815.2+                                             AGCATGTTCTACCTCCACT
SRR944635.11655522.1+                                               CATGTTCTACCTCCACT
SRR944639.9343527.1+                                                CATGTTCTACCTCCACT
SRR944631.43349604.2+                                                ATGTTCTACCTCCACT
SRR944633.96164481.1+                                                ATGTTCTACCTCCACT
SRR944633.100415876.2+                                                TGTTCTACCTCCACT
SRR944630.70037776.2+                                                  GTTCTACCTCCACT
SRR944633.66408716.2+                                                  GTTCTACCTCCACT
SRR944633.54109791.2-                                                   TTCTCCCTCCACT
SRR944634.36413891.1-                                                   TTCTCCCTCCACT
SRR944633.58893957.2+                                                    TCTACCTCCACT
SRR944633.70260610.1+                                                    TCTACCTCCACT
SRR944632.195484.2+                                                        CTACCTCCACT
SRR944632.96398654.2+                                                      CTACCTCCACT
```

```
SRR944636.32951693.1+                                                      TACCTCCACT
SRR944634.96353591.1-                                                       CCCTCCACT
SRR944637.32814322.2-                                                       CCCTCCACT
SRR944635.13217570.2-                                                        CCTCTACT
SRR944639.41608196.2+                                                        CCTCCACT
SRR944633.49669968.1-                                                         CTCCACT
SRR944636.40446523.1-                                                          TCCACT
SRR944632.98092518.2+                                                           CCACT
SRR944634.95930937.2-                                                           CCACT
SRR944637.41492408.2+                                                            CACT
SRR944639.35501012.2+                                                            CACT
SRR944630.3862609.2-                                                              ACT
SRR944630.32863949.2-                                                              CT
SRR944633.33685071.1-                                                              CT
SRR944633.37543341.2-                                                              CT
SRR944633.91746479.2+                                                               T
SRR944634.8289892.1+                                                                T

consensus                CACCTCCACTGCTGCGTTTCCCCACCTTATTGGATCCTTCCAGCATGTTCTACCTCCACT

                             .    :    .    :    .    :    .    :    .    :    .    :
SRR944634.36413891.2+    GGG
SRR944636.39659613.2+    GGG
SRR944637.48110385.1-    GGG
SRR944632.54147278.1+    GGGA
SRR944632.59154265.1-    GGGA
SRR944639.40454281.2+    GGGA
SRR944638.19970532.1+    GGGAC
SRR944638.4524910.1+     GGGAC
SRR944639.27712427.2+    GGGAC
SRR944639.8021464.2+     GGGAC
SRR944631.38021789.1+    GGGACC
SRR944631.45456746.1+    GGGACC
SRR944631.46791067.1+    GGGACC
SRR944639.40231068.1+    GGGACC
SRR944632.58349281.2-    GGGACCA
SRR944632.68184668.2-    GGGACCA
SRR944638.26820928.1+    GGGACCAC
SRR944630.121406811.1-   GGGACCACG
SRR944630.22625207.1-    GGGACCACG
SRR944630.63102084.1-    GGGACCACG
SRR944630.89008962.1-    GGGACCACG
SRR944637.42475624.1+    GGGACCACGA
SRR944632.44981717.1+    GGGACCACGAGGA
SRR944633.37543341.1+    GGGACCACGAGGAAC
SRR944636.41076715.1+    GGGACCACGAGGAAC
SRR944636.4281731.2+     GGGACCACGAGGAAC
SRR944636.608439.1+      GGGACCACGAGGAAC
SRR944639.25479476.1+    GGGACCACGAGGAACA
SRR944636.64757170.2+    GGGACCACGAGGAACAGG
SRR944636.73199438.1+    GGGACCACGAGGAACAGG
SRR944636.85911281.1+    GGGACCACGAGGAACAGG
SRR944632.18161690.2+    GGGACCACGAGGAACAGGA
SRR944632.20945602.2+    GGGACCACGAGGAACAGGA
SRR944635.8277624.2-     GGGACCACGAGGAACAGGA
SRR944637.29882792.1-    GGGACCACGAGGAACAGGA
SRR944634.95930937.1+    GGGACCACTAGGAACAGGAG
SRR944634.96353591.2+    GGGACCACGAGGAACAGGAG
SRR944632.113114427.2+   GGGACCACGAGGAACAGGAGC
SRR944636.66276717.1+    GGGACCACGAGGAACAGGAGC
SRR944635.19297688.2+    GGGACCACGAGGAACAGGAGCTG
SRR944639.91169517.1-    GGGACCACGAGGAACAGGAGCTG
SRR944634.54009543.1-    GGGACCACGAGGAACAGGAGCTGA
SRR944636.82506820.2+    GGGACCACGAGGAACAGGAGCTGA
SRR944638.23438986.2+    GGGACCACGAGGAACAGGAGCTGAT
SRR944638.61445856.2+    GGGACCACGAGGAACAGGAGCTGAT
SRR944638.80030283.2+    GGGACCACGAGGAACAGGAGCTGAT
SRR944636.31398530.1-    GGGACCACGAGGAACAGGAGCTGATGA
SRR944632.66509157.2-    GGGACCACGAGGAACAGGAGCTGATGAGG
SRR944633.33685071.2+    GGGACCACGAGGAACAGGAGCTGATGAGG
SRR944633.82191784.2-    GGGACCACGAGGAACAGGAGCTGATGAGGT
SRR944636.1766768.1-     GGGACCACGAGGAACAGGAGCTGATGAGGT
SRR944636.61934370.1-    GGGACCACGAGGAACAGGAGCTGATGAGGT
SRR944635.75573623.2-    GGGACCACGAGGAACAGGAGCTGATGAGGTT
SRR944639.9343527.1+     GGGACCACGAGGAACAGGAGCTGATGAGGTTG
SRR944631.43349604.2+    GGGACCACGAGGAACAGGAGCTGATGAGGTTGA
SRR944634.61033746.2-    GGGACCACGAGGAACAGGAGCTGATGAGGTTGA
SRR944634.56993620.1+    GGGACCACGAGGAACAGGAGCTGATGAGGTTGAG
SRR944630.70037776.2+    GGGACCACGAGGAACAGGAGCTGATGAGGTTGAGC
SRR944633.49669968.2+    GGGACCACGAGGAACAGGAGCTGATGAGGTTGAGCT
SRR944634.94919320.1-    GGGACCACGAGGAACAGGAGCTGATGAGGTTGAGCT
SRR944632.195484.2+      GGGACCACGAGGAACAGGAGCTGATGAGGTTGAGCTGC
SRR944632.96398654.2+    GGGACCACGAGGAACAGGAGCTGATGAGGTTGAGCTGC
SRR944636.32951693.1+    GGGACCACGAGGAACAGGAGCTGATGAGGTTGAGCTGCA
```

```
SRR944634.34545057.1-    GGGACCACGAGGAACAGGAGCTGATGAGGTTGAGCTGCAC
SRR944639.41608196.2+    GGGACCACGAGGAACAGGAGCTGATGAGGTTGAGCTGCACA
SRR944634.63040457.1-    GGGACCACGAGGAACAGGAGCTGATGAGGTTGAGCTGCACAT
SRR944636.40446523.1-    GGGACCACGAGGAACAGGAGCTGATGAGGTTGAGCTGCACATC
SRR944632.98092518.2+    GGGACCACGAGGAACAGGAGCTGATGAGGTTGAGCTGCACATCC
SRR944637.27932713.2+    GGGACCACGAGGAACAGGAGCTGATGAGGTTGAGCTGCACATCC
SRR944635.49884631.1-    GGGACCACGAGGAACAGGAGCTGATGAGGTTGAGCTGCACATCCA
SRR944639.35501012.2+    GGGACCACGAGGAACAGGAGCTGATGAGGTTGAGCTGCACATCCA
SRR944630.1730641.2-     GGGACCACGAGGAACAGGAGCTGATGAGGTTGAGCTGCACATCCAC
SRR944630.32863949.2-    GGGACCACGAGGAACAGGAGCTGATGAGGTTGAGCTGCACATCCAC
SRR944630.3862609.2-     GGGACCACGAGGAACAGGAGCTGATGAGGTTGAGCTGCACATCCAC
SRR944633.22219140.1-    GGGACCACGAGGAACAGGAGCTGATGAGGTTGAGCTGCACATCCAC
SRR944634.27747891.1+    GGGACCACGAGGAACAGGAGCTGATGAGGTTGAGCTGCACATCCACA
SRR944633.6245784.1+     GGGACCACGAGGAACAGGAGCTGATGAGGTTGAGCTGCACATCCACAC
SRR944630.25461350.2-    GGGACCACGAGGAACAGGAGCTGATGAGGTTGAGCTGCACATCCACACA
SRR944630.5400523.2-     GGGACCACGAGGAACAGGAGCTGATGAGGTTGAGCTGCACATCCACACA
SRR944630.74999846.2-    GGGACCACGAGGAACAGGAGCTGATGAGGTTGAGCTGCACATCCACACA
SRR944633.90674241.1-    GGGACCACGAGGAACAGGAGCTGATGAGGTTGAGCTGCACATCCACACAA
SRR944634.3508483.1-     GGGACCACGAGGAACAGGAGCTGATGAGGTTGAGCTGCACATCCACACAA
SRR944637.66585051.1-    GGGACCACGAGGAACAGGAGCTGATGAGGTTGAGCTGCACATCCACACAACTGG
SRR944634.10483507.1+    GGGACCACGAGGAACAGGAGCTGATGAGGTTGAGCTGCACATCCACACAACTGGC
SRR944634.69071785.2-    GGGACCACGAGGAACAGGAGCTGATGAGGTTGAGCTGCACATCCACACAACTGGC
SRR944635.36734830.2+    GGGACCACGAGGAACAGGAGCTGATGAGGTTGAGCTGCACATCCCCACAACTGGC
SRR944634.18725566.1+    GGGACCACGAGGAACAGGAGCTGATGAGGTTGAGCTGCACATCCACACAACTGGCT
SRR944635.13217570.2-    GGGACCTCGAGGAACAGGAGCTGATGAGGGTGAGCTGCCCATCCACACAACTGGCT
SRR944634.44547124.1+    GGGACCACGAGGAACAGGAGCTGATGAGGTTGAGCTGCACATCCACACAACTGGCTG
SRR944633.100415876.2+   GGGACCACGAGGAACAGGAGCTGATGAGGTTGAGCTGCACATCCACACAACTGGCTGGAA
SRR944633.33685071.1-    GGGACCACGAGGAACAGGAGCTGATGAGGTTGAGCTGCACATCCACACAACTGGCTGGAT
SRR944633.37543341.2-    GGGACCACGAGGAACAGGAGCTGATGAGGTTGAGCTGCACATCCACACAACTGGCTGGAT
SRR944633.49669968.1-    GGGACCACGAGGAACAGGAGCTGATGAGGTTGAGCTGCACATCCACACAACTGGCTGGAT
SRR944633.5304783.2+     GGGACCACGAGGAACAGGAGCTGATGAGGTTGAGCTGCACATCCACACAACTGGCTGGAT
SRR944633.54109791.2-    GGGACCACGAGGAACAGGAGCTGATGAGGTTGAGCTGCACATCCACACAACTGGCTGGAT
SRR944633.58893957.2+    GGGACCACGAGGAACAGGAGCTGATGAGGTTGAGCTGCACATCCACACAACTGGCTGGAA
SRR944633.6245784.2-     GGGACCACGAGGAACAGGAGCTGATGAGGTTGAGCTGCACATCCACACAACTGGCTGGAT
SRR944633.66408716.2+    GGGACCACGAGGAACAGGAGCTGATGAGGTTGAGCTGCACATCCACACAACTGGCTGGAT
SRR944633.70260610.1+    GGGACCACGAGGAACAGGAGCTGATGAGGTTGAGCTGCACATCCACACAACTGGCTGGAT
SRR944633.91746479.2+    GGGACCACGAGGAACAGGAGCTGATGAGGTTGAGCTGCACATCCACACAACTGGCTGGAT
SRR944633.96164481.1+    GGGACCACGAGGAACAGGAGCTGATGAGGTTGAGCTGCACATCCACACAACTGGCTGGAT
SRR944634.36413891.1-    GGGACCACGAGGAACAGGAGCTGATGAGGTTGAGCTGCACATCCACACAACTGGCTGGAT
SRR944634.71474468.1-    GGGACCACGAGGAACAGGAGCTGATGAGGTTGAGCTGCACATCCACACAACTGGCTGGAT
SRR944634.8289892.1+     GGGACCACGAGGAACAGGAGCTGATGAGGTTGAGCTGCACATCCACACAACTGGCTGGAT
SRR944634.90107683.2+    GGGACCACGAGGAACAGGAGCTGATGAGGTTGAGCTGCACATCCACACAACTGGCTGGAT
SRR944634.95930937.2-    GGGACCACGAGGAACAGGAGCTGATGAGGTTGAGCTGCACATCCACACAACTGGCTGGAT
SRR944634.96353591.1-    GGGACCACGAGGAACAGGAGCTGATGAGGTTGAGCTGCACATCCACACAACTGGCTGGAT
SRR944635.11655522.1+    GGGACCACGAGGAACAGGAGCTGATGAGGTTGAGCTGCACATCCACACAACTGGCTGGAT
SRR944635.35994372.1-    GGGACCACGAGGAACAGGAGCTGATGAGGTTGAGCTGCACATCCACACAACTGGCTGGAT
SRR944635.57178392.1+    GGGACCACGAGGAACAGGAGCTGATGAGGTTGAGCTGCACATCCACACAACTGGCTGGAT
SRR944635.6426379.1-     GGGACCACGAGGAACAGGAGCTGATGAGGTTGAGCTGCACATCCACACAACTGGCTGGAT
SRR944635.81118867.1-    GGGACCACGAGGAACAGGAGCTGATGAGGTTGAGCTGCACATCCACACAACTGGCTGGAT
SRR944635.90005215.1-    GGGACCACGAGGAACAGGAGCTGATGAGGTTGAGCTGCACATCCACACAACTGGCTGGAT
SRR944637.21269815.2+    GGGACCACGAGGAACAGGAGCTGATGAGGTTGAGCTGCACATCCACACAACTGGCTGGAT
SRR944637.2949431.2-     GGGACCACGAGGAACAGGAGCTGATGAGGTTGAGCTGCACATCCACACAACTGGCTGGAT
SRR944637.32814322.2-    GGGCCCACGAGGAACAGGAGCTGATGAGGTTGAGCTGCACATCCACACAACTGGCTGGAT
SRR944637.41492408.2+    GGGACCACGAGGAACAGGAGCTGATGAGGTTGAGCTGCACATCCACACAACTGGCTGGAT
SRR944637.60761079.2+    GGGACCACGAGGAACAGGAGCTGATGAGGTTGAGCTGCACATCCACACAACTGGCTGGAT
SRR944636.61145333.1+       ACCACGAGGAACAGGAGCTGATGAGGTTGAGCTGCACATCCACACAACT
SRR944636.88140504.1-       ACCACGAGGAACAGGAGCTGATGAGGTTGAGCTGCACATCCACACAACT
SRR944630.3117193.2+         CCACGAGGAACAGGAGCTGATGAGGTTGAGCTGCACATCCACACAACTG
SRR944630.39077923.2+        CCACGAGGAACAGGAGCTGATGAGGTTGAGCTGCACATCCACACAACTG
SRR944630.87553480.2+        CCACGAGGAACAGGAGCTGATGAGGTTGAGCTGCACATCCACACAACTG
SRR944636.8868960.2+          CACGAGGAACAGGAGCTGATGAGGTTGAGCTGCACATCCACACAACTGG
SRR944632.39371924.2+            GAGGAACAGGAGCTGATGAGGTTGAGCTGCACATCCACACAACTGGCTG
SRR944632.6491554.2-             GAGGAACAGGAGCTGATGAGGTTGAGCTGCACATCCACACAACTGGCTG
SRR944633.26132114.1+            GAGGAACAGGAGCTGATGAGGTTGAGCTGCACATCCACACAACTGGCTGGAT
SRR944632.102059811.1+            AGGAACAGGAGCTGATGAGGTTGAGCTGCACATCCACACAACTGGCTGG
SRR944634.18725566.2-              GGAACAGGAGCTGATGAGGTTGAGCTGCACATCCACACAACTGGCTGGAT
SRR944633.72364928.2+                 ACAGGAGCTGATGAGGTTGAGCTGCACATCCACACAACTGGCTGGAT
SRR944638.34568035.2+                   AGGAGCTGATGAGGTTGAGCTGCACATCCACACAACTGGCTGGAT
SRR944638.39020624.2+                   AGGAGCTGATGAGGTTGAGCTGCACATCCACACAACTGGCTGGAT
SRR944634.44547124.2-                    GGAGCTGATGAGGTTGAGCTGCACATCCACACAACTGGCTGGAT
SRR944635.57173909.1+                    GGAGCTGATGAGGTTGAGCTGCACATCCACACAACTGGCTGGAT
SRR944632.13271979.1-                      AGCTGATGAGGTTGAGCTGCACATCCACACAACTGGCTGGAT
SRR944632.30320650.1-                      AGCTGATGAGGTTGAGCTGCACATCCACACAACTGGCTGGAT
SRR944635.59792053.2+                      AGCTGATGAGGTTGAGCTGCACATCCACACAACTGGCTGGAT
SRR944636.71511825.1-                       GCTGATGAGGTTGAGCTGCACATCCACACAACTGGCTGGAT
SRR944634.56993620.2-                        CTGATGAGGTTGAGCTGCACATCCACACAACTGGCTGGAT
SRR944635.26842443.1-                        CTGATGAGGTTGAGCTGCACATCCACACAACTGGCTGGAT
SRR944635.55524370.2+                        CTGATGAGGTTGAGATGCACATCCACACAACTGGCAGGAT
SRR944635.67387987.2+                        CTGATGAGGTTGAGCTGCACATCCACACAACTGGCTGGAT
SRR944630.53677100.2+                         TGATGAGGTTGAGCTGCACATCCACACAACTGGCTGGAT
SRR944634.60100148.2+                         TGATGAGGTTGAGCTGCACATCCACACAACTGGCTGGAT
SRR944639.50819808.2+                         TGATGAGGTTGAGCTGCACATCCACACAACTGGCTGGAT
SRR944636.7358424.1+                           GATGAGGTTGAGCTGCACATCCACACAACTGGCTGGAT
SRR944634.27747891.2-                           ATGAGGTTGAGCTGCACATCCACACAACTGGCTGGAT
```

```
SRR944637.18002670.2-                            TGAGGTGGAGCTGCACATCCACACAACTGGCTGGAT
SRR944636.38241941.1+                             GAGGTTGAGCTGCACATCCACACAACTGGCTGGAT
SRR944636.26614040.1+                               GGTTGAGCTGCACATCCACACAACTGGCTGGAT
SRR944632.2794312.1+                                 GTTGAGCTGCACATCCACACAACTGGCTGGAT
SRR944632.52740312.1+                                GTTGAGCTGCACATCCACACAACTGGCTGGAT
SRR944634.10483507.2-                                 TTGAGCTGCATCTCCACACAACAGGCTGGAT
SRR944635.81516722.2-                                 TTGAGCTGCACATCCACACAACTGGCTGGAT
SRR944632.100497177.1+                                  GAGCTGCACATCCACACAACTGGCTGGAT
SRR944632.47848021.1-                                   GAGCTGCACATCCACACAACTGGCTGGAT
SRR944638.33077779.1-                                   GAGCTGCACATCCACACAACTGGCTGGAT
SRR944638.50525901.1-                                   GAGCTGCACATCCACACAACTGGCTGGAT
SRR944638.9113724.1-                                    GAGCTGCACATCCACACAACTGGCTGGAT
SRR944635.25751445.2-                                    AGCTGCCCATCCACACAACTGGCTGGAT
SRR944635.48675781.2-                                      CTGCACATCCACACAACTGGCTGGAT
SRR944637.18612130.2+                                      CTGCACATCCACACAACTGGCTGGAT
SRR944636.6219837.1+                                        TGCACATCCACACAACTGGCTGGAT
SRR944637.48990847.2-                                        GCACATCCACACAACTGGCTGGAT
SRR944633.55088954.1+                                          ACATCCACACAACTGGCTGGAT
SRR944636.14867043.2-                                          ACATCCACACAACTGGCTGGAT
SRR944639.36153102.1+                                          ACATCCACACAACTGGCTGGAT
SRR944635.48191009.2-                                            ATCCACACAACTGGCTGGAT
SRR944638.17491490.2-                                            ATCCACACAACTGGCTGGAT
SRR944638.24792007.2-                                            ATCCACACAACTGGCTGGAT
SRR944636.47374346.2-                                              CCACACAACTGGCTGGAT
SRR944638.63988209.1+                                               CACACAACTGGCTGGAT
SRR944639.95430747.1-                                                ACACAACTGGCTGGAT
SRR944633.58893957.1-                                                 CACAACTGGCTGGAT
SRR944632.61973592.2+                                                  ACAACTGGCTGGAT
SRR944633.22731044.1+                                                  ACAACTGGCTGGAT
SRR944633.5304783.1-                                                     AACTGGCTGGAT
SRR944634.90107683.1-                                                     ACTGGCTGGAT
SRR944634.62072555.1+                                                       TGGCTGGAT
SRR944638.363258.2-                                                          GGCTGGAT
SRR944634.36751401.2+                                                         GCTGGAT
SRR944637.41203321.1+                                                          CTGGAT
SRR944639.83356177.2+                                                          CTGGAT
SRR944630.111468291.2-                                                          TGGAT
SRR944630.115766130.2-                                                          TGGAT
SRR944630.22557989.2-                                                           TGGAT
SRR944638.29797893.1+                                                            GGAT
SRR944638.77656764.1+                                                            GGAT
SRR944633.100415876.1-                                                            GAT
SRR944632.102005917.2+                                                             AT
SRR944632.30311077.2+                                                              AT
SRR944633.70260610.2-                                                              AT
SRR944633.66408716.1-                                                               T
SRR944639.71512403.1+                                                               T

consensus                GGGACCACGAGGAACAGGAGCTGATGAGGTTGAGCTGCACATCCACACAACTGGCTGGAT

                             .    :    .    :    .    :    .    :    .    :    .    :
SRR944637.21269815.2+    GGC
SRR944633.100415876.2+   GGCA
SRR944634.71474468.1-    GGCA
SRR944638.34568035.2+    GGCA
SRR944638.39020624.2+    GGCA
SRR944635.81118867.1-    GGCAT
SRR944632.13271979.1-    GGCATCT
SRR944632.30320650.1-    GGCATCT
SRR944636.71511825.1-    GGCATCTG
SRR944636.7358424.1+     GGCATCTGG
SRR944630.53677100.2+    GGCATCTGGA
SRR944639.50819808.2+    GGCATCTGGA
SRR944633.6245784.2-     GGCATCTGGATT
SRR944635.6426379.1-     GGCATCTGGATT
SRR944635.90005215.1-    GGCATCTGGATTCA
SRR944636.38241941.1+    GGCATCTGGATTCA
SRR944637.32814322.2-    GGCATCTGGATTCA
SRR944635.57178392.1+    GGCATCTGGATTCAGC
SRR944636.26614040.1+    GGCATCTGGATTCAGC
SRR944637.2949431.2-     GGCATCTGGATTCAGC
SRR944632.2794312.1+     GGCATCTGGATTCAGCC
SRR944632.52740312.1+    GGCATCTGGATTCAGCC
SRR944632.100497177.1+   GGCATCTGGATTCAGCCCTC
SRR944632.47848021.1-    GGCATCTGGATTCAGCCCTC
SRR944634.90107683.2+    GGCATCTGGATTCAGCCCTC
SRR944638.33077779.1-    GGCATCTGGATTCAGCCCTC
SRR944638.50525901.1-    GGCATCTGGATTCAGCCCTC
SRR944638.9113724.1-     GGCATCTGGATTCAGCCCTC
SRR944633.96164481.1+    GGGATCTGGATTCAGCCCTCATG
SRR944635.11655522.1+    GGCATCTGGATTCAGCCCTCATG
SRR944636.6219837.1+     GGCATCTGGATTCAGCCCTCATGG
SRR944633.58893957.2+    GGCATCTGGATTCAGCCCTCCTGGAG
SRR944633.66408716.2+    GGCATATGGATTCAGCCCTCATGGAG
```

```
SRR944634.36413891.1-    GGCATCTGGATTCAGCCCTCATGGAGA
SRR944634.96353591.1-    GGCATCTGGATTCAGCCCTCATGGAGA
SRR944636.14867043.2-    GGCATCTGGATTCAGCCCTCATGGAGA
SRR944639.36153102.1+    GGCATCTGGATTCAGCCCTCATGGAGA
SRR944633.70260610.1+    GGCATCTGGATTCAGCCCTCATGGAGAG
SRR944638.17491490.2-    GGCATCTGGATTCAGCCCTCATGGAGAGC
SRR944638.24792007.2-    GGCATCTGGATTCAGCCCTCATGGAGAGC
SRR944636.47374346.2-    GGCATCTGGATTCAGCCCTCATGGAGAGCTG
SRR944638.63988209.1+    GGCATCTGGATTCAGCCCTCATGGAGAGCTGC
SRR944633.49669968.1-    GGCATCTGGATTCAGCCCTCATGGAGAGCTGCC
SRR944639.95430747.1-    GGCATCTGGATTCAGCCCTCATGGAGAGCTGCC
SRR944632.61973592.2+    GGCATCTGGATTCAGCCCTCATGGAGAGCTGCCTG
SRR944634.95930937.2-    GGCATCTGGATTCAGCCCTCATGGAGAGCTGCCTG
SRR944637.41492408.2+    GGCATCTGGATTCAGCCCTCCTGGAGAGCTGCCTG
SRR944633.37543341.2-    GGCATCTGGATTCAGCCCTCATGGAGAGCTGCCTGG
SRR944633.33685071.1-    GGCATCTGGATTCAGCCCTCATGGAGAGCTGCCTGGAT
SRR944635.35994372.1-    GGCATCTGGATTCAGCCCTCATGGAGAGCTGCCTGGAT
SRR944633.91746479.2+    GGCATCTGGATTCAGCCCTCATGGAGAGCTGCCTGGATA
SRR944634.8289892.1+     GGCATCTGGATTCAGCCCTCATGGAGAGCTGCCTGGATA
SRR944638.363258.2-      GGCATCTGGATTCAGCCCTCATGGAGAGCTGCCTGGATATA
SRR944639.83356177.2+    GGCATCTGGATTCAGCCCTCATGGAGAGCTGCCTGGATATAAC
SRR944630.111468291.2-   GGCATCTGGATTCAGCCCTCATGGAGAGCTGCCTGGATATAACA
SRR944630.115766130.2-   GGCATCTGGATTCAGCCCTCATGGAGAGCTGCCTGGATATAACA
SRR944630.22557989.2-    GGCATCTGGATTCAGCCCTCATGGAGAGCTGCCTGGATATAACA
SRR944635.55524370.2+    GGCATCTGGATTCAGGCCTCATGGAGAGCTGCCCGGATATAACA
SRR944638.29797893.1+    GGCATCTGGATTCAGCCCTCATGGAGAGCTGCCTGGATATAACAT
SRR944638.77656764.1+    GGCATCTGGATTCAGCCCTCATGGAGAGCTGCCTGGATATAACAT
SRR944632.102005917.2+   GGCATCTGGATTCAGCCCTCATGGAGAGCTGCCTGGATATAACATTG
SRR944632.30311077.2+    GGCATCTGGATTCAGCCCTCATGGAGAGCTGCCTGGATATAACATTG
SRR944633.26132114.1+    GGCATCTGGATTCAGCCCTCATGGAGAGCTGCCTGGATATAACATTGT
SRR944639.71512403.1+    GGCATCTGGATTCAGCCCTCATGGAGAGCTGCCTGGATATAACATTGT
SRR944633.72364928.2+    GGCATCTGGATTCAGCCCTCATGGAGAGCTGCCTGGATATAACATTGTGA
SRR944634.18725566.2-    GGCATCTGGATTCAGCCCTCATGGAGAGCTGCCTGGATATAACATTGTGA
SRR944635.59792053.2+    GGCATCTGGATTCAGCCCTCATGGAGAGCTGCCTGGATATAACATTGTGAC
SRR944634.60100148.2+    GGCATCTGGATTCAGCCCTCATGGAGAGCTGCCTGGGTATAACATTGTGACAGGG
SRR944635.57173909.1+    GGGATATGGGTTCAGCCCTCATTGAGAGCTGCCTGGATATAACATTGTGACAGGG
SRR944634.44547124.2-    GGCATCTGGATTCAGCCCTCATGGAGAGCTGCCTGGATATAACATTGTGACAGGAG
SRR944634.10483507.2-    GGCATCTGGATTCAGCCCTCATGGAGAGCTGCCTGGATATAACATTGTGACAGGAGGT
SRR944637.18002670.2-    GGCATCTGGATTCAGCCCTCATGGAGAGCTGCCTGGATATAACATTGTGACAGGAGGT
SRR944633.100415876.1-   GGCATCTGGATTCAGCCCTCATGGAGAGCTGCCTGGATATAACATTGTGACAGGAGGTGC
SRR944633.12567580.1+    GGCATCTGGATTCAGCCCTCATGGAGAGCTGCCTGGATATAACATTGTGACAGGAGGTGC
SRR944633.22731044.1+    GGCATCTGGATTCAGCCCTCATGGAGAGCTGCCTGGATATAACATTGTGACAGGAGGNGC
SRR944633.5304783.1-     GGCATCTGGATTCAGCCCTCATGGAGAGCTGCCTGGATATAACATTGTGACAGGAGGTGC
SRR944633.55088954.1+    GGCATCTGGATTCAGCCCTCATGGAGAGCTGCCTGGATATAACATTGTGACAGGAGGGGC
SRR944633.58893957.1-    GGCATCTGGATTCAGCCCTCATGGAGAGCTGCCTGGATATAACATTGTGACAGGAGGTGC
SRR944633.66408716.1-    GGCATATGGATTCAGCCCTCATGGAGAGCTGCCTGGATATAACATTGTGACAGGAGGTGC
SRR944633.70260610.2-    GGCATCTGGATTCAGCCCTCATGGAGAGCTGCCTGGATATAACATTGTGACAGGAGGTGC
SRR944634.27747891.2-    GGCATCTGGATTCAGCCCTCATGGAGAGCTGCCTGGATATAACATTGTGACAGGAGGTGC
SRR944634.36751401.2+    GGCATCTGGATTCAGCCCTCATGGAGAGCTGCCTGGATATAACATTGTGACAGGCGGGGC
SRR944634.56993620.2-    GGCATCTGGATTCAGCCCTCATGGAGAGCTGCCTGGATATAACATTGTGACAGGAGGTGC
SRR944634.62072555.1+    GGCATCTGGATTCAGCCCTCATGGAGAGCTGCCTGGATATAACATTGTGACAGGAGGTGC
SRR944634.90107683.1-    GGCATCTGGATTCAGCCCTCATGGAGAGCTGCCTGGATATAACATTGTGACAGGAGGTGC
SRR944635.25751445.2-    GGCATCTGGATTCAGCCCTCATGGAGAGCTGCCTGGATATAACATTGTGACAGGAGGTGC
SRR944635.26842443.1-    GGCATCTGGATTCAGCCCTCATGGAGAGCTGCCTGGATATAACATTGTGACAGGAGGTGC
SRR944635.48191009.2-    GGCATCTGGATTCAGCCCTCATGGAGAGCTGCCTGGATATAACATTGTGACAGGAGGTGC
SRR944635.48675781.2-    GGCATCTGGATTCAGCCCTCATGGAGAGCTGCCTGGATATAACATTGTGACAGGAGGTGC
SRR944635.67387987.2+    GGCATCTGGATTCAGCCCTCATGGAGAGCTGCCTGGATATAACATTGTGACAGGAGGTGC
SRR944635.81516722.2-    GGCATCTGGATTCAGCCCTCATGGAGAGCTGCCTGGATATAACATTGTGACAGGAGGTGC
SRR944637.18612130.2+    GGCATCTGGATTCAGCCCTCATGGAGAGCTGCCTGGATATAACATTGTGACAGGGGGTGC
SRR944637.3204364.1-     GGCATCTGGATTCAGCCCTCATGGAGAGCTGCCTGGATATAACATTGTGACAGGAGGTGC
SRR944637.41203321.1+    GGCATCTGGATTCAGCCCTCATGGAGAGCTGCCTGGATATAACATTGTGACAGGAGGTGC
SRR944637.48990847.2-    GGCATCTGGATTCAGCCCTCATGGAGAGCTGCCTGGATATAACATTGTGACAGGAGGTGC
SRR944637.570564.1+        CATCTGGATTCAGCCCTCATGGAGAGCTGCCTGGATATAACATTGTGACAGGAGGTGC
SRR944636.23172184.1+       ATCTGGATTCAGCCCTCATGGAGAGCTGCCTGGATATAACATTGTGACA
SRR944633.26132114.2-          TGGATTCAGCCCTCATGGAGAGCTGCCTGGATATAACATTGTGACAGGAGGTGC
SRR944635.24423685.1-          TGGATTCAGCCCTCATGGAGAGCTGCCTGGATATAACATTGTGACAGGAGGTGC
SRR944635.13764978.1-            GATTCAGCCCTCATGGAGAGCTGCCTGGATATAACATTGTGACAGGAGGTGC
SRR944635.82600026.1-            GATTCAGCCCTCATGGAGAGCTGCCTGGATATAACATTGTGACAGGAGGTGC
SRR944635.10104664.2-             ATTCAGCCCTCATGGAGAGCTGCCTGGATATAACATTGTGACAGGAGGTGC
SRR944637.66277957.1+              TTCAGCCCTCATGGAGAGCTGCCTGGATATAACATTGTGACAGGAGGTGC
SRR944636.31854196.2+               TCAGCCCTCATGGAGAGCTGCCTGGATATAACATTGTGACAGGAGGTGC
SRR944634.83589188.1+                CAGCCCTCATGGAGATCTGCCTGGATATAACATTGTGACAGGAGGTGC
SRR944634.59210810.1+                 AGCCCTCATGGAGAGCTGCCTGGATATAACATTGTGACAGGAGGTGC
SRR944639.35025396.2+                  GCCCTCATGGAGAGCTGCCTGGATATAACATTGTGACAGGAGGTGC
SRR944639.88519028.2+                  GCCCTCATGGAGAGCTGCCTGGATATAACATTGTGACAGGAGGTGC
SRR944638.48431457.1-                   CCCTCATGGAGAGCTGCCTGGATATAACATTGTGACAGGAGGTGC
SRR944638.64645947.1-                   CCCTCATGGAGAGCTGCCTGGATATAACATTGTGACAGGAGGTGC
SRR944638.80128535.1-                   CCCTCATGGAGAGCTGCCTGGATATAACATTGTGACAGGAGGTGC
SRR944637.4770778.1+                     CCTCATGGAGAGCTGCCTGGATATAACATTGTGACAGGAGGTGC
SRR944639.20592150.1+                       CATGGAGAGCTGCCTGGATATAACATTGTGACAGGAGGTGC
SRR944633.91746479.1-                        ATGGAGAGCTGCCTGGATATAACATTGTGACAGGAGGTGC
SRR944635.6485714.1-                          TGGAGAGCTGCCTGGATATAACATTGTGACAGGAGGTGC
SRR944635.44560005.1-                          GGAGAGCTGCCTGGATATAACATTGTGACAGGAGGTGC
SRR944637.64626416.2-                              AGCTGCCTGGATATAACATTGTGACAGGAGGTGC
```

```
SRR944633.72364928.1-                               GCTGCCTGGATATAACATTGTGAGAGGAGGTGC
SRR944634.60100148.1-                               GCTGCCTGGATATAACATTGTGACAGGAGGTGC
SRR944634.8289892.2-                                GCTGCCTGGATATAACATTGTGACAGGAGGTGC
SRR944638.76380308.1-                               GCTGCCTGGATATAACATTGTGACAGGAGGTGC
SRR944635.14783008.2-                                CTGCCTGGATATAACATTGTGACAGGAGGTGC
SRR944635.5046535.1-                                     CTGGATATAACATTGTGACAGGAGGTGC
SRR944630.46910315.2-                                     TGGATATAACATTGTGACAGGAGGTGC
SRR944630.56113758.2-                                     TGGATATAACATTGTGACAGGAGGTGC
SRR944639.4474450.1-                                      TGGATATAACATTGTGACAGGAGGTGC
SRR944635.73418382.1-                                      GGATATAACATTGTGACAGGAGGTGC
SRR944639.34202714.2+                                      GGATATAACATTGTGACAGGAGGTGC
SRR944636.2781029.1-                                        GATATAACATTGTGACAGGAGGTGC
SRR944638.39279198.2+                                         TATAACATTGTGACAGGAGGTGC
SRR944638.43657872.2+                                         TATAACATTGTGACAGGAGGTGC
SRR944632.26472867.1-                                          ATAACATTGTGACAGGAGGTGC
SRR944633.777688.1-                                            ATAACATTTTGACAGGAGGTGC
SRR944637.12320160.1-                                          ATAACATTGTGACAGGAGGTGC
SRR944639.11134324.2-                                           TAACATTGTGACAGGAGGTGC
SRR944639.26937907.2-                                           TAACATTGTGACAGGAGGTGC
SRR944639.29003334.2+                                            AACATTGTGACAGGAGGTGC
SRR944638.59244387.1+                                             ACATTGTGACAGGAGGTGC
SRR944639.56854205.2+                                             ACATTGTGACAGGAGGTGC
SRR944639.76938662.1+                                             ACATTGTGACAGGAGGTGC
SRR944633.55088954.2-                                               ATTGTGACAGGAGGTGC
SRR944634.36751401.1-                                                  GTGACAGGAGGTTC
SRR944638.71262487.1-                                                  GTGACAGGAGGTGC
SRR944637.8913243.2+                                                    TGACAGGAGGTGC
SRR944635.30293163.1+                                                    GACAGGAGGTGC
SRR944630.28885318.1-                                                     ACAGGAGGTGC
SRR944632.4798276.2+                                                        AGGAGGTGC
SRR944635.19092406.2+                                                       AGGAGGTGC
SRR944630.102667012.1+                                                       GGAGGTGC
SRR944630.2203921.2-                                                            GGTGC
SRR944637.40151612.2+                                                            GTGC
SRR944634.51557407.2+                                                               C

consensus                GGCATCTGGATTCAGCCCTCATGGAGAGCTGCCTGGATATAACATTGTGACAGGAGGTGC

                             .    :    .    :    .    :    .    :    .    :    .    :
SRR944634.27747891.2-    CTC
SRR944639.35025396.2+    CTC
SRR944639.88519028.2+    CTC
SRR944635.81516722.2-    CTCC
SRR944638.48431457.1-    CTCC
SRR944638.64645947.1-    CTCC
SRR944638.80128535.1-    CTCC
SRR944635.48675781.2-    CTCCCC
SRR944637.18612130.2+    CTCCCC
SRR944639.20592150.1+    CTCCCCAA
SRR944637.48990847.2-    CTCCCCAAAT
SRR944635.25751445.2-    CTCCCCAAATGG
SRR944634.36751401.2+    CTCCCCAAATGGCAG
SRR944638.76380308.1-    CTCCCCAAATGGCAGC
SRR944633.55088954.1+    CTCCCCCAATGGCAGCAT
SRR944633.22731044.1+    CTCCCCAAATGGCAGCATCT
SRR944635.48191009.2-    CTCCCCAAATGGCAGCATCT
SRR944630.46910315.2-    CTCCCCAAATGGCAGCATCTGA
SRR944630.56113758.2-    CTCCCCAAATGGCAGCATCTGA
SRR944639.4474450.1-     CTCCCCAAATGGCAGCATCTGA
SRR944639.34202714.2+    CTCCCCAAATGGCAGCATCTGAT
SRR944636.2781029.1-     CTCCCCAAATGGCAGCATCTGATT
SRR944633.58893957.1-    CTCCCCAAATGGCAGCATCTGATTC
SRR944638.39279198.2+    CTCCCCAAATGGCAGCATCTGATTCT
SRR944638.43657872.2+    CTCCCCAAATGGCAGCATCTGATTCT
SRR944632.26472867.1-    CTCCCCAAATGGCAGCATCTGATTCTC
SRR944633.5304783.1-     CTCCCCAAATGGCAGCATCTGATTCTCT
SRR944639.11134324.2-    CTCCCCAAATGGCAGCATCTGATTCTCT
SRR944639.26937907.2-    CTCCCCAAATGGCAGCATCTGATTCTCT
SRR944639.29003334.2+    CTCCCCAAATGGCAGCATCTGATTCTCT
SRR944634.90107683.1-    CTCCCCAAATGGCAGCATCTGATTCTCTg
SRR944638.59244387.1+    CTCCCCAAATGGCAGCATCTGATTCTCTgt
SRR944639.56854205.2+    CTCCCCAAATGGCAGCATCTGATTCTCTgt
SRR944639.76938662.1+    CTCCCCAAATGGCAGCATCTGATTCTCTgt
SRR944634.62072555.1+    CTCCCCAAATGGCAGCATCTGATTCTCTgta
SRR944637.41203321.1+    CTCCCCAAATGGCAGGATCTGATTCTCTgtaagt
SRR944638.71262487.1-    CTCCCCAAATGGCAGCATCTGATTCTCTgtaagtt
SRR944637.66277957.1+    CTCCCCAAATGGCAGCATCTGATTCTCTgtaagttg
SRR944633.100415876.1-   CTCCCCAAATGGCAGCATCTGATTCTCTgtaagttga
SRR944630.28885318.1-    CTCCCCAAATGGCAGCATCTGATTCTCTgtaagttgaa
SRR944633.70260610.2-    CTCCCCAAATGGCAGCATCTGATTCTCTgtaagttgaa
SRR944633.66408716.1-    CTCCCCAAATGGCAGCATCTGATTCTCTgtaagttgaac
SRR944632.4798276.2+     CTCCCCAAATGGCAGCATCTGATTCTCTgtaagttgaaca
SRR944633.12567580.1+    CTCCCCAAATGGCAGCATCTGATTCTCTgtaagttgaaca
SRR944637.3204364.1-     CTCCCCAAATGGCAGCATCTGATTCTCTgtaagttgaaca
```

```
SRR944630.102667012.1+   CTCCCCAAATGGCAGCATCTGATTCTCTgtaagttgaacag
SRR944637.570564.1+      CTCCCCAAATGGCAGCATCTGCTTCTCTgtaagttgaacaga
SRR944630.2203921.2-     CTCCCCAAATGGCAGCATCTGATTCTCTgtaagttgaacagatt
SRR944633.26132114.2-    CTCCCCAAATGGCAGCATCTGATTCTCTgtaagttgaacagatttc
SRR944635.24423685.1-    CTCCCCAAATGGCAGCATCTGATTCTCTgtaagttgaacagatttc
SRR944635.13764978.1-    CTCCCCAAATGGCAGCATCTGATTCTCTgtaagttgaacagatttcct
SRR944635.82600026.1-    CTCCCCAAATGGCAGCATCTGATTCTCTgtaagttgaacagatttcct
SRR944635.10104664.2-    CTCCCCAAATGGCAGCATCTGATTCTCTgtaagttgaacagatttcctc
SRR944634.83589188.1+    CTCCCCAAATGGCAGCATCTGATTCTCTgtaagttgaacagatttcctcttc
SRR944634.59210810.1+    CTCCCCAAATGGCAGCATCTGATTCTCTgtaagttgaacagatttcctcttca
SRR944637.4770778.1+     CTCCCCAAATGGCAGCATCTGATTCTCTgtaagttgaacagatttcctcttcatcc
SRR944637.64626416.2-    CTCCCCAAATGGCAGCATCTGATTCTCTgtaagttgaacagatttcctcttcatcccc
SRR944633.55088954.2-    CTCCCCAAATGGCAGCATCTGATTCTCTgtaagttgaacagatttcctcttcatccccct
SRR944633.72364928.1-    CTCCCCAAATGGCAGCATCTGATTCTCTgtaagttgaacagatttcctcttcatccccct
SRR944633.777688.1-      CTCCCCAAATGGCAGCATCTGATTCTCTgtaagtttaacagatttcctcttcatccccct
SRR944633.91746479.1-    CTCCCCAAATGGCAGCATCTGATTCTCTgtaagttgaacagatttcctcttcatccccct
SRR944634.36751401.1-    CTCCCCAAATGGCAGCATCTGATTCTCTgtaagttgaacagatttcctcttcatccccct
SRR944634.51557407.2+    CTCCCCAAATGGCAGCATCTGATTCTCTgtaagttgaacagatttcctcttcatccccct
SRR944634.60100148.1-    CTCCCCAAATGGCAGCATCTGATTCTCTgtaagttgaacagatttcctcttcatccccct
SRR944634.8289892.2-     CTCCCCAAATGGCAGCATCTGATTCTCTgtaagttgaacagatttcctcttcatccccct
SRR944635.14783008.2-    CTCCCCAAATGGCAGCATCTGATTCTCTgtaagttgaacagatttcctcttcatccccct
SRR944635.19092406.2+    CTCCCCAAATGGCAGAATCTGGTTCTCTgtaagttgaacagatttcctcttcatccccct
SRR944635.30291594.1+    CTCCCCAAATGGCAGCATCTGATTCTCTgtaagttgaacagatttcctcttcatccccct
SRR944635.30293163.1+    CTCCCCAAATGGCAGCATCTGATTCTCTgtaagttgaacagatttcctcttcatccccct
SRR944635.44560005.1-    CTCCCCAAATGGCAGCATCTGATTCTCTgtaagttgaacagatttcctcttcatccccct
SRR944635.5046535.1-     CTCCCCAAATGGCAGCATCTGATTCTCTgtaagttgaacagatttcctcttcatccccct
SRR944635.6485714.1-     CTCCCCAAATGGCAGCATCTGATTCTCTgtaagttgaacagatttcctcttcatccccct
SRR944635.73418382.1-    CTCCCCAAATGGCAGCATCTGATTCTCTgtaagttgaacagatttcctcttcatccccct
SRR944637.12320160.1-    CTCCCCAAATGGCAGCATCTGATTCTCTgtaagttgaacagatttcctcttcatccccct
SRR944637.40151612.2+    CTCCCCAAATGGCAGCATCTGATTCTCTgtaagttgaacagatttcctcttcatccccct
SRR944637.8913243.2+     CTCCCCAAATGGCAGCATCTGATTCTCTgtaagttgaacagatttcctcttcatcccccc
SRR944636.71590752.2-     TCCCCAAATGGCAGCATCTGATTCTCTgtaagttgaacagatttcc
SRR944635.33395560.1-      CCCCAAAGGGGAGCCTCTGATTCTCTgtaagttgaacagatttcctcttcatccccct
SRR944634.1024513.1-        CCCAAATGGCAGCATCTGATTCTCTgtaagttgaacagatttcctcttcatccccct
SRR944637.50501063.1+       CCCAAATGGCAGCATCTGATTCTCTgtaagttgaacagatttcctcttcatccccct
SRR944633.22731044.2-         CAAATGGCAGCATCTGATTCTCTgtaagttgaacagatttcctcttcatccccct
SRR944633.9318354.1+           AAATGGCAGCATCTGATTCTCTgtaagttgaacagatttcctcttcatccccct
SRR944634.62072555.2-          AAATGGCAGCATCTGATTCTCTgtaagttgaacagatttcctcttcatccccct
SRR944635.62658282.2-            ATGGCAGCTGCTGATTCTCTgtaagttgaacagatttcctcttcacccccct
SRR944637.66942762.1-            ATGGCAGCATCTGATTCTCTgtaagttgaacagatttcctcttcatccccct
SRR944630.56258503.1+             TGGCAGCATCTGATTCTCTgtaagttgaacagatttcctcttcatcccc
SRR944633.12567580.2-             TGGCAGCATCTGATTCTCTgtaagttgaacagatttcctcttcatccccct
SRR944635.5861948.1-              TGGCAGCATCTGATTCTCTgtaagttgaacagatttcctcttcatccccct
SRR944639.41083331.2+              GGCAGCATCTGATTCTCTgtaagttgaacagatttcctcttcatccccc
SRR944637.4900912.2-               GGCAGCATCTGATTCTCTgtaagttgaacagatttcctcttcatccccct
SRR944634.92106320.2-                CAGCATCTGATTCTCTgtaagttgaacagatttcctcttcatccccct
SRR944637.31551384.1-                CAGCCTCTGATTCTCTgtaagttgaacagatttcctcttcatccccct
SRR944639.81939970.2-                CAGCATCTGATTCTCTgtaagttgaacagatttcctcttcatccccct
SRR944636.70931640.2-                 AGCATCTGATTCTCTgtaagttgaacagatttcctcttcatccccct
SRR944634.64662410.2+                  GCATCTGATTCTCTgtaagttgaacagatttcctcttcatccccct
SRR944635.2449031.1+                     ATCTGATTCTCTgtaagttgaacagatttcctcttcatccccct
SRR944635.31533306.1-                    ATCTGATTCTCTgtaagttgaacagatttcctcttcatccccct
SRR944637.52689694.2+                    ATCTGATTCTCTgtaagttgaacagatttcctcttcatccccct
SRR944637.55178119.2+                    ATCTGATTCTCTgtaagttgaacagatttcctcttcatccccct
SRR944637.57331907.1+                    ATCTGATTCTCTgtaagttgaacagatttcctcttcatccccct
SRR944635.8222046.2-                      TCTGATTCTCTgtaagttgaacagatttcctcttcatccccct
SRR944633.102269773.1+                     CTGATTCTCTgtaagttgaacagatttcctcttcatccccct
SRR944634.59210810.2-                      CTGATTCTCTgtaagttgaacagatttcctcttcatccccct
SRR944633.61494335.1+                       TGATTCTCTgtaagttgaacagatttcctcttcatccccct
SRR944633.9318354.2-                        TGATTCTCTgtaagttgaacagatttcctcttcatccccct
SRR944634.46413615.1+                       TGATTCTCTgtaagttgaacagatttcctcttcatccccct
SRR944636.14213886.1+                       TGATTCTCTgtaagttgaacagatttcctcttcatccccct
SRR944637.70490964.1-                       TGATTCTCTgtaagttgaacagatttcctcttcatccccct
SRR944635.57447282.2+                        GATTCTCTgtaagttgaacagatttcctcttcatccccct
SRR944634.83589188.2-                         ATTCTCTgtaagttgaacagatttcctcttcatccccct
SRR944635.6074553.2-                          ATTCTCTgtaagttgaacagatttcctcttcatccccct
SRR944636.20771813.1-                         ATTCTCTgtaagttgaacagatttcctcttcatccccct
SRR944636.66207103.1+                         ATTCTCTgtaagttgaacagatttcctcttcatccccct
SRR944637.64082347.2-                         ATTCTTTgtaagttgaacagatttcctcttcatccccct
SRR944633.21943774.1+                          TTCTCTgtaagttgaacagatttcctcttcatccccct
SRR944631.16715231.2+                           TCTCTgtaagttgaacagatttcctcttcatccccct
SRR944632.123407711.2-                              Tgtaagttgaacagatttcctcttcatccccct
SRR944635.60156403.2-                               Tgtaagttgaacagatttcctcttcatccccct
                         ------------------------------------------------------------
consensus                CTCCCCAAATGGCAGCATCTGATTCTCTgtaagttgaacagatttcctcttcatccccct
```

C. Hooded crow (No. 4), exon 1, WGS data

- CAP3 alignment of WGS data spanning exon 1 (uppercase letters)

```
                             .    :    .    :    .    :    .    :    .    :    .    :
SRR1271606.18225242.2-   tgcccgaggtgtcagcaagactcagaaactgcagtgA
SRR1265418.3326891.1+    tgcccgaggtgtcagcaacactcagaaactgcagtgAT
SRR1265429.4576456.1-    tgcccgaggtgtcagcaagactcagaaactgcagtgAT
SRR1271631.39804293.2-   tgcccgaggtgtcagcaagactcagaaactgcagtgAT
SRR1271641.7006039.1-    tgcccgaggtgtcagcaagactcagaaactgcagtgAT
SRR1265486.2988089.1+    tgcccgaggtgtcagcaagactcagaaactgcagtgATG
SRR1266957.4360269.2-    tgcccgaggtgtcagcaagactcagaaactgcagtgATG
SRR1265421.11833894.2-   tgcccgaggtgtcagcaagactcagaaactgcagtgATGAT
SRR1265108.1960595.2-    tgcccgaggtgtcagcaagactcagaaactgcagtgATGATG
SRR1265425.6191113.2-    tgcccgaggtgtcagcaagactcagaaactgcagtgATGATG
SRR1265487.14960037.2-   tgcccgaggtgtcagcaagactcagaaactgcagtgATGATG
SRR1266947.19667874.2-   tgcccgaggtgtcagcaagacacagaaactgcagtgATGATG
SRR1271617.43257682.1+   tgcccgaggtgtcagcaagactcagaaactgcagtgATGATG
SRR1271634.1326286.2-    tgcccgaggtgtcagcaagactcagaaactgcagtgATGATG
SRR1271662.5325320.2-    tgcctgaggtgtcagcaagactcagaaactgcagtgATGATG
SRR1269720.8521946.2+    tgcccgaggtgtcagcaagacacagaaactgcagtgATGATGG
SRR1271628.1578656.1+    tgcccgaggtgtcagcaagactcagaaactgcagtgATGATGG
SRR1271664.3024191.2-    tgcccgaggtgtcagcaagactcagaaactgcagtgATGATGG
SRR1271617.67611451.2-   tgcccgaggtgtcagcaagactcagaaactgcagtgATGATGGT
SRR1271641.2170364.2-    tgcccgaggtgtcagcaagacacagaaactgcagtgATGATGGT
SRR1271656.41698454.2-   tgcccgaggtgtcagcaagacacagaaactgcagtgATGATGGT
SRR1265494.6720823.1-    tgcccgaggtgtcagcaagactcagaaactgcagtgATGATGGTG
SRR1271614.4869115.2-    tgcccgaggtgtcagcaagactcagaaactgcagtgATGATGGTG
SRR1269721.297214.1-     tgcccgaggtgtcagcaagacacagaaactgcagtgATGATGGTGA
SRR1271617.70351078.1+   tgcccgaggtgtcagcaagactcagaaactgcagtgATGATGGTGA
SRR1271625.4440646.2+    tgcccgaggtgtcagcaagactcagaaactgcagtgATGATGGTGA
SRR1265093.3579230.2-    tgcccgaggtgtcagcaagactcagaaactgcagtgATGATGGTGAT
SRR1265425.9555102.1-    tgcccgaggtgtcagcaagactcagaaactgcagtgATGATGGTGAT
SRR1271631.3124822.1-    tgcccgaggtgtcagcaagactcagaaactgcagtgATGATGGTGAT
SRR1271631.31626497.1-   tgcccgaggtgtcagcaagactcagaaactgcagtgATGATGGTGAT
SRR1271631.95991717.2-   tgcccgaggtgtcagcaagactcagaaactgcagtgATGATGGTGAT
SRR1271660.10781231.2-   tgcccgaggtgtcagcaagactcagaaactgcagtgATGATGGTGAT
SRR1265116.4878633.1+    tgcccgaggtgtcagcaagactcagaaactgcagtgATGATGGTGATG
SRR1269723.13204824.1-   tgcccgaggtgtcagcaagacacagaaactgcagtgATGATGGTGATG
SRR1271624.11221728.2+   tgcccgaggtgtcagcaagactcagaaactgcagtgATGATGGTGATG
SRR1271631.88026372.2-   tgcccgaggtgtcagcaagactcagaaactgcagtgATGATGGTGATG
SRR1271631.88107554.1+   tgcccgaggtgtcagcaagactcagaaactgcagtgATGATGGTGATG
SRR1271642.3577506.1+    tgcccgaggtgtcagcaagactcagaaactgcagtgATGATGGTGATG
SRR1265101.7450306.1+    tgcccgaggtgtcagcaagactcagaaactgcagtgATGATGGTGATGC
SRR1265102.8168674.2-    tgcccgaggtgtcagcaagactcagaaactgcagtgATGATGGTGATGC
SRR1271617.37836118.1-   tgcccgaggtgtcagcaagactcagaaactgcagtgATGATGGTGATGC
SRR1271642.3018005.1+    tgcccgaggtgtcagcaagactcagaaactgcagtgATGATGGTGATGC
SRR1265425.3746284.2-    tgcccgaggtgtcagcaagactcagaaactgcagtgATGATGGTGATGCT
SRR1265486.14250218.1-   tgcccgaggtgtcagcaagactcagaaactgcagtgATGATGGTGATGCT
SRR1265960.19147369.2-   tgcccgaggtgtcagcaagactcagaaactgcagtgATGATGGTGATGCT
SRR1269721.4085403.2+    tgcccgaggtgtcagcaagacacagaaactgcagtgATGATGGTGATGCT
SRR1271605.9271138.2-    tgcccgaggtgtcagcaagactcagaaactgcagtgATGATGGTGATGCT
SRR1271660.204492.1+     tgcccgaggtgtcagcaagactcagaaactgcagtgATGATGGAGATGCT
SRR1265423.1464723.1+    tgcccgaggtgtcagcaagactcagaaactgcagtgATGATGGTGATGCTCT
SRR1266947.23270046.1+   tgcccgaggtgtcagcaagacacagaaactgcagtgATGATGGTGATGCTCT
SRR1271628.2167064.2-    tgcccgaggtgtcagcaagactcagaaactgcagtgATGATGGTGATGCTCT
SRR1271631.31329934.2-   tgcccgaggtgtcagcaagactcagaaactgcagtgATGATGGTGATGCTCT
SRR1271637.3241382.2+    tgcccgaggtgtcagcaagactcagaaactgcagtgATGATGGGGATGCTCT
SRR1265961.10160280.2-   tgcccgaggtgtcagcaagactcagaaactgcagtgATGATGGTGATGCTCTT
SRR1266947.12369096.2-   tgcccgaggtgtcagcaagacacagaaactgcagtgATGATGGTGATGCTCTT
SRR1266956.668321.1-     tgcccgaggtgtcagcaagacacagaaactgcagtgATGATGGTGATGCTCTT
SRR1266962.10827261.1-   tgcccgaggtgtcagcaagacacagaaactgcagtgATGATGGTGATGCTCTT
SRR1271642.249531.1+     tgcccgaggtgtcagcaagactcagaaactgcagtgATGATGGTGATGCTCTT
SRR1271665.3197988.1+    tgcccgaggtgtcagcaagactcagaaactgcagtgATGATGGTGATGCTCTT
SRR1265107.4659856.2-    tgcccgaggtgtcagcaagactcagaaactgcagtgATGATGGTGATGCTCTTC
SRR1269727.11297167.1-   tgcccgaggtgtcagcaagactcagaaactgcagtgATGATGGTGATGCTCTTC
SRR1271626.39747880.1+   tgcccgaggtgtcagcaagactcagaaactgcagtgATGATGGTGATGCTCTTC
SRR1266956.2653211.1+    tgcccgaggtgtcagcaagactcagaaactgcagtgATGATGGTGATGCTCTTCT
SRR1269690.2995201.1-    tgcccgaggtgtcagcaagactcagaaactgcagtgATGATGGTGATGCTCTTCT
SRR1269699.1770369.1-    tgcccgaggtgtcagcaagacacagaaactgcagtgATGATGGTGATGCTCTTCT
SRR1271633.1518080.1-    tgcccgaggtgtcagcaagactcagaaactgcagtgATGATGGTGATGCTCTTCT
SRR1271656.16279216.1-   tgcccgaggtgtcagcaagactcagaaactgcagtgATGATGGTGATGCTCTTCT
SRR1266948.11632586.1-   tgcccgaggtgtcagcaagactcagaaactgcagtgATGATGGTGATGCTCTTCTC
SRR1269695.4227406.1-    tgcccgaggtgtcagcaagactcagaaactgcagtgATGATGGTGATGCTCTTCTC
SRR1271631.75576578.2+   tgcccgaggtgtcagcaagactcagaaactgcagtgATGATGGTGATGCTCTTCTC
SRR1265102.6323108.1-    tgcccgaggtgtcagcaagactcagaaactgcagtgATGATGGTGATGCTCTTCTCA
SRR1265421.26066836.2-   tgcccgaggtgtcagcaaggctcagaaactgcagtgATGATGGTGATGCTCTTCTCA
SRR1271611.53197193.1-   tgcccgaggtgtcagcaagactcagaaactgcagtgATGATGGTGATGCTCTTCTCA
SRR1271656.31699611.1+   tgcccgaggtgtcagcaagactcagaaactgcagtgATGATGGTGATGCTCTTCTCA
SRR1271661.4142288.1-    tgcctgaggtgtcagcaagactcagaaactgcagtgATGATGGTGATGCTCTTCTCA
```

SRR1271620.12588857.1- tgcccgaggtgtcagcaagactcagaaactgcagtgATGATGGTGATGCTCTTCTCAG
SRR1271626.10299387.1+ tgcccgaggtgtcagcaagactcagaaactgcagtgATGATGGTGATGCTCTTCTCAG
SRR1271631.77415545.2- tgcccgaggtgtcagcaagactcagaaactgcagtgATGATGGTGATGCTCTTCTCAG
SRR1265094.790368.2+ tgcccgaggtgtcagcaagactcagaaactgcagtgATGATGGTGATGCTCTTCTCAGG
SRR1265492.5585323.1+ tgcccgaggtgtcagcaagactcagaaactgcagtgATGATGGTGATGCTCTTCTCAGG
SRR1265959.4685497.2+ tgcccgaggtgtcagcaagactcagaaactgcagtgATGATGGTGATGCTCTTCTCAGG
SRR1269696.5605733.1+ tgcccgaggtgtcagcaagactcagaaactgcagtgATGATGGTGATGCTCTTCTCAGG
SRR1271617.28309534.1- tgcccgaggtgtcagcaagactcagaaactgcagtgATGATGGTGATGCTCTTCTCAGG
SRR1271636.12517782.2+ tgcccgaggtgtcagcaagactcagaaactgcagtgATGATGGTGATGCTCTTCTCAGG
SRR1265091.7293804.2+ tgcccgaggtgtcagcaagactcagaaactgcagtgATGATGGTGATGCTCTTCTCAGGG
SRR1265092.8008049.2- tgcccgaggtgtcagcaagactcagaaactgcagtgATGATGGTGATGCTCTTCTCAGGG
SRR1265094.1737834.1- tgcccgaggtgtcagcaagactcagaaactgcagtgATGATGGTGATGCTCTTCTCAGGG
SRR1265100.7029791.1+ tgcccgaggtgtcagcaagactcagaaactgcagtgATGATGGTGATGCTCTTCTCAGGG
SRR1265103.985070.1+ tgcccgaggtgtcagcaagactcagaaactgcagtgATGATGGCGATGCTCTTCTCAGGG
SRR1265107.1205589.1+ tgcccgaggtgtcagcaagactcagaaactgcagtgATGATGGTGATGCTCTTCTCAGGG
SRR1265107.5946926.1+ tgcccgaggtgtcagcaagactcagaaactgcagtgATGATGGTGATGCTCTTCTCAGGG
SRR1265108.3192987.1- tgcccgaggtgtcagcaagactcagaaactgcagtgATGATGGTGATGCTCTTCTCAGGG
SRR1265108.9817332.2- tgcccgaggtgtcagcaaggctcagaacctgcagtgATGATGGTGATGCTCTTCTCAGGG
SRR1265116.9489829.1- tgcccgaggtgtcagcaagactcagaaactgcagtgATGATGGTGATGCTCTTCTCAGGG
SRR1265117.5894445.1- tgcccgaggtgtcagcaagactcagaaactgcagtgATGATGGTGATGCTCTTCTCAGGG
SRR1265117.8111828.1+ tgcccgaggtgtcagcaagactcagaaactgcagtgATGATGGTGATGCTCTTCTCAGGG
SRR1265120.12719577.1+ tgcccgaggtgtcagcaagactcagaaactgcagtgATGATGGTGATGCTCTTCTCAGGG
SRR1265419.5834061.1+ tgcccgaggtgtcagcaagactcagaaactgcagtgATGATGGTGATGCTCTTCTCAGGG
SRR1265419.9630529.2+ tgcccgaggtgtcagcaagactcagaaactgcagtgATGATGGTGATGCTCTTCTCAGGG
SRR1265419.9760671.2+ tgcccgaggtgtcagcaagactcagaaactgcagtgATGATGGTGATGCTCTTCTCAGGG
SRR1265420.8209771.1+ tgcccgaggtgtcagcaagactcagaaactgcagtgATGATGGGGATGCTCTTCTCAGGG
SRR1265420.8458569.1+ tgcccgaggtgtcagcaagactcagaaactgcagtgATGATGGTGATGCTCTTCTCAGGG
SRR1265420.9671347.2- tgcccgaggtgtcagcaagactcagaaactgcagtgATGATGGTGATGCTCTTCTCAGGG
SRR1265421.22002067.1+ tgcccgaggtgtcagcaagactcagaaactgcagtgATGATGGTGATGCTCTTCTCAGGG
SRR1265422.16304480.2+ tgcccgaggcgtcagcaagactcagaaactgcagtgATGATTGTGACGCTCTTCTCAGGG
SRR1265423.8357279.2- tgcccgaggtgtcagcaagactcagaaactgcagtgATGATGGTGATGCTCTTCTCAGGG
SRR1265425.3715610.1- tgcccgaggtgtcagcaagactcagaaactgcagtgATGATGGTGATGCTCTTCTCAGGG
SRR1265427.10252303.2+ tgcccgaggtgtcagcaagactcagaaactgcagtgATGATGGTGATGCTCTTCTCAGGG
SRR1265427.143510.1- tgcccgaggtgtcagcaagactcagaaactgcagtgATGATGGTGATGCTCTTCTCAGGG
SRR1265429.13415617.2+ tgcccgaggtgtcagcaagactcagaaactgcagtgATGATGGTGATGCTCTTCTCAGGG
SRR1265429.1518485.1+ tgcccgaggtgtcagcaagactcagaaactgcagtgATGATGGTGATGCTCTTCTCAGGG
SRR1265485.7131594.1- tgcccgaggtgtcagcaagactcagaaactgcagtgATGATGGTGATGCTCTTCTCAGGG
SRR1265486.14296408.1- tgcccgaggtgtcagcaagactcagaaactgcagtgATGATGGTGATGCTCTTCTCAGGG
SRR1265486.16127387.1- tgcccgaggtgtcagcaagactcagaaactgcagtgATGATGGTGATGCTCTTCTCAGGG
SRR1265486.4188250.2- tgcccgaggtgtcagcaagactcagaaactgcagtgATGATGGTGATGCTCTTCTCAGGG
SRR1265486.5317212.2- tgcccgaggtgtcagcaagactcagaaactgcagtgATGATGGTGATGCTCTTCTCAGGG
SRR1265487.3611936.1+ tgcccgaggtgtcagcaagactcagaaactgcagtgATGATGGTGATGATCTTCTCAGGG
SRR1265490.6979773.2- tgcccgaggtgtcagcaagactcagaaactgcagtgATGATGGTGATGCTCTTCTCAGGG
SRR1265492.1503780.2- tgcccgaggtgtcagcaagactcagaaactgcagtgATGATGGTGATGCTCTTCTCAGGG
SRR1265492.2995376.1- tgcccgaggtgtcagcaagactcagaaactgcagtgATGATGGTGATGCTCTTCTCAGGG
SRR1265492.6404320.2+ tgcccgaggtgtcagcaagactcagaaactgcagtgATGATGGTGATGCTCTTCTCAGGG
SRR1265959.10232564.2- tgcccgaggtgtcagcaagactcagaaactgcagtgATGATGGTGATGCTCTTCTCAGGG
SRR1265961.17164072.2+ tgcccgaggtgtcagcaagactcagaaactgcagtgATGATGGTGATGCTCTTCTCAGGG
SRR1265961.20492731.2- tgcccgaggtgtcagcaagactcagaaactgcagtgATGATGGTGATGCTCTTCTCAGGG
SRR1265961.5907868.2- tgcccgaggtgtcagcaagactcagaaactgcagtgATGATGGTGATGCTCTTCTCAGGG
SRR1265962.10464106.1+ tgcccgaggtgtcagcaagactcagaaactgcagtgATGATGGTGATGCTCTTCTCAGGG
SRR1265962.6217934.1+ tgcccgaggggtcagcaagactcagaaactgcagcgATGATGGTGATGCTCTTCTCAGGG
SRR1266947.12676414.2- tgcccgaggtgtcagcaagactcagaaactgcagtgATGATGGTGATGCTCTTCTCAGGG
SRR1266949.3696068.1- tgcccgaggtgtcagcaagacacagaaactgcagtgATGATGGTGATGCTCTTCTCAGGG
SRR1266949.9294787.1+ tgcccgaggtgtcagcaagacacagaaactgcagtgATGATGGTGATGCTCTTCTCAGGG
SRR1266952.1269097.2- tgcccgaggtgtcagcaagactcagaaactgcagtgATGATGGTGATGCTCTTCTCAGGG
SRR1266952.18524365.1- tgcccgaggtgtcagcaagactcagaaactgcagtgATGATGGTGATGCTCTTCTCAGGG
SRR1266956.5970423.2- tgcccgaggtgtcagcaagactcagaaactgcagtgATGATGGTGATGCTCTTCTCAGGG
SRR1266957.1004692.2+ tgcccgaggtgtcagcaagacacagaaactgcagtgATGATGGTGATGCTCTTCTCAGGG
SRR1266957.3246519.1+ tgcccgaggtgtcagcaagactcagaaactgcagtgATGATGGTGATGCTCTTCTCAGGG
SRR1266959.12079654.1- tgcccgaggtgtcagcaagacacagaaactgcagtgATGATGGTGATGCTCTTCTCAGGG
SRR1266960.9103031.1- tgcccgaggtgtcagcaagactcagaaactgcagtgATGATGGTGATGCTCTTCTCAGGG
SRR1266961.140544.1+ tgcccgaggtgtcagcaagacacagaaactgcagtgATGATGGTGATGCTCTTCTCAGGG
SRR1266961.2852570.2- tgcccgaggtgtcagcaagactcagaaactgcagtgATGATGGTGATGCTCTTCTCAGGG
SRR1266962.3776588.2+ tgcccgaggtgtcagcaagactcagaaactgcagtgATGATGGTGATGCTCTTCTCAGGG
SRR1266963.14458810.2- tgcccgaggtgtcagcaagactcagaaactgcagtgATGATGGTGATGCTCTTCTCAGGG
SRR1266964.8313196.1- tgcccgaggtgtcagcaagactcagaaactgcagtgATGATGGTGATGCTCTTCTCAGGG
SRR1266964.9455231.1- tgcccgaggtgtcagcaagactcagaaactgcagtgATGATGGTGATGCTCTTCTCAGGG
SRR1266965.7238566.2- tgcccgaggtgtcagcaagactcagaaactgcagtgATGATGGTGATGCTCTTCTCAGGG
SRR1266966.3487586.1+ tgcccgaggtgtcagcaagactcagaaactgcagtgATGATGGTGATGCTCTTCTCAGGG
SRR1269689.12646918.2- tgcccgaggtgtcagcaagactcagaaactgcagtgATGATGGTGATGCTCTTCTCAGGG
SRR1269689.6208067.2- tgcccgaggtgtcagcaagactcagaaactgcagtgATGATGGTGATGCTCTTCTCAGGG
SRR1269690.2081589.2+ tgcccgaggtgtcagcaagactcagaaactgcagtgATGATGGTGATGCTCTTCTCAGGG
SRR1269691.2790772.1+ tgcccgaggtgtcagcaagactcagaaactgcagtgATGATGGTGATGCTCTTCTCAGGG
SRR1269691.4350208.1+ tgcccgaggtgtcagcaagactcagaaactgcagtgATGATGGTGATGCTCTTCTCAGGG
SRR1269691.4350208.2- tgcccgaggtgtcagcaagactcagaaactgcagtgATGATGGTGATGCTCTTCTCAGGG
SRR1269693.7819029.1- tgcccgaggtgtcagcaagactcagaaactgcagtgATGATGGTGATGCTCTTCTCAGGG
SRR1269694.4040988.2- tgcccgaggtgtcagcaagactcagaaactgcagtgATGATGGTGATGCTCTTCTCAGGG
SRR1269694.9908338.2+ tgcccgaggtgtcagcaagactcagaaactgcagtgATGATGGTGATGCTCTTCTCAGGG
SRR1269697.3282854.1- tgcccgaggtgtcagcaagactcagaaactgcagtgATGATGGTGATGCTCTTCTCAGGG
SRR1269699.10407563.2- tgcccgaggtgtcagcaagacacagaaactgcagtgATGATGGTGATGCTCTTCTCAGGG
SRR1269700.5425205.1- tgcccgaggtgtcagcaagacacagaaactgcagtgATGATGGTGATGCTCTTCTCAGGG
SRR1269702.9206585.2+ tgcccgaggtgtcagcaagacacagaaactgcagtgATGATGGTGATGCTCTTCTCAGGG
SRR1269721.4648312.1+ tgcccgaggtgtcagcaagacacagaaactgcagtgATGATGGTGATGCTCTTCTCAGGG

```
SRR1269724.10149594.1+   tgcccgaggtgtcagcaagactcagaaactgcagtgATGATGGTGATGCTCTTCTCAGGG
SRR1269724.10149594.2-   tgcccgaggtgtcagcaagactcagaaactgcagtgATGATGGTGATGCTCTTCTCAGGG
SRR1269725.4281425.1+    tgcccgaggtgtcagcaagactcagaaactgcagtgATGATGGTGATGCTCTTCTCAGGG
SRR1269727.8305397.1+    tgcccgaggtgtcagcaagactcagaaactgcagtgATGATGGTGATGCTCTTCTCAGGG
SRR1271606.2596332.2+    tgcccgaggtgtcagcaagactcagaaactgcagtgATGATGGTGATGCTCTTCTCAGGG
SRR1271606.4361690.2-    tgcccgaggtgtcagcaagactcagaaactgcagtgATGATGGTGATGCTCTTCTCAGGG
SRR1271611.10904168.2-   tgcccgaggtgtcagcaagactcagaaactgcagtgATGATGGTGATGCTCTTCTCAGGG
SRR1271611.35773627.2-   tgcccgaggtgtcagcaagactcagaaactgcagtgATGATGGTGATGCTCTTCTCAGGG
SRR1271611.37255256.2-   tgcccgaggtgtcagcaagactcagaaactgcagtgATGATGGTGATGCTCTTCTCAGGG
SRR1271611.44404236.2-   tgcccgaggtgtcagcaagactcagaaactgcagtgATGATGGTGATGCTCTTCTCAGGG
SRR1271611.58180054.1+   tgcccgaggtgtcagcaagactcagaaactgcagtgATGATGGTGATGCTCTTCTCAGGG
SRR1271611.8284106.1+    tgcccgaggtgtcagcaagactcagaaactgcagtgATGATGGTGATGCTCTTCTCAGGG
SRR1271612.1636746.1+    tgcccgaggtgtcagcaagaatcagaaactgcagtgATGATGGTGATGCTCTTCTCAGGG
SRR1271612.6155372.1-    tgcccgaggtgtcagcaagactcagaaactgcagtgATGATGGTGATGCTCTTCTCAGGG
SRR1271616.8117819.1-    tgcccgaggtgtcagcaagactcagaaactgcagtgATGATGGTGATGCTCTCCTCAGGG
SRR1271617.2641915.1+    tgcccgaggtgtcagcaagactcagaaactgcagtgATGATGGTGATGCTCTTCTCAGGG
SRR1271617.61779939.1-   tgcccgaggtgtcagcaagactcagaaactgcagtgATGATGGTGATGCTCTTCTCAGGG
SRR1271617.63603202.2-   tgcccgaggtgtcagcaagactcagaaactgcagtgATGATGGTGATGCTCTTCTCAGGG
SRR1271617.9057728.1+    tgcccgaggtgtcagcaagactcagaaactgcagtgATGATGGTGATGCTCTTCTCAGGG
SRR1271617.91836522.2-   tgcccgaggtgtcagcaagactcagaaactgcagtgATGATGGTGATGCTCTTCTCAGGG
SRR1271618.2332801.2-    tgcccgaggtgtcagcaagactcaaaaactgcagtgATGATGGTTATGCTCTTCTCAGGG
SRR1271619.11721387.2+   tgcccgaggtgtaagcaagactcagaaactgcagtgATGATGGTGATGCTCTTCTCAGGG
SRR1271619.5245116.1+    tgcccgaggtgtcagcaagactcagaaactgcagtgATGATGGGGATGCTCTTCTCAGGG
SRR1271620.7033507.1-    tgcccgaggtgtcagcaagactcagaaactgcagtgATGATGGTGATGCTCTTCTCAGGG
SRR1271620.7196430.1-    tgcccgaggtgtcagcaagactcagaaactgcagtgATGATGGTGATGCTCTTCTCAGGG
SRR1271621.604681.2-     tgcccgaggtgtcagcaagactcagaaactgcagtgATGATGGTGATGCTCTTCTCAGGG
SRR1271623.13311270.2-   tgcccgaggtgtcagcaagactcagaaactgcagtgATGATGGTGATGCTCTTCTCAGGG
SRR1271624.16956133.2-   tgcccgaggtgtcagcaagactcagaaactgcagtgATGATGGTGATGCTCTTCTCAGGG
SRR1271624.17619079.2-   tgcccgaggtgtcagcaagactcagaaactgcagtgATGATGGTGATGCTCTTCTCAGGG
SRR1271624.3358682.1+    tgcccgaggtgtcagcaagactcagaaactgcagtgATGATGGTGATGCTCTTCTCAGGG
SRR1271624.5552917.2+    tgcccgaggtgtcagcaagactcagaaactgcagtgATGATGGTGATGCTCTTCTCAGGG
SRR1271626.1129978.1-    tgcccgaggtgtcagcaagactcagaaactgcagtgATGATGGTGATGCTCTTCTCAGGG
SRR1271626.15408811.1-   tgcccgaggtgtcagcaagactcagaaactgcagtgATGATGGTGATGCTCTTCTCAGGG
SRR1271626.15519384.2+   tgcccgaggtgtcagcaagactcagaaactgcagtgATGATGGTGATGCTCTTCTCAGGG
SRR1271626.18186673.1-   tgcccgaggtgtcagcaagactcagaaactgcagtgATGATGGTGATGCTCTTCTCAGGG
SRR1271626.23923475.2+   tgcccgaggtgtcagcaagactcagaaactgcagtgATGATGGTGATGCTCTTCTCAGGG
SRR1271626.39792399.1-   tgcccgaggtgtcagcaagactcagaaactgcagtgATGATGGTGATGCTCTTCTCAGGG
SRR1271628.74690.2+      tgcccgaggtgtcagcaagactcagaaactgcagtgATGATGGTGATGCTCTTCTCAGGG
SRR1271628.8325711.2-    tgcccgaggtgtcagcaagactcagaaactgcagtgATGATGGTGATGCTCTTCTCAGGG
SRR1271630.5638436.1-    tgcccgaggtgtcagcaagactcagaaactgcagtgATGATGGTGATGCTCTTCTCAGGG
SRR1271630.6944205.2+    tgcccgaggtgtcagcaagactcagaaactgcagtgATGATGGTTATTTTCTTCTCAGGG
SRR1271631.104145897.1+  tgcccgaggtgtcagcaagactcagaaactgcagtgATGATGGTGATGCTCTTCTCAGGG
SRR1271631.116517047.2-  tgcccgaggtgtcagcaagactcagaaactgcagtgATGATGGTGATGCTCTTCTCAGGG
SRR1271631.15126426.1-   tgcccgaggtgtcagcaagactcagaaactgcagtgATGATGGTGATGCTCTTCTCAGGG
SRR1271631.29333027.2-   tgcccgaggtgtcagcaagactcagaaactgcagtgATGATGGTGATGCTCTTCTCAGGG
SRR1271631.51751938.2+   tgcctgaggtgtcagcaagactcagaaactgcagtgATGATGGTGATGCTCTTCTCAGGG
SRR1271631.53104753.1+   tgcccgaggtgtcagcaagactcagaaactgcagtgATGATGGTGATGCTCTTCTCAGGG
SRR1271631.54114509.1-   tgcccgaggtgtcagcaagactcagaaactgcagtgATGATGGTGATGCTCTTCTCAGGG
SRR1271631.60482848.2+   tgcccgaggtgtcagcaagactcagaaactgcagtgATGATGGTGATGCTCTTCTCAGGG
SRR1271631.75535824.1+   tgcccgaggggtcagcaagactcagaaactgcagtgATGATGGTGATGCTCTTCTCAGGG
SRR1271631.75871170.1-   tgcccgaggtgtcagcaagactcagaaactgcagtgATGATGGTGATGCTCTTCTCAGGG
SRR1271631.78235890.2+   tgcccgaggtgtcagcgagactcagaaactgcagtgATGATGGTGATGCTCTTCTCAGGG
SRR1271631.7995179.2+    tgcacgaggtgtcagcaagactcagaaactgcagtgATGATGGTGATGCTCTTCTCAGGG
SRR1271631.96785903.1-   tgcccgaggtgtcagcaagactcagaaactgcagtgATGATGGTGATGCTCTTCTCAGGG
SRR1271635.2853328.1-    tgcccgaggtgtcagcaagactcagaaactgcagtgATGATGGTGATGCTCTTCTCAGGG
SRR1271636.10973309.2+   tgcccgaggtgtcagcaagactcagaaactgcagtgATGATGGTGATGCTCTTCTCAGGG
SRR1271636.22676106.2-   tgcccgaggtgtcagcaagactcagaaactgcagtgATGATGGTGATGCTCTTCTCAGGG
SRR1271636.29905606.2-   tgcccgaggtgtcagcaagactcagaaactgcagtgATGATGGTGATGCTCTTCTCAGGG
SRR1271636.7067284.1+    tgcccgaggtgtcagcaagactcagaaactgcagtgATGATGGTGATGCTCTTCTCAGGG
SRR1271637.7917479.2+    tgcccgaggtgtcagcaagactcagaaactgcagtgATGATGGGGATGCTCTTCTCAGGG
SRR1271638.16782324.2-   tgcccgaggtgtcagcaagactcagaaactgcagtgATGATGGTGATGCTCTTCTCAGGG
SRR1271638.4121191.2-    tgcccgaggtgtcagcaagactcagaaactgcagtgATGATGGTGATGCTCTTCTCAGGG
SRR1271639.5769975.2+    tgcccgaggtgtcagcaagactcagaaactgcagtgATGATGGTGATGCTCTTCTCAGGG
SRR1271640.3286459.2-    tgcccgaggtgtcagcaagactcagaaactgcagtgATGATGGTGATGCTCTTCTCAGGG
SRR1271641.1828285.2+    tgcccgaggtgtcagcaagacacagaaactgcagtgATGATGGTGATGCTCTTCTCAGGG
SRR1271641.4994077.1-    tgcccgaggtgtcagcaagactcagaaactgcagtgATGATGGTGATGCTCTTCTCAGGG
SRR1271641.5711711.1+    tgcccgaggtgtcagcaagactcagaaactgcagtgATGATGGTGATGCTCTTCTCAGGG
SRR1271641.8508693.1-    tgcccgaggtgtcagcaagacacagaaactgcagtgATGATGGTGATGCTCTTCTCAGGG
SRR1271644.6211925.2+    tgcccgaggtgtcagcaagacacagaaactgcagtgATGATGGTGATGCTCTTCTCAGGG
SRR1271656.14780067.1-   tgcccgaggtgtcagcaagactcagaaactgcagtgATGATGGTGATGCTCTTCTCAGGG
SRR1271656.32340673.1-   tgcccgaggtgtcagcaagactcagaaactgcagtgATGATGGTGATGCTCTTCTCAGGG
SRR1271656.37726636.2+   tgcccgaggtgtcagcaagactcagaaactgcagtgATGATGGTGATGCGCTTCTCAGGG
SRR1271656.40813652.1+   tgcccgaggtgtcagcaagactcagaaactgcagtgATGATGGTGATGCTCTTCTCAGGG
SRR1271656.45546724.1-   tgcccgaggtgtcagcaagactcagaaactgcagtgATGATGGTGATGCTCTTCTCAGGG
SRR1271656.45740696.2+   tgcccgaggtgtcagcaagacacagaaactgcagtgATGATGGTGATGCTCTTCTCAGGG
SRR1271656.51406426.2-   tgcccgaggtgtcagcaagacacagaaactgcagtgATGATGGTGATGCTCTTCTCAGGG
SRR1271660.15638503.1+   tgcctgaggtgtcagcaagactcagaaactgcagtgATGATGGTGATGCTCTTCTCAGGG
SRR1271660.22996263.2-   tgcctgaggtgtcagcaagactcagaaactgcagtgATGATGGTGATGCTCTTCTCAGGG
SRR1271661.7415342.1-    tgcctgaggtgtcagcaagactcagaaactgcagtgATGATGGTGATGCTCTTCTCAGGG
SRR1271661.7471914.1+    tgcctgaggtgtcagcaagactcagaaactgcagtgATGATGGGGATGCTCTTCTCAGGG
SRR1271661.7818147.1+    tgcctgaggtgtcagcaagactcagaaactgcagtgATGATGGTGATGCTCTTCTCAGGG
SRR1271664.6779123.1+    tgcccgaggtgtcagcaagactcagaaactgcagtgATGATGGTGATGCTCTTCTCAGGG
SRR1271665.12472605.2-   tgcccgaggtgtcagcaagactcagaaactgcagtgATGATGGTGATGCTCTTCTCAGGG
SRR1271665.4483121.1-    tgcccgaggtgtcagcaagactcagaaactgcagtgATGATGGTGATGCTCTTCTCAGGG
```

```
SRR1265421.3532440.1-     gcccgaggtgtcagcaagactcagaaactgcagtgATGATGGTGATGCTCTTCTCAGGG
SRR1266951.538073.2+      gcccgaggtgtcagcaagactcagaaactgcagtgATGATGGTGATGCTCTTCTCAGGG
SRR1266961.140544.2-      gcccgaggtgtcagcaagacacagaaactgcagtgATGATGGTGATGCTCTTCTCAGGG
SRR1265959.14596287.2+     cccgaggtgtcagcaagactcagaaactgcagtgATGATGGTGATGCTCTTCTCAGGG
SRR1266948.20090114.2-     cccgaggtgtcagcaagactcagaaactgcagtgATGATGGTGATGCTCTTCTCAGGG
SRR1265429.4871220.2-       ccgaggtgtcagcaagactcagaaactgcagtgATGATGGTGATGCTCTTCTCAGGG
SRR1271612.777884.2-         cgaggtgtcagcaagactcagaaactgcagtgATGATGGTGATGCTCTTCTCAGGG
SRR1265485.4295901.1+         gaggtgtcagcaagactcagaaactgcagtgATGATGGTGATGCTCTTCTCAGGG
SRR1266953.6459693.2-         gaggtgtcagcaagactcagaaactgcagtgATGATGGTGATGCTCTTCTCAGGG
SRR1266955.9340457.2+         gaggtgtcagcaagactcagaaactgcagtgATGATGGTGATGCTCTTCTCAGGG
SRR1266960.7618362.2+         gaggtgtcagcaagactcagaaactgcagtgATGATGGTGATGCTCTTCTCAGGG
SRR1266960.863619.1+          gaggtgtcagcaagactcagaaactgcagtgATGATGGCGGTGCTCTTCTCAGGG
SRR1271611.51705152.2+        gaggtgtcagcaagactcagaaactgcagtgATGATGGTGATGCTCTTCTCAGGG
SRR1271620.10933616.1+        gaggtgtcagcaagactcagaaactgcagtgATGATGGTGATGCTCTTCTCAGGG
SRR1271626.5381257.2+         gaggtgtcagcaagactcagaaactgcagtgATGATGGTGATGCTCTTCTCAGGG
SRR1265430.10056360.2+         aggtgtcagcaagactcagaaactgcagtgATGATGGTGATGCTCTTCTCAGGG
SRR1271656.21063060.2+         aggtgtcagcaagacacagaaactgcagtgATGATGGTGATGCTCTTCTCAGGG
SRR1265961.21125338.1+          ggtgtcagcaagactcagaaactgcagtgATGATGGTGATGCTCTTCTCAGGG
SRR1266964.9037991.2-           ggtgtcagcaagactcagaaactgcagtgATGATGGTGATGCTCTTCTCAGGG
SRR1271617.107030387.1-         ggtgtcagcaagactcagaaactgcagtgATGATGGTGATGCTCTTCTCAGGG
SRR1271664.9315059.2+           ggtgtcagcaagactcagaaactgcagtgATGATGGTGATGCTCTTCTCAGGG
SRR1265108.11035394.1+           gtgtcagcaagactcagaaactgcagtgATGATGGTGATGCTCTTCTCAGGG
SRR1265425.8225295.2+            gtgtcagcaagactcagaaactgcagtgATGATGGTGATGCTCTTCTCAGGG
SRR1269700.383429.2+             gtgtcagcaagacacagaaactgcagtgATGATGGTGATGCTCTTCTCAGGG
SRR1271626.30577518.1-           gtgtcagcaagactcagaaactgcagtgATGATGGTGATGCTCTTCTCAGGG
SRR1265492.3432458.1+             tgtcagcaagactcagaaactgcagtgATGATGGTGATGCTCTTCTCAGGG
SRR1266954.5286348.2-             tgtcagcaagactcagaaactgcagtgATGATGGTGATGCTCTTCTCAGGG
SRR1266962.1283028.2-             tgtcagcaagactcagaaactgcagtgATGATGGTGATGCTCTTCTCAGGG
SRR1271631.18001001.2-            tgtcagcaagactcagaaactgcagtgATGATGGTGATGCTCTTCTCAGGG
SRR1266951.9077736.1-              gtcagcaagactcagaaactgcagtgATGATGGTGATGCTCTTCTCAGGG
SRR1266959.5837465.2+              gtcagcaagacacagaaactgcagtgATGATGGTGATGCTCTTCTCAGGG
SRR1266961.10240545.2-             gtcagcaagactcagaaactgcagtgATGATGGTGATGCTCTTCTCAGGG
SRR1271617.13985324.1-             gtcagcaagactcagaaactgcagtgATGATGGTGATGCTCTTCTCAGGG
SRR1271635.2818304.1-               tcagcaagactcagaaactgcagtgATGATGGTGATGCTCTTCTCAGGG
SRR1265960.10540450.1+               cagcaagactcagaaactgcagtgATGATGGTGATGCTCTTCTCAGGG
SRR1269723.820781.2+                 cagcaagacacagaaactgcagtgATGATGGTGATGCTCTTCTCAGGG
SRR1271606.5849136.2+                cagcaagactcagaaactgcagtgATGATGGTGATGCTCTTCTCAGGG
SRR1265120.5268240.2+                 agcaagactcagaaactgcagtgATGATGGAGATGCTCTTCTCAGGG
SRR1265421.25226457.2+                agcaagactcagaaactgcagtgATGATGGTGATGCTCTTCTCAGGG
SRR1271605.16567794.1+                agcaagactcagaaactgcagtgATGATGGTGATGCTCTTCTCAGGG
SRR1266966.13263794.2-                 gcaagactcagaaactgcagtgATGATGGTGATGCTCTTCTCAGGG
SRR1269721.8669041.1+                  gcaagacacagaaactgcagtgATGATGGTGATGCTCTTCTCAGGG
SRR1271656.20184311.1+                 gcaagactcagaaactgcagtgATGATGGTGATGCTCTTCTCAGGG
SRR1271656.31545570.2+                 gcaagactcagaaactgcagtgATGATGGTGATGCTCTTCTCAGGG
SRR1271611.40265863.2+                  caagactcagaaactgcagtgATGATGGTGATGCTCTTCTCAGGG
SRR1271641.8238182.2+                    aagactcagaaactgcagtgATGATGGTGATGCTCTTCTCAGGG
SRR1271665.16214124.1+                   aagactcagaaactgcagtgATGATGGTGATGCTCTTCTCAGGG
SRR1271631.53790917.2+                    agactcagaaactgcagtgATGATGGTGATGCTCTTCTCAGGG
SRR1271636.20236431.2+                    agactcagaaactgcagtgATGATGGTGATGCTCTTCTCAGGG
SRR1265424.13096215.1+                     gactcagaaactgcagtgATGATGGTGATGCTCTTCTCAGGG
SRR1269694.10704014.2-                     gactcagaaactgcagtgATGATGGTGATGCTCTTCTCAGGG
SRR1271618.4133814.1-                      gactcagaaactgcagtgATGATGGTGATGCTCTTCTCAGGG
SRR1271631.61406029.2+                     gactcagaaactgcagtgATGATGGTGATGCTCTTCTCAGGG
SRR1271611.1989144.2+                       actcagaaactgcagtgATGATGGTGATGCTCTTCTCAGGG
SRR1271626.12852273.2-                      actcagaaactgcagtgATGATGGTGATGCTCTTCTCAGGG
SRR1271626.6889032.2-                       actcagaaactgcagtgATGATGGTGATGCTCTTCTCAGGG
SRR1271637.7730229.1+                       actcagaaactgcagtgATGATGGTGATGCTCTTCTCAGGG
SRR1266953.271055.1-                         ctcagaaactgcagtgATGATGGTGATGCTCTTCTCAGGG
SRR1271642.3577506.2-                        ctcagaaactgcagtgATGATGGTGATGCTCTTCTCAGGG
SRR1271656.42884483.2+                       cacagaaactgcagtgATGATGGTGATGCTCTTCTCAGGG
SRR1269723.7787279.2+                         acagaaactgcagtgATGATGGTGATGCTCTTCTCAGGG
SRR1271618.10995740.1-                        tcagaaactgcagtgATGATGGTGATGCTCTTCTCAGGG
SRR1271631.17796850.1+                        tcagaaactgcagtgATGATGGTGATGCTCTTCTCAGGG
SRR1265418.1061795.1-                          cagaaactgcagtgATGATGGTGATGCTCTTCTCAGGG
SRR1271617.65162719.1+                         cagaaactgcagtgATGATGGTGATGCTCTTCTCAGGG
SRR1271631.73182052.1+                         cagaaactgcagtgATGATGGTGATGCTCTTCTCAGGG
SRR1265959.5289237.2+                           agaaactgcagtgATGATGGTGATGCTCTTCTCAGGG
SRR1269691.1086423.1+                           agaaactgcagtgATGATGGTGATGCTCTTCTCAGGG
SRR1271661.3541586.2+                           agaaactgcagtgATGATGGTGATGCTCTTCTCAGGG
SRR1265429.662140.1-                             gaaactgcagtgATGATGGTGATGCTCTTCTCAGGG
SRR1271622.14489347.2+                           gaaactgcagtgATGATGGTGATGCTCTTCTCAGGG
SRR1265101.6853887.1-                             aaactgcagtgATGATGGTGATGCTCTTCTCAGGG
SRR1266954.10031696.1-                            aaactgcagtgATGATGGTGATGCTCTTCTCAGGG
SRR1271611.54109106.1-                            aaactgcagtgATGATGGTGATGCTCTTCTCAGGG
SRR1271627.817121.1-                              aaactgcagtgATGATGGTGATGCTCTTCTCAGGG
SRR1271631.12525033.2+                            aaactgcagtgATGATGGTGATGCTCTTCTCAGGG
SRR1271638.7137901.1+                             aaactgcagtgATGATGGTGATGCTCTTCTCAGGG
SRR1271665.15301974.1-                            aaactgcagtgATGATGGTGATGCTCTTCTCAGGG
SRR1271611.3494094.2-                              aactgcagtgATGATGGTGATGCTCTTCTCAGGG
SRR1271629.12769352.1-                              actgcagtgATGATGGTGATGCTCTTCTCAGGG
SRR1271640.2387601.1-                               actgcagtgATGATGGTGATGCTCTTCTCAGGG
SRR1265961.2477855.1+                                ctgcagtgATGATGGTGATGCTCTTCTCAGGG
SRR1265962.4168719.2-                                ctgcagtgATGATGGTGATGCTCTTCTCAGGG
SRR1266947.19802521.2+                               ctgcagtgATGATGGTGATGCTCTTCTCAGGG
```

SRR1266966.437734.2+	ctgcagtgATGATGGTGATGCTCTTCTCAGGG
SRR1271620.2068946.1-	ctgcagtgATGATGGTGATGCTCTTCTCAGGG
SRR1271631.70375615.1-	ctgcagtgATGATGGTGATGCTCTTCTCAGGG
SRR1265105.2962579.1-	tgcagtgATGATGGTGATGCTCTTCTCAGGG
SRR1266961.9230787.2-	tgcagtgATGATGGTGATGCTCTTCTCAGGG
SRR1271642.4563788.2-	tgcagtgATGATGGTGATGCTCTTCTCAGGG
SRR1265102.11716845.1+	gcagtgATGATGGTGATGCTCTTCTCAGGG
SRR1265424.11995558.2+	gcagggATGATGGTGATGCTCTTCTCAGGG
SRR1266956.13063849.1+	gcagtgATGATGGTGATGCTCTTCTCAGGG
SRR1266962.4122587.1+	gcagtgATGATGGTGATGCTCTTCTCAGGG
SRR1271609.4569674.1-	gcagtgATGATGGTGATGCTCTTCTCAGGG
SRR1271627.6371503.1-	gcagtgATGATGGTGATGCTCTTCTCAGGG
SRR1271631.66929254.2-	gcagtgATGATGGTGATGCTCTTCTCAGGG
SRR1271639.961470.1-	gcagtgATGATGGTGATGCTCTTCTCAGGG
SRR1265486.10320482.1-	cagtgATGATGGTGATGCTCTTCTCAGGG
SRR1266958.1653120.2-	cagtgATGATGGTGATGCTCTTCTCAGGG
SRR1265107.8255291.2-	gtgATGATGGTGATGCTCTTCTCAGGG
SRR1271631.13703327.2+	gtgATGATGGTGATGCTCTTCTCAGGG
SRR1265094.4259059.2+	tgATGATGGTGATGCTCTTCTCAGGG
SRR1265419.2098789.1+	tgATGATGGTGATGCTCTTCTCAGGG
SRR1266948.8806088.2+	tgATGATGGTGATGCTCTTCTCAGGG
SRR1265487.11821367.2+	gATGATGGTGATGCTCTTCTCAGGG
SRR1269690.6866648.2+	gATGATGGTGATGCTCTTCTCAGGG
SRR1269701.5896733.1+	gATGATGGTGATGCTCTTCTCAGGG
SRR1271605.649185.1+	gATGATGGTGATGCTCTTCTCAGGG
SRR1271611.30562393.2+	gATGATGGTGATGCTCTTCTCAGGG
SRR1265423.6251649.1-	ATGATGGTGATGCTCTTCTCAGGG
SRR1266962.11554845.2+	ATGATGGTGATGCTCTTCTCAGGG
SRR1269696.1594481.2+	ATGATGGTGATGCTCTTCTCAGGG
SRR1271643.6174127.1+	ATGATGGTGATGCTCTTCTCAGGG
SRR1271610.5829611.1+	TGATGGTGATGCTCTTCTCAGGG
SRR1271624.3306484.1+	TGATGGTGATGCTCTTCTCAGGG
SRR1265106.6015462.2+	GATGGTGATGCTCTTCTCGGGA
SRR1265108.2515604.1+	GATGGTGATGCTCTTCTCAGGG
SRR1265424.15339550.2-	GATGGTGATGCTCTTCTCAGGT
SRR1271611.16853764.2+	GATGGTGATGCTCTTCTCAGGG
SRR1271622.14382387.1+	GATGGTGATGCTCTTCTCAGGG
SRR1271626.3653174.1-	GATGGTGATGCTCTTCTCAGGG
SRR1271639.12989136.2+	GATGGTGATGCTCTTCTCAGGG
SRR1271626.19282964.2+	ATGGTGATGCTCTTCTCAGGG
SRR1265090.6845871.1+	TGGTGATGCTCTTCTCAGGG
SRR1266962.7593491.1+	TGGTGATGCTCTTCTCAGGG
SRR1271614.6562104.2-	TGGTGATGCTCTTCACAGGG
SRR1271666.15761999.1-	TGGTGATGCTCTTCTCAGGG
SRR1265422.11840911.2+	GGTGATGCTCTTCTCAGGG
SRR1265423.4185373.1-	GGTGATGCTCTTCTCAGGG
SRR1265494.4458321.1-	GGTGATGCTCTTCTCAGGG
SRR1271625.17771509.2+	GGTGATGCTCTTCTCAGGG
SRR1271665.16602572.1+	GGTGATGCTCTTCTCAGGG
SRR1269726.363820.1+	GTGATGCTCTTCTCAGGG
SRR1271616.2011111.2+	TGATGCTCTTCTCAGGG
SRR1271626.27417879.2+	TGATGCTCTTCTCAGGG
SRR1271630.3176113.1+	TGATGCTCTTCTCAGGG
SRR1271664.2748803.2-	TGATGCTCTTCTCAGGG
SRR1265423.845833.1+	GATGCTCTTCTCAGGG
SRR1265425.678706.1+	GATGCTCTTCTCAGGG
SRR1265960.14555341.2+	GATGCTCTTCTCAGGG
SRR1266948.14467911.1+	GATGCTCTTCTCAGGG
SRR1269695.4570879.2-	GATGCTCTTCTCAGGG
SRR1271631.92663139.1+	GATGCTCTTCTCAGGG
SRR1266946.18245078.1-	ATGCTCTTCTCAGGG
SRR1271620.711931.1-	ATGCTCTTCTCAGGG
SRR1265424.9055674.2-	TGCTCTTCTCAGGG
SRR1269690.5523621.2-	TGCTCTTCTCAGGG
SRR1271611.24840466.2-	TGCTCTTCTCAGGG
SRR1271637.6571114.2-	TGCTCTTCTCAGGG
SRR1265102.5685651.2+	GCTCTTCTCAGGG
SRR1265485.1858508.1-	GCTCTTCTCAGGG
SRR1266952.14069486.2+	GCTCTTCTCAGGG
SRR1271627.4158392.1-	GCTCTTCTCAGGG
SRR1271661.6840898.1-	GCTCTTCTCAGGG
SRR1271666.12193922.1-	GCTCTTCTCAGGG
SRR1265420.9704864.2+	CTCTTCTCAGGG
SRR1265960.4362410.1+	CTCTTCTCAGGG
SRR1266950.19035200.2+	CTCTTCTCAGGG
SRR1269721.13769927.2-	CTCTTCTCAGGG
SRR1271617.21164005.1+	TCTTCTCAGGG
SRR1271631.15064325.1-	TCTTCTCAGGG
SRR1271661.9678304.1+	TCTTCTCAGGG
SRR1265485.16069283.1-	CTTCTCAGGG
SRR1265493.882472.2+	CTTCTCAGGG
SRR1265960.20313739.2-	CTTCTCAGGG
SRR1271626.35055944.1-	CTTCTCAGGG
SRR1271629.8867623.1-	CTTCTCAGGG

```
SRR1271663.7532180.2-                                                      CTTCTCAGGG
SRR1271609.1031300.1+                                                       TTCTCAGGG
SRR1271611.10364010.1+                                                      TTCTCAGGG
SRR1271631.56849156.1+                                                      TTCTCAGGG
SRR1271635.1018397.2+                                                       TTCTCAGGG
SRR1265485.8142050.2-                                                        TCTCAGGG
SRR1266960.6717190.2-                                                        TCTCAGGG
SRR1265103.11096904.1+                                                        CTCAGGG
SRR1265106.6372305.2-                                                         CTCAGGG
SRR1266947.14185884.1+                                                        CTCAGGG
SRR1266951.18466248.1-                                                        CTCAGGG
SRR1269700.1764719.1+                                                         CTCAGGG
SRR1265421.11935315.2-                                                         TCAGGG
SRR1265959.15507634.1-                                                         TCAGGG
SRR1269720.6697396.2+                                                          TCAGGG
SRR1271611.18839548.2-                                                         TCAGGG
SRR1271611.38267951.1-                                                         TCAGGG
SRR1271618.11716789.1-                                                         TCAGGG
SRR1271635.4911972.1-                                                          TCAGGG
SRR1265485.10372300.1+                                                          CAGGG
SRR1265486.1000359.1-                                                           CAGGG
SRR1265487.14014666.1+                                                          CAGGG
SRR1271611.5176815.1+                                                           CAGGG
SRR1271642.1942769.2-                                                           CAGGA
SRR1271656.28111822.2+                                                          CAGGG
SRR1271656.48186978.2-                                                          CAGGG
SRR1265493.1938206.2-                                                            AGGG
SRR1271617.107171253.2-                                                          AGGG
SRR1265093.9367806.1+                                                             GGG
SRR1265429.3960386.1-                                                             GGG
SRR1271656.40523025.1-                                                            GGG
SRR1266952.5817620.2-                                                              GG
SRR1266956.8774588.1-                                                              GG
SRR1266965.13605504.1-                                                             GG
SRR1269696.7386843.1-                                                              GG
SRR1271656.16911801.1-                                                             GG
SRR1265100.8844830.1-                                                               G
SRR1271617.106361713.2+                                                             G
SRR1271617.35068629.2+                                                              G
SRR1271617.93090910.1+                                                              G

consensus                tgcccgaggtgtcagcaagactcagaaactgcagtgATGATGGTGATGCTCTTCTCAGGG

                             .    :    .    :    .    :    .    :    .    :    .    :
SRR1265425.3715610.1-    A
SRR1265962.6217934.1+    A
SRR1266963.14458810.2-   A
SRR1266960.9103031.1-    AT
SRR1271661.7818147.1+    AT
SRR1269724.10149594.1+   ATC
SRR1271636.7067284.1+    ATC
SRR1271639.5769975.2+    ATC
SRR1271660.15638503.1+   ATC
SRR1265108.9817332.2-    ATCA
SRR1265961.20492731.2-   ATCA
SRR1266956.5970423.2-    ATCA
SRR1271611.58180054.1+   ATCA
SRR1271635.2853328.1-    ATCA
SRR1271611.10904168.2-   ATCAA
SRR1271626.18186673.1-   ATCAA
SRR1271631.75871170.1-   ATCAA
SRR1271636.29905606.2-   ATCAA
SRR1265420.8458569.1+    ATCAAG
SRR1271606.4361690.2-    ATCAAG
SRR1271611.35773627.2-   ATCAAG
SRR1271631.29333027.2-   ATCAAG
SRR1265103.985070.1+     ATCAAGA
SRR1265420.8209771.1+    ATCAAGA
SRR1266961.140544.1+     ATCAAGA
SRR1266966.3487586.1+    ATCAAGA
SRR1271612.1636746.1+    ATCAAGA
SRR1265961.17164072.2+   ATCAAGAG
SRR1266964.8313196.1-    ATCAAGAG
SRR1271631.104145897.1+  ATCAAGAG
SRR1271660.22996263.2-   ATCAAGAG
SRR1271612.6155372.1-    ATCAAGAGA
SRR1265486.14296408.1-   ATCAAGAGAA
SRR1271624.3358682.1+    ATCAAGAGAA
SRR1271628.74690.2+      ATCAAGAGAA
SRR1265117.5894445.1-    ATCAAGAGAAT
SRR1271617.2641915.1+    ATCAAGAGAAT
SRR1271617.63603202.2-   ATCAAGAGAAT
SRR1271619.5245116.1+    ATCAAGAGAAT
SRR1271626.15408811.1-   ATCAAGAGAAT
```

SRR1271656.45546724.1-   ATCAAGAGAAT
SRR1265423.8357279.2-   ATCAAGAGAATGC
SRR1265959.10232564.2-   CTCAAGAGAATGC
SRR1271626.1129978.1-   ATCAAGAGAATGC
SRR1271631.78235890.2+   ATCAAGAGAATGC
SRR1265117.8111828.1+   ATCAAGAGAATGCT
SRR1265486.4188250.2-   ATCAAGAGAATGCT
SRR1269690.2081589.2+   ATCAAGAGAATGCT
SRR1269694.9908338.2+   ATCAAGAGAATGCT
SRR1271631.116517047.2-   ATCAAGAGAATGCT
SRR1271626.15519384.2+   ATCAAGAGAATGCTC
SRR1271661.7415342.1-   ATCAAGAGAATGCTC
SRR1271619.11721387.2+   ATCAAGAGAATGCTCT
SRR1271638.16782324.2-   ATCAAGAGAATGCTCT
SRR1271638.4121191.2-   ATCAAGAGAATGCTCT
SRR1266949.3696068.1-   ATCAAGAGAATGCTCTT
SRR1269697.3282854.1-   ATCAAGAGAATGCTCTT
SRR1265429.1518485.1+   ATCAAGAGAATGCTCTTC
SRR1271621.604681.2-   ATCAAGAGAATGCTCTTC
SRR1271640.3286459.2-   ATCAAGAGAATGCTCTTC
SRR1269693.7819029.1-   ATCAAGAGAATGCTCTTCC
SRR1269724.10149594.2-   ATCAAGAGAATGCTCTTCC
SRR1271637.7917479.2+   ATCAAGAGAATGCTCTTCC
SRR1271656.32340673.1-   ATCAAGAGAATGCTCTTCC
SRR1265492.2995376.1-   ATCAAGAGAATGCTCTTCCT
SRR1269702.9206585.2+   ATCAAGAGAATGCTCTTCCT
SRR1271641.4994077.1-   ATCAAGAGAATGCTCTTCCT
SRR1271656.14780067.1-   ATCAAGAGAATGCTCTTCCT
SRR1271623.13311270.2-   ATCAAGAGAATGCTCTTCCTC
SRR1271628.8325711.2-   ATCAAGAGAATGCTCTTCCTC
SRR1265486.5317212.2-   ATCAAGAGAATGCTCTTCCTCT
SRR1266961.2852570.2-   ATCAAGAGAATGCTCTTCCTCT
SRR1266964.9455231.1-   ATCAAGAGAATGCTCTTCCTCT
SRR1271620.7033507.1-   ATCAAGAGAATGCTCTTCCTCT
SRR1271631.15126426.1-   ATCAAGAGAATGCTCTTCCTCT
SRR1265094.1737834.1-   ATCAAGAGAATGCTCTTCCTCTT
SRR1265485.7131594.1-   ATCAAGAGAATGCTCTTCCTCTT
SRR1265961.5907868.2-   ATCAAGAGAATGCTCTTCCTCTT
SRR1266952.1269097.2-   ATCAAGAGAATGCTCTTCCTCTT
SRR1271664.6779123.1+   ATCAAGAGCATGCTCTTCCTCTT
SRR1265116.9489829.1-   ATCAAGAGAATGCTCTTCCTCTTG
SRR1265962.10464106.1+   ATCAAGAGAATGCTCTTCCTCTTG
SRR1271631.51751938.2+   ATCAAGAGAATGCTCTTCCTCTTG
SRR1271631.75535824.1+   ATCAAGAGAATGCTCTTCCTCTTG
SRR1265427.10252303.2+   ATCAAGAGAATGCTCTTCCTCTTGT
SRR1271606.2596332.2+   ATCAAGAGAATGCTCTTCCTCTTGT
SRR1271636.10973309.2+   ATCAAGAGAATGCTCTTCCTCTTGT
SRR1265108.3192987.1-   ATCAAGAGAATGCTCTTCCTCTTGTT
SRR1269694.4040988.2-   ATCAAGAGAATGCTCTTCCTCTTGTT
SRR1269721.4648312.1+   ATCAAGAGAATGCTCTTCCTCTTGTT
SRR1271624.17619079.2-   ATCAAGAGAATGCTCTTCCTCTTGTT
SRR1271665.12472605.2-   ATCAAGAGAATGCTCTTCCTCTTGTT
SRR1265429.13415617.2+   ATCAAGAGAATGCTCTTCCTCTTGTTC
SRR1271656.37726636.2+   ATCAAGAGAATGCTCTTCCTCTTGTTC
SRR1266947.12676414.2-   ATCAAGAGAATGCTCTTCCTCTTGTTCC
SRR1266965.7238566.2-   ATCAAGAGAATGCTCTTCCTCTTGTTCC
SRR1271630.5638436.1-   ATCAAGAGAATGCTCTTCCTCTTGTTCC
SRR1265091.7293804.2+   ATCAAGAGAATGCTCTTCCTCTTGTTCCT
SRR1265419.9630529.2+   ATCAAGAGAATGCTCTTCCTCTTGTTCCT
SRR1265422.16304480.2+   TTCAAGAGAATGCTCTTCCTCTCGTTCCT
SRR1269691.4350208.1+   ATCAAGAGAATGCTCTTCCTCTTGTTCCT
SRR1269691.4350208.2-   ATCAAGAGAATGCTCTTCCTCTTGTTCCT
SRR1269700.5425205.1-   ATCAAGAGAATGCTCTTCCTCTTGTTCCT
SRR1271626.23923475.2+   ATCAAGAGAATGCTCTTCCTCTTGTTCCT
SRR1271631.54114509.1-   ATCAAGAGAATGCTCTTCCTCTTGTTCCT
SRR1265120.12719577.1+   ATCAAGAGAATGCTCTTCCTCTTGTTCCTC
SRR1265490.6979773.2-   ATCAAGAGAATGCTCTTCCTCTTGTTCCTC
SRR1269689.6208067.2-   ATCAAGAGAATGCTCTTCCTCTTGTTCCTC
SRR1271656.45740696.2+   ATCAAGAGAATGCTCTTCCTCTTGTTCCTC
SRR1271656.51406426.2-   ATCAAGAGAATGCTCTTCCTCTTGTTCCTC
SRR1269691.2790772.1+   ATCAAGAGAATGCGCTTCCTCTTGTTCCTCC
SRR1271611.44404236.2-   ATCAAGAGAATGCTCTTCCTCTTGTTCCTCC
SRR1271617.91836522.2-   ATCAAGAGAATGCTCTTCCTCTTGTTCCTCC
SRR1271656.40813652.1+   ATCAAGAGAATGCTCTTCCTCTTGTTCCTCC
SRR1271661.7471914.1+   ATCAAGAGAATGCTCTTCCTCTTGTTCCTCC
SRR1265419.5834061.1+   ATCAAGAGAATGCTCTTCCTCTTGTTCCTCCC
SRR1269699.10407563.2-   ATCAAGAGAATGCTCTTCCTCTTGTTCCTCCC
SRR1271617.9057728.1+   ATCAAGAGAATGCTCTTCCTCTTGTTCCTCCC
SRR1271630.6944205.2+   AGCAAGAGAATGCTCTTCCTCTTGTTCCTCCC
SRR1271665.4483121.1-   ATCAAGAGAATGCTCTTCCTCTTGTTCCTCCC
SRR1265420.9671347.2-   ATCAAGAGAATGCTCTTCCTCTTGTTCCTCCCA
SRR1265427.143510.1-   ATCAAGAGAATGCTCTTCCTCTTGTTCCTCCCA
SRR1265492.1503780.2-   ATCAAGAGAATGCTCTTCCTCTTGTTCCTCCCA
SRR1271618.2332801.2-   ATCAAGAGAATGCTCTTCCTCTTGTTCCTCCCA

```
SRR1265421.22002067.1+   ATCAAGAGAATGCTCTTCCTCTTGTTCCTCCCAT
SRR1266959.12079654.1-   ATCAAGAGAATGCTCTTCCTCTTGTTCCTCCCAT
SRR1271617.61779939.1-   ATCAAGAGAATGCTCTTCCTCTTGTTCCTCCCAT
SRR1271631.96785903.1-   ATCAAGAGAATGCTCTTCCTCTTGTTCCTCCCAT
SRR1271641.8508693.1-    ATCAAGAGAATGCTCTTCCTCTTGTTCCTCCCAT
SRR1265092.8008049.2-    ATCAAGAGAATGCTCTTCCTCTTGTTCCTCCCATG
SRR1265107.5946926.1+    ATCAAGAGAATGCTCTTCCTCTTGTTCCTCCCATG
SRR1266949.9294787.1+    ATCAAGAGAATGCTCTTCCTCTTGTTCCTCCCATG
SRR1266952.18524365.1-   ATCAAGAGAATGCTCTTCCTCTTGTTCCTCCCATG
SRR1269725.4281425.1+    ATCAAGAGAATGCTCTTCCTCTTGTTCCTCCCATG
SRR1271611.8284106.1+    ATCAAGAGAATGCTCTTCCTCTTGTTCCTCCCATG
SRR1271626.39792399.1-   ATCAAGAGAATGCTCTTCCTCTTGTTCCTCCCATG
SRR1271631.53104753.1+   ATCAAGAGAATGCTCTTCCTCTTGTTCCTCCCATG
SRR1271631.60482848.2+   ATCAAGAGAATGCTCTTCCTCTTGTTCCTCCCATG
SRR1271631.7995179.2+    ATCAAGAGAATGCTCTTCCTCTTGTTCCTCCCATG
SRR1265492.6404320.2+    ATCAAGAGAATGCTCTTCCTCTTGTTCCTCCCATGC
SRR1266962.3776588.2+    ATCAAGAGAATGCTCTTCCTCTTGTTCCTCCCATGC
SRR1266957.1004692.2+    ATCAAGAGAATGCTCTTCCTCTTGTTCCTCCCATGCT
SRR1269689.12646918.2-   ATCAAGAGAATGCTCTTCCTCTTGTTCCTCCCATGCT
SRR1269727.8305397.1+    ATCAAGAGAATGCTCTTCCTCTTGTTCCTCCCATGCT
SRR1271641.5711711.1+    ATCAAGAGAATGCTCTTCCTCTTGTTCCTCCCATGCT
SRR1271644.6211925.2+    ATCAAGAGAATGCTCTTCCTCTTGTTCCTCCCATGCT
SRR1271624.16956133.2-   ATCAAGAGAATGCTCTTCCTCTTGTTCCTCCCATGCTT
SRR1271636.22676106.2-   ATCAAGAGAATGCTCTTCCTCTTGTTCCTCCCATGCTT
SRR1265419.9760671.2+    ATCAAGAGAATGCTCTTCCTCTTGTTCCTCCCATGCTTT
SRR1265486.16127387.1-   ATCAAGAGAATGCTCTTCCTCTTGTTCCTCCCATGCTTT
SRR1266957.3246519.1+    ATCAAGAGAATGCTCTTCCTCTTGTTCCTCCCATGCTTT
SRR1271616.8117819.1-    ATGAAGAGAATGCTCTACCTCTTGTTCCTCCCATGCTTTT
SRR1271620.7196430.1-    ATCAAGAGAATGCTCTTCCTCTTGTTCCTCCCATGCTTTT
SRR1271624.5552917.2+    ATCAAGAGAATGCTCTTCCTCTTGTTCCTCCCATGCTTTT
SRR1271641.1828285.2+    ATCAAGAGAATGCTCTTCCTCTTGTTCCTCCCATGCTTTT
SRR1265421.3532440.1-    ATCAAGAGAATGCTCTTCCTCTTGTTCCTCCCATGCTTTTT
SRR1266951.538073.2+     ATCAAGAGAATGCTCTTCCTCTTGTTCCTCCCATGATTTTT
SRR1266961.140544.2-     ATCAAGAGAATGCTCTTCCTCTTGTTCCTCCCATGCTTTTT
SRR1265959.14596287.2+   ATCAAGAGAATGCTCTTCCTCTTGTTCCTCCCATGCTTTTGT
SRR1266948.20090114.2-   ATCAAGAGAATGCTCTTCCTCTTGTTCCTCCCATGCTTTTGT
SRR1265429.4871220.2-    ATCAAGAGAATGCTCTTCCTCTTGTTCCTCCCATGCTTTTGTT
SRR1265430.10056360.2+   ATCAAGAGAATGCTCTTCCTCTTGTTCCTCCCATGCTTTTGTT
SRR1271612.777884.2-     ATCAAGAGAATGCTCTTCCTCTTGTTCCTCCCATGCTTTTGTTC
SRR1265485.4295901.1+    ATCAAGAGAATGCTCTTCCTCTTGTTCCTCCCATGCTTTTGTTCT
SRR1266953.6459693.2-    ATCAAGAGAATGCTCTTCCTCTTGTTCCTCCCATGCTTTTTTTCT
SRR1266955.9340457.2+    ATCAAGAGAATGCTCTTCCTCTTGTTCCTCCCATGCTTTTGTTCT
SRR1266960.7618362.2+    ATCAAGAGAATGCTCTTCCTCTTGTTCCTCCCATGCTTTTGTTCT
SRR1266960.863619.1+     ATCAAGAGAATGCTCTTCCTCTTGTTCCTCCCATGCTTTTTTTCT
SRR1271611.51705152.2+   ATCAAGAGAATGCTCTTCCTCTTGTTCCTCCCATGCTTTTGTTCT
SRR1271620.10933616.1+   ATCAAGAGAATGCTCTTCCTCTTGTTCCTCCCATGCTTTTGTTCT
SRR1271626.5381257.2+    ATCAAGAGAATGCTCTTCCTCTTGTTCCTCCCATGCTTTTGTTCT
SRR1266961.10240545.2-   ATCAAGAGAATTCTCTTCCTCTTGTTCCTCCCATGCCTTTGTTCTG
SRR1266962.1283028.2-    ATCAAGAGAATGCTCTTCCTCTTCTTCCTCCCATGCTTTTGTTCTG
SRR1271656.21063060.2+   ATCAAGAGAATGCTCTTCCTCTTGTTCCTCCCATGCTTTTTTTCTG
SRR1265961.21125338.1+   ATCAAGAGAATGCTCTTCCTCTTGTTCCTCCCATGCTTTTGTTCTGG
SRR1266964.9037991.2-    ATCAAGAGAATGCTCTTCCTCTTGTTCCTCACATGCTTTTGTTCTGG
SRR1271617.107030387.1-  ATCAAGAGAATGCTCTTCCTCTTGTTCCTCCCATGCTTTTGTTCTGG
SRR1271664.9315059.2+    ATCAAGAGAATGCTCTTCCTCTTGTTCCTCCCATGCTTTTGTTCTGG
SRR1265108.11035394.1+   ATCAAGAGAATGCTCTTCCTCTTGTTCCTCCCATGCTTTTGTTCTGGC
SRR1265425.8225295.2+    ATCAAGAGAATGCTCTTCCTCTTGTTCCTCCCATGCTTTTGTTCTGGC
SRR1269700.383429.2+     ATCAAGAGAATGCTCTTCCTCTTGTTCCTCCCATGCTTTTTTTCTGGC
SRR1271626.30577518.1-   ATCAAGAGAATGCTCTTCCTCTTGTTCCTCCCATGCTTTTTTTCTGGC
SRR1271635.2818304.1-    ATCAAGAGAATGCTCTTCCTCTTGTTCCTCCCATGCTTTTTTTCTGGC
SRR1265492.3432458.1+    ATCAAGAGAATGCTCTTCCTCTTGTTCCTCCCATGCTTTTTTTCTGGCC
SRR1266954.5286348.2-    ATCAAGAGAATGCTCTNCCTCTTGTTCCTCCCATGCTTTTGTTCTGGCC
SRR1271631.18001001.2-   ATCAAGAGAATGCTCTTCCTCTTGTTCCTCCCATGCTTTTGTTCTGGCC
SRR1265421.25226457.2+   ATCAAGAGAATGCTCTTCCTCTTGTTCCTCCCATGCTTTTGTTCTGGCCA
SRR1266951.9077736.1-    ATCAAGAGAATGCTCTTCCTCTTGTTCCTCCCATGCTTTTTTTCTGGCCA
SRR1266959.5837465.2+    ATCAAGAGAATGCTCTTCCTCTTGTTCCTCCCATGCTTTTTTTCTGGCCA
SRR1271617.13985324.1-   ATCAAGAGAATGCTCTTCCTCTTGTTCCTCCCATGCTTTTGTTCTGGCCA
SRR1265960.10540450.1+   ATCAAGAGAATGCTCTTCCTCTTGTTCCTCCCATGCTTTTGTTCTGGCCAGC
SRR1269723.820781.2+     ATCAAGAGAATGCGCTTCCTCTTGTTCCTCCCATGCTTTTTTTCTGGCCAGC
SRR1271606.5849136.2+    CTCAAGAGAATGCTCTTCCTCTTGTTCCTCCCATGCTTTTGTTCTGGCCAGC
SRR1265120.5268240.2+    ATCAAGAGAATGCTCTTCCTCTTCTTCCTCCCATGCTTTTTTTCTGGCCAGCT
SRR1271605.16567794.1+   ATCAAGAGAATGCTCTTCCTCTTGTTCCTCCCATGCTTTTGTTCTGACCAGCT
SRR1266966.13263794.2-   ATCAAGAGAATGCTCTTCCTCTTGTTCCTCCCATGCTTTTGTTCTGGCCAGCTT
SRR1269721.8669041.1+    ATCAAGAGAATGCTCTTCCTCTTGTTCCTCCCATGCTTTTTTTCTGGCCAGCTT
SRR1271656.20184311.1+   ATCAAGAGAATGCTCTTCCTCTTGTTCCTCCCATGCTTTTTTTCTGGCCAGCTT
SRR1271656.31545570.2+   ATCAAGAGAATGCTCTTCCTCTTGTTCCTCCCATGCTTTTTTTCTGGCCAGCTT
SRR1271611.40265863.2+   ATCAAGAGAATGCTCTTCCTCTTGTTCCTCCCATGCTTTTTTTCTGGCCAGCTTG
SRR1271641.8238182.2+    ATCAAGAGAATGCTCTTCCTCTTGTTCCTCCCATGCTTTTTTTCTGGCCAGCTTGC
SRR1271665.16214124.1+   ATCAAGAGAATGCTCTTCCTCTTGTTCCTCCCATGCTTTTGTTCTGGCCAGCTTGC
SRR1271631.53790917.2+   ATCAAGAGAATGCTCTTCCTCTTGTTCCTCCCATGCTTTTGTTCTGGCCAGCTTGCA
SRR1271636.20236431.2+   ATCAAGAGAATGCTCTTCCTCTTGTTCCTCCCATGCTTTTTTTCTGGCCAGCTTGCA
SRR1265424.13096215.1+   ATCAAGAGAATGCTCTTCCTCTTGTTCCTCCCATGCTTTTTTTCTGGCCAGCTTGCAC
SRR1269694.10704014.2-   ATCAAGAGAATGCTCTTCCTCTTGTTCCTCCCATGCTTTTTTTCTGGCCAGCTTGCAC
SRR1271618.4133814.1-    ATCAAGAGAATGCTCTTCCTCTTGTTCCTCCCATGCTTTTGTTCTGGCCAGCTTGCAC
SRR1271631.61406029.2+   ATCAAGAGAATGCTCTTCCTCTTGTTCCTCCCATGCTTTTTTTCTGGCCAGCTTGCAC
```

SRR1271611.1989144.2+   ATCAAGAGAATGCTCTTCCTCTTGTTCCTCCCATGCTTTTGTTCTGGCCAGCTTGCACC
SRR1271626.12852273.2-   ATCAAGAGAATGCTCTTCCTCTTGTTCCTCCCATGCTTTTTTTCTGGCCAGCTTGCACC
SRR1271626.6889032.2-   ATCAAGAGAATGCTCTTCCTCTTGTTCCTCCCATGCTTTTGTTCTGGCCAGCTTGCACC
SRR1271637.7730229.1+   ATCAAGAGAATGCTCTTCCTCTTGTTCCTCCCATGCTTTTTTTCTGGCCAGCTTGCACC
SRR1265090.6845871.1+   ATCAAGAGAATGCTCTTCCTCTTGTTCCTCCCATGCTTTTTTTCTGGCCAGCTTGCACCT
SRR1265093.9367806.1+   ATCAAGAGAATGCTCTTCCTCTTGTTCCTCCCATGCTTTTGTTCTGGCCAGCTTGCACCT
SRR1265094.4259059.2+   ATCAAGAGAATGCTCTTCCTCTTGTTCCTCCCATGCTTTTTTTCTGGCCAGCTTGCACCT
SRR1265100.8844830.1-   ATCAAGAGAATGCTCTTCCTCTTGTTCCTCCCATGCTTTTTTTCTGGCCAGCTTGCACCT
SRR1265101.6853887.1-   ATCAAGAGAATGCTCTTCCTCTTGTTCCTCCCATGCTTTTTTTCTGGCCAGCTTGCACCT
SRR1265102.11716845.1+   ATCAAGAGAATGCTCTTCCTCTTGTTCCTCCCATGCTTTTTTTCTGGCCAGCTTGCACCT
SRR1265102.5685651.2+   ATCAAGAGAATGCTCTTCCTCTTGTTCCTCCCATGCTTTTTTTCTGGCCAGCTTGCACCT
SRR1265103.11096904.1+   ATCAAGAGAATGCTCTTCCTCTTGTTCCTCCCATGCTTTTTTTCTGGCCAGCTTGCACCT
SRR1265105.2962579.1-   ATCAAGAGAATGCTCTTCCTCTTGTTCCTCCCATGCTTTTGTTCTGGCCAGCTTGCACCT
SRR1265106.6015462.2+   ATCAAGAGAATGCTCTTCCTCTTGTTCCTCCCATGCTTTTGTTCTGGCCAGCTTGCACCT
SRR1265106.6372305.2-   ATCAAGAGAATGCTCTTCCTCTTGTTCCTCCCATGCTTTTGTTCTGGCCAGCTTGCACCT
SRR1265107.8255291.2-   ATCAAGAGAATGCTCTTCCTCTTGTTCCTCCCATGCTTTTGTTCTGGCCAGCTTGCACCT
SRR1265108.2515604.1+   ATCAAGAGAATGCTCTTCCTCTTGTTCCTCCCATGCTTTTGTTCTGGCCAGCTTGCACCT
SRR1265118.9445284.1+   ATCAAGAGAATGCTCTTCCTCTTGTTCCTCCCATGCTTTTTTTCTGGCCAGCTTGCACCT
SRR1265418.1061795.1-   ATCAAGAGAATGCTCTTCCTCTTGTTCCTCCCATGCTTTTTTTCTGGCCAGCTTGCACCT
SRR1265419.2098789.1+   ATCAAGAGAATGCTCTTCCTCTTGTTCCTCCCATGCTTTTGTTCTGGCCAGCTTGCACCT
SRR1265420.9704864.2+   ATCAAGAGAATGCTCTTCCTCTTGTTCCTCCCATGCTTTTTTTCTGGCCAGCTTGCACCT
SRR1265421.11935315.2-   ATCAAGAGAATGCTCTTCCTCTTGTTCCTCCCATGCTTTTTTTCTGGCCAGCTTGCACCT
SRR1265422.11840911.2+   ATCAAGAGAATGCTCTTCCTCTTGTTCCTCCCATGCTTTTTTTCTGGCCAGCTTGCACCT
SRR1265423.4185373.1-   ATCAAGAGAATGCTCTTCCTCTTGTTCCTCCCATGCTTTTGTTCTGGCCAGCTTGCACCT
SRR1265423.6251649.1-   ATCAAGAGAATGCTCTTCCTCTTGTTCCTCCCATGCTTTTGTTCTGGCCAGCTTGCACCT
SRR1265423.845833.1+   ATCAAGAGAATGCTCTTCCTCTTGTTCCTCCCATGCTTTTGTTCTGGCCAGCTTGCACCT
SRR1265424.11995558.2+   ATTAAGCGAATGCTCTTCTTCTTGCTCCTCCCATGCTTTTTTTCTGCTCAGCTTGCACCT
SRR1265424.15339550.2-   ATCAAGAGAATGCTCTTCCTCTTGCTCCTCCCATGCTTTTGTTCTGGCCAGCTTGCACCT
SRR1265424.9055674.2-   ATCAAGAGAATGCTCTTCCTCTTGTTCCTCCCATGCTTTTGTTCTGGCCAGCTTGCACCT
SRR1265425.678706.1+   ATCAAGAGAATGCTCTTCCTCTTGTTCCTCCCATGCTTTTTTTCTGGCCAGCTTGCACCT
SRR1265429.3960386.1-   ATCAAGAGAATTCTCTTCCTCTTGGTCCTCCCATGCTTTTTTTCTGGCCAGCTTGCACCT
SRR1265429.662140.1-   ATCAAGAGAATGCTCTTCCTCTTGTTCCTCCCATGCTTTTTTTCTGGCCAGCTTGCACCT
SRR1265485.10372300.1+   ATCAAGAGAATGCTCTTCCTCTTGTTCCTCCCATGCTTTTGTTCTGGCCAGCTTGCACCT
SRR1265485.16069283.1-   ATCAAGAGAATGCTCTTCCTCTTGTTCCTCCCATGCTTTTGTTCTGGCCAGCTTGCACCT
SRR1265485.1858508.1-   ATCAAGAGAATGCTCTTCCTCTTGTTCCTCCCATGCTTTTGTTCTGGCCAGCTTGCACCT
SRR1265485.8142050.2-   ATCAAGAGAATGCTCTTCCTCTTGTTCCTCCCATGCTTTTTTTCTGGCCAGCTTGCACCT
SRR1265486.1000359.1-   ATCAAGAGAATGCTCTTCCTCTTGTGCCTCCCATGCTTTTTTTCTGGCCAGCTTGCACCT
SRR1265486.10320482.1-   ATCAAGAGAATGCTCTTCCTCTTGTTCCTCCCATGCTTTTGTTCTGGCCAGCTTGCCCCT
SRR1265487.11821367.2+   ATCAAGAGAATGCTCTTCCTCTTGTTCCTCCCATGCTTTTTTTCTGGCCAGCTTGCACCT
SRR1265487.14014666.1+   ATCAAGAGAATGCTCTTCCTCTTGTTCCTCCCATGCTTTTGTTCTGGCCAGCTTGCACCT
SRR1265493.1938206.2-   ATCAAGAGAATGCTCTTCCTCTTGTTCCTCCCATGCTTTTTTTCTGGCCAGCTTGCACCT
SRR1265493.882472.2+   ATCAAGAGAATGCTCTTCCTCTTGTTCCTCCCATGCTTTTTTTCTGGCCAGCTTGCACCT
SRR1265494.4458321.1-   ATCAAGAGAATGCTCTTCCTCTTGTTCCTCCCATGCTTTTTTTCTGGCCAGCTTGCACCT
SRR1265959.15507634.1-   ATCAAGAGAATGCTCTTCCTCTTGTTCCTCCCATGCTTTTGTTCTGGCCAGCTTGCACCT
SRR1265959.5289237.2+   ATCAAGAGAATGCTCTTCCTCTTGTTCCTCCCATGCTTTTGTTCTGGCCAGCTTGCACCT
SRR1265960.14555341.2+   ATCAAGAGAATGCTCTTCCTCTTGTTCCTCCCATGCTTTTGTTCTGGCCAGCTTGCACCT
SRR1265960.20313739.2-   ATCAAGAGAATGCTCTTCCTCTTGTTCCTACCATGCTTATGTTCTGGCCAGCTTGCACCT
SRR1265960.4362410.1+   ATCAAGAGAATGCTCTTCCTCTTGTTCCTCCCATGCTTTTGTTCTGGCCAGCTTGCACCT
SRR1265961.2477855.1+   ATCAAGAGAATGCTCTTCCTCTTGTTCCTCCCATGCTTTTGTTCTGGCCAGCTTGCACCT
SRR1265962.4168719.2-   ATCAAGAGAATGCTCTTCCTCTTGTTCCTCCCATGCTTTTGTTCTGGCCAGCTTGCACCT
SRR1266946.18245078.1-   ATCAAGAGAATGCTCTTCCTCTTGTTCCTCCCATGCTTTTTTTCTGGCCAGCTTGCACCT
SRR1266947.14185884.1+   ATCAAGAGAATGCTCTTCCTCTTGTTCCTCCCATGCTTTTTTTCTGGCCAGCTTGCACCT
SRR1266947.19802521.2+   ATCAAGAGAATGCTCTTCCTCTTGTTCCTCCCATGCTTTTGTTCTGGCCAGCTTGCACCT
SRR1266948.14467911.1+   ATCAAGAGAATGCTCTTCCTCTTGTTCCTCCCATGCTTTTGTTCTGGCCAGCTTGCACCT
SRR1266948.8806088.2+   ATCAAGAGAATGCTCTTCCTCTTGTTCCTCCCATGCTTTTGTTCTGGCCAGCTTGCACCT
SRR1266950.19035200.2+   ATCAAGAGAATGCTCTTCCTCTTGTTCCTCCCATGCTTTTTTTCTGGCCAGCTTGCACCT
SRR1266951.18466248.1-   ATCAAGAGAATTCTCTTCCTCTTGTTCCTCCCATGCTTTTTTTCTGGCCAGCTTGCGCCT
SRR1266952.14069486.2+   ATCAAGAGAATGCTCTTCCTCTTGTTCCTCCCATGCTTTTTTTCTGGCCAGCTTGCACCT
SRR1266952.5817620.2-   ATCAAGAGAATGCTCTTCCTCTTGTTCCTCCCATGCTTTTTTTCTGGCCAGCTTGCACCT
SRR1266953.271055.1-   ATCAAGAGAATGCTCTTCCTCTTGTTCCTCCCATGCTTTTTTTCTGGCCAGCTTGCACCT
SRR1266954.10031696.1-   ATCAAGAGAATGCTCTTCCTCTTGTTCCTCCCATGCTTTT-TTCTGGCCAGCTTGCACCT
SRR1266956.13063849.1+   ATCAAGAGAATGCTCTTCCTCTTGTTCCTCCCATGCTTTTGTTCTGGCCAGCTTGCACCT
SRR1266956.8774588.1-   ATCAAGAGAATGCTCTTCCTCTTGTTCCGCCCATGCTTTTGTTCTGGCCAGCTTGCACCT
SRR1266958.1653120.2-   ATCAAGAGAATGCTCTTCCTCTTGTTCCTCCCATGCTTTTGTTCTGGCCAGCTTGCACCT
SRR1266960.6717190.2-   ATCAAGAGAATGCTCTTCCTCTTGTTCCTCCCATGCTTTTTTTCTGGCCAGCTTGCACCT
SRR1266961.9230787.2-   ATCAAGAGAATGCTCTTCCTCTTGTTCCTCCCATGCTTTTTTTCTGGCCAGCTTGCACCT
SRR1266962.11554845.2+   ATCAAGAGAATGCTCTTCCTCTTGTTCCTCCCATGCTTTTTTTCTGGCCAGCTTGCACCT
SRR1266962.4122587.1+   ATCAAGAGAATGCTCTTCCTCTTGTTCCTCCCATGCTTTTGTTCTGGCCAGCTTGCACCT
SRR1266962.7593491.1+   ATCAAGAGAATGCTCTTCCTCTTGTTCCTCCCATGCTTTTGTTCTGGCCAGCTTGCACCT
SRR1266965.13605504.1-   ATCAAGAGAATGCTCTTCCTCTTGTTCCTCCCATGCTTTTTTTCTGGCCAGCTTGCACCT
SRR1266966.437734.2+   ATCAAGAGAATGCTCTTCCTCTTGTTCCTCCCATGCTTTTGTTCTGGCCAGCTTGCCCCT
SRR1269690.5523621.2-   ATCAAGAGAATGCTCTTCCTCTTGTTCCTCCCATGCTTTTTTTCTGGCCAGCTTGCACCT
SRR1269690.6866648.2+   ATCAAGAGAATGCTCTTCCTCTTGTTCCTCCCATGCTTTTTTTCTGGCCAGCTTGCACCT
SRR1269691.1086423.1+   ATCAAGAGAATGCTCTTCCTCTTGTTCCTCCCATGCTTTTTTTCTGGCCAGCTTGCACCT
SRR1269695.4570879.2-   ATCAAGAGAATGCTCTTCCTCTTGTTCCTCCCATGCTTTTGTTCTGGCCAGCTTGCACCT
SRR1269696.1594481.2+   ATCAAGAGAATGCTCTTCCTCTTGTTCCTCCCATGCTTTTTTTCTGGCCAGCTTGCACCT
SRR1269696.7386843.1-   ATCAAGAGAATGCTCTTCCTCTTGTTCCTCCCATGCTTTTGTTCTGGCCAGCTTGCACCT
SRR1269700.1764719.1+   ATCAAGAGAATGCTCTTCCTCTTGTTCCTCCCATGCTTTTTTTCTGGCCAGCTTGCACCT
SRR1269701.5896733.1+   ATCAAGAGAATGCTCTTCCTCTTGTTCCTCCCATGCTTTTTTTCTGTCCAGCTTGCACCT
SRR1269720.6697396.2+   ATCAATAGAATGCTCTTCCTCTTTCTCCTCCCATTCTTTTTTTCTGGCCAGCTTGAACCT
SRR1269721.13769927.2-   ATCAAGAGAAGGCTCTTCCTCGTGTTCCTCCCAGGCTTTTTTTCTGGCCAGCCTGCGCCT
SRR1269723.7787279.2+   ATCAAGAGAATGCTCTTCCTCTTGTTCCTCCCATGCTTTTTTTCTGGCCAGCTTGCACCT
SRR1269726.363820.1+   ATCAAGAGAATGCTCTTCCTCTTGTTCCTCCCATGCTTTTTTTCTGGCCAGCTTGCACCT
SRR1271605.649185.1+   ATCAAGAGAATGCTCTTCCTCTTGTTCCTCCCATGCTTTTGTTCTGGCCAGCTTGCACCT

```
SRR1271609.1031300.1+    ATCAAGAGAATGCTCTTCCTCTTGTTCCTCCCATGCTTTTTTTCTGGCCAGCTTGCACCT
SRR1271609.4569674.1-    ATCAAGAGAATGCTCTTCCTCTTGTTCCTCCCATGCTTTTTTTCTGGCCAGCTTGCACCT
SRR1271610.5829611.1+    ATCAAGAGAATGCTCTTCCTCTTGTTCCTCCCATGCTTTTGTTCTGGCCAGCTTGCACCT
SRR1271611.10364010.1+   ATCAAGAGAATGCTCTTCCTCTTGTTCCTCCCATGCTTTTGTTCTGGCCAGCTTGCACCT
SRR1271611.16853764.2+   ATCAAGAGAATGCTCTTCCTCTTGTTCCTCCCATGCTTTTGTTCTGGCCAGCTTGCACCT
SRR1271611.18839548.2-   ATCAAGAGAATGCTCTTCCTCTTGTTCCTCCCATGCTTTTGTTCTGGCCAGCTTGCACCT
SRR1271611.24840466.2-   ATCAAGAGAATGCTCTTCCTCTTGTTCCTCCCATGCTTTTGTTCTGGCCAGCTTGCACCT
SRR1271611.30562393.2+   ATCAAGAGAATGCTCTTCCTCTTGTTCCTCCCATGCTTTTTTTCTGGCCAGCTTGCACCT
SRR1271611.3494094.2-    ATCAAGAGAATGCTCTTCCTCTTGTTCCTCCCATGCTTTGGTTCTGGCCAGCTTGCACCT
SRR1271611.38267951.1-   ATCAAGAGAATGCTCTTCCTCTTGTTCCTCCCATGCTTTTGTTCTGGCCAGCTTGCACCT
SRR1271611.5176815.1+    ATCAAGAGAATGCTCTTCCTCTTGTTCCTCCCATGCTTTTGTTCTGGCCAGCTTGCACCT
SRR1271611.54109106.1-   ATCAAGAGAATGCTCTTCCTCTTGTTCCTCCCATGCTTTTTTTCTGGCCAGCTTGCACCT
SRR1271614.6562104.2-    ATCAAGAGAATGCTCTTCCTCTTGTTCCTCCCATGCTTTTGTTCTGGCCAGCTTGCACCT
SRR1271616.2011111.2+    ATCAAGAGAATGCTCTTCCTCTTGTTCCTCCCATGCTTTTGTTCTGGCCAGCTTGCACCT
SRR1271617.106361713.2+  ATCAAGAGAATGCGCTTCCTCTTGTTCCTCCCATGCTTTTGTTCTGGCCAGCTTGCACCT
SRR1271617.107171253.2-  ATCAAGAGAATGCTCTTCCTCTTGTTCCTCCCATGCTTTTGTTCTGGCCAGCTTGCACCT
SRR1271617.21164005.1+   ATCAAGAGAATGCTCTTCCTCTTGTTCCTCCCATGCTTTTGTTCTGGCCAGCTTGCACCT
SRR1271617.35068629.2+   ATCAAGAGAATGCTCTTCCTCTTGTTCCTCCCATGCTTTTGTTCTGGCCAGCTTGCACCT
SRR1271617.65162719.1+   ATCAAGAGAATGCTCTTCCTCTTGTTCCTCCCATGCTTTTGTTCTGGCCAGCTTGCACCT
SRR1271617.93090910.1+   ATCAAGAGAATGCTCTTCCTCTTGTTCCTCCCATGCTTTTGTTCTGGCCAGCTTGCACCT
SRR1271618.10995740.1-   ATCAAGAGAATGCTCTTCCTCTTGTTCCTCCCATGCTTTTGTTCTGGCCAGCTTGCACCT
SRR1271618.11716789.1-   ATCAAGAGAATGCTCTTCCTCTTGTTCCTCCCATGCTTTTGTTCTGGCCAGCTTGCACCT
SRR1271620.2068946.1-    ATCAAGAGAATGCTCTTCCTCTTGTTCCTCCCATGCTTTTGTTCTGGCCAGCTTGCACCT
SRR1271620.711931.1-     ATCAAGAGAATGCTCTTCCTCTTGTTCCTCCCATGCTTTTGTTCTGGCCAGCTTGCACCT
SRR1271621.3138183.1+    ATCAAGAGAATGCTCTTCCTCTTGTTCCTCCCATGCTTTTGTTCTGGCCAGCTTGCACCT
SRR1271622.14382387.1+   ATCAAGAGAATGCTCTTCCTCTTGTTCCTCCCATGCTTTTTTTCTGGCCAGCTTGCACCT
SRR1271622.14489347.2+   ATCAAGAGAATGCTCTTCCTCTTGTTCCTCCCATGCTTTTGTTCTGGCCAGCTTGCACCT
SRR1271624.3306484.1+    ATCAAGAGAATGCTCTTCCTCTTGTTCCTCCCATGCTTTTTTTCTGGCCAGCTTGCACCT
SRR1271625.17771509.2+   ATCAAGAGAATGCTCTTCCTCTTGTTCCTCCCATGCTTTTGTTCTGGCCAGCTTGCACCT
SRR1271626.19282964.2+   ATCAAGAGAATGCTCTTCCTCTTGTTCCTCCCATGCTTTTGTTCTGGCCAGCTTGCACCT
SRR1271626.27417879.2+   ATCAAGAGAATGCTCTTCCTCTTGTTCCTCCCATGCTTTTGTTCTGGCCAGCTTGCACCT
SRR1271626.35055944.1-   ATCAAGAGAATGCTCTTCCTCTTGTTCCTCCCATGCTTTTTTTCTGGCCAGCTTGCACCT
SRR1271626.3653174.1-    ATCAAGAGAATGCTCTTCCTCTTGTTCCTCCCATGCTTTTTTTCTGGCCAGCTTGCACCT
SRR1271627.4158392.1-    ATCAAGAGAATGCTCTGCCTCTTGTTCCTCCCATGCTTTTTTTCTGGCCAGCTTGCACCT
SRR1271627.6371503.1-    ATCAAGAGAATGCTCTTCCTCTTGTTCCTCCCATGCTTTTGTTCTGGCCAGCTTGCACCT
SRR1271627.817121.1-     ATCAAGAGAATGCTCTTCCTCTTGTTCCTCCCATGCTTTTTTTCTGGCCAGCTTGCACCT
SRR1271629.12769352.1-   ATCAAGAGAATGCTCTTCCTCTTGTTCCTCCCATGCTTTTTTTCTGGCCAGCTTGCACCT
SRR1271629.8867623.1-    ATCAAGAGAATGCTCTTCCTCTTGTTCCTCCCATGCTTTTGTTCTGGCCAGCTTGCACCT
SRR1271630.3176113.1+    ATCAAGAGAATGCTCTTCCTCTTGTTCCTCCCATGCTTTTGTTCTGGCCAGCTTGCACCT
SRR1271631.12525033.2+   ATCAAGAGAATGCTCTTCCTCTTGTTCCTCCCATGCTTTTTTTCTGGCCAGCTTGCACCT
SRR1271631.13703327.2+   ATCAAGAGAATGCTCTTCCTCTTGTTCGTCCCATGCTTTTGTTCTGGCCAGCTTGCACCT
SRR1271631.15064325.1-   ATCAAGAGAATGCTCTTCCTCTTGTTCCTCCCATGCTTTTTTTCTGGCCAGCTTGCACCT
SRR1271631.17796850.1+   ATCAAGAGAATGCTCTTCCTCTTGTTCCTCCCATGCTTTTGTTCTGGCCAGCTTGCACCT
SRR1271631.56849156.1+   ATCAAGAGAATGCTCTTCCTCTTGTTCCTCCCATGCTTTTGTTCTG-CCAGCTTGCACCT
SRR1271631.66929254.2-   ATCAAGAGAATGCTCTTCCTCTTGTTCCTCCCATGCTTTTGTTCTGGCCAGCTTGCACCT
SRR1271631.70375615.1-   ATCAAGAGAATGCTCTTCCTCTTGTTCCTCCCATGCTTTTTTTCTGGCCAGCTTGCACCT
SRR1271631.73182052.1+   ATCAAGAGAATGCTCTTCCTCTTGTTCCTCCCATGCTTTTGTTCTGGCCAGCTTGCACCT
SRR1271631.92663139.1+   ATCAAGAGAATGCTCTTCCTCTTGTTCCTCCCATGCTTTTTTTCTGGCCAGCTTGCACCT
SRR1271635.1018397.2+    ATCAAGAGAATGCTCTTCCTCTTGTTCCTCCCATGCTTTTGTTCTGGCCAGCTTGCACCT
SRR1271635.4911972.1-    ATCAAGAGAATGCTCTTCCTCTTGTTCCTCCCATGCTTTTGTTCTGGCCAGCTTGCACCT
SRR1271637.6571114.2-    ATCAAGAGAATTCTCTTCCTCTTGTTCCTCCCATGCTTTTTTTCTGGCCAGCTTGCACCT
SRR1271638.7137901.1+    ATCAAGAGAATGCTCTTCCTCTTGTTCCTCCCATGCTTTTTTTCTGGCCAGCTTGCACCT
SRR1271639.12989136.2+   ATCAAGAGAATGCTCTTCCTCTTGTTCCTCCCATGCTTTTGTTCTGGCCAGCTTGCACCT
SRR1271639.5769975.1-    ATCAAGAGAATGCTCTTCCTCTTGTTCCTCCCATGCTTTTTTTCTGGCCAGCTTGCACCT
SRR1271639.961470.1-     ATCAAGAGAATGCTCTTCCTCTTGTTCCTCCCATGCTTTTTTTCTGGCCAGCTTGCACCT
SRR1271640.2387601.1-    ATCAAGAGAATGCTCTTCCTCTTGTTCCTCCCATGCTTTTTTTCTGGCCAGCTTGCACCT
SRR1271642.1942769.2-    ATCAAGATAATGCTCTTCCTCTTGTTCCTCCCATGCTATTTTGCTGGCCAGCTTGCACCT
SRR1271642.3577506.2-    ATCAAGAGAATGCTCTTCCTCTTGTTCCTCCCATGCTTTTTTTCTGGCCAGCTTGCACCT
SRR1271642.4563788.2-    ATCAAGAGAATGCTCTTCCTCTTGTTCCTCCCATGCTTTTTTTCTGGCCAGATTGCACCT
SRR1271643.6174127.1+    ATCAAGAGAATGCTCTTCCTCTTGTTCCTCCCATGCTTTTTTTCTGGCCAGCTTGCACCT
SRR1271656.16911801.1-   ATCAAGAGAATGCTCTTCCTCTTGTTCCTCCCATGCTTTTTTTCTGGCCAGCTTGCACCT
SRR1271656.28111822.2+   ATCAAGAGAATGCTCTTCCTCTTGTTCCTCCCATGCTTTTTTTCTGGCCAGCTTGCACCT
SRR1271656.40523025.1-   ATCAAGAGAATGCTCTTCCTCTTGTTCCTCCCATGCTTTTTTTCTGGCCAGCTTGCACCT
SRR1271656.42884483.2+   ATCAAGAGAATGCTCTTCCTCTTGTTCCTCCCATGCTTTTTTTCTGGCCAGCTTGCACCT
SRR1271656.48186978.2-   ATCAAGAGAATGCTCTTCCTCTTGTTCCTCCCATGCTTTTTTTCTGGCCAGCTTGCACCT
SRR1271661.3541586.2+    ATCAAGAGAATGCTCTTCCTCTTGTTCCTCCCATGCTTTTGTTCTGGCCAGCTTGCACCT
SRR1271661.6840898.1-    ATCAAGAGAATGCTCTTCCTCTTGTTCCTCCCATGCTTTTGTTCTGGCCAGCTTGCACCT
SRR1271661.9678304.1+    ATCAAGAGAATGCTCTTCCTCTTGTTCCTCCCATGCTTTTGTTCTGGCCAGCTTGCACCT
SRR1271663.7532180.2-    ATCAAGAGAATGCTCTTCCTCTTTTTCCTCCCATGCTTTTGTTCTGGCCAGCTTGCACCT
SRR1271664.2748803.2-    ATCAAGAGAATGCTCTTCCTCTTGTTCCTCCCATGCTTTTTTTCTGGCCAGCTTGCACCT
SRR1271665.15301974.1-   ATCAAGAGAATGCTCTTCCTCTTGTTCCTCCCATGCTTTTGTTCTGGCCAGCTTGCACCT
SRR1271665.16602572.1+   ATCAAGAGAATGCTCTTCCTCTTGTTCCTCCCATGCTTTTTTTCTGGCCAGCTTGCACCT
SRR1271666.12193922.1-   ATCAAGAGAATGCTCTTCCTCTTGTTCCTCCCATGCTTTTTTTCTGGCCAGCTTGCACCT
SRR1271666.15761999.1-   ATCAAGAGAATGCTCTTCCTCTTGTTCCTCCCATGCTTTTGTTCTGGCCAGCTTGCACCT
SRR1269723.11892874.1+    TCAAGAGAATGCTCTTCCTCTTGTTCCTCCCATGCTTTTTTTCTGGCCAGCTTGCACCT
SRR1265421.10156975.1-      CAAGAGAATGCTCTTCCTCTTGTTCCTCCCATGCTTTTTTTCTGGCCAGCTTGCACCT
SRR1265961.17342409.2+      CAAGAGAATGCTCTTCCTCTTGTTCCTCCCATGCTTTTGTTCTGGCCAGCTTGCACCT
SRR1269722.612784.1+        CAAGAGAATGCTCTTCCTCTTGTTCCTCCCATGCTTTTTTTCTGGCCAGCTTGCACCT
SRR1271616.12361.2+         CAAGAGAATGCTCTTCCTCTTGTTCCTCCCATGCTTTTGTTCTGGCCAGCTTGCACCT
SRR1271631.2131030.2+       CAAGAGAATGCTCTTCCTCTTGTTCCTCCCATGCTTTTTTTCTGGCCAGCTTGCACCT
SRR1271637.2457105.1-       CAAGAGAATGCTCTTCCTCTTGTTCCTCCCATGCTTTTGTTCTGGCCAGCTTGCACCT
SRR1266960.8454366.2+        AAGAGAATGCTCTTCCTCTTGTTCCTCCCATGCTTTTTTTCTGGCCAGCTTGCACCT
SRR1269691.6982732.2+        AAGAGAATGCTCTTCCTCTTGTTCCTCCCATGCTTTTTTTCTGGCCAGCTTGCACCT
SRR1269703.1669213.2+        AAGAGAATGCTCTTCCTCTTGTTCCTCCCATGCTTTTTTTCTGGCCAGCTTGCACCT
```

```
SRR1265103.11542175.1+       AGAGAATGCTCTTCCTCTTGTTCCTCCCATGCTTTTTTTCTGGCCAGCTTGCACCT
SRR1269721.3127720.1-        AGAGAATGCTCTTCCTCTTGTGCCTCCCATGCTTTTTTTCTGGCCAGCTTGCACCT
SRR1271612.6153622.1+        AGAGAATGCTCTTCCTCTTGTTCCTCCCATGCTTTTGTTCTGGCCAGCTTGCACCT
SRR1271617.49734526.1+       AGAGAATGCTCTTCCTCTTGTTCCTCCCATGCTTTTGTTCTGGCCAGCTTGCACCT
SRR1271624.1867358.2+        AGAGAATGCTCTTCCTCTTGTTCCTCCCATGCTTTTGTTCTGGCCAGCTTGCACCT
SRR1266947.868250.2+          GAGAATGCTCTTCCTCTTGTTCCTCCCATGCTTTTTTTCTGGCCAGCTTGCACCT
SRR1271636.20102713.1-        GAGAATGCTCTTCCTCTTGTTCCTCCCATGCTTTTTTTCTGGCCAGCTTGCACCT
SRR1265116.2223311.1-          AGAATGCTCTTCCTCTTGTTCCTCCCATGCTTTTTTTCTGGCCAGCTTGCACCT
SRR1265420.9541940.2+          AGAATGCTCTTCCTCTTGTTCCTCCCATGCTTTTGTTCTGGCCAGCTTGCACCT
SRR1265423.5540826.1+          AGAATGCTCTTCCTCTTGTTCCTCCCATGCTTTTGTTCTGGCCAGCTTGCACCT
SRR1266954.13274081.2+         AGAATGCTCTTCCTCTTGTTCCTCCCATGCTTTTTTTCTGGCCAGCTTGCACCT
SRR1271626.5495113.1+          AGAATGCTCTTCCTCTTGTTCCTCCCATGCTTTTTTTCTGGCCAGCTTGCACCT
SRR1271629.12293870.2-         AGAATGCTCTTCCTCTTGTTCCTCCCATGCTTTTTTTCTGGCCAGCTTGCACCT
SRR1271644.2407654.1-          AGAATGCTCTTCCTCTTGTTCCTCCCATGCTTTTTTTCTGGCCAGCTTGCACCT
SRR1271664.496248.1+            GAATGCTCTTCCTCTTGTTCCTCCCATGCTTTTGTTCTGGCCAGCTTGCACCT
SRR1265492.11023875.2+           AATGCTCTTCCTCTTGTTCCTCCCATGCTTTTTTTCTGGCCAGCTTGCACCT
SRR1271620.8170287.2+            AATGCTCTTCCTCTTGTTCCTCCCATGCTTTTGTTCTGGCCAGCTTGCACCT
SRR1271626.25836168.2-           AATTCTCTTCCTCTTGTTCCTCCCATGCTTTTGTTCTGGCCAGCTTGCACCT
SRR1271605.19857960.1-            ATGCTCTTCCTCTTGTTCCTCCCATGCGTTTTTCCTGGCCAGCTTGCACCT
SRR1271617.31861062.2+            ATGCTCTTCCTCTTGTTCCTCCCATGCTTTTGTTCTGGCCAGCTTGCACCT
SRR1271631.13391857.2+            ATGCTCTTCCTCTTGTTCCTCCCATGCTTTTGTTCTGGCCAGCTTGCACCT
SRR1269694.9029316.2-              TGCTCTTCCTCTTGTTCCTCCCATGCTTTTTTTCTGGCCAGCTTGCCCCT
SRR1271639.4180019.1+              TGCTCTTCCTCTTGTTCCTCCCATGCTTTTTTTCTGGCCAGCTTGCACCT
SRR1271611.49730989.1+              GCTCTTCCTCTTGTTCCTCCCATGCTTTTGTTCTGGCCAGCTTGCACCT
SRR1265103.11542175.2-               CTCTTCCTCTTGTTCCTCCCATGCTTTTTTTCTGGCCAGCTTGCACCT
SRR1266950.10231735.2+               CTCTTCCTCTTGTTCCTCCCATGCTTTTTTTCTGGCCAGCTTGCACCT
SRR1266959.358352.2-                 CTCTTCCTCTTGTTCCTCCCATGCTTTTGCTCTGGCCAGCTTGCCCCT
SRR1271618.11957621.2+               CTCTTCCTCTTGTTCCTCCCATGCTTTTGTTCTGGCCAGCTTGCACCT
SRR1271630.6754689.2+                CTCTTCCTCTTGGTCCTCCCATGCTTTTGTTCTGGCCAGCTTGCACCT
SRR1265419.2274158.2+                 TCTTCCTCTTGTTCCTCCCATGCTTTTGTTCTGGCCAGCTTGCACCT
SRR1269696.95546.1-                   TCTTCCTCTTGTTCCTCCCATGCTTTTGTTCTGGCCAGCTTGCACCT
SRR1269698.2485360.2-                 TCTTCCTCTTGTTCCTCCCATGCTTTTGTTCTGGCCAGCTTGCACCT
SRR1269723.12292891.1+                TCTTCCTCTTGTTCCTCCCATGCTTTTTTTCTGGCCAGCTTGCACCT
SRR1271623.14004856.2+                TCTTCCTCTTGTTCCTCCCATGCTTTTGTTCTGGCCAGCTTGCACCT
SRR1265419.9204382.2-                  CTTCCTCTTGTTCTTCCCATGCTTTTGTTCTGGCCAGCTTGCACCT
SRR1265488.2451011.1-                  CTTCCTTTTGTTCCTCCCATGCTTTTTTTCTGGCCAGCTTGCACCT
SRR1265962.6556046.2-                  CTTCCTCTTTTTGCTCCCATGCTTTTGTTCTGGCCAGCTTGCCCCC
SRR1271610.18281570.1+                  TTCCTCTTGTTCCTCCCATGCTTTTTTTCTGGCCAGCTTGCACCT
SRR1265100.6999977.1-                    TCCTCTTGTTCCTCCCATGCTTTTTTTCTGGCCAGCTTGCACCT
SRR1271612.6407329.2+                    TCCTCTTGTTCCTCCCATGCTTTTGTTCTGGCCAGCTTGCACCT
SRR1271660.19233729.1+                   TCCTCTTGTTCCTCCCATGCTTTTGTTCTGGCCAGCTTGCACCT
SRR1265091.8211546.1+                     CCTCTTGTTCCTCCCATGCTTTTTTTCTGGCCAGCTTGCACCT
SRR1265102.1899426.1+                     CCTCTTGTTCCTCCCATGCTTTTTTTCTGGCCAGCTTGCACCT
SRR1265429.12954956.2-                    CCTCNTGTTCCTCCCATGCTTTTGTTCTGGCCAGCTTGCACCT
SRR1266948.400501.2+                      CCTCTTGTTCCTCCCATGCTTTTTTTCTGGCCAGCTTGCACCT
SRR1269700.2113418.1+                     CCTCTTGTTCCTCCCATGCTTTTTTTCTGGCCAGCTTGCACCT
SRR1271621.4075809.1-                     CCTCTTGTTCCTCCCATGCTTTTGTTCTGGCCAGCTTGCACCT
SRR1271628.228721.1+                      CCTCTTGTTCCTCCCATGCTTTTGTTCTGGCCAGCTTGCACCT
SRR1271631.42457297.2-                    CCTCTTGTTCCTCCCATGCTTTTGTTCTGGCCAGCTTGCACCT
SRR1271631.89798142.2+                    CCTCTTGTTCCTCCCATGCTTTTGTTCTGGCCAGCTTGCACCT
SRR1271640.8629898.2+                     CCTCTTGTTCCTCCCATGCTTTTTTTCTGGCCAGCTTGCACCT
SRR1265421.26923308.1+                     CTCTTGTTCCTCCCATGCTTTTGTTCTGGCCAGCTTGCACCT
SRR1266948.7331073.2-                      CTCTTGTTCCTCCCATGCTGTTGTTCTGGCCAGCTTGCACCT
SRR1269694.1056175.1+                       TCTTGTTCCTCCCATGCTTTTTTTCTGGCCAGCTTGCACCT
SRR1269695.5358702.1+                       TCTTGTTCCTCCCATGCTTTTTTTCTGGCCAGCTTGCACCT
SRR1265424.13096215.2-                       CTTGTTCCTCCCATGCTTTTTTTCTGGCCAGCTTGCACCT
SRR1269692.1681865.1-                        CTTGTTCCTCCCATGCTTTTGTTCTGGCCAGCTTGCACCT
SRR1269700.8551703.1+                        CTTGTTCCTCCCATGCTTTTTTTCTGGCCAGCTTGCACCT
SRR1265107.10953111.1+                        TTGTTCCTCCCATGCTTTTGTTCTGGCCAGCTTGCACCT
SRR1265108.4121277.1-                         TTGTTCCTCCCATGCTTTTGTTCTGGCCAGCTTGCACCT
SRR1265422.12487509.2-                         TGTTCCTCCCATGCTTTTTTTCTGGCCAGCTTGCACCT
SRR1271626.8851170.1+                          TGTTCCTCCCATGCTTTTTTTCTGGCCAGCTTGCACCT
SRR1271639.16935460.1+                         TGTTCCTCCCATGCTTTTGTTCTGGCCAGCTTGCACCT
SRR1266954.9977358.1-                           GTTCCTCCCATGCTTTTGTTCTGGCCAGCTTGCCCCT
SRR1266955.12775171.2-                          GTTCCTCCCATGCTTTTTTTCTGGCCAGCTTGCACCT
SRR1266958.3227986.1-                           GTTCCTCCCATGCTTTTGTTCTGGCCAGCTTGCACCT
SRR1265103.4526646.2+                            TTCCTCCCATGCTTTTGTTCTGGCCAGCTTGCACCT
SRR1266947.12981212.1-                           TTCCTCCCATGCTTTTGTTCTGGCCAGCTTGCGCCT
SRR1266953.3952558.2+                            TTCCTCCCATGCTTTTTTTCTGGCCAGCTTGCACCT
SRR1269700.9464373.2+                            TTCCTCCCATGCTTTTTTTCTGGCCAGCTTGCACCT
SRR1271634.8456855.2-                            TTCCTCCCATGCTTTTTTTCTGGCCAGCTTGCACCT
SRR1271639.11541096.1-                           TTCCTCCCATGCTTTTGTTCTGGCCAGCTTGCACCT
SRR1266965.6800008.2-                             TCCTCCCATGCGTTTGTTCTGGCCAGCTTGCACCT
SRR1271617.105521641.1-                           TCCTCCCATGCTTTTGTTCTGGCCAGCTTGCACCT
SRR1269693.1880844.2+                              CCTCCCATGCTTTTGTTCTGGCCAGCTTGCACCT
SRR1271660.11124812.1-                             CCTCCCATGCTTTTGTTCTGGCCAGCTTGCACCT
SRR1271614.6603009.1-                               CTCCCATGCTTTTGTTCTGGCCAGCTTGCACCT
SRR1271641.6014124.1+                               CTCCCATGCTTTTTTTCTGGCCAGCTTGCACCT
SRR1265486.10961790.2-                               TCCCATGCTTTTGTTCTGGCCTGCTTGCCCCT
SRR1266947.22170370.1+                               TCCCATGCTTTTTTTCTGGCCAGCTTGCACCT
SRR1271617.101618594.2+                              TCCCATGCTTTTGTTCTGGCCAGCTTGCACCT
SRR1271617.36691224.1-                               TCCCATGCTTTTGTTCTGGCCAGCTTGCACCT
SRR1271620.12578244.2+                               TCCCATGCTTTTGTTCTGGCCAGCTTGCACCT
SRR1265120.3033993.2+                                 CCCATGCTTTTTTTCTGGCCAGCTTGCACCT
```

```
SRR1271625.13588244.2+                                CCCATGCTTTTTTTCTGGCCAGCTTGCACCT
SRR1265959.16757127.1-                                 CCATGCTTTTGTTCTGGCCAGCTTGCCCCT
SRR1269724.4208407.1+                                  CCATGCTTTTTTTCTGGCCAGCTTGCACCT
SRR1265485.8802153.2-                                   CATGCTTTTGTTCTGGCCAGCTTGCACCT
SRR1269690.1324197.2-                                   CATGCTTTTTTTCTGGCCAGCTTGCACCT
SRR1266955.7154550.1+                                    ATGCTTTTGTTCTGGCCAGCTTGCACCT
SRR1271611.5929085.1+                                    ATGCTTTTGTTCTGGCCAGCTTGCACCT
SRR1271617.46820496.1+                                   ATGCTTTTGTTCTGGCCAGCTTGCACCT
SRR1265107.2088771.2-                                     TGCTTTTGTTCTGGCCAGCTTGCACCT
SRR1266966.1422141.1+                                      GCTTTTTTTCTGGCCAGCTTGCACCT
SRR1271626.29048032.1+                                     GCTTTTGTTCTGGCCAGCTTGCACCT
SRR1271612.6213795.1+                                       CTTTTGTTCTGGCCAGCTTGCACCT
SRR1271636.5617421.2+                                       CTTTTGTTCTGGCCAGCTTGCACCT
SRR1271662.3330309.1+                                       CTTTTGTTCTGGCCAGCTTGCACCT
SRR1265103.8617061.1-                                          TTTTTCTGGCCAGCTTGCACCT
SRR1266957.3666759.1+                                           TTTTCTGGCCAGCTTGCACCT
SRR1271617.8325012.1+                                           TGTTCTGGCCAGCTTGCACCT
SRR1265107.11198169.1+                                           GTTCTGGCCAGCTTGCACCT
SRR1265959.13791605.2+                                           GTTCTGGCCAGCTTGCACCT
SRR1271619.10258847.1-                                           GTTCTGGCCAGCTTGCACCT
SRR1271624.16629241.2-                                           TTTCTGGCCAGCTTGCACCT
SRR1265424.14499483.2-                                            TTCTGGCCAGCTTGCACCT
SRR1266957.1389486.2+                                             TTCTGGCCAGCTTGCACCT
SRR1269721.7475901.1+                                             TTCTGGCCAGCTTGCACCT
SRR1271626.39375321.1-                                            TTCTGGCCAGCTTGCACCT
SRR1271643.2019678.1+                                             TTCTGGCCAGCTTGCACCT
SRR1266957.10574795.2-                                             TCTGGCCAGCTTGCTCCT
SRR1271642.5331247.1+                                              TCTGGCCAGCTTGCACCT
SRR1266965.6865544.1+                                               CTGGCCAGCTTGCACCT
SRR1271611.45328290.1+                                              CTGGCCAGCTTGCACCT
SRR1265107.9116199.1-                                                TGGCCAGCTTGCACCT
SRR1265421.10976887.1+                                                GGCCAGCTTGCACCT
SRR1265421.28186333.1+                                                GGCCAGCTTGCACCT
SRR1265487.10026670.1-                                                GGCCAGCTTGCCCCT
SRR1265487.15510276.2-                                                GGCCAGCTTGCCCCT
SRR1269689.606782.2+                                                  GGCCAGCTTGCACCT
SRR1265493.2249139.2-                                                  GCCAGCTTGCACCT
SRR1266963.4385495.1+                                                  GCCAGCTTGCACCT
SRR1271664.7577323.2+                                                  GCCAGCTTGCACCT
SRR1266950.11307404.1-                                                   CAGCTTGCACCT
SRR1271639.17341141.1+                                                   CAGCTTGCACCT
SRR1265092.2139034.1-                                                     AGCTTGCACCT
SRR1265101.2621422.2-                                                     AGCTTGCACCT
SRR1266960.4673203.1-                                                     AGCTTGCACCT
SRR1269689.10802270.2+                                                    AGCTTGCACCT
SRR1269690.6701197.1+                                                     AGCTTGCACCT
SRR1269691.5900866.2+                                                     AGCTTGCACCT
SRR1269698.4930714.1-                                                     AGCTTGCACCT
SRR1271616.4976712.2+                                                     AGCTTGCACCT
SRR1271618.649812.2+                                                      AGCTTGCACCT
SRR1265102.11843773.1-                                                     GCTTGCACCT
SRR1265421.28387803.2+                                                     GCTTGCACCT
SRR1265427.12651809.2-                                                     GCTTGCCCCT
SRR1265427.6605459.1+                                                      GCTTGCACCT
SRR1271631.56868617.2-                                                     GCTTGCACCT
SRR1266962.4519620.1-                                                        TTGCACCT
SRR1271661.2368772.1+                                                        TTGCACCT
SRR1265424.4163374.2+                                                         TGCACCT
SRR1266949.1088605.1-                                                         TGCCCCT
SRR1269690.8024392.2+                                                         TGCACCT
SRR1271623.12702077.1+                                                        TGCACCT
SRR1271660.66535.1-                                                             CACCT
SRR1265107.6644656.1-                                                            ACCT
SRR1271631.25901633.1+                                                           ACCT
SRR1271644.4072249.1-                                                            ACCT
SRR1265430.10892354.1+                                                            CCT
SRR1266962.2111129.1-                                                             CCT
SRR1269695.709605.2-                                                              CCT
SRR1271605.5228759.1+                                                             CCT
SRR1269695.5539655.2+                                                              CT
SRR1271624.11221728.1-                                                             CT
SRR1265116.6928798.1+                                                               T
SRR1265419.2667566.2-                                                               T
SRR1265488.7425864.1-                                                               T
SRR1269697.3110262.1+                                                               T
SRR1271617.81306830.2+                                                              T
                         ____________________________________________________________
consensus                ATCAAGAGAATGCTCTTCCTCTTGTTCCTCCCATGCTTTTGTTCTGGCCAGCTTGCACCT

                             .    :    .    :    .    :    .    :    .    :    .    :
SRR1269723.7787279.2+    C
SRR1271618.10995740.1-   C
SRR1271631.17796850.1+   C
SRR1271617.65162719.1+   CC
```

SRR1271631.73182052.1+	CC
SRR1271642.4563788.2-	CC
SRR1265959.5289237.2+	CCA
SRR1269691.1086423.1+	CCA
SRR1271661.3541586.2+	CCA
SRR1265429.662140.1-	CCAC
SRR1271622.14489347.2+	CCAC
SRR1265101.6853887.1-	CCACT
SRR1266954.10031696.1-	CCACT
SRR1271611.54109106.1-	CCACT
SRR1271627.817121.1-	CCACT
SRR1271631.12525033.2+	CCACT
SRR1271638.7137901.1+	CCACT
SRR1271665.15301974.1-	CCACT
SRR1265485.8142050.2-	CCACTG
SRR1271611.3494094.2-	CCACTG
SRR1271629.12769352.1-	CCACTGC
SRR1271640.2387601.1-	CCACTGC
SRR1265961.2477855.1+	CCACTGCT
SRR1265962.4168719.2-	CCACTGCT
SRR1266947.19802521.2+	CCACTGCT
SRR1266966.437734.2+	CCACTGCT
SRR1271620.2068946.1-	CCACTGCT
SRR1271631.70375615.1-	CCACTGCT
SRR1265105.2962579.1-	CCACTGCTG
SRR1266961.9230787.2-	CCACTGCTG
SRR1265102.11716845.1+	CCACTGCTGC
SRR1265102.5685651.2+	CCACTGCTGC
SRR1265424.11995558.2+	CCACTGCTGC
SRR1266956.13063849.1+	CCACTGCTGC
SRR1266962.4122587.1+	CCACTGCTGC
SRR1271609.4569674.1-	CCACTGCTGC
SRR1271627.6371503.1-	CCACTGCTGC
SRR1271631.66929254.2-	CCACTGCTGC
SRR1271639.961470.1-	CCACTGCTGC
SRR1265486.10320482.1-	CCACTGCTGCG
SRR1266958.1653120.2-	CCACTGCTGTG
SRR1271631.13703327.2+	CCACTGCTGCG
SRR1265107.8255291.2-	CCACTGCTGTGTT
SRR1271611.30562393.2+	CCACTGCTGCGTT
SRR1265094.4259059.2+	CCACTGCTGCGTTT
SRR1265106.6015462.2+	CCACTGCTGTGTTT
SRR1265419.2098789.1+	CCACTGCTGTGTTT
SRR1266948.8806088.2+	CCACTGCTGCGTTT
SRR1265429.12954956.2-	CCACTGCTGCGTTTC
SRR1265487.11821367.2+	CCACTGCTGCGTTTC
SRR1269690.6866648.2+	CCACTGCTGCGTTTC
SRR1269701.5896733.1+	CCACTGCTGCGTTTC
SRR1271605.649185.1+	CCACTGCTGCGTTTC
SRR1265423.6251649.1-	CCACTGCTGCGTTTCC
SRR1266946.18245078.1-	CCACTGCTGCGTTTCC
SRR1266962.11554845.2+	CCACTGCTGCGTTTCC
SRR1269696.1594481.2+	CCACTGCTGCGTTTCC
SRR1271643.6174127.1+	CCACTGCTGCGTTTCC
SRR1271610.5829611.1+	CCACTGCTGCGTTTCCC
SRR1271622.14382387.1+	CCACTGCTGCGTTTCCC
SRR1271624.3306484.1+	CCACTGCTGCGTTTCCC
SRR1265108.2515604.1+	CCACTGCTGCGCTTCCCC
SRR1265424.15339550.2-	CCACTGCTGCGTTTCCCC
SRR1271611.16853764.2+	CCACTGCTGCGTTTCCCC
SRR1271626.3653174.1-	CCACTGCTGCGTTTCCCC
SRR1271639.12989136.2+	CCACTGCTGCGTTTCCCC
SRR1271614.6562104.2-	CCACTGCTGCGTTTCCCCA
SRR1271626.19282964.2+	CCACTGCTGCGTTTCCCCA
SRR1265090.6845871.1+	CCACTGCTGCGTTTCCCCAC
SRR1266962.7593491.1+	CCACTGCTGCGTTTCCCCAC
SRR1269721.13769927.2-	CCACTGCTGCGTTTCACCAC
SRR1271666.15761999.1-	CCACTGCTGCGTTTCCCCAC
SRR1265422.11840911.2+	CCACTGCTGCGTTTCCCCACC
SRR1265423.4185373.1-	CCACTGCTGCGTTTCCCCACC
SRR1265494.4458321.1-	CCACTGCTGCGTTTCCCCACC
SRR1271625.17771509.2+	CCACTGCTGCGGTTCCCCACC
SRR1271665.16602572.1+	CCACTGCTGCGTTTCCCCACC
SRR1269726.363820.1+	CCACTGCTGCGTTTCCCCACCT
SRR1271616.2011111.2+	CCACTGCTGCGTTTACCCACCTT
SRR1271626.27417879.2+	CCACTGCTGCGTTTCCCCACCTT
SRR1271630.3176113.1+	CCACTGCTGCGTTTCCCCACCTT
SRR1271664.2748803.2-	CCACTGCTGCGTTTCCCCACCTT
SRR1265423.845833.1+	CCACTGCTGCGTTTCCCCACCTTA
SRR1265425.678706.1+	CCACTGCTGCGTTTCCCCACCTTA
SRR1265960.14555341.2+	CCACTGCTGCGTTTCCCCACCTTA
SRR1266948.14467911.1+	CCACTGCTGCGTTTCCCCACCTTA
SRR1269695.4570879.2-	CCACTGCTGCGTTTCCCCACCTTA
SRR1271631.92663139.1+	CCACTGCTGCGTTTCCCCACCTTA

SRR1271620.711931.1- CCACTGCTGTGTTTCCCCACCTTAT
SRR1271637.6571114.2- CCACTGCTGCGTTTCCCCACCTTAT
SRR1265424.9055674.2- CCACTGCTGCGTTTCCCCACCTTATT
SRR1269690.5523621.2- CCACTGCTGCGTTTCCCCACCTTATT
SRR1271611.24840466.2- CCACTGCTGCGTTTCCCCACCTTATT
SRR1265485.1858508.1- CCACTGCTGCGTTTCCCCACCTTATTG
SRR1266952.14069486.2+ CCACTGCTGCGTTTCCCCACCTTATTG
SRR1271627.4158392.1- CCACTGCTGCGTTTCCCCACCTTATTG
SRR1271661.6840898.1- CCACTGCTGTGTTTCCCCACCTTATTG
SRR1271666.12193922.1- CCACTGCTGCGTTTCCCCACCTTATTG
SRR1265420.9704864.2+ CCACTGCTGCGTTTCCCCACCTTATTGG
SRR1265960.4362410.1+ CCACTGCTGCGTTTCCCCACCTTATTGG
SRR1266950.19035200.2+ CCACTGCTGCGTTTCCCCACCTTATTGG
SRR1271617.21164005.1+ CCACTGCTGCGTTTCCCCACCTTATTGGA
SRR1271631.15064325.1- CCACTGCTGCGTTTCCCCACCTTATTGGA
SRR1271661.9678304.1+ CCACTGCTGCGTTTCCCCACCTTATTGGA
SRR1271663.7532180.2- CCACTGCTGCGTTTCCCCACCTTATTGGA
SRR1265485.16069283.1- CCACTGCTGCGTTTCCCCACCTTATTGGAT
SRR1265493.882472.2+ CCACTGCTGCGTTTCCCCACCTTATTGGAT
SRR1265960.20313739.2- CCACTGCTGCGTTTCCCCACCTTATTGGAT
SRR1271626.35055944.1- CCACTGCTGCGTTTCCCCACCTTATTGGAT
SRR1271629.8867623.1- CCACTGCTGCGTTTCCCCACCTTATTGGAT
SRR1269720.6697396.2+ CCACTGCTGCGTTTCCCCACTTTATTGGATC
SRR1271609.1031300.1+ CCACTGCTGCGTTTCCCCACCTTATTGGATC
SRR1271611.10364010.1+ CCACTGCTGCGTTTCCCCACCTTATTGGATC
SRR1271635.1018397.2+ CCACTGCTGCGTTTCCCCACCTTATTGGATC
SRR1266960.6717190.2- CCACTGCTGCGTTTCCCCACCTTATTGGATCC
SRR1271631.56849156.1+ CCACTGCTGCGTTTCCCCACCTTATTGGATCC
SRR1265103.11096904.1+ CCACTGCTGCGTTTCCCCACCTTATTGGATCCT
SRR1265106.6372305.2- CCACTGCTGCGTTTCCCCACCTTATTGGATCCT
SRR1266947.14185884.1+ CCACTGCTGCGTTTCCCCACCTTATTGGATCCT
SRR1266951.18466248.1- CCACTGCTGCGTTTCCCCACCTTATTGGATCCT
SRR1269700.1764719.1+ CCACTGCTGCGTTTCCCCACCTTATTGGATCCT
SRR1265421.11935315.2- CCACTGCTGCGTTTCCCCACCTTATTGGATCCTT
SRR1265959.15507634.1- CCACTGCTGCGTTTCCCCACCTTATTGGATCCTT
SRR1271611.18839548.2- CCACTGCTGCGTTTCCCCACCTTATTGGATCCTT
SRR1271611.38267951.1- CCACTGCTGCGTTTCCCCACCTTATTGGATCCTT
SRR1271618.11716789.1- CCACTGCTGTGTTTCCCCACCTTATTGGATCCTT
SRR1271635.4911972.1- CCACTGCTGCGTTTCCCCACCTTATTGGATCCTT
SRR1271642.1942769.2- CCACTGCAGCGTTTCCCCACCTTATTGGATCCTT
SRR1265485.10372300.1+ CCACTGCTGCGTTTCCCCACCTTATTGGATCCTTC
SRR1265486.1000359.1- CCACTGCTGCGTTTCCCCACCTTATTGGATCCTTC
SRR1265487.14014666.1+ CCACTGCTGCGTTTCCCCACCTTATTGGATCCTTC
SRR1271611.5176815.1+ CCACTGCTGCGTTTCCCCACCTTATTGGATCCTTC
SRR1271656.28111822.2+ CCACTGCTGCGTTTCCCCACCTTATTGGATCCTTC
SRR1271656.48186978.2- CCACTGCTGCGTTTCCCCACCTTATTGGATCCTTC
SRR1265493.1938206.2- CCACTGCTGCGTTTCCCCACCTTATTGGATCCTTCC
SRR1271617.107171253.2- CCACTGCTGCGTTTCCCCACCTTATTGGATCCTTCC
SRR1265093.9367806.1+ CCACTGCTGCGTTTCCCCACCTTATTGGATCCTTCCA
SRR1265429.3960386.1- CCACTGCTGCGTTTCCCCACCTTATTGGATCCTTCCA
SRR1271656.40523025.1- CCACTGCTGCGTTTCCCCACCTTATTGGATCCTTCCA
SRR1266952.5817620.2- CCACTGCTGCGTTTCCCCACCTTATTGGATCCTTCCAG
SRR1266956.8774588.1- CCACTGCTGCGTTTCCCCACCTTATTGGATCCTTCCAG
SRR1266965.13605504.1- CCACTGCTGCGTTTCCCCACCTTATTGGATCCTTCCAG
SRR1269696.7386843.1- CCACTGCTGCGTTTCCCCACCTTATTGGATCCTTCCAG
SRR1271656.16911801.1- CCACTGCTGCGTTTCCCCACCTTATTGGATCCTTCCAG
SRR1265100.8844830.1- CCACTGCTGCGTTTCCCCACCTTATTGGATCCTTCCAGC
SRR1271617.106361713.2+ CCACTGCTGCGTTTCCCCACCTTATTGGATCCTTCCAGC
SRR1271617.35068629.2+ CCACTGCTGCGTTTCCCCACCTTATTGGATCCTTCCAGC
SRR1271617.93090910.1+ CCACTGCTGCGTTTCCCCACCTTATTGGATCCTTCCAGC
SRR1265118.9445284.1+ CCACTGCTGCGTTTCCCCACCTTATTGGATCCTTCCAGCA
SRR1271621.3138183.1+ CCACTGCTGTGTTTCCCCACCTTATTGGATCCTTCCAGCA
SRR1271639.5769975.1- CCACTGCTGCGTTTCCCCACCTTATTGGATCCTTCCAGCA
SRR1269723.11892874.1+ CCACTGCTGCGTTTCCCCACCTTATTGGATCCTTCCAGCAT
SRR1265421.10156975.1- CCACTGCTGCGTTTCCCCACCTTATTGGATCCTTCCAGCATG
SRR1265961.17342409.2+ CCACTGCTGCGTTTCCCCACCTTATTGGCTCCTTCCAGCATG
SRR1269722.612784.1+ CCACTGCTGCGTTTCCCCACCTTATTGGATCCTTCCAGCATG
SRR1271616.12361.2+ CCACTGCTGCGTTTCCCCACCTTATTGGATCCTTCCAGCATG
SRR1271631.2131030.2+ CCACTGCTGCGTTTCCCCACCTTATTGGATCCTTCCAGCATG
SRR1271637.2457105.1- CCACTGCTGCGTTTCCCCACCTTATTGGATCCTTCCAGCATG
SRR1266960.8454366.2+ CCACTGCTGCGTTTCCCCACCTTATTGGATCCTTCCAGCATGT
SRR1269691.6982732.2+ CCACTGCTGCGTTTCCCCACCTTATTGGATCCTTCCAGCATGT
SRR1269703.1669213.2+ CCACTGCTGCGTTTCCCCACCTTATTGGATCCTTCCAGCATGT
SRR1265103.11542175.1+ CCACTGCTGCGTTTCCCCACCTTATTGGATCCTTCCAGCATGTT
SRR1269721.3127720.1- CCACTGCTGCGTTTCCCCACCTTATTGGATCCTTCCAGCATGTT
SRR1271612.6153622.1+ CCACTGCTGCGTTTCCCCACCTTATTGGATCCTTCCAGCATGTT
SRR1271617.49734526.1+ CCACTGCTGCGTTTCCCCACCTTATTGGATCCTTCCAGCATGTT
SRR1271624.1867358.2+ CCACTGCTGCGTTTCCCCACCTTATTGGATCCTTCCAGCATGTT
SRR1266947.868250.2+ CCACTGCTGCGTTTCCCCACCTTATTGGATCCTTCCAGCATGTTC
SRR1271636.20102713.1- CCACTGCTGCGTTTCCCCACCTTATTGGATCCTTCCAGCATGTTC
SRR1265116.2223311.1- CCACTGCTGCGTTTCCCCACCTTATTGGATCCTTCCAGCATGTTCT
SRR1265420.9541940.2+ CCACTGCTGTGTTTCCCCACCTTATTGGATCCTTCCAGCATGTTCT
SRR1265423.5540826.1+ CCACTGCTGCGTTTCCCCACCTTATTGGATCCTTCCAGCATGTTCT
SRR1265486.10961790.2- CCACTGCTGCGTTTCCCCACCTTATTGGATCCTTCCAGCATCTTCT

SRR1266954.13274081.2+ CCACTGCTGCGTTTCCCCACCTTATTGGATCCTTCCAGCATGTTCT
SRR1271626.5495113.1+ CCACTGCTGCGTTTCCCCACCTTATTGGATCCTTCCAGCATGTTCT
SRR1271629.12293870.2- CCACTGCTGCGTTTCCCCACCTTATTGGATCCTTCCAGCATGTTCT
SRR1271644.2407654.1- CCACTGCTGCGTTTCCCCACCTTATTGGATCCTTCCAGCATGTTCT
SRR1271664.496248.1+ CCACTGCTGCGTTTCCCCACCTTATTGGATCCTTCCAGCATGTTCTA
SRR1265492.11023875.2+ CCACTGCTGCGTTTCCCCACCTTATTGGATCCTTCCAGCATGTTCTAC
SRR1266948.7331073.2- CCACTGCTGCGTTTCCCCACCTTATTGGATCCTTCCAGCATTTTCTAC
SRR1271605.19857960.1- CCACTGCTGCGTTTCCCCACCTTATTGGATCCTTCCAGCATGTTCTAC
SRR1271611.49730989.1+ CCACTGCTGCGTTTCCCCACCTTATTGGATCCTTCCAGCATGTTCTAC
SRR1271620.8170287.2+ CCACTGCTGTGTTTCCCCACCTTATTGGATCCTTCCAGCATGTTCTAC
SRR1271626.25836168.2- CCACTGCTGCGTTTCCCCACCTTATTGGATCCTTCCAGCATGTTCTAC
SRR1271617.31861062.2+ CCACTGCTGCGTTTCCCCACCTTATTGGATCCTTCCAGCATGTTCTACC
SRR1271631.13391857.2+ CCACTGCTGCGTTTCCCCACCTTATTGGATCCTTCCAGCATGTTCTACC
SRR1266959.358352.2- CCACTGCTGCGTTTCCCCCCCTTATTGGATCCTTCCAGCATGTTCTCCCT
SRR1269694.9029316.2- CCACTGCTGCGTTTCCCCACCTTATTGGATCCTTCCAGCATGTTCTACCT
SRR1271639.4180019.1+ CCACTGCTGCGTTTCCCCACCTTATTGGATCCTTCCAGCATGTTCTACCT
SRR1271630.6754689.2+ CCACTGCTGCGTTTCCCCACCTTATTGGATCCTTCCAGCATGTTCTACCTC
SRR1265103.11542175.2- CCACTGCTGCGTTTCCCCACCTTATTGGATCCTTCCAGCATGTTCTACCTCC
SRR1266950.10231735.2+ CCACTGCTGCGTTTCCCCACCTTATTGGATCCTTCCAGCATGGTCTCCCTCC
SRR1271618.11957621.2+ CCACTGCTGTGTTTCCCCACCTTATTGGATCCTTCCAGCATGTTCTACCTCC
SRR1265419.2274158.2+ CCACTGCTGTGTTTCCCCACCTTATTGGATCCTTCCAGCATGTTCTACCTCCA
SRR1269696.95546.1- CCACTGCTGCGTTTCCCCACCTTATTGGATCCTTCCAGCATGTTCTACCTCCA
SRR1269698.2485360.2- CCACTGCTGCGTTTCCCCACCTTATTGGATCCTTCCAGCATGTTCTACCTCCA
SRR1269723.12292891.1+ CCACTGCTGCGTTTCCCCACCTTATTGGATCCTTCCAGCATGTTCTACCTCCA
SRR1271623.14004856.2+ CCACTGCTGCGTTTCCCCACCTTATTGGATCCTTCCAGCATGTTCTACCTCCA
SRR1265419.9204382.2- CCACTGCTGTGTTTCCCCACCTTATTGGATCCTTCCAGCATGTTCTACCTCCAC
SRR1265488.2451011.1- CCACTGCTGCGTTTCCCCACCTTATTGGATCCTTCCAGCATGTTCTACCTCCAC
SRR1265962.6556046.2- CCACTGCTGCGTTTCCCCACCTTATTGGATCCTTCCAGCATGTTCTACCTCCAC
SRR1271610.18281570.1+ CCACTGCTGCGTTTCCCCACCTTATTGGATCCTTCCAGCATGTTCTACCTCCACT
SRR1265100.6999977.1- CCACTGCTGCGTTTCCCCACCTTATTGGATCCTTCCAGCATGTTCTACCTCCACTG
SRR1271612.6407329.2+ CCACTGCTGCGTTTCCCCACCTTATTGGATCCTTCCAGCATGTTCTACCTCCACTG
SRR1271660.19233729.1+ CCACTGCTGCGTTTCCCCACCTTATTGGATCCTTCCAGCATGTTCTACCTCCACTG
SRR1265091.8211546.1+ CCACTGCCGCGTTTCCCCACCTTATTGGATCCTTCCAGCATGTTCTACCTCCACTGG
SRR1265102.1899426.1+ CCACTGCTGCGTTTCCCCACCTTATTGGATCCTTCCAGCATGTTCTACCTCCCCTGG
SRR1266948.400501.2+ CCACTGCTGCGTTTCCCCACCTTATTGGATCCTTCCAGCATGTTCTACCTCCACTGG
SRR1269700.2113418.1+ CCACTGCTGCGTTTCCCCACCTTATTGGATCCTTCCAGCATGTTCTACCTCCACTGG
SRR1271621.4075809.1- CCACTGCTGTGTTTCCCCACCTTATTGGATCCTTCCAGCATGTTCTACCTCCACTGG
SRR1271628.228721.1+ CCACTGCTGCGTTTCCCCACCTTATTGGATCCTTCCAGCATGTTCTACCTCCACTGG
SRR1271631.42457297.2- CCACTGCTGCGTTTCCCCACCTTATTGGATCCTTCCAGCATGTTCTACCTCCACTGG
SRR1271631.89798142.2+ CCACTGCTGCGTTTCCCCACCTTATTGGATCCTTCCAGCATGTTCTACCTCCACTGG
SRR1271640.8629898.2+ CCACTGCTGCGTTTCCCCACCTTATTGGATCCTTCCAGCATGTTCTACCTCCACTGG
SRR1265421.26923308.1+ CCACTGCTGTGTTTCCCCACCTTATTGGATCCTTCCAGCATGTTCTACCTCCACTGGG
SRR1269694.1056175.1+ CCACTGCTGCGTTTCCCCACCTTATTGGATCCTTCCAGCATGTTCTACCTCCACTGGGA
SRR1269695.5358702.1+ CCACTGCTGCGTTTCCCCACCTTATTGGATCCTTCCAGCATGTTCTACCTCCACTGGGA
SRR1265092.2139034.1- CCACTGCTGCGTTTCCCCACCTTATTGGATCCTTCCAGCATGTTCTCCCTCCACTGGGAC
SRR1265101.2621422.2- CCACTGCTGCGTTTCCCCACCTTATTGGATCCTTCCAGCATGTTCTACCTCCACTGGGAC
SRR1265102.11843773.1- CCACTGCTGCGTTTCCCCACCTTATTGGATCCTTCCAGCATGTTCTACCTCCACTGGGAC
SRR1265103.4526646.2+ CCACTGCTGCGTTTCCCCACCTTATTGGATCCTTCCAGCATGTTCTACCTCCACTGGGAC
SRR1265103.8617061.1- CCACTGCTGCGTTTCCCCACCTTATTGGATCCTTCCAGCATGTTCTACCTCCACTGGGAC
SRR1265107.10953111.1+ CCACTGCTGTGTTTCCCCACCTTATTGGATCCTTCCAGCATGTTCTACCTCCACTGGGAC
SRR1265107.11198169.1+ CCACTGCTGCGTTTCCCCACCTTATTGGATCCTTCCAGCATGTTCTACCTCCACTGGGAC
SRR1265107.2088771.2- CCACTGCTGTGTTTCCCCACCTTATTGGATCCTTCCAGCATGTTCTACCTCCACTGGGAC
SRR1265107.6644656.1- CCACTGCTGTGTTTCCCCACCTTATTGGATCCTTCCAGCATGTTCTACCTCCACTGGGAC
SRR1265107.9116199.1- CCACTGCTGTGTTTCCCCACCTTATTGGATCCTTCCAGCATGTTCTACCTCCACTGGGAC
SRR1265108.4121277.1- CCACTGCTGCGTTTCCCCACCTTATTGGATCCTTCCAGCATGTTCTACCTCCACTGGGAC
SRR1265116.6928798.1+ CCACTGCTGCGTTTCCCCACCTTATTGGATCCTTCCAGCATGTTCTACCTCCACTGGGAC
SRR1265120.3033993.2+ CCACTGCTGCGTTTCCCCACCTTATTGGATCCTTCCAGCATGTTCTACCTCCACTGGGAC
SRR1265419.2667566.2- CCACTGCTGTGTTTCCCCACCTTATTGGATCCTTCCAGCATGTTCTACCTCCACTGGGAC
SRR1265421.10976887.1+ CCACTGCTGTGTTTCCCCACCTTATTGGATCCTTCCAGCATGTTCTACCTCCACTGGGAC
SRR1265421.28186333.1+ CCACTGCTGCGTTTCCCCACCTTATTGGATCCTTCCAGCATGTTCTACCTCCACTGGGAC
SRR1265421.28387803.2+ CCACTGCTGCGTTTCCCCACCTTATTGGATCCTTCCAGCATGTTCTACCTCCACTGGGAC
SRR1265422.12487509.2- CCACTGCTGCGTTTCCCCACCTTATTGGATCCTTCCAGCATGTTCTACCTCCACTGGGAC
SRR1265424.13096215.2- CCACTGCTGCGTTTCCCCACCTTATTGGATCCTTCCAGCATGTTCTACCTCCACTGGGAC
SRR1265424.14499483.2- CCACTGCTGCGTTTCCCCACCTTATTGGATCCTTCCAGCATGTTCTACCTCCACTGGGAC
SRR1265424.4163374.2+ CCACTGCTGCGTTTCCCCACCTTATTGGATCCTTCCAGCATGTTCTACCTCCACTGGGAC
SRR1265427.12651809.2- CCACTGCTGCGTTTCCCCACCTTATTGGATCCTTCCAGCATGTTCTACCTCCACTGGGAC
SRR1265427.6605459.1+ CCACTGCTGCGTTTCCCCACCTTATTGGATCCTTCCAGCATGTTCTACCTCCACTGGGAC
SRR1265430.10892354.1+ CCACTGCTGCGTTTCCCCACCTTATTGGATCCTTCCAGCATGTTCTACCTCCACTGGGAC
SRR1265485.8802153.2- CCACTGCTGCGTTTCCCCACCTTATTGGATCCTTCCAGCATGTTCTACCTCCACTGGGAC
SRR1265487.10026670.1- CCACTGCTGCGTTTCCCCACCTTATTGGATCCTTCCAGCATGTTCTACCTCCACTGGGAC
SRR1265487.15510276.2- CCACTGCTGCGTTTCCCCACCTTATTGGATCCTTCCAGCATGTTCTACCTCCACTGGGAC
SRR1265488.7425864.1- CCACTGCTGCGTTTCCCCACCTTATTGGATCCTTCCAGCATGTTCTACCTCCACTGGGAC
SRR1265490.7959942.2+ CCACTGCTGCGTTTCCCCACCTTATTGGATCCTTCCAGCATGTTCTACCTCCACTGGGAC
SRR1265493.2249139.2- CCACTGCTGCGTTTCCCCACCTTATTGGATCCTTCCAGCATGTTCTACCTCCACTGGGAC
SRR1265959.13791605.2+ CCACTGCTGCGTTTCCCCACCTTATTGGATCCTTCCAGCATGTTCTACCTCCACTGGGAC
SRR1265959.16757127.1- CCACTGCTGCGTTTCCCCACCTTATTGGATCCTTCCAGCATGTTCTACCTCCACTGGGAC
SRR1265960.6622381.1+ CCACTGCTGCGTTTCCCCACCTTATTGGATCCTTCCAGCATGTTCTACCTCCACTGGGAC
SRR1266947.12981212.1- CCACTGCTGCGTTTCCCCACCTTATTGGATCCTTCCAGCATGTTCTACCTCCACTGGGAC
SRR1266947.22170370.1+ CCACTGCTGCGTTTCCCCACCTTATTGGATCCTTCCAGCATGTTCTACCTCCACTGGGAC
SRR1266949.1088605.1- CCACTGCTGCGTTTCCCCACCTTATTGGATCCTTCCAGCATGTTCTACCTCCACTGGGAC
SRR1266950.11307404.1- CCACTGCTGCGTTTCCCCACCTTATTGGATCCTTCCAGCATGTTCTACCTCCACTGGGAC
SRR1266950.4890096.2- CCACTGCTGCTTTTCCCCACCTTATTGGATCCTTCCAGCATGTTCTACCTCCACTGGGTC
SRR1266953.3952558.2+ CCACTGCTGCGTTTCCCCACCTTATTGGATCCTTCCAGCATGTTCTACCTCCACTGGGAC
SRR1266954.9977358.1- CCACTGCTGCGTTTCCCCACCTTATTGGATCCTTCCAGCATGTTCTACCTCCACTGGGAC

```
SRR1266955.12775171.2-   CCACTGCTGCGTTTCCCCACCTTATTGGATCCTTCCAGCATGTTCTACCTCCACTGGGAC
SRR1266955.7154550.1+    CCACTGCTGCGTTTCCCCACCTTATTGGATCCTTCCAGCATGTTCTACCTCCACTGGGAC
SRR1266957.10574795.2-   CCACTGCTGCGTTTCCCCACCTTATTGGATCCTTCCAGCATGTTCTACCTCCACTGGGAC
SRR1266957.1389486.2+    CCACTGCTGCGTTTCCCCACCTTATTGGATCCTTCCAGCATGTTCTACCTCCACTGGGAC
SRR1266957.3666759.1+    CCACTGCTGCGTTTCCCCACCTTATTGGATCCTTCCAGCATGTTCTACCTCCACTGGGAC
SRR1266958.3227986.1-    CCACTGCTGCGTTTCCCCACCTTATTGGATCCTTCCAGCATGTTCTACCTCCACTGGGAC
SRR1266960.4673203.1-    CCACTGCTGCGTTTCCCCACCTTATTGGATCCTTCCAGCATGTTCTACCTCCACTGGGAC
SRR1266962.2111129.1-    CCACTGCTGCGTTTCCCCACCTTATTGGATCCTTCCAGCATGTTCTACCTCCACTGGGAC
SRR1266962.4519620.1-    CCACGGCGGCGTTTCCCCACCTTATTGGATCCTTCCAGCATGTTCTACCTCCACTGGGAC
SRR1266963.4385495.1+    CCACTGCTGCGTTTCCCCACCTTATTGGATCCTTCCAGCATGTTCTACCTCCACTGGGAC
SRR1266965.6800008.2-    CCACTGCTGCGTTTCCCCACCTTATTGGATCCTTCCAGCATGTTCTCCCTCCACTGGGAC
SRR1266965.6865544.1+    CCACTGCTGCGTTTCCCCACCTTATTGGATCCTTCCAGCATGTTCTACCTCCACTGGGAC
SRR1266966.1422141.1+    CCACTGCTGCGTTTCCCCACCTTATTGGATCCTTCCAGCATGTTCTACCTCCACTGGGAC
SRR1269689.10802270.2+   CCACTGCTGCGTTTCCCCACCTTATTGGATCCTTCCCGCATGGTCTACCTCCACTGGGAC
SRR1269689.606782.2+     CCACTGCTGTGTTTCCCCACCTTATTGGATCCTTCCAGCATGTTCTACCTCCACTGGGAC
SRR1269690.1324197.2-    CCACTGCTGCGTTTCCCCACCTTATTGGATCCTTCCAGCATGTTCTACCTCCACTGGGAC
SRR1269690.6701197.1+    CCACTGCTGTGTTTCCCCACCTTATTGGATCCTTCCAGCATGTTCTACCTCCACTGGGAC
SRR1269690.8024392.2+    CCACTGCTGCGTTTCCCCACCTTATTGGATCCTTCCAGCATGTTCTACCTCCACTGGGAC
SRR1269691.5900866.2+    CCACTGCTGTGTTTCCCCACCTTATTGGATCCTTCCAGCATGTTCTACCTCCACTGGGAC
SRR1269692.1681865.1-    CCACTGCTGTGTTTCCCCACCTTATTGGATCCTTCCAGCATGTTCTACCTCCACTGGGAC
SRR1269693.1880844.2+    CCACTGCTGTGTTTCCCCACCTTATTGGATCCTTCCAGCATGTTCTACCTCCACTGGGAC
SRR1269695.5539655.2+    CCACTGCTGCGTTTCCCCACCTTATTGGATCCTTCCAGCATGTTCTACCTCCACTGGGAC
SRR1269695.709605.2-     CCACTGCTGCGTTTCCCCACCTTATTGGATCCTTCCAGCATGTTCTACCTCCACTGGGAC
SRR1269697.3110262.1+    CCACTGCTGCGTTTCCCCACCTTATTGGATCCTTCCAGCATGTTCTACCTCCACTGGGAC
SRR1269698.4930714.1-    CCACTGCTGCGTTTCCCCACCTTATTGGATCCTTCCAGCATGTTCTACCTCCACTGGGAC
SRR1269700.8551703.1+    CCACTGCTGCGTTTCCCCACCTTATTGGATCCTTCCAGCATGTTCTACCTCCACTGGGAC
SRR1269700.9464373.2+    CCACTGCTGCGTTTCCCCACCTTATTGGATCCTTCCAGCATGTTCTACCTCCACTGGGAC
SRR1269721.7475901.1+    CCACTGCTGCGTTTCCCCACCTTATTGGATCCTTCCAGCATGTTCTACCTCCACTGGGAC
SRR1269724.4208407.1+    CCACTGCTGCGATTCCCCACCTTATTGGATCCTTCCAGCATGTTCTACCTCCACTGGGAC
SRR1271605.5228759.1+    CCACTGCTGCGTTTCCCCACCTTATTGGATCCTTCCAGCATGTTCTACCTCCACTGGGAC
SRR1271611.45328290.1+   CCACTGCTGCGTTTCCCCACCTTATTGGATCCTTCCAGCATGTTCTACCTCCACTGGGAC
SRR1271611.5929085.1+    CCACTGCTGCGTTTCCCCACCTTATTGGATCCTTCCAGCATGTTCTACCTCCACTGGGAC
SRR1271612.6213795.1+    CCACTGCTGCGTTTCCCCACCTTATTGGATCCTTCCAGCATGTTCTACCTCCACTGGGAC
SRR1271614.6603009.1-    CCACTGCTGCGTTTCCCCACCTTATTGGATCCTTCCAGCATGTTCTACCTCCACTGGGAC
SRR1271616.4976712.2+    CCACTGCTGCGTTTCCCCACCTTATTGGATCCTTCCAGCATGTTCTACCTCCACTGGGAC
SRR1271617.101618594.2+  CCACTGCTGCGTTTCCCCACCTTATTGGATCCTTCCAGCATGTTCTACCTCCACTGGGAC
SRR1271617.105521641.1-  CCACTGCTGCGTTTCCCCACCTTATTGGATCCTTCCAGCATGTTCTACCTCCACTGGGAC
SRR1271617.36691224.1-   CCACTGCTGCGTTTCCCCACCTTATTGGATCCTTCCAGCATGTTCTACCTCCACTGGGAC
SRR1271617.46820496.1+   CCACTGCTGCGTTTCCCCACCTTATTGGATCCTTCCAGCATGTTCTACCTCCACTGGGAC
SRR1271617.81306830.2+   CCACTGCTGCGTTTCCCCACCTTATTGGATCCTTCCAGCATGTTCTACCTCCACTGGGAC
SRR1271617.8325012.1+    CCACTGCTGCGTTTCCCCACCTTATTGGATCCTTCCAGCATGTTCTACCTCCACTGGGAC
SRR1271618.649812.2+     CCACTGCTGTGTTTCCCCACCTTATTGGATCCTTCCAGCATGTTCTACCTCCACTGGGAC
SRR1271619.10258847.1-   CCACTGCTGTGTTTCCCCACCTTATTGGATCCTTCCAGCATGTTCTACCTCCACTGGGAC
SRR1271620.12578244.2+   CCACTGCTGTGTTTCCCCACCTTATTGGATCCTTCCAGCATGTTCTACCTCCACTGGGAC
SRR1271623.12702077.1+   CCACTGCTGCGTTTCCCCACCTTATTGGATCCTTCCAGCATGTTCTACCTCCACTGGGAC
SRR1271624.11221728.1-   CCACTGCTGCGTTTCCCCACCTTATTGGATCCTTCCAGCATGTTCTACCTCCACTGGGAC
SRR1271624.16629241.2-   CCACTGCTGCGTTTCCCCACCTTATTGGATCCTTCCAGCATGTTCTACCTCCACTGGGAC
SRR1271625.13588244.2+   CCACTGCTGCGGTTCCCCACCTTATTGGATCCTTCCAGCATGTTCTACCTCCACTGGGAC
SRR1271626.29048032.1+   CCACTGCTGCGTTTCCCCACCTTATTGGATCCTTCCAGCATGTTCTACCTCCACTGGGAC
SRR1271626.39375321.1-   CCACTGCTGCGTTTCCCCACCTTATTGGATCCTTCCAGCATGTTCTACCTCCACTGGGAC
SRR1271626.8851170.1+    CCACTGCTGCGTTTCCCCACCTTATTGGATCCTTCCAGCATGTTCTACCTCCACTGGGAC
SRR1271631.25901633.1+   CCACTGCTGCGTTTCCCCACCTTATTGGATCCTTCCAGCATGTTCTACCTCCACTGGGAC
SRR1271631.56868617.2-   CCACTGCTGCGTTTCCCCACCTTATTGGATCCTTCCAGCATGTTCTACCTCCACTGGGAC
SRR1271634.8456855.2-    CCACTGCTGCGTTTCCCCACCTTATTGGATCCTTCCAGCATGTTCTACCTCCACTGGGAC
SRR1271636.5617421.2+    CCACTGCTGCGTTTCCCCACCTTATTGGATCCTTCCAGCATGTTCTACCTCCACTGGGAC
SRR1271639.11541096.1-   CCACTGCTGCGTTTCCCCACCTTATTGGATCCTTCCAGCATGTTCTACCTCCACTGGGAC
SRR1271639.16935460.1+   CCACTGCTGCGTTTCCCCACCTTATTGGATCCTTCCAGCATGTTCTACCTCCACTGGGAC
SRR1271639.17341141.1+   CCACTGCTGCGTTTCCCCACCTTATTGGATCCTTCCAGCATGTTCTACCTCCACTGGGAC
SRR1271641.6014124.1+    CCACTGCTGCGTTTCCCCACCTTATTGGATCCTTCCAGCATGTTCTACCTCCACTGGGAC
SRR1271642.5331247.1+    CCACTGCTGCGTTTCCCCACCTTATTGGATCCTTCCAGCATGTTCTACCTCCACTGGGAC
SRR1271643.2019678.1+    CCACTGCTGCGTTTCCCCACCTTATTGGATCCTTCCAGCATGTTCTACCTCCACTGGGAC
SRR1271644.4072249.1-    CCACTGCTGCGTTTCCCCACCTTATTGGATCCTTCCAGCATGTTCTACCTCCACTGGGAC
SRR1271660.11124812.1-   CCACTGCTGTGTTTCCCCACCTTATTGGATCCTTCCAGCATGTTCTACCTCCACTGGGAC
SRR1271660.66535.1-      CCAATGCTGCGTTCCCCCACCTTATTGGATCCGCCCAGCATGTTCTACCTCCACTGGGAC
SRR1271661.2368772.1+    CCACTGCTGCGTTTCCCCACCTTATTGGATCCTTCCAGCATGTTCTACCTCCACTGGGAC
SRR1271662.3330309.1+    CCACTGCTGCGTTTCCCCACCTTATTGGATCCTTCCAGCATGTTCTACCTCCACTGGGAC
SRR1271664.7577323.2+    CCACTGCTGCGTTTCCCCACCTTATTGGATCCTTCCAGCATGTTCTACCTCCACTGGGAC
SRR1271664.7610027.1+    CCACTGCTGCGTTTCCCCACCTTATTGGATCCTTCCAGCATGTTCTACCTCCACTGGGAC
SRR1265429.2980691.2-     CACTGCTGCGTTTCCCCACCTTATTGGATCCTTCCAGCATGTTCTACCTCCACTGGGAC
SRR1265485.4295901.2-     CACTGCTGCGTTTCCCCACCTTATTGGATCCTTCCAGCATGTTCTACCTCCACTGGGAC
SRR1271629.16583.1-       CACTGCTGCGTTTCCCCCCCTTATTGGATCCTTCCAGCATGTTCTACCTCCACTGGGAC
SRR1271617.94327348.2+     ACTGCTGCGTTTCCCCACCTTATTGGATCCTTCCAGCATGTTCTACCTCCACTGGGAC
SRR1265118.3539444.2-       CTGCTGCGTTTCCCCACCTTATTGGATCCTTCCAGCATGTTCTACCTCCACTGGGAC
SRR1269701.5896733.2-       CTGCTGCGTTTCCCCACCTTATTGGATCCTTCCAGCATGTTCTACCTCCACTGGGAC
SRR1269722.10861522.2+      CTGCTGCGTTTCCCCACCTTATTGGATCCTTCCAGCATGTTCTACCTCCACTGGGAC
SRR1265117.1014705.1-        TGCTGCGTTTCCCCACCTTATTGGATCCTTCCAGCATGTTCTACCTCCACTGGGAC
SRR1266963.7668173.1+        TGCTGCGTTTCCCCACCTTATTGGATCCTTCCAGCATGTTCTACCTCCACTGGGAC
SRR1271606.19163420.1+       TGCTGCGTTTCCCCACCTTATTGGATCCTTCCAGCATGTTCTACCTCCACTGGGAC
SRR1271611.41723090.1-       TGCTGCGTTTCCCCACCTTATTGGATCCTTCCAGCATGTTCTACCTCCACTGGGAC
SRR1271660.27380513.2-       TGCTGTGTTTCCCCACCTGATTGGATCCTTCCAGCATGTTCTACCTCCACTGGGAC
SRR1266959.4156269.1+         GCTGCGTTTCCCCACCTTATTGGATCCTTCCAGCATGTTCTACCTCCACTGGGAC
SRR1271620.8170287.1-         GCTGTGTTTCCCCACCTTATTGGATCCTTCCAGCATGTTCTACCTCCACTGGGAC
SRR1271625.17771509.1-        GCTGCGTTTCCCCACCTTATTGGATCCTTCCAGCATGTTCTCCCTCCACTGGGAC
SRR1271630.7036120.2-         GCTGCGTTTCCCCACCTTATTGGATCCTTCCAGCATGTTCTACCTCCACTGGGGC
```

SRR1271631.77846051.2-	GCTGCGTTTCCCCACCTTATTGGATCCTTCCAGCATGTTCTACCTCCACTGGGAC
SRR1265959.16039523.1-	CTGCGTTTCCCCACCTTATTGGATCCTTCCAGCATGTTCTACCTCCACTGGGAC
SRR1271656.12001727.1-	CTGCGTTTCCCCTCCTTATTGGATCCTTCCAGCATGTTCTGCCTCCACTGGGAC
SRR1271656.38998198.1+	CTGCGTTTCCCCACCTTATTGGATCCTTCCAGCATGTTCTACCTCCACTGGGAC
SRR1271635.5444837.2-	TGCGTTTCCCCACCTTATTGGATCCTTCCAGCATGTTCTACCTCCACTGGGAC
SRR1265102.11790677.1+	GCGTTTCCCCACCTTATTGGATCCTTCCAGCATGTTCTACCTCCACTGGGAC
SRR1271631.9620063.1+	GCGTTTCCCCACCTTATTGGATCCTTCCAGCATGTTCTACCTCCACTGGGAC
SRR1269700.230054.1-	CGTTTCCCCCCCTTATTGGATCCTTCCAGCATGTTCTACCTCCACTGGGAC
SRR1269726.6324567.1+	CGTTTCCCCACCTTATTGGATCCTTCCAGCATGTTCTACCTCCACTGGGAC
SRR1265423.4843600.2+	GTTTCCCCACCTTATTGGATCCTTCCAGCATGTTCTACCTCCACTGGGAC
SRR1266955.9100423.2+	GTTTCCCCACCTTATTGGATCCTTCCAGCATGTTCTACCTCCACTGGGAC
SRR1266961.12229708.1-	GTTTCCCCACCTTATTGGATCCTTCCAGCATGTTCTACCTCCACTGGGAC
SRR1271635.8499862.2+	GTTTCCCCACCTTATTGGATCCTTACAGCATGTTCTACCTCCACTGGGAC
SRR1266963.15703496.1-	TTTCCCCACCTTATTGGATCCTTCCAGCATGTTCTCCCTCCACTGGGAC
SRR1269699.10804333.2-	TTTCCCCACCTTATTGGATCCTTCCAGCATGTTCTACCTCCACTGGGAC
SRR1271633.8205548.2-	TTTCCCCACCTTATTGGATCCTTCCAGCATGTTCTACCTCCACTGGGAC
SRR1271637.6456421.1+	TTCCCCACCTTATTGGATCCTTCCAGCATGTTCTACCTCCACTGGGAC
SRR1266952.10330331.1+	TCCCCACCTTATTGGATCCTTCCAGCATGTTCTACCTCCACTGGGAC
SRR1271614.6488833.1-	TCCCCACCTTATTGGATCCTTCCAGCATGTTCTCCCTCCACTGGGAC
SRR1271617.92587535.2-	TCCCCACCTTATTGGATCCTTCCAGCATGTTCTGCCTCCACTGGGAC
SRR1265102.5685651.1-	CCCCACCTTATTGGATCCTTCCAGCATGTTCTACCTCCACTGGGAC
SRR1265424.13766781.2+	CCCCACCTTATTGGATCCTTCCAGCATGTTCTACCTCCACTGGGAC
SRR1269725.3278979.1+	CCCCACCTTATTGGATCCTTCCAGCATGTTCTACCTCCACTGGGAC
SRR1266963.9363809.1+	CCCACCTTATTGGATCCTTCCAGCATGTTCTACCTCCACTGGGAC
SRR1269692.5539705.1+	CCCACCTTATTGGATCCTCCCAGCATGTTCTACCTCCACTGGGAC
SRR1271626.8342312.2+	CCCACCTTATTGGATCCTTCCAGCATGTTCTACCTCCACTGGGAC
SRR1271628.3667565.2+	CCCACCTTATTGGATCCTTCCAGCATGTTCTACCTCCACTGGGAC
SRR1271656.28111822.1-	CCCACCTTATTGGATCCTTCCAGCATGTTCTACCTCCACTGGGAC
SRR1266951.4137283.1+	CCACCTTATTGGATCCTTCCAGCATGTTCTACCTCCACTGGGAC
SRR1271617.101746624.1-	CCACCTTATTGGATCCTTCCAGCATGTTCTACCTCCACTGGGAC
SRR1271622.6836463.2-	CCACCTTATTGGATCCTTCCAGCATGTTCTACCTCCACTGGGAC
SRR1265961.2742222.1-	CACCTTATTGGATCCTTCCAGCATGTTCTACCTCCACTGGGAC
SRR1266952.1463982.2-	CACCTTATTGGATCCTTCCAGCATGTTCTACCGCCGCTGGGAC
SRR1271614.8837036.1+	CACCTTATTGGATCCTTCCAGCATGTTCTACCTCCACTGGGAC
SRR1271617.32725188.2-	ACCTTATTGGATCCTTCCAGCATGTTCTACCTCCACTGGGAC
SRR1271631.87281188.2-	ACCTTATTGGATCCTTCCAGCATGTTCTACCTCCACTGGGAC
SRR1265421.19234411.1-	CCTTATTGGATCCTTCCAGCATGTTCTACCTCCACTGGGAC
SRR1271611.42557115.2-	CCTTATTGGATCCTTCCAGCATGTTCTACCTCCACTGGGAC
SRR1271625.3981531.1+	CCTTATTGGATCCTTCCAGCATGTTCTACCTCCACTGGGAC
SRR1265118.3202418.2+	CTTATTGGATCCTTCCAGCATGTTCTACCTCCACTGGGAC
SRR1269720.8521946.1-	CTTATTGGATCCTTCCAGCATGTTCTACCTCCACTGGGAC
SRR1265118.8469183.1+	TTATTGGATCCTTCCAGCATGTTCTACCTCCACTGGGAC
SRR1265427.10426011.1+	TTATTGGATCCTTCCAGCATGTTCTACCTCCACTGGGAC
SRR1266964.4594838.2-	TTATTGGATCCTTCCAGCATGTTCTACCTCCACTGGGAC
SRR1271656.18693034.2-	TTATTGGATCCTTCCAGCATGTTCTACCTCCACTGGGAC
SRR1266959.6559141.2+	TATTGGATCCTTCCAGCATGTTCTACCTCCACTGGGAC
SRR1266962.3051106.2+	TATTGGATCCTTCCAGCATGTTCTACCTCCACTGGGAC
SRR1271623.14435698.2+	TATTGGATCCTTCCAGCATGTTCTACCTCCACTGGGAC
SRR1271625.4462362.2-	TATTGGATCCTTCCAGCATGTTCTACCTCCACTGGGAC
SRR1265090.573951.1+	ATTGGATCCTTCCAGCATGTTCTACCTCCACTGGGAC
SRR1265419.5834061.2-	ATTGGATCCTTCCAGCATGTTCTACCTCCACTGGGAC
SRR1266962.8268942.2-	ATTGGATCCTTCCAGCATGTTCTACCTCCACTGGGAC
SRR1269694.6400404.1+	ATTGGATCCTTCCAGCATGTTCTACCTCCACTGGGAC
SRR1269727.8758172.1+	ATTGGATCCTTCCAGCATGTTCTACCTCCACTGGGAC
SRR1271617.87539143.2+	ATTGGATCCTTCCAGCATGTTCTACCTCCACTGGGAC
SRR1271631.51412202.2-	ATTGGATCCTTCCAGCATGTTCTACCTCCACTGTGAC
SRR1265490.7565460.2+	TTGGATCCTTCCAGCATGTTCTACCTCCACTGGGAC
SRR1271611.32939716.2+	TTGGATCCTTCCAGCATGTTCTACCTCCACTGGGAC
SRR1265107.8184323.2+	TGGATCCTTCCAGCATGTTCTACCTCCACTGGGAC
SRR1265430.3999536.2-	TGGAGCCTTCCAGCATGTTCTTCCTCCACTGGGAC
SRR1271625.4672091.1-	TGGATCCTTCCAGCATGTTCTACCTCCACTGGGAC
SRR1271625.4974445.2-	TGGATACTTCCAGCATGTTCTCCCTCCACGGGGAC
SRR1265490.12123742.2-	GGATCCTTCCAGCATGTTCTACCTCCACTGGGAC
SRR1269725.9198123.1+	GGATCCTTCCAGCATGTTCTACCTCCACTGGGAC
SRR1271666.9606214.1-	GGATCCTTCCAGCATGTTCTACCTCCACTGGGAC
SRR1265108.11287257.2+	GATCCTTCCAGCATGTTCTACCTCCACTGGGAC
SRR1269723.12879211.1+	GATCCTTCCAGCATGTTCTACCTCCACTGGGAC
SRR1271611.35735582.1-	GATCCTTCCAGCATGTTCTACCTCCACTGGGAC
SRR1271611.45105264.1+	GATCCTTCCAGCATGTTCTACCTCCACTGGGAC
SRR1271637.7730229.2-	GATCCTTCCAGCATGTTCTACCTCCACTGGGAC
SRR1271663.9467739.2+	GATCCTTCCAGCATGTTCTACCTCCACTGGGAC
SRR1271605.19945219.1+	ATCCTTCCAGCATGTTCTACCTCCACTGGGAC
SRR1271606.10370241.1+	ATCCTTCCAGCATGTTCTACCTCCACTGGGAC
SRR1271618.8223150.2+	ATCCTTCCAGCATGTTCTACCTCCACTGGGAC
SRR1271617.24478703.2+	TCCTTCCAGCATGTTCTACCTCCACTGGGAC
SRR1271656.16215479.1-	TCCTTCCAGCATTTTCTACCTCCACTGGGAC
SRR1271656.38998198.2-	TCCTTCCAGCATGTTCTACCTCCACTGGGAC
SRR1265427.13084996.1+	CCTTCCAGCATGTTCTACCTCCACTGGGAC
SRR1266946.1503677.1-	CCTTCCAGCATGTTCTACCTCCACTGGGGC
SRR1271656.4976182.2+	CTTCCAGCATGTTCTACCTCCACTGGGAC
SRR1265490.5635851.1+	TTCCAGCATGTTCTACCTCCACTGGGAC
SRR1269724.1613007.2+	TTCCAGCATGTTCTACCTCCACTGGGAC
SRR1271628.1578656.2-	TTCCAGCATGTTCTACCTCCACTGGGAC
SRR1271631.73787149.2-	TTCCAGCATGTTCTACCTCCACTGGGAC

SRR1271660.7768350.2+	TTCCAGCATGTTCTACCTCCACTGGGAC
SRR1271617.29201909.1-	TCCAGCATGTTCTACCTCCACTGGGAC
SRR1271656.27642211.2-	TCCAGCATGTTCTACCTCCACTGGGAC
SRR1271617.18340985.1-	CCAGCATGTTCTACCTCCACTGGGAC
SRR1271643.6252990.1+	CCAGCATGTTCTACCTCCACTGGGAC
SRR1265486.4816291.2+	CAGCATGTTCTACCTCCACTGGGAC
SRR1265490.1647534.2+	CAGCATGTTCTACCTCCACTGGGAC
SRR1266947.1797376.1+	CAGCATGTTCTACCTCCACTGGGAC
SRR1269693.657451.2-	CAGCATGTTCTACCTCCACTGGGAC
SRR1269724.7736550.2-	CAGCATGTTCTACCTCCACGGGGAC
SRR1271625.4440646.1-	CAGCATGTTCTACCTCCACTGGGAC
SRR1271626.7870290.2+	CAGCATGTTCTACCTCCACTGGGAC
SRR1265101.7863435.2+	AGCATGTTCTACCTCCACTGGGAC
SRR1265119.2075759.2+	AGCATGTTCTACCTCCACTGGGAC
SRR1271660.19489729.2-	AGCATGTTCTTCCTCCACTGGGAC
SRR1265425.11836318.2+	GCATGTTCTACCTCCACTGGGAC
SRR1266959.12652072.2-	GCATGTTCTACCTCCACTGGGAC
SRR1271638.10108003.1-	GCATGTTCTACCTCCACTGGGAC
SRR1265107.9762562.1+	CATGTTCTACCTCCACTGGGAC
SRR1271630.7776502.2+	CATGTTCTACCTCCACTGGGAC
SRR1266964.9683545.2+	ATGTTCTACCTCCACTGGGAC
SRR1269724.7878642.2-	ATGTTCTACCTCCACTGGGAC
SRR1271617.106361713.1-	ATGTTCTACCTCCACTGGGAC
SRR1271631.38795764.1+	ATGTTCTACCTCCACTGGGAC
SRR1271637.8462724.1-	ATGTTCTACCTCCACTGGGAC
SRR1269724.2351101.2-	TGTTTTCCCTCCCCTGGGAC
SRR1265424.825035.1+	GTTCTACCTCCACTGGGAC
SRR1266962.933875.2-	GTTCTACCTCCCCTGGGAC
SRR1269695.4605288.1-	GTTCTACCTCCACTGGGAC
SRR1269701.962293.1+	GTTCTACCTCCACTGGGAC
SRR1265094.9258610.2-	TTCTACCTCCACTGGGAC
SRR1266963.14618912.2+	TTCTACCTCCACTGGGAC
SRR1271627.2433963.2+	TTCTACCTCCACTGGGAC
SRR1266962.10797533.1+	TCTACCTCCACTGGGAC
SRR1269702.9206585.1-	TCTACCTCCACTGGGAC
SRR1271636.32970902.1-	TCTACCTCCACTGGGAC
SRR1265108.5336322.1+	CTACCTCCACTGGGAC
SRR1265485.927570.2+	CTACCTCCACTGGGAC
SRR1266948.20521025.2-	CTACCTCCACTGGGAC
SRR1269691.4799971.1+	CTACCTCCACTGGGAC
SRR1269722.8669002.1+	CTACCTCCACTGGGAC
SRR1265487.3611936.2-	TCCCTCCACTGGGAC
SRR1265487.5948289.1+	TACCTCCACTGGGAC
SRR1271656.19755138.1+	TACCTCCACTGGGAC
SRR1265120.10380681.2+	ACCTCCACTGGGAC
SRR1265430.9435022.1-	ACCTCCACTGGGAC
SRR1266959.1925156.1+	ACCTCCACTGGGAC
SRR1271637.9483811.1+	ACCTCCACTGGGAC
SRR1265103.11024313.1+	CCTCCACTGGGAC
SRR1265960.6641003.1-	CCTCCACTGGGAC
SRR1269689.11150721.2+	CCTCCACTGGGAC
SRR1269701.8482472.1+	CCTCCACTGGGAC
SRR1269726.8967180.1-	CCTCCACTGGGAC
SRR1266946.22292515.1-	CTCCACTGGGAC
SRR1265100.7029791.2-	TCCACTGGGAC
SRR1266952.10100495.1-	CCACTGGGAC
SRR1265116.2215467.2+	CACTGGGAC
SRR1265486.9625524.2+	CACTGGGAC
SRR1266948.4974750.1+	CACTGGGAC
SRR1271626.39747880.2-	CACTGGGAC
SRR1265424.14641649.2+	ACTGGGAC
SRR1265100.6825972.2+	CTGGGAC
SRR1265420.6477611.2+	CTGGGAC
SRR1265486.2988089.2-	CTGGGAC
SRR1271634.2225500.2-	CTGGGAC
SRR1271641.1475015.2-	CTGGGAC
SRR1271643.11732587.1-	CTGGGAC
SRR1271656.19605949.2+	CTGGGAC
SRR1265091.7293804.1-	TGGGAC
SRR1265494.10552009.1+	TGGGAC
SRR1266951.1123610.1+	TGGGAC
SRR1269725.4823828.2+	TGGGAC
SRR1271617.7158444.2-	TGGGAC
SRR1271626.27554490.1+	TGGGAC
SRR1269689.1433856.2+	GGGAC
SRR1269726.4838196.2-	GGAC
SRR1271605.3457147.2+	AC
SRR1271614.7695441.1+	AC
SRR1271631.94542648.1+	AC
SRR1269689.9751428.2+	C
SRR1271663.9621731.2+	C
consensus	CCACTGCTGCGTTTCCCCACCTTATTGGATCCTTCCAGCATGTTCTACCTCCACTGGGAC

```
                                .    :    .    :    .    :    .    :    .    :    .    :
SRR1265107.10953111.1+   C
SRR1265108.4121277.1-    C
SRR1266965.6800008.2-    C
SRR1265422.12487509.2-   CA
SRR1271626.8851170.1+    CA
SRR1271639.16935460.1+   CA
SRR1266954.9977358.1-    CAC
SRR1266955.12775171.2-   CAC
SRR1266958.3227986.1-    CAC
SRR1265102.11843773.1-   CACG
SRR1265103.4526646.2+    CACG
SRR1266947.12981212.1-   CACG
SRR1266953.3952558.2+    CACG
SRR1269700.9464373.2+    CACG
SRR1271634.8456855.2-    CACG
SRR1271639.11541096.1-   CACG
SRR1271617.105521641.1-  CACGA
SRR1269693.1880844.2+    CACGAG
SRR1271660.11124812.1-   CACGAG
SRR1271614.6603009.1-    CACGAGG
SRR1271641.6014124.1+    CACGAGG
SRR1266947.22170370.1+   CACGAGGA
SRR1271617.101618594.2+  CACGAGGA
SRR1271617.36691224.1-   CACGAGGA
SRR1271620.12578244.2+   CACGAGGA
SRR1265120.3033993.2+    CACGAGGAA
SRR1271625.13588244.2+   CACGAGGAA
SRR1265959.16757127.1-   CACGAGGAAC
SRR1269724.4208407.1+    CACGAGGAAC
SRR1265485.8802153.2-    CACGAGGAACA
SRR1269690.1324197.2-    CACGAGGAACA
SRR1266955.7154550.1+    CACGAGGAACAG
SRR1271611.5929085.1+    CACGAGGAACAG
SRR1271617.46820496.1+   CACGAGGAACAG
SRR1265107.2088771.2-    CACGAGGAACAGG
SRR1266966.1422141.1+    CACGAGGAACAGGA
SRR1271626.29048032.1+   CACGAGGAACAGGA
SRR1271612.6213795.1+    CACGAGGAACAGGAG
SRR1271636.5617421.2+    CACGAGGAACAGGAG
SRR1271662.3330309.1+    CACGAGGAACAGGAG
SRR1265103.8617061.1-    CACGAGGAACAGGAGCTG
SRR1266957.3666759.1+    CACGAGGAACAGGAGCTGA
SRR1271617.8325012.1+    CACGAGGAACAGGAGCTGA
SRR1265107.11198169.1+   CACGAGGAACAGGAGCTGAT
SRR1265959.13791605.2+   CACGAGGAACAGGAGCTGAT
SRR1271619.10258847.1-   CACGAGGAACAGGAGCTGAT
SRR1271624.16629241.2-   CACGAGGAACAGGAGCTGAT
SRR1265424.14499483.2-   CACGAGGAACAGGAGCTGATG
SRR1265487.15510276.2-   CACGAGGAACAGGAGCTGATG
SRR1266957.1389486.2+    CACGAGGAACAGGAGCTGATG
SRR1269721.7475901.1+    CACGAGGAACAGGAGCTGATG
SRR1271626.39375321.1-   CACGAGGAACAGGAGCTGATG
SRR1271643.2019678.1+    CACGAGGAACAGGAGCTGATG
SRR1266957.10574795.2-   CACGAGGAACAGGAGCTGATGA
SRR1271642.5331247.1+    CACGAGGAACAGGAGCTGATGA
SRR1266964.4594838.2-    CACGAGGAACANGAGCTGATGAG
SRR1266965.6865544.1+    CACGAGGAACAGGAGCTGATGAG
SRR1271611.45328290.1+   CACGAGGAACAGGAGCTGATGAG
SRR1265107.9116199.1-    CACGAGGAACAGGAGCTGATGAGG
SRR1265421.10976887.1+   CACGAGGAACAGGAGCTGATGAGGT
SRR1265421.28186333.1+   CACGAGGAACAGGAGCTGATGAGGT
SRR1265487.10026670.1-   CACGAGGAACAGGAGCTGATGAGGT
SRR1269689.606782.2+     CACGAGGAACAGGAGCTGATGAGGT
SRR1265493.2249139.2-    CACGAGGAACAGGAGCTGATGAGGTT
SRR1266963.4385495.1+    CACGAGGAACAGGAGCTGATGAGGTT
SRR1271664.7577323.2+    CACGAGGAACAGGAGCTGATGAGGTT
SRR1265421.28387803.2+   CACGAGGAACAGGAGCTGATGAGGTTG
SRR1266950.11307404.1-   CACGAGGAACAGGAGCTGATGAGGTTGA
SRR1271639.17341141.1+   CACGAGGAACAGGAGCTGATGAGGTTGA
SRR1265092.2139034.1-    CACGAGGAACAGGAGCTGATGAGGTTGAG
SRR1265101.2621422.2-    CACGAGGAACAGGAGCTGATGAGGTTGAG
SRR1266960.4673203.1-    CACGAGGAACAGGAGCTGATGAGGTTGAG
SRR1269689.10802270.2+   CACGAGGAACAGGAGCTGATGAGGATGAG
SRR1269690.6701197.1+    CACGAGGAACAGGAGCTGATGAGGTTGAG
SRR1269691.5900866.2+    CACGAGGAAAAGGAGCTGATGAGGTTGAG
SRR1269698.4930714.1-    CACGAGGAACAGGAGCTGATGAGGTTGAG
SRR1271616.4976712.2+    CACGAGGAACAGGAGCTGATGAGGTTGAG
SRR1271618.649812.2+     CACGAGGAACAGGAGCTGATGAGGTTGAG
SRR1265427.12651809.2-   CACGAGGAACAGGAGCTGATGAGGTTGAGC
SRR1265427.6605459.1+    CACGAGGAACAGGAGCTGATGAGGTTGAGC
SRR1266950.4890096.2-    CACGAGGAACAGGAGCTGATGAGGTTGAGC
SRR1271631.56868617.2-   CACGAGGAACAGGAGCTGATGAGGTTGAGC
SRR1266962.4519620.1-    CACGAGGAACAGGAGCTGATGAGGTTGAGCTG
```

SRR1271661.2368772.1+ CACGAGGAACAGGAGCAGATGAGGTTGAGCTG
SRR1265424.4163374.2+ CACGAGGAACAGGAGCTGATGAGGTTGAGCTGC
SRR1266949.1088605.1- CACGAGGAACAGGAGCTGATGAGGTTGAGCTGC
SRR1269690.8024392.2+ CACGAGGAACAGGAGCTGATGAGGTTGAGCTGC
SRR1271623.12702077.1+ CACGAGGAACAGGAGCTGATGAGGTTGAGCTGC
SRR1271660.66535.1- CACGAGGAACAGGAGCTGATGAGGTTGAGCTGCCC
SRR1265107.6644656.1- CACGAGGAACAGGAGCTGATGAGGTTGAGCTGCACA
SRR1271631.25901633.1+ CACGAGGAACAGGAGCTGATGAGGTTGAGCTGCACA
SRR1271644.4072249.1- CACGAGGAACAGGAGCTGATGAGGTTGAGCTGCACA
SRR1265430.10892354.1+ CACGAGGAACAGGAGCTGATGAGGTTGAGCTGCACAT
SRR1266962.2111129.1- CACGAGGAACAGGAGCTGATGAGGTTGAGCTGCACAT
SRR1269695.709605.2- CACGAGGAACAGGAGCTGATGAGGTTGAGCTGCACAT
SRR1271605.5228759.1+ CACGAGGAACAGGAGCTGATGAGGTTGAGCTGCACAT
SRR1269695.5539655.2+ CACGAGGAACAGGAGCTGATGAGGTTGAGCTGCCCATC
SRR1271624.11221728.1- CACGAGGAACAGGAGCTGATGAGGTTGAGCTGCCCATC
SRR1265116.6928798.1+ CACGAGGAACAGGAGCTGATGAGGTTGAGCTGCACATCC
SRR1265419.2667566.2- CACGAGGAACAGGAGCTGATGAGGTTGAGCTGCACATCC
SRR1265488.7425864.1- CACGAGGAACAGGAGCTGATGAGGTTGAGCTGCCCATCC
SRR1269697.3110262.1+ CACGAGGAACAGGAGCTGATGAGGTTGAGCTGCACATCC
SRR1271617.81306830.2+ CACGAGGAACAGGAGCTGATGAGGTTGAGCTGCACATCC
SRR1265490.7959942.2+ CACGAGGAACAGGAGCTGATGAGGTTGAGCTGCACATCCA
SRR1265960.6622381.1+ CACGAGGAACAGGAGCTGATGAGGTTGAGCTGCCCATCCA
SRR1271664.7610027.1+ CACGAGGAACAGGAGCTGATGAGGTTGAGCTGCCCATCCA
SRR1265429.2980691.2- CACGAGGAACAGGAGCTGATGAGGTTGAGCTGCACATCCAC
SRR1265485.4295901.2- CACGAGGAACAGGAGCTGATGAGGTTGAGCTGCCCATCCAC
SRR1271629.16583.1- CACGAGGAACAGGAGCTGATGAGGTTGAGCTGCCCATCCAC
SRR1271617.94327348.2+ CACGAGGAACAGGAGCTGATGAGGTTGAGCTGCACATCCACA
SRR1271631.77846051.2- CACGAGGAACAGGAGCTGACGAGGTTGAGCTGCCCATCCACA
SRR1265118.3539444.2- CACGAGGAACAGGAGCTGATGAGGTTGAGCTGCACATCCACAC
SRR1269701.5896733.2- CACGAGGAACAGGAGCTGATGAGGTTGAGCTGCACATCCACAC
SRR1269722.10861522.2+ CACGAGGAACAGGAGCTGATGAGGTTGAGCTGCACATCCACAC
SRR1265117.1014705.1- CACGAGGAACAGGAGCTGATGAGGTTGAGCTGCACATCCACACA
SRR1266963.7668173.1+ CACGAGGAACAGGAGCTGATGAGGTTGAGCTGCACATCCACACA
SRR1271606.19163420.1+ CACGAGGAACAGGAGCTGATGAGGTTGAGCTGAACATCCACACA
SRR1271611.41723090.1- CACGAGGAACAGGAGCTGATGAGGTTGAGCTGCACATCCACACA
SRR1271660.27380513.2- CACGAGGAACAGGAGCTGATGAGGTTGAGCTGCACATCCACACA
SRR1266959.4156269.1+ CACGAGGAACAGGAGCTGATGAGGTTGAGCTGCCCATCCACACAA
SRR1271620.8170287.1- CACGAGGAACAGGAGCTGATGAGGTTGAGCTGCACATCCACACAA
SRR1271625.17771509.1- CACGAGGAACAGGAGCTGATGAGGTTGAGCTGCCCATCCACACAA
SRR1271630.7036120.2- CACGAGGAACAGGAGCTGATGAGGTTGAGCTGCACATCCACACAA
SRR1265959.16039523.1- CACGAGGAACAGGAGCTGATGAGGTTGAGCTGCCCATCCACACAAC
SRR1271656.12001727.1- CACGAGGAACAGGAGCTGATGAGGTTGAGCTGCACATCCACACAAC
SRR1271656.38998198.1+ CACGAGGAACAGGAGCTGATGAGGTTGAGCTGCACATCCACACAAC
SRR1271635.5444837.2- CACGAGGAACAGGAGCTGATGAGGTTGAGCTGCACATCCACACAACT
SRR1265102.11790677.1+ CACGAGGAACAGGAGCTGATGAGGTTGAGCTGCCCATCCACACAACTG
SRR1271631.9620063.1+ CACGAGGAACAGGAGCTGATGAGGTTGAGCTGCACATCCACACAACTG
SRR1269700.230054.1- CACGAGGAACAGGAGCTGATGAGGTTGAGCTGCACATCCACACAACTGG
SRR1269726.6324567.1+ CACGAGGAACAGGAGCTGATGAGGTTGAGCTGCACATCCACACAACTGG
SRR1265423.4843600.2+ CACGAGGAACAGGAGCTGATGAGGTTGAGCTGCCCATCCACACAACTGGC
SRR1266955.9100423.2+ CACGAGGAACAGGAGCTGATGAGGTTGAGCTGCCAATCCACACAACTGGC
SRR1266961.12229708.1- CACGAGGAACAGGAGCTGATGAGGTTGAGCTGCCCATCCACACAACTGGC
SRR1271635.8499862.2+ CACGAGGAACAGGAGCTGATGAGGTTGAGCTGCCCATCCACACAACTGGC
SRR1266963.15703496.1- CACGAGGAACAGGAGCTGATGAGGTTGAGCTGCACATCCACACAACTGGCT
SRR1269699.10804333.2- CACGAGGAACAAGAGCTGATGAGGTTGAGCTGCACATCCACACAACTGGCT
SRR1271633.8205548.2- CACGAGGAACAGGAGCTGATGAGGTTGAGCTGCCCATCCACACAACTGGCT
SRR1271637.6456421.1+ CACGAGGAACAGGAGCTGATGAGGTTGAGCTGCACATCCACACAACTGGCTG
SRR1266952.10330331.1+ CACGAGGAACAGGAGCTGATGAGGTTGAGCTGCACATCCACACAACTGGCTGG
SRR1271614.6488833.1- CACGAGGAACAGGAGCTGATGAGGTTGAGCTGCACATCCACACAACTGGCTGG
SRR1271617.92587535.2- CACGAGGAACAGGAGCTGATGAGGTTGAGCTGCACATCCACACAACTGGCTGG
SRR1265102.5685651.1- CACGAGGAACAGGAGCTGATGAGGTTGAGCTGCACATCCACACAACTGGCTGGA
SRR1265424.13766781.2+ CACGAGGAACAGGAGCTGATGAGGTTGAGCTGCACATCCACACAACTGGCTGGA
SRR1269725.3278979.1+ CACGAGGAACAGGAGCTGATGAGGTTGAGCTGCACATCCACACAACTGGCTGGA
SRR1271630.7776502.2+ CACAAGGAACAGGAGCTGACGAGGTTGAGCTGCACATCCACACAACTGGCTGGA
SRR1266952.1463982.2- CACGAGGACCAGGATCTGATGAGGTTGAGCTGCACATCCACACAACTGGCTGGAT
SRR1266963.9363809.1+ CACGAGGAACAGGAGCTGATGAGGTTGAGCTGCCCATCCACACAACTGGCTGGAT
SRR1269692.5539705.1+ CACGAGGAACAGGAGCTGATGAGGTTGAGCTGCACATCCACACAACTGGCTGGAT
SRR1271622.6836463.2- CACGAGGAACAGGAGCTGATGAGGTTGAGCTGCCCATCCACACAACTGGCTGGAT
SRR1271625.3981531.1+ CACGAGGAACAGGAGCTGATGAGGTTGAGCTGCCCATCCACACAACTGGCTGGAT
SRR1271626.8342312.2+ CACGAGGAACAGGAGCTGATGAGGTTGAGCTGCCCATCCACACAACTGGCTGGAT
SRR1271628.3667565.2+ CACGAGGAACAGGAGCTGATGAGGTTGAGCTGCCCATCCACACAACTGGCTGGAT
SRR1271656.28111822.1- CACGAGGAACAGGAGCTGATGAGGTTGAGCTGCACATCCACACAACTGGCTGGAT
SRR1266951.4137283.1+ CACGAGGAACAGGAGCTGATGAGGTTGAGCTGCACATCCACAAAACTGGCTGGATG
SRR1271611.42557115.2- CACGAGGAACAGGAGCTGATGAGGTTGAGCTGCACATCCACACAACTGGCTGGATG
SRR1271617.101746624.1- CACGAGGAACAGGAGCTGATGAGGTTGAGCTGCACATCCACACAACTGGCTGGATG
SRR1265961.2742222.1- CACGAGGAACAGGAGCTGATGAGGTTGAGCTGCCCATCCACACAACTGGCTGGATGG
SRR1271614.8837036.1+ CACGAGGAACAGGAGCTGATGAGGTTGAGCTGCACATCCACACAACTGGCTGGATGG
SRR1265421.19234411.1- CACGAGGAACAGGAGCTGATGAGGTTGAGCTGCACATCCACACAACTGGCTGGATGGC
SRR1271617.32725188.2- CACGAGGAACAGGAGCTGATGAGGTTGAGCTGCACATCCACACAACTGGCTGGATGGC
SRR1271631.87281188.2- CACGAGGAACAGGAGCTGATGAGGTTGAGCTGCCCATCCACACAACTGGCTGGATGGC
SRR1265090.573951.1+ CACGAGGAACAGGAGCTGATGAGGTTGAGCTGCACATCCACACAACTGGCTGGATGGCAT
SRR1265091.7293804.1- CACGAGGAACAGGAGCTGATGAGGTTGAGCTGCACATCCACACAACTGGCTGGATGGCAT
SRR1265094.9258610.2- CACGAGGAACAGGAGCTGATGAGGTTGAGCTGCACATCCACACAACTGGCTGGATGGCAT
SRR1265100.6825972.2+ CACGAGGAACAGGAGCTGATGAGGTTGAGCTGCCCATCCACACAACTGGCTGGATGGCAT
SRR1265100.7029791.2- CACGAGGAACAGGAGCTGATGAGGTTGAGCTGCCCATCCACACAACTGGCTGGATGGCAT

SRR1265101.7863435.2+   CACGAGGAACAGGAGCTGATGAGGTTGAGCTGCACATCCACACAACTGGCTGGATGGCAT
SRR1265103.11024313.1+   CACGAGGAACAGGAGCTGATGAGGTTGAGCTGCCCATCCACACAACTGGCTGGATGGCAT
SRR1265103.9204126.1-   CACGAGGAACAGGAGCTGATGAGGTTGAGCTGCACATCCACACAACTGGCTGGATGGCAT
SRR1265107.8184323.2+   CACGAGGAACAGGAGCTGATGAGGTTGAGCTGCACATCCACACAACTGGCTGGATGGCAT
SRR1265107.9762562.1+   CACGAGGAACAGGAGCTGATGAGGTTGAGCTGCACATCCACACAACTGGCTGGATGGCAT
SRR1265108.11287257.2+   CACGAGGAACAGGAGCTGATGAGGTTGAGCTGCACATCCACACAACTGGCTGGATGGCAT
SRR1265108.5336322.1+   CACGAGGAACAGGAGCTGATGAGGTTGAGCTGCACATCCACACAACTGGCTGGATGGCAT
SRR1265116.2215467.2+   CACGAGGAACAGGAGCTGATGAGGTTGAGCTGCACATCCACACAACTGGCTGGATGGCAT
SRR1265118.3202418.2+   CACGAGGAACAGGAGCTGATGAGGTTGAGCTGCACATCCACACAACTGGCTGGATGGCAT
SRR1265118.8469183.1+   CACGAGGAACAGGAGCTGATGAGGTTGAGCTGCACATCCACACAACTGGCTGGATGGCAT
SRR1265119.2075759.2+   CACGAGGAACAGGAGCTGATGAGGTTGAGCTGCACATCCACACAACTGGCTGGATGGCAT
SRR1265120.10380681.2+   CACGAGGAACAGGAGCTGATGAGGTTGAGCTGCACATCCACACAACTGGCTGGATGGCAT
SRR1265419.5834061.2-   CACGAGGAACAGGAGCTGATGAGGTTGAGCTGCACATCCACACAACTGGCTGGATGGCAT
SRR1265420.6477611.2+   CACGAGGAACAGGAGCTGATGAGGTTGAGCTGCACATCCACACAACTGGCTGGATGGCAT
SRR1265424.14641649.2+   CACGAGGAACAGGAGCTGATGAGGTTGAGCTGCACATCCACACAACTGGCTGGATGGCAT
SRR1265424.825035.1+   CACGAGGAACAGGAGCTGATGAGGTTGAGCTGCACATCCACACAACTGGCTGGATGGCAT
SRR1265425.11836318.2+   CACGAGGAACAGGAGCTGATGAGGTTGAGCTGCCCATCCACACAACTGGCTGGATGGCAT
SRR1265427.10426011.1+   CACGAGGAACAGGAGCTGATGAGGTTGAGCTGCACATCCACACAACTGGCTGGATGGCAT
SRR1265427.13084996.1+   CACGAGGAACAGGAGCTGATGAGGTTGAGCTGCCCATCCACACAACTGGCTGGATGGCAT
SRR1265430.3999536.2-   CACGAGGAACAGGAGCTGATGAGGTTGAGCTGCACATCCACACAACTGGCTGGATGGCAT
SRR1265430.9435022.1-   CACGAGGAACAGGAGCTGATGAGGTTGAGCTGCACATCCACACAACTGGCTGGATGGCAT
SRR1265485.927570.2+   CACGAGGAACANGAGCTGATGAGGTTGAGCTGCCCATCCACACAACTGGCTGGATGGCAT
SRR1265486.2988089.2-   CACGAGGAACAGGAGCTGATGAGGTTGAGCTGCACATCCACACAACTGGCTGGATGGCAT
SRR1265486.4816291.2+   CACGAGGAACAGGAGCTGATGAGGTTGAGCTGCCGATCCACACAACTGGCTGGATGGCAT
SRR1265486.9625524.2+   CACGAGGAACAGGAGCTGATGAGGTTGAGCTGCCCATCCACACAACTGGCTGGATGGCAT
SRR1265487.3611936.2-   CACGAGGAACAGGAGCTGATGAGGTTGAGCTGCCCATCCACACAACTGGCTGGATGGCAT
SRR1265487.5948289.1+   CACGAGGAACAGGAGCTGATGAGGTTGAGCTGACCATCCACACAACTGGCTGGATGGCAT
SRR1265490.12123742.2-   CACGAGGAACAGGAGCTGATGAGGTTGAGCTGCACATCCACACAACTGGCTGGATGGCAT
SRR1265490.1647534.2+   CACGAGGAACAGGAGCTGATGAGGTTGAGCTGCACATCCACACAACTGGCTGGATGGCAT
SRR1265490.5635851.1+   CACGAGGAACAGGAGCTGATGAGGTTGAGCTGCACATCCACACAACTGGCTGGATGGCAT
SRR1265490.7565460.2+   CACGAGGAACAGGAGCTGATGAGGTTGAGCTGCACATCCACACAACTGGCTGGATGGCAT
SRR1265494.10552009.1+   CACGAGGAACAGGAGCTGATGAGGTTGAGCTGCACATCCACACAACTGGCTGGATGGCAT
SRR1265960.6641003.1-   CACGAGGAACAGGAGCTGATGAGGTTGAGCTGCCCATCCACACAACTGGCTGGATGGCAT
SRR1266946.1503677.1-   CACGAGGAACAGGAGCTGATGAGGTTGAGCTGCACATCCACACAACTGGCTGGATGGCAT
SRR1266946.22292515.1-   CACGAGGAACAGGAGCTGATGAGGTTGAGCTGCACATCCACACAACTGGCTGGATGGCAT
SRR1266947.1797376.1+   CACGAGGAACAGGAGCTGATGAGGTTGAGCTGCACATCCACACAACTGGCTGGATGGCAT
SRR1266948.20521025.2-   CACGAGGAACAGGAGCTGATGAGGTTGAGCTGCCCATCCACACAACTGGCTGGATGGCAT
SRR1266948.4974750.1+   CACGAGGAACAGGAGCTGATGAGGTTGAGCTGCCCATCCACACAACTGGCTGGATGGCAT
SRR1266951.1123610.1+   CACGAGGAACAGGAGCTGATGAGGTTGAGCTGCACATCCACACAACTGGCTGGATGGCAT
SRR1266952.10100495.1-   CACGAGTAACAGGAGCTGATGAGGTTGAGCTGCACATCCACACAACTGGCTGGATGGCAT
SRR1266959.12652072.2-   CACGAGGAACAGGAGCTGATGAGGTTGAGCTGCACATCCACACAACTGGCTGGATGGCAT
SRR1266959.1925156.1+   CACGAGGAACAGGAGCTGATGAGGTTGAGCTGCACATCCACACAACTGGCTGGATGGCAT
SRR1266959.6559141.2+   CACGAGGAACAGGAGCTGATGAGGTTGAGCTGCACATCCACACAACTGGCTGGATGGCAT
SRR1266962.10797533.1+   CACGAGGAACAGGAGCTGATGAGGTTGAGCTGCCCATCCACACAACTGGCTGGATGGCAT
SRR1266962.3051106.2+   CACGAGGAACAGGAGCTGATGAGGTTGAGCTGCACATCCACACAACTGGCTGGATGGCAT
SRR1266962.8268942.2-   CACGAGGAACAGGAGCTGATGAGGTTGAGCTGCCCATCCACACAACTGGCTGGATGGCAT
SRR1266962.933875.2-   CACGAGGAACAGGAGCTGATGAGGTTGAGCTGCACATCCACACAACTGGCTGGATGGCAT
SRR1266963.14618912.2+   CACGAGGAACAGGAGCTGATGAGGTTGAGCTGCACATCCACACAACTGGCTGGATGGCAT
SRR1266964.9683545.2+   CACGAGGAACAGGAGCTGATGAGGTTGAGCTGCACATCCACACAACTGGCTGGATGGCAT
SRR1269689.11150721.2+   CACGAGGAACAGGAGCTGATGAGGTTGAGCTGCACATCCACACAACTGGCTGGATGGCAT
SRR1269689.1433856.2+   CACGAGGAACAGGAGCTGATGAGGTTGAGCTGCACATCCACACAACTGGCTGGATGGCAT
SRR1269689.9751428.2+   CACGAGGAACAGGAGCTGATGAGGTTGAGCTGCACATCCACACAACTGGCTGGATGGCAT
SRR1269691.4799971.1+   CACGAGGAACAGGAGCTGATGAGGTTGAGCTGCACATCCACACAACTGGCTGGATGGCAT
SRR1269693.657451.2-   CACGAGGAACAGGAGCTGATGAGGTTGAGCTGCACATCCACACAACTGGCTGGATGGCAT
SRR1269694.6400404.1+   CACGAGGAACAGGAGCTGATGAGGTTGAGCTGCCCATCCACACAACTGGCTGGATGGCAT
SRR1269695.4605288.1-   CACGAGGAACAGGAGCTGATGAGGTTGAGCTGCACATCCACACAACTGGCTGGATGGCAT
SRR1269701.8482472.1+   CACGAGGAACAGGAGCTGATGAGGTTGAGCTGCACATCCACACAACTGGCTGGATGGCAT
SRR1269701.962293.1+   CACGAGGAACAGGAGCTGATGAGGTTGAGCTGCACATCCACACAACTGGCTGGATGGCAT
SRR1269702.9206585.1-   CACGAGGAACAGGAGCTGATGAGGTTGAGCTGCACATCCACACAACTGGCTGGATGGCAT
SRR1269720.8521946.1-   CACGAGGAACAGGAGCTGATGAGGTTGAGCTGCACATCCACACAACTGGCTGGATGGCAT
SRR1269722.8669002.1+   CACGAGGAACAGGAGCTGATGAGGTTGAGCTGCACATCCACACAACTGGCTGGATGGCAT
SRR1269723.12879211.1+   CACGAGGAACAGGAGCTGATGAGGTTGAGCTGCACATCCACACAACTGGCTGGATGGCAT
SRR1269724.1613007.2+   CACGAGGAACAGGAGCTGATGAGGTTGAGCTGCACATCCACACAACTGGCTGGATGGCAT
SRR1269724.2351101.2-   CACGAGGAACAGGAGCTGATGAGGTTGAGCTGCACATCCACACAACTGGCTGGATGGCAT
SRR1269724.7736550.2-   CACGAGGAACAGGAGCTGATGAGGTTGAGCTGCACATCCACACAACTGGCTGGATGGCAT
SRR1269724.7878642.2-   CACGAGGAACAGGAGCTGATGAGGTTGAGCTGCACATCCACACAACTGGCTGGATGGCAT
SRR1269725.4823828.2+   CACGAGGAACAGGAGCTGATGAGGTTGAGCTGCACATCCACACAACTGGCTGGATGGCAT
SRR1269725.9198123.1+   CACGAGGAACAGGAGCTGATGAGGTTGAGCTGCACATCCACACAACTGGCTGGATGGCAT
SRR1269726.4838196.2-   CACGAGGAACAGGAGCTGATGAGGTTGAGCTGCACATCCACACAACTGGCTGGATGGCAT
SRR1269726.8967180.1-   CACGAGGAACAGGAGCTGATGAGGTTGAGCTGCACATCCACACAACTGGCTGGATGGCAT
SRR1269727.8758172.1+   CACGAGGAACAGGAGCTGATGAGGTTGAGCTGCACATCCACACAACTGGCTGGATGGCAT
SRR1271605.19945219.1+   CACGAGGAACAGGAGCTGATGCGGTTGAGCTGCACATCCACACAACTGGCTGGATGGCAT
SRR1271605.3457147.2+   CACGAGGAACAGGAGCTGATGAGGTTGAGCTGCACATCCACACAACTGGCTGGATGGCAT
SRR1271606.10370241.1+   CACGAGGAACAGGAGCTGATGAGGTTGAGCTGCACATCCACACAACTGGCTGGGTGGAAT
SRR1271611.32939716.2+   CACGAGGAACAGGAGCTGATGAGGTTGAGCTGCACATCCACACAACTGGCTGGATGGCAT
SRR1271611.35735582.1-   CACGAGGAACAGGAGCTGATGAGGTTGAGCTGCACATCCACACAACTGGCTGGATGGCAT
SRR1271611.45105264.1+   CACGAGGAACAGGAGCTGATGAGGTTGAGCTGCACATCCACACAACTGGCTGGATGGCAT
SRR1271614.7695441.1+   CACGAGGAACAGGAGCTGATGAGGTTGAGCTGCACATCCACACAACTGGCTGGATGGCAT
SRR1271617.106361713.1- CACGAGGAACAGGAGCTGATGAGGTTGAGCTGCACATCCACACAACTGGCTGGATGGCAT
SRR1271617.18340985.1-   CACGAGGAACAGGAGCTGATGAGGTTGAGCTGCACATCCACACAACTGGCTGGATGGCAT
SRR1271617.24478703.2+   CACGAGGAACAGGAGCTGATGAGGTTGAGCTGCACATCCACACAACTGGCTGGCTGGAAC
SRR1271617.29201909.1-   CACGAGGAACAGGAGCTGATGAGGTTGAGCTGCACATCCACACAACTGGCTGGATGGCAT
SRR1271617.7158444.2-   CACGAGGAACAGGAGCTGATGAGGTTGAGCTGCACATCCACACAACTGGCTGGATGGCAT
SRR1271617.87539143.2+   CACGAGGAACAGGAGCTGATGAGGTTGAGCTGCACATCCACACAACTGGCTGGATGGCAT

```
SRR1271618.8223150.2+    CACGAGGAACAGGAGCTGATGAGGTTGAGCTGCACATCCACACAACTGGCTGGATGGCAT
SRR1271623.14435698.2+   CACGAGGAACAGGAGCTGATGAGGTTGAGCTGCCCATCCACACAACTGGCTGGATGGCAT
SRR1271625.4440646.1-    CACGAGGAACAGGAGCTGATGAGGTTGAGCTGCCCATCCACACAACTGGCTGGATGGCAT
SRR1271625.4462362.2-    CACGAGGAACAGGAGCTGATGAGGTTGAGCTGCACATCCACACAACTGGCTGGATGGCAT
SRR1271625.4672091.1-    CACGAGGAACAGGAGCTGATGAGGTTGAGCTGCCCATCCACACAACTGGCTGGATGGCAT
SRR1271625.4974445.2-    CACGAGGAACAGGAGCTGATGAGGTTGAGCTGCCCATCCACACAACTGGCTGGATGGCAT
SRR1271626.27554490.1+   CACGAGGAACAGGAGCTGATGAGGTTGAGCTGCCCATCCACACAACTGGCTGGATGGCAT
SRR1271626.39100757.2+   CACGAGGAACAGGAGCTGATGAGGTTGAGCTGCCCATCCACACAACTGGCTGGATGGCAT
SRR1271626.39747880.2-   CACGAGGAACAGGAGCTGATGAGGTTGAGCTGCCCATCCACACAACTGGCTGGATGGCAT
SRR1271626.7870290.2+    CACGAGGAACAGGAGCTGATGAGGTTGAGCTGCACATCCACACAACTGGCTGGATGGCAT
SRR1271627.2433963.2+    CACGAGGAACAGGAGCTGATGAGGTTGAGCTGCCCATCCACACAACTGGCTGGATGGCAT
SRR1271628.1578656.2-    CACGAGGAACAGGAGCTGATGAGGTTGAGCTGCACATCCACACAACTGGCTGGATGGCAT
SRR1271631.38795764.1+   CACGAGGAACAGGAGCTGATGAGGTTGAGCTGCCCATCCACACAACTGGCTGGATGGCAT
SRR1271631.51412202.2-   CACGAGGAACAGGAGCTCATGAGCTTCAGCTGCCCATCCACACAACTGGCTGGATGGCAT
SRR1271631.73787149.2-   CACGAGGAACAGGAGCTGATGAGGTTGAGCTGCCCATCCACACAACTGGCTGGATGGCAT
SRR1271631.94542648.1+   CACGAGGAACAGGAGCTGATGAGGTTGAGCTGCACATCCACACAACTGGCTGGATGGCAT
SRR1271634.2225500.2-    CACGAGGAAAAGGAGCTGATGGTGTTGAGCTGCCCAACCACACAACTGGCTGGATGGGAT
SRR1271636.32970902.1-   CACGAGGAACAGGAGCTGATGAGGTTGAGCTGCACATCCACACAACTGGCTGGATGGCAT
SRR1271637.7730229.2-    CACGAGGAACAGGAGCTGATGAGGTTGAGCTGCACATCCACACAACTGGCTGGATGGCAT
SRR1271637.8462724.1-    CACGAGGAACAGGAGCTGATGAGGTTGAGCTGCCCATCCACACAACTGGCTGGATGGCAT
SRR1271637.9483811.1+    CACGAGGAACAGGAGCTGATGAGGTTGAGCTGCACATCCACACAACTGGCTGGATGGCAT
SRR1271638.10108003.1-   CACGAGGAACAGGAGCTGATGAGGTTGAGCTGCACATCCACACAACTGGCTGGATGGCAT
SRR1271641.1475015.2-    CACGAGGAACAGGAGCTGATGAGGTTGAGCTGCACATCCACACAACTGGCTGGATGGCAT
SRR1271643.11732587.1-   CACGAGGAACAGGAGCTGATGAGGTTGAGCTGCACATCCACACAACTGGCTGGATGGCAT
SRR1271643.6252990.1+    CACGAGGAACAGGAGCTGATGAGGTTGAGCTGCACATCCACACAACTGGCTGGATGGCAT
SRR1271656.16215479.1-   CACGAGGAACAGGAGCTGATGAGGTTGAGCTGCACATCCACACAACTGGCTGGATGGCAT
SRR1271656.18693034.2-   CACGAGGAACAGGAGCTGATGAGGTTGAGCTGCACATCCACACAACTGGCTGGATGGCAT
SRR1271656.19605949.2+   CACGAGGAACAGGAGCTGATGAGGTTGAGCTGCACATCCACACAACTGGCTGGATGGCAT
SRR1271656.19755138.1+   CACGAGGAACAGGAGCTGATGAGGTTGAGCTGCACATCCACACAACTGGCTGGATGGCAT
SRR1271656.27642211.2-   CACGAGGAACAGGAGCTGATGAGGTTGAGCTGCACATCCACACAACTGGCTGGATGGCAT
SRR1271656.38998198.2-   CACGAGGAACAGGAGCTGATGAGGTTGAGCTGCACATCCACACAACTGGCTGGATGGCAT
SRR1271656.4976182.2+    CACGAGGAACAGGAGCTGATGAGGTTGAGCTGCACATCCACACAACTGGCTGGATGGCAT
SRR1271660.19489729.2-   CACGAGGAACAGGAGCTGATGAGGTTGAGCTGCACATCCACACAACTGGCTGGATGGCAT
SRR1271660.7768350.2+    CACGAGGAACAGGAGCTGATGAGGTTGAGCTGCACATCCACACAACTGGCTGGATGGCAT
SRR1271663.9467739.2+    CACGAGGAACAGGAGCTGATGAGGTTGAGCTGCCCATCCACACAACTGGCTGGATGGCAT
SRR1271663.9621731.2+    CACGAGGAACAGGAGCTGATGAGGTTGAGCTGCCCATCCACACAACTGGCTGGATGGCAT
SRR1271666.9606214.1-    CACGAGGAACAGGAGCTGATGAGGTTGAGCTGCACATCCACACAACTGGCTGGATGGCAT
SRR1266966.10206312.2+    ACGAGGAACAGGAGCTGATGAGGTTGAGCTGCACATCCACACAACTGGCTGGATGGCAT
SRR1271617.46091166.2-    ACGAGGAACAGGAGCTGATGAGGTTGAGCTGCACATCCACACAACTGGCTGGATGGCAT
SRR1271626.2595264.2+     ACGAGGAACAGGAGCTGATGAGGTTGAGCTGCCCATCCACACAACTGGCTGGATGGCAT
SRR1271631.36011479.1+    ACGAGGAACAGGAGCTGATGAGGTTGAGCTGCACATCCACACAACTGGCTGGATGGCAT
SRR1271638.17228052.2+    ACGAGGAACAGGAGCTGATGAGGTTGAGCTGCCCATCCACACAACTGGCTGGATGGCAT
SRR1265118.2132389.2+      CGAGGAACAGGAGCTGATGAGGTTGAGCTGCACATCCACACAACTGGCTGGATGGCAT
SRR1269693.1981773.1+      CGAGGAACAGGAGCTGATGAGGTTGAGCTGCACATCCACACAACTGGCTGGATGGCAT
SRR1271617.15492594.1-     CGAGGAACAGGAGCTGATGAGGTTGAGCTGCACATCCACACAACTGGCTGGATGGCAT
SRR1271624.3358682.2-      CGAGGAACAGGAGCTGATGAGGTTGAGCTGCCCATCCACACAACTGGCTGGATGGCAT
SRR1265424.7293.2+          GAGGAACAGGAGCTGATGAGGTTGAGCTGCCCATCCACACAACTGGCTGGATGGCAT
SRR1266948.19095128.2+      GAGGAACAGGAGCTGATGAGGTTGAGCTCCCCATCCACACAACTGGCTGGATGGCAT
SRR1266957.3666759.2-       GAGGAACAGGAGCTGATGAGGTTGAGCTGCACATCCACACAACTGGCTGGATGGCAT
SRR1269700.1764719.2-       GAGGAACAGGAGCTGATGAGGTTGAGCTGCACATCCACACAACTGGCTGGATGGCAT
SRR1271617.12164168.1+      GAGGAACAGGAGCTGATGAGGTTGAGCTGCACATCCACACAACTGGCTGGATGGCAT
SRR1271621.2349329.1+       GAGGAACAGGAGCTGATGAGGTTGAGCTGCACATCCACACAACTGGCTGGATGGCAT
SRR1271631.60122186.1+      GAGGAACAGGAGCTGATGAGGTTGAGCTGCACATCCACACAACTGGCTGGATGGCAT
SRR1271631.75576578.1-      GAGGAACAGGAGCTGATGAGGTTGAGCTGCCCATCCACACAACTGGCTGGATGGCAT
SRR1265107.9747432.1-        AGGAACAGGAGCTGATGAGGTTGAGCTGCACATCCACACAACTGGCTGGATGGCAT
SRR1265428.3438079.1+        AGGAACAGGAGCTGATGAGGTTGAGCTGCACATCCACACAACTGGCTGGATGGCAT
SRR1265487.9618712.2+        AGGAACAGGAGCTGATGAGGTTGAGCTGCACATCCACACAACTGGCTGGATGGCAT
SRR1266960.47540.2-          AGGAACAGGAGCTGATGAGGTTGAGCTGCACATCCACACAACTGGCTGGATGGCAT
SRR1271617.99985877.2+       AGGAACAGGAGCTGATGAGGTTGAGCTGCACATCCACACAACTGGCTGGATGGCAT
SRR1265418.2535443.1+          GAACAGGAGCTGATGAGGTTGAGCTGCACATCCACACAACAGGCTGGATGGCAT
SRR1265486.11495028.2+         GAACAGGAGCTGATGAGGTTGAGCTGCCCATCCACACAACTGGCTGGATGGCAT
SRR1269702.3793239.2-           AACAGGAGCTGATGAGGTTGAGCTGCACATCCACACAACTGGCTGGATGGCAT
SRR1271617.46820496.2-          AACAGGAGCTGATGAGGTTGAGCTGCACATCCACACAACTGGCTGGATGGCAT
SRR1271617.81204720.2+          AACAGGAGCTGATGAGGTTGAGCTGCACATCCACACAACTGGCTGGATGGCAT
SRR1271640.16324249.2+          AACAGGAGCTGATGAGGTTGAGCTGCACATCCACACAACTGGCTGGATGGCAT
SRR1271656.37410994.2-          AACAGGAGCTGATGAGGTTGAGCTGCACATCCACACAACTGGCTGGATGGCAT
SRR1265106.3654183.1+             CAGGAGCTGATGAGGTTGAGCTGCACATCCACACAACTGGCTGGATGGCAT
SRR1265428.3817667.1+             CAGGAGCTGATGAGGTTGAGCTGCCCATCCACACAACTGGCTGGATGGCAT
SRR1265429.2745098.2+              AGGAGCTGATGAGGTTGAGCTGCCCATCCACACAACTGGCTGGATGGCAT
SRR1271639.6412786.2+              AGGAGCTGATGAGGTTGAGCTGCACATCCACACAACTGGCTGGATGGCAT
SRR1271656.50764191.2+             AGGAGCTGATGAGGTTGAGCTGCACATCCACACAACTGGCTGGATGGCAT
SRR1265421.1577740.1+               GGAGCTGATGAGGTTGAGCTGCACATCCACACAACTGGCTGGATGGCAT
SRR1265959.4685497.1-               GGAGCTGATGAGGTTGAGCTGCCCATCCACACAACTGGCTGGATGGCAT
SRR1266946.20413741.1+              GGAGCTGATGAGGTTGAGCTGCCCATCCACACAACTGGCTGGATGGCAT
SRR1266948.20676083.1-              GGAGCTGATGAGGTTGAGCTGCCCATCCACACAACTGGCTGGATGGCAT
SRR1271611.58180054.2-              GGAGCTGATGAGGTTGAGCTGCACATCCACACAACTGGCTGGATGGCAT
SRR1269721.4085403.1-                GAGCTGATGAGGTTGAGCTGCACATCCACACAACTGGCTGGATGGCAT
SRR1271616.7303942.1-                 AGCTGATGAGGTTGAGCTGCACATCCACACAACTGGCTGGATGGCAT
SRR1271617.43257682.2-                AGCTGATGAGGTTGAGCTGCACATCCACACAACTGGCTGGATGGCAT
SRR1266952.5487495.1+                  GCTGATGAGGTTGAGCTGCACATCCACACAACTGGCTGGATGGCAT
SRR1266964.9267740.1-                  GCTGATGAGGTTGAGCTGCCCATCCAAACAAATGGCTGGATGGCAT
SRR1265962.10995514.2+                  CTGATGAGGTTGAGCTGCCCATCCACACAACTGGCTGGATGGCAT
SRR1266948.1989161.1-                   CTGATGAGGTTGAGCTGCCCATCCACACAACTGGCTGGATGGCAT
SRR1269720.2054067.1+                   CTGATGAGGTTGAGCTGCACATCCACACAACTGGCTGGATGGCAT
```

SRR1271617.70351078.2-	CTGATGAGGTTGAGCTGCACATCCACACAACTGGCTGGATGGCAT
SRR1265493.861628.2-	TGATGAGGTTGAGCTGCACATCCACACAACTGGCTGGATGGCAT
SRR1266956.10452827.2+	TGATGAGGTTGAGCTGCACATCCACACAACTGGCTGGATGGCAT
SRR1269692.5660364.2+	TGATGAGGTTGAGCTGCACATCCACACAACTGGCTGGATGGCAT
SRR1271606.11910612.2+	TGATGAGGTTGAGCTGCACATCCACACAACTGGCTGGATGGCAT
SRR1271618.3593897.1+	TGATGAGGTTGAGCTGCACATCCACACAACTGGCTGGATGGCAT
SRR1271631.70543548.2+	TGATGAGGTTGAGCTGCCCATCCACACAACTGGCTGGATGGCAT
SRR1271632.1398829.2+	TGATGAGGTTGAGCTGCCCATCCACACAACTGGCTGGATGGCAT
SRR1271640.643478.1+	TGATGAGGTTGAGCTGCCCATCCACACAACTGGCTGGATGGCAT
SRR1271644.8110496.1+	GATGAGGTTGAGCTGCACATCCACACAACTGGCTGGATGGCAT
SRR1271617.89166641.1+	ATGAGGTTGAGCTGCACATCCACACAACTGGCTGGATGGCAT
SRR1266956.8706797.2+	TGAGGTTGAGCTGCACATCCACACAACTGGCTGGATGGCAT
SRR1265107.10559184.2+	GAGGTTGAGCTGCACATCCACACAACTGGCTGGATGGCAT
SRR1269727.8680671.1+	GAGGTTGAGCTGCACATCCACACAACTGGCTGGATGGCAT
SRR1271617.76678834.2-	GAGGTTGAGCTGCACATCCACACAACTGGCTGGATGGCAT
SRR1265494.8124625.1-	AGGTTGAGCTGCACATCCACACAACTGGCTGGATGGCAT
SRR1271636.10089274.2+	AGGTTGAGCTGCCCATCCACACAACTGGCTGGATGGCAT
SRR1271643.6397.1+	AGGTTGAGCTGCACATCCACACAACTGGCTGGATGGCAT
SRR1265429.12762650.1+	GGTTGAGCTGCACATCCACACAACTGGCTGGATGGCAT
SRR1265488.2054623.2+	GGTTGAGCTGCACATCCACACAACTGGCTGGATGGCAT
SRR1265962.6107415.1+	GGTTGAGCTGCCCATCCACACAACTGGCTGGATGGCAT
SRR1266949.6573322.2+	GGTTGAGCTGCCCATCCACACAACTGGCTGGGTGGCAT
SRR1266966.3487586.2-	GGTTGAGCTGCACAACCACACAACTGGCTGGATGGCAT
SRR1271637.17744220.1+	GGTTGAGCTGCACATCCACACAACTGGCTGGATGGCAT
SRR1271637.17744220.2-	GGTTGAGCTGCACATCCACACAACTGGCTGGATGGCAT
SRR1265106.5466905.1+	TTGAGCTGCACATCCACACAACTGGCTGGATGGCAT
SRR1266960.6111811.2-	TTGAGCTGCACATCCACACAACTGGCTGGATGGCAT
SRR1271656.23576076.2+	TTGAGCTGCACATCCACACAACTGGCTGGATGGCAT
SRR1271626.7548380.2+	TGAGCTGCACATCCACACAACTGGCTGGATGGCAT
SRR1271631.42268717.2+	TGAGCTGCACATCCACACAACTGGCTGGATGGCAT
SRR1271644.5773655.2+	TGAGCTGCACATCCACACAACTGGCTGGATGGCAT
SRR1266957.2266659.2+	GAGCTGCACATCCACACAACTGGCTGGATGGCAT
SRR1271616.2239278.1-	GAGCTGCACATCCACACAACTGGCTGGATGGCAT
SRR1265429.617391.2+	AGCTGCACATCCACACAACTGGCTGGATGGCAT
SRR1271617.2641915.2-	AGCTGCACATCCACACAACTGGCTGGATGGCAT
SRR1271626.10299387.2-	AGCTGCACATCCACACAACTGGCTGGATGGCAT
SRR1265430.11367129.2-	GCTGCCCATCCACACAACTGGCTGGATGGCAT
SRR1269691.6146778.1+	GCTGCACATCCACACAACTGGCTGGATGGCAT
SRR1269723.7787279.1-	GCTGCACATCCACACAACTGGCTGGATGGCAT
SRR1271631.37762412.1+	GCTGCCCATCCACACAACTGGCTGGATGGCAT
SRR1271656.13812009.2-	GCTGCACATCCACACAACTGGCTGGATGGCAT
SRR1271660.204492.2-	GCTGCCCATCCACACAACTGGCTGGATGGGAT
SRR1271661.7818147.2-	GCTGCCCGTCCACACAACTGGCTGGATGGCAT
SRR1265961.17164072.1-	CTGCCCATCCACACAACTGGCTGGATGGCAT
SRR1266956.11010848.2+	CTGCACATCCACACAACTGGCTGGATGGCAT
SRR1269723.7352283.2+	CTGCACATCCACACAACTGGCTGGATGGCAT
SRR1271606.4403075.1+	CTGCACATCCACACAACTGGCTGGATGGCAT
SRR1271611.8284106.2-	CTGCACATCCACACAACTGGCTGGATGGCAT
SRR1271626.15519384.1-	CTGCACATCCACACAACTGGCTGGATGGCAT
SRR1271631.30275332.1+	CTGCACATCCACACAACTGGCTGGATGGCAT
SRR1271664.6779123.2-	CTGCGCATCCACTCAACTGGCTGGATGCCAT
SRR1271605.4262505.1+	TGCACATCCACACAACTGGCTGGATGGCAT
SRR1271606.2596332.1-	TGCACATCCACACAACTGGCTGGATGGCAT
SRR1271631.33921289.2+	TGCACATCCACACAACTGGCTGGATGGCAT
SRR1271665.3828750.1+	TGCACATCCACACAACTGGCTGGATGGCAT
SRR1271666.15049755.2+	TGCCCATCCACACAACTGGCTGGATGGCAT
SRR1265420.8209771.2-	GCACATCCACACAACTGGCTGGATGGCAT
SRR1265420.9704864.1-	GCACATCCACACAACTGGCTGGATGGCAT
SRR1265103.985070.2-	CACATCCACACAACTGGCTGGATGGCAT
SRR1271610.12892437.1+	CACATCCACACAACTGGCTGGATGGCAT
SRR1271626.2251061.2+	CACATCCACACAACTGGCTGGATGGCAT
SRR1269690.2081589.1-	ACATCCACACAACTGGCTGGATGGCAT
SRR1265101.7450306.2-	CATCCACACAACTGGCTGGATGGCAT
SRR1265427.1292820.1+	CATCCACACAACTGGCTGGATGGCAT
SRR1265430.2865913.1+	CATCCACACAACTGGCTGGATGGCAT
SRR1266946.20535265.1+	CATCCACACAACTGGCTGGATGGCAT
SRR1271632.2107455.1+	CATCCACACAACTGGCTGGATGGCAT
SRR1265118.653206.1-	ATCCACACAACTGGCTGGATGGCAT
SRR1266957.1389486.1-	ATCCACACAACTGGCTGGATGGCAT
SRR1266958.5348149.1-	ATCCACACAACTGGCTGGATGGCAT
SRR1266964.3568258.1+	ATCCACACAACTGGCTGGATGGCAT
SRR1271626.37697061.1+	ATCCACACAACTGGCTGGATGGCAT
SRR1271631.78235890.1-	ATCCACACAACTGGCTGGATGGCAT
SRR1271631.88107554.2-	ATCCACACAACTGGCTGGATGGCAT
SRR1271667.9388496.1+	ATCCACACAACTGGCTGGATGGCAT
SRR1265962.6217934.2-	TCCACACAACTGGCTGGATGGCAT
SRR1266949.9294787.2-	TCCACACAACTGGCTGGATGGCAT
SRR1271639.15930769.1+	TCCACACAACTGGCTGGATGGCAT
SRR1271640.3344063.1+	CCACACAACTGGCTGGATGGCAT
SRR1265119.1351842.2+	CACACAACTGGCTGGATGGCAT
SRR1269694.10059460.1+	CACACAACTGGCTGGATGGCAT
SRR1271636.10973309.1-	CACACAACTGGCTGGATGGCAT
SRR1265108.11035394.2-	ACACAACTGGCTGGATGGCAT
SRR1265961.21125338.2-	ACACAACTGGCTGGATGGCAT

```
SRR1271631.31017128.2+                                          ACACAACTGGCTGGATGGCAT
SRR1265487.11821367.1-                                           CACAACTGGCTGGATGGCAT
SRR1271637.3241382.1-                                            CACAACTGGCTGGATGGCAT
SRR1271636.12517782.1-                                            ACAACTGGCTGGATGGCAT
SRR1265429.1518485.2-                                              CAACTGGCTGGATGGCAT
SRR1271619.1235877.2+                                              CAACTGGCTGGATGGCAT
SRR1265425.5275455.1+                                               AACTGGCTGGATGGCAT
SRR1266953.243746.1+                                                AACTGGCTGGATGGCAT
SRR1271624.5552917.1-                                               AACTGGCTGGATGGCAT
SRR1269721.4648312.2-                                                ACTGGCTGGATGGCAT
SRR1271642.11184403.2-                                               ACTGGCTGGATGGCAT
SRR1271643.6252990.2-                                                ACTGGCTGGATGGCAT
SRR1265094.790368.1-                                                  CTGGCTGGATGGCAT
SRR1271611.53462766.1+                                                CTGGCTGGATGGCAT
SRR1271626.23923475.1-                                                CTGGCTGGATGGCAT
SRR1271638.1270149.1+                                                 CTGGCTGGATGGCAT
SRR1271665.3197988.2-                                                 CTGGCTGGATGGCAT
SRR1265091.3132398.2+                                                  TGGCTGGATGGCAT
SRR1271626.33842030.2+                                                  GGCTGGATGGCAT
SRR1271639.3293122.2+                                                   GGCTGGATGGCAT
SRR1265959.5926745.2+                                                    GCTGGATGGCAT
SRR1269721.8669041.2-                                                    GCTGGATGGCAT
SRR1271622.14489347.1-                                                   GCTGGATGGCAT
SRR1266949.13627167.1-                                                    CTGGATGGCAT
SRR1266964.3108962.2-                                                     CTGGATGGCAT
SRR1265430.10056360.1-                                                     TGGATGGCAT
SRR1265488.8390944.1+                                                      TGGATGGCAT
SRR1271617.20893667.1+                                                     TGGATGGCAT
SRR1271631.16099619.2-                                                     TGGATGGCAT
SRR1271631.75834610.2-                                                     TGGATGGCAT
SRR1271636.7067284.2-                                                      TGGATGGCAT
SRR1271637.7917479.1-                                                      TGGATGGCAT
SRR1265118.3202418.1-                                                       GGATGGCAT
SRR1265419.9760671.1-                                                       GGATGGCAT
SRR1265959.8541929.1+                                                       GGATGGCAT
SRR1269724.3215048.1+                                                       GGATGGCAT
SRR1271660.19161575.1+                                                      GGATGGCAT
SRR1271661.7471914.2-                                                       GGATGGCAT
SRR1271631.51751938.1-                                                       GATGGCAT
SRR1265429.13415617.1-                                                        ATGGCAT
SRR1269694.7238249.2+                                                         ATGGCAT
SRR1271631.56849156.2-                                                        ATGGCAT
SRR1265959.14596287.1-                                                         TGGCAT
SRR1271626.5381257.1-                                                          TGGCAT
SRR1265960.10540450.2-                                                          GGCAT
SRR1269727.6578407.2+                                                           GGCAT
SRR1271611.50005129.1+                                                          GGCAT
SRR1265103.1752770.1+                                                            GCAT
SRR1265101.3000620.1+                                                             CAT
SRR1265420.8458569.2-                                                             CAT
SRR1265962.10464106.2-                                                            CAT
SRR1271628.74690.1-                                                               CAT
SRR1265119.9232704.2+                                                              AT
SRR1265486.8872663.2+                                                              AT
SRR1266948.8806088.1-                                                              AT
SRR1269690.4935460.1+                                                              AT
SRR1271631.92695975.2+                                                             AT
SRR1265422.16304480.1-                                                              T
SRR1271609.5981094.1+                                                               T
SRR1271661.10105368.1+                                                              T
                         ____________________________________________________________
consensus                CACGAGGAACAGGAGCTGATGAGGTTGAGCTGCACATCCACACAACTGGCTGGATGGCAT

                             .    :    .    :    .    :    .    :    .    :    .    :
SRR1265118.8469183.1+    C
SRR1265427.10426011.1+   C
SRR1265430.3999536.2-    C
SRR1271656.18693034.2-   C
SRR1266959.6559141.2+    CT
SRR1266962.3051106.2+    CT
SRR1271623.14435698.2+   CT
SRR1271625.4462362.2-    CT
SRR1265090.573951.1+     CTG
SRR1265419.5834061.2-    CTG
SRR1266962.8268942.2-    CTG
SRR1269694.6400404.1+    CTG
SRR1269727.8758172.1+    CTG
SRR1271617.87539143.2+   CTG
SRR1271631.51412202.2-   CTG
SRR1265490.7565460.2+    CTGG
SRR1271611.32939716.2+   CTGG
SRR1271663.9467739.2+    CTGG
SRR1265107.8184323.2+    CTGGA
SRR1271625.4672091.1-    CTGGA
```

SRR1271625.4974445.2-	CTGGA
SRR1265490.12123742.2-	CTGGAT
SRR1269725.9198123.1+	CTGGAT
SRR1271666.9606214.1-	CTGGAT
SRR1265108.11287257.2+	CTGGATT
SRR1269723.12879211.1+	CTGGATT
SRR1271611.35735582.1-	CTGGATT
SRR1271611.45105264.1+	CTGGATT
SRR1271637.7730229.2-	CTGGATT
SRR1271605.19945219.1+	CTGGATTC
SRR1271606.10370241.1+	CTGGATTC
SRR1271618.8223150.2+	CTGGATTC
SRR1271617.24478703.2+	CTGGATTCA
SRR1271656.16215479.1-	CTGGATTCA
SRR1271656.38998198.2-	CTGGATTCA
SRR1265427.13084996.1+	CTGGATTCAG
SRR1266946.1503677.1-	CTGGATTCAG
SRR1271656.4976182.2+	CTGGATTCAGC
SRR1265490.5635851.1+	CTGGATTCAGCC
SRR1269724.1613007.2+	CTGGATTCAGCC
SRR1271628.1578656.2-	CTGGATTCAGCC
SRR1271631.73787149.2-	CTGGATTCAGCC
SRR1271634.2225500.2-	CTGGATTCAGCC
SRR1271660.7768350.2+	CTGGATTCAGCC
SRR1265490.1647534.2+	CTGGATTCAGCCC
SRR1271617.29201909.1-	CTGGATTCAGCCC
SRR1271656.27642211.2-	CTGGATTCAGCCC
SRR1271617.18340985.1-	CTGGATTCAGCCCT
SRR1271643.6252990.1+	CTGGATTCAGCCCT
SRR1265486.4816291.2+	CTGGATTCAGCCTTC
SRR1266947.1797376.1+	CTGGATTCCGCCCTC
SRR1269693.657451.2-	CTGGATTCAGCCCTC
SRR1269724.7736550.2-	CTGGATTCAGCCCTC
SRR1269724.7878642.2-	CTGGATTCAGCCCTC
SRR1271625.4440646.1-	CTGGATTCAGCCTTC
SRR1271626.7870290.2+	CTGGATTCAGCCCTC
SRR1271663.9621731.2+	CTGGATTCAGCCTTC
SRR1265101.7863435.2+	CTGGATTCAGCCCTCA
SRR1265119.2075759.2+	CTGGATTCAGCCCTCA
SRR1271660.19489729.2-	ATGGATTCAGCCCTCA
SRR1265425.11836318.2+	CTGGATTCAGCCTTCAT
SRR1266959.12652072.2-	CTGGATTCAGCCCTCAT
SRR1271638.10108003.1-	CTGGATTCAGCCCTCAT
SRR1265107.9762562.1+	CTGGATTCAGCCCTCATG
SRR1266964.9683545.2+	CTGGATTCAGCCCTCATGG
SRR1271617.106361713.1-	CTGGATTCAGCCCTCATGG
SRR1271631.38795764.1+	CTGTATTCAGCCTTCATGG
SRR1271637.8462724.1-	CTGGATTCAGCCTTCATGG
SRR1269724.2351101.2-	CTGGATTCAGCCCTCATGGA
SRR1265424.825035.1+	CTGGATTCAGCCCTCATGGAG
SRR1266962.10797533.1+	CTGGATTCAGCCTTCATGGAG
SRR1266962.933875.2-	CTGGATTCAGCCCTCATGGAG
SRR1269695.4605288.1-	CTGGATTCAGCCCTCATGGAG
SRR1269701.962293.1+	CTGGATTCAGCCCTCATGGAG
SRR1265094.9258610.2-	CTGGATTCAGCCCTCATGGAGA
SRR1266963.14618912.2+	CTGGATTCAGCCCTCATGGAGA
SRR1271627.2433963.2+	CTGGATTCAGCCTTCATGGAGA
SRR1265960.6641003.1-	CTGGATTCAGCCTTCATGGAGAG
SRR1266959.1925156.1+	CTGGATTCAGCCCTCATGGAGAG
SRR1269702.9206585.1-	CTGGATTCAGCCCTCATGGAGAG
SRR1271636.32970902.1-	CTGGATTCAGCCCTCATGGAGAG
SRR1265108.5336322.1+	CTGGATTCAGCCCTCATGGAGAGC
SRR1265485.927570.2+	CTGGATTCAGCCTTCATGGAGAGC
SRR1266948.20521025.2-	CTGGATTCAGCCTTCATGGAGAGC
SRR1269691.4799971.1+	CTGGATTCAGCCCTCATGGAGAGC
SRR1269722.8669002.1+	CTGGATTCAGCCCTCATGGAGAGC
SRR1265487.3611936.2-	CTGGATTCAGCCTTCATGGAGAGCT
SRR1265487.5948289.1+	CTGGATTCAGCCTTCATGGAGAGCT
SRR1271656.19755138.1+	CTGGATTCAGCCCTCATGGAGAGCT
SRR1265120.10380681.2+	CTGGATTCAGCCCTCATGGAGAGCTG
SRR1265430.9435022.1-	CTGGATTCAGCCCTCATGGAGAGCTG
SRR1269700.1764719.2-	CTGGATTCAGCCCTCATGGAGAGCTG
SRR1271637.9483811.1+	CTGGATTCAGCCCTCATGGAGAGCTG
SRR1265103.11024313.1+	CTGGATTCAGCCTTCATGGAGAGCTGC
SRR1269689.11150721.2+	CTGGATTCAGCCCTCATGGAGAGCTGC
SRR1269701.8482472.1+	CTGGATTCAGCCCTCATGGAGAGCTGC
SRR1269726.8967180.1-	CTGGATTCAGCCCTCATGGAGAGCTGC
SRR1266946.22292515.1-	CTGGATTCAGCCCTCATGGAGAGCTGCC
SRR1266948.4974750.1+	CTGGATTCAGCCTTCATGGAGAGCTGCC
SRR1265100.7029791.2-	CTGGATTCAGCCTTCATGGAGAGCTGCCT
SRR1266960.6111811.2-	CTGGATTCAGCCCTCATGGAGAGCTGCCT
SRR1266952.10100495.1-	CTGGATTCAGCCCTCATGGAGAGCTGCCTG
SRR1265116.2215467.2+	CTGGATTCAGCCCTCATGGAGAGCTGCCTGG
SRR1265486.9625524.2+	CTGGATTCAGCCTTCATGGAGAGCTGCCTGG

SRR1271626.39747880.2- CTGGATTCAGCCTTCATGGAGAGCTGCCTGG
SRR1265424.14641649.2+ CTGGATTCAGCCCTCATGGAGAGCTGCCTGGA
SRR1265100.6825972.2+ CTGGATTCAGCCTTCATGGAGAGCTGCCTGGAT
SRR1265420.6477611.2+ CTGGATTCAGCCCTCATGGAGAGCTGCCTGGAT
SRR1265486.2988089.2- CTGGATTCAGCCCTCATGGAGAGCTGCCTGGAT
SRR1271641.1475015.2- CTGGATTCAGCCCTCATGGAGAGCTGCCTGGAT
SRR1271643.11732587.1- CTGGATTCAGCCCTCATGGAGAGCTGCCTGGAT
SRR1271656.19605949.2+ CTGGATTCAGCCCTCATGGAGAGCTGCCTGGAT
SRR1265091.7293804.1- CTGGATTCAGCCCTCATGGAGAGCTGCCTGGATA
SRR1265494.10552009.1+ CTGGATTCAGCCCTCATGGAGAGCTGCCTGGATA
SRR1266951.1123610.1+ CTGGATTCAGCCCTCATGGAGAGCTGCCTGGATA
SRR1269725.4823828.2+ CTGGATTCACCCCTCATGGGGAGCTGCCTGGATA
SRR1271617.7158444.2- CTGGATTCAGCCCTCATGGAGAGCTGCCTGGATA
SRR1271626.27554490.1+ CTGGATTCAGCCTTCATGGAGAGCTGCCTGGATA
SRR1271631.94542648.1+ CTGGATTCAGCCCTCATGGAGAGCTGCCTGGATA
SRR1269689.1433856.2+ CTGGATTCAGCCCTCATGGAGAGCGGCCTGGATAT
SRR1269726.4838196.2- CTGGATTCAGCCCTCATGGAGAGCTGCCTGGATATG
SRR1271605.3457147.2+ CTGGATTCAGCCCTCATGGAGAGCTGCCTGGATATGAC
SRR1271614.7695441.1+ CTGGATTCAGCCCTCATGGAGAGCTGCCTGGATATGAC
SRR1269689.9751428.2+ CTGGATTCAGCCCTCATGGAGAGCTGCCTGGATATGACA
SRR1265103.9204126.1- CTGGATTCAGCCCTCATGGAGAGCTGCCTGGATATGACAT
SRR1271626.39100757.2+ CTGGATTCAGCCTTCATGGAGAGCTGCCTGGATATGACAT
SRR1266966.10206312.2+ CTGGATTCAGCCCTCATGGAGAGCTGCCTGGATATGACATT
SRR1271617.46091166.2- CTGGATTCAGCCCTCATGGAGAGCTGCCTGGATATGACATT
SRR1271626.2595264.2+ CTGGATTCAGCCTTCATGGAGAGCTGCCTGGATATGACATT
SRR1271631.36011479.1+ CTGGATTCAGCCCTCATGGAGAGCTGCCTGGATATGACATT
SRR1271638.17228052.2+ CTGGATTCAGCCTTCATGGAGAGCTGCCTGGATATGACATT
SRR1265118.2132389.2+ CTGGATTCAGCCCTCATGGAGAGCTGCCTGGATATGACATTG
SRR1269693.1981773.1+ CTGGATTCAGCCCTCATGGAGAGCTGCCTGGATATGACATTG
SRR1271617.15492594.1- CTGGATTCAGCCCTCATGGAGAGCTGCCTGGATATGACATTG
SRR1271624.3358682.2- CTGGATTCAGCCTTCATGGAGAGCTGCCTGGATATGACATTG
SRR1265424.7293.2+ CTGGATTCAGCCTTCATGGAGAGCTGCCTGGATATGACATTGT
SRR1266948.19095128.2+ CTGGATTCAGCCTTCATGGAGAGCTGCCTGGATATGACATTGT
SRR1266957.3666759.2- CTGGATTCAGCCCTCATGGAGAGCTGCCTGGATATGACATTGT
SRR1271617.12164168.1+ CTGGATTCAGCCCTCATGGAGAGCTGCCTGGATATGACATTGT
SRR1271621.2349329.1+ CTGGATTCAGCCCTCATGGAGAGCTGCCTGGATATGACATTGT
SRR1271631.60122186.1+ CTGGATTCAGCCCTCATGGAGAGCTGCCTGGATATGACATTGT
SRR1271631.75576578.1- CTGGATTCAGCCTTCATGGAGAGCTGCCTGGATATGACATTGT
SRR1265107.9747432.1- CTGGATTCAGCCCTCATGGAGAGCTGCCTGGATATGACATTGTG
SRR1265428.3438079.1+ CTGGATTCAGCCCTCATGGAGAGCTGCCTGGATATGACATTGTG
SRR1265487.9618712.2+ CTGGATTCAGCCCTCATGGAGAGCTGCCTGGATATGACATTGTG
SRR1266960.47540.2- CTGGATTCAGCCCTCATGGAGAGCTGCCTGGATATGACATTGTG
SRR1271617.99985877.2+ CTGGATTCAGCCCTCATGGAGAGCTGCCTGGATATGACATTGTG
SRR1265418.2535443.1+ CTGGATTCAGCCCTCATGGAGAGCTGCCTGGATATGACATTGTGA
SRR1265486.11495028.2+ CTGGATTCAGCCTTCATGGAGAGCTGCCTGGATATGACATTGTGAC
SRR1269702.3793239.2- CTGGATTCAGCCCTCATGGAGAGCTGCCTGGATATGACATTGTGACA
SRR1271617.46820496.2- CTGGATTCAGCCCTCATGGAGAGCTGCCTGGATATGACATTGTGACA
SRR1271617.81204720.2+ CTGGATTCAGCCCTCATGGAGAGCTGCCTGGATATGACATTGTGACA
SRR1271640.16324249.2+ CTGGATTCAGCCCTCATGGAGAGCTGCCTGGATATGACATTGTGACA
SRR1271656.37410994.2- CTGGATTCAGCCCTCATGGAGAGCTGCCTGGATATGACATTGTGACA
SRR1265106.3654183.1+ CTGGATTCAGCCCTCATGGAGAGCTGCCTGGATATGACATTGTGACAGG
SRR1265428.3817667.1+ CTGGATTCAGCCTTCATGGAGAGCTGCCTGGATATGACATTGTGACAGG
SRR1265429.2745098.2+ CTGGCTTCAGCCTTCATGGAGAGCTGCCTGGATATGACATTGTGACAGGA
SRR1271639.6412786.2+ CTGGATTCAGCCCTCATGGAGAGCTGCCTGGATATGACATTGTGACAGGA
SRR1271656.50764191.2+ CTGGATTCAGCCCTCATGGAGAGCTGCCTGGATATGACATTGTGACAGGA
SRR1265421.1577740.1+ CTGGATTCAGCCCTCATGGAGAGCTGCCTGGATATGACATTGTGACAGGAG
SRR1265959.4685497.1- CTGGATTCAGCCTTCATGGAGAGCTGCCTGGATATGACATTGTGACAGGAG
SRR1266946.20413741.1+ CTGGATTCAGCCTTCATGGAGAGCTGCCTGGATATGACATTGTGACAGGAG
SRR1266948.20676083.1- CTGGATTCAGCCTTCATGGAGAGCTGCCTGGATATGACATTGTGACAGGAG
SRR1271611.58180054.2- CTGGATTCAGCCCTCATGGAGAGCTGCCTGGATATGACATTGTGACAGGAG
SRR1269721.4085403.1- CTGGATTCAGCCCTCATGGAGAGCTGCCTGGATATGACATTGTGACAGGAGG
SRR1271616.7303942.1- CTGGATTCAGCCCTCATGGAGAGCTGCCTGGATATGACATTGTGACAGGAGGT
SRR1271617.43257682.2- CTGGATTCAGCCCTCATGGAGAGCTGCCTGGATATGACATTGTGACAGGAGGT
SRR1266952.5487495.1+ CTGGATTCAGCCCTCATGGAGAGCTGCCTGGATATGACATTGTGACAGGAGGTG
SRR1266964.9267740.1- CTGGATTCAGCCTTCCTGGAGAGATGCCTGGATATGAAATTGTGACAGGAGGTG
SRR1265962.10995514.2+ CTGGATTCAGCCTTCATGGAGAGCTGCCTGGATATGACATTGTGACAGGAGGTGC
SRR1266948.1989161.1- CTGGATTCAGCCTTCATGGAGAGCTGCCTGGATATGACATTGTGACAGGAGGTGC
SRR1269720.2054067.1+ CTGGATTCAGCCCTCATGGAGCGCTGCCTGGATATGACATTGTGACAGGAGGGGC
SRR1271617.70351078.2- CTGGATTCAGCCCTCATGGAGAGCTGCCTGGATATGACATTGTGACAGGAGGTGC
SRR1271637.17744220.1+ CTGGATTCAGCCCTCATGGAGAGCTGCCTGGATATGACATTGTGACAGGTGGTGC
SRR1271637.17744220.2- CTGGATTCAGCCCTCATGGAGAGCTGCCTGGATATGACATTGTGACAGGAGGTGC
SRR1265493.861628.2- CTGGATTCAGCCCTCATGGAGAGCTGCCTGGATATGACATTGTGACAGGAGGTGCC
SRR1266956.10452827.2+ CTGGATTCAGCCCTCATGGAGAGCTGCCTGGATATGACATTGTGACAGGAGGGGCC
SRR1269692.5660364.2+ CTGGATTCAGCCCTCATGGAGAGCTGCCTGGATATGACATTGTGACAGGAGGTGCC
SRR1271606.11910612.2+ CTGGATTCAGCCCTCATGGAGAGCTGCCTGGATATGACATTGTGACAGGAGGTGCC
SRR1271618.3593897.1+ CTGGATTCAGCCCTCATGGAGAGCTGCCTGGATATGACATTGTGACAGGAGGTGCC
SRR1271631.70543548.2+ CTGGATTCAGCCTTCATGGAGAGCTGCCTGGATATGACATTGTGACAGGAGGTGCC
SRR1271632.1398829.2+ CTGGATTCAGCCTTCATGGAGAGCTGCCTGGATATGACATTGTGACAGGAGGTGCC
SRR1271640.643478.1+ CTGGATTCAGCCTTCATGGAGAGCTGCCTGGATATGACATTGTGACAGGAGGTGCC
SRR1271644.8110496.1+ CTGGATTCAGCCCTCATGGAGAGCTGCCTGGATATGACATTGTGACAGGAGGTGCCT
SRR1271617.89166641.1+ CTGGATTCAGCCCTCATGGAGAGCTGCCTGGATATGACATTGTGACAGGAGGTGCCTC
SRR1266956.8706797.2+ CTGGATTCAGCCCTCATGGAGAGCTGCCTGGATATGACATTGTGACAGGAGGTGCCTCC
SRR1265091.3132398.2+ CTGGATTCAGCCTTCATGGAGAGCTGCCTGGATATGACATTGTGACAGGAGGTGCCTCCC
SRR1265094.790368.1- CTGGATTCAGCCTTCATGGAGAGCTGCCTGGATATGACATTGTGACAGGAGGTGCCTCCC

SRR1265101.3000620.1+   CTGGATTCAGCCTTCATGGAGAGCTGCCTGGATATGACATTGTGACAGGAGGTGCCTCCC
SRR1265101.7450306.2-   CTGGATTCAGCCTTCATGGAGAGCTGCCTGGATATGACATTGTGACAGGAGGTGCCTCCC
SRR1265103.1752770.1+   CTGGATTCAGCCTTCATGGAGAGCTGCCTGGATATGACATTGTGACAGGAGGTGCCTCCC
SRR1265103.985070.2-   CTGGATTCAGCCCTCATGGAGAGCTGCCTGGATATGACATTGTGACAGGAGGTGCCTCCC
SRR1265106.5466905.1+   CTGGATTCAGCCCTCATGGAGAGCTGCCTGGATATGACATTGTGACAGGAGGTGCCTCCC
SRR1265107.10559184.2+   CTGGATTCAGCCCTCATGGAGAGCTGCCTGGATATGACATTGTGACAGGAGGTGCCTCCC
SRR1265107.5946926.2-   CTGGATTCAGCCCTCATGGAGAGCTGCCTGGATATGACATTGTGACAGGAGGTGCCTCCC
SRR1265108.11035394.2-   CTGGATTCAGCCCTCATGGAGAGCTGCCTGGATATGACATTGTGACAGGAGGTGCCTCCC
SRR1265118.3202418.1-   CTGGATTCAGCCCTCATGGAGAGCTGCCTGGATATGACATTGTGACAGGAGGTGCCTCCC
SRR1265118.653206.1-   CTGGATTCAGCCCTCATGGAGAGCTGCCTGGATATGACATTGTGACAGGAGGTGCCTCCC
SRR1265119.1351842.2+   CTGGATTCAGCCCTCATGGAGAGCTGCCTGGATATGACATTGTGACAGGAGGTGCCTCCC
SRR1265119.9232704.2+   CTGGATTCAGCCCTCATGGAGAGCTGCCTGGATATGACATTGTGACAGGAGGTGCCTCCC
SRR1265419.9630529.1-   CTGGATTCAGCCCTCATGGAGAGCTGCCTGGATATGACATTGTGACAGGAGGTGCCTCCC
SRR1265419.9760671.1-   CTGGATTCAGCCCTCATGGAGAGCTGCCTGGATATGACATTGTGACAGGAGGTGCCTCCC
SRR1265420.8209771.2-   CTGGATTCAGCCCTCATGGAGAGCTGCCTGGATATGACATTGTGACAGGAGGTGCCTCCC
SRR1265420.8458569.2-   CTGGATTCAGCCCACATGGAGAGCTGCCTGGATATGACATTGTGACATGAGGTGCCTCCC
SRR1265420.9704864.1-   CTGGATTCAGCCCTCATGGAGAGCTGCCTGGATATGACATTGTGACAGGAGGTGCCTCCC
SRR1265422.16304480.1-   CTGGATTCAGCCCTCATGGAGAGCTGCCTGGATATTACATTGTGACAGGAGGTGCCTCCC
SRR1265425.5275455.1+   CTGGATTCAGCCCTCATGGAGAGCTGCCTGGATATGACATTGTGACAGGAGGTGCCTCCC
SRR1265427.1292820.1+   CTGGATTCAGCCTTCATGGAGAGCTGCCTGGATATGACATTGTGACAGGAGGTGCCTCCC
SRR1265429.12762650.1+   CTGGATTCAGCCCTCATGGAGAGCTGCCTGGATATGACATTGTGACAGGAGGGGCCTCCC
SRR1265429.13415617.1-   CTGGATTCAGCCCTCATGGAGAGCTGCCTGGATATGACATTGTGACAGGAGGTGCCTCCC
SRR1265429.1518485.2-   CTGGATTCAGCCTTCATGGAGAGCTGCCTGGATATGACATTGTGACAGGAGGTGCCTCCC
SRR1265429.617391.2+   CTGGATTCAGCCCTCATGGAGAGCTGCCTGGATATGACATTGTGACAGGAGGTGCCTCCC
SRR1265430.10056360.1-   CTGGATTCAGCCTTCATGGAGAGCTGCCTGGATATGACATTGTGACAGGAGGTGCCTCCC
SRR1265430.11367129.2-   CTGGATTCAGCCTTCATGGAGAGCTGCCTGGATATGACATTGTGACAGGAGGTGCCTCCC
SRR1265430.2865913.1+   CTGGATTCAGCCTTCATGGAGAGCTGCCTGGATATGACATTGTGACAGGAGGTGCCTCCC
SRR1265486.8872663.2+   CTGGATTCAGCCCTCATGGAGAGCTGCCTGGATATGACATTGTGACAGGAGGTGCCTCCC
SRR1265487.11821367.1-   CTGGATTCAGCCCTCATGGAGAGCTGCCTGGATATGACATTGTGACAGGAGGTGCCTCCC
SRR1265488.2054623.2+   CTGGATTCAGCCCTCATGGAGAGCTGCCTGGATATGACATTGTGACAGGGGGGGCCTCCC
SRR1265488.8390944.1+   CTGGATTCAGCCTTCATGGAGAGCTGCCTGGATATGACATTGTGACAGGAGGTGCCTCCC
SRR1265494.8124625.1-   CTGGATTCAGCCCTCATGGAGAGCTGCCTGGATATGACATTGTGACAGGAGGTGCCTCCC
SRR1265959.14596287.1-   CTGGATTCAGCCTTCATGGAGAGCTGCCTGGATATGACATTGTGACAGGAGGTGCCTCCC
SRR1265959.5926745.2+   CTGGATTCAGCCTTCATGGAGAGCTGCCTGGATATGACATTGTGACAGGAGGTGCCTCCC
SRR1265959.8541929.1+   CTGGATTCAGCCTTCATGGAGAGCTGCCTGGATATGACATTGTGACAGGAGGTGCCTCCC
SRR1265960.10540450.2-   CTGGATTCAGCCTTCATGGAGAGCTGCCTGGATATGACATTGTGACAGGAGGTCCCTCCC
SRR1265961.17164072.1-   CTGGATTCAGCCTTCATGGAGAGCTGCCTGGATATGACATTGTGACAGGAGGTGCCTCCC
SRR1265961.21125338.2-   CTGGATTCAGCCTTCATGGAGAGCTGCCTGGATATGACATTGTGACAGGAGGTGCCTCCC
SRR1265962.10464106.2-   CTGGATTCAGCCTTCATTGAGAGCTGCCTGGATATGACATTGTGACAGGAGGTGCCTCCC
SRR1265962.6107415.1+   CTGGATTCAGCCTTCATGGAGAGCTGCCTGGATATGACATTGTGACAGGGGGTGCCTCCC
SRR1265962.6217934.2-   CTGGATTCAGCCTTCATGGAGAGCTGCCTGGATATGACATTGTGACAGGAGGTGCCTCCC
SRR1266946.20535265.1+   CTGGATTTAGCCTTCATGGAAAGCTGCCTGGATATGACATTGTGACAGGAGGTGCCTCCC
SRR1266948.8806088.1-   CTGGATTCAGCCTTCATGGAGAGCTGCCTGGATATGACATTGTGACAGGAGGTGCCTCCC
SRR1266949.13627167.1-   CTGGATTCAGCCCTCATGGAGAGCTGCCTGGATATGACATTGTGACAGGAGGTGCCTCCC
SRR1266949.6573322.2+   CTGGATTCAGCCTTCATGGAGAGCTGCCTGGATATGACATTGTGACAGGAGGTGCCTCCC
SRR1266949.9294787.2-   CTGGATTCAGCCCTCATGGAGAGCTGCCTGGATATGACATTGTGACAGGAGGTGCCTCCC
SRR1266951.538073.1-   CTGGATTCAGCCCTCATGGAGAGCTGCCTGGATATGACATTGTGACAGGAGGTGCCTCCC
SRR1266953.243746.1+   CTGGATTCAGCCCTCATGGAGAGCTGCCTGGATATGACATTGTGACAGGAGGTGCCTCCC
SRR1266956.11010848.2+   CTGGATTCAGCCCTCATGGAGAGCTGCCTGGATATGACATTGTGACAGGAGGTGCCTCCC
SRR1266957.1389486.1-   CTGGATTCAGCCCTCATGGAGAGCTGCCTGGATATGACATTGTGACAGGAGGTGCCTCCC
SRR1266957.2266659.2+   CTGGATTCAGCCCTCATGGAGAGCTGCCTGGATATGACATTGTGACAGGAGGTGCCTCCC
SRR1266958.5348149.1-   CTGGATTCAGCCCTCATGGAGAGCTGCCTGGATATGACATTGTGACAGGAGGTGCCTCCC
SRR1266964.3108962.2-   CTGGATTCAGCCTTCATGGAGAGCTGCCTGGATATGACATTGTGACAGGAGGTGCCTCCC
SRR1266964.3568258.1+   CTGGATTCAGCCTTCATGGAGAGCTGCCTGGATATGACATTGTGACAGGAGGTGCCTCCC
SRR1266966.3487586.2-   CTGAATTCAGCCCTCATGGAGAGCTGCCTGGATATGACATTGTGACAGGAGGTGCCTCCC
SRR1269690.2081589.1-   CTGGATTCAGCCCTCATGGAGAGCTGCCTGGATATGACATTGTGACAGGAGGTGCCTCCC
SRR1269690.4935460.1+   CTGGATTCAGCCCTCATGGAGAGCTGCCTGGATATGACATTGTGACAGGAGGTGCCTCCC
SRR1269691.6146778.1+   CTGGATTCAGCCCTCATGGAGAGCTGCCTGGATATGACATTGTGACAGGAGGTGCCTCCC
SRR1269694.10059460.1+   CTGGATTCAGCCTTCATGGAGAGCTGCCTGGATATGACATTGTGACAGGAGGGGCCTCCC
SRR1269694.7238249.2+   CTGGATTCAGCCTTCATGGAGAGCTGCCTGGATATGACATTGTGACAGGAGGTGCCTCCC
SRR1269721.4648312.2-   CTGGATTCAGCCCTCATGGAGAGCTGCCTGGATATGACATTGTGACAGGAGGTGCCTCCC
SRR1269721.8669041.2-   CTGGATTCAGCCCTCATGGAGAGCTGCCTGGATATGACATTGTGACAGGAGGTGCCTCCC
SRR1269723.7352283.2+   CTGGATTCAGCCCTCATGGAGAGCTGCCTGGATATGACATTGTGACAGGAGGTGCCTCCC
SRR1269723.7787279.1-   CTGGATTCAGCCCTCATGGAGAGCTGCCTGGATATGACATTGTGACAGGAGGTGCCTCCC
SRR1269724.3215048.1+   CTGGATTCAGCCCTCATGGAGAGCTGCCTGGATATGACATTGTGACAGGAGGTGCCTCCC
SRR1269727.6578407.2+   CTGGATTCAGCCCTCATGGAGAGCTGCCTGGATATGACATTGTGACAGGAGGTGCCTCCC
SRR1269727.8680671.1+   CTGGATTCAGCCCTCATGGAGAGCTGCCTGGATATGACATTGTGACAGGAGGTGCCTCCC
SRR1271605.1607310.2+   CTGGATTCAGCCCTCATGGAGAGCTGCCTGGATATGACATTGTGACAGGAGGTGCCTCCC
SRR1271605.4262505.1+   CTGGATTCAGCCCTCATGGAGAGCTGCCTGGATATGACATTGTGACAGGAGGTGCCTCCC
SRR1271606.2596332.1-   CTGGATTCAGCCCTCATGGAGAGCTGCCTGGATATGACATTGTGACAGGAGGTGCCTCCC
SRR1271606.4403075.1+   CTGGATTCAGCCCTCATGGAGAGCTGCCTGGATATGACATTGTGACAGGAGGTGCCTCCC
SRR1271609.5981094.1+   CTGGATTCAGCCCTCATGGAGAGCTGCCTGGATATGACATTGTGACAGGAGGTGCCTCCC
SRR1271610.12892437.1+   CTGGATTCAGCCCTCATGGAGAGCTGCCTGGATATGACATTGTGACAGGAGGTGCCTCCC
SRR1271611.50005129.1+   CTGGATTCAGCCCTCATGGAGAGCTGCCTGGATATGACATTGTGACAGGAGGTGCCTCCC
SRR1271611.53462766.1+   CTGGATTCAGCCCTCATGGAGAGCTGCCTGGATATGACATTGTGACAGGAGGTGCCTCCC
SRR1271611.8284106.2-   CTGGATTCAGCCCTCATGGAGAGCTGCCTGGATATGACATTGTGACAGGAGGTGCCTCCC
SRR1271616.2239278.1-   CTGGATTCAGCCCTCATGGAGAGCTGCCTGGATATGACATTGTGACAGGAGGTGCCTCCC
SRR1271617.20893667.1+   CTGGATTCAGCCCTCATGGAGAGCTGCCTGGATATGACATTGTGACAGGAGGTGCCTCCC
SRR1271617.2641915.2-   CTGGATTCAGCCCTCATGGAGAGCTGCCTGGATATGACATTGTGACAGGAGGTGCCTCCC
SRR1271617.76678834.2-   CTGGATTCAGCCCTCATGGAGAGCTGCCTGGATATGACATTGTGACAGGAGGTGCCTCCC
SRR1271617.9057728.2-   CTGGATTCAGCCCTCATGGAGAGCTGCCTGGATATGACATTGTGACAGGAGGTGCCTCCC
SRR1271619.1235877.2+   CTGGATTCAGCCCTCATGGAGAGCTGCCTGGATATGACATTGTGACAGGAGGCGCCTCCC
SRR1271622.14489347.1-   CTGGATTCAGCCTTCATGGAGAGCTGCCTGGATATGACATTGTGACAGGAGGTGCCTCCC
SRR1271624.5552917.1-   CTGGATTCAGCCTTCATGGAGAGCTGCCTGGATATGACATTGTGACAGGAGGTGCCTCCC

```
SRR1271626.10299387.2-   CTGGATTCAGCCCTCATGGAGAGCTGCCTGGATATGACATTGTGACAGGAGGTGCCTCCC
SRR1271626.15519384.1-   CTGGATTCAGCCCTCATGGAGAGCTGCCTGGATATGACATTGTGACAGGAGGTGCCTCCC
SRR1271626.2251061.2+    CTGGATTCAGCCCTCATGGAGAGCTGCCTGGATATGACATTGTGACAGGAGGTGCCTCCC
SRR1271626.23923475.1-   CTGGATTCAGCCCTCATGGAGAGCTGCCTGGATATGACATTGTGACAGGAGGTGCCTCCC
SRR1271626.33842030.2+   CTGGATTCAGCCCTCATGGAGAGCTGCCTGGATATGACATTGTGACAGGAGGTGCCTCCC
SRR1271626.37697061.1+   CTGGATTCAGCCCTCATGGAGAGCTGCCTGGATATGACATTGTGACAGGAGGTGCCTCCC
SRR1271626.37703222.1+   CTGGATTCAGCCTTCATGGAGAGCTGCCTGGATATGACATTGTGACAGGAGGTGCCTCCC
SRR1271626.5381257.1-    CTGGATTCAGCCTTCATGGAGAGCTGCCTGGATATGACATTGTGACAGGAGGTGCCTCCC
SRR1271626.7548380.2+    CTGGATTCAGCCCTCATGGAGAGCTGCCTGGATATGACATTGTGACAGGAGGTGCCTCCC
SRR1271628.74690.1-      CTGGATTCAGCCTTCATGGAGAGCTGCCTGGATATGACATTGTGACAGGAGGTGCCTCCC
SRR1271631.104145897.2-  CTGGATTCAGCCCTCATGGAGAGCTGCCTGGATATGACATTGTGACAGGAGGTGCCTCCC
SRR1271631.16099619.2-   CTGGATTCAGCCCTCATGGAGAGCTGCCTGGATATGACATTGTGACAGGAGGTGCCTCCC
SRR1271631.30275332.1+   CTGGATTCAGCCCTCATGGAGAGCTGCCTGGATATGACATTGTGACAGGAGGTGCCTCCC
SRR1271631.31017128.2+   CTGGATTCAGCCTTCATGGAGAGCTGCCTGGATATGACATTGTGACAGGAGGTGCCTCCC
SRR1271631.33921289.2+   CTGGATTCAGCCCTCATGGAGAGCTGCCTGGATATGACATTGTGACAGGAGGTGCCTCCC
SRR1271631.37762412.1+   CTGGATTCAGCCTTCATGGAGAGCTGCCTGGATATGACATTGTGACAGGAGGTGCCTCCC
SRR1271631.42268717.2+   CTGGATTCAGCCCTCATGGAGAGCTGCCTGGATATGACATTGTGACAGGAGGTGCCTCCC
SRR1271631.51751938.1-   CTGGATTCAGCCTTCATGGAGAGCTGCCTGGATATGACATTGTGACAGGAGGTGCCTCCC
SRR1271631.56849156.2-   CTGGATTCAGCCTTCATGGAGAGCTGCCTGGATATGACATTGTGACAGGAGGTGCCTCCC
SRR1271631.75834610.2-   CTGGATTCAGCCGTCATGGAGAGCTGCCTGGATATGACATTGTGACAGGAGGTGCCTCCC
SRR1271631.78235890.1-   CTGGATTCAGCCCTCATGGAGAGCTGCCTGGATATGACATTGTGACAGGAGGTGCCTCCC
SRR1271631.88107554.2-   CTGGATTCAGCCTTCATGGAGAGCTGCCTGGATATGACATTGTGACAGGAGGTGCCTCCC
SRR1271631.92695975.2+   CTGGATTCAGCCTTCATGGAGAGCTGCCTGGATATGACATTGTGACAGGAGGAGCCTCCC
SRR1271632.2107455.1+    CTGGATTCAGCCCTCATGGAGAGCTGCCTGGATATGACATTGTGACAGGAGGTGCCTCCC
SRR1271636.10089274.2+   CTGGATTCAGCCTTCATGGAGAGCTGCCTGGATATGACATTGTGACAGGAGGTGCCTCCC
SRR1271636.10973309.1-   CTGGATTCAGCCCTCATGGAGAGCTGCCTGGATATGACATTGTGACAGGAGGTGCCTCCC
SRR1271636.12517782.1-   CTGGATTCAGCCTTCATGGAGAGCTGCCTGGATATGACATTGTGACAGGAGGTGCCTCCC
SRR1271636.7067284.2-    CTGGATTCAGCCCTCATGGAGAGCTGCCTGGATATGACATTGTGACAGGAGGTGCCTCCC
SRR1271637.3241382.1-    CTGGATTCAGCCTTCATGGAGAGCTGCCTGGATATGACATTGTGACAGGAGGTGCCTCCC
SRR1271637.7917479.1-    CTGGATTCAGCCTTCATGGAGAGCTGCCTGGATATGACATTGTGACAGGAGGTGCCTCCC
SRR1271638.1270149.1+    CTGGATTCAGCCCTCATGGAGAGCTGCCTGGATATGACATTGTGACAGGAGGTGCCTCCC
SRR1271639.15930769.1+   CTGGATTCAGCCTTCATGGAGAGCTGCCTGGATATGACATTGTGACAGGAGGTGCCTCCC
SRR1271639.3293122.2+    CTGGATTCAGCCTTCATGGAGAGCTGCCTGGATATGACATTGTGACAGGAGGGGCCTCCC
SRR1271640.16967275.2+   CTGGATTCAGCCCTCATGGAGAGCTGCCTGGATATGACATTGTGACAGGAGGTGCCTCCC
SRR1271640.3344063.1+    CTGGATTCAGCCTTCATGGAGAGCTGCCTGGATATGACATTGTGACAGGAGGTGCCTCCC
SRR1271642.11184403.2-   CTGGATTCAGCCCTCATGGAGAGCTGCCTGGATATGACATTGTGACAGGAGGTGCCTCCC
SRR1271643.6252990.2-    CTGGATTCAGCCCTCATGGAGAGCTGCCTGGATATGACATTGTGACAGGAGGTGCCTCCC
SRR1271643.6397.1+       CTGGATTCAGCCCTCATGGAGAGCTGCCTGGATATGACATTGTGACAGGAGGTGCCTCCC
SRR1271644.5773655.2+    CTGGATTCAGCCCTCATGGAGAGCTGCCTGGATATGACATTGTGACAGGAGGTGCCTCCC
SRR1271656.13812009.2-   CTGGATTCAGCCCTCATGGAGAGCTGCCTGGATATGACATTGTGACAGGAGGTGCCTCCC
SRR1271656.23576076.2+   CTGGATTCAGCCCTCATGGAGAGCTGCCTGGATATGACATTGTGACAGGAGGTGCCTCCC
SRR1271660.19161575.1+   CTGGATTCAGCCTTCATGGAGAGCTGCCTGGATATGACATTGTGACAGGAGGTGCCTCCC
SRR1271660.204492.2-     CTGGATTCAGCCTTCATGGAGAGCTGCCTGGATATGACATTGTGACAGGAGGTGCCTCCC
SRR1271661.10105368.1+   CTGGATTCAGCCCTCATGGAGAGCTGCCTGGATATGACATTGTGACAGGAGGTGCCTCCC
SRR1271661.7471914.2-    CTGGATTCAGCCCTCATGGAGAGCTGCCTGGATATGACATTGTGACAGGAGGTGCCTCCC
SRR1271661.7818147.2-    CTGGATTCAGCCCTCATGGAGAGCTGCCTGGATATGACATTGTGACAGGAGGTGCCTCCC
SRR1271664.6779123.2-    CTGGATTCAGCCCTCATGGAGAGCTGCCTGTATATGACATTGTGACAGGAGGTGCCTCCC
SRR1271665.3197988.2-    CTGGATTCAGCCCTCATGGAGAGCTGCCTGGATATGACATTGTGACAGGAGGTGCCTCCC
SRR1271665.3828750.1+    CTGGATTCAGCCCTCATGGAGAGCTGCCTGGATATGACCTTGTGACAGGAGGTGCCTCCC
SRR1271666.15049755.2+   CTGGATTCAGCCTTCATGGAGAGCTGCCTGGATATGACATTGTGACAGGAGGCGCCTCCC
SRR1271667.9388496.1+    CTGGATTCAGCCCTCATGGAGAGCTGCCTGGATATGACATTGTGACAGGAGGTGCCTCCC
SRR1265100.3569680.1+     TGGATTCAGCCTTCATGGAGAGGTGCCTGGATATGACATTGTGACAGGAGGTGCCTCCC
SRR1271620.12742265.2+    TGGATTCAGCCCTCATGGAGAGCTGCCTGGATATGACATTGTGACAGGAGGTGCCTCCC
SRR1271642.9849776.1+     TGGATTCAGCCCTCATGGAGAGCTGCCTGGATATGACATTGTGACAGGAGGGGCCTCCC
SRR1271665.16214124.2-    TGGATTCAGCCTTCATGGAGAGCTGCCTGGATATGACATTGTGACAGGAGGTGCCTCCC
SRR1269724.4223359.2+      GGATTCAGCCCTCATGGAGAGCTGCCTGGATATGACATTGTGACAGGAGGTGCCTCCC
SRR1271617.37162146.1+     GGATTCAGCCCTCATGGAGAGCTGCCTGGATATGACATTGTGACAGGAGGTGCCTCCC
SRR1271639.4756923.2+      GGATTCAGCCTTCATGGAGAGCTGCCTGGATATGACATTGTGACAGGAGGTGCCTCCC
SRR1271643.10483940.1+     GGATTCAGCCCTCATGGAGAGCTGCCTGGATATGACATTGTGACAGGAGGTGCCTCCC
SRR1271631.7995179.1-       GATTCAGCCTTCATGGAGAGCTGCCTGGATATGACATTGTGACAGGAGGTGCCTCCC
SRR1265423.4002997.2+        ATTCAGCCCTCATGGAGAGCTGCCTGGATATGACATTGTGACAGGAGGTGCCTCCC
SRR1265427.10252303.1-       ATTCAGCCCTCATGGAGAGCTGCCTGGATATGACATTGTGACAGGAGGTGCCTCCC
SRR1266964.13711499.2+       ATTCAGCCTTCATGGAGAGCTGCCTGGATATGACATTGTGACAGGAGGTGCCTCCC
SRR1269723.820781.1-         ATTCAGCCCTCATGGAGAGCTGCCTGGATATGACATTGTGACAGGAGGTGCCTCCC
SRR1269724.764340.1-         ATTCAGCCCTCATGGAGAGCTGCCTGGATATGACATTGTGACAGGAGGTGCCTCCC
SRR1271631.115122576.1+      ATTCAGCCTTCATGGAGAGCTGCCTGGATATGACATTGTGACAGGAGGTGCCTCCC
SRR1271639.6929131.1+        ATTCAGCCCTCATGGAGAGCTGCCTGGATATGACATTGTGACAGGAGGTGCCTCCC
SRR1271656.4106422.1+         TTCAGCCCTCATGGAGAGCTGCCTGGATATGACATTGTGACAGGAGGTGCCTCCC
SRR1265962.9313085.1+          TCAGCCTTCATGGAGAGCTGCCTGGATATGACATTGTGACAGGAGGTGCCTCCC
SRR1265419.10177100.1+          CAGCCCTCATGGAGAGCTGCCTGGATATGACATTGTGACAGGAGGTGCCTCCC
SRR1265487.6650089.1+           CAGCCTTCATGGAGAGCTGCCTGGATATGACATTGTGACAGGAGGGGCCTCAC
SRR1265492.6404320.1-           CAGCCCTCATGGAGAGCTGCCTGGATATGACATTGTGACAGGAGGTGCCTCCC
SRR1271614.9511385.2+           CAGCCCTCATGGAGAGCTGCCTGGATATGACATTGTGACAGGAGGGGCCTCCC
SRR1271661.3541586.1-           CAGCCCTCATGGAGAGCTGCCTGGATATGACATTGTGACAGGAGGTGCCTCCC
SRR1265422.11220844.2+           AGCCTTCATGGAGAGCTGCCTGGATATGACATTGTGACAGGAGGTGCCTCCC
SRR1269694.9908338.1-             GCCTTCATGGAGAGCTGCCTGGATATGACATTGTGACAGGAGGTGCCTCCC
SRR1271618.3593897.2-             GCCCTCATGGAGAGCTGCCTGGATATGACATTGTGACAGGAGGTGCCTCCC
SRR1271624.3306484.2-             GCCCTCATTGAGAGCTGCCTGGATATGACATTGTGACAGGAGGTGCCTCCC
SRR1265117.8111828.2-              CCCTCATGGAGAGCTGCCTGGATATGACATTGTGACAGGAGGTGCCTCCC
SRR1269689.1433856.1-              CCCTCATGGAGAGCTGCCTGGATATGACATTGTGACAGGAGGTGCCTCCC
SRR1269690.8024392.1-              CCCTCATGGAGAGCTGCCTGGATATGACATTGTGACAGGAGGTGCCTCCC
SRR1269695.4463182.2+              CCCTCATGGAGAGCTGCCTGGATATGACATTGTGACAGGAGGTGCCTCCC
SRR1269700.383429.1-               CCCTCATGGAGAGCTGCCTGGATATGACATTGTGACAGGAGGTGCCTCCC
SRR1271611.16853764.1-             CCCTCATGGAGAGCTGCCTGGATATGACATTGTGACAGGAGGTGCCTCCC
```

SRR1271617.50700751.1+	CCCTCATGGAGAGCTGCCTGGATATGACATTGTGACAGGAGGTGCCTCCC
SRR1271631.43237953.1+	CCCTCATGGAGAGCTGCCTGGATATGACATTGTGACAGGAGGTGCCTCCC
SRR1271660.15638503.2-	CCCTCATGGAGAGCTGCCTGGATATGACATTGTGACAGGAGGTGCCTCCC
SRR1265486.13491181.2+	CCTCATGGAGAGCTGCCTGGATATGACATTGTGACAGGAGGTGCCTCCC
SRR1269691.1086423.2-	CCTCATGGAGAGCTGCCTGGATATGACATTGTGACAGGAGGTGCCTCCC
SRR1269696.5605733.2-	CTTCATGGAGAGCTGCCTGGATATGACATTGTGACAGGAGGTGCCTCCC
SRR1271606.5702810.2+	CCTCATGGAGAGCTGCCTGGATATGACATTGTGACAGGAGGTGCCTCCC
SRR1271617.65162719.2-	CCTCATGGAGAGCTGCCTGGATATGACATTGTGACAGGAGGTGCATCCC
SRR1271640.12546842.1+	CTTCATGGAGAGCTGCCTGGATATGACATTGTGACAGGAGGTGCCTCCC
SRR1266947.12390140.1+	TTCATGGAGAGCTGCCTGGATATGACATTGTGACAGGAGGTGCCTCCC
SRR1271611.139187.1+	CTCATGGAGAGCTGCCTGGATATGACATTGTGACAGGAGGTGCCTCCC
SRR1271627.8664492.2+	TCATGGAGAGCTGCCTGGATATGACATTGTGACAGGAGGTGCCTCCC
SRR1265119.703716.2+	CATGGAGAGCTGCCTGGATATGACATTGTGACAGGAGGTGCCTCCC
SRR1269689.3378410.1+	CATGGAGAGCTGCCTGGATATGACATTGTGACAGGAGGTGCCTCCC
SRR1269724.4049028.1+	CATGGAGAGCTGCCTGGATATGACATTGTGACAGGAGGTGCCTCCC
SRR1271664.2243521.1-	CATGGAGAGCTGCCTGGATATGACATTGTGACAGGAGGTGCCTCCC
SRR1265092.2522035.2+	ATGGAGAGCTGCCTGGATATGACATTGTGACAGGAGGTGCCTCCC
SRR1265427.6605459.2-	ATGGAGAGCTGCCTGGATATGACATTGTGACAGGAGGTGCCTCCC
SRR1265488.15177506.2+	ATGGAGAGCTGCCTGGATATGACATTGTGACAGGAGGTGCCTCCC
SRR1271609.12516496.1+	ATGGAGAGCTGCCTGGATATGACATTGTGACAGGAGGTGCCTCCC
SRR1271638.17228052.1-	ATGGAGAGCTGCCTGGATATGACATTGTGACAGGAGGTGCCTCCC
SRR1265102.10463059.1+	TGGAGAGCTGCCTGGATATGACATTGTGACAGGAGGTGCCTCCC
SRR1265961.17342409.1-	GGAGGGCTGCCTGGATATGACATTGTGACAGGAGGTGCCTCCC
SRR1271605.649185.2-	GGAGAGCTGCCTGGATATGACATTGTGACAGGAGGTGCCTCCC
SRR1271610.20241530.2+	GGAGAGCTGCCTGGATATGACATTGTGACAGGAGGTGCCTCCC
SRR1271626.19553110.1+	GGAGAGCTGCCTGGATATGACATTGTGACAGGAGGTGCCTCCC
SRR1271660.7892962.2+	GGAGAGCTGCCTGGATATGACATTGTGACAGGAGGTGCCTCCC
SRR1265961.2477855.2-	GAGAGCTGCCTGGATATGCCAATGTGACAGGAGGTGCCTCCC
SRR1266955.131900.1-	GAGAGCTGCCTGGATATGACATTGTGACAGGAGGTGCCTCCC
SRR1271610.8517146.1+	GAGAGCTGCCTGGATATGACATTGTGACAGGAGGTGCCTCCC
SRR1271611.48572042.1+	GAGAGCTGCCTGGATATGACATTGTGACAGGAGGTGCCTCCC
SRR1271622.14382387.2-	GAGAGCTGCCTTGATATGACATTGTGACAGGAGGTGCCTCCC
SRR1271631.58668872.1+	GAGAGCTGCCTGGATATGACATTGTGACAGGAGGTGCCTCCC
SRR1271619.5245116.2-	AGAGCTGCCTGGATATGACATTGTGACAGGAGGTGCCTCCC
SRR1271622.11118149.2+	AGAGCTGCCTGGATATGACATTGTGACAGGAGGTGCCTCCC
SRR1271637.2965041.1+	AGAGCTGCCTGGATATGACATTGTGACAGGAGGTGCCTCCC
SRR1271638.5730963.1+	AGAGCTGCCTGGATATGACATTGTGACAGGAGGTGCCTCCC
SRR1265106.6015462.1-	GAGCTGCCTGGATATGACATTGTGACAGGAGGTGCCTCCC
SRR1265494.7123982.2+	GAGCTGCCTGGATATGACATTGTGACAGGAGGTGCCTCCC
SRR1271615.5772241.2+	GAGCTGCCTGGATATGACATTGTGACAGGAGGTGCCTCCC
SRR1266966.10206312.1-	AGCTGCCTGGATATGACATTGTGACAGGAGGTGCCTCCC
SRR1271631.8158897.1+	AGCTGCCTGGATATGACATTGTGACAGGAGGTGCCTCCC
SRR1271664.1051398.2+	AGCTGCCTGGATATGACATTGTGACAGGAGGTGCCTCCC
SRR1269727.2683943.1-	GCTGCCTGTATATGACATTGTGACAGGAGGTGCCTCCC
SRR1271619.11721387.1-	GCTGCCTGGATATGACATTGTGACAGGAGGTGCCTCCC
SRR1271660.28173172.1+	GCTGCCTGGATATGACATTGTGACAGGAGGTGCCTCCC
SRR1271661.66238.1+	GCTGCCTGGATATGACATTGTGACAGGAGGTGCCTCCC
SRR1265120.12719577.2-	CTGCCTGGATATGACATTGTGACAGGAGGTGCCTCCC
SRR1265423.1464723.2-	CTGCCTGGATATGACATTGTGACAGGAGGTGCCTCCC
SRR1265487.5948289.2-	CTGCCTGGATATGACATTGTGACAGGAGGTGCCTCCC
SRR1271620.4399325.2+	CTGCCTGGATATGACATTGTGACAGGAGGTGCCTCCC
SRR1266956.2653211.2-	TGCCTGGATATGACATTGTGACAGGAGGTGCCTCCC
SRR1271624.5962316.1-	TGCCTGGATATGACATTGTGACAGGAGGTGCCTCCC
SRR1271638.7137901.2-	TGCCTGGATATGACATTGTGACAGGAGGTGCCTCCC
SRR1269722.6911698.1+	GCCTGGATATGACATTGTGACAGGAGGTGCCTCCC
SRR1265094.4259059.1-	CTGGTTATGACATTGTGACAGGAGGTGCCTCCC
SRR1266963.7668173.2-	CTGGATATGACATTGTGACAGGAGGTGCCTCCC
SRR1266964.11823893.2+	CTGGATATGACATTGTGACAGGAGGTGCCTCCC
SRR1271631.60482848.1-	CTGGATATGACATTGTGACAGGAGGTGCCTCCC
SRR1271631.37714877.2+	TGGATATGACATTGTGACAGGAGGTGCCTCCC
SRR1271641.2977584.2+	TGGATATGACATTGTGACAGGAGGTGCCTCCC
SRR1271660.18416623.1+	TGGATATGACATTGTGACAGGAGGTGCCTCCC
SRR1265492.5585323.2-	GGATATGACATTGTGACAGGAGGTGCCTCCC
SRR1269690.5971251.1+	GGATATGACATTGTGACAGGAGGTGCCTCCC
SRR1269703.1669213.1-	GGATATGACATTGTGACAGGAGGTGCCTCCC
SRR1271631.65104355.1+	GGATATGACATTGTGACAGGAGGTGCCTCCC
SRR1269700.9312299.2+	GATATGACATTGTGANAGGAGGTGCCTCCC
SRR1269723.1055715.2+	GATATGACATTGTGACAGGAGGTGCCTCCC
SRR1271610.8517146.2-	GATATGACATTGTGACAGGAGGTGCCTCCC
SRR1271612.6153622.2-	GATATGACATTGTGACAGGAGGTGCCTCCC
SRR1271616.6540135.1+	GATATGACATTGTGACAGGAGGTGCCTCCC
SRR1271624.1867358.1-	GATATGACATTGTGACAGGAGGTGCCTCCC
SRR1271609.12903351.2+	ATATGACATTGTGACAGGAGGTGCCTCCC
SRR1265424.7293.1-	TATGACATTGTGACAGGAGGTGCCTCCC
SRR1265485.15551348.1+	TATGACATTGTGACAGGAGGTGCCTCCC
SRR1265103.225186.1+	ATGACATTGTGACAGGAGGTGCCTCCC
SRR1266962.7560910.1+	ATGACATTGTGACAGGAGGTGCCTCCC
SRR1271636.20236431.1-	ATGACATTGTGACAGGAGGTGCCTCCC
SRR1271665.17092941.1+	ATGACATTGTGACAGGAGGTGCCTCCC
SRR1271667.7988823.1+	ATGACATTGTGACAGGAGGTGCCTCCC
SRR1265090.6109847.1+	TGACATTGTGACAGGAGGTGCCTCCC
SRR1265100.3569680.2-	TGACATTGTGACAGGAGGTGCCTCCC
SRR1266947.19802521.1-	TGACATTGTGACAGGAGGTGCCTCCC
SRR1266952.1314922.2+	TGACATTGTGACAGGAGGTGCCTCCC

SRR1266957.11077584.1- TGACATTGCGACAGGAGGTGCCTCCC
SRR1265116.4878633.2- GACATTGTGACAGGAGGTGCCTCCC
SRR1269725.4246389.1+ GACATTGTGACAGGAGGTGCCTCCC
SRR1271610.19119218.1+ GACATTGTGACAGGAGGTGCCTCCC
SRR1271660.25394364.1+ GACATTGTGACAGGAGGTGCCTCCC
SRR1265423.1668798.1+ ACATTGTGACAGGAGGTGCCTCCC
SRR1265429.2745098.1- ACATTGTGACAGGAGGTGCCTCCC
SRR1265485.10372300.2- ACATTGTGACAGGAGGTGCCTCCC
SRR1266946.10714685.1+ ACATTGTGACAGGAGGTGCCTCCC
SRR1271656.47447557.1+ ACATTGTGACAGGAGGTGCCTCCC
SRR1265120.5268240.1- CATTGTGACAGGAGGTGCCTCCC
SRR1265485.16445595.1+ CATTGTGACAGGAGGTGCCTCCC
SRR1269689.6495585.2+ CATTGTGACAGGAGGTGCCTCCC
SRR1269700.8196703.2+ CATTGTGACAGGAGGTGCCTCCC
SRR1269721.10925497.1+ CATTGTGACAGGAGGTGCCTCCC
SRR1269722.612784.2- CATTGTGACAGGAGGTGCCTCCC
SRR1269725.3442595.2+ CATTGTGACAGGAGGTGCCTCCC
SRR1271626.19282964.1- CATTGTGACAGGAGGTGCCTCCC
SRR1271665.16602572.2- CATTGTGACAGGAGGTGCCTCCC
SRR1266960.4486561.1+ ATTGTGACAGGAGGTGCCTCCC
SRR1269727.8907245.1- ATTGTGACAGGAGGTGCCTCCC
SRR1271610.5829611.2- ATTGTGACAGGAGGTGCCTCCC
SRR1271611.51705152.1- ATTGTGACAGGAGGTGCCTCCC
SRR1271617.108645868.2+ ATTGTGACAGGAGGTGCCTCCC
SRR1271626.27417879.1- ATTGTGACAGGAGGTGCCTCCC
SRR1271637.9483811.2- ATTGTGACAGGAGGTGCATCCC
SRR1265960.14555341.1- TTGTGACAGGAGGTGCCTCCC
SRR1271626.8851170.2- TTGTGACAGGAGGTGCCTCCC
SRR1271642.3018005.2- TTGTGACAGGAGGTGCGTCCC
SRR1265421.22002067.2- TGTGACAGGAGGTGCCTCCC
SRR1271606.11910612.1- TGTGACAGGAGGTGCCTCCC
SRR1271611.45475124.1+ TGTGACAGGAGGTGCCTCCC
SRR1271631.53104753.2- TGTGACAGGAGGTGCCTCCC
SRR1271656.31699611.2- TGTGACCGGAGGTGCCTCCC
SRR1265103.11096904.2- GTGACAGGAGGTGCCTCCC
SRR1265421.25226457.1- GTGACAGGAGGTGCCTCCC
SRR1269692.7437558.2+ GTGACAGGAGGTGCCTCCC
SRR1271631.13703327.1- GTGACAGGAGGTGCCTCCC
SRR1271631.17796850.2- GTGACAGGAGGTGCCTCCC
SRR1271644.6211925.1- GTGACAGGAGGTGCCTCCC
SRR1271656.37726636.1- GTGACAGGAGGTGCCTCCC
SRR1265421.10386528.1- TGACAGGAGGTGCCTCCC
SRR1266949.6049032.2+ TGACAGGAGGTGCCTCCC
SRR1266952.14069486.1- TGACAGGAGGTGCCTCCC
SRR1266959.5837465.1- TGACAGGAGGTGCCTCCC
SRR1269689.257048.1+ TGACAGGAGGTGCCTCCC
SRR1271617.21164005.2- TGACAGGAGGTGCCTCCC
SRR1271630.6944205.1- TGACAGGAGGTGCCTCCC
SRR1271641.1828285.1- TGACAGGAGGTGCCTCCC
SRR1266947.868250.1- GACAGGAGGTGCCTCCC
SRR1266957.2266659.1- GACAGGAGGTGCCTCCC
SRR1266964.10482596.2+ GACAGGAGGTGCCTCCC
SRR1269690.6866648.1- GACAGGAGGTGCCTCCC
SRR1269693.3627362.1+ GACAGGAGGTGCCTCCC
SRR1271611.30562393.1- GACAGGAGGTGCCTCCC
SRR1271631.47115470.1+ GACAGGAGGTGCCTCCC
SRR1265090.6845871.2- ACAGGAGGTGCCTCCC
SRR1265962.8694253.2+ ACAGGAGGTGCCTCCC
SRR1271606.5849136.1- ACAGTAGGTGCCTCCC
SRR1271609.1031300.2- ACAGGAGGTGCCTCCC
SRR1271666.2776816.1- ACAGGAGGTGCCTCCC
SRR1265090.4559545.1+ CAGGAGGTGCCTCCC
SRR1271616.2011111.1- CAGGAGGTGCCTCCC
SRR1265959.13979314.2+ AGGAGGTNCCTCCN
SRR1271611.5176815.2- AGGAGGTGCCTCCC
SRR1271636.11917632.2+ AGGAGGTGCCTCCC
SRR1266948.14467911.2- GGAGGTGCCTCCC
SRR1269699.8216561.2+ GGAGGTGCCTCCC
SRR1269700.4999243.2+ GAGGTGCCTCCC
SRR1269703.1602551.2+ GAGGTGCCTCCC
SRR1269720.6697396.1- GAGGTGCCTCCC
SRR1271631.39606297.2+ GAGGTGCCTCCC
SRR1271642.249531.2- GAGGTGCCTCCC
SRR1265488.8390944.2- AGGTGCCTCCC
SRR1271617.72923099.1+ AGGTGCCTCCC
SRR1265959.5289237.1- GGTGCCTCCC
SRR1269722.11583054.1+ GGTGCCTCCC
SRR1269722.7488875.1+ GGTGCCTCCC
SRR1265102.11716845.2- GTGCCTCCC
SRR1266966.2152787.1+ GTGCCTCCC
SRR1269699.6395347.1+ GTGCCTCCC
SRR1269703.7122945.1+ GTGCCTCCC
SRR1271631.38215481.1+ GTGCCTCCC
SRR1266962.4122587.2- TGCCTCCC

```
SRR1271617.6275825.1+                                                        TGCCTCCC
SRR1271639.12989136.1-                                                       TGCCTCCC
SRR1265116.2981073.2+                                                         GCCTCCC
SRR1265423.10735190.2+                                                        GCCTCCC
SRR1265425.6471841.1+                                                         GCCTCCC
SRR1265960.2099829.1+                                                         GCCTCCC
SRR1269723.11892874.2-                                                        GCCTCCC
SRR1265107.6639546.1+                                                          CCTCCC
SRR1269723.5270004.2+                                                          CCTCCC
SRR1271617.35095789.1+                                                         CCTCCC
SRR1271626.29048032.2-                                                         CCTCCC
SRR1271611.49933549.1+                                                          CTCCC
SRR1271617.72688277.1+                                                           TCCC
SRR1271620.10933616.2-                                                           TCCC
SRR1271630.6773779.2+                                                            TCCC
SRR1269694.995920.2+                                                              CCC
SRR1269723.12292891.2-                                                            CCC
SRR1266961.10701038.1+                                                             CC
SRR1269691.6982732.1-                                                              CC
SRR1271617.49734526.2-                                                             CC
SRR1271617.65566596.2+                                                             CC
SRR1271631.53790917.1-                                                             CC
SRR1271656.42884483.1-                                                             CC
SRR1271664.9315059.1-                                                              CC
SRR1266960.7618362.1-                                                               C

consensus                CTGGATTCAGCCCTCATGGAGAGCTGCCTGGATATGACATTGTGACAGGAGGTGCCTCCC

                             .    :    .    :    .    :    .    :    .    :    .    :
SRR1265494.8124625.1-    C
SRR1271636.10089274.2+   C
SRR1271643.6397.1+       C
SRR1265429.12762650.1+   CA
SRR1265488.2054623.2+    CA
SRR1265962.6107415.1+    CA
SRR1266949.6573322.2+    CA
SRR1266966.3487586.2-    CA
SRR1265106.5466905.1+    CAAA
SRR1265962.6217934.2-    CAAA
SRR1271656.23576076.2+   CAAA
SRR1265961.17164072.1-   CAAAT
SRR1271626.7548380.2+    CAAAT
SRR1271631.42268717.2+   CAAAT
SRR1271644.5773655.2+    CAAAT
SRR1266957.2266659.2+    CAAATG
SRR1271606.2596332.1-    CAAATG
SRR1271616.2239278.1-    CAAATG
SRR1265429.617391.2+     CAAATGG
SRR1271617.2641915.2-    CAAATGG
SRR1271626.10299387.2-   CAAATGG
SRR1265430.11367129.2-   CAAATGGC
SRR1269691.6146778.1+    CAAATGGC
SRR1269723.7787279.1-    CAAATGGC
SRR1271631.37762412.1+   CAAATGGC
SRR1271656.13812009.2-   CAAATGGC
SRR1271660.204492.2-     CAAATGGC
SRR1271661.7818147.2-    CAAATGGC
SRR1266956.11010848.2+   CAAATGGCA
SRR1269723.7352283.2+    CAAATGGCA
SRR1271606.4403075.1+    CAAATGGCA
SRR1271611.8284106.2-    CAAATGGCA
SRR1271626.15519384.1-   CAAATGGCA
SRR1271631.30275332.1+   CAAATGGCA
SRR1271664.6779123.2-    CAAATGGCA
SRR1266946.20535265.1+   CAAATGGCAG
SRR1271605.4262505.1+    CAAATGGCAG
SRR1271631.33921289.2+   CAAATGGCAG
SRR1271665.3828750.1+    CAAATGGCAG
SRR1271666.15049755.2+   CAAATGGCAG
SRR1265420.8209771.2-    CAAATGGCAGC
SRR1265420.9704864.1-    CAAATGGCAGC
SRR1265430.2865913.1+    CAAATGGCAGC
SRR1265103.985070.2-     CAAATGGCAGCA
SRR1271610.12892437.1+   CAAATGGCAGCA
SRR1271626.2251061.2+    CAAATGGCAGCA
SRR1269690.2081589.1-    CAAATGGCAGCAT
SRR1265101.7450306.2-    CAAATGGCAGCATC
SRR1265427.1292820.1+    CAAATGGCAGCATC
SRR1271632.2107455.1+    CAAATGGCAGCATC
SRR1265118.653206.1-     CAAATGGCAGCATCT
SRR1266957.1389486.1-    CAAATGGCAGCATCT
SRR1266958.5348149.1-    CAAATGGCAGCATCT
SRR1266964.3568258.1+    CAAATGGCAGCATCT
SRR1271626.37697061.1+   CAAATGGCAGCATCT
```

SRR1271631.78235890.1-   CAAATGGCAGCATCT
SRR1271631.88107554.2-   CAAATGGCAGCATCT
SRR1271667.9388496.1+   CAAATGGCAGCATCT
SRR1266949.9294787.2-   CAAATGGCAGCATCTG
SRR1271639.15930769.1+   CAAATGGCAGCATCTG
SRR1271640.3344063.1+   CAAATGGCAGCATCTGA
SRR1265119.1351842.2+   CAAATGGCAGCACCTGAT
SRR1269694.10059460.1+   CAAATGGCAGCATCTGAT
SRR1271636.10973309.1-   CAAATGGCAGCATCTGAT
SRR1265108.11035394.2-   CAAATGGCAGCATCTGATT
SRR1265961.21125338.2-   CAAATGGCAGCATCTGATT
SRR1271619.1235877.2+   CAAATGGCAGCATCTGATT
SRR1271631.31017128.2+   CAAATGGCAGCATCTGATT
SRR1265420.8458569.2-   CAAATGGCAGCATTTGATTC
SRR1265429.1518485.2-   CAAATGGCAGCATCTGATTC
SRR1265487.11821367.1-   CAAATGGCAGCATCTGATTC
SRR1271637.3241382.1-   CAAATGGCAGCATCTGATTC
SRR1271636.12517782.1-   CAAATGGCAGCATCTGATTCT
SRR1265425.5275455.1+   CAAATGGCAGCATCTGATTCTCT
SRR1266953.243746.1+   CAAATGGCAGCATCTGATTCTCT
SRR1271624.5552917.1-   CAAATGGCAGCATCTGATTCTCT
SRR1269721.4648312.2-   CAAATGGCAGCATCTGATTCTCTg
SRR1271642.11184403.2-   CAAATGGCAGCATCTGATTCTCTg
SRR1271643.6252990.2-   CAAATGGCAGCATCTGATTCTCTg
SRR1265094.790368.1-   CAAATGGCAGCATCTGATTCTCTgt
SRR1271611.53462766.1+   CAAATGGCAGCATCTGATTCTCTgt
SRR1271626.23923475.1-   CAAATGGCAGCATCTGATTCTCTgt
SRR1271638.1270149.1+   CAAATGGCAGCATCTGATTCTCTgt
SRR1271665.3197988.2-   CAAATGGCAGCATCTGATTCTCTgt
SRR1265091.3132398.2+   CAAATGGCAGCATCTGATTCTCTgta
SRR1271626.33842030.2+   CAAATGGCAGCATCTGATTCTCTgtaa
SRR1271639.3293122.2+   CAAATGGCAGCATCTGATTCTCTgtaa
SRR1271661.7471914.2-   CAAATGGCAGCATCTGATTCTCTgtaa
SRR1265959.5926745.2+   CAAATGGCAGCATCTGATTCTCTgtaag
SRR1265960.10540450.2-   CAAATGGCAGCATCTGATTCTCTgtaag
SRR1269721.8669041.2-   CAAATGGCAGCATCTGATTCTCTgtaag
SRR1271622.14489347.1-   CAAATGGCAGCATCTGATTCTCTgtaag
SRR1266949.13627167.1-   CAAATGGCAGCATCTGATTCTCTgtaagt
SRR1266964.3108962.2-   CAAATGGCAGCATCTGATTCTCTgtaagt
SRR1265430.10056360.1-   CAAATGGCAGCATCTGATTCTCTgtaagtt
SRR1265488.8390944.1+   CAAATGGCAGCATCTGATTCTCTgtaagtt
SRR1269724.4223359.2+   CAAATGGCAGCATCTGATTATCTgtaagtt
SRR1271617.20893667.1+   CAAATGGCAGCATCTGATTCTCTgtaagtt
SRR1271631.16099619.2-   CAAATGGCAGCATCTGATTCTCTgtaagtt
SRR1271631.75834610.2-   CAAATGGCAGCATCTGATTCTCTgtaagtt
SRR1271636.7067284.2-   CAAATGGCAGCATCTGATTCTCTgtaagtt
SRR1271637.7917479.1-   CAAATGGCAGCATCTGATTCTCTgtaagtt
SRR1265118.3202418.1-   CAAATGGCAGCATCTGATTCTCTgtaagttg
SRR1265419.9760671.1-   CAAATGGCAGCATCTGATTCTCTgtaagttg
SRR1265959.8541929.1+   CAAATGGCAGCATCTGATTCTCTgtaagttg
SRR1269724.3215048.1+   CAAATGGCAGCATCTGATTCTCTgtaagttg
SRR1271660.19161575.1+   CAAATGGCAGCATCTGATTCTCTgtaagttg
SRR1269727.6578407.2+   CAAATGGCAGCATCTGATTCTCTgtaagttga
SRR1271631.51751938.1-   CAAATGGCAGCATCTGATTCTCTgtaagttga
SRR1265429.13415617.1-   CAAATGGCAGCATCTGATTCTCTgtaagttgaa
SRR1269694.7238249.2+   CAAATGGCAGCATCTGATTCTCTgtaagttgaa
SRR1271631.56849156.2-   CAAATGGCAGCATCTGATTCTCTgtaagttgaa
SRR1265959.14596287.1-   CAAATGGCAGCATCTGATTCTCTgtaagttgaac
SRR1271626.5381257.1-   CAAATGGCAGCATCTGATTCTCTgtaagttgaac
SRR1271611.50005129.1+   CAAATGGCAGCATCTGATTCTCTgtaagttgaaca
SRR1265103.1752770.1+   CAAATGGCAGCATCTGATTCTCTgtaagttgaacag
SRR1265962.10464106.2-   CAAATGGCAGCATCTGATTCTCTgtaagttgaacag
SRR1265101.3000620.1+   CAAATGGCAGCATCTGATTCTCTgtaagttgaacaga
SRR1271628.74690.1-   CAAATGGCAGCATCTGATTCTCTgtaagttgaacaga
SRR1265119.9232704.2+   CAAATGGCAGCATCTGATTCTCTgtaagttgaacagat
SRR1265486.8872663.2+   CAAATGGCAGCATCTGATTCTCTgtaagttgaacagat
SRR1266948.8806088.1-   CAAATGGCAGCATCTGATTCTCTgtaagttgaacagat
SRR1269690.4935460.1+   CAAATGGCAGCATCTGATTCTCTgtaagttgaacagat
SRR1271631.92695975.2+   CAAATGGCAGCATCTGATTCTCTgtaagttgaacagat
SRR1265422.16304480.1-   CTAATGGCAGCATCTGATTCTCTgtaagttgaacagatt
SRR1271609.5981094.1+   CAAATGGCAGCATCTGATTCTCTgtaagttgaacagatt
SRR1271661.10105368.1+   CAAATGGCAGCATCTGATTCTCTgtaagttgaacagatt
SRR1265107.5946926.2-   CAAATGGCAGCATCTGATTCTCTgtaagttgaacagattt
SRR1265419.9630529.1-   CAAATGGCAGCATCTGATTCTCTgtaagttgaacagattt
SRR1266951.538073.1-   CAAATGGCAGCATCTGATTCTCTgtaagttgaacagattt
SRR1271605.1607310.2+   CAAATGGCAGCATCTGATTCTCTgtaagttgaacagattt
SRR1271617.9057728.2-   CAAATGGCAGCATCTGATTCTCTgtaagttgaacagattt
SRR1271626.37703222.1+   CAAATGGCAGCATCTGATTCTCTgtaagttgaacagattt
SRR1271631.104145897.2- CAAATGGCAGCATCTGATTCTCTgtaagttgaacagattt
SRR1271639.4756923.2+   CAAATGGCAGAATCTGATTCTCTgtaagttgaacagattt
SRR1271640.16967275.2+   CAAATGGCAGCATCTGATTCTCTgtaagttgaacagattt
SRR1265100.3569680.1+   CAAATGGCAGCATCTGATTCTCTgtaagttgaacagatttc
SRR1271620.12742265.2+   CAAATGGCAGCATCTGATTCTCTgtaagttgaacagatttc
SRR1271642.9849776.1+   CAAATGGCAGCATCTGATTCTCTgtaagttgaacagatttc

```
SRR1271665.16214124.2-   CAAATGGCAGCATCTGATTCTCTgtaagttgaacagatttc
SRR1271617.37162146.1+   CAAATGGCAGCATCTGATTCTCTgtaagttgaacagatttcc
SRR1271643.10483940.1+   CAAATGGCAGCATCTGATTCTCTgtaagttgaacagatttcc
SRR1271631.7995179.1-    CAAATGGCAGCATCTGATTCTCTgtaagttgaacagatttcct
SRR1265423.4002997.2+    CAAATGGCAGCATCTGATTCTCTgtaagttgaacagatttcctc
SRR1265427.10252303.1-   CAAATGGCAGCATCTGATTCTCTgtaagttgaacagatttcctc
SRR1266964.13711499.2+   CAAATGGCAGCATCTGATTCTCTgtaagttgaacagatttcctc
SRR1269723.820781.1-     CAAATGGCAGCATCTGATTCTCTgtaagttgaacagatttcctc
SRR1269724.764340.1-     CAAATGGCAGCATCTGATTCTCTgtaagttgaacagatttcctc
SRR1271631.115122576.1+  CAAATGGCAGCATCTGATTCTCTgtaagttgaacagatttcctc
SRR1271639.6929131.1+    CAAATGGCAGCATCTGATTCTCTgtaagttgaacagatttcctc
SRR1271656.4106422.1+    CAAATGGCAGCATCTGATTCTCTgtaagttgaacagatttcctct
SRR1265962.9313085.1+    CAAATGGCAGCATCTGATTCTCTgtaagttgaacagatttcctctt
SRR1265419.10177100.1+   CAAATGGCAGCATCTGATTCTCTgtaagttgaacagatttcctcttc
SRR1265487.6650089.1+    CAAATGGCAGCATCTGATTCTCTgtaagttgaacagatttactcttc
SRR1265492.6404320.1-    CAAATGGCAGCATCTGATTCTCTgtaagttgaacagatttcctcttc
SRR1271614.9511385.2+    CAAATGGCAGCATCTGATTCTCTgtaagttgaacagatttcctcttc
SRR1271661.3541586.1-    CAAATGGCAGCATCTGATTCTCTgtaagttgaacagatttcctcttc
SRR1265422.11220844.2+   CAAATGGCAGCATCTGATTCTCTgtaagttgaacagacttcctcttca
SRR1269694.9908338.1-    CAAATGGCAGCATCTGATTCTCTgtaagttgaacagatttcctcttcat
SRR1271618.3593897.2-    CAAATGGCAGCATCTGATTCTCTgtaagttgaacagatttcctcttcat
SRR1271624.3306484.2-    CAAATGGCAGCATCTGATTCTCTgtaagttgaacagatttcctcttcat
SRR1271627.8664492.2+    CAAATGGCAGCATCTGATTCTCTgtaagttgaacagatttcctcttcat
SRR1265117.8111828.2-    CAAATGGCAGCATCTGATTCTCTgtaagttgaacagatttcctcttcatc
SRR1269689.1433856.1-    CAAATGGCAGCATCTGATTCTCTgtaagttgaacagatttcctcttcatc
SRR1269690.8024392.1-    CAAATGGCAGCATCTGATTCTCTgtaagttgaacagatttcctcttcatc
SRR1269695.4463182.2+    CAAATGGCAGCATCTGATTCTCTgtaagttgaacagatttcctcttcatc
SRR1269700.383429.1-     CAAATGGCAGCATCTGATTCTCTgtaagttgaacagatttcctcttcatc
SRR1271611.16853764.1-   CAAATGGCAGCATCTGATTCTCTgtaagttgaacagatttcctcttcatc
SRR1271617.50700751.1+   CAAATGGCAGCATCTGATTCTCTgtaagttgaacagatttcctcttcatc
SRR1271631.43237953.1+   CAAATGGCAGCATCTGATTCTCTgtaagttgaacagatttcctcttcatc
SRR1271660.15638503.2-   CAAATGGCAGCATCTGATTCTCTgtaagttgaacagatttcctcttcatc
SRR1265486.13491181.2+   CAAATGGCAGCATCTGATTCTCTgtaagttgaacagatttcctcttcatcc
SRR1269691.1086423.2-    CAAATGGCAGCATCTGATTCTCTgtaagttgaacagatttcctcttcatcc
SRR1269696.5605733.2-    CAAATGGCAGCATCTGATTCTCTgtaagttgaacagatttcctcttcatcc
SRR1271606.5702810.2+    CAAATGGCAGCATCTGATTCTCTgtaagttgaacagatttcctcttcatcc
SRR1271617.65162719.2-   CAAATGGCAGCATCTGATTCTCTgtaagttgaacagatttcctcttcatcc
SRR1271640.12546842.1+   CAAATGGCAGCATCTGATTCTCTgtaagttgaacagatttcctcttcatcc
SRR1265961.2477855.2-    AAAATGGCAGCATCTGCTTCTCTgtaagttgaaccgatttcctcttcatccc
SRR1266947.12390140.1+   CAAATGGCAGCATCTGATTCTCTgtaagttgaacagatttcctcttcatccc
SRR1271611.139187.1+     CAAATGGCAGCATCTGATTCTCTgtaagttgaacagatttcctcttcatccc
SRR1265119.703716.2+     CAAATGGCAGCATCTGATTCTCTgtaagttgaacagatttcctcttcatccccc
SRR1269689.3378410.1+    CAAATGGCAGCATCTGATTCTCTgtaagttgaacagatttcctcttcatccccc
SRR1269724.4049028.1+    CAAATGGCAGCATCTGATTCTCTgtaagttgaacagatttcctcttcatccccc
SRR1271664.2243521.1-    CAAATGGCAGCATCTGATTCTCTgtaagttgaacagatttcctcttcatccccc
SRR1265092.2522035.2+    CAAATGGCAGCATCTGATTCTCTgtaagttgaacagatttcctcttcatccccct
SRR1265427.6605459.2-    CAAATGGCAGCATCTGATTCTCTgtaagttgaacagatttcctcttcatccccct
SRR1265488.15177506.2+   CAAATGGCAGCATCTGATTCTCTgtaagttgaacagatttccccttcatccccct
SRR1271609.12516496.1+   CAAATGGCAGCATCTGATTCTCTgtaagttgaacagatttcctcttcatccccct
SRR1271622.11118149.2+   CAAATGGCAGCATCTGATTCTCTttaagttgaacagatttcctcttcataccccct
SRR1271638.17228052.1-   CAAATGGCAGCATCTGATTCTCTgtaagttgaacagatttcctcttcatccccct
SRR1265102.10463059.1+   CAAATGGCAGCATCTGATTCTCTgtaagttgaacagatttcctcttcatcccccctt
SRR1265961.17342409.1-   CAAATGGCAGCATCTGATTCTCTgtaagttgaacagatttcctcttcatcccccttt
SRR1271605.649185.2-     CAAATGGCAGCATCTGATTCTCTgtaagttgaacagatttcctcttcatcccccttt
SRR1271610.20241530.2+   CAAATGGCAGCATCTGATTCTCTgtaagttgaacagatttcctcttcatcccccttt
SRR1271626.19553110.1+   CAAATGGCAGCATCTGATTCTCTgtaagttgaacagatttcctcttcatcccccttt
SRR1271660.7892962.2+    CAAATGGCAGCATCTGATTCTCTgtaagttgaacagatttcctcttcatcccccttt
SRR1266955.131900.1-     CAAATGGCAGCATCTGATTCTCTgtaagttgaacagatttcctcttcatcccccctttg
SRR1271610.8517146.1+    CAAATGGCAGCATCTGATTCTCTgtaagttgaacagatttcctcttcatcccccctttg
SRR1271611.48572042.1+   CAAATGGCAGCATCTGATTCTCTgtaagttgaacagatttcctcttcatcccccctttg
SRR1271622.14382387.2-   CAAATGGCAGCATCTGATTCTCTgtaagttgaacagatttccacttcatcccccctttg
SRR1271631.58668872.1+   CAAATGGCAGCATCTGATTCTCTgtaagttgaacagatttcctcttcatcccccctttg
SRR1271619.5245116.2-    CAAATGGCAGCATCTGATTCTCTgtaagttgaacagatttcctcttcatcccccctttgc
SRR1271637.2965041.1+    CAAATGGCAGCATCTGATTCTCTgtaagttgaacagatttcctcttcatcccccctttgc
SRR1271638.5730963.1+    CAAATGGCAGCATCTGATTCTCTgtaagttgaacagatttcctcttcatcccccctttgc
SRR1265090.4559545.1+    CAAATGGCAGCATCTGATTCTCTgtaagttgaacagatttcctcttcatcccccctttgcc
SRR1265090.6109847.1+    CAAATGGCAGCATCTGATTCTCTgtaagttgaacagatttcctcttcatcccccctttgcc
SRR1265090.6845871.2-    CAAATGGCAGCATCTGATTCTCTgtaagttgaacagatttcctcttcatcccccctttgcc
SRR1265094.4259059.1-    CAAATGGCAGCATCTGATTCTCTgtaagttgaacagatttcctcttcatcccccctttgcc
SRR1265100.3569680.2-    CAAATGGCAGCATCTGATTCTCTgtaagttgaacagatttcctcttcatcccccctttgcc
SRR1265102.11716845.2-   CAAATGGCAGCATCTGATTCTCTgtaagttgaacagatttcctcttcatcccccctttgcc
SRR1265103.11096904.2-   CAAATGGCAGCATCTGATTCTCTgtaagttgaacagatttcctcttcatcccccctttgcc
SRR1265103.225186.1+     CAAATGGCAGCATCTGATTCTCTgtaagttgaacagatttcctcttcatcccccctttgcc
SRR1265106.6015462.1-    CAAATGGCAGCATCTGATTCTCTgtaagttgaacagatttcctcttcatcccccctttgcc
SRR1265107.6639546.1+    CAAATGGCAGCATCTGATTCTCTgtaagttgaacagatttcctcttcatcccccctttgcc
SRR1265116.2981073.2+    CAAATGGCAGCATCTGATTCTCTgtaagttgaacagatttcctcttcatcccccctttgcc
SRR1265116.4878633.2-    CAAATGGCAGCATCTGATTCTCTgtaagttgaacagatttcctcttcatcccccctttgcc
SRR1265120.12719577.2-   CAAATGGCAGCATCTGATTCTCTgtaagttgaacagatttcctcttcatcccccctttgcc
SRR1265120.5268240.1-    CAAATGGCAGCATCTGATTCTCTgtaagttgaacagatttcctcttcatcccccctttgcc
SRR1265421.10386528.1-   CAAATGGCAGCATCTGATTCTCTgtaagttgaacagatttcctcttcatcccccctttgcc
SRR1265421.22002067.2-   CAAATGGCAGCATCTGATTCTCTgtaagttgaacagatttcctcttcatcccccctttgcc
SRR1265421.25226457.1-   CAAATGGCAGCATCTGATTCTCTgtaagttgaacagatttcctcttcatcccccctttgcc
SRR1265423.10735190.2+   CAAATGGCAGCATCTGATTCTCTgtaagttgaacagatttcctcttcatcccccctttgcc
SRR1265423.1464723.2-    CAAATGGCAGCATCTGATTCTCTgtaagttgaacagatttcctcttcatcccccctttgcc
```

```
SRR1265423.1668798.1+    CAAATGGCAGCATCTGATTCTCTgtaagttgaacagatttcctcttcatccccctttgcc
SRR1265424.7293.1-       CAAATGGCAGCATCTGATTCTCTgtaagttgaacagatttcctcttcatccccctttgcc
SRR1265425.6471841.1+    CAAATGGCAGCATCTGATTCTCTgtaagttgaacagatttcctcttcatccccctttgcc
SRR1265429.2745098.1-    CAAATGGCAGCATCTGATTCTCTgtaagttgaacagatttcctcttcaccccccctttgcc
SRR1265485.10372300.2-   CAAATGGCAGCATCTGATTCTCTgtaagttgaacagatttcctcttcatccccctttgcc
SRR1265485.15551348.1+   CAAATGGCAGCATCTGATTCTCTgtaagttgaacagatttcctcttcatccccctttgcc
SRR1265485.16445595.1+   CAAATGGCAGCATCTGATTCTCTgtaagttgaacagatttcctcttcatccccctttgcc
SRR1265487.5948289.2-    CAAATGGCAGCATCTGATTCTCTgtaagttgaacagatttcctcttcatccccctttgcc
SRR1265488.8390944.2-    CAAATGGCAGCATCTGATTCTCTgtaagttgaacagatttcctcttcatccccctttgcc
SRR1265492.5585323.2-    CAAATGGCAGCATCTGATTCTCTgtaagttgaacagatttcctcttcatccccctttgcc
SRR1265492.5869691.1+    CAAATGGCAGCATCTGATTCTCTgtaagttgaacagatttcctcttcatccccctttgcc
SRR1265494.7123982.2+    CAAATGGCAGCATCTGATTCTCTgtaagttgaacagatttcctcttcatccccctttgcc
SRR1265959.13979314.2+   CAAATGGCAGCATCTGATTCTCTgtaagttgaacagatttcctcttcatccccctttgcc
SRR1265959.5289237.1-    CAAATGGCAGCATCTGATTCTCTgtaagttgaacagatttcctcttcatccccctttgcc
SRR1265960.14555341.1-   CAAATGGCAGCATCTGATTCTCTgtaagttgaacagatttcctcttcatccccctttgcc
SRR1265960.2099829.1+    CAAATGGCAGCATCTGATTCTCTgtaagttgaacagatttcctctgcatcaccatttgcc
SRR1265962.8694253.2+    CAAATGGCAGCATCTGATTCTCTgtaagttgaacagatttcctcttcatccccctttgcc
SRR1266946.10714685.1+   CAAATGGCAGCATCTGATTCTCTgtaagttgaacagatttcctcttcatccccctttgcc
SRR1266947.19802521.1-   CAAATGGCAGCATCTGATTCTCTgtaagttgaacagatttcctcttcatccccctttgcc
SRR1266947.868250.1-     CAAATGGCAGCATCTGATTCTCTgtaagttgaacagatttcctcttcatccccctttgcc
SRR1266948.14467911.2-   CAAATGGCAGCATCTGATTCTCTgtaagttgaacagatttcctcttcatccccctttgcc
SRR1266949.6049032.2+    CAAATGGCAGCATCTGATTCTCTgtaagttgaacagatttcctcttcatccccctttgcc
SRR1266952.1314922.2+    CAAATGGCAGCATCTGATTCTCTgtaagttgaacagatttcctcttcatccccctttgcc
SRR1266952.14069486.1-   CAAATGGCAGCATCTGATTCTCTgtaagttgaacagatttcctcttcatccccctttgcc
SRR1266956.2653211.2-    CAAATGGCAGCATCTGATTCTCTgtaagttgaacagatttcctcttcatccccctttgcc
SRR1266957.11077584.1-   CAAATGGCAGCATCTGATTCTCTgtaagttgaacagatttcctcttcatccccctttgcc
SRR1266957.2266659.1-    CAAATGGCAGCATCTGATTCTCTgtaagttgaacagatttcctcttcatccccctttgcc
SRR1266959.5837465.1-    CAAATGGCAGCATCTGATTCTCTgtaagttgaacagatttcctcttcatccccctttgcc
SRR1266960.4486561.1+    CAAATGGCAGCATCTGATTCTCTgtaagttgaacagatttcctcttcatccccctttgcc
SRR1266960.7618362.1-    CAAATGGCAGCATCTGATTCTCTgtaagttgaacagatttcctcttcatccccctttgcc
SRR1266961.10701038.1+   CAAATGGCAGCATCTGATTCTCTgtaagttgaacagatttcctctttatccccctttgcc
SRR1266962.4122587.2-    CAAATGGCAGCATCTGATTCTCTgtaagttgaacagatttcctcttcatccccctttncc
SRR1266962.7560910.1+    CAAATGGCAGCATCTGATTCTCTgtaagttgaacagatttcctcttcatccccctttgcc
SRR1266963.7668173.2-    CAAATGGCAGCATCTGATTCTCTgtaagttgaacagatttcctcttcatccccctttgcc
SRR1266964.10482596.2+   CAAATGGCAGCATCTGATTCTCTgtaagttgaacagatttcctcttcatccccctttgcc
SRR1266964.11823893.2+   CAAATGGCAGCATCTGATTCTCTgtaagttgaacagatttcctcttcatccccctttgcc
SRR1266966.10206312.1-   CAAATGGCAGCATCTGATTCTCTgtaagttgaacagatttcctcttcatccccctttgcc
SRR1266966.2152787.1+    CAAATGGCAGCATCTGATTCTCTgtaagttgaacagatttcctcttcatccccctttgcc
SRR1269689.257048.1+     CAAATGGCAGCATCTGATTCTCTgtaagttgaacagatttcctcttcatccccctttgcc
SRR1269689.6495585.2+    CAAATGGCAGCATCTGATTCTCTgtaagttgaacagatttcctcttcatccccctttgcc
SRR1269690.5971251.1+    CAAATGGCAGCATCTGATTCTCTgtaagttgaacagatttcctcttcatccccctttgcc
SRR1269690.6866648.1-    CAAATGGCAGCATCTGATTCTCTgtaagttgaacagatttcctcttcatccccctttgcc
SRR1269691.5900866.1-    CAAATGGCAGCATCTGATTCTCTttaagttgaacagatttcctcttcatccccctttgcc
SRR1269691.6982732.1-    CAAATGGCAGCATCTGATTCTCTgtaagttgaacagatttcctcttcatccccctttgcc
SRR1269692.7437558.2+    CAAATGGCAGCATCTGATTCTCTgtaagttgaacagatttcctcttcatccccctttgcc
SRR1269693.3627362.1+    CAAATGGCAGCATCTGATTCTCTgtaagttgaacagatttcctcttcatccccctttgcc
SRR1269694.995920.2+     CAAATGGCAGCATCTGATTCTCTgtaagttgaacagatttcctcttcatccccctttgcc
SRR1269699.6395347.1+    CAAATGGCAGCATCTGATTCTCTgtaagttgaacagatttcctcttcatccccctttgcc
SRR1269699.8216561.2+    CAAATGGCAGCATCTGATTCTCTgtaagttgaacagatttcctcttcatccccctttgcc
SRR1269700.4999243.2+    CAAATGGCAGCATCTGATTCTCTgtaagttgaacagatttcctcttcatccccctttgcc
SRR1269700.8196703.2+    CAAATGGCAGCATCTGATTCTCTgtaagttgaacagatttcctcttcatccccctttgcc
SRR1269700.9312299.2+    CAAATGGCAGCATCTGATTCTCTgtaagttgaacagatttcctcttcatccccctttgcc
SRR1269703.1602551.2+    CAAATGGCAGCATCTGATTCTCTgtaagttgaacagatttcctcttcatccccctttgcc
SRR1269703.1669213.1-    CAAATGGCAGCATCTGATTCTCTgtaagttgaacagatttcctcttcatccccctttgcc
SRR1269703.7122945.1+    CAAATGGCAGCATCTGATTCTCTgtaagttgaacagatttcctcttcatccccctttgcc
SRR1269720.6697396.1-    CAAATTGCAGCAGCTGACTCTCTgtaagttgaacatatttcctcttcatccccctttgcc
SRR1269721.10925497.1+   CAAATGGCAGCATCTGATTCTCTgtaagttgaacagatttcctcttcatccccctttgcc
SRR1269722.11583054.1+   CAAATGGCAGCATCTGATTCTCTgtaagttgaacagatttcctcttcatccccctttgcc
SRR1269722.612784.2-     CAAATGGCAGCATCTGATTCTCTgtaagttgaacagatttcctcttcatccccctttgcc
SRR1269722.6911698.1+    CAAATGGCAGCATCTGATTCTCTgtaagttgaacagatttcctcttcatccccctttgcc
SRR1269722.7488875.1+    CAAATGGCAGCATCTGATTCTCTgtaagttgaacagatttcctcttcatccccctttgcc
SRR1269723.1055715.2+    CAAATGGCAGCATCTGATTCTCTgtaagttgaacagatttcctcttcatccacctttgcc
SRR1269723.11892874.2-   CAAATGGCAGCATCTGATTCTCTgtaagttgaacagatttcctcttcatccccctttgcc
SRR1269723.12292891.2-   CAAATGGCAGCATCTGATTCTCTgtaagttgaacagatttcctcttcaccccccctttgcc
SRR1269723.5270004.2+    CAAATGGCAGCATCTGATTCTCTgtaagttgaacagatttcctcttcatccccctttgcc
SRR1269725.3442595.2+    CAAATGGCAGCATCTGATTCTCTgtaagttgaacagatttcctcttcatccccctttgcc
SRR1269725.4246389.1+    CAAATGGCAGCATCTGATTCTCTgtaagttgaacagatttcctcttcatccccctttgcc
SRR1269727.2683943.1-    CAAATGGCAGCATCTGATTCTCTgtaatttgaacagatttcctcttcatccccctttgcc
SRR1269727.8907245.1-    CAAATGGCAGCATCTGATTCTCTgtaagttgaacagatttcctcttcatccccctttgcc
SRR1271606.11910612.1-   CAAATGGCAGCATCTGATTCTCTgtaagttgaacagatttcctcttcatccccctttgcc
SRR1271606.5849136.1-    CAAATGGCAGCATCTGATTCTCTgtaagttgaacagatttcctcttcatccccctttgcc
SRR1271609.1031300.2-    CAAATGGCAGCATCTGATTCTCTgtaagttgaacagatttcctcttcatccccctttgcc
SRR1271609.12903351.2+   CAAATGGCAGCATCTGATTCTCTgtaagttgaacagatttcctcttcatccccctttgcc
SRR1271610.19119218.1+   CAAATGGCAGCATCTGATTCTCTgtaagttgaacagatttcctcttcatccccctttgcc
SRR1271610.5829611.2-    CAAATGGCAGCATCTGATTCTCTgtaagttgaacagatttcctcttcatccccctttgcc
SRR1271610.8517146.2-    CAAATGGCAGCATCTGATTCTCTgtaagttgaacagatttcctcttcatccccctttgcc
SRR1271611.30562393.1-   CAAATGGCAGCATCTGATTCTCTgtaagttgaacagatttcctcttcatccccctttgcc
SRR1271611.45475124.1+   CAAATGGCAGCATCTGATTCTCTgtaagttgaacagatttcctcttcatccccctttgcc
SRR1271611.49933549.1+   CAAATGGCAGCATCTGATTCTCTgtaagttgaacagatttcctcttcatccccctttgcc
SRR1271611.51705152.1-   CAAATGGCAGCATCTGATTCTCTgtaagttgaacagatttcctcttcatccccctttgcc
SRR1271611.5176815.2-    CAAATGGCAGCATCTGATTCTCTgtaagttgaacagatttcctcttcatccccctttgcc
SRR1271612.6153622.2-    CAAATGGCAGCATCTGATTCTCTgtaagttgaacagatttcctcttcatccccctttgcc
SRR1271615.5772241.2+    CAAATGGCAGCATCTGATTCTCTgtaagttgaacagatttcctcttcatccccctttgcc
SRR1271616.2011111.1-    CAAATGGCAGCATCTGATTCTCTgtaagttgaacagatttcctcttcatccccctttgcc
```

```
SRR1271616.6540135.1+    CAAATGGCAGCATCTGATTCTCTgtaagttgaacagatttcctcttcatccccctttgcc
SRR1271617.108645868.2+  CAAATGACAGCATCTGATTCTCTgtaagttggacagatttcctcttcatccacctttgcc
SRR1271617.21164005.2-   CAAATGGCAGCATCTGATTCTCTgtaagttgaacagatttcctcttcatccccctttgcc
SRR1271617.35095789.1+   CAAATGGCAGCATCTGATTCTCTgtaagttgaacagatttcctcttcatccccctttgcc
SRR1271617.49734526.2-   CAAATGGCAGCATCTGATTCTCTgtaagttgaacagatttcctcttcatccccctttgcc
SRR1271617.6275825.1+    CAAATGGCAGCATCTGATTCTCTgtaagttgaacagatttcctcttcatccccctttgcc
SRR1271617.65566596.2+   CAAATGGCAGCATCTGATTCTCTgtaagttgaacagatttcctcttcatccccctttgcc
SRR1271617.72688277.1+   CAAATGGCAGCATCTGATTCTCTgtaagttgaacagatttcctcttcatccccctttgcc
SRR1271617.72923099.1+   CAAATGGCAGCATCTGATTCTCTgtaagttgaacagatttcctcttcatccccctttgcc
SRR1271619.11721387.1-   CAAATGGCAGCATCTGATTCTCTgtaagttgaacagatttcctcttcatccccctttgcc
SRR1271620.10933616.2-   CAAATGGCAGCATCTGATTCTCTgtaagttgaacagatttcctcttcatccccctttgcc
SRR1271620.4399325.2+    CAAATGGCAGCATCTGATTCTCTgtaagttgaacagatttcctcttcatccccctttgcc
SRR1271624.1867358.1-    CAAATGGCAGCATCTGATTCTCTgtaagttgaacagatttcctcttcatccccctttgcc
SRR1271624.5962316.1-    CAAATGGCAGCATCTGATTCTCTgtaagttgaacagatttcctcttcatccccctttgcc
SRR1271626.19282964.1-   CAAATGGCAGCATCTGATTCTCTgtaagttgaacagatttcctcttcatccccctttgcc
SRR1271626.27417879.1-   CAAATGGCAGCATCTGATTCTCTgtaagttgaacagatttcctcttcatccccctttgcc
SRR1271626.29048032.2-   CAAATGGCAGCATCTGATTCTCTgtaagttgaacagatttcctcttcatccccctttgcc
SRR1271626.8851170.2-    CAAATGGCAGCATCTGATTCTCTgtaagttgaacagatttcctcttcatccccctttgcc
SRR1271630.6773779.2+    CAAATGGCAGCATCGGATTCTCTgtaagttgaacagatttcctcttcatccccctttgcc
SRR1271630.6944205.1-    CAAATGGCAGCATCTGATTCTCTgtaagttgaacagatttcctcttcatccccctttgcc
SRR1271631.13703327.1-   CAAATGGCAGCATCTGATTCTCTgtaagttgaacagatttcctcttcatccccctttgcc
SRR1271631.17796850.2-   CAAATGGCAGCATCTGATTCTCTgtaagttgaacagatttcctcttcatccccctttgcc
SRR1271631.37714877.2+   CAAATGGCAGCATCTGATTCTCTgtaagttgaacagatttcctcttcatccccctttgcc
SRR1271631.38215481.1+   CAAATGGCAGCATCTGATTCTCTgtaagttgaacagatttcctcttcatccccctttgcc
SRR1271631.39606297.2+   CAAATGGCAGCATCTGATTCTCTgtaagttgaacagatttcctcttcatccccctttgcc
SRR1271631.47115470.1+   CAAATGGCAGCATCTGATTCTCTgtaagttgaacagatttcctcttcatccccctttgcc
SRR1271631.53104753.2-   CAAATGGCAGCATCTGATTCTCTgtaagttgaacagatttcctcttcatccccctttgcc
SRR1271631.53790917.1-   CAAATGGCAGCATCTGATTCTCTgtaagttgaacagatttcctcttcatccccctttgcc
SRR1271631.60482848.1-   CAAATGGCAGCATCTGATTCTCTgtaagttgaacagatttcctcttcatccccctttgcc
SRR1271631.65104355.1+   CAAATGGCAGCATCTGATTCTCTgtaagttgaacagatttcctcttcatccccctttgcc
SRR1271631.8158897.1+    CAAATGGCAGCATCTGATTCTCTgtaagttgaacagatttcctcttcatccccctttgcc
SRR1271636.11917632.2+   CAAATGGCAGCATCTGATTCTCTgtaagttgaacagatttcctcttcatccccctttgcc
SRR1271636.20236431.1-   CAAATGGCAGCATCTGATTCTCTgtaagttgaacagatttcctcttcatccccctttgcc
SRR1271637.9483811.2-    CAAATGGCAGCATCTGATTCTCTgtaagttgaacagatttcctcttcatccccctttgcc
SRR1271638.7137901.2-    CAAATGGCAGCATCTGATTCTCTgtaagttgaacagatttcctcttcatccccctttgcc
SRR1271639.12989136.1-   CAAATGGCAGCATCTGATTCTCTgtaagttgaacagatttcctcttcatccccctttgcc
SRR1271641.1828285.1-    CAAATGGCAGCATCTGATTCTCTgtaagttgaacagatttcctcttcatccccctttgcc
SRR1271641.2977584.2+    CAAATGGCAGCATCTGATTCTCTgtaagttgaacagatttcctcttcatccccctttgcc
SRR1271642.249531.2-     CAAATGGCAGCATCTGATTCTCTgtaagttgaacagatttcctcttcatccccctttgcc
SRR1271642.3018005.2-    CAAATGGCAGCATCTGATTCTCTgtaagttgaacagatttcctcttcatccccctttgcc
SRR1271644.6211925.1-    CAAATGGCAGCATCTGATTCTCTgtaagttgaacagatttcctcttcatccccctttgcc
SRR1271656.31699611.2-   CAAATGGCAGCATCTGATTCTCTgtaagttgaacagatttcctcttcatccccctttgcc
SRR1271656.37726636.1-   CAAATGGCAGCATCTGATTCTCTgtaagttgaacagatttcctcttcatccccctttgcc
SRR1271656.42884483.1-   CAAATGGCAGCATCTGATTCTCTgtaagttgaacagatttcctcttcatccccctttgcc
SRR1271656.47447557.1+   CAAATGGCAGCATCTGATTCTCTgtaagttgaacagatttcctcttcatccccctttgcc
SRR1271660.12843375.2+   CAAATGGCAGCATCTGATTCTCTgtaagttgaacagatttcctcttcatccccctttgcc
SRR1271660.18416623.1+   CAAATGGCAGCATCTGATTCTCTgtaagttgaacagatttcctcttcatccccctttgcc
SRR1271660.25394364.1+   CAAATGGCAGCATCTGATTCTCTgtaagttgaacagatttcctcttcatccccctttgcc
SRR1271660.28173172.1+   CAAATGGCAGCATCTGATTCTCTgtaagttgaacagatttcctcttcatccccctttgcc
SRR1271661.66238.1+      CAAATGGCAGCATCTGATTCTCTgtaagttgaacagatttcctcttcatccccctttgcc
SRR1271661.9678304.2-    CAAAAGGCAGCATCTGATTCTCTgtaagttgaacagatttcctcttcatccccctttgcc
SRR1271664.1051398.2+    CAAATGGCAGCATCTGATTCTCTgtaagttgaacagatttcctcttcatccccctttgcc
SRR1271664.9315059.1-    CAAATGGCAGCATCTGATTCTCTgtaagttgaacagatttcctcttcatccccctttgcc
SRR1271665.16602572.2-   CAAATGGCAGCATCTGATTCTCTgtaagttgaacagatttcctcttcatccccctttgcc
SRR1271665.17092941.1+   CAAATGGCAGCATCTGATTCTCTgtaagttgaacagatttcctcttcataccccctttgcc
SRR1271666.2776816.1-    CAAATGGCAGCATCTGATGCTCTgtaagttgaacagatttcctcttcatccccctttgcc
SRR1271667.7988823.1+    CAAATGGCAGCATCTGATTCTCTgtaagttgaacagatttcctcttcatccccctttgcc
SRR1265959.13791605.1-    AAATGGCAGCATCTGATTCTCTgtaagttgaacagatttcctcttcatccccctttgcc
SRR1266955.9340457.1-      AATGGCAGCATCTGATTCTCTgtaagttgaacagatttcctcttcatccccctttgcc
SRR1271631.89798142.1-     AATGGCAGCATCTGATTCTCTgtaagttgaacagatttcctcttcatccccctttgcc
SRR1271636.28227184.1+     AATGGCAGCATCTGATTCTCTgtaagttgaacagatttcctcttcatccccctttgcc
SRR1271636.5133246.1+      AATGGCAGCATCTGATTCTCTgtaagttgaacagatttcctcttcatccccctttgcc
SRR1271643.5399607.2+      AATGGCAGCATCTGATTCTCTgtaagttgaaaagatttcctcttcatccccctttgcc
SRR1271617.18167979.2+      ATGGCAGCATCTGATTCTCTgtaagttgaacagatttcctcttcatccccctttgcc
SRR1271627.9333660.2+       ATGGCAGCATCTGATTCTCTgtaagttgaacagatttcctcttcatccccctttgcc
SRR1265423.845833.2-         TGGCAGCATCTGATTCTCTgtaagttgaacagatttcctcttcatccccctttgcc
SRR1266966.437734.1-         TGGCAGCATCTGATTCTCTgtaagttgaacagatttcctcttcatccccctttgcc
SRR1265105.380641.1+          GGCAGCATCTGATTCTCTgtaagttgaacagatttcctcttcatccccctttgcc
SRR1266957.3246519.2-         GGCAGCATCTGATTCTCTgtaagttgaacagatttcctcttcacccccctttgcc
SRR1269727.7734711.1-         GGCAGCATCTGATTCTCTgtaagttgaacagatttcctcttcatccccctttgcc
SRR1271618.11104575.2+        GGCAGCATCTGATTCTCTgtaagttgaacagatttcctcttcatccccctttgcc
SRR1271656.45740696.1-         GCAGCATCTGATTCTCTgtaagttgaacagatttcctcttcatccccctttgcc
SRR1266955.5156734.1-           CAGCATCTGATTCTCTgtaagtagaaaagatttcctcttcatccccctttgcc
SRR1269725.9933502.1+           CAGCATCTGATTCTCTgtaagttgaacagatttcctcttcatccccctttgcc
SRR1271623.13691659.2+          CAGCATCTGATTCTCTgtaagttgaacagatttcctcttcatccccctttgcc
SRR1265093.569959.2+             AGCATCTGATTCTCTgtaagttgaacagatttcctcttcatccccctttgcc
SRR1265418.2535443.2-            AGCATCTGATTCTTTgtaagttgaacagatttcctcttcatccccctttgcc
SRR1265485.16227498.1+           AGCATCTGATTCTCTgtaagttgaacagatttcctcttcatccccctttgcc
SRR1271631.73182052.2-           AGCATCTGATTCTCTgtaagttgaacagatttcctcttcatccccctttgcc
SRR1271640.6710261.2+            AGCATCTGATTCTCTgtaagttgaacagatttcctcttcatccccctttgcc
SRR1266957.1004692.1-             GCATCTGATTCTCTgtaagttgaacagatttcctcttcatccccctttgcc
SRR1271624.7572085.1+             GCATCTGATTCTCTgtaagttgaacagatttcctcttcatccccctttgcc
SRR1271660.17854389.2+            GCATCTGATTCTCTgtaagttgaacagatttcctcttcatccccctttgcc
SRR1265093.9367806.2-              CATCTTATTCTCTgtaagttgaacagatttcctcttcatccccctttgcc
```

```
SRR1266954.5050757.1+             CATCTGATTCTGTgtaagttgaacagatttcctcttcatccccctttgcc
SRR1266957.3195693.1-             CATCTTATTCTCTgtaagttgaacagatttcctcttcacccccctttgcc
SRR1269703.3019169.1+             CATCTGATTCTCTgtaagttgaacagatttcctcttcatccccctttgcc
SRR1271606.314344.1+              CATCTGATTCTCTgtaagttgaacagatttcctcttcatccccctttgcc
SRR1271639.16935460.2-            CATCTGATTCTCTgtaagttgaacagatttcctcttcatccccctttgcc
SRR1266951.7331141.2+               TCTGATTCTCTgtaagttgaacagatttcctcttcatccccctttgcc
SRR1266947.22343126.1+               CTGATTCTCTgtaagttgaacagatttcctcttcatccccctttgcc
SRR1266963.20780247.2+               CTGATTCTCTgtaagttgaacagatttcctcttcatccccctttgcc
SRR1265428.8823863.2+                 TGATTCTCTgtaagttgaacagatttcctcttcatccccctttgcc
SRR1265487.506939.1+                  TGATTCTCTgtaagttgaacagatttcctcttcatccccctttgcc
SRR1266955.7154550.2-                 TGATTCTCTgtaagttgaacagatttcctcttcatccccctttgcc
SRR1271611.1989144.1-                 TGATTCTCTgtaagttgaacagatttcctcttcatccccctttgcc
SRR1271660.19233729.2-                   TTCTCTgtaagttgaacagatttcctcttcatccccctttgcc
SRR1271611.10364010.2-                    TCTCTgtaagtttaacagatttcctcttcaaccccctttgcc
SRR1271623.3169311.1+                     TCTCTgtaagttgaacagatttcctcttcatccccctttgcc
SRR1271606.223591.2+                       CTCTgtaagttgaacagatttcctcttcatccccctttgcc
SRR1271630.3176113.2-                      CTCTgtaagttaaacagatttcctcttcatccccctttgcc
SRR1271666.16618310.2+                     CTCTgtaagttgaacagatttcctcttcatccccctttgcc
SRR1271617.69327822.1+                      TCTgtaagttgaacagatttcctcttcatccccctttgcc
SRR1265108.2919013.2+                        CTgtaagttgaacagatttcctcttcatccccctttgcc
SRR1265425.8225295.1-                        CTgtaaggtgaacagatttcctcttcatccccctttccc
SRR1265108.2515604.2-                         Tgtaagttgaacagctttccgcttcatccccctttgcc
SRR1265427.10637217.2+                        Tgtaagttgaacagatttcctcttcatccccctttgcc
SRR1271617.85496141.1+                        Tgtaagttgaacagatttcctcttcatccccctttgcc
SRR1271623.14435698.1-                        Tgtaagttgaacagatttcctcttcatccccctttgcc
SRR1271635.8499862.1-                         Tgtaagttgaacagattccctcttcatccccctttgcc
SRR1271664.496248.2-                          Tgtaagttgaacagatttcctcttcatccccctttgcc

consensus               CAAATGGCAGCATCTGATTCTCTgtaagttgaacagatttcctcttcatccccctttgcc
```

D. Ground tit (No. 5), exon 1, WGS data

- CAP3 alignment of WGS data spanning exon 1 (uppercase letters)

```
                              .    :    .    :    .    :    .    :    .    :    .    :
SRR765711.127108613.2+   ctgcccaaggtatcagcaagacttagaggtaccagagactgcagagA
SRR765714.96762027.1-    ctgcccaaggtatcagcaagacttagaggtaccagagactgcagagAT
SRR765715.28535392.2-    ctgcccaaggtatcagcaagacttagaggtaccagagactgcagagATG
SRR765715.40711716.2+    ctgcccaaggtatcagcaagacttagaggtaccagagactgcagagATG
SRR765715.15325357.1+    ctgcccaaggtatcagcaagacttagaggtaccagagactgcagagATGA
SRR765715.20079751.1+    ctgcccaaggtatcagcaagacttagaggtaccagagactgcagagATGA
SRR765711.98788597.1+    ctgcccaaggtatcagcaagacttagaggtaccagagactgcagagATGAT
SRR765712.191725402.2-   ctgcccaaggtatcagcaagacttagaggtaccagagactgcagagATGAT
SRR765712.221242791.2+   ctgcccaaggtatcagcaagacttagaggtaccagagactgcagagATGAT
SRR765714.120567258.1-   ctgcccaaggtatcagcaagacttagaggtaccagagactgcagagATGAT
SRR765714.60800763.1-    ctgcccaaggtatcagcaagacttagaggtaccagagactgcagagATGAT
SRR765714.90916047.1-    ctgcccaaggtatcagcaagacttagaggtaccagagactgcagagATGAT
SRR765710.43360571.2-    ctgcccaaggtatcagcaagacttagaggtaccagagactgcagagATGATAG
SRR765711.93189275.1+    ctgcccaaggcatcagcaagacttagaggtaccagagactgcagagATGATAG
SRR765714.80472540.1-    ctgcccaaggtatcagcaagacttagaggtaccagagactgcagagATGATAG
SRR747825.25590730.1-    ctgcccaaggtatcagcaagacttagaggtaccagagactgcagagATGATAGT
SRR747825.25736700.1-    ctgcccaaggtatcagcaagacttagaggtaccagagactgcagagATGATAGT
SRR765711.43336047.1+    ctgcccaaggtatcagcaagacttagaggtaccagagactgcagagATGATAGC
SRR765712.202793541.2-   ctgcccaaggtatcagcaagacttagaggtaccagagactgcagagATGATAGTG
SRR765709.3594615.1-     ctgcccaaggtatcagcaagacttagaggtaccagagactgcagagATGATAGTGAT
SRR765711.27058616.1+    ctgcccaaggtatcagcaagacttagaggtaccagagactgcagagATGATAGTGAT
SRR765712.201848235.2+   ctgcccaaggtatcagcaagacttagaggtaccagagactgcagagATGATAGTGAT
SRR765712.223930320.1+   ctgcccaaggtatcagcaagacttagaggtaccagagactgcagagATGATAGTGAT
SRR765712.71905088.1-    ctgcccaaggtatcagcaagacttagaggtaccagagactgcagagATGATAGTGAT
SRR765714.96238274.1-    ctgcccaaggtatcagcaagacttagaggtaccagagactgcagagATGATAGTGAT
SRR765715.25873242.1+    ctgcccaaggtatcagcaagacttagaggtaccagagactgcagagATGATAGCGAT
SRR765709.50792463.1-    ctgcccaaggtatcagcaagacttagaggtaccagagactgcagagATGATAGCGATG
SRR765709.10978190.1-    ctgcccaaggtatcagcaagacttagaggtaccagagactgcagagATGATAGCGATGTT
SRR765709.11048444.2-    ctgcccaaggtatcagcaagacttagaggtaccagagactgcagagATGATAGTGATGTT
SRR765709.15051614.2+    ctgcccaaggtatcagcaagacttagaggtaccagagactgcagagATGATAGTGATGTT
SRR765709.17947042.1+    ctgcccaaggtatcagcaagacttagaggtaccagagactgcagagATGATAGTGATGTT
SRR765709.84981878.1-    ctgcccaaggtatcagcaagacttagaggtaccagagactgcagagATGATAGCGATGTT
SRR765710.14520134.2+    ctgcccaaggtatcagcaagacttagaggtaccagagactgcagagATGATAGCGATGTT
SRR765710.20061203.1+    ctgcccaaggtatcagcaagacttagaggtaccagagactgcagagATGATAGCGATGTT
SRR765710.30559922.2-    ctgcccaaggtatcagcaagacttagaggtaccagagactgcagagATGATAGTGATGTT
SRR765710.48969246.1+    ctgcccaaggtatcagcaagacttagaggtaccagagactgcagagATGATAGTGATGTT
SRR765710.52432273.1+    ctgcccaaggtatcagcaagacttagaggtacgagagtctgcagagATGATAGCGATGTT
SRR765710.52899931.2+    ctgcccaaggtatcagcaagacttagaggtaccagagactgcagagATGATAGCGATGTT
SRR765710.58473373.1-    ctgcccaaggtatcagcaagacttagaggtaccagagactgcagagATGATAGCGATGTT
SRR765710.59709380.1-    ctgcccaaggtatcagcaagacttagaggtaccagagactgcagagATGATAGTGATGTT
SRR765710.62324211.2+    ctgcccaaggtatcagcaagacttagaggtaccagagactgcagagATGATAGTGATGTT
SRR765710.6314.2+        ctgcccaaggtatcagcaagacttagaggtaccagagactgcagagATGATAGTGATGTT
SRR765710.72900439.2+    ctgcccaaggtatcagcaagacttagaggtaccagagactgcagagATGATAGCGATGTT
SRR765710.78927757.2-    ctgcccaaggtatcagcaagacttagaggtaccagagactgcagagATGATAGTGATGTT
SRR765710.83849557.2+    ctgcccaaggtatcagcaagacttagaggtaccagagactgcagagATGATAGCGATGTT
SRR765711.115303767.1+   ctgcccaaggtatcagcaagacttagaggtaccagagactgcagagATGATAGCGATGTT
SRR765711.116926262.2+   ctgcccaaggtatcagcaagacttagaggtaccagagactgcagagATGATAGCGATGTT
SRR765711.12467307.2-    ctgcccaaggtatcagcaagacttagaggtaccagagactgcagagATGATAGCGATGTT
SRR765711.125009762.2+   ctgcccaaggtatcagcaagacttagaggtaccagagactgcagagATGATAGCGATGTT
SRR765711.136262268.1+   ctgcccaaggtatcagcaagacttagaggtaccagagactgcagagATGATAGCGATGTT
SRR765711.136914716.1+   ctgcccaaggtatcagcacgacttagaggtaccagagactgcagagATGATAGTGATGTT
SRR765711.141606739.1+   ctgcccaaggtatcagcaagacttagaggtaccagagactgcagagATGATAGTGATGTT
SRR765711.39537275.2+    ctgcccaaggtatcagcaagacttagaggtaccagagactgcagagATGATAGCGATGTT
SRR765711.39985328.1+    ctgcccaaggtatcagcaagacttagaggtaccagagactgcagagATGATAGTGATGTT
SRR765711.57382922.1+    ctgcccaaggtatcagcaagacttagaggtaccagagactgcagagATGATAGTGATGTT
SRR765711.58178683.2+    ctgcccaaggtatcagcaagacttagaggtaccagagactgcagagATGATAGCGATGTT
SRR765711.65007596.1+    ctgcccaaggtatcagcaagacttagaggtaccagagactgcagagATGATAGCGATGTT
SRR765711.75189809.1+    ctgcccaaggtatcagcaagacttagaggtaccagagactgcagagATGATAGCGATGTT
SRR765711.75336142.1+    ctgcccaaggtatcagcaagacttagaggtaccagagactgcagagATGATAGTGATGTT
SRR765711.90300589.2+    ctgcccaaggtatcagcaagacttagaggtaccagagactgcagagATGATAGTGATGTT
SRR765711.96129668.2+    ctgcccaagggatcagcaagacttagaggtaccagagactgcagagATGATAGCGATGTT
SRR765712.102108300.1+   ctgcccaaggtatcagcaagacttagaggtaccagagactgcagagATGATAGTGATGTT
SRR765712.103022563.2-   ctgcccaaggtatcagcaagacttagaggtaccagagactgcagagATGATAGTGATGTT
SRR765712.108514153.2+   ctgcccaaggtatcagcaagacttagaggtaccagagactgcagagATGATAGTGATGTT
SRR765712.128813487.2-   ctgcccaaggtatcagcaagacttagaggtaccagagactgcacagATGATAGTGATGTT
SRR765712.170972669.2+   ctgcccaaggtatcagcaagacttagaggtaccagagactgcagagATGATAGTGATGTT
SRR765712.178874795.1+   ctgcccaagatatcagcaagacttagaggtaccagagactgcagagATGATAGTGATGTT
SRR765712.19285418.2-    ctgcccaaggtatcagcaagacttagaggtaccagagactgcagagATGATAGTGATGTT
SRR765712.204517588.1-   ctgcccaaggtatcagcaagacttagaggtaccagagactgcagagATGATAGTGATGTT
SRR765712.209293117.1-   ctgcccaaggtatcagcaagacttagaggtaccagagactgcagagATGATAGCGATGTT
SRR765712.221242791.1-   ctgcccaaggtatcagcaagacttagaggtaccagagactgcagagATGATAGCGATGTT
SRR765712.227153236.1+   ctgcccaaggtatcagcaagacttagaggtaccagagactgcagagATGATAGCGATGTT
SRR765712.227855078.2-   ctgcccaaggtatcagcaagacttagaggtaccagagactgcagagATGATAGTGATGTT
SRR765712.36663455.2-    ctgcccaaggtatcagcaagacttagaggtaccagagactgcagagATGATAGTGATGTT
SRR765712.39001705.1-    ctgcccaaggtatcagcaagacttagaggtaccagagactgcagagATGATAGTGATGTT
```

```
SRR765712.44471210.2+     ctgcccaaggtatcagcaagacttagaggtaccagagactgcagagATGATAGCGATGTT
SRR765712.89074862.2-     ctgcccaaggtatcagcaagacttagaggtaccagagactgcagagATGATAGTGATGTT
SRR765712.9126008.2+      ctgcccaaggtatcagcaagacttagaggtaccagagactgcagagATGATAGCGATGTT
SRR765714.103477576.1-    ctgcccaaggtatcagcaagacttagaggtaccagagactgcagagATGATAGTGATGTT
SRR765714.104135391.1-    ctgcccaaggtatcagcaagacttagaggtaccagagactgcagagATGATAGTGATGTT
SRR765714.115256777.1-    ctgcccaaggtatcagcaagacttagaggtaccagagactgcagagATGATAGTGATGTT
SRR765714.118843472.1-    ctgcccaaggtatcagcaagacttagaggtaccagagactgcagagATGATAGTGATGTT
SRR765714.1288495.1-      ctgcccaaggtatcagcaagacttagaggtaccagagactgcagagATGATAGCGATGTT
SRR765714.129290600.1-    ctgcccaaggtatcagcaagacttagaggtaccagagactgcagagATGATAGTGATGTT
SRR765714.139482311.1+    ctgcccaaggtatcagcaagacttagaggtaccagagactgcagagATGATAGCGATGTT
SRR765714.144000723.2-    ctgcccaaggtatcagcaagacttagaggtaccggagactgcagagATGATAGTGATGTT
SRR765714.152535865.1-    ctgcccaaggtatcagcaagacttagaggtaccagagactgcagagATGATAGTGATGTT
SRR765714.179739684.1-    ctgcccaaggtatcagcaagacttagaggtaccagagactgcagagATGATAGTGATGTT
SRR765714.181419219.2-    ctgcccaaggtatcagcaagacttagaggtaccagagactgcagagATGATAGTGATGTT
SRR765714.185244055.1-    ctgcccaaggtatcagcaagacttagaggtaccagagactgcagagATGATAGCGATGTT
SRR765714.196951609.2-    ctgcccaaggtatcagcaagacttagaggtaccagagactgcagagATGATAGCGATGTT
SRR765714.20194193.2+     ctgcccaaggtatcagcaagacttagaggtaccagagactgcagagATGATAGCGATGTT
SRR765714.20771235.1-     ctgcccaaggtatcagcaagacttagaggtaccagagactgcagagATGATAGTGATGTT
SRR765714.632864.1-       ctgcccaaggtatcagcaagacttagaggtaccagagactgcagagATGATAGTGATGTT
SRR765714.71369973.2-     ctgcccaaggtatcagcaagacttagaggtaccagagactgcagagATGATAGCGATGTT
SRR765714.87789096.1-     ctgcccaaggtatcagcaagacttagaggtaccagagactgcagagATGATAGTGATGTT
SRR765714.95133774.1-     ctgcccaaggtatcagcaagacttagaggtaccagagactgcagagATGATAGTGATGTT
SRR765715.12220715.1-     ctgcccaaggtatcagcaagacttagaggtaccagagactgcagagATGATAGTGATGTT
SRR765715.15093018.1-     ctgcccaaggtatcagcaagacttagaggtaccagagactgcagagATGATAGTGATGTT
SRR765715.29951049.1+     ctgcccaaggtatcagcaagacttagaggtaccagagactgcagagATGATAGTGATGTT
SRR765715.39302854.1-     ctgcccaaggtatcagcaagacttagaggtaccagagactgcagagATGATAGTGATGTT
SRR765716.412165.1+       ctgcccaaggtatcagcaagacttagaggtaccagagactgcagagATGATAGTGATGTT
SRR765716.5724759.1+      ctgcccaaggtatcagcaagacttagaggtaccagagactgcagagATGATAGTGATGTT
SRR765716.7847479.2+      ctgcccaaggtatcagcaagacttagaggtaccagagactgcagagATGATAGTGATGTT
SRR765710.52880643.1-      tgcccaaggtatcagcaagacttagaggtaccagagactgcagagATGATAGTGATGTT
SRR765711.16163481.2-       gcccaaggtatcagcaagacttagaggtaccagagactgcagagATGATAGCGATGTT
SRR765711.76231448.1+       gcccaaggtatcagcaagacttagaggtaccagagactgcagagATGATAGTGATGTT
SRR765715.17513876.2+       gcccaaggtatcagcaagacttagaggtaccagagactgcagagATGATAGCGATGTT
SRR765711.106750007.1-       cccaaggtatcagcaagacttagaggtaccagagactgcagagATGATAGCGATGTT
SRR765709.19446554.1-         ccaaggtatcagcaagacttagaggtaccagagactgcagagATGATAGCGATGTT
SRR765711.78666991.2-         ccaaggtatcagcaagacttagaggtaccagagactgcagagATGATAGTGATGTT
SRR765710.58041512.1-          caaggtatcagcaagacttagaggtaccagagactgcagagATGATAGCGATGTT
SRR765711.115598291.2-          aaggtatcagcaagacttagaggtaccagagactgcagagATGATAGTGATGTT
SRR765710.50972813.1-            aggtatcagcaagacttagaggtaccagagactgcagagATGATAGTGATGTT
SRR765712.166853237.1+           aggtatcagcaagacttagaggtacaagagactgcagagATGATAGTGATGTT
SRR765712.7129698.2-             aggtatcagcaagacttagaggtaccagagactgcagagATGATAGCGATGTT
SRR765711.24558845.1-              gtatcagcaagacttagaggtaccagagactgcagagATGATAGCGATGTT
SRR765711.79934409.2-              gtatcagcaagacttagaggtaccagagactgcagagATGATAGTGATGTT
SRR765712.73428311.2+              gtatcagcaagacttagaggtaccagagactgcagagATGATAGCGATGTT
SRR765709.56879908.2+               tatcagcaagacttagaggtaccagagactgcagagATGATAGTGATGTT
SRR765712.70145514.2-                  cagcaagacttagaggtaccagagactgcagagATGATAGTGATGTT
SRR765711.79688649.1+                   agcaagacttagaggtaccagagactgcagagATGATAGTGATGTT
SRR765712.106710182.1+                  agcaagacttagaggtaccagagactgcagagATGATAGCGATGTT
SRR765712.82312151.1-                   agcaagacttagaggtaccagagactgcagagATGATAGTGATGTT
SRR765714.105503876.2-                  agcaagacttagaggtaccagagactgcagagATGATAGTGATGTT
SRR765714.115172876.2-                  agcaagacttagaggtaccagagactgcagagATGATAGTGATGTT
SRR765712.141280360.1-                   gcaagacttagaggtaccagagactgcagagATGATAGCGATGTT
SRR765710.6874784.2+                        agacttagaggtaccagagactgcagagATGATAGCGATGTT
SRR765711.70787159.1+                         acttagaggtaccagagactgcagagATGATAGTGATGTT
SRR765715.41596894.1-                            tagaggtaccagagactgcagagATGATAGTGATGTT
SRR765711.81304240.1+                             agaggtaccagagactgcagagATGATAGCGATGTT
SRR765712.80351652.1+                             agaggtaccagagactgcagagATGATAGTGATGTT
SRR765714.90251705.1-                             agaggtaccagagactgcagagATGATAGCGATGTT
SRR765710.77934178.2+                              gaggtaccagagactgcagagATGATAGTGATGTT
SRR765711.42060791.1+                              gaggtaccagagactgcagagATGATAGCGATGTT
SRR747825.15514581.2+                               aggtaccagagactgcagagATGATAGTGATGTT
SRR747825.2399014.1+                                aggtaccagagactgcagagATGATAGTGATGTT
SRR747825.25077350.1+                               aggtaccagagactgcagagATGATAGTGATGTT
SRR747825.32125711.2+                               aggtaccagagactgcagagATGATAGTGATGTT
SRR747825.34186382.2+                               aggtaccagagactgcagagATGATAGTGATGTT
SRR747825.36676712.1+                               aggtaccagagactgcagagATGATAGTGATGTT
SRR747825.36796222.1+                               aggtaccagagactgcagagATGATAGTGATGTT
SRR747825.37802110.2+                               aggtaccagagactgcagagATGATAGTGATGTT
SRR747825.41358891.2+                               aggtaccagagactgcagagATGATAGTGATGTT
SRR747825.42101809.1+                               aggtaccagatactgcagagATGATAGTGATGTT
SRR747825.42675926.1+                               aggtaccagagactgcagagATGATAGTGATGTT
SRR747825.7479104.2+                                aggtaccagagactgcagagATGATAGTGATGTT
SRR747825.9216947.2+                                aggtaccagagactgcagagATGATAGTGATGTT
SRR765709.89392740.2-                                 gtaccagagactgcagagATGATAGCGATGTT
SRR765711.58856886.1-                                 gtaccagagactgcagagATGATAGCGATGTT
SRR765712.223930320.2-                                 taccagagactgcagagATGATAGTGATGTT
SRR765714.15028586.1-                                  taccagagactgcagagATGATAGCGATGTT
SRR765716.250216.2-                                    taccagagactgcagagATGATAGCGATGTT
SRR765716.25173572.2-                                  taccagagactgcagagATGATAGCGATGTT
SRR765716.26119942.2-                                  taccagagactgcagagATGATAGCGATGTT
SRR765710.48848881.2+                                    ccagagactgcagagATGATAGCGATGTT
SRR765715.8423602.1+                                     ccagagactgcagagATGATAGCGATGTT
SRR765710.83849557.1-                                      agagactgcagagATGATAGCGATGTT
SRR765710.62324211.1-                                       gagactgcagagATGATAGTGATGTT
```

```
SRR765709.27281621.2-                                       agactgcagagATGATAGCGATGTT
SRR765711.127108613.1-                                      agactgcagagATGATAGTGATGTT
SRR765716.23347563.1+                                        gactgcagagATGATAGTGATGTT
SRR765710.52899931.1-                                         actgcagagATGATAGCGATGTT
SRR765710.53338134.1+                                         actgcagagATGATAGTGATGTT
SRR765711.41431931.1+                                         actgcagagATGATAGCGATGTT
SRR765712.225295283.2-                                        actgcagagATGATAGTGATGTT
SRR765714.92358977.1+                                         actgcagagATGATAGCGATGTT
SRR765712.73551137.1+                                          ctgcagagATGATAGCGATGTT
SRR765712.201848235.1-                                           gcagagATGATAGTGATGTT
SRR765710.72900439.1-                                             cagagATGATAGCGATGTT
SRR765711.120800792.2+                                            cagagATGATAGTGATGTT
SRR765711.93189275.2-                                             cagagATGATAGTGATGTT
SRR765712.108514153.1-                                             agagATGATAGTGATGTT
SRR765714.45571282.1-                                                gagATGATAGTGATGTT
SRR765711.13528758.1+                                                 agATGATAGCGATGTT
SRR765711.43336047.2-                                                 agATGATAGCGATGTT
SRR765711.50432206.2+                                                 agATGATAGCGATGTT
SRR765714.123035075.2-                                                agATGATAGCGATGTT
SRR765714.162066143.2+                                                agATGATAGCGATGTT
SRR765709.5623476.2+                                                    ATGATAGCGATGTT
SRR765711.98788597.2-                                                   ATGATAGCGATGTT
SRR765712.9126008.1-                                                    ATGATAGCGATGTT
SRR765714.12218431.2+                                                   ATGATAGCGATGTT
SRR765710.6314.1-                                                        TGATAGTGATGTT
SRR765710.14520134.1-                                                     GATAGCGATGTT
SRR765715.16533716.2+                                                     GATAGTGATGTT
SRR765711.96129668.1-                                                      ATAGCGATGTT
SRR765709.16812281.2-                                                       TAGCGATGTT
SRR765710.48969246.2-                                                        AGTGATGTT
SRR765714.181565026.1+                                                       AGCGATGTT
SRR765711.15445266.2+                                                         GTGATGTT
SRR765712.227153236.2-                                                        GCGATGTT
SRR765716.28097872.2+                                                         GCGATGTT
SRR765711.136914716.2-                                                         TGATGTT
SRR765709.80799489.2+                                                            ATGTT
SRR765714.73397308.2-                                                            ATGTT
SRR765714.76267865.2+                                                            ATGTT
SRR765711.27058616.2-                                                             TGTT
SRR765712.208044178.1+                                                            TGTT
SRR765715.22238685.2+                                                             TGTT
SRR765715.3175093.1-                                                              TGTT
SRR765715.3175093.2+                                                              TGTT
SRR765710.52432273.2-                                                              GTT
SRR765711.39985328.2-                                                              GTT
SRR765712.22158653.2+                                                              GTT
SRR765711.141606739.2-                                                              TT
SRR765712.29757431.1+                                                               TT
SRR765715.22238685.1-                                                               TT
SRR765712.71088928.1+                                                                T
                         ____________________________________________________________
consensus                ctgcccaaggtatcagcaagacttagaggtaccagagactgcagagATGATAGTGATGTT

                             .    :    .    :    .    :    .    :    .    :    .    :
SRR765715.29951049.1+    CT
SRR765710.62324211.2+    CTT
SRR765712.227153236.1+   CTTT
SRR765714.1288495.1-     CTTT
SRR765714.179739684.1-   CTTT
SRR765715.17513876.2+    CTTT
SRR765714.103477576.1-   CTTTT
SRR765714.152535865.1-   CTTTT
SRR765714.632864.1-      CTTTT
SRR765714.95133774.1-    CTTTT
SRR765709.10978190.1-    CTTTTC
SRR765712.128813487.2-   CTTTTCA
SRR765712.227855078.2-   CTTTTCA
SRR765714.20194193.2+    CTTTTCA
SRR765715.12220715.1-    CTTTTCAGG
SRR765711.39985328.1+    CTTTTCAGGA
SRR765712.204517588.1-   CTTTTCAGGA
SRR765710.52899931.2+    CTTTTCAGGAA
SRR765711.141606739.1+   CTTTTCAGGAATC
SRR765714.181419219.2-   CTTTTCAGGAATC
SRR765711.96129668.2+    CTTTTCAGGAATCA
SRR765710.52432273.1+    CTTTTCAGGAATCAAG
SRR765716.412165.1+      CTTTTCAGGAATCAAG
SRR765716.5724759.1+     CTTTTCAGGAATCAAG
SRR765716.7847479.2+     CTTTTCAGGAATCAAG
SRR765710.14520134.2+    CTTTTCAGGAATCAAGAG
SRR765710.83849557.2+    CTTTTCAGGAATCAAGAG
SRR765714.139482311.1+   CTTTTCAGGAATCAAGAG
SRR765709.15051614.2+    CTTTTCAGGAATCAAGAGA
SRR765711.58178683.2+    CTTTTCAGGAATCAAGAGAA
```

SRR765714.129290600.1- CTTTTCAGGAATCAAGAGAA
SRR765714.20771235.1- CTTTTCAGGAATCAAGAGAA
SRR765714.87789096.1- CTTTTCAGGAATCAAGAGAA
SRR765711.136914716.1+ CTTTTCAGGAATCAAGAGAAT
SRR765712.36663455.2- CTTTTCAGGAATCAAGAGAAT
SRR765712.103022563.2- CTTTTCAGGAATCAAGAGAATG
SRR765714.104135391.1- CTTTTCAGGAATCAAGAGAATG
SRR765715.15093018.1- CTTTTCAGGAATCAAGAGAATG
SRR765709.17947042.1+ CTTTTCAGGAATCAAGAGAATGCTC
SRR765711.39537275.2+ CTTTTCAGGAATCAAGAGAATGCTC
SRR765712.178874795.1+ CTTTTCAGGAATCAAGAGAATGCTC
SRR765712.221242791.1- CTTTTCAGGAATCAAGAGAATGCTC
SRR765712.39001705.1- CTTTTCAGGAATCAAGAGAATGCTC
SRR765710.72900439.2+ CTTTTCAGGAATCAAGAGAATGCTCT
SRR765711.125009762.2+ CTTTTCAGGAATCAAGAGAATGCTCT
SRR765711.75189809.1+ CTTTTCAGGAATCAAGAGAATGCTCT
SRR765712.89074862.2- CTTTTCAGGAATCAAGAGAATGCTCT
SRR765714.115256777.1- CTTTTCAGGAATCAAGAGAATGCTCT
SRR765714.185244055.1- CTTTTCAGGAATCAAGAGAATGCTCT
SRR765711.115303767.1+ CTTTTCAGGAATCAAGAGAATGCTCTT
SRR765710.6314.2+ CTTTTCAGGAATCAAGAGAATGCTCTTC
SRR765711.90300589.2+ CTTTTCAGGAATCAAGAGAATGCTCTTC
SRR765712.102108300.1+ CTTTTCAGGAATCAAGAGAATGCTCTTC
SRR765714.196951609.2- CTTTTCAGGAATCAAGAGAATGCTCTTC
SRR765714.71369973.2- CTTTTCAGGAATCAAGAGAATGCTCTTC
SRR765710.59709380.1- CTTTTCAGGAATCAAGAGAATGCTCTTCC
SRR765714.144000723.2- CTTTTCAGGAATCAAGAGAATGCTCTTCC
SRR765709.84981878.1- CTTTTCAGGAATCAAGAGAATGCTCTTCCTCT
SRR765712.19285418.2- CTTTTCAGGAATCAAGAGAATGCTCTTCCTCT
SRR765715.8423602.1+ CTTTTCAGGAATCAAGAGAATGCTCTTCCTCT
SRR765710.48969246.1+ CTTTTCAGGAATCAAGAGAATGCTCTTCCTCTT
SRR765711.116926262.2+ CTTTTCAGGAATCAAGAGAATGCTCTTCCTCTT
SRR765711.136262268.1+ CTTTTCAGGAATCAAGAGAATGCTCTTCCTCTT
SRR765709.11048444.2- CTTTTCAGGAATCAAGAGAATGCTCTTCCTCTTG
SRR765710.30559922.2- CTTTTCAGGAATCAAGAGAATGCTCTTCCTCTTG
SRR765711.12467307.2- CTTTTCAGGAATCAAGAGAATGCTCTTCCTCTTG
SRR765712.170972669.2+ CTTTTCAGGAATCAAGAGAATGCTCTTCCTCTTG
SRR765715.39302854.1- CTTTTCAGGAATCAAGAGAATGCTCTTCCTCTTG
SRR765710.20061203.1+ CTTTTCAGGAATCAAGAGAATGCTCTTCCTCTTGT
SRR765711.75336142.1+ CTTTTCAGGAATCAAGAGAATGCTCTTCCTCTTGT
SRR765712.209293117.1- CTTTTCAGGAATCAAGAGAATGCTCTTCCTCTTGT
SRR765711.57382922.1+ CTTTTCAGGAATCAAGAGAATGCTCTTCCTCTTGTT
SRR765712.108514153.2+ CTTTTCAGGAATCAAGAGAATGCTCTTCCTCTTGTTC
SRR765712.44471210.2+ CTTTTCAGGAATCAAGAGAATGCTCTTCCTCTTGTTCC
SRR765711.65007596.1+ CTTTTCAGGAATCAAGAGAATGCTCTTCCTCTTGTTCCT
SRR765714.118843472.1- CTTTTCAGGAATCAAGAGAATGCTCTTCCTCTTGTTCCT
SRR765710.78927757.2- CTTTTCAGGAATCAAGAGAATGCTCTTCCTCTTGTTCCTC
SRR765715.16533716.2+ CTTTTCAGGAATCAAGAGAGTGCTCTTCCTCTTGTTCCTCC
SRR765710.52880643.1- CTTTTCAGGAATCAAGAGAATGCTCTTCCTCTTGTTCCTCCC
SRR765711.16163481.2- CTTTTCAGGAATCAAGAGAATGCTCTTCCTCTTGTTCCTCCCA
SRR765711.76231448.1+ CTTTTCAGGAATCAAGAGAATGCTCTTCCTCTTGTTCCTCCCA
SRR765711.106750007.1- CTTTTCAGGAATCAAGAGAATGCTCTTCCTCTTGTTCCTCCCAT
SRR765709.19446554.1- CTTTTCAGGAATCAAGAGAATGCTCTTCCTCTTGTTCCTCCCATG
SRR765711.78666991.2- CTTTTCAGGAATCAAGAGAATGCTCTTCCTCTTGTTCCTCCCATG
SRR765710.58041512.1- CTTTTCAGGAATCAAGAGAATGCTCTTCCTCTTGTTCCTCCCATGC
SRR765711.115598291.2- CTTTTCAGGAATCAAGAGAATGCTCTTCCTCTTGTTCCTCCCATGCT
SRR765710.50972813.1- CTTTTCAGGAATCAAGAGAATGCTCTTCCTCTTGTTCCTCCCATGCTC
SRR765712.166853237.1+ CTTTTCAGGAATCAAGAGAATGCTCTTCCTCTTGTTCCTCCCATGCTC
SRR765712.7129698.2- CTTTTCAGGAATCAAGAGAATGCTCTTCCTCTTGTTCCTCCCATGCTC
SRR765711.24558845.1- CTTTTCAGGAATCAAGAGAATGCTCTTCCTCTTGTTCCTCCCATGCTCTT
SRR765711.79934409.2- CTTTTCAGGAATCAAGAGAATGCTCTTCCTCCTGTTCCTCCCATGCTCTT
SRR765712.73428311.2+ CTTTTCAGGAATCAAGAGAATGCTCTTCCTCTTGTTCCTCCCATGCTCTT
SRR765709.56879908.2+ CTTTTCAGGAATCAAGAGAATGCTCTTCCTCTTGTTCCTCCCATGCTCTTG
SRR765711.79688649.1+ CTTTTCAGGAATCAAGAGAATGCTCTTCCTCTTGTTCCTCCCATGCTCTTGTT
SRR765712.70145514.2- CTTTTCAGGAATCAAGAGAATGCTCTTCCTCTTGTTCCTCCCATGCTCTTGTTC
SRR765714.105503876.2- CTTTTCAGGAATCAAGAGAATGCTCTTCCTCTTGTTCCTCCCATGCTCTTGTTC
SRR765714.115172876.2- CTTTTCAGGAATCAAGAGAATGCTCTTCCTCTTGTTCCTCCCATGCTCTTGTTC
SRR765712.106710182.1+ CTTTTCAGGAATCAAGAGAATGCTCTTCCTCTTGTTCCTCCCATGCTCTTGTTCT
SRR765712.82312151.1- CTTTTCAGGAATCAAGAGAATGCTCTTCCTCTTGTTCCTCCCATGCTCTTGTTCT
SRR765712.141280360.1- CTTTTCAGGAATCAAGAGAATGCTCTTCCTCTTGTTCCTCCCATGCTCTTGTTCTG
SRR765710.6874784.2+ CTTTTCAGGAATCAAGAGAATGCTCTTCCTCTTGTTCCTCCCATGCTCTTGTTCTGGCC
SRR747825.15514581.2+ CTTTTCAGGAATCAAGAGAATGCTCTTCCTCTTGTTCCTCCCATGCTCTTGTTCTGGCCA
SRR747825.2399014.1+ CTTTTCAGGAATCAAGAGAATGCTCTTCCTCTTGTTCCTCCCATGCTCTTGTTCTGGCCA
SRR747825.25077350.1+ CTTTTCAGGAATCAAGAGAATGCTCTTCCTCTTGTTCCTCCCATGCTCTTGTTCTGGCCA
SRR747825.32125711.2+ CTTTTCAGGAATCAAGAGAATGCTCTTCCTCTTGTTCCTCGCATGCTCTTGTTCTGGCCA
SRR747825.34186382.2+ CTTTTCGGGAATCAAGAGAATGCTCTTCCTCTTGTTCCTCCCATGCTCTTGTTCTGGCCA
SRR747825.36676712.1+ CTTTTCAGGAATCAAGAGAATGCTCTTCCTGTTGTTCCTCCCATGCTCTTGTTCTGGCCA
SRR747825.36796222.1+ CTTTTCAGGAATCAAGAGAATGCTCTTCCTCTTGTTCCTCCCATGCTCTTGTTCTGCGCA
SRR747825.37802110.2+ CTTTTCAGGAATCAAGAGAATGCTCTTCCTCTTGTTCCTCCCATGCTCTTGTTCTGGCCA
SRR747825.41358891.2+ CTTTTCAGGAATCAAGAGAATGCTCTTCCTCTTGTTCCTCCCATGCTCTTGTTCTGGCCA
SRR747825.42101809.1+ CTTTTCAGGAATCAAGAGAATGCTCTTCCTCTTGTTCCTCCCATGCTCTTGTTCTGGCCA
SRR747825.42675926.1+ CTTTTCAGGAATCAAGAGAATGCTCTTCCTCTTGTTCCTCCCATGCTCTTGTTCTGGCCA
SRR747825.7479104.2+ CTTTTCAGGAATCAAGAGAATGCTCTTCCTCTTGTTCCTCCCATGCTCTTGTTCTGGCCA
SRR747825.9216947.2+ CTTTTCAGGAATCAAGAGAATGCTCTTCCTCTTGTTCCTCCCATGCTCTTGTTCTGGCCA

```
SRR765709.16812281.2-    CTTTTCAGGAATCAAGAGAATGCTCTTCCTCTTGTTCCTCCCATGCTCTTGTTCTGGCCA
SRR765709.27281621.2-    CTTTTCAGGAATCAAGAGAATGCTCTTCCTCTTGTTCCTCCCATGCTCTTGTTCTGGCCA
SRR765709.5623476.2+     CTTTTCAGGAATCAAGAGAATGCTCTTCCTCTTGTTCCTCCCATGCTCTTGTTCTGGCCA
SRR765709.80799489.2+    CTTTTCAGGAATCAAGAGAATGCTCTTCCTCTTGTTCCTCCCATGCTCTTGTTCTGGCCA
SRR765709.89392740.2-    CTTTTCAGGAATCAAGAGAATGCTCTTCCTCTTGTTCCTCCCATGCTCGTGTTCTGGCCA
SRR765710.14520134.1-    CTTTTCAGGAATCAAGAGAATGCTCTTCCTCTTGTTCCTCCCATGCTCTTGTTCTGGCCA
SRR765710.48848881.2+    CTTTTCAGGAATCAAGAGAATGCTCTTCCTCTTGTTCCTCCCATGCTCTTGTTCTGGCCA
SRR765710.48969246.2-    CTTTTCAGGAATCAAGAGAATGCTCTTCCTCTTGTTCCTCCCATGCTCTTGTTCTGGCCA
SRR765710.52432273.2-    CTTTTCAGAAATCAAGAGAATGCTCTTCCTCTTGTTCCTCCCATGCTCTTGTTCTGGCCA
SRR765710.52899931.1-    CTTTTCAGGAATCAAGAGAATGCTCTTCCTCTTGTTCCTCCCATGCTCTTGTTCTGGCCA
SRR765710.53338134.1+    CTTTTCAGGAATCCAGAGAATGCTCTTCCTCTTGTTCCTCCCATGCTCTTGTTCTGGCCA
SRR765710.62324211.1-    CTTTTCAGGAATCAAGAGAATGCTCTTCCTCTTGTTCCTCCCATGCTCTTGTTCTGGCCA
SRR765710.6314.1-        CTTTTCAGGAATCAAGAGAATGCTCTTCCTCTTGTTCCTCCCATGCTCTTGTTCTGGCCA
SRR765710.72900439.1-    CTTTTCAGGAATCAAGAGAATGCTCTTCCTCTTGTTCCTCCCATGCTCTTGTTCTGGCCA
SRR765710.77934178.2+    CTTTTCAGGAATCAAGAGAATGCTCTTCCTCTTGTTCCTCCCATGCTCTTGTTCTGGCCA
SRR765710.83849557.1-    CTTTTCAGGAATCAAGAGAATGTTCTTCCTCTTGTTCCTCCCATGCTCTTGTTCTGGCCA
SRR765711.120800792.2+   CTTTTCAGGAATCAAGAGAATGCTCTTCCTCTTGTTCCTCCCATGCTCTTGTTCTGGCCA
SRR765711.127108613.1-   CTTTTCAGGAATCAAGAGAATGCTCTTCCTCTTGTTCCTCCCATGCTCTTGTTCTGGCCA
SRR765711.13528758.1+    CTTTTCAGGAATCAAGAGAATGCTCTTCCTCTTGTTCCTCCCATGCTCTTGTTCTGGCCA
SRR765711.136914716.2-   CTTTTCAGGAATCAAGAGAATGCTCTTCCTCTTGTTCCTCCCATGCTCTTGTTCTGGCCA
SRR765711.141606739.2-   CTTTTCAGGAATCAAGAGAATGCTCTTCCTCTTGTTCCTCCCATGCTCTTGTTCTGGCCA
SRR765711.15445266.2+    CTTTTCAGGAATCAAGAGAATGCTCTTCCTCTTGTTCCTCCCATGCTCTTGTTCTGGCCA
SRR765711.27058616.2-    CTTTTCAGGAATCAAGAGAATGCTCTTCCTCTTGTTCCTCCCATGCTCTTGTTCTGGCCA
SRR765711.39985328.2-    CTTTTCAGGAATCAAGAGAATGCTCTTCCTCTTGTTCCTCCCATGCTCTTGTTCTGGCCA
SRR765711.41431931.1+    CTTTTCAGGAATCACGAGAATGCTCTTCCTCTTGTTCCTCCCATGCTCTTGTTCTGGCCA
SRR765711.42060791.1+    CTTTTCAGGAATCAAGAGAATGCTCTTCCTCTTGTTCCTCCCATGCTCTTGTTCTGGCCA
SRR765711.43336047.2-    CTTTTCAGGAATCAAGAGAATGCTCTTCCTCTTGTTCCTCCCATGCTCTTGTTCTGGCCA
SRR765711.50432206.2+    CTTTTCAGGAATCAAGAGAATGCTCTTCCTCTTGTTCCTCCCATGCTCTTGTTCTGGCCA
SRR765711.58856886.1-    CTTTTCAGGAATCAAGAGAATGCTCTTCCTCTTGTTCCTCCCATGCTCTTGTTCTGGCCA
SRR765711.70787159.1+    CTTTTCAGGAATCAAGAGAATGCTCTTCCTCTTGTTCCTCCCATGCTCTTGTTCTGGCCA
SRR765711.81304240.1+    CTTTTCAGGAATCAAGAGAATGCTCTTCCTCTTGTTCCTCCCATGCTCTTGTTCTGGCCA
SRR765711.93189275.2-    CTTTTCAGGAATCAAGAGAATGCTCTTCCTCTTGTTCCTCCCATGCTCTTGTTCTGGCCA
SRR765711.96129668.1-    CTTTTCAGGAATCAAGAGAATGCTCTTCCTCTTGTTCCTCCCATGCTCTTGTTCTGGCCA
SRR765711.98788597.2-    CTTTTCAGGAATCAAGAGAATGCTCTTCCTCTTGTTCCTCCCATGCTCTTGTTCTGGCCA
SRR765712.108514153.1-   CTTTTCAGGAATCAAGAGAATGCTCTTCCTCTTGTTCCTCCCATGCTCTTGTTCTGGCCA
SRR765712.201848235.1-   CTTTTCAGGAATCAAGAGAATGCTCTTCCTCTTGTTCCTCCCATGCTCTTGTTCTGGCCA
SRR765712.208044178.1+   CTTTTCAGGAATCAAGAGAATGCTCTTCCTCTTGTTCCTCCCATGCTCTTGTTCTGGCCA
SRR765712.22158653.2+    CTTTTCAGGAATCAAGAGAATGCTCTTCCTCTTGTTCCTCCCATGCTCTTGTTCTGGCCA
SRR765712.223930320.2-   CTTTTCAGGAATCAAGAGAATGCTCTTCCTCTTGTTCCTCCCATGCTCTTGTTCTGGCCA
SRR765712.225295283.2-   CTTTTCAGGAATCAAGAGAATGCTCTTCCTCTTGTTCCTCCCATGCTCTTGTTCTGGCCA
SRR765712.227153236.2-   CTTTTCAGGAATCAAGAGAATGCTCTTCCTCTTGTTCCTCCCATGCTCTTGTTCTGGCCA
SRR765712.29757431.1+    CTTTTCAGGAATCAAGAGAATGCTCTTCCTCTTGTTCCTCCCATGCTCTTGTTCTGGCCA
SRR765712.71088928.1+    CTTTTCAGGAATCAAGAGAATGCTCTTCCTCTTGTTCCTCCCATGCTCTTGTTCTGGCCA
SRR765712.73551137.1+    CTTTTCAGGAATCAAGAGAATGCTCTTCCTCTTGTTCCTCCCATGCTCTTGTTCTGGCCA
SRR765712.80351652.1+    CTTTTCAGGAATCAAGAGAATGCTCTTCCTCTTGTTCCTCCCATGCTCTTGTTCTGGCCA
SRR765712.9126008.1-     CTTTTCAGGAATCAAGAGAATGCTCTTCCTCTTGTTCCTCCCATGCTCTTGTTCTGGCCA
SRR765714.12218431.2+    CTTTTCAGGAATCAAGAGAATGCTCTTCCTCTTGTTCCTCCCATGCTCTTGTTCTGGCCA
SRR765714.123035075.2-   CTTTTCAGGAATCAAGAGAATGCTCTTCCTCTTGTTCCTCCCATGCTCTTGCTCTGGCCA
SRR765714.15028586.1-    CTTTTCAGGAATCAAGAGAATGCTCTTCCTCTTGTTCCTCCCATGCTCTTGTTCTGGCCA
SRR765714.162066143.2+   CTTTTCAGGAATCAAGAGAATGCTCTTCCTCTTGTTCCTCCCATGCTCTTGTTCTGGCCA
SRR765714.181565026.1+   CTTTTCAGGAATCAAGAGAATGCTCTTCCTCTTGTTCCTCCCATGCTCTTGTTCTGGCCA
SRR765714.45571282.1-    CTTTTCAGGAATCAAGAGAATGCTCTTCCTCTTGTTCCTCCCATGCTCTTGTTCTGGCCA
SRR765714.73397308.2-    CTTTTCAGGAATCAAGAGAATGCTCTTCCTCTTGTTCCTCCCATGCTCTTGTTCTGGCCA
SRR765714.76267865.2+    CTTTTCAGGAATCAAGAGAATGCTCTTCCTCTTGTTCCTCCCATGCTCTTGTTCTGGCCA
SRR765714.90251705.1-    CTTTTCAGGAAGCAAGAGAATGCTCTTCCTCTTGTTCCTCCCATGCTCTTGTTCTGGCCA
SRR765714.92358977.1+    CTTTTCAGGAATCAAGAGAATGCTCTTCCTCTTGTTCCTCCCATGCTCTTGTTCTGGCCA
SRR765715.19822467.1+    CTTTTCAGGAATCAAGAGAATGCTCTTCCTCTTGTTCCTCCCATGCTCTTGTTCTGGCCA
SRR765715.22238685.1-    CTTTTCAGGAATCAAGAGAATGCTCTTCCTCTTGTTCCTCCCATGCTCTTGTTCTGGCCA
SRR765715.22238685.2+    CTTTTCAGGAATCAAGAGAATGCTCTTCCTCTTGTTCCTCCCATGCTCTTGTTCTGGCCA
SRR765715.3175093.1-     CTTTTCAGGAATCAAGAGAATGCTCTTCCTCTTGTTCCTCCCATGCTCTTGTTCTGGCCA
SRR765715.3175093.2+     CTTTTCAGGAATCAAGAGAATGCTCTTCCTCTTGTTCCTCCCATGCTCTTGTTCTGGCCA
SRR765715.41596894.1-    CTTTTCAGGAATCAAGAGAATGCTCTTCCTCTTGTTCCTCCCATGCTCTTGTTCTGGCCA
SRR765715.4936771.1+     CTTTTCAGGAATCAAGAGAATGCTCTTCCTCTTGTTCCTCCCATGCTCTTGTTCTGGCCA
SRR765716.23347563.1+    CTTTTCAGGAATCAAGAGAATGCTCTTCCTCTTGTTCCTCCCATGCTCTTGTTCTGGCCA
SRR765716.250216.2-      CTTTTCAGGAATCAAGAGAATGCTCTTCCTCTTGTTCCTCCCATGCTCTTGTTCTGGCCA
SRR765716.25173572.2-    CTTTTCAGGAATCAAGAGAATGCTCTTCCTCTTGTTCCTCCCATGCTCTTGTTCTGGCCA
SRR765716.26119942.2-    CTTTTCAGGAATCAAGAGAATGCTCTTCCTCTTGTTCCCCCCATGCTCCTGTTCTGGCCA
SRR765716.28097872.2+    CTTTTCAGGAATCAAGAGAATTCTCTTCCTCTTGTTCCTCCCATGCTCTTATTCTGGCCA
SRR765711.57382922.2-     TTTTCAGGAATCAAGAGAATGCTCTTCCTCTTGTTCCTCCCATGCTCTTGTTCTGGCCA
SRR765711.9180360.2+         CAGGAATCAAGAGAATGCTCTTCCTCTTGTTCCTCCCATGCTCTTGTTCTGGCCA
SRR765712.44471210.1-        CAGGAATCAAGAGAATGCTCTTCCTCTTGTTCCTCCCATGCTCTTGTTCTGGCCA
SRR765711.125009762.1-        AGGAATCAAGAGAATGCTCTTCCTCTTGTTCCTCCCATGCTCTTGTTCTGGCCA
SRR765711.76231448.2-         AGGAATCAAGAGAATGCTCTTCCTCTTGTTCCTCCCATGCTCTTGTTCTGGCCA
SRR765712.57478692.2+         AGGAATCAAGAGAATGCTCTTCCTCTTGTTCCTCCCATGCTCTTGTTCTGGCCA
SRR765712.73886187.2+         AGGAATCAAGAGAATGCTCTTCCTCTTGTTCCTCCCATGCTCTTGTTCTGGCCA
SRR765712.15635258.2+          GGAATCAAGAGAATGCTCTTCCTCTTGTTCCTCCCATGCTCTTGTTCTGGCCA
SRR765712.178874795.2-         GGAATCAAGAGAATGCTCTTCCTCTTGTTCCTCCCATGCTCTTGTTCTGGCCA
SRR765712.165805728.2+           AATCAAGAGAATGCTCTTCCTCTTGTTCCTCCCATGCTCTTGTTCTGGCCA
SRR765710.11930567.1+             ATCAAGAGAATGCTCTTCCTCTTGTTCCTCCCATGCTCTTGTTCTGGCCA
SRR765710.46047202.2+              TCAAGAGAATGCTCTTCCTCTTGTTCCTCCCATGCTCTTGTTCTGGCCA
SRR765711.65007596.2-              TCAAGAGAATGCTCTTCCTCTTGTTCCTCCCATGCTCTTGTTCTGGCCA
SRR765712.58838374.2+              TCAAGAGAATGCTCTTCCTCTTGTTCCTCCCATGCTCTTGTTCTGGCCA
SRR765710.20061203.2-               CAAGAGAATGCTCTTCCTCTTGTTCCTCCCATGCTCTTGTTCTGGCCA
SRR765712.126177480.1+              CAAGAGAATGCTCTTCCTCTTGTTCCTCCCATGCTCTTGTTCTGGCCA
```

```
SRR765714.149627387.1+               CAAGAGAATGCTCTTCCTCTTGTTCCTCCCATGCTCTTGTTCTGGCCA
SRR765712.21952163.1+                 AAGAGAATGCTCTTCCTCTTGTTCCTCCCATGCTCTTGTTCTGGCCA
SRR765711.58178683.1-                  AGAGAATGCTCTTCCTCTTGTTCCTCCCATGCTCTTGTTCTGGCCA
SRR765714.11920695.2+                  AGAGAATGCTCTTCCTCTTGTTCCTCCCATGCTCTTGTTCTGCCCA
SRR765711.116926262.1-                  GAGAATGCTCTTCCTCTTGTTCCTCCCATGCTCTTGTTCTGGCCA
SRR765712.152915721.1+                  GAGAATGCTCTTCCTCTTGTTCCTCCCATGCTCTTGTTCTGGCCA
SRR765711.75336142.2-                    AGAATGCTCTTCCTCTTGTTCCTCCCATGCTCTTGTTCTGGCCA
SRR765712.102108300.2-                   AGAATGCTCTTCCTCTTGTTCCTCCCATGCTCTTGTTCTGGCCA
SRR765712.117148243.2+                   AGAATGCTCTTCCTCTTGTTCCTCCCATGCTCTTGTTCTGGCCA
SRR765711.117156605.2+                    GAATGCTCTTCCTCTTGTTCCTCCCATGCTCTTGTTCTGGCCA
SRR765713.24757498.1+                     GAATGCTCTTCCTCTTGTTCCTCCCATGCTCTTGTTCTGGCCA
SRR765709.13162642.2+                      AATGCTCTTCCTCTTGTTCCTCCCATGCTCTTGTTCTGGCCA
SRR765712.170972669.1-                     AATGCTCTTCCTCTTGTTCCTCCCATGCTCTTGTTCTGGCCA
SRR765712.232059580.1+                     AATGCTCTTCCTCTTGTTCCTCCCATGCTCTTGTTCTGGCCA
SRR765711.115303767.2-                      ATGCTCTTCCTCTTGTTCCTCCCATGCTCTTGTTCTGGCCA
SRR765712.21124108.2+                       ATGCTCTTCCTCTTGTTCCTCCCATGCTCTTGTTCTGGCCA
SRR765712.228231813.2+                      ATGCTCTTCCTCTTGTTCCTCCCATGCTCTTGTTCTGGCCA
SRR765711.136262268.2-                        GCTCTTCCTCTTGTTCCTCCCATGCTCTTGTTCTGGCCA
SRR765710.1716354.2+                           CTCTTCCTCTTGTTCCTCCCATGCTCTTGTTCTGGCCA
SRR765714.39979308.2-                          CTCTTCCTCTTGTTCCTCCCATGCTCTTGTTCTGGCCA
SRR765715.15378963.1+                           TCTTCCTCTTGTTCCTCCCATGCTCTTGTTCTGGCCA
SRR765711.75189809.2-                            CTTCCTCTTGTTCCTCCCATGCTCTTGTTCTGGCCA
SRR765712.106710182.2-                             TCCTCTTGTTCCTCCCATGCTCTTGTTCTGGCCA
SRR765712.11807389.1+                                CTCTTGTTCCTCCCATGCTCTTGTTCTGGCCA
SRR765715.22812006.1-                                CTCTTGTTCCTCCCATGCTCTTGTTCTGGCCA
SRR765714.149230942.1+                                TCTTGTTCCTCCCATGCTCTTGTTCTGGCCA
SRR765710.6874784.1-                                   CTTGTTCCTCCCATGCTCTTGTTCTGGCCA
SRR765711.39537275.1-                                  CTTGTTCCTCCCATGCTCTTGTTCTGGCCA
SRR765711.90300589.1-                                    TGTTCCTCCCATGCTCTTGTTCTGGCCA
SRR765712.166853237.2-                                   TGTTCCTCCCATGCTCTTGTTCTGGCCA
SRR765711.15546394.2+                                     GTTCCTCCCATGCTCTTGTTCTGGCCA
SRR765711.92360717.1+                                     GTTCCTCCCATGCTCTTGTTCTGGCCA
SRR765715.30571678.1+                                     GTTCCTCCCATGCTCTTGTTCTGGCCA
SRR765715.36409904.1+                                     GTTCCTCCCATGCTCTTGTTCTGGCCA
SRR765710.77934178.1-                                       TCCTCCCATGCTCTTGTTCTGGCCA
SRR765712.179891936.1+                                         TCCCATGCTCTTGTTCTGGCCA
SRR765711.42060791.2-                                           CCCATGCTCTTGTTCTGGCCA
SRR765711.70787159.2-                                           CCCATGCTCTTGTTCTGGCCA
SRR765712.203325839.1+                                              TGCTCTTGTTCTGGCCA
SRR765710.48848881.1-                                                 CTCTTGTTCTGGCCA
SRR765711.83011887.1+                                                  TCTTGTACTGGCCA
SRR765712.46974017.1+                                                  TCTTGTTCTGGCCA
SRR765709.26128387.2+                                                   CTTGTTCTGGCCA
SRR765710.29061231.2+                                                   CTTGTTCTGGCCA
SRR765716.24592103.2+                                                    TTGTTCTGGCCA
SRR765716.287615.2+                                                      TTGTTCTGGCCA
SRR765716.12328120.2+                                                     TGTTCTGGCCA
SRR765716.14688142.2+                                                     TGTTCTGGCCA
SRR765716.19440677.2+                                                     TGTTCTGGCCA
SRR765716.25986022.2+                                                     TGTTCTGGCCA
SRR765711.31552385.1+                                                      GTTCTGGCCA
SRR765711.120800792.1-                                                      TTCTGGCCA
SRR765711.81304240.2-                                                        TCTGGCCA
SRR765713.15891820.1-                                                        TCTGGCCA
SRR765715.46017009.2+                                                         CTGGCCA
SRR765710.53338134.2-                                                           GGCCA
SRR765712.73428311.1-                                                           GGCCA
SRR765711.39834426.2+                                                            GCCA
SRR765712.29757431.2-                                                              CA
SRR765712.76576490.1+                                                               A

consensus                CTTTTCAGGAATCAAGAGAATGCTCTTCCTCTTGTTCCTCCCATGCTCTTGTTCTGGCCA

                             .    :    .    :    .    :    .    :    .    :    .    :
SRR765711.70787159.1+    G
SRR747825.25077350.1+    GC
SRR765714.90251705.1-    GCTT
SRR765715.41596894.1-    GCTT
SRR765711.81304240.1+    GCTTG
SRR765712.80351652.1+    GCTTG
SRR747825.15514581.2+    GCTTGC
SRR747825.2399014.1+     GCTTGC
SRR747825.32125711.2+    GCTTGC
SRR747825.34186382.2+    GCTTGC
SRR747825.36676712.1+    GCTTGC
SRR747825.36796222.1+    GCTTGC
SRR747825.37802110.2+    GCTTGC
SRR747825.41358891.2+    GCTTGC
SRR747825.42101809.1+    GCTTGC
SRR747825.42675926.1+    GCTTGC
SRR747825.7479104.2+     GCTTGC
SRR747825.9216947.2+     GCTTGC
SRR765710.77934178.2+    GCTTGC
SRR765711.42060791.1+    GCTTGC
```

SRR765709.89392740.2-	GCTTGCATC
SRR765711.58856886.1-	GCTTGCATC
SRR765714.15028586.1-	GCTTGCATC
SRR765712.223930320.2-	GCTTGCATCT
SRR765716.250216.2-	GCTTGCATCT
SRR765716.25173572.2-	GCTTGCATCT
SRR765716.26119942.2-	GCTTGCATCT
SRR765710.48848881.2+	GCTTGCATCTCT
SRR765710.83849557.1-	GCTTGCATCTCTGC
SRR765710.62324211.1-	GCTTGCATCTCTGCT
SRR765709.27281621.2-	GCTTGCATCTCTGCTG
SRR765711.127108613.1-	GCTTGCATCTCTGCTG
SRR765714.92358977.1+	GCTTGCATCTCTGCTGC
SRR765716.23347563.1+	GCTTGCATCTCTGCTGC
SRR765710.52899931.1-	GCTTGCATCTCTGCTGCT
SRR765710.53338134.1+	GCTTGCATCTCTGCTGCT
SRR765711.41431931.1+	GCTTGCATCTCTGCTGCT
SRR765712.225295283.2-	GCTTGCATCTCTGCTGCT
SRR765712.73551137.1+	GCTTGCATCTCTGCTGCTG
SRR765712.201848235.1-	GCTTGCATCTCTGCTGCTGAG
SRR765710.72900439.1-	GCTTGCATCTCTGCTGCTGAGT
SRR765711.120800792.2+	GCTTGCATCTCTGCTGCTGAGT
SRR765711.93189275.2-	GCTTGCATCTCTGCTGCTGAGT
SRR765712.108514153.1-	GCTTGCATCTCTGCTGCTGAGTT
SRR765714.45571282.1-	GCTTGCATCTCTGCTGCTGAGTT
SRR765714.123035075.2-	GCTTGCATCTCTGCTGCTGAGTTT
SRR765714.162066143.2+	GCTTGCATCTCTGCTGCTGAGTTT
SRR765711.13528758.1+	GCTTGCATCTCTGCTGCTGAGTTTC
SRR765711.43336047.2-	GCTTGCATCTCTGCTGCTGAGTTTC
SRR765711.50432206.2+	GCTTGCATCTCTGCTGCTGAGTTTC
SRR765714.12218431.2+	GCTTGCATCTCTGCTGCTGAGTTTCC
SRR765709.5623476.2+	GCTTGCATCTCTGCTGCTGAGTTTCCC
SRR765711.98788597.2-	GCTTGCATCTCTGCTGCTGAGTTTCCC
SRR765712.9126008.1-	GCTTGCATCTCTGCTGCTGAGTTTCCC
SRR765710.6314.1-	GCTTGCATCTCTGCTGCTGAGTTTCCCC
SRR765710.14520134.1-	GCTTGCATCTCTGCTGCTGAGTTTCCCCA
SRR765715.22238685.1-	GCTTGCATCTCTGCTGCTGAGTTTCCCCA
SRR765715.22238685.2+	GCTTGCATCTCTGCCGCTGAGTTTCCCCA
SRR765715.3175093.1-	GCTTGCATCTCTGCTGCTGAGTTTCCCCA
SRR765715.3175093.2+	GCTTGCATCTCTGCTGCTGAGTTTCCCCA
SRR765709.16812281.2-	GCTTGCATCTCTGCTGCTGAGTTTCCCCAC
SRR765711.96129668.1-	GCTTGCATCTCTGCTGCTGAGTTTCCCCAC
SRR765716.28097872.2+	GCTTGCATCTCTGCTGCTGAGTTTCCCCAC
SRR765714.181565026.1+	GCTTGCATCTCTGCTGCTGAGTTTCCCCACC
SRR765710.48969246.2-	GCTTGCATCTCTGCTGCTGAGTTTCCCCACCT
SRR765711.15445266.2+	GCTTGCATCTCTGCTGCTGAGTTTCCCCACCTT
SRR765712.227153236.2-	GCTTGCATCTCTGCTGCTGAGTTTCCCCACCTT
SRR765711.136914716.2-	GCTTGCATCTCTGCTGCTGAGTTTCCCCACCTTC
SRR765714.73397308.2-	GCTTGCATCTCTGCTGCTGAGTTTCCCCACCTTCC
SRR765714.76267865.2+	GCTTGCATCTCTGCTGCTGAGTTTCCCCACCTTCC
SRR765709.80799489.2+	GCTTGCATCTCTGCTGCTGAGTTTCCCCACCTTCCT
SRR765711.27058616.2-	GCTTGCATCTCTGCTGCTGAGTTTCCCCACCTTCCTG
SRR765712.208044178.1+	GCTTGCATCTCTGCTGCTGAGTTTCCCCACCTTCCTG
SRR765710.52432273.2-	GCTTGCATCTCTGCTGCTGAGTTTCCCCACCTTCCTGG
SRR765711.39985328.2-	GCTTGCATCTCTGCTGCTGAGTTTCCCCACCTTCCTGG
SRR765712.22158653.2+	GCTTGCATCTCTGCTGCTGAGTTTCCCCACCTTCCTGG
SRR765711.141606739.2-	GCTTGCATCTCTGCTGCTGAGTTTCCCCACCTTCCTGGA
SRR765712.29757431.1+	GCTTGCATCTCTGCTGCTGAGTTTCCCCACCTTCCTGGA
SRR765712.71088928.1+	GCTTGCATCTCTGCTGCTGAGTTTCCCCACCTTCCTGGAT
SRR765715.19822467.1+	GCTTGCATCTGTGCTGCTGAGTTTCCCCACCTTCCTGGATC
SRR765715.4936771.1+	GCTTGCATCTCTGCTGCTGAGTTTCCCCACCTTCCTGGATC
SRR765716.24592103.2+	GCTTGCATCTCTGCTGCTGAGTTTCCCCACCTTCCTGGATC
SRR765716.287615.2+	GCTTGCATCTCTGCTGCTGAGTTTCCCCACCTTCCTGGATC
SRR765711.57382922.2-	GCTTGCATCTCTGCTGCTGAGTTTCCCCACCTTCCTGGATCC
SRR765715.30571678.1+	GCTTGCATCTCTGCTGCTGAGTTTCCCCACCTTCCTGGATCCT
SRR765715.36409904.1+	GCTTGCATCTCTGCTGCTGAGTTTCCCCACCTTCCTGGATCCT
SRR765711.9180360.2+	GCTTGCATCTCTGCTGCTGAGTTTCCCCACCTTCCTGGATCCTTCC
SRR765712.44471210.1-	GCTTGCATCTCTGCTGCTGAGTTTCCCCACCTTCCTGGATCCTTCC
SRR765711.125009762.1-	GCTTGCATCTCTGCTGCTGAGTTTCCCCACCTTCCTGGATCCTTCCA
SRR765711.76231448.2-	GCTTGCATCTCTGCTGCTGAGTTTCCCCACCTTCCTGGATCCTTCCA
SRR765712.57478692.2+	GCTTGCATCTCTGCTGCTGAGTTTCCCCACCTTCCTGGATCCTTCCA
SRR765712.73886187.2+	GCTTGCATCTCTGCTGCTGAGTTTCCCCACCTTCCTGGATCCTTCCA
SRR765712.15635258.2+	GCTTGCATCTCTGCTGCTGAGTTTCCCCACCTTCCTGGATCCTTCCAG
SRR765712.178874795.2-	GCTTGCATCTCTGCTGCTGAGTTTCCCCACCTTCCTGGATCCTTCCAG
SRR765712.165805728.2+	GCTTGCATCTCTGCTGCTGAGTTTCCCCACCTTCCTGGATCCTTCCAGCA
SRR765710.11930567.1+	GCTTGCATCTCTGCTGCTGAGTTTCCCCACCTTCCTGGATCCTTCCAGCAT
SRR765710.46047202.2+	GCTTGCATCTCTGCTGCTGAGTTTCCCCACCTTCCTGGATCCTTCCAGCATG
SRR765711.65007596.2-	GCTTGCATCTCTGCTGCTGAGTTTCCCCACCTTCCTGGATCCTTCCAGCATG
SRR765712.58838374.2+	GCTTGCATCTCTGCTGCTGAGTTTCCCCACCTTCCTGGATCCTTCCAGCATG
SRR765714.149627387.1+	GCTTGCATCTCTGCTGCTGAGTTTCCCCACCTTCCTGGATCCTTCCAGCATG
SRR765710.20061203.2-	GCTTGCATCTCTGCTGCTGAGTTTCCCCACCTTCCTGGATCCTTCCAGCATGT
SRR765712.126177480.1+	GCTTGCATCTCTGCTGCTGAGTTTCCCCACCTTCCTGGATCCTTCCAGCATGT
SRR765712.21952163.1+	GCTTGCATCTCTGCTGCTGAGTTTCCCCACCTTCCTGGATCCTTCCAGCATGTT
SRR765714.11920695.2+	GCTTGCATCTCTGCTGCTGAGTTTCCCCACCTTCCTGGATCCTTCCAGCATGTT

SRR765711.58178683.1- GCTTGCATCTCTGCTGCTGAGTTTCCCCACCTTCCTGGATCCTTCCAGCATGTTC
SRR765711.116926262.1- GCTTGCATCTCTGCTGCTGAGTTTCCCCACCTTCCTGGATCCTTCCAGCATGTTCT
SRR765712.152915721.1+ GCTTGCATCTCTGCTGCTGAGTTTCCCCACCTTCCTGGATCCTTCCAGCATGTTCT
SRR765711.75336142.2- GCTTGCATCTCTGCTGCTGAGTTTCCCCACCTTCCTGGATCCTTCCAGCTTGTTCTA
SRR765712.102108300.2- GCTTGCATCTCTGCTGCTGAGTTTCCCCACCTTCCTGGATCCTTCCAGCATGTTCTA
SRR765712.117148243.2+ GCTTGCATCTCTGCTGCTGAGTTTCCCCACCTTCCTGGATCCTTCCAGCATGTTCTA
SRR765713.24757498.1+ GCTTGCATCTCTGCTGCTGAGTTTCCCCACCTTCCTGGATCCTTCCAGCATGTTCTA
SRR765711.117156605.2+ GCTTGCATCTCTGCTGCTGAGTTTCCCCACCTTCCTGGATCCTTCCAGCATGTTCTAC
SRR765709.13162642.2+ GCTTGCATCTCTGCTGCTGAGTTTCCCCACCTTCCTGGATCCTTCCAGCATGTTCTACC
SRR765712.170972669.1- GCTTGCATCTCTGCTGCTGAGTTTCCCCACCTTACTGGATCCTTCCAGCATGTTCTACC
SRR765712.232059580.1+ GCTTGCATCTCTGCTGCTGAGTTTCCCCACCTTCCTGGATCCTTCCAGCATGTTCTACC
SRR765709.26128387.2+ GCTTGCATCTCTGCTGCTGAGTTTCCCCACCTTCCTGGATCCTTCCAGCATGTTCTACCT
SRR765710.1716354.2+ GCTTGCATCTCTGCTGCTGAGTTTCCCCACCTTCCTGGATCCTTCCAGCATGTTCTACCT
SRR765710.29061231.2+ GCTTGCATCTCTGCTGCTGAGTTTCCCCACCTTCCTGGATCCTTCCAGCATGTTCTACCT
SRR765710.48848881.1- GCTTGCATCTCTGCTGCTGAGTTTCCCCACCTTCCTGGATCCTTCCAGCATGTTCTACCT
SRR765710.53338134.2- GCTTGCATCTCTGCTGCTGAGTTTCCCCACCTTCCTGGATCCTTCCAGCATGTTCTACCT
SRR765710.6874784.1- GCTTGCATCTCTGCTGCTGAGTTTCCCCACCTTCCTGGATCCTTCCAGCATGTTCTACCT
SRR765710.77934178.1- GCTTGCATCTCTGCTGCTGAGTTTCCCCACCTTCCTGGATCCTTCCAGCATGTTCTACCT
SRR765711.115303767.2- GCTTGCATCTCTGCTGCTGAGTTTCCCCACCTTCCTGGATCCTTCCAGCATGTTCTACCT
SRR765711.120800792.1- GCTTGCATCTCTGCTGCTGAGTTTCCCCACCTTCCTGGATCCTTCCAGCATGTTCTACCT
SRR765711.136262268.2- GCTTGCATCTCTGCTGCTGAGTTTCCCCACCTTCCTGGATCCTTCCAGCATGTTCTACCT
SRR765711.15546394.2+ GCTTGCATCTCTGCTGCTGAGTTTCCCCACCTTCCTGGATCCTTCCAGCATGTTCTACCT
SRR765711.31552385.1+ GCTTGCATCTCTGCTGCTGAGTTTCCCCACCTTCCTGGATCCTTCCAGCATGTTCTACCT
SRR765711.39537275.1- GCTTGCATCTCTGCTGCTGAGTTTCCCCACCTTCCTGGATCCTTCCAGCATGTTCTACCT
SRR765711.39834426.2+ GCTTGCATCTCTGCTGCTGAGTTTCCCCACCTTCCTGGATCCTTCCAGCATGTTCTACCT
SRR765711.42060791.2- GCTTGCATCTCTGCTGCTGAGTTTCCCCACCTTCCTGGATCCTTCCAGCATGTTCTACCT
SRR765711.70787159.2- GCTTGCATCTCTGCTGCTGAGTTTCCCCACCTTCCTGGATCCTTCCAGCATGTTCTACCT
SRR765711.75189809.2- GCTTGCATCTCTGCTGCTGAGTTTCCCCACCTTCCTGGATCCTTCCAGCATGTTCTACCT
SRR765711.79688649.2- GCTTGCATCTCTGCTGCTGAGTTTCCCCACCTTCCTGGATCCTTCCAGCATGTTCTACCT
SRR765711.81304240.2- GCTTGCATCTCTGCTGCTGAGTTTCCCCACCTTCCTGGATCCTTCCAGCATGTTCTACCT
SRR765711.83011887.1+ GCTTGCATCTCTGCTGCTGAGTTTCCCCACCTTCCTGGATCCTTCCAGCATGTTCTACCT
SRR765711.90300589.1- GCTTGCATCTCTGCTGCTGAGTTTCCCCACCTTCCTGGATCCTTCCAGCATGTTCTACCT
SRR765711.92360717.1+ GCTGGCATCTCTGCTGCTGCGTTTCCCTACCTTCCTGGATCCTTACAGCATGTTCTACCT
SRR765712.106710182.2- GCTTGCATCTCTGCTGCTGAGTTTCCCCACCTTCCTGGATCCTTCCAGCATGTTCTACCT
SRR765712.11807389.1+ GCTTGCATCTCTGCTGCTGAGTTTCCCCACCTTCCTGGATCCTTCCAGCATGTTCTACCT
SRR765712.166853237.2- GCTTGCATCTCTGCTGCTGAGTTTCCCCACCTTCCTGGATCCTTCCAGCATGTTCTACCT
SRR765712.179891936.1+ GCTTGCATCTCTGCTGCTGAGTTTCCCCACCTTCCTGGATCCTTCCAGCATGTTCTACCT
SRR765712.203325839.1+ GCTTGCATCTCTGCTGCTGAGTTTCCCCACCTTCCTGGATCCTTCCAGCATGTTCTACCT
SRR765712.21124108.2+ GCTTGCATCTCTGCTGCTGAGTTTCCCCACCTTCCTGGATCCTTCCAGCATGTTCTACCT
SRR765712.228231813.2+ GCTTGCATCTCTGCTGCTGAGTTTCCCCACCTTCCTGGATCCTTCCAGCATGTTCTACCT
SRR765712.29757431.2- GCTTGCATCTCTGCTGCTGAGTTTCCCCACCTTCCTGGATCCTTCCAGCATGTTCTACCT
SRR765712.46974017.1+ GCTTGCATCTCTGCTGCTGAGTTTCCCCACCTTCCTGGATCCTTCCAGCATGTTCTACCT
SRR765712.73428311.1- GCTTGCATCTCTGCTGCTGAGTTTCCCCACCTTCCTGGATCCTTCCAGCATGTTCTACCT
SRR765712.76576490.1+ GCTTGCATCTCTGCTGCTGAGTTTCCCCACCTTCCTGGATCCTTCCAGCATGTTCTACCT
SRR765713.15891820.1- GCTTGCATCTCTGCTGCTGAGTTTCCCCACCTTCCTGGATCCTTCCAGCATGTTCTACCT
SRR765714.149230942.1+ GCTTGCATCTCTGCTGCTGAGTTTCCCCACCTTCCTGGATCCTTCCAGCATGTTCTACCT
SRR765714.39979308.2- GCTTGCATCTCTGCTGCTGAGTTTCCCCACCTTCCTGGATCCTTCCAGCATGTTCTACCT
SRR765715.15378963.1+ GCTTGCATCTCTGCTGCTGAGTTTCCCCACCTTCCTGGATCCTTCCAGCATGTTCTACCT
SRR765715.22812006.1- GCTTGCATCTCTGCTGCTGAGTTTCCCCACCTTCCTGGATCCTTCCAGCATGTTCTACCT
SRR765715.46017009.2+ GCTTGCATCTCTGCTGCTGAGTTTCCCCACCTTCCTGGATCCTTCCAGCATGTTCTACCT
SRR765716.12328120.2+ GCTTGCATCTCTGCTGCTGAGTTTCCCCACCTTCCTGGATCCTTCCAGCATGTTCTACCT
SRR765716.14688142.2+ GCTTGCATCTCTGCTGCTGAGTTTCCCCACCTTCCTGGATCCTTCCAGCATGTTCTACCT
SRR765716.19440677.2+ GCTTGCATCTCTGCTGCTGAGTTTCCCCACCTTCCTGGATCCTTCCAGCATGTTCTACCT
SRR765716.25986022.2+ GCTTGCATCTCTGCTGCTGAGTTTCCCCACCTTCCTGGATCCTTCCAGCATGTTCTACCT
SRR765714.68056853.2- CTTGCATCTCTGCTGCTGAGTTTCCCCACCTTCCTGGATCCTTCCAGCATGTTCTACCT
SRR765714.88849388.2- CTTGCATCTCTGCTGCTGAGTTTCCCCACCTTCCTGGATCCTTCCAGCATGTTCTACCT
SRR765710.78316546.1+ ATCTCTGCTGCTGAGTTTCCCCACCTTCCTGGATCCTTCCAGCATGTTCTACCT
SRR765711.42145081.2+ ATCTCTGCTGCTGAGTTTCCCCACCTTCCTGGATCCTTCCAGCATGTTCTACCT
SRR765711.50432206.1- ATCTCTGCTGCTGAGTTTCCCCACCTTCCTGGATCCTTCCAGCATGTTCTACCT
SRR765713.12243610.2+ ATCTCTGCTGCTGAGTTTCCCCACCTTCCTGGATCCTTCCAGCATGTTCTACCT
SRR765714.142532987.2+ ATCTCTGCTGCTGAGTTTCCCCACCTTCCTGGATCCTTCCAGCATGTTCTACCT
SRR765714.54269538.2+ ATCTCTGCTGCTGAGTTTCCCCACCTTCCTGGATCCTTCCAGCATGTTCTACCT
SRR765714.55316615.2+ ATCTCTGCTGCTGAGTTTCCCCACCTTCCTGGATCCTTCCAGCATGTTCTACCT
SRR765712.25672489.1+ TCTCTGCTGCTGAGTTTCCCCACCTTCCTGGATCCTTCCAGCATGTTCTACCT
SRR765712.73551137.2- TCTCTGCTGCTGAGTTTCCCCACCTTCCTGGATCCTTCCAGCATGTTCTACCT
SRR765711.51569470.1+ CTCTGCTGCTGAGTTTCCCCACCTTCCTGGATCCTTCCAGCATGTTCTACCT
SRR765712.139323924.1+ CTCTGCTGCTGAGTTTCCCCACATTCCTGGATCCTTCCAGCATGTTCTACCT
SRR765714.88899945.1+ CTCTGCTGCTGAGTTTCCCCACCTTCCTGGATCCTTCCAGCATGTTCTACCT
SRR765713.15730043.1- TCTGCTGCTGAGTTTCCCCACCTTCCCGGATCCTTCCAGCATGTTCTACCT
SRR765712.27955158.1+ TGCTGCTGAGTTTCCCCACCTTCCTGGATCCTTCCAGCATGTTCTACCT
SRR765709.3017797.2+ TGCTGAGTTTCCCCACCTTCCTGGATCCTTCCAGCATGTTCTACCT
SRR765709.15051614.1- GCTGAGTTTCCCCACCTTCCTGGATCCTTCCAGCATGTTCTACCT
SRR765709.56879908.1- GCTGAGTTTCCCCACCTTCCTGGATCCTTCCAGCATGTTCTACCT
SRR765711.15445266.1- GCTGAGTTTCCCCACCTTCCTGGATCCTTCCAGCATGTTCTGCCT
SRR765714.45211402.1+ GCTGAGTTTCCCCACCTTCCTGGATCCTTCCAGCATGTTCTACCT
SRR765714.71098406.2- CTGAGTTTCCCCACCTTCCTGGATCCTTCCAGCATGTTCTACCT
SRR765714.126051084.2- TGAGTTTCCCCACCTTCCTGGATCCTTCCAGCATGTTCTACCT
SRR765710.11110602.1+ GAGTTTCCCCACCTTCCTGGATCCTTCCAGCATGTTCTACCT
SRR765715.11770606.2- AGTTTCCCCACCTTCCTGGATCCTTCCAGCATGTTCTACCT
SRR765715.41985107.2- AGTTTCCCCACCTTCCTGGATCCTTCCAGCATGTTCTACCT
SRR765710.66135762.2+ GTTTCCCCACCTTCCTGGATCCTTCCAGCATGTTCTACCT
SRR765714.103329039.2+ GTTTCCCCACCTTCCTGGATCCTTCCAGCATGTTCTACCT
SRR765710.11930567.2- TTCCCCACCTTCCTGGATCCTTCCAGCATGTTCTACCT
SRR765709.44632583.2+ CCCACCTTCCTGGATCCTTCCAGCATGTTCTACCT

```
SRR765712.117148243.1-                            CCCACCTTCCTGGATCCTTCCAGCATGTTCTACCT
SRR765715.5481277.1+                              CCCACCTTCCTGGATCCTTCCAGCATGTTCTACCT
SRR765712.208044178.2-                              CACCTTCCTGGATCCTTCCAGCATGTTCTACCT
SRR765712.165805728.1-                                CCTTCCTGGATCCTTCCAGCATGTTCTACCT
SRR765712.21124108.1-                                 CCTTCCTGGATCCTTCCAGCATGTTCTACCT
SRR765712.71088928.2-                                 CCTTCCTGGATCCTTCCAGCATGTTCTACCT
SRR765710.1716354.1-                                   CTTCCTGGATCCTTCCAGCATGTTCTACCT
SRR765715.16262666.1+                                  CTTCCTGGATCCTTCCAGCATGTTCTACCT
SRR765715.18589469.1+                                  CTTCCTGGATCCTTCCAGCATGTTCTACCT
SRR765712.15635258.1-                                   TTCCTGGATCCTTCCAGCATGTTCTACCT
SRR765711.117156605.1-                                   TCCTGGATCCTTCCAGCATGTTCTACCT
SRR765712.21952163.2-                                    TCCTGGATCCTTCCAGCATGTTCTACCT
SRR765709.17947042.2-                                     CCTGGATCCTTCCAGCATGTTCTACCT
SRR765712.173925922.1+                                    CCTGGATCCTTCCAGCATGTTCTACNT
SRR765715.19898449.2-                                        GGATCCTTCCAGCATGTTCTCCCT
SRR765715.28871379.2-                                        GGATCCTTCCAGCATGTTCTACCT
SRR765710.14715271.1+                                          ATCCTTCCAGCATGTTCTACCT
SRR765712.165744054.2+                                         ATCCTTCCAGCATGTTCTACCT
SRR765712.73886187.1-                                          ATCCTTCCAGCATGTTCTACCT
SRR765711.9180360.1-                                             CCTTCCAGCATGTTCTACCT
SRR765712.88008628.1+                                             CTTCCAGCATGTTCTACCT
SRR765712.125893357.1+                                              TCCAGCATGTTCTACCT
SRR765712.138777141.2+                                              TCCAGCATGTTCTACCT
SRR765712.228231813.1-                                              TCCAGCATGTTCTACCT
SRR765711.125330306.2+                                               CCAGCATGTTCTACCT
SRR765712.126177480.2-                                               CCAGCATGTTCTACCT
SRR765712.232059580.2-                                               CCAGCATGTTCTACCT
SRR765712.57478692.1-                                                CCAGCATGTTCTACCT
SRR765715.27991329.2+                                                CCAGCATGTTCTACCT
SRR765715.48806277.2+                                                CCAGCATGTCCTACCT
SRR765709.21897156.1+                                                 CAGCATGTTCTACCT
SRR765712.22158653.1-                                                   GCATGTTCTACCT
SRR765713.31177874.1+                                                    CATGTTCTACCT
SRR765712.136103372.2+                                                    ATGTTCTACCT
SRR765712.58838374.1-                                                     ATGTTCTACCT
SRR765712.152915721.2-                                                      GTTCTACCT
SRR765712.43404440.1+                                                       GTTCTACCT
SRR765709.5623476.1-                                                         TTCTACCT
SRR765711.15546394.1-                                                        TTCTGCCT
SRR765711.51569470.2-                                                        TTCTACCT
SRR765712.195958303.1-                                                       TTCTACCT
SRR765713.39071978.2-                                                         TCTACCT
SRR765712.11807389.2-                                                           TACCT
SRR765712.99870371.1+                                                           TACCT
SRR765712.46974017.2-                                                             CCT
SRR765712.203325839.2-                                                             CT
SRR765709.80799489.1-                                                               T
                         ------------------------------------------------------------
consensus                GCTTGCATCTCTGCTGCTGAGTTTCCCCACCTTCCTGGATCCTTCCAGCATGTTCTACCT

                             .    :    .    :    .    :    .    :    .    :    .    :
SRR765711.136262268.2-   CC
SRR765714.39979308.2-    CC
SRR765710.1716354.2+     CCG
SRR765715.15378963.1+    CCGC
SRR765711.75189809.2-    CCGCT
SRR765712.106710182.2-   CCGCTGG
SRR765712.11807389.1+    CCGCTGGCA
SRR765714.149230942.1+   CCGCTGGCA
SRR765715.22812006.1-    CCGCTGGCA
SRR765710.6874784.1-     CCGCTGGCACT
SRR765711.39537275.1-    CCGCTGGCACT
SRR765711.90300589.1-    CCGCTGGCACTAT
SRR765712.166853237.2-   CCGCTGGCACTAT
SRR765711.15546394.2+    CCGCTGGCACTATA
SRR765711.92360717.1+    CCGCGGGCACTATA
SRR765710.77934178.1-    CCGCTGGCACTATAAG
SRR765712.179891936.1+   CCGCTGGCACTATAAGGAA
SRR765714.71098406.2-    CCGCTGGCACTATAAGGAA
SRR765711.42060791.2-    CCGCTGGCACTATAAGGAAC
SRR765711.70787159.2-    CCGCTGGCACTATAAGGAAC
SRR765712.203325839.1+   CCGCTGGCACTATAAGGAACAGGA
SRR765710.48848881.1-    CCGCTGGCACTATAAGGAACAGGAGC
SRR765711.83011887.1+    CCGCTGGCACTATAAGGAACAGGAGCT
SRR765712.46974017.1+    CCGCTGGCACTATAAGGAACAGGAGCT
SRR765709.26128387.2+    CCGCTGGCACTATAAGGAACAGGAGCTG
SRR765710.29061231.2+    CCGCTGGCACTATAAGGAACAGGAGCTG
SRR765716.12328120.2+    CCGCTGGCGCTATAAGGAACAGGAGCTGAT
SRR765716.14688142.2+    CCGCTGGCACTATAAGGAACAGGAGCTGAT
SRR765716.19440677.2+    CCGCTGGCACTATAAGGAACAGGAGCTGAT
SRR765716.25986022.2+    CCGCTGGCACTATAAGGAACAGGAGCTGAT
SRR765711.31552385.1+    CCGCTGGCACTATAAGGAACAGGAGCTGATG
SRR765711.120800792.1-   CCGCTGGCACTATAAGGAACAGGAGCTGATGG
SRR765713.15891820.1-    CCGCTGGCACTATAAGGAACAGGAGCTGATGG
```

SRR765711.81304240.2-   CCGCTGGCACTATAAGGAACAGGAGCTGATGGG
SRR765715.46017009.2+   CCGCTGGCACTATAAGGAACAGGAGCTGATGGGG
SRR765710.53338134.2-   CCGCTGGCACTATAAGGAACAGGAGCTGATGGGGTT
SRR765712.73428311.1-   CCGCTGGCACTATAAGGAACAGGAGCTGATGGGGTT
SRR765711.39834426.2+   CCGCTGGCACTATAAGGAACAGGAGCTGATGGGGTTG
SRR765712.29757431.2-   CCGCTGGCACTATAAGGAACAGGAGCTGATGGGGTTGAG
SRR765712.76576490.1+   CCGCTGGCACTATAAGGAACAGGAGCTGATGGGGTTGAGC
SRR765711.79688649.2-   CCGCTGGCACTATAAGGAACAGCAGCTGATGGGGTTGAGCT
SRR765714.68056853.2-   CCGCTGGCACTATAAGGAACAGGAGCTGATGGGGTTGAGCT
SRR765714.88849388.2-   CCGCTGGCACTATAAGGAACAGGAGCTGATGGGGTTGAGCT
SRR765713.12243610.2+   CCGCTGGCACTATAAGGAACAGGAGCTGATGGGGTTGAGCTGCACA
SRR765714.142532987.2+   CCGCTGGCACTATAAGGAACAGGAGCTGATCGGGTTGAGCTGCACA
SRR765714.54269538.2+   CCGCTGGCACTATAAGGAACAGGAGCTGATGGGGTTGAGCTGCACA
SRR765714.55316615.2+   CCGCTGGCACTATAAGGAACAGGAGCTGATGGGGTTGAGCTGCACA
SRR765710.78316546.1+   CCGCTGGCACTATAAGGAACAGGAGCTGATGGGGTTGAGCTGCACAT
SRR765711.42145081.2+   CCGCTGGCACTATAAGGAACAGGAGCTGATGGGGTTGAGCTGCACAT
SRR765711.50432206.1-   CCGCTGGCACTATAAGGAACAGGAGCTGATGGGGTTGAGCTGCACAT
SRR765712.25672489.1+   CCGCTGGCACTATAAGGAACAGGAGCTGATGGGGTTGAGCTGCACATC
SRR765712.73551137.2-   CCGCTGGCACTATAAGGAACAGGAGCTGATGGGGTTGAGCTGCACATC
SRR765714.88899945.1+   CCGCTGGCACTATAAGGAACAGGAGCTGATGGGGTTGAGCTGCACATC
SRR765711.51569470.1+   CCGCTGGCACTATAAGGAACAGGAGCTGATGGGGTTGAGCTGCACATCC
SRR765712.139323924.1+   CCGCTGGCACTATAAGGAACAGGAGCTGATGGGGTTGAGCTGCACATCC
SRR765713.15730043.1-   CCGCTGGCACTATAAGGAACAGGAGCTGATGGGGTTGAGCTGCACATCC
SRR765712.27955158.1+   CCGCTGGCACTATAAGGAACAGGAGCTGATGGGGTTGAGCTGCACATCCACA
SRR765709.3017797.2+   CCGCTGGCACTATAAGGAACAGGAGCTGATGGGGTTGAGCTGCACATCCACACAA
SRR765714.45211402.1+   CCGCTGGCACTATAAGGAACAGGAGCTGATGGGGTTGAGCTGCACATCCACACAA
SRR765709.15051614.1-   CCGCTGGCACTATAAGGAACAGGAGCTGATGGGGTTGAGCTGCACATCCACACAAT
SRR765709.56879908.1-   CCGCTGGCACTATAAGGAACAGGAGCTGATGGGGTTGAGCTGCACATCCACACAAT
SRR765711.15445266.1-   CCGCTGGCACTATAAGGAACAGGAGCTGATGGGGTTGAGCTGCACATCCACACAAT
SRR765714.126051084.2-   CCGCTGGCACTATAAGGAACAGGAGCTGATGGGGTTGAGCTGCACATCCACACAATT
SRR765710.11110602.1+   CCGCTGGCACTATAAGGAACAGGAGCTGATGGGGTTGAGCTGCACATCCACACAATTGG
SRR765709.17947042.2-   CCGCTGGCACTATAAGGAACAGGAGCTGATGGGGTTGAGCTGCACATCCACACAATTGGC
SRR765709.21897156.1+   CCGCTGGCACTATAAGGAACAGGAGCTGATGGGGTTGAGCTGCACATCCACACAATTGGC
SRR765709.44632583.2+   CCGCTGGCACTATAAGGAACAGGAGCTGATGGGGTTGAGCTGCACATCCACACAATTGGC
SRR765709.5623476.1-   CCGCTGGCACTATAAGGAACAGGAGCTGATGGGGTTGAGCTGCACATCCACACAATTGGC
SRR765709.80799489.1-   CCGCTGGCACTATAAGGAACAGGAGCTGATGGGGTTGAGCTGCACATCCACACAATTGGC
SRR765710.11930567.2-   CCGCTGGCACTATAAGGAACAGGAGCTGATGGGGTTGAGCTGCACATCCACACAATTGGC
SRR765710.14715271.1+   CCGCTGGCACTATAAGGAACAGGAGCTGATGGGGTTGAGCTGCACATCCACACAATTGGC
SRR765710.1716354.1-   CCGCTGGCACTATAAGGAACAGGAGCTGATGGGGTTGAGCTGCACATCCACACAATTGGC
SRR765710.66135762.2+   CCGCTGGCACTATAAGGAACAGGAGCTGATGGGGTTGAGCTGCCCATCCACACAATTGGC
SRR765711.117156605.1-   CCGCTGGCACTATAAGGAACAGGAGCTGATGGGGTTGAGCTGCACATCCACACAATTGGC
SRR765711.125330306.2+   CCGCTGGCACTATAAGGCACAAGAGCTGATGGGGTTGAGCTGCACATCCACACAATTGGC
SRR765711.15546394.1-   CCGCTGGCACTATAAGGAACAGGAGCTGATGGGGTTGAGCTGCACATCCACACAATTGGC
SRR765711.51569470.2-   CCGCTGGCACTATAAGGAACAGGAGCTGATGGGGTTGAGCTGCACATCCACACAATTGGC
SRR765711.9180360.1-   CCGCTGGCAGTATAAGGACCAGGAGCTGATGGGGTTGAGCTGCCCATCCACACAATTGGC
SRR765712.117148243.1-   CCGCTGGCACTATAAGGAACAGGAGCTGATGGGGTTGAGCTGCACATCCACACAATTGGC
SRR765712.11807389.2-   CCGCTGGCACTATAAGGAACAGGAGCTGATGGGGTTGAGCTGCACATCCACACAATTGGC
SRR765712.125893357.1+   CCGCTGGCACTATAAGGAACAGGAGCTGATGGGGTTGAGCTGCACATCCACACAATTGGC
SRR765712.126177480.2-   CCGCTGGCACTATAAGGAACAGGAGCTGATGGGGTTGAGCTGCACATCCACACAATTGGC
SRR765712.136103372.2+   CCGCTGGCACTATAAGGAACAGGAGCTGATGGGGTTGAGCTGCACATCCACACAATTGGC
SRR765712.138777141.2+   CCGCTGGCACTATAAGGAACAGGAGCTGATGGGGTTGAGCTGCACATCCACACAATTGGC
SRR765712.152915721.2-   CCGCTGGCACTATAAGGAACAGGAGCTGATGGGGTTGAGCTGCACATCCACACAATTGGC
SRR765712.15635258.1-   CCGCTGGCACTATAAGGAACAGGAGCTGATGGGGTTGAGCTGCACATCCACACAATTGGC
SRR765712.165744054.2+   CCGCTGGCACTATAAGGAACAGGAGCTGATGGGGTTGAGCTGCACATCCACACAATTGGC
SRR765712.165805728.1-   CCGCTGGCACTATAAGGAACAGGAGCTGATGGGGTTGAGCTGCATATCCACACAATTGGC
SRR765712.173925922.1+   CCGCTGGCACTATAAGGAACAGGAGCTGATGGGGTTGAGCTGCACATCCACACAATTGGC
SRR765712.195958303.1-   CCGCTGGCACTATAAGGAACAGGAGCTGATGGGGTTGAGCTGCACATCCACACAATTGGC
SRR765712.203325839.2-   CCGCTGGCACTATAAGGAACAGGAGCTGATGGGGTTGAGCTGCACATCCACACAATTGGC
SRR765712.208044178.2-   CCGCTGGCACTATAAGGAACAGGAGCTGATGGGGTTGAGCTGCACATCCACACAATTGGC
SRR765712.21124108.1-   CCGCTGGCACTATAAGGAACAGGAGCTGATGGGGTTGAGCTGCACATCCACACAATTGGC
SRR765712.21952163.2-   CCGCTGGCACTATAAGGAACAGGAGCTGATGGGGTTGAGCTGCACATCCACACAATTGGC
SRR765712.22158653.1-   CCGCTGGCACTATAAGGAACAGGAGCTGATGGGGTTGAGCTGCACATCCACACAATTGGC
SRR765712.228231813.1-   CCGCTGGCACTATAAGGAACAGGAGCTGATGGGGTTGAGCTGCACATCCACACAATTGGC
SRR765712.232059580.2-   CCGCTGGCACTATAAGGAACAGGAGCTGATGGGGTTGAGCTGCACATCCACACAATTGGC
SRR765712.43404440.1+   CCGCTGGCACTATAAGGAACAGGAGCTGATGGGGTTGAGCTGCACATCCACACAATTGGC
SRR765712.46974017.2-   CCGGTGGCACTATAAGGAACAGGAGCTGATGGGGTTGAGCTGCACATCCACACAATTGGC
SRR765712.57478692.1-   CCGCTGGCACTATAAGGAACAGGAGCTGATGGGGTTGAGCTGCACATCCACACAATTGGC
SRR765712.58838374.1-   CCGCTGGCACTATAAGGAACAGGAGCTGATGGGGTTGAGCTGCACATCCACACAATTGGC
SRR765712.71088928.2-   CCGCTGGCACTATAAGGAACAGGAGCTGATGGGGTTGAGCTGCACATCCACACAATTGGC
SRR765712.73886187.1-   CCGCTGGCACTATAAGGAACAGGAGCTGATGGGGTTGAGCTGCACATCCACACAATTGGC
SRR765712.88008628.1+   CCGCTGGCACTATAAGGAACAGGAGCTGATGGGGTTGAGCTGCACATCCACACAATTGGC
SRR765712.99870371.1+   CCGCTGGCACTATAAGGAACAGGTGCTGATGGGGTTGAGCTGCACATCCACACAATTGGC
SRR765713.31177874.1+   CCGCTGGCACTATAAGGAACAGGAGCTGATGGGGTTGAGCTGCACATCCACACAATTGGC
SRR765713.39071978.2-   CCGCTGGCACTATAAGGAACAGGAGCTGATGGGGTTGAGCTGCACATCCACACAATTGGC
SRR765714.103329039.2+   CCGCTGGCACTATAAGGAACAGGAGCTGATGGGGTTGAGCTGCACATCCACACAATTGGC
SRR765715.11770606.2-   CCGCTGGCACTATAAGGAACAGGAGCTGATGGGGTTGAGCTGCACATCCACACAATTGGC
SRR765715.16262666.1+   CCGCTGGCACTATAAGGAACAGGAGCTGATGGGGTTGAGCTGCACATCCACACAATTGGC
SRR765715.18589469.1+   CCGCTGGCACTATAAGGAACAGGAGCTGATGGGGTTGAGCTGCACATCCACACAATTGGC
SRR765715.19898449.2-   CCGCTGGCACTATAAGGACCAGGAGCTGATGGGGTTGAGCTGCACATCCACACAATTGGC
SRR765715.27991329.2+   CCGCTGGCACTATAAGGAACAGGAGCTGATGGGGTTGAGCTGCACATCCACACAATTGGC
SRR765715.28871379.2-   CCGCTGGCACTATAAGGAACAGGAGCTGATGGGGTTGAGCTGCACATCCACACAATTGGC
SRR765715.41985107.2-   CCGCTGGCACTATAAGGAACAGGAGCTGATGGGGTTGAGCTGCACATCCACACAATTGGC
SRR765715.48806277.2+   CCGCTGGCACTATAAGGAACAGGAGCTGATGGGGTTGAGCTGCACATCCACACAATTGGC
SRR765715.5481277.1+   CCGCTGGCACTATAAGGAACAGGAGCTGATGGGGTTGAGCTGCACATCCACACAATTGGC

```
SRR765713.24490767.2-     CGCTGGCACTATAAGGAACAGGAGCTGATGGGGTTGAGCTGCACATCCACACAATTGGC
SRR765713.901098.2-       CGCTGGCACTATAAGGAACAGGAGCTGATGGGGTTGAGCTGCACATCCACACAATTGGC
SRR765714.106732036.2-    CGCTGGCACTATAAGGAACAGGAGCTGATGGGGTTGAGCTGCACATCCACACAATTGGC
SRR765709.27935475.2+      GCTGGCACTATAAGGAACAGGAGCTGATGGGGTTGAGCTGCACATCCACACAATTGGC
SRR765710.12300792.2+      GCTGGCACTATAAGGAACAGGAGCTGATGGGGTTGAGCTGCACATCCACACAATTGGC
SRR765711.92360717.2-      GCTGGCACTATAAGGAACAGGAGCTGATGGGGTTGAGCTGCACATCCACACAATTGGC
SRR765712.183278959.1+       TGGCACTATAAGGAACAGGAGCTGAGGGGGTTGAGCTGCACATCCACACAATTGGC
SRR765713.30731876.1-        TGGCACTATAAGGAACAGGAGCTGATGGGGTTGAGCTGCACATCCACACAATTGGC
SRR765714.180169356.1-       TGGCACTATAAGGAACAGGAGCTGATGGGGTTGAGCTGCACATCCACACAATTGGC
SRR765714.30609878.1+        TGGCACTATAAGGAACAGGAGCTGATGGGGTTGAGCTGCACATCCACACAATTGGC
SRR765712.125325157.1+        GGCACTATAAGGAACAGGAGCTGATGGGGTTGAGCTGCACATCCACACAATTGGC
SRR765714.154250096.1-         GCACTATAAGGAACAGGAGCTGATGGGGTTGAGCTGCACATCCACACAATTGGC
SRR765712.179891936.2-          CACTATAAGGAACAGGAGCGGATTGGGTTGAGCTTCACATCCACTCAATTGGC
SRR765709.82540197.1+            ACTATAAGGAACAGGAGCTGATGGGGTTGAGCTGCACATCCACACAATTGGC
SRR765710.29061231.1-            ACTATAAGGAACAGGAGCTGATGGGGTTGAGCTGCACATCCACACAATTGGC
SRR765712.158301577.1+           ACTATAAGGAACAGGAGCTGAAGGGGTTGAGCTGCACATCCACACAATTGGC
SRR765714.129507513.1+            CTATAAGGAACAGGAGCTGATGGGGTTGAGCTGCACATCCACACAATTGGC
SRR765714.140601593.2+            CTATAAGGAACAGGAGCTGATGGGGTTGAGCTGCACATCCACACAATTGGC
SRR765714.168159457.1+            CTATAAGGAACAGGAGCTGATGGGGTTGAGCTGCACATCCACACAATTGGC
SRR765715.31628901.1+              TATAAGGAACAGGAGCTGATGGGGTTGAGCTGCACATCCACACAATTGGC
SRR765715.45770009.1+              TATAAGGAACAGGAGCTGATGGGGTTGAGCTGCACATCCACACAATTGGC
SRR765709.85918077.1+               ATAAGGAACAGGAGCTGATGGGGTTGAGCTGCACATCCACACAATTGGC
SRR765712.139323924.2-              ATAAGGAACAGGAGCTGATGGGGTTGAGCTGCACATCCACACAATTGGC
SRR765712.85106693.1+               ATAAGGAACAGGAGCTGATGGGGTTGAGCTGCACATCCACACAATTGGC
SRR765712.171142138.1+               TAAGGAACAGGAGCTGATGGGGTTGAGCTGCACATCCACACAATTGGC
SRR765712.128159580.2+                 AGGAACAGGAGCTGATGGGGTTGAGCTGCACATCCACACAATTGGC
SRR765714.149178143.2-                 AGGAACAGGAGCTGATGGGGTTGAGCTGCACATCCACACAATTGGC
SRR765710.73677951.2+                   GGAACAGGAGCTGATGGGGTTGAGCTGCACATCCACACAATTGGC
SRR765711.125330306.1-                    AACAGGAGCTGATGGGGTTGAGCTGCACATCCACACAATTGGC
SRR765711.83011887.2-                       CAGGAGCTGATGGGGTTGAGCTGCACATCCACACAATTGGC
SRR765714.149867042.1+                      CAGGAGCTGATGGGGTTGAGCTGCACATCCACACAATTGGC
SRR747825.14576590.2+                        AGGAGCTGATGGGGTTGAGCTGCACATCCACACAATTGGC
SRR747825.14585113.1+                        AGGAGCTGATGGGGTTGAGCTGCACATCCACACAATTGGC
SRR747825.19360308.2+                        AGGAGCTGATGGGGTTGAGCTGCACATCCACACAATTGGC
SRR747825.1972727.1+                         AGGAGCTGATGGGGTTGAGCTGCACATCCACACAATTGGC
SRR747825.21619580.2+                        AGGAGCTGATGGGGTTGAGCTGCACATCCACACAATTGGC
SRR747825.22167783.1+                        AGGAGCTGATGGGGTTGAGCTGCACATCCACACAATTGGC
SRR747825.22770718.1+                        AGGAGCTGATGGGGTTGAGCTGCACATCCACACAATTGGC
SRR747825.26682374.2+                        AGGAGCTGATGGGGTTGAGCTGCACATCCACACAATTGGC
SRR747825.26883748.1+                        AGGAGCTGATGGGGTTGAGCTGCACATCCACACAATTGGC
SRR747825.31272404.2+                        AGGAGCTGATGGGGTTGAGCTGCACATCCACACAATTGGC
SRR747825.33273337.2+                        AGGAGCTGATGGGGTTGAGCTGCACATCCACACAATTGGC
SRR747825.33273338.2+                        AGGAGCTGATGGGGTTGAGCTGCACATCCACACAATTGGC
SRR747825.33533006.2+                        AGGAGCTGATGGGGTTGAGCTGCACATCCACACAATTGGC
SRR747825.35561440.2+                        AGGAGCTGATGGGGTTGAGCTGCACATCCACACAATTGGC
SRR747825.36118365.1+                        AGGAGCTGATGGGGTTGAGCTGCACATCCACACAATTGGC
SRR747825.37409548.2+                        AGGAGCTGATGGGGTTGAGCTGCACATCCACACAATTGGC
SRR747825.3942779.2+                         AGGAGCTGATGGGGTTGAGCTGCACATCCACACAATTGGC
SRR747825.6652060.2+                         AGGAGCTGATGGGGTTGAGCTGCACATCCACACAATTGGC
SRR747825.7537942.2+                         AGGAGCTGATGGGGTTGAGCTGCACATCCACACAATTGGC
SRR747825.8318391.2+                         AGGAGCTGATGGGGTTGAGCTGCACATCCACACAATTGGC
SRR747825.8487474.2+                         AGGAGCTGATGGGGTTGAGCTGCACATCCACACAATTGGC
SRR747825.8731386.1+                         AGGAGCTGATGGGGTTGAGCTGCACATCCACACAATTGGC
SRR765710.27338352.2+                        AGGAGCTGATGGGGTTGAGCTGCACATCCACACAATTGGC
SRR765712.51340874.2+                        AGGAGCTGATGGGGTTGAGCTGCACATCCACACAATTGGC
SRR765712.72190442.2+                        AGGAGCTGATGGGGTTGAGCTGCACATCCACACAATTGGC
SRR765713.33934669.1+                        AGGAGCTGATGGGGTTGAGCTGCACATCCACACAATTGGC
SRR765713.9198519.1+                         AGGAGCTGATGGGGTTGAGCTGCACATCCACACAATTGGC
SRR765714.114351789.1+                       AGGAGCTGATGGGGTTGAGCTGCACATCCACACAATTGGC
SRR765711.13566352.1+                         GGAGCTGATGGGGTTGAGCTGCACATCCACACAATTGGC
SRR765712.76576490.2-                         GGAGCTGATGGGGTTGAGCTGCACATCCACACAATTGGC
SRR765711.39834426.1-                          GAGCTGATGGGGTTGAGCTGCACATCCACACAATTGGC
SRR765711.79664446.2+                          GAGCTGATGGGGTTGAGCTGCACATCCACACAATTGGC
SRR765709.3017797.1-                             GCTGATGGGGTTGAGCTGCACATCCACACAATTGGC
SRR747825.12487751.2-                             CTGATGGGGTTGAGCTGCACATCCACACAATTGGC
SRR747825.12965830.2-                             CTGATGGGGTTGAGCTGCACATCCACACAATTGGC
SRR747825.14196148.2-                             CTGATGGGGTTGAGCTGCACATCCACACAATTGGC
SRR747825.17726022.2-                             CTGATGGGGTTGAGCTGCACATCCACACAATTGGC
SRR747825.21410409.2-                             CTGATGGGGTTGAGCTGCACATCCACACAATTGGC
SRR747825.26095160.2-                             CTGATGGGGTTGAGCTGCACATCCACACAATTGGC
SRR747825.26346030.2-                             CTGATGGGGTTGAGCTGCACATCCACACAATTGGC
SRR747825.35226945.2-                             CTGATGGGGTTGAGCTGCACATCCACACAATTGGC
SRR747825.36267467.2-                             CTGATGGGGTTGAGCTGCACATCCACACAATTGGC
SRR747825.4608608.2-                              CTGATGGGGTTGAGCTGCACATCCACACAATTGGC
SRR747825.7988087.2-                              CTGATGGGGTTGAGCTGCACATCCACACAATTGGC
SRR747825.8350873.2-                              CTGATGGGGTTGAGCTGCACATCCACACAATTGGC
SRR765710.11110602.2-                             CTGATGGGGTTGAGCGGCACATCCACACAATTGGC
SRR765714.63395510.2-                             CTGATGGGGTTGAGCTGCACATCCACACAATTGGC
SRR765714.82008612.2-                             CTGATGGGGTTGAGCTGCACATCCACACAATTGGC
SRR747825.42525078.2-                              TGATGGGGTTGAGCTGCACATCCACACAATTGGC
SRR765711.6290287.2+                               TGATGGGGTTGAGCTGCACATCCACACAATTGGC
SRR765712.197777944.1+                             TGATGGGGTTGAGCTGCACATCCACACAATTGGC
SRR765710.78316546.2-                               GATGGGGTTGAGCTGCACATCCACACAATTGGC
SRR765709.13217291.1+                                ATGGGGTTGAGCTGCACATCCACACAATTGGC
```

```
SRR765715.16262666.2-                                ATGGGGTTGAGCTGCACATCCGCACAATTGGC
SRR765715.18589469.2-                                ATGGGGTTGAGCTGCACATCCACACAATTGGC
SRR765716.10147584.2+                                 TGGGGTTGAGCTGCACATCCACACAATTGGC
SRR765716.18110791.2+                                 TGGGGTTGAGCTGCACATCCACACAATTGGC
SRR765709.13162642.1-                                  GGGGTTGAGCTGCACATCCACACAATTGGC
SRR765710.41723497.1+                                    GGTTGAGCTGCACATCCACACAATTGGC
SRR765714.77066962.1+                                    GGTTGAGCTGCACATCCACACAATTGGC
SRR765711.31552385.2-                                     GTTGAGCTGCACATCCACACAATTGGC
SRR765711.35892352.2+                                     GTTGAGCTGCACATCCACACAATTGGC
SRR765714.181917353.1-                                    GTTGAGCTGCACATCCACACAATTGGC
SRR765714.23956215.1+                                      TTGAGCTGCACATCCACACAATTGGC
SRR765710.66135762.1-                                       TGAGCTGCACATCCACACGATTGGC
SRR765712.88008628.2-                                        GAGCTGCACATCCACACAATTGGC
SRR765711.102739620.2+                                        AGCTGCACATCCACACAACTGGC
SRR765714.181600550.1-                                        AGCTGCACATCCACACAATTGGC
SRR765712.148429673.1+                                         GCTGCACATCCACACAATTGGC
SRR765714.178949095.1-                                          CTGCACATCCACACAATTGGC
SRR765711.56339811.1+                                            TGCACATCCACACAATTGGC
SRR765709.40884677.1+                                             GCACATCCACACAATTGGC
SRR765712.25672489.2-                                              CACATCCACACAATTGGC
SRR765712.220295908.2+                                              ACATCCACACAATTGGC
SRR765712.27955158.2-                                               ACATCCACACAATTGGC
SRR765712.151632343.1+                                                ATCCACACAATTGGC
SRR765713.8355185.1+                                                  ATCCACACAATTGGC
SRR765710.10250671.1+                                                   CCACACAATTGGC
SRR765710.6584671.2+                                                     CACACAATTGGC
SRR765714.86734657.2-                                                     ACACAATTGGC
SRR765712.125893357.2-                                                     CACAATTGGC
SRR765710.14715271.2-                                                        ACAATGGGC
SRR765714.195543010.2-                                                       ACAATTGGC
SRR765714.43208061.2+                                                        ACAATTGGC
SRR765715.27991329.1-                                                        ACAATTGGC
SRR765715.48806277.1-                                                        ACAATTGGC
SRR765711.42145081.1-                                                         CAATTGGC
SRR765712.136103372.1-                                                        CAATTGGC
SRR765714.102557296.2-                                                        CAATTGGC
SRR765714.111689744.2-                                                        CAATTGGC
SRR765709.44632583.1-                                                          AATTGGC
SRR765709.62403711.1+                                                          AATTGGC
SRR765712.121630012.2+                                                         AATTGGC
SRR765712.189246974.2+                                                         AATTGGC
SRR765716.13424455.2-                                                           ATTGGC
SRR765716.27196679.2-                                                           ATTGGC
SRR765716.6716252.2-                                                            ATTGGC
SRR765711.55682203.2+                                                             TGGC
SRR765714.4956404.2-                                                              TGGC
SRR765709.15951076.2+                                                              GGC
SRR765709.87544394.2-                                                              GGC
SRR765712.173925922.2-                                                              GC
SRR765716.13051448.2-                                                               GC
                         ------------------------------------------------------------
consensus                CCGCTGGCACTATAAGGAACAGGAGCTGATGGGGTTGAGCTGCACATCCACACAATTGGC

                             .    :    .    :    .    :    .    :    .    :    .    :
SRR765710.66135762.2+    T
SRR765710.11930567.2-    TGG
SRR765709.44632583.2+    TGGGTG
SRR765712.117148243.1-   TGGGTG
SRR765715.5481277.1+     TGGGTG
SRR765712.208044178.2-   TGGGTGGC
SRR765712.165805728.1-   TGGGTGGCAC
SRR765712.21124108.1-    TGGGTGGCAC
SRR765712.71088928.2-    TGGGTGGCAC
SRR765710.1716354.1-     TGGGTGGCACA
SRR765715.16262666.1+    TGGGTGGCACA
SRR765715.18589469.1+    TGGGTGGCACA
SRR765712.15635258.1-    TGGGTGGCACAT
SRR765711.117156605.1-   TGGGTGGCACATG
SRR765712.21952163.2-    TGGGTGGCACATG
SRR765709.17947042.2-    TGGGTGGCACATGG
SRR765712.173925922.1+   TGGGTGGCACATGG
SRR765715.19898449.2-    TGGGTGGCACATGGAGA
SRR765715.28871379.2-    TGGGTGGCACATGGAGA
SRR765711.92360717.2-    TGGGTGGCACATGGAGAG
SRR765710.14715271.1+    TGGGTGGCACATGGAGAGC
SRR765712.165744054.2+   TGGGTGGCACATGGAGAGC
SRR765712.73886187.1-    TGGGTGGCACATGGAGAGC
SRR765711.9180360.1-     TGGGTGGCACATGGAGAGCCC
SRR765712.88008628.1+    TGGGTGGCACATGGAGAGCCCT
SRR765712.125893357.1+   TGGGTGGCACATGGAGAGCCCTCA
SRR765712.138777141.2+   TGGGTGGCACATGGAGAGCCCTCA
SRR765712.228231813.1-   TGGGTGGCACATGGAGAGCCCTCA
SRR765711.125330306.2+   TGGGTGGCACATGGAGAGCCCTCAT
SRR765712.126177480.2-   TGGGTGGCACATGGAGAGCCCTCAT
```

SRR765712.232059580.2-	TGGGTGGCACATGGAGAGCCCTCAT
SRR765712.57478692.1-	TGGGTGGCACATGGAGAGCCCTCAT
SRR765715.27991329.2+	TGGGTGGCACATGGAGAGCCCTCAT
SRR765715.48806277.2+	TGGGTGGCACATGGAGAGCCCTCAT
SRR765709.21897156.1+	TGGGTGGCACATGGAGAGCCCTCATG
SRR765712.22158653.1-	TGGGTGGCACATGGAGAGCCCTCATGGA
SRR765713.31177874.1+	TGGGTGGCACATGGAGAGCCCTCATGGA
SRR765712.136103372.2+	TGGGTGGCACATGGAGAGCCCTCATGGAGA
SRR765712.58838374.1-	TGGGTGGCACATGGAGAGCCCTCATGGAGA
SRR765712.152915721.2-	TGGGTGGCACATGGAGAGCCCTCATGGAGAGC
SRR765712.43404440.1+	TGGGTGGCACATGGAGAGCCCTCATGGAGAGC
SRR765709.5623476.1-	TGGGTGGCACATGGAGAGCCCTCATGGAGAGCT
SRR765711.15546394.1-	TGGGTGGCACATGGAGAGCCCTCATGGAGAGCT
SRR765711.51569470.2-	TGGGTGGCACATGGAGAGCCCTCATGGAGAGCT
SRR765712.195958303.1-	TGGGTGGCACATGGAGAGCCCTCATGGAGAGCT
SRR765713.39071978.2-	TGGGTGGCACATGGAGAGCCCTCATGGAGAGCT
SRR765712.11807389.2-	TGGGTGGCACATGGAGAGCCCTCATGGAGAGCTGCC
SRR765712.99870371.1+	TGGGTGGCACATGGAGAGCCCTCATGGAGAGCTGCC
SRR765712.46974017.2-	TGGGTGGCACATGGAGAGCCCTCATGGAGAGCTGCCTG
SRR765712.203325839.2-	TGGGTGGCACATGGAGAGCCCTCATGGAGAGCTGCCTGG
SRR765709.80799489.1-	TGGGTGGCACATGGAGAGCCCTCATGGAGAGCTGCCTGGA
SRR765713.24490767.2-	TGGGTGGCACATGGAGAGCCCTCATGGAGAGCTGCCTGGAT
SRR765713.901098.2-	TGGGTGGCACATGGAGAGCCCTCATGGAGAGCTGCCTGGAT
SRR765714.106732036.2-	TGGGTGGCACATGGAGAGCCCTCATGGAGAGCTGCCTGGAT
SRR765709.27935475.2+	TGGGTGGCACATGGAGAGCCCTCATGGAGAGCTGCCTGGATCT
SRR765710.12300792.2+	TGGGTGGCACATGGAGAGCCCTCATGGAGAGCTGCCTGGATCT
SRR765713.30731876.1-	TGGGTGGCACATGGAGAGCCCTCATGGAGAGCTGCCTGGATCTG
SRR765714.180169356.1-	TGGGTGGCACATGGAGAGCCCTCATGGAGAGCTGCCTGGATCTG
SRR765714.30609878.1+	TGGGTGGCACATGGAGAGCCCTCATGGAGAGCTGCCTGGATCTG
SRR765712.158301577.1+	TGGGTGGCACATGGAGAGCCCTCATGGAGAGCTGCCTGGATCTGA
SRR765712.183278959.1+	TGGGTGGCACATGGAGAGCCCTCATGGAGAGCTGCCTGGATCTGA
SRR765712.125325157.1+	TGGGTGGCACATGGAGAGCCCTCATGGAGAGCTGCCTGGATCTGAC
SRR765714.154250096.1-	TGGGTGGCACATGGAGAGCCCTCATGGAGAGCTGCCTGGATCTGAC
SRR765712.179891936.2-	TGGGTGGCACATGGAGAGCCCTCATGGAGAGCTGCCTGGATCTGACAC
SRR765709.82540197.1+	TGGGTGGCACATGGAGAGCCCTCATGGAGAGCTGCCTGGATCTGACACT
SRR765710.29061231.1-	TGGGTGGCACATGGAGAGCCCTCATGGAGAGCTGCCTGGATCTGACACT
SRR765714.129507513.1+	TGGGTGGCACATGGAGAGCCCTCATGGAGAGCTGCCTGGATCTGACACT
SRR765714.140601593.2+	TGGGTGGCACATGGAGAGCCCTCATGGAGAGCTGCCTGGATCTGACACT
SRR765714.168159457.1+	TGGGTGGCACATGGAGAGCCCTCATGGAGAGCTGCCTGGATCTGACACT
SRR765715.45770009.1+	TGGGTGGCACATGGAGAGCCCTCATGGAGAGCTGCCTGGATCTGACACTG
SRR765715.31628901.1+	TGGGTGGCACATGGAGAGCCCTCATGGAGAGCTGCCTGGATCTGACACTGT
SRR765709.85918077.1+	TGGGTGGCACATGGAGAGCCCTCATGGAGAGCTGCCTGGATCTGACACTGTG
SRR765712.139323924.2-	TGGGTGGCACATGGAGAGCCCTCATGGAGAGCTGCCTGGATCTGACACTGTG
SRR765712.85106693.1+	TGGGGGGCACATGGAGAGCCCTCATGGAGAGCTGCCTGGATCTGACACTGTG
SRR747825.3942779.2+	TGGGTGGCACATGGAGAGCCCTCATGGAGAGCAGCCTGGAGCTGACACTGTGT
SRR765712.171142138.1+	TGGGTGGCACATGGAGAGCCCTCATGGAGAGCTGCCTGGATCTGACACTGTGT
SRR765714.149178143.2-	TGGGTGGCACATGGAGAGCCCTCATGGAGAGCTGCCTGGATCTGACACTGTGTC
SRR747825.22167783.1+	TGGGGGGCACATGGAGAGCCCGCATGGAGAGCTGCCTGGATCTGACACTGTGTCA
SRR765712.128159580.2+	TGGGTGGCACATGGAGAGCCCTCATGGAGAGCTGCCTGGATCTGACACTGTGTCA
SRR765710.73677951.2+	TGGGTGGCACATGGAGAGCCCTCATGGAGAGCTGCCTGGATCTGACACTGTGTCAG
SRR747825.22770718.1+	TGGGTGGCACATGGAGAGCCCTCATGGAGAGCTGCCTGGATCTGACACTGTGGCAGG
SRR765714.149867042.1+	TGGGTGGCACATGGAGAGCCCTCATGGAGAGCTGCCTGGATCTGACACTGTGTCAGG
SRR747825.8487474.2+	TGGGTGGCACATGGAGAGCCCTCATGGAGAGCTGCCTGGATCTGACACTGTGTCAGGA
SRR765711.125330306.1-	TGGGTGGCACATGGAGAGCCCTCATGGAGAGCTGCCTGGATCTGACACTGTGTCAGGA
SRR747825.19360308.2+	TGGGTGGCACATGGAGAGCCCTCATGGAGAGCTGCCTGGATCTGACACTGTGCCAGGAG
SRR747825.26682374.2+	TGGGTGGCACATGGAGAGCCCTCATGGAGAGCTGCCTGGATCTGACACTGTGTCAGGAG
SRR747825.6652060.2+	TGGGTGGCACATGGAGAGCCCTCATGGAGAGCTGCCTGGATCTGACACTGTGTCAGGAG
SRR765713.9198519.1+	TGGGTGGCACATGGAGAGCCCTCATGGAGAGCTGCCTGGATCTGACACTGTGTCAGGAG
SRR747825.12487751.2-	TGGGTGGCACATGGAGAGCCCTCATGGAGAGCTGCCTGGATCTGACACTGTGTCAGGAGA
SRR747825.12965830.2-	TGGGTGGCACATGGAGAGCCCTCATGGAGAGCTGCCTGGATCTGACACTGTGTCAGGAGA
SRR747825.14196148.2-	TGGGTGGCACATGGAGAGCCCTCATGGAGAGCTGCCTGGATCTGACACTGTGTCAGGAGA
SRR747825.14576590.2+	TGGGTGGCACATGGAGAGCCCTCATGGAGAGCTGCCTGGATCTGACACTGTGTCAGGAGA
SRR747825.14585113.1+	TGGGTGGCACATGGAGAGCCCTCATGGAGAGCTGCCTGGATCTGACACTGTGTCAGGAGA
SRR747825.17726022.2-	TGGGTGGCACATGGAGAGCCCTCATGGAGAGCTGCCTGGATCTGACACTGTGTCAGGAGA
SRR747825.1972727.1+	TGGGTGGCACATGGAGAGCCCTCATGGAGAGCTGCCTGGATCTGACACTGTGTCAGGAGA
SRR747825.21410409.2-	TGGGTGGCACATGGAGAGCCCTCATGGAGAGCTGCCTGGATCTGACACTGTGTCAGGAGA
SRR747825.21619580.2+	TGGGGGGCACATGGAGAGCCCTCATGGAGAGCTGCCTGGATCTGACACTGTGTCAGGAGA
SRR747825.26095160.2-	TGGGTGGCACATGGAGAGCCCTCATGGAGAGCTGCCTGGATCTGACACTGTGTCAGGAGA
SRR747825.26346030.2-	TGGGTGGCACATGGAGAGCCCTCATGGAGAGCTGCCTGGATCTGACACTGTGTCAGGAGA
SRR747825.26883748.1+	TGGGGGGCACATGGAGAGCCCTCATGGAGAGCTGCCTGGATCTGACACTGTGTCAGGAGA
SRR747825.31272404.2+	TGGGTGGCACATGGAGAGCCCTCATGGAGAGCTGCCTGGATCTGACACTGTGTCAGGAGA
SRR747825.33273337.2+	TGGGTGGCACATGGAGAGCCCACATGGAAAGCTGCCTGGATCTGACACTGTGTCAGGAGA
SRR747825.33273338.2+	TGGGTGGCACATGGAGAGCCCTCATGGAGAGCTGCCTGGATCTGACACTGTGTCAGGAGA
SRR747825.33533006.2+	TGGGTGGCACATGGAGAGCCCTCATGGAGAGCTGCCTGGATCTGACACTGTGTCAGGAGA
SRR747825.35226945.2-	TGGGTGGCACATGGAGAGCCCTCATGGAGAGCTGCCTGGATCTGACACTGTGTCAGGAGA
SRR747825.35561440.2+	TGGGTGGCACATGGAGAGCCCTCATGGAGAGCTGCCTGGATCTGACACTGTGTCAGGAGA
SRR747825.36118365.1+	TGGGTGGCACATGGAGAGCCCTCATGGAGAGCTGCCTGGATCTGACACTGTGTCAGGAGA
SRR747825.36267467.2-	TGGGTGGCACATGGAGAGCCCTCATGGAGAGCTGCCTGGATCTGACACTGTGTCAGGAGA
SRR747825.37409548.2+	TGGGTGGCACATGGAGAGCCCTCATGGAGAGCTGCCTGGATCTGACACTGTGTCAGGAGA
SRR747825.42525078.2-	TGGGTGGCACATGGAGAGCCCTCATGGAGAGCTGCCTGGATCTGACACTGTGTCAGGAGA
SRR747825.4608608.2-	TGGGTGGCACATGGAGAGCCCTCATGGAGAGCTGCCTGGATCTGACACTGTGTCAGGAGA
SRR747825.7537942.2+	TGGGTGGCACATGGAGAGCCCTCATGGAGAGCTGCCTGGATCTGACACTGTGTCAGGAGA
SRR747825.7988087.2-	TGGGTGGCACATGGAGAGCCCTCATGGAGAGCTGCCTGGATCTGACACTGTGTCAGGAGA
SRR747825.8318391.2+	TGGGTGGCACATGGAGAGCCCTCATGGAGAGCTGCCTGGATCTGACACTGTGTCAGGAGA

```
SRR747825.8350873.2-      TGGGTGGCACATGGAGAGCCCTCATGGAGAGCTGCCTGGATCTGACACTGTGTCAGGAGA
SRR747825.8731386.1+      TGGGTGGCACATGGAGAGCCCTCATGGAGAGCCGCCTGGATCTGACACTGTGTCAGGAGA
SRR765709.13162642.1-     TGGGTGGCACATGGAGAGCCCTCATGGAGAGCTGCCTGGATCTGACACTGTGTCAGGAGA
SRR765709.13217291.1+     TGGGGGGCACATGGAGAGCCCTCATGGAGAGCTACCTGGATCTGACACTGTGTCAGGAGA
SRR765709.15951076.2+     TGGGTGGCACATGGAGAGCCCTCATGGAGAGCTGCCTGGATCTGACACTGTGTCAGGAGA
SRR765709.3017797.1-      TGGGTGGCACATGGAGAGCCCTCATGGAGAGCTGCCTGGATCTGACACTGTGTCAGGAGA
SRR765709.40884677.1+     TGGGTGGCACATGGAGAGCCCTCATGTAGAGCTGCCTGGATCTGACACTGTGTCAGGAGA
SRR765709.44632583.1-     TGGGTGGCACATGGAGAGCCCTCATGGAGAGCTGCCTGGATCTGACACTGTGTCAGGAGA
SRR765709.44907493.2+     TGGGTGGCACATGGAGAGCCCTCATGGAGAGCTGCCTGGATCTGACACTGTGTCAGGAGA
SRR765709.62403711.1+     TGGGTGGCACATGGAGAGCCCTCATGGAGAGCTGCCTGGATCTGACACTGTGTCAGGAGA
SRR765709.87544394.2-     TGGGTGGCACATGGAGAGCCCTCATGGAGAGCTGCCTGGATCTGACACTGTGTCAGGAGA
SRR765710.10250671.1+     TGGGTGGCACATGGAGAGCCCTCATGGAGAGCTGCCTGGATCTGACACTGTGTCAGGAGA
SRR765710.11110602.2-     TGGGTGGCACATGGAGAGCCCTCATGGAGAGCTGCCTGGATCTGACACTGTGTCAGGAGA
SRR765710.14715271.2-     TGGGCGGCACATGGAGAGCCCTCATGGAGAGCTGCCTGGATCTGACACTGTGTCAGGAGA
SRR765710.27338352.2+     TGGGTGGCACATGGAGAGCCCTCATGGAGAGCTGCCTGGATCTGACACTGTGTCAGGAGA
SRR765710.41723497.1+     TGGGTGGCACATGGAGAGCCCTCATGGAGAGCTGCCTGGATCTGACACTGTGTCAGGAGA
SRR765710.6584671.2+      TGGGTGGCACATGGAGAGCCCTCATGGAGAGCTGCCTGGATCTGACACTGTGTCAGGAGA
SRR765710.66135762.1-     TGGGTGGCACATGGAGAGCCCTCATGGAGAGCTGCCTGGATCTGACACTGTGTCAGGAGA
SRR765710.78316546.2-     TGGGTGGCACATGGAGAGCCCTCATGGAGAGCTGCCTGGATCTGACACTGTGTCAGGAGA
SRR765711.102739620.2+    TGGGTGGCACATGGAGAGCCCTCATGGAGAGCTGCCTGGATCTGACACTGTGTCAGGAGA
SRR765711.13566352.1+     TGGGGGGCACATGGAGAGCCCTCATGGAGAGCTGCCTGGATCTGACACTGTGTCAGGAGA
SRR765711.31552385.2-     TGGGTGGCACATGGAGAGCCCTCATGGAGAGCTGCCTGGATCTGACACTGTGTCAGGAGA
SRR765711.35892352.2+     TGGGTGGCACATGGAGAGCCCTCATGGAGAGCTGCCTGGATCTGACACTGTGTCAGGAGA
SRR765711.39834426.1-     TGGGTGGCACATGGAGAGCCCTCATGGAGAGCTGCCTGGATCTGACACTGTGTCAGGAGA
SRR765711.42145081.1-     TGGGTGGCACATGGAGAGCCCTCATGGAGAGCTGCCTGGATCTGACACTGTGTCAGGAGA
SRR765711.55682203.2+     TGGGTGGCACATGGAGAGCCCTCATGGAGAGCTGCCTGGATCTGACACTGTGTCAGGAGA
SRR765711.56339811.1+     TGGGTGGCACATGGAGAGCCCTCATGGAGAGCTGCCTGGATCTGACACTGTGTCAGGAGA
SRR765711.6290287.2+      TGGGTGGCACATGGAGAGCCCTCATGGAGAGCTGCCTGGATCTGACACTGTGTCAGGAGA
SRR765711.79664446.2+     TGGGTGGCACATGGAGAGCCCTCATGGAGAGCTGCCTGGATCTGACACTGTGTCAGGAGA
SRR765711.83011887.2-     TGGGTGGCACATGGAGAGCCCTCATGGAGAGCTGCCTGGATCTGACACTGTGTCAGGAGA
SRR765712.121630012.2+    TGGGTGGCACATGGAGAGCCCTCATGGAGAGCTGCCTGGATCTGACACTGTGTCAGGAGA
SRR765712.125893357.2-    TGGGTGGCACATGGAGAGCCCTCATGGAGAGCTGCCTGGATCTGACACTGTGTCAGGAGA
SRR765712.136103372.1-    TGGGTGGCACATGGAGAGCCCTCATGGAGAGCTGCCTGGATCTGACACTGTGTCAGGAGA
SRR765712.148429673.1+    TGGGTGGCACATGGAGAGCCCTCATGGAGAGCTGCCTGGATCTGACACTGTGTCAGGAGA
SRR765712.151632343.1+    TGGGTGGCACATGGAGAGCCCTCATGGAGAGCTGCCTGGATCTGACACTGTGTCAGGAGA
SRR765712.173925922.2-    TGGGTGGCACATGGAGAGCCCTCATGGAGAGCTGCCTGGATCTGACACTGTGTCAGGAGA
SRR765712.189246974.2+    TGGGTGGAACATGGAGAGCCCTCATGGAGAGCTGCCTGGATCTGACACTGTGTCAGGAGA
SRR765712.197777944.1+    TGGGTGGCACATGGAGAGCCCTCATGGAGAGCTGCCGGGATCTGACACTGTGTCAGGAGA
SRR765712.202510854.2+    TGGGTGGCACATGGAGAGCCCTCATGGAGAGCTGCCTGGATCTGACACTGTGTCAGGAGA
SRR765712.220295908.2+    TGGGTGGCACATGGAGAGCCCTCATGGAGAGCTGCCTGGATCTGACACTGTGTCAGGAGA
SRR765712.25672489.2-     TGGGTGGCACATGGAGAGCCCTCATGGAGAGCTGCCTGGATCTGACACTGTGTCAGGAGA
SRR765712.27955158.2-     TGGGTGGCACATGGAGAGCCCTCATGGAGAGCTGCCTGGATCTGACACTGTGTCAGGAGA
SRR765712.51340874.2+     TGGGTGGCACATGGAGAGCCCTCATGGAGAGCTGCCTGGATCTGACACTGTGTCAGGAGA
SRR765712.72190442.2+     TGGGTGGCACATGGAGAGCCCTCATGGAGAGCTGCCTGGATCTGACACTGTGTCAGGAGA
SRR765712.76576490.2-     TGGGTGGCACATGGAGAGCCCTCATGGAGAGCTGCCTGGATCTGACACTGTGTCAGGAGA
SRR765712.88008628.2-     TGGGTGGCACATGGAGAGCCCTCATGGAGAGCTGCCTGGATCTGACACTGTGTCAGGAGA
SRR765713.33934669.1+     TGGGTGGCACATGGAGAGCCCTCATGGAGAGCTGCCTGGATCTGACACTGTGTCAGGAGA
SRR765713.8355185.1+      TGGGTGGCACATGGAGAGCCCTCATGGAGAGCCGCCTGGATCTGACACTGTGTCAGGAGA
SRR765714.102557296.2-    TGGGTGGCACATGGAGAGCCCTCATGGAGAGCTGCCTGGATCTGACACTGTGTCAGGAGA
SRR765714.111689744.2-    TGGGTGGCACATGGAGAGCCCTCATGGAGAGCTGCCTGGATCTGACACTGTGTCAGGAGA
SRR765714.114351789.1+    TGGGTGGCACATGGAGAGCCCTCATGGAGAGCTGCCTGGATCTGACACTGTGTCAGGAGA
SRR765714.178949095.1-    TGGGTGGCACATGGAGAGCCCTCATGGAGAGCTGCCTGGATCTGACACTGTGTCAGGAGA
SRR765714.181600550.1-    TGGGTGGCACATGGAGAGCCCTCATGGAGAGCTGCCTGGATCTGACACTGTGTCAGGAGA
SRR765714.181917353.1-    TGGGTGGCACATGGAGAGCCCTCATGGAGAGCTGCCTGGATCTGACACTGTGTCAGGAGA
SRR765714.195543010.2-    TGGGTGGCACATGGAGAGCCCTCATGGAGAGCTGCCTGGATCTGACACTGTGTCAGGAGA
SRR765714.23956215.1+     TGGGTGGCACATGGAGAGCCCTCATGGAGAGCTGCCTGGATCTGACACTGTGTCAGGAGA
SRR765714.43208061.2+     TGGGTGGCACATGGAGAGCCCTCATGGAGAGCTGCCTGGATCTGACACTGTGTCAGGAGA
SRR765714.4956404.2-      TGGGTGGCACATGGAGAGCCCTCATGGAGAGCTGCCTGGATCTGACACTGTGTCAGGAGA
SRR765714.63395510.2-     TGGGTGGCACATGGAGAGCCCTCATGGAGAGCTGCCTGGATCTGACACTGTGTCAGGAGA
SRR765714.77066962.1+     TGGGTGGCACATGGAGAGCCCTCATGGAGAGCTGCCTGGATCTGACACTGTGTCAGGAGA
SRR765714.82008612.2-     TGGGTGGCACATGGAGAGCCCTCATGGAGAGCTGCCTGGATCTGACACTGTGTCAGGAGA
SRR765714.86734657.2-     TGGGTGGCACATGGAGAGCCCTCATGGAGAGCTGCCTGGATCTGACACTGTGTCAGGAGA
SRR765715.16262666.2-     TGGGTGGCACATGGAGAGCCCTCATGGAGAGCTGCCTGGATCTGACACTCTGTCAGGAGA
SRR765715.18589469.2-     TGGGTGGCACATGGAGAGCCCTCATGGAGAGCTGCCTGGATCTGACCCTGTGTCAGGAGA
SRR765715.27991329.1-     TGGGTGGCACATGGAGAGCCCTCATGGAGAGCTGCCTGGATCTGACACTGTGTCAGGAGA
SRR765715.48806277.1-     TGGGTGGCACATGGAGAGCCCTCATGGAGAGCTGCCTGGATCTGACACTGTGTCAGGAGA
SRR765716.10147584.2+     TGGGTGGCACATGGAGAGCCCTCATGGAGAGCTGCCTGGATCTGACACTGTGTCACGAGA
SRR765716.13051448.2-     TGTGTGGCACATGGAGAGCCCTCATGGAGAGCTGCCTGGATCTGACACTGTGTCAGGAGA
SRR765716.13424455.2-     TGGGTGGCACATGGAGAGCCCTCATGGAGAGCTGCCTGGATCTGACACTGTGTCAGGAGA
SRR765716.18110791.2+     TGGGTGGCACATGGAGAGCCCTCATGGAGAGCTGCCTGGATCTGACACTGTGTCAGGAGA
SRR765716.27196679.2-     TGGGTGGCACATGGAGAGCCCTCATGGAGAGCTGCCTGGATCTGACACTGTGTCAGGAGA
SRR765716.6716252.2-      TGGGTGGCACATGGAGAGCCCTCATGGAGAGCTGCCTGGATCTGACACTGTGTCAGGAGA
SRR765716.29034131.2-      GGGTGGCACATGGAGAGCCCTCATGGAGAGCTGCCTGGATCTGACACTGTGTCAGGAGA
SRR765712.138777141.1-        TGGCACATGGAGAGCCCTCATGGAGAGCTGCCTGGATCTGACACTGTGTCAGGAGA
SRR765715.30571678.2-         TGGCACATGGAGAGCCCTCATGGAGAGCTGCCTGGATCTGACACTGTGTCAGGAGA
SRR765715.36409904.2-         TGGCACATGGAGAGCCCTCATGGAGAGCTGCCTGGATCTGACACTGTGTCAGCAGA
SRR765711.68524181.1+             ACATGGAGAGCCCTCATGGAGAGCTGCCTGGATCTGACACTGTGTCAGGAGA
SRR765712.64070721.2+             ACATGGAGAGCCCTCATGGAGAGCTGCCTGGATCTGACACTGTGTCAGGAGA
SRR765713.25918438.2-             ACATGGAGAGCCCTCATGGAGAGCTGCCTGGATCTGACACTGTGTCAGGAGA
SRR765712.205681324.2+              ATGGAGAGCCCTCATGGAGAGCTGCCTGGATCTGACACTGTGTCAGGAGA
SRR765713.13238228.2+               ATGGAGAGCCCTCATGGAGAGCTGCCTGGATCTGACACTGTGTCAGGAGA
SRR765714.135078852.2+              ATGGAGAGCCCTCATGGAGAGCTGCCTGGATCTGACACTGTGTCAGGAGA
SRR765712.218218238.1+               TGGAGAGCCCTCATGGAGAGCTGCCTGGATCTGACACTGTGTCAGGAGA
SRR765710.70286255.1-                 GGAGAGCCCTCATGGAGAGCTGCCTGGATCTGACGCTGTGTCAGGAGA
```

```
SRR765712.122158002.1+               GGAGAGCCCTCATGGAGAGCTGCCTGGATCTGACACTGTGTCAGGAGA
SRR765711.131295664.2+                 AGAGCCCTCATGGAGAGCTGCCTGGATCTGACACTGTGTCAGGAGA
SRR765712.185452286.1+                 AGAGCCCTCATGGAGAGCTGCCTGGATCTGACACTGTGTCAGGAGA
SRR765713.23757933.2-                   GAGCCCTCATGGAGAGCTGCCTGGATCTGACACTGTGTCAGGAGA
SRR765713.28931355.2+                   GAGCCCTCATGGAGAGCTGCCTGGATCTGACACTGTGTCAGGAGA
SRR765712.165744054.1-                     CCCTCATGGAGAGCTGCCTGGATCTGACACTGTGTCAGGAGA
SRR765714.167377556.1+                     CCCTCATGGAGAGCTGCCTGGATCTGACACTGTGTCAGGAGA
SRR765710.12300792.1-                       CCTCATGGAGAGCTGCCTGGATCTGACACTGTGTCAGGAGA
SRR765710.75143655.1+                           ATGGAGAGCTGCCTGGATCTGACACTGTGTCAGGAGA
SRR765713.13781601.2+                           ATGGAGAGCTGCCTGGATCTGACACTGTGTCAGGAGA
SRR765713.22750873.1+                           ATGGAGAGCTGCCTGGATCTGACACTGTGTCAGGAGA
SRR765715.16533716.1-                           ATGGAGAGCTGCCTGGATCTGACACTGTGTCAGGAGA
SRR765712.128159580.1-                           TGGAGAGCTGCCTGGATCTGACACTGTGTCAGGAGA
SRR765712.99870371.2-                            TGGAGAGCTGCCTGGATCTGACACTGTGTCAGGAGA
SRR765714.2480924.2+                             TGGAGAGCTGCCTGGATCTGACACTGTGTCAGGAGA
SRR765714.40452062.2+                            TGGAGAGCTGCCTGGATCTGACACTGTGTCAGGAGA
SRR765709.7414701.1+                              GGAGAGCTGCCTGGATCTGACACTGTGTCAGGAGA
SRR765712.158301577.2-                             GAGAGCTGCCTGGATCTGACACTGTGTCAGGAGA
SRR765712.183278959.2-                              AGAGCTGCCTGGATCTGACACTGTGTCAGGAGA
SRR765712.214292832.1+                              AGAGCTGCCTGGATCTGACACTGTGTCAGGAGA
SRR765710.73677951.1-                                GAGCTGCCTGGATCTGACACTGTGTCAGGAGA
SRR765712.43404440.2-                                  GCTGCCTGGATCTGACACTGTGTCAGGAGA
SRR765712.51340874.1-                                    TGCCTGGATCTGACACTGTGTCAGGAGA
SRR765709.21897156.2-                                     GCCTGGATCTGACACTGTGTCAGGAGA
SRR765711.13388429.1+                                     GCCTGGATCTGACACTGTGTCAGGAGA
SRR765712.121630012.1-                                     CCTGGATCTGACACTGTGTCAGGAGA
SRR765711.74112939.1+                                       CTGGATCTGACACTGTGTCAGGAGA
SRR765712.72190442.1-                                       CTGGATCTGACACTGTGTCAGGAGA
SRR765712.171142138.2-                                             TGACACTGTGTCAGGAGA
SRR765709.26128387.1-                                                ACACTGTGTCAGGAGA
SRR765711.132150187.1+                                               ACACTGTGTCAGGAGA
SRR765711.79664446.1-                                                 CACTGTGTCAGGAGA
SRR765712.85106693.2-                                                 CACTGTGTCAGGAGA
SRR765710.27338352.1-                                                   CTGTGTCAGGAGA
SRR765712.122158002.2-                                                  CTGTGTCAGGAGA
SRR765709.62253164.1+                                                    TGTGTCAGGAGA
SRR765714.186542268.1-                                                   TGTGTCAGGAGA
SRR765711.33485371.2+                                                     GTGTCAGGAGA
SRR765714.151210991.2-                                                    GTGTCAGGAGA
SRR765712.36675453.1+                                                       GTCAGGAGA
SRR765712.217980781.2+                                                       TCAGGAGA
SRR765712.66526516.1+                                                        TCAGGAGA
SRR765715.38198709.1-                                                        TCAGGAGA
SRR765709.56584448.2+                                                         CAGGAGA
SRR765711.23637391.2+                                                         CAGGAGA
SRR765714.129672082.1-                                                         AGGAGA

consensus                TGGGTGGCACATGGAGAGCCCTCATGGAGAGCTGCCTGGATCTGACACTGTGTCAGGAGA

                             .    :    .    :    .    :    .    :    .    :    .    :
SRR765710.27338352.2+    T
SRR765711.13566352.1+    T
SRR765712.51340874.2+    T
SRR765712.72190442.2+    T
SRR765712.76576490.2-    TG
SRR765711.39834426.1-    TGC
SRR765711.79664446.2+    TGC
SRR747825.12487751.2-    TGCCT
SRR747825.12965830.2-    TGCCT
SRR747825.14196148.2-    TGCCT
SRR747825.17726022.2-    TGCCT
SRR747825.21410409.2-    TGCCT
SRR747825.26095160.2-    TGCCT
SRR747825.26346030.2-    TGCCT
SRR747825.35226945.2-    TGCCT
SRR747825.36267467.2-    TGCCT
SRR747825.42525078.2-    TGCCT
SRR747825.4608608.2-     TGCCT
SRR747825.7988087.2-     TGCCT
SRR747825.8350873.2-     TGCCT
SRR765709.3017797.1-     TGCCT
SRR765714.63395510.2-    TGCCT
SRR765714.82008612.2-    TGCCT
SRR765710.11110602.2-    TGCCTA
SRR765711.6290287.2+     TGCCTAC
SRR765712.197777944.1+   TGCCTAC
SRR765710.78316546.2-    TGCCTACC
SRR765709.13217291.1+    TGCCTACCG
SRR765715.16262666.2-    TGCCTACCG
SRR765715.18589469.2-    TGCCTACCG
SRR765716.10147584.2+    TGCCTACCGG
SRR765716.18110791.2+    TGCCTACCGG
SRR765709.13162642.1-    TGCCTACCGGA
SRR765710.41723497.1+    TGCCTACCGGAG
```

SRR765714.77066962.1+ TGCCTACCGGAG
SRR765714.181917353.1- TGCCTACCGGAGT
SRR765711.31552385.2- TGCCTACCGGAGTG
SRR765711.35892352.2+ TGCCTACCGGAGTG
SRR765714.23956215.1+ TGCCTACCGGAGTG
SRR765710.66135762.1- TGCCTACCGGAGTGGC
SRR765712.88008628.2- TGCCTACCGGAGTGGCA
SRR765714.181600550.1- TGCCTACCGGAGTGGCA
SRR765711.102739620.2+ TGCCTACCGGAGTGGCAG
SRR765712.148429673.1+ TGCCTACCGGAGTGGCAGC
SRR765714.178949095.1- TGCCTACCGGAGTGGCAGC
SRR765711.56339811.1+ TGCCTACCGGAGTGGCAGCAT
SRR765709.40884677.1+ TGCCTACCGGAGTGGCAGCATC
SRR765712.151632343.1+ TGCCTACCGGAGTGGCAGCATC
SRR765712.25672489.2- TGCCTACCGGAGTGGCAGCATCT
SRR765712.220295908.2+ TGCCTACCGGAGTGGCAGCATCTG
SRR765712.27955158.2- TGCCTACCGGAGTGGCAGCATCTG
SRR765713.8355185.1+ TGCCTACCGGAGTGGCAGCATCTGC
SRR765710.10250671.1+ TGCCTACCGGAGTGGCAGCATCTGCTTC
SRR765710.6584671.2+ TGCCTACCGGAGTGGCAGCATCTGCTTCC
SRR765714.86734657.2- TGCCTACCGGAGTGGCAGCATCTGCTTCC
SRR765709.62403711.1+ TGCCTACCGGAGTGGCAGCATCTGCCTCCC
SRR765712.125893357.2- TGCCTACCGGAGTGGCAGCATCTGCTTCCCT
SRR765714.195543010.2- TGCCTACCGGAGTGGCAGCATCTGCTTCCCT
SRR765714.43208061.2+ TGCCTACCGGAGTGGCAGCATCTGCTTCCCT
SRR765710.14715271.2- TGCCTACCGGAGTGGCAGCATCTGCTTCCCTg
SRR765714.102557296.2- TGCCTACCGGAGTGGCAGCATCTGCTTCCCTg
SRR765714.111689744.2- TGCCTACCGGAGTGGCAGCATCTGCTTCCCTg
SRR765715.27991329.1- TGCCTACCGGAGTGGCAGCATCTGCTTCCCTg
SRR765715.48806277.1- TGCCTACCGGAGTGGCAGCATCTGCTTCCCTg
SRR765711.42145081.1- TGCCTACCGGAGTGGCAGCATCTGCTTCCCTgt
SRR765712.136103372.1- TGCCTACCGGAGTGGCAGCATCTGCTTCCCTgt
SRR765709.44632583.1- TGCCTACCGGAGTGGCAGCATCTGCTTCCCTgta
SRR765712.121630012.2+ TGCCTACCGGAGTGGCAGCATCTGCTTCCCTgta
SRR765712.189246974.2+ TGCCTACCGGAGTGGCAGCATCTGCTTCCCTgta
SRR765716.13051448.2- TGCCTACCGGAGTGGCAGCATCTGCTTCCCTgtaa
SRR765716.13424455.2- TGCCTACCGGATTGGCAGCATCTGCTTCCCTgtaa
SRR765716.27196679.2- TGCCTACCGGAGTGGCAGCATCTGCTTCCCTgtaa
SRR765716.29034131.2- TGCCTACCGGAGTGGCAGCATCTGCTTCCCTgtaa
SRR765716.6716252.2- TGCCTACCGGAGTGGCAGCATCTGCTTCCCTgtaa
SRR765714.4956404.2- TGCCTACCGGAGTGGCAGCATCTGCTTCCCTgtaag
SRR765711.55682203.2+ TGCCTACCGGAGTGGCAGCATCTGCTTCCCTgtaagc
SRR765709.15951076.2+ TGCCTACCGGAGTGGCAGCATCTGCTTCCCTgtaagct
SRR765709.87544394.2- TGCGTACCGGAGTGGCAGCCTCTGCTTCCCTgtaagct
SRR765712.173925922.2- TGCCTACCGGAGTGGCAGCATCTGCTTCCCTgtaagctg
SRR765709.44907493.2+ TGCCTACCGGAGTGGCAGCATCTGCTTCCCTgtaagctgaa
SRR765712.202510854.2+ TGCCTACCGGAGTGGCAGCATCTGCTTCCCTgtaagctgaa
SRR765712.138777141.1- TGCCTACCGGAGTGGCAGCATCTGCTTCCCTgtaagctgaacaga
SRR765715.30571678.2- TGCCTACCGGAGTGGCAGCATCTGCTTCCCTgtaagctgaacaga
SRR765715.36409904.2- TGCCTACCGGAGTGGCAGCAACTGCTTCCCTgtaagctgaacaga
SRR765713.25918438.2- TGCCTACCGGAGTGGCAGCATCTGCTTCCCTgtaagctgaacagattt
SRR765711.68524181.1+ TGCCTACCGGAGTGGCAGCATCTGCTTCCCTgtaagctgaacagatttc
SRR765712.64070721.2+ TGCCTACCGGAGTGGCAGCATCTGCTTCCCTgtaagctgaacagatttc
SRR765713.13238228.2+ TGCCTACCGGAGTGGCAGCATCTGCTTCCCTgtaagctgaacagatttcc
SRR765714.135078852.2+ TGCCTACCAGAGTGGCAGCATCTGCTTCCCTgtaagctgaacagatttcc
SRR765712.205681324.2+ TGCCTACCGGAGTGGCAGCATCTGCTTCCCTgtaagctgaacagatttcct
SRR765712.218218238.1+ TGCCTACCGGAGTGGCAGCATCTGCTTCCCTgtaagctgaacagatttcctc
SRR765710.70286255.1- TGCCTACCGGAGTGGCAGCATCTGCTTCCCTgtaagctgaacagatttcctct
SRR765712.122158002.1+ TGCCTACCGGAGTGGCAGCATCTGCTTCCCTgtaagctgaacagatttcctct
SRR765711.131295664.2+ TGCCTACCGGAGTGGCAGCATCTGCTTCCCTgtaagctgaacagatttcctcttc
SRR765712.185452286.1+ TGCCTACCGGAGTGGCAGCATCTGCTTCCCTgtaagctgaacagatttcctcttc
SRR765713.23757933.2- TGCCTACCGGAGTGGCAGCATCTGCTTCCCTgtaagctgaacagatttcctcttc
SRR765713.28931355.2+ TGCCTACCGGCGTGGCAGCATCTGCTTCCCTgtaagctgaacagatttcctcttc
SRR765714.167377556.1+ TGCCTACCGGAGTGGCAGCATCTGCTTCCCTgtaagctgaacagatttcctcttcaac
SRR765712.165744054.1- TGCCTACCGGAGTGGCAGCATCTGCTTCCCTgttagctgaacagatttcctcttcaacc
SRR765709.21897156.2- TGCCTACCGGAGTGGCAGCATCTGCTTCCCTgtaagctgaacagatttcctcttcaaccc
SRR765709.26128387.1- TGCCTACCGGAGTGGCAGCATCTGCTTCCCTgtaagctgaacagatttcctcttcaaccc
SRR765709.56584448.2+ TGCCTACCGGAGTGGCAGCATCTGCTTCCCTgtaagctgaacagagttcctcttcaaccc
SRR765709.62253164.1+ TGCCTACCGGAGTGGCAGCATCTGCTTCCCTgtaagctgaacagatttcctcttcaaccc
SRR765709.7414701.1+ TGCCTACCGGAGTGGCAGCATCTGCTTCCCTgtaagctgaacagatttcctcttcaaccc
SRR765710.12300792.1- TGCCTACCGGAGTGGCAGCATCTGCTTCCCTgtaagctgaacagatttcctcttcaaccc
SRR765710.27338352.1- TGCCTACCGGAGTGGCAGCATCTGCTTCCCTgtaagctgaacagatttcctcttcaaccc
SRR765710.73677951.1- TGCCTACCGGAGTGGCAGCATCTGCTTCCCTgtaagctgaacagatttcctcttcaaccc
SRR765710.75143655.1+ TGCCTACCGGAGTGGCAGCATCTGCTTCCCTgtaagctgaacagatttcctcttcaaccc
SRR765711.132150187.1+ TGCCTACCGGAGTGGCAGCATCTGCTTCCCTgtaagctgaacagatttcctcttcaaccc
SRR765711.13388429.1+ TGCCTACCGGAGTGGCAGCATCTGCTTCCCTgtaagctgaacagatttcctcttcaaccc
SRR765711.23637391.2+ TGCCTACCGGAGTGGCAGCATCTGCTTCCCTgtaagctgaacagatttcctcttcaaccc
SRR765711.33485371.2+ TGCCTACCGGAGTGGCAGCATCTGCTTCCCTgtaagctgaacagatttcctcttcaaccc
SRR765711.74112939.1+ TGCCTACCGGAGTGGCAGCATCTGCTTCCCTgtaagctgaacagatttcctcttcaaccc
SRR765711.79664446.1- TGCCTACCGGAGTGGCAGCATCTGCTTCCCTgtaagctgaacagatttcctcttcaaccc
SRR765712.121630012.1- TGCCTACCGGAGTGGCAGCATCTGCTTCCCTgtaagctgaacagatttcctcttcaaccc
SRR765712.122158002.2- TGCCTACCGGAGTGGCAGCATCTGCTTCCCTgtaagctgaacagatttcctcttcaaccc
SRR765712.128159580.1- TGCCTACCGGAGTGGCAGCATCTGCTTCCCTgtaagctgaacagaattcctcttcaaccc
SRR765712.158301577.2- TGCCTACCGGAGTGGCAGCATCTGCTTCCCTgtaagctgaacagatttcctcttcaaccc

```
SRR765712.171142138.2-   TGCCTACCGGAGTGGCAGCATCTGCTTCCCTgtaagctgaacagatttcctcttcaaccc
SRR765712.183278959.2-   TGCCTACCGGAGTGGCAGCATCTGCTTCCCTgtaagctgaacagatttcctcttcaaccc
SRR765712.214292832.1+   TGCCTACCGGAGTGGCAGCATCTGCTTCCCTgtaagctgaacagatttcctcttcaaccc
SRR765712.217980781.2+   TGCCTACCGGAGTGGCAGCATCTGCCTCCCTgtaagctgaacagatttcctcttcaaccc
SRR765712.36675453.1+    TGCCTACCGGAGTGGCAGCATCTGCTTCCCTgtaagctgaacagatttcctcttcaaccc
SRR765712.43404440.2-    TGCCTACCGGAGTGGCAGCATCTGCTTCCCTgtaagctgaacagatttcctcttcaaccc
SRR765712.51340874.1-    TGCCTACCGGAGTGGCAGCATCTGCTTCCCTgtaagctgaacagatttcctcttcaaccc
SRR765712.66526516.1+    TGCCTACCGGAGTGGCAGCATCTGCTTCCCTgtaagctgaacagatttcctcttcaaccc
SRR765712.72190442.1-    TGCCTACCGGAGTGGCAGCATCTGCTTCCCTgtaagctgaacagatttcctcttcaaccc
SRR765712.85106693.2-    TGCCTACCGGAGTGGCAGCATCTGCTTCCCTgtaagctgaacagatttcctcttcaaccc
SRR765712.99870371.2-    TGCCTACCGGAGTGCCAGCATCTGCTTCCCTgtaagctgaacagatttcctcttcaaccc
SRR765713.13781601.2+    TGCCTACCGGAGTGGCAGCATCTGCTTCCCTgtaagctgaacagatttcctcttcaaccc
SRR765713.22750873.1+    TGCCTACCGGAGTGGCAGCATCTGCTTCCCTgtaagctgaacagatttcctcttcaaccc
SRR765714.129672082.1-   TGCCTACCGGAGTGGCAGCATCTGCTTCCCTgtaagctgaacagatttcctcttcaaccc
SRR765714.151210991.2-   TGCCTACCGGAGTGGCAGCATCTGCTTCCCTgtaagctgaacagatttcctcttcaaccc
SRR765714.186542268.1-   TGCCTACCGGAGTGGCAGCATCTGCTTCCCTctacgctgaacagatttcctcttcaaccc
SRR765714.2480924.2+     TGCCTACCGGAGTGGCAGCATCTGCTTCCCTgtaagctgaacagatttcctcttcaaccc
SRR765714.40452062.2+    TGCCTACCGGAGTGGCAGCATCTGCTTCCCTgtaagctgaacagatttcctcttcaaccc
SRR765715.16533716.1-    TGCCTACCGGAGTGGCAGCATCTGCTTCCCTgtaagctgaacagatttcctcttcaaccc
SRR765715.38198709.1-    TGCCTACCGGAGTGGCAGCATCTGCTTCCCTgtaagctgaacagatttcctcttcaaccc

consensus                TGCCTACCGGAGTGGCAGCATCTGCTTCCCTgtaagctgaacagatttcctcttcaaccc
```

E. Collared flycatcher (No. 6), exon 1, WGS data

- CAP3 alignment of WGS data spanning exon 1 (uppercase letters)

```
                             .    :    .    :    .    :    .    :    .    :    .    :
ERR117212.6476186.2-     cccaaggtatcagcaggactcagagactgcagagATGATGGTTTTGTTCTTCTCAGGAAT
ERR117201.2485870.1-                         cagagactgcagcgATGATGGTTTTGTTCTTCTCAGGAAT
ERR117191.3942459.1+                            agactgcagagATGATGGTTTTGTTCTTCTCAGGAAT
ERR117214.14172132.2+                             ctgcagagATGATGGTTTTGTTCTTCTCAGGAAT
ERR117209.2234770.2+                                 gcagagATGATGGTTTTGTTCTTCTCAGGAAT
ERR117172.15932939.1-                                    agATGATGGTTTTGTTCTTCTCAGGAAT
ERR117162.17731664.1-                                      ATGATGGTTTTGTTCTTCTCAGGAAT
ERR117162.20390690.1-                                      ATGATGGTTTTGTTCTTCTCAGGAAT
ERR117162.15239877.1-                                       TGATTGTTTTGTTCTTCTCAGGAAT
ERR117162.24743150.1+                                         ATGGTTTTGTTCTTCTCAGGAAT
ERR117187.1846104.1+                                          ATGGTTTTGTTCTTCTCAGGAAT
ERR117195.8720933.1-                                             GTTTTGTTCTTCTCAGGAAT
ERR117195.13585097.1-                                             TTTTGTTCTTCTCAGGAAT
ERR117212.8693972.1-                                              TTTTGTGCTTCTCAGGAAT
ERR117213.20630415.2-                                                TGTTCTTCTCAGGAAT
ERR117188.6260249.1-                                                  GTTCTTCTCAGGAAT
ERR117189.3627525.1+                                                  GTTCTTCTCAGGAAT
ERR117167.13563954.1+                                                       TCTCAGGAAT
ERR117184.14295738.2-                                                       TCTCAGTAAT
ERR117211.10510244.1+                                                         TCAGGAAT
ERR117203.460754.1-                                                            CAGGAAT
ERR117214.14172132.1-                                                           AGGAAT
ERR117190.4710130.1-                                                              GAAT
ERR117186.15182709.1-                                                                T
ERR117186.19827414.1-                                                                T

consensus                cccaaggtatcagcaggactcagagactgcagagATGATGGTTTTGTTCTTCTCAGGAAT

                             .    :    .    :    .    :    .    :    .    :    .    :
ERR117212.6476186.2-     CAAGAAAATGCTCTTCCTCTAGTTCCTCTC
ERR117153.26125281.2+    CAAGAAAATGCTCTTCCTCTAGTTCCTCTCATGCTTTTGTTCTGGCCTGCTTGCATCTCC
ERR117154.46542049.2+    CAAGAAAATGCTCTTCCTCTAGTTCCTCTCATGCTTTTGTTCTGGCCTGCTTGCATCTCC
ERR117154.7676116.2+     CAAGAAAATGCTCTTCCTCTAGTTCCTCTCATGCTTTTGTTCTGGCCTGCTTGCATCTCC
ERR117155.48606278.2+    CAAGAAAATGCTCTTCCTCTAGTTCCTCTCATGCTTTTGTTCTGGCCTGCTTGCATCTCC
ERR117162.15239877.1-    CAAGAAAATGCTCTTCCTCTAGTTCCTCTCATGCTTTTGTTCTGGCCTGCTTGCATCTCC
ERR117162.17731664.1-    CAAGAAAATGCTCTTCCTCTAGTTCCTCTCATGCTTTTGTTCTGGCCTGCTTGCATCTCC
ERR117162.20390690.1-    CAAGAAAATGCTCTTCCTCTAGTTCCTCTCATGCTTTTGTTCTGGCCTGCTTGCATCTCC
ERR117162.24743150.1+    CAAGAAAATGCTCTTCCTCTAGTTCCTCTCATGCTTTTGTTCTGGCCTGCTTGCATCTCC
ERR117167.13563954.1+    CAAGAAAATGCTCTTCCTCTAGTTCCTCTCATGCTTTTGTTCTGGCCTGCTTGCATCTCC
ERR117169.908271.1-      CAAGAAAATGCTCTTCCTCTAGTTCCTCTCATGCTTTTGTTCTGGCCTGCTTGCATCTCC
ERR117172.15932939.1-    CAAGAAAATGCTCTTCCTCTAGTTCCTCTCATGCTTTTGTTCTGGCCTGCTTGCATCTCC
ERR117184.14295738.2-    CAAGAAAATGCTCTTCCTCTAGTTCCTCTCATGCTTTTGTTCTGGCCTGCTTGCATCTCC
ERR117186.15182709.1-    CAAGAAAATGCTCTTCCTCTAGTTCCTCTCATGCTTTTGTTCTGGCCTGCTTGCATCTCC
ERR117186.19827414.1-    CAAGAAAATGCTCTTCCTCTAGTTCCTCTCATGCTTTTGTTCTGGCCTGCTTGCATCTCC
ERR117187.1846104.1+     CAAGAAAATGCTCTTCCTCTAGTTCCTCTCATGCTTTTGTTCTGGCCTGCTTGCATCTCC
ERR117188.6260249.1-     CAAGAAAATGCTCTTCCTCTAGTTCCTCTCATGCTTTTGTTCTGGCCTGCTTGCATCTCC
ERR117189.3627525.1+     CAAGAAAATGCTCTTCCTCTAGTTCCTCTCATGCTTTTGTTCTGGCCTGCTTGCATCTCC
ERR117190.4710130.1-     CAAGAAAATGCTCTTCCTCTAGTCCCTCTCATGCTTTTGTTCTGGCCTGCTTGCATCTCC
ERR117191.3942459.1+     CAAGAAAATGCTCTTCCTCTAGTTCCTCTCATGCTTTTGTTCTGGCCTGCTTGCATCTCC
ERR117195.13585097.1-    CAAGAAAATGCTCTTCCTCTAGTTCCTCTCATGCTTTTGTTCTGGCCTGCTTGCATCTCC
ERR117195.8720933.1-     CAAGAAAATGCTCTTCCTCTAGTTCCTCTCATGCTTTTGTTCTGGCCTGCTTGCATCTCC
ERR117201.2485870.1-     CAAGAAAATGCTCTTCCTCTAGTTCCTCTCATGCTTTTGTTCTGGCCTGCTTGCATCTCC
ERR117203.460754.1-      CAAGAAAATGCTCTTCCTCTAGTTCCTCTCATGCTTTTGTTCTGGCCTGCTTGCATCTCC
ERR117209.2234770.2+     CAAGAAAATGCTCTTCCTCTAGTTCCTCTCATGCTTTTGTTCTGGCCTGCTTGCATCTCC
ERR117211.10510244.1+    CAAGAAAATGCTCTTCCTCTAGTTCCTCTCATGCTTTTGTTCTGGCCTGCTTGCATCTCC
ERR117212.8693972.1-     CAAGAAAATGCTCTTCCTCTAGTTCCTCGCATGCTTTTGTTCTGGCCTGCTTGCATCTCC
ERR117213.20630415.2-    CAAGAAAATTCTCTTCCTCTAGTTCCTCTCATGCTTTTGTTCTGGCCTGCTTGCATCTCC
ERR117214.14172132.1-    CAAGAAAATGCTCTTCCTCTAGTTCCTCTCATGCTTTTGTTCTGGCCTGCTTGCATCTCC
ERR117214.14172132.2+    CAAGAAAATGCTCTTCCTCTAGTTCCTCTCATGCTTTTGTTCTGGCCTGCTTGCATCTCC
ERR117153.25248628.2+     AAGAAAATGCTCTTCCTCTAGTTCCTCTCATGCTTTTGTTCTGGCCTGCTTGCATCTCC
ERR117153.28715906.2+     AAGAAAATGCTCTTCCTCTAGTTCCTCTCATGCTTTTGTTCTGGCCTGCTTGCATCTCC
ERR117153.39929989.2+     AAGAAAATGCTCTTCCTCTAGTTCCTCTCATGCTTTTGTTCTGGCCTGCTTGCATCTCC
ERR117153.50977464.2+     AAGAAAATGCTCTTCCTCTAGTTCCTCTCATGCTTTTGTTCTGGCCTGCTTGCATCTCC
ERR117153.52435271.1+     AAGAAAATGCTCTTCCTCTAGTTCCTCTCATGCTTTTGTTCTGGCCTGCTTGCATCTCC
ERR117153.9559693.1+      AAGAAAATGCTCTTCCTCTAGTTCCTCTCATGCTTTTGTTCTGGCCTGCTTGCATCTCC
ERR117154.24132143.2+     AAGAAAATGCTCTTCCTCTAGTTCCTCTCATGCTTTTGTTCTGGCCTGCTTGCATCTCC
ERR117154.28182887.1+     AAGAAAATGCTCTTCCTCTAGTTCCTCTCATGCTTTTGTTCTGGCCTGCTTGCATCTCC
ERR117154.32244393.2+     AAGAAAATGCTCTTCCTCTAGTTCCTCTCATGCTTTTGTTCTGGCCTGCTTGCATCTCC
ERR117154.51549766.2+     AAGAAAATGCTCTTCCTCTAGTTCCTCTCATGCTTTTGTTCTGGCCTGCTTGCATCTCC
ERR117154.52572361.2+     AAGAAAATGCTCTTCCTCTAGTTCCTCTCATGCTTTTGTTCTGGCCTGCTTGCATCTCC
ERR117154.53521277.1+     AAGAAAATGCTCTTCCTCTAGTTCCTCTCATGCTTTTGTTCTGGCCTGCTTGCATCTCC
ERR117154.55843371.2+     AAGAAAATGCTCTTCCTCTAGTTCCTCTCATGCTTTTGTTCTGGCCTGCTTGCATCTCC
ERR117155.1676696.1+      AAGAAAATGCTCTTCCTCTAGTTCCTCTCATGCTTTTGTTCTGGCCTGCTTGCATCTCC
ERR117155.24901097.2+     AAGAAAATGCTCTTCCTCTAGTTCCTCTCATGCTTTTGTTCTGGCCTGCTTGCATCTCC
ERR117155.27398178.1+     AAGAAAATGCTCTTCCTCTAGTTCCTCTCATGCTTTTGTTCTGGCCTGCTTGCATCTCC
```

```
ERR117155.30544527.2+     AAGAAAATGCTCTTCCTCTAGTTCCTCTCATGCTTTTGTTCTGGCCTGCTTGCATCTCC
ERR117155.32031392.1+     AAGAAAATGCTCTTCCTCTAGTTCCTCTCATGCTTTTGTTCTGGCCTGCTTGCATCTCC
ERR117155.35114720.1+     AAGAAAATGCTCTTCCTCTAGTTCCTCTCATGCTTTTGTTCTGGCCTGCTTGCATCTCC
ERR117155.56017163.2+     AAGAAAATGCTCTTCCTCTAGTTCCTCTCATGCTTTTGTTCTGGCCTGCTTGCATCTCC
ERR117155.58927721.1+     AAGAAAATGCTCTTCCTCTAGTTCCTCTCATGCTTTTGTTCTGGCCTGCTTGCATCTCC
ERR117155.5975449.2+      AAGAAAATGCTCTTCCTCTAGTTCCTCTCATGCTTTTGTTCTGGCCTGCTTGCATCTCC
ERR117158.24629168.2-     AAGAAAATTCTCTTCCTCTAGTTCCTCTCATGCTTTTGTTCTGGCCTGCTTGCATCTCC
ERR117168.6760065.1+       AGAAAATGCTCTTCCTCTAGTTCCTCTCATGCTTTTGTTCTGGCCTGCTTGCATCTCC
ERR117185.11582461.2-      AGAAAATGCTCTTCCTCTAGTTCCTCTCATGCTTTTGTTCTGGCCTGCTTGCATCTCC
ERR117187.8211208.1-       AGAAAATGCTCTTCCTCTAGTTCCTCTCATGCTTTTGTTCTGGCCTGCTTGCATCTCC
ERR117189.16427324.1+       GAAAATGCTCTTCCTCTAGTTCCTCTCATGCTTTTGTTCTGGCCTGCTTGCATCTCC
ERR117189.16427324.2-       GAAAATGCTCTTCCTCTAGTTCCTCTCATGCTTTTGTTCTGGCCTGCTTGCATCTCC
ERR117158.4154112.1+          AAATGCTCTTCCTCTAGTTCCTCTCATGCTTTTGTTCTGGCCTGCTTGCATCTCC
ERR117160.9073880.1-          AAATGCTCTTCCTCTAGTTCCTCTCATGCTTTTGTTCTGGCCTGCTTGCATCTCC
ERR117162.29804872.1-          AATGCTCTTCCTCTAGTTCCTCTCGTGCTTTTGTTCTGGCCTGCTTGCATCTCC
ERR117170.149546.1-            AATGCTCTTCCTCTAGTTCCTCTCATGCTTTTGTTCTGGCCTGCTTGCATCTCC
ERR117186.14041801.1+          AATGCTCTTCCTCTAGTTCCTCTCATGCTTTTGTTCTGGCCTGCTTGCATCTCC
ERR117195.13433153.1+          AATGCTCTTCCTCTAGTTCCTCTCATGCTTTTGTTCTGGCCTGCTTGCATCTCC
ERR117195.19866522.1+          AATGCTCTTCCTCTAGTTCCTCTCATGCTTTTGTTCTGGCCTGCTTGCATCTCC
ERR117195.20596627.1+          AATGCTCTTCCTCTAGTTCCTCTCATGCTTTTGTTCTGGCCTGCTTGCATCTCC
ERR117171.21382053.1+           ATGCTCTTCCTCTAGTTCCTCTCATGCTTTTGTTCTGGCCTGCTTGCATCTCC
ERR117187.13695262.1-            TGCTCTTCCTCTAGTTCCTCTCATGCTTTTGTTCTGGCCTGCTTGCATCTCC
ERR117190.26390213.1-            TGCTCTTCCTCTAGTTCCTCTCATGCTTTTGTTCTGGCCTGCTTGCATCTCC
ERR117168.17100909.1-             GCTCTTCCTCTAGTTCCTCTCATGCTTTTGTTCTGGCCTGCTTGCATCTCC
ERR117172.12496667.1-             GCTCTTCCTCTAGTTCCTCTCATGCTTTTGTTCTGGCCTGCTTGCATCTCC
ERR117189.17247817.1+             GCTCTTCCTCTAGTTCCTCTCATGCTTTTGTTCTGGCCTGCTTGCATCTCC
ERR117195.13729654.1+             GCTCTTCCTCTAGTTCCTCTCATGCTTTTGTTCTGGCCTGCTTGCATCTCC
ERR117195.13884831.1+             GCTCTTCCTCTAGTTCCTCTCATGCTTTTGTTCTGGCCTGCTTGCATCTCC
ERR117211.11635529.1+              CTCTTCCTCTAGTTCCTCTCATGCTTTTGTTCTGGCCTGCTTGCATCTCC
ERR117213.10610064.2+               TCTTCCTCTAGTTCCTCTCATGCTTTTGTTCTGGCCTGCTTGCATCTCC
ERR117173.232780.1+                   TTCCTCTAGTTCCTCTCATGCTTTTGTTCTGGCCTGCTTGCATCTCC
ERR117172.1851309.1+                    CCTCTAGTTCCTCTCATGCTTTTGTTCTGGCCTGCTTGCATCTCC
ERR117207.3319922.1-                    CCTCTAGTTCCTCTCATGCTTTTGTGCTGGCCTGCTTGCATCTCC
ERR117190.38583922.2+                     TCTAGTTCCTCTCATGCTTTTGTTCTGGCCTGCTTGCATCTCC
ERR117173.12302743.2+                        AGTTCCTCTCATGCTTTTGTTCTGGCCTGCTTGCATCTCC
ERR117170.12478522.2+                         GTTCCTCTCATGCTTTTGTTCTGGCCTGCTTGCATCTCC
ERR117189.19092939.2+                          TTCCTCTCATGCTTTTGTTCTGGCCTGCTTGCATCTCC
ERR117190.29386046.1+                           TCCTCTCATGCTTTTGTTCTGGCCTGCTTGCATCTCC
ERR117195.6901054.1-                              CTCTCACGCTTTTGTTCTGGCCTGCTTGCATCTCC
ERR117167.544228.1+                                 CTCATGCTTTTGTTCTGGCCTGCTTGCATCTCC
ERR117186.4205347.1-                                CTCATGCTTTTGTTCTGGCCTGCTTGCATCTCC
ERR117195.5115980.1-                                 TCATGCTTTTCTGCTGGCCTGCTTGCATCTCC
ERR117195.6900905.1-                                 TCATGCTTTTTTTCGGGCCTGCTTGCATCTCC
ERR117170.11495570.1+                                 CATGCTTTTGTTCTGGCCTGCTTGCATCTCC
ERR117173.1405665.2-                                   ATGCTTTTGTGCTGGCCTGCTTGCATCTCC
ERR117186.21463358.2+                                  ATGCTTTTGTTCTGGCCTGCTTGCATCTCC
ERR117160.6004183.1+                                     GCTTTTGTTCTGGCCTGCTTGCATCTCC
ERR117156.1297884.2+                                        TTTGTTCTGGCCTGCTTGCATCTCC
ERR117156.16352577.2+                                       TTTGTTCTGGCCTGCTTGCATCTCC
ERR117157.38560318.2+                                       TTTGTTCTGGCCTGCTTGCATCTCC
ERR117158.9230335.2+                                        TTTGTTCTGGCCTGCTTGCATCTCC
ERR117188.5462950.1-                                         TTGTTCTGGCCTGCTTGCATCTCC
ERR117188.5463136.1-                                         TTGTTCTGGCCTGCTTGCATCTCC
ERR117195.16676120.2-                                         TGTTCTGGCCTGCTTGCATCTCC
ERR117211.10960067.1+                                         TGTTCTGGCCTGCTTGCATCTCC
ERR117167.12360378.1+                                            TCTGGCCTGCTTGCATCTCC
ERR117170.1901259.2+                                             TCTGGCCTGCTTGCATCTCC
ERR117170.6017029.1-                                             TCTGGCCTGCTTGCATCTCC
ERR117170.7975740.2+                                               TGGCCTGCTTGCATCTCC
ERR117190.31524659.2+                                              TGGCCTGCTTGCATCTCC
ERR117162.21063772.1-                                                 CCTGCTTGCATCTCC
ERR117156.12339954.1+                                                  CTGCTTGCATCTCC
ERR117156.14079381.1+                                                  CTGCTTGCATCTCC
ERR117156.15679746.1+                                                  CTGCTTGCATCTCC
ERR117156.1899448.1+                                                   CTGCTTGCATCTCC
ERR117156.27998490.1+                                                  CTGCTTGCATCTCC
ERR117156.384374.1+                                                    CTGCTTGCATCTCC
ERR117156.412636.1+                                                    CTGCTTGCATCTCC
ERR117156.51082258.1+                                                  CTGCTTGCATCTCC
ERR117156.55492752.1+                                                  CTGCTTGCATCTCC
ERR117156.686079.1+                                                    CTGCTTGCATCTCC
ERR117157.10175429.1+                                                  CTGCTTGCATCTCC
ERR117157.21083951.1+                                                  CTGCTTGCATCTCC
ERR117157.35348797.1+                                                  CTGCTTGCATCTCC
ERR117157.47818519.1+                                                  CTGCTTGCATCTCC
ERR117157.48178658.1+                                                  CTGCTTGCATCTCC
ERR117157.48288776.1+                                                  CTGCTTGCATCTCC
ERR117157.52982400.1+                                                  CTGCTTGCATCTCC
ERR117157.6127110.1+                                                   CTGCTTGCATCTCC
ERR117162.19230106.1+                                                  CTGCTTGCATCTCC
ERR117172.10868533.2-                                                   TGCTTGCATCTCC
ERR117186.6868637.1+                                                     GCTTGCATCTCC
ERR117168.8755584.1-                                                         TGCATCTCC
ERR117169.2607605.1-                                                              TCC
```

```
ERR117195.20988009.2-                                                             TCC
ERR117172.19560183.2-                                                              CC
ERR117186.13986380.1-                                                              CC
ERR117187.9292650.1-                                                               CC
ERR117194.922317.2-                                                                 C

consensus                CAAGAAAATGCTCTTCCTCTAGTTCCTCTCATGCTTTTGTTCTGGCCTGCTTGCATCTCC

                             .    :    .    :    .    :    .    :    .    :    .    :
ERR117187.1846104.1+     CCTGCTGCGTTTCCCCA
ERR117209.2234770.2+     CCTGCTGCGTTTCCCCACC
ERR117153.26125281.2+    CCTGCTGCGTTTCCCCACCT
ERR117154.46542049.2+    CCTGCTGCGTTTCCCCACCT
ERR117154.7676116.2+     CCTGCTGCGTTTCCCCACCT
ERR117155.48606278.2+    CCTGCTGCGTTTCCCCACCT
ERR117153.25248628.2+    CCTGCTGCGTTTCCCCACCTT
ERR117153.28715906.2+    CCTGCTGCGTTTCCCCACCTT
ERR117153.39929989.2+    CCTGCTGCGTTTCCCCACCTT
ERR117153.50977464.2+    CCTGCTGCGTTTCCCCACCTT
ERR117153.52435271.1+    CCTGCTGCGTTTCCCCACCTT
ERR117153.9559693.1+     CCTGCTGCGTTTCCCCACCTT
ERR117154.24132143.2+    CCTGCTGCGTTTCCCCACCTT
ERR117154.28182887.1+    CCTGCTGCGTTTCCCCACCTT
ERR117154.32244393.2+    CCTGCTGCGTTTCCCCACCTT
ERR117154.51549766.2+    CCTGCTGCGTTTCCCCACCTT
ERR117154.52572361.2+    CCTGCTGCGTTTCCCCACCTT
ERR117154.53521277.1+    CCTGCTGCGTTTCCCCACCTT
ERR117154.55843371.2+    CCTGCTGCGTTTCCCCACCTT
ERR117155.1676696.1+     CCTGCTGCGTTTCCCCACCTT
ERR117155.24901097.2+    CCTGCTGCGTTTCCCCACCTT
ERR117155.27398178.1+    CCTGCTGCGTTTCCCCACCTT
ERR117155.30544527.2+    CCTGCTGCGTTTCCCCACCTT
ERR117155.32031392.1+    CCTGCTGCGTTTCCCCACCTT
ERR117155.35114720.1+    CCTGCTGCGTTTCCCCACCTT
ERR117155.56017163.2+    CCTGCTGCGTTTCCCCACCTT
ERR117155.58927721.1+    CCTGCTGCGTTTCCCCACCTT
ERR117155.5975449.2+     CCTGCTGCGTTTCCCCACCTT
ERR117172.15932939.1-    CCTGCTGCGTTTCCCCACCTT
ERR117189.3627525.1+     CCTGCTGCGTTTCCCCACCTT
ERR117162.15239877.1-    CCTGCTGCGTTTCCCCACCTTCC
ERR117162.17731664.1-    CCTGCTGCGTTTCCCCACCTTCC
ERR117162.20390690.1-    CCTGCTGCGTTTCCCCACCTTCC
ERR117188.6260249.1-     CCTGCTGCGTTTCCCCACCTTCCTG
ERR117162.24743150.1+    CCTGCTGCGTTTCCCCACCTTCCTGG
ERR117190.4710130.1-     CCTGCTGCGTTTCCCCACCTTCCTGGATCCTTCCAG
ERR117167.13563954.1+    CCTGCTGCGTTTCCCCACCTTCCTGGATCCTTCCAGCAT
ERR117184.14295738.2-    CCTGCTGCGTTTCCCCACCTTCCTGGATCCTTCCAGCAT
ERR117187.8211208.1-     CCTGCTGCGTTTCCCCACCTTCCTGGATCCTTCCAGCATGTT
ERR117189.16427324.1+    CCTGCTGCGTTTCCCCACCTTCCTGGATCCTTCCAGCATGTT
ERR117189.16427324.2-    CCTGCTGCGTTTCCCCACCTTCCTGGATCCTTCCAGCATGTT
ERR117186.15182709.1-    CCTGCTGCGTTTCCCCAGCTTCCTGGATCCTTCCAGCATGTTCTACCT
ERR117186.19827414.1-    CCTGCTGCGTTTCCCCACCTTCCTGGATCCTTCCAGCATGTTCTACCT
ERR117187.13695262.1-    CCTGCTGCGTTTCCCCACCTTCCTGGATCCTTCCAGCATGTTCTACCT
ERR117190.26390213.1-    CCTGCTGCGTTTCCCCACCTTCCTGGATCCTTCCAGCATGTTCTACCT
ERR117201.2485870.1-     CCTGCTGCGTTTCCCCACCTTCCTGGATCCTTCCAGCATGTTCTACCT
ERR117169.908271.1-      CCTGCTGCGTTTCCCCACCTTCCTGGATCCTTCCNGCATGTTCTACCTC
ERR117189.17247817.1+    CCTGCTGCGTTTCCCCACCTTCCTGGATCCTTCCAGCATGTTCTACCTC
ERR117168.6760065.1+     CCTGCTGCGTTTCCCCACCTTCCTGGATCCTTCCAGCATGTTCTACCTCTG
ERR117185.11582461.2-    CCTGCTGCGTTTCCCCACCTTCCTGGATCCTTCCAGCATGTTCTACCTCTG
ERR117191.3942459.1+     CCTGCTGCGTTTCCCCACCTTCCTGGATCCTTCCAGCATGTTCTACCTCTGCT
ERR117160.9073880.1-     CCTGCTGCGTTTCCCCACCTTCCTGGATCCTTCCAGCATGTTCTACCTCTGCTG
ERR117162.29804872.1-    CCTGCTGCGTTTCCCCACCTTCCTGGATCCTTCCAGCATGTTCTCCCTCTGCTG
ERR117171.21382053.1+    CCTGCTGCGTTTCCCCACCTTCCCGGATCCTTCCAGCACGTTCTACCTCTGCTG
ERR117156.1297884.2+     CCTGCTGCGTTTCCCCACCTTCCTGGATCCTTCCAGCATGTTCTACCTCTGCTGG
ERR117156.16352577.2+    CCTGCTGCGTTTCCCCACCTTCCTGGATCCTTCCAGCATGTTCTACCTCTGCTGG
ERR117157.38560318.2+    CCTGCTGCGTTTCCCCACCTTCCTGGATCCTTCCAGCATGTTCTACCTCTGCTGG
ERR117170.149546.1-      CCTGCTGCGTTTCCCCACCTTCCTGGATCCTTCCAGCATGTTCTACCTCTGCTGG
ERR117186.14041801.1+    CCTGCTGCGTTTCCCCACCTTCCTGGATCCTTCCAGCATGTTCTACCTCTGCTGG
ERR117214.14172132.2+    ACTGCTGCGTTTCCCCACCTTCCTGGATCCTTCCAGCATGTTCTACCTCTGCTGGG
ERR117190.38583922.2+    CCTGCTGCGTTTCCCCACCTTCCTGGATCCTTCCAGCATGTTCTACCTCTGCTGGGA
ERR117168.17100909.1-    CCTGCTGCGTTTCCCCACCTTCCTGGATCCTTCCAGCATGTTCTACCTCTGCTGGGAC
ERR117172.12496667.1-    CCTGCTGCGTTTCCCCACCTTCCTGGATCCTTCCAGCATGTTCTACCTCTGCTGGGAC
ERR117195.16676120.2-    CCTACTGCGTTTCCCCACCTTCCTGGATCCTTCCAGCATGTTCTACCTCTGCTGGGACC
ERR117156.12339954.1+    CCTGCTGCGTTTCCCCACCTTCCTGGATCCTTCCAGCATGTTCTACCTCTGCTGGGACCA
ERR117156.14079381.1+    CCTGCTGCGTTTCCCCACCTTCCTGGATCCTTCCAGCATGTTCTACCTCTGCTGGGACCA
ERR117156.15679746.1+    CCTGCTGCGTTTCCCCACCTTCCTGGATCCTTCCAGCATGTTCTACCTCTGCTGGGACCA
ERR117156.1899448.1+     CCTGCTGCGTTTCCCCACCTTCCTGGATCCTTCCAGCATGTTCTACCTCTGCTGGGACCA
ERR117156.27998490.1+    CCTGCTGCGTTTCCCCACCTTCCTGGATCCTTCCAGCATGTTCTACCTCTGCTGGGACCA
ERR117156.384374.1+      CCTGCTGCGTTTCCCCACCTTCCTGGATCCTTCCAGCATGTTCTACCTCTGCTGGGACCA
ERR117156.412636.1+      CCTGCTGCGTTTCCCCACCTTCCTGGATCCTTCCAGCATGTTCTACCTCTGCTGGGACCA
ERR117156.51082258.1+    CCTGCTGCGTTTCCCCACCTTCCTGGATCCTTCCAGCATGTTCTACCTCTGCTGGGACCA
ERR117156.55492752.1+    CCTGCTGCGTTTCCCCACCTTCCTGGATCCTTCCAGCATGTTCTACCTCTGCTGGGACCA
ERR117156.686079.1+      CCTGCTGCGTTTCCCCACCTTCCTGGATCCTTCCAGCATGTTCTACCTCTGCTGGGACCA
ERR117157.10175429.1+    CCTGCTGCGTTTCCCCACCTTCCTGGATCCTTCCAGCATGTTCTACCTCTGCTGGGACCA
```

```
ERR117157.21083951.1+    CCTGCTGCGTTTCCCCACCTTCCTGGATCCTTCCAGCATGTTCTACCTCTGCTGGGACCA
ERR117157.35348797.1+    CCTGCTGCGTTTCCCCACCTTCCTGGATCCTTCCAGCATGTTCTACCTCTGCTGGGACCA
ERR117157.47818519.1+    CCTGCTGCGTTTCCCCACCTTCCTGGATCCTTCCAGCATGTTCTACCTCTGCTGGGACCA
ERR117157.48178658.1+    CCTGCTGCGTTTCCCCACCTTCCTGGATCCTTCCAGCATGTTCTACCTCTGCTGGGACCA
ERR117157.48288776.1+    CCTGCTGCGTTTCCCCACCTTCCTGGATCCTTCCAGCATGTTCTACCTCTGCTGGGACCA
ERR117157.52982400.1+    CCTGCTGCGTTTCCCCACCTTCCTGGATCCTTCCAGCATGTTCTACCTCTGCTGGGACCA
ERR117157.6127110.1+     CCTGCTGCGTTTCCCCACCTTCCTGGATCCTTCCAGCATGTTCTACCTCTGCTGGGACCA
ERR117158.24629168.2-    CCTGCTGCGTTTCCCCACCTTCCTGGATCCTTCCAGCATGTTCTACCTCTGCTGGGACCA
ERR117158.4154112.1+     CCTGCTGCGTTTCCCCACCTTCCTGGATCCTTCCAGCATGTTCTACCTCTGCTGGGACCA
ERR117158.9230335.2+     ACTGCTGCGTTTCCCCACCTTCCTGGATCCTTCCAGCATGTTCTACCTCTGCTGGGCCCA
ERR117160.6004183.1+     CCTGCTGCGTTTCCCCACCTTCCTGGATCCTTCCAGCATGTTCTACCTCTGCTGGGACCA
ERR117162.19230106.1+    CCTGCTGCGTTTCCCCACCTTCCTGGATCCTTCCAGCATGTTCTACCTCTGCTGGGACCA
ERR117162.21063772.1-    CCGGCTGCGTTTCCCCACCTTCCTGGATCCTTCCAGCATGTTCTACCTCTGCTGGGACCA
ERR117167.12360378.1+    CCTGCTGCGTTTCCCCACCTTCCTGGATCCTTCCAGCATGTTCTACCTCTGCTGGGACCA
ERR117167.544228.1+      CCTGCTGCGTTTCCCCACCTTCCTGGATCCTTCCAGCATGTTCTACCTCTGCTGGGACCA
ERR117168.8755584.1-     CCTGCTGCGTTTCCCCACCTTCCTGGATCCTTCCAGCATGTTCTACCTCTGCTGGGACCA
ERR117169.2607605.1-     CCTTCTGCGTTTCCCCACCGTCCTGGATCCTTCCAGCATGTTCTACCTCTGCTGGGACCA
ERR117170.11495570.1+    CCTGCTGCGTTTCCCCACCTTCCTGGATCCTTCCAGCATGTTCTACCTCTGCTGGGACCA
ERR117170.12478522.2+    CCTGCTGCGTTTCCCCACCTTCCTGGATCCTTCCAGCATGTTCTACCTCTGCTGGGACCA
ERR117170.1901259.2+     CCTGCTGCGTTTCCCCACCTTCCTGGATCCTTCCAGCATGTTCTACCTCTGCTGGGACCA
ERR117170.6017029.1-     CCTGCTGCGTTTCCCCACCTTCCTGGATCCTTCCAGCATGTTCTACCTCTGCTGGGACCA
ERR117170.7975740.2+     CCTGCTGCGTTTCCCCACCTTCCTGGATCCTTCCAGCATGTTCTACCTCTGCTGGGACCA
ERR117172.10868533.2-    CCTGCTGCGTTTCCCCACCTTCCTGGATCCTTCCAGCATGTTCTACCTCTGCTGGGACCA
ERR117172.1851309.1+     CCTGCTGCGTTTCCCCACCTTCCTGGATCCTTCCAGCATGTTCTACCTCTGCTGGGACCA
ERR117172.19560183.2-    CCTGCTGCGTTTCCCCACCTTCCTGGATCCTTCCAGCATGTTCTACCTCTGCTGGGACCA
ERR117173.12302743.2+    CCTGCTGCGTTTCCCCACCTTCCTGGATCCTTCCAGCATGTTCTACCTCTGCTGGGACCA
ERR117173.1405665.2-     CCTGCTGCGTTTCCCCACCTTCCTGGATCCTTCCAGCATGTTCTACCTCTGCTGGGACCA
ERR117173.232780.1+      CCTGCTGCGTTTCCCCACCTTCCTGGATCCTTCCAGCATGTTCTACCTCTCCTGGGACCA
ERR117186.13986380.1-    CCTGCTGCTTTCCCCCCCTTCCTGGATCCTTCCAGCATGTTCTACCTCTGCTGGGACCA
ERR117186.21463358.2+    CCTGCTGCGTTTCCCCACCTTCCTGGATCCTTCCAGCATGTTCTACCTCTGCTGGGACCA
ERR117186.4205347.1-     CCTGCTGCGTTTCCCCACCTTCCTGGATCCTTCCAGCATGTTCTACCTCTGCTGGGACCA
ERR117186.6868637.1+     CCTGCTGCGTTTCCCCACCTTCCTGGATCCTTCCAGCATGTTCTACCTCTGCTGGGACCA
ERR117187.9292650.1-     CCTGCTGCGTTTCCCCACCTTCCTGGATCCTTCCAGCATGTTCTACCTCTGCTGGGACCA
ERR117188.5462950.1-     CCTGCTGCGTTTCCCCACCTTCCTGGATCCTTCCAGCATGTTCTACCTCTGCTGGGACCA
ERR117188.5463136.1-     CCTGCTGCGTTTCCCCACCTTCCTGGATCCTTCCAGCATGTTCTACCTCTGCTGGGACCA
ERR117189.19092939.2+    CCTGCTGCGTTTCCCCACCTTCCTGGATCCTTCCAGCATGTTCTACCTCTGCTGGGACCA
ERR117190.29386046.1+    CCTGCTGCGTTTCCCCACCTTCCTGGATCCTTCCAGCATGTTCTACCTCTGCTGGGACCA
ERR117190.31524659.2+    CCTGCTGCGTTTCCCCACCTTCCTGGATCCTTCCAGCATGTTCTACCTCTGCTGGGACCA
ERR117194.922317.2-      CCTGCTTCGTTTCCCCACCTTCCTGGATCCTTCCATCATGTTCTACCTCTGCTGGGACCA
ERR117195.13433153.1+    CCTGCTGCGTTTCCCCACCTTCCTGGATCCTTCCAGCATGTTCTACCTCTGCTGGGACCA
ERR117195.13585097.1-    CCTGCTGCGTTTCCCCACCTTCCTGGATCCTTCCAGCATGTTCTACCTCTGCTGGGACCA
ERR117195.13729654.1+    CCTGCTGCGTTTCCCCACCTTCCTGGATCCTTCCAGCATGTTCTACCTCTGCTGGGACCC
ERR117195.13884831.1+    CCTGCTGCGTTTCCCCACCTTCCTGGATCCTTCCAGCCTGTTCTACCTCTGCTGGGACCA
ERR117195.19866522.1+    CCTGCTGCGTTTCCCCACCTTCCTGGATCCTTCCAGCATGTTCTACCTCTGCTGGGACCA
ERR117195.20596627.1+    CCTGCTGCGTTTCCCCACCTTCCTGGATCCTTCCAGCATGTTCTACCTCTGCTGGGACCA
ERR117195.20988009.2-    CCTTCTGCGTTTCCCCCCCTTCCTGGATCCTTCCAGCATGTTCTGCCTCTGCTGGGACCA
ERR117195.5115980.1-     CCTGCTGCGTTTCCCCCCCTTCCTGGATCCTTCCAGCATGTTCTACCTCTGCTGGGACCA
ERR117195.6900905.1-     CCTGCCGCGTTTCCCCACCTTCCTGGATCCTTCCAGCATGTTCTACCTCTGCTGGGACCA
ERR117195.6901054.1-     CCTGCTGCGTTTCCCCACCGTCCTGGATCCTTCCAGCATGTTCTACCTCTGCTGGGACCA
ERR117195.8720933.1-     CCTGCTGCGTTTCCCCACCTTCCTGGATCCTTCCAGCATGTTCTACCTCTGCTGGGACCA
ERR117203.460754.1-      CCTGCTGCGTTTCCCCACCTTCCTGGATCCTTCCAGCATGTTCTACCTCTGCTGGGACCA
ERR117207.3319922.1-     CCGGCTGCGTTTCCCCACCTTCCTGGATCCTTCCAGCATGTTCTACCTCTGCTGGGACCA
ERR117209.2148070.1+     CCTGCTGCGTTTCCCCACCTTCCTGGATCCTTCCAGCATGTTCTACCTCTGCTGGGACCA
ERR117211.10510244.1+    CCTGCTGCGTTTCCCCACCTTCCTGGATCCTTCCAGCATGTTCTACCTCTGCTGGGACCA
ERR117211.10960067.1+    CCTGCTGCGTTTCCCCACCTTCCTGGATCCTTCCAGCATGTTCTACCTCTGCTGGGACCA
ERR117211.11635529.1+    CCTGCTGCGTTTCCCCACCTTCCTGGATCCTTCCAGCATGTTCTACCTCTGCTGGGACCA
ERR117212.8693972.1-     CCTGCTGCGTTTCCCCACCTTCCTGGATCCTTCCAGCATGTTCTACCTCTGCTGGGACCA
ERR117213.10610064.2+    ACTGCTGCGTTTCCCCACCTTCCTGGATCCTTCCAGCCTGTTCTACCTCTGCTCGGCCCA
ERR117213.20630415.2-    ACTGCTGCGTTTCCCCACCTTCCTGGATCCTTCCAGCATGTTCTACCTCTGCTGGGCCCA
ERR117214.14172132.1-    ACTGCTGCGTTTCCCCACCTTCCTGGATCCTTCCAGCATGTTCTACCTCTGCTGGGCCCA
ERR117185.7576876.1-       TGCTGCGTTTCCCCACCTTCCTGGATCCTTCCAGCATGTTCTACCTCTGCTGGGACCA
ERR117186.13703774.1-      TGCTGCGTTTCCCCCCCTTCCTGGATCCTTCCAGCATGTTCTACCTCTGCTGGGACCA
ERR117185.14382460.1+       GCTGCGTTTCCCCACCTTCCTGGATCCTTCCAGCATGTTCTACCTCTGCTGGGACCA
ERR117187.481263.1-         GCTGCGTTTCCCCACCTTCCTGGATCCTTCCAGCATGTTCTACCTCTGCTGGGACCA
ERR117196.3093370.1+        GCTGCGTTTCCCCACCTTCCTGGATCCTTCCAGCATGTTCTACCTCTGCTGGGACCA
ERR117213.70641.1+          GCTGCGTTTCCCCACCTTCCTGGATCCTTCCAGCATGTTCTCCCTCTGCTGGGACAA
ERR117161.30642625.1+        CTGCGTTTCCCCACCTTCCTGGATCCTTCCAGCATGTTCTACCTCTGCTGGGACCA
ERR117173.10378971.1+         TGCGTTTCCCCACCTTCCTGGATCCTTCCAGCATGTTCTACCTCTGCTGGGACCA
ERR117191.1712169.1-           GCGTTTCCCCGCCTNCCTGGATCCTTCCAGCATGTTCTACCTCTGCTGGGACCA
ERR117170.17484957.2+           CGTTTCCCCACCTTCCTGGATCCTTCCAGCATGTTCTACCTCTGCTGGGACCA
ERR117192.4993405.1+            CGTTTCCCCACCTTCCTGGATCCTTCCAGCATGTTCTACCTCTGCTGGGACCA
ERR117162.23912075.1+            GTTTCCCCACCTTCCTGGATCCTTCCAGCATGTTCTCCCTCTGCTGGGACCA
ERR117173.14111596.1+            GTTTCCCCACCTTCCTGGATCCTTCCAGCATGTTCTACCTCTGCTGGGACCA
ERR117197.6040782.1+             GTTTCCCCACCTTCCTGGATCCTTCCAGCATGTTCTACCTCTGCTGGGACCA
ERR117189.29044564.1-             TTTCCCCACCTTCCTGGATCCTTCCAGCATGTTCTACCTCTGCTGGGACCA
ERR117170.4755144.1-                TCCCCCCTGCCTGGATCCTTCCAGCATGTTCTACCTCTGCTGGGACCA
ERR117211.3135314.2+                TCCCCACCTTCCTGGATCCTTCCAGCATGTTCTACCTCTGCTGGGACCA
ERR117200.106648.1+                   CCCACCTTCCTGGATCCTTCCAGCATGTTCTACCTCTGCTGGGACCA
ERR117208.1138650.2-                  CCCACCTTCCGGGATCCTTCCAGCATGTTCTACCTCTGCTGGGACCA
ERR117212.13913207.2+                 CCCACCTTCCTGGATCCTTCCAGCATGTTCTACCTCTGCTGGGACCA
ERR117197.421777.1-                    CCACCTTCCTGGATCCTTCCAGCATGTTCTACCTCTGCTGGGACCA
ERR117190.5382735.1+                        TTCCTGGATCCTTCCAGCATGTTCTACCTCTGCTGGGACCA
ERR117213.24762755.1-                         CCTGGATCCTTCCAGCATGTTCTACCTCTGCTGGGCCCA
ERR117157.48953716.1-                          CTGGATCCTTCCAGCATGTTCTACCTCTGCTGGGACCA
```

```
ERR117190.29798443.1-                           TGGATCCTTCCAGCATGTTCTACCTCTGCTGGGACCA
ERR117159.22106723.1+                             GATCCTTCCAGCATGTTCTACCTCTGCTGGGACCA
ERR117188.12796779.1+                             GATCCTTCCAGCATGTTCTACCTCTGCTGGGACCA
ERR117161.21646014.1-                              ATCCTTCCAGCATGTTCTACCTCTGCTGGGACCA
ERR117186.7854080.1-                                TCCTTCCAGCATGTTCTACCTCTGCTGGGACCA
ERR117160.14580897.1+                                 CTTCCAGCATGTTCTACCTCTGCTGGGACCA
ERR117205.20017840.1+                                 CTTCCAGCATGTTCTACCTCTGCTGGGACCA
ERR117205.3454310.1+                                  CTTCCAGCATGTTCTACCTCTGCTGGGACCA
ERR117162.7505659.1-                                      CAGCATGTTCGACCTCTGCTGGGACCG
ERR117168.6995690.1-                                        GCATGTTCTCCCTCTGCTGGGACCA
ERR117170.3438595.2+                                        GCATGTTCTACCTCTGCTGGGACCA
ERR117211.8033843.2-                                        GCATGTTCTACCTCGGCTGGGCCCA
ERR117169.1769409.1+                                         CATGTTCTACCTCTGCTGGGACCA
ERR117172.25891693.1+                                         ATGTTCTACCTCTGCTGGGACCA
ERR117191.5032878.2+                                          ATGTTCTACCTCTGCTGGGACCA
ERR117189.19822430.1+                                          TGTTCTACCTCTGCTGGGACCA
ERR117214.17283903.1-                                            TTCTACCTCTGCTGGGCCCA
ERR117168.9275607.1+                                               CTACCTCTGCTGGGACCA
ERR117210.12667077.1+                                               TACCTCTGCTGGGACCA
ERR117210.15363689.1+                                               TACCTCTGCTGGGACCA
ERR117210.19301345.1+                                               TACCTCTGCTGGGACCA
ERR117210.2000732.1+                                                TACCTCTGCTGGGACCA
ERR117210.20471729.1+                                               TACCTCTGCTGGGACCA
ERR117210.21361233.1+                                               TACCTCTGCTGGGACCA
ERR117160.14706551.1-                                                CCCTGTGCTGGGACCG
ERR117158.4821344.1+                                                  CCTCTGCTGGGACCA
ERR117172.21418257.2-                                                 CCTCTGCGGGGACCA
ERR117159.18195311.1+                                                   TCTGCTGGGACCA
ERR117189.15711126.2-                                                    CTGCTGGGACCA
ERR117195.16895887.2-                                                    CTGCTGGGACCA
ERR117171.7573199.1+                                                      TGCTGGGACCA
ERR117171.11301750.1-                                                      GCTGGGACCA
ERR117193.5201767.1-                                                       GCTGGGACCA
ERR117195.17135423.1-                                                      GCTGGGACCA
ERR117160.876206.1-                                                         CGGGGACCA
ERR117189.5341307.2-                                                        CTGGGACCA
ERR117195.7556643.1-                                                        CGGGGACCA
ERR117199.6229760.2+                                                         TGGGACCA
ERR117158.14364574.1-                                                         GGGACCA
ERR117185.1830563.1+                                                            GACCA
ERR117214.5815958.1+                                                            GCCCA
ERR117191.5032878.1-                                                             ACCA
ERR117193.4247774.2-                                                              CCA
ERR117153.23642243.1+                                                              CA
ERR117153.55281904.1+                                                              CA
ERR117154.19671490.1+                                                              CA
ERR117154.51411665.1+                                                              CA
ERR117154.6725085.1+                                                               CA
ERR117155.1262030.1+                                                               CA
ERR117155.31097123.1+                                                              CA
ERR117155.39985310.1+                                                              CA
ERR117155.44201871.1+                                                              CA
                         ____________________________________________________________
consensus                CCTGCTGCGTTTCCCCACCTTCCTGGATCCTTCCAGCATGTTCTACCTCTGCTGGGACCA

                             .    :    .    :    .    :    .    :    .    :    .    :
ERR117167.544228.1+      C
ERR117173.232780.1+      CA
ERR117189.19092939.2+    CA
ERR117190.29386046.1+    CAA
ERR117172.1851309.1+     CAAG
ERR117156.12339954.1+    CAAGGA
ERR117156.14079381.1+    CAAGGA
ERR117156.15679746.1+    CAAGGA
ERR117156.1899448.1+     CAAGGA
ERR117156.27998490.1+    CAAGGA
ERR117156.384374.1+      CAAGGA
ERR117156.412636.1+      CAAGGA
ERR117156.51082258.1+    CAAGGA
ERR117156.55492752.1+    CAAGGA
ERR117156.686079.1+      CAAGGA
ERR117157.10175429.1+    CAAGGA
ERR117157.21083951.1+    CAAGGA
ERR117157.35348797.1+    CAAGGA
ERR117157.47818519.1+    CAAGGA
ERR117157.48178658.1+    CAAGGA
ERR117157.48288776.1+    CAAGGA
ERR117157.52982400.1+    CAAGGA
ERR117157.6127110.1+     CAAGGA
ERR117173.12302743.2+    CAAGGAACA
ERR117170.12478522.2+    CAAGGCACAG
ERR117212.8693972.1-     CAAGGAACAGG
ERR117211.10510244.1+    CAAGGAACAGGA
ERR117213.20630415.2-    CAAGGAACAGGAGC
```

ERR117186.4205347.1-	CAAGGAACAGGAGCTG
ERR117188.5462950.1-	CAAGGAACAGGAGCTG
ERR117188.5463136.1-	CAAGGAACAGGAGCTG
ERR117170.11495570.1+	CAAGGAACAGGAGCTGAT
ERR117203.460754.1-	CAAGGAACAGGAGCTGAT
ERR117172.19560183.2-	CAAGGAACAGGAGCTGATG
ERR117173.1405665.2-	CAAGGAACAGGAGCTGATG
ERR117186.21463358.2+	CAAGGAACAGGAGCTGATG
ERR117160.6004183.1+	CAAGGAACAGGAGCTGATGGG
ERR117190.31524659.2+	CAAGGAACAGGAGCTGATGGGG
ERR117195.13884831.1+	CAAGGAACAGGAGCTGATGGGG
ERR117214.14172132.1-	CAAGGAACAGGAGCTGATGGGGTT
ERR117214.17283903.1-	CAAGGAACAGGAGCTGATGGGGTTG
ERR117195.13433153.1+	CAAGGAACAGGAGCTGCTGGGGTTGA
ERR117195.19866522.1+	CAAGGAACAGGAGCTCATGGGGTTGA
ERR117195.20596627.1+	CAAGGAACAGGAGCTGATGGGGTTGA
ERR117167.12360378.1+	CAAGGAACAGGAGCTGATGGGGTTGAGCT
ERR117170.1901259.2+	CAAGGAACAGGAGCTGATGGGGTTGAGCT
ERR117170.6017029.1-	CAAGGAACAGGAGCTGATGGGGTTGAGCT
ERR117195.13729654.1+	CAAGGAACAGGAGCTGATGGGGTTGAGCT
ERR117211.11635529.1+	CAAGGAACAGGAGCTGATGGGGTTGAGCTG
ERR117158.24629168.2-	CAAGGAACAGGAGCTGATGGGGTTGAGCTGC
ERR117170.7975740.2+	CAAGGAACAGGAGCTGATGGGGTTGAGCTGC
ERR117162.21063772.1-	CAAGGAACAGGAGCTGATGGGGTTGAGCTGCACA
ERR117213.10610064.2+	CAAGGCACAGGAGCTGATGGGGTAGAGCTGCACA
ERR117158.4154112.1+	CAAGGAACAGGAGCTGATGGGGTTGAGCTGCACAT
ERR117162.19230106.1+	CAAGGAACAGGAGCTGATGGGGTTGAGCTGCACAT
ERR117168.8755584.1-	CAAGGAACAGGAGCTGATGGGGTTGAGCTGCACATC
ERR117172.10868533.2-	CAAGGAACAGGAGCTGATGGGGTTGAGCTGCACATC
ERR117186.6868637.1+	CAAGGAACAGGAGCTGATGGGGTTGAGCTGCACATCC
ERR117187.9292650.1-	CAAGGAACAGGAGCTGATGGGGTTGAGCTGCACATCCA
ERR117195.6900905.1-	CAAGGAACAGGAGCTGATGGGGTTGAGCTGCACATCCA
ERR117195.6901054.1-	CAAGGAACAGGAGCTGATGGGGTTGAGCTGCACATCCA
ERR117186.13986380.1-	CAAGGAACAGGAGCTGATGGGGTTGAGCTGCACATCCACA
ERR117157.48953716.1-	CAAGGAACAGGAGCTGATGGGGTTGAGCTGCACATCCACACA
ERR117189.5341307.2-	CAAGGAACAGGAGCTGATGGGGTTGAGCTGCACATCCACACA
ERR117187.481263.1-	CAAGGAACAGGAGCTGATGGGGTTGAGCTGCACATCCACACAA
ERR117207.3319922.1-	CAAGGAACAGGAGCTGATGGGGTTGAGCTGCACATCCACACAACT
ERR117169.2607605.1-	CAAGGAACAGGAGCTGATGGGGTTGAGCTGCACATCCACACAACTG
ERR117195.5115980.1-	CAAGGAACAGGAGCTGATGGGGTTGAGCTGCACATCCACACAACTGGC
ERR117189.29044564.1-	CAAGGAACAGGAGCTGATGGGGTTGAGCTGCACATCCACACAACTGGCT
ERR117158.9230335.2+	CAAGGAACAGGAGCTGATGGGGTTGAGCTGCACATCCACACAACTGGCTG
ERR117185.7576876.1-	CAAGGAACAGGAGCTGATGGGGTTGAGCTGCACATCCACACAACTGGCTGG
ERR117186.13703774.1-	CAAGGAACAGGAGCTGATGGGGTTGAGCTGCACATCCACACAACTGGCTGG
ERR117173.10378971.1+	AAAGGAACAGGAGCTGATGCGGTTGAGCTGCACATCCACACAACTGGCTGGG
ERR117185.14382460.1+	CAAGGAACAGGAGCTGCTGGGGTTGAGCTGCCCATCCACACCACTGGCTGGG
ERR117161.30642625.1+	CAAGGAACAGGAGCTGATGGGGTTGAGCTGCACATCCACACAACTGGCTGGGT
ERR117173.14111596.1+	CAAGGAACAGGAGCTGATGGGGGTGAGCTGCACATCCACACAACTGGCTGGGGGG
ERR117170.17484957.2+	CAAGGAACAGGAGCTGATGGGGTTGAGCTGCACATCCACACAACTGGCTGGGGGGC
ERR117162.23912075.1+	CAAGGAACAGGAGCTGATGGGGTTGAGCTGCACATCCACACAACTGTCTGGGTGGCA
ERR117211.10960067.1+	CAAGGAACAGGAGCTGATGGGGTTGAGCTGCACATCCACACAACTGGCTGGGTGGCA
ERR117190.5382735.1+	CAAGGAACAGGAGCTGATGGGGTTGAGCTGCACATCCACACAACTGGCTGGGGGGCATC
ERR117153.23642243.1+	CAAGGAACAGGAGCTGATGGGGTTGAGCTGCACATCCACACAACTGGCTGGGTGGCATCT
ERR117153.55281904.1+	CAAGGAACAGGAGCTGATGGGGTTGAGCTGCACATCCACACAACTGGCTGGGTGGCATCT
ERR117154.19671490.1+	CAAGGAACAGGAGCTGATGGGGTTGAGCTGCACATCCACACAACTGGCTGGGTGGCATCT
ERR117154.51411665.1+	CAAGGAACAGGAGCTGATGGGGTTGAGCTGCACATCCACACAACTGGCTGGGTGGCATCT
ERR117154.6725085.1+	CAAGGAACAGGAGCTGATGGGGTTGAGCTGCACATCCACACAACTGGCTGGGTGGCATCT
ERR117155.1262030.1+	CAAGGAACAGGAGCTGATGGGGTTGAGCTGCACATCCACACAACTGGCTGGGTGGCATCT
ERR117155.31097123.1+	CAAGGAACAGGAGCTGATGGGGTTGAGCTGCACATCCACACAACTGGCTGGGTGGCATCT
ERR117155.39985310.1+	CAAGGAACAGGAGCTGATGGGGTTGAGCTGCACATCCACACAACTGGCTGGGTGGCATCT
ERR117155.44201871.1+	CAAGGAACAGGAGCTGATGGGGTTGAGCTGCACATCCACACAACTGGCTGGGTGGCATCT
ERR117158.14364574.1-	CAAGGAACAGGAGCTGATGGGGTTGAGCTGCACATCCACACAACTGGCTGGGTGGCATCT
ERR117158.4821344.1+	CAAGGAACAGGAGCTGATGGGGTTGAGCTGCCCATCCACACAACTGGCTGGGGGGCCTCT
ERR117159.10361209.1+	CAAGGAACAGGAGCTGATGGGGTTGAGCTGCACATCCACACAACTGGCTGGGTGGCATCT
ERR117159.18195311.1+	CAAGGAACAGGAGCTGATGGGGTTGAGCTGCACATCCACACAACTGGCTGGGTGGCATCT
ERR117159.22106723.1+	CAAGGAACAGGAGCTGATGGGGTTGAGCTGCACATCCACACAACTGGCTGGGGGGCCTCC
ERR117160.14580897.1+	CAAGGAACAGGAGCTGATGGGGTTGAGCTGCACATCCACACAACTGGCTGGGTGGCATCT
ERR117160.14706551.1-	CAAGGAACAGGAGCTGATGGGGTTGAGCTGCACATCCACACAACTGGCTGGGTGGCATCT
ERR117160.876206.1-	CAAGGAACAGGGGCTGAGGGGGTTGAGCTGCCCATCCACACAACTGGCTGGGTGGCATCT
ERR117161.10441001.1+	CAAGGAACAGGAGCTGATGGGGTTGAGCTGCACATCCACACAACTGGCTGGGTGGCATCT
ERR117161.21646014.1-	CAAGGAACAGGAGCTGATGGGGTTGAGCTGCACATCCACACAACTGGCTGGGTGGCATCT
ERR117162.7505659.1-	CAAGGAACAGGAGCTGATGGGGTTGAGCTGCACATCCACACAACTGGCTGGGTGGCATCT
ERR117168.6995690.1-	CAAGGAACAGGAGCTGATGGGGTTGAGCTGCACATCCACACAACTGGCTGGGTGGCATCT
ERR117168.9275607.1+	CAAGGAACAGGAGCTGATGGGGTTGAGCTGCACATCCACACAACTGGCTGGGTGGCATCT
ERR117169.1769409.1+	CAAGGAACAGGAGCTGATGGGGTTGAGCTGCACATCCACACAACTGGCTGGGGGGCATCT
ERR117170.3438595.2+	CAAGGAACAGGAGCTGATGGGGTTGAGCTGCACATCCACACAACTGGCTGGGTGGCATCT
ERR117170.4755144.1-	CAAGGAACAGGAGCTGATGGGGTTGAGCTGCACATCCACACAACTGGCTGGGTGGCATCT
ERR117171.11301750.1-	CAAGGAACAGGAGCTGATGGGGTTGAGCTGCACATCCACACAACTGGCTGGGTGGCATCT
ERR117171.7573199.1+	CAAGGAACAGGAGCTGATGGGGTTGAGCTGCACATCCACACAACTGGCTGGGTGGCATCT
ERR117172.21418257.2-	CAAGGAACAGGAGCTGATGGGGTTGAGCTGCACATCCACACAACTGGCTGGGTGGCATCT
ERR117172.25891693.1+	CAAGGAACAGGAGCTGATGGGGTTGAGCTGCACATCCACACAACTGGCTGGGTGGCATCT
ERR117185.1830563.1+	CAAGGAACAGGAGCTGATGGGGTTGAGCTGCACATCCACACAACTGGCTGGGTGGCATCT
ERR117186.7854080.1-	CAAGGAACAGGAGCTGATGGGGTTGAGCTGCACATCCACACAACTGGCTGGGTGGCATCT
ERR117188.12796779.1+	CAAGGAACAGGAGCTGATGGGGTTGAGCTGCACATCCACACAACTGGCTGGGTGGCATCT

```
ERR117189.15711126.2-     CCAGGAACAGGAGCTGATGGGGTTGAGCTGCACATCCACACAACTGGCTGGGTGGCATCT
ERR117189.19822430.1+     CAAGGAACAGGAGCTGATGGGGTTGAGCTGCACATCCACACAACTGGCTGGGTGGCATCT
ERR117190.29798443.1-     CAAGGAACAGGAGCTGATGGGGTTGAGCTGCACATCCACACAACTGGCTGGGTGGCATCT
ERR117191.1712169.1-      CAAGGAACAGGAGCTGATGGGGTTGAGCTGCACATCCACACAACTGCCTGGGTGGCATCT
ERR117191.5032878.1-      CAAGGAACAGGAGCTGATGGGGTTGAGCTGCACATCCACACAACTGGCTGGGTGGCATCT
ERR117191.5032878.2+      CAAGGAACAGGAGCTGATGGGGTTGAGCTGCACATCCACACAACTGGCTGGGTGGCATCT
ERR117192.4993405.1+      CAAGGAACAGGAGCTGATGGGGTTGAGCTGCACATCCACACAACTGGCTGGGTGGCATCT
ERR117193.4247774.2-      CAAGGAACGGGAGCTGATGTGGTTGAGCTGCACATGCACACAAGTGGCTGGGTGGCATCT
ERR117193.5201767.1-      CAAGGAACAGGAGCTGAGGGGGTTGAGCTGCACATCCACACAACGGGCTGGGTGGCATCT
ERR117194.922317.2-       CAAGGAACAGGAGCTGATGGGGTTGAGCTGCACATCCACACAACTGCCTGGGTGGCATCT
ERR117195.16895887.2-     CAAGGAACAGGAGCTGATGGGGTTGAGCTGCACATCCACACAACTGGCTGGGTGGCATCT
ERR117195.17135423.1-     CAGGGAACGGGAGCTGATGGGGTTGAGCTGCACATCCACACAACTGGCTGGGTGGCATCT
ERR117195.20988009.2-     CAAGGAACAGGAGCTGATGGGGTTGAGCTGCACATCCACACAACTGGCTGGGTGGCATCT
ERR117195.7556643.1-      CAAGGAACAGGAGCTGAGGGGGTTGAGCTGCACATCCACACAACTGGCTGGGTGGCATCT
ERR117196.3093370.1+      CAAGGAACAGGAGCTGATGGGGTTGAGCTGCACATCCACACAACTGCCTGGGTGGCATCT
ERR117197.421777.1-       CAAGGAACAGGAGCTGATGGGGTTGAGCTGCACATCCACACAACTGCCTGGGTGGCATCT
ERR117197.6040782.1+      CAAGGAACAGGAGCTGATGGGGTTGAGCTGCACATCCACACAACTGCCTGGGTGGCATCT
ERR117199.6229760.2+      CAAGGAACAGGAGCTGATGGGGTTGAGCTGCACATCCACACAACTGCCTGGGTGGCATCT
ERR117200.106648.1+       CAAGGAACAGGAGCTGATGGGGTTGAGCTGCACATCCACACAACTGCCTGGGTGGCATCT
ERR117205.20017840.1+     CAAGGAACAGGAGCTGATGGGGTTGAGCTGCACATCCACACAACTGCCTGGGTGGCATCT
ERR117205.3454310.1+      CAAGGAACAGGAGCTGATGGGGTTGAGCTGCACATCCACACAACTGCCTGGGTGGCATCT
ERR117208.1138650.2-      CAAGGAACAGGAGCTGATGGGGTTGAGCTGCACATCCACACAACTGCCTGGGTGGCATCT
ERR117209.2148070.1+      CAAGGAACAGGAGCTGATGGGGTTGAGCTGCACATCCACACAACTGCCTGGGGGGCATCT
ERR117210.12667077.1+     CAAGGAACAGGAGCTGATGGGGTTGAGCTGCACATCCACACAACTGCCTGGGTGGCATCT
ERR117210.15363689.1+     CAAGGAACAGGAGCTGATGGGGTTGAGCTGCACATCCACACAACTGCCTGGGTGGCATCT
ERR117210.19301345.1+     CAAGGAACAGGAGCTGATGGGGTTGAGCTGCACATCCACACAACTGCCTGGGTGGCATCT
ERR117210.2000732.1+      CAAGGAACAGGAGCTGATGGGGTTGAGCTGCACATCCACACAACTGCCTGGGTGGCATCT
ERR117210.20471729.1+     CAAGGAACAGGAGCTGATGGGGTTGAGCTGCACATCCACACAACTGCCTGGGTGGCATCT
ERR117210.21361233.1+     CAAGGAACAGGAGCTGATGGGGTTGAGCTGCACATCCACACAACTGCCTGGGTGGCATCT
ERR117211.3135314.2+      CAAGGAACAGGAGCTGATGGGGTTGAGCTGCACATCCACACAACTGGCTGGGTGGCATCT
ERR117211.8033843.2-      CAAGGAACAGGAGCTGATGGGGTTGAGCTGCACATCCACACAACTGGCTGGGTGGCATCT
ERR117212.13913207.2+     CAAGGAACAGGAGCTGATGGGGTTGAGCTGCACATCCACACAACTGGCTGGGTGGCATCC
ERR117213.24762755.1-     CAAGGAACAGGAGCTGATGGGGTTGAGCTGCACATCCACACAACTGGCTGGGTGGCATCT
ERR117213.70641.1+        CACGGAACCGGCGCTGATGGGGTTGAGCTGCACATCCACACAACTGGCTGGGTGGCATCT
ERR117214.11189135.1-     CAAGGAACGGGGGCTGATGGGGTTGAGCTGCACATCCACACAACTGGCTGGGTGGCATCT
ERR117214.5815958.1+      CAAGGAACAGGAGCTGATGGGGTTGAGCTGCACATCCACACAACTGGCTGGGTGGCATCT
ERR117161.23788703.1+        GGAACAGGAGCTGATGGGGTTGAGCTGCACATCCACACAACTGGCTGGGTGGCATCT
ERR117167.14056574.1-        GGAACGGGAGCTGATGGGGTTGAGCTGCACATCCACACAACTGGCTGGGTGGCATCT
ERR117171.15015481.2+         GAACAGGAGCTGATGGGGTTGAGCTGCACATCCACACAACTGGCTGGGTGGCATCT
ERR117200.20534766.1-         GAACAGGAGCTGATGGGGTTGAGCTGCACATCCACACAACTGCCTGGGTGGCATCT
ERR117200.20534879.1-         GAACAGGAGCTGATGGGGTTGAGCTGCACATCCACACAACTGCCTGGGTGGCATCT
ERR117211.11264989.1+         GAACAGGAGCTGATGGGGTTGAGCTGCACATCCACACAACTGGCTGGGTGGCATCT
ERR117153.16400562.2+           ACAGGAGCTGATGGGGTTGAGCTGCACATCCACACAACTGGCTGGGTGGCATCT
ERR117153.22012558.2+           ACAGGAGCTGATGGGGTTGAGCTGCACATCCACACAACTGGCTGGGTGGCATCT
ERR117154.25543571.2+           ACAGGAGCTGATGGGGTTGAGCTGCACATCCACACAACTGGCTGGGTGGCATCT
ERR117168.3687101.1+            ACAGGAGCTGATGGGGTTGAGCTGCACATCCACACAACTGGCTGGGTGGCATCT
ERR117187.4682199.1+            ACAGGAGCTGATGGGGTTGAGCTGCACATCCACACAACTGGCTGGGTGGCATCT
ERR117190.36997267.1+           ACAGGAGCTGATGGGGTTGAGCTGCACATCCACACAACTGGCTGGGTGGCATCT
ERR117153.27720594.2+            CAGGAGCTGATGGGGTTGAGCTGCACATCCACACAACTGGCTGGGTGGCATCT
ERR117153.45094634.2+            CAGGAGCTGATGGGGTTGAGCTGCACATCCACACAACTGGCTGGGTGGCATCT
ERR117154.16009785.2+            CAGGAGCTGATGGGGTTGAGCTGCACATCCACACAACTGGCTGGGTGGCATCT
ERR117154.46529470.2+            CAGGAGCTGATGGGGTTGAGCTGCACATCCACACAACTGGCTGGGTGGCATCT
ERR117154.522286.2+              CAGGAGCTGATGGGGTTGAGCTGCACATCCACACAACTGGCTGGGTGGCATCT
ERR117155.24331140.2+            CAGGAGCTGATGGGGTTGAGCTGCACATCCACACAACTGGCTGGGTGGCATCT
ERR117155.46378739.2+            CAGGAGCTGATGGGGTTGAGCTGCACATCCACACAACTGGCTGGGTGGCATCT
ERR117190.11895861.1+             AGGAGCTGATGGGGTTGAGCTGCACATCCACACAACTGGCTGGGTGGCATCT
ERR117190.30731870.1+             AGGAGCTGATGGGGTTGAGCTGCACATCCACACAACTGGCTGGGTGGCATCT
ERR117197.400966.1-               AGGAGCTGATGGGGTTGAGCTGCACATCCACACAACTGCCTGGGTGGCATCT
ERR117199.3208434.1+              AGGAGCTGATGGGGTTGAGCTGCACATCCACACAACTGCCTGGGTGGCATCT
ERR117170.5195913.1+                GAGCTGATGGGGTTGAGCTGCACATCCACACAACTGGCTGGGTGGCATCT
ERR117167.17752603.1+                 GCTGATGGGGTTGAGCTGCACATCCACACAACTGGCTGGGTGGCATCT
ERR117188.13021263.1+                  CTGATGGGGTTGAGCTGCACATCCACACAACTGGCTGGGTGGCATCT
ERR117202.1843529.1-                   CTGATGGGGTTGAGCTGCACATCCACACAACTGCCTGGGTGGCATCT
ERR117167.7857224.1-                    TGATGGGGTTGAGCTGCACATCCACACAACTGGCTGGGTGGCATCT
ERR117185.11043877.1+                   TGATGGGGTTGAGCTGCACATCCACACAACTGGCTGGGTGGCATCT
ERR117190.45442737.1+                    GATGGGGTTGAGCTGCACATCCACACAACTGGCTGGGTGGCATCT
ERR117155.44519310.2+                     ATGGGGTTGAGCTGCACATCCACACAACTGGCTGGGTGGCATCT
ERR117211.6459571.2+                      ATGGGGTTGAGCTGCACATCCACACAACTGGCTGGGTGGCATCT
ERR117158.10800048.1+                      TGGGGTTGAGCTGCACATCCACACAACTGGCTGGCTGGCATCT
ERR117202.276018.2-                        TGGGGTTGAGCTGCACATCCACACAGCTGCCGGGGTGGCATCT
ERR117186.18226816.1-                        GGGTTGAGCTGCACATCCACACAACTGGCTGGGTGGCATCT
ERR117210.15005312.2+                        GGGTTGAGCTGCACATCCACACAACTGCCTGGGTGGCATCT
ERR117210.16500830.2+                        GGGTTGAGCTGCACATCCACACAACTGCCTGGGTGGCATCT
ERR117210.16854114.2+                        GGGTTGAGCTGCACATCCACACAACTGCCTGGGTGGCATCT
ERR117210.21340502.2+                        GGGTTGAGCTGCACATCCACACAACTGCCTGGGTGGCATCT
ERR117210.4356481.2+                         GGGTTGAGCTGCACATCCACACAACTGCCTGGGTGGCATCT
ERR117210.6082569.2+                         GGGTTGAGCTGCACATCCACACAACTGCCTGGGTGGCATCT
ERR117214.12669516.2+                         GGTTGAGCTGCACATCCACACAACTGGCTGGGTGGCATCT
ERR117189.21830335.2-                           TTGAGCTGCACATCCACACAACTGGCTGGGTGGCATCT
ERR117200.11764044.1-                           TTGAGCTGCACATCCACACAACTGCCTGGGTGGCATCT
ERR117172.844435.1-                               GAGCTGCACATCCACACAACTGGCTGGGTGGCATCT
ERR117190.3171531.1-                               AGCTGCACATCCACACAACTGGCTGGGTGGCATCT
ERR117170.8626134.1+                                GCTGCACATCCACACAACTGGCTGGGTGGCATCT
ERR117213.25383985.2+                               GCTGCACATCCACACAACTGGCTGGGTGGCATCT
```

```
ERR117187.1846104.2-                                 TGCACATCCACACAGCTGGCTGGGTGGCATCT
ERR117169.17408627.1-                                 GCACATCCCCACAACTGGCTGGGTGGCATCT
ERR117173.10552519.1-                                 GCACACCCACACAGCTGGCGGGGTGGCATCT
ERR117173.20651546.1+                                  CACATCCACACAACTGGCTGGGTGGCATCT
ERR117184.7371422.1+                                   CACATCCACACAACTGGCTGGGTGGCATCT
ERR117185.11325256.2+                                  CACATCCACACAACTGGCTGGGTGGCATCT
ERR117160.24336321.1-                                   ACATCCACACAGCTGGCTGGGTGGCATCT
ERR117171.23237058.2+                                    CATCCACACAACTGGCTGGGTGGCATCT
ERR117187.1165299.1-                                         CACACAACTGGCTGGGTGTCATCT
ERR117167.16748331.1-                                         ACACAACTGGCTGGGTGGCATCT
ERR117185.23115514.1-                                         ACACAACTGGCTGGGTGGCATCT
ERR117168.721185.1-                                              CAACTGGGTGGGTGGCAGCT
ERR117170.14720665.1+                                              ACTGGCTGGGTGGCATCT
ERR117195.19851398.2-                                              ACTGCCTGGGTGGCATCT
ERR117161.1948449.1+                                                CTGGCTGGGTGGCATCT
ERR117159.702483.1-                                                  TGGCTGGGTGGCATCT
ERR117160.22579884.1-                                                GGGCTGGGTGGCATCT
ERR117187.16354200.1-                                                TGGCTGGGTGGCATCT
ERR117158.17464169.2+                                                 GGCTGGGTGGCATCT
ERR117173.13365762.2-                                                 GGCGGGGTGGCATCT
ERR117185.21262523.2-                                                 GGCGGGGTGGCATCT
ERR117185.5549859.2-                                                  GGCTGGGTGGCATCT
ERR117203.2780230.2-                                                  GCCTGGGTGGCATCT
ERR117212.9510103.1-                                                  GGCTGGGTGGCATCT
ERR117190.28070050.2-                                                  GCTGGGTGGCATCT
ERR117209.2234770.1-                                                    CGGGGTGGCATCT
ERR117184.14949727.1+                                                       GTGGCATCT
ERR117212.18402684.1-                                                       GTGGCATCT
ERR117191.2740877.1-                                                         TGGCATCT
ERR117167.8827916.1-                                                          GGCATCT
ERR117186.8377888.2-                                                          GGCATCT
ERR117172.11772034.2-                                                          GCATCA
ERR117195.20783572.2-                                                             TCT
ERR117211.13982774.2-                                                             TCT
ERR117190.16447003.1-                                                              CT
ERR117200.4919005.1-                                                               CG
ERR117210.15005312.1-                                                              CT
ERR117210.16500830.1-                                                              CG
ERR117210.21340502.1-                                                              CG
ERR117210.4356481.1-                                                               CT
ERR117210.6082569.1-                                                               CT
ERR117212.13913207.1-                                                              CT
ERR117190.8741966.1+                                                                T

consensus                CAAGGAACAGGAGCTGATGGGGTTGAGCTGCACATCCACACAACTGGCTGGGTGGCATCT

                             .    :    .    :    .    :    .    :    .    :    .    :
ERR117190.29798443.1-    GGA
ERR117188.12796779.1+    GGATT
ERR117159.22106723.1+    GGATTCAGCCCTCC
ERR117161.21646014.1-    GGATTCAGCCCTCAT
ERR117162.7505659.1-     GGATTCAGCCCTCAT
ERR117186.7854080.1-     GGATTCAGCCCTCATG
ERR117208.1138650.2-     GGATTCAGCCCTCATG
ERR117195.16895887.2-    GGATTCAGCCCTCATGG
ERR117195.20988009.2-    GGATTCAGCCCTCATGG
ERR117153.23642243.1+    GGATTCAGCCCTCATGGA
ERR117153.55281904.1+    GGATTCAGCCCTCATGGA
ERR117154.19671490.1+    GGATTCAGCCCTCATGGA
ERR117154.51411665.1+    GGATTCAGCCCTCATGGA
ERR117154.6725085.1+     GGATTCAGCCCTCATGGA
ERR117155.1262030.1+     GGATTCAGCCCTCATGGA
ERR117155.31097123.1+    GGATTCAGCCCTCATGGA
ERR117155.39985310.1+    GGATTCAGCCCTCATGGA
ERR117155.44201871.1+    GGATTCAGCCCTCATGGA
ERR117160.14580897.1+    GGATTCAGCCCTCATGGA
ERR117189.19822430.1+    GGATTCAGACCTCCTGGA
ERR117194.922317.2-      GGATTCAGCCCTCATGGAGAGCT
ERR117168.6995690.1-     GGATTCAGCCCTCATGGAGAGCTG
ERR117170.3438595.2+     GGATTCAGCCCTCATGGAGAGCTG
ERR117169.1769409.1+     GGATTCAGCCCTCATGGAGAGCTGC
ERR117153.16400562.2+    GGATTCAGCCCTCATGGAGAGCTGCC
ERR117153.22012558.2+    GGATTCAGCCCTCATGGAGAGCTGCC
ERR117154.25543571.2+    GGATTCAGCCCTCATGGAGAGCTGCC
ERR117172.25891693.1+    GGATTCAGCCCTCATGGAGAGCTGCC
ERR117189.15711126.2-    GGATTCAGCCCTCATGGAGAGCTGCC
ERR117213.70641.1+       GGATTCAGCCCTCATGGAGAGCTGCC
ERR117153.27720594.2+    GGATTCAGCCCTCATGGAGAGCTGCCT
ERR117153.45094634.2+    GGATTCAGCCCTCATGGAGAGCTGCCT
ERR117154.16009785.2+    GGATTCAGCCCTCATGGAGAGCTGCCT
ERR117154.46529470.2+    GGATTCAGCCCTCATGGAGAGCTGCCT
ERR117154.522286.2+      GGATTCAGCCCTCATGGAGAGCTGCCT
ERR117155.24331140.2+    GGATTCAGCCCTCATGGAGAGCTGCCT
ERR117155.46378739.2+    GGATTCAGCCCTCATGGAGAGCTGCCT
```

ERR117209.2148070.1+	GGATTCAGCCCTCATGGAGAGCTGCCTGGG
ERR117168.9275607.1+	GGATTCAGCCCTCATGGAGAGCTGCCTGGGT
ERR117172.21418257.2-	GGATTCAGCCCTCATGGAGAGCTGCCTGGGT
ERR117211.3135314.2+	GGATTCAGCCCTCCTGGAGAGCCGCCTGGGG
ERR117159.18195311.1+	GGATTCAGCCCTCACGGAGAGCTGCCTGGGGC
ERR117160.14706551.1-	GGATTCAGCCCTCATGGAGAGCTGCCTGGGTCT
ERR117191.1712169.1-	GGATTCAGCCCTCATGGAGAGCTGCCTGGGTCT
ERR117196.3093370.1+	GGATTCAGCCCTCATGGAGAGCTGCCTGGGTCT
ERR117200.106648.1+	GGATTCAGCCCTCATGGAGAGCTGCCTGGGTCT
ERR117155.44519310.2+	GGATTCAGCCCTCATGGAGAGCTGCCTGGGTCTGAC
ERR117171.7573199.1+	GGATTCAGCCCTCATGGAGAGCTGCCTGGGGCTGACG
ERR117192.4993405.1+	GGATTCAGCCCTCATGGAGAGCTGCCTGGGTCTGACA
ERR117197.6040782.1+	GGATTCAGCCCTCATGGAGAGCTGCCTGGGTCTGACAT
ERR117171.11301750.1-	GGATTCAGCCCTCATGGAGAGCTGCCTGGGTCTGACGTT
ERR117160.876206.1-	GGATTCAGCCCTCATGGAGAGCTGCCTGGGTCTGACGTTG
ERR117212.13913207.2+	GGATTCAGACCTCACGGAGAGCTGCCTGGGGCCGACATTG
ERR117185.1830563.1+	GGATTCAGCCCTCATGGAGAGCTGCCTGGGTCTGACGTTGTGAC
ERR117197.421777.1-	GGATTCAGCCCTCATGGAGAGCTGCCTGGGTCTGACATTGTGAC
ERR117187.4682199.1+	GGATTCAGCCCTCATGGAGAGCTGCCTGGGTCTGACATTGTGACAA
ERR117190.36997267.1+	GGATTCAGCCCTCATGGAGAGCTGCCTGGGTCTGACATTGTGACAA
ERR117190.11895861.1+	GGATTCAGCCCTCATGGAGAGCTGCCTGGGTCTGACATTGTGACAAGA
ERR117190.30731870.1+	GGATTCAGCCCTCATGGAGAGCTGCCTGGGTCTGACATTGTGACAAGA
ERR117159.10361209.1+	GGATTCAGCCCTCATGGAGAGCTGCCTGGGTCTGACATTGTGACAAGAG
ERR117161.10441001.1+	GGATTCAGCCCTCATGGAGAGCTGCCTGGGTCTGACATTGTGACAAGAG
ERR117205.20017840.1+	GGATTCAGCCCTCATGGAGAGCTGCCTGGGTCTGACATTGTGACAAGAG
ERR117205.3454310.1+	GGATTCAGCCCTCATGGAGAGCTGCCTGGGTCTGACATTGTGACCAGAG
ERR117171.15015481.2+	GGATTCAGCCCTCATGGAGAGCTGCCTGGGTCTGACGTTGTGACAAGAGG
ERR117213.24762755.1-	GGATTCAGCCCTCATGGAGAGCTGCCTGGGTCTGACATTGTGACAAGAGGT
ERR117158.4821344.1+	GGATTCAGCCCTCATGGAGAGCTGCCCGGGTGTGACATTGTGACAAAAGGTG
ERR117161.23788703.1+	GGATTCAGCCCTCATGGAGAGCTGCCTGGGTCTGACATTGTGACAAGAGGTG
ERR117167.14056574.1-	GGATTCAGCCCTCATGGAGAGCTGCCTGGGTCTGACATTGTGACAAGAGGTG
ERR117188.13021263.1+	GGATTCAGCCCTCATGGAGAGCTGCCTGGGTCTGACATTGTGACAAGAGGTGC
ERR117211.8033843.2-	GGATTCAGCCCTCATGGAGAGCTGCCTGGGTCTGACATTGTGACAAGAGGTGCC
ERR117168.3687101.1+	GGATTCAGCCCTCATGGAGAGCTGCCTGGGTCTGACATTGTGACAAGAGGTGCCT
ERR117190.45442737.1+	GGATTCAGCCCTCATGGAGAGCTGCCTGGGTCTGACGTTGTGACAAGAGGTGCCT
ERR117170.5195913.1+	GGATTCAGCCCTCATGGAGAGCTGCCTGGGTCTGACATTGTGACAAGAGGGGCCTTCCA
ERR117158.10800048.1+	GGATTCAGCCCTCATGGAGAGCTGCCTGGGTCTGACATTGTGACAAGAGGTGCCTTCCAA
ERR117158.14364574.1-	GGATTCAGCCCTCATGGAGAGCTGCCTGGGTCTGACATTGTGACAAGAGGTGCCTTCCAA
ERR117158.17464169.2+	GGATTCAGCCCTCATGGAGAGCTGCCTGGGTCTGACATTGTGACAAGAGGTGCCTTCCAA
ERR117159.702483.1-	GGATTCAGCCCTCATGGAGAGCTGCCTGGGTCTGACATTGTGACAAGAGGTGCCTTCCAA
ERR117160.22579884.1-	GGATTCAGCCCTCATGGAGAGCTGCCTGGGTCTGACGTTGTGACAAGAGGTGCCTTCCAA
ERR117160.24336321.1-	GGATTCAGCCCTCATGGAGAGCTGCCTGGGTCTGACATTGTGACAAGAGGTGCCTTCCAA
ERR117161.1948449.1+	GGATTCAGCCCTCATGGAGAGCTGCCTGGGTCTGACGTTGTGACAAGAGGTGCCTTCCAA
ERR117167.16748331.1-	GGATTCAGCCCTCATGGAGAGCTGCCTGGGTCTGACGTTGTGACAAGAGGTGCCTTCCAA
ERR117167.17752603.1+	GGATTCAGCCCTCATGGAGAGCTGCCTGGGTCTGACATTGTGACAAGAGGTGCCTTCCAA
ERR117167.7857224.1-	GGATTCAGCCCTCATGGAGAGCTGCCTGGGTCTGACGTTGTGACAAGAGGTGCCTTCCAA
ERR117167.8827916.1-	GGATTCAGCCCTCAGGGAGAGCTGCCTGGGTCTGACGTTGTGACAAGAGGTGCCTTCCAA
ERR117168.721185.1-	GGATTCAGCCCTCATGGAGAGCTGCCTGGGTCTGACGTTGTGACAAGAGGTGCCTTCCAA
ERR117169.17408627.1-	GGATTCAGCCCTCATGGAGAGCTGCCTGGGTCTGACGTTGTGACAAGAGGTGCCTTCCAA
ERR117170.14720665.1+	GGATTCAGCCCTCATGGAGAGCTGCCTGGGGCTGACGGTGTGGCCAGAGGTGCCTTCCAA
ERR117170.8626134.1+	GGATTCAGCCCTCATGGAGAGCTGCCTGGGTCTGACATTGTGACAAGAGGTGCCTTCCAA
ERR117171.23237058.2+	GGATTCAGCCCTCATGGAGAGCTGCCTGGGTCTGACGTTGTGACAAGAGGTGCCTTCCAA
ERR117172.11772034.2-	GGATTCAGCCCGCAGGGAGAGCTGCGTGGGTCTGACATTGTGACGAGAGGCGCCTTCCAA
ERR117172.844435.1-	GGATTCAGCCCTCATGGAGAGCTGCCTGGGTCTGACATTGTGACAAGAGGTGCCTTCCAA
ERR117173.10552519.1-	GGATTCAGCCCTCATGGAGAGCTGCCTGGGTCTGACATTGTGACAAGAGGTGCCTTCCAA
ERR117173.13365762.2-	GGATTCAGCCCTCATGGAGAGCTGCCTGGGTCTGACATTGTGACAAGAGGTGCCTTCCAA
ERR117173.20651546.1+	GGATTCAGCCCTCATGGAGAGCTGCCTGGGTCTGACGTTGTGACAAGAGGTGCCTTCCAC
ERR117184.14949727.1+	GGATTCAGCCCTCATGGAGAGCTGCCTGGGTCTGACATTGTGACAAGAGGTGCCTTCCAA
ERR117184.7371422.1+	GGATTCAGCCCTCATGGAGAGGCGCCTGGGTCTGACGTTGTGACAAGAGGTGCCTTCCAA
ERR117185.10160607.2+	GGATTCAGCCCTCATGGAGAGCTGCCTGGGTCTGACGTTGTGACAAGAGGTGCCTTCCAA
ERR117185.11043877.1+	GGATTCAGCCCTCATGGAGAGCTGCCTGGGTCTGACATTGTGACAAGAGGTGCCTTCCAA
ERR117185.11325256.2+	GGATTCAGCCCTCATGGAGAGCTGCCTGGGTCTGACATTGTGACAAGAGGTGCCTTCCAA
ERR117185.21262523.2-	GGATTCAGCCCTCATGGAGAGCTGCCTGGGTCTGACATTGTGACAAGAGGTGCCTTCCAA
ERR117185.23115514.1-	GGATTCAGCCCTCATGGAGAGCTGCCTGGGTCTGACGTTGTGACAAGAGGTGCCTTCCAA
ERR117185.23254860.1+	GGATTCAGCCCTCATGGAGAGCTGCCTGGGTCTGACGTTGTGACAAGAGGTGCCTTCCAA
ERR117185.5549859.2-	GGAGTCAGCCCTCATGGAGAGCTGCCTGGGTCTGACATTGTGACAAGAGGTGCCTTCCAA
ERR117186.18226816.1-	GGATTCAGCCCTCATGGAGAGCTGCCTGGGTCTGACATTGTGACAAGAGGTGCCTTCCAA
ERR117186.8377888.2-	GGATTCAGCCCTCATGGAGAGCTGCCTGGGTCTGACATTGTGACAAGAGGTGCCTTCCAA
ERR117187.1165299.1-	GGATTCAGCCCTCATGGAGAGCTGCCTGGGTCTGACATTGTGACAAGAGGTGCCTTCCAA
ERR117187.16354200.1-	GGATTCAGCCCTCATGGAGAGCTGCCTGGGTCTGACGTTGTGACAAGAGGTGCCTTCCAA
ERR117187.1846104.2-	GGATTCAGCCCTCATGGAGAGCTGCCTGGGTCTGACATTGTGACAAGAGGTGCCTTCCAA
ERR117189.21830335.2-	GGATTCAGCCCTCATGGAGAGCTGCCTGGGTCTGACGTTGTGACAAGAGGTGCCTTCCAA
ERR117190.16447003.1-	GGATTCAGCCCTCATGGAGAGCTGCCTGGGTCTGACATTGTGACAAGAGGTGCCTTCCAA
ERR117190.28070050.2-	GGATTCAGCCCTCATGGAGAGCTGCCTGGGTCTGACGTTGTGACAAGAGGTGCCTTCCAA
ERR117190.3171531.1-	GGATTCAGCCCTCATGGAGAGCTGCCTGGGTCTGACATTGTGACAAGAGGTGCCTTCCAA
ERR117190.8741966.1+	GGATTCAGCCCTCATGGAGAGCTGCCTGGGTCTGACGTTGTGACAAGAGGTGCCTTCCAA
ERR117191.2740877.1-	GGATTCAGCCCTCATGGAGAGCTGCCTGGGTCTGACATTGTGACAAGAGGTGCCTTCCAA
ERR117191.5032878.1-	GGATTCAGCCCTCATGGAGAGCTGCCTGGGTCTGACATTGTGACAAGAGGTGCCTTCCAA
ERR117191.5032878.2+	GGATTCAGCCCTCATGGAGAGCTGCCTGGGTCTGACATCGTGACAAGAGGTGCCTTCCAA
ERR117193.4247774.2-	GGATTCAGCCCTCATGGAGAGCTGCCTGGGTCTGACATTGTGACAAGAGGGGCCTTCCAA
ERR117193.5201767.1-	GGATTCAGCCCTCATGGAGAGCTGCCTGGGTCTGACATTGTGACAAGAGGTGCCTTCCAA
ERR117195.17135423.1-	GGATTCAGCCCTCATGGAGAGCTGCCTGGGTCTGACATTGTGACAAGAGGTGCCTTCCAA
ERR117195.19851398.2-	GGATTCAGCCCTCATGGAGAGCTGCCTGGGTCTGACATTGTGACAAGAGGTGCCTTCCAA
ERR117195.20783572.2-	GGATTCGTCCCTGATGGAGAGCTGCCTGGGTCGGACAGTGTGACAAGAGGTGCCTTCCAA

ERR117195.7556643.1- GGATTCAGCCCTCATGGAGAGCTGCCTGGGTCTGACATTGTGACAAGAGGTGCCTTCCAA
ERR117197.400966.1- GGATTCAGCCCTCATGGAGAGCTGCCTGGGTCTGACATTGTGACAAGAGGTGCCTTCCAA
ERR117199.3208434.1+ GGATTCAGCCCTCATGGAGAGCTGCCTGGGTCTGACATTGTGACAAGAGGTGCCTTCCAA
ERR117199.6229760.2+ GGATTCAGCCCTCATGGAGAGCTGCCTGGGTCTGACATTGTGACAAGAGGTGCCTTCCAC
ERR117200.11764044.1- GGATTCAGCCCTCATGGAGAGCTGCCTGGGTCTGACATTGTGACAAGAGGTGCCTTCCAA
ERR117200.20534766.1- GGATTCAGCCCTCATGGAGAGCTGCCTGGGTCTGACATTGTGACAAGAGGTGCCTTCCAA
ERR117200.20534879.1- GGATTCAGCCCTCATGGAGAGCTGCCTGGGTCTGACATTGTGACAAGAGGTGCCTTCCAA
ERR117200.4919005.1- GGAGTCAGCCCTCATGGAGAGCTGCCTGGGTCTGACATTGTGACAAGAGGTGCCTTCCAA
ERR117202.1843529.1- GGATTCAGCCCTCATGGAGAGCTGCCTGGGTCTGACATTGTGACAAGAGGTGCCTTCCAA
ERR117202.276018.2- GGATTCAGCCCTCATGGAGAGCTGCCTGGGTCTGACATTGTGACAAGAGGTGCCTTCCAA
ERR117203.2780230.2- GGATTCAGCCCTCATGGAGAGCTGCCTGGGTCTGACATTGTGACAAGAGGTGCCTTCCAA
ERR117209.2234770.1- GGATTCAGCGCGCATGGAGAGCTGCCTGGGTCTGACATTGTGACAAGAGGTGCCTTCCAA
ERR117210.12667077.1+ GGATTCAGCCCTCATGGAGAGCTGCCTGGGTCTGACATTGTGACAAGAGGTGCCTTCCAA
ERR117210.15005312.1- GGATTCAGCCCTCATGGAGAGCTGCCTGGGTCTGACATTGTGACAAGAGGTGCCTTCCAA
ERR117210.15005312.2+ GGATTCAGCCCTCATGGAGAGCTGCCTGGGTCTGACATTGTGACAAGAGGTGCCTTCCAA
ERR117210.15363689.1+ GGATTCAGCCCTCATGGAGAGCTGCCTGGGTCTGACATTGTGACAAGAGGTGCCTTCCAA
ERR117210.16500830.1- GGACTCAGCCCTCATGGAGAGCTGCCTGGGTCTGACATTGTGACAAGAGGTGCCTTCCAA
ERR117210.16500830.2+ GGATTCAGCCCTCATGGAGAGCTGCCTGGGTCTGACATTGTGACAAGAGGTGCCTTCCAA
ERR117210.16854114.2+ GGATTCAGCCCTCATGGAGAGCTGCCTGGGTCTGACATTGTGACAAGAGGTGCCTTCCAA
ERR117210.19301345.1+ GGATTCAGCCCTCATGGAGAGCTGCCTGGGTCTGACATTGTGACAAGAGGTGCCTTCCAA
ERR117210.2000732.1+ GGATTCAGCCCTCATGGAGAGCTGCCTGGGTCTGACATTGTGACAAGAGGTGCCTTCCAA
ERR117210.20471729.1+ GGATTCAGCCCTCATGGAGAGCTGCCTGGGTCTGACATTGTGACAAGAGGTGCCTTCCAA
ERR117210.21340502.1- GGATTCAGCCCTCATGGAGAGCTGCCTGGGTCTGACATTGTGACAAGAGGTGCCTTCCAA
ERR117210.21340502.2+ GGATTCAGCCCTCATGGAGAGCTGCCTGGGTCTGACATTGTGACAAGAGGTGCCTTCCAA
ERR117210.21361233.1+ GGATTCAGCCCTCATGGAGAGCTGCCTGGGTCTGACATTGTGACAAGAGGTGCCTTCCAA
ERR117210.4356481.1- GGATTCAGCCCTCATGGAGAGCTGCCTGGGTCTGACATTGTGACAAGAGGTGCCTTCCAA
ERR117210.4356481.2+ GGATTCAGCCCTCATGGAGAGCTGCCTGGGTCTGACATTGTGACAAGAGGTGCCTTCCAA
ERR117210.6082569.1- GGATTCAGCCCTCATGGAGAGCTGCCTGGGTCTGACATTGTGACAAGAGGTGCCTTCCAA
ERR117210.6082569.2+ GGATTCAGCCCTCATGGAGAGCTGCCTGGGTCTGACATTGTGACAAGAGGTGCCTTCCAA
ERR117211.11264989.1+ GGATTCAGCCCTCATGGAGAGCTGCCTGGGTCTGACATTGTGACAAGAGGTGCCTTCCAA
ERR117211.13982774.2- GGATTCAGCCCTCATGGAGAGCTGCCTGGGTCTGACATTGTGACAAGAGGTGCCTTCCAA
ERR117211.6459571.2+ GGATTCAGCCCTCATGGAGAGCTGCCTGGGTCTGACATTGTGACAAGAGGTGCCTTCCAA
ERR117212.13913207.1- GGATTCAGCCGTCATGGAGAGCTGCCTGGGTCTGACATTGTGACAAGAGGTGCCTTCCAA
ERR117212.18402684.1- GGATTCGGCCCTCATGGAGAGCTGCCTGGGTCTGACATTGTGACAAGAGGTGCCTTCCAA
ERR117212.9510103.1- GGATTCAGCCCTCATGGAGAGCTGCCTGGGTCTGACATTGTGACAAGAGGTGCCTTCCAA
ERR117213.25383985.2+ GGATTCAGCCCTCATGGAGAGCTGCCTGGGTCTGACATTGTGACAAGAGGTGCCTTCCAA
ERR117214.11189135.1- GGATTCAGCCCTCATGGAGAGCTGCCTGGGTCTGACATTGTGACAAGAGGTGCCTTCCAA
ERR117214.12669516.2+ GGATTCAGCCCTCATGGAGAGCTGCCTGGGTCTGACATTGGGACAAGAGGTGCCTTCCAA
ERR117214.5815958.1+ GGATTCAGCCCTCATGGAGAGCTGCCTGGGTCTGACATTGTGACAAGAGGTGCCTTCCAA
ERR117173.20977760.1+ GATTCAGCCCTCATGGAGAGCTGCCTGGGTCTGACATTGTGACAAGAGGTGCCTTCCAA
ERR117195.13241995.1- GATTCAGCCCTCATGGAGAGCTGCCTGGGTCTGACATTGTGACAAGAGGTGCCTTCCAA
ERR117195.14059793.1- GATTCAGCCGTCAGGGAGAGCTGCCTGGGTCTGACATTGTGACAAGAGGTGCCTTCCAA
ERR117195.19875258.1- GATCCAGCCCTCAGGGAGAGCGGCCTGGGTCTGACATTGTGACAAGAGGTGCCTTCCAA
ERR117195.6845458.1- GATTCGGCCCTCATGGAGGGCTGCCTGGGTCTGACATTGTGACAAGAGGTGCCTTCCAA
ERR117203.3734878.2- ATTCAGCCCTCATGTAGGGCTGCCGGGGTCTGACATTGTGACAAGAGGTGCGTTCCAA
ERR117211.7038507.2- ATTCACCCCTCAGGGAGAGCTGCCTGGGTCTGACATTGTGACAAGAGGTGCCTTCCAA
ERR117184.20758333.1+ TCAGCCCTCATGGAGAGCTGCCTGGGTCTGACATTGTGACAAGAGGTGCCTTCCAA
ERR117186.6680280.2- TCAGCCCTCATGGAGAGCTGCCTGGGTCTGACATTGTGACAAGAGGTGCCTTCCAA
ERR117172.22363800.1- AGCCCTCATGGAGAGCTGCCTGGGTCTGACATTGTGACAAGAGGTGCCTTCCAA
ERR117211.6530188.1- AGCCCTCATGGAGAGCTGCCTGGGTCTGACATTGTGACAAGAGGTGCCTTCCAA
ERR117160.10839667.1+ GCCCTCATGGAGAGCTGCCTGGGTCTGACATTGTGACAAGAGGTGCCTTCCAA
ERR117193.4162248.2- GCCCTCATGGAGAGCTGGCTGGGTCTGACATTGTGACAAGAGGTGCCTTCCAA
ERR117184.1045359.1+ CCCTCATGGAGAGCTGCCTGGGTCTGACATTGTGACAAGAGGTGCCTTCCAA
ERR117205.11725674.1+ CCCTCATGGAGAGCTGCCTGGGTCTGACATTGTGACAAGAGGTGCCTTCCAA
ERR117205.12765034.1+ CCCTCATGGAGAGCTGCCTGGGTCTGACATTGTGACAAGAGGTGCCTTCCAA
ERR117205.18084573.1+ CCCTCATGGAGAGCTGCCTGGGTCTGACATTGTGACAAGAGGTGCCTTCCAA
ERR117205.20836240.1+ CCCTCATGGAGAGCTGCCTGGGTCTGACATTGTGACAAGAGGTGCCTTCCAA
ERR117173.10498732.1- CATGGAGAGCTGCCTGGGTCTGACGTTGTGACAAGAGGTGCCTTCCAA
ERR117185.25418354.1- CATGGAGAGCTGCCTGGGTCTGACGTTGTGACAAGAGGTGCCTTCCAA
ERR117214.17076092.1+ CATGGAGAGCTGCCTGGGTCTGACATTGTGACAAGAGGTGCCTTCCAA
ERR117195.5194609.2- AGGGAGAGCGGCCGGTGTCTGACATTGTGACAAGAGGTGCCTTCCAA
ERR117206.2892479.2+ TGGAGAGCTGCCTGGGTCTGACATTGTGACAAGAGGTGCCTTCCAA
ERR117202.4492296.1+ GGAGAGCTGCCTGGGTCTGACATTGTGACAAGAGGTGCCTTCCAA
ERR117212.16686714.1- GAGAGCGGCCTGGGTCTGACATTGTGACAAGAGGTGCCTTCCAA
ERR117162.29184588.1- AGGGCTGCCTGGGTCTGACGTTGTGACAAGAGGTGCCTTCCAA
ERR117191.3942459.2- AGAGCTGCCTGGTGCTGACATTGTGACGAGAGGTGCCTTCCAA
ERR117171.22484358.1- GGGCTGCCTGGGGCTGACGTTGTGACAAGAGGTGCCTTCCAA
ERR117185.18468589.1- GAGCTGCCTGGGTCTGACATTGTGACAAGAGGTGCCTTCCAA
ERR117185.19578803.1- GAGCTGCCTGGGTCTGACATTGTGACAAGAGGTGCCTTCCAA
ERR117158.9230335.1- AGCGGCCTGGGTCTGACATTGTGACAAGAGGTGCCTTCCAA
ERR117159.12488008.1+ AGCTGCCTGGGTCTGACGTTGTGACAAGAGGTGCCTTCCAA
ERR117210.16854114.1- GCGGCCGGGGTCGGACATTGTGACAAGAGGTGCCTTCCAA
ERR117186.18182009.1- CTGCCTGGGTCTGACGTTGTGACAAGAGGTGCCTTCCAA
ERR117189.21787522.1- TGCCGGGGTCTGACGTTGTGACAAGAGGTGCCGTCCAA
ERR117167.20252206.1+ CCTGGGTCTGACATTGTGACAAGAGGTGCCTTCCAA
ERR117170.18019832.1- CTGGGTCTGACATTGTGACAAGAGGTGCCTTCCAA
ERR117158.17464169.1- TGGGTCTGACATTGTGACAAGAGGTGCCTTCCAA
ERR117169.15062876.1- GGGTCTGACATTGTGACAAGAGGTGCCTTCCAA
ERR117173.386521.1- GTCTGACATTGTGACAAGAGGTGCCTTCCAA
ERR117158.11622681.1- CTGACATTGTGACAAGAGGTGCCTTCCAA
ERR117195.24277877.1+ CTGACATTGTGACAAGAGGTGCCTTCCAA
ERR117195.25470877.1+ CTGACATTGTGACAAGAGGTGCCTTCCAA
ERR117195.25471015.1+ CTGACATTGTGACAAGAGGTGCCTTCCAA
ERR117170.18440732.2- GACGTGGTGAGAAGAGGTGCCTTCCAA

```
ERR117213.25383985.1-                                      ACATTGTGACAAGAGGTGCCTTCCAA
ERR117209.1866596.1+                                        CATTGTGACAAGAGGTGCCTTCCAA
ERR117159.15086134.1+                                        GTTGTGACAAGAGGTGCCTTCCAA
ERR117212.3472658.1+                                          TTGTGACAAGAGGTGCCTTCCAA
ERR117212.3472658.2-                                          TTGTGACAAGAGGTGCCTTCCAA
ERR117189.14477456.2-                                          TGTGACAAGAGGTGCCTTCCAA
ERR117186.15047856.2-                                            TGACAAGAGGTGCCTTCCAG
ERR117168.10534830.1+                                              ACAAGAGGTGCCTTCCAA
ERR117189.17247817.2-                                              ACAAGAGGTGCCTTCCAA
ERR117214.16394658.1-                                               CAAGAGGTGCCTTCCAA
ERR117168.19739446.1+                                                AAGAGGTGCCTTCCAA
ERR117171.22772451.1+                                                AAGAGGTGCCTTCCAA
ERR117191.5977567.2+                                                  AGAGGTGCCTTCCAA
ERR117211.10773022.2+                                                  GAGGTGCCTTCCAA
ERR117170.3438595.1-                                                      GTGCCTTCCAA
ERR117162.7835727.1+                                                        GCCTTCCAA
ERR117205.21325428.2-                                                       GCCTTCCAA
ERR117205.3064670.2-                                                        GCCTTCCAA
ERR117211.4852441.2-                                                        GCCTTCCAA
ERR117211.4957486.2-                                                        GCCTTCCAA
ERR117214.5729010.2+                                                        GCCTTCCAA
ERR117167.6958174.1+                                                         CCTTCCAA
ERR117200.11660475.1+                                                        CCTTCCAA
ERR117200.4827260.1+                                                         CCTTCCAA
ERR117188.12142941.1+                                                         CTTCCAA
ERR117190.12805217.1+                                                         CTTCCAA
ERR117167.9426509.1+                                                           TTCCAA
ERR117171.23237058.1-                                                         TTCCAA
ERR117205.21962995.2-                                                         TTCCAA
ERR117159.1161003.1+                                                             CCAA
ERR117185.10160607.1-                                                            CCAA
ERR117172.14663654.2-                                                              AA
ERR117184.9889636.1-                                                                A

consensus                GGATTCAGCCCTCATGGAGAGCTGCCTGGGTCTGACATTGTGACAAGAGGTGCCTTCCAA

                             .    :    .    :    .    :    .    :    .    :    .    :
ERR117167.17752603.1+    A
ERR117191.5032878.2+     A
ERR117167.7857224.1-     AAG
ERR117185.11043877.1+    AAG
ERR117210.12667077.1+    AAG
ERR117210.19301345.1+    AAG
ERR117210.2000732.1+     AAG
ERR117210.20471729.1+    AAG
ERR117210.21361233.1+    AAG
ERR117190.3171531.1-     AAGGG
ERR117186.18226816.1-    AAGGGCAG
ERR117187.1846104.2-     AAGGGCAG
ERR117195.17135423.1-    AAGGGCAGC
ERR117195.7556643.1-     AAGGGCAGC
ERR117159.702483.1-      AAGGGCAGCATCT
ERR117172.844435.1-      AAGGGCAGCATCT
ERR117173.10552519.1-    AAGGGCAGCATCTG
ERR117170.8626134.1+     AAGGGCAGCATCTGC
ERR117211.6459571.2+     AAGGGCAGCAACTGC
ERR117187.1165299.1-     AAGGGCAGCATCTGCT
ERR117199.6229760.2+     AAGGGCAGCATCTGCT
ERR117169.17408627.1-    AAGGGCAGCATCTGCTTC
ERR117173.20651546.1+    AAGGGCAGCATCTGCTTCT
ERR117184.7371422.1+     AAGGGCAGCATCTGCTTCT
ERR117185.11325256.2+    AAGGGCAGCATCTGCTTCT
ERR117193.4247774.2-     AAGGGCAGCATCTGCTTCT
ERR117160.24336321.1-    AAGGGCAGCATCTGCTTCTC
ERR117170.14720665.1+    AAGGGCAGCCTCTGCTTCTC
ERR117193.5201767.1-     AAGGGCAGCATCTGCTTCTC
ERR117160.22579884.1-    AAGGGCAGCATCTGCTTCTCT
ERR117171.23237058.2+    AAGGGCAGCATCTGCTTCTCT
ERR117158.14364574.1-    AAGGGCAGCATCTGCTTCTCTgt
ERR117168.721185.1-      AAGGGCAGCATCTGCTTCTCTgt
ERR117184.14949727.1+    AAGGGCAGCATCTGCTTCTCTgt
ERR117187.16354200.1-    AAGGGCAGCATCTGCTTCTCTgta
ERR117200.20534766.1-    AAGGGCAGCATCTGCTTCTCTgta
ERR117200.20534879.1-    AAGGGCAGCATCTGCTTCTCTgta
ERR117211.11264989.1+    AAGGGCAGCATCTGCTTCTCTgta
ERR117214.5815958.1+     AAGGGCAGCATCTGCTTCTCTgtaa
ERR117167.16748331.1-    AAGGGCAGCATCTGCTTCTCTgtaag
ERR117185.23115514.1-    AAGGGCAGCATCTGCTTCTCTgtaag
ERR117190.28070050.2-    AAGGGCAGCATCTGCTTCTCTgtaag
ERR117191.5032878.1-     AAGGGCAGCATCTGCTTCTCTgtaag
ERR117212.3472658.1+     AAGGGCAGCATCTGCTTCTCTgtaagtt
ERR117212.3472658.2-     AAGGGCAGCATCTGCTTCTCTgtaagtt
ERR117214.11189135.1-    AAGGGCAGCATCTGCTTCTCTgtaagttga
ERR117161.1948449.1+     AAGGGCAGCATCTGCTTCTCTgtaagttgaac
```

ERR117190.8741966.1+	AAGGGCAGCATCTGCTTCTCTgtaaggtgaaca
ERR117173.13365762.2-	AAGGGCAGCATCTGCTTCTCTgtaagttgaacag
ERR117185.21262523.2-	AAGGGCAGCATCTGCTTCTCTgtaagttgaacag
ERR117185.5549859.2-	AAGGGCAGCATCTGCTTCTCTgtaagttgaacag
ERR117193.4162248.2-	AAGGGCGGCAGCTGCTTCTCTgtaagttgaacag
ERR117190.16447003.1-	AAGGGCAGCATCTGCTTCTCTgtaagttgaacaggttt
ERR117197.400966.1-	AAGGGCAGCATCTGCTTCTCTgtaagttgaacaggttt
ERR117199.3208434.1+	AAGGGCAGCATCTGCTTCTCTgtaagttgaacaggttt
ERR117210.15005312.2+	AAGGGCAGCATCTGCTTCTCTgtaagttgaacaggtttc
ERR117210.16500830.2+	AAGGGCAGCATCTGCTTCTCTgtaagttgaacaggtttc
ERR117210.16854114.2+	AAGGGCAGCATCTGCTTCTCTgtaagttgaacaggtttc
ERR117210.21340502.2+	AAGGGCAGCATCTGCTTCTCTgtaagttgaacaggtttc
ERR117210.4356481.2+	AAGGGCAGCATCTGCTTCTCTgtaagttgaacaggtttc
ERR117210.6082569.2+	AAGGGCAGCATCTGCTTCTCTgtaagttgaacaggtttc
ERR117202.276018.2-	AAGGGCAGCATCTGCTTCTCTgtaagttgaacaggtttcct
ERR117203.2780230.2-	AAGGGCAGCATCTGCTTCTCTgtaagttgaacaggtttcct
ERR117167.8827916.1-	AAGGGCAGCATCTGCTTCTCTgtaagttgaacaggtttcctc
ERR117172.11772034.2-	GAGGGCAGCATCTGCTTCTCTgtaagttgagcaggtttcctc
ERR117186.8377888.2-	AAGGGCAGCATCTGCTTCTCTgtaagttgaacaggtttcctc
ERR117200.11764044.1-	AAGGGCAGCATCTGCTTCTCTgtaagttgaacaggtttcctc
ERR117202.1843529.1-	AAGGGCAGCATCTGCTTCTCTgtaagttgaacaggtttcctct
ERR117159.12488008.1+	AAGGGCAGCATCTGCTTCTCTgtaagttgaaaaggtttactcttc
ERR117185.23254860.1+	AAGGGCAGCATCTGCTTCTCTgtaagttgaacaggtttcctcttcg
ERR117158.10800048.1+	AAGGGCAGCATCTGCTTCTCTgtaagttgaacaggtttcttcttcgt
ERR117185.10160607.2+	AAGGGCAGCATCTGCTTCTCTgtaagttgaacaggtttcctcttcgtcc
ERR117173.20977760.1+	AAGGGCAGCATCTGCTTCTCTgtaagttgaacaggtttcctctttgtccc
ERR117186.6680280.2-	AAGGGCAGCATCTGCTTCTCTgtaagttgaacaggtttcctctttgtccc
ERR117214.12669516.2+	ACGGGCAGCATCTGCTTCTCTgtaccttgaacaggtttcttcttcgtccc
ERR117184.20758333.1+	AAGGGCAGCATCTGCTTCTCTgtacgttgaacaggtttcctctttgtcccacg
ERR117172.22363800.1-	AAGGGCAGCATCTGCTTCTCTgtaagttgaacaggtttcctctttgtcccacgct
ERR117160.10839667.1+	AAGGGCAGCATCTGCTTCTCTgtaagttgaacaggtttcctctttgtcccacgctg
ERR117213.25383985.2+	AAGGGCAGCATCTGCTTCTCTgtaagttgaacaggtttcttcttcgtcccacgctg
ERR117184.1045359.1+	AAGGGCAGCATCTGCTTCTCTgtaagttgaacaggtttcctctttgtcccacgctgc
ERR117158.11622681.1-	AAGGGCAGCATCTGCTTCTCTgtaagttgaacaggtttcttcttcgtcccacgctgccaa
ERR117158.17464169.1-	AAGGGCAGCATCTGCTTCTCTgtaagttgaacaggtttcttcttcgtcccacgctgccaa
ERR117158.17464169.2+	AAGGGCAGCATCTGCTTCTCTgtaagttgaacaggtttcttcttcgtcccacgctgccaa
ERR117158.9230335.1-	AAGGGCAGCATCTGCTTCTCTgtaagttgaacaggtttcttcttcgtcccacgctgccaa
ERR117159.1161003.1+	AAGGGCAGCATCTGCTTCTCTgtaagttgaacaggtttcctcttcgtcccacactgccaa
ERR117159.15086134.1+	AAGGGCAGCATCTGCTTCTCTgtaagttgaacaggtttcctcttcgtcccacactgccaa
ERR117162.29184588.1-	AAGGGCAGCATCTGCTTCTCTgtaagttgaacaggtttcctcttcgtcccacactgccaa
ERR117162.7835727.1+	AAGGGCAGCATCTGCTTCTCTgtaagttgaacaggtttcctctttgtcccacgctgccaa
ERR117167.20252206.1+	AAGGGCAGCATCTGCTTCTCTgtaagttgaacaggtttcctctttgtcccacgctgccaa
ERR117167.6958174.1+	AAGGGCAGCATCTGCTTCTCTgtaagttgaacaggtttcctctttgtcccacgctgccaa
ERR117167.9426509.1+	AAGGGCAGCATCTGCTTCTCTgtaagttgaacaggtttcctcttcgtcccacactgccaa
ERR117168.10534830.1+	AAGGGCAGCATCTGCTTCTCTgtaagttgaacaggtttcctcttcgtcccacactgccaa
ERR117168.19739446.1+	AAGGGCAGCATCTGCTTCTCTgtaagttgaacaggtttcctcttcgtcccacactgccaa
ERR117169.15062876.1-	AAGGGCAGCATCTGCTTCTCTgtaagttgaacaggtttcctctttgtcccacgctgccaa
ERR117170.18019832.1-	AAGGGCAGCATCTGCTTCTCTgtaagttgaacaggtttcctctttgtcccacgctgccaa
ERR117170.18440732.2-	AAGGGCAGCATCTGCTTCTCTgtaagttgaacaggtttcctctttgtcccacgctgccaa
ERR117170.3438595.1-	AAGGGCAGCATCTGCTTCTCTgtaagttgaacaggtttcctctttgtcccacgctgccaa
ERR117171.22484358.1-	AAGGGCAGCATCTGCTTCTCTgtaagttgaacaggtttcctcttcgtcccacactgccaa
ERR117171.22772451.1+	AAGGGCAGCATCTGCTTCTCTgtaagttgaacaggtttcctctttgtcccacgctgccaa
ERR117171.23237058.1-	AAGGGCAGCATCTGCTTCTCTgtaagttgaacaggtttcctcttcgtcccacactgccaa
ERR117172.14663654.2-	AAGGGCAGCATCTGCGTCTCTgtaaggtgaacaggtttcctctttgtcccacgctgccaa
ERR117173.10498732.1-	AAGGGCAGCATCTGCTTCTCTgtaagttgaacaggtttcctcttcgtcccacactgccaa
ERR117173.386521.1-	AAGGGCAGCATCTGCTTCTCTgtaagttgaacaggtttcctctttgtcccacgctgccaa
ERR117184.9889636.1-	AAGGGCAGCATCTGCTTCTCTgtaagttgaacaggtttcctctttgtcccacgctgccaa
ERR117185.10160607.1-	AAGGGCAGCATCTGCTTCTCTgtaagttgaacaggtttcctcttcgtcccacactgccaa
ERR117185.18468589.1-	AAGGGCAGCATCTGCTTCTCTgtaagttgaacaggtttcctctttgtcccacgctgccaa
ERR117185.19578803.1-	AAGGGCAGCATCTGCTTCTCTgtaagttgaacaggtttcctctttgtcccacgctgccaa
ERR117185.25418354.1-	AAGGGCAGCATCTGCTTCTCTgtaagttgaacaggtttcctcttcgtcccacactgccaa
ERR117186.15047856.2-	AAGGGCAGCATCTGCTTCTCTgtaagttgaacaggtttcctctttgtcccacgctgccaa
ERR117186.18182009.1-	AAGGGCAGCATCTGCTTCTCTgtaagttgaacaggtttcctcttcgtcccacactgccaa
ERR117188.12142941.1+	AAGGGCAGCATCTGCTTCTCTgtaagttgaacaggtttcctctttgtcccacgctgccaa
ERR117189.14477456.2-	AAGGGCAGCATCTGCTTCTCTgtaagttgaacaggtttcctcttcgtcccacactgccaa
ERR117189.17247817.2-	AAGGGCAGCATCTGCTTCTCTgtaagttgaacaggtttcctcttcgtcccacactgccaa
ERR117189.21787522.1-	AAGGGCAGCATCTGCTTCTCTgtaagttgaacaggtttcctcttcgtcccacactgccaa
ERR117190.12805217.1+	AAGGGCAGCATCTGCTTCTCTgtaagttgaacaggtttcctctttgtcccacgctgccaa
ERR117191.2740877.1-	AAGGGCAGCATCTGCTTCTCTgtaagttgaacaggtttcctcttcgtcccacgctgccaa
ERR117191.3942459.2-	AAGGGCAGCATCTGCTTCTCTgtaagttgaacaggtttcctcttcgtcccacgctgccaa
ERR117191.5977567.2+	AAGGGCAGCATCTGCTTCTCTgtaagttgaacaggtttcctcttcgtcccacgctgccaa
ERR117195.13241995.1-	AAGGGCAGCATCTGCTTCTCTgtaagttgaacaggtttcctcttcgtcccacgctgccaa
ERR117195.14059793.1-	AAGGGCAGCATCTGCTTCTCTgtaagttgaacaggtttcctcttcgtcccacgctgccaa
ERR117195.19851398.2-	AAGGGCAGCATCTGCTTCTCTgtaagttgaacaggtttcctcttcgtcccacgctgccaa
ERR117195.19875258.1-	AAGGGCAGCATCTGCTTCTCTgtaagttgaacaggtttcctcttcgtcccacgctgccaa
ERR117195.20783572.2-	AAGGGCAGCATCTGCTTCTCTgtaagttgaacaggtttcctcttcgtcccacgctgccaa
ERR117195.24277877.1+	AAGGGCAGCATCTGCTTCTCTgtaagttgaacaggtttcctcttcgtcccacgctgccaa
ERR117195.25470877.1+	AAGGGCAGCATCTGCTTCTCTgtaagttgaacaggtttcctcttcgtcccacgctgccaa
ERR117195.25471015.1+	AAGGGCAGCATCTGCTTCTCTgtaagttgaacaggtttcctcttcgtcccacgctgccaa
ERR117195.5194609.2-	AAGGGCAGCATCTGCTTCTCTgtaagttgaacaggtttcctcttcgtcccacgctgccaa
ERR117195.6845458.1-	AAGGGCAGCATCTGCTTCTCTgtaagttgaacaggtttcctcttcgtcccacgctgccaa
ERR117200.11660475.1+	AAGGGCAGCATCTGCTTCTCTgtaagttgaacaggtttcctcttcgtcccacgctgccaa
ERR117200.4827260.1+	AAGGGCAGCATCTGCTTCTCTgtaagttgaacaggtttcctcttcgtcccacgctgccaa
ERR117200.4919005.1-	AAGGGCAGCATCTGCTTCTCTgtaagttgaacaggtttcctcttcgtcccacgctgccaa

```
ERR117202.4492296.1+     AAGGGCAGCATCTGCTTCTCTgtaagttgaacaggtttcctctttgtcccacgctgccaa
ERR117203.3734878.2-     AAGGGCAGCATCTGCTTCTCTgtaagttgaacaggtttcctctttgtcccacgctgccaa
ERR117205.11725674.1+    AAGGGCAGCATCTGCTTCTCTgtaagttgaacaggtttcctctttgtcccacgctgccaa
ERR117205.12765034.1+    AAGGGCAGCATCTGCTTCTCTgtaagttgaacaggtttcctctttgtcccacgctgccaa
ERR117205.18084573.1+    AAGGGCAGCATCTGCTTCTCTgtaagttgaacaggtttcctctttgtcccacgctgccaa
ERR117205.20836240.1+    AAGGGCAGCATCTGCTTCTCTgtaagttgaacaggtttcctctttgtcccacgctgccaa
ERR117205.21325428.2-    AAGGGCAGCATCTGCTTCTCTgtaagttgaacaggtttcctctttgtcccacgctgccaa
ERR117205.21962995.2-    AAGGGCAGCATCTGCTTCTCTgtaagttgaacaggtttcctctttgtcccacgctgccaa
ERR117205.3064670.2-     AAGGGCAGCATCTGCTTCTCTgtaagttgaacaggtttcctctttgtcccacgctgccaa
ERR117206.2892479.2+     AAGGGCAGCATCTGCTTCTCTgtaagttgaacaggcttcctcttcgtcccacgctgccaa
ERR117209.1866596.1+     AAGGGCAGCATCTGCTTCTCTgtaagttgaacaggtttcctcttcgtcccacgctgccaa
ERR117209.2234770.1-     AAGGGCAGCATCTGCTTCTCTgtaagttgaacaggtttcctcttcgtcccacgctgccaa
ERR117210.15005312.1-    AAGGGCAGCATCTGCTTCTCTgtaagttgaacaggtttcctcttcgtcccacgctgccaa
ERR117210.16500830.1-    AAGGGCAGCATCTGCTTCTCTgtaagttgaacaggtttcctcttcgtcccacgctgccaa
ERR117210.16854114.1-    AAGGGCAGCATCTGCTTCTCTgtaagttgaacaggtttcctcttcgtcccacgctgccaa
ERR117210.21340502.1-    AAGGGCAGCATCTGCTTCTCTgtaagttgaacaggtttcctcttcgtcccacgctgccaa
ERR117210.4356481.1-     AAGGGCAGCATCTGCTTCTCTgtaagttgaacaggtttcctcttcgtcccacgctgccaa
ERR117210.6082569.1-     AAGGGCAGCATCTGCTTCTCTgtaagttgaacaggtttcctcttcgtcccacgctgccaa
ERR117211.10773022.2+    AAGGGCAGCATCTGCTTCTCTgtaagttgaacaggtttcctcttcgtcccacgctgccaa
ERR117211.13982774.2-    AAGGGCAGCATCTGCTTCTCTgtaagttgaacaggtttcctcttcgtcccacactgccaa
ERR117211.4852441.2-     AAGGGCAGCATCTGCTTCTCTgtaagttgaacaggtttcctcttcgtcccacactgccaa
ERR117211.4957486.2-     AAGGGCAGCATCTGCTTCTCTgtaagttgaacaggtttcctcttcgtcccacactgccaa
ERR117211.6530188.1-     AAGGGCAGCATCTGCTTCTCTgtaagttgaacaggtttcctcttcgtcccacactgccaa
ERR117211.7038507.2-     AAGGGCAGCATCTGCTTCTCTgtaagttgaacaggtttcctcttcgtcccacgctgccaa
ERR117212.13913207.1-    AAGGGCAGCATCTGCTTCTCTgtaagttgaacaggtttcctcttcgtcccacactgccaa
ERR117212.16686714.1-    AAGGGCAGCATCTGCTTCTCTgtaagttgaacaggtttcctcttcgtcccacactgccaa
ERR117212.18402684.1-    AAGGGCAGCATCTGCTTCTCTgtaagttgaacaggtttcctcttcgtcccacactgccaa
ERR117212.9510103.1-     AAGGGCAGCATCTGCTTCTCTgtaagttgaacaggtttcctcttcgtcccacactgccaa
ERR117213.25383985.1-    AAGGGCAGCATCTGCTTCTCTgtaagttgaacaggtttcttcttcgtcccacgctgccaa
ERR117214.16394658.1-    AAGGGCAGCATCTGCTTCTCTgtaagttgaacaggtttcttcttcgtcccacgctgccaa
ERR117214.17076092.1+    AAGGGCAGCATCTGCTTCTCTgtaagttgaacaggtttcttcttcgtcccacgctgccaa
ERR117214.5729010.2+     AAGGGCAGCATCTGCTTCTCTgtaagttgaacaggtttcttcttcgtcccacgctgccaa
ERR117185.10163404.1+      GGGCAGCATCTGCTTCTCTgtaagttgaacaggtttcctcttcgtcccacactgccaa
ERR117209.885141.2+            AGCATCTGCTTCTCTgtaagttgaacaggtttcctcttcgtcccacgctgccaa
ERR117162.26586686.1-           GCATCTGCTTCTCTgtaagttgaacaggtttcctctttgtcccacgctgccaa
ERR117173.12302743.1-               CTGCTTCTCTgtaagttgaacaggtttcctcttcgtcccacactgccaa
ERR117170.12241048.1+                TGCTTCTCTgtaagttgaacaggtttcctcttcgtcccacactgccaa
ERR117188.9453026.2-                      CTCTgtaagttgaacaggtttcctcttcgtcccaccctgccaa
ERR117213.20416582.2+                        Tgtaagttgaacaggtttcttcttcgtcccacgctgccaa
                         ____________________________________________________________
consensus                AAGGGCAGCATCTGCTTCTCTgtaagttgaacaggtttcctcttcgtcccacgctgccaa
```

F. Zebra finch (No. 7), exon 1, WGS data

- CAP3 alignment of WGS data spanning exon 1 (uppercase letters)

```
                             .    :    .    :    .    :    .    :    .    :    .    :
1239865472+                  gtttctcctggctgcccaatgtaccagcaagactcagagactgcagagATGAT
1219534330-                  gtttctcctagctgcccaaggtaccagcaagactcagagactgcagagATGATGGTGATC
1230596782+                  gtttctcctagctgcccaaggtaccagcaagactcagagactgcagagATGATGGTGATC
1231322579-                  gtttctcctggctgcccaaggtaccagcaagactcagagactgcagagATGATGGTGATC
1231538640-                  gtttctcctggctgcccaaggtaccagcaagactcagagactgcagagATGATGGTGATC
1404858879-                  gtttctcctggctgcccaaggtaccagcaagactcagagactgcagagATGATGGTGATC
1267622776-                                             caagactcagagactgcagagATGATGGTGATC
1252412210-                                                      gagactgcagagATGATGGTGATC
1423366051-                                                                        GTGATC
                             ____________________________________________________________
consensus                    gtttctcctggctgcccaaggtaccagcaagactcagagactgcagagATGATGGTGATC

                             .    :    .    :    .    :    .    :    .    :    .    :
1219534330-                  TTTTCAGGAATCAAG-AGAATGCTCTTCCTTTTGTTCTCTTGCCTCTGTTCTGG-CCAGC
1230596782+                  TTTTCAGGAATCAAG-AGAATGCTCTTCCTTTTGTTCTCTTGCCTCTGTTCTGG-CCAGC
1231322579-                  TTTTCAGGAATCAAG-AGAATGCTCTTCCTTTTGTTCTCTTGCCTCTGTTCTGG-CCAGC
1231538640-                  -TTTCAGGAATCAAG-AGAATGCTCTTCCTTTTGTTCTCTTGCCTCTGTTCTGG-CCAGC
1252412210-                  TTTTCAGGAATCAAGAAGAATGCTCTTCCTTTTGTTCTCTTGCCTCTGT-CT-GACCAGC
1267622776-                  TTTTCAGGAATCAAG-AGAATGCTCTTCCTTTTGTTCTCTTGCCTCTGTTCTGGCCCAGC
1404858879-                  TTTTCAGGAATCAAG-AGAATGCTCTTCCTTTTGTTCTCTTGCCTCTGTTCTGG-CCAGC
1423366051-                  TTTTCAGGAATCAAG-AGAATGCTCTT-CATTTGTTCTCTTGCCTCTGTTCTGG-CCAGC
1230149097-                                                        CTTGCATCTGTTCTGG-CCAGC
                             ____________________________________________________________
consensus                    TTTTCAGGAATCAAG-AGAATGCTCTTCCTTTTGTTCTCTTGCCTCTGTTCTGG-CCAGC

                             .    :    .    :    .    :    .    :    .    :    .    :
1219534330-                  TTGCATCT-CCACTGTTGAGTTTCTCCACCTT-CCTGGATCCTTCTAGCATGTTCCACCT
1230149097-                  TTGCATCT-CCACTGCTGCGTTTCTCCTCCTT-CCTGGATCCTTCTAGCATGTTCCACCT
1230596782+                  TTGCATCT-CCACTGTTGAGTTTCTCCACCTT-CCTGGATCCTTCTAGCATGTTCCACCT
1231322579-                  TTGCATCT-CCACTGCTGCGTTTCTCCACCTT-CCTGGATCCTTCTAGCATGTTCCACCT
1231538640-                  TTGCATCT-CCACTGCTGCGTTTCTCCACCTTTCCTGGATCTTTCTAGCATGTTCCACCT
1252412210-                  TTGCATCT-CCACTGTTGAGTTTCTCCACCTTCCTGGATCCTTTCTAGCATGTTCCACCT
1267622776-                  TTGCATCT-CCACTGTTGAGTTTCTCCACCTTTCCTGGATCCTTCTAGCATGTTCCACCT
1404858879-                  TTGCATCT-CCACTGCTGCGTTTCTCCACCTT-CCTGGATCCTTCTAGCATGTTCCACCT
1423366051-                  TTGCATCTACCACTGGTGAGTTTCTCCACCTTTCCTGGATC-ATCTAGCATGTTCCACCT
1400577008-                                                                            CT
                             ____________________________________________________________
consensus                    TTGCATCT-CCACTGCTGCGTTTCTCCACCTT-CCTGGATCCTTCTAGCATGTTCCACCT

                             .    :    .    :    .    :    .    :    .    :    .    :
1230149097-                  C
1219534330-                  CTGCTGGG-ACCATGAGGAACAGGAGCTGATGGGGTTGAGCTGCACATCCACGCTGCCTC
1230596782+                  CTGCTGGG-ACCATGAGGAACAGGAGCTGATGGGGTTGAGCTGCACATCCACGCTGCCTC
1231322579-                  CTGCTGGG-ACCATGAGGAACAGGAGCTGATGGGGTTGAGCTGCACATCCACACTGCCTC
1231538640-                  CTGCTGGG-ACCATGAGGAACAGGAGCTGATGGGGTTGAGCTGCACATCCACACTGCCTC
1252412210-                  CTGCTGGG-ACCATGAGGAACAGGAGCTGATGGGGTTGAGCTGCACATCCACGCTGCCTC
1267622776-                  CTGCTGGG-ACCATGAGGAACAGGAGCTGATGGGGTTGAGCTGCACATCCACGCTGCCTC
1400577008-                  CTGCTGGG-ACCATGAGGAACAGGAGCTGATGGGGTTGAGCTGCACATCCACGCTGCCTC
1404858879-                  CTGCTGGG-ACCATGAGGAACAGGAGCTGATGGGGTTGAGCTGCACATCCACACTGCCTC
1423366051-                  CTGCTGGGAACCATGAGGAACAGGAGCTGATGGGGTTGAGCTGCACATCCACGCTGCCTC
                             ____________________________________________________________
consensus                    CTGCTGGG-ACCATGAGGAACAGGAGCTGATGGGGTTGAGCTGCACATCCACGCTGCCTC

                             .    :    .    :    .    :    .    :    .    :    .    :
1404858879-                  ATCCACACTGTAGATTCAGCTATCATGGAGAGCTGCCTGGATC
1231322579-                  ATCCACACTGTAGATTCAGCTATCATGGAGAGCTGCCTGGATCTGACAT
1219534330-                  ATCCACACTGTAGATTCAGCTATCATGGAGAGCTGCCTGGATCTGACATTGTGACAGGAG
1230596782+                  ATCCACACTGTAGATTCAGCTATCATGGAGAGCTGCCTGGATCTGACATTGTGACAGGA-
1231538640-                  ATCCACACTGTAGATTCAGCTATCATGGAGAGCTGCCTGGATCTGACATTGTGACAGGAG
1252412210-                  ATCCACACTGTAGATTCAGCTATCATGGAGAGCTGCCTGGATCTGACATTGTGACAGGAG
1267622776-                  ATCCACACTGTAGATTCAGCTATCATGGAGAGCTGCCTGGATCTGACATTGTGACAGGAG
1400577008-                  ATCCACACTGTAGATTCAGCTATCATGGAGAGCTGCCTGGATCTGACATTGTGACAGGAG
1423366051-                  ATCCACACTGTAGATTCAGCTATCATGGAGAGCTGCCTGGATCTGACATTGTGACAGGAG
1389006827-                     CACACTGTAGATTCAGCTATCATGGAGAGCTGCCTGGATCTGACA-TGTGACAGGAG
1286248790-                                  CAGCTATCATGGAGAGCTGCCTGGATCTGACATTGTGACAGGAG
                             ____________________________________________________________
consensus                    ATCCACACTGTAGATTCAGCTATCATGGAGAGCTGCCTGGATCTGACATTGTGACAGGAG

                             .    :    .    :    .    :    .    :    .    :    .    :
1230596782+                  GTGCTTTCTGAAAT-GGCAGCATCTGCATCTCTgtaagttgaacagatat
1219534330-                  GTGCTTTCTGAAAT-GGCAGCATCTGCATCTCTgtaagttgaacagatatcctcttcatc
1231538640-                  GTGCTTTCTGAAAT-GGCAGCATCTGCATCTCTgtaagttgaacagatatcctcttcatc
1252412210-                  GTGCTTTCTGAAAT-GGCAGCATCTGCATCTCTgtaagttgaacagatatcctcttcatc
1267622776-                  GTGCTTTCTGAAAT-GGCAGCATCTGCATCTCTgtaagttgaacagatatcctcttcatc
1286248790-                  GTGCTTTCTGAAAT-GGCAGCATCTGCATCTCTgtaag-tgaacagatatcctct----c
```

```
1389006827-                GTGCTTTCTGAAAT-GGCAGCATCTGCATCTCTgtaagttgaacagatatcctcttcatc
1400577008-                GTGC-TTCTGAAAT-GGCAGCATCTGCATCTCTgtaagttgaacagatatcctcttcatc
1423366051-                GTGCTTTCTGAAATGGGCAGCATCTGCATCTCTgtaagttgaacagatatcctcttcatc
                           ____________________________________________________________
consensus                  GTGCTTTCTGAAAT-GGCAGCATCTGCATCTCTgtaagttgaacagatatcctcttcatc
```

G. Medium ground finch (No. 9), exon 1, WGS data

- CAP3 alignment of WGS data spanning exon 1 (uppercase letters)

```
                             .    :    .    :    .    :    .    :    .    :    .    :
SRR448675.100479892.1+   ctaactgcccaaggtatcagcaagacttggagactgagagATG
SRR448675.69549168.1+    ctaactgcccaaggtatcagcaagacttagagactgagagATG
SRR448681.82948658.1+    ctaactgcccaaggtatcagcaagacttggagactgagagATG
SRR448675.1007017.1+     ctaactgcccaaggtatcagcaagacttggagactgagagATGAT
SRR448675.5778478.1-     ctaactgcccaaggtatcagcaagacttggagactgagagATGAT
SRR448681.104812713.1-   ctaactgcccaaggtatcagcaagacttggagactgagagATGAT
SRR448681.113966051.1-   ctaactgcccaaggtatcagcaagacttggagactgagagATGATG
SRR448690.3092766.2+     ctaactgcccaaggtatcagcaagacttggagactgagagATGATGT
SRR448690.41354884.2+    ctaactgcccaaggtatcagcaagacttggagactgagagATGATGT
SRR448683.104535904.1+   ctaactgcccaaggtatcagcaagacttggagactgagagATGATGTT
SRR448688.92491247.2+    ctaactgcccaaggtatcagcaagacttggagactgagagATGATGTTG
SRR448686.103495169.2-   ctaactgcccaaggtatcagcaagacttggagactgagagATGATGTTGA
SRR448687.77379032.1-    ctaactgcccaaggtatcagcaagacttggagactgagagATGATGTTGAT
SRR448681.13943690.2+    ctaactgcccaaggtatcagcaagacttggaggctgagagATGATGTTGATG
SRR448681.90158569.2-    ctaactgcccaaggtatcagcaagacttggagactgagagATGATGTTGATG
SRR448683.93320995.1-    ctaactgcccaaggtatcagcaagacttggagactgagagATGATGTTGATG
SRR448686.26288542.2-    ctaactgcccaaggtatcagcaagacttggagactgagagATGATGTTGATG
SRR448686.27936370.2+    ctaactgcccaaggtatcagcaagacttggagactgagagATGATGTTGATG
SRR448686.49278550.2+    ctaactgcccaaggtatcagcaagacttggagactgagagATGATGTTGATG
SRR448687.51377636.1-    ctaactgcccaaggtatcagcaagacttggagactgagagATGATGTTGATG
SRR448681.14983340.1+    ctaactgcccaaggtatcagcaagacttggagactgagagATGATGTTGATGT
SRR448687.3421469.1-     ctaactgcccaaggtatcagcaagacttggagactgagagATGATGTTGATGT
SRR448686.31721987.2+    ctaactgcccaaggtatcagcaagacttggagactgagagATGATGTTGATGTT
SRR448686.95288214.2+    ctaactgcccaaggtatcagcaagacttggagactgagagATGATGTTGCTGTT
SRR448681.107485952.2-   ctaactgcccaaggtatcagcaagacttggagactgagagATGATGTTGATGTTC
SRR448683.91763429.1-    ctaactgcccaaggtatcagcaagacttggagactgagagATGATGTTGATGTTCT
SRR448687.500212.2-      ctaactgcccaaggtatcagcaagacttggagactgagagATGATGTTGATGTTCT
SRR448681.37781787.1-    ctaactgcccaaggtatcagcaagacttggagactgagagATGATGTTGATGTTCTT
SRR448675.105142019.2+   ctaactgcccaaggtatcagcaagacttggagactgagagATGATGTTGATGTTCTTCCC
SRR448675.30107343.1-    ctaactgcccaaggtatcagcaagacttggagactgagagATGATGTTGATGTTCTTCCC
SRR448675.34632732.2+    ctaactgcccaaggtatcagcaagacttggagactgagagATGATGTTGATGTTCTTCCC
SRR448675.47505994.2-    ctaactgcccaaggtatcagcaagacttggagactgagagATGATGTTGATGTTCTTCCC
SRR448675.48792349.2-    ctaactgcccaaggtatcagcaagacttggagactgagagATGATGTTGATGTTCTTCCC
SRR448675.59624787.1-    ctaactgcccaaggtatcagcaagacttggagactgagagATGATGTTGATGTTCTTCCC
SRR448675.61033335.1+    ctaactgcccaaggtatcagcaagacttggagactgagagATGATGTTGATGTTCTTCCC
SRR448675.61454794.1+    ctaactgcccaaggtatcagcaagacttggagactgagagATGATGTTGATGTTCTTCCC
SRR448675.68583665.1-    ctaactgcccaaggtatcagcaagacttggagactgagagATGATGTTGATGTTCTTCCC
SRR448675.73902527.2+    ctaactgcccaaggtatcagcaagacttggagactgagagATGATGTTGATGTTCTTCCC
SRR448675.76019197.1-    ctaactgcccaaggtatcagcaagacttggagactgagagATGATGTTGATGTTCTTCCC
SRR448675.89618126.2+    ctaactgcccaaggtatcagcaagacttggagactgagagATGATGTTGATGTTCTTCCC
SRR448675.91257091.1+    ctaactgcccaaggtatcagcaagacttggagactgagagATGATGTTGATGTTCTTCCC
SRR448681.100625135.2-   ctaactgcccaaggtatcagcaagacttggagactgagagATGATGTTGATGTTCTTCCC
SRR448681.103745259.1+   ctaactgcccaaggtatcagcaagacttggagactgagagATGATGTTGATGTTCTTCCC
SRR448681.114016387.2-   ctaactgcccaaggtatcagcaagacttggagactgagagATGATGTTGATGTTCTTCCC
SRR448681.24063966.1-    ctaactgcccaaggtatcagcaagacttggagactgagagATGATGTTGATGTTCTTCCC
SRR448681.4029888.1+     ctaactgcccaaggtatcagcaagacttggagactgagagATGATGTTGATGTTCTTCCC
SRR448681.59142577.1+    ctaactgcccaaggtatcagcaagacttggagactgagagATGATGTTGATGTTCTTCCC
SRR448681.66970561.2+    ctaactgcccaaggtatcagcaagacttggagactgagagATGATGTTGATGTTCTTCCC
SRR448681.70988830.1+    ctaactgcccaaggtatcagcaagacttggagactgagagATGATGTTGATGTTCTTCCC
SRR448681.7905433.2+     ctaactgcccaaggtatcagcaagacttggagactgagagATGATGTTGATGTTCTTCCC
SRR448681.80008393.2+    ctaactgcccaaggtatcagcaagacttggagactgagagATGATGTTGATGTTCTTCCC
SRR448681.80676895.1+    ctaactgcccaaggtatcagcaagacttggagactgagagATGATGTTGATGTTCTTCCC
SRR448681.8573045.1+     ctaactgcccaaggtatcggcaagacttggagactgagagATGATGTTGATGTTCTTCCC
SRR448681.94680159.2+    ctaactgcccaaggtatcagcaagacttggagactgagagATGATGTTGATGTTCTTCCC
SRR448681.98576512.1-    ctaactgcccaaggtatcagcaagacttggagactgagagATGATGTTGATGTTCTTCCC
SRR448683.100160277.1-   ctaactgcccaaggtatcagcaagacttggagactgagagATGATGTTGATGTTCTTCCC
SRR448683.36676326.2-    ctatctgcccaaggtatcagcaagacttggagactgagagATGATGTTTATGTTCTTCCC
SRR448683.37283289.2-    ctaactgcccaaggtatcagcaagacttggagactgagagATGATGTTGATGTTCTTCCC
SRR448683.39705.1+       ctaactgcccaaggtatcagcaagacttggagactgagagATGATGTTGATGTTCTTCCC
SRR448683.57387180.2+    ctaactgcccaaggtatcagcaagacttggagactgagagATGATGTTGATGTTCTTCCC
SRR448683.75617016.1-    ctaactgcccaaggtatcagcaagacttggagactgagagATGATGTTGATGTTCTTCCC
SRR448683.77248776.2-    ctaactgcccaaggtatcagcaagacttggagactgagagATGATGTTGATGTTCTTCCC
SRR448683.8327371.2+     ctaactgcccaaggtatcagcaagacttggagactgagagATGATGTTGATGTTCTTCCC
SRR448683.94768504.1-    ctaactgcccaaggtatcagcaagacttggagactgagagATGATGTTGATGTTCTTCCC
SRR448683.99125569.1-    ctaactgcccaaggtatcagcaagacttggagactgagagATGATGTTGATGTTCTTCCC
SRR448686.14816400.2+    ctaactgcccaaggtatcagcaagacttggagactgagagATGATGTTGATGTTCTTCCC
SRR448686.18360119.1-    ctaactgcccaaggtatcagcaagacttggagactgagagATGATGTTGATGTTCTTCCC
SRR448686.36602792.2+    ctaactgcccaaggtatcagcaagacttggagactgagagATGATGTTGATGTTCTTCCC
SRR448686.37906446.1+    ctaactgcccaaggtatcagcaagacttggagactgagagATGATGTTGATGTTCTTCCC
SRR448686.99095226.1-    ctaactgcccaaggtatcagcaagacttggagactgagagATGATGTTGATGTTCTTCCC
SRR448687.1959505.1-     ctaactgcccaaggtatcagcaagacttggagactgagagATGATGTTGATGTTCTTCCC
SRR448687.19667835.1-    ctaactgcccaaggtatcagcaagacttggagactgagagATGATGTTGATGTTCTTCCC
SRR448687.30037127.2+    ctaactgcccaaggtatcagcaagacttggagactgagagATGATGTTGATGTTCTTCCC
SRR448687.3580770.1-     ctaactgcccaaggtatcagcaagacttggagactgagagATGATGTTGATGTTCTTCCC
SRR448687.52301939.1-    ctaactgcccaaggtatcagcaagacttggagactgagagATGATGTTGATGTTCTTCCC
```

```
SRR448687.67270793.1+     ctaactgcccaaggtatcagcaagacttggagactgagagATGATGTTGATGTTCTTCCC
SRR448687.78219029.1+     ctaactgcccaaggaatcagcaagacttggagactgagagATGATGTTGATGTTCTTCCC
SRR448687.8466327.1+      ctaactgcccaaggtatcagcaagacttggagactgagagATGATGTTGATGTTCTTCCC
SRR448687.85478227.2-     ctaactgcccaaggtatcagcaagacttggagactgagagATGATGTTGATGTTCTTCCC
SRR448687.91479653.1-     ctaactgcccaaggtatcagcaagacttggagactgagagATGATGTTGATGTTCTTCCC
SRR448687.93646354.1+     ctaactgcccaaggtatcagcaagacttggagactgagagATGATGTTGATGTTCTTCCC
SRR448686.14112130.1+      taactgcccaaggtatcagcaagacttggagactgagagATGATGTTGATGTTCTTCCC
SRR448686.29349064.2-      taactgcccaaggtatcagcaagacttggagactgagagATGATGTTGATGTTCTTCCC
SRR448687.26806833.2+      taactgcccaaggtatcagcaagacttggagactgagagATGATGTTGATGTTTTTCCC
SRR448675.55807871.1-        actgcccaaggtatcagcaagacttggagactgagagATGATGTTGATGTTCTTCCC
SRR448686.58001144.2+        actgcccaaggtatcagcaagacttggagactgagagATGATGTTGATGTTCTTCCC
SRR448694.31414169.1-          tgcccaaggtatcagcaagacttggagactgagagATGATGTTGATGTT
SRR448675.100479892.2-         tgcccaaggtatcagcaagacttggagactgagagATGATGTTGATGTTCTTCCC
SRR448675.69549168.2-          tgcccaaggtatcagcaagacttagagactgagagATGATGTTGATGTTCTTCCC
SRR448681.51654459.2-          tgcccaaggtatcagcaagacttggagactgagagATGATGTTGATGTTCTTCCC
SRR448702.17838171.2+           gcccaaggtatcagcaagacttggagactgagagATGATGTTGATGTTC
SRR448702.22794598.2+           gcccaaggtatcagcaagacttggagactgagagATGATGTTGATGTTC
SRR448702.25453408.2+           gcccaaggtatcagcaagacttggagactgagagATGATGTTGATGTTC
SRR448702.3330112.2+            gcccaaggtatcagcaagacttggagactgagagATGATGTTGATGTTC
SRR448702.45203147.2+           gcccaaggtatcagcaagacttggagactgagagATGATGTTGATGTTC
SRR448702.51353857.2+           gcccaaggtatcagcaagacttggagactgagagATGATGTTGATGTTC
SRR448702.52718704.2+           gcccaaggtatcagcaagacttggagactgagagATGATGTTGATGTTC
SRR448702.54146257.2+           gcccaaggtatcagcaagacttggagactgagagATGATGTTGATGTTC
SRR448702.88727150.2+           gcccaaggtatcagcaagacttggagactgagagATGATGTTGATGTTC
SRR448681.111896412.2-          gcccaaggtatcagcaagacttggagactgagagATGATGTTGATGTTCTTCCC
SRR448683.85289444.2+           gcccaaggtatcagcaagacttggagactgagagATGATGTTGATGTTCTTCCC
SRR448675.24488131.1+            cccaaggtatcagcaagacttggagactgagagATGATGTTGATGTTCTTCCC
SRR448681.22542882.2+            cccaaggtatcagcaagacttggagactgagagATGATGTTGATGTTCTTCCC
SRR448690.74370714.1-             ccaaggtatcagcaagacttggagactgagagATGATGTTGATGTTCTT
SRR448690.94429022.1-             ccaaggtatcagcaagacttggagactgagagATGATGTTGATGTTCTT
SRR448694.63844295.1+              caaggtatcagcaagacttggagactgagagATGATGTTGATGTTCTTC
SRR448694.7228368.1+               caaggtatcagcaagacttggagactgagagATGATGTTGATGTTCTTC
SRR448683.80039025.1+              caaggtatcagcaagacttggagactgagagATGATGTTGATGTTCTTCCC
SRR448702.15399809.2+               aaggtatcagcaagacttggagactgagagATGATGTTGATGTTCTTCC
SRR448702.24642302.2+               aaggtatcagcaagacttggagactgagagATGATGTTGATGTTCTTCC
SRR448702.37895200.2+               aaggtatcagcaagacttggagactgagagATGATGTTGATGTTCTTCC
SRR448702.38797500.2+               aaggtatcagcaagacttggagactgagagATGATGTTGATGTTCTTCC
SRR448702.48115865.2+               aaggtatcagcaagacttggagactgagagATGATGTTGATGTTCTTCC
SRR448702.87787709.2+               aaggtatcagcaagacttggagactgagagATGATGTTGATGTTCTTCC
SRR448702.91460362.2+               aaggtatcagcaagacttggagactgagagATGATGTTGATGTTCTTCC
SRR448702.93050215.2+               aaggtatcagcaagacttggagactgagagATGATGTTGATGTTCTTCC
SRR448702.94097808.2+               aaggtatcagcaagacttggagactgagagATGATGTTGATGTTCTTCC
SRR448681.68433421.1-               aaggtatcagcaagacttggagactgagagATGATGTTGATGTTCTTCCC
SRR448683.12657253.1+               aaggtatcagcaagacttggagactgagagATGATGTTGATGTTCTTCCC
SRR448687.27436536.1-               aaggtatcagcaagacttggagactgagagATGATGTTGATGTTCTTCCC
SRR448681.82948658.2-                aggtatcagcaagacttggagactgagagATGATGTTGATGTTCTTCCC
SRR448681.107967200.2-                 gtatcagcaagacttggagactgagagATGATGTTGATGTTCTTCCC
SRR448686.102284400.1+                 gtatcagcaagacttggagactgagagATGATGTTGATGTTCTTCCC
SRR448687.54908066.2+                   tatcagcaagacttggagactgagagATGATGTTGATGTTCTTCCC
SRR448675.26299051.2+                    atcagcaagacttggagactgagagATGATGTTGATGTTCTTCCC
SRR448683.38738038.2+                    atcagcaagacttggagactgagagATGATGTTGATGTTCTTCCC
SRR448683.85263611.2+                    atcagcaagacttggagactgagagATGATGTTGATGTTCTTCCC
SRR448686.92859673.1+                    atcagcaagacttggagactgagagATGATGTTGATGTTCTTCCC
SRR448688.1339075.2+                     atcagcaagacttggagactgagagATGATGTTGATGTTCTTCCC
SRR448688.37172419.2+                    atcagcaagacttggagactgagagATGATGTTGATGTTCTTCCC
SRR448688.39785364.2+                    atcagcaagacttggagactgagagATGATGTTGATGTTCTTCCC
SRR448702.21178808.2-                    atcagcaagacttggagactgagagATGATGTTGATGTTCTTCCC
SRR448702.32548642.2-                    atcagcaagacttggagactgagagATGATGTTGATGTTCTTCCC
SRR448702.35387138.2-                    atcagcaagacttggagactgagagATGATGTTGATGTTCTTCCC
SRR448702.52659794.2-                    atcagcaagacttggagactgagagATGATGTTGATGTTCTTCCC
SRR448702.57467735.2-                    atcagcaagacttggagactgagagATGATGTTGATGTTCTTCCC
SRR448702.60595664.2-                    atcagcaagacttggagactgagagATGATGTTGATGTTCTTCCC
SRR448702.86000697.2-                    atcagcaagacttggagactgagagATGATGTTGATGTTCTTCCC
SRR448702.91690059.2-                    atcagcaagacttggagactgagagATGATGTTGATGTTCTTCCC
SRR448694.50680781.1-                      cagcaagacttggagactgagagATGATGTTGATGTTCTTCCC
SRR448699.92483900.2-                      cagcaagacttggagactgagagATGATGTTGATGTTCTTCCC
SRR448675.1007017.2-                        agcaagacttggagactgagagATGATGTTGATGTTCTTCCC
SRR448683.26530291.1-                       agcaagacttggagactgagagATGATGTTGATGTTCTTCCC
SRR448686.37030654.1+                       agcaagacttggagactgagagATGATGTTGATGTTCTTCCC
SRR448686.96851970.2-                       agcaagacttggagactgagagATGATGTTGATGTTCTTCCC
SRR448688.29503588.2-                       agcaagacttggagactgagagATGATGTTGATGTTCTTCCC
SRR448681.14983340.2-                        gcaagacttggagactgagagATGATGTTGATGTTCTTCCC
SRR448683.99769950.2-                         caagacttggagactgagagATGATGTTGATGTTCTTCCC
SRR448687.16463223.1+                          aagacttggagactgagagATGATGTTGATGTTCTTCCC
SRR448694.109946173.2+                         aagacttggagactgagagATGATGTTGATGTTCTTCCC
SRR448688.27885175.1+                           agacttggagactgagagATGATGTTGATGTTCTTCCC
SRR448699.30571204.2+                            gacttggagactgagagATGATGTTGATGTTCTTCCC
SRR448686.86332807.2-                              cttggagactgagagATGATGTTGATGTTCTTCCC
SRR448687.85324970.2-                               ttggagactgagagATGATGTTGATGTTCTTCCC
SRR448702.3724550.1+                                ttggagactgagagATGATGTTGATGTTCTTCCC
SRR448702.50322092.1+                               ttggagactgagagATGATGTTGATGTTCTTCCC
SRR448702.51265716.1+                               ttggagactgagagATGATGTTGATGTTCTTCCC
SRR448702.57520481.1+                               ttggagactgagagATGATGTTGATGTTCTTCCC
SRR448702.59530418.1+                               ttggagactgagagATGATGTTGATGTTCTTCCC
```

SRR448702.72123323.1+	ttggagactgagagATGATGTTGATGTTCTTCCC
SRR448702.81468057.1+	ttggagactgagagATGATGTTGATGTTCTTCCC
SRR448702.82704114.1+	ttggagactgagagATGATGTTGATGTTCTTCCC
SRR448694.5380521.2-	tggagactgagagATGATGTTGATGTTCTTCCC
SRR448694.55971147.2-	tggagactgagagATGATGTTGATGTTCTTCCC
SRR448694.65213546.2-	tggagactgagagATGATGTTGATGTTCTTCCC
SRR448699.43913294.2-	tggagactgagagATGATGTTGATGTTCTTCCC
SRR448681.13943690.1-	ggagactgagagATGATGTTGATGTTCTTCCC
SRR448699.111251328.1-	ggagactgagagATGATGTTGATGTTCTTCCC
SRR448699.17531339.1-	ggagactgagagATGATGTTGATGTTCTTCCC
SRR448699.87618487.1-	ggagactgagagATGATGTTGATGTTCTTCCC
SRR448699.93424159.1-	ggagactgagagATGATGTTGATGTTCTTCCC
SRR448699.94858830.2+	ggagactgagagATGATGTTGATGTTCTTCCC
SRR448699.95214740.1-	ggagactgagagATGATGTTGATGTTCTTCCC
SRR448687.40599867.1-	gagactgagagATGATGTTGATGTTCTTCCC
SRR448690.28845541.1-	gagactgagagATGATGTTGATGTTCTTCCC
SRR448690.63910124.1-	gagactgagagATGATGTTGATGTTCTTCCC
SRR448690.83247711.1-	gagactgagagATGATGTTGATGTTCTTCCC
SRR448694.50469539.1+	gagactgagagATGATGTTGATGTTCTTCCC
SRR448675.54641135.2+	agactgagagATGATGTTGATGTTCTTCCC
SRR448683.77711223.2+	actgagagATGATGTTGATGTTCTTCCC
SRR448686.116154652.1-	actgagagATGATGTTGATGTTCTTCCC
SRR448702.10099410.1-	actgagagATGATGTTGATGTTCTTCCC
SRR448702.16389027.1-	actgagagATGATGTTGATGTTCTTCCC
SRR448702.19024048.1-	actgagagATGATGTTGATGTTCTTCCC
SRR448702.26223669.1-	actgagagATGATGTTGATGTTCTTCCC
SRR448702.26905409.1-	actgagagATGATGTTGATGTTCTTCCC
SRR448702.37178889.1-	actgagagATGATGTTGATGTTCTTCCC
SRR448702.38429770.1-	actgagagATGATGTTGATGTTCTTCCC
SRR448702.39094754.1-	actgagagATGATGTTGATGTTCTTCCC
SRR448702.42431471.1-	actgagagATGATGTTGATGTTCTTCCC
SRR448702.63301725.1-	actgagagATGATGTTGATGTTCTTCCC
SRR448702.72901452.1-	actgagagATGATGTTGATGTTCTTCCC
SRR448702.81094837.1-	actgagagATGATGTTGATGTTCTTCCC
SRR448702.81330689.1-	actgagagATGATGTTGATGTTCTTCCC
SRR448702.83257465.1-	actgagagATGATGTTGATGTTCTTCCC
SRR448702.93112878.1-	actgagagATGATGTTGATGTTCTTCCC
SRR448681.12918655.1+	tgagagATGATGTTGATGTTCTTCCC
SRR448683.63839307.1+	tgagagATGATGTTGATGTTCTTCCC
SRR448683.82663094.2-	gagagATGATGTTGATGTTCTTCCC
SRR448681.103745259.2-	agagATGATGTTGATGTTCTTCCC
SRR448681.66970561.1-	agagATGATGTTGATGTTCTTCCC
SRR448686.112672816.2-	agagATGATGTTGATGTTCTTCCC
SRR448688.33222151.1-	agagATGATGTTGATGTTCTTCCC
SRR448688.48422888.2-	agagATGATGTTGATGTTCTTCCC
SRR448688.5754810.1-	agagATGATGTTGATGTTCTTCCC
SRR448688.60065196.1-	agagATGATGTTGATGTTCTTCCC
SRR448688.71027850.1-	agagATGATGTTGATGTTCTTCCC
SRR448694.37023895.1+	agagATGATGTTGATGTTCTTCCC
SRR448694.82493274.2-	agagATGATGTTGATGTTCTTCCC
SRR448675.61454794.2-	gagATGATGTTGATGTTCTTCCC
SRR448681.9386500.2+	gagATGATGTTGATGTTCTTCCC
SRR448694.28589359.1-	gagATGATGTTGATGTTCTTCCC
SRR448690.2512208.2+	agATGATGTTGATGTTCTTCCC
SRR448675.82235779.1+	ATGATGTTGATGTTCTTCCC
SRR448694.18700495.1+	ATGATGTTGATGTTCTTCCC
SRR448675.37623604.2+	GATGTTGATGTTCTTCCC
SRR448681.70988830.2-	GATGTTGATGTTCTTCCC
SRR448675.105142019.1-	TGTTGATGTTCTTCCC
SRR448681.80008393.1-	TGTTGATGTTCTTCCC
SRR448686.109419161.1-	TGTTGATGTTCTTCCC
SRR448675.73902527.1-	GTTGATGTTCTTCCC
SRR448683.11200816.2-	GTTGATGTTCTTCCC
SRR448694.7585788.1-	GTTGATGTTCTTCCC
SRR448694.87477746.2-	GTTGATGTTCTTCCC
SRR448699.85949089.2+	GTTGATGTTCTTCCC
SRR448681.59142577.2-	TTGATGTTCTTCCC
SRR448699.11831588.1-	GATGTTCTTCCC
SRR448699.59187151.2-	GATGTTCTTCCC
SRR448702.7291062.2+	GATGTTCTTCCC
SRR448675.24551560.1+	TGTTCTTCCC
SRR448675.34632732.1-	GTTCTTCCC
SRR448687.49807054.1+	GTTCTTCCC
SRR448687.78234188.2+	TTCTTCCC
SRR448699.78372155.2-	TTCTTCCC
SRR448681.4029888.2-	TCTTCCC
SRR448690.52210119.2+	TCTTCCC
SRR448690.7743621.2+	TCTTCCC
SRR448699.104884924.1+	TCTTCCC
SRR448688.56502178.2+	CTTCCC
SRR448699.26148630.2-	CTTCCC
SRR448686.105571365.2+	TTCCC
SRR448702.17456697.1-	TTCCC
SRR448702.28402283.1-	TTCCC

```
SRR448702.6523881.1-                                                                TTCCC
SRR448702.82938393.1-                                                               TTCCC
SRR448702.99898637.1-                                                               TTCCC
SRR448686.102868232.2-                                                               TCCC
SRR448675.61033335.2-                                                                 CCC
SRR448675.91257091.2-                                                                 CCC
SRR448686.41318453.1-                                                                 CCC
SRR448687.55441481.1+                                                                 CCC
SRR448687.68896953.1+                                                                  CC
SRR448694.100352330.2-                                                                 CC
SRR448681.88931306.1+                                                                   C
SRR448683.69969975.2+                                                                   C

consensus                ctaactgcccaaggtatcagcaagacttggagactgagagATGATGTTGATGTTCTTCCC

                             .    :    .    :    .    :    .    :    .    :    .    :
SRR448683.36676326.2-    A
SRR448675.30107343.1-    AGG
SRR448681.98576512.1-    AGG
SRR448683.8327371.2+     AGG
SRR448675.76019197.1-    AGGA
SRR448688.1339075.2+     AGGA
SRR448688.37172419.2+    AGGA
SRR448688.39785364.2+    AGGA
SRR448702.21178808.2-    AGGA
SRR448702.32548642.2-    AGGA
SRR448702.35387138.2-    AGGA
SRR448702.52659794.2-    AGGA
SRR448702.57467735.2-    AGGA
SRR448702.60595664.2-    AGGA
SRR448702.86000697.2-    AGGA
SRR448702.91690059.2-    AGGA
SRR448675.68583665.1-    AGGAA
SRR448687.8466327.1+     AGGAA
SRR448681.100625135.2-   AGGAAT
SRR448694.50680781.1-    AGGAAT
SRR448699.92483900.2-    AGGAAT
SRR448688.29503588.2-    AGGAATC
SRR448686.37906446.1+    AGGAATCA
SRR448687.30037127.2+    AGGAATCA
SRR448675.47505994.2-    AGGAATCAAG
SRR448681.114016387.2-   AGGAATCAAG
SRR448683.100160277.1-   AGGAATCAAG
SRR448683.57387180.2+    AGGAATCAAG
SRR448694.109946173.2+   AGGAATCAAG
SRR448681.59142577.1+    AGGAATCAAGA
SRR448688.27885175.1+    AGGAATCAAGA
SRR448675.105142019.2+   AGGAATCAAGAG
SRR448675.61454794.1+    AGGAATCAAGAG
SRR448675.73902527.2+    AGGAATCAAGAG
SRR448681.66970561.2+    AGGAATCAAGAG
SRR448699.30571204.2+    AGGAATCAAGAG
SRR448686.36602792.2+    AGGAATCAAGAGAAT
SRR448687.52301939.1-    AGGAATCAAGAGAAT
SRR448702.3724550.1+     AGGAATCAAGAGAAT
SRR448702.50322092.1+    AGGAATCAAGAGAAT
SRR448702.51265716.1+    AGGAATCAAGAGAAT
SRR448702.57520481.1+    AGGAATCAAGAGAAT
SRR448702.59530418.1+    AGGAATCAAGAGAAT
SRR448702.72123323.1+    AGGAATCAAGAGAAT
SRR448702.81468057.1+    AGGAATCAAGAGAAT
SRR448702.82704114.1+    AGGAATCAAGAGAAT
SRR448675.59624787.1-    AGGAATCAAGAGAATG
SRR448694.5380521.2-     AGGAATCAAGAGAATG
SRR448694.55971147.2-    AGGAATCAAGAGAATG
SRR448694.65213546.2-    AGGAATCAAGAGAATG
SRR448699.43913294.2-    AGGAATCAAGAGAATG
SRR448681.70988830.1+    AGGAATCAAGAGAATGC
SRR448699.111251328.1-   AGGAATCAAGAGAATGC
SRR448699.17531339.1-    AGGAATCAAGAGAATGC
SRR448699.87618487.1-    AGGAATCAAGAGAATGC
SRR448699.93424159.1-    AGGAATCAAGAGAATGC
SRR448699.94858830.2+    AGGAATCAAGAGAATGC
SRR448699.95214740.1-    AGGAATCAAGAGAATGC
SRR448683.39705.1+       AGGAATCAAGAGAATGCT
SRR448687.93646354.1+    AGGAATCAAGAGAATGCT
SRR448690.28845541.1-    AGGAATCAAGAGAATGCT
SRR448690.63910124.1-    AGGAATCAAGAGAATGCT
SRR448690.83247711.1-    AGGAATCAAGAGAATGCT
SRR448694.50469539.1+    AGGAATCAAGAGAATGCT
SRR448675.34632732.2+    AGGAATCAAGAGAATGCTC
SRR448681.24063966.1-    AGGAATCAAGAGAATGCTC
SRR448681.8573045.1+     AGGAATCAAGAGAATGCTCT
SRR448683.99125569.1-    AGGAATCAAGAGAATGCTCT
```

SRR448702.10099410.1-   AGGAATCAAGAGAATGCTCTT
SRR448702.16389027.1-   AGGAATCAAGAGAATGCTCTT
SRR448702.19024048.1-   AGGAATCAAGAGAATGCTCTT
SRR448702.26223669.1-   AGGAATCAAGAGAATGCTCTT
SRR448702.26905409.1-   AGGAATCAAGAGAATGCTCTT
SRR448702.37178889.1-   AGGAATCAAGAGAATGCTCTT
SRR448702.38429770.1-   AGGAATCAAGAGAATGCTCTT
SRR448702.39094754.1-   AGGAATCAAGAGAATGCTCTT
SRR448702.42431471.1-   AGGAATCAAGAGAATGCTCTT
SRR448702.63301725.1-   AGGAATCAAGAGAATGCTCTT
SRR448702.72901452.1-   AGGAATCAAGAGAATGCTCTT
SRR448702.81094837.1-   AGGAATCAAGAGAATGCTCTT
SRR448702.81330689.1-   AGGAATCAAGAGAATGCTCTT
SRR448702.83257465.1-   AGGAATCAAGAGAATGCTCTT
SRR448702.93112878.1-   AGGAATCAAGAGAATGCTCTT
SRR448687.3580770.1-   AGGAATCAAGAGAATGCTCTTC
SRR448688.33222151.1-   AGGAATCAAGAGAATGCTCTTCCTC
SRR448688.48422888.2-   AGGAATCAAGAGAATGCTCTTCCTC
SRR448688.5754810.1-   AGGAATCAAGAGAATGCTCTTCCTC
SRR448688.60065196.1-   AGGAATCAAGAGAATGCTCTTCCTC
SRR448688.71027850.1-   AGGAATCAAGAGAATGCTCTTCCTC
SRR448694.37023895.1+   AGGAATCAAGAGAATGCTCTTCCTC
SRR448694.82493274.2-   AGGAATCAAGAGAATGCTCTTCCTC
SRR448675.89618126.2+   AGGAATCAAGAGAATGCTCTTCCTCT
SRR448694.28589359.1-   AGGAATCAAGAGAATGCTCTTCCTCT
SRR448681.4029888.1+   AGGAATCAAGAGAATGCTCTTCCTCTT
SRR448690.2512208.2+   AGGAATCAAGAGAATGCTCTTCCTCTT
SRR448675.48792349.2-   AGGAATCAAGAGAATGCTCTTCCTCTTG
SRR448675.91257091.1+   AGGAATCAAGAGAATGCTCTTCCTCTTG
SRR448683.37283289.2-   AGGAATCAAGAGAATGCTCTTCCTCTTG
SRR448683.94768504.1-   AGGAATCAAGAGAATGCTCTTCCTCTTG
SRR448686.18360119.1-   AGGAATCAAGAGAATGCTCTTCCTCTTG
SRR448687.78219029.1+   AGGAATCAAGAGAATGCTCTTCCTCTTG
SRR448694.18700495.1+   AGGAATCAAGAGAATGCTCTTCCTCTTGT
SRR448686.14816400.2+   AGGAATCAAGAGAATGCTCTTCCTCTTGTTC
SRR448687.91479653.1-   AGGAATCAAGAGAATGCTCTTCCTCTTGTTC
SRR448675.61033335.1+   AGGAATCAAGAGAATGCTCTTCCTCTTGTTCC
SRR448687.67270793.1+   AGGAATCAAGAGAATGCTCTTCCTCTTGTTCCTC
SRR448694.7585788.1-   AGGAATCAAGAGAATGCTCTTCCTCTTGTTCCTC
SRR448694.87477746.2-   AGGAATCAAGAGAATGCTCTTCCTCTTGTTCCTC
SRR448699.85949089.2+   AGGAATCAAGAGAATGCTCTTCCTCTTGTTCCTC
SRR448681.94680159.2+   AGGAATCAAGAGAATGCTCTTCCTCTTGTTCCTCC
SRR448683.75617016.1-   AGGAATCAAGAGAATGCTCTTCCTCTTGTTCCTCC
SRR448681.7905433.2+   AGGAATCAAGAGAATGCTCTTCCTCTTGTTCCTCCC
SRR448683.77248776.2-   AGGAATCAAGAGAATGCTCTTCCTCTTGTTCCTCCC
SRR448687.26806833.2+   AGGAATCAAGAGAATGCTCTTACTCTTGTTCATCCC
SRR448702.7291062.2+   AGGAATCAAGAGAATGCTCTTCCTCTTGTTCCTCCC
SRR448699.11831588.1-   AGGAATCAAGAGAATGCTCTTCCTCTTGTTCCTCCCA
SRR448699.59187151.2-   AGGAATCAAGAGAATGCTCTTCCTCTTGTTCCTCCCA
SRR448681.80676895.1+   AGGAATCAAGAGAATGCTCTTCCTCTTGTTCCTCCCAT
SRR448687.19667835.1-   AGGAATCAAGAGAATGCTCTTCCTCTTGTTCCTCCCAT
SRR448687.85478227.2-   AGGAATCAAGAGAATGCTCTTCCTCTTGTTCCTCCCAT
SRR448687.1959505.1-   AGGAATCAAGAGAATGCTCTTCCTCTTGTTCCTCCCATG
SRR448686.14112130.1+   AGGAATCAAGAGAATGCTCTTCCTCTTGTTCCTCCCATGCT
SRR448686.29349064.2-   AGGAATCAAGAGAATGCTCTTCCTCTTGTTCCTCCCATGCT
SRR448699.78372155.2-   AGGAATCAAGAGAATGCTCTTCCTCTTGTTCCTCCCATGCT
SRR448690.52210119.2+   AGGAATCAAGAGAATGCTCTTCCTCTTGTTCCTCCCATGCTT
SRR448690.7743621.2+   AGGAATCAAGAGAATGCTCTTCCTCTTGTTCCTCCCATGCTT
SRR448699.104884924.1+   AGGAATCAAGAGAATGCTCTTCCTCTTGTTCCTCCCATGCTT
SRR448675.55807871.1-   AGGAATCAAGAGAATGCTCTTCCTCTTGTTCCTCCCATGCTTT
SRR448686.58001144.2+   AGGAATCAAGAGAATGCTCTTCCTCTTGTTCCTCCCATGCTTT
SRR448688.56502178.2+   AGGAATCAAGAGAATGCTCTTCCTCTTGTTCCTCCCATGCTTT
SRR448699.26148630.2-   AGGAATCAAGAGAATGCTCTTCCTCTTGTTCCTCCCATGCTTT
SRR448702.17456697.1-   AGGAATCAAGAGAATGCTCTTCCTCTTGTTCCTCCCATGCTTTT
SRR448702.28402283.1-   AGGAATCAAGAGAATGCTCTTCCTCTTGTTCCTCCCATGCTTTT
SRR448702.6523881.1-   AGGAATCAAGAGAATGCTCTTCCTCTTGTTCCTCCCATGCTTTT
SRR448702.82938393.1-   AGGAATCAAGAGAATGCTCTTCCTCTTGTTCCTCCCATGCTTTT
SRR448702.99898637.1-   AGGAATCAAGAGAATGCTCTTCCTCTTGTTCCTCCCATGCTTTT
SRR448675.100479892.2-   AGGAATCAAGAGAATGCTCTTCCTCTTGTTCCTCCCATGCTTTTG
SRR448675.69549168.2-   AGGAATCAAGAGAATGCTCTTCCTCTTGTTCCTCCCATGCTTTTG
SRR448681.51654459.2-   AGGAATCAAGAGAATGCTCTTCCTCTTGTTCCTCCCATGCTTTTG
SRR448681.111896412.2-   AGGAATCAAGAGAATGCTCTTCCTCTTGTTCCTCCCATGCTTTTGT
SRR448683.85289444.2+   AGGAATCAAGAGAATGCTCTTCCTCTTGTTCCTCCCATGCTTTTGT
SRR448675.24488131.1+   AGGAATCAAGAGAATGCTCTTCCTCTTGTTCCTCCCATGCTTTTGTT
SRR448681.22542882.2+   AGGAATCAAGAGAATGCTCTTCCTCTCGTTCCTCCCATGCTTTTGTT
SRR448694.100352330.2-   AGGAATCAAGAGAATGCTCTTCCTCTTGTTCCTCCCATGCTTTTGTT
SRR448683.80039025.1+   AGGAATCAAGAGAATGCTCTTCCTCTTGTTCCTCCCATGCTTTTGTTCT
SRR448688.24392558.1+   AGGAATCAAGAGAATGCTCTTCCTCTTGTTCCTCCCATGCTTTTGTTCT
SRR448688.70490207.1+   AGGAATCAAGAGAATGCTCTTCCTCTTGTTCCTCCCATGCTTTTGTTCT
SRR448688.79793784.1+   AGGAATCAAGAGAATGCTCTTCCTCTTGTTCCTCCCATGCTTTTGTTCT
SRR448688.85235348.1+   AGGAATCAAGAGAATGCTCTTCCTCTTGTTCCTCCCATGCTTTTGTTCT
SRR448681.68433421.1-   AGGAATCAAGAGAATGCTCTTCCTCTTGTTCCTCCCATGCTTTTGTTCTG
SRR448683.12657253.1+   AGGAATCAAGAGAATGCTCTTCCTCTTGTTCCTCCCATGCTTTTGTTCTG
SRR448687.27436536.1-   AGGAATCAAGAGAATGCTCTTCCTCTTGTTCCTCCCATGCTTTTGTTCTG

```
SRR448681.82948658.2-    AGGAATCAAGAGAATGCTCTTCCTCTTGTTCCTCCCATGCTTTTGTTCTGG
SRR448681.107967200.2-   AGGAATCAAGAGAATGCTCTTCCTCTTGTTCCTCCCATGCTTTTGTTCTGGCC
SRR448686.102284400.1+   AGGAATCAAGAGAATGCTCTTCCTCTTGTTCCTCCCATGCTTTTGTTCTGGCC
SRR448687.54908066.2+    AGGAATCAAGAGAATGCTCTTCCTCTTGTTCCTCCCATGCTTTTGTTCTGGCCA
SRR448675.26299051.2+    AGGAATCAAGAGAATGCTCTTCCTCTTGTTCCTCCCATGCTTTTGTTCTGGCCAG
SRR448683.38738038.2+    AGGAATCAAGAGAATGCTCTTCCTCTTGTTCCTCCCATGCTTTTGTTCTGGCCAG
SRR448683.85263611.2+    AGGAATCAAGAGAATGCTCTTCCTCTTGTTCCTCCCATGCTTTTGTTCTGGCCAG
SRR448686.92859673.1+    AGGAATCAAGAGAATGCTCTTCCTCTTGTTCCTCCCATGCTTTTGTTCTGGCCAG
SRR448675.1007017.2-     AGGAATCAAGAGAATGCTCTTCCTCTTGTTCCTCCCATGCTTTTGTTCTGGCCAGCTT
SRR448683.26530291.1-    AGGAATCAAGAGAATGCTCTTCCTCTTGTTCCTCCCATGCTTTTGTTCTGGCCAGCTT
SRR448686.37030654.1+    AGGAATCAAGAGAATGCTCTTCCTCTTGTTCCTCCCATGCTTTTGTTCTGGCCAGCTT
SRR448686.96851970.2-    AGGAATCAAGAGAATGCTCTTCCTCTTGTTCCTCCCATGCTTTTGTTCTGGCCAGCTT
SRR448681.14983340.2-    AGGAATCAAGAGAATGCTCTTCCTCTTGTTCCTCCCATGCTTTTGTTCTGGCCAGCTTG
SRR448675.105142019.1-   AGGAATCAAGAGAATGCTCTTCCTCTTGTTCCTCCCATGCTTTTGTTCTGGCCAGCTTGC
SRR448675.24551560.1+    AGGAATCAAGAGAATGCTCTTCCTCTTGTTCCTCCCATGCTTTTGTTCTGGCCAGCTTGC
SRR448675.34632732.1-    AGGAATCAAGAGAATGCTCTTCCTCTTGTTCCTCCCATGCTTTTGTTCTGGCCAGCTTGC
SRR448675.37623604.2+    AGGAATCAAGAGAATGCTCTTCCTCTTGTTCCTCCCATGCTTTTGTTCTGGCCAGCTTGC
SRR448675.54641135.2+    AGGAATCAAGAGAATGCTCTTCCTCTTGTTCCTCCCATGCTTTTGTTCTGGCCAGCTTGC
SRR448675.61033335.2-    AGGAATCAAGAGAATGCTCTTCCTCTTGTTCCTCCCATGCTTTTGTTCTGGCCAGCTTGC
SRR448675.61454794.2-    AGGAATCAAGAGAATGCTCTTCCTCTTGTTCCTCCCATGCTTTTGTTCTGGCCAGCTTGC
SRR448675.73902527.1-    AGGAATCAAGAGAATGCTCTTCCTCTTGTTCCTCCCATGCTTTTGTTCTGGCCAGCTTGC
SRR448675.82235779.1+    AGGAATCAAGAGAATGCTCTTCCTCTTGTTCCTCCCATGCTTTTGTTCTGGCCAGCTTGC
SRR448675.91257091.2-    AGGAATCAAGAAAATGCTCTTCCTCTTGTTCCTCCCATGCTTTTGTTCTGGCCAGCTTGC
SRR448681.103745259.2-   AGGAATCAAGAGAATGCTCTTCCTCTTGTTCCTCCCATGCTTTTGTTCTGGCCAGCTTGC
SRR448681.12918655.1+    AGGAATCAAGAGAATGCTCTTCCTCTTGTTCCTCCCATGCTTTTGTTCTGGCCAGCTTGC
SRR448681.13943690.1-    AGGAATCAAGAGAATGCTCTTCCTCTTGTTCCTCCCATGCTTTTGTTCTGGCCAGCTTGC
SRR448681.4029888.2-     AGGAATCAAGAGAATGCTCTTCCTCTTGTTCCTCCCATGCTTTTGTTCTGGCCAGCTTGC
SRR448681.59142577.2-    AGGAATCAAGAGAATGCTCTTCCTCTTGTTCCTCCCATGCTTTTGTTCTGGCCAGCTTGC
SRR448681.66970561.1-    AGGAATCAAGAGAATGCTCTTCCTCTTGTTCCTCCCATGCTTTTGTTCTGGCCAGCTTGC
SRR448681.70988830.2-    AGGAATCAAGAGAATGCTCTTCCTCTTGTTCCTCCCATGCTTTTGTTCTGGCCAGCTTGC
SRR448681.80008393.1-    AGGAATCAAGAGAATGCTCTTCCTCTTGTTCCTCCCATGCTTTTGTTCTGGCCAGCTTGC
SRR448681.88931306.1+    AGGAATCAAGAGAATGCTCTTCCTCTTGTTCCTCCCATGCTTTTGTTCTGGCCAGCTTGC
SRR448681.9386500.2+     AGGAATCAAGAGAATGCTCTTCCTCTTGTTCCTCCCATGCTTTTGTTCTGGCCAGCTTGC
SRR448683.11200816.2-    AGGAATCAAGAGAATGCTCTTCCTCTTGTTCCTCCCATGCTTTTGTTCTGGCCAGCTTGC
SRR448683.63839307.1+    AGGAATCAAGAGAATGCTCTTCCTCTTGTTCCTCCCATGCTTTTGTTCTGGCCAGCTTGC
SRR448683.69969075.2+    AGGAATCAAGAGAATGCTCTTCCTCTTGTTCCTCCCATGCTTTTGTTCTGGCCAGCTTGC
SRR448683.77711223.2+    AGGAATCAAGAGAATGATCTTACTCTTGTTCCTCCCATGCTTTTGTTCTGGCCAGCTTGC
SRR448683.82663094.2-    AGGAATCAAGAGAATGCTCTTCCTCTTGTTCCTCCCATGCTTTTGTTCTGGCCAGCTTGC
SRR448683.99769950.2-    AGGAATCAAGAGAATGCTCTTCCTCTTGTTCCTCCCATGCTTTTGTTCTGGCCAGCTTGC
SRR448686.102868232.2-   AGGAATCAAGAGAATGCTCTTCCTCTTGTTCCTCCCATGCTTTTGTTCTGGCCAGCTTGC
SRR448686.105571365.2+   AGGAATCAAGAGAATGCTCTTCCTCTTGTTCCTCCCATGCTTTTGTTCTGGCCAGCTTGC
SRR448686.106701110.2+   AGGAATCAAGAGAATGCTCTTCCTCTTGTTCCTCCCATGCTTTTGTTCTGGCCAGCTTGC
SRR448686.109419161.1-   AGGAATCAAGAGAATGCTCTTCCTCTTGTTCCTCCCATGCTTTTGTTCTGGCCAGCTTGC
SRR448686.112672816.2-   AGGAATCAAGAGAATTGTCTGCCTCTTGTTCCTCCCATGCTTTTGTTCTGGCCAGCTTGC
SRR448686.116154652.1-   AGGAATCAAGAGAATGCTCTTCCTCTTGTTCCTCCCATGCTTTTGTTCTGGCCAGCTTGC
SRR448686.41318453.1-    AGGAATCAAGAGAATGCTCTTCCTCTTGTTCCTCCCATGCTTTTGTTCTGGCCAGCTTGC
SRR448686.51187136.1+    AGGAATCAAGAGAATGCTCTTCCTCTTGTTCCTCCCATGCTTTTGTTCTGGCCAGCTTGC
SRR448686.86332807.2-    AGGAATCAAGAGAATGCTCTTCCTCTTGTTCCTCCCATGCTTTTGTTCTGGCCAGCTTGC
SRR448687.16463223.1+    AGGAATCAAGAGAATGCTCTTCCTCTTGTTCCTCCCATGCTTTTGTTCTGGCCAGCTTGC
SRR448687.40599867.1-    AGGAATCAAGAGAATGCTCTTCCTCTTGTTCCTCCCATGCTTTTGTTCTGGCCAGCTTGC
SRR448687.49807054.1+    AGGAATCAAGAGAATGCTCTTCCTCTTGTTCCTCCCATGCTTTTGTTCTGGCCAGCTTGC
SRR448687.55441481.1+    AGGAATCAAGAGAATGCTCTTCCTCTTGTTCCTCCCATGCTTTTGTTCTGGCCAGCTTGC
SRR448687.68896953.1+    AGGAATCAAGAGAATGCTCTTCCTCTTGTTCCTCCCATGCTTTTGTTCTGGCCAGCTTGC
SRR448687.78234188.2+    AGGAATCAAGAGAATGCTCTTCCTCTTGTTCCTCCCATGCTTTTGTTCTGGCCAGCTTGC
SRR448687.85324970.2-    AGGAATCAAGAGAATGCTCTTCCTCTTGTTCCTCCCATGCTTTTGTTCTGGCCAGCTTGC
SRR448686.23979878.1+     GGAATCAAGAGAATGCTCTTCCTCTTGTTCCTCCCATGCTTTTGTTCTGGCCAGCTTGC
SRR448687.86864824.1-     GGAATCAAGAGAATGCTCTTCCTCTTGTTCCTCCCATGCTTTTGTTCTGGCCAGCTTGC
SRR448681.8573045.2-       GAATCAAGAGAATGCTCTTCCTCTTGTTCCTCCCATGCTTTTGTTCTGGCCAGCTTGC
SRR448687.3343710.2+       GAATCAAGAGAATGCTCTTCCTCTTGTTCCTCCCATGCTTTTGTTCTGGCCAGCTTGC
SRR448694.17743245.1-       AATCAAGAGAATGCTCTTCCTCTTGTTCCTCCCATGCTTTTGTTCTGGC
SRR448699.14233604.2+        ATCAAGAGAATGCTCTTCCTCTTGTTCCTCCCATGCTTTTGTTCTGGCC
SRR448681.7905433.1-         ATCAAGAGAATGCTCTTCCTCTTGTTCCTCCCATGCTTTTGTTCTGGCCAGCTTGC
SRR448683.7171667.2+         ATCAAGAGAATGCTCTTCCTCTTGTTCCTCCCATGCTTTTGTTCTGGCCAGCTTGC
SRR448694.92432333.2-         TCAAGAGAATGCTCTTCCTCTTGTTCCTCCCATGCTTTTGTTCTGGCCA
SRR448683.54936564.2-         TCAAGAGAATGCTCTTCCTCTTGTTCCTCCCATGCTTTTGTTCTGGCCAGCTTGC
SRR448675.89618126.1-          CAAGAGAATGCTCTTCCTCTTGTTCCTCCCATGCTTTTGTTCTGGCCAGCTTGC
SRR448686.45764778.1+          CAAGAGAATGCTCTTCCTCTTGTTCCTCCCATGCTTTTGTTCTGGCCAGCTTGC
SRR448686.62615618.2+          CAAGAGAATGCTCTTCCTCTTGTTCCTCCCATGCTTTTGTTCTGGCCAGCTTGC
SRR448687.74845974.2+          CAAGAGAATGCTCTTCCTCTTGTTCCTCCCATGCTTTTGTTCTGGCCAGCTTGC
SRR448675.41712885.1+           AAGAGAATGCTCTTCCTCTTGTTCCTCCCATGCTTTTGTTCTGGCCAGCTTGC
SRR448686.78672064.1+            AGAGAATGCTCTTCCTCTTGTTCCTCCCATGCTTTTGTTCTGGCCAGCTTGC
SRR448687.56549641.1+            AGAGAATGCTCTTCCTCTTGTTCTTCCCACGCTTTTGTTCTGGCCAGCTTGC
SRR448690.61093805.1+              AGAATGCTCTTCCTCTTGTTCCTCCCATGCTTTTGTTCTGGCCAGCTTG
SRR448690.80600913.1+              AGAATGCTCTTCCTCTTGTTCCTCCCATGCTTTTGTTCTGGCCAGCTTG
SRR448681.80676895.2-               GAATGCTCTTCCTCTTGTTCCTCCCATGCTTTTGTTCTGGCCAGCTTGC
SRR448681.94680159.1-                AATGCTCTTCCTCTTGTTCCTCCCATGCTTTTGTTCTGGCCAGCTTGC
SRR448687.11814241.1-                AATGCTCTTCCTCTTGTTCCTCCCATGCTTTTGTTCTGGCCAGCTTGC
SRR448683.7145084.1+                  ATGCTCTTCCTCTTGTTCCTCCCATGCTTTTGTTCTGGCCAGCTTGC
SRR448683.8612468.1+                  ATGCTCTTCCTCTTGTTCCTCCCATGCTTTTGTTCTGGCCAGCTTGC
SRR448683.28756459.1+                  TGCTCTTCCTCTTGTTCCTCCCATGCTTTTGTTCTGGCCAGCTTGC
SRR448681.58636992.1+                   GCTCTTCCTCTTGTTCCTCCCATGCTTTTGTTCTGGCCAGCTTGC
SRR448687.46257036.1+                   GCTCTTCCTCTTGTTCCTCCCATGCTTTTGTTCTGGCCAGCTTGC
SRR448681.69796216.1+                    CTCTTCCTCTTGTTCCTCCCATGCTTTTGTTCTGGCCAGCTTGC
SRR448686.109816047.1+                    TCTTCCTCTTGTTCCTCCCATGCTTTTGTTCTGGCCAGCTTGC
```

SRR448699.27636387.2-	TCTTCCTCTTGTTCCTCCCATGCTTTTGTTCTGGCCAGCTTGC
SRR448702.18743593.2+	TCTTCCTCTTGTTCCTCCCATGCTTTTGTTCTGGCCAGCTTGC
SRR448702.36153067.2+	TCTTCCTCTTGTTCCTCCCATGCTTTTGTTCTGGCCAGCTTGC
SRR448686.67616577.2+	CTTCCTCTTGTTCCTCCCATGCTTTTGTTCTGGCCAGCTTGC
SRR448681.18143961.1+	TTCCTCTTGTTCCTCCCATGCTTTTGTTCTGGCCAGCTTGC
SRR448686.43492870.2+	TTCCTCTTGTTCCTCCCATGCTTTTGTTCTGGCCAGCTTGC
SRR448687.46659143.2-	TTCCTCTTGTTCCTCCCATGCTTTTGTTCTGGCCAGCTTGC
SRR448699.23567757.2+	TTCCTCTTGTTCCTCCCATGCTTTTGTTCTGGCCAGCTTGC
SRR448699.61488415.2+	TTCCTCTTGTTCCTCCCATGCTTTTGTTCTGGCCAGCTTGC
SRR448686.81987744.1+	TCCTCTTGTTCCTCCCATGCTTTTGTTCTGGCCAGCTTGC
SRR448690.29380968.2-	TCCTCTTGTTCCTCCCATGCTTTTGTTCTGGCCAGCTTGC
SRR448681.47301511.1+	CTCTTGTTCCTCCCATGCTTTTGTTCTGGCCAGCTTGC
SRR448686.114576736.2+	CTCTTGTTCCTCCCATGCTTTTGTTCTGGCCAGCTTGC
SRR448702.53790654.2+	CTCTTGTTCCTCCCATGCTTTTGTTCTGGCCAGCTTGC
SRR448702.79376348.2+	CTCTTGTTCCTCCCATGCTTTTGTTCTGGCCAGCTTGC
SRR448702.80270161.2+	CTCTTGTTCCTCCCATGCTTTTGTTCTGGCCAGCTTGC
SRR448702.88283448.2+	CTCTTGTTCCTCCCATGCTTTTGTTCTGGCCAGCTTGC
SRR448686.4636378.1+	TCTTGTTCCTCCCATGCTTTTGTTCTGGCCAGCTTGC
SRR448690.25858161.1+	TCTTGTTCCTCCCATGCTTTTGTTCTGGCCAGCTTGC
SRR448690.36379102.1+	TCTTGTTCCTCCCATGCTTTTGTTCTGGCCAGCTTGC
SRR448690.62562223.1+	TCTTGTTCCTCCCATGCTTTTGTTCTGGCCAGCTTGC
SRR448690.78220875.1+	TCTTGTTCCTCCCATGCTTTTGTTCTGGCCAGCTTGC
SRR448681.86267206.2+	CTTGTTCCTCCCATGCTTTTGTTCTGGCCAGCTTGC
SRR448690.78945613.2-	TTGTTCCTCCCATGCTTTTGTTCTGGCCAGCTTGC
SRR448675.24488131.2-	GTTCCTCCCATGCTTTTGTTCTGGCCAGCTTGC
SRR448687.32853383.2+	GTTCCTCCCATGCTTTTGTTCTGGCCAGCTTGC
SRR448675.26299051.1-	TTCCTCCCATGCTTTTGTTCTGGCCAGCTTGC
SRR448687.52280250.1+	TCCTCCCATGCTTTTGTTCTGGCCAGCTTGC
SRR448690.11864772.2+	TCCTCCCATGCTTTTGTTCTGGCCAGCTTGC
SRR448690.331272.2+	TCCTCCCATGCTTTTGTTCTGGCCAGCTTGC
SRR448690.36200876.2+	TCCTCCCATGCTTTTGTTCTGGCCAGCTTGC
SRR448690.80958684.2+	TCCTCCCATGCTTTTGTTCTGGCCAGCTTGC
SRR448690.81660724.2+	TCCTCCCATGCTTTTGTTCTGGCCAGCTTGC
SRR448675.24700540.1+	CCTCCCATGCTTTTGTTCTGGCCAGCTTGC
SRR448681.12918655.2-	CCTCCCATGCTTTTGTTCTGGCCAGCTTGC
SRR448681.22542882.1-	CCTCCCATGCTTTTGTTCTGGCCAGCTTGC
SRR448686.77809999.2-	CCTCCCATGCTTTTGTTCTGGCCAGCTTGC
SRR448694.75898948.2+	CCTCCCATGCTTTTGTTCTGGCCAGCTTGC
SRR448675.76497237.2+	CTCCCATGCTTTTGTTCTGGCCAGCTTGC
SRR448690.6903614.1-	CTCCCATGCTTTTGTTCTGGCCAGCTTGC
SRR448675.3711092.1+	TCCCATGCTTTTGTTCTGGCCAGCTTGC
SRR448686.16841256.2+	TCCCATGCTTTTGTTCTGGCCAGCTTGC
SRR448683.16399903.2-	CCATGCTTTTGTTCTGGCCAGCTTGC
SRR448686.24823209.1+	CCATGCTTTTGTTCTGGCCAGCTTGC
SRR448675.54641135.1-	CATGCTTTTGTTCTGGCCAGCTTGC
SRR448687.737468.2+	ATGCTTTTGTTCTGGCCAGCTTGC
SRR448675.34371245.1+	TGCTTTTGTTCTGGCCAGCTTGC
SRR448690.32482994.2-	GCTTTTGTTCTGGCCAGCTTGC
SRR448690.34924895.1-	GCTTTTGTTCTGGCCAGCTTGC
SRR448690.35709700.1-	GCTTTTGTTCTGGCCAGCTTGC
SRR448690.68631968.2-	GCTTTTGTTCTGGCCAGCTTGC
SRR448690.88441719.1-	GCTTTTGTTCTGGCCAGCTTGC
SRR448675.26176825.1+	TTTTGTTCTGGCCAGCTTGC
SRR448690.33783403.2+	TTTTGTTCTGGCCAGCTTGC
SRR448690.39466210.2+	TTTTGTTCTGGCCAGCTTGC
SRR448690.4401988.2+	TTTTGTTCTGGCCAGCTTGC
SRR448690.60907104.2+	TTTTGTTCTGGCCAGCTTGC
SRR448699.87323970.1-	TTTTGTTCTGGCCAGCTTGC
SRR448699.104976401.2+	TTGTTCTGGCCAGCTTGC
SRR448675.37623604.1-	TTCTGGCCAGCTTGC
SRR448681.9386500.1-	TTCTGGCCAGCTTGC
SRR448687.96116175.2+	TCTGGCCAGCTTGC
SRR448687.40049376.1-	CTGGCCAGCTTGC
SRR448694.94535128.2-	CTGGCCAGCTTGC
SRR448686.90650419.2+	TGGCCAGCTTGC
SRR448686.111978264.2-	GGCCAGCTTGC
SRR448687.4804306.2+	GGCCAGCTTGC
SRR448694.98441280.2-	GGCCAGCTTGC
SRR448683.58306359.1+	CAGCTTGC
SRR448687.57896852.1+	CAGCTTGC
SRR448687.58131717.1-	CAGCTTGC
SRR448688.28024589.1-	CAGCTTGC
SRR448688.9125292.1-	CAGCTTGC
SRR448694.6034041.2-	CAGCTTGC
SRR448694.98363864.2+	AGCTTGC
SRR448675.24551560.2-	CTTGC
SRR448675.82235779.2-	CTTGC
SRR448675.59564553.1+	TTGC
SRR448675.81560484.2+	TTGC
SRR448694.29420155.1+	TTGC
SRR448683.13133273.1+	TGC
SRR448686.35001025.1+	TGC
SRR448686.78011325.2-	GC
SRR448688.44162718.2+	GC

```
SRR448688.47771318.2+                                                                      GC
SRR448688.51806068.2+                                                                      GC
SRR448688.71711909.1+                                                                      GC
SRR448688.73119745.1+                                                                      GC
SRR448688.78619446.2+                                                                      GC
SRR448688.89980412.1+                                                                      GC

consensus                AGGAATCAAGAGAATGCTCTTCCTCTTGTTCCTCCCATGCTTTTGTTCTGGCCAGCTTGC

                             .    :    .    :    .    :    .    :    .    :    .    :
SRR448687.16463223.1+    A
SRR448686.86332807.2-    ATCTC
SRR448686.116154652.1-   ATCTCC
SRR448687.85324970.2-    ATCTCC
SRR448699.27636387.2-    ATCTCC
SRR448702.18743593.2+    ATCTCC
SRR448702.36153067.2+    ATCTCC
SRR448681.13943690.1-    ATCTCCTC
SRR448699.23567757.2+    ATCTCCTC
SRR448699.61488415.2+    ATCTCCTC
SRR448687.40599867.1-    ATCTCCTCT
SRR448690.29380968.2-    ATCTCCTCT
SRR448675.54641135.2+    ATCTCCTCTG
SRR448702.53790654.2+    ATCTCCTCTGA
SRR448702.79376348.2+    ATCTCCTCTGA
SRR448702.80270161.2+    ATCTCCTCTGA
SRR448702.88283448.2+    ATCTCCTCTGA
SRR448683.77711223.2+    ATCTCCTATGAT
SRR448690.25858161.1+    ATCTCCTCTGAT
SRR448690.36379102.1+    ATCTCCTCTGAT
SRR448690.62562223.1+    ATCTCCTCTGAT
SRR448690.78220875.1+    ATCTCCTCTGAT
SRR448681.12918655.1+    ATCTCCTCTGATGC
SRR448683.63839307.1+    CTCTCCTCTGATGC
SRR448690.78945613.2-    ATCTCCTCTGATGC
SRR448683.82663094.2-    ATCTCCTCTGATGCT
SRR448681.103745259.2-   ATCTCCTCTGATGCTT
SRR448681.66970561.1-    ATCTCCTCTGATGCTT
SRR448686.112672816.2-   ATCTCCTCTGATGCTT
SRR448675.61454794.2-    ATCTCCTCTGATGCTTT
SRR448681.9386500.2+     ATCTCCTCTGATGCTTT
SRR448690.11864772.2+    ATCTCCTCTGATGCTTTT
SRR448690.331272.2+      ATCTCCTCTGATGCTTTT
SRR448690.36200876.2+    ATCTCCTCTGATGCTTTT
SRR448690.80958684.2+    ATCTCCTCTGATGCTTTT
SRR448690.81660724.2+    ATCTCCTCTGATGCTTTT
SRR448694.75898948.2+    ATCTCCTCTGATGCTTTTC
SRR448675.82235779.1+    ATCTCCTCTGATGCTTTTCC
SRR448690.6903614.1-     ATCTCCTCTGATGCTTTTCC
SRR448675.37623604.2+    ATCTCCTCTGATGCTTTTCCCC
SRR448681.70988830.2-    ATCTCCTCTGATGCTTTTCCCC
SRR448675.105142019.1-   ATCTCCTCTGATGCTTTTCCCCAC
SRR448681.80008393.1-    ATCTCCTCTGATGCTTTTCCCCAC
SRR448686.109419161.1-   ATCTCCTCTGATGCTTTTCCCCAC
SRR448675.73902527.1-    ATCTCCTCTGATGCTTTTCCCCACC
SRR448683.11200816.2-    ATCTCCTCTGATGCTTTTCCCCACC
SRR448681.59142577.2-    ATCTCCTCTGATGCTTTTCCCCACCT
SRR448690.32482994.2-    ATCTCCTCTGATGCTTTTCCCCACCTT
SRR448690.34924895.1-    ATCTCCTCTGATGCTTTTCCCCACCTT
SRR448690.35709700.1-    ATCTCCTCTGATGCTTTTCCCCACCTT
SRR448690.68631968.2-    ATCTCCTCTGATGCTTTTCCCCACCTT
SRR448690.88441719.1-    ATCTCCTCTGATGCTTTTCCCCACCTT
SRR448690.33783403.2+    ATCTCCTCTGATGCTTTTCCCCACCTTCC
SRR448690.39466210.2+    ATCTCCTCTGATGCTTTTCCCCACCTTCC
SRR448690.4401988.2+     ATCTCCTCTGATGCTTTTCCCCACCTTCC
SRR448690.60907104.2+    ATCTCCTCTGATGCTTTTCCCCACCTTCC
SRR448699.87323970.1-    ATCTCCTCTGATGCTTTTCCCCACCTTCC
SRR448675.24551560.1+    ATCTCCTCTGATGCTTTTCCCCACCTTCCT
SRR448675.34632732.1-    ATCTCCTCTGATGCTTTTCCCCACCTTCCTG
SRR448687.49807054.1+    ATCTCCTCTGATGCTTTTCCCCACCTTCCTG
SRR448699.104976401.2+   ATCTCCTCTGATGCTTTTCCCCACCTTCCTG
SRR448687.78234188.2+    ATCTCCTCTGATGCTTTTCCCCACCTTCCTGG
SRR448681.4029888.2-     ATCTCCTCTGATGCTTTTCCCCACCTTCCTGGA
SRR448675.91257091.2-    ATCTCCTCTGATGCTTTTCCCCACCTTCCTGGATC
SRR448686.105571365.2+   ATCTCCTCTGATGCTTTTCCCCACCTTCCTGGATC
SRR448686.102868232.2-   ATCTCCTCTGATGCTTTTCCCCACCTTCCTGGATCC
SRR448694.94535128.2-    ATCTCCTCTGATGCTTTTCCCCACCTTCCTGGATCC
SRR448675.61033335.2-    ATCTCCTCTGATGCTTTTCCCCACCTTCCTGGATCCT
SRR448686.41318453.1-    ATCTCCTCTGATGCTTTTCCCCACCTTCCTGGATCCT
SRR448687.55441481.1+    ATCTCCTCTGATGCTTTTCCCCACCTTCCTGGATCCT
SRR448687.56549641.1+    ATCTCCTCTGATGCTTTTCCCCACTTTCCTTGATCCTT
SRR448687.68896953.1+    ATCTCCTCTGATGCTTTTCCCCACCTTCCTGGATCCTT
SRR448694.98441280.2-    ATCTCCTCTGATGCTTTTCCCCACCTTCCTGGATCCTT
SRR448681.88931306.1+    ATCTCCTCTGATGCTTTTCCCCACCTTCCTGGATCCTTC
```

SRR448683.69969075.2+   ATCTCCTCTGATGCTTTTCCCCACCTTCCTGGATCCTTC
SRR448686.106701110.2+   ATCTCCTCTGAGGCTTTTCACCACCTTCCTGGACCCTTCC
SRR448686.51187136.1+   ATCTCCTCTGATGCTTTTCCCCACCTTCCTGGATCCTTCC
SRR448686.23979878.1+   ATCTCCTCTGATGCTTTTCCCCACCTTCCTGGATCCTTCCA
SRR448687.86864824.1-   ATCTCCTCTGATGCTTTTCCCCACCTTCCTGGATCCTTCCA
SRR448688.28024589.1-   ATCTCCTCTGATGCTTTTCCCCACCTTCCTGGATCCTTCCA
SRR448688.9125292.1-   ATCTCCTCTGATGCTTTTCCCCACCTTCCTGGATCCTTCCA
SRR448694.6034041.2-   ATCTCCTCTGATGCTTTTCCCCACCTTCCTGGATCCTTCCA
SRR448681.8573045.2-   ATCTCCTCTGATGCTTTTCCCCACCTTCCTGGATCCTTCCAG
SRR448687.3343710.2+   ATCTCCTCTGATGCTTTTCCCCACCTTCCTGGATCCTTCCAG
SRR448694.98363864.2+   ATCTCCTCTGATGCTTTTCCCCACCTTCCTGGATCCTTCCAG
SRR448681.7905433.1-   ATCTCCTCTGATGCTTTTCCCCACCTTCCTGGATCCTTCCAGCA
SRR448683.7171667.2+   ATCTCCTCTGATGCTTTTCCCCACCTTCCTGGATCCTTCCAGCA
SRR448687.74845974.2+   ATCTCCTCTGATGCTTTTCCCCACCTTCCTGGATCCTTCCAGCA
SRR448683.54936564.2-   ATCTCCTCTGATGCTTTTCCCCACCTTCCTGGATCCTTCCAGCAT
SRR448694.29420155.1+   ATCTCCTCTGATGCTTTTCCCCACCTTCCTGGATCCTTCCAGCAT
SRR448675.89618126.1-   ATCTCCTCTGATGCTTTTCCCCACCTTCCTGGATCCTTCCAGCATG
SRR448686.45764778.1+   ATCTCCTCTGATGCTTTTCCCCACCTTCCTGGATCCTTCCAGCATG
SRR448686.62615618.2+   ATCTCCTCTGATGCTTTTCCCCACCTTCCTGGATCCTTCCAGCATG
SRR448675.41712885.1+   ATCTCCTCTGATGCTTTTCCCCACCTTCCTGGATCCTTCCAGCATGT
SRR448688.44162718.2+   ATCTCCTCTGATGCTTTTCCCCACCTTCCTGGATCCTTCCAGCATGT
SRR448688.47771318.2+   ATCTCCTCTGATGCTTTTCCCCACCTTCCTGGATCCTTCCAGCATGT
SRR448688.51806068.2+   ATCTCCTCTGATGCTTTTCCCCACCTTCCTGGATCCTTCCAGCATGT
SRR448688.71711909.1+   ATCTCCTCTGATGCTTTTCCCCACCTTCCTGGATCCTTCCAGCATGT
SRR448688.73119745.1+   ATCTCCTCTGATGCTTTTCCCCACCTTCCTGGATCCTTCCAGCATGT
SRR448688.78619446.2+   ATCTCCTCTGATGCTTTTCCCCACCTTCCTGGATCCTTCCAGCATGT
SRR448688.89980412.1+   ATCTCCTCTGATGCTTTTCCCCACCTTCCTGGATCCTTCCAGCATGT
SRR448686.78672064.1+   ATCTCCTCTGATGCTTTTCCCCACCTTCCTGGATCCTTCCAGCATGTT
SRR448681.80676895.2-   ATCTCCTCTGATGCTTTTCCCCACCTTCCTGGATCCTTCCAGCATGTTCT
SRR448681.94680159.1-   ATCTCCTCTGATGCTTTTCCCCACCTTCCTGGATCCTTCCAGCATGTTCTAC
SRR448687.11814241.1-   ATCTCCTCTGATGCTTTTCCCCACCTTCCTGGATCCTTCCAGCATGTTCTAC
SRR448683.7145084.1+   ATCTCCTCTGATGCTTTTCCCCACCTTCCTGGATCCTTCCAGCATGTTCTACC
SRR448683.8612468.1+   ATCTCCTCTGATGCTTTTCCCCACCTTCCTGGATCCTTCCAGCATGTTCTACC
SRR448683.28756459.1+   ATCTCCTCTGATGCTTTTCCCCACCTTCCTGGATCCTTCCAGCATGTTCTACCT
SRR448681.58636992.1+   ATCTCCTCTGATGCTTTTCCCCACCTTCCTGGATCCTTCCAGCATGTTCTACCTC
SRR448687.46257036.1+   ATCTCCTCTGATGCTTTTCCCCACCTTCCTGGATCCTTCCAGCATGTTCTACCTC
SRR448681.69796216.1+   ATCTCCTCTGATGCTTTTCCCCACCTTCCTGGATCCTTCCAGCATGTTCTACCTCT
SRR448686.109816047.1+   ATCTCCTCTGATGCTTTTCCCCACCTTCCTGGATCCTTCCAGTATGTTCTACCTCTG
SRR448686.67616577.2+   ATCTCCTCTGATGCTTTTCCCCACCTTCCTGGATCCTTCCAGCATGTTCTACCTCTGC
SRR448681.18143961.1+   ATCTCCTCTGATGCTTTTCCCCACCTTCCTGGATCCTTCCAGCATGTTCTACCTCTGCT
SRR448686.43492870.2+   ATCTCCTCTGATGCTTTTCCCCAACTTCCTGGATCCTTCCAGCATGTTCTACCTCTGCT
SRR448687.46659143.2-   ATCTCCTCTGATGCTTTTCCCCACCTTCCTGGATCCTTCCAGCATGTTCTACCTCTGCT
SRR448675.104363339.1+   ATCTCCTCTGATGCTTTTCCCCACCTTCCTGGATCCTTCCAGCATGTTCTACCTCTGCTG
SRR448675.24488131.2-   ATCTCCTCTGATGCTTTTCCCCACCTTCCTGGATCCTTCCAGCATGTTCTACCTCTGCTG
SRR448675.24551560.2-   ATCTCCTCTGATGCTTTTCCCCACCTTCCTGGATCCTTCCAGCATGTTCTACCTCTGCTG
SRR448675.24700540.1+   ATCTCCTCTGATGCTTTTCCCCACCTTCCTGGATCCTTCCAGCATGTTCTACCTCTGCTG
SRR448675.24783207.2+   ATCTCCTCTGATGCTTTTCCCCACCTTCCTGGATCCTTCCAGCATGTTCTACCTCTGCTG
SRR448675.26176825.1+   ATCTCCTCTGATGCTTTTCCCCACCTTCCTGGATCCTTCCAGCATGTTCTACCTCTGCTG
SRR448675.26299051.1-   ATCTCCTCTGATGCTTTTCCCCACCTTCCTGGATCCTTCCAGCATGTTCTACCTCTGCTG
SRR448675.34371245.1+   ATCTCCTCTGATGCTTTTCCCCACCTTCCTGGATCCTTCCAGCATGTTCTACCTCTGCTG
SRR448675.3711092.1+   ATCTCCTCTGATGCTTTTCCCCACCTTCCTGGATCCTTCCAGCATGTTCTACCTCTGCTG
SRR448675.37623604.1-   ATCTCCTCTGATGCTTTTCCCCACCTTCCTGGATCCTTCCAGCATGTTCTACCTCTGCTG
SRR448675.54641135.1-   ATCTCCTCTGATGCTTTTCCCCACCTTCCTGGATCCTTCCAGCATGTTCTACCTCTGCTG
SRR448675.59564553.1+   ATCTCCTCTGATGCTTTTCCCCACCTTCCTGGATCCTTCCAGCATGTTCTACCTCTGCTG
SRR448675.76497237.2+   ATCTCCTCTGATGCTTTTCCCCACCTTCCTGGATCCTTCCAGCATGTTCTACCTCTGCTG
SRR448675.81560484.2+   ATCTCCTCTGATGCTTTTCCCCACCTTCCTGGATCCTTCCAGCATGTTCTACCTCTGCTG
SRR448675.82235779.2-   ATCTCCTCTGATGCTTTTCCCCACCTTCCTGGATCCTTCCAGCATGTTCTACCTCTGCTG
SRR448681.12918655.2-   ATCTCCTCTGATGCTTTTCCCCACCTTCCTGGATCCTTCCAGCATGTTCTACCTCTGCTG
SRR448681.22542882.1-   ATCTCCTCTGATGCTTTTCCCCACCTTCCTGGATCCTTCCAGCATGTTCTACCTCTGCTG
SRR448681.47301511.1+   ATCTCCTCTGATGCTTTTCCCCACCTTCCTGGATCCTTCCAGCATGTTCTACCTCTGCTG
SRR448681.86267206.2+   ATCTCCTCTGATGCTTTTCCCCACCTTCCTGGATCCTTCCAGCATGTTCTACCTCTGCTG
SRR448681.9386500.1-   ATCTCCTCTGATGCTTTTCCCCACCTTCCTGGATCCTTCCAGCATGTTCTACCTCTGCTG
SRR448683.13133273.1+   ATCTCCTCTGATGCTTTTCCCCACCTTCCTGGATCCTTCCAGCATGTTCTACCTCTGCTG
SRR448683.16399903.2-   ATCTCCTCTGATGCTTTTCCCCACCTTCCTGGATCCTTCCAGCATGTTCTACCTCTGCTG
SRR448683.58306359.1+   ATCTCCTCTGATGCTTTTCCCCACCTTCCTGGATCCTTCCAGCATGTTCTACCTCTGCTG
SRR448686.1056904.2+   ATCTCCTCTGATGCTTTTCCCCACCTTCCTGGATCCTTCCAGCATGTTCTACCTCTGCTG
SRR448686.111978264.2-   ATCTCCTCTGATGCTTTTCCCCACCTTCCTGGATCCTTCCAGCATGTTCTACCTCTGCTG
SRR448686.114576736.2+   ATCTCCTCTGATGCTTTTCCCCACCTTCCTGGATCCTTCCAGCATGTTCTACCTCTGCTG
SRR448686.16841256.2+   ATCTCCTCTGATGCTTTTCCCCACCTTCCTGGATCCTTCCAGCATGTTCTACCTCTGCTG
SRR448686.24823209.1+   ATCTCCTCTGATGCTTTTCCCCACCTTCCTGGATCCTTCCAGCATGTTCTACCTCTGCTG
SRR448686.35001025.1+   ATCTCCTCTGATGCTTTTCCCCACCTTCCTGGATCCTTCCAGCATGTTCTACCTCTGCTG
SRR448686.4636378.1+   ATCTCCTCTGATGCTTTTCCCCACCTTCCTGGATCCTTCCAGCATGTTCTACCTCTGCTG
SRR448686.77809999.2-   ATCTCCTCTGATGCTTTTCCCCACCTTCCTGGATCCTTCCAGCATGTTCTACCTCTGCTG
SRR448686.78011325.2-   ATCTCCTCTGATGCTTTTCCCCACCTTCCTGGATCCTTCCAGCATGTTCTACCTCTGCTG
SRR448686.81987744.1+   ATCTCCTCTGATGCTTTTCCCCACCTTCCTGGATCCTTCCAGCATGTTCTACATCTGCTG
SRR448686.90650419.2+   ATCTCCTCTGATGCTTTTCCCCACCTTCCTGGATCCTTCCAGCATGTTCTACCTCTGCTG
SRR448687.32853383.2+   ATCTCCTCTGATGCTTTTCCCCACCTTCCTGGATCCTTCCAGCATGTTCTACCTCTGCTG
SRR448687.40049376.1-   ATCTCCTCTGATGCTTTTCCCCTCCTTCCTGGATCCTTCCAGCATGTTCTACCTCTGCTG
SRR448687.4804306.2+   ATCTCCTCTGATGCTTTTCCCCACCTTCCTGGATCCTTCCAGCATGTTCTACCTCTGCTG
SRR448687.52280250.1+   ATCTCCTCTGATGCTTTTCCCCACCTTCCTGGATCCTTCCAGCATGTTCTACCTCTGCTG
SRR448687.57896852.1+   ATCTCCTCTGATGCTTTTCCCCACCTTCCTGGATCCTTCCAGCATGTTCTACCTCTGCTG
SRR448687.58131717.1-   ATCTCCTCTGATGCTTTTCCCCACCTTCCTGGATCCTTCCAGCATGTTCTACCTCTGCTG
SRR448687.737468.2+   ATCTCCTCTGATGCTTTTCCCCACCTTCCTGGATCCTTCCAGCATGTTCTACCTCTGCTG
SRR448687.96116175.2+   ATCTCCTCTGATGCTTTTCCCCACCTTCCTGGATCCTTCCAGCATGTTCTACCTCTGTTG

```
SRR448687.88915780.1+     TCTCCTCTGATGCTTTTCCCCACCTTCCTGGATCCTTCCAGCATGTTCTACCTCTGCTG
SRR448690.33179760.1+      CTCCTCTGATGCTTTTCCCCACCTTCCTGGATCCTTCCAGCATGTTCTA
SRR448690.4517109.1+       CTCCTCTGATGCTTTTCCCCACCTTCCTGGATCCTTCCAGCATGTTCTA
SRR448690.55728553.1+      CTCCTCTGATGCTTTTCCCCACCTTCCTGGATCCTTCCAGCATGTTCTA
SRR448690.82753351.1+      CTCCTCTGATGCTTTTCCCCACCTTCCTGGATCCTTCCAGCATGTTCTA
SRR448699.60328335.2-      CTCCTCTGATGCTTTTCCCCACCTTCCTGGATCCTTCCAGCATGTTCTA
SRR448699.60338778.2+      CTCCTCTGATGCTTTTCCCCACCTTCCTGGATCCTTCCAGCATGTTCTA
SRR448699.79010841.2+      CTCCTCTGATGCTTTTCCCCACCTTCCTGGATCCTTCCAGCATGTTCTA
SRR448683.4919531.2+       CTCCTCTGATGCTTTTCCCCACCTTCCTGGATCCTTCCAGCATGTTCTACCTCTGCTG
SRR448683.90888613.2+      CTCCTCTGATGCTTTTCCCCTCCTTCCTTGTTCCTTCCAGCATGTTCTACCTCTGCTG
SRR448687.55340056.2-       TCCTCTGATGCTTTTCCCCACCTTCCTGGATCCTTCCAGCATGTTCTACCTCTGCTG
SRR448694.35914305.1-           CTGATGCTTTTCCCCACCTTCCTGGATCCTTCCAGCATGTTCTACCTCT
SRR448694.5122634.1-            CTGATGCTTTTCCCCACCTTCCTGGATCCTTCCAGCATGTTCTACCTCT
SRR448699.50118699.1-           CTGATGCTTTTCCCCACCTTCCTGGATCCTTCCAGCATGTTCTACCTCT
SRR448699.60282870.1-           CTGATGCTTTTCCCCACCTTCCTGGATCCTTCCAGCATGTTCTACCTCT
SRR448675.92739025.2+           CTGATGCTTTTCCCCACCTTCCTGGATCCTTCCAGCATGTTCTACCTCTGCTG
SRR448683.53372918.2-           CTGATGCTTTTCCCGACCTTCCTGGATCCTTCCAGCATGTTCTACCTCTGCTG
SRR448686.20664457.2+           CTGATGCTTTTCCCCACCTTCCTGGATCCTTCCAGCATGTTCTACCTCTGCTG
SRR448681.7497961.1+             TGATGCTTTTCCCCACCTTCCTGGATCCTTCCAGCATGTTCTACCTCTGCTG
SRR448686.83118909.2+                 CTTTTCCCCACCTTCCTGGATCCTTCCAGCATGTTCTACCTCTGCTG
SRR448702.50912846.1+                 CTTTTCCCCACCTTCCTGGATCCTTCCAGCATGTTCTACCTCTGCTG
SRR448694.36209705.2+                  TTTTCCCCACCTTCCTGGATCCTTCCAGCATGTTCTACCTCTGCTG
SRR448688.60587352.2-                   TTTCCCCACCTTCCTGGATCCTTCCAGCATGTTCTACCTCTGCTG
SRR448688.83480597.2-                   TTTCCCCACCTTCCTGGATCCTTCCAGCATGTTCTACCTCTGCTG
SRR448694.89521764.1-                    TTCCCCACCTTCCTGGATCCTTCCAGCATGTTCTACCTCTGCTG
SRR448681.69796216.2-                     TCCCCACCTTCCTGGATCCTTCCAGCATGTTCTACCTCTGCTG
SRR448681.88931306.2-                     TCCCCCCCTTCCTGGATCCTTCCAGCATGTTCTACCTCTGCTG
SRR448686.86029142.1-                      CCCCACCTTCCTGGATCCTTCCAGCATGTTCTACCTCTGCTG
SRR448683.59297888.2+                       CCCACCTTCCTGGATCCTTCCAGCATGTTCTACCTCTGCTG
SRR448702.56722010.2+                        CCACCTTCCTGGATCCTTCCAGCATGTTCTACCTCTGCTG
SRR448699.57717956.1-                          ACCTTCCTGGATCCTTCCAGCATGTTCTACCTCTGCTG
SRR448699.97721361.1-                          ACCTTCCTGGATCCTTCCAGCATGTTCTACCTCTGCTG
SRR448690.1725560.1-                            CCTTCCTGGATCCTTCCAGCATGTTCTACCTCTGCTG
SRR448690.68594978.1-                           CCTTCCTGGATCCTTCCAGCATGTTCTACCTCTGCTG
SRR448690.69634400.1-                           CCTTCCTGGATCCTTCCAGCATGTTCTACCTCTGCTG
SRR448687.994456.2+                              CTTCCTGGATCCTTCCAGCATGTTCTACCTCTGCTG
SRR448675.41712885.2-                             TTCCTGGATCCTTCCAGCATGTTCTACCTCTGCTG
SRR448683.45914950.1-                             TTCCTGGATCCTTCCAGCATGTTCTACCTCTGCTG
SRR448694.92755853.2+                             TTCCTGGATCCTTCCAGCATGTTCTACCTCTGCTG
SRR448699.41278410.2-                             TTCCTGGATCCTTCCAGCATGTTCTACCTCTGCTG
SRR448699.4367997.1-                              TTCCTGGATCCTTCCAGCATGTTCTACCTCTGCTG
SRR448688.75840989.2+                              TCCTGGATCCTTCCAGCATGTTCTACCTCTGCTG
SRR448699.101976960.2+                             TCCTGGATCCTTCCAGCATGTTCTACCTCTGCTG
SRR448681.58636992.2-                               CCTGGATCCTTCCAGCATGTTCTACCTCTGCTG
SRR448686.34129339.2+                               CCTGGATCCTTCCAGCATGTTCTACCTCTGCTG
SRR448681.34420081.1+                                 TGGATCCTTCCAGCATGTTCTACCTCTGCTG
SRR448699.102129511.2-                                TGGATCCTTCCAGCATGTTCTACCTCTGCTG
SRR448699.26262351.2-                                 TGGATCCTTCCAGCATGTTCTACCTCTGCTG
SRR448686.99783446.1-                                  GGATCCTTCCAGCATGTTCTACCTCTGCTG
SRR448705.2172827.1-                                   GGATCCTTCCAGCATGTTCTACCTCTGCTG
SRR448705.22697971.1-                                  GGATCCTTCCAGCATGTTCTACCTCTGCTG
SRR448705.24783751.1-                                  GGATCCTTCCAGCATGTTCTACCTCTGCTG
SRR448705.35110401.1-                                  GGATCCTTCCAGCATGTTCTACCTCTGCTG
SRR448705.41751292.1-                                  GGATCCTTCCAGCATGTTCTACCTCTGCTG
SRR448705.43820714.1-                                  GGATCCTTCCAGCATGTTCTACCTCTGCTG
SRR448705.62173839.1-                                  GGATCCTTCCAGCATGTTCTACCTCTGCTG
SRR448705.73492024.1-                                  GGATCCTTCCAGCATGTTCTACCTCTGCTG
SRR448705.76600513.1-                                  GGATCCTTCCAGCATGTTCTACCTCTGCTG
SRR448705.78624254.1-                                  GGATCCTTCCAGCATGTTCTACCTCTGCTG
SRR448705.88689082.1-                                  GGATCCTTCCAGCATGTTCTACCTCTGCTG
SRR448690.19251526.1-                                    ATCCTTCCAGCATGTTCTACCTCTGCTG
SRR448690.35177987.1-                                    ATCCTTCCAGCATGTTCTACCTCTGCTG
SRR448690.8793826.1-                                     ATCCTTCCAGCATGTTCTACCTCTGCTG
SRR448694.88915619.1+                                    ATCCTTCCAGCATGTTCTACCTCTGCTG
SRR448681.86267206.1-                                      CCTTCCAGCATGTTCTACCTCTGCTG
SRR448687.65214307.1+                                      CCTTCCAGCATGTTCTACCTCTGCTG
SRR448683.14290636.1-                                       CTTCCAGCATGTTCTACCTCTGCTG
SRR448686.46585949.2-                                       CTTCGAGCATGTTCTACCTCTGCTG
SRR448687.62503016.1+                                       CTTCCAGCATGTTCTACCTCTGCTG
SRR448705.94463881.2+                                       CTTCCAGCATGTTCTACCTCTGCTG
SRR448681.47301511.2-                                        TTCCAGCATGTTCTACCTCTGCTG
SRR448690.100388792.1+                                       TTCCAGCATGTTCTACCTCTGCTG
SRR448690.91289910.1+                                        TTCCAGCATGTTCTACCTCTGCTG
SRR448699.10605196.1+                                        TTCCAGCATGTTCTACCTCTGCTG
SRR448675.104128282.1+                                        TCCAGCATGTTCTACCTCTGCTG
SRR448675.24700540.2-                                         TCCAGCATGTTCTACCTCTGCTG
SRR448688.23026856.2+                                         TCCAGCATGTTCTACCTCTGCTG
SRR448688.26519151.1+                                         TCCAGCATGTTCTACCTCTGCTG
SRR448688.4725251.1+                                          TCCAGCATGTTCTACCTCTGCTG
SRR448688.75228225.1+                                         TCCAGCATGTTCTACCTCTGCTG
SRR448688.85519119.1+                                         TCCAGCATGTTCTACCTCTGCTG
SRR448688.9307577.1+                                          TCCAGCATGTTCTACCTCTGCTG
SRR448675.26176825.2-                                          CCAGCATGTTCTACCTCTGCTG
SRR448687.71782978.2+                                          CCAGCATGTTCTACCTCTGCTG
```

```
SRR448699.28603845.1+                                          CCAGCATGTTCTACCTCTGCTG
SRR448681.50099907.1+                                           CAGCATGTTCTACCTCTGCTG
SRR448687.89910530.2+                                           CAGCATGTTCTACCTCTGCTG
SRR448683.19193053.2-                                            AGCATGTTCTACCTCTGCTG
SRR448675.3711092.2-                                              GCATGTTCTACCTCTGCTG
SRR448681.18143961.2-                                             GCATGTTCTACCTCTGCTG
SRR448688.623745.1+                                               GCATGTTCTACCTCTGCTG
SRR448690.59500894.1-                                             GCATGTTCTACCTCTGCTG
SRR448690.82179856.1-                                             GCATGTTCTACCTCTGCTG
SRR448699.51426342.2-                                             GCATGTTCTACCTCTGCTG
SRR448687.59753368.2+                                              CATGTTCTACCTCTGCTG
SRR448683.91457999.2-                                               ATGTTCTACCTCTGCTG
SRR448699.74102933.2+                                                TGTTCTACCTCTGCTG
SRR448683.53184158.2-                                                 GTTCTACCTCTGCTG
SRR448694.13441487.2-                                                 GTTCTACCTCTGCTG
SRR448687.10881609.1+                                                  TTCTACCTCTGCTG
SRR448687.88021195.1+                                                  TTCTACCTCTGCTG
SRR448694.2375465.1-                                                   TTCTACCTCTGCTG
SRR448675.76497237.1-                                                     TACCTCTGCTG
SRR448681.68030745.1+                                                     TACCTCTGCTG
SRR448681.21545235.2+                                                      ACCTCTGCTG
SRR448690.52257033.2+                                                      ACCTCTGCTG
SRR448687.42732058.1-                                                       CCTCTGCTG
SRR448687.47390517.2+                                                       CCTCTGCTG
SRR448699.47956681.2+                                                       CCTCTGCTG
SRR448675.34371245.2-                                                        CTCTGCTG
SRR448681.32854092.1+                                                          CTGCTG
SRR448681.3793565.1+                                                            TGCTG
SRR448681.7337942.2+                                                            TGCTG
SRR448694.19498543.1-                                                            GCTG
SRR448687.6574725.1-                                                              CTG
SRR448694.5989384.1+                                                               TG
SRR448694.71735553.1+                                                              TG
SRR448694.92596162.1+                                                              TG
SRR448686.24956981.2-                                                               G
SRR448688.25191493.1-                                                               G
SRR448688.28589525.1-                                                               G
SRR448688.32670682.1-                                                               G
                         ------------------------------------------------------------
consensus                ATCTCCTCTGATGCTTTTCCCCACCTTCCTGGATCCTTCCAGCATGTTCTACCTCTGCTG

                             .    :    .    :    .    :    .    :    .    :    .    :
SRR448681.47301511.1+    GG
SRR448686.114576736.2+   GG
SRR448702.50912846.1+    GG
SRR448686.4636378.1+     GGA
SRR448694.36209705.2+    GGA
SRR448681.86267206.2+    GGAG
SRR448688.60587352.2-    GGAG
SRR448688.83480597.2-    GGAG
SRR448694.89521764.1-    GGAGC
SRR448675.24488131.2-    GGAGCAC
SRR448687.32853383.2+    GGAGCAC
SRR448675.26299051.1-    GGAGCACA
SRR448687.52280250.1+    GGAGCACAA
SRR448702.56722010.2+    GGAGCACAA
SRR448675.24700540.1+    GGAGCACAAG
SRR448681.12918655.2-    GGAGCACAAG
SRR448681.22542882.1-    GGAGCACAAG
SRR448686.77809999.2-    GGAGCACAAG
SRR448675.76497237.2+    GGAGCACAAGG
SRR448699.57717956.1-    GGAGCACAAGG
SRR448699.97721361.1-    GGAGCACAAGG
SRR448675.3711092.1+     GGAGCACAAGGA
SRR448686.16841256.2+    GGAGCACAAGGA
SRR448690.1725560.1-     GGAGCACAAGGA
SRR448690.68594978.1-    GGAGCACAAGGA
SRR448690.69634400.1-    GGAGCACAAGGA
SRR448683.16399903.2-    GGAGCACAAGGAAC
SRR448686.24823209.1+    GGAGCACAAGGAAC
SRR448694.92755853.2+    GGAGCACAAGGAAC
SRR448699.41278410.2-    GGAGCACAAGGAAC
SRR448699.4367997.1-     GGAGCACAAGGAAC
SRR448675.54641135.1-    GGAGCACAAGGAACA
SRR448688.75840989.2+    GGAGCACAAGGAACA
SRR448699.101976960.2+   GGAGCACAAGGAACA
SRR448687.737468.2+      GGAGCACAAGGAACAG
SRR448675.34371245.1+    GGAGCACAAGGAACAGG
SRR448699.102129511.2-   GGAGCACAAGGAACAGGA
SRR448699.26262351.2-    GGAGCACAAGGAACAGGA
SRR448705.2172827.1-     GGAGCACAAGGAACAGGAG
SRR448705.22697971.1-    GGAGCACAAGGAACAGGAG
SRR448705.24783751.1-    GGAGCACAAGGAACAGGAG
SRR448705.35110401.1-    GGAGCACAAGGAACAGGAG
```

SRR448705.41751292.1-   GGAGCACAAGGAACAGGAG
SRR448705.43820714.1-   GGAGCACAAGGAACAGGAG
SRR448705.62173839.1-   GGAGCACAAGGAACAGGAG
SRR448705.73492024.1-   GGAGCACAAGGAACAGGAG
SRR448705.76600513.1-   GGAGCACAAGGAACAGGAG
SRR448705.78624254.1-   GGAGCACAAGGAACAGGAG
SRR448705.88689082.1-   GGAGCACAAGGAACAGGAG
SRR448675.26176825.1+   GGAGCACAAGGAACAGGAGC
SRR448690.19251526.1-   GGAGCACAAGGAACAGGAGCT
SRR448690.35177987.1-   GGAGCACAAGGAACAGGAGCT
SRR448690.8793826.1-   GGAGCACAAGGAACAGGAGCT
SRR448694.88915619.1+   GGAGCACAAGGAACAGGAGCT
SRR448705.94463881.2+   GGAGCACAAGGAACAGGAGCTGAT
SRR448675.37623604.1-   GGAGCACAAGGAACAGGAGCTGATG
SRR448681.9386500.1-   GGAGCACAAGGAACAGGAGCTGATG
SRR448690.100388792.1+   GGAGCACAAGGAACAGGAGCTGATG
SRR448690.91289910.1+   GGAGCACAAGGAACAGGAGCTGATG
SRR448699.10605196.1+   GGAGCACAAGGAACAGGAGCTGATG
SRR448687.96116175.2+   GGAGCACAAGGAACAGGAGCTGATGG
SRR448688.23026856.2+   GGAGCACAAGGAACAGGAGCTGATGG
SRR448688.26519151.1+   GGAGCACAAGGAACAGGAGCTGATGG
SRR448688.4725251.1+   GGAGCACAAGGAACAGGAGCTGATGG
SRR448688.75228225.1+   GGAGCACAAGGAACAGGAGCTGATGG
SRR448688.85519119.1+   GGAGCACAAGGAACAGGAGCTGATGG
SRR448688.9307577.1+   GGAGCACAAGGAACAGGAGCTGATGG
SRR448687.40049376.1-   GGAGCACAAGGAACAGGAGCTGATGGG
SRR448699.28603845.1+   GGAGCACAAGGAACAGGAGCTGATGGG
SRR448686.90650419.2+   GGAGCACAAGGAACAGGAGCTGATGGGG
SRR448686.111978264.2-   GGAGCACAAGGAACAGGAGCTGATGGGGT
SRR448687.4804306.2+   GGAGCACAAGGAACAGGAGCTGATGGGGT
SRR448688.623745.1+   GGAGCACAAGGAACAGGAGCTGATGGGGTT
SRR448690.59500894.1-   GGAGCACAAGGAACAGGAGCTGATGGGGTT
SRR448690.82179856.1-   GGAGCACAAGGAACAGGAGCTGATGGGGTT
SRR448699.51426342.2-   GGAGCACAAGGAACAGGAGCTGATGGGGTT
SRR448683.58306359.1+   GGAGCACAAGGAACAGGAGCTGATGGGGTTGA
SRR448687.57896852.1+   GGAGCACAAGGTACAGGAGCTGATGGGGTTGA
SRR448687.58131717.1-   GGAGCACAAGGAACAGGAGCTGATGGGGTTGA
SRR448699.74102933.2+   GGAGCACAAGGAACAGGAGCTGATGGGGTTGAG
SRR448694.13441487.2-   GGAGCACAAGGAACAGGAGCTGATGGGGTTGAGC
SRR448675.24551560.2-   GGAGCACAAGGAACAGGAGCTGATGGGGTTGAGCT
SRR448675.82235779.2-   GGAGCACAAGGAACAGGAGCTGATGGGGTTGAGCT
SRR448694.2375465.1-   GGAGCACAAGGAACAGGAGCTGATGGGGTTGAGCT
SRR448675.59564553.1+   GGAGCACAAGGAACAGGAGCTGATGGGGTTGAGCTG
SRR448675.81560484.2+   GGAGCACAAGGAACAGGAGCTGATGGGGTTGAGCTG
SRR448683.13133273.1+   GGAGCACAAGGAACAGGAGCTGATGGGGTTGAGCTGC
SRR448686.35001025.1+   GGAGCACAAGGAACAGGAGCTGATGGGGTTGAGCTGC
SRR448686.78011325.2-   GGAGCACAAGGAACAGGAGCTGATGGGGTTGAGCTGCA
SRR448690.52257033.2+   GGAGCACAAGGAACAGGAGCTGATGGGGTTGAGCTGCAC
SRR448675.104363339.1+   GGAGCACAAGGAACAGGAGCTGATGGGGTTGAGCTGCACA
SRR448675.24783207.2+   GGAGCACAAGGAACAGGAGCTGATGGGGTTGAGCTGCACA
SRR448686.1056904.2+   GGAGCACAAGGAACAGGAGCTGATGGGGTTGAGCTGCACA
SRR448699.47956681.2+   GGAGCACAAGGAACAGGAGCTGATGGGGTTGAGCTGCACA
SRR448683.90888613.2+   GGAGCACACGGAACAGGAGCTGATGGGGGTGGGCTGCACAT
SRR448687.88915780.1+   GGAGCACAAGGAACAGGAGCTGATGGGGTTGAGCTGCACAT
SRR448683.4919531.2+   GGAGCACAAGGAACAGGAGCTGATGGGGTTGAGCTGCACATC
SRR448687.55340056.2-   GGAGCACAAGGAACAGGAGCTGATGGGGTTGAGCTGCACATCC
SRR448683.53372918.2-   GGAGCCCAAGGAACAGGAGCTGATGGGGTTGAGCTGCACATCCA
SRR448694.19498543.1-   GGAGCACAAGGAACAGGAGCTGATGGGGTTGAGCTGCACATCCAC
SRR448675.92739025.2+   GGAGCACAAGGAACAGGAGCTGATGGGGTTGAGCTGCACATCCACAC
SRR448686.20664457.2+   GGAGCACAAGGAACAGGAGCTGATGGGGTTGAGCTGCACATCCACAC
SRR448694.5989384.1+   GGAGCACAAGGAACAGGAGCTGATGGGGTTGAGCTGCACATCCACAC
SRR448694.71735553.1+   GGAGCACAAGGAACAGGAGCTGATGGGGTTGAGCTGCACATCCACAC
SRR448694.92596162.1+   GGAGCACAAGGAACAGGAGCTGATGGGGTTGAGCTGCACATCCACAC
SRR448681.7497961.1+   GGAGCACAAGGAACAGGAGCTGATGGGGTTGAGCTGCACATCCACACT
SRR448688.25191493.1-   GGAGCACAAGGAACAGGAGCTGATGGGGTTGAGCTGCACATCCACACT
SRR448688.28589525.1-   GGAGCACAAGGAACAGGAGCTGATGGGGTTGAGCTGCACATCCACACT
SRR448688.32670682.1-   GGAGCACAAGGAACAGGAGCTGATGGGGTTGAGCTGCACATCCACACT
SRR448694.76767889.1+   GGAGCACAAGGAACAGGAGCTGATGGGGTTGAGCTGCACATCCACACTG
SRR448686.83118909.2+   GGAGCACAAGGAACAGGAGCTGATGGGGTTGAGCTGCACATCCACACTGGGTG
SRR448681.69796216.2-   GGAGCACAAGGAACAGGAGCTGATGGGGTTGAGCTGCACATCCACACTGGGTGGATG
SRR448681.88931306.2-   GGAGCACAAGGAACAGGAGCTGATGGGGTTGAGCTGCACATCCACACTGGGTGGATG
SRR448686.86029142.1-   GGAGCACAAGGAACAGGAGCTGATGGGGTTGAGCTGCACATCCACACTGGGTGGATGG
SRR448683.59297888.2+   GGAGCACAAGGAACAGGAGCTGATGGGGTTGAGCTGCACATCCACACTGGGTGGATGGC
SRR448675.104128282.1+   GGATCACAAGGAACAGGAGCTGATGGGGTTGAGCTGCACATCCACACTGGGTGGATGGCA
SRR448675.24700540.2-   GGAGCACAAGGAACAGGAGCTGATGGGGTTGAGCTGCACATCCACACTGGGTGGATGGCA
SRR448675.26176825.2-   GGAGCACAAGGAACAGGAGCTGATGGGGTTGAGCTGCACATCCACACTGGGTGGATGGCA
SRR448675.34371245.2-   GGAGCACAAGGAACAGGAGCTGATGGGGTTGAGCTGCACATCCACACTGGGTGGATGGCA
SRR448675.3711092.2-   GGAGCACAAGGAACAGGAGCTGATGGGGTTGAGCTGCACATCCACACTGGGTGGATGGCA
SRR448675.41712885.2-   GGAGCACAAGGAACAGGAGCTGATGGGGTTGAGCTGCACATCCACACTGGGTGGATGGCA
SRR448675.76497237.1-   GGAGCACAAGGAACAGGAGCTGATGGGGTTGAGCTGCACATCCACACTGGGTGGATGGCA
SRR448681.18143961.2-   GGAGCACAAGGAACAGGAGCTGATGGGGTTGAGCTGCACATCCACACTGGGTGGATGGCA
SRR448681.21545235.2+   GGAGCACAAGGAACAGGAGCTGATGGGGTTGAGCTGCACATCCACACTGGGTGGATGGCA
SRR448681.32854092.1+   GGAGCACAAGGAACAGGAGCTGATGGGGTTGAGCTGCACATCCACACTGGGTGGATGGCA
SRR448681.34420081.1+   GGAGCACAAGGAACAGGAGCTGATGGGGTTGAGCTGCACATCCACACTGTGTGGATGGCA

```
SRR448681.3793565.1+      GGAGCACAAGGAACAGGAGCTGATGGGGTTGAGCTGCACATCCACACTGGGTGGATGGCA
SRR448681.47301511.2-     GGAGCACAAGGAACAGGAGCTGATGGGGTTGAGCTGCACATCCACACTGGGTGGATGGCA
SRR448681.50099907.1+     GGAGCACAAGGAACAGGAGCTGATGGGGTTGAGCTGCACATCCACACTGGGTGGATGGCA
SRR448681.58636992.2-     GGAGCACAAGGAACAGGAGCTGATGGGGTTGAGCTGCACATCCACACTGGGTGGATGGCA
SRR448681.68030745.1+     GGAGCACAAGGAACAGGAGCTGATGGGGTTGAGCTGCACATCCACACTGGGTGGATGGCA
SRR448681.7337942.2+      GGAGCACAAGGAACAGGAGCTGATGGGGTTGAGCTGCACATCCACACTGGGTGGATGGCA
SRR448681.86267206.1-     GGAGCACAAGGAACAGGAGCTGATGGGGTTGAGCTGCACATCCACACTGGGTGGATGGCA
SRR448683.14290636.1-     GGAGCACAAGGAACAGGAGCTGATGGGGTTGAGCTGCACATCCACACTGGGTGGATGGCA
SRR448683.19193053.2-     GGAGCACAAGGAACAGGAGCTGATGGGGTTGAGCTGCACATCCACACTGGGTGGATGGCA
SRR448683.45914950.1-     GGAGCACAAGGAACAGGAGCTGATGGGGTTGAGCTGCACATCCACACTGGGTGGATGGCA
SRR448683.53184158.2-     GGAGCACAAGGAACAGGAGCTGATGGGGTTGAGCTGCACATCCACACTGGGTGGATGGCA
SRR448683.91457999.2-     GGAGCACAAGGAACAGGAGCTGATGGGGTTGAGCTGCACATCCACACTGGGTGGATGGCA
SRR448686.24956981.2-     GGAGCACAAGGACCAGGAGCTGATGGGGTTGAGCTGCACATCCACACTGGGTGGATGGCA
SRR448686.25320476.1-     GGAGCACAAGGAACAGGAGCTGATGGGGTTGAGCTGCACATCCACACTGGGTGGATGGCA
SRR448686.34129339.2+     GGAGCACAAGGAACAGGAGCTGATGGGGTTGAGCTGCACATCCACACTGGGTGGATGGCA
SRR448686.46585949.2-     GGAGCACAAGGAACAGGAGCTGATGGGGTTGAGCTGCACATCCACACTGGGTGGATGGCA
SRR448686.99783446.1-     GGAGCACAAGGAACAGGAGCTGATGGGGTTGAGCTGCACATCCACACTGGGTGGATGGCA
SRR448687.10881609.1+     GGAGCACAAGGAACAGGAGCTGATGGGGTTGAGCTGCACATCCACACTGGGTGGATGGCA
SRR448687.42732058.1-     GGAGCACAAGGAACAGGAGCTGATGGGGTTGAGCTGCACATCCACACTGGGTGGATGGCA
SRR448687.47390517.2+     GGAGCACAAGGAACAGGAGCTGATGGGGTTGAGCTGCACATCCACACTGGGTGGATGGCA
SRR448687.59753368.2+     GGAGCACAAGGAACAGGAGCTGATGGGGTTGAGCTGCACATCCACACTGGGTGGATGGCA
SRR448687.62503016.1+     GGAGCACAAGGAACAGGAGCTGATGGGGTTGAGCTGCACATCCACACTGGGTGGATGGCA
SRR448687.65214307.1+     GGAGCACAAGGAACAGGAGCTGATGGGGTTGAGCTGCACATCCACACTGGGTGGATGGCA
SRR448687.6574725.1-      GAAGCACAAGGAACAGGAGCTGATGGGGTTGAGCTGCACATCCACACTGGGTGGATGGCA
SRR448687.71782978.2+     GGAGCACAAGGAACAGGAGCTGATGGGGTTGAGCTGCACATCCACACTGGGTGGATGGCA
SRR448687.88021195.1+     GGAGCACAAGGAACAGGAGCTGATGGGGTTGAGCTGCACATCCACACTGGGTGGATGGCA
SRR448687.89910530.2+     GGAGCACAAGGAACAGGAGCTGATGGGGTTGAGCTGCACATCCACACTGGGTGGATGGCA
SRR448687.994456.2+       GGAGCACAAGGAACAGGAGCTGATGGGGTTGAGCTGCACATCCACACTGGGTGGATGGCA
SRR448688.48262799.2-      GAGCACAAGGAACAGGAGCTGATGGGGTTGAGCTGCACATCCACACTGG
SRR448688.48699444.2-      GAGCACAAGGAACAGGAGCTGATGGGGTTGAGCTGCACATCCACACTGG
SRR448688.7762234.2-       GAGCACAAGGAACAGGAGCTGATGGGGTTGAGCTGCACATCCACACTGG
SRR448699.46619104.2-      GAGCACAAGGAACAGGAGCTGATGGGGTTGAGCTGCACATCCACACTGG
SRR448675.81560484.1-      GAGCACAAGGAACAGGAGCTGATGGGGTTGAGCTGCACATCCACACTGGGTGGATGGCA
SRR448683.5610403.1+         GCACAAGGAACAGGAGCTGATGGGGTTGAGCTGCACATCCACACTGGGTGGATGGCA
SRR448681.54132602.1+         CACAAGGAACAGGAGCTGATGGGGTTGAGCTGCACATCCACACTGGGTGGATGGCA
SRR448699.68479777.2+          ACAAGGAACAGGAGCTGATGGGGTTGAGCTGCACATCCACACTGGGTGG
SRR448699.81987907.2+          ACAAGGAACAGGAGCTGATGGGGTTGAGCTGCACATCCACACTGGGTGG
SRR448694.104011077.2-          CAAGGAACAGGAGCTGATGGGGTTGAGCTGCACATCCACACTGGGTGGA
SRR448694.85217927.2-           CAAGGAACAGGAGCTGATGGGGTTGAGCTGCACATCCACACTGGGTGGA
SRR448699.5431226.1+            CAAGGAACAGGAGCTGATGGGGTTGAGCTGCACATCCACACTGGGTGGA
SRR448694.75858924.1+            AAGGAACAGGAGCTGATGGGGTTGAGCTGCACATCCACACTGGGTGGAT
SRR448683.54546641.2+            AAGGAACAGGAGCTGATGGGGTTGAGCTGCACATCCACACTGGGTGGATGGCA
SRR448687.72215272.1+             AGGAACAGGAGCTGATGGGGTTGAGCTGCACATCCACACTGGGTGGATGGCA
SRR448686.95438255.1-               GAACAGGAGCTGATGGGGTTGAGCTGCACATCCACACTGGGTGGATGGCA
SRR448683.90493668.1+                AACAGGAGCTGATGGGGTTGAGCTGCACATCCACACTGGGTGGATGGCA
SRR448688.77432328.1+                AACAGGAGCTGATGGGGTTGAGCTGCACATCCACACTGGGTGGATGGCA
SRR448681.21344736.2+                 ACAGGAGCTGATGGGGTTGAGCTGCACATCCACACTGGGTGGATGGCA
SRR448687.61688358.2-                 ACAGGAGCTGATGGGGTTGAGCTGCACATCCACACTGGGTGGATGGCA
SRR448683.81894875.1+                   AGGAGCTGATGGGGTTGAGCTGCACATCCACACTGGGTGGATGGCA
SRR448683.37959705.1-                    GGAGCTGATGGGGTTGAGCTGCACATCCACACTGGGTGGATGGCA
SRR448675.34322357.2+                      AGCTGATGGGGTTGAGCTGCACATCCACACTGGGTGGATGGCA
SRR448699.110207694.1-                      GCTGATGGGGTTGAGCTGCACATCCACACTGGGTGGATGGCA
SRR448675.104363339.2-                       CTGAGGGGGTTGAGCTGCACATCCACACTGGGTGGATGGCA
SRR448675.24783207.1-                        CTGATGGGGTTGAGCTGCACATCCACACTGGGTGGATGGCA
SRR448681.63302088.2+                        CTGATGGGGTTGAGCTGCACATCCACACTGGGTGGATGGCA
SRR448683.16844924.2+                        CTGATGGGGTTGAGCTGCACATCCACACTGGGTGGATGGCA
SRR448694.46724614.2+                        CTGATGGGGTTGAGCTGCACATCCACACTGGGTGGATGGCA
SRR448694.58076743.2-                        CTGATGGGGTTGAGCTGCACATCCACACTGGGTGGATGGCA
SRR448694.70323075.2-                        CTGATGGGGTTGAGCTGCACATCCACACTGGGTGGATGGCA
SRR448681.82661095.1+                         TGATGGGGTTGAGCTGCACATCCACACTGGGTGGATGGCA
SRR448683.31908163.1-                          GATGGGGTTGAGCTGCACATCCACACTGGGTGGATGGCA
SRR448687.39768395.1-                          GATGGGGTTGAGCTGCACATCCACACTGGGTGGATGGCA
SRR448675.59564553.2-                           ATGGGGTTGAGCTGCACATCCACACTGGGTGGATGGCA
SRR448686.59598963.1-                           ATGGGGTTGAGCTGCACATCCACACTGGGTGGATGGCA
SRR448681.73558260.2+                            TGGGGTTGAGCTGCACATCCACACTGGGTGGATGGCA
SRR448681.7497961.2-                             TGGGGTGGAGCTGCACATCCACACTGGGTGGATGGCA
SRR448683.56266056.2+                            TGGGGTTGAGCTGCACATCCACACTGGGTGGATGGCA
SRR448683.9537018.2-                             TGGGGTTGAGCTGCACATCCACACTGGGTGGATGGCA
SRR448687.31855042.1+                            TGGGGTTGAGCTGCACATCCACACTGGGTGGATGGCA
SRR448699.22573002.2+                             GGGGTTGAGCTGCACATCCACACTGGGTGGATGGCA
SRR448675.92739025.1-                              GGGTTGAGCTGCACATCCACACTGGGTGGATGGCA
SRR448675.103252359.1+                              GGTTGAGCTGCACATCCACACTGGGTGGATGGCA
SRR448683.42166054.2-                               GGTTGAGCTGCACATCCACACTGGGTGGATGGCA
SRR448699.12313355.1-                                GTTGAGCTGCACATCCACACTGGGTGGATGGCA
SRR448699.3628409.1-                                 GTTGAGCTGCACATCCACACTGGGTGGATGGCA
SRR448687.76742462.2+                                 TTGAGCTGCACATCCACACTGGGTGGATGGCA
SRR448683.2954521.2-                                    GAGCGGCACATCCACACTGGGTGGATGGGA
SRR448686.108023284.1-                                  GAGCTGCACATCCACACTGGGTGGATGGCA
SRR448687.51419033.1-                                   GAGCTGCACATCCACACTGGGTGGATGGCA
SRR448699.67140813.2+                                   GAGCTGCACATCCACACTGGGTGGATGGCA
SRR448699.68906004.1-                                   GAGCTGCACATCCACACTGGGTGGATGGCA
SRR448702.16481712.1+                                   GAGCTGCACATCCACACTGGGTGGATGGCA
SRR448702.18421897.1+                                   GAGCTGCACATCCACACTGGGTGGATGGCA
SRR448702.18996655.1+                                   GAGCTGCACATCCACACTGGGTGGATGGCA
```

```
SRR448702.22776614.1+                                  GAGCTGCACATCCACACTGGGTGGATGGCA
SRR448702.30902813.1+                                  GAGCTGCACATCCACACTGGGTGGATGGCA
SRR448702.75582168.1+                                  GAGCTGCACATCCACACTGGGTGGATGGCA
SRR448687.14912879.2-                                   AGCTGCACATCCACCCTGGGTGGATGGCA
SRR448681.97626341.1+                                    GCTGCACATCCACACTGGGTGGATGGCA
SRR448675.29937986.2+                                     CTGCACATCCACACTGGGTGGATGGCA
SRR448686.106844158.2+                                    CTGCACATCCACACTGGGTGGATGGCA
SRR448694.44732468.1-                                     CTGCACATCCACACTGGGTGGATGGCA
SRR448699.87157854.1-                                      TGCACATCCACACTGGGTGGATGGCA
SRR448681.66489052.2+                                       GCACATCCACACTGGGTGGATGGCA
SRR448683.88276819.2-                                       GCACATCCACACTGGGTGGATGGCA
SRR448683.92212587.1-                                       GCACATCCACGCTGGGTGGATGGCA
SRR448702.56722010.1-                                       GCACATCCACACTGGGTGGATGGCA
SRR448675.100859301.1+                                       CACATCCACACTGGGTGGATGGCA
SRR448687.65209642.1+                                        CACATCCACACTGGGTGGATGGCA
SRR448694.110412111.1-                                       CACATCCACACTGGGTGGATGGCA
SRR448694.13924592.1-                                        CACATCCACACTGGGTGGATGGCA
SRR448694.85516447.1-                                        CACATCCACACTGGGTGGATGGCA
SRR448683.79752369.1-                                         ACATCCACACTGGGTGGATGGCA
SRR448687.23078728.1-                                         ACATCCACACTGGGTGGATGGCA
SRR448694.88407078.1+                                         ACATCCACACTGGGTGGATGGCA
SRR448686.1930452.1-                                            ATCCACACTGGGTGGATGGCA
SRR448675.88926626.1+                                            TCCACACTGGGTGGATGGCA
SRR448687.74386594.2+                                            TCCACACTGGGTGGATGGCA
SRR448687.89718471.2+                                             CCACACTGGGTGGATGGCA
SRR448690.81061662.2+                                              CACACTGGGTGGATGGCA
SRR448690.91569127.2+                                              CACACTGGGTGGATGGCA
SRR448694.85881197.1-                                              CACACTGGGTGGATGGCA
SRR448683.67390564.1-                                                CACTGGGTGGATGGCA
SRR448683.69167326.1-                                                  CTGGGTGGATGGCA
SRR448675.104128282.2-                                                  TGGGTGGATGGCA
SRR448683.72556731.1-                                                    GGGTGGATGCCT
SRR448675.65770019.1+                                                      GTGGATGGCA
SRR448690.52612250.1-                                                       TGGATGGCA
SRR448683.15990850.1-                                                        GGATGGCA
SRR448681.50099907.2-                                                         GATGGCA
SRR448688.40173627.2-                                                            GGCA
SRR448686.91310581.1+                                                             GCA
SRR448681.39820355.1+                                                              CA
SRR448683.64053908.2+                                                              CA
SRR448687.69057351.2-                                                              CA
SRR448694.18506634.2+                                                              CA
SRR448694.67361378.2+                                                              CA
SRR448687.33039334.1+                                                               A
SRR448699.87199114.1-                                                               A
                         ------------------------------------------------------------
consensus                GGAGCACAAGGAACAGGAGCTGATGGGGTTGAGCTGCACATCCACACTGGGTGGATGGCA

                             .    :    .    :    .    :    .    :    .    :    .    :
SRR448687.994456.2+      TGTG
SRR448675.41712885.2-    TGTGG
SRR448683.45914950.1-    TGTGG
SRR448681.58636992.2-    TGTGGAT
SRR448686.34129339.2+    TGTGGAT
SRR448699.110207694.1-   TGTGGAT
SRR448694.46724614.2+    TGTGGATT
SRR448694.58076743.2-    TGTGGATT
SRR448694.70323075.2-    TGTGGATT
SRR448681.34420081.1+    CGTGGATTC
SRR448686.99783446.1-    TGTGGATTCA
SRR448699.22573002.2+    TGTGGATTCAGCC
SRR448681.86267206.1-    TGTGGATTCAGCCC
SRR448687.65214307.1+    TGTGGATTCAGCCC
SRR448683.14290636.1-    TGTGGATTCAGCCCT
SRR448686.46585949.2-    TGTGGATTCAGCCCT
SRR448687.62503016.1+    TGTGGATTCAGCCCT
SRR448681.47301511.2-    TGTGGATTCAGCCCTC
SRR448699.12313355.1-    TGTGGATTCAGCCCTC
SRR448699.3628409.1-     TGTGGATTCAGCCCTC
SRR448675.104128282.1+   TGTGGATTCAGCCCTCA
SRR448675.24700540.2-    TGTGGATTCAGCCCTCA
SRR448675.26176825.2-    TGTGGATTCAGCCCTCAT
SRR448687.71782978.2+    TGTGGATTCAGCCCTCAT
SRR448681.50099907.1+    TGTGGATTCAGCCCTCATG
SRR448687.89910530.2+    TGTGGATTCAGCCCTCATG
SRR448699.67140813.2+    TGTGGATTCAGCCCTCATG
SRR448699.68906004.1-    TGTGGATTCAGCCCTCATG
SRR448702.16481712.1+    TGTGGATTCAGCCCTCATG
SRR448702.18421897.1+    TGTGGATTCAGCCCTCATG
SRR448702.18996655.1+    TGTGGATTCAGCCCTCATG
SRR448702.22776614.1+    TGTGGATTCAGCCCTCATG
SRR448702.30902813.1+    TGTGGATTCAGCCCTCATG
SRR448702.75582168.1+    TGTGGATTCAGCCCTCATG
SRR448683.19193053.2-    TGTGGATTCAGCCCTCATGG
```

SRR448687.59753368.2+ TGTGGATTCAGCACTCATGG
SRR448675.3711092.2- TGTGGATTCAGCCCTCATGGA
SRR448681.18143961.2- TGTGGATTCAGCCCTCATGGA
SRR448694.44732468.1- TGTGGATTCAGCCCTCATGGAG
SRR448683.91457999.2- TGTGGATTCAGCCCTCATGGAGA
SRR448699.87157854.1- TGTGGATTCAGCCCTCATGGAGA
SRR448702.56722010.1- TGTGGATTCAGCCCTCATGGAGAG
SRR448683.53184158.2- TGTGGATTCAGCCCTCATGGAGAGC
SRR448694.110412111.1- TGTGGATTCAGCCCTCATGGAGAGC
SRR448694.13924592.1- TGTGGATTCAGCCCTCATGGAGAGC
SRR448694.85516447.1- TGTGGATTCAGCCCTCATGGAGAGC
SRR448687.10881609.1+ TGTGGATTTAGCCCTCATGGAGAGCT
SRR448687.88021195.1+ TGTGGATTCAGCCCTCATGGAGAGCT
SRR448694.88407078.1+ TGTGGATTCAGCCCTCATGGAGAGCT
SRR448675.76497237.1- TGTGGATTCAGCCCTCATGGAGAGCTGCC
SRR448681.68030745.1+ TGTGGATTCAGCCCTCATGGAGAGCTGCC
SRR448681.21545235.2+ TGTGGATTCAGCCCTCATGGAGAGCTGCCT
SRR448687.42732058.1- TGTGGATTCAGCCCTCATGGAGAGCTGCCTG
SRR448687.47390517.2+ TGTGGATTCAGCCCTCATGGAGAGCTGCCTG
SRR448690.81061662.2+ TGTGGATTCAGCCCTCATGGAGAGCTGCCTG
SRR448690.91569127.2+ TGTGGATTCAGCCCTCATGGAGAGCTGCCTG
SRR448694.85881197.1- TGTGGATTCAGCCCTCATGGAGAGCTGCCTG
SRR448675.34371245.2- TGTGGATTCAGCCCTCATGGAGAGCTGCCTGC
SRR448681.32854092.1+ TGTGGATTCAGCCCTCATGGAGAGCTGCCTGCAT
SRR448681.3793565.1+ TATGGATTCAGCCCTCATGGAGAGCTGCCTGGATC
SRR448681.7337942.2+ TGTGGATTCAGCCCTCATGGAGAGCTGCCTGGATC
SRR448687.6574725.1- TGTGGATTCAGCCCTCATGGAGAGCTGCCTGGATCTG
SRR448686.24956981.2- TGTGGATTCAGCCCTCATGGAGAGCTGCCTGGATCTGAC
SRR448686.25320476.1- TGTGGATTCAGCCCTCATGGAGAGCTGCCTGGATCTGACA
SRR448690.52612250.1- TGTGGATTCAGCCCTCATGGAGAGCTGCCTGCATCTGACA
SRR448675.81560484.1- TGTGGATTCAGCCCTCATGGAGAGCTGCCTGGATCTGACAT
SRR448683.5610403.1+ TGTGGATTCAGCCCTCATGGAGAGCTGCCTGGATCTGACATTG
SRR448681.54132602.1+ TGTGGATTCAGCCCTCATGGAGAGCTGCCTGCATCTGACATTGT
SRR448688.40173627.2- TGTGGATTCAGCCCTCATGGAGAGCTGCCTGGATCTGACATTGTG
SRR448683.54546641.2+ TGTGGATTCAGCCCTCATGGAGAGCTGCCTGCATCTGACATTGTGAG
SRR448694.18506634.2+ TGTGGATTCAGCCCTCATGGAGAGCTGCCTGGATCTGACATTGTGAG
SRR448694.67361378.2+ TGTGGATTCAGCCCTCATGGAGAGCTGCCTGGATCTGACATTGTGAG
SRR448687.72215272.1+ TGTGGATTCAGCCCTCATGGAGAGCTGCCTGCATCTGGCATTGTGAGA
SRR448699.87199114.1- TGTGGATTCAGCCCTCATGGAGAGCTGCCTGCATCTGACATTGTGAGA
SRR448688.43048732.1+ TGTGGATTCAGCCCTCATGGAGAGCTGCCTGCATCTGACATTGTGAGAG
SRR448699.1447497.2- TGTGGATTCAGCCCTCATGGAGAGCTGCCTGGATCTGACATTGTGAGAG
SRR448686.95438255.1- TGTGGATTCAGCCCTCATGGAGAGCTGCCTGGATCTGACATTGTGAGAGG
SRR448683.90493668.1+ TGTGGATTCAGCCCTCATGGAGAGCTGCCTGGATCTGACATTGTGAGAGGA
SRR448681.21344736.2+ TGTGGATTCAGCCCTCATGGAGAGCTGCCTGGATCTGACATTGTGAGAGGAG
SRR448683.31908163.1- TGTGGATTCAGCCCTCATGGAGAGCTGCCTGCATCTGACATTGTGAGAGGAG
SRR448687.61688358.2- TGTGGATTCAGCCCTCATGGAGAGCTGCCTGGATCTGACATTGTGAGAGGAG
SRR448681.66489052.2+ TGTGGATTCAGCCCTCATGGAGAGCTGCCTGCATCAGACATTGTGAGAGGAGG
SRR448683.81894875.1+ TGTGGATTCAGCCCTCATGGAGAGCTGCCTGGATCTGACATTGTGAGAGGAGGT
SRR448683.37959705.1- TGTGGATTCAGCCCTCATGGAGAGCTGCCTGCATCTGACATTGTGAGAGGAGGTG
SRR448675.34322357.2+ TGTGGATTCAGCCCTCATGGAGAGCTGCCTGGATCTGACATTGTGAGAGGAGGTGCC
SRR448675.104363339.2- TGTGGATTCATCCCTCATGGAGAGCTGCCTGCATCTGACATTGTGAGAGGAGGTGCCTT
SRR448675.24783207.1- TGTGGATTCAGCCCTCATGGAGAGCTGCCTGCATCTGACATTGTGAGAGGAGGTGCCTT
SRR448681.63302088.2+ TGTGGATTCAGCCCTCATGGAGAGCTGCCTGCATCTGACATTGTGAGAGGAGGTGCCTT
SRR448683.16844924.2+ TGTGGATTCAGCCCTCATGGAGAGAAGCCTGCATCTGACATTGTGAGAGGAGGTGCCTT
SRR448675.100859301.1+ TGTGGATTCAGCCCTCATGGAGAGCTGCCTGGATCTGACATTGTGAGAGGAGGTTCCTTC
SRR448675.103252359.1+ TGTGGATTCAGCCCTCATGGAGAGCTGCCTGCATCTGACATTGTGAGAGGAGGTGCCTTC
SRR448675.104128282.2- TGTGGATTCAGCCCTCATGGAGAGCTGCCTGGATCTGACATTGTGAGAGGAGGTGCCTTC
SRR448675.29937986.2+ TGTGGATTCAGCCCTCATGGAGAGCTGCCTGCATCTGACATTGTGAGAGGAGGTGCCTTC
SRR448675.59564553.2- TGTGGATTCAGCCCTCATGGAGAGCTGCCTGGATCTGACATTGTGAGAGGAGGTGCCTTC
SRR448675.65770019.1+ TGTGGATTCAGCCCTCATGGAGAGCTGCCTGCATCTGACATTGTGAGAGGAGGTGCCTTC
SRR448675.88926626.1+ TGTGGATTCAGCCCTCATGGAGAGCTGCCTGCATCTGACATTGTGAGAGGAGGTGCCTTC
SRR448675.92739025.1- TGTGGATTCAGCCCTCATGGAGAGCTGCCTGGATCTGACATTGTGAGAGGAGGTGCCTTC
SRR448681.39820355.1+ TGTGGATTCAGCCCTCATGGAGAGCTGCCTGCATCTGACATTGTGAGAGGAGGTGCCTTC
SRR448681.50099907.2- TGTGGATTCAGCCCTCATGGAGAGCTGCCTGGATCTGACATTGTGAGAGGAGGTGCCTTC
SRR448681.73558260.2+ TGTGGATTCAGCCCTCATGGAGAGCTGCCTGGATCTGACATTGTGAGAGGAGGTGCCTTC
SRR448681.7497961.2- TGTGGATTCAGCCTTCATGGAGAGCTGCCTGCATCTGACATTGTGAGAGGAGGTGCCTTC
SRR448681.82661095.1+ TGTGGATTCAGCCCTCATGGAGAGCTGCCTGCATCTGACATTGTGAGAGGCGGCGCCTTC
SRR448681.97626341.1+ TGTGGATTCAGCCCTCATGGAGAGCTGCCTGCATCTGACATTGTGAGAGGAGGTGCCTTC
SRR448683.15990850.1- TGTGGATTCAGCCCTCATGGAGAGCTGCCTGGATCTGACATTGTGAGAGGAGGTGCCTTC
SRR448683.2954521.2- TGTGGGTTCAGCCCTCATGGAGAGCTGCCTGCATCTGACATTGTGAGAGGAGGTGCCTTC
SRR448683.42166054.2- TGTGGATTCAGCCCTCATGGAGAGCTGCCTGGATCTGACATTGTGAGAGGAGGTGCCTTC
SRR448683.56266056.2+ TGTGGATTCAGCCCTCATGGAGAGCTGCCTGCATCTGACATTGTGAGAGGAGGTGCCTTC
SRR448683.64053908.2+ TGTGGATTCAGCCCTCATGGAGAGCTGCCTGGATCTGACATTGTGAGAGGAGGTGCCTTC
SRR448683.67390564.1- TGTGGATTCAGCCCTCATGGAGAGCTGCCTGGATCTGACATTGTGAGAGGAGGTGCCTTC
SRR448683.69167326.1- TGTGGATTCAGCCCTCATGGAGAGCTGCCTGGATCTGACATTGTGAGAGGAGGTGCCTTC
SRR448683.72556731.1- TGTGGATTCAGCCCTCATGGGGAGCTGCCTGCATCTGACATTGTGAGAGGAGGTGCCTTC
SRR448683.79752369.1- TGTGGATTCAGCCCTCATGGAGAGCTGCCTGGATCTGACATTGTGAGAGGAGGTGCCTTC
SRR448683.88276819.2- TGTGGATTCAGCCCTCATGGAGAGCTGCCTGCATCTGACATTGTGAGAGGAGGTGCCTTC
SRR448683.92212587.1- TGTGGATTCAGCCCTCATGGAGAGCTGCCTGCATCTGACATTGTGAGAGGAGGTGCCTTC
SRR448683.9537018.2- TGTGGATTCAGCCCTCATGGAGAGCTGCCTGGATCTGACATTGTGAGAGGAGGTGCCTTC
SRR448686.106844158.2+ TGTGGATTCAGCCCTCATGGAGAGCTGCCTGGATCTGACATTGTGAGAGGAGGTGCCTTC
SRR448686.108023284.1- TGTGGATTCAGCCCTCATGGAGAGCTGCCTGCATCTGACATTGTGAGAGGAGGTGCCTTC
SRR448686.1930452.1- TGTGGATTCAGCCCTCATGGAGAGCTGCCTGGATCTGACATTGTGAGAGGAGGTGCCTTC
SRR448686.59598963.1- TGTGGATTCAGCCCTCATGGAGAGCTGCCTGGATCTGACATTGTGAGAGGAGGTGCCTTC

```
SRR448686.91310581.1+    TGTGGATTCAGCCCTCATGGAGAGCTGCCTGCATCTGACATTGTGAGAGGAGGTGCCTTC
SRR448687.14912879.2-    TGTGGATTCATCCCTCATGGAGAGCTGCCTGCATCTGACATTGTGAGAGGAGGTGCCTTC
SRR448687.23078728.1-    TGTGGATTCAGCCCTCATGGAGAGCTGCCTGCATCTGACATTGTGAGAGGAGGTGCCTTC
SRR448687.31855042.1+    TGTGGATTCAGCCCTCATGGAGAGCTGCCTGCATCTGACATTGTGAGAGGAGGTGCCTTC
SRR448687.33039334.1+    TGTGGATTCAGCCCTCATGGAGAGCTGCCTGCATCTGACATTGTGAGAGGAGGTGCCTTC
SRR448687.39768395.1-    TGTGGATTCAGCCCTCATGGAGAGCTGCCTGGATCTGACATTGTGAGAGGAGGTGCCTTC
SRR448687.51419033.1-    TGTGGATTCAGCCCTCATGGAGAGCTGCCTGCATCTGACATTGTGAGAGGAGGTGCCTTC
SRR448687.65209642.1+    TGTGGATTCAGCCCTCATGGAGAGCTGCCTGGATCTGACATTGTGAGAGGAGGTGCCTTC
SRR448687.69057351.2-    TGTGGATTCAGCCCTCATGGAGAGCTGCCTGCATCTGACATTGTGAGAGGAGGTGCCTTC
SRR448687.74386594.2+    TGTGGATTCAGCCCTCATGGAGAGCTGCCTGCATCTGACATTGTGAGAGGAGGTGCCTTC
SRR448687.76742462.2+    TGTGGATTCAGCCCTCATGGAGAGCTGCCTGCATCTGACATTGTGAGAGGAGGCGCCTTC
SRR448687.89718471.2+    TGTGGATTCAGCCCTCATGGAGAGCTGCCTGCATCTGACATTGTGAGAGGAGGTGCCTTC
SRR448694.90058685.1-     GTGGATTCAGCCCTCATGGAGAGCTGCCTGCATCTGACATTGTGAGAGG
SRR448699.57300782.1+      TGGATTCAGCCCTCATGGAGAGCTGCCTGCATCTGACATTGTGAGAGGA
SRR448699.105936149.1-      GGATTCAGCCCTCATGGAGAGCTGCCTGGATCTGACATTGTGAGAGGAG
SRR448686.94018257.1-       GGATTCAGCCCTCATGGAGAGCTGCCTGCATCTGACATTGTGAGAGGAGGTGCCTTC
SRR448681.21545235.1-         ATTCAGCCCTCATGGAGAGCTGCCTGCATCTGACATTGTGAGAGGAGGTGCCTTC
SRR448681.4625807.1+          ATTCAGCCCTCATGGAGAGCTGCCTGCATCTGACATTGTGAGAGGAGGTGCCTTC
SRR448687.19754537.1-          TTCAGCCCTCATGGAGAGCTGCCTGGATCTGACATTGTGAGAGGAGGTGCCTTC
SRR448690.24936622.1-             AGCCCTCATGGAGAGCTGCCTGGATCTGACATTGTGAGAGGAGGTGCCT
SRR448690.60572380.1-             AGCCCTCATGGAGAGCTGCCTGGATCTGACATTGTGAGAGGAGGTGCCT
SRR448690.64980178.1-             AGCCCTCATGGAGAGCTGCCTGGATCTGACATTGTGAGAGGAGGTGCCT
SRR448690.66342636.1-             AGCCCTCATGGAGAGCTGCCTGGATCTGACATTGTGAGAGGAGGTGCCT
SRR448690.94974465.1-             AGCCCTCATGGAGAGCTGCCTGGATCTGACATTGTGAGAGGAGGTGCCT
SRR448675.92803720.2+             AGCCCTCATGGAGAGCTGCCTGGATCTGACATTGTGAGAGGAGGTGCCTTC
SRR448681.7337942.1-              AGCCCTCATGGAGAGCTGCCTGGATCTGACATTGTGAGAGGAGGTGCCTTC
SRR448687.50111023.1+             AGCCCTCATGGAGAGCTGCCTGGATCTGACATTGTGAGAGGAGGTGCCTTC
SRR448690.58786536.1-              GCCCTCATGGAGAGCTGCCTGCATCTGACATTGTGAGAGGAGGTGCCTT
SRR448690.66186920.1-              GCCCTCATGGAGAGCTGCCTGCATCTGACATTGTGAGAGGAGGTGCCTT
SRR448681.24459844.2+              GCCCTCATGGAGAGCTGCCTGGATCTGACATTGTGAGAGGAGGTGCCTTC
SRR448681.3793565.2-               GCCCTCATGGAGAGCTGCCTGGATCTGACATTGTGAGAGGAGGTGCCTTC
SRR448686.105629760.1-             GCCCTCCTGGAGAGCTGCCTGGATCTGACATTGTGAGAGGAGGTGCCTTC
SRR448681.68030745.2-               CCCTCAGGGAGAGCTGCCTGCATCTGACATTGTGAGAGGAGGTGCCTTC
SRR448683.76951253.2-               CCCTCAGGGACAGCTGCCTGCATCTGACATTGTGAGAGGAGGTGCCTTC
SRR448683.89708988.1-               CCCTCAGGGAGAGCTGCCTGCATCTGACATTGTGAGAGGAGGTGCCTTC
SRR448694.31238796.2-               CCCTCATGGAGAGCTGCCTGGATCTGACATTGTGAGAGGAGGTGCCTTC
SRR448694.69001203.1-               CCCTCATGGAGAGCTGCCTGGATCTGACATTGTGAGAGGAGGTGCCTTC
SRR448686.30719737.1+                  TCATGGAGAGCTGCCTGCATCTGACATTGTGAGAGGAGGTGCCTTC
SRR448681.32854092.2-                    ATGGAGAGCTGCCTGCATCTGACATTGTGAGAGGAGGTGCCTTC
SRR448686.67600101.2+                    ATGGAGAGCTGCCTGGATCTGACATTGTGAGAGGAGGTGCCTTC
SRR448694.30972732.1-                    ATGGAGAGCTGCCTGGATCTGACATTGTGAGAGGAGGTGCCTTC
SRR448694.77446518.1-                    ATGGAGAGCTGCCTGGATCTGACATTGTGAGAGGAGGTGCCTTC
SRR448699.62917460.1+                    ATGGAGAGCTGCCTGCATCTGACATTGTGAGAGGAGGTGCCTTC
SRR448702.18987201.1-                    ATGGAGAGCTGCCTGGATCTGACATTGTGAGAGGAGGTGCCTTC
SRR448702.56960720.1-                    ATGGAGAGCTGCCTGGATCTGACATTGTGAGAGGAGGTGCCTTC
SRR448702.65613870.1-                    ATGGAGAGCTGCCTGGATCTGACATTGTGAGAGGAGGTGCCTTC
SRR448681.85152453.1-                     TGGAGAGTTGCCTGCATCTGACATTGTGAGAGGAGGTGCCTTC
SRR448681.54132602.2-                      GGAGAGCTGCCTGCATCTGACATTGTGAGAGGAGGTGCCTTC
SRR448686.106659351.2-                     GGAGAGCTGCCTGCATCTGACATTGTGAGAGGAGGTGCCTTC
SRR448687.35771328.2-                      GGAGAGCTGCCTGGATCTGACATTGTGAGAGGAGGTGCCTTC
SRR448699.71823992.2+                      GGAGAGCTGCCTGCATCTGACATTGTGAGAGGAGGTGCCTTC
SRR448681.21344736.1-                       GAGAGCTGCCTGGATCTGACATTGTGAGAGGAGGTGCCTTC
SRR448699.2138811.1+                          GAGCTGCCTGCATCTGACATTGTGAGAGGAGGTGCCTTC
SRR448699.31495046.1+                         GAGCTGCCTGCATCTGACATTGTGAGAGGAGGTGCCTTC
SRR448699.59251159.1+                         GAGCTGCCTGCATCTGACATTGTGAGAGGAGGTGCCTTC
SRR448690.55829523.1+                          AGCTGCCTGCATCTGACATTGTGAGAGGAGGTGCCTTC
SRR448690.86020218.2+                          AGCTGCCTGCATCTGACATTGTGAGAGGAGGTGCCTTC
SRR448690.99451614.2+                          AGCTGCCTGCATCTGACATTGTGAGAGGAGGTGCCTTC
SRR448694.16806995.2-                          AGCTGCCTGGATCTGACATTGTGAGAGGAGGTGCCTTC
SRR448694.44498965.2-                          AGCTGCCTGGATCTGACATTGTGAGAGGAGGTGCCTTC
SRR448675.103252359.2-                           CTGCCTGCATCTGACATTGTGAGAGGAGGTGCCTTC
SRR448686.3872877.2-                             CTGCCTGGATCTGACATTGTGAGAGGAGGTGCCTTC
SRR448687.21493552.2-                            CTGCCTGGATCTGACATTGTGAGAGGAGGTGCCTTC
SRR448683.74926472.1+                             TGCCTGGATCTGACATTGTGAGAGGAGGTGCCTTC
SRR448675.55211359.1+                               CCTGGATCTGACATTGTGAGAGGAGGTGCCTTC
SRR448686.54291032.1+                               CCTGCATCTGACATTGTGAGAGGAGGTGCCTTC
SRR448681.63302088.1-                                CTGCATCTGACATTGTGAGAGGAGGTGCCTTC
SRR448687.2419817.2-                                    CATCTGACATTGTGAGAGGAGGTGCCTTC
SRR448681.82661095.2-                                     TCTGACATTGTGAGAGGAGGTGCCTTC
SRR448686.54830197.1-                                     TCTGACATGGTGAGAGGAGGTGCCTTC
SRR448694.1529471.1-                                      TCTGACATTGTGAGAGGAGGTGCCTTC
SRR448694.3705273.1-                                      TCTGACATTGTGAGAGGAGGTGCCTTC
SRR448694.98235980.2-                                      CTGACATTGTGAGAGGAGGTGCCTTC
SRR448675.34322357.1-                                       TGACATTGTGAGAGGAGGTGCCTTC
SRR448681.52262572.1+                                       TGACATTGTGAGAGGAGGTGCCTTC
SRR448681.66489052.1-                                         ACATTGTGAGAGGAGGTGCCTTA
SRR448686.60047141.1-                                         ACATTGTGAGAGGAGGTGCCTTC
SRR448699.53858420.2-                                         ACATTGTGAGAGGAGGTGCCTTC
SRR448687.53588449.2+                                          CATTGTGAGAGGAGGTGCCTTC
SRR448681.73558260.1-                                           ATTGTGAGAGGAGGTGCCTTC
SRR448681.74492780.2+                                           ATTGTGAGAGGAGGTGCCTTC
SRR448681.97626341.2-                                           ATTGTGAGAGGAGGTGCCTTC
SRR448683.38609304.2-                                             TGTGAGAGGAGGTGCCTTC
SRR448675.88926626.2-                                              GTGAGAGGAGGTGCCTTC
```

```
SRR448687.79714711.2-                                               TGAGAGGAGGTGCCTTC
SRR448690.101626234.1-                                              TGAGAGGAGGTGCCTTC
SRR448690.9489931.1-                                                TGAGAGGAGGTGCCTTC
SRR448683.6008353.2+                                                 GAGAGGAGGTGCCTTC
SRR448688.24653805.1-                                                GAGAGGAGGTGCCTTC
SRR448675.29937986.1-                                                 AGAGGAGGTGCCTTC
SRR448694.89586431.1+                                                 AGAGGAGGTGCCTTC
SRR448683.41964796.2-                                                  GAGGAGGTGCCTTC
SRR448687.67472025.1-                                                  GAGGAGGTGCCTTC
SRR448683.85260721.2-                                                   AGGAGGTGCCTTC
SRR448699.75512887.1+                                                   AGGAGGTGCCTTC
SRR448675.100859301.2-                                                   GGAGGTTCCTTC
SRR448686.18126246.1-                                                     GAGGTGCCTTC
SRR448683.103280204.1+                                                      GGTGCCTTC
SRR448688.19366612.2-                                                       GGTGCCTTC
SRR448687.20474580.1-                                                        GTGCCTTC
SRR448694.104960237.1-                                                       GTGCCTTC
SRR448694.43393625.1-                                                        GTGCCTTC
SRR448687.25169379.1-                                                         TGCCTTC
SRR448675.55058720.2+                                                          GCCTTC
SRR448699.11267604.1-                                                           CCTTC
SRR448699.112474019.1+                                                           CTTC
SRR448699.17013684.1+                                                            CTTC
SRR448699.3359303.1+                                                             CTTC
SRR448687.8856975.2-                                                              TTC
SRR448683.26439403.1+                                                              TC
SRR448683.12692921.1-                                                               C
SRR448686.593803.1-                                                                 C
                         ____________________________________________________________
consensus                TGTGGATTCAGCCCTCATGGAGAGCTGCCTGCATCTGACATTGTGAGAGGAGGTGCCTTC

                             .    :    .    :    .    :    .    :    .    :    .    :
SRR448675.59564553.2-    TA
SRR448686.59598963.1-    TA
SRR448681.73558260.2+    TAA
SRR448681.7497961.2-     TAA
SRR448683.56266056.2+    TAA
SRR448683.9537018.2-     TAA
SRR448687.31855042.1+    TAA
SRR448687.76742462.2+    TAAA
SRR448702.65613870.1-    TAAA
SRR448675.92739025.1-    TAAAA
SRR448694.30972732.1-    TAAAA
SRR448694.77446518.1-    TAAAA
SRR448699.62917460.1+    TAAAA
SRR448702.18987201.1-    TAAAA
SRR448702.56960720.1-    TAAAA
SRR448675.103252359.1+   TAAAAT
SRR448683.2954521.2-     TAAAAT
SRR448683.42166054.2-    TAAAAT
SRR448687.51419033.1-    TAAAAT
SRR448699.71823992.2+    TAAAATG
SRR448686.108023284.1-   TAAAATGGCA
SRR448699.2138811.1+     TAAAATGGCA
SRR448699.31495046.1+    TAAAATGGCA
SRR448699.59251159.1+    TAAAATGGCA
SRR448686.106844158.2+   TAAAATGGCAG
SRR448687.14912879.2-    TAAAATGGCAG
SRR448690.55829523.1+    TAAAATGGCAG
SRR448690.86020218.2+    TAAAATGGCAG
SRR448690.99451614.2+    TAAAATGGCAG
SRR448694.16806995.2-    TAAAATGGCAG
SRR448694.44498965.2-    TAAAATGGCAG
SRR448681.97626341.1+    TAAAATGGCAGC
SRR448675.29937986.2+    TAAAATGGCAGCA
SRR448687.74386594.2+    TAAAATGGCAGCA
SRR448683.88276819.2-    TAAAATGGCAGCATC
SRR448683.92212587.1-    TAAAATGGCAGCATC
SRR448675.100859301.1+   TAAAATGGCAGCATCT
SRR448687.65209642.1+    TAAAATGGCAGGATCT
SRR448683.79752369.1-    TAAAATGGCAGCATCTG
SRR448687.23078728.1-    TAAAATGGCAGCATCTG
SRR448687.33039334.1+    TAAAATGGCAGCCTCTG
SRR448686.1930452.1-     TAAAATGGCAGCATCTGCT
SRR448675.88926626.1+    TAAAATGGCAGCATCTGCTT
SRR448687.89718471.2+    TAAAATGGCAGCATCTGCTTC
SRR448694.1529471.1-     TAAAATGGCAGCATCTGCTTCT
SRR448694.3705273.1-     TAAAATGGCAGCATCTGCTTCT
SRR448694.98235980.2-    TAAAATGGCAGCATCTGCTTCTC
SRR448683.67390564.1-    TAAAATGGCAGCATCTGCTTCTCT
SRR448683.72556731.1-    TAAAATGGCAGCATCTGCTTCTCT
SRR448686.105629760.1-   TAAAATGGCAGCATCTGCTTCTCT
SRR448683.69167326.1-    TAAAATGGCAGCATCTGCTTCTCTgt
SRR448699.53858420.2-    TAAAATGGCAGCATCTGCTTCTCTgt
```

```
SRR448675.104128282.2-   TAAAATGGCAGCATCTGCTTCTCTgta
SRR448675.65770019.1+    TAAAATGGCAGCATCTGCTTCTCTgtaagt
SRR448683.15990850.1-    TAAAATGGCAGCATCTGCTTCTCTgtaagttg
SRR448690.101626234.1-   TAAAATGGCAGCATCTGCTTCTCTgtaagttg
SRR448690.9489931.1-     TAAAATGGCAGCATCTGCTTCTCTgtaagttg
SRR448681.50099907.2-    TAAAATGGCAGCATCTGCTTCTCTgtaagttga
SRR448688.24653805.1-    TAAAATGGCAGCATCTGCTTCTCTgtaagttga
SRR448694.89586431.1+    TAAAATGGCAGCATCTGCTTCTCTgtaagttgaa
SRR448699.75512887.1+    TAAAATGGCAGCATCTGCTTCTCTgtaagttgaaca
SRR448686.91310581.1+    TAAAATGGCAGCATCTGCTTCTCTttaagttggacag
SRR448681.39820355.1+    TAAAATGGCAGCATCTGCTTCTCTgtaagttgaacaga
SRR448683.64053908.2+    TAAAATGGCAGCATCTGCTTCTCTgtaagttgaacaga
SRR448687.69057351.2-    TAAAATGGCAGCATCTGCTTCTCTgtaagttgaacaga
SRR448688.19366612.2-    TAAAATGGCAGCATCTGCTTCTCTgtaagttgaacagatt
SRR448699.11267604.1-    TAAAATGGCAGCATCTGCTTCTCTgtaagttgaacagatt
SRR448694.104960237.1-   TAAAATGGCAGCATCTGCTTCTCTgtaagttgaacagattt
SRR448694.43393625.1-    TAAAATGGCAGCATCTGCTTCTCTgtaagttgaacagattt
SRR448686.94018257.1-    TAAAATGGCAGCATCTGCTTCTCTgtaagttgaacagatttcc
SRR448681.85152453.1-    TAAAATGGCAGCATCTGCTTCTCTgtaagttgaacagatttcct
SRR448681.21545235.1-    TAAAATGGCAGCATCTGCTTCTCTgtaagttgaacagatttcctc
SRR448681.4625807.1+     TAAAATGGCAGCATCTGCTTCTCTgtaagttgaacagatttcctc
SRR448699.112474019.1+   TAAAATGGCAGCATCTGCTTCTCTgtaagttgaacagatttcctc
SRR448699.17013684.1+    TAAAATGGCAGCATCTGCTTCTCTgtaagttgaacagatttcctc
SRR448699.3359303.1+     TAAAATGGCAGCATCTGCTTCTCTgtaagttgaacagatttcctc
SRR448681.68030745.2-    TAAAATGGCAGCATCTGCTTCTCTgtaagttgaacagatttcctct
SRR448687.19754537.1-    TAAAATGGCAGCATCTGCTTCTCTgtaagttgaacagatttcctct
SRR448681.32854092.2-    TAAAATGGCAGCATCTGCTTCTCTgtaagttgaacagatttcctcttc
SRR448675.92803720.2+    TAAAATGGCAGCATCTGCTTCTCTgtaagttgaacagatttcctcttca
SRR448681.7337942.1-     TAAAATGGCAGCATCTGCTTCTCTgtaagttgaacagatttcctcttca
SRR448687.50111023.1+    TAAAATGGCAGCATCTGCTTCTCTgtaagttggacagatttcctcttca
SRR448699.19251753.1+    TAAAATGGCAGCATCTGCTTCTCTgtaagttgaacagatttcctcttca
SRR448699.60284909.1+    TAAAATGGCAGCATCTGCTTCTCTgtaagttgaacagatttcctcttca
SRR448699.93854624.1+    TAAAATGGCAGCATCTGCTTCTCTgtaagttgaacagatttcctcttca
SRR448681.24459844.2+    TAAAATGGCAGCATCTGCTTCTCTgtaagttgaacagatttcctcttcac
SRR448681.3793565.2-     TAAAATGGCAGCATCTGCTTCTCTgtaagttgaacagatttcctcttcac
SRR448683.76951253.2-    TAAAATGGCAGCATCTGCTTCTCTgtaagttgaacagatttcctcttcac
SRR448683.89708988.1-    TAAAATGGCAGCATCTGCTTCTCTgtaagttgaacagatttcctcttcacc
SRR448686.30719737.1+    TAAAATGGCAGCATCTGCTTCTCTgtaagttgaacagatttcctcttcaccccc
SRR448686.67600101.2+    TAAAATGGCAGCATCTGCTTCTCTgtaagttgaacagattttctcttcacccccct
SRR448681.54132602.2-    TAAAATGGCAGCATCTGCTTCTCTgtaagttgaacagatttcctcttcacccccccttt
SRR448686.106659351.2-   TAAAATGGCAGCATCTGCTTCTCTgtaagttgaacagatttcctcttcacccccccttt
SRR448686.54291032.1+    TAAAATGGCAGCATCTGCTTCTCTgtaagttgaacagatttcctcttcacccccccttt
SRR448687.35771328.2-    TAAAATGGCAGCATCTGCTTCTCTgtaagttgaacagatttcctcttcacccccccttt
SRR448681.21344736.1-    TAAAATGGCAGCATCTGCTTCTCTgtaagttgaacagatttcctcttcacccccccttttg
SRR448675.100859301.2-   TAAAATGGCAGCATCTGCTTCTCTgtaagttgaacagatttcctcttcaccccccctttgc
SRR448675.103252359.2-   TAAAATGGCAGCATCTGCTTCTCTgtaagttgaacagatttcctcttcaccccccctttgc
SRR448675.27813751.2+    TAAAATGGCAGCATCTGCTTCTCTgtaagttgaacagatttcctcttcaccccccctttgc
SRR448675.29937986.1-    TAAAATGGCAGCATCTGCTTCTCTgtaagttgaacagatttcctcttcaccccccctttgc
SRR448675.34322357.1-    TAAAATGGCAGCATCTGCTTCTCTgtaagttgagcagatttcctcttcaccccccctttgc
SRR448675.55058720.2+    TAAAATGGCAGCATCTGCTTCTCTgtaagttgaacagatttcctcttcaccccccctttgc
SRR448675.55211359.1+    TAAAATGGCAGCATCTGCTTCTCTgtaagttgaacagatttcctcttcaccccccctttgc
SRR448675.88926626.2-    TAAAATGGCAGCATCTGCTTCTCTgtaagttgaacagatttcctcttcaccccccctttgc
SRR448681.52262572.1+    TAAAATGGCAGCATCTGCTTCTCTgtaagttgaacagatttcctcttcaccccccctttgc
SRR448681.63302088.1-    TAAAATGGCAGCATCTGCTTCTCTgtaagttgaacagatttcctcttcaccccccctttgc
SRR448681.66489052.1-    TAAAATGACAGCATCTGCTTCTCTgtaagttgaacagatttcctcttcaccccccctttgc
SRR448681.73558260.1-    TAAAATGGCAGCATCTGCTTCTCTgtaagttgaacagatttcctcttcaccccccctttgc
SRR448681.74492780.2+    TAAAATGGCAGCATCTGCTTCTCTgtaagttgaacagatttcctcttcaccccccctttgc
SRR448681.82661095.2-    TAAAATGGCAGCATCTGCTTCTCTgtaagttgaacagatttcctcttcaccccccctttgc
SRR448681.97626341.2-    TAAAATGGCAGCATCTGCTTCTCTgtaagttgaacagatttcctcttcaccccccctttgc
SRR448683.103280204.1+   TAAAATGGCAGCATCTGCTTCTCTggaagtttaacagatttcctcttcaccccccctttgc
SRR448683.12692921.1-    TAAAATGGCAGCATCTGCTTCTCTgtaagttgaacagatttcctcttcaccccccctttgc
SRR448683.26439403.1+    TAAAATGGCAGCATCTGCTTCTCTgtaagttgaacagatttcctcttcaccccccctttgc
SRR448683.38609304.2-    TAAAATGGCAGCATCTGCTTCTCTgtaagttgaacagatttcctcttcaccccccctttgc
SRR448683.41964796.2-    TAAAATGGCAGCATCTGCTTCTCTgtaagttgaacagatttcctcttcaccccccctttgc
SRR448683.6008353.2+     TAAAATGGCAGCATCTGCTTCTCTgtaagttgaacagatttcctcttcacccccccttttc
SRR448683.74926472.1+    TAAAATGGCAGCATCTGCTTTTCTgtaagttgaacagatttcctcttcacaccccctttgc
SRR448683.85260721.2-    TAAAATGCCAGCATCTGCTTCTCTgtaagttgaacagatttcctcttcaccccccctttgc
SRR448686.18126246.1-    TAAAATGGCAGCATCTGCTTCTCTgtaagttgaacagatttcctcttcaccccccctttgc
SRR448686.3872877.2-     TAAAATGGCAGCATCTGCTTCTCTgtaagttggacagatttcctcttcaccccccctttgc
SRR448686.54830197.1-    TAAAATGGCAGCATCTGCTTCTCTgtaagttgaacagatttcctcttcaccccccctttgc
SRR448686.593803.1-      TAAAATGGCAGCATCTGCTTCTCTgtaagttgaacagatttcctcttcaccccccctttgc
SRR448686.60047141.1-    TAAAATGGCAGCATCTGCTTCTCTgtaagttgaacagatttcctcttcaccccccctttgc
SRR448687.20474580.1-    TAAAATGGCAGCATCTGCTTCTCTgtaagttgaacagatttcctcttcaccccccctttgc
SRR448687.21493552.2-    TAAAATGGCAGCATCTGCTTCTCTgtaagttgaacagatttcctcttcaccccccctttgc
SRR448687.2419817.2-     TAAAATGGCAGCATCTGCTTCTCTgtaagttgaacagatttcctcttcaccccccctttgc
SRR448687.25169379.1-    TAAAATGGCAGCATCTGCTTCTCTgtaagttgaacagatttcctcttcaccccccctttgc
SRR448687.53588449.2+    TAAAATGGCAGCATCTGCTTCTCTgtaagttgaacagatttactcttcaccccccctttgc
SRR448687.67472025.1-    TAAAATGGCAGCATCTGCTTCTCTgtaagttgaacagatttcctcttcaccccccctttgc
SRR448687.79714711.2-    TAAAATGGCAGCATCTGCTTCTCTgtaagttgaacagatttcctcttcaccccccctttgc
SRR448687.8856975.2-     TAAAATGGCAGCATCTGCTTCTCTgtaagttgaacagatttcctctacacagccctttgc
SRR448675.65770019.2-     AAAATGGCAGCATCTGCTTCTCTgtaagttgaacagatttcctcttcaccccccctttgc
SRR448687.96591513.1+     AAAATGGCAGCATCTGCTTCTCTgtaagttgaacagatttcctcttcaccccccctttgc
SRR448675.83276572.2+      AAATGGCAGCATCTGCTTCTCTgtaagttgaacagatttcctcttcaccccccctttgc
SRR448683.5269535.2-       AAATGGCAGCATCTGCTTCTCTgtaagttgaacagatttcctcttcaccccccctttgc
```

```
SRR448681.48531411.2+       AATGGCAGCATCTGCTTCTCTgtaagttgaacagatttcctcttcacccccctttgc
SRR448688.43502939.1-        ATGGCAGCATCTGCTTCTCTgtaagttgaacagatttcctcttcacccc
SRR448686.11759748.1-        ATGGCAGCATCTGCTTCTCTgtaagttgaacagatttcctcttcacccccctttgc
SRR448686.77036164.2+        ATGGCAGCATCTGCTTCTCTgtaagttgaacagatttcctcttcacccccctttgc
SRR448675.107261507.1+        TGGCAGCATCTGCTTCTCTgtaagttgaacagatttcctcttcacccccctttgc
SRR448686.51959512.2-         TGGCAGCATCTGCTTCTCTgtaagttgaacagatttcctcttcacccccctttgc
SRR448686.112999163.1-         GGCAGCATCTGCTTCTCTgtaagttgaacagatttcctcttcacccccctttgc
SRR448687.28327880.2-          GGCAGCATCTGCTTCTCTgtaagttgaacagatttcctcttcacccccctttgc
SRR448686.68556123.2+           GCAGCATCTGCTTCTCTgtaagttgaacagatttcctcttcacccccctttgc
SRR448694.39287712.2-            CAGCATCTGCTTCTCTgtaagttgaacagatttcctcttcacccccctt
SRR448694.64893080.2-            CAGCATCTGCTTCTCTgtaagttgaacagatttcctcttcacccccctt
SRR448702.24699021.2-            CAGCATCTGCTTCTCTgtaagttgaacagatttcctcttcacccccctt
SRR448702.26760819.2-            CAGCATCTGCTTCTCTgtaagttgaacagatttcctcttcacccccctt
SRR448702.27931894.2-            CAGCATCTGCTTCTCTgtaagttgaacagatttcctcttcacccccctt
SRR448702.29785359.2-            CAGCATCTGCTTCTCTgtaagttgaacagatttcctcttcacccccctt
SRR448702.61954721.2-            CAGCATCTGCTTCTCTgtaagttgaacagatttcctcttcacccccctt
SRR448702.71414955.2-            CAGCATCTGCTTCTCTgtaagttgaacagatttcctcttcacccccctt
SRR448702.72279098.2-            CAGCATCTGCTTCTCTgtaagttgaacagatttcctcttcacccccctt
SRR448702.74637775.2-            CAGCATCTGCTTCTCTgtaagttgaacagatttcctcttcacccccctt
SRR448702.81793783.2-            CAGCATCTGCTTCTCTgtaagttgaacagatttcctcttcacccccctt
SRR448702.9104901.2-             CAGCATCTGCTTCTCTgtaagttgaacagatttcctcttcacccccctt
SRR448702.9955743.2-             CAGCATCTGCTTCTCTgtaagttgaacagatttcctcttcacccccctt
SRR448690.22501808.2+             AGCATCTGCTTCTCTgtaagttgaacagatttcctcttcaccccccttt
SRR448690.36155819.2+             AGCATCTGCTTCTCTgtaagttgaacagatttcctcttcaccccccttt
SRR448690.57282333.2+             AGCATCTGCTTCTCTgtaagttgaacagatttcctcttcaccccccttt
SRR448690.83673025.2+             AGCATCTGCTTCTCTgtaagttgaacagatttcctcttcaccccccttt
SRR448699.56150436.1+             AGCATCTGCTTCTCTgtaagttgaacagatttcctcttcaccccccttt
SRR448686.91959985.2-             AGCATCTGCTTCTCTgtaagttgaacagatttcctcttcacccccctttgc
SRR448687.95495483.2-              GCATCTGCTTCTCTgtaagttgaacagatttcctcttcacccccctttgc
SRR448683.90592100.2-               CATCTGCTTCTCTgtaagtttaacagatttccccttcacccccctttgg
SRR448687.93736509.1-               CATCTGCTTCTCTgtaagttgaacagatttcctcttcacccccctttgc
SRR448702.22005023.2+               CATCTGCTTCTCTgtaagttgaacagatttcctcttcacccccctttgc
SRR448702.57417831.2+               CATCTGCTTCTCTgtaagttgaacagatttcctcttcacccccctttgc
SRR448702.71283349.2+               CATCTGCTTCTCTgtaagttgaacagatttcctcttcacccccctttgc
SRR448702.71354704.2+               CATCTGCTTCTCTgtaagttgaacagatttcctcttcacccccctttgc
SRR448702.75737206.2+               CATCTGCTTCTCTgtaagttgaacagatttcctcttcacccccctttgc
SRR448702.79322973.2+               CATCTGCTTCTCTgtaagttgaacagatttcctcttcacccccctttgc
SRR448702.96383647.2+               CATCTGCTTCTCTgtaagttgaacagatttcctcttcacccccctttgc
SRR448681.4625807.2-                  TCTGCTTCTCTgtaagttgaacagatttcctcttcacccccctttgc
SRR448683.87004555.2+                  CTGCTTCTCTgtaagttgaacagatttcctcttcacccccctttgc
SRR448687.6190006.2-                   CTGCTTCTCTgtaagttgaacagatttcctcttcacccccctttgc
SRR448688.43220813.1+                   TGCTTCTCTgtaagttgaacagatttcctcttcacccccctttgc
SRR448688.52106493.1+                   TGCTTCTCTgtaagttgaacagatttcctcttcacccccctttgc
SRR448688.62588768.1+                   TGCTTCTCTgtaagttgaacagatttcctcttcacccccctttgc
SRR448688.990692.1+                     TGCTTCTCTgtaagttgaacagatttcctcttcacccccctttgc
SRR448681.24459844.1-                    GCTTCTCTgtaagttgaacagatttcctcttcacccccctttgc
SRR448683.76750984.2+                    GCTTCTCTgtaagttgaacagatttcctcttcacccccctttgc
SRR448690.80864321.2-                    GCTTCTCTgtaagttgaacagatttcctcttcacccccctttgc
SRR448675.81395938.2-                     CTTCTCTgtaagttgaacagatttcctcttcacccccctttgc
SRR448688.39740332.2+                     CTTCTCTgtaagttgaacagatttcctcttcacccccctttgc
SRR448675.92803720.1-                      TTCTCTgtaagttgaacagatttcctcttcacccccctttgc
SRR448681.39820355.2-                      TTCTCTgtaagttgaacagatttcctcttcacccccctttgc
SRR448683.2563223.1+                       TTCTCTgtaagttgaacagatttcctcttcacccccctttgc
SRR448686.40703985.2-                      TTCTCTgtaagttgaacagatttcctcttcacccccctttgc
SRR448690.46976751.1+                       TCTCTgtaagttgaacagatttcctcttcacccccctttgc
SRR448675.50850970.2+                        CTCTgtaagttgaacagatttcctcttcacccccctttgc
SRR448694.45712566.1-                         TCTgtaagttgaacagatttcctcttcacccccctttgc
SRR448675.65170588.2+                          CTgtaagttgaacagatttcctcttcacccccctttgc

consensus                TAAAATGGCAGCATCTGCTTCTCTgtaagttgaacagatttcctcttcacccccctttgc
```

H. Rock pigeon (No. 47), exon 1, WGS data

- CAP3 alignment of WGS data spanning exon 1 (uppercase letters)

```
                                   .    :    .    :    .    :    .    :    .    :    .    :
SRR511912.10904274.2+    attggcaagactccgaaattgcagtggtcctggtgataA
SRR516981.37304474.2+    attggcaagactccgaaattgcagtggtcctggtgataA
SRR516996.43960638.2+    attggcaagactccgaaattgcagtggtcctggtgataA
SRR517006.10231357.2+    attggcaagactccgaaattgcagtggtcctggtgataA
SRR517006.16071194.2+    attggcaagactccgaaattgcagtggtcctggtgataA
SRR516967.148303398.1+   attggcaagactccgaaattgcagtggtcctggtgataAT
SRR516989.56316629.1+    attggcaagactccgaaattgcagtgatcctggtgataAT
SRR516999.97066835.2+    attggcaagactccgaaattgcagtggtcctggtgataAT
SRR516967.125704511.2+   attggcaagactccgaaattgcagtggtcctggtgataATG
SRR516995.40185498.2-    attggcaagactccgaaattgcagtggtcctggtgataATG
SRR516996.45030954.2-    attggcaagactccgaaattgcagtggtcctggtgataATG
SRR516997.75919883.2-    atcggcaagactccgaaatcgcagtggtcctggtgataATG
SRR511911.13083061.2-    attggcaagactccgaaattgcagtggtcctggtgataATGG
SRR511915.55090700.1+    attggcaagactccgaaattgcagtggtcctggtgataATGG
SRR511917.19484665.2+    attggcaagactccgaaattgcagtggtcctggtgataATGG
SRR516968.171429418.1-   attggcaagactccgcaattgcagtggtcctggtgataATGG
SRR516973.26502918.1+    attggcaagactccgaaattgcagtggtcctggtgataATGG
SRR516990.26399022.1+    attggcaagactccgaaattgcagtggtcctggtgataATGG
SRR516998.3146409.2+     attggcaagactccgaaattgcagtggtcctggtgataATGG
SRR517000.20199727.1+    attggcaagactccgaaattgcagtggtcctggtgataATGG
SRR511915.76881432.1+    attggcaagactccgaaattgcagtggtcctggtgataATGGC
SRR511917.13212508.1+    attggcaagactccgaaattgcagtggtcctggtgataATGGC
SRR516973.13923651.1+    atcggcaagactccgaaatcgcagtggtcatggtgataATGGC
SRR516981.56307628.2+    attggcaagactccgaaattgcagtggtcctggtgataATGGC
SRR516999.66646424.1-    attggcaagactccgaaattgcagtggtcctggtgataATGGC
SRR517003.52529179.1-    attggcaagactccgaaattgcagtggtcctggtgataATGGC
SRR516967.121468181.2+   attggcaagactccgaaattgcagtggtcctggtgataATGGCA
SRR516967.5677042.2+     attggcaagactccgaaattgcagtggtcctggggataATGGCA
SRR516972.14209986.1+    attggcaagactccgaaattgcagtggtcctggtgataATGGCA
SRR511913.18817834.2-    attggcaagactccgaaattgcagtggtcctggtgataATGGCAG
SRR516975.17854041.1+    atcggcaagactccgaaatcgcagtggtcctggtgataATGGCAG
SRR516986.4527098.1-     attggcaagactccgaaattgcagtggtcctggtgataATGGCAG
SRR517002.35534784.2-    attggcaagactccgaaattgcagtggtcctggtgataATGGCAG
SRR511913.32159204.2-    attggcaagactccgaaattgcagtggtcctggtgataATGGCAGT
SRR511913.60163149.2-    attggcaagactccgaaattgcagtggtcctggtgataATGGCAGT
SRR511916.41456611.2+    attggcaagactccgaaattgcagtggtcctggtgataATGGCAGG
SRR511916.870334.1+      attggcaagactccgaaattgcagtggtcctggtgataATGGCAGT
SRR511917.59277348.2+    attggcaagactccgaaattgcagtggtcctggtgataATGGCAGG
SRR511917.73764240.1+    attggcaagactccgaaattgcagtggtcctggtgataATGGCAGT
SRR511917.74847508.2+    attggcaagactccgaaattgcagtggtcctggtgataATGGCAGG
SRR516991.12327481.1-    atcggcaagactccgaaatcgcagtggtcctggtgataATGGCAGT
SRR511913.7237934.2-     attggcaagactccgaaattgcagtggtcctggtgataATGGCAGTT
SRR511914.85920285.1+    attggcaagactccgaaattgcagtggtcctggtgataATGGCAGGT
SRR516983.601653.1-      attggcaagactccgaaattgcagtggtcctggtgataATGGCAGTT
SRR516989.69959453.2-    attggcaagactccgaaattgcagtggtcctggtgataATGGCAGTT
SRR516993.12254620.1-    attggcaagactccgaaattgcagtggtcctggtgataATGGCAGTT
SRR516995.30697500.2+    attggcaagactccgaaattgcagtggtcctggtgataATGGCAGTT
SRR517003.68088813.1+    attggcaagactccgaaattgcagtggtcctggtgataATGGCAGGT
SRR511912.6039191.2-     attggcaagactccgaaattgcagtggtcctggtgataATGGCAGTTC
SRR511915.71616142.2-    attggcaagactccgaaattgcagtggtcctggtgataATGGCAGTTC
SRR516987.71247483.1+    atcggcaagactccgaaatcgcagtggtcctggtgataATGGCAGGTT
SRR517006.75902656.1+    attggcaagactccgaaattgcagtggtcctggtgataATGGCAGGTC
SRR517007.10616984.1+    attggcaagactccgaaattgcagtggtcctggtgataATGGCAGGTT
SRR511892.27528962.2+    attggcaagactccgaaattgcagtggtcctggtgataATGGCAGGTTT
SRR516981.26225422.2-    attggcaagactccgaaattgcagtggtcctggtgataATGGCAGTTCT
SRR516981.56501324.1-    attggcaagactccgaaattgcagtggtcctggtgataATGGCAGTTCT
SRR516990.13617951.1-    attggcaagactccgaaattgcagtggtcctggtgataATGGCAGTTCT
SRR516995.63783405.1+    attggcaagactccgaaattgcagtggtcctggtgataATGGCAGGTCT
SRR516997.26908206.1-    attggcaagactccgaaattgcagtggtcctggtgataATGGCAGTTCT
SRR516997.92109894.2-    atcggcaagactccgaaatcgcagtggtcctggtgataATGGCAGTTCT
SRR516975.22235529.1-    attggcaagactccgaaattgcagtggtcctggtgataATGGCAGTTCTT
SRR516982.63238094.1-    atcggcaagactccgaaatcgcagtggtcctggtgataATGGCAGTTCTT
SRR516990.2434805.2-     attggcaagactccgaaattgcagtggtcctggtgataATGGCAGTTCTT
SRR516990.48830689.2-    attggcaatactccgaaattgcagtggtcctggtgataATGGCAGTTCTT
SRR516968.60181167.2-    attggcaagactccgaaattgcagtggtcctggtgataATGGCAGTTCTTT
SRR516990.39880558.2-    attggcaagactccgaaattgcagtggtcctggtgataATGGCAGTTCTTT
SRR516996.76886931.2+    attggcaagactccgaaattgcagtggtcctggtgataATGGCAGGTCTTTT
SRR517007.12538010.1-    attggcaagactccgaaattgcagtggtcctggtgataATGGCAGTTCTTTT
SRR517007.58137463.2-    attggcaagactccgaaattgcagtggtcctggtgataATGGCAGTTCTTTT
SRR511911.69454746.2-    attggcaagactccgaaattgcagtggtcctggtgataATGGCAGTTCTTTTC
SRR516986.269274.2-      attggcaagactccgaaattgcagtggtcctggtgataATGGCAGTTCTTTTC
SRR517003.81748180.2-    atcggcaagactccgaaattgcagtggtcctggtgataATGGCAGTTCTTTTC
SRR516978.51401599.2-    atcggcaagactccgaaattgcagtggtcctggtgataATGGCAGTTCTTTTCT
SRR516984.72157789.1+    atcggcaagactccgaaattgcagtggtcctggtgataATGGCAGGTGTTTTCT
SRR516998.97862365.2+    attggcaagactccgaaattgcagtggtcctggtgataATGGCAGTTTTTTTTT
```

```
SRR517000.108297177.2-   atcggcaagactccgaaattgcagtggtcctggtgataATGGCAGTTCTTTTCT
SRR517000.76398852.2+    attggcaagactccgaaattgcagtggtcctggtgataATGGCAGGTCTTTTCT
SRR517004.35366889.1-    atcggcaagactccgaaattgcagtggtcctggtgataATGGCAGTTCTTTTCT
SRR516968.109083542.2+   attggcaagactccgaaattgcagtggtcctggtgataATGGCAGTTCTTTTCTC
SRR516981.17610135.2-    attggcaagactccgaaattgcagtggtcctggtgataATGGCAGTTCTTTTCTC
SRR516993.43367430.1+    atcggcaagactccgaaattgcagtggtcctggtgataATGGCAGGTCTTTTCTC
SRR517008.2647950.2+     atcggcaagactccgaaattgcagtggtcctggtgataATGGCAGTTATTTTCTC
SRR516967.149002341.1-   attggcaggactccgaaatcgcagtggtcctggtgataATTGCAGTTCTTTTCTCA
SRR516967.63778390.1+    attngcaagactccgaaattgcagtggtcctggtgataATGGCAGTTCTTTTCTCA
SRR516977.47084677.2+    atcggcaagactccgaaattgcagtggtcctggtgataATGGCAGGTCTTTTCTCA
SRR516979.50136254.2+    atcggcaagactccgaaatcgcagtggtcctggtgataATGGCAGCTCTTTTTTCA
SRR516989.38263129.1-    attggcaagactccgaaattgcagtggtcctggtgataATGGCAGTTCTTTTCTCA
SRR511915.64407574.2-    attggcaagactccgaaattgcagtggtcctggtgataATGGCAGTTCTTTTCTCAA
SRR511915.64407713.2-    attggcaagactccgaaattgcagtggtcctggtgataATGGCAGTTCTTTTCTCAA
SRR516994.45722230.2+    atcggcaagactccgaaattgcagtggtcctggtgataATGGCAGTTCTTTTCTCAA
SRR511911.51563603.2-    attggcaagactccgaaattgcagtggtcctggtgataATGGCAGTTCTTTTCTCAAG
SRR511913.74486978.2-    attggcaagactccgaaattgcagtggtcctggtgataATGGCAGTTCTTTTCTCAAG
SRR511915.39596443.1+    attggcaagactccgaaattgcagtggtcctggtgataATGGCAGGTCTTTTCTCAAA
SRR516967.59477310.2+    attggcaagactccgaaattgcagtggtcctggtgataATGGCAGTTCTTTTCTCAAG
SRR516973.3345899.1+     attggcaagactccgaaattgcagtggtcctggtgataATGGCAGGTCTTTTCTCAAG
SRR516975.11381701.2+    attggcaagactccgaaattgcagtggtcctggtgataATGGCAGTTCTTTTCTCAAG
SRR516976.25681997.1+    attggcaagactccgaaattgcagtggtcctggtgataATGGCAGGTCTTTTCTCAAG
SRR516991.24958347.1+    atcggcaagactccgaaatcgcagtggtcctggtgataATGGCAGGTCTTTTCTCAAG
SRR516991.63054458.1+    atcggcaagactccgaaatcgcagtggtcctggtgataATGGCAGTTCTTTTCTCAAG
SRR516993.7655946.1+     atcggcaagactccgaaattgcagtggtcctggtgataATGGCAGGTCTTTTCTCAAG
SRR517001.63430876.2+    atcggcaagactccgaaattgcagtggtcctggtgataATGGCAGGTCTTTTCTCAAG
SRR517002.29006876.1+    attggcaagactccgaaattgcagtggtcctggtgataATGGCAGGTCTTTTCTCAAG
SRR517006.16479440.1+    attggcaagactccgaaattgcagtggtcctggtgataATGGCAGGTCTTTTCTCAAG
SRR516968.106610860.2+   attggcaagactccgaaattgcagtggtcctggtgataATGGCAGTTCTTTTCTCAAGT
SRR516981.52610643.1+    attggcaagactccgaaattgcagtggtcctggtgataATGGCAGTTCTTTTCTCAAGT
SRR516994.13908675.1-    atcggcaagactccgaaattgcagtggtcctggtgataATGGCAGTTCTTTTCTCAAGT
SRR516994.19939914.1-    atcggcaagactccgaaattgcagtggtcctggtgataATGGCAGTTCTTTTCTCAAGT
SRR516999.53729530.2-    attggcaagactccgaaattgcagtggtcctggtgataATGGCAGTTCTTTTCTCAAGT
SRR517000.24850193.1-    atcggcaagactccgaaattgcagtggtcctggtgataATGGCAGTTCTTTTCTCAAGT
SRR511892.31593589.1-    attggcaagactccgaaattgcagtggtcctggtgataATGGCAGTTCTTTTCTCAAGTA
SRR511892.45941781.1+    attggcaagactccgaaattgcagtggtcctggtgataATGGCAGGTCTTTTCTCAAGTA
SRR511892.55624723.1-    attggcaagactccgaaattgcagtggtcctggtgataATGGCAGTTCTTTTCTCAAGTA
SRR511892.68477751.1-    attggcaagactccgaaattgcagtggtcctggtgataATGGCAGTTCTTTTCTCAAGTA
SRR511892.9740145.1-     attggcaagactccgaaattgcagtggtcctggtgataATGGCAGTTCTTTTCTCAAGTA
SRR511892.9740146.1-     attggcaagactccgaaattgcagtggtcctggtgataATGGCAGTTCTTTTCTCAAGTA
SRR511911.17781748.1-    attggcaagactccgaaattgcagtggtcctggtgataATGGCAGTTCTTTTCTCAAGTA
SRR511911.37480006.2-    attggcaagactccgaaattgcagtggtcctggtgataATGGCAGTTCTTTTCTCAAGTA
SRR511911.40076074.2-    attggcaagactccgaaattgcagtggtcctggtgataATGGCAGTTCTTTTCTCAAGTA
SRR511911.45670450.2-    attggcaagactccgaaattgcagtggtcctggtgataATGGCAGTTCTTTTCTCAAGTA
SRR511911.61131441.1+    attggcaagactccgaaattgcagtggtcctggtgataATGGCAGTTTTTTTTCCAGGA
SRR511911.71659560.2+    attggcaagactccgaaattgcagtggtcctggtgataATGGCAGTTCTTTTCTCAAGTA
SRR511912.1014472.1-     attggcaagactccgaaattgcagtggtcctggtgataATGGCAGTTCTTTTCTCAAGTA
SRR511912.14258493.1-    attggcaagactccgaaattgcagtggtcctggtgataATGGCAGTTCTTTTCTCAAGTA
SRR511912.14258649.1-    attggcaagactccgaaattgcagtggtcctggtgataATGGCAGTTCTTTTCTCAAGTA
SRR511912.19294174.1+    attggcaagactccgaaattgcagtggtcctggtgataATGGCAGGTCTTTTCTCAAGCA
SRR511912.19294330.1+    attggcaagactccgaaattgcagtggtcctggtgataATGGCAGTTCTTTTCTCAAGTA
SRR511912.20631234.2-    attggcaagactccgaaattgcagtggtcctggtgataATGGCAGTTCTTTTCTCAAGTA
SRR511912.4156394.1-     attggcaagactccgaaattgcagtggtcctggtgataATGGCAGTTCTTTTCTCAAGTA
SRR511912.72370488.2+    attggcaagactccgaaattgcagtggtcctggtgataATGGCAGTTCTTTTCTCAAGTA
SRR511913.1169864.1+     attggcaagactccgaaattgcagtggtcctggtgataATGGCAGGTCTTTTCTCCAGGA
SRR511913.18852311.1-    attggcaagactccgaaattgcagtggtcctggtgataATGGCAGTTCTTTTCTCAAGTA
SRR511913.20668125.2-    attggcaagactccgaaattgcagtggtcctggtgataATGGCAGTTCTTTTCTCAAGTA
SRR511913.51810263.1-    attggcaagactccgaaattgcagtggtcctggtgataATGGCAGTTCTTTTCTCAAGTA
SRR511913.52001769.2-    attggcaagactccgaaattgcagtggtcctggtgataATGGCAGTTCTTTTCTCAAGTA
SRR511913.8247697.2+     attggcaagactccgaaattgcagtggtcctggtgataATGGCAGGTTTTTTCTCCAGTA
SRR511914.65840186.2-    attggcaagactccgaaattgcagtggtcctggtgataATGGCAGTTCTTTTCTCAAGTA
SRR511914.68007108.2-    attggcaagactccgaaattgcagtggtcctggtgataATGGCAGTTCTTTTCTCAAGTA
SRR511915.2956436.1-     attggcaagactccgaaattgcagtggtcctggtgataATGGCAGTTCTTTTCTCAAGTA
SRR511915.2956742.1-     attggcaagactccgaaattgcagtggtcctggtgataATGGCAGTTCTTTTCTCAAGTA
SRR511915.46810937.2+    attggcaagactccgaaattgcagtggtcctggtgataATGGCAGGTCTTTTCTCCAGTA
SRR511915.67432792.1+    attggcaagactccgaaattgcagtggtcctggtgataATGGCAGTTCTTTTCTCAAGTA
SRR516967.146282791.1-   attggcaagactccgaaattgcagtggtcctggtgataATGGCAGTTCTTTTCTCAAGTA
SRR516967.14759904.2+    attggcaagactccgaaattgcagtggtcctggtgataATGGCAGTTCTTTTCTCAAGTA
SRR516967.54383831.1-    attggcaagactccgaaattgcagtggtcctggtgataATGGCAGTTCTTTTCTCAAGTA
SRR516967.6221170.2+     attggcaagactccgaaattgcagtggtcctggtgataATGGCAGTTCTTTTCTCAAGTA
SRR516967.94416127.2+    attggcatgactccgaaattgcagtggtcctggtgataATGGCAGTTCTTTTCTCAAGTA
SRR516968.105088970.1-   attggcaagactccgaaattgcagtggtcctggtgataATGGCAGTTCTTTTCTCAAGTA
SRR516968.115657864.2-   attggcaagactccgaaattgcagtggtcctggtgataATGGCAGTTCTTTTCTCAAGTA
SRR516968.118272353.2+   atcggcaagactccgaaattgcagtggtcctggtgataATGGCAGTTCTTTTCTCAAGTA
SRR516968.140796502.2+   attggcaagactccgaaattgcagtggtcctggtgataATGGCAGTTCTTTTCTCAAGTA
SRR516968.145184287.1+   attggcaagactccgaaattgcagtggtcctggtgataATGGCAGTTCTTTTCTCAAGTA
SRR516968.15347757.1+    atcggcaagactccgaaattgcagtggtcctggtgataATGGCAGTTCTTTTCTCAAGTA
SRR516968.156018396.2+   attggcaagactccgaaattgcagtggtcctggtgataATGGCAGTTCTTTTCTCAAGTA
SRR516968.32902413.1-    atcggcaagactccgaaattgcagtggtcctggtgataATGGCAGTTCTTTTCTCAAGTA
SRR516968.43850003.1+    atcggcaagactccgaaattgcagtggtcctggtgataATGGCAGTTCTTTTCTCAAGTA
SRR516968.6323756.1-     attggcaagactccgaaattgcagtggtcctggtgataATGGCAGTTCTTTTCTCAAGTA
SRR516968.98150509.1-    atcggcaagactccgaaattgcagtggtcctggtgataATGGCAGTTCTTTTCTCAAGTA
SRR516970.7640225.2+     atcggcaagactccgaaattgcagtggtcctggtgataATGGCAGTTCTTTTCTCAAGTA
SRR516971.1110705.2+     atcggcaagactccgaaattgcagtggtcctggtgataATGGCAGTTCTTTTCTCAAGTA
```

```
SRR516971.15887332.2-    atcggcaagactccgaaattgcagtggtcctggtgataATGGCAGTTCTTTTCTCAAGTA
SRR516971.24445351.1+    atcggcaagactccgaaattgcagcggtcctggtgataATGGCAGTTCTTTTCTCAAGTA
SRR516971.5736893.1-     atcggcaagactccgaaattgcagtggtcctggtgataATGGCAGTTCTTTTCTCAAGTA
SRR516972.51498046.1-    attggcaagactccgaaattgcagtggtcctggtgataATGGCAGTTCTTTTCTCAAGTA
SRR516972.54921884.2+    attggcaagactccgaaattgcagtggtcctggtgataATGGCAGTTCTTTTCTCTAGTA
SRR516972.58439524.1-    attggcaagactccgaaattgcagtggtcctggtgataATGGCAGTTCTTTTCTCAAGTA
SRR516972.67844320.1-    attggcaagactccaaaattgcagtggtcctggtgataATGGCAGTTCTTTTCTCAAGTA
SRR516973.16283767.2+    attggcaagactccgaaattgcagtggtcctggtgataATGGCAGTTCTTTTCTCAAGTA
SRR516973.16890128.2+    atcggcaagactccgaaatcgcagtggtcctggtgataATGGCAGGTCTTTTCTCCAGTA
SRR516973.17477597.1-    attggcaagactccgaaattgcagtggtcctggtgataATGGCAGTTCTTTTCTCAAGTA
SRR516973.19946759.2-    atcggcaagactccgaaatcgcagtggtcctggtgataATGGCAGTTCTTTTCTCAAGTA
SRR516973.50577545.1+    attggcaagactccgaaattgcagtggtcctggtgataATGGCAGTTTTTTTCTCAAGCA
SRR516973.52994151.1-    attggcaagactccgaaattgcagtggtcctggtgataATGGCAGTTCTTTTCTCAAGTA
SRR516974.36849866.2-    attggcaagactccgaaattgcagtggtcctggtgataATGGCAGTTCTTTTCTCAAGTA
SRR516974.42555202.2+    attggcaagactccgaaattgcagtggtcctggtgataATGGCAGTTCTTTTCTCAAGTA
SRR516974.47709711.1+    attggcaagactccgaaattgcagtggtcctggtgataATGGCAGTTCTTTTCTCAAGTA
SRR516974.65206690.1-    attggcaagactccgaaattgcagtggtcctggtgataATGGCAGTTCTTTTCTCAAGTA
SRR516974.65598927.2+    attggcaagactccgaaattgcagtggtcctggtgataATGGCAGTTCTTTTCTCAAGTA
SRR516974.72485008.1-    attggcaagactccgaaattgcagtggtcctggtgataATGGCAGTTCTTTTCTCAAGTA
SRR516975.14702766.2-    atcggcaagactccgaaatcgcagtggtcctggtgataATGGCAGTTCTTTTCTCAAGTA
SRR516975.27931365.1-    attggcaagactccgaaattgcagtggtcctggtgataATGGCAGTTCTTTTCTCAAGTA
SRR516975.9441702.2-     attggcaagactccgaaattgcagtggtcctggtgataATGGCAGTTCTTTTCTCAAGTA
SRR516976.12707927.1+    atcggcaagactccgaaatcgcagtggtcctggtgataATGGCAGTTCTTTTCTCAAGTA
SRR516976.24305739.2+    attggcaagactccgaaattgcagtggtcctggtgataATGGCAGGTCTTTTCTCAAGTA
SRR516976.5159916.1+     attggcaagactccgaaattgcagtggtcctggtgataATGGCAGGTCTTTTCTCAAGTA
SRR516976.8894843.2-     attggcaagactccgaaattgcagtggtcctggtgataATGGCAGTTCTTTTCTCAAGTA
SRR516977.1612623.2-     atcggcaagactccgaaattgcagtggtcctggtgataATGGCAGTTCTTTTCTCAAGTA
SRR516977.42905173.2-    atcggcaagactccgaaattgcagtggtcctggtgataATGGCAGTTCTTTTCTCAAGTA
SRR516977.50923694.1-    atcggcaagactccgaaattgcagtggtcctggtgataATGGCAGTTCTTTTCTCAAGTA
SRR516977.63657923.1+    atcggcaagactccgaaattgcagtggtcctggtgataATGGCAGTTCTTTTCTCAAGTA
SRR516977.73763692.1-    atcggcaagactccgaaattgcagtggtcctggtgataATGGCAGTTCTTTTCTCAAGTA
SRR516978.20535847.2-    atcggcaacactccgaaattgcagtggtcctggtgataATGGCAGTTCTTTTCTCAAGTA
SRR516978.4630760.1+     atcggcaagactccgaaattgcagtggtcctggtgataATGGCAGGTCTTTTCTCAAGTA
SRR516978.48507581.2+    atcggcaagactccgaaattgcagtggtcctggtgataATGGCAGTTCTTTTCTCAAGTA
SRR516978.57147863.2-    atcggcaagactccgaaattgcagtggtcctggtgataATGGCAGTTCTTTTCTCAAGTA
SRR516978.75075983.1-    atcggcaagactccgaaattgcagtggtcctggtgataATGGCAGTTCTTTTCTCAAGTA
SRR516978.9119600.2+     atcggcaagactccgaaattgcagtggtcctggtgataATGGCAGTTCTTTTCTCAAGTA
SRR516979.11039952.1-    atcggcaagactccgaaattgcagtggtcctggtgataATGGCAGTTCTTTTCTCAAGTA
SRR516979.16080749.1+    atcggcaagactccgaaatcgcagtggtcctggtgataATGGCAGTTCTTTTCTCAAGTA
SRR516979.43816476.2+    atcggcaagactccgaaattgcagtggtcctggtgataATGGCAGGTCTTTTCTCAAGTA
SRR516979.45053249.1-    atcggcaagactccgaaattgcagtggtcctggtgataATGGCAGTTCTTTTCTCAAGTA
SRR516979.5438653.1+     atcggcaagactccgaaattgcagtggtcctggtgataATGGCAGTTCTTTTCTCAAGTA
SRR516980.12879649.1-    atcggcaagactccgaaattgcagtggtcctggtgataATGGCAGTTCTTTTCTCAAGTA
SRR516980.57095254.1+    atcggcaagactccgaaattgcagtggtcctggtgataATGGCAGGTCTTTTCTCCAGTA
SRR516980.7236138.2-     atcggcaagactccgaaattgcagtggtcctggtgataATGGCAGTTCTTTTCTCAAGTA
SRR516980.8816673.2-     atcggcaagactccgaaattgcagtggtcctggtgataATGGCAGTTCTTTTCTCAAGTA
SRR516981.23321590.2-    attggcaagactccgaaattgcagtggtcctggtgataATGGCAGTTCTTTTCTCAAGTA
SRR516981.26964373.2+    attggcaagactccgaaattgcagtggtcctggtgataATGGCAGTTCTTTTCTCAAGTA
SRR516981.35251407.1-    attggcaagactccgaaattgcagtggtcctggtgataATGGCAGTTCTTTTCTCAAGTA
SRR516981.35677677.2-    attggcaagactccgaaattgcagtggtcctcgtgataATGGCAGTTCTTTTCTCAAGTA
SRR516981.76497254.2-    attggcaagactcctaaattgcagtggtcctggtgataATGGCAGTTCTTTTCTCAAGTA
SRR516981.85604642.1-    attggcaagactccgaaattgcagtggtcctggtgataATGGCAGTTCTTTTCTCAAGTA
SRR516982.31349844.1-    atcggcaagactccgaaatcgcagtggtcctggtgataATGGCAGTTCTTTTCTCAAGTA
SRR516982.50768215.2-    atcggcaagactccgaaatcgcagtggtcctggtgataATGGCAGTTCTTTTCTCAAGTA
SRR516982.55151074.2+    atcggcaagactccgaaatcgcagtggtcctggtgataATGGCAGTTCTTTTCTCCAGGA
SRR516983.19283942.1+    attggcaagactccgaaattgcagtggtcctggtgataATGGCAGTTCTTTTCTCAAGAA
SRR516983.72008022.1-    attggcaagactccgaaattgcagtggtcctggtgataATGGCAGTTCTTTTCTCAAGTA
SRR516984.11685539.1+    atcggcaagactccgaaattgcagtggtcctggtgataATGGCAGGTCTTTTCTCAAGTA
SRR516984.23515395.1+    attggcaagactccgaaattgcagtggtcctggtgataATGGCAGTTCTTTTCTCAAGTA
SRR516984.64702111.2-    attggcaagactccgaaattgcagtggtcctggtgataATGGCAGTTCTTTTCTCAAGTA
SRR516985.219617.1+      atcggcaagactccgaaattgcagtggtcctggtgataATGGCAGTTCTTTTCTCAAGTA
SRR516985.35211582.2-    atcggcaagactccgaaattgcagtggtcctggtgataATGGCAGTTCTTTTCTCAAGTA
SRR516985.49435852.1-    atcggcaagactccgaaattgcagtggtcctggtgataATGGCAGTTCTTTTCTCAAGTA
SRR516985.9120641.2+     atcggcaagactccgaaattgcagtggtcctggtgataATGGCAGTTCTTTTCTCCAGTA
SRR516986.14794457.1+    attggcaagactccgaaattgcagtggtcctggtgataATGGCAGTTCTTTTTTCAAGTA
SRR516986.15967055.2-    attggcaagactccgaaattgcagtggtcctggtgataATGGCAGTTCTTTTCTCAAGTA
SRR516986.21413362.2+    atcggcaagactccgaaattgcagtggtcctggtgataATGGCAGTTCTTTTCTCAAGTA
SRR516986.24103450.2-    atcggcaagactccgaaattgcagtggtcctggtgataATGGCAGTTCTTTTCTCAAGTA
SRR516986.37049200.1-    attggcaagactccgaaattgcagtggtcctggtgataATGGCAGTTCTTTTCTCAAGTA
SRR516986.44931411.2+    atcggcaagactccgaaattgcagtggtcctggtgataATGGCAGGTCTTTTCTCAAGTA
SRR516987.79339821.1-    atcggcaagactccgaaatcgcagtggtcctggtgataATGGCAGTTCTTTTCTCAAGTA
SRR516988.3500447.1-     atcggcaagactccgaaattgcagtggtcctggtgataATGGCAGTTCTTTTCTCAAGTA
SRR516988.42192760.1-    atcggcaagactccgaaattgcagtggtcctggtgataATGGCAGTTCTTTTCTCAAGTA
SRR516989.22778442.2+    attggcaagactccgaaattgcagtggtcctggtgataATGGCAGTTCTTTTCTCAAGTA
SRR516989.32990384.1-    attggcaagactccgaaattgcagtggtcctggtgataATGGCAGTTCTTTTCTCAAGTA
SRR516989.44612348.1-    attggcaagactccgaaattgcagtggtcctggtgataATGGCAGTTCTTTTCTCAAGTA
SRR516989.50454159.2-    attggcaagactccgaaattgcagtggtcctggtgataATGGCAGTTCTTTTCTCAAGTA
SRR516989.69704079.1+    attggcaagactccgaaattgcagtggtcctggtgataATGGCAGGTTCTTTCTCAAGGA
SRR516989.75191770.1+    attggcaagactccgaaattgcagtggtcctggtgataATGGCAGGTCTTTTCTCAAGTA
SRR516989.8213552.2-     attggcaagactccgaaattgcagtggtcctggtgataATGGCAGTTCTTTTCTCAAGTA
SRR516990.15863859.2+    attggcaagactccgaaattgcagtggtcctggtgataATGGCAGTTCTTTTCTCAAGTA
SRR516990.20604046.1-    attggcaagactccgaaattgcagtggtcctggtgataATGGCAGTTCTTTTCTCAAGTA
SRR516990.40763061.2+    attggcaagactccgaaattgcagtggtcctggtgataATGGCAGTTCTTTTCTCCAGTA
SRR516991.3697767.1+     attggcaagactccgaaattgcagtggtcctggtgataATGGCAGTTCTTTTCTCAAGTA
```

```
SRR516991.70672748.1-    atcggcaagactccgaaatcgcagtggtcctggtgataATGGCAGTTCTTTTCTCAAGTA
SRR516991.89333198.1+    attggcaagactccgaaattgcagtggtcctggtgataATGGCAGTTCTTTTCTCAAGTA
SRR516993.33273789.1-    atcggcaagactccgaaattgcagtggtcctggtgataATGGCAGTTCTTTTCTCAAGTA
SRR516993.77596703.1+    attggcaagactccgaaattgcagtggtcctggtgataATGGCAGTTCTTTTCTCAAGTA
SRR516993.84060022.1-    attggcaagactccgaaattgcagtggtcctggtgataATGGCAGTTCTTTTCTCAAGTA
SRR516994.27805045.1-    atcggcaagactccgaaattgcagtggtcctggtgataATGGCAGTTCTTTTCTCAAGTA
SRR516995.17755192.1+    atcggcaagactccgaaattgcagtggtcctggtgataATGGCAGGTCTTTTCTCCAGTA
SRR516995.41614722.2-    atcggcaagactccgaaattgcagtggtcctggtgataATGGCAGTTCTTTTCTCAAGTA
SRR516995.43843685.1-    attggcaagactccgaaattgcagtggtcctggtgataATGGCAGTTCTTTTCTCAAGTA
SRR516995.83414144.2-    atcggcaagactccgaaattgcagtggtcctggtgataATGGCAGTTCTTTTCTCAAGTA
SRR516995.87128240.2-    atcggcaagactccgaaattgcagtggtcctggtgataATGGCAGTTCTTTTCTCAAGTA
SRR516996.31113264.1-    attggcaagactccgaaattgcagtggtcctggtgataATGGCAGTTCTTTTCTCAAGTA
SRR516996.33042242.1-    attggcaagactccgaaattgcagtggtcctggtgataATGGCAGTTCTTTTCTCAAGTA
SRR516996.52401398.1+    attggcaagactccgaaattgcagtggtcctggtgataATGGCAGTTCTTTTCTCAAGTA
SRR516996.66223613.1+    attggcaagactccgaaattgcagtggtcctggtgataATGGCAGTTCTTTTCTCAAGGA
SRR516996.76179188.2+    attggcaagactccgaaattgcagtggtcctggtgataATGGCAGTTCTTTTCTCAAGTA
SRR516997.4512656.1-     attggcaagactccgaaattgcagtggtcctggtgataATGGCAGTTCTTTTCTCAAGTA
SRR516997.55886851.2-    atcggcaagactccgaaatcgcagtggtcctggtgataATGGCAGTTCTTTTCTCAAGTA
SRR516997.58614765.1+    atcggcaagactccgaaatcgcagtggtcctggtgataATGGCAGGTCTTTTCTCCAGTA
SRR516997.63356024.2-    attggcaagactccgaaattgcagtggtcctggtgataATGGCAGTTCTTTTCTCAAGTA
SRR516997.90536592.1+    atcggcaagactccgaaatcgcagtggtcctggtgataATGGCAGGTCTTTTCTCAAGTA
SRR516998.100156656.1+   attggcaagactccgaaattgcagtggtcctggtgataATGGCAGGTCTTTTCTCAAGTA
SRR516998.105122847.1-   atcggcaagactccgaaattgcagtggtcctggtgataATGGCAGTTCTTTTCTCAAGTA
SRR516998.28200586.2-    attggcaagactccgaaattgcagtggtcctggtgataATGGCAGTTCTTTTCTCAAGTA
SRR516998.29687080.2-    atcggcaagactccgaaattgcagtggtcctggtgataATGGCAGTTCTTTTCTCAAGTA
SRR516998.53967676.2-    attggcaagactccgaaattgcagtggtcctggtgataATGGCAGTTCTTTTCTCAAGTA
SRR516998.54079381.1-    attggcaagactccgaaattgcagtggtcctggtgataATGGCAGTTCTTTTCTCAAGTA
SRR516998.88264383.1+    attggcaagactccgaaattgcagtggtcctggtgataATGGCAGGTCTTTTCTCAAGTA
SRR516999.100313301.1+   attggcaagactccgaaattgcagtggtcctggtgataATGGCAGTTCTTTTTTCAAATA
SRR516999.3393409.2-     attggcaagactccgaaattgcagtggtcctggtgataATGGCAGTTCTTTTCTCAAGTA
SRR516999.39456345.2-    attggcaagactccgaaattgcagtggtcctggtgataATGGCAGTTCTTTTCTCAAGTA
SRR516999.44920014.1+    attggcaagactccgaaattgcagtggtcctggtgataATGGCAGGTCTTTTCTCAAGTA
SRR516999.4718727.1+     attggcaagactccgaaattgcagtggtcctggtgataATGGCAGGTCTTTTCTCAAGTA
SRR516999.5917.1+        attggcaagactccgaaattgcagtggtcctggtgataATGGCAGTTCTTTTCTCAAGAA
SRR516999.78625944.1+    attggcaagactccgaaattgcagtggtcctggtgataATGGCAGTTCTTTTCTCAAGTA
SRR516999.92446757.1-    attggcaagactccgaaattgcagtggtcctggtgataATGGCAGTTCTTTTCTCAAGTA
SRR516999.93433337.2-    attggcaagactccgaaattgcagtggtcctggtgataATGGCAGTTCTTTTCTCAAGTA
SRR517000.36678948.1+    attggcaagactccgaaattgcagtggtcctggtgataATGGCAGTTCTTTTCTCAAGTA
SRR517000.54991829.2+    atcggcaagactccgaaattgcagtggtcctggtgataATGGCAGTTCTTTTCTCAAGTA
SRR517000.63898591.1-    atcggcaagactccgaaattgcagtggtcctggtgataATGGCAGTTCTTTTCTCAAGTA
SRR517001.63940723.2+    atcggcaagactccgaaattgcagtggtcctggtgataATGGCAGTTCTTTTCTCAAGTA
SRR517001.87930230.1-    atcggcaagactccgaaattgcagtggtcctggtgataATGGCAGTTCTTTTCTCAAGTA
SRR517002.15955724.1-    attggcaagactccgaaattgcagtggtcctggtgataATGGCAGTTCTTTTCTCAAGTA
SRR517002.31507094.2-    attggcaagactccgaaattgcagtggtcctggtgataATGGCAGTTCTTTTCTCAAGTA
SRR517002.32972405.2-    attggcaagactccgaaattgcagtggtcctggtgataATGGCAGTTCTTTTCTCAAGTA
SRR517002.54888386.2-    attggcaagactccgaaattgcagtggtcctggtgataATGGCAGTTCTTTTCTCAAGTA
SRR517002.90607223.1+    attggcaagactccgaaattgcagtggtcctggtgataATGGCAGTTCTTTTTTCAAGTA
SRR517002.97801972.1-    attggcaagactccgaaattgcagtggtcctggtgataATGGCAGTTCTTTTCTCAAGTA
SRR517003.12649627.2-    attggcaagactccgaaattgcagtggtcctggtgataATGGCAGTTCTTTTCTCAAGTA
SRR517003.21132339.1-    attggcaagactccgaaattgcagtggtcctggtgataATGGCAGTTCTTTTCTCAAGTA
SRR517003.33620522.1-    atcggcaagactccgaaattgcagtggtcctggtgataATGGCAGTTCTTTTCTCAAGTA
SRR517003.39098715.2-    atcggcaagactccgaaattgcagtggtcctggtgataATGGCAGTTCTTTTCTCAAGTA
SRR517004.23920483.1+    atcggcaagactccgaaattgcagtggtcctggtgataATGGCAGTTCTTTTCTCAAGTA
SRR517004.41059246.1+    atcggcaagactccgaaattgcagtggtcctggtgataATGGCAGGTCTTTTCTCAAGTA
SRR517004.42185234.1-    atcggcaagactccgaaattgcagtggtcctggtgataATGGCAGTTCTTTTCTCAAGTA
SRR517004.51768700.2-    atcggcaagactccgaaattgcagtggtcctggtgataATGGCAGTTCTTTTCTCAAGTA
SRR517005.61532069.2-    atcggcaagactccgaaattgcagtggtcctggtgataATGGCAGTTCTTTTCTCAAGTA
SRR517005.73305592.2-    atcggcaagactccgaaattgcagtggtcctggtgataATGGCAGTTCTTTTCTCAAGTA
SRR517005.7777984.1+     atcggcaagactccgaaattgcagtggtcctggtgataATGGCAGGTCTTTTCTCAAGGA
SRR517006.47673006.2-    attggcaagactccgaaattgcagtggtcctggtgataATGGCAGTTCTTTTCTCAAGTA
SRR517006.55093488.2-    attggcaagactccgaaattgcagtggtcctggtgataATGGCAGTTCTTTTCTCAAGTA
SRR517006.5583744.1-     attggcaagactccgaaattgcagtggtcctggtgataATGGCAGTTCTTTTCTCAAGTA
SRR517007.13507481.1+    attggcaagactccgaaattgcagtggtcctggtgataATGGCAGGTCTTTTCTCAAGGA
SRR517007.35305430.2-    attggcaagactccgaaattgcagtggtcctggtgataATGGCAGTTCTTTTCTCAAGTA
SRR517007.68739089.1+    attggcaagactccgaaattgcagtggtcctggtgataATGGCAGTTCTTTTCTCCAGTA
SRR517007.7845014.1-     attggcaagactccgaaattgcagtggtcctggtgataATGGCAGTTCTTTTCTCAAGTA
SRR517007.8592877.1-     attggcaagactccgaaattgcagtggtcctggtgataATGGCAGTTCTTTTCTCAAGTA
SRR517008.41558359.1+    atcggcaagactccgaaattgcagtggtcctggtgataATGGCAGGTCTTTTCTCAAGTA
SRR517008.63631025.2-    atcggcaagactccgaaattgcagtggtcctggtgataATGGCAGTTCTTTTCTCAAGTA
SRR517008.75782648.1-    atcggcaagactccgaaattgcagtggtcctggtgataATGGCAGTTCTTTTCTCAAGTA
SRR517009.19677786.1-    attggcaagactccgaaattgcagtggtcctggtgataATGGCAGTTCTTTTCTCAAGTA
SRR517009.22120276.1+    attggcaagactccgaaattgcagtggtcctggtgataATGGCAGTTCTTTTCTCAAGTA
SRR517009.26922977.2+    atcggcaagactccgaaattgcagtggtcctggtgataATGGCAGGTCTTTTCTCAAGTA
SRR517009.26982378.2+    attggcaagactccgaaattgcagtggtcctggtgataATGGCAGGTCTTTTCTCAAGTA
SRR517009.73111776.1-    attggcaagactccgaaattgcagtggtcctggtgataATGGCAGTTCTTTTCTCAAGTA
SRR517009.7493725.1-     attggcaagactccgaaattgcagtggtcctggtgataATGGCAGTTCTTTTCTCAAGTA
SRR517009.85179959.2-    attggcaagactccgaaattgcagtggtcctggtgataATGGCAGTTCTTTTCTCAAGTA
SRR511917.12998789.2+     ttggcaagactccgaaattgcagtggtcctggtgataATGGCAGGT
SRR511917.12998927.2+     ttggcaagactccgaaattgcagtggtcctggtgataATGGCAGGT
SRR511917.51708370.2+     ttggcaagactccgaaattgcagtggtcctggtgataATGGCAGGT
SRR511911.29686714.2-     ttggcaagactccgaaattgcagtggtcctggtgataATGGCGGTTCTTTTCTCAAGTA
SRR517000.66457751.1+     ttggcaagactccgaaattgcagtggtcctggtgataATGGCAGTTCTTTTCTCAAGTA
SRR517002.10945551.1-     ttggcaagactccgaaattgcagtggtcctggtgataATGGCAGTTCTTTTCTCAAGTA
SRR516975.4624241.1+       tggcaagactccgaaattgcagtggtcctggtgataATGGCAGTTCTTTTCTCAAGTA
```

```
SRR516987.76366948.2-     tggcaagactccgaaattgcagtggtcctggtgataATGGCAGTTCTTTTCTCAAGTA
SRR516990.64951029.2+     tggcaagactccgaaattgcagtggtcctggtgataATGGCAGTTCTTTTCTCAAGTA
SRR516993.11025707.2-     tggcaagactccgaaattgcagtggtcctggtgataATGGCAGTTCTTTTCTCAAGTA
SRR516998.78835527.1+     tggcaagactccgaaattgcagtggtcctggtgataATGGCAGTTCTTTTCTCAAGTA
SRR516998.83658111.2+     tggcaagactccgaaattgcagtggtcctggtgataATGGCAGTTCTTTTCTCAAGTA
SRR517000.39640223.2+     tggcaagactccgaaattgcagtggtcctggtgataATGGCAGTTCTTTTCTCAAGGA
SRR517002.44210664.1-     tggcaagactccgaaattgcagtggtcctggtgataATGGCAGTTCTTTTCTCAAGTA
SRR511914.19951607.1-      ggcaagactccgaaattgcagtggtcctggtgataATGGCAGTTCTTTTCTCAAGTA
SRR516967.129799720.1-     ggcaagactccgaaattgcagtggtcctggtgataATGGCAGTTCTTTTCTCAAGTA
SRR516974.46565740.2-      ggcaagactccgaaattgcagtggtcctggtgataATGGCAGTTCTTTTCTCAAGTA
SRR516978.52604776.1-      ggcaagactccgaaattgcagtggtcctggtgataATGGCAGTTCTTTTCTCAAGTA
SRR517002.50187769.1+      ggcaagactccgaaattgcagtggtcctggtgataATGGCGGGTCTTTTTTCCAGTA
SRR511916.54430845.1-       gcaagactccgaaattgcagtggtcctggtgataATGGCAGTTCTTTTC
SRR511917.35012429.2-       gcaagactccgaaattgcagtggtcctggtgataATGGCAGTTCTTTTC
SRR516969.9631131.1+        gcaagactccgaaattgcagtggtcctggtgataATGGCAGTTCTTTTCTCAAGTA
SRR516978.38239451.2-       gcaagactccgaaattgcagtggtcctggtgataATGGCAGTTCTTTTCTCAAGTA
SRR516991.87080495.1-       gcaagactccgaaatcgcagtggtcctggtgataATGGCAGTTCTTTTCTCAAGTA
SRR516999.14760583.2-       gcaagactccgaaattgcagtggtcctggtgataATGGCAGTTCTTTTCTCAAGTA
SRR517008.76003299.2-       gcaagactccgaaattgcagtggtcctggtgataATGGCAGTTCTTTTCTCAAGTA
SRR516967.102120548.1-       caagactccgaaattgcagtggtcctggtgataATGGCAGTTCTTTTCTCAAGTA
SRR516967.59801249.2-        caagactccgaaattgcagtggtcctggtgataATGGCAGTTCTTTTCTCAAGTA
SRR516973.60429677.2+        caagactccgaaatcgcagtggtcctggtgataATGGCAGTTCTTTTCTCAAGTA
SRR516978.14537581.1+        caagactccgaaattgcagtggtcctggtgataATGGCAGGTCTTTTCTCAAGTA
SRR516980.14193627.1+        caagactccgaaattgcagtggtcctggtgataATGGCAGTTCTTTTCTCAAGTA
SRR516985.4837674.2+         caagactccgaaattgcagtggtcctggtgataATGGCAGTTCTTTTCTCAAGTA
SRR516987.56665405.2+        caagactccgaaattgcagtggtcctggtgataATGGCAGTTCTTTTCTCAAGTA
SRR516993.66881456.2+        caagactccgaaattgcagtggtcctggtgataATGGCAGTTCTTTTCTCAAGTA
SRR517000.5336928.1-         caagactccgaaattgcagtggtcctggtgataATGGCAGTTCTTTTCTCAAGTA
SRR511892.53930910.2+         aagactccgaaatagcagtggtcctggtgataATGGCAGTTCTTTTCTCAAGTA
SRR511911.45879133.1+         aagactccgaaattgcagtggtcctggtgataATGGCAGTTCTTTTCTCAAGTA
SRR516983.47760221.1-         aagactccgaaattgcagtggtcctggtgataATGGCAGTTCTTTTCTCAAGTA
SRR516992.6818043.1-          aagactccgaaattgcagtggtcctggtgataATGGCAGTTCTTTTCTCAAGTA
SRR516994.75464207.2+         aagactccgaaattgcagtggtcctggtgataATGGCAGTTCTTTTCTCAAGTA
SRR516996.89309997.1+         aagactccgaaattgcagtggtcctggtgataATGGCAGTTCTTTTCTCAAGTA
SRR516974.1040705.1+           agactccgaaattgcagtggtcctggtgataATGGCAGTTCTTTTCTCTAGTA
SRR516979.60567804.1+          agactccgaaatcgcagtggtcctggtgataATGGCAGTTCTTTTCTCAAGTA
SRR516990.13785933.1-          agactccgaaattgcagtggtcctggtgataATGGCAGTTCTTTTCTCAAGTA
SRR516990.60703481.2-          agactccgaaattgcagtggtcctggtgataATGGCAGTTCTTTTCTCAAGTA
SRR517008.30257719.1+          agactccgaaattgcagtggtcctggtgataATGGCAGTTCTTTTCTCAAGTA
SRR511892.22460703.1+           gactccgaaattgcagtggtcctggtgataATGGCAGTTCTTTTCTCAAGTA
SRR516967.118206337.1-          gactccgaaattgcagtggtcctggtgataATGGCAGTTCTTTTCTCAAGTA
SRR516977.47216005.2+           gactccgaaattgcagtggtcctggtgataATGGCAGTTCTTTTCTCAAGTA
SRR516984.23605175.2+           gactccgaaattgcagtggtcctggtgataATGGCAGTTCTTTTCTCAAGTA
SRR517006.41660503.1-           gactccgaaattgcagtggtcctggtgataATGGCAGTTCTTTTCTCAAGTA
SRR516972.58990870.1-            actccgaaattgcagtggtcctggtgataATGGCAGTTCTTTTCTCAAGTA
SRR516975.45005523.1-            actccgaaattgcagtggtcctggtgataATGGCAGTTCTTTTCTCAAGTA
SRR516977.70276963.1+            actccgaaattgcagtggtcctggtgataATGGCAGTTCTTTTCTCAAGTA
SRR517004.33967467.1+            actccgaaattgcagtggtcctggtgataATGGCAGTTCTTTTCTCAAGTA
SRR517007.68737803.1-            actccgaaattgcagtggtcctggtgataATGGCAGTTCTTTTCTCAAGTA
SRR511917.30057537.2+             ctccgaaattgcagtggtcctggtgataATGGCAGGTCTTTTCTCAAGT
SRR516967.132651451.2+            ctccgaaattgcagtggtcctggtgataATGGCAGTTCTTTTCTCAAGTA
SRR516967.94039320.2+             ctccgaaattgcagtggtcctggtgataATGGCAGTTCTTTTCTCAAGTA
SRR516973.18821850.2-             ctccgaaattgcagtggtcctggtgataATGGCAGTTCTTTTCTCAAGTA
SRR516973.18821853.2-             ctccgaaattgcagtggtcctggtgataATGGCAGTTCTTTTCTCAAGTA
SRR516973.42617646.2-             ctccgaaattgcagtggtcctggtgataATGGCAGTTCTTTTCTCAAGTA
SRR516974.6201811.2+              ctccgaaattgcagtggtcctggtgataATGGCAGTTCTTTTCTCAAGTA
SRR516999.97278751.1+             ctccgaaattgcagtggtcctggtgataATGGCAGTTCTTTTCTCAAGTA
SRR511912.61545448.1+              tccgaaattgcagtggtcctggtgataATGGCAGTTCTTTTCTCAAGTA
SRR511912.74095538.1-              tccgaaattgcagtggtcctggtgataATGGCAGTTCTTTTCTCAAGTA
SRR517008.5489452.2-               tccgaaattgcagtggtcctggtgataATGGCAGTTCTTTTCTCAAGTA
SRR516967.149674807.1+              ccgaaattgcagtggtcctggtgataATGGCATTTCTTTTCTCAAGTA
SRR516969.22222210.2-               ccgaaattgcagtggtcctggtgataATGGCAGTTCTTTTCTCAAGTA
SRR516972.57623491.1-               ccgaaattgcagtggtcctggtgataATGGCAGTTCTTTTCTCAAGTA
SRR516975.29785312.1-               ccgaaattgcagtggtcctggtgataATGGCAGTTCTTTTCTCAAGTA
SRR516976.70234108.2-               ccgaaagtgcagtggtcctggtgataATGGCAGTTCTTTTCTCAAGTA
SRR516981.43595596.1-               ccgaaattgcagtggtcctggtgataATGGCAGTTCTTTTCTCAAGTA
SRR516983.13010793.1-               ccgaaattgcagtggtcctggtgataATGGCAGTTCTTTTCTCAAGTA
SRR516987.1841637.2-                ccgaaattgcagtggtcctggtgataATGGCAGTTCTTTTCTCAAGTA
SRR516989.66572423.2+               ccgaaattgcagtggtcctggtgataATGGCAGTTCTTTTCTCAAGTA
SRR516999.1552100.2-                ccgaaattgcagtggtcctggtgataATGGCAGTTCTTTTCTCAAGTA
SRR517005.67585549.2-               ccgaaattgcagtggtcctggtgataATGGCAGTTCTTTTCTCAAGTA
SRR516975.41855794.2+                cgaaatcgcagtggtcctggtgataATGGCAGTTCTTTTCTCAAGTA
SRR516986.39849151.2-                cgaaattgcagtggtcctggtgataATGGCAGTTCTTTTCTCAAGTA
SRR517000.91150091.2+                cgaaattgcagtggtcctggtgataATGGCAGTTCTTTTTTCAAGTA
SRR517004.53237476.2+                cgaaattgcagtggtcctggtgataATGGCAGTTCTTTTCTCAAGTA
SRR517005.54024085.1-                cgaaattgcagtggtcctggtgataATGGCAGTTCTTTTCTCAAGTA
SRR516968.40141403.2+                 gaaattgcagtggtcctggtgataATGGCAGTTCTTTTCTCAAGTA
SRR516970.30184580.1-                 gaaattgcagtggtcctggtgataATGGCAGTTCTTTTCTCAAGTA
SRR516984.75040283.2-                 gaaattgcagtggtcctggtgataATGGCAGTTCTTTTCTCAAGTA
SRR516990.22073434.1-                 gaaattgcagtggtcctggtgataATGGCAGTTCTTTTCTCAAGTA
SRR516995.43463521.1-                 gaaattgcagtggtcctggtgataATGGCAGTTCTTTTCTCAAGTA
SRR516997.21480163.2-                 gaaattgcagtggtcctggtgataATGGCAGTTCTTTTCTCAAGTA
SRR516997.47194944.1-                 gaaattgcagtggtcctggtgataATGGCAGTTCTTTTCTCAAGTA
SRR517006.12195593.1+                 gaaattgcagtggtcctggtgataATGGCAGTTCTTTTCTCAAGTA
```

```
SRR517009.52663891.2+      gaaattgcagtggtcctggtgataATGGCAGTTCTTTTCTCAAGTA
SRR511913.45378464.2+       aaattgcagtggtcctggtgataATGGCAGTTCTTTTCTCAAGTA
SRR516975.25984190.1-       aaattgcagtggtcctggtgataATGGCAGTTCTTTTCTCAAGTA
SRR516983.70623253.2-       aaattgcagtggtcctggtgataATGGCAGTTCATTTCTCAAGTA
SRR516989.30730922.2+       aaattgcagtggtcctggtgataATGGCAGTTCTTTTCTCAAGTA
SRR517003.23333068.2-       aaattgcagtggtcctggtgataATGGCAGTTCTTTTCTCAAGTA
SRR517006.80972378.1-       aaattgcagtggtcctggtgataATGGCAGTTCTTTTCTCAAGTA
SRR511911.73201164.1-        aattgcagtggtcctggtgataATGGCAGTTCTTTTCTCAAGTA
SRR516968.21564094.1+        aattgcagtggtcctggtgataATGGCAGTTCTTTTCTCAAGTA
SRR516969.13918852.2+        aattgcagtggtcctggtgataATGGCAGTTCTTTTCTCAAGTA
SRR516971.6332637.2+         aattgcagtggtcctggtgataATGGCAGTTCTTTTCTCAAGTA
SRR516974.33490101.2+        aattgcagtggtcctggtgataATGGCAGGTCTTTTCTCAAGTA
SRR516977.1264704.2-         aattgcagtggtcctggtgataATGGCAGTTCTTTTCTCAAGTA
SRR516985.95116448.1-        aattgcagtggtcctggtgataATGGCAGTTCTTTTCTCAAGTA
SRR516991.27367214.1+        aattgcagtggtcctggtgataATGGCAGTTCTTTTCTCAAGTA
SRR516998.88169944.2+        aattgcagtggtcctggtgataATGGCAGTTCTTTTCTCAAGTA
SRR516999.94992807.1+        aattgcagtggtcctggtgataATGGCAGTTCTTTTCTCAAGGA
SRR516967.138148601.2+        attgcagtggtcctggtgataATGGCAGTTCTTTTCTCAAGTA
SRR517001.52537783.2+         attgcagtggtcctggtgataATGGCAGTTCCTTTCTCAAGTA
SRR516967.121342033.1-         ttgcagtggtcctggtgataATGGCAGTTCTTTTCTCAAGTA
SRR516972.87344728.1+          ttgcagtggtcctggtgataATGGCAGTTCTTTTCTCAAGTA
SRR511911.13404865.2+           tgcagtggtcctggtgataATGGCAGTTCTTTTCTCAAGTA
SRR516969.34784658.2-           tgcagtggtcctggtgataATGGCAGTTCTTTTCTCAAGTA
SRR516980.39425242.2-           tgcagtggtcctggtgataATGGCAGTTCTTTTCTCAAGTA
SRR516991.44566949.1+           cgcagtggtcctggtgataATGGCAGTTCTTTTCTCAAGTA
SRR516999.92033228.2-           tgcagtggtcctggtgataATGGCAGTTCTTTTCTCAAGTA
SRR517003.11858841.1+           tgcagtggtcctggtgataATGGCAGTTCTTTTCTCAAGTA
SRR517008.88421506.1+           tgcagtggtcctggtgataATGGCAGTTCTTTTCTCAAGTA
SRR511914.75736196.2+            gcagtggtcctggtgataATGGCAGTTCTTTTCTCAAGTA
SRR516991.36445672.2+            gcagtggtcctggtgataATGGCAGTTCTTTTCTCAAGTA
SRR517009.56817958.1-            gcagtggtcctggtgataATGGCAGTTCTTTTCTCAAGTA
SRR511914.17741304.2+             cagtggtcctggtgataATGGCAGGTCTTTTCTCAAGTA
SRR511916.23406501.2+             cagtggtcctggtgataATGGCAGGTCTTTTCTCAAGTA
SRR516967.22260303.1+             cagtggtcctggtgataATGGCAGTTCTTTTCTCAAGTA
SRR516967.30937619.1+             cagtggtcctggtgataATGGCAGTTCTTTTCTCAAGTA
SRR516986.15638143.1-             cagtggtcctggtgataATGGCAGTTCTTTTCTCAAGTA
SRR516994.22414233.2+             cagtggtcctggtgataATGGCAGTTCTTTTCTCAAGTA
SRR517001.66298626.2+             cagtggtcctggtgataATGGCAGTTCTTTTCTCAAGTA
SRR517008.59060241.2-             cagtggtcctggtgataATGGCAGTTCTTTTCTCAAGTA
SRR511915.10990174.2-              agtggtcctggggataATGGCAGTTCTGTTCTCAAGTA
SRR516967.128996446.2+             agtggtcctggtgataATGGCAGTTCTTTTCTCAAGTA
SRR516968.75435688.2+              agtggtcctggtgataATGGCAGTTCTTTTCTCAAGTA
SRR516971.7392543.1+               agtggtcctggtgataATGGCAGTTCTTTTCTCAAGTA
SRR516974.47716003.1+              agtggtcctggtgataATGGCAGTTCTTTTCTCAAGTA
SRR516975.13576344.2-              agtggtcctggtgataATGGCAGTTCTTTTCTCAAGTA
SRR516984.84534820.2+              agtggtcctggtgataATGGCAGTTCTTTTCTCAAGTA
SRR516994.76783997.1-              agtggtcctggtgataATGGCAGTTCTTTTCTCAAGTA
SRR517005.62626634.1+              agtggtcctggtgataATGGCAGTTCTTTTCTCAAGTA
SRR517006.45290877.1+              agtggtcctggtgataATGGCAGTTCTTTTCTCAAGTA
SRR511892.27528962.1-               gtggtcctggtgataATGGCAGTTCTTTTCTCAAGTA
SRR516968.155018007.1-              gtggtcctggtgataATGGCAGTTCTTTTCTCAAGTA
SRR516968.53031658.1+               gtggtcctggtgataATGGCAGTTCTTTTCTCAAGTA
SRR516969.1737591.2+                gtggtcctggtgataATGGCAGTTCTTTTCTCAAGTA
SRR516982.4572095.1-                gtggtcctggtgataATGGCAGTTCTTTTCTCAAGTA
SRR516985.59438916.2-               gtggtcctggtgataATGGCAGTTCTTTTCTCAAGTA
SRR516988.29683805.2-               gtggtcctggtgataATGGCAGTTCTTTTCTCAAGTA
SRR516991.4931934.2-                gtggtcctggtgataATGGCAGTTCTTTTCTCAAGTA
SRR516998.32153278.1-               gtggtcctggtgataATGGCAGTTCTTTTCTCAAGTA
SRR516998.43339497.2-               gtggtcctggtgataATGGCAGTTCTTTTCTCAAGTA
SRR517006.89664623.2-               gtggtcctggtgataATGGCAGTTCTTTTCTCAAGTA
SRR516976.39031867.1+                tggtcctggtgataATGGCAGTTCTTTTCTCAAGTA
SRR516986.10018581.2-                tggtcctggtgataATGGCAGTTCTTTTCTCAAGTA
SRR516994.61946968.1-                tggtcctggtgataATGGCAGTTCTTTTCTCAAGTA
SRR517000.73288031.2-                tggtcctggtgataATGGCAGTTCTTTTCTCAAGTA
SRR516971.1738878.1-                  ggtcctggtgataATGGCAGTTCTTTTCTCAAGTA
SRR516985.37644018.1-                 ggtcctggtgataATGGCAGTTCTTTTCTCAAGTA
SRR516998.76032854.1+                 ggtcctggtgataATGGCAGTTCTTTTCTCAAGTA
SRR511915.2718550.2+                   gtcctggtgataATGGCAGTTCTTTTCTCAAGTA
SRR511915.2718704.2+                   gtcctggtgataATGGCAGTTCTTTTCTCAAGTA
SRR516989.14400971.1-                  gtcctggtgataATGGCAGTTCTTTTCTCAAGTA
SRR516989.56753297.1+                  gtcctggtgataATGGCAGTTCTTTTCTCAAGTA
SRR516993.46849885.2-                  gtcctggtgataATGGCAGTTCTTTTCTCAAGTA
SRR516995.55107914.1+                  gtcctggtgataATGGCAGTTCTTTTCTCAAGTA
SRR517006.56172332.1-                  gtcctggtgataATGGCAGTTCTTTTCTCAAGTA
SRR516979.12930171.1-                   tcctggtgataATGGCAGTTCTTTTCTCAAGTA
SRR516987.12110999.2-                   tcctggtgataATGGCAGTTCTTTTCTCAAGTA
SRR516989.7567542.2-                    tcctggtgataATGGCAGTTCTTTTCTCAAGTA
SRR516997.21795180.1-                   tcctggtgataATGGCAGTTCTTTTCTCAAGTA
SRR516999.38660980.2+                   tcctggtgataATGGCAGTTCTTTTCTCAAGTA
SRR517002.85671319.1+                   tcctggtgataATGGCAGTTCTTTTCTCAAGTA
SRR516973.1546854.2-                     cctggtgataATGGCAGTTCTTTTCTCAAGTA
SRR516979.61078971.2+                    cctggtgataATGGCAGTTCTTTTCTCAAGTA
SRR517005.77446950.2+                    cctggtgataATGGCAGTTCTTTTCTCAAGTA
SRR517007.27729441.2+                    cctggtgataATGGCAGTTCTTTTCTCAAGTA
```

SRR516973.36002427.1+ ctggtgataATGGCAGTTCTTTTCTCAAGGA
SRR517004.18234910.1- ctggtgataATGGCAGTTCTTTTCTCAAGTA
SRR517004.23357618.2- ctggtgataATGGCAGTTCTTTTCTCAAGTA
SRR517009.46479798.1+ ctggtgataATGGCAGTTCTTTTCTCAAGTA
SRR511892.28500594.1+ tggtgataATGGCAGTTCTTTTCTCAAGTA
SRR511911.53964321.2+ tggtgataATGGCAGTTCTTTTCTCAAGTA
SRR511914.39483852.2+ tggtgataATGGCAGTTCTTTTCTCAAGTA
SRR516974.30867255.1+ tggtgataATGGCAGTTCTTTTCTCAAGTA
SRR516976.22199380.2+ tggtgataATGGCAGTTCTTTTCTCAAGTA
SRR516985.1366211.2+ tggtgataATGGCAGTTCTTTTCTCAAGGA
SRR516989.56916901.2+ tggtgataATGGCAGTTCTTTTCTCAAGTA
SRR516999.6894145.1- tggtgataATGGCAGTTCTTTTCTCAAGTA
SRR511914.44813836.2+ ggtgataATGGCAGTTCTTTTCTCAAGTA
SRR511916.28951157.1+ ggtgataATGGCAGTTCTTTTCTCAAGTA
SRR511917.39957849.1+ ggtgataATGGCAGTTCTTTTCTCAAGTA
SRR516973.23808767.1- ggtgataATGGCAGTTCTTTTCTCAAGTA
SRR516973.23808771.1- ggtgataATGGCAGTTCTTTTCTCAAGTA
SRR516975.39272850.2- ggtgataATGGCAGTTCTTTTCTCAAGTA
SRR516988.60003491.2- ggtgataATGGCAGTTCTTTTCTCAAGTA
SRR516991.63425507.1+ ggtgataATGGCAGTTCTTTTCTCAAGTA
SRR517003.20939478.2- ggtgataATGGCAGTTCTTTTCTCAAGTA
SRR511892.35285726.2+ gtgataATGGCAGTTCTTTTCTCAAGTA
SRR511913.5222172.1- gtgataATGGCAGTTCTTTTCTCAAGTA
SRR516971.4236129.1- tgataATGGCAGTTCTTTTCTCAAGTA
SRR516999.47140010.1+ tgataATGGCAGTTCTTTTCTCAAGTA
SRR517000.83969593.2- tgataATGGCAGTTCTTTTCTCAAGTA
SRR517008.31099343.2- tgataATGGCAGTTCTTTTCTCAAGTA
SRR516968.128087344.1+ gataATGGCAGTTCTTTTCTCAAGTA
SRR516970.37367691.1+ gataATGGCAGTTCTTTTCTCAAGTA
SRR516971.34030929.1- gataATGGCAGTTCTTTTCTCAAGTA
SRR516972.31320955.1+ gataATGGCAGTTCTTTTCTCAAGTA
SRR516972.60791475.1+ gataATGGCAGTTCTTTTCTCAAGTA
SRR516984.7283570.1+ gataATGGCAGTTCTTTTCTCAAGTA
SRR516987.86762093.1- gataATGGCAGTTCTTTTCTCAAGTA
SRR516991.40998329.1- gataATGGCAGTTCTTTTCTCAAGTA
SRR516993.14043843.2- gataATGGCAGTTCTTTTCTCAAGTA
SRR516993.47859690.2- gataATGGCAGTTCTTTTCTCAAGTA
SRR516996.4820423.1- gataATGGCAGTTCTTTTCTCAAGTA
SRR517005.42320821.2- gataATGGCAGTTCTTTTCTCAAGTA
SRR516967.23066803.1+ ataATGGCAGTTCTTTTCTCAAGTA
SRR516968.73747636.2+ ataATGGCAGTTCTTTTCTCAAGTA
SRR516970.17885639.1- ataATGGCAGTTCTTTTCTCAAGTA
SRR516977.71210552.2- ataATGGCAGTTCTTTTCTCAAGTA
SRR516981.16534572.1+ ataATGGCAGTTCTTTTCTCAAGTA
SRR516985.84026477.1+ ataATGGCAGTTCTTTTCTCAAGTA
SRR517008.8977763.1- ataATGGCAGTTCTTTTCTCAAGTA
SRR511911.48293933.1+ taATGGCAGTTCTTTTCTCAAGTA
SRR511911.61131441.2- taATGGCAGTTCTTTTCTCAAGTA
SRR516967.41353207.1- taATGGCAGTTCTTTTCTCAAGTA
SRR516968.165290408.2- taATGGCAGTTCTTTTCTCAAGTA
SRR516986.10651932.2+ taATGGCAGTTCTTTTCTCAAGTA
SRR516995.20779522.2- taATGGCAGTTCTTTTCTCAAGTA
SRR516997.49184054.2+ taATGGCAGTTCTTTTCTCAAGTA
SRR517000.95262135.1- taATGGCAGTTCTTTTCTCAAGTA
SRR516969.33135240.1+ aATGGCAGTTCTTTTCTCAAGTA
SRR516969.867824.1+ aATGGCAGTTCTTTTCTCAAGTA
SRR516975.51826312.2- aATGGCAGTTCTTTTCTCAAGTA
SRR516989.80624643.2+ aATGGCAGTTCTTTTCTCAAGTA
SRR516992.65303336.1+ aATGGCAGTTCTTTTCTCAAGTA
SRR516993.19338558.2+ aATGGCAGTTCTTTTCTCAAGTA
SRR516993.35664507.1- aATGGCAGTTCTTTTCTCAAGTA
SRR516997.32779052.2+ aATGGCAGTTCTTTTCTCAAGTA
SRR517001.50757283.1- aATGGCAGTTCTTTTCTCAAGTA
SRR517002.67211818.1- aATGGCAGTTCTTTTCTCAAGTA
SRR517009.70549529.2+ aATGGCAGTTCTTTTCTCAAGTA
SRR516967.72187734.1+ ATGGCAGTTCTTTTCTCAAGTA
SRR516978.60261999.1- ATGGCAGTTCTTTTCTCAAGTA
SRR516985.6369730.2- ATGGCAGTTCTTTTCTCAAGTA
SRR516994.13045831.2+ ATGGCAGTTCTTTTCTCAAGTA
SRR516997.72118786.2+ ATGGCAGTTCTTTTCTCAAGTA
SRR516998.27579449.2+ ATGGCAGTTCTTTTCTCAAGTA
SRR516999.40240407.2+ ATGGCAGTTCTTTTCTCAAGTA
SRR517002.25042325.1+ ATGGCAGTTCTTTTCTCAAGTA
SRR517003.19342168.1+ ATGGCAGTTCTTTTCTCAAGTA
SRR516984.13867668.1- TGGCAGTTCTTTTCTCAAGTA
SRR516985.794078.1+ TGGCAGTTCTTTTCTCAAGTA
SRR516991.64722198.2- TGGCAGTTCTTTTCTCAAGTA
SRR517001.13478608.1+ TGGCAGTTCTTTTCTCAAGTA
SRR511915.2208112.2- GGCAGTTCTTTTCTCAAGTA
SRR511915.2208399.2- GGCAGTTCTTTTCTCAAGTA
SRR516968.80862039.2- GGCAGTTCTTTTCTCAAGTA
SRR516971.6695405.1+ GGCAGTTCTTTTCTCAAGTA
SRR516973.25479726.2+ GGCAGTTCTTTTCTCAAGTA
SRR511914.43073009.1+ GCAGTTCTTTTCTCAAGTA

SRR516985.90328884.2+	GCAGTTCTTTTCTCAAGTA
SRR516990.36711687.2-	GCAGTTCTTTTCTCAAGTA
SRR516991.52505003.2-	GCAGTTCTTTTCTCAAGTA
SRR516998.95088687.1-	GCAGTTCTTTTCTCAAGTA
SRR517002.26060078.2-	GCAGTTCTTTTCTCAAGTA
SRR511912.16411003.2-	CAGTTCTTTTCTCAAGTA
SRR511912.9044568.1+	CAGTTCTTTTCTCAAGTA
SRR511915.50178675.2-	CAGTTCTTTTCTCAAGTA
SRR511916.51401451.2-	CAGTTCTTTTCTCAAGTA
SRR516967.143090323.1-	CAGTTCTTTTCTCAAGTA
SRR516967.99920687.2-	CAGTTCTTTTCTCAAGTA
SRR516984.51860923.2+	CAGTTCTTTTCTCAAGTA
SRR516985.38800584.1+	CAGTTCTTTTCTCAAGTA
SRR517006.82333459.1-	CAGTTCTTTTCTCAAGTA
SRR511911.24447502.2-	AGTTCTTTTCTCAAGTA
SRR511915.12387403.1+	AGTTCTTTTCTCAAGTA
SRR516968.31712935.1-	AGTTCTTTTCTCAAGTA
SRR516972.48121654.2-	AGTTCTTTTCTCAAGTA
SRR516978.69371288.1-	AGTTCTGTTCTCAAGTA
SRR516980.49332165.2+	AGTTCTTTTCTCAAGTA
SRR516981.26581776.2+	AGTTCTTTTCTCAAGTA
SRR516986.22724390.1-	AGTTCTTTTCTCAAGTA
SRR516990.46955085.1+	AGTTCTTTTCTCAAGTA
SRR516993.77981446.2+	AGTTCTTTTCTCAAGTA
SRR516998.40607410.2-	AGTTCTTTTCTCAAGTA
SRR517009.19164909.2-	AGTTCTTTTCTCAAGTA
SRR517009.5972409.1-	AGTTCTTTTCTCAAGTA
SRR516974.48843984.2-	GTTCTTTTCTCAAGTA
SRR516978.14547831.1+	GTTCTTTTCTCAAGTA
SRR516985.70870904.1-	GTTCTTTTCTCAAGTA
SRR516995.16662105.2-	GTTCTTTTCTCAAGTA
SRR516967.114035844.1+	TTCTTTTCTCAAGTA
SRR516974.24280528.2-	TTCTTTTCTCAAGTA
SRR516999.51381955.2-	TTCTTTTCTCAAGTA
SRR517004.28032599.1-	TTCTTTTCTCAAGTA
SRR517006.64062576.1-	TTCTTTTCTCAAGTA
SRR517007.60890697.2+	TTCTTTTCTCAAGTA
SRR517009.45770063.2+	TTCTTTTCTCAAGTA
SRR511913.41367866.2+	TCTTTTCTCAAGTA
SRR511917.2380844.2-	TCTTTTCTCAAGTA
SRR511917.9454744.2-	TCTTTTCTCAAGTA
SRR511917.9454874.2-	TCTTTTCTCAAGTA
SRR516988.4949152.1-	TCTTTTCTCAAGTA
SRR516997.73355617.2+	TCTTTTCTCAAGTA
SRR516998.87531343.2-	TCTTTTCTCAAGTA
SRR517002.82258575.2-	TCTTTTCTCAAGTA
SRR517006.4335776.2-	TCTTTTCTCAAGTA
SRR517006.52685296.2-	TCTTTTCTCAAGTA
SRR516968.111104702.2-	CTTTTCTCAAGTA
SRR516982.49581501.1-	CTTTTCTCAAGTA
SRR516983.35103124.2+	CTTTTCTCAAGTA
SRR516967.3860522.2+	TTTTCTCAAGTA
SRR516970.33098467.1-	TTTTCTCAAGTA
SRR516974.1759552.2+	TTTTCTCAAGTA
SRR516979.30065340.2+	TTTTCTCAAGTA
SRR516984.6951606.2+	TTTTCTCAAGTA
SRR516986.17707572.1-	TTTTCTCAAGTA
SRR516999.32513950.1-	TTTTCTCAAGTA
SRR517000.67695666.1-	TTTTCTCAAGTA
SRR517007.47743023.1-	TTTTCTCAAGTA
SRR516967.71626809.2+	TTTCTCAAGTA
SRR516970.33098640.1-	TTTCTCAAGTA
SRR516974.33187826.2-	TTTCTCAAGTA
SRR516977.65941636.2-	TTTCTCAAGTA
SRR516979.7087082.1-	TTTCTCAAGTA
SRR516981.69540153.2-	TTTCTCAAGTA
SRR517000.44752129.1-	TTTCTCAAGTA
SRR516968.108115361.1+	TTCTCAAGTA
SRR516981.34446750.1-	TTCTCAAGTA
SRR516991.54630322.1-	TTCTCAAGTA
SRR517000.59906460.1+	TTCTCAAGTA
SRR511911.14454908.2+	TCTCAAGTA
SRR516972.30802147.2-	TCTCAAGTA
SRR517006.75846793.2+	TCTCAAGTA
SRR511914.85292277.1+	CTCAAGTA
SRR516992.59812008.2-	CTCAAGTA
SRR516992.60599956.2-	CTCAAGTA
SRR516994.13973863.1+	CTCAAGTA
SRR516996.47443095.1+	CTCAAGTA
SRR516996.84831988.2+	CTCAAGTA
SRR516999.45267731.1+	CTCAAGTA
SRR517002.81950718.1+	CTCAAGTA
SRR517007.25883181.1+	CTCAAGTA
SRR511915.68961737.2+	TCAAGTA

```
SRR516967.119505550.2-                                                          TCAAGTA
SRR516970.26934.1+                                                              TCAAGTA
SRR516972.53472127.1-                                                           TCAAGTA
SRR516972.77543660.1+                                                           TCAAGTA
SRR516978.70829906.1+                                                           TCAAGTA
SRR516995.4091683.2+                                                            TCAAGTA
SRR516999.38940100.1+                                                           TCAAGTA
SRR516999.42713432.1+                                                           TCAAGTA
SRR511915.72842805.1+                                                            CAAGTA
SRR516981.56879022.2+                                                            CAAGTA
SRR516997.12935966.2-                                                            CAAGTA
SRR517002.91703218.2+                                                            CAAGTA
SRR516972.67116381.2-                                                             AAGTA
SRR516985.71587022.2-                                                             AAGTA
SRR516990.23106415.2+                                                             AAGTA
SRR516992.42779638.1-                                                             AAGTA
SRR516995.27817063.1+                                                             AAGTA
SRR516996.41067957.1-                                                             AAGTA
SRR516996.64162159.1-                                                             AAGTA
SRR517001.43143516.2+                                                             AAGTA
SRR511914.83943589.1-                                                              AGTA
SRR516967.140688955.1+                                                             AGTA
SRR516984.11603344.2+                                                              AGTA
SRR516985.95011897.2+                                                              AGTA
SRR516990.30495340.2+                                                              AGTA
SRR516996.40869314.1+                                                              AGTA
SRR517007.50962620.2+                                                              AGTA
SRR516969.1709162.1-                                                                GTA
SRR516974.24221041.1-                                                               GTA
SRR516977.3418701.1+                                                                GTA
SRR516979.27449937.1+                                                               GTA
SRR516983.18957782.2+                                                               GTA
SRR516996.25148019.2+                                                               GTA
SRR516996.96865981.1-                                                               GTA
SRR516998.89996681.2+                                                               GTA
SRR516967.88384226.2+                                                                TA
SRR516977.2738721.1-                                                                 TA
SRR516992.78710121.1+                                                                TA
SRR516996.345191.1+                                                                  TA
SRR516999.41559143.1-                                                                TA
SRR516999.68726039.1-                                                                TA
SRR517006.8320014.2+                                                                 TA
SRR517009.40247008.2+                                                                TA
SRR511911.2560931.2-                                                                  A
SRR516968.114845186.2-                                                                A
SRR516968.37738736.1-                                                                 A
SRR516974.66712393.1+                                                                 A
SRR516978.44746730.2+                                                                 A
SRR516978.55364120.2-                                                                 A
SRR516981.43172628.1-                                                                 A
SRR516984.26297997.1+                                                                 A
SRR516999.58849034.2-                                                                 A
SRR516999.76775311.2+                                                                 A
SRR517003.5667689.1-                                                                  A
SRR517007.51506783.2+                                                                 A
SRR517008.35456369.2+                                                                 A
SRR517008.67351581.2+                                                                 A
                         ____________________________________________________________
consensus                attggcaagactccgaaattgcagtggtcctggtgataATGGCAGTTCTTTTCTCAAGTA

                             .    :    .    :    .    :    .    :    .    :    .    :
SRR516968.145184287.1+   T
SRR516973.16890128.2+    T
SRR516977.63657923.1+    T
SRR516997.90536592.1+    T
SRR517009.73111776.1-    T
SRR511911.61131441.1+    TC
SRR516976.5159916.1+     TC
SRR516981.85604642.1-    TC
SRR516990.15863859.2+    TC
SRR517002.54888386.2-    TC
SRR511892.9740145.1-     TCA
SRR511892.9740146.1-     TCA
SRR516967.6221170.2+     TCA
SRR516968.105088970.1-   TCA
SRR516977.1612623.2-     TCA
SRR516978.9119600.2+     TCA
SRR516982.50768215.2-    TCA
SRR516995.43843685.1-    TCA
SRR516996.33042242.1-    TCA
SRR516999.93433337.2-    TCA
SRR516973.16283767.2+    TCAA
SRR516989.75191770.1+    TCCA
SRR516989.8213552.2-     TCAA
```

SRR517003.12649627.2-	TCAA
SRR516968.15347757.1+	TCAAG
SRR516968.32902413.1-	TCAAG
SRR516972.54921884.2+	TCAAG
SRR516974.65206690.1-	TCAAG
SRR516977.42905173.2-	TCAAG
SRR516985.35211582.2-	TCAAG
SRR516988.3500447.1-	TCAAG
SRR516988.42192760.1-	TCAAG
SRR517006.47673006.2-	TCAAG
SRR516980.12879649.1-	TCAAGG
SRR516981.35251407.1-	TCAAGG
SRR516990.20604046.1-	TCAAGG
SRR517004.41059246.1+	TCAAGG
SRR517006.55093488.2-	TCAAGG
SRR517007.7845014.1-	TCAAGG
SRR511892.55624723.1-	TCAAGGG
SRR516968.156018396.2+	TCAAGGG
SRR516976.24305739.2+	TCAAAGG
SRR516993.84060022.1-	TCAAGGG
SRR517002.32972405.2-	TCAAGGG
SRR517007.35305430.2-	TCAAGGG
SRR516968.43850003.1+	TCAAGGGT
SRR516975.14702766.2-	TCAAGGGT
SRR516975.27931365.1-	TCAAGGGT
SRR516982.55151074.2+	TCAAGGGT
SRR516984.64702111.2-	TCAAGGGT
SRR516986.44931411.2+	TCAAGGGT
SRR516989.69704079.1+	TCAAGGGT
SRR516998.105122847.1-	TCAAGGGT
SRR516998.29687080.2-	TCAAGGGT
SRR517007.13507481.1+	TCAAGGGT
SRR517009.22120276.1+	TCAAGGGG
SRR511912.14258493.1-	TCAAGGGTA
SRR511912.14258649.1-	TCAAGGGTA
SRR516978.57147863.2-	TCAAGGGTA
SRR516996.31113264.1-	TCAAGGGTA
SRR516997.63356024.2-	TCAAGGGTA
SRR511916.23406501.2+	TCAAGGGGAT
SRR516968.98150509.1-	TCAAGGGTAT
SRR516971.15887332.2-	TCAAGGGTAT
SRR516976.8894843.2-	TCAAGGGTAT
SRR516985.49435852.1-	TCAAGGGTAT
SRR516989.32990384.1-	TCAAGGGTAT
SRR517002.15955724.1-	TCAAGGGTAT
SRR517006.5583744.1-	TCAAGGGTAT
SRR511913.51810263.1-	TCAAGGGTATG
SRR516968.6323756.1-	TCAAGGGTATG
SRR516971.24445351.1+	TCCAGGGTATG
SRR516978.75075983.1-	TCAAGGGTATG
SRR516983.72008022.1-	TCAAGGGTATG
SRR516974.65598927.2+	TCAAGGGCATGC
SRR516984.11685539.1+	TCAAGGGTATGC
SRR516985.9120641.2+	TCAAGGGTATGC
SRR516989.50454159.2-	TCAAGGGTATGC
SRR516998.54079381.1-	TCAAGGGTATGC
SRR517002.90607223.1+	TCAAGGGTATGC
SRR511911.40076074.2-	TCAAGGGTATGCT
SRR516978.4630760.1+	TCAAGGGTATGCT
SRR516986.15967055.2-	TCAAGGGTATGCT
SRR516991.70672748.1-	TCAAGGGTATGCT
SRR516996.66223613.1+	TCAAGGGTATGCT
SRR516999.3393409.2-	TCAAGGGTATGCT
SRR516999.78625944.1+	TCAAGGGGATGCT
SRR517005.73305592.2-	TCAAGGGTATGCT
SRR516977.50923694.1-	TCAAGGGTATGCTC
SRR516979.43816476.2+	TCAAGGGGCTGCTC
SRR516980.7236138.2-	TCAAGGGTATGCTCT
SRR516987.79339821.1-	TCAAGGGTATGCTCT
SRR516993.33273789.1-	TCAAGGGTATGCTCT
SRR516996.76179188.2+	TCAAGGGGATGCTCT
SRR516998.88264383.1+	TCCAGGGGATGCTCT
SRR517001.87930230.1-	TCAAGGGTATGCTCT
SRR517009.19677786.1-	TCAAGGGTATGCTCT
SRR511912.4156394.1-	TCAAGGGTATGCTCTT
SRR516972.51498046.1-	TCAAGGGTATGCTCTT
SRR516975.9441702.2-	TCAAGGGTATGCTCTT
SRR516981.76497254.2-	TCAAGGGTATGCTCTT
SRR517005.61532069.2-	TCAAGGGTATGCTCTT
SRR517005.7777984.1+	TCAAGGGGATGCTCTT
SRR517008.41558359.1+	TCAAGGGTATGCTCTT
SRR517009.26922977.2+	TCAAGGGGATGCTCTT
SRR511913.20668125.2-	TCAAGGGTATGCTCTTC
SRR511914.65840186.2-	TCAAGGGTATGCTCTTC

SRR516972.67844320.1-	TCAAGGGTATGCTCTTC
SRR516977.73763692.1-	TCAAGGGTATGCTCTTC
SRR516980.57095254.1+	TCAAGGGGATGCTCTTT
SRR516986.14794457.1+	TCAAGGGTATGCTCTTC
SRR516991.3697767.1+	TCAAGGGGATGCTCTTC
SRR516995.17755192.1+	TCAAGGGCATGCTCTTC
SRR516995.83414144.2-	TCAAGGGTATGCTCTTC
SRR516999.44920014.1+	TCAAGGGTATGGTCTTT
SRR516999.4718727.1+	TCAAGGGGATGCTCTTC
SRR517002.31507094.2-	TCAAGGGTATGCTCTTC
SRR511913.52001769.2-	TCAAGGGTATGCTCTTCC
SRR517003.39098715.2-	TCAAGGGTATGCTCTTCC
SRR516971.5736893.1-	TCAAGGGTATGCTCTTCCT
SRR516974.47709711.1+	TCAAGGGGATGCTCTTCCT
SRR516979.11039952.1-	TCAAGGGTATGCTCTTCCT
SRR516979.45053249.1-	TCAAGGGTATGCTCTTCCT
SRR516999.100313301.1+	TCAAGGGTATGCTCTTCCT
SRR511916.28951157.1+	TCAAGGGTATGCTCTTCCTC
SRR511917.39957849.1+	TCAAGGGTATGCTCTTCCTC
SRR516993.77596703.1+	TCAAGGGTATGCTCTTCCTC
SRR516996.52401398.1+	TCAAGGGTATGCTCTTCCTC
SRR511915.2956436.1-	TCAAGGGTATGCTCTTCCTCT
SRR511915.2956742.1-	TCAAGGGTATGCTCTTCCTCT
SRR516983.19283942.1+	TCAAGGGGATGCTCTTCCTCT
SRR516997.4512656.1-	TCAAGGGTATGCTCTTCCTCT
SRR511912.1014472.1-	TCAAGGGTATGCTCTTCCTCTT
SRR516967.94416127.2+	TCAAGGGTATGCTCTTCCTCTT
SRR516974.36849866.2-	TCAAGGGTATGCTCTTCCTCTT
SRR516979.16080749.1+	TCAAGGGTATGCTCTTCCTCTT
SRR516980.8816673.2-	TCAAGGGTATGCTCTTCCTCTT
SRR516981.35677677.2-	TCAAGGGTATGCTCTTCCTCTT
SRR516986.21413362.2+	TCAAGGGTATGCTCTTCCTCTT
SRR516986.37049200.1-	TCAAGGGTATGCTCTTCCTCTT
SRR516995.87128240.2-	TCAAGGGTATGCTCTTCCTCTT
SRR516998.28200586.2-	TCAAGGGTATGCTCTTCCTCTT
SRR516999.92446757.1-	TCAAGGGTATGCTCTTCCTCTT
SRR517002.97801972.1-	TCAAGGGTATGCTCTTCCTCTT
SRR517003.21132339.1-	TCAAGGGTATGCTCTTCCTCTT
SRR517004.51768700.2-	TCAAGGGTATGCTCTTCCTCTT
SRR517007.8592877.1-	TCAAGGGTATGCTCTTCCTCTT
SRR511911.29686714.2-	TCAAGGGTATGCTCTTCCTCTTG
SRR511911.37480006.2-	TCAAGGGTATGCTCTTCCTCTTG
SRR511915.46810937.2+	TCAAGGGTATGCTCTTCCTCTTG
SRR516968.115657864.2-	TCAAGGGTATGCTCTTCCTCTTG
SRR516973.50577545.1+	TCAAGGGTAAGCTCTTCCCCTTG
SRR516976.12707927.1+	TCAAGGGGATGCTCTTCCTCTTG
SRR516998.53967676.2-	TCAAGGGTATGCTCTTCCTCTTG
SRR516978.20535847.2-	TCAAGGGTATGCTCTTCCTCTTGC
SRR516999.39456345.2-	TCAAGGGTATGCTCTTCCTCTTGC
SRR517007.68739089.1+	TCAAGGGGATGCTCTTCCTCTTGC
SRR517008.75782648.1-	TCAAGGGTATGCTCTTCCTCTTGC
SRR511913.1169864.1+	TCAAGGGTATGGTCTTCCTCTTGCT
SRR511913.18852311.1-	TCAAGGGTATGCTCTTCCTCTTGCT
SRR516967.146282791.1-	TCAAGGGTACGCTCTTCCTCTTGCT
SRR516971.1110705.2+	TCAAGGGTATGCTCTTCCTCTTGCT
SRR516973.17477597.1-	TCAAGGGTATGCTCTTCCTCTTGCT
SRR516973.19946759.2-	TCAAGGGTATGCTCTTCCTCTTGCT
SRR516974.72485008.1-	TCAAGGGTATGCTCTTCCTCTTGCT
SRR516979.5438653.1+	TCAAGGGTATGCTCTTCCTCTTGCT
SRR516982.31349844.1-	TCAAGGGTATGCTCTTCCTCTTGCT
SRR516991.89333198.1+	TCAAGGGGATGCTCTTCCTCTTGCT
SRR517000.36678948.1+	TCAAGGGGATGCTCTTCCTCTTGCT
SRR517001.63940723.2+	TCCAGGGTATGCTCTTTCTCTTGCT
SRR517004.42185234.1-	TCAAGGGTATGCTCTTCCTCTTGCT
SRR517009.7493725.1-	TCAAGGGTATGCTCTTCCTCTTGCT
SRR511911.45670450.2-	TCAAGGGTATGCTCTTCCTCTTGCTCC
SRR516972.58439524.1-	TCAAGGGTATGCTCTTCCTCTTGCTCC
SRR516978.48507581.2+	TCAAGGGTATGCTCTTCCTCATGCTCC
SRR516981.23321590.2-	TCAAGGGTATGCTCTTCCTCTTGCTCC
SRR516984.23515395.1+	TCAAGGGTATGCTCTTCCTCTTGCTCC
SRR516995.41614722.2-	TCAAGGGTATGCTCTTCCTCTTGCTCC
SRR517004.23920483.1+	TCAAGGGGATGCTCTTCCTCTTGCTCC
SRR511892.31593589.1-	TCAAGGGTATGCTCTTCCTCTTGCTCCT
SRR516968.118272353.2+	TCAAGGGTATGCTCTTCCTCTTGCTCCT
SRR516970.7640225.2+	TCAAGGGTATGCTCTTCCTCTTGCTCCT
SRR516986.24103450.2-	TCAAGGGTATGCTCTTCCTCTTGCTCCT
SRR516989.22778442.2+	TCAAGGGTATGCTCTTCCTCTTGCTCCT
SRR517000.54991829.2+	TCAAGGGCATGCTCTTCCTCTTGCTCCT
SRR517009.26982378.2+	TCAAGGGGATGCTCTTCCTCTTGCTCCT
SRR511912.20631234.2-	TCAAGGGTATGCTCTTCCTCTTGCTCCTC
SRR516973.52994151.1-	TCAAGGGTATGCTCTTCCTCTTGCTCCTC
SRR516981.26964373.2+	TCAAGGGTATGCTCTTCCTCTTGCTCCTC
SRR516985.219617.1+	TCAAGGGGATGGTCTTCCTCTTGCTCCTC
SRR516989.44612348.1-	TCAAGGGTATGCTCTTCCTCTTGCTCCTC

SRR517008.63631025.2- TCAAGGGTATGCTCTTCCTCTTGCTCCTC
SRR516974.42555202.2+ TCAAGGGGATGCTCTTCCTCTTGCTCCTCC
SRR516990.40763061.2+ TCAAGGGGATGCTCTTCCTCTTGCTCCTCC
SRR516994.27805045.1- TCAAGGGTATGCTCTTCCTCTTGCTCCTCC
SRR516997.55886851.2- TCAAGGGTATGCTCTTCCTCTTGCTCCTCC
SRR516997.58614765.1+ TCAAGGGTATGCTCTTTCTCTTTCTCCTCC
SRR516998.100156656.1+ TCAAGGGGATGCTCTTCCTCTTGCTCCTCC
SRR516999.5917.1+ TCAAGGGGATGCTCTTCCTCTTGCTCCTCC
SRR511916.51401451.2- TCAAGGGTATGCTCTTCCTCTTGCTCCTCCC
SRR516974.1040705.1+ TCAAGGGGATGCTCTTCCTCTTGCTTCTCCC
SRR517000.66457751.1+ TCAAGGGTATGCTCTTCCTCTTGCTCCTCCC
SRR517002.10945551.1- TCAAGGGTATGCTCTTCCTCTTGCTCCTCCC
SRR511911.17781748.1- TCAAGGGTATGCTCTTCCTCTTGCTCCTCCCA
SRR516975.4624241.1+ TCAAGGGTATGCTCTTCCTCTTGCTCCTCCCA
SRR516987.76366948.2- TCAAGGGTATGCTCTTCCTCTTGCTCCTCCCA
SRR516990.64951029.2+ TCAAGGGTATGCTCTTCCTCTTGCTCCTCCCA
SRR516993.11025707.2- TCAAGGGTATGCTCTTCCTCTTGCTCCTCCCA
SRR516998.78835527.1+ TCAAGGGTATGCTCTTCCTCTTGCTCCTCCCA
SRR516998.83658111.2+ TCAAGGGTATGCTCTTCCTCTTGCTCCTCCCA
SRR517000.39640223.2+ TCAAGGGTATGCTCTTCCTCTTGCTCCTCCCA
SRR517002.44210664.1- TCAAGGGTATGCTCTTCCTCTTGCTCCTCCCA
SRR517002.50187769.1+ TCAAGGGGATGCTCTTCCTCTTGCTCCTCCCC
SRR511911.71659560.2+ TCAAGGGTATGCTCTTCCTCTTGCTCCTCCCCT
SRR511913.8247697.2+ TCAAGGGTATGCTCTTCCTCTTGCTCCTCCCCT
SRR516974.46565740.2- TCAAGGGTATGCTCTTCCTCTTGCTCCTCCCAT
SRR516978.52604776.1- TCAAGGGTATGCTCTTCCTCTTGCTCCTCCCAT
SRR516985.4837674.2+ TCTAGGGGAAGCTCTTCCTCTTGCTCCTTCCCT
SRR511912.72370488.2+ TCAAGGGGATGCTCTTCCTCTTGCTCCTCCCCTC
SRR516978.38239451.2- TCAAGGGTATGCTCTTCCTCTTGCTCCTCCCATA
SRR516991.87080495.1- TCAAGGGTATGCTCTTCCTCTTGCTCCTCCCATA
SRR516999.14760583.2- TCAAGGGTATGCTCTTCCTCTTGCTCCTCCCATA
SRR517008.76003299.2- TCAAGGGTATGCTCTTCCTCTTGCTCCTCCCATA
SRR511917.2380844.2- TCAAGGGTATGCTCTTCCTCTTGCTCCTCCCATAC
SRR511917.9454744.2- TCAAGGGTATGCTCTTCCTCTTGCTCCTCCCATAC
SRR511917.9454874.2- TCAAGGGTATGCTCTTCCTCTTGCTCCTCCCATAC
SRR516967.54383831.1- TCAAGGGTATGCTCTTCCTCTTGCTCCTCCCATAC
SRR516973.60429677.2+ TCAAGGGGATGCTCTTCCTCTTGCTCCTCCCCTAC
SRR516978.14537581.1+ TCAAGGGGATGCTCTTCCTCTTGCTCCTCCCCTAC
SRR516980.14193627.1+ TCAAGGGGATGCTCTTCCTCTTGCTCCTCCCATAC
SRR516987.56665405.2+ TCAAGGGTATGCTCTTCCTCTTGCTCCTCCCATAC
SRR516993.66881456.2+ TCAAGGGTATGCTCTTCCTCTTGCTCCTCCCATAC
SRR517000.5336928.1- TCAAGGGTATGCTCTTCCTCTTGCTCCTCCCATAC
SRR511892.45941781.1+ TCAAGGGTATGCTCTTCCTCTTGCTCCTCCCCTACT
SRR516968.140796502.2+ TCAAGGGTATGCTCTTCCTCTTGCTCCTCCCATACT
SRR516983.47760221.1- TCAAGGGTATGCTCTTCCTCTTGCTCCTTCCATACT
SRR516992.6818043.1- TCAAGGGTATGCTCTTCCTCTTGCTCCTCCCATACT
SRR516994.75464207.2+ TCAAGGGTATGCTCTTCCTCTTGCTCCTCCCCTACT
SRR516996.89309997.1+ TCAAGGGTATGCTCTTCCTCTTGCTCCTCCCCTACT
SRR516979.60567804.1+ TCAAGGGTATGCTCTTCCTCTTGCTCCTCCCCTACTT
SRR516990.13785933.1- TCAAGGGTATGCTCTTCCTCTTGCTCCTCCCATACTT
SRR516990.60703481.2- TCAAGGGTATGCTCTTCCTCTTGCTCCTCCCATACTT
SRR517008.30257719.1+ TCAAGGGTATGCTCTTCCTCTTTCTCCTCCCCTACTT
SRR511914.68007108.2- TCAAGGGTATGCTCTTCCTCTTGCTCCTCCCATACTTT
SRR511915.67432792.1+ TCAAGGGGATGCTCTTCCTCTTGCTCCTCCCATACTTT
SRR516976.70234108.2- TCAAGGGTATGCTCTTCCTCTTGCTCCTCCCATACTTT
SRR516977.47216005.2+ TCAAGGGGATGCTCTTCCTCTTGCTCCTCCCATACTTT
SRR516984.23605175.2+ TCAAGGGTATGCTCTTCCTCTTGCTCCTCCCATACTTT
SRR516987.1841637.2- TCAAGGGTATGCGCTTCCTCTTGCTCCTCCCATACTTT
SRR517006.41660503.1- TCAAGGGTATGCTCTTCCTCTTGCTCCTCCCATACTTT
SRR511912.19294174.1+ TCAAGGGGATGCTCTTCCCCTTGCTCCTCCCATAATTTT
SRR511912.19294330.1+ TCAAGGGGATGCTCTTCCTCTTGCTCCTCCCCTACTTTT
SRR516972.58990870.1- TCAAGGGTATGCTCTTCCTCTTGCTCCTCCCATACTTTT
SRR516975.45005523.1- TCAAGGGTATGCTCTTCCTCTTGCTCCTCCCATACTTTT
SRR516977.70276963.1+ TCAAGGGTATGCTCTTCCTCTTGCTCCTCCCATACTTTT
SRR517004.33967467.1+ TCAAGGGTATGCTCTTCCTCTTGCTCCTCCCATACTTTT
SRR517007.68737803.1- TCAAGGGTATGCTCTTCCTCTTGCTCCTCCCATACTTTT
SRR511892.68477751.1- TCAAGGGTATGCTCTTCCTCTTGCTCCTCCCATACTTTTG
SRR516973.18821850.2- TCAAGGGTATGCTCTTCCTCTTGCTCCTCCCATACTTTTG
SRR516973.18821853.2- TCAAGGGTATGCTCTTCCTCTTGCTCCTCCCATACTTTTG
SRR516973.42617646.2- TCAAGGGTATGCTCTTCCTCTTGCTCCTCCCATACTTTTG
SRR516974.6201811.2+ TCAAGGGGATGCTCTTCCTCTTGCTCCTCCCATACTTTTG
SRR516999.97278751.1+ TCAAGGGGATGCTCTTCCCCTTGCTCCTCCCATACTTTTG
SRR516967.14759904.2+ TCAAGGGTATGCTCTTCCTCTTGCTGCTCCCATCCTTTTGT
SRR517008.5489452.2- TCAAGGGTATGCTCTTCCTCTTGCTCCTCCCATACTTTTGT
SRR516972.57623491.1- TCAAGGGTATGCTCTTCCTCTTGCTCCTCCCATACTTTTGTT
SRR516975.29785312.1- TCAAGGGTATGCTCTTCCTCTTGCTCCTCCCATACTTTTGTT
SRR516981.43595596.1- TCAAGGGTATGCTCTTCCTCTTGCTCCTCCCATACTTTTGTT
SRR516983.13010793.1- TCAAGGGTATGCTCTTCCTCTTGCTCCTCCCATACTTTTGTT
SRR516989.66572423.2+ TCAAGGGTATGCTCTTCCTCTTGCTCCTCCCATACTTTTGTT
SRR516999.1552100.2- TCAAGGGTATGCTCTTCCTCTTGCTCCTCCCATACTTTTGTT
SRR517005.67585549.2- TCAAGGGTATGCTCTTCCTCTTGCTCCTCCCATACTTTTGTT
SRR511914.19951607.1- TCAAGGGTATGCTCTTCCTCTTGCTCCTCCCATACTTTTGTTT
SRR516969.9631131.1+ TCAAGGGTATGCTCTTCCTCTTGCTCCTCCCATACTTTTGTTT
SRR516975.41855794.2+ TCAAGGGTATGCTCTTCCTCTTGCTCCTCCCATACTTTTGTTT

SRR516986.39849151.2- TCAAGGGTATGCTCTTCCTCTTGCTCCTCCCATACTTTTGTTT
SRR517000.91150091.2+ TCAAGGGTATGCTCTTCCTCTTGCTCCTCCCATACTTTTGTTT
SRR517004.53237476.2+ TCAAGGGTATGCTCTTCCTCTTGCTCCTCCCATACTTTTGTTT
SRR517005.54024085.1- TCAAGGGTATGCTCTTCCTCTTGCTCCTCCCATACTTTTGTTT
SRR516967.129799720.1- TCAAGGGTATGCTCTTCCTCTTGCTCCTCCCATACTTTTGTTTT
SRR516984.75040283.2- TCAAGGGTATGCTCTTCCTCTTGCTCCTCCCATACTTTTGTTTT
SRR516990.22073434.1- TCAAGGGTATGCTCTTCCTCTTGCTCCTCCCATACTTTTGTTTT
SRR516995.43463521.1- TCAAGGGTATGCTCTTCCTCTTGCTCCTCCCATACTTTTGTTTT
SRR516997.21480163.2- TCAAGGGTATGCTCTTCCTCTTGCTCCTCCCATACTTTTGTTTT
SRR516997.47194944.1- TCAAGGGTATGCTCTTCCTCTTGCTCCTCCCATACTTTTGTTTT
SRR517006.12195593.1+ TCAAGGGGATGCTCTTCCTCTTGCTCCTCCCATACTTTTGTTTT
SRR517009.52663891.2+ TCAAGGGTATGCTCTTCCTCTTGCTCCTCCCATACTTTTGTTTT
SRR516975.25984190.1- TCAAGGGTATGCTCTTCCTCTTGCTCCTCCCATACTTTTGTTTTG
SRR516983.70623253.2- TCAAGGGTATGCTCTTCCTCTAGCTCCTCCCATACTTTTCTTTTG
SRR516989.30730922.2+ TCAAGGGTATGCTCTTCCTCTTGCTCCTCCCATACTTTTGTTTTG
SRR517003.23333068.2- TCAAGGGTATGCTCTTCCTCTTGCTCCTCCCATACTTTTGTTTTG
SRR517006.80972378.1- TCAAGGGTATGCTCTTCCTCTTGCTCCTCCCATACTTTTGTTTTG
SRR511892.53930910.2+ TCAAGGGTATGCTCTTCCTCTTGCTCCTCCCATACTTTTGTTTTTG
SRR511911.45879133.1+ TCAAGGGTATGCTCTTCCTCTTGCTCCTCCCATACTTTTGTTTTGG
SRR516967.102120548.1- TCAAGGGTATGCTCTTCCTCTTGCTCCTCCCATACTTTTGTTTTGG
SRR516967.59801249.2- TCAAGGGTATGCTCTTCCTCTTGCTCCTCCCNTACTTTTGTTTTGG
SRR516974.33490101.2+ TCCAGGGTATGCTCTTCCTCTTGCTCCTCCCATACTTTTGTTTTGG
SRR516977.1264704.2- TCAAGGGTATGCTCTTCCTCTTGCTCCTCCCATACTTTTGTTTTGG
SRR516985.95116448.1- TCAAGGGTATGCTCTTCCTCTTGCTCCTCCCATACTTTTGTTTTGG
SRR516991.27367214.1+ TCAAGGGTATGCTCTTCCTCTTGCTCCTCCCATACTTTTGTTTTGG
SRR516998.88169944.2+ TCAAGGGTATGCTCTTCCTCTTGCTCCTCCCATACTTTTGTTTTGG
SRR516999.94992807.1+ TCAAGGGTATGCTCTTCCTCTTGCTCCTCCCATACTTTTGTTTTTG
SRR511915.10990174.2- TCAAGGGTATGCTCTTCCTCTTGCTCCTCCCATTCTTTTGTTTTGGT
SRR511915.2718704.2+ TCAAGGGTATGCTCTTCCTCTTGCTCCTCCCATACTTTTGTTTTTGT
SRR517001.52537783.2+ TCAAGGGTATGCTCTTCCTCTTGCTCCTCCCATACTTTTGTTTTGGT
SRR511892.22460703.1+ TCAAGGGTATGCTCTTCCTCTTGCTCCTCCCATACTTTTGTTTTGGTC
SRR516972.87344728.1+ TCAAGGGGATGCTCTTCCTCTTGCTCCTCCCATACTTTTGTTTTGGTC
SRR516984.84534820.2+ TCAAGGGTATGCTCTTCCTCTTGCTCCTCCCATACTTTTGTTTTGGTC
SRR516967.118206337.1- TCAAGGGTATGCTCTTCCTCTTGCTCCTCCCATACTTTTGTTTTGGTCA
SRR516980.39425242.2- TCAAGGGTATGCTCTTCCTCTTGCTCCTCCCATACTTTTGTTTTGGTCA
SRR516991.44566949.1+ TCAAGGGTATGCTCTTCCTCTTGCTCCTCCCATACTTTTGTTTTGGTCA
SRR516999.92033228.2- TCAAGGGTATGCTCTTCCTCTTGCTCCTCCCATACTTTTGTTTTGGTCA
SRR517003.11858841.1+ TCAAGGGTATGCTCTTCCTCTTGCTCCTCCCATACTTTTGTTTTGGTCA
SRR517008.88421506.1+ TCAAGGGTATGCTCTTCCTCTTGCTCCTCCCATACTTTTGTTTTGGTCA
SRR516973.36002427.1+ TCAAGGGTATGCTCTTCCTCTTGCTCCTCCCATACTTTTGTTTTGGTCAG
SRR516991.36445672.2+ TCAAGGGGATGCTCTTCCTCTTGCCCCTCCCATACTTTTGTTTTGGTCAG
SRR517009.56817958.1- TCAAGGGTATGCTCTTCCTCTTGCTCCTCCCATACTTTTGTTTTGGTCAG
SRR511912.61545448.1+ TCAAGGGTATGCTCTTCCTCTTGCTCCTCCCATACTTTTGTTTTTGTCAGC
SRR511912.74095538.1- TCAAGGGTATGCTCTTCCTCTTGCTCCTCCCATACTTTTGTTTTGGTCAGC
SRR516967.132651451.2+ TCAAGGGTATGCTCTTCCTCTTGCTCCTCCCATACTTTTGTTTTGGTCAGC
SRR516967.94039320.2+ TCAAGGGTATGCTCTTCCTCTTGCTCCTCCCATACTTTTGTTTTGGTCAGC
SRR516969.22222210.2- TCAAGGGTATGCTCTTCCTCTTGCTCCTCCCATACTTTTGTTTTGGTCAGC
SRR516986.15638143.1- TCAAGGGTATGCTCTTCCTCTTGCTCCTCCCATACTTTTGTTTTGGTCAGC
SRR516994.22414233.2+ TCAAGGGTATGCTCTTCCTCTTGCTCCTCCCATACTTTTGTTTTGGTCAGC
SRR517001.66298626.2+ TCAAGGGTATGCTCTTCCTCTTGCTCCTCCCATACTTTTGTTTTGGTCAGC
SRR517008.59060241.2- TCAAGGGTATGCTCTTCCTCTTGCTCCTCCCATACTTTTGTTTTGGTCAGC
SRR516974.47716003.1+ TCAAGGGTATGCTCTTCCTCTTGCTCCTCCCATACTTTTGTTTTGGTCAGCC
SRR516975.13576344.2- TCAAGGGTATGCTCTTCCTCTTGCTCCTCCCATACTTTTGTTTTGGTCAGCC
SRR516994.76783997.1- TCAAGGGTATGCTCTTCCTCTTGCTCCTCCCATACTTTTGTTTTGGTCAGCC
SRR517005.62626634.1+ TCAAGGGTATGCTCTTCCTCTTGCTCCTCCCATACTTTTGTTTTGGTCAGCC
SRR517006.45290877.1+ TCAAGGGTATGCTCTTCCTCTTGCTCCTCCCATTCTTTTGTTTTGGTCAGCC
SRR516967.149674807.1+ TCAAGGGTATGCTCTTCCTCTTGCTCCTCCCATACTTTTGTTTTGGTCAGCCT
SRR516970.30184580.1- TCAAGGGTATGCTCTTCCTCTTGCTCCTCCCATACTTTTGTTTTGGTCAGCCT
SRR516982.4572095.1- TCAAGGGTATGCTCTTCCTCTTGCTCCTCCCATACTTTTGTTTTGGTCAGCCT
SRR516985.37644018.1- TCAAGGGTATGCTCTTCCTCTTGCTCCTCCCATACTTTTGTTTTGGTCAGCCT
SRR516985.59438916.2- TCAAGGGTATGCTCTTCCTCTTGCTCCTCCCATACTTTTGTTTTGGTCAGCCT
SRR516988.29683805.2- TCAAGGGTATGCTCTTCCTCTTGCTCCTCCCATACTTTTGTTTTGGTCAGCCT
SRR516991.4931934.2- TCAAGGGTATGCTCTTCCTCTTGCTCCTCCCATACTTTTGTTTTGGTCAGCCT
SRR516998.32153278.1- TCAAGGGTATGCTCTTCCTCTTGCTCCTCCCATACTTTTGTTTTGGTCAGCCT
SRR516998.43339497.2- TCAAGGGTATGCTCTTCCTCTTGCTCCTCCCATACTTTTGTTTTGGTCAGCCT
SRR517006.89664623.2- TCAAGGGTATGCTCTTCCTCTTGCTCCTCCCATACTTTTGTTTTGGTCAGCCT
SRR516976.39031867.1+ TCAAGGGGATGCTCTTCCTCTTGCTCCTCCCATACTTTTGTTTTGGTCAGCCTG
SRR516986.10018581.2- TCAAGGGTATGCTCTTCCTCTTGCTCCTCCCATACTTTTGTTTTGGTCAGCCTG
SRR516994.61946968.1- TCAAGGGTATGCTCTTCCTCTTGCTCCTCCCATACTTTTGTTTTGGTCAGCCTG
SRR517000.73288031.2- TCAAGGGTATGCTCTTCCTCTTGCTCCTCCCATACTTTTGTTTTGGTCAGCCTG
SRR511913.45378464.2+ TCAAGGGGATGCTCTTCCTCTTGCTCCTCCCATACTTTTGTTTTGGTTAGCCTGC
SRR516968.40141403.2+ TCAAGGGTATGCTCTTCCTCTTGCTCCTCCCATACTTTTGTTTTGGTCAGCCTGC
SRR516969.13918852.2+ TCAAGGGGATGCTCTTCCTCTTGCTCCTCCCATACTTTTGTTTTGGTCAGCCTGC
SRR516971.6332637.2+ TCAAGGGTATGCTCTTCCTCTTGCTCCTCCCATACTTTTGTTTTGGTCAGCCTGC
SRR516998.76032854.1+ TCAAGGGTATGCTCTTCCTCTTGCTCCTCCCATACTTTTGTTTTGGTCAGCCTGC
SRR511911.73201164.1- TCAAGGGTATGCTCTTCCTCTTGCTCCTCCCATACTTTTGTTTTGGTCAGCCTGCA
SRR516985.1366211.2+ TCAAGGGAATGCTCTTCCTCTTGCTCCTCCCAGACTTTTGTTTTTGTCCGCCTGCA
SRR516989.14400971.1- TCAAGGGTATGCTCTTCCTCTTGCTCCTCCCATACTTTTGTTTTGGTCAGCCTGCA
SRR516989.56753297.1+ TCAAGGGTATGCTCTTCCTCTTGCTCCTCCCATACTTTTGTTTTGGTCAGCCTGCA
SRR516993.46849885.2- TCAAGGGTATGCTCTTCCTCTTGCTCCTCCCATACTTTTGTTTTGGTCAGCCTGCA
SRR516995.55107914.1+ TCAAGGGTATGCTCTTCCTCTTGCTCCTCCCATACTTTTGTTTTGGTCAGCCTGCA
SRR517006.56172332.1- TCAAGGGTATGCTCTTCCTCTTGCTCCTCCCATACTTTTGTTTTGGTCAGCCTGCA
SRR516968.21564094.1+ TCAAGGGTATGCTCTTCCTCTTGCTCCTCCCATACTTTTGTTTTGGTCAGCCTGCAC
SRR516979.12930171.1- TCAAGGGTATGCTCTTCCTCTTGCTCCTCCCATACTTTTGTTTTGGTCAGCCTGCAC

SRR516987.12110999.2-   TCAAGGGTATGCTCTTCCTCTTGCTCCTCCCATACTTTTGTTTTGGTCAGCCTGCAC
SRR516989.7567542.2-   TCAAGGGTATGCTCTTCCTCTTGCTCCTCCCATACTTTTGTTTTGGTCAGCCTGCAC
SRR516997.21795180.1-   TCAAGGGTATGCTCTTCCTCTTGCTCCTCCCATACTTTTGTTTTGGTCAGCCTGCAC
SRR516999.38660980.2+   TCAAGGGTATGCTCTTCCTCTTGCTCCTCCCATACTTTTGTTTTGGTCAGCCTGCAC
SRR517002.85671319.1+   TCAAGGGTATGCTCTTCCTCTTGCTCCTCCCATACTTTTGTTTTGGTCAGCCTGCAC
SRR511915.2718550.2+   TCTAGGGTATGCTCCTCCTCTTGCTCCTCCCATACTTTTGTTTTGGTCAGCCTTCACC
SRR516967.138148601.2+   TCAAGGGTATGCTCTTCCTCTTGCTCCTCCCATACTTTTGTTTTGGTCAGCCTGCACC
SRR516969.34784658.2-   TCAAGGGTATGCTCTTCCTCTTGCTCCTCCCATACTTTTGTTTTGGTCAGCCTGCACC
SRR516973.1546854.2-   TCAAGGGTATGCTCTTCCTCTTGCTCCTCCCATACTTTTGTTTTGGTCAGCCTGCACC
SRR516979.61078971.2+   TCAAGGGTATGCTCTTCCTCTTGCTCCTCCCATACTTTTGTTTTGGTCAGCCTGCACC
SRR517005.77446950.2+   TCAAGGGTATGCTCTTCCTCTTGCTCCTCCCATACTTTTGTTTTGGTCAGCCTGCACC
SRR517007.27729441.2+   TCAAGGGTATGCTCTTCCTCTTGCTCCTCCCATACTTTTGTTTTGGTCAGCCTGCACC
SRR511911.13404865.2+   TCAAGGGTATGCTCTTCCTCTTGCTCCTCCCATACTTTTGTTTTGGTCAGCCTGCACCT
SRR516967.121342033.1-   TCAAGGGTATGCTCTTCCTCTTGCTCCTCCCATACTTTTGTTTTGGTCAGCCTGCACCT
SRR517004.18234910.1-   TCAAGGGTATGCTCTTCCTCTTGCTCCTCCCATACTTTTGTTTTGGTCAGCCTGCACCT
SRR517004.23357618.2-   TCAAGGGTATGCTCTTCCTCTTGCTCCTCCCATACTTTTGTTTTGGTCAGCCTGCACCT
SRR517009.46479798.1+   TCAAGGGTATGCTCTTCCTCTTGCTCCTCCCATACTTTTGTTTTGGTCAGCCTGCACCT
SRR511892.27528962.1-   TCAAGGGTATGCTCTTCCTCTTGCTCCTCCCATACTTTTGTTTTGGTCAGCCTGCACCTT
SRR511892.28500594.1+   TCAAGGGGATGCTCTTCCTCTTGCTCCTCCCATACTTTTGTTTTGGTCAGCCTGCACCTT
SRR511892.35285726.2+   TCAAAGGTATGCTCTTCCTCTTGCTCCTCCCATACTTTTGTTTTGGTCAGCCTGCACCTT
SRR511911.14454908.2+   TCAAGGGTATGCTCTTCCTCTTGCTCCTCCCATACTTTTGTTTTGGTCAGCCTGCACCTT
SRR511911.24447502.2-   TCAAGGGTATGCTCTTCCTCTTGCTCCTCCCATACTTTTGTTTTGGTCAGCCTGCACCTT
SRR511911.2560931.2-   TCAAGGGTATGCTCTTCCTCTTGCTCCTCCCATACTTTTGTTTTGGTCAGCCTGCACCTT
SRR511911.48293933.1+   TCAAGGGTATGCTCTTCCTCTTGCTCCTCCCATACTTTTGTTTTGGTCAGCCTGCACCTT
SRR511911.53964321.2+   TCAAGGGTATGCTCTTCCTCTTGCTCCTCCCATACTTTTGTTTTGGTCAGCCTGCACCTT
SRR511911.61131441.2-   TCAAGGGTATGCTCTTCCTCTTGCTCCTCCCATACTTTTGTTTTGGTCAGCCTGCACCTT
SRR511912.16411003.2-   TCAAGGGTATGCTCTTCCTCTTGCTCCTCCCATACTTTTGTTTTGGTCAGCCTGCACCTT
SRR511912.9044568.1+   TCAAGGGTATGCTCTTCCTCTTGCTCCTCCCATACTTTTGTTTTGGTCAGCCTGCACCTT
SRR511913.41367866.2+   TCAAGGGTATGCTCTTCCTCTTGCTCCTCCCATACTTTTGTTTTGGTCAGCCTGCACCTT
SRR511913.5222172.1-   TCAAGGGTATGCTCTTCCTCTTGCTCCTCCCATACTTTTGTTTTGGTCAGCCTGCACCTT
SRR511914.17741304.2+   TCAAGGGGATGCTCTTCCTCTTGCTCCTCCCATACTTTTGTTTTGGTCAGCCTGCACCTT
SRR511914.39483852.2+   TCAAGGGTATGCTCTTCCTCTTGCTCCTCCCATACTTTTGTTTTGGTCAGCCTGCACCTT
SRR511914.43073009.1+   TCAAGGGTATGCTCTTCCTCTTGCTCCTCCCATACTTTTGTTTTGGTCAGCCTGCACCTT
SRR511914.44813836.2+   TCAAGGGTATGCTCTTCCTCTTGCTCCTCCCATACTTTTGTTTTGGTCAGCCTGCCCCTT
SRR511914.75736196.2+   TCAAGGGTATGCTCTTCCTCTTGCTCCTCCCATACTTTTGTTTTGGTCAGCCTGCACCTT
SRR511914.83943589.1-   TCAAGGGTATGCTCTTCCTCTTGCTCCTCCCATACTTTTGTTTTGGTCAGCCTGCACCTT
SRR511914.85292277.1+   TCAAGGGTATGCTCTTCCTCTTGCTCCTCCCATACTTTTGTTTTGGTCAGCCTGCACCTT
SRR511915.12387403.1+   TCAAGGGTATGCTCTTCCTCTTGCTCCTCCCATACTTTTGTTTTGGTCAGCCTGCACCTT
SRR511915.2208112.2-   TCAAGGGTATGCTCTTCCTCTTGCTCCTCCCATACTTTTGTTTTGGTCAGCCTGCACCTT
SRR511915.2208399.2-   TCAAGGGTATGCTCTTCCTCTTGCTCCTCCCATACTTTTGTTTTGGTCAGCCTGCACCTT
SRR511915.50178675.2-   TCAAGGGTATGCTCTTCCTCTTGCTCCTCCCATACTTTTGTTTTGGTCAGCCTGCACCTT
SRR511915.68961737.2+   TCAAGGGTATGCTCTTCCTCTTGCTCCTCCCATACTTTTGTTTTGGTCAGCCTGCACCTT
SRR511915.72842805.1+   TCAAGGGTATGCTCTTCCTCTTGCTCCTCCCATACTTTTGTTTTGGTCAGCCTGCACCTT
SRR516967.114035844.1+   TCAAGGGTATGCTCTTCCTCTTGCTCCTCCCATACTTTTGTTTTGGTCAGCCTGCACCTT
SRR516967.119505550.2-   TCAAGGGTATGCTCTTCCTCTTGCTCCTCCCATACTTTTGTTTTGGTCAGCCTGCACCTT
SRR516967.128996446.2+   TCAAGGGTATGCTCTTCCTCTTGCTCCTCCCATACTTTTGTTTTGGTCAGCCTGCACCTT
SRR516967.140688955.1+   TCAAGGGTATGCTCTTCCTCTTGCTCCTCCCATACTTTTGTTTTGGTCAGCCTGCACCTT
SRR516967.143090323.1-   TCAAGGGTATGCTCTTCCTCTTGCTCCTCCCATACTTTTGTTTTGGTCAGCCTGCACCTT
SRR516967.22260303.1+   TCAAGGGTATGCTCTTCCTCTTGCTCCTCCCATACTTTTGTTTTGGTCAGCCTGCACCTT
SRR516967.23066803.1+   TCAAGGGTATGCTCTTCCTCTTGCTCCTCCCATACTTTTGTTTTGGTCAGCCTGCACCTT
SRR516967.25613572.1-   TCAAGGGTATGCTCTTCCTCTTGCTCCTCCCATACTTTTGTTTTGGTCAGCCTGCACCTT
SRR516967.30937619.1+   TCAAGGGTATGCTCTTCCTCTTGCTCCTCCCATACTTTTGTTTTGGTCAGCCTGCACCTT
SRR516967.3860522.2+   TCAAGGGTATGCTCTTCCTCTTGCTCCTCCCATACTTTTGTTTTGGTCAGCCTGCACCTT
SRR516967.41353207.1-   TCAAGGGTATGCTCTTCCTCTTGCTCCTCCCATACTTTTGTTTTGGTCAGCCTGCACCTT
SRR516967.71626809.2+   TCANGGGTATGCTCTTCCTCTTGCTCCTCCCATACTTTTGTTTTGGTCAGCCTGCACCTT
SRR516967.72187734.1+   TCAAGGGTATGCTCTTCCTCTTGCTCNTCCCATACTTTTGTTTTGGTCAGCCTGCACCTT
SRR516967.88384226.2+   TCAAGGGTATGCTCTTCCTCTTGCTCCTCCCATACTTTTGTTTTGGTCAGCCTGCACCTT
SRR516967.99920687.2-   TCAAGGGTATGCTCTTCCTCTTGCTCCTCCCATACTTTTGTTTTGGTCAGCCTGCACCTT
SRR516968.108115361.1+   TCAAGGGTATGCTCTTCCTCTTGCTCCTCCCATACTTTTGTTTTGGTCAGCCTGCACCTT
SRR516968.111104702.2-   TCAAGGGTATGCTCTTCCTCTTGCTCCTCCCATACTTTTGTTTTGGTCAGCCTGCACCTT
SRR516968.114845186.2-   TCAAGGGTATGCTCTTCCTCTTGCTCCTCCCATACTTTTGTTTTGGTCAGCCTGCACCTT
SRR516968.128087344.1+   TCAAGGGTATGCTCTTCCTCTTGCTCCTCCCATACTTTTGTTTTGGTCAGCCTGCACCTT
SRR516968.155018007.1-   TCAAGGGTATGCTCTTCCTCTTGCTCCTCCCATACTTGTGTTTTGGTCAGCCTGCACCTT
SRR516968.165290408.2-   TCAAGGGTATGCTCTTCCTCTTGCTCCTCCCATACTTTTGTTTTGGTCAGCCTGCACCTT
SRR516968.31712935.1-   TCAAGGGTATGCTCTTCCTCTTGCTCCTCCCATACTTTTGTTTTGGTCAGCCTGCACCTT
SRR516968.37738736.1-   TCAAGGGTATGCTCTTCCTCTTGCTCCTCCCATACTTTTGTTTTGGTCAGCCTGCACCTT
SRR516968.53031658.1+   TCAAGGGTATGCTCTTCCTCTTGCTCCTCCCATACTTTTGTTTTTGTCAGCCTGCACCTT
SRR516968.73747636.2+   TCAAGGGTATGCTCTTCCTCTTGCTCCTCCCATACTTTTGTTTTGGTCAGCCTGCACCTT
SRR516968.75435688.2+   TCAAGGGTATGCTCTTCCTCTTGCTCCTCCCATACTTTTGTTTTGGTCAGCCTGCACCTT
SRR516968.80862039.2-   TCAAGGGTCTGCTCTTCCTCTTGCTCCTCCCATACTTTTGTTTTGGTCAGCCTGCACCTT
SRR516969.1709162.1-   TCAAGGGTATGCTCTTCCTCTTGCTCCTCCCATACTTTTGTTTTGGTCAGCCTGCACCTT
SRR516969.1737591.2+   TCAAGGGGATGCTCCTCCTCTTGCTCCCCCCATACTTTTGTTTTGGTCAGCCTGCACCTT
SRR516969.33135240.1+   TCAAGGGTATGCTCTTCCTCTTGCTCCTCCCATACTTTTGTTTTGGTCAGCCTGCACCTT
SRR516969.867824.1+   TCAAGGGTATGCCCTTCCTCTTGCTCCTCCCATACTTTTGTTTTGGTCAGCCTGCACCTT
SRR516970.17885639.1-   TCAAGGGTATGCTCTTCCTCTTGCTCCTCCCATACTTTTGTTTTGGTCAGCCTGCACCTT
SRR516970.26934.1+   TCAAGGGTATGCTCTTCCTCTTCCTCCTCCCATACTTTTGCTTTGGTCAGCCTGCACCTT
SRR516970.33098467.1-   TCAAGGGTATGCTCTTCCTCTTGCTCCTCCCATCCTTTTGTTTTGGTCAGCCTGCCCCTT
SRR516970.33098640.1-   TCAAGGGTATGCTCTTCCTCTTGCTCCTCCCATACTTTTGTTTTGGTCAGCCTGCACCTT
SRR516970.37367691.1+   TCAAGGGTATGCTCTTCCTCTTGCTCCTCCCATACTTTTGTTTTGGTCAGCCTGCACCTT
SRR516971.1738878.1-   TCAAGGGTATGCTCTTCCTCTTGCTCCTCCCATACTTTTGTTTTGGTCAGCCTGCACCTT
SRR516971.34030929.1-   TCAAGGGTATGCTCTTCCTCTTGCTCCTCCCATACTTTTGTTTTGGTCAGCCTGCACCTT
SRR516971.4236129.1-   TCAAGGGTATGCTCTTCCTCTTGCTCCTCCCATACTTTTGTTTTGGTCAGCCTGCACCTT
SRR516971.6695405.1+   TCAAGGGTATGCTCTTCCTCTTGCTCCTCCCATACTTTTGTTTTGGTCAGCCTGCACCTT
SRR516971.7392543.1+   TCAAGGGTATGCTCTTCCTCTTGCTCCTCCCATACTTTTGTTTTGGTCAGCCTGCACCTT

SRR516972.30802147.2-   TCAAGGGTATGCTCTTCCTCTTGCTCCTCCCATACTTTTGTTTTGGTCAGCCTGCACCTT
SRR516972.31320955.1+   TCAAGGGTATGCTCTTCCTCTTGCTCCTCCCATACTTTTGTTTTGGGCAGCCTGCACCTT
SRR516972.48121654.2-   TCAAGGGTATGCTCTTCCTCTTGCTCCTCCCATACTTTTGTTTTGGTCAGCCTGCACCTT
SRR516972.53472127.1-   TCAAGGGTATGCTCTTCCTCTTGCTCCTCCCATACTTTTGTTTTGGTCAGCCTGCACCTT
SRR516972.60791475.1+   TCAAGGGTATGCTCTTCCTCTTGCTCCTCCCATACTTTTGTTTTGGTCAGCCTGCACCTT
SRR516972.67116381.2-   TCAAGGGTATGCTCTTCCTCTTGCTCCTCCCATACTTTTGTTTTGGTCAGCCTGCACCTT
SRR516972.77543660.1+   TCAAGGGTATGCTCTTCCTCTTGCTCCTCCCATACTTTTGTTTTGGTCAGCCTGCACCTT
SRR516973.23808767.1-   TCAAGGGTATGCTCTTCCTCTTGCTCCTCCCATACTTTTGTTTTGGTCAGCCTGCACCTT
SRR516973.23808771.1-   TCAAGGGTATGCTCTTCCTCTTGCTCCTCCCATACTTTTGTTTTGGTCAGCCTGCACCTT
SRR516973.25479726.2+   TCAAGGGTATGCTCTTCCTCTTGCTCCTCCCATACTTTTGTTTTGGTCAGCCTGCACCTT
SRR516974.1759552.2+   TCAAGGGTATGCTCTTCCTCTTGCTCCTCCCATACTTTTGTTTTGGTCAGCCTGCACCTT
SRR516974.24221041.1-   TCAAGGGTATGCTCTTCCTCTTGCTCCTCCCATACTTTTGTTTTGGTCAGCCTGCACCTT
SRR516974.24280528.2-   TCAAGGGTATGCTCTTCCTCTTGCTCCTCCCATACTTTTGTTTTGGTCAGCCTGCACCTT
SRR516974.30867255.1+   TCCAGGGTATGCTCTTCCTCTTGCTCCTCCCATACTTTTGTTTTGGTCAGCCTGCACCTT
SRR516974.33187826.2-   TCAAGGGTATGCTCTTCCTCTTGCTCCTCCCATACTTTTGTTTTGGTCAGCCTGCACCTT
SRR516974.48843984.2-   TCAAGGGTATGCTCTTCCTCTTGCTCCTCCCATACTTTTGTTTTGGTCAGCCTGCACCTT
SRR516974.66712393.1+   TCAAGGGTATGCTCTTCCTCTTGCTCCTCCCATACTTTTGTTTTGGTCAGCCTGCACCTT
SRR516975.39272850.2-   TCAAGGGTATGCTCTTCCTCTTGCTCCTCCCATACTTTTGTTTTGGTCAGCCTGCACCTT
SRR516975.51826312.2-   TCAAGGGTATGCTCTTCCTCTTGCTCCTCCCATACTTTTGTTTTGGTCAGCCTGCACCTT
SRR516976.22199380.2+   TCAAGGGTATGCTCTTCCTCTTGCTACTCCCCTACTTTTGTTTTTGTACGCCTGCACCTT
SRR516977.2738721.1-   TCAAGGGTATGCTCTTCCTCTTGCTCCTCCCATACTTTTGTTTTGGTCAGCCTGCACCTT
SRR516977.3418701.1+   TCAAGGGTATGCTCTTCCTCTTGCTCCTCCCATACTTTTGTTTTGGTCAGCCTGCACCTT
SRR516977.65941636.2-   TCAAGGGTATGCTCTTCCTCTTGCTCCTCCCATACTTTTGTTTTGGTCAGCCTGCACCTT
SRR516977.71210552.2-   TCAAGGGTATGCTCTTCCTCTTGCTCCTCCCATACTTTTGTTTTGGTCAGCCTGCACCTT
SRR516978.14547831.1+   TCAAGGGTATGCTCTTCCTCTTGCTCCTCCCATACTTTTGTTTTGGTCAGCCTGCACCTT
SRR516978.44746730.2+   TCAAGGGTATGCTCTTCCTCTTGCTCCTCCCATACTTTTGTTTTGGTCAGCCTGCACCTT
SRR516978.55364120.2-   TCAAGGGTATGCTCTTCCTCTTGCTCCTCCCATACTTTTGTTTTGGTCAGCCTGCACCTT
SRR516978.60261999.1-   TCAAGGGTATGCTCTTCCTCTTGCTCCTCCCATACTTTTGTTTTGGTCAGCCTGCACCTT
SRR516978.69371288.1-   TCAAGGGTATGCTCTTCCTCTTGCTCCTCCCATACTTTTGTTTTGGTCAGCCTGCACCTT
SRR516978.70829906.1+   TCAAGGGTATGCTCTTCCTCTTGCTCCTCCCATACTTTTGTTTTGGTCAGCCTGCACCTT
SRR516979.27449937.1+   TCAAGGGTATGCTCTTCCTCTTGCTCCTCCCATACTTTTGTTTTGGTCAGCCTGCACCTT
SRR516979.30065340.2+   TCAAGGGTATGCTCTTCCTCTTGCTCCTCCCATACTTTTGTTTTGGTCAGCCTGCACCTT
SRR516979.7087082.1-   TCAAGGGTATGCTCTTCCTCTTGCTCCTCCCATACTTTTGTTTTGGTCAGCCTGCACCTT
SRR516980.49332165.2+   TCAAGGGTATGCTCTTCCTCTTGCTCCTCCCATACTTTTGTTTTGGTCAGCCTGCACCTT
SRR516981.16534572.1+   TCAAGGGTATGCTCTTCCTCTTGCTCCTCCCATACTTTTGTTTTGGTCAGCCTGCACCTT
SRR516981.26581776.2+   TCAAGGGTATGCTCTTCCTCTTGCTCCTCCCATACTTTTGTTTTGGTCAGCCTGCACCTT
SRR516981.34446750.1-   TCAAGGGTATGCTCTTCCTCTTGCTCCTCCCATACTTTTGTTTTGGTCAGCCTGCACCTT
SRR516981.43172628.1-   TCAAGGGTATGCTCTTCCTCTTGCTCCTCCCATACTTTTGTTTTGGTCAGCCTGCACCTT
SRR516981.56879022.2+   TCAAGGGTATGCTCTTCCTCTTGCTCCTCCCATACTTTTGTTTTGGTCAGCCTGCACCTT
SRR516981.69540153.2-   TCAAGGGTATGCTCTTCCTCTTGCTCCTCCCATACTTTTGTTTTGGTCAGCCTGCACCTT
SRR516982.49581501.1-   TCAAGGGTATGCTCTTCCTCTTGCTCCTCCCATACTTTTGTTTTGGTCAGCCTGCACCTT
SRR516983.18957782.2+   TCAAGGGGATGCTCTTCCTCTTGCTCCTCCCATACTTTTGTTTTGGTCAGCCTGCACCTT
SRR516983.2660585.2+   TCAAGGGTATGCTCTTCCTCTTGCTCCTCCCATACTTTTGTTTTGATCAGCCTGCACCTT
SRR516983.34256945.1+   TCAAGGGTATGCTCTTCCTCTTGCTCCTCCCATACTTTTGTTTTGGTCAGCCTGCACCTT
SRR516983.35103124.2+   TCAAGGGTATGCTCTTCCTCTTGCTCCTCCCATACTTTTGTTTTGGTCAGCCTGCACCTT
SRR516984.11603344.2+   TCAAGGGTATGCTCTTCCTCTTGCTCCTCCCATACTTTTGTTTTGGTCAGCCTGCACCGT
SRR516984.13867668.1-   TCAAGGGTATGCTCTTCCTCTTGCTCCTCCCATACTTTTGTTTTGGTCAGCCTGCACCTT
SRR516984.26297997.1+   TCAAGGGTATGCTCTTCCTCTTGCTCCTCCCATACTTTTGTTTTGGTCAGCCTGCACCTT
SRR516984.51860923.2+   TCAAGGGTATGCTCTTCCTCTTGCTCCTCCCATACTTTTGTTTTGGTCAGCCTGCACCTT
SRR516984.67024304.1-   TCAAGGGTATGCTCTTCCTCTTGCTCCTCCCATACTTTTGTTTTGGTCAGCCTGCACCTT
SRR516984.6951606.2+   TCAAGGGTATGCTCTTCCTCTTGCTCCTCCCATACTTTTGTTTTGGTCAGCCTGCACCTT
SRR516984.7283570.1+   TCAAGGGTATGCTCTTCCTCTTGCTCCTCCCATACTTTTGTTTTGGTCAGCCTGCACCTT
SRR516985.38800584.1+   TCAAGGGTATGCTCTTCCTCTTGCTCCTCCCATACTTTTGTTTTGGTCAGCCTGCACCTT
SRR516985.6369730.2-   TCAAGGGTATGCTCTTCCTCTTGCTCCTCCCATACTTTTGTTTTGGTCAGCCTGCACCTT
SRR516985.70870904.1-   TCAAGGGTATGCTCTTCCTCTTGCTCCTCCCATACTTTTGTTTTGGTCAGCCTGCACCTT
SRR516985.71587022.2-   TCAAGGGTATGCTCTTCCTCTTGCTCCTCCCATACTTTTGTTTTGGTCAGCCTGCACCTT
SRR516985.794078.1+   TCAAGGGTATGCTCTTCCTCTTGCTCCTCCCATACTTTTGTTTTGGTCAGCCTGCACCTT
SRR516985.84026477.1+   TCAAGGGTATGCTCTTCCTCTTGCTCCTCCCATACTTTTGTTTTGGTCAGCCTGCACCTT
SRR516985.90328884.2+   TCAAGGGTATGCTCTTCCTCTTGCTCCTCCCATACTTTTGTTTTGGTCAGCCTGCACCTT
SRR516985.95011897.2+   TCAAGGGTATGCTCTTCCTCTTGCTCCTCCCATACTTTTGTTTTGGTCAGCCTGCACCTT
SRR516986.10651932.2+   TCAAGGGTATGCTCTTCCTCTTGCTCCTCCCATACTTTTGTTTTGGTCAGCCTGCACCTT
SRR516986.17707572.1-   TCAAGGGTATGCTCTTCCTCTTGCTCCTCCCATACTTTTGTTTTGGTCAGCCTGCACCTT
SRR516986.22724390.1-   TCAAGGGTATGCTCTTCCTCTTGCTCCTCCCATACTTTTGTTTTGGTCAGCCTGCACCTT
SRR516987.86762093.1-   TCAAGGGTATGCTCTTCCTCTTGCTCCTCCCATACTTTTGTTTTGGTCAGCCTGCACCTT
SRR516988.4949152.1-   TCAAGGGTATGCTCTTCCTCTTGCTCCTCCCATACTTTTGTTTTGGTCAGCCTGCACCTT
SRR516988.60003491.2-   TCAAGGGTATGCTCTTCCTCTTGCTCCTCCCATACTTTTGTTTTGGTCAGCCTGCACCTT
SRR516989.56916901.2+   TCAAGGGTATGCTCTTCCTCTTGCTCCTCCCATACTTTTGTTTTGGTCAGCCTGCACCTT
SRR516989.80624643.2+   TCAAGGGTATGCTCTTCCTCTTGCTCCTCCCATACTTTTGTTTTGGTCAGCCTGCACCTT
SRR516990.23106415.2+   TCAAGGGTATGCTCTTCCTCTTGCTCCTCCCATACTTTTGTTTTGGTCAGCCTGCACCTT
SRR516990.30495340.2+   TCAAGGGTATGCTCTTCCTCTTGCTCCTCCCATACTTTTGTTTTGGTCAGCCTGCACCTT
SRR516990.36711687.2-   TCAAGGGTATGCTCTTCCTCTTGCTCCTCCCATACTTTTGTTTTGGTCAGCCTGCACCTT
SRR516990.46955085.1+   TCAAGGGTATGCTCTTCCTCTTGCTCCTCCCATACTTTTGTTTTGGTCAGCCTGCACCTT
SRR516991.40998329.1-   TCAAGGGTATGCTCTTCCTCTTGCTCCTCCCATACTTTTGTTTTGGTCAGCCTGCACCTT
SRR516991.52505003.2-   TCAAGGGTATGCTCTTCCTCTTGCTCCTCCCATACTTTTGTTTTGGTCAGCCTGCACCTT
SRR516991.54630322.1-   TCAAGGGTATGCTCTTCCTCTTGCTCCTCCCATACTTTTGTTTTGGTCAGCCTGCACCTT
SRR516991.63425507.1+   TCAAGGGTATGCTCTTCCTCTTGCTCCTCCCATACTTTTGTTTTGGTCAGCCTGCACCTT
SRR516991.64722198.2-   TCAAGGGTATGCTCTTCCTCTTGCTCCTCCCATACTTTTGTTTTGGTCAGCCTGCACCTT
SRR516992.42779638.1-   TCAAGGGTATGCTCTTCCTCTTGCTCCTCCCATACTTTTGTTTTGGTCAGCCTGCACCTT
SRR516992.59812008.2-   TCAAGGGTATGCTCTTCCTCTTGCTCCTCCCATACTTTTGTTTTGGTCAGCCTGCACCTT
SRR516992.60599956.2-   TCAAGGGTATGCTCTTCCTCTTGCTCCTCCCATACTTTTGTTTTGGTCAGCCTGCACCTT
SRR516992.65303336.1+   TCAAGGGTATGCTCTTCCTCTTGCTCCTCCCATACTTTTGTTTTGGTCAGCCTGCACCTT
SRR516992.78710121.1+   TCAAGGGTATGCTCTTCCTCTTGCTCCTCCCATACTTTTGTTTTGGTCAGCCTGCACCTT
SRR516993.14043843.2-   TCAAGGGTATGCTCTTCCTCTTGCTCCTCCCATACTTTTGTTTTGGTCAGCCTGCACCTT
SRR516993.19338558.2+   TCAAGGGTATGCTCTTCCTCTTGCTCCTCCCATACTTTTGTTTTGGTCAGCCTGCACCTT

```
SRR516993.35664507.1-    TCAAGGGTATGCTCTTCCTCTTGCTCCTCCCATACTTTTGTTTTGGTCAGCCTGCACCTT
SRR516993.47859690.2-    TCAAGGGTATGCTCTTCCTCTTGCTCCTCCCATACTTTTGTTTTGGTCAGCCTGCACCTT
SRR516993.77981446.2+    TCAAGGGTATGCTCTTCCTCTTGCTCCTCCCATACTTTTGTTTTGGTCAGCCTGCACCTT
SRR516994.13045831.2+    TCAAGGGTATGCTCTTCCTCTTGCTCCTCCCATACTTTTGTTTTGGTCAGCCTGCACCTT
SRR516994.13973863.1+    TCAAGGGTATGCTCTTCCTCTTGCTCCTCCCATACTTTTGTTTTGGTCAGCCTGCACCTT
SRR516994.22273890.1+    TCAAGGGTATGCTCTTCCTCTTGCTCCTCCCATACTTTTGTTTTGGTCAGCCTGCACCTT
SRR516995.16662105.2-    TCAAGGGTATGCTCTTCCTCTTGCTCCTCCCATACTTTTGTTTTGGTCAGCCTGCACCTT
SRR516995.20779522.2-    TCAAGGGTATGCTCTTCCTCTTGCTCCTCCCATGCTTTTGTTTTGGTCAGCCTGCCCCTT
SRR516995.27817063.1+    TCAAGGGTATGCTCTTCCTCTTGCTCCTCCCATACTTTTGTTTTGGTCAGCCTGCACCTT
SRR516995.4091683.2+     TCAAGGGTATGCTCTTCCTCTTGCTCCTCCCATACTTTTGTTTTGGTCAGCCTGCACCTT
SRR516996.25148019.2+    TCAAGGGTATGCTCTTCCTCTTGCTCCTCCCATACTTTTGTTTTGGTCAGCCTGCACCTT
SRR516996.345191.1+      TCAAGGGTATGCTCTTCCTCTTGCTCCTCCCATACTTTTGTTTTGGTCAGCCTGCACCTT
SRR516996.40869314.1+    TCAAGGGTATGCTCTTCCTCTTGCTCCTCCCATACTTTTGTTTTGGTCAGCCTGCACCTT
SRR516996.41067957.1-    TCAAGGGTATGCTCTTCCTCTTGCTCCTCCCATACTTTTGTTTTGGTCAGCCTGCACCTT
SRR516996.47443095.1+    TCAAGGGTATGCTCTTCCTCTTGCTCCTCCCATACTTTTGTTTTGGTCAGCCTGCACCTT
SRR516996.4820423.1-     TCAAGGGTATGCTCTTCCTCTTGCTCCTCCCATACTTTTGTTTTGGTCAGCCTGCACCTT
SRR516996.64162159.1-    TCAAGGGTATGCTCTTCCTCTTGCTCCTCCCATACTTTTGTTTTGGTCAGCCTGCACCTT
SRR516996.84831988.2+    TCAAGGGTATGCTCTTCCTCTTGCTCCTCCCATACTTTTGTTTTGGTCAGCCTGCACCTT
SRR516996.96865981.1-    TCAAGGGTATGCTCTTCCTCTTGCTCCTCCCATACTTTTGTTTTGGTCAGCCTGCACCTT
SRR516997.12935966.2-    TCAAGGGTATGCTCTTCCTCTTGCTCCTCCCATACTTTTGTTTTGGTCAGCCTGCACCTT
SRR516997.32779052.2+    TCAAGGGTATGCTCTTCCTCTTGCTCCTCCCATACTTTTGTTTTGGTCAGCCTGCACCTT
SRR516997.49184054.2+    TCAAGGGTATGCTCTTCCTCTTGCTCCTCCCATACTTTTGTTTTGGTCAGCCTGCACCTT
SRR516997.72118786.2+    TCAAGGGTATGCTCTTCCTCTTGCTCCTCCCATACTTTTGTTTTGGTCAGCCTGCACCTT
SRR516997.73355617.2+    TCAAGGGTATGCTCTTCCTCTTGCTCCTCCCATACTTTTGTTTTGGTCAGCCTGCACCTT
SRR516998.27579449.2+    TCAAGGGTATGCTCTTCCTCTTGCTCCTCCCATACTTTTGTTTTGGTCAGCCTGCACCTT
SRR516998.40607410.2-    TCAAGGGTATGCTCTTCCTCTTGCTCCTCCCATACTTTTGTTTTGGTCAGCCTGCACCTT
SRR516998.87531343.2-    TCAAGGGTATGCTCTTCCTCTTGCTCCTCCCATACTTTTGTTTTGGTCAGCCTGCACCTT
SRR516998.89996681.2+    TCAAGGGTATGCTCTTCCTCTTGCTCCTCCCATACTTTTGTTTTGGTCAGCCTGCACCTT
SRR516998.95088687.1-    TCAAGGGTATGCTCTTCCTCTTGCTCCTCCCATACTTTTGTTTTGGTCAGCCTGCACCTT
SRR516999.32513950.1-    TCAAGGGTATGCTCTTCCTCTTGCTCCTCCCATACTTTTGTTTTGGTCAGCCTGCACCTT
SRR516999.38940100.1+    TCAAGGGTATGCTCTTCCTCTTGCTCCTCCCATACTTTTGTTTTGGTCAGCCTGCACCTT
SRR516999.40240407.2+    TCAAGGGTATGCTCTTCCTCTTGCTCCTCCCATACTTTTGTTTTGGTCAGCCTGCACCTT
SRR516999.41559143.1-    TCAAGGGTATGCTCTTCCTCTTGCTCCTCCCATACTTTTGTTTTGGTCAGCCTGCACCTT
SRR516999.42713432.1+    TCAAGGGTATGCTCTTCCTCTTGCTCCTCCCATACTTTTGTTTTGGTCAGCCTGCACCTT
SRR516999.45267731.1+    TCAAGGGTATGCTCTTCCTCTTGCTCCTCCCATACTTTTGTTTTGGTCAGCCTGCACCTT
SRR516999.47140010.1+    TCAAGGGTATGCTCTTCCTCTTGCTCCTCCCATACTTTTGTTTTGGTCAGCCTGCACCTT
SRR516999.51381955.2-    TCAAGGGTATGCTCTTCCTCTTGCTCCTCCCATACTTTTGTTTTGGTCAGCCTGCACCTT
SRR516999.58849034.2-    TCAAGGGTATGCTCTTCCTCTTGCTCCTCCCATACTTTTGTTTTGGTCAGCCTGCCCCTT
SRR516999.68726039.1-    TCAAGGGTATGCTCTTCCTCTTGCTCCTCCCATACTTTTGTTTTGGTCAGCCTGCACCTT
SRR516999.6894145.1-     TCAAGGGTATGCTCTTCCTCTTGCTCCTCCCATACTTTTGTTTTGGTCAGCCTGCACCTT
SRR516999.76775311.2+    TCAAGGGTATGCTCTTCCTCTTGCTCCTCCCATACTTTTGTTTTGGTCAGCCTGCACCTT
SRR517000.44752129.1-    TCAAGGGTATGCTCTTCCTCTTGCTCCTCCCATACTTTTGTTTTGGTCAGCCTGCACCTT
SRR517000.59906460.1+    TCAAGGGTATGCTCTTCCTCTTGCTCCTCCCATACTTTTGTTTTGGTCAGCCTGCACCTT
SRR517000.67695666.1-    TCAAGGGTATGCTCTTCCTCTTGCTCCTCCCATACTTTTGTTTTGGTCAGCCTGCACCTT
SRR517000.83969593.2-    TCAAGGGTATGCTCTTCCTCTTGCTCCTCCCATACTTTTGTTTTGGTCAGCCTGCACCTT
SRR517000.95262135.1-    TCAAGGGTATGCTCTTCCTCTTGCTCCTCCCATACTTTTGTTTTGGTCAGCCTGCACCTT
SRR517001.13478608.1+    TCAAGGGTATGCTCTTCCTCTTGCTCCTCCCATACTTTTGTTTTGGTCAGCCTGCACCTT
SRR517001.43143516.2+    TCAAGGGTATGCTCTTCCTCTTGCTCCTCCCATACTTTTGTTTTGGTCAGCCTGCACCTT
SRR517001.50757283.1-    TCAAGGGTATGCTCTTCCTCTTGCTCCTCCCATACTTTTGTTTTGGTCAGCCTGCACCTT
SRR517002.25042325.1+    TCAAGGGCATGCTCTTCCTCTTGCTCCTCCCATACTTTTGTTTTGGTCAGCCTGCACCTT
SRR517002.26060078.2-    TCAAGGGTATGCTCTTCCTCTTGCTCCTCCCATACTTTTGTTTTGGTCAGCCTGCACCTT
SRR517002.67211818.1-    TCAAGGGTATGCTCTTCCTCTTGCTCCTCCCATACTTTTGTTTTGGTCAGCCTGCACCTT
SRR517002.81950718.1+    TCAAGGGTATGCTCTTCCTCTTGCTCCTCCCATACTTTTGTTTTGGTCAGCCTGCACCTT
SRR517002.82258575.2-    TCAAGGGTATGCTCTTCCTCTTGCTCCTCCCATACTTTTGTTTTGGTCAGCCTGCACCTT
SRR517002.91703218.2+    TCAAGGGTATGCTCTTCCTCTTGCTCCTCCCATACTTTTGTTTTGGTCAGCCTGCACCTT
SRR517003.19342168.1+    TCAAGGGTATGCTCTTCCTCTTGCTCCTCCCATACTTTTGTTTTGGTCAGCCTGCACCTT
SRR517003.20939478.2-    TCAAGGGTATGCTCTTCCTCTTGCTCCTCCCATACTTTTGTTTTGGTCAGCCTGCACCTT
SRR517003.5667689.1-     TCAAGGGTATGCTCTTCCTCTTGCTCCTCCCATACTTTTGTTTTGGTCAGCCTGCACCTT
SRR517004.28032599.1-    TCAAGGGTATGCTCTTCCTCTTGCTCCTCCCATACTTTTGTTTTGGTCAGCCTGCACCTT
SRR517005.42320821.2-    TCAAGGGTATGCTCTTCCTCTTGCTCCTCCCATACTTTTGTTTTGGTCAGCCTGCACCTT
SRR517006.4335776.2-     TCAAGGGTATGCTCTTCCTCTTGCTCCTCCCATACTTTTGTTTTGGTCAGCCTGCACCTT
SRR517006.52685296.2-    TCAAGGGTATGCTCTTCCTCTTGCTCCTCCCATACTTTTGTTTTGGTCAGCCTGCACCTT
SRR517006.64062576.1-    TCAAGGGTATGCTCTTCCTCTTGCTCCTCCCATACTTTTGTTTTGGTCAGCCTGCACCTT
SRR517006.75846793.2+    TCAAGGGTATGCTCTTCCTCTTGCTCCTCCCATACTTTTGTTTTGGTCAGCCTGCACCTT
SRR517006.82333459.1-    TCAAGGGTATGCTCTTCCTCTTGCTCCTCCCATACTTTTGTTTTGGTCAGCCTGCACCTT
SRR517006.8320014.2+     TCAAGGGTATGCTCTTCCTCTTGCTCCTCCCATACTTTTGTTTTGGTCAGCCTGCACCTT
SRR517007.25883181.1+    TCAAGGGTATGCTCTTCCTCTTGCTCCTCCCATACTTTTGTTTTGGTCAGCCTGCACCTT
SRR517007.47743023.1-    TCAAGGGTATGCTCTTCCTCTTGCTCCTCCCATACTTTTGTTTTGGTCAGCCTGCACCTT
SRR517007.50962620.2+    TCAAGGGTATGCTCTTCCTCTTGCTCCTCCCATACTTTTGTTTTGGTCAGCCTGCACCTT
SRR517007.51506783.2+    TCAAGGGTATGCTCTTCCTCTTGCTCCTCCCATACTTTTGTTTTGGTCAGCCTGCACCTT
SRR517007.60890697.2+    TCAAGGGTATGCTCTTCCTCTTGCTCCTCCCATACTTTTGTTTTGGTCAGCCTGCACCTT
SRR517008.31099343.2-    TCAAGGGTATGCTCTTCCTCTTGCTCCTCCCATACTTTTGTTTTGGTCAGCCTGCACCTT
SRR517008.35456369.2+    TCAAGGGTATGCTCTTCCTCTTGCTCCTCCCATACTTTTGTTTTGGTCAGCCTGCACCTT
SRR517008.67351581.2+    TCAAGGGTATGCTCTTCCTCTTGCTCCTCCCATCCTTTTGTTTTGGTCAGCCTGCACCTT
SRR517008.8977763.1-     TCAAGGGTATGCTCTTCCTCTTGCTCCTCCCATACTTTTGTTTTGGTCAGCCTGCACCTT
SRR517009.19164909.2-    TCAAGGGTATGCTCTTCCTCTTGCTCCTCCCATACTTTTGTTTTGGTCAGCCTGCACCTT
SRR517009.40247008.2+    TCAAGGGTATGCTCTTCCTCTTGCTCCTCCCATACTTTTGTTTTGGTCAGCCTGCACCTT
SRR517009.45770063.2+    TCAAGGGTATGCTCTTCCTCTTGCTCCTCCCATACTTTTGTTTTGGTCAGCCTGCACCTT
SRR517009.5972409.1-     TCAAGGGTATGCTCTTCCTCTTGCTCCTCCCATACTTTTGTTTTGGTCAGCCTGCACCTT
SRR517009.70549529.2+    TCAAGGGGATGCTCTTCCTCTTGCTCCTCCCATACTTTTGTTTTGGTCAGCCTGCACCTT
SRR511892.28064592.1+     CAAGGGTATGCTCTTCCTCTTGCTCCTCCCATACTTTTGTTTTGGTCAGCCTGCACCTT
SRR511892.41096124.1+     CAAGGGTATGCTCTTCCTCTTGCTCCTCCCATACTTTTGTTTTGGTCAGCCTGCACCTT
SRR516973.1417145.2-      CAAGGGTATGCTCTTCCTCTTGCTCCTCCCATACTTTTGTTTTGGTCAGCCTGCACCTT
SRR516973.1417146.2-      CAAGGGTATGCTCTTCCTCTTGCTCCTCCCATACTTTTGTTTTGGTCAGCCTGCACCTT
```

```
SRR516991.89756659.2-     CAAGGGTATGCTCTTCCTCTTGCTCCTCCCATACTTTTGTTTTGGTCAGCCTGCACCTT
SRR516992.7333033.1+      CAAGGGTATGCTCTTCCTCTTGCTCCTCCCATACTTTTGTTTTGGTCAGCCTGCACCTT
SRR516998.65411337.1+     CAAGGGTATGCTCTTCCTCTTGCTCCTCCCATACTTTTGTTTTGGTCAGCCTGCACCTT
SRR517006.59401473.1-     CAAGGGTATGCTCTTCCTCTTGCTCCTCCCATACTTTTGTTTTGGTCAGCCTGCACCTT
SRR516968.109900230.1+     AAGGGTATGCTCTTCCTCTTGCTCCTCCCATACTTTTGTTTTGGTCAGCCTGCACCTT
SRR516972.63489975.1+      AAGGGTATGCTCTTCCTCTTGCTCCTCCCATACTTTTGTTTTGGTCAGCCTGCACCTT
SRR516974.43659576.1+      AAGGGTATGCTCTTCCTCTTGCTCCTCCCATACTTTTGTTTTGGTCAGCCTGCACCTT
SRR516986.47033107.2+      AAGGGTATGCTCTTCCTCTTGCTCCTCCCATACTTTTGTTTTGGTCAGCCTGCACCTT
SRR516988.14405849.2+      AAGGGTATGCTCTTCCTCTTGCTCCTCCCATACTTTTGTTTTGGTCAGCCTGCACCTT
SRR516991.84387060.1+      AAGGGTATGCTCTTCCTCTTGCTCCTCCCATACTTTTGTTTTGGTCAGCCTGCACCTT
SRR516993.73666384.1+      AAGGGTATGCTCTTCCTCTTGCTCCTCCCATACTTTTGTTTTGGTCAGCCTGCACCTT
SRR516995.73341151.2+      AAGGGAATGCTCTTCCTCTTGCTCCTCCCATAGTTTGGTTTTGGACAGCCTGCAGCTT
SRR516996.17100141.2-      AAGGGTATGCTCTTCCTCTTGCTCCTCCCATACTTTTGTTTTGGTCAGCCTGCACCTT
SRR516998.784538.1+        AAGGGTATGCTCTTCCTCTTGCTCCTCCCATACTTTTGTTTTGGTCAGCCTGCACCTT
SRR517001.57642820.1+      AAGGGTATGCTCTTCCTCTTGCTCCTCCCATACTTTTGTTTTGGTCAGCCTGCACCTT
SRR517008.22652667.1+      AAGGGTATGCTCTTCCTCTTGCTCCTCCCATACTTTTGTTTTGGTCAGCCTGCACCTT
SRR511913.5587657.2+        AGGGTATGCTCTTCCTCTTGCTCCTCCCATACTTTTGTTTTGGTCAGCCTGCACCTT
SRR516967.113781662.1+      AGGGTATGCTCTTCCTCTTGCTCCTCCCATACTTTTGTTTTGGTCAGCCTGCACCTT
SRR516967.75977499.2-       AGGGTATGCTCTTCCTCTTGCTCCTCCCATACTTTTGTTTTGGTCAGCCTGCACCTT
SRR516968.13503998.1-       AGGGTATGCTCTTCCTCTTGCTCCTCCCATACTTTTGTTTTGGTCAGCCTGCACCTT
SRR516974.46129857.2-       AGGGTATGCTCTTCCTCTTGCTCCTCCCATACTTTTGTTTTGGTCAGCCTGCACCTT
SRR516992.26195657.2-       AGGGTATGCTCTTCCTCTTGCTCCTCCCATACTTTTGTTTTGGTCAGCCTGCACCTT
SRR516994.51970256.2-       AGGGTATGCTCTTCCTCTTGCTCCTCCCATACTTTTGTTTTGGTCAGCCTGCACCTT
SRR517002.87764725.1+       AGGGTATGCTCTTCCTCTTGCTCCTCCCATACTTTTGTTTCGGTCAGCCTGCACCTT
SRR517009.33285239.1+       AGGGTATGCTCTTCCTCTTGCTCCTCCCATACTTTTGTTTTGGTCAGCCTGCACCTT
SRR516979.72185467.1-        GGGTATGCTCTTCCTCTTGCTCCTCCCATACTTTTGTTTTGGTCAGCCTGCACCTT
SRR516995.50080094.1+        GGGTATGCTCTTCCTCTTGCTCCTCCCATACTTTTGTTTTGGTCAGCCTGCACCTT
SRR517006.37525580.1+        GGGTATGCTCTTCCTCTTGCTCCTCCCATACTTTTGTTTTGGTCAGCCTGCACCTT
SRR517009.46221980.2+        GGGTATGCTCTTCCTCTTGCTCCTCCCATACTTTTGTTTTGGTCAGCCTGCACCTT
SRR516967.692820.1+           GGTATGCTCTTCCTCTTGCTCCTCCCATACTTTTGTTTTGGTCAGCCTGCACCTT
SRR516970.33702206.1-         GGTATGCTCTTCCTCTTGCTCCTCCCATACTTTTGTTTTGGTCAGCCTGCACCTT
SRR516972.26613549.2+         GGTATGCTCTTCCTCTTGCTCCTCCCATACTTTTGTTTTGGTCAGCCTGCACCTT
SRR516973.19319985.2+         GGTATGCTCTTCCTCTTGCTCCTCCCATACTTTTGTTTTGGTCAGCCTGCACCTT
SRR516983.80722236.2+         GGTATGCTCTTCCTCTTGCTCCTCCCATACTTTTGTTTTGGTCAGCCTGCACCTT
SRR511892.39300111.2+          GTATGCTCTTCCTCTTGCTCCTCCCATACTTTTGTTTTGGTCAGCCTGCACCTT
SRR516967.143302175.2-         GTATGCTCTTCCTCTTGCTCCTCCCATACTTTTGTTTTGGTCAGCCTGCACCTT
SRR516967.40446194.1+          GTATGCTCTTCCTCTTGCTCCTCCCATACTTTTGTTTTGGTCACCCTGCCCCTT
SRR516975.16339360.1+          GTATGCTCTTCCTCTTGCTCCTCCCATACTTTTGTTTTGGTCAGCCTGCACCTT
SRR516993.45140548.2-          GTATGCTCTTCCTCTTGCTCCTCCCATACTTTTGTTTTGGTCAGCCTGCACCTT
SRR516996.39193734.1-          GTATGCTCTTCCTCTTGCTCCTCCCATACTTTTGTTTTGGTCAGCCTGCACCTT
SRR517008.15407290.1+          GTATGCTCTTCCTCTTGCTCCTCCCATACTTTTGTTTTGGTCAGCCTGCACCTT
SRR511914.74202240.2+           TATGCTCTTCCTCTTGCTCCTCCCATACTTTTGTTTTGGTCAGCCTGCACCTT
SRR511914.74202383.2+           TATGCTCTTCCTCTTGCTCCTCCCATACTTTTGTTTTGGTCAGCCTGCACCTT
SRR511915.77154079.1+           TATGCTCTTCCTCTTGCTCCTCCCATACTTTTGTTTTGGTCAGCCTGCACCTT
SRR516967.3323960.1+            TATGCTCTTCCTCTTGCTCCTCCCATACTTTTGTTTTGGTCAGCCTGCACCTT
SRR516973.17670450.1+           TATGCTCTTCCTCTTGCTCCTCCCATACTTTTGTTTTGGTCAGCCTGCACCTT
SRR516973.41289849.2+           TATGCTCTTCCTCTTGCTCCTCCCATACTTTTGTTTTGGTCAGCCTGCACCTT
SRR516981.50701184.1+           TATGCTCTTCCTCTTGCTCCTCCCATACTTTTGTTTTGGTCAGCCTGCACCTT
SRR516990.1457908.1-            TATGCTCTTCCTCTTGCTCCTCCCATACTTTTGTTTTGGTCAGCCTGCACCTT
SRR516995.57519330.1-           TATGCTCTTCCTCTTGCTCCTCCCATTCTTTTGTTTTGGTCAGCCTGCACCTT
SRR516998.95947533.1+           TATGCTCTTCCTCTTGCTCCTCCCATACTTTTGTTTTGGTCAGCCTGCACCTT
SRR517001.86378213.2-           TATGCTCTTCCTCTTGCTCCTCCCATACTTTTGTTTTGGTCAGCCTGCACCTT
SRR516967.51922488.1+            ATGCTCTTCCTCTTGCTCCTCCCATACTTTTGTTTTGGTCAGCCTGCACCTT
SRR516967.66315071.1+            ATGCTCTTCCTCTTGCTCCTCCCATACTTTTGTTTTGGTCAGCCTGCANCTT
SRR516982.12131511.1+            ATGCTCTTCCTCTTGCTCCTCCCATACTTTTGTTTTGGTCAGCCTGCACCTT
SRR516992.74240970.2+            ATGCTCTTCCTCTCGCTCCTCCCATACTTTTGTTTTGGTCAGCCTGCACCTT
SRR511916.38138441.2+             TGCTCTTCCTCTTGCTCCTCCCATACTTTTGTTTTGGTCAGCCTGCACC
SRR511917.60034213.2+             TGCTCTTCCTCTTGCTCCTCCCATACTTTTGTTTTGGTCAGCCTGCACC
SRR516971.33071473.1+             TGCTCTTCCTCTTGCTCCTCCCATACTTTTGTTTTGGTCAGCCTGCACCTT
SRR516978.27059321.2+             TGCTCTTCCTCTTGCTCCTCCCATACTTTTGTTTTGGTCAGCCTGCACCTT
SRR516983.33407963.2+             TGCTCTTCCTCTTGCTCCTCCCATACTTTTGTTTTGGTCAGCCTGCACCTT
SRR516984.90374479.1-             TGCTCTTCCTCTTGCTCCTCCCATACTTTTGTTTTGGTCAGCCTGCACCTT
SRR516991.17053852.2+             TGCTCTTCCTCTTGCTCCTCCCATACTTTTGTTTTGGTCAGCCTGCACCTT
SRR516997.61814545.2+             TGCTCTTCCTCGTGCTCCTCCCATACTTTTGTTTTGGTCAGCCTGCACCTT
SRR516998.100162957.2-            TGCTCTTCCTCTTGCTCCTCCCATACTTTTGTTTTGGTCAGCCTGCACCTT
SRR511912.43117119.2+              GCTCTTCCTCTTGCTCCTCCCATACTTTTGTTTTGGTCAGCCTGCACCTT
SRR511915.70587641.1-              GCTCTTCCTCTTGCTCCTCCCATACTTTTGTTTTGGTCAGCCTGCACCTT
SRR511915.70587944.1-              GCTCTTCCTCTTGCTCCTCCCATACTTTGGTTTTGGTCAGCCTGCACCTT
SRR516973.53561779.1-              GCTCTTCCTCTTGCTCCTCCCATACTTTTGTTTTGGTCAGCCTGCACCTT
SRR516978.47021888.1+              GCTCTTCCTCTTGCTCCTCCCATACTTTTGTTTTGGTCAGCCTGCACCTT
SRR516985.87014315.2-              GCTCTTCCTCTTGCTCCTCCCATACTTTTGTTTTGGTCAGCCTGCACCTT
SRR516986.24845211.1-              GCTCTTCCTCTTGCTCCTCCCATACTTTTGTTTTGGTCAGCCTGCACCTT
SRR516988.60315129.1-              GCTCTTCCTCTTGCTCCTCCCATACTTTTGTTTTGGTCAGCCTGCACCTT
SRR516999.52172306.2+              GCTCTTCCTCTTGCTCCTCCCATACTTTTGTTTTGGTCAGCCTGCACCTT
SRR517000.8953178.1+               GCTCTTCCTCTTGCTCCTCCCATACTTTTGTTTTGGTCAGCCTGCACCTT
SRR511892.65397717.2+               CTCTTCCTCTTGCTCCTCCCATACTTTTGTTTTGGTCAGCCTGCACCTT
SRR516989.72004672.2+               CTCTTCCTCTTGCTCCTCCCATACTTTTGTTTTGGTCAGCCTGCACCTT
SRR516998.14101174.1-               CTCTTCCTCTTTCTCCTCCCATACTTTTGTTTTGGTCAGCCTGCACCTT
SRR517000.44421459.2-               CTCTTCCTCTTGCTCCTCCCATACTTTTGTTTTGGTCAGCCTGCACCTT
SRR517009.47221478.1+               CTCTTCCTCTTGCTCCTCCCATACTTTTGTTTTGGTCAGCCTGCACCTT
SRR511892.37274156.1+                TCTTCCTCTTGCTCCTCCCATACTTTTGTTTTGGTCAGCCTGCACCTT
SRR511911.71659560.1-                TCTTCCTCTTGCTCCTCCCATACTTTTGTTTTGGTCAGCCTGCACCTT
SRR516971.18124229.1-                TCTTCCTCTTGCTCCTCCCATGCTTTTGTTTTGGTCAGCCTGCACCTT
SRR516972.23056341.2+                TCTTCCTCTTGCTCCTCCCATACTTTTGTTTTGGTCAGCCTGCACTTT
```

```
SRR516972.63004169.2+                   TCTTCCTCTTGCTCCTCCCATACTTTTGTTTTGGTCAGCCTGCACCTT
SRR516981.4191635.1+                    TCTTCCTCTTGCTCCTCCCATACTTTTGTTTTGGTCAGCCTGCACCTT
SRR516986.987228.1+                     TCTTCCTCTTGCTCCTCCCATACTTTTGTTTTGGTCAGCCTGCACCTT
SRR516987.3530565.1-                    TCTTCCTCTTGCTCCTCCCATACTTTTGTTTTGGTCAGCCTGCACCTT
SRR516990.47650251.1+                   TCTTCCTCTTGCTCCTCCCATACTTTTGTTTTGGTCAGCCTGCACCTT
SRR516975.38510117.1+                    CTTCCTCTTGCTCCTCCCATACTTTTGTTTTGGTCAGCCTGCACCTT
SRR516978.25674765.2+                    CTTCCTCTTGCTCCTCCCATACTTTTGTTTTGGTCAGCCTGCACCTT
SRR516983.15102679.2+                    CTTCCTCTTGCTCCTCCCATACTTTTGTTTTGGTCAGCCTGCACCTT
SRR516994.34198741.2-                    CTTCCTCTTGCTCCTCCCATACTTTTGTTTTGGTCAGCCTGCACCTT
SRR516997.43562335.1+                    CTTCCTCTTGCTCCTCCCATACTTTTGTTTTGGTCAGCCTGCACCTT
SRR516997.84310008.2+                    CTTCCTCTTGCTCCTCCCATACTTTTGTTTTGGTCAGCCTGCACCTT
SRR517005.97398522.1+                    CTTCCTCTTGCTCCTCCCATACTTTTGTTTTGGTCAGCCTGCACCTT
SRR511915.38060972.2+                     TTCCTCTTGCTCCTCCCATACTTTTGTTTTGGTCAGCCTGCACCTT
SRR516972.63924756.1+                     TTCCTCTTGCTCCTCCCATACTTTTGTTTTGGTCAGCCTGCACCTT
SRR516985.13798813.2-                     TTCCTCTTGCTCCTCCCATACTTTTGTTTTGGTCAGCCTGCACCTT
SRR516985.51865401.1+                     TTCCTCTTGCTCCTCCCATACTTTTGTTTTGGTCAGCCTGCACCTT
SRR516993.69967585.2+                     TTCCTCTTGCTCCTCCCATACTTTTGTTTTGGTCAGCCTGCACCTT
SRR516995.78799244.2+                     TTCCTCTTGCTCCTCCCATACTTTTGTTTTGGTCAGCCTGCACCTT
SRR516998.18257531.2-                     TTCCTCTTGCTCCTCCCATACTTTTGTTTTGGTAAAGCTGCAACTT
SRR517005.13267808.2+                     TTCCTCTTGCTCCTCCCATACTTTTGTTTTGGTCAGCCTGCACCTT
SRR511892.45941781.2-                      TCCTCTTGCTCCTCCCATACTTTTGTTTTGGTCAGCCTGCACCTT
SRR511917.65115730.1+                      TCCTCTTGCTCCTCCCATACTTTTGTTTTGGTCAGCCTGCACCTT
SRR516980.32139732.1+                      TCCTCTTGCTCCTCCCATACTTTTGTTTTGGTCAGCCTGCACCTT
SRR516981.35675221.2-                      TCCTCTTGCTCCTCCCATACTTTTGTTTTGGTCAGCCTGCACCTT
SRR516991.64036010.1+                      TCCTCTTGCTCCTCCCATACTTTTGTTTTGGTCAGCCTGCACCTT
SRR516992.27044560.2+                      TCCTCTTGCTCCTCCCATACTTTTGTTTTGGTCAGCCTGCACCTT
SRR516994.58264551.2+                      TCCTCTTGCTCCTCCCATACTTTTGTTTTGGTCAGCCTGCACCTT
SRR516996.11016954.1+                      TCCTCTTGCTCCTCCCATACTTTTGTTTTGGTCAGCCTGCACCTT
SRR516999.1643008.1-                       TCCTCTTGCTCCTCCCATACTTTTGTTTTGGTCAGCCTGCACCTT
SRR516999.85502800.1-                      TCCTCTTGCTCCTCCCATACTTTTGTTTTGGTCAGCCTGCTCCTT
SRR516999.85502801.1-                      TCCTCTTGCTCCTCCCATACTTTTGTTTTGGTCAGCCTGCACCTT
SRR517004.61579126.2+                      TCCTCGTGCTCCTCCCATACTTTTGTTTTGGTCAGCCTGCACCTT
SRR516967.14216035.1-                       CCTCTTGCTCCTCCCATACTTTTGTTTTGGTCAGCCTGCACCTT
SRR516968.120253034.1-                      CCTCTTGCTCCTCCCATACTTTTGTTTTGGTCAGCCTGCACCTT
SRR516977.50716105.1-                       CCTCTTGCTCCTCCCATACTTTTGTTTTGGTCAGCCTGCACCTT
SRR516979.5196665.1+                        CCTCTTGTTCCTCCCATACTTTTGTTTTGGTCAGCCTGCACCTT
SRR516993.85753445.2-                       CCTCTTGCTCCTCCCATACTTTTGTTTTGGTCAGCCTGCACCTT
SRR516997.91295456.2-                       CCTCTTGCTCCTCCCATACTTTTGTTTTGGTCAGCCTGCACCTT
SRR511911.38218078.1+                        CTCTTGCTCCTCCCATACTTTTGTTTTGGTCAGCCTGCACCTC
SRR511911.45879133.2-                        CTCTTGCTCCTCCCATACTTTTGTTTTGGTCAGCCTGCACCTT
SRR511913.35212029.2-                        CTCTTGCTCCTCCCATACTTTTGTTTTGGTCAGCCTGCACCTT
SRR516967.2436532.1+                         CTCTTGCTCCTCCCATACTTTTGTTTTGGTCAGCCTGCACCTT
SRR516993.25377542.2-                        CTCTTGCTCCTCCCATACTTTTGTTTTGGTCAGCCTGCACCTT
SRR516998.23421238.1-                        CTCTTGCTCCTCCCATACTTTTGTTTTGGTCAGCCTGCACCTT
SRR517000.38312514.2+                        CTCTTGCTCCTCCCATACTTTTGTTTTGGTCAGCCTGCACCTT
SRR517003.87401309.2+                        CTCTTGCTCCTCCCATACTTTTGTTTTGGTCAGCCTGCACCTT
SRR511892.24906029.1-                         TCTTGCTCCTCCCATTCTTTTGTTTTGGTCAGCCTGCACCTT
SRR511912.52540950.1+                         TCTTGCTCCTCCCATACTTTTGTTTTGGTCAGCCTGCACCTT
SRR511913.44631486.2+                         TCTTGCTCCTCCCATACTTTTGTTTTGGTCAGCCTGCACCTT
SRR516971.10078918.1+                         TCTTGCTCCTCCCATACTTTTGTTTTGGTCAGCCTGCACCTT
SRR516982.63179189.1-                         TCTTGCTCCTCCCATACTTTTGTTTTGGTCAGCCTGCACCTT
SRR517000.55314048.2-                         TCTTGCTCCTCCCATACTTTTGTTTTGGTCAGCCTGCACCTT
SRR511915.51707777.1-                          CTTGCTCCTCCCATACTTTTGTTTTGGTCAGCCTGCACCTT
SRR516974.55348987.1-                          CTTGCTCCTCCCATACTTTTGTTTTGGTCAGCCTGCACCTT
SRR516992.48824419.1-                          CTTGCTCCTCCCATACTTTTGTTTTGGTCAGCCTGCACCTT
SRR516994.12134146.1-                          CTTGCTCCTCCCATACTTTTGTTTTGGTCAGCCTGCACCTT
SRR516995.10684909.1+                          CTTGCTCCTCCCATACTTTTGTTTTGGTCAGCCTGCACCTT
SRR516999.103440235.2+                         CTTGCTCCTCCCATACTTTTGTTTTGGTCAGCCTGCACCTT
SRR516999.10410465.2-                          CTTGCTCCTCCCATACTTTTGTTTTGGTCAGCCTGCACCTT
SRR517000.64874633.2+                          CTTGCTCCTCCCATACTTTTGTTTTGGTCAGCCTGCACCTT
SRR517003.26284114.2+                          CTTGCTCCTCCCATACTTTTGTTTTGGTCAGCCTGCACCTT
SRR517003.71529016.1-                          CTTGCTCCTCCCATACTTTTGTTTTGGTCAGCCTGCACCTT
SRR511917.9086576.1+                            TTGCTCCTCCCATACTTTTGTTTTGGTCAGCCTGCACCTT
SRR516973.14872432.2+                           TTGCTCCTCCCATACTTTTGTTTTGGTCAGCCTGCACCTT
SRR516974.65117480.1+                           TTGCTCCTCCCATACTTTTGTTTTGGTCAGCCTGCACCTT
SRR516976.8246559.2+                            TTGCTCCTCCCATACTTTTGTTTTGGTCAGCCTGCACCTT
SRR516986.40277712.2+                           TTGCTCCTCCCATACTTTTGTTTTGGTCAGCCTGCACCTT
SRR517001.76210543.1+                           TTGCTCCTCCCATACTTTTGTTTTGGTCAGCCTGCACCTT
SRR516972.31596629.1-                            TGCTCCTCCCATACTTTTGTTTTGGTCAGCCTGCACCTT
SRR516972.85177199.1+                            TGCTCCTCCCATACTTTTGTTTTGGTCAGCCTGCACCTT
SRR516978.40529185.1-                            TGCTCCTCCCATACTTTTGTTTTGGTCAGCCTGCACCTT
SRR516988.42046286.1+                            TGCTCCTCCCATACTTTTGTTTTGGTCAGCCTGCACCTT
SRR516992.573765.1-                              TGCTCCTCCCATACTTTTGTTTTGGTCAGCCTGCACCTT
SRR516993.28392126.2-                            TGCTCCTCCCATACTTTTGTTTTGGTCAGCCTGCACCTT
SRR517002.75362667.2-                            TGCTCCTCCCATACTTTTGTTTTGGTCAGCCTGCACCTT
SRR511912.83753137.1-                             GCTCCTCCCATACTTTTGTTTTGGTCAGCCTGCACCTT
SRR516968.125235180.1-                            GCTCCTCCCATACTTTTGTTTTGGTCAGCCTGCACCTT
SRR516973.46174374.2-                             GCTCCTCCCATACTTTTGTTTTGGTCAGCCTGCACCTT
SRR516978.2051635.2+                              GCTCCTCCCATACTTTTGTTTTGGTCAGCCTGCACCTT
SRR516983.77073621.2+                             GCTCCTCCCATACTTTTGTTTTGGTCAGCCTGCACCTT
SRR516985.42939042.2+                             GCTCCTCCCATACTTTTGTTTTGGTCAGCCTGCACCTT
SRR517001.74321810.2+                             GCTCCTCCCATACTTTTGTTTTGGTCAGCCTGCACCTT
SRR517004.29961337.2-                             GCTCCTCCCATACTTTTGTTTTGGTCAGCCTGCACCTT
SRR517006.172048.1-                               GCTCCTCCCATACTTTTGTTTTGGTCAGCCTGCACCTT
```

SRR517006.43830014.2+	GCTCCTCCCATACTTTTGTTTTGGTCAGCCTGCACCTT
SRR517007.20420514.2+	GCTCCTCCCATACTTTTGTTTTGGTCAGCCTGCACCTT
SRR516972.64059213.1-	CTCCTCCCATACTTTTGTTTTGGTCAGCCTGCACCTT
SRR516974.65696664.1+	CTCCTCCCATACTTTTGTTTTGGTCAGCCTGCACCTT
SRR516977.4731991.2+	CTCCTCCCATACTTTTGTTTTGGTCAGCCTGCACCTT
SRR516985.18999080.2+	CTCCTCCCATACTTTTGTTTTGGTCAGCCTGCACCTT
SRR516985.37325423.1+	CTCCTCCCATACTTTTGTTTTGGTCAGCCTGCACCTT
SRR516993.30875953.1+	CTCCTCCCATACTTTTGTTTTGGTCAGCCTGCACCTT
SRR517000.62796423.1+	CTCCTCCCATACTTTTGTTTTGGTCAGCCTGCACCTT
SRR517003.52120889.1-	CTCCTCCCATACTTTTGTTTTGGTCAGCCTGCACCTT
SRR517005.98675652.1+	CTCCTCCCATACTTTTGTTTTGGTCAGCCTGCACCTT
SRR516968.170088452.1-	TCCTCCCATACTTTTGTTTTGGTCAGCCTGCACCTT
SRR516972.52377834.2-	TCCTCCCATACTTTTGTTTTGGTCAGCCTGCACCTT
SRR516975.50269754.2+	TCCTCCCATACTTTTGTTTTGGTCAGCCTGCACCTT
SRR516981.37293974.2+	TCCTCCCATACTTTTGTTTTGGACAGACTGCACCTT
SRR516984.12015783.2+	TCCTCCCATACTTTTGTTTTGGTCAGCCTGCACCTT
SRR516991.78169676.2+	TCCTCCCATACTTTTGTTTTGGTCAGCCTGCACCTT
SRR516998.99573345.2+	TCCTCCCATACTTTTGTTTTGGTCAGCCTGCACCTT
SRR517000.62408706.2+	TCCTCCCATACTTTTGTTTTGGTCAGCCTGCACCTT
SRR517004.44571193.1+	TCCTCCCATACTTTTGTTTTGGTCAGCCTGCACCTT
SRR517004.7608079.2+	TCCTCCCATACTTTTGTTTTGGTCAGCCTGCACCTT
SRR511915.45075036.2+	CCTCCCATACTTTTGTTTTGGTCAGCCTGCACCTT
SRR516968.42660308.2+	CCTCCCATACTTTTGTTTTGGTCAGCCTGCACCTT
SRR516977.51389208.2+	CCTCCCATACTTTTGTTTTGGTCAGCCTGCACCTT
SRR516978.9550445.2-	CCTCCCATACTTTTGTTTTGGTCAGCCTGCCCCTT
SRR516989.35030363.2-	CCTCCCATATTTTTGTTTTGGCCAGCCTGCCCCTT
SRR516991.90343348.1+	CCTCCCATACTTTTGTTTTGGTCAGCCTGCACCTT
SRR516998.25981085.1-	CCTCCCATACTTTTGTTTTGGTCAGCCTGCACCTT
SRR517001.47934217.2+	CCTCCCATACTTTTGTTTTGGTCAGCCTGCACCTT
SRR517002.20414411.2-	CCTCCCATACTTTTGTTTTGGTCAGCCTGCACCTT
SRR517005.84338736.2-	CCTCCCATACTTTTGTTTTGGTCAGCCTGCACCTT
SRR517008.63673114.2-	CCTCCCATACTTTTGTTTTGGTCAGCCTGCCCCTT
SRR511892.53930910.1-	CTCCCATACTTTTGTTTTGGTCAGCCTGCACCTT
SRR511911.36873194.2+	CTCCCATACTTTTGTTTTGGTCAGCCTGCACCTT
SRR511914.67737508.2+	CTCCCATACTTTTGTTTTGGTCAGCCTGCACCTT
SRR511914.67737642.2+	CTCCCATACTTTTGTTTTGGTCAGCCTGCACCTT
SRR516978.3904335.2+	CTCCCATACTTTTGTTTTGGTCAGCCTGCACCTT
SRR516980.55772569.1+	CTCCCATACTTTTGTTTTGGTCAGCCTGCACCTT
SRR516983.11462340.1+	CTCCCATATTTTTGTTTTGGTCAGCCTGCACCTT
SRR516996.61810277.2-	CTCCCATACTTTTGTTTTGGTCAGCCTGCACCTT
SRR516998.602129.1+	CTCCCATACTTTTGTTTTGGTCAGCCTGCACCTT
SRR516999.110390232.2+	CTCCCATACTTTTGTTTTGGTCAGCCTGCACCTT
SRR517002.59657103.2+	CTCCCATACTTTTGTTTTGGTCAGCCTGCACCTT
SRR511912.40363221.2+	TCCCATACTTTTGTTTTGGTCAGCCTGCACCTT
SRR516968.7574142.1+	TCCCATACTTTTGTTTTGGTCAGCCTGCACCTT
SRR516986.3278042.1+	TCCCATACTTTTGTTTTGGTCAGCCTGCACCTT
SRR516987.11536053.2+	TCCCATACTTTTGTTTTGGTCAGCCTGCACCTT
SRR516988.19872809.2-	TCCCATACTTTTGTTTTGGTCAGCCTGCACCTT
SRR516989.88613591.1-	TCCCATACTTTTGTTTTGGTCAGCCTGCACCTT
SRR516994.39637294.1+	TCCCATACTTTTGTTTTGGTCAGCCTGCACCTT
SRR517001.54436281.1+	TCCCATACTTTTGTTTTGGTCAGCCTGCACCTT
SRR517002.22893072.1-	TCCCATACTTTTGTTTTGGTCAGCCTGCACCTT
SRR517005.31804048.2+	TCCCATACTTTTGTTTTGGTCAGCCTGCACCTT
SRR517009.24352435.2-	TCCCATACTTTTGTTTTGGTCAGCCTGCACCTT
SRR511911.13404865.1-	CCCATACTTTTGTTTTGGTCAGCCTGCACCTT
SRR516967.20886732.1+	CCCATACTTTTGTTTTGGTCAGCCTGCACCTT
SRR516985.49238935.1-	CCCATACTTTTGTTTTGGTCAGCCTGCACCTT
SRR516991.10395258.2+	CCCATACTTTTGTTTTGGTCAGCCTGCACCTT
SRR516999.61931820.2+	CCCATACTTTTGTTTTGGTCAGCCTGCACCTT
SRR516999.79892526.2-	CCCATACTTTTGTTTTGGTCAGCCTGCACCTT
SRR517002.72952186.2+	CCCATACTTTTGTTTTGGTCAGCCTGCACCTT
SRR511892.22460703.2-	CCATACTTTTGTTTTGGTCAGCCTGCACCTT
SRR511892.28500594.2-	CCATACTTTTGTTTTGGTCAGCCTGCTCCTT
SRR516970.9984608.2-	CCATACTTTTGTTTTGGTCAGCCTGCACCTT
SRR516974.62115179.1+	CCATACTTTTGTTTTGGTCAGCCTGCACCTT
SRR516991.23709560.2-	CCATCCTTTTGTTTTGGTCAGCCTGCACCTT
SRR516992.64410587.2+	CCATACTTTTGTTTTGGTCAGCCTGCACCTT
SRR517000.60951698.1+	CCATACTTTTGTTTTGGTCAGCCTGCACCTT
SRR517009.21051867.2+	CCATACTTTTGTTTTGGTCGGCCTGCACCTT
SRR511892.6007403.2+	CATACTTTTGTTTTGGTCAGCCTGCACCTT
SRR511914.68550111.1-	CATACTTTTGTTTTGGTCAGCCTGCACCTT
SRR511914.68550259.1-	CATACTTTTGTTTTGTTCAGCCTGCACCTT
SRR516968.81828792.2+	CATACTTTTGTTTTGGTCAGCCTGCACCTT
SRR516978.43361919.1-	CATACTTTTGTTTTGGTCAGCCTGCACCTT
SRR516983.52186797.1-	CATCCTTTTGTTTTGGTCAGCCTGCACCTT
SRR516985.65479664.1-	CATACTTTTGTTTTGGTCAGCCTGCACCTT
SRR516986.15810916.2-	CATACTTTTGTTTTGGTCAGCCTGCACCTT
SRR516991.25212876.1-	CATACTTTTGTTTTGGTCAGCCTGCACCTT
SRR516992.30084668.1+	CATACTTTTGTTTTGGTCAGCCTGCACCTT
SRR516996.21145455.1-	CATACTTTTGTTTTGGTCAGCCTGCACCTT
SRR516996.47831185.1+	CATACTTTTGTTTTGGTCAGCCTGCACCTT
SRR516998.96704581.1+	CATACTTTTGTTTTGGTCAGCCTGCACCTT
SRR517007.4964232.2+	CATACTTTTGTTTTGGTCAGCCTGCACCTT
SRR517007.75509732.1+	CATACTTTTGTTTTGGTCAGCCTGCACCTT

SRR517008.31103292.1-	CATACTTTTGTTTTGGTCAGCCTGCACCTT
SRR516968.37214890.1+	ATACTTTTGTTTTGGTCAGCCTGCACCTT
SRR516990.25197712.2+	ATACTTTTGTTTTGGTCAGCCTGCACCTT
SRR516990.28337778.2+	ATACTTTTGTTTTGGTCAGCCTGCACCTT
SRR517008.17734492.1+	ATACTTTTGTTTTGGTCAGCCTGCACCTT
SRR516978.76490935.1-	TACTTTTGTTTTGGTCAGCCTGCACCTT
SRR516983.22296978.1-	TACTTTTGTTTTGGTCAGCCTGCACCTT
SRR516997.92691745.1+	TACTTTTGTTTTGGTCAGCCTGCACCTT
SRR517001.54839630.2+	TACTTTTGTTTTGGTCAGCCTGCACCTT
SRR517009.22763744.1+	TACTTTTGTTTTGGTCAGCCTGCACCTT
SRR511912.3464697.2-	ACTTTTGTTTTGGTCAGCCGGCACCTT
SRR511913.30633233.1+	ACTTTTGTTTTGGTCAGCCTGCACCTT
SRR511913.52630668.1+	ACTTTTGTTTTGGTCAGCCTGCACCTT
SRR516968.75914217.2-	CCTTTTGTTTTGGTCAGCCTGCACCTT
SRR516978.51856587.2-	ACTTTTGTTTTGGTCAGCCTGCACCTT
SRR516993.14999698.2+	ACTTTTGTTTTGGTCAGCCTGCACCTT
SRR517005.29004911.1-	ACTTTTGTTTTGGTCAGCCTGCACCTT
SRR511914.66457590.1+	CTTTTGTTTTGGTCAGCCTGCACCTT
SRR516967.13632766.2+	CTTTTGTTTTGGTCAGCCTGCACCTT
SRR516972.66728747.1-	CTTTTGTTTTGGTCAGCCTGCACCTT
SRR516974.18523926.1+	CTTTTGTTTTGGTCAGCCTGCACCTT
SRR516976.50060483.1+	CTTTTGTTTTGGTCAGCCTGCACCTT
SRR516984.85335095.2-	CTTTTGTTTTGGTCAGCCTGCACCTT
SRR517004.11493321.2-	CTTTTGTTTTGGTCAGCCTGCACCTT
SRR517006.60516129.2-	CTTTTGTTTTGGTCAGCCTGCACCTT
SRR517008.72452430.1-	CTTTTGTTTTGGTCAGCCTGCACCTT
SRR511911.7460180.2+	TTTTGTTTTGGTCAGCCTGCACCTT
SRR511913.70659514.2-	TTTTGTTTTGGTCAGCCTGCACCTT
SRR511913.72818344.2-	TTTTGTTTTGGTCAGCCTGCACCTT
SRR516972.40257819.2+	TTTTGTTTTGGTCAGCCTGCACCTT
SRR516976.35412479.1-	TTTTGTTTTGGTCAGCCTGCACCTT
SRR516985.24934961.2-	TTTTGTTTTGGTCAGCCTGCACCTT
SRR516988.48142203.1-	TTTTGTTTTGGTAAGCCTGCACCTT
SRR516992.45298055.2+	TTTTGTTTTGGTCAGCCTGCACCTT
SRR516993.79889276.2-	TTTTGTTTTGGTCAGCCTGCACCTT
SRR516994.50421693.1-	TTTTGTTTTGGTCAGCCTGCACCTT
SRR516996.22277693.1-	TTTTGTTTTGGTCAGCCTGCACCTT
SRR516998.103714982.2-	TTTTGTTTTGGTCAGCCTGCACCTT
SRR517000.64893284.2-	TTTTGTTTTGGTCAGCCTGCACCTT
SRR517002.35600907.2-	TTTTGTTTTGGTCAGCCTGCACCTT
SRR511914.2434148.2-	TTTGTTTTGGTCAGCCTGCACCTT
SRR516968.78983771.2+	TTTGTTTTGGTCAGCCTGCACCTT
SRR516971.818087.2-	TTTGTTTTGGTCAGCCTGCACCTT
SRR516972.7252980.1-	TTTGTTTTGGTCAGCCTGCACCTT
SRR516974.34088399.1+	TTTGTTTTGGTCAGCCTGCACCTT
SRR516975.50255788.1-	TTTGTTTTGGTCAGCCTGCACCTT
SRR516996.4359948.2+	TTTGTTTTGGTCAGCCTGCACCTT
SRR516999.101662647.1-	TTTGTTTTGGTCAGCCTGCACCTT
SRR517004.15232910.1+	TTTGTTTTGGTCAGCCTGCACCTT
SRR517005.38635366.1-	TTTGTTTTGGTCAGCCTGCACCTT
SRR511892.64175574.1+	TTGTTTTGGTCAGCCTGCACCTT
SRR511912.78794364.2-	TTGTTTGGGTCAGCCTGCACCTT
SRR511913.54466415.2-	TTGTTTTGGTCAGCCTGCACCTT
SRR511914.49593496.1-	TTGTTTTGGTCAGCCTGCACCTT
SRR511914.68003041.1-	TTGTTTTGGTCAGCCTGCACCTT
SRR516978.13762672.2+	TTGTTTTGGTCAGCCTGCACCTT
SRR516984.19458384.2-	TTGTTTTGGTCAGCCTGCACCTT
SRR516991.40101822.2+	TTGTTTTGGTCAGCCTGCACCTT
SRR516997.46341269.2+	TTGTTTTGGTCAGCCTGCACCTT
SRR517006.3014885.2+	TTGTTTTGGTCAGCCTGCACCTT
SRR511916.12623440.2+	TGTTTTGGTCAGCCTGCACCTT
SRR511916.12623444.2+	TGTTTTGGTCAGCCTGCACCTT
SRR511917.23628752.2+	TGTTTTGGTCAGCCTGCACCTT
SRR516983.99315750.2-	TGTTTTGGTCATCCTGCACCTT
SRR516994.69104660.2-	TGTTTTGGTCAGCCTGCACCTT
SRR516995.44976834.2+	TGTTTTGGTCAGCCTGCACCTT
SRR516985.3722256.2-	GTTTTGGCCAGCCTGCACCTT
SRR516991.88390963.2-	GTTTTGGTCAGCCTGCTCCTT
SRR516994.3915521.1-	GTTTTGGTCAGCCTGCACCTT
SRR516994.39508980.2-	GTTTTGGTCAGCCTGCACCTT
SRR517001.21007961.1-	GTTTTGGTCAGCCTGCACCTT
SRR517004.39340770.2-	GTTTTGGTCAGCCTGCACCTT
SRR517005.36327145.1+	GTTTTGGTCAGCCTGCACCTT
SRR517005.3636097.2-	GTTTTGGTCAGCCTCCACCTT
SRR517005.92632228.1+	GTTTTGGTCAGCCTGCACCTT
SRR517008.46362816.2-	GTTTTGGTCAGCCTGCACCTT
SRR517009.32725362.1-	GTTTTGGTCAGCCTGCACCTT
SRR511918.23041134.2+	TTTTGGTCAGCCTGCACCTT
SRR511918.23041136.2+	TTTTGATCAGCCTGCACCTT
SRR511918.36794599.2+	TTTTGGTCAGCCTGCACCTT
SRR511920.10061090.2-	TTTTGGTCAGCCTGCACCTT
SRR511920.20487711.2-	TTTTGGTCAGCCTGCACCTT
SRR511920.20487970.2-	TTTTGGTCAGCCTGCACCTT
SRR511920.23071387.2-	TTTTGGTCAGCCTGCACCTT

SRR511920.26852972.2-	TTTTGGTCAGCCTGCACCTT
SRR511920.26853216.2-	TTTTGGTCAGCCTGCACCTT
SRR511920.30130909.2-	TTTTGGTCAGCCTGCACCTT
SRR511920.403516.2-	TTTTGGTCAGCCTGCACCTT
SRR511920.59478020.2-	TTTTGGTCAGCCTGCACCTT
SRR511920.8740493.2-	TTTTGGTCAGCCTGCACCTT
SRR516968.96419618.2+	TTTTGGTCAGCCTGCACCTT
SRR516972.63526588.1-	TTTTGGTCAGCCTGCACCTT
SRR516973.2205347.2-	TTTTGGTCAGCCTGCACCTT
SRR516978.68244002.1+	TTTTGGTCAGCCTGCACCTT
SRR516979.22734254.1+	TTTTGGTCAGCCTGCACCTT
SRR516989.78285929.2+	TTTTGGTCAGCCTGCACCTT
SRR516993.6142128.1-	TTTTGGTCAGCCTGCACCTT
SRR517004.18294620.1+	TTTTGGTCAGCCTGCACCTT
SRR517007.27000447.1-	TTTTGGTCAGCCTGCACCTT
SRR516981.64156231.1-	TTTGGTCAGCCTGCACCTT
SRR517000.22124310.1+	TTTGGTCAGCCTGCACCTT
SRR516980.56485916.1+	TTGGTTAGCCTGCACCTT
SRR511913.74026630.2+	TGGTCAGCCTGCACCTT
SRR516979.70135575.2+	TGGTCAGCCTGCACCTT
SRR516987.73050149.1-	TGGTCAGCCTGCACCTT
SRR516994.71971275.2-	TGGTCAGCCTGCACCTT
SRR517009.41211152.1-	TGGTCAGCCTGCACCTT
SRR516970.28438184.2+	GGTCAGCCTGCACCTT
SRR516972.46769474.2+	GGTCAGCCTGCACCTT
SRR516982.11686504.2-	GGTCAGCCTGCACCTT
SRR516984.39380541.1-	GGTCAGCCTGCACCTT
SRR517009.58624417.1-	GGTCAGCCTGCACCTT
SRR511892.63390781.1+	GTCAGCCTGCACCTT
SRR511916.30499491.1-	GTCAGCCTGCACCTT
SRR511917.13648601.1-	GTCAGCCTGCACCTT
SRR516967.56639881.1+	GTCAGCCTGCACCTT
SRR516976.3885854.1-	GTCAGCCTGCACCTT
SRR516985.21667072.2-	GTCAGCCTGCACCTT
SRR517001.20598275.2-	GTCAGCCTGCACCTT
SRR517004.38117563.1-	GTCAGCCTGCACCTT
SRR517004.57378084.1+	GTCAGCCTGCACCTT
SRR511912.71061316.2+	TCAGCCTGCACCTT
SRR516979.54060768.1-	TCAGCCTGCACCTT
SRR516982.20957450.2+	TCAGCCTGCACCTT
SRR516989.31478451.2-	TCAGCCTGCACCTT
SRR516990.26352168.1-	TCAGCCTGCACCTT
SRR516999.8565899.2+	TCAGCCTGCACCTT
SRR517001.99395117.2-	TCAGCCTGCACCTT
SRR516985.49910327.1-	CAGCCTGCCCCTT
SRR516990.18276182.1-	CAGCCTGCACCTT
SRR516995.7000561.2+	CAGCCTGCACCTT
SRR516999.43689059.1-	CAGCCTGCACCTT
SRR517001.65137644.1-	CAGCCTGCACCTT
SRR511912.51896762.2+	AGCCTGCACCTT
SRR516975.14717207.2-	AGCCTGCACCTT
SRR516976.15724427.2+	AGCCTGCACCTT
SRR516983.60185222.2+	AGCCTGCACCTT
SRR516985.58622247.1+	AGCCTGCACCTT
SRR517001.24229462.2-	AGCCTGCACCTT
SRR517008.47368909.1+	AGCCTGCACCTT
SRR511892.42564732.1+	GCCTGCACCTT
SRR511911.53964321.1-	GCCTGCACTTT
SRR511915.30420375.2-	GCCTGCACCTT
SRR516968.179954570.2-	GCCTGCACCTT
SRR516980.641302.1-	GCCTGCACCTT
SRR516985.88521940.2-	GCCTGCACCTT
SRR517002.43430166.1-	GCCTGCACCTT
SRR511892.35285726.1-	CCTGCACCTT
SRR511917.54558919.1-	CCTGCACCTT
SRR516975.41667615.2-	CCTGCACCTT
SRR516978.65142163.2+	CCTGCCCATT
SRR516981.51713088.2-	CCTGCACCTT
SRR516985.18771290.2-	CCTGCACCTT
SRR516989.46948006.2+	CCTGCACCTT
SRR516989.48094546.1+	CCTGCACCTT
SRR516991.8406901.1-	CCTGCACCTT
SRR516978.54979818.2-	CTGCACCTT
SRR516982.7942053.2-	CTGCACCTT
SRR516987.14379181.1-	CTGCACCTT
SRR516988.28221388.1-	CTGCACCTT
SRR516988.8503657.1+	CTGCACCTT
SRR517000.91798223.1+	CTGCACCTT
SRR517001.32526871.1+	CTGCACCTT
SRR517006.42774934.1-	CTGCACCTT
SRR517009.37989736.1+	CTGCACCTT
SRR511892.56496978.2+	TGCACCTT
SRR511912.67114670.1+	TGCACCTT
SRR511918.3846685.1+	TGCACCTT

```
SRR516984.46881394.2+                                                        TGCACCTT
SRR516991.38701220.2+                                                        TGCACCTT
SRR516991.83096318.2+                                                        TGCACCTT
SRR516995.17155730.1-                                                        TGCACCTT
SRR516998.30978040.1-                                                        TGCACCTT
SRR516999.35189572.2+                                                        TGCACCTT
SRR517004.15247676.2-                                                        TGCCCCTT
SRR516969.19690168.1-                                                         GCACCTT
SRR516990.52881947.2-                                                         GCACCTT
SRR517000.84699913.1+                                                         GCACCTT
SRR517007.46550915.1+                                                         GCACCTT
SRR517008.30759488.1+                                                         GCACCTT
SRR516979.15989227.1-                                                          CACCTT
SRR516982.57333576.1-                                                          CACCTT
SRR516992.10463572.2+                                                          CACCTT
SRR511892.22200889.1+                                                           ACCTT
SRR511892.25732978.1-                                                           CCCTT
SRR516971.36450695.2+                                                           ACCTT
SRR516986.21282883.1-                                                           ACCTT
SRR516997.1368394.1+                                                            ACCTT
SRR516999.79336674.2-                                                           CCCTT
SRR511913.46683201.1-                                                            CCTT
SRR516973.44104519.1-                                                            CCTT
SRR516975.59671997.1+                                                            CCTT
SRR516981.71035524.1-                                                            CCTT
SRR516983.26943128.1-                                                            CCTT
SRR516998.7520652.2+                                                             CCTT
SRR516999.536846.1-                                                              CCTT
SRR517000.79804010.1-                                                            CCTT
SRR517003.32276918.1+                                                            CCTT
SRR517006.63434435.1-                                                            CCTT
SRR511912.25061802.2+                                                             CTT
SRR511918.10389351.2-                                                             CTT
SRR511918.19943510.2-                                                             CTT
SRR511918.26089337.2-                                                             CTT
SRR516971.1149505.2+                                                              CTT
SRR516994.60807912.2+                                                             CTT
SRR517006.92771968.1+                                                             CTT
SRR517009.39648041.1+                                                             CTT
SRR511911.48293933.2-                                                              TT
SRR516975.29998471.1+                                                              TT
SRR516975.42749277.1-                                                              TT
SRR516980.26511072.2-                                                              TT
SRR516982.62820607.2+                                                              TT
SRR516985.94632745.2+                                                              TT
SRR516996.41370267.1-                                                              TT
SRR517006.7636423.2-                                                               TT
SRR516973.26618435.2+                                                               T
SRR516979.13778629.2+                                                               T
SRR517000.59205461.2-                                                               T
SRR517008.53562741.2+                                                               T

consensus                TCAAGGGTATGCTCTTCCTCTTGCTCCTCCCATACTTTTGTTTTGGTCAGCCTGCACCTT

                             .    :    .    :    .    :    .    :    .    :    .    :
SRR511914.17741304.2+    C
SRR516967.40446194.1+    C
SRR516971.7392543.1+     C
SRR516973.23808767.1-    C
SRR516973.23808771.1-    C
SRR516975.39272850.2-    C
SRR516988.60003491.2-    C
SRR516991.63425507.1+    C
SRR517003.20939478.2-    C
SRR516967.22260303.1+    CA
SRR516967.30937619.1+    CA
SRR516969.1737591.2+     CA
SRR511892.27528962.1-    CAC
SRR516967.128996446.2+   CAC
SRR516968.75435688.2+    CAC
SRR516999.47140010.1+    CAC
SRR517000.83969593.2-    CAC
SRR517008.31099343.2-    CAC
SRR511917.65115730.1+    CACT
SRR516968.155018007.1-   CACT
SRR516968.53031658.1+    CACT
SRR516971.1738878.1-     CACT
SRR516972.31320955.1+    CACT
SRR516972.60791475.1+    CACT
SRR516984.7283570.1+     CACT
SRR516987.86762093.1-    CACT
SRR516991.40998329.1-    CACT
SRR516993.14043843.2-    CACT
SRR516993.47859690.2-    CACT
```

SRR516996.4820423.1-	CACT
SRR517005.42320821.2-	CACT
SRR516977.71210552.2-	CACTG
SRR516981.16534572.1+	CACTG
SRR516985.84026477.1+	CACTG
SRR517008.8977763.1-	CACTG
SRR516986.10651932.2+	CACTGC
SRR516995.20779522.2-	CACTGC
SRR516997.49184054.2+	CACTGC
SRR517000.95262135.1-	CACTGC
SRR516975.51826312.2-	CACTGCT
SRR516989.80624643.2+	CACTGCT
SRR516992.65303336.1+	CACTGCT
SRR516993.19338558.2+	CACTGCT
SRR516993.35664507.1-	CACTGCT
SRR516997.32779052.2+	CACTGCT
SRR517001.50757283.1-	CACTGCT
SRR517002.67211818.1-	CACTGCT
SRR517009.70549529.2+	CACTGCT
SRR516978.60261999.1-	CACTGCTG
SRR516985.6369730.2-	CACTGCTG
SRR516994.13045831.2+	CACTGCTG
SRR516997.72118786.2+	CACTGCTG
SRR516998.27579449.2+	CACTGCTG
SRR516999.40240407.2+	CACTGCTG
SRR517002.25042325.1+	CACTGCTG
SRR517003.19342168.1+	CACTGCTG
SRR511917.9086576.1+	CACTGCTGC
SRR516981.26581776.2+	CACTGCTGC
SRR516984.13867668.1-	CACTGCTGC
SRR516985.794078.1+	CACTGCTGC
SRR516991.64722198.2-	CACTGCTGC
SRR517001.13478608.1+	CACTGCTGC
SRR511892.28500594.1+	CACTGCTGCA
SRR511911.53964321.2+	CACTGCTGCA
SRR511914.39483852.2+	CACTGCTGCA
SRR516973.25479726.2+	CACTGCTGCA
SRR511914.44813836.2+	TACTGCTGCAT
SRR516985.90328884.2+	CACTGCTGCGT
SRR516990.36711687.2-	CACTGCTGCAT
SRR516991.52505003.2-	CACTGCTGCGT
SRR516998.95088687.1-	CACTGCTGCGT
SRR517002.26060078.2-	CACTGCTGCAT
SRR511892.35285726.2+	CACTGCTGCATT
SRR511913.5222172.1-	CACTGCTGCATT
SRR516971.4236129.1-	CACTGCTGCGTT
SRR516984.51860923.2+	CACTGCTGCGTT
SRR516985.38800584.1+	CACTGCTGCGTT
SRR517006.82333459.1-	CACTGCTGCATT
SRR516970.37367691.1+	CACTGCTGCGTTT
SRR516971.34030929.1-	CACTGCTGCGTTT
SRR516972.48121654.2-	CACTGCTGCATTT
SRR516978.69371288.1-	CACTGCTGCGTTT
SRR516980.49332165.2+	CACTGCTGCGTTT
SRR516986.22724390.1-	CACTGCTGCATTT
SRR516990.46955085.1+	CACTGCTGCATTT
SRR516993.77981446.2+	CACTGCTGCGTTT
SRR516998.40607410.2-	CACTGCTGCGTTT
SRR517009.19164909.2-	CACTGCTGCGTTT
SRR517009.5972409.1-	CACTGCTGCGTTT
SRR516970.17885639.1-	CACTGCTGCGTTTC
SRR516974.48843984.2-	CACTGCTGCATTTC
SRR516978.14547831.1+	CACTGCTGCGTTTC
SRR516985.70870904.1-	CACTGCTGCGTTTC
SRR516995.16662105.2-	CACTGCTGCGTTTC
SRR516995.73341151.2+	CACTGCTGCGTTTC
SRR516968.128087344.1+	CACTGCTGCATTTCT
SRR516974.24280528.2-	CACTGCTGCATTTCT
SRR516999.51381955.2-	CACTGCTGCATTTCT
SRR517004.28032599.1-	CACTGCTGCGTTTCT
SRR517006.64062576.1-	CACTGCTGCATTTCT
SRR517007.60890697.2+	CACTGCTGCATTTCT
SRR517009.45770063.2+	CACTGCTGCGTTTCT
SRR511911.48293933.1+	CACTGCTGCATTTCTC
SRR511911.61131441.2-	CACTGCTGCATTTCTC
SRR516967.23066803.1+	CACTGCTGCATTTCTC
SRR516968.73747636.2+	CACTGCTGCATTTCTC
SRR516969.33135240.1+	CACTGCTGCGTTTCTC
SRR516969.867824.1+	CACTGCTGCGTTTCTC
SRR516988.4949152.1-	CACTGCTGCGTTTCTC
SRR516997.73355617.2+	CACTGCTGCATTTCTC
SRR516998.87531343.2-	CACTGCTGCATTTCTC
SRR517002.82258575.2-	CACTGCTGCATTTCTC
SRR517006.4335776.2-	CACTGCTGCATTTCTC

SRR517006.52685296.2-	CACTGCTGCATTTCTC
SRR516967.41353207.1-	CACTGCTGCATTTCTCC
SRR516968.165290408.2-	CACTGCTGCATTTCTCC
SRR516982.49581501.1-	CACTGCTGCGTTTCTCC
SRR516983.35103124.2+	CACTGCTGCATTTCTCC
SRR516974.1759552.2+	CACTGCTGCATTTCTCCA
SRR516979.30065340.2+	CACTGCTGCGTTTCTCCA
SRR516984.6951606.2+	CACTGCTGCATTTCTCCA
SRR516986.17707572.1-	CACTGCTGCATTTCTCCA
SRR516999.32513950.1-	CACTGCTGCATTTCTCCA
SRR517000.67695666.1-	CACTGCTGCGTTTCTCCA
SRR517007.47743023.1-	CACTGCTGCATTTCTCCA
SRR516967.72187734.1+	CACTGCTGCATTTCTCCAT
SRR516971.6695405.1+	CACTGCTGCGTTTCTCCAT
SRR516974.33187826.2-	CACTGCTGCATTTCTCCAT
SRR516977.65941636.2-	CACTGCTGCGTTTCTCCAT
SRR516979.7087082.1-	CACTGCTGCGTTTCTCCAT
SRR516981.69540153.2-	CACTGCTGCATTTCTCCAT
SRR517000.44752129.1-	CACTGCTGCATTTCTCCAT
SRR511915.2208112.2-	CACTGCTGCATTTCTCCATC
SRR511915.2208399.2-	CACTGCTGCATTTCTCCATC
SRR516981.34446750.1-	CACTGCTGCATTTCTCCATC
SRR516991.54630322.1-	CACTGCTGCATTTCTCCATC
SRR517000.59906460.1+	CACTGCTGCGTTTCTCCATC
SRR511914.43073009.1+	CACTGCTGCATTTCTCCATCT
SRR516968.80862039.2-	CACTGCTGCGTTTCTCCATCT
SRR516972.30802147.2-	CACTGCTGCATTTCTCCATCT
SRR517006.75846793.2+	CACTGCTGCATTTCTCCATCT
SRR511912.16411003.2-	CACTGCTGCATTTCTCCATCTT
SRR511912.9044568.1+	CACTGCTGCATTTCTCCATCTT
SRR511915.50178675.2-	CACTGCTGCATTTCTCCATCTT
SRR516992.59812008.2-	CACTGCTGCATTTCTCCATCTT
SRR516992.60599956.2-	CACTGCTGCATTTCTCCATCTT
SRR516994.13973863.1+	CACTGCTGCGTTTCTCCATCTT
SRR516996.47443095.1+	CACTGCTGCATTTCTCCATCTT
SRR516996.84831988.2+	CACTGCTGCATTTCTCCATCTT
SRR516999.45267731.1+	CACTGCTGCATTTCTCCATCTT
SRR517002.81950718.1+	CACTGCTGCATTTCTCCATCTT
SRR517007.25883181.1+	CACTGCTGCATTTCTCCATCTT
SRR511911.24447502.2-	CACTGCTGCATTTCTCCATCTTC
SRR511915.12387403.1+	CACTGCTGCATTTCTCCATCTTC
SRR516967.143090323.1-	CACTGCTGCATTTCTCCATCTTC
SRR516967.99920687.2-	CACTGCTGCATTTCTCCATCTTC
SRR516972.53472127.1-	CACTGCTGCATTTCTCCATCTTC
SRR516972.77543660.1+	CACTGCTGCATTTCTCCATCTTC
SRR516978.70829906.1+	CACTGCTGCGTTTCTCCATCTTC
SRR516995.4091683.2+	CACTGCTGCGTTTCTCCATCTTC
SRR516999.38940100.1+	CACTGCTGCATTTCTCCATCTTC
SRR516999.42713432.1+	CACTGCTGCATTTCTCCATCTTC
SRR516968.31712935.1-	CACTGCTGCATTTCTCCATCTTCC
SRR516972.67116381.2-	CACTGCTGCATTTCTCCATCTTCC
SRR516981.56879022.2+	CACTGCTGCATTTCTCCATCTTCC
SRR516997.12935966.2-	CACTGCTGCATTTCTCCATCTTCC
SRR517002.91703218.2+	CACTGCTGCATTTCTCCATCTTCC
SRR516985.71587022.2-	CACTGCTGCGTTTCTCCATCTTCCT
SRR516990.23106415.2+	CACTGCTGCATTTCTCCATCTTCCT
SRR516992.42779638.1-	CACTGCTGCATTTCTCCATCTTCCT
SRR516995.27817063.1+	CACTGCTGCGTTTCTCCATCTTCCT
SRR516996.41067957.1-	CACTGCTGCATTTCTCCATCTTCCT
SRR516996.64162159.1-	CACTGCTGCATTTCTCCATCTTCCT
SRR517001.43143516.2+	CACTGCTGCGTTTCTCCATCTTCCT
SRR511913.41367866.2+	CACTGCTGCATTTCTCCATCTTCCTG
SRR516967.114035844.1+	CACTGCTGCATTTCTCCATCTTCCTG
SRR516970.33098467.1-	CACTGCTGCGTTTCTCCATCTTCCTG
SRR516984.11603344.2+	CACTGCTGCGTTTCTCCATCTTCCTG
SRR516985.95011897.2+	CACTGCTGCGTTTCTCCATCTTCCTG
SRR516990.30495340.2+	CACTGCTGCATTTCTCCATCTTCCTG
SRR516996.40869314.1+	CACTGCTGCATTTCTCCATCTTCCTG
SRR517007.50962620.2+	CACTGCTGCATTTCTCCATCTTCCTG
SRR511916.12623440.2+	CACTGCTGCATTTCTCCATCTTCCTGG
SRR511916.12623444.2+	CACTGCTGCATTTCTCCATCTTCCTGG
SRR511917.23628752.2+	CACTGCTGCATTTCTCCATCTTCCTGG
SRR516974.24221041.1-	CACTGCTGCATTTCTCCATCTTCCTGG
SRR516977.3418701.1+	CACTGCTGCGTTTCTCCATCTTCCTGG
SRR516978.44746730.2+	CACTGCTGCGTTTCTCCATCTTCCTGG
SRR516979.27449937.1+	CACTGCTGCGTTTCTCCATCTTCCTGG
SRR516983.18957782.2+	CACTGCTGCATTTCTCCCTCTTCCTGG
SRR516996.25148019.2+	CACTGCTGCATTTCTCCATCTTCCTGG
SRR516996.96865981.1-	CACTGCTGCATTTCTCCATCTTCCTGG
SRR516998.89996681.2+	CACTGCTGCATTTCTCCATCTTCCTGG
SRR516968.111104702.2-	CACTGCTGCGTTTCTCCATCTTCCTGGA
SRR516970.33098640.1-	CACTGCTGCGTTTCTCCATCTTCCTGGA
SRR516977.2738721.1-	CACTGCTGCGTTTCTCCATCTTCCTGGA
SRR516981.43172628.1-	CACTGCTGCATTTCTCCATCTTCCTGGA

SRR516992.78710121.1+	CACTGCTGCATTTCTCCATCTTCCTGGA
SRR516996.345191.1+	CACTGCTGCATTTCTCCATCTTCCTGGA
SRR516998.18257531.2-	CACTGCTGCATTTCTCCATCTTCCTGGA
SRR516999.41559143.1-	CACTGCTGCATTTCTCCATCTTCCTGGA
SRR516999.68726039.1-	CACTGCTGCATTTCTCCATCTTCCTGGA
SRR517006.8320014.2+	CACTGCTGCATTTCTCCATCTTCCTGGA
SRR517009.40247008.2+	CACTGCTGCGTTTCTCCATCTTCCTGGA
SRR511918.23041134.2+	CACTGCTGCATTTCTCCATCTTCCTGGAG
SRR511918.23041136.2+	CACTGCTGCATTTCTCCATCTTCCTGGAG
SRR511918.36794599.2+	CACTGCTGCATTTCTCCATCTTCCTGGAG
SRR511920.10061090.2-	CACTGCTGCATTTCTCCATCTTCCTGGAG
SRR511920.20487711.2-	CACTGCTGCATTTCTCCATCTTCCTGGAG
SRR511920.20487970.2-	CACTGCTGCATTTCTCCATCTTCCTGGAG
SRR511920.23071387.2-	CACTGCTGCATTTCTCCATCTTCCTGGAG
SRR511920.26852972.2-	CACTGCTGCATTTCTCCATCTTCCTGGAG
SRR511920.26853216.2-	CACTGCTGCATTTCTCCATCTTCCTGGAG
SRR511920.30130909.2-	CACTGCTGCATTTCTCCATCTTCCTGGAG
SRR511920.403516.2-	CACTGCTGCATTTCTCCATCTTCCTGGAG
SRR511920.59478020.2-	CACTGCTGCATTTCTCCATCTTCCTGGAG
SRR511920.8740493.2-	CACTGCTGCATTTCTCCATCTTCCTGGAG
SRR516967.3860522.2+	CACTGCTGCATTTCTCCATCTTCCTGGAG
SRR516974.66712393.1+	CACTGCTGCATTTCTCCATCTTCCTGGAG
SRR516978.55364120.2-	CACTGCTGCGTTTCTCCATCTTCCTGGAG
SRR516984.26297997.1+	CACTGCTGCGTTTCTCCATCTTCCTGGAG
SRR516999.58849034.2-	CACTGCTGCATTTCTCCATCTTCCTGGAG
SRR516999.76775311.2+	CACTGCTGCATTTCTCCATCTTCCTGGAG
SRR517003.5667689.1-	CACTGCTGCATTTCTCCATCTTCCTGGAG
SRR517007.51506783.2+	CACTGCTGCATTTCTCCATCTTCCTGGAG
SRR517008.35456369.2+	CACTGCTGCGTTTCTCCATCTTCCTGGAG
SRR517008.67351581.2+	CACTGCTGCGTTTCTCCATCTTACTGGAG
SRR516967.71626809.2+	CACTGCTGCATTTCTCCATCTTCCTGGAGC
SRR516983.2660585.2+	CACTGCTGCATTTCTCCATCTTCCTGGAGC
SRR516983.34256945.1+	CACTGCTGCATTTCTCCATCTTCCTGGAGC
SRR516984.67024304.1-	CACTGCTGCGTTTCTCCATCTTCCTGGAGC
SRR516994.22273890.1+	CACTGCTGCGTTTCTCCATCTTCCTGGAGC
SRR511911.14454908.2+	CACTGCTGCATTTCTCCATCTTCCTGGAGCC
SRR516968.108115361.1+	CACTGCTGCGTTTCTCCATCTTCCTGGAGCC
SRR516973.1417145.2-	CACTGCTGCGTTTCTCCATCTTCCTGGAGCC
SRR516973.1417146.2-	CACTGCTGCGTTTCTCCATCTTCCTGGAGCC
SRR516991.89756659.2-	CACTGCTGCATTTCTCCATCTTCCTGGAGCC
SRR516992.7333033.1+	CACTGCTGCGTTTCTCCATCTTCCTGGAGCC
SRR516998.65411337.1+	CACTGCTGCATTTCTCCATCTTCCTGGAGCC
SRR517006.59401473.1-	CACTGCTGCATTTCTCCATCTTCCTGGAGCC
SRR511914.85292277.1+	CACTGCTGCATTTCTCCATCTTCCTGGAGCCT
SRR516970.26934.1+	CACTGCTGCGTTTCCCCATCTTCCTGGAGCCT
SRR516972.63489975.1+	CACTGCTGCATTTCTCCATCTTCCTGGAGCCT
SRR516974.43659576.1+	CACTGCTGCATTTCTCCATCTTCCTGGAGCCT
SRR516986.47033107.2+	CACTGCTGCGTTTCTCCATCTTCCTGGAGCCT
SRR516988.14405849.2+	CACTGCTGCGTTTCTCCATCTTCCTGGAGCCT
SRR516991.84387060.1+	CACTGCTGCGTTTCTCCATCTTCCTGGAGCCT
SRR516993.73666384.1+	CACTGCTGCGTTTCTCCATCTTCCTGGAGCCT
SRR516996.17100141.2-	CACTGCTGCATTTCTCCATCTTCCTGGAGCCT
SRR516998.784538.1+	CACTGCTGCATTTCTCCATCTTCCTGGAGCCT
SRR517001.57642820.1+	CACTGCTGCGTTTCTCCATCTTCCTGGAGCCT
SRR517008.22652667.1+	CACTGCTGCGTTTCTCCATCTTCCTGGAGCCT
SRR511915.68961737.2+	CACTGCTGCATTTCTCCATCTTCCTGGAGCCTA
SRR516974.46129857.2-	CACTGCTGCATTTCTCCATCTTCCTGGAGCCTA
SRR516992.26195657.2-	CACTGCTGCGTTTCTCCATCTTCCTGGAGCCTA
SRR516994.51970256.2-	CACTGCTGCGTTTCTCCATCTTCCTGGAGCCTA
SRR517002.87764725.1+	CACTGCTGCATTTCTCCATCTTCCTGGAGCCTA
SRR517009.33285239.1+	CACTGCTGCGTTTCTCCATCTTCCTGGAGCCTA
SRR511915.72842805.1+	CACTGCTGCATTTCTCCATCTTCCTGGAGCCTAC
SRR511916.30499491.1-	CACTGCTGCATTTCTCCATCTTCCTGGAGCCTAC
SRR511917.13648601.1-	CACTGCTGCATTTCTCCATCTTCCTGGAGCCTAC
SRR516967.119505550.2-	CACTGCTGCATTTCTCCATCTTCCTGGAGCCTAC
SRR516979.72185467.1-	CACTGCTGCGTTTCTCCATCTTCCTGGAGCCTAC
SRR516995.50080094.1+	CACTGCTGCGTTTCTCCATCTTCCTGGAGCCTAC
SRR517006.37525580.1+	CACTGCTGCATTTCTCCATCTTCCTGGAGCCTAC
SRR517009.46221980.2+	CACTGCTGCGTTTCTCCATCTTCCTGGAGCCTAC
SRR516972.26613549.2+	CACTGCTGCATTTCTCCATCTTCCTGGAGCCTACA
SRR516973.19319985.2+	CACTGCTGCGTTTCTCCATCTTCCTGGAGCCTACA
SRR516983.80722236.2+	CACTGCTGCATTTCTCCATCTTCCTGGAGCCTACA
SRR511914.83943589.1-	CACTGCTGCATTTCTCCATCTTCCTGGAGCCTACAA
SRR516969.1709162.1-	CACTGCTGCGTTTCTCCATCTTCCTGGAGCCTACAA
SRR516975.16339360.1+	CACTGCTGCATTTCTCCATCTTCCTGGAGCCTACAA
SRR516993.45140548.2-	CACTGCTGCGTTTCTCCATCTTCCTGGAGCCTACAA
SRR516996.39193734.1-	CACTGCTGCATTTCTCCATCTTCCTGGAGCCTACAA
SRR517008.15407290.1+	CACTGCTGCGTTTCTCCATCTTCCTGGAGCCTACAA
SRR511917.54558919.1-	CACTGCTGCATTTCTCCATCTTCCTGGAGCCTACAAT
SRR516967.140688955.1+	CACTGCTGCATTTCTCCATCTTCCTGGAGCCTACAAT
SRR516973.17670450.1+	CACTGCTGCGTTTCTCCATCTTCCTGGAGCCTACAAT
SRR516973.41289849.2+	CACTGCTGCGTTTCTCCATCTTCCTGGAGCCTACAAT
SRR516981.50701184.1+	CACTGCTGCATTTCTCCATCTTCCTGGAGCCTACAAT
SRR516985.87014315.2-	CACTGCTGCGTTTCTCCATCTTCCTGGAGCCTACAAT

SRR516990.1457908.1-	CACTGCTGCATTTCTCCATCTTCCTGGAGCCTACAAT
SRR516992.74240970.2+	CACTGCTGCATTTCTCCATCTTCCGGGAGCCTACAAT
SRR516995.57519330.1-	CACTGCTGCGTTTCTCCATCTTCCTGGAGCCTACAAT
SRR516998.95947533.1+	CACTGCTGCGTTTCTCCATCTTCCTGGAGCCTACAAT
SRR517001.86378213.2-	CACTGCTGCGTTTCTCCATCTTCCTGGAGCCTACAAT
SRR516982.12131511.1+	CACTGCTGCGTTTCTCCATCTTCCTGGAGCCTACAATC
SRR511911.2560931.2-	CACTGCTGCATTTCTCCATCTTCCTGGAGCCTACAATCA
SRR516967.88384226.2+	CACTGCTGCATTTCTCCATCTTCCTGGAGCCTACAATCA
SRR516978.27059321.2+	CACTGCTGCGTTTCTCCATCTTCCCGGAGCCTACAATCA
SRR516983.33407963.2+	CACTGCTGCATTTCTCCATCTTCCTGGAGCCTACAATCA
SRR516984.90374479.1-	CACTGCTGCGTTTCTCCATCTTCCTGGAGCCTACAATCA
SRR516991.17053852.2+	CACTGCTGCATTTCTCCATCTTCCTGGAGCCTACAATCA
SRR516997.61814545.2+	CACTGCTGCATTTCTCCATCTTCCTGGAGCCTACAATCA
SRR516998.100162957.2-	CACTGCTGCATTTCTCCATCTTCCTGGAGCCTACAATCA
SRR516998.14101174.1-	CACTGCTGCATTTCTCCATCTTCCTGGAGCCTACAATCA
SRR517006.43830014.2+	CACTGCTTCATTTCTCAATCTTCCTTGAGCCTACAATCA
SRR516968.114845186.2-	CACTGCTGCATTTCTCCATCTTCCTGGAGCCTACAATCAT
SRR516968.37738736.1-	CACTGCTGCGTTTCTCCATCTTCCTGGAGCCTACAATCAT
SRR516973.53561779.1-	CACTGCTGCGTTTCTCCATCTTCCTGGAGCCTACAATCAT
SRR516978.47021888.1+	CACTGCTGCGTTTCTCCATCTTCCTGGAGCCTACAATCAT
SRR516986.24845211.1-	CACTGCTGCATTTCTCCATCTTCCTGGAGCCTACAATCAT
SRR516988.60315129.1-	CACTGCTGCGTTTCTCCATCTTCCTGGAGCCTACAATCAT
SRR516999.52172306.2+	CACTGCTGCATTTCTCCATCTTCCTGGAGCCTACAATCAT
SRR517000.8953178.1+	CACTGCTGCATTTCTCCATCTTCCTGGAGCCTACAATCAT
SRR511892.28064592.1+	CACTGCTGCATTTCTCCATCTTCCTGGAGCCTACAATCATG
SRR511892.41096124.1+	CACTGCTGCATTTCTCCATCTTCCTGGAGCCTACAATCATG
SRR511918.3846685.1+	CACTGCTGCATTTCTCCATCTTCCTGGAGCCTACAATCATG
SRR516967.25613572.1-	CACTGCTGCATTTCTCCATCTTCCTGGAGCCTACAATCATG
SRR516989.72004672.2+	CACTGCTGCATTTCTCCATCTTCCTGGAGCCTACAATCATG
SRR517000.44421459.2-	CACTGCTGCGTTTCTCCATCTTCCTGGAGCCTACAATCATG
SRR517009.47221478.1+	CACTGCTGCGTTTCTCCATCTTCCTGGAGCCTACAATCATG
SRR516972.23056341.2+	CACTGCTGCATTTCTCCATCTTCCTGGAGCCTACAATCATGG
SRR516972.63004169.2+	CACTGCTGCATTTCTCCATCTTCCTGGAGCCTACAATCATGG
SRR516981.4191635.1+	CACTGCTGCATTTCTCCATCTTCCTGGAGCCTACAATCATGG
SRR516986.987228.1+	CACTGCTGCGTTTCTCCATCTTCCTGGAGCCTACAATCATGG
SRR516987.3530565.1-	CACTGCTGCGTTTCTCCATCTTCCTGGAGCCTACAATCATGA
SRR516990.47650251.1+	CACTGCTGCATTTCTCCATCTTCCTGGAGCCTACAATCATGG
SRR511913.5587657.2+	CACTGCTGCATTTCTCCATCTTCCTGGAGCCTACAATCATGGT
SRR516968.109900230.1+	CACTGCTGCATTTCTCCATCTTCCTGGAGCCTACAATCATGGT
SRR516975.38510117.1+	CACTGCTGCATTTCTCCATCTTCCTGGAGCCTACAATCATGGT
SRR516978.25674765.2+	CACTGCTGCGTTTCTCCATCTTCCTGGAGCCTACAATCATGGT
SRR516983.15102679.2+	CACTGCTGCATTTCTCCATCTTCCTGGAGCCTACAATCATGGT
SRR516994.34198741.2-	CACTGCTGCGTTTCTCCATCTTCCTGGAGCCTACAATCATGGT
SRR516997.43562335.1+	CACTGCTGCGTTTCTCCATCTTCCTGGAGCCTACAATCATGAT
SRR516997.84310008.2+	CACTGCTGCGTTTCTCCATCTTCCTGGAGCCTACAATCATGAT
SRR516999.85502800.1-	CCCTGCTGCATTTCTCCATCTTCCTGGAGCCTACAATCATGGT
SRR517005.97398522.1+	CACTGCTGCATTTCTCCATCTTCCTGGAGCCTACAATCATGGT
SRR516967.113781662.1+	CACTGCTGCATTTCTCCATCTTCCTGGAGCCTACAATCATGGTT
SRR516967.75977499.2-	CACTGCTGCATTTCTCCATCTTCCTGGAGNCTACAATCATGGTT
SRR516968.13503998.1-	CACTGCTGCGTTTCTCCATCTTCCTGGAGCCTACAATCATGGTT
SRR516970.33702206.1-	CACTGCTGCGTTTCTCCATCTTCCTGGAGCCTACAATCATGGTT
SRR516972.63924756.1+	CACTGCTGCATTTCTCCATCTTCCTGGAGCCTACAATCATGGTT
SRR516985.13798813.2-	CACTGCTGCGTTTCTCCATCTTCCTGGAGCCTACAATCATGGTT
SRR516985.51865401.1+	CACTGCTGCGTTTCTCCATCTTCCTGGAGCCTACAATCATGGTT
SRR516993.69967585.2+	CACTGCTGCGTTTCTCCATCTTCCTGGAGCCTACACTCATGGTT
SRR516995.78799244.2+	CACTGCTGCGTTTCTCCATCTTCCTGGAGCCTACAATCATGGTT
SRR517005.13267808.2+	CACTGCTGCATTTCTCCATCTTCCTGGAGCCTACAATCATGGTT
SRR516980.32139732.1+	CACTGCTGCGTTTCTCCATCTTCCTGGAGCCTACAATCATGGTTT
SRR516981.35675221.2-	CACTGCTGCATTTCTCCATCTTCCTGGAGCCTACAATCATGGTTT
SRR516991.64036010.1+	CACTGCTGCGTTTCTCCATCTTCCTGGAGCCTACAATCATGATTT
SRR516992.27044560.2+	CACTGCTGCATTTCTCCATCTTCCTGGAGCCTACAATCATGGTTT
SRR516994.58264551.2+	CACTGCTGCGTTTCTCCATCTTCCTGGAGCCTACAATCATGGTTT
SRR516996.11016954.1+	CACTGCTGCATTTCTCCATCTTCCTGGAGCCTACAATCATGGTTT
SRR516999.1643008.1-	CACTGCTGCATTTCTCCATCTTCCTGGAGCCTACAATCATGGTTT
SRR516999.85502801.1-	CACTGCTGCATTTCTCCATCTTCCTGGAGCCTACAATCATGGTTT
SRR517004.61579126.2+	CACTGCTGCGTTTCTCCATCTTCCTGGAGCCTACAATCATGGTTT
SRR511892.39300111.2+	CACTGCTGCATTTCTCCATCTTCCTGGAGCCTACAATCATGGTTTA
SRR511918.10389351.2-	CACTGCTGCATTTCTCCATCTTCCTGGAGCCTACAATCATGGTTTA
SRR511918.19943510.2-	CACTGCTGCATTTCTCCATCTTCCTGGAGCCTACAATCATGGTTTA
SRR511918.26089337.2-	CACTGCTGCATTTCTCCATCTTCCTGGAGCCTACAATCATGGTTTA
SRR516967.692820.1+	CACTGCTGCATTTCTCCATCTTCCTGGAGCCTACAATCATGGTTTA
SRR516977.50716105.1-	CACTGCTGCGTTTCTCCATCTTCCTGGAGCCTACAATCATGGTTTA
SRR516979.5196665.1+	CACTGCTGCGTTTCTCCATCTTCCTGGAGCCTACAATCATGGTTTA
SRR516993.85753445.2-	CACTGCTGCGTTTCTCCATCTTCCTGGAGCCTACAATCATGGTTTA
SRR516997.91295456.2-	CACTGCTGCATTTCTCCATCTTCCTGGAGCCTACAATCATGGTTTA
SRR511914.74202240.2+	CACTGCTGCATTTCTCCATCTTCCTGGAGCCTACAATCATGGTTTAC
SRR511914.74202383.2+	CACTGCTGCATTTCTCCATCTTCCTGGAGCCTACAATCATGGTTTAC
SRR511915.77154079.1+	CACTGCTGCATTTCTCCATCTTCCTGGAGCCTACAATCATGGTTTAC
SRR516967.143302175.2-	CACTGCTGCATTTCTCCATCTTCCTGGAGCCTACAATCATGGTTTAC
SRR516993.25377542.2-	CACTGCTGCGTTTCTCCATCTTCCTGGAGCCTACAATCATGGTTTAC
SRR516998.23421238.1-	CACTGCTGCATTTCTCCATCTTCCTGGAGCCTACAATCATGGTTTAC
SRR517000.38312514.2+	CACTGCTGCATTTCTCCATCTTCCTGGAGCCTACAATCATGGTTTAC
SRR517003.87401309.2+	CACTGCTGCATTTCTCCATCTTCCTGGAGCCTACAATCATGGTTTAC
SRR516967.3323960.1+	CACTGCTGCATTTCTCCATCTTCCTGGAGCCTACAATCATGGTTTACC

SRR516971.33071473.1+ CACTGCTGCGTTTCTCCATCTTCCTGGAGCCTACAATCATGGTTTACC
SRR516982.63179189.1- CACTGCTGCGTTTCTCCATCTTCCTGGAGCCTACAATCATGATTTACC
SRR517000.55314048.2- CACTGCTGCATTTCTCCATCTTCCTGGAGCCTACAATCATGGTTTACC
SRR516967.51922488.1+ CACTGCTGCATTTCTCCATCTTCCTGGAGCCTACAATCATGGTTTACCT
SRR516967.66315071.1+ CACTGCTGCATTTCTCCATCTTCCTGGAGCCTACAATCATGGTTTACCT
SRR516974.55348987.1- CACTGCTGCATTTCTCCATCTTCCTGGAGCCTACAATCATGGTTTACCT
SRR516992.48824419.1- CACTGCTGCGTTTCTCCATCTTCCTGGAGCCTACAATCATGGTTTACCT
SRR516994.12134146.1- CACTGCTGCGTTTCTCCATCTTCCTGGAGCCTACAATCATGGTTTACCT
SRR516995.10684909.1+ CACTGCTGCGTTTCTCCATCTTCCTGGAGCCTACAATCATGGTTTACCT
SRR516999.103440235.2+ CACTGCTGCATTTCTCCATCTTCCTGGAGCCTACAATCATGGTTTACCT
SRR516999.10410465.2- CACTGCTGCATTTCTCCATCTTCCTGGAGCCTACAATCATGGTTTACCT
SRR517000.64874633.2+ CACTGCTGCGTTTCTCCATCTTCCTGGAGCCTACAATCATGGTTTACCT
SRR517003.26284114.2+ CACTGCTGCGTTTCTCCATCTTCCTGGAGCCTACAATCATGGTTTACCT
SRR517003.71529016.1- CACTGCTGCGTTTCTCCATCTTCCTGGAGCCTACAATCATGGTTTACCT
SRR511912.43117119.2+ CACTGCTGCATTTCTCCATCTTCCTGGAGCCTACAATCATGGTTTACCTC
SRR511915.70587641.1- CACTGCTGCATTTCTCCATCTTCCTGGAGCCTACAATCATGGTTTACCTC
SRR511915.70587944.1- CACTGCTGCATTTCTCCATCTTCCTGGAGCCTACAATCATGGTTTACCTC
SRR516973.14872432.2+ CACTGCTGCATTTCTCCATCTTCCTGGAGCCTACAATCATGGTTTACCTC
SRR516974.65117480.1+ CACTGCTGCATTTCTCCATCTTCCTGGAGCCTACAATCATGGTTTACCTC
SRR516976.8246559.2+ CACTGCTGCGTTTCTCCATCTTCCTGGCGCCTACAATCATGGTTTCCCTC
SRR516986.40277712.2+ CACTGCTGCATTTCTCCATCTTCCTGGAGCCTACAATCATGGTTTACCTC
SRR516989.35030363.2- CACTGCTGCTTTTCTCCATCTCCCTGGAGCCTACAATCATGGTTTCCCTC
SRR517001.76210543.1+ CACTGCTGCGTTTCTCCATCTTCCTGGAGCCTACAATCATGGTTTACCTC
SRR511892.65397717.2+ CACTGCTGCATTTCTCCATCTTCCTGGAGCCTACAATCATGGTTTACCTCT
SRR516971.18124229.1- CACTGCTGCGTTTCTCCATCTTCCTGGAGCCTACAATCATGGTTTACCTCT
SRR516972.31596629.1- CACTGCTGCATTTCTCCATCTTCCTGGAGCCTACAATCATGGTTTACCTCT
SRR516972.85177199.1+ CACTGCTGCATTTCTCCATCTTCCTGGAGCCTACAATCATGGTTTACCTCT
SRR516978.40529185.1- CACTGCTGCGTTTCTCCATCTTCCTGGAGCCTACAATCATGGTTTACCTCT
SRR516988.42046286.1+ CACTGCTGCGTTTCTCCATCTTCCTGGAGCCTACAATCATGGTTTACCTCT
SRR516992.573765.1- CACTGCTGCATTTCTCCATCTTCCTGGAGCCTACAATCATGGTTTACCTCT
SRR516993.28392126.2- CACTGCTGCGTTTCTCCATCTTCCTGGAGCCTACAATCATGGTTTACCTCT
SRR517002.75362667.2- CACTGCTGCATTTCTCCATCTTCCTGGAGCCTACAATCATGGTTTACCTCT
SRR511892.37274156.1+ CACTGCTGCATTTCTCCATCTTCCTGGAGCCTACAATCATGGTTTACCTCTG
SRR511911.71659560.1- CACTGCTGCATTTCTCCATCTTCCTGGAGCCTACAATCATGGTTTACCTCTG
SRR516973.46174374.2- CACTGCTGCATTTCTCCATCTTCCTGGAGCCTACAATCATGGTTTACCTCTG
SRR516978.2051635.2+ CACTGCTGCGTTTCTCCATCTTCATGGAGCCTACCATCATGGTTTACCTCTG
SRR516978.9550445.2- CACTGCTGCGTTTCTCCATCTTCCTGGAGCCTACAATCATGGTTTACCTCTG
SRR516983.77073621.2+ CACTGCTGCATTTCTCCATCTTCCTGGAGCCTACAATCATGGTTTACCTCTG
SRR516985.42939042.2+ CACTGCTGCGTTTCTCCATCTTCCTGGAGCCTACAATCATGGTTTACCTCTG
SRR517001.74321810.2+ CACTGCTGCGTTTCTCCATCTTCCTGGAGCCTACAATCATGGTTTACCTCTG
SRR517004.29961337.2- CACTGCTGCGTTTCTCCATCTTCCTGGAGCCTACGATCATGGTTTACCTCTG
SRR517006.172048.1- CACTGCTGCATTTCTCCATCTTCCTGGAGCCTACAATCATGGTTTACCTCTG
SRR517007.20420514.2+ CACTGCTGCATTTCTCCATCTTCCTGGAGCCTACAATCATGGTTTACCTCTG
SRR516972.64059213.1- CACTGCTGCATTTCTCCATCTTCCTGGAGCCTACAATCATGGTTTACCTCTGC
SRR516974.65696664.1+ CACTGCTGCATTTCTCCATCTTCCTGGAGCCTACAATCATGGCTTACCTCTGC
SRR516977.4731991.2+ CACTGCTGCGTTTCTCCATCTTCCTGGAGCCTACAATCATGGTTTACCTCTGC
SRR516985.18999080.2+ CACTGCTGCGTTTCTCCATCTTCCTGGAGCCTACAATCATGGTTTACCTCTGC
SRR516985.37325423.1+ CACTGCTGCGTTTCTCCATCTTCCTGGAGCCTACAATCATGGTTTACCTCTGC
SRR516993.30875953.1+ CACTGCTGCGTTTCTCCATCTTCCTGGAGCCTACAATCATGGTTTACCTCTGC
SRR517000.62796423.1+ CACTGCTGCGTTTCTCCATCTTCCTGGAGCCTACAATCATGGTTTACCTCTGC
SRR517003.52120889.1- CACTGCTGCATTTCTCCATCTTCCTGGAGCCTACAATCATGGTTTACCTCTGC
SRR517005.98675652.1+ CACTGCTGCGTTTCTCCATCTTCCTGGAGCCTACAATCATGTTTTACCTCTGC
SRR517008.63673114.2- CACTGCTGCGTTTCTCCATCTTCCTGGAGCCTACAATCATGGTTTACCTCTGC
SRR511915.38060972.2+ CACTGCTGCATTTCTCCATCTTCCTGGAGCCTACAATCATGGTTTACCTCTGCT
SRR516972.52377834.2- CACTGCTGCATTTCTCCATCTTCCTGGAGCCTACAATCATGGTTTACCTCTGCT
SRR516975.50269754.2+ CACTGCTGCATTTCTCCATCTTCCTGGAGCCTACAATCATGGTTTACCTCTGCT
SRR516981.37293974.2+ CACTGCTGCATTTCTCCATCTTCCTGGAGCCTACAATCATGGTATAACTCTGCT
SRR516984.12015783.2+ CACTGCTGCGTTTCTCCATCTTCCTGGAGCCTACAATCATGGTTTACCTCTGCT
SRR516991.78169676.2+ CACTGCTGCGTTTCTCCATCTTCCTGGAGCCTACAATCATGATTTACCTCTGCT
SRR516998.99573345.2+ CACTGCTGCATTTCTCCATCTTCCTGGAGCCTACAATCATGGTTTACCTCTGCT
SRR517000.62408706.2+ CACTGCTGCGTTTCTCCATCTTCCTGGAGCCTACAATCATGGTTTACCTCTGCT
SRR517004.44571193.1+ CACTGCTGCGTTTCTCCATCTTCCTGGAGCCTACAATCATGGTTTACCTCTGCT
SRR517004.7608079.2+ CACTGCTGCGTTTCTCCATCTTCCTGGAGCCTACAATCATGGTTTACCTCTGCT
SRR511892.45941781.2- CACTGCTGCATTTCTCCATCTTCCTGGAGCCTACAATCATGGTTTACCTCTGCTG
SRR516977.51389208.2+ CACTGCTGCGTTTCTCCATCTTCCTGGAGCCTACAATCATGGTTTACCTCTGCTG
SRR516991.90343348.1+ CACTGCTGCGTTTCTCCATCTTCCTGGAGCCTACAATCATGATTTACCTCTGCTG
SRR516996.61810277.2- CACTGCTGCATTTCTCCATCTTCCTGGAGCCTACAATCATGGTTTCCCTCTGCTG
SRR516998.25981085.1- CACTGCTGCATTTCTCCATCTTCCTGGAGCCTACAATCATGGTTTACCTCTGCTG
SRR517001.47934217.2+ CACTGCTGCGTTTCTCCATCTTCCTGGAGCCTACAATCATGGTTTACCTCTGCTG
SRR517002.20414411.2- CACTGCTGCATTTCTCCATCTTCCTGGAGCCTACAATCATGGTTTACCTCTGCTG
SRR517005.84338736.2- CACTGCTGCATTTCTCCATCTTCCTGGAGCCTACAATCATGGTTTACCTCTGCTG
SRR516978.3904335.2+ CACTGCTGCGTTTCTCCATCTTCCTGGAGCCTACAATCATGGTTTAACTCTGCTGA
SRR516980.55772569.1+ CACTGCTGCGTTTCTCCATCTTCCTGGAGCCTACAATCATGGTTTACCTCTGCTGA
SRR516983.11462340.1+ CACTGCTGCATTTCTCCATCTTCCTGGAGCCTACAATCATGGTTTACCTCTGCTGA
SRR516998.602129.1+ CACTGCTGCATTTCTCCATCTTCCTGGAGCCTACAATCATGGTTTACCTCTGCTGA
SRR516999.110390232.2+ CACTGCTGCATTTCTCCATCTTCCTGGAGCCTACAATCATGGTTTACCTCTGCTGA
SRR517002.59657103.2+ CACTGCTGCATTTCTCCATCTTCCTGGAGCCTACAATCATGGTTTACCTCTGCTGA
SRR511892.53930910.1- CACTGCTGCATTTCTCCATCTTCCTGGAGCCTACAATCATGGTTTACCTCTGCTGAG
SRR511911.38218078.1+ CACTGCTGCATTTCTCCATCTTCCTGGAGCCTACAATCATGGTTTACCTCTGCTGAG
SRR511911.45879133.2- CACTGCTGCATTTCTCCATCTTCCTGGAGCCTACAATCATGGTTTACCTCTGCTGAG
SRR511913.35212029.2- CACTGCTGCATTTCTCCATCTTCCTGGAGCCTACAATCATGGTTTACCTCTGCTGAG
SRR516967.14216035.1- CACTGCTGCATTTCTCCATCTTCCTGGAGCCTACAATCATGGTTTACCTCTGCTGAG
SRR516968.120253034.1- CACTGCTGCATTTCTCCATCTTCCTGGAGCCTACAATCATGGTTTACCTCTGCTGAG
SRR516971.10078918.1+ CACTGCTGCGTTTCTCCATCTTCCTGGAGCCTACAATCATGGTTTACCTCTGCTGAG

SRR516986.3278042.1+	CACTGCTGCATTTCTCCATCTTCCTGGAGCCTACAATCATGGTTTACCTCTGCTGAG
SRR516987.11536053.2+	CACTGCTGCATTTCTCCATCTTCCTGGAGCCTACAATCATGGTTTACCTCTGCTGAG
SRR516988.19872809.2-	CACTGCTGCGTTTCTCCATCTTCCTGGAGCCTACAATCATGGTTTACCTCTGCTGAG
SRR516989.88613591.1-	CACTGCTGCATTTCTCCATCTTCCTGGAGCCTACAATCATGGTTTACCTCTGCTGAG
SRR516994.39637294.1+	CACTGCTGCGTTTCTCCATCTTCCTGGAGCCTACAATCATGGTTTACCTCTGCTGAG
SRR517001.54436281.1+	CACTGCTGCGTTTCTCCATCTTCCTGGAGCCTACAATCATGGTTTACCTCTGCTGAG
SRR517002.22893072.1-	CACTGCTGCATTTCTCCATCTTCCTGGAGCCTACAATCATGGTTTACCTCTGCTGAG
SRR517002.72952186.2+	CACTGCTGCATTTCTCCATCTTCCTGGAGACTACAATCATGGTTTAACTCTGCTGAG
SRR517005.31804048.2+	CACTGCTGCATTTCTCCATCTTCCTGGAGCCTACAATCATGGTTTACCTCTGCTGAG
SRR517009.24352435.2-	CACTGCTGCGTTTCTCCATCTTCCTGGAGCCTACAATCATGGTTTACCTCTGCTGAG
SRR511892.24906029.1-	CACTGCTGCATTTCTCCATCTTCCTGGAGCCTACAATCATGGTTTACCTCTGCTGAGA
SRR511912.52540950.1+	CACTGCTGCATTTCTCCATCTTCCTGGAGCCTACAATCATGGTTTACCTCTGCTGAGA
SRR511913.44631486.2+	CACTGCTGCATTTCTCCATCTTCCTGGAGCCTACAATCATGGTTTACCTCTGCTGAGA
SRR516967.2436532.1+	CACTGCTGCATTTCTCCATCTTCCTGGAGCCTACAATCATGGTTTACCTCTGCTGAGA
SRR516985.49238935.1-	CACTGCTGCGTTTCTCCATCTTCCTGGAGCCTACAATCATGGTTTACCTCTGCTGAGA
SRR516991.10395258.2+	CACTGCTGCGTTTCTCCATCTTCCTGGAGCCTACAATCATGATTTACCTCTGCTGAGA
SRR516999.61931820.2+	CACTGCTGCATTTCTCCATCTTCCTGGAGCCTACAATCATGGTTTACCTCTGCTGAGA
SRR516999.79892526.2-	CACTGCTGCATTTCTCCATCTTCCTGGAGCCTACAATCATGGTTTACCTCTGCTGAGA
SRR511915.51707777.1-	CACTGCTGCATTTCTCCATCTTCCTGGAGCCTACAATCATGGTTTACCTCTGCTGAGAC
SRR516974.62115179.1+	CACTGCTGCATTTCTCCATCTTCCTGGAGCCTACAATCATGGTTTACCTCTGCTGAGAC
SRR516991.23709560.2-	CACTGCTGCGTTTCTCCATCTTCCTGGAGCCTACAATCATGATTTACCTCTGCTGAGAC
SRR516992.64410587.2+	CACTGCTGCATTTCTCCATCTTCCTGGAGCCTACAATCATGGTTTACCTCTGCTGAGAC
SRR517000.60951698.1+	CACTGCTGCGTTTCTCCATCTTCCTGGAGCCTACAATCATGGTTTACCTCTGCTGAGAC
SRR517009.21051867.2+	CACTGCTGCGTTTCTCGATCTTCCTGGAGCCTACAATCATGGTTTACCACTGCTGAGAC
SRR511892.22200889.1+	CACTGCTTCATTTCTCCATCTTCCTGGAGCCTACAATCATGGTTTACCTCTGCTGAGACC
SRR511892.22460703.2-	CACTGCTGCATTTCTCCATCTTCCTGGAGCCTACAATCATGGTTTACCTCTGCTGAGACC
SRR511892.25732978.1-	CACTGCTGCATTTCTCCATCTTCCTGGAGCCTACAATCATGGTTTACCTCTGCTGAGACC
SRR511892.28500594.2-	CACTGCTGCATTTCTCCATCTTCCTGGAGCCTACAATCATGGTTTACCTCTGCTGAGACC
SRR511892.35285726.1-	CACTGCTGCATTTCTCCATCTTCCTGGAGCCTACAATCATGGTTTACCTCTGCTGAGACC
SRR511892.42564732.1+	CACTGCTGCATTTCTCCATCTTCCTGGAGCCTACAATCATGGTTTACCTCTGCTGAGACC
SRR511892.56496978.2+	CACTGCTGCATTTCTCCATCTTCCTGGAGCCTACAATCATGGTTTACCTCTGCTGAGACC
SRR511892.6007403.2+	CACTGCTGCATTTCTCCATCTTCCTGGAGCCTACAATCATGGTTTACCTCTGCTGAGACC
SRR511892.63390781.1+	CACTGCTGCATTTCTCCATCTTCCTGGAGCCTACAATCATGGTTTACCTCTGCTGAGACC
SRR511892.64175574.1+	CACTGCTGCATTTCTCCATCTTCCTGGAGCCTACAATCATGGTTTACCTCTGCTGAGACC
SRR511911.13404865.1-	CACTGCTGCATTTCTCCATCTTCCTGGAGCCTACAATCATGGTTTACCTCTGCTGAGACC
SRR511911.36873194.2+	CACTGCTGCATTTCTCCATCTTCCTGGAGCCTACAATCATGGTTTACCTCTGCTGAGACC
SRR511911.48293933.2-	CACTGCTGCATTTCTCCATCTTCCTGGAGCCTACAATCATGGTTTACCTCTGCTGAGACC
SRR511911.53964321.1-	CACTGCTGCATTTCTCCATCTTCCTGGAGCCTACAATCATGGTTTACCTCTGCTGAGACC
SRR511911.7460180.2+	CACTGCTGCATTTCTCCATCTTCCTGGAGCCTACAATCATGGTTTACCTCTGCTGAGACC
SRR511912.25061802.2+	CACTGCTGCATTTCTCCATCTTCCTGGAGCCTACAATCATGGTTTACCTCTGCTGAGACC
SRR511912.3464697.2-	CACTGCTGCATTTCTCCATCTTCCTGGAGCCTACAATCATGGTTTGCCTCTGCTGAGACC
SRR511912.40363221.2+	CACTGCTGCATTTCTCCATCTTCCTGGAGCCTACAATCATGGTTTACCTCTGCTGAGACC
SRR511912.51896762.2+	CACTGCTGCATTTCTCCATCTTCCTGGAGCCTACAATCATGGTTTACCTCTGCTGAGACC
SRR511912.67114670.1+	CACTGCTGCATTTCTCCATCTTCCTGGAGCCTACAATCATGGTTTACCTCTGCTGAGACC
SRR511912.71061316.2+	CACTGCTGCATTTCTCCATCTTCCTGGAGCCTACAATCATGGTTTACCTCTGCTGAGACC
SRR511912.78794364.2-	CACTGCTGCATTTCTCCATCTTCCTGGAGCCTACAATCATGGTTTACCTCTGCTGAGACC
SRR511912.83753137.1-	CACTGCTGCATTTCTCCATCTTCCTGGAGCCTACAATCATGGTTTACCTCTGCTGAGACC
SRR511913.30633233.1+	CACTGCTGCATTTCTCCATCTTCCTGGAGCCTACAATCATGGTTTACCTCTGCTGAGACC
SRR511913.46683201.1-	CACTGCTGCATTTCTCCATCTTCCTGGAGCCTACAATCATGGTTTACCTCTGCTGAGACC
SRR511913.52630668.1+	CACTGCTGCATTTCTCCATCTTCCTGGAGCCTACAATCATGGTTTACCTCTGCTGAGACC
SRR511913.54466415.2-	CACTGCTGCATTTCTCCATCTTCCTGGAGCCTACAATCATGGTTTACCTCTGCTGAGACC
SRR511913.70659514.2-	CACTGCTGCATTTCTCCATCTTCCTGGAGCCTACAATCATGGTTTACCTCTGCTGAGACC
SRR511913.72818344.2-	CACTGCTGCATTTCTCCATCTTCCTGGAGCCTACAATCATGGTTTACCTCTGCTGAGACC
SRR511913.74026630.2+	CACTGCTGCATTTCTCCATCTTCCTGGAGCCTACAATCATGGTTTACCTCTGCTGAGACC
SRR511914.2434148.2-	CACTGCTGCATTTCTCCATCTTCCTGGAGCCTACAATCATGGTTTACCTCTGCTGAGACC
SRR511914.49593496.1-	CACTGCTGCATTTCTCCATCTTCCTGGAGCCTACAATCATGGTTTACCTCTGCTGAGACC
SRR511914.66457590.1+	CACTGCTGCATTTCTCCATCTTCCTGGAGCCTACAATCATGGTTTACCTCTGCTGAGACC
SRR511914.67737508.2+	CACTGCTGCATTTCTCCATCTTCCTGGAGCCTACAATCATGGTTTACCTCTGCTGAGACC
SRR511914.67737642.2+	CACTGCTGCATTTCTCCATCTTCCTGGAGCCTACAATCATGGTTTACCTCTGCTGAGACC
SRR511914.68003041.1-	CACTGCTGCATTTCTCCATCTTCCCGGAGCCTACAATCATGGTTTACCTCTGCTGAGACC
SRR511914.68550111.1-	CACTGCTGCATTTCTCCATCTTCCTGGAGCCTACAATCATGGTTTACCTCTGCTGAGACC
SRR511914.68550259.1-	CACTGCTGCATTTCTCCATCTTCCTGGAGCCTACAATCATGGTTTACCTCTGCTGAGACC
SRR511915.30420375.2-	CACTGCTGCATTTCTCCATCTTCCTGGAGCCTACAATCATGGTTTACCTCTGCTGAGACC
SRR511915.45075036.2+	CACTGCTGCATTTCTCCATCTTCCTGGAGCCTACAATCATGGTTTACCTCTGCTGAGACC
SRR516967.13632766.2+	CACTGCTGCATTTCTCCATCTTCCTGGAGCCTACAATCATGGTTTACCTCTGCTGAGACC
SRR516967.20886732.1+	CACTGCTGCATTTCTCCATCTTCCTGGAGCCTACAATCATGGTTTACCTCTGCTGAGACC
SRR516967.56639881.1+	CACTGCTGCATTTCTCCATCTTCCTGGAGCCTACAATCATGGTTTACCTCTGCTGAGACC
SRR516968.125235180.1-	CACTGCTGCATTTCTCCATCTTCCTGGAGCCTACAATCATGGTTTACCTCTGCTGAGACC
SRR516968.170088452.1-	CACTGCTGCGTTTCTCCATCTTCCTGGAGCCTACAATCATGGTTTACCTCTGCTGAGACC
SRR516968.179954570.2-	CACTGCTGCGTTTCTCCATCTTCCTGGAGCCTACAATCATGGTTTACCTCTGCTGAGACC
SRR516968.37214890.1+	CACTGCTGCGTTTCTCCATCTTCCTGGAGCCTACAGTCATGGTTTACCTCTGCTGAGACC
SRR516968.42660308.2+	CACTGCTGCATTTCTCCATCTTCCTGGAGCCTACAATCATGGTTTACCTCTGCTGAGACC
SRR516968.7574142.1+	CACTGCTGCGTTTCTCCATCTTCCTGGAGCCTACAATCATGGTTTACCTCTGCTGAGACC
SRR516968.75914217.2-	CACTGCTGCATTTCTCCATCTTCCTGGAGCCTACAATCATGGTTTACCTCTGCTGAGACC
SRR516968.78983771.2+	CACTGCTGCATTTCTCCATCTTCCTGGAGCCTACAATCATGGTTTACCTCTGCTGAGACC
SRR516968.81828792.2+	CACTGCTGCGTTTCTCCATCTTCCTGGAGCCTACAATCATGGTTTACCTCTGCTGAGACC
SRR516968.96419618.2+	CACTGCTGCATTTCTCCATCTTCCTGGAGCCTACAATCATGGTTTACCTCTGCTGAGACC
SRR516969.19690168.1-	CACTGCTGCGTTTCTCCAGCTTCCTGGAGCCTACAATCATGGTTTACCTCTGCTGAGACC
SRR516970.28438184.2+	CACTGCTGCGTTTCTCCATCTTCCTGGAGCCTACAATCATGGTTTACCTCTGCTGAGACC
SRR516970.9984608.2-	CACTGCTGCGTTTCTCCATCTTCCTGGAGCCTACAATCATGGTTTACCTCTGCTGAGACC
SRR516971.1149505.2+	CACTGCTGCGTTTCTCCATCTTCCTGGAGCCTACAATCATGGTTTACCTCTGCTGAGACC
SRR516971.36450695.2+	CACTGCTGCGTTTCTCCATCTTCCTGGAGCCTACAATCATGGTTTACCTCTGCTGAGACC
SRR516971.818087.2-	CACTGCTGCGTTTCTCCATCTTCCTGGAGCCTACAATCATGGTTTACCTCTGCTGAGACC
SRR516972.40257819.2+	CACTGCTGCATTTCTCCATCTTCCTGGAGCCTACAATCATGGTTTACCTCTGCTGAGACC

SRR516972.46769474.2+ CACTGCTGCATTTCTCCATCTTCCTGGAGCCTACAATCATGGTTTACCTCTGCTGAGACC
SRR516972.63526588.1- CACTGCTGCATTTCTCCATCTTCCTGGAGCCTACAATCATGGTTTACCTCTGCTGAGACC
SRR516972.66728747.1- CACTGCTGCATTTCTCCATCTTCCTGGAGCCTACAATCATGGTTTACCTCTGCTGAGACC
SRR516972.7252980.1- CACTGCTGCATTTCTCCATCTTCCTGGAGCCTACAATCATGGTTTACCTCTGCTGAGACC
SRR516973.2205347.2- CACTGCTGCGTTTCTCCATCTTCCTGGAGCCTACAATTATGATTTACCTCTGCTGAGACC
SRR516973.26618435.2+ CACTGCTGCGTTTCTCCATCTTCCTGGAGCCTACAATCATGATTTACCTCTGCTGAGACC
SRR516973.44104519.1- CACTGCTGCGTTTCTCCATCTTCCTGGAGCCTACAATCATGATTTACCTCTGCTGAGACC
SRR516974.18523926.1+ CACTGCTGCATTTCTCCATCTTCCTGGAGCCTACAATCATGGTTTACCTCTGCTGAGACC
SRR516974.34088399.1+ CACTGCTGCATTTCTCCATCTTCCTGGAGCCTACAATCATGGTTTACCTCTGCTGAGACC
SRR516975.14717207.2- CACTGCTGCGTTTCTCCATCTTCCTGGAGCCTACAATCATGATTTACCTCTGCTGAGACC
SRR516975.29998471.1+ CACTGCTGCGTTTCTCCATCTTCCTGGAGCCTACAATCATGATTTACCTCTGCTGAGACC
SRR516975.41667615.2- CACTGCTGCATTTCTCCATCTTCCTGGAGCCTACAATCATGGTTTACCTCTGCTGAGACC
SRR516975.42749277.1- CACTGCTGCGTTTCTCCATCTTCCTGGAGCCTACAATCATGATTTACCTCTGCTGAGACC
SRR516975.50255788.1- CACTGCTGCATTTCTCCATCTTCCTGGAGCCTACAATCATGGTTTACCTCTGCTGAGACC
SRR516975.59671997.1+ CACTGCTGCATTTCTCCATCTTCCTGGAGCCTACAATCATGGTTTACCTCTGCTGAGACC
SRR516976.15724427.2+ CACTGCTGCGTTTCTCCATCTTCCTGGAGCCTACAATCATGATTTACCTCTGCTGAGACC
SRR516976.35412479.1- CACTGCTGCGTTTCTCCATCTTCCTGGAGCCTACAATCATGGTTTACCTCTGCTGAGACC
SRR516976.3885854.1- CACTGCTGCGTTTCTCCAGCTTCCTGGAGCCTACAATCATGGTTTACCTCTGCTGAGACC
SRR516976.50060483.1+ CACTGCTGCGTTTCTCCATCTTCCTGGAGCCTACAATCATGATTTACCTCTGCTGAGACC
SRR516978.13762672.2+ CACTGCTGCGTTTCTCCATCTTCCTGGAGCCTACAATCATGGTTTACCTCTGCTGAGAAC
SRR516978.43361919.1- CACTGCTGCGTTTCTCCATCTTCCTGGAGCCTACAATCATGGTTTACCTCTGCTGAGACC
SRR516978.51856587.2- CACTGCTGCGTTTCTCCATCTTCCTGGAGCCTACAATCATGGTTTACCTCTGCTGAGACC
SRR516978.54979818.2- CACTGCTGCGTTTCTCCATCTTCCTGGAGCCTACAATCATGGTTTACCTCTGCTGAGACC
SRR516978.65142163.2+ CCCTGCTGCGTTTCTCCATCTTCCTGGAGCCTACAATCATGGTTTACATCTGCTGAGACC
SRR516978.68244002.1+ CACTGCTGCGTTTCTCCATCTTCCTGGAGCCTACAATCATGGTTTACCTCTGCTGAGACC
SRR516978.70371063.2- CACTGCTGCGTTTCTCCATCTTCCTGGAGCCTACAATCATGGTTTACCTCTGCTGAGACC
SRR516978.76490935.1- CACTGCTGCGTTTCTCCATCTTCCTGGAGCCTACAATCATGGTTTACCTCTGCTGAGACC
SRR516979.13778629.2+ CACTGCTGCGTTTCTCCATCTTCCTGGAGCCTACAATCATGGTTTACCTCTGCTGAGACC
SRR516979.15989227.1- CACTGCTGCGTTTCTCCATCTTCCTGGAGCCTACAATCATGGTTTACCTCTGCTGAGACC
SRR516979.22734254.1+ CACTGCTGCGTTTCTCCATCTTCCTGGAGCCTACAATCATGATTTACCTCTGCTGAGACC
SRR516979.54060768.1- CACTGCTGCGTTTCTCCATCTTCCTGGAGCCTACAATCATGATTTACCTCTGCTGAGACC
SRR516979.70135575.2+ CACTGCTGCGTTTCTCCATCTTCCTGGAGCCTACAATCATGATTTACCTCTGCTGAGACC
SRR516980.26511072.2- CACTGCTGCGTTTCTCCATCTTCCTGGAGCCTACAATCATGGTTTACCTCTGCTGAGACC
SRR516980.56485916.1+ CACTGCTGCGTTTCTCCATCTTCCTGGAGCCTACAATCATGGTTTACCTCTGCTGAGACC
SRR516980.641302.1- CACTGCTGCGTTTCTCCATCTTCCTGGAGCCTACAATCATGGTTTACCTCTGCTGAGACC
SRR516981.51713088.2- CACTGCTGCATTTCTCCATCTTCCTGGAGCCTACAATCATGGTTTACCTCTGCTGAGACC
SRR516981.64156231.1- CACTGCTGCATTTCTCCATCTTCCTGGAGCCTACAATCATGGTTTACCTCTGCTGAGACC
SRR516981.71035524.1- CACTGCTGCATTTCTCCATCTTCCTGGAGCCTACAATCATGGTTTACCTCTGCTGAGACC
SRR516982.11686504.2- CACTGCTGCGTTTCTCCATCTTCCTGGAGCCTACAATCATGATTTACCTCTGCTGAGACC
SRR516982.20957450.2+ CACTGCTGCGTTTCTCCATCTTCCTGGAGCCTACAATCATGATTTACCTCTGCTGAGACC
SRR516982.57333576.1- CACTGCTGCGTTTCTCCATCTTCCTGGAGCCTACAATCATGATTTACCTCTGCTGAGACC
SRR516982.62820607.2+ CACTGCTGCGTTTCTCCATCTTCCTGGAGCCTACAATCATGATTTACCTCTGCTGAGACC
SRR516982.7942053.2- CACTGCTGCGTTTCTCCATCTTCCTGGAGCCTACAATCATGATTTACCTCTGCTGAGACC
SRR516983.22296978.1- CACTGCTGCATTTCTCCATCTTCCTGGAGCCTACAATCATGGTTTACCTCTGCTGAGACC
SRR516983.26943128.1- CACTGCTGCATTTCTCCATCTTCCTGGAGCCTACAATCATGGTTTACCTCTGCTGAGACC
SRR516983.52186797.1- CACTGCTGCATTTCTCCATCTTCCTGGAGCCTACAATCATGGTTTACCTCTGCTGAGACC
SRR516983.60185222.2+ CACTGCTGCATTTCTCCATCTTCCTGGAGCCTACAATCATGGTTTACCTCTGCTGAGACC
SRR516983.99315750.2- CACTGCTGCATTTCTTCATCTTCCTGGAGCCTACAATCATGGTTTACCTCTGCTGAGACC
SRR516984.19458384.2- CACTGCTGCATTTCTCCATCTTCCTGGAGCCTACAATCATGGTTTACCTCTGCTGAGACC
SRR516984.39380541.1- CACTGCTGCGTTTCTCCATCTTCCTGGAGCCTACAATCATGGTTTACCTCTGCTGAGACC
SRR516984.46881394.2+ CACTGCTGCGTTTCTCCATCTTCCTGGAGCCTACAATCATGGTTTACCTCTGCTGAGACC
SRR516984.85335095.2- CACTGCTGCATTTCTCCATCTTCCTGGAGCCTACAATCATGGTTTACCTCTGCTGAGACC
SRR516985.18771290.2- CACTGCTGCGTTTCTCCATCTTCCTGGAGCCTACAATCATGGTTTACCTCTGCTGAGACC
SRR516985.21667072.2- CACTGCTGCGTTTCTCCATCTTCCTGGAGCCTACAATCATGGTTTACCTCTGCTGAGACC
SRR516985.24934961.2- CACTGCTGCGTTTCTCCATCTTCCTGGAGCCTACAATCATGGTTTACCTCTGCTGAGACC
SRR516985.3722256.2- CACTGCTGCGTTTCTCCATCTTCCTGGAGCCTACAATCATGGTTTACCTCTGCTGAGACC
SRR516985.49910327.1- CACTGCTGCGTTTCTCCATCTTCCTGGAGCCTACAATCATGGTTTACCTCTGCTGAGACC
SRR516985.58622247.1+ CACTGCTGCGTTTCTCCATCTTCCTGGAGCCTACAATCATGGTTTACCTCTGCTGAGACC
SRR516985.65479664.1- CACTGCTGCGTTTCTCCATCTTCCTGGAGCCTACAATCATGGTTTACCTCTGCTGAGACC
SRR516985.88521940.2- CACTGCTGCGTTTCTCCATCTTCCTGGAGCCTACAATCATGGTTTACCTCTGCTGAGACC
SRR516985.94632745.2+ CACTGCTGCGTTTCTCTATCTTCCTGGAGCCTACAATCATGGTTTACCTCTGCTGAGACC
SRR516986.15810916.2- CACTGCTGCATTTCTCCATCTTCCTGGAGCCTACAATCATGGTTTACCTCTGCTGAGACC
SRR516986.21282883.1- CACTGCTGCATTTCTCCATCTTCCTGGAGCCTACAATCATGGTTTACCTCTGCTGAGACC
SRR516986.6287834.2+ CACTGCTGCATTTCTCCATCTTCCTGGAGCCTACAATCATGGTTTACCTCTGCTGAGACC
SRR516987.14379181.1- CACTGCTGCATTTCTCCATCTTCCTGGAGCCTACAATCATGGTTTACCTCTGCTGAGACC
SRR516987.73050149.1- CACTGCTGCATTTCTCCATCTTCCTGGAGCCTACAATCATGGTTTACCTCTGCTGAGACC
SRR516988.28221388.1- CACTGCTGCGTTTCTCCATCTTCCTGGAGCCTACAATCATGGTTTACCTCTGCTGAGACC
SRR516988.48142203.1- CACTGCTGCGTTTCTCCATCTTCCTGGAGCCTACAATCATGGTTTACCTCTGCTGAGACC
SRR516988.8503657.1+ CACTGCTGCGTTTCTCCATCTTCCTGGAGCCTACAATCATGGTTTACCTCTGCTGAGACC
SRR516989.31478451.2- CACTGCTGCATTTCTCCATCTTCCTGGAGCCTACAATCATGGTTTACCTCTGCTGAGACC
SRR516989.46948006.2+ CACTGCTGCATTTCTCCATCTTCCTGGAGCCTACAATCATGGTTTACCTCTGCTGAGACC
SRR516989.48094546.1+ CACTGCTGCATTTCTCCATCTTCCTGGAGCCTACAATCATGGTTTACCTCTGCTGAGACC
SRR516989.78285929.2+ CACTGCTGCATTTCTCCATCTTCCTGGAGCCTACAATCATGGTTTACCTCTGCTGAGACC
SRR516990.18276182.1- CACTGCTGCATTTCTCCATCTTCCTGGAGCCTACAATCATGGTTTACCTCTGCTGAGACC
SRR516990.25197712.2+ CACTGCTGCATTTCTCCATCTTCCTGGAGCCTACAATCATGGTTTACCTCTGCTGAGACC
SRR516990.26352168.1- CACTGCTGCATTTCTCCATCTTCCTGGAGCCTACAATCATGGTTTACCTCTGCTGAGACC
SRR516990.28337778.2+ CACTGCTGCATTTCTCCATCTTCCTGGAGCCTACAATCATGGTTTACCTCTGCTGAGACC
SRR516990.52881947.2- CACTGCTGCATTTCTCCATCTTCCTGGAGCCTACAATCATGGTTTACCTCTGCTGAGACC
SRR516991.25212876.1- CACTGCTGCATTTCTCCATCTTCCTGGAGCCTACAATCATGGTTTACCTCTGCTGAGACC
SRR516991.38701220.2+ CACTGCTGCATTTCTCCATCTTCCTGGAGCCTACAATCATGGTTTACCTCTGCTGAGACC
SRR516991.40101822.2+ CACTGCTGCATTTCTCCATCTTCCTGGAGCCTACAATCATGGTTTACCTCTGCTGAGACC
SRR516991.55763720.1+ CACTGCTGCATTTCTCCATCTTCCTGGAGCCTACAATCATGGTTTACCTCTGCTGAGACC
SRR516991.83096318.2+ CACTGCTGCGTTTCTCCATCTTCCTGGAGCCTACAATCATGATTTACCTCTGCTGAGACC
SRR516991.8406901.1- CACTGCTGCGTTTCTCCATCTTCCTGGAGCCTACAATCATGATTTACCTCTGCTGAGACC

SRR516991.88390963.2-   CACTGCTGCGTTTCTCCATCTTCCTGGAGCCTACAATCATGATTTACCTCTGCTGAGACC
SRR516992.10463572.2+   CACTGCTGCGTTTCTCCATCTTCCTGGAGCCTACAATCATGGTTTACCTCTGCTGAGACC
SRR516992.30084668.1+   CACTGCTGCGTTTCTCCATCTTCCTGGAGCCTACAATCATGGTTTACCTCTGCTGAGACC
SRR516992.45298055.2+   CACTGCTGCATTTCTCCATCTTCCTGGAGCCTACAATCATGGTTTACCTCAGCTGAGACC
SRR516993.14999698.2+   CACTGCTGCGTTTCTCCATCTTCCTGGAGCCTACAATCATGGTTTACCTCTGCTGAGACC
SRR516993.6142128.1-   CACTGCTGCGTTTCTCCATCTTCCTGGAGCCTACAATCATGGTTTACCTCTGCTGAGACC
SRR516993.79889276.2-   CACTGCTGCGTTTCTCCATCTTCCTGGAGCCTACAATCATGGTTTACCTCTGCTGAGACC
SRR516994.3915521.1-   CACTGCTGCGTTTCTCCATCTTCCTGGAGCCTACAATCATGGTTTACCTCTGCTGAGACC
SRR516994.39508980.2-   CACTGCTGCGTTTCTCCATCTTCCTGGAGCCTACAATCATGGTTTACCTCTGCTGAGACC
SRR516994.50421693.1-   CACTGCTGCGTTTCTCCATCTTCCTGGAGCCTACAATCATGGTTTACCTCTGCTGAGACC
SRR516994.60807912.2+   CACTGCTGCGTTTCTCCATCTTCCTGGAGCCTACAATCATGGTTTACCTCTGCTGAGACC
SRR516994.69104660.2-   CACTGCTGCGTTTCTCCATCTTCCTGGAGCCTACAATCATGGTTTACCTCTGCTGAGACC
SRR516994.71971275.2-   CACTGCTGCGTTTCTCCATCTTCCTGGAGCCTACAATCATGGTTTACCTCTGCTGAGACC
SRR516995.17155730.1-   CACTGCTGCGTTTCTCCATCTTCCTGGAGCCTACAATCATGGTTTACCTCTGCTGAGACC
SRR516995.44976834.2+   CACTGCTGCGTTTCTCCATCTTCCTGGAGCCTACAATCATGGTTTACCTCTGCTGAGACC
SRR516995.7000561.2+   CACTGCTGCGTTTCTCCATCTTCCTGGAGCCTACAATCATGGTTTACCTCTGCTGAGACC
SRR516996.21145455.1-   CACTGCTGCATTTCTCCATCTTCCTGGAGCCTACAATCATGGTTTACCTCTGCTGAGACC
SRR516996.22277693.1-   CACTGCTGCATTTCTCCATCTTCCTGGAGCCTACAATCATGGTTTACCTCTGCTGAGACC
SRR516996.41370267.1-   CACTGCTGCATTTCTCCATCTTCCTGGAGCCTACAATCATGGTTTACCTCTGCTGAGACC
SRR516996.4359948.2+   CACTGCTGCATTTCTCCATCTTCCTGGAGCCTACAATCATGGTTTACCTCTGCTGAGACC
SRR516996.47831185.1+   CACTGCTGCATTTCTCCATCTTCCTGGAGCCTACAATCATGGTTTACCTCTGCTGAGACC
SRR516997.1368394.1+   CACTGCTGCATTTCTCCATCTTCCTGGAGCCTACAATCATGGTTTACCTCTGCTGAGACC
SRR516997.46341269.2+   CACTGCTGCGTTTCTCCATCTTCCTGGAGCCTACAATCATGATTTACCTCTGCTGAGACC
SRR516997.92691745.1+   CACTGCTGCGTTTCTCCATCTTCCTGGAGCCTACAATCATGATTTACCTCTGCTGAGACC
SRR516998.103714982.2-   CACTGCTGCGTTTCTCCATCTTCCTGGAGCCTACAATCATGGTTTACCTCTGCTGAGACC
SRR516998.30978040.1-   CACTGCTGCATTTCTCCATCTTCCTGGAGCCTACAATCATGGTTTACCTCTGCTGAGACC
SRR516998.7520652.2+   CACTGCTGCGTTTCTCCATCTTCCTGGAGCCTACAATCATGGTTTACCTCTGCTGAGACC
SRR516998.96704581.1+   CACTGCTGCATTTCTCCATCTTCCTGGAGCCTACACTCATGGTTTACCTCTGCTGAGACC
SRR516999.101662647.1-   CACTGCTGCATTTCTCCATCTTCCTGGAGCCTACAATCATGGTTTACCTCTGCTGAGACC
SRR516999.35189572.2+   CACTGCTGCATTTCTCCATCTTCCTGGAGCCTACAATCATGGTTTACCTCTGCTGAGACC
SRR516999.43689059.1-   CACTGCTGCATTTCTCCATCTTCCTGGAGCCTACAATCATGGTTTACCTCTGCTGAGACC
SRR516999.536846.1-   CACTGCTGCATTTCTCCATCTTCCTGGAGCCTACAATCATGGTTTACCTCTGCTGAGACC
SRR516999.79336674.2-   CACTGCTGCATTTCTCCATCTTCCTGGAGCCTACAATCATGGTTTACCTCTGCTGAGACC
SRR516999.8565899.2+   CACTGCTGCATTTCTCCCTCTTCCTGGAGCCTACAATCATGGGTTACCTCTGCTGAGACC
SRR517000.22124310.1+   CACTGCTGCGTTTCTCCATCTTCCTGGAGCCTACAATCATGGTTTACCTCTGCTGAGACC
SRR517000.59205461.2-   CACTGCTGCATTTCTCCATCTTCCTGGAGCCTACAATCATGGTTTACCTCTGCTGAGACC
SRR517000.64893284.2-   CACTGCTGCGTTTCTCCATCTTCCTGGAGCCTACAATCATGGTTTACCTCTGCTGAGACC
SRR517000.79804010.1-   CACTGCTGCGTTTCTCCATCTTCCTGGAGCCTACAATCATGGTTTACCTCTGCTGAGACC
SRR517000.84699913.1+   CACTGCTGCGTTTCTCCATCTTCCTGGAGCCTACAATCATGGTTTACCTCTGCTGAGACC
SRR517000.91798223.1+   CACTGCTGCATTTCTCCATCTTCCTGGAGCCTACAATCATGGTTTACCTCTGCTGAGACC
SRR517001.20598275.2-   CACTGCTGCGTTTCTCCATCTTCCTGGAGCCTACAATCATGGTTTACCTCTGCTGAGACC
SRR517001.21007961.1-   CACTGCTGCGTTTCTCCATCTTCCTGGAGCCTACAATCATGGTTTACCTCTGCTGAGACC
SRR517001.24229462.2-   CACTGCTGCGTTTCTCCATCTTCCTGGAGCCTACAATCATGGTTTACCTCTGCTGAGACC
SRR517001.32526871.1+   CACTGCTGCGTTTCTCCATCTTCCTGGAGCCTACAATCATGGTTTACCTCTGCTGAGACC
SRR517001.54839630.2+   CACTGCTGCGTTTCTCCATCTTCCTGGAGCCTACAATCATGGTTTACCTCTGCTGAGACC
SRR517001.65137644.1-   CACTGCTGCGTTTCTCCATCTTCCTGGAGCCTACAATCATGGTTTACCTCTGCTGAGACC
SRR517001.70785490.2-   CACTGCTGCGTTTCTCCATCTTCCTGGAGCCTACAATCATGGTTTACCTCTGCTGAGACC
SRR517001.99395117.2-   CACTGCTGCGTTTCTCCATCTTCCTGGAGCCTACAATCATGGTTTACCTCTGCTGAGACC
SRR517002.35600907.2-   CACTGCTGCATTTCTCCATCTTCCTGGAGCCTACAATCATGGTTTACCTCTGCTGAGACC
SRR517002.43430166.1-   CACTGCTGCATTTCTCCATCTTCCTGGAGCCTACAATCATGGTTTACCTCTGCTGAGACC
SRR517003.32276918.1+   CACTGCTGCATTTCTCCATCTTCCTGGAGCCTACAATCATGGTTTACCTCTGCTGAGACC
SRR517004.11493321.2-   CACTGCTGCGTTTCTCCATCTTCCTGGAGCCTACAATCATGGTTTACCTCTGCTGAGACC
SRR517004.15232910.1+   CACTGCTGCGTTTCTCCATCTTCCTGGAGCCTACAATCATGGTTTACCTCTGCTGAGACC
SRR517004.15247676.2-   CACTGCTGCGTTTCTCCAGCTTCCTGGAGCCTACAATCATGGTTTCCCTCTGCTGAGACC
SRR517004.18294620.1+   CACTGCTGCGTTTCTCCATCTTCCTGGAGCCTACAATCATGGTTTACCTCTGCTGAGACC
SRR517004.38117563.1-   CACTGCTGCGTTTCTCCATCTTCCTGGAGCCTACAATCATGGTTTACCTCTGCTGAGACC
SRR517004.39340770.2-   CACTGCTGCGTTTCTCCATCTTCCTGGAGCCTACAATCATGGTTTACCTCTGCTGAGACC
SRR517004.57378084.1+   CACTGCTGCGTTTCTCCATCTTCCTGGAGCCTACAATCATGGTTTACCTCTGCTGAGACC
SRR517005.29004911.1-   CACTGCTGCATTTCTCCATCTTCCTGGAGCCTACAATCATGGTTTACCTCTGCTGAGACC
SRR517005.36327145.1+   CACTGCTGCATTTCTCCATCTTCCTGGAGCCTACAATCATGGTTTACCTCTGCTGAGACC
SRR517005.3636097.2-   CACTGCTGCATTTCTCCATCTTCCTGGAGCCTACAATCATGGTTTACCTCTGCTGAGACC
SRR517005.38635366.1-   CACTGCTGCATTTCTCCATCTTCCTGGAGCCTACAATCATGGTTTACCTCTGCTGAGACC
SRR517005.71623618.2-   CACTGTTGCGTTTCTCCATCATCCTGGAGCCTACAATCATGGTTTACCTCTGCTGAGACC
SRR517005.92632228.1+   CACTGCTGCATTTCTCCATCTTCCTGGAGCCTACAATCATGGTTTACCTCTGCTGAGACC
SRR517006.3014885.2+   CACTGCTGCATTTCTCCATCTTCCTGGAGCCTACAATCATGGTTTACCTCTGCTGAGACC
SRR517006.42774934.1-   CACTGCTGCATTTCTCCATCTTCCTGGAGCCTACAATCATGGTTTACCTCTGCTGAGACC
SRR517006.60516129.2-   CACTGCTGCATTTCTCCATCTTCCTGGAGCCTACAATCATGGTTTACCTCTGCTGAGACC
SRR517006.63434435.1-   CACTGCTGCATTTCTCCATCTTCCTGGAGCCTACAATCATGGTTTACCTCTGCTGAGACC
SRR517006.7636423.2-   CACTGCTGCATTTCTCCATCTTCCTGGAGCCTACAATCATGGTTTACCTCTGCTGAGACC
SRR517006.92771968.1+   CACTGCTGCATTTCTCCATCTTCCTGGAGCCTACAATCATGGTTTACCTCTGCTGAGACC
SRR517007.27000447.1-   CACTGCTGCATTTCTCCATCTTCCTGGAGCCTACAATCATGGTTTACCTCTGCTGAGACC
SRR517007.46550915.1+   CACTGCTGCATTTCTCCATCTTCCTGGAGCCTACAATCATGGTTTACCTCTGCTGAGACC
SRR517007.4964232.2+   CACTGCTGCATTTCTCCATCTTCCTGGAGCCTACAATCATGGTTTACCTCTGCTGAGACC
SRR517007.75509732.1+   CACTGCTGCATTTCTCCATCTTCCTGGAGCCTACAATCATGGTTTACCTCTGCTGAGACC
SRR517008.17734492.1+   CACTGCTGCGTTTCTCCATCTTCCTGGAGCCTACAATCATGGTTTACCTCTGCTGAGACC
SRR517008.30759488.1+   CACTGCTGCGTTTCTCCATCTTCCTGGAGCCTACAATCATGGTTTACCTCTGCTGAGACC
SRR517008.31103292.1-   CACTGCTGCGTTTCTCCATCTTCCTGGAGCCTACAATCATGGTTTACCTCTGCTGAGACC
SRR517008.46362816.2-   CACTGCTGCGTTTCTCCATCTTCCTGGAGCCTACAATCATGGTTTACCTCTGCTGAGACC
SRR517008.47368909.1+   CACTGCTGCGTTTCTCCATCTTCCTGGAGCCTACAATCATGGTTTACCTCTGCTGAGACC
SRR517008.53562741.2+   CACTGCTGCGTTTCTCCATCTTCCTGGAGCCTACAATCATGGTTTACCTCTGCTGAGACC
SRR517008.72452430.1-   CACTGCTGCGTTTCTCCATCTTCCTGGAGCCTACAATCATGGTTTACCTCTGCTGAGACC
SRR517009.22763744.1+   CACTGCTGCGTTTCTCCATCTTCCTGGAGCCTACAATCATGGTTTACCTCTGCTGAGACC
SRR517009.32725362.1-   CACTGCTGCGTTTCTCCATCTTCCTGGAGCCTACAATCATGGTTTACCTCTGCTGAGACC
SRR517009.37989736.1+   CACTGCTGCGTTTCTCCATCTTCCTGGAGCCTACAATCATGGTTTACCTCTGCTGAGACC

SRR517009.39648041.1+   CACTGCTGCGTTTCTCCATCTTCCTGGAGCCTACAATCATGGTTTACCTCTGCTGAGACC
SRR517009.41211152.1-   CACTGCTGCGTTTCTCCATCTTCCTGGAGCCTACAATCATGGTTTACCTCTGCTGAGACC
SRR517009.58624417.1-   CACTGCTGCGTTTCTCCATCTTCCTGGAGCCTACAATCATGGTTTACCTCTGCTGAGACC
SRR511913.20105001.2+   ACTGCTGCATTTCTCCATCTTCCTGGAGCCTACAATCATGGTTTACCTCTGCTGAGACC
SRR511913.20105241.2+   ACTGCTGCATTTCTCCATCTTCCTGGAGCCTACAATCATGGTTTACCTCTGCTGAGACC
SRR516983.45197941.1+   ACTGCTGCATTTCTCCATCTTCCTGGAGCCTACAATCATGGTTTACCTCTGCTGAGACC
SRR516989.45104008.1-   ACTGCTGCATTTCTCCATCTTCCTGGAGCCTACAATCATGGTTTACCTCTGCTGAGACC
SRR516992.26374669.1+   ACTGCTGCGTTTCTCCATCTTCCTGGAGCCTACAATCATGGTTTACCTCTGCTGAGACC
SRR516996.46495058.2-   ACTGCTGCATTTCTCCATCTTCCTGGAGCCTACAATCATGGTTTACCTCTGCTGAGACC
SRR516999.29788274.1+   ACTGCTGCATTTCTCCATCTTCCTGGAGCCTACAATCATGGTTTACCTCTGCTGAGACC
SRR517001.18025361.1+   ACTGCTGCGTTTCTCCATCTTCCTGGAGCCTACAATCATGGTTTACCTCTGCTGAGACC
SRR517002.51898282.2-   ACTGCTGCATTTCTCCATCTTCCTGGAGCCTACAATCATGGTTTACCTCTGCTGAGACC
SRR517009.39987367.2-   ACTGCTGCGTTTCTCCATCTTCCTGGAGCCTACAATCATGGTTTACCTCTGCTGAGACC
SRR511892.53446249.2+   CTGCTGCATTTCTCCATCTTCCTGGAGCCTACAATCATGGTTTACCTCTGCTGAGACC
SRR516967.125704511.1-   CTGCTGCATTTCTCCATCTTCCTGGAGCCTACAATCATGGTTTACCTCTGCTGAGACC
SRR516977.56066574.1+   CTGCTGCGTTTCTCCATCTTCCTGGAGCCTACAATCATGGTTTACCTCTGCTGAGACC
SRR516982.24144878.1-   CTGCTGCGTTTCTCCATCTTCCTGGAGCCTACAATCATGATTTACCTCTGCTGAGACC
SRR516995.48904445.1-   CTGCTGCGTTTCTCCATCTTCCTGGAGCCTACAATCATGGTTTACCTCTGCTGAGACC
SRR516998.74061869.1-   CTGCTGCGTTTCCCCATCTGCCTGGAGCCTACAATCATGGTTTACCTCTGCTGAGACC
SRR516967.6221170.1-   TGCTGCATTTCTCCATCTTCCTGGAGCCTACAATCATGGTTTACCTCTGCTGAGACC
SRR516974.46978934.2+   TGCTGCATTTCTCCATCTTCCTGGAGCCTACAATCATGGCTTACCTCTGCTGAGACC
SRR516975.49935850.2+   TGCTGCATTTCTCCATCTTCCTGGAGCCTACAATCATGGTTTACCTCTGCTGAGACC
SRR516979.61681085.2+   TGCTGCGTTTCTCCATCTTCCTGGAGCCTACAATCATGGTTTACCTCTGCTGAGACC
SRR516983.89585195.2+   TGCTGCATTTCTCCATCTTCCTGGAGCCTACAATCATGGTTTACCTCTGCTGAGACC
SRR516991.41770914.1-   TGCTGCGTTTCTCCATCTTCCTGGAGCCTACAATCATGATTTACCTCTGCTGAGACC
SRR511913.25527344.1+   GCTGCATTTCTCCATCTTCCTGGAGCCTACAATCATGGTTTACCTCTGCTGTGACC
SRR516968.39655396.2+   GCTGCATTTCTCCATCTTCCTGGAGCCTACAATCATGGTTTACCTCTGCTGAGACC
SRR516998.54361396.1+   GCTGCATTTCTCCATCTTCCTGGAGCCTACAATCATGGTTTACCTCTGCTGAGACC
SRR516999.99949277.1-   GCTGCATTTCTCCATCTTCCTGGAGCCTACAATCATGGTTTACCTCTGCTGAGACC
SRR517009.6644834.2-   GCTGCGTTGCTCCATCTTCCTGGAGCCTACAATCATGGTTTACCTCTGCTGAGACC
SRR516976.6204118.1+   CTGCGTTTCTCCATCTTCCTGGAGCCTACAATCATGGTTTACCTCTGCTGAGACC
SRR516980.42600802.2-   CTGCGTTCCTCCATCTTCCTGGAGCCTACAATCATGGTTTCCCTCTGCTGAGACC
SRR516990.6461511.1+   CTGCATTTCTCCATCTTCCTGGAGCCTACAATCATGGTTTACCTCTGCTGAGACC
SRR516991.19768136.1+   CTGCATTTCTCCATCTTCCTGGAGCCTACAATCATGGTTTACCTCTGCTGAGACC
SRR516994.26780695.2+   CTGCGTTTCTCCATCTTCCTGGAGCCTACAATCATGGTTTACCTCTGCTGAGACC
SRR516994.30789737.1+   CTGCGTTTCTCCATCTTCCTGGAGCCTACAATCATGGTTTACCTCTGCTGAGACC
SRR511916.56328020.2+   TGCATTTCTCCATCTTCCTGGAGCCTACAATCATGGTTTACCTCTG
SRR516974.54299546.1+   TGCATTTCTCCATCTTCCTGGAGCCTACAATCATGGTTTACCTCTGCTGAGACC
SRR516977.65083064.2+   TGCGTTTCTCCATCTTCCTGGAGCCTACAATCATGGTTTACCTCTGCTGAGACC
SRR516982.31054443.1-   TGCGTTTCTCCATCTTCCTGGAGCCTACAATCATGATTTACCTCTGCTGAGACC
SRR516983.15530144.1+   TGCATTTCTCCATCTTCCTGGAGCCTACAATCATGGTTTACCTCTGCTGAGACC
SRR516985.1978329.2-   TGCGTTTCTCCATCTTCCTGGAGCCTACAATCATGGTTTACCTCTGCTGAGACC
SRR516996.18713822.1-   TGCATTTCTCCATCTTCCTGGAGCCTACAATCATGGTTTACCTCTGCTGAGACC
SRR516997.5355646.1-   TGCATTTCTCCATCTTCCTGGAGCCTACAATCATGGTTTACCTCTGCTGAGACC
SRR516999.108304884.1+   TGCATTTCTCCATCTTCCTGGAGCCTACAATCATGGTTTACCTCTGCTGAGACC
SRR517001.3087830.1+   TGCGTTTCTCCATCTTCCTGGAGCCTACAATCATGGTTTACCTCTGCTGAGACC
SRR517004.41718493.2+   TGCGTTTCTCCATCTTCCTGGAGCCTACAATCATGGTTTACCTCTGCTGAGACC
SRR516973.25012163.1-   GCATTTCTCCATCTTCCTGGAGCCTACAATCATGGTTTACCTCTGCTGAGACC
SRR516978.43288050.1-   GCGTTTCTCCATCTTCCTGGAGCCTACAATCATGGTTTACCTCTGCTGAGACC
SRR516980.5795258.2-   GCGTTTCTCCATCTTCCTGGAGCCTACAATCATGGTTTACCTCTGCTGAGACC
SRR516992.11057517.2-   GCATTTCTCCATCTTCCTGGAGCCTACAATCATGGTTTACCTCTGCTGAGACC
SRR511915.47098295.1+   CATTTCTCCATCTTCCTGGAGCCTACAATCATGGTTTACCTCTGCTGAGACC
SRR516968.145184287.2-   CATTTCTCCATCTTCCTGGAGCCTACAATCATGGTTTACCTCTGCTGAGACC
SRR516968.15347757.2-   CGTTTCTCCATCTTCCTGGAGCCTACAATCATGGTTTACCTCTGCTGAGACC
SRR516973.63984660.2+   CGTTTCTCCATCTTCCTGGAGCCTACAATCATGATTTACCTCTGCTGAGACC
SRR516978.9966331.1-   CGTTTCTCCATCTTCCTGGAGCCTACAATCATGGTTTACCTCTGCTGAGACC
SRR516985.58077220.2+   CGTTTCTCCATCTTCCTGGAGCCTACAATCATGGTTTACCTCTGCTGAGACC
SRR516996.71975786.2-   CATTTCTCCATCTTCCTGGAGCCTACAATCATGGTTTACCTCTGCTGAGACC
SRR517001.103617359.1+   CGTTTCTCCATCTTCCTGGAGCCTACAATCATGGTTTACCTCTGCTGAGACC
SRR517001.22577401.1-   CGTTTCTCCATCTTCCTGGAGCCTACAATCATGGTTTACCTCTGCTGAGACC
SRR511912.34406775.1-   ATTTCTCCATCTTCCTGGAGCCTACAATCATGGTTTACCTCTGCTGAGACC
SRR516976.12584702.2+   GTTTCTCCATCTTCCTGGAGCCTACAATCATGGTTTACCTCTGCTGAGACC
SRR516987.5180076.1-   GTTTCTCCATCTTCCTGGAGCCTACAATCATGATTTACCTCTGCTGAGACC
SRR516990.58587583.1-   ATTTCTCCATCTTCCTGGAGCCTACAATCATGGTTTACCTCTGCTGAGACC
SRR516991.44385216.1+   GTTTCTCCATCTTCCTGGAGCCTACAATCATGATTTACCTCTGCTGAGACC
SRR516995.80032724.1-   GTTTCTCCATCGTCCTGGAGCCTACAATCATGGTTTACCTCTGCTGAGACC
SRR511892.28064592.2-   TTTCTCCATCTTCCTGGAGCCTACAATCATGGTTTACCTCTGCTGAGACC
SRR516969.26985112.1+   TTTCTCCATCTTCCTGGAGCCTACAATCATGGTTTACCTCTGCTGAGACC
SRR516970.19833631.2-   TTTCTCCATCTTCCTGGAGCCTACAATCATGGTTTACCTCTGCTGAGACC
SRR516987.6864091.2-   TTTCTCCATCTTCCTGGAGCCTACAATCATGATTTACCTCTGCTGAGACC
SRR516989.43322421.2+   TTTCTCCATCTTCCTGGAGCCTACAATCATGGTTTACCTCTGCTGAGACC
SRR516993.55737172.2-   TTTCTCCATCTTCCTGGAGCCTACAATCATGGTTTACCTCTGCTGAGACC
SRR516993.83112824.1+   TTTCTCCATCTTCCTGGAGCCTACAATCATGGTTTACCTCTGCTGAGACC
SRR516994.61678342.1+   TTTCTCCATCTTCCTGGAGCCTACAATCATGGTTTACCTCTGCTGAGACC
SRR516997.96113566.1-   TTTCTCCATCTTCCTGGAGCCTACAATCATGGTTTACCTCTGCTGAGACC
SRR517002.26924755.2+   TTTCTCCATCTTCCTGGAGCCTACAATCATGGTTTACCTCTGCTGAGACC
SRR517005.50448398.2+   TTTCTCCATCTTCCTGGAGCCTACAATCATGGTTTACCTCTGCTGAGACC
SRR511911.29777200.1-   TTCTCCATNTGCCTGGAGCCTACAATCATGGTTTACCTCTGCTGAGACC
SRR511913.75322811.2-   TTCTCCATCTTCCTGGAGCCTACAATCATGGTTTACCTCTGCTGAGACC
SRR516977.63372655.1+   TTCTCCATCTTCCTGGAGCCTACAATCATGGTTTACCTCTGCTGAGACC
SRR516981.4478016.2-   TTCTCCATCTTCCTGGAGCCTACAATCATGGTTTACCTCTGCTTAGACC
SRR517001.46019341.1+   TTCTCCATCTTCCTGGAGCCTACAATCATGGTTTACCTCTGCTGAGACC
SRR517003.9557651.2+   TTCTCCATCTTCCTGGAGCCTACAATCATGGTTTACCTCTGCTGAGACC
SRR516973.57501785.2-   TCTCCATCTTCCTGGAGCCTACAATCATGATTTACCTCTGCTGAGACC

```
SRR516981.55431738.2+                TCTCCATCTTCCTGGAGCCTACAATCATGGTTTACCTCTGCTGAGACC
SRR516982.67688043.2+                TCTCCATCTTCCTGGAGCCTACAATCATGATTTACCTCTGCTGAGACC
SRR516983.95786046.1+                TCTCCATCTTCCTGGAGCCTACAATCATGGTTTACCTCTGCTGAGACC
SRR516991.16236160.2+                TCTCCATCTTCCTGGAGCCTACAATCATGATTTACCTCTGCTGAGACC
SRR516996.49068306.1-                TCTCCATCTTCCTGGAGCCTACAATCATGGTTTACCTCTGCTGAGACC
SRR516998.94026502.1+                TCTCCATCTTCCTGGAGCCTACAATCATGGTTTACCTCTGCTGAGACC
SRR517000.23065522.1+                TCTCCATCTTCCTGGAGCCTACAATCATGGTTTACCTCTGCTGAGACC
SRR517002.88240524.1+                TCTCCATCTTCCTGGAGCCTACAATCATGGTTTACCTCTGCTGAGACC
SRR511892.41096124.2-                 CTCCATCTTCCTGGAGCCTACAATCATGGTTTACCTCTGCTGAGACC
SRR511911.14454908.1-                 CTCCATCTTCCTGGAGCCTACAATCATGGTTTACCTCTGCTGAGACC
SRR516983.46519373.2-                 CTCCATCTTCCTGGAGCCTACAATCATGGTTTACCTCTGCTGAGACC
SRR516983.96419424.1-                 CTCCATCTTCCTGGAGCCTACAATCATGGTTTACCTCTGCTGAGACC
SRR516996.25489938.2-                 CTCCATCTTCCTGGAGCCTACAATCATGGTTTACCTCTGCTGAGACC
SRR517003.43535674.1+                 CTCCATCTTCCTGGAGCCTACAATCATGGTTTACCTCTGCTGAGACC
SRR517006.48499023.1+                 CTCCATCTTCCTGGAGCCTACAATCATGGTTTACCTCTGCTGAGACC
SRR511911.21547512.1+                  TCCATCTTCCTGGAGCCTACAATCATGGTTTACCTCTGCTGAGACC
SRR516968.100131508.2+                 TCCATCTTCCTGGAGCCTACAATCATGGTTTACCTCTGCTGAGACC
SRR516970.20795711.2-                  TCCATCTTCCTGTGGCCTACAATCGTGGTTTACCTCTGCTGAGACC
SRR516975.46689364.1+                  TCCATCTTCCTGGAGCCTACAATCATGGTTTACCTCTGCTGAGACC
SRR516998.101916190.1-                 TCCATCTTCCTGGAGCCTACAATCATGGTTTACCTCTGCTGAGACC
SRR517000.91504937.1+                  TCCATCTTCCTGGAGCCTACAATCATGGTTTACCTCTGCTGAGACC
SRR517001.900897.1+                    TCCATCTTCCTGGAGCCTACAATCATGGTTTACCTCTGCTGAGACC
SRR517007.25961977.2-                  TCCATCTTCCTGGAGCCTACAATCATGGTTTACCTCTGCTGAGACC
SRR517008.21405715.2-                  TCCATCTTCCTGGAGCCTACAATCATGGTTTACCTCTGCTGAGACC
SRR517009.83112268.1+                  TCCATCTTCCTGGAGCCTACAATCATGGTTTACCTCTGCTGAGACC
SRR511892.65397717.1-                   CCATCTTCCTGGAGCCTACAATCATGGTTTACCTCTGCTGAGACC
SRR516967.87829969.2-                   CCATCTTCCTGGAGCCTACAATCATGGTTGACCTCTGCTGAGACC
SRR516984.44849129.2+                   CCATCTTCCTGGAGCCTACAATCATGGTTTACCTCTGCTGAGACC
SRR516986.35116064.1+                   CCATCTTCCTGGAGCCTACAATCATGGTTTACCTCTGCTGAGACC
SRR516995.55914942.1-                   CCATCTTCCTGGAGCCTACAATCATGGTTTACCTCTGCTGAGACC
SRR517000.73971487.1+                   CCATCTTCCTGGAGCCTACAATCATGGTTTACCTCGGCTGAGACC
SRR511912.30395815.2+                    CATCTTCCTGGAGCCTACAATCATGGTTTACCTCTGCTGAGACC
SRR516972.84821035.1-                    CATCTTCCTGGAGCCTACAATCATGGTTTACCTCTGCTGAGACC
SRR516974.64263409.1-                    CATCTTCCTGGAGCCTACAATCATGGTTTACCTCTGCTGAGACC
SRR516980.6938514.1+                     CATCTTCCTGGAGCCTACAATCATGGTTTACCTCTGCTGAGACC
SRR516987.45900545.1+                    CATCTTCCTGGAGCCTACAATCATGATTTACCTCTGCTGAGACC
SRR516987.74463633.2+                    CATCTTCCTGGAGCCTACAATCATGGTTTACCTCTGCTGAGACC
SRR516999.1489221.1-                     CATCTTCCTGGAGCCTACAATCATGGTTTACCTCTGCTGAGACC
SRR516999.15834111.2-                    CATCTTCCTGGAGCCTACAATCATGGTTTACCTCTGCTGAGACC
SRR517008.35566824.2+                    CATCTTCCTGGAGCCTACAATCATGGTTTACCTCTGCTGAGACC
SRR511892.39300111.1-                     ATCTTCCTGGAGCCTACAATCATGGTTTACCTCTGCTGAGACC
SRR516968.109083542.1-                    ATCTTCCTGGAGCCTACAATCATGGTTTACCTCTGCTGAGACC
SRR516968.140796502.1-                    ATCTTCCTGGAGCCTACAATCATGGTTTACCTCTGCTGAGACC
SRR516972.17444908.2-                     ATCTTCCTGGAGCCTACAATCATGGTTTACCTCTGCTGAGACC
SRR516972.29398207.1-                     ATCTTCCTGGAGCCTACAATCATGGTTTACCTCTGCTGAGACC
SRR516977.18216728.2+                     ATCTTCCTGGAGCCTACAATCATGGTTTACCTCTGCTGAGACC
SRR516977.77183349.2+                     ATCTTCCTGGAGCCTACAATCATGGTTTACCTCTGCTGAGACC
SRR516979.10219896.2+                     ATCTTCCTGGAGCCTACAATCATGGTTTACCTCTGCTGAGACC
SRR516985.43613602.2+                     ATCTTCCTGGAGCCTACAATCATGGTTTACCTCTGCTGAGACC
SRR516993.60327009.1-                     ATCTTCCTGGAGCCTACAATCATGGTTTACCTCTGCTGAGACC
SRR516997.92588309.1-                     ATCTTCCTGGAGCCTACAATCATGGTTTACCTCTGCTGAGACC
SRR516998.96836905.2-                     ATCTTCCTGGAGCCTACAATCATGGTTTACCTCTGCTGAGACC
SRR516974.49070472.1-                      TCTTCCTGGAGCCTACAATCATGGTTTACCTCTGCTGAGACC
SRR516975.64845215.1-                      TCTTCCTGGAGCCTACAATCATGGTTTACCTCTGCTGAGACC
SRR516993.85425731.1-                      TCTTCCTGGAGCCTACAATCATGGTTTACCTCTGCTGAGACC
SRR517000.15878174.2-                      TCTTCCTGGAGCCTACAATCATGGTTTACCTCTGCTGAGACC
SRR517000.27952472.1+                      TCTTCCTGGAGCCTACAATCATGGTTTACCTCTGCTGAGACC
SRR517000.52705672.2+                      TCTTCCTGGAGCCTACAATCATGGTTTACCTCTGCTGAGACC
SRR517004.46455486.1-                      TCTTCCTGGAGCCTACAATCATGGTTTCCCTCTGCTGAGACC
SRR511912.63931928.2+                       CTTCCTGGAGCCTACAATCATGGTTTACCTCTGCTGAGACC
SRR511914.1000674.2+                        CTTCCTGGAGCCTACAATCATGGTTTACCTCTGCTGAGACC
SRR516967.83672796.1-                       CTTCCTGGAGCCTACAATCATGGTTTACCTCTGCTGAGACC
SRR516974.13682622.1+                       CTTCCTGGAGCCTACAATCATGGTTTACCTCTGCTGAGACC
SRR516976.30705361.2-                       CTTCCTGGAGCCTACAATCATGGTTTACCTCTGCTGAGACC
SRR516977.79316740.2-                       CTTCCTGGAGCCTACAATCATGGTTTACCTCTGCTGAGACC
SRR516979.63870114.2-                       CTTCCTGGAGCCTACAATCATGGTTTACCTCTGCTGAGACC
SRR516990.30827191.1+                       CTTCCTGGAGCCTACAATCATGGTTTACCTCTGCTGAGACC
SRR516997.85778454.1+                       CTTCCTGGAGCCTACAATCATGATTTACCTCTGCTGAGACC
SRR517003.47760361.1-                       CTTCCTGGAGCCTACAATCATGGTTTACCTCTGCTGAGACC
SRR517009.80280823.1-                       CTTCCTGGAGCCTACAATCATGGTTTACCTCTGCTGAGACC
SRR511914.45719171.1+                        TTCCTGGAGCCTACAATCATGGTTTACCTCTGCTGAGACC
SRR516967.121468181.1-                       TTCCTGGAGCCTACAATCATGGTTTACCTCTGCTGAGACC
SRR516987.59068064.1-                        TTCCTGGAGCCTACAATCATGGTTTACCTCTGCTGAGACC
SRR516992.47548931.2+                        TTCCTGGAGCCTACAATCATGGTTTACCTCTGCTGAGACC
SRR517000.49039794.2-                        TTCCTGGAGCCTACAATCATGGTTTACCTCTGCTGAGACC
SRR517000.59218275.1+                        TTCCTGGAGCCTACAATCATGGTTTACCTCTGCTGAGACC
SRR517000.86755698.2-                        TTCCTGGAGCCTACAATCATGGTTTACCTCTGCTGAGACC
SRR517005.68411118.2+                        TTCCTGGAGCCTACAATCATGGTTTACCTCTGCTGAGACC
SRR516968.40141403.1-                         TCCTGGAGCCTACAATCATGGTTTACCTCTGCTGAGAGC
SRR516991.45959728.2-                         TCCTGGAGCCTACAATCATGATTTACCTCTGCTGAGACC
SRR516992.5218916.1-                          TCCTGGAGCCTACAATCATGGTTTACCTCTGCTGAGACC
SRR516996.43104832.1-                         TCCTGGAGCCTACAATCATGGTTTACCTCTGCTGAGACC
SRR516997.19843563.2+                         TCCTGGAGCCTACAATCATGGTTTACCTCTGCTGAGACC
SRR516998.77015275.2+                         TCCTGGAGCCTACAATCATGGTTTACCTCTGCTGAGACC
```

```
SRR516999.56136092.1+    TCCTGGAGCCTACAATCATGGTTTACCTCTGCTGAGACC
SRR517001.87349540.2-    TCCTGGAGCCTACAATCATGGTTTACCTCTGCTGAGACC
SRR511912.20243841.1-     CCTGGAGCCTACAATCATGGTTTACCTCTGCTGAGACC
SRR516968.106610860.1-    CCTGGAGCCTACAATCATGGTTTACCTCTGCTGAGACC
SRR516979.67226952.1+     CCTGGAGCCTACAATCATGGTTTACCTCTGCTGAGACC
SRR516986.21942167.1+     CCTGGAGCCTACAATCATGGTTTACCTCTGCTGAGACC
SRR516998.58191126.1+     CCTGGAGCCTACAATCATGGTTTACCTCTGCTGAGACC
SRR517000.42847360.2-     CCTGGAGCCTACAATCATGGTTTACCTCTGCTGAGACC
SRR517003.50373066.2+     CCTGGAGCCTACAATCATGGTTTACCTCTGCTGAGACC
SRR517003.66035176.2+     CCTGGAGCCTACAATCATGGTTTACCTCTGCTGAGACC
SRR517009.44679586.2-     CCTGGAGCCTACAATCATGGTTTACCTCTGCTGAGACC
SRR511913.72838419.1+      CTGGAGCCTACAATCATGGTTTACCTCTGCTGAGACC
SRR511914.19779621.1-      CTGGAGCCTACAATCATGGTTTACCTCTGCTGAGACC
SRR511918.12327870.2+      CTGGAGCCTACAATCATGGTTTACCTCTGCTGAGACC
SRR511918.22802077.2+      CTGGAGCCTACAATCATGGTTTACCTCTGCTGAGACC
SRR511918.6049292.2+       CTGGAGCCTACAATCATGGTTTACCTCTGCTGAGACC
SRR516976.6568357.2+       CTGGAGCCTACAATCATGATTTACCTCTGCTGAGACC
SRR516980.29666170.2+      CTGGAGCCTACAATCATGGTTTACCTCTGCTGAGACC
SRR516985.12986836.1+      CTGGAGCCTACAATCATGGTTTACCTCTGCTGAGACC
SRR516989.7050322.1-       CTGGAGCCTACAATCATGGTTTACCTCTGCTGAGACC
SRR516998.65330415.1-      CTGGAGCCTACAATCATGGTTTACCTCTGCTGAGACC
SRR511915.51271900.2+       TGGAGCCTACAATCATGGTTTACCTCTGCTGAGACC
SRR511916.29120870.1+       TGGAGCCTACAATCATGGTTTACCTCTGCTGAGACC
SRR516967.115389681.2+      TGGAGCCTACAATCATGGTTTACCTCTGCTGAGACC
SRR516985.56923558.1+       TGGAGCCTACAATCATGGTTTACCTCTGCTGAGACC
SRR516987.91428967.1-       TGGAGCCTACAATCATGATTTACCTCTGCTGAGACC
SRR516998.39972283.1+       TGGAGCCTACAATCATGGTTTACCTCTGCTGAGACC
SRR516998.60100976.2+       TGGAGCCTACAATCATGGTTTACCTCTGCTGAGACC
SRR516998.76618156.1+       TGGAGCCTACAATCATGGTTTACCTCTGCTGAGACC
SRR517001.26973912.2-       TGGAGCCTACAATCATGGTTTACCTCTGCTGAGACC
SRR517006.28491749.1+       TGGAGCCTACAATCATGGTTTACCTCTGCTGAGACC
SRR516967.148303398.2-       GGAGCCTACAATCATGGTTTACCTCTGCTGAGACC
SRR516994.74877170.1+        GGAGCCTACAATCATGGTTTACCTCTGCTGAGACC
SRR516996.88052646.2+        GGAGCCTACAATCATGGTTTACCTCTGCTGAGACC
SRR516998.13962236.2-         GAGCCTACAATCATGGTTTACCTCTGCTGAGACC
SRR517000.29169337.1-         GAGCCTACAATCATGGTTTACCTCTGCTGAGACC
SRR517005.99973486.1-         GAGCCTACAATCATGGTTTACCTCTGCTGAGACC
SRR517008.11106831.2+         GAGCCTACAATCATGGTTTACCTCTGCTGAGACC
SRR517009.71578868.2-         GAGCCTACAATCATGGTTTACCTCTGCTGAGACC
SRR511915.56868915.2-          AGCCTACAATCATGGTTTACCTCTGCTGAGACC
SRR516967.94416127.1-          AGCCTACAATCATGGTTTACCTCTGATGAGACC
SRR516971.15050413.1-          AGCCTACAATCATGGTTTACCTCTGCTGAGACC
SRR516989.78968928.1+          AGCCTACAATCATGGTTTACCTCTGCTGAGACC
SRR516993.72207497.1-          AGCCTACAATCATGGTTTACCTCTGCTGAGACC
SRR517008.87918252.1+          AGCCTACAATCATGGTTTACCTCTGCTGAGACC
SRR511911.38218078.2-           GCCTACAATCATGGTTTACCTCTGCTGAGACC
SRR516973.20554683.1-           GCCTACAATCATGGTTTACCTCTGCTGAGACC
SRR516973.35471726.2+           GCCTACAATCATGATTTACCTCTGCTGAGACC
SRR516973.35471727.2+           GCCTACAATCATGATTTACCTCTGCTGAGACC
SRR516982.30415927.2-           GCCTACAATCATGATTTACCTCTGCTGAGACC
SRR516996.24708888.2+           GCCTACAATCATGGTTTACCTCTGCTGAGACC
SRR517004.31599976.2-           GCCTACAATCATGGTTTACCTCTGCTGAGACC
SRR517004.39008760.1-           GCCTACAATCATGGTTTACCTCTGCTGAGACC
SRR511916.21038728.2+            CCTACAATCATGGTTTACCTCTGCTGAGACC
SRR511918.21279607.2-            CCTACAATCATGGTTTACCTCTGCTGAGACC
SRR516968.43850003.2-            CCTACAATCATGGTTTACCTCTGCTGAGACC
SRR516987.61859958.2-            CCTACAATCATGATTTACCTCTGCTGAGACC
SRR516989.36199378.2+            CCTACAATCATGGTTTACCTCTGCTGAGACC
SRR516989.52379004.2-            CCTACAATCATGGTTTACCTCTGCTGAGACC
SRR517002.27629774.1+            CCTACAATCATGGTTTACCTCTGCTGAGACC
SRR516967.100239201.1+            CTACAATCATGGTTTACCTCTGCTGAGACC
SRR516968.54367312.2+             CTACAATCATGGTTTACCTCTGCTGAGACC
SRR516982.23500814.2+             CTACAATCATGATTTACCTCTGCTGAGACC
SRR516982.56632493.1+             CTACAATCATGATTTACCTCTGCTGAGACC
SRR516993.83529308.2+             CTACAATCATGGCTTACCTCTGCTGAGACC
SRR517002.48542308.2-             CTACAATCATGGTTTACCTCTGCTGAGACC
SRR517003.48327823.1+             CTACAATCATGGTTTACCTCTGCTGAGACC
SRR516976.50893891.2-              TACAATCATGGTTTACCTCTGCTGAGACC
SRR516984.50030865.2+              TACAATCATGGTTTACCTCTGCTGAGACC
SRR516988.2929307.2-               TACAATCATGGTTTACCTCTGCTGAGACC
SRR517000.23191480.1+              TACAATCATGGTTTACCTCTGCTGAGACC
SRR517003.42514893.2-              TACAATCATGGTTTACCTCTGCTGAGACC
SRR517006.79064283.2-              TACAATCATGGTTTACCTCTGCTGAGACC
SRR517007.20187658.2-              TACAATCATGGTTTACCTCTGCTGAGACC
SRR511916.58161197.1+               ACAATCATGGTTTACCTCTGCTGAGACC
SRR516967.135591639.1+              ACAATCATGGTTTACCTCTGCTGAGACC
SRR516967.57741754.1+               ACAATCATGGTTTACCTCTGCTGAGACC
SRR516973.4884301.2-                ACAATCATGGTTTACCTCTGCTGAGACC
SRR516978.1992911.1+                ACAATCATGGTTTACCTCTGCTGAGACC
SRR516988.32844820.2-               ACAATCATGGTTTACCTCTGCTGAGACC
SRR517001.31582093.1-               ACAATCATGGTTTACCTCTGCTGAGACC
SRR517002.29394887.2+               ACAATCATGGTTTACCTCTGCTGAGACC
SRR517009.28589393.1-               ACAATCATGGTTTACCTCTGCTGAGACC
SRR516968.108661732.1-               CAATCATGGTTTACCTCTGCTGAGACC
```

SRR516977.75947199.2+	CAATCATGGTTTACCTCTGCTGAGACC
SRR516989.46904169.2+	CAATCATGGTTTACCTCTGCTGAGACC
SRR516996.36234607.1+	CAATCATGGTTTACCTCTGCTGAGACC
SRR517003.73936435.2-	CAATCATGGTTTACCTCTGCTGAGACC
SRR517004.53693916.2-	CAATCATGTTTTACCTCTGCTGAGACC
SRR517008.37417547.1+	CAATCATGGTTTACCTCTGCTGAGACC
SRR511917.45677079.2-	AATCATGGTTTACCTCTGCTGAGACC
SRR511917.69281775.2-	AATCATGGTTTACCTCTGCTGAGACC
SRR516991.30740799.1+	AATCATGGTTTACCTCTGCTGAGACC
SRR516993.49833109.2-	AATCATGGTTTACCTCTGCTGAGACC
SRR517001.16403167.2-	AATCATGGTTTACCTCTGCTGAGACC
SRR517001.89049728.2+	AATCATGGTTTACCTCTGCTGAGACC
SRR517007.5128092.2-	AATCATGGTTTACCTCTGCTGAGACC
SRR511915.717767.2+	ATCATGGTTTACCTCTGCTGAGACC
SRR516978.60869002.2+	ATCATGGTTTACCTCTGCTGAGACC
SRR516994.76691379.2-	ATCATGGTTTACCTCTGCTGAGACC
SRR517005.54428488.2+	ATCATGGTTTACCTCTGCTGAGACC
SRR511912.83293905.1+	TCATGGTTTACCTCTGCTGAGACC
SRR516967.138148601.1-	TCATGGTTTACCTCTGCTGAGACC
SRR516968.164894710.1+	TCATGGTTTACCTGTGCTGAGACC
SRR516978.43755925.2-	TCATGGTTTACCTCTGCTGAGACC
SRR517003.63008308.1-	TCATGGTTTACCTCTGCTGAGACC
SRR517005.97095694.1-	TCATGGTTTACCTCTGCTGAGACC
SRR517006.43208825.1-	TCATGGTTTACCTCTGCTGAGACC
SRR517006.8352318.1-	TCATGGTTTACCTCTGCTGAGACC
SRR511913.22705507.2-	CATGGGTTACCTCTGCTGAGACC
SRR516968.8958322.1+	CATGGTTTACCTCTGCTGAGACC
SRR516974.66440091.2-	CATGGTTTACCTCTGCTGAGACC
SRR516975.15347664.1-	CATGATTTACCTCTGCTGAGACC
SRR516975.39714728.2+	CATGATTTACCTCTGCTGAGACC
SRR516976.2015671.2+	CATGATTTACCTCTGCTGAGACC
SRR516980.19111556.1-	CATGGTTTACCTCTGCTGAGACC
SRR516980.32208306.1-	CATGGTTTACCTCTGCTGAGACC
SRR516985.77238548.2-	CATGGTTTACCTCTGCTGAGACC
SRR517000.94486378.2-	CATGGTTTACCTCTGCTGAGACC
SRR517003.87371479.1+	CATGGTTTACCTCTGCTGAGACC
SRR511914.41285120.1-	ATGATTTCCCTCTGCTGAGACC
SRR511914.56122848.1+	ATGGTTTACCTCTGCTGAGACC
SRR511915.73501967.1-	ATGGTTTACCTCTGCTGAGACC
SRR511916.31954449.1+	ATGGTTTACCTCTGCTGAGACC
SRR516974.29282850.1+	ATGGTTTACCTCTGCTGAGACC
SRR516977.10878176.2-	ATGGTTTACCTCTGCTGAGACC
SRR516986.10287761.2+	ATGGTTTACCTCTGCTGAGACC
SRR516991.49815040.2+	ATGATTTACCTCTGCTGAGACC
SRR517003.70967985.2+	ATGGTTTACCTCTGCTGAGACC
SRR517004.40113226.2+	ATGGTTTACCTCTGCTGAGACC
SRR516977.12820030.2+	TGGTTTACCTCTGCTGAGACC
SRR516989.8534348.1-	TGGTTTACCTCTGCTGAGACC
SRR516991.17318347.2-	TGGTTTACCTCTGCTGAGACC
SRR516991.1931100.1-	TGATTTACCTCTGCTGAGACC
SRR516991.24878492.1-	TGGTTTACCTCTGCTGAGACC
SRR516995.74299668.1+	TGGTTTACCTCTGCTGAGACC
SRR517000.37586981.1-	TGGTTTACCTCTGCTGAGACC
SRR517001.36464132.1-	TGGTTTACCTCTGCTGAGACC
SRR517005.886243.1-	TGGTTTACCTCTGCTGAGACC
SRR511911.7460180.1-	GGTTTACCTCTGCTGAGACC
SRR511912.59893234.1+	GGTTTACCTCTGCTGAGACC
SRR516967.34973661.1+	GGTTTACCTCTGCTGAGACC
SRR516975.22417896.2+	GATTTACCTCTGCTGAGACC
SRR516976.36876145.1+	GGTTTACCTCTGCTGAGACC
SRR516979.6140788.2+	GGTTTACCTCTGCTGAGACC
SRR516994.78306274.2+	GGTTTACCTCTGCTGAGACC
SRR516997.5744761.2-	GGTTTACCTCTGCTGAGACC
SRR517002.68785810.2+	GGTTTACCTCTGCTGAGACC
SRR511911.60280636.2+	GTTTACCTCTGCTGAGACC
SRR516996.52900642.2-	GTTTACCTCTGCTGAGACC
SRR516967.55179281.2+	TTTACCTCTGCTGAGACC
SRR516967.63778390.2-	TTTACCTCTGCTGAGACC
SRR516998.57969943.1-	TTTACCTCTGCTGAGACC
SRR516999.61456648.1+	TTTACCTCTGCTGAGACC
SRR517001.8564550.1+	TTTACCTCTGCTGAGACC
SRR511892.6007403.1-	TTACCTCTGCTGAGACC
SRR516968.75435688.1-	TTACCTCTGCTGAGACC
SRR516992.10103608.1+	TTACCTCTGCTGAGACC
SRR516992.79918999.2+	TTACCTCTGCTGAGACC
SRR516993.1447601.1+	TTACCTCTGCTGAGACC
SRR516999.7152611.2+	TTACCTCTGCTGAGACC
SRR517002.8665886.2-	TTACCTCTGCTGAGACC
SRR517005.66404474.1+	TTACCTCTGCTGAGACC
SRR516973.16904597.1-	TACCTCTGCTGAGACC
SRR516973.16906873.1-	TACCTCTGCTGAGACC
SRR516976.57348554.2-	TACCTCTGCTGAGACC
SRR516980.63928031.1-	TACCTCTGCTGAGACC
SRR516989.14777251.1+	TACCTCTGCTGAGACC

SRR517004.34678058.2-	TACCTCTGCTGAGACC
SRR517008.53563245.1-	TACCTCTGCTGAGACC
SRR511912.63185812.1+	ACCTCTGCTGAGACC
SRR511912.63185956.1+	ACCTCTGCTGAGACC
SRR516990.68475801.1+	ACCTCTGCTGAGACC
SRR516994.35326807.2-	ACCTCTGCTGAGACC
SRR516994.82514123.2+	ACCTCTGCTGAGACC
SRR516979.10675489.2+	CCTCTGCTGAGACC
SRR516985.70259889.1-	CCTCTGCTGAGACC
SRR516987.36431135.1-	CCTCTGCTGAGACC
SRR516989.21621378.1+	CCTCTGCTGAGACC
SRR516991.7741780.1-	CCTCTGCTGAGACC
SRR516995.15254844.2-	CCTCTGCTGAGACC
SRR516968.73747636.1-	CTCTGCTGAGACC
SRR516980.37499938.1+	CTCTGCTGAGACC
SRR516983.43684823.1-	CTCTGCTGAGACC
SRR516983.94153626.2+	CTCTGCTGAGACC
SRR516995.43808656.1-	CTCTGCTGAGACC
SRR516997.25946564.1-	CTCTGCTGAGACC
SRR516997.44735578.1-	CTCTGCTGAGACC
SRR516999.61850545.2+	CTCTGCTGAGACC
SRR517000.49710061.1-	CTCTGCTGAGACC
SRR517003.70018023.2-	CTCTGCTGAGACC
SRR511915.12934870.2-	TCTGCTGAGACC
SRR516967.59477310.1-	TCTGCTGAGACC
SRR516975.59637801.2-	TCTGCTGAGACC
SRR516977.57408704.1+	TCTGCTGAGACC
SRR516983.60674262.1-	TCTGCTGAGACC
SRR516989.75746913.1-	TCTGCTGAGACC
SRR516992.63008789.1-	TCTGCTGAGACC
SRR516997.93541116.1-	TCTGCTGAGACC
SRR517007.55411417.1+	TCTGCTGAGACC
SRR517009.31830082.2-	TCTGCTGAGACC
SRR517009.74653167.2-	TCTGCTGAGACC
SRR511892.63390781.2-	CTGCTGAGACC
SRR511892.64175574.2-	CTGCTGAGACC
SRR511917.54829842.1-	CTGCTGAGACC
SRR511917.69338839.1-	CTGCTGAGACC
SRR516978.37638949.1-	CTGCTGAGACC
SRR516979.1430425.1-	CTGCTGAGACC
SRR516988.31050597.2+	CTGCTGAGACC
SRR517006.62045893.2-	CTGCTGAGACC
SRR517007.44465454.2-	CTGCTGAGACC
SRR511911.51765582.1+	TGCTGAGACC
SRR516967.132651451.1-	TGCTGAGACC
SRR516967.94039320.1-	TGCTGAGACC
SRR516968.137574947.2+	TGCTGAGACC
SRR516976.29296347.1-	TGCTGAGACC
SRR516977.13189923.1+	TGCTGAGACC
SRR516984.56347101.1-	TGCTGAGACC
SRR516994.1794759.2+	TGCTGAGACC
SRR517009.38612123.1+	TGCTGAGACC
SRR511911.36873194.1-	GCTGAGACC
SRR511914.80423043.1-	GCTGAGACC
SRR516968.58859238.1+	GCTGAGACC
SRR516973.61866208.2+	GCTGAGACC
SRR516976.10068918.1-	GCTGAGACC
SRR516976.8946365.1-	GCTGAGACC
SRR516980.12955843.2-	GCTGAGACC
SRR516982.42917165.2+	GCTGAGACC
SRR516992.78662263.1-	GCTGAGACC
SRR516996.85884676.2-	GCTGAGACC
SRR516997.66381769.1-	GCTGAGACC
SRR517000.39251131.1+	GCTGAGACC
SRR517004.58862282.1+	GCTGAGACC
SRR511892.9511611.2+	CTGAGACC
SRR516973.16005217.1-	CTGAGACC
SRR516983.41490614.2-	CTGAGACC
SRR516983.54306814.2-	CTGAGACC
SRR516992.23178703.2-	CTGAGACC
SRR516993.12312704.1-	CTGAGACC
SRR516993.73082870.1-	CTGAGACC
SRR516996.88424796.2-	CTGAGTCC
SRR517000.81566989.1+	CTGAGACC
SRR511911.21547512.2-	TGAGACC
SRR511913.18165035.2-	TGAGACC
SRR511913.52308827.1-	TGAGACC
SRR516968.156018396.1-	TGAGACC
SRR516972.49653018.2-	TGAGACC
SRR516993.83887266.1+	TGAGACC
SRR516999.81087074.2+	TGAGACC
SRR511911.19148709.2+	GAGACC
SRR511912.70385865.1-	GAGACC
SRR516992.37277386.2-	GAGACG

```
SRR516996.1617304.1+                                                              GAGACC
SRR516998.43983662.1-                                                             GAGACC
SRR517001.43347006.1-                                                             GAGACC
SRR511892.23600637.2+                                                              AGACC
SRR511911.3389924.2+                                                               AGACC
SRR516967.96216344.1+                                                              AGACC
SRR516990.32569854.2-                                                              AGACC
SRR516990.55302390.2-                                                              AGACC
SRR516993.75065526.2+                                                              AGACC
SRR516996.29500035.2-                                                              AGACC
SRR516999.96819542.2-                                                              AGACC
SRR511913.70154375.2-                                                               GACC
SRR516989.36153179.1+                                                               GACC
SRR516999.48060851.1-                                                               GACC
SRR517000.9053207.1-                                                                GACC
SRR517001.26562166.2-                                                               GACC
SRR517002.51438896.1-                                                               GACC
SRR516990.20970250.1+                                                                ACC
SRR516974.21816576.2-                                                                 CC
SRR517004.45940969.1+                                                                 CC
SRR516977.49992915.2-                                                                  C
SRR516978.65078285.1-                                                                  C
SRR517009.51801045.2+                                                                  C
SRR517009.70794059.2+                                                                  C
SRR517009.89416108.1+                                                                  C
                         ____________________________________________________________
consensus                CACTGCTGCATTTCTCCATCTTCCTGGAGCCTACAATCATGGTTTACCTCTGCTGAGACC

                             .    :    .    :    .    :    .    :    .    :    .    :
SRR516990.25197712.2+    A
SRR516990.28337778.2+    A
SRR517008.17734492.1+    A
SRR511912.83753137.1-    AT
SRR516978.76490935.1-    AT
SRR516983.22296978.1-    AT
SRR516997.92691745.1+    AT
SRR517001.54839630.2+    AT
SRR517009.22763744.1+    AT
SRR516968.125235180.1-   ATG
SRR516978.51856587.2-    ATG
SRR516983.99315750.2-    ATG
SRR516993.14999698.2+    ATG
SRR517005.29004911.1-    ATG
SRR516972.66728747.1-    ATGA
SRR516974.18523926.1+    ATGA
SRR516976.50060483.1+    ATGA
SRR516984.85335095.2-    ATGA
SRR517004.11493321.2-    ATGA
SRR517006.60516129.2-    ATGA
SRR517008.72452430.1-    ATGA
SRR511915.45075036.2+    ATGAC
SRR516968.170088452.1-   ATGAC
SRR516972.40257819.2+    ATGAC
SRR516976.35412479.1-    ATGAC
SRR516985.24934961.2-    ATGAC
SRR516988.48142203.1-    ATGAC
SRR516992.45298055.2+    ATGAC
SRR516993.79889276.2-    ATGAC
SRR516994.50421693.1-    ATGAC
SRR516996.22277693.1-    ATGAC
SRR516998.103714982.2-   ATGAC
SRR517000.64893284.2-    ATGAC
SRR517002.35600907.2-    ATGAC
SRR511911.36873194.2+    ATGACG
SRR511914.67737508.2+    ATGACG
SRR511914.67737642.2+    ATGACG
SRR516968.42660308.2+    ATGACG
SRR516972.7252980.1-     ATGACG
SRR516974.34088399.1+    ATGACG
SRR516975.50255788.1-    ATGACG
SRR516996.4359948.2+     ATGACG
SRR516999.101662647.1-   ATGACG
SRR517004.15232910.1+    ATGACG
SRR517005.38635366.1-    ATGACG
SRR511912.40363221.2+    ATGACGA
SRR516978.13762672.2+    ATGACGA
SRR516984.19458384.2-    ATGACGA
SRR516991.40101822.2+    ATGACGA
SRR516997.46341269.2+    ATGATGA
SRR517006.3014885.2+     ATGACGA
SRR511911.13404865.1-    ATGACGAA
SRR516968.7574142.1+     ATGACGAA
SRR516970.9984608.2-     ATGACGAA
SRR516994.69104660.2-    ATGACGAA
```

SRR516995.44976834.2+	ATGACGAA
SRR511892.22460703.2-	ATGACGAAC
SRR511892.28500594.2-	ATGACGAAC
SRR516967.20886732.1+	ATGACGAAC
SRR516985.3722256.2-	ATGACGAAC
SRR516991.88390963.2-	ATGATGAAC
SRR516994.3915521.1-	ATGACGAAC
SRR516994.39508980.2-	ATGACGAAC
SRR517001.21007961.1-	ATGACGAAC
SRR517004.15247676.2-	ATGACGAAC
SRR517004.39340770.2-	ATGACGAAC
SRR517005.36327145.1+	ATGACGAAC
SRR517005.3636097.2-	ATGACGAAC
SRR517005.92632228.1+	ATGACGAAC
SRR517008.46362816.2-	ATGACGAAC
SRR517009.32725362.1-	ATGACGAAC
SRR511892.6007403.2+	ATGACGAACA
SRR511914.68550111.1-	ATGACGAACA
SRR511914.68550259.1-	ATGACGAACA
SRR516972.63526588.1-	ATGACGAACA
SRR516973.2205347.2-	ATGATGAACA
SRR516978.68244002.1+	ATGACGAACA
SRR516979.22734254.1+	ATGATGAACA
SRR516989.78285929.2+	ATGACGAACA
SRR516993.6142128.1-	ATGACGAACA
SRR517004.18294620.1+	ATGACGAACA
SRR517007.27000447.1-	ATGACGAACA
SRR516968.81828792.2+	ATGACGAACAG
SRR516981.64156231.1-	ATGACGAACAG
SRR517000.22124310.1+	ATGACGAACAG
SRR511918.12327870.2+	ATGACGAACAGG
SRR511918.22802077.2+	ATGACGAACAGG
SRR511918.6049292.2+	ATGACGAACAGG
SRR516968.37214890.1+	ATGACGAACAGG
SRR516980.56485916.1+	ATGACGAACAGG
SRR511912.3464697.2-	ATGACGAACAGGA
SRR511913.30633233.1+	ATGACGAACAGGA
SRR511913.52630668.1+	ATGACGAACAGGA
SRR511916.29120870.1+	ATGACGAACAGGA
SRR516979.70135575.2+	ATGATGAACAGGA
SRR516982.20957450.2+	CTGATGAACAGGA
SRR516987.73050149.1-	ATGACGAACAGGA
SRR516994.71971275.2-	ATGACGAACAGGA
SRR517009.41211152.1-	ATGACGAACAGGA
SRR511914.66457590.1+	ATGACGAACAGGAG
SRR516968.75914217.2-	ATGACGAACAGGAG
SRR516972.46769474.2+	ATGACGAACAGGAG
SRR516982.11686504.2-	ATGATGAACAGGAG
SRR516984.39380541.1-	ATGACGAACAGGAG
SRR517009.58624417.1-	ATGACGAACAGGAG
SRR511911.7460180.2+	ATGACGAACAGGAGG
SRR511913.70659514.2-	ATGACGAACAGGAGG
SRR511913.72818344.2-	ATGACGAACAGGAGG
SRR516967.13632766.2+	ATGACGAACAGGAGG
SRR516971.818087.2-	ATGACGAACAGGAGG
SRR516976.3885854.1-	ATGACGAACAGGAGG
SRR516983.60185222.2+	ATGACGAACAGGAGG
SRR516985.21667072.2-	ATGACGAACAGGAGG
SRR516999.8565899.2+	ATGACGGACAGGAGG
SRR517001.20598275.2-	ATGACGAACAGGAGG
SRR517004.38117563.1-	ATGACGAACAGGAGG
SRR517004.57378084.1+	ATGACGAACAGGAGG
SRR511914.2434148.2-	ATGACGAACAGGAGGT
SRR516979.54060768.1-	ATGATGAACAGGAGGT
SRR516989.31478451.2-	ATGACGAACAGGAGGT
SRR516990.26352168.1-	ATGACGAACAGGAGGT
SRR517001.99395117.2-	ATGACGAACAGGAGGT
SRR511892.64175574.1+	ATGACGAACAGGAGGTG
SRR511912.78794364.2-	ATGACGAACAGGAGGTG
SRR511913.54466415.2-	ATGACGAACAGGAGGTG
SRR511914.49593496.1-	ATGACGAACAGGAGGTG
SRR511914.68003041.1-	ATGACGAACAGGAGGTG
SRR516968.78983771.2+	ATGACGAACAGGAGGTG
SRR516985.49910327.1-	ATGACGAACAGGAGGTG
SRR516990.18276182.1-	ATGACGAACAGGAGGTG
SRR516995.7000561.2+	ATGACGAACAGGAGGTG
SRR516999.43689059.1-	ATGACGAACAGGAGGTG
SRR517001.65137644.1-	ATGACGAACAGGAGGTG
SRR511916.21038728.2+	ATGACGAACAGGAGGTGA
SRR511918.21279607.2-	ATGACGAACAGGAGGTGA
SRR516975.14717207.2-	ATGATGAACAGGAGGTGA
SRR516976.15724427.2+	ATGATGAACAGGAGGTGA
SRR516985.58622247.1+	ATGACGAACAGGAGGTGA
SRR517001.24229462.2-	ATGACGAACAGGAGGTGA

SRR517008.47368909.1+ ATGACGAACAGGAGGTGA
SRR516980.641302.1- ATGACGAACAGGAGGTGAT
SRR516985.88521940.2- ATGACGAACAGGAGGTGAT
SRR517002.43430166.1- ATGACGAACAGGAGGTGAT
SRR516975.41667615.2- ATGACGAACAGGAGGTGATG
SRR516978.65142163.2+ ATGACGAACAGGAGGTGATG
SRR516981.51713088.2- ATGACGAACAGGAGGTGATG
SRR516985.18771290.2- ATGACGAACAGGAGGTGATG
SRR516989.46948006.2+ ATGACGAACAGGAGGTGATG
SRR516989.48094546.1+ ATGACGAACAGGAGGTGATG
SRR516991.8406901.1- ATGATGAACAGGAGGTGATG
SRR511916.58161197.1+ ATGACGAACAGGAGGTGATGA
SRR516968.96419618.2+ ATGACGAACAGGAGGTGATGA
SRR516978.54979818.2- ATGACGAACAGGAGGTGATGA
SRR516982.7942053.2- ATGATGAACAGGAGGTGATGA
SRR516987.14379181.1- ATGACGAACAGGAGGTGATGA
SRR516988.28221388.1- ATGACGAACAGGAGGTGATGA
SRR516988.8503657.1+ ATGACGAACAGGAGGTGATGA
SRR517000.91798223.1+ ATGACGAACAGGAGGTGATGA
SRR517001.32526871.1+ ATGACGAACAGGAGGTGATGA
SRR517006.42774934.1- ATGACGAACAGGAGGTGATGA
SRR517009.37989736.1+ ATGACGAACAGGAGGTGATGA
SRR516984.46881394.2+ ATGACGAACAGGAGGTGATGAC
SRR516991.38701220.2+ ATGACGAACAGGAGGTGATGAC
SRR516991.83096318.2+ ATGATGAACAGGAGGTGATGAC
SRR516995.17155730.1- ATGACGAACAGGAGGTGATGAC
SRR516998.30978040.1- ATGACGAACAGGAGGTGATGAC
SRR516999.35189572.2+ ATGACGAACAGGAGGTGATGAC
SRR511913.74026630.2+ ATGACGAACAGGAGGTGATGACA
SRR511917.45677079.2- ATGACGAACAGGAGGTGATGACA
SRR511917.69281775.2- ATGACGAACAGGAGGTGATGACA
SRR516970.28438184.2+ ATGACGAACAGGAGGTGATGACA
SRR516990.52881947.2- ATGACGAACAGGAGGTGATGACA
SRR517000.84699913.1+ ATGACGAACAGGAGGTGATGACA
SRR517007.46550915.1+ ATGACGAACAGGAGGTGATGACA
SRR517008.30759488.1+ ATGACGAACAGGAGGTGATGACA
SRR516979.15989227.1- ATGACGAACAGGAGGTGATGACAT
SRR516982.57333576.1- ATGATGAACAGGAGGTGATGACAT
SRR516992.10463572.2+ ATGACGAACAGGAGGTGATGACAT
SRR511892.63390781.1+ ATGACGAACAGGAGGTGATGACATT
SRR516986.21282883.1- ATGACGAACAGGAGGTGATGACATT
SRR516997.1368394.1+ ATGACGAACAGGAGGTGATGACATT
SRR516999.79336674.2- ATGACGAACAGGAGGTGATGACATT
SRR511912.71061316.2+ ATGACGAACAGGAGGTTATGACATTT
SRR516967.56639881.1+ ATGACGAACAGGAGGTGATGACATTT
SRR516973.44104519.1- ATGATGAACAGGAGGTGATGACATTT
SRR516975.59671997.1+ ATGACGAACAGGAGGTGATGACATTT
SRR516981.71035524.1- ATGACGAACAGGAGGTGATGACATTT
SRR516983.26943128.1- ATGACGAACAGGAGGTGATGACATTT
SRR516998.7520652.2+ ATGACGAACAGGAGGTGATGACATTT
SRR516999.536846.1- ATGACGAACAGGAGGTGATGACATTT
SRR517000.79804010.1- ATGACGAACAGGAGGTGATGACATTT
SRR517003.32276918.1+ ATGACGAACAGGAGGTGATGACATTT
SRR517006.63434435.1- ATGACGAACAGGAGGTGATGACATTT
SRR511916.31954449.1+ ATGACGAACAGGAGGTGATGACATTTG
SRR516994.60807912.2+ ATGACGAACAGGAGGTGATGACATTTG
SRR517006.92771968.1+ ATGACGAACAGGAGGTGATGACATTTG
SRR517009.39648041.1+ ATGACGAACAGGAGGTGATGACATTTG
SRR511912.51896762.2+ ATGACGAACAGGAGGTGATGACATTTGA
SRR516975.29998471.1+ ATGATGAACAGGAGGTGATGACATTTGA
SRR516975.42749277.1- ATGATGAACAGGAGGTGATGACATTTGA
SRR516980.26511072.2- ATGACGAACAGGAGGTGATGACATTTGA
SRR516982.62820607.2+ ATGATGAACAGGAGGTGATGACATTTGA
SRR516985.94632745.2+ ATGACGAACAGGAGGTGATGACATTTGA
SRR516996.41370267.1- ATGACGAACAGGAGGTGATGACATTTGA
SRR517006.7636423.2- ATGACGAACAGGAGGTGATGACATTTGA
SRR511892.42564732.1+ ATGACGAACAGGAGGTGATGACATTTGAG
SRR511911.53964321.1- ATGACGAACAGGAGGTGATGACATTTGAG
SRR511915.30420375.2- ATGACGAACAGGAGGTGATGACATTTGAG
SRR516973.26618435.2+ ATGATGAACAGGAGGTGATGACATTTGAG
SRR516974.46978934.2+ ATGACGAACAGGAGGTGATGACATTTGAG
SRR516979.13778629.2+ ATGACGAACAGGAGGTGATGACATTTGAG
SRR517000.59205461.2- ATGACGAACAGGAGGTGATGACATTTGAG
SRR517008.53562741.2+ ATGACGAACAGGAGGTGATGACATTTGAG
SRR511892.35285726.1- ATGACGAACAGGAGGTGATGACATTTGAGC
SRR516968.179954570.2- ATGACGAACAGGAGGTGATGACATTTGAGC
SRR516978.70371063.2- ATGACGAACAGGAGGTGATGACATTTGAGC
SRR516986.6287834.2+ ATGACGAACAGGAGGTGATGACATTTGAGC
SRR516991.55763720.1+ ATGACGAACAGGAGGTGATGACATTTGAGC
SRR517001.70785490.2- ATGACGAACAGGAGGTGATGACATTTGAGC
SRR517005.71623618.2- ATGACGAACAGGAGGTGATGACATTTGAGC
SRR516983.45197941.1+ ATGACGAACAGGAGGTGATGACATTTGAGCC
SRR516989.45104008.1- ATGACGAACAGGAGGTGATGACATTTGAGCC
SRR516992.26374669.1+ ATGACGAACAGGAGGTGATGACATTTGAGCC

SRR516996.46495058.2- ATGACGAACAGGAGGTGATGACATTTGAGCC
SRR516999.29788274.1+ ATGACGAACAGGAGGTGATGACATTTGAGCC
SRR517001.18025361.1+ ATGACGAACAGGAGGTGATGACATTTGAGCC
SRR517002.51898282.2- ATGACGAACAGGAGGTGATGACATTTGAGCC
SRR517009.39987367.2- ATGACGAACAGGAGGTGATGACATTTGAGCC
SRR511892.56496978.2+ ATGACGAACAGGAGGTGATGACATTTGAGCCG
SRR511912.67114670.1+ ATGACGAACAGGAGGTGATGACATTTGAGCCG
SRR516969.19690168.1- ATGACGAACAGGAGGTGATGACATTTGAGCCG
SRR516977.56066574.1+ ATGACGAACAGGAGGTGATGACATTTGAGCCG
SRR516980.42600802.2- ATGACGAACAGGAGGTGATGACATTTGAGCCG
SRR516982.24144878.1- ATGATGAACAGGAGGTGATGACATTTGAGCCG
SRR516995.48904445.1- ATGACGAACAGGAGGTGATGACATTTGAGCCG
SRR516998.74061869.1- ATGACGAACAGGAGGTGATGACATTTGAGCCG
SRR516975.49935850.2+ ATGACGAACAGGAGGTGATGACATTTGAGCCGC
SRR516979.61681085.2+ ATGACGAACAGGCGGTGATGACATTTGCGCCGC
SRR516983.89585195.2+ ATGACGAACAGGAGGTGATGACATTTGAGCCGC
SRR516991.41770914.1- ATGATGAACAGGAGGTGATGACATTTGAGCCGC
SRR516971.36450695.2+ ATGACGAACAGGAGGTGATGACATTTGAGCCGCA
SRR516998.54361396.1+ ATGACGAACAGGAGGTGATGACATTTGAGCCGCA
SRR516999.99949277.1- ATGACGAACAGGAGGTGATGACATTTGAGCCGCA
SRR517009.6644834.2- ATGACGAACAGGAGGTGATGACATTTGAGCCGCA
SRR511892.22200889.1+ ATGACGAACAGGAGGTGATGACATTTGAGCCGCAA
SRR511892.25732978.1- ATGACGAACAGGAGGTGATGACATTTGAGCCGCAA
SRR516976.6204118.1+ ATGACGAACAGGAGGTGATGACATTTGAGCTGCAA
SRR516990.6461511.1+ ATGACGAACAGGAGGTGATGACATTTGAGCCGCAA
SRR516991.19768136.1+ ATGACGAACAGGAGGTGATGACATTTGAGCCGCAA
SRR516994.26780695.2+ ATGACGAACAGGAGGTGCTGACATTTGAGCCGCAA
SRR516994.30789737.1+ ATGACGAACAGGAGGTGATGACATTTGAGCCGCAA
SRR511913.46683201.1- ATGACGAACAGGAGGTGATGACATTTGAGCCGCAAG
SRR516971.1149505.2+ ATGACGAACAGGAGGTGATGACATTTGAGCCGCAAG
SRR516974.54299546.1+ ATGACGAACAGGAGGTGATGACATTTGAGCCGCAAG
SRR516977.65083064.2+ ATGACGAACAGGAGGTGATGACATTTGAGCCGCAAG
SRR516980.29666170.2+ ATGACGAACAGGAGGTGATGACATTTGAGCCGCAAG
SRR516982.31054443.1- ATGATGAACAGGAGGTGATGACATTTGAGCCGCAAG
SRR516983.15530144.1+ ATGACGAACAGGAGGTGATGACATTTGAGCCGCAAG
SRR516985.1978329.2- ATGACGAACAGGAGGTGATGACATTTGAGCCGCAAG
SRR516996.18713822.1- ATGACGAACAGGAGGTGATGACATTTGAGCCGCAAG
SRR516997.5355646.1- ATGACGAACAGGAGGTGATGACATTTGAGCCGCAAG
SRR516999.108304884.1+ ATGACGAACAGGAGGTGATGACATTTGAGCCGCAAG
SRR517001.3087830.1+ ATGACGAACAGGAGGTGATGACATTTGAGCCGCAAG
SRR517004.41718493.2+ ATGACGAACAGGAGGTGATGACATTTGAGCCGCAAG
SRR511912.25061802.2+ ATGACGAACAGGAGGTGATGACATTTGCGCCGCAAGT
SRR516973.25012163.1- ATGACGAACAGGAGGTGATGACATTTGAGCCGCAAGT
SRR516978.43288050.1- ATGACGAACAGGAGGTGATGACATTTGAGCCGCAAGT
SRR516980.5795258.2- ATGACGAACAGGAGGTGATGACATTTGAGCCGCAAGT
SRR516992.11057517.2- ATGACGAACAGGAGGTGATGACATTTGAGCCGCAAGT
SRR516993.55737172.2- ATGACGAACAGGAGGTGATGACATTTGAGCCGCAAGT
SRR511911.48293933.2- ATGACGAACAGGAGGTGATGACATTTGAGCCGCAAGTC
SRR511917.54829842.1- ATGACGAACAGGAGGTGATGACATTTGAGCCGCAAGTC
SRR511917.69338839.1- ATGACGAACAGGAGGTGATGACATTTGAGCCGCAAGTC
SRR516973.63984660.2+ ATGATGAACAGGAGGTGATGACATTTGAGCCGCAAGTC
SRR516978.9966331.1- ATGACGAACAGGAGGTGATGACATTTGAGCCGCAAGTC
SRR516985.58077220.2+ ATGACGAACAGGAGGTGATTACATTTGAGCCGCAAGTC
SRR516996.71975786.2- ATGACGAACAGGAGGTGATGACATTTGAGCCGCAAGTC
SRR517001.103617359.1+ ATGACGAACAGGAGGTGATGACATTTGAGCCGCAAGTC
SRR517001.22577401.1- ATGACGAACAGGAGGTGATGACATTTGAGCCGCAAGTC
SRR516976.12584702.2+ ATGACGAACAGGAGGCGATGACATTTGAGATGCACGTCC
SRR516987.5180076.1- ATGATGAACAGGAGGTGATGACATTTGAGCCGCAAGTCC
SRR516990.58587583.1- ATGACGAACAGGAGGTGATGACATTTGAGCCGCAAGTCC
SRR516991.44385216.1+ ATGATGAACAGGAGGTGATGACATTTGAGCCGCAAGTCC
SRR516995.80032724.1- ATGACGAACAGGAGGTGATGACATTTGAGCTGCAAGTCC
SRR516981.4478016.2- ATGACGAACAGGAGGTGATGACATTTGAGCCGCAAGTCCA
SRR516987.6864091.2- ATGATGAACAGGAGGTGATGACATTTGAGCCGCAAGTCCA
SRR516989.43322421.2+ ATGACGAACAGGAGGTGATGACATTTGAGCCGCAAGTCCA
SRR516993.83112824.1+ ATGACGAACAGGAGGTGATGACATTTGAGCCGCAAGTCCA
SRR516994.61678342.1+ ATGACGAACAGGAGGTGATGACATTTGAGCCGCAAGTCCA
SRR516997.96113566.1- ATGACGAACAGGAGGTGATGACATTTGAGCCGCAAGTCCA
SRR517002.26924755.2+ ATGACGAACAGGAGGTGATGACATTTGAGCCGCAAGTCCA
SRR517005.50448398.2+ ATGACGAACAGGAGGTGATGACATTTGAGCCGCAAGTCCA
SRR517008.21405715.2- ATGACGAACAGGAGGTGATGACATTTGAGCCGCAAGTCCA
SRR511913.20105001.2+ ATGACGAACAGGAGGTGATGACATTTGAGCCGCAAGTCCAT
SRR511913.20105241.2+ ATGACGAACAGGAGGTGATGACATTTGAGCCGCAAGTCCAT
SRR516977.63372655.1+ ATGACGAACAGGAGGTGATGACATTTGAGCCGCAAGTCCAT
SRR517001.46019341.1+ ATGACGAACAGGAGGGGATGACATTTGAGCCGCAAGTCCAT
SRR517003.9557651.2+ ATGACGAACAGGAGGTGATGACATTTGAGCCGCAAGTCCAT
SRR511892.53446249.2+ ATGACGAACAGGAGGTGATGACATTTGAGCCGCAAGTCCATA
SRR516973.57501785.2- ATGATGAACAGGAGGTGATGACATTTGAGCCGCAAGTCCATA
SRR516981.55431738.2+ ATGACGAACAGGAGGTGATGACATTTGAGCCGCAAGTCCATA
SRR516982.67688043.2+ ATGATGAACAGGAGGTGATGACATTTGAGCCGCAAGTCCATA
SRR516983.95786046.1+ ATGACGAACAGGAGGTGATGACATTTGAGCCGCAAGTCCATA
SRR516991.16236160.2+ ATGATGAACAGGAGGTGATGACATTTGAGCCGCAAGTCCATA
SRR516996.49068306.1- ATGACGAACAGGAGGTGATGACATTTGAGCCGCAAGTCCATA
SRR516998.94026502.1+ ATGACGAACAGGAGGTGATGACATTTGAGCCGCAAGGCCATA
SRR517000.23065522.1+ ATGACGAACAGGAGGTGATGACATTTGAGCCGCAAGTCCATA

SRR517002.88240524.1+ ATGACGAACAGGAGGTGATGACATTTGAGCCGCAAGTACATA
SRR516967.125704511.1- ATGACGAACAGGAGGTGATGACATTTGAGCCGCAAGTCCATAC
SRR516983.46519373.2- ATGACGAACAGGAGGTGATGACATTTGAGCCGCAAGTCCATAC
SRR516983.96419424.1- ATGACGAACAGGAGGTGATGACATTTGAGTCGCAAGTCCATAC
SRR516996.25489938.2- ATGACGAACAGGAGGTGATGACATTTGAGCCGCAAGTCCATAC
SRR517003.43535674.1+ ATGACGAACAGGAGGTGATGACATTTGAGCCGCAAGTCCATAC
SRR517006.48499023.1+ ATGACGAACAGGAGGCGATGACATTTGAGCCGCAAGTCCATAC
SRR511913.25527344.1+ ATGACGAACAGGAGGTGATGACATTTGAGCCGCAAGTCCATACA
SRR516967.6221170.1- ATGACGAACAGGAGGTGATGACATTTGAGCCGCAAGTCCATACA
SRR516975.46689364.1+ ATGACGAACAGGAGGTGATGACATTTGAGCCGCAAGTCCATACA
SRR516998.101916190.1- ATGACGAACAGGAGGTGATGACATTTGAGCCGCAAGTCCATACA
SRR517000.91504937.1+ ATGACGAACAGGAGGTGATGACATTTGAGCCGCAAGTCCATACA
SRR517001.900897.1+ ATGACGAACAGGAGGTGATGACATTTGAGCCGCAAGTCCATACA
SRR517007.25961977.2- ATGACGAACAGGAGGTGATGACATTTGAGCCGCAAGTCCATACA
SRR517009.83112268.1+ ATGACGAACAGGAGGTGATGACATTTGAGCCGCAAGTCCATACA
SRR516968.39655396.2+ ATGACGAACAGGAGGTGATGACATTTGAGCCGCAAGTCCATACAA
SRR516984.44849129.2+ ATGACGAACAGGAGGTGATGACATTTGAGCCGCAAGTCCATACAA
SRR516986.35116064.1+ ATGACGAACAGGAGGTGATGACATTTGAGCCGCAAGTCCATACAA
SRR516995.55914942.1- ATGACGAACAGGAGGTGATGACATTTGAGCTGCAAGTCCATACAA
SRR516997.92588309.1- ATGACGAACAGGAGGTGATGACATTTGAGCCGCAAGTCCATACAA
SRR517000.73971487.1+ ATGACGAACAGGAGGAGATGACATTTGAGCCGCAAGTCCATACAA
SRR511911.29777200.1- ATGACGAACAGGAGGTGATGACATTTGAGCCTCAAGTCCATACAAC
SRR516972.84821035.1- ATGACGAACAGGAGGTGATGACATTTGAGCCGCAAGTCCATACAAC
SRR516974.64263409.1- ATGACGAACAGGAGGTGATGACATTTGAGCCGCAAGTCCATACAAC
SRR516980.6938514.1+ ATGACGAACAGGAGGTGATGACATTTGAGCCGCAAGTCCATACAAC
SRR516987.45900545.1+ ATGATGAACAGGAGGTGATGACATTTGAGCCGCAAGTCCATACAAC
SRR516987.74463633.2+ ATGACGAACAGGAGGTGATGACATTTGAGCCGCAAGTCCATACAAC
SRR516999.1489221.1- ATGACGAACAGGAGGTGATGACATTTGAGCCGCAAGTCCATACAAC
SRR516999.15834111.2- ATGACGAACAGGAGGTGATGACATTTGAGCCGCAAGTCCATACAAC
SRR517008.35566824.2+ ATGACGAACAGGAGGTGATGACATTTGAGCCGCAAGTCCATACAAC
SRR516970.19833631.2- ATGACGAACAGGAGGTGATGACATTTGAGCCGCAAGTCCATACAACT
SRR516972.17444908.2- ATGACGAACAGGAGGTGATGACATTTGAGCCGCAAGTCCATACAACT
SRR516972.29398207.1- ATGACGAACAGGAGGTGATGACATTTGAGCCGCAAGTCCATACAACT
SRR516977.18216728.2+ ATGACGAACAGGAGGTGATGACATTTGAGCCGCAAGTCCATACAACT
SRR516977.77183349.2+ ATGACGAACAGGAGGTGATGACATTTGAGCCGCAAGTCCATACAACT
SRR516979.10219896.2+ ATGACGAACAGGAGGTGATGACATTTGAGCCGCAAGTCCATACAACT
SRR516985.43613602.2+ ATGACGAACAGGAGGTGATGACATTTGAGCCGCAAGTCCATACAACT
SRR516993.60327009.1- ATGACGAACAGGAGGTGATGACATTTGAGCCGCAAGTCCATACAACT
SRR516998.96836905.2- ATGCCGAACAGGAGGTGATGACATTTGAGCCGCAAGTCCATACAACT
SRR511911.14454908.1- ATGACGAACAGGAGGTGATGACATTTGAGCCGCAAGTCCATACAACTG
SRR511915.47098295.1+ ATGACGAACAGGAGGTGATGACATTTGAGCCGCAAGTCCATACAACTG
SRR516974.49070472.1- ATGACGAACAGGAGGTGATGACATTTGAGCCGCAAGTCCATACAACTG
SRR516975.64845215.1- ATGACGAACAGGAGGTGATGACATTTGAGCCGCAAGTCCATACAACTG
SRR516993.85425731.1- ATGACGAACAGGAGGTGATGACATTTGAGCTGCAAGTCCATACAACTG
SRR517000.15878174.2- ATGACGAACAGGAGGTGATGACATTTGAGCCGCAAGTCCATACAACTG
SRR517000.27952472.1+ ATGACGAACAGGAGGTGATGACATTTGAGCCGCAAGTCCATACAACTG
SRR517000.42847360.2- ATGACGAACAGGAGGTGATGACATTTGAGCCGCAAGTCCATTCAACTG
SRR517000.52705672.2+ ATGACGAACAGGAGGTGATGACATTTGAGCCGCAAGTCCATACAACTG
SRR517004.46455486.1- ATGACGAACAGGAGGTGATGACATTTGAGCCGCAAGTCCATACAACTG
SRR511912.34406775.1- ATGACGAACAGGAGGTGATGACATTTGAGCCGCAAGTCCATACAACTGG
SRR516968.145184287.2- ATGACGAACAGGAGGTGATGACATTTGAGCCGCAAGTCCATACAACTGG
SRR516968.15347757.2- ATGACGAACAGGAGGTGATGACATTTGAGCCGCAAGTCCATACAACTGG
SRR516969.26985112.1+ ATGACGAACAGGAGGTGATGACATTTGAGCCGCAAGTCCATACAACTGG
SRR516974.13682622.1+ ATGACGAACAGGAGGTGATGACATTTGAGCCGCAAGTCCATACAACTGG
SRR516976.30705361.2- ATGACGAACAGGAGGTGATGACATTTGAGCTGCAAGTCCATACAACTGG
SRR516976.6568357.2+ ATGATGCACAGGAGGTGATGTCATTTGAGCCGCAAGTCAATACAACTGG
SRR516977.79316740.2- ATGACGAACAGGAGGTGATGACATTTGAGCCGCAAGTCCATACAACTGG
SRR516979.63870114.2- ATGACGAACAGGAGGTGATGACATTTGAGCCGCAAGTCCATACAACTGG
SRR516990.30827191.1+ ATGACGAACAGGAGGTGATGACATTTGAGCCGCAAGTCCATACAACTGG
SRR516997.85778454.1+ ATGATGAACAGGAGGTGATGACATTTGAGCCGCAAGTCCATACAACTGG
SRR517003.47760361.1- ATGACGAACAGGAGGTGATGACATTTGAGCCGCAAGTCCATACAACTGG
SRR517009.80280823.1- ATGACGAACAGGAGGTGATGACATTTGAGCTGCAAGTCCATACAACTGG
SRR511892.28064592.2- ATGACGAACAGGAGGTGATGACATTTGAGCCGCAAGTCCATACAACTGGC
SRR516987.59068064.1- ATGACGAACAGGAGGTGATGACATTTGAGCCGCATGTCCATACAACTGGC
SRR516992.47548931.2+ ATGACGAACAGGAGGTGATGACATTTGAGCCGCAAGTCCATACAACTGGC
SRR517000.49039794.2- ATGACGAACAGGAGGTGATGACATTTGAGCCGCAAGTCCATACAACTGGC
SRR517000.59218275.1+ ATGACGAACAGGAGGTGATGACATTTGAGCCGCAAGTCCATACAACTGGC
SRR517000.86755698.2- ATGACGAACAGGAGGTGATGACATTTGAGCCGCAAGTCCATACAACTGGC
SRR517005.68411118.2+ ATGACGAACAGGAGGTGATGACATTTGAGCCGCAAGTCCATACAACTGGC
SRR511913.75322811.2- ATGACGAACAGGAGGTGATGACATTTGAGCCGCAAGTCCATACAACTGGCT
SRR516991.45959728.2- ATGATGAACAGGAGGTGATGACATTTGAGCCGCAAGTCCATACAACTGGCT
SRR516992.5218916.1- ATGACGAACAGGAGGTGATGACATTTGAGCCGCAAGTCCATACAACTGGCT
SRR516996.43104832.1- ATGACGAACAGGAGGTGATGACATTTGAGCCGCAAGTCCATACAACTGGCT
SRR516997.19843563.2+ ATGACGAACAGGAGGTGATGACATTTGAGCCGCAAGTCCATACAACTGGCT
SRR516998.77015275.2+ ATGACGAACAGGAGGTGATGACATTTGAGCCGCAAGTCCATACAACTGGCT
SRR516999.56136092.1+ ATGACGAACAGGAGGTGATGACATTTGAGCCGCAAGTCCATACAACTGGCT
SRR517001.87349540.2- ATGACGAACAGGAGGTGATGACATTTGAGCCGCAAGTCCATACAACTGGCT
SRR516979.67226952.1+ ATGACGAACAGGAGGTGATGACATTTGAGCCGCAAGTCCATACAACTGGCTG
SRR516986.21942167.1+ ATGACGAACAGGAGGTGATGACATTTGAGCCGCAAGTCCATACAACTGGCTG
SRR516998.58191126.1+ ATGACGAACAGGAGGTGATGACATTTGAGCCGCAAGTCCATACAACTGGCTG
SRR517003.50373066.2+ ATGACGAACAGGAGGTGATGACATTTGAGCCGCAAGTCCATACAACTGGCTG
SRR517003.66035176.2+ ATGACGAACAGGAGGTGATGACATTTGAGCCGCAAGTCCATACAACTGGCTG
SRR517009.44679586.2- ATGACGAACAGGAGGTGATGACATTTGAGCTGCAAGTCCATACAACTGGCTG
SRR511892.41096124.2- ATGACGAACAGGAGGTGATGACATTTGAGCCGCAAGTCCATACAACTGGCTGG

SRR516970.20795711.2-   ATGACGAACAGGAGGTGATGACATTTGAGCCGCAAGTCCATACAACTGGCTGG
SRR516985.12986836.1+   ATGACGAACAGGAGGTGATGACATTTGAGCCGCAAGTCCATACAACTGGCTGG
SRR516989.7050322.1-   ATGACGAACAGGAGGTGATGACATTTGAGCCGCAAGTCCATACAACTGGCTGG
SRR516998.65330415.1-   ATGACGAACAGGAGGTGATGACATTTGAGCCGCAAGTCCATACAACTGGCTGG
SRR517005.99973486.1-   ATGACGAACAGGAGGTGATGACATTTGAGCCGCAAGTCCATACAACTGGCTGG
SRR511911.21547512.1+   ATGACGAACAGGAGGTGATGACATTTGAGCCGCAAGTCCATACAACTGGCTGGG
SRR516985.56923558.1+   ATGACGAACAGGAGGTGATGACATTTGAGCCGCAAGTCCATACAACTGGCTGGT
SRR516987.91428967.1-   ATGATGAACAGGAGGTGATGACATTTGAGCCGCAAGTCCATACAACTGGCTGGT
SRR516998.39972283.1+   ATGACGAACAGGAGGGGATGACATTTGAGCCGCAAGTCCATACAACTGGCTGGG
SRR516998.60100976.2+   ATGACGAACAGGAGGTGATGACATTTGAGCCGCAAGTCCATACAACTGGCTGGG
SRR516998.76618156.1+   ATGACGAACAGGAGGTGATGACATTTGAGCCGCAAGTCCATACAACTGGCTGGG
SRR517001.26973912.2-   ATGACGAACAGGAGGTGATGACATTTGAGCCGCAAGTCCATACAACTGGCTGGG
SRR517006.28491749.1+   ATGACGAACAGGAGGTGATGACATTTGAGCCGCAAGTCCATACAACTGGCTGGG
SRR511892.65397717.1-   ATGACGAACAGGAGGTGATGACATTTGAGCCGCAAGTCCATACAACTGGCTGGGT
SRR516968.100131508.2+   ATGACGAACAGGAGGTGATGACATTTGAGCCGCAAGTCCATACAACTGGCTGGGT
SRR516973.35471727.2+   ATGATGAACAGGAGGTGATGACATTTGAGCCGCAAGTCCATACAACTGGCTGGGT
SRR516994.74877170.1+   ATGACGAACAGGAGGTGATGACATTTGAGCCGCAAGTCCATACAACTGGCTGGGT
SRR516996.88052646.2+   ATGACGAACAGGAGGTGATGACATTTGAGCCGCAAGTCCATACAACTGGCTGGGG
SRR511912.30395815.2+   ATGACGAACAGGAGGTGATGACATTTGAGCCGCAAGTCCATACAACTGGCTGGGTG
SRR516967.87829969.2-   ATGACGAACAGGAGGTGATGACATTTGAGCCGCAAGTCCATACAACTGGCTGGGTG
SRR516998.13962236.2-   ATGACGAACAGGAGGTGATGACATTTGAGCCGCAAGTCCATACAACTGGCTGGGTG
SRR517000.29169337.1-   ATGACGAACAGGAGGTGATGACATTTGAGCCGCAAGTCCATACAACTGGCTGGGTG
SRR517008.11106831.2+   ATGACGAACAGGAGGTGATGACATTTGAGCCGCAAGTCCATACAACTGGCTGGGTG
SRR517009.71578868.2-   ATGACGAACAGGAGGTGATGACATTTGAGCTGCAAGTCCATACAACTGGCTGGGTG
SRR511892.39300111.1-   ATGACGAACAGGAGGTNATGACATTTGAGCCGCAAGTCCATACAACTGGCTGGGTGG
SRR516989.78968928.1+   ATGACGAACAGGAGGTGATGACATTTGAGCCGCAAGTCCATACAACTGGCTGGGGGG
SRR516993.72207497.1-   ATGACGAACAGGAGGTGATGACATTTGAGCCGCAAGTCCATACAACTGGCTGGGTGG
SRR517008.87918252.1+   ATGACGAACAGGAGGTGATGACATTTGAGCCGCAAGTCCATACAACTGGCTGGGTGG
SRR516968.109083542.1-   ATGACGAACAGGAGGTGATGACATTTGAGCCGCAAGTCCATACAACTGGCTGGGTGGC
SRR516968.140796502.1-   ATGACGAACACGAGGTGATGACATTTGAGCCGCAAGTCCATACAACTGGCTGGGTGGC
SRR516973.20554683.1-   ATGACGAACAGGAGGTGATGACATTTGAGCCGCAAGTCCATACAACTGGCTGGGTGGC
SRR516973.35471726.2+   ATGATGAACAGGAGGTGATGACATTTGAGCCGCAAGTCCATACAACTGGCTGGGTGGC
SRR516982.23500814.2+   ATGATGAACAGGAGGTGATGACATTTGAGCCGCAAGTCCATACAACTGGCTGGGTGGG
SRR516982.30415927.2-   ATGATGAACAGGAGGTGATGACATTTGAGCCGCAAGTCCATACAACTGGCTGGTTGGC
SRR516982.42917165.2+   ATGATGAACAGGAGGTGATGCCATTTGAGCCGCAAGTCCATACAACTGGCTGGGTGGC
SRR516993.83529308.2+   ATGACGAACAGGAGGTGATGACATTTGAGCTGCAAGTCCATACAACTGGCTGGGTGGG
SRR516996.24708888.2+   ATGACGAACAGGAGGTGATGACATTTGAGCCGCAAGTCCATACAACTGGCTGGGTGGC
SRR517004.31599976.2-   ATGACGAACAGGAGGTGATGACATTTGAGCCGCAAGTCCATACAACTGGCTGGGTGGC
SRR517004.39008760.1-   ATGACGAACAGGAGGTGATGACATTTGAGCCGCAAGTCCATACAACTGGCTGGGTGGC
SRR511912.63931928.2+   ATGACGAACAGGAGGTGATGACATTTGAGCCGCAAGTCCATACAACTGGCTGGGGGGCA
SRR511914.1000674.2+   ATGACGAACAGGAGGTGATGACATTTGAGCCGCAAGTCCATACAACTGGCTGGGGGGCC
SRR516987.61859958.2-   ATGATGAACAGGAGGTGATGACATTTGAGCCGCAAGTCCATACAACTGGCTGGTTGGCA
SRR516989.36199378.2+   ATGACGAACAGGAGGTGATGACATTTGAGCCGCAAGTCCATACAACTGGCTGGGGGGCA
SRR516989.52379004.2-   ATGACGAACAGGAGGTGATGACATTTGAGCCGCAAGTCCATACAACTGGCTGGGTGGCA
SRR517002.27629774.1+   ATGACGAACAGGAGGTGATGACATTTGAGCCGCAAGTCCATACAACTGGCTGGGGGGCA
SRR511892.23600637.2+   ATGACGAACAGGAGGTGATGACATTTGAGCCGCAAGTCCATACAACTGGCTGGGTGGCAT
SRR511892.45271449.2+   ATGACGAACAGGAGGTGATGACATTTGAGCCGCAAGTCCATACAACTGGCTGGGTGGCAT
SRR511892.6007403.1-   ATGACGAACAGGAGGTGATGACATTTGAGCCGCAAGTCCATACAACTGGCTGGGTGGCAT
SRR511892.63390781.2-   ATGACGAACAGGAGGTGATGACATTTGAGCCGCAAGTCCATACAACTGGCTGGGTGGCAT
SRR511892.64175574.2-   ATGACGAACAGGAGGTGATGACATTTGAGCCGCAAGTCCATACAACTGGCTGGGTGGCAT
SRR511892.9511611.2+   ATGACGAACAGGAGGTGATGACATTTGAGCCGCAAGTCCATACAACTGGCTGGGTGGCCT
SRR511911.19148709.2+   ATGACGAACAGGAGGTGATGACATTTGAGCCGCAAGTCCATACAACTGGCTGGGGGGCAT
SRR511911.21547512.2-   ATGACGAACAGGAGGTGATGACATTTGAGCCGCAAGTCCATACAACTGGCTGGGTGGCAT
SRR511911.3389924.2+   ATGACGAACAGGAGGTGATGACATTTGAGCCGCAAGTCCATACAACTGGCTGGGTGGCAT
SRR511911.36873194.1-   ATGACGAACAGGAGGTGATGACATTTGAGCCGCAAGTCCATACAACTGGCTGGGTGGCAT
SRR511911.38218078.2-   ATGACGAACAGGAGGTGATGACATTTGAGCCGCAAGTCCATACAACTGGCTGGGTGGCAT
SRR511911.51765582.1+   ATGACGAACAGGAGGTGATGACATTTGAGCCGCAAGTCCATACAACTGGCTGGGTGGCAT
SRR511911.58123630.1+   ATGACGAACAGGAGGTGATGACATTTGAGCCGCAAGTCCATACAACTGGCTGGGTGGCAT
SRR511911.60280636.2+   ATGACGAACAGGAGGGGATGACATTTGAGCCGCAAGTCCATACAACTGGCTGGGTGGGAT
SRR511911.7460180.1-   ATGACGAACAGGAGGTGATGACATTTGAGCCGCAAGTCCATACAACTGGCTGGGTGGCAT
SRR511912.20243841.1-   ATGACGAACAGGAGGTGATGACATTTGAGCCGCAAGTCCATACAACTGGCTGGGTGGCAT
SRR511912.59893234.1+   ATGACGAACAGGAGGTGATGACATTTGAGCCGCAAGTCCATACAACTGGCTGGGTGGCAT
SRR511912.63185812.1+   ATGACGAACAGGAGGTGATGACATTTGAGCCGCAAGTCCATACAACTGGCTGGGTGGCAT
SRR511912.63185956.1+   ATGACGAACAGGAGGTGATGACATTTGAGCCGCAAGTCCATACAACTGGCTGGGGGGCAT
SRR511912.70385865.1-   ATGACGAACAGGAGGTGATGACATTTGAGCCGCAAGTCCATACAACTGGCTGGGTGGCAT
SRR511912.83293905.1+   ATGACGAACAGGAGGTGATGACATTTGAGCCGCAAGTCCATACAACTGGCTGGGGGGCAT
SRR511913.18165035.2-   ATGACGAACAGGAGGTGATGACATTTGAGCCGCAAGTCCATACAACTGGCTGGGTGGCAT
SRR511913.22705507.2-   ATGACGAACAGGAGGTGATGACATTTGAGCCGCAAGTCCATACAACTGGCTGGGTGGCAT
SRR511913.52308827.1-   ATGACGAACAGGAGGTGATGACATTTGAGCCGCAAGTCCATACAACTGGCTGGGTGGCAT
SRR511913.70154375.2-   ATGACGAACAGGAGGTGATGACATTTGAGCCGCAAGTCCATACAACTGGCTGGGTGGCAT
SRR511913.72838419.1+   ATGACGAACAGGAGGTGATGACATTTGAGCCGCAAGTCCATACAACTGGCTGGGTGGCAT
SRR511914.19779621.1-   ATGACGAACAGGAGGTGATGACATTTGAGCCGCAAGTCCATACAACTGGCTGGGTGGCAT
SRR511914.41285120.1-   ATGACGAACAGGAGGTGATGACATTTGAGCCGCAAGTCCATACAACTGGCTGGGTGGCAT
SRR511914.45719171.1+   ATGACGAACAGGAGGTGATGACATTTGAGCCGCAAGTCCATACAACTGGCTGGGTGGCAT
SRR511914.56122848.1+   ATGACGAACAGGAGGTGATGACATTTGAGCCGCAAGTCCATACAACTGGCTGGGTGGCAT
SRR511914.80423043.1-   AGGACGAACAGGAGGTGATGACATTTGAGCCGCAAGTCCATACAACTGGCTGGGTGGCAT
SRR511915.12934870.2-   ATGACGAACAGGAGGTGATGACATTTGAGCCGCAAGTCCATACAACTGGCTGGGTGGCAT
SRR511915.51271900.2+   ATGACGAACAGGAGGTGATGACATTTGAGCCGCAAGTCCATACAACTGGCTGGGTGGCAT
SRR511915.56868915.2-   ATGACGAACAGGAGGTGATGACATTTGAGCCGCAAGTCCATACAACTGGCTGGGTGGCAT
SRR511915.717767.2+   ATGACGAACAGGAGGTGATGACATTTGAGCCGCAAGTCCATACAACTGGCTGCGGGGCAT
SRR511915.73501967.1-   ATGACGAACAGGAGGTGATGACATTTGAGCCGCAAGTCCATACAACTGGCTGGGTGGCAT
SRR516967.100239201.1+   ATGACGAACAGGGGGTGATGACATTTGAGCCGCAAGTCCATACAACTGGCTGGGTGGCAT
SRR516967.115389681.2+   ATGACGAACAGGAGGTGATGACATTTGAGCCGCAAGTCCATACAACTGGCTGGGTGGCAT
SRR516967.121468181.1-   ATGACGAACAGGAGGTGATGACATTTGAGCCGCAAGTCCATACAACTGGCTGGGTGGCAT

```
SRR516967.132651451.1-   ATGACGAACAGGAGGTGATGACATTTGAGCCGCAAGTCCATACAACTGGCTGGGTGGCAT
SRR516967.135591639.1+   ATGACGAACAGGAGGTGATGACATTTGAGCCGCAAGTCCATACAACTGGCTGGGTGGCAT
SRR516967.138148601.1-   ATGACGAACAGGAGGTGATGACATTTGAGCCGCAAGTCCATACAACTGGCTGGGTGGCAT
SRR516967.148303398.2-   ATGACGAACAGGAGGTGATGACATTTGAGCCGAAAGTCCATACAACTGGCTGGGTGGCAT
SRR516967.34973661.1+    ATGACGAACAGGAGGTGATGACATTTGAGCCGCAAGTCCATACAACTGGCTGGGTGGCAT
SRR516967.55179281.2+    ATGACGAACAGGAGGTGATGACATTTGAGCCGCAAGTCCATACAACTGGCTGGGTGGCAT
SRR516967.57741754.1+    ATGACGAACAGGAGGTGATGACATTTGAGCCGCAAGTCCATACAACTGGCTGGGTGGCAT
SRR516967.59477310.1-    ATGACGAACAGGAGGTGATAACATTTGAGCCGCAAGTCCANACAACTGGCTGGGTGGCAT
SRR516967.63778390.2-    ATGACGAACAGGAGGTGATGACATTTGAGCCGAAAGTCCATACAACTGGCTGGGTGGCAT
SRR516967.81487147.2+    ATGACGAACAGGAGNTGATGACATTTGAGCCGCAAGTCCATACAACTGGCTGGGTGGCAT
SRR516967.83672796.1-    ATGACGAACAGNAGGTGATGACATTTGAGCCGCAAGTCCATACAACTGGCTGGGTGGCAT
SRR516967.94039320.1-    ATGACGAACAGGAGGTGATGACATTTGAGCCGCAAGTCCATACAACTGGCTGGGTGGCAT
SRR516967.94416127.1-    ATGACGAACAGGAGGTGATGACATTTGAGCCGCAAGTCCATACAACTGGCTGGGTCGCAT
SRR516967.96216344.1+    ATGACGAACAGGAGGTGATGACATTTGAGCCGCAAGTCCATACAACTGGCTGGGTGGCAT
SRR516968.106610860.1-   ATGACGAACAGGAGGTGATGACATTTGAGCCGCAAGTCCATACAACTGGCTGGGTGGCAT
SRR516968.108661732.1-   ATGACGAACAGGAGGTGATGACATTTGAGCCGCAAGTCCATACAACTGGCTGGGTGGCAT
SRR516968.137574947.2+   ATGACGAACAGGAGGTGATGACATTTGAGCCGCAAGTCCATACAACTGGCTGGGTGGCAT
SRR516968.156018396.1-   ATGACGAACAGGAGGTGATGACATTTGAGCCGCAAGTCCATACAACTGGCTGGGTGGCAT
SRR516968.164894710.1+   ATGACGAACAGGAGGTGATGACATTTGAGCCGCAAGTCCATACAACTGGCTGGGTGGCAT
SRR516968.40141403.1-    ATGACGAACAGGAGGTGATGACATTTGAGCCGCAAGTCCATACAACTGGCTGGGTGGCAT
SRR516968.43850003.2-    ATGACGAACAGGAGGTGATGACATTTGAGCCGCAAGTCCATACAACTGGCTGGGTGGCAT
SRR516968.54367312.2+    ATGACGAACAGGAGGTGATGACATTTGAGCCGCAAGTCCATACAACTGGCTGGGTGGCAT
SRR516968.58859238.1+    ATGACGAACAGGAGGGGATGACATTTGAGCCGCAAGTCCATACAACTGGCTGGGCGGCAT
SRR516968.73747636.1-    ATGACGAACAGGAGGTGATGACATTTGAGCCGCAAGTCCATACAACTGGCTGGGTGGCAT
SRR516968.75435688.1-    ATGACGAACAGGAGGTGATGACATTTGAGCCGCAAGTCCATACAACTGGCTGGGTGGCAT
SRR516968.8958322.1+     ATGACGAACAGGAGGTGATGACATTTGAGCCGCAAGTCCATACAACTGGCTGGGTGGCAT
SRR516971.15050413.1-    ATGACGAACAGGAGGTGATGACATTTGAGCCGCAAGTCCATACAACTGGCTGGGTGGCAT
SRR516972.49653018.2-    ATGACGAACAGGAGGTGATGACATTTGAGCCGCAAGTCCATACAACTGGCTGGGTGGCAT
SRR516973.16005217.1-    ATGACGAACAGGAGGTGATGACATTTGAGCCGCAAGTCCATACAACTGGCTGGGTGGCAT
SRR516973.16904597.1-    ATGATGAACAGGAGGTGATGACATTTGAGCCGCAAGTCCATACAACTGGCTGGTTGGCAT
SRR516973.16906873.1-    ATGATGAACAGGAGGTGATGACATTTGAGCCGCAAGTCCATACAACTGGCTGGTTGGCAT
SRR516973.4884301.2-     ATGACGAACAGGAGGTGATGACATTTGAGCCGCAAGTCCATACAACTGGCTGGGTGGCAT
SRR516973.61866208.2+    ATGACGAACAGGAGGTGATGACATTTGAGCCGCAAGTCCATACAACTGGCTGGGTGGCAT
SRR516974.21816576.2-    ATGACGAACAGGAGGTGATGACATTTGAGCCGCAAGTCCATACAACTGGCTGGGTGGCAT
SRR516974.29282850.1+    ATGACGAACAGGAGGTGATGACATTTGAGCCGCAAGTCCATACAACTGGCTGGGTGGCAT
SRR516974.66440091.2-    ATGACGAACAGGAGGTGATGACATTTGAGCCGCAAGTCCATACAACTGGCTGGGTGGCAT
SRR516975.15347664.1-    ATGATGAACAGGAGGTGATGACATTTGAGCCGCAAGTCCATACAACTGGCTGGTTGGCAT
SRR516975.22417896.2+    ATGATGAACAGGAGGTGATGACATTTGAGCCGCAAGTCCATACAACTGGCTGGTTGGCAT
SRR516975.39714728.2+    ATGATGAACAGGAGGTGATGACATTTGAGCCGCAAGTCCATACAACTGGCTGGTTGGCAT
SRR516975.59637801.2-    ATGACGAACAGGAGGTGATGACATTTGAGCCGCAAGTCCATACAACTGGCTGGGTGGCAT
SRR516976.10068918.1-    ATGACGAACAGGAGGTGATGACATTTGAGCTGCAAGTCCATACAACTGGCTGGGTGGCAT
SRR516976.2015671.2+     ATGATGAACAGGAGGTGATGACATTTGAGCCGCAAGTCCATACAACTGGCTGGTTGGCAT
SRR516976.29296347.1-    ATGACGAACAGGAGGTGATGACATTTGAGCTGCAAGTCCATACAACTGGCTGGGTGGCAT
SRR516976.36876145.1+    ATGACGAACAGGAGGTGATGACATTTGAGCTGCAAGTCCATACAACTGGCTGGGTGGCAT
SRR516976.50893891.2-    ATGACGAACAGTAGGTGATGACATTTGAGCTGCAAGTCCATACAACTGGCTGGGTGGCAT
SRR516976.57348554.2-    ATGATGAACAGGAGGTGATGACATTTGAGCCGCAAGTCCATACAACTGGCTGGTTGGCAT
SRR516976.8946365.1-     ATGATGAACAGGAGGTGATGACATTTGAGCCGCAAGTCCATACAACTGGCTGGTTGGCAT
SRR516977.10878176.2-    ATGACGAACAGGAGGTGATGACATTTGAGCCGCAAGTCCATACAACTGGCTGGGTGGCAT
SRR516977.12820030.2+    ATGACGAACAGGAGGTGATGACATTTGAGCCGCAAGTCCATACAACTGGCTGGGGGGCAT
SRR516977.13189923.1+    ATGACGAACAGGAGGTGATGACATTTGAGCCGCAAGTCCATACAACTGGCTGGGTGGCAT
SRR516977.49992915.2-    ATGACGAACAGGAGGTGATGACATTTGAGCCGCAAGTCCATACAACTGGCTGGGTGGCAT
SRR516977.57408704.1+    ATGACGAACAGGAGGTGATGACATTTGAGCCGCAAGTCCATACAACTGGCTGGGTGGCAT
SRR516977.75947199.2+    ATGACGAACAGGAGGTGATGACATTTGAGCCGCAAGTCCATACAACTGGCTGGGTGGCAT
SRR516978.1992911.1+     ATGACGAACAGGAGGTGATGACATTTGAGCCGCAAGTCCATACAACTGGCTGGGGGGCAT
SRR516978.37638949.1-    ATGACGAACAGGAGGTGAGGACATTTGAGCCGCAAGTCCATACAACTGGCTGGGTGGCAT
SRR516978.43755925.2-    ATGACGAACAGGAGGTGATGACATTTTAGCCGCAAGTCCATACAACTGGCTGGGTGGCAT
SRR516978.60869002.2+    ATGACGAACAGGAGGTGATGACATTTGAGCCGCAAGTCCATACAACTGGCTGGGTGGCAT
SRR516978.65078285.1-    ATGACGAACAGGAGGTGATGACATTTGAGCCGCAAGTCCATACAACTGGCTGGGTGGCAT
SRR516979.10675489.2+    ATGATGAACAGGAGGTGATGACATTTGAGCCGCAAGTCCATACAACTGGCTGGTTGGCAT
SRR516979.1430425.1-     ATGACGAACAGGAGGTGATGACATTTGAGCCGCAAGTCCATACAACTGGCTGGGTGGCAT
SRR516979.6140788.2+     ATGACGAACAGGAGGTGATGACATTTGAGCCGCAAGTCCATACAACTGGCTGGGTGGCAT
SRR516980.12955843.2-    ATGACGAACAGGAGGTGATGACATTTGAGCCGCAAGTCCATACAACTGGCTGGGTGGCAT
SRR516980.19111556.1-    ATGACGAACAGGAGGTGATGACATTTGAGCCGCAAGTCCATACAACTGGCTGGGTGGCAT
SRR516980.30549936.1-    ATGACGAACAGGAGGTGATGACATTTGAGCCGCAAGTCCATACAACTGGCTGGGTGGCAT
SRR516980.32208306.1-    ATGACGAACAGGAGGTGATGACATTTGAGCCGCAAGTCCATACAACTGGCTGGGTGGCAT
SRR516980.37499938.1+    ATGACGAACAGGAGGTGATGACATTTGAGCCGCAAGTCCATACAACTGGCTGGGGGGCAT
SRR516980.63928031.1-    ATGACGAACAGGAGGTGATGACATTTGAGCCGCAAGTCCATACAACTGGCTGGGTGGCAT
SRR516982.56632493.1+    ATGATGAACAGGAGGTGATGACATTTGAGCCGCAAGTCCATACAACTGGCTGGGTGGCAT
SRR516983.41490614.2-    ATGACGAACAGGAGGTGATGACATTTGAGCCGCAAGTCCATACAACTGGCTGGGTGGCAT
SRR516983.43684823.1-    ATGACGAACAGGAGGTGATGACATTTGAGCCGCAAGTCCATACAACTGGCTGGGTGGCAT
SRR516983.54306814.2-    ATGACGAACAGGAGGTGATGACATTTGAGCCGCAAGTCCATACAACTGGCTGGGTGGCAT
SRR516983.60674262.1-    ATGACGAACAGGAGGTGATGACATTTGAGCCGCAAGTCCATACAACTGGCTGGGTGGCAT
SRR516983.94153626.2+    ATGACGAACAGGAGGTGATGACATTTGAGCCGCAAGTCCATACAACTGGCTGGGTGGCAT
SRR516984.50030865.2+    ATGACGAACAGGAGGTGATGACATTTGAGCCGCAAGTCCATACAACTGGCTGGGGGGCAT
SRR516984.56347101.1-    ATGACGAACAGGAGGTGATGACATTTGAGCCGCAAGTCCATACAACTGGCTGGGTGGCAT
SRR516984.6819096.2-     ATGACGATCAGGAGGTGATGACATTTGAGCCGCAAGTCCATACAACTGGCTGGGTGGCAT
SRR516985.70259889.1-    ATGACGAACAGGAGGTGATGACATTTGAGCCGCAAGTCCATACAACTGGCTGGTTGGCAT
SRR516985.77238548.2-    ATGACGAACAGGAGGTGATGACATTTGAGCCGCAAGTCCATACAACTGGCTGGTTGGCAT
SRR516986.10287761.2+    ATGACGAACAGGAGGTGATGACATTTGAGCCGCAAGTCCATACAACTGGCTGGGTGGCAT
SRR516987.36431135.1-    ATGACGAACAGGAGGTGATGACATTTGAGCCGCAAGTCCATACAACTGGCTGGGTGGCAT
SRR516988.2929307.2-     ATGACGAACAGGAGGTGATGACATTTGAGCCGCAAGTCCATACAACTGGCTGGGTGGCAT
SRR516988.31050597.2+    ATGACGAACAGGAGGTGATGACATTTGAGCCGCAAGTCCATACAACTGGCTGGGTGGCAT
SRR516988.32844820.2-    ATGACGAACAGGAGGTGATGACATTTGAGCCGCAAGTCCATACAACTGGCTGGGTGGCAT
SRR516989.14777251.1+    ATGACGAACAGGAGGTGATGACATTTGAGCCGCAAGTCCATACAACTGGCTGGGGGGGAA
```

SRR516989.21621378.1+	ATGACGAACAGGAGGTGATGACATTTGAGCCGCAAGTCCATACAACTGGCTGGGTGGCAT
SRR516989.36153179.1+	ATGACGAACAGGAGGTGATGACATTTGAGCCGCAAGTCCATACAACTGGCTGGGTGGCAT
SRR516989.46904169.2+	ATGACGAACAGGAGGTGATGACATTTGAGCCGCAAGTCCATACAACTGGCTGGGGGGCAT
SRR516989.75746913.1-	ATGACGAACAGGAGGTGATGACATTTGAGCCGCAAGTCCATACAACTGGCTGGGTGGCAT
SRR516989.8534348.1-	ATGACGAACAGGAGGTGATGACATTTGAGCCGCAAGTCCATACAACTGGCTGGGTGGCAT
SRR516990.20970250.1+	ATGACGAACAGGAGGTGATGACATTTGAGCCGCAAGTCCATACAACTGGCTGGGGGGCAT
SRR516990.21735027.2-	ATGACGAACAGGAGGTGATGACATTTGAGCCGCAAGTCCATACAACTGGCTGGGTGGCAT
SRR516990.32569854.2-	ATGACGAACAGGAGGTGATGACATTTGAGCCGCAAGTCCATACAACTGGCTGGGTGGCAT
SRR516990.55302390.2-	ATGACGAACAGGAGGTGATGACATTTGAGCCGCAAGTCCATACAACTGGCTGGGTGGCAT
SRR516990.68475801.1+	ATGACGAACAGGAGGTGATGACATTTGAGCCGCAAGTCCATACAACTGGCTGGGTGGCAT
SRR516991.17318347.2-	ATGACGAACAGGAGGTGATGACATTTGAGCCGCAAGTCCATACAACTGGCTGGGTGGCAT
SRR516991.1931100.1-	ATGATGAACAGGAGGTGATGACATTTGAGCCGCAAGTCCATACAACTGGCTGGTTGGCAT
SRR516991.24878492.1-	ATGACGAACAGGAGGTGATGACATTTGAGCCGCAAGTCCATACAACTGGCTGGGTGGCAT
SRR516991.30740799.1+	ATGACGAACAGGAGGTGATGACATTTGAGCCGCAAGTCCATACAACTGGCTGGGTGGCAT
SRR516991.49815040.2+	ATGATGAACAGGAGGTGATGACATTTGAGCCGCAAGTCCATACAACTGGCTGGTTGGCAT
SRR516991.7741780.1-	ATGATGAACAGGAGGTGATGACATTTGAGCCGCAAGTCCATACAACTGGCTGGTTGGCAT
SRR516992.10103608.1+	ATGACGAACAGGAGGTGATGACATTTGAGCCGCAAGTCCATACAACTGGCTGGGTGGCAT
SRR516992.23178703.2-	ATGACGAACAGGAGGTGATGACATTTGAGCCGCAAGTCCATACAACTGGCTGGGTGGCAT
SRR516992.37277386.2-	ATGACGAACAGGAGGTGATGACATTTGAGCAGCAAGACCATACAACTGGCTGGGTTGCAT
SRR516992.63008789.1-	ATGACGAACAGGAGGTGATGACATTTGAGCCGCAAGTCCATACAACTGGCTGGGTGGCAT
SRR516992.78662263.1-	ATGACGAACAGGAGGTGATGACATTTGAGCCGCAAGTCCATACAACTGGCTGGGTGGCAT
SRR516992.79918999.2+	ATGACGAACAGGAGGTGATGACATTTGAGCCGCAAGTCCATACAACTGGCTGGGTGGCAT
SRR516993.12312704.1-	ATGACGAACAGGAGGTGATGACATTTGAGCTGCAAGTCCATACAACTGGCTGGGTGGCAT
SRR516993.1447601.1+	ATGACGAACAGGAGGTGATGACATTTGAGCTGCAAGTCCATACAACTGGCTGGGTGGCAT
SRR516993.49833109.2-	ATGACGAACAGGAGGTGATGACATTTGAGCTGCAACTCCATACAACTGGCTGTGTGGCAT
SRR516993.73082870.1-	ATGACGAACAGGAGGTGATGACATTTGAGCTGCAAGTCCATACAACTGGCTGGGTGGCAT
SRR516993.75065526.2+	ATGACGAACAGGAGGTGATGACATTTGAGCCGCAAGTCCATACAACTGGCTGGGTGGCAT
SRR516993.83887266.1+	ATGACGAACAGGAGGTGATGACATTTGAGCTGCAAGTCCATACAACTGGCTGGGAGGCAT
SRR516994.1794759.2+	ATGACGAACAGGAGGTGATGACATTTGAGCCGCAAGTCCATACAACTGGCTGGGTGGCAT
SRR516994.35326807.2-	ATGACGAACAGGAGGTGATGACATTTGAGCCGCAAGTCCATACAACTGGCTGGGTGGCAT
SRR516994.76691379.2-	ATGACGAACAGGAGGTGATGACATTTGAGCCGCAAGTCCATACAACTGGCTGGGTGGCAT
SRR516994.78306274.2+	ATGACGAACAGGAGGTGATGACATTTGAGCCGCAAGTCCATACAACTGGCTGGGTGGCAT
SRR516994.82514123.2+	ATGACGAACAGGAGGTGATGACATTTGAGCCGCAAGTCCATACAACTGGCTGGGTGGCAT
SRR516995.15167538.1+	ATGACGAACAGGAGGTGATGACATTTGAGCTGCAAGTCCATACAACTGGCTGGGTGGCAT
SRR516995.15254844.2-	ATGACGAACAGGAGGTGATGACATTTGAGCTGCAAGTCCATACAACTGGCTGGGTGGCAT
SRR516995.43808656.1-	ATGACGAACAGGAGGTGATGACATTTGAGCCGCAAGTCCATACAACTGGCTGGGTGGCAT
SRR516995.74299668.1+	ATGACGAACAGGAGGTGATGACATTTGAGCCGCAAGTCCATACAACTGGCTGGGTGGCAT
SRR516996.1617304.1+	ATGACGAACAGGAGGTGATGACATTTGAGCCGCAAGTCCATACAACTGGCTGGGGGGCAT
SRR516996.29500035.2-	ATGACGAACAGGAGGTGATGACATTTGAGCCGCAAGTCCATACAACTGGCTGGGTGGCAT
SRR516996.36234607.1+	ATGACGAACAGGAGGTGATGACATTTGAGCCGCAAGTCCATACAACTGGCTGGGGGGCAT
SRR516996.52900642.2-	ATGACGAACAGGAGGTGATGACATTTGAGCCGCAAGTCCATACAACTGGCTGGGTGGCAT
SRR516996.85884676.2-	ATGACGAACAGGAGGTGATGACATTTGAGCCGCAAGTCCATACAACTGGCTGGGTGGCAT
SRR516996.88424796.2-	ATGACGAACAGGAGGTGATGACATTTGAGCCGCAAGTCCATACAACTGGCTGGGTGGCAT
SRR516997.25946564.1-	ATGATGAACAGGAGGTGATGACATTTGAGCCGCAAGTCCATACAACTGGCTGGTTGGCAT
SRR516997.44735578.1-	ATGACGAACAGGAGGTGATGACATTTGAGCCGCAAGTCCATACAACTGGCTGGGTGGCAT
SRR516997.5744761.2-	ATGACGAACAGGAGGTGATGACATTTGAGCCGCAAGTCCATACAACTGGCTGGGTGGCAT
SRR516997.66381769.1-	ATGACGAACAGGAGGTGATGACATTTGAGCCGCAAGTCCATACAACTGGCTGGGTGGCAT
SRR516997.93541116.1-	ATGACGAACAGGAGGTGATGACATTTGAGCCGCAAGTCCATACAACTGGCTGGGTGGCAT
SRR516998.43983662.1-	ATGACGAACAGGAGGTGATGACATTTGAGCCGCAAGTCCATACAACTGGCTGGGTGGCAT
SRR516998.57969943.1-	ATGACGAACAGGAGGTGATGGCATTTGAGCCGCAAGTCCATACAACTGGCTGGGTGGCAT
SRR516999.15040710.1-	ATGACGAACAGGAGGTGATGACATTTGAGCCGCAAGTCCATACAACTGGCTGGGTGGCAT
SRR516999.48060851.1-	ATGACGAACAGGAGGTGATGACATTTGAGCCGCAAGTCCATACAACTGGCTGGGTGGCAT
SRR516999.61456648.1+	ATGACGAACAGGAGGTGATGACATTTGAGCCGCAAGTCCATACAACTGGCTGGGGGGCAT
SRR516999.61850545.2+	ATGACGAACAGGAGGTGATGACATTTGAGCCGCAAGTCCATACAACTGGCTGGGTGGCAT
SRR516999.7152611.2+	ATGACGAACAGGAGGTGATGACATTTGAGCCGCAAGTCCATACAACTGGCTGGGTGGCAT
SRR516999.81087074.2+	ATGACGAACAGGAGGTGATGACATTTGAGCCGCAAGTCCATACAACTGGCTGGGTGGCAT
SRR516999.96819542.2-	ATGACGAACAGGAGGTGATGACATTTGAGCCGCAAGTCCATACAACTGGCTGGGTGGCAT
SRR517000.23191480.1+	ATGACGAACAGGAGGTGATGACATTTGAGCCGCAAGTCCATACAACTGGCTGGGTGGCAT
SRR517000.37586981.1-	ATGACGAACAGGAGGTGATGACATTTGAGCCGCAAGTCCATACAACTGGCTGGGTGGCAT
SRR517000.39251131.1+	ATGACGAACAGGAGGTGATGACATTTGAGCCGCAAGTCCATACAACTGGCTGGGGGGCAT
SRR517000.49710061.1-	ATGACGAACAGGAGGTGATGACATTTGAGCCGCAAGTCCATACAACTGGCTGGGTGGCAT
SRR517000.81566989.1+	ATGACGAACAGGAGGTGATGACATTTGAGCCGCAAGTCCATACAACTGGCTGGGTGGCAT
SRR517000.9053207.1-	ATGACGAACAGGAGGTGATGACATTTGAGCCGCAAGTCCATACAACTGGCTGGGTGGCAT
SRR517000.94486378.2-	ATGACGAACAGGAGGTGATGACATTTGAGCCGCAAGTCCATACAACTGGCTGGGTGGCAT
SRR517001.16403167.2-	ATGACGAACAGGAGGTGATGACATTTGAGCCGCAAGTCCATACAACTGGCTGGGTGGCAT
SRR517001.26562166.2-	ATGACGAACAGGAGGTGATGACATTTGAGCCGCAAGTCCATACAACTGGCTGGGTGGCAT
SRR517001.31582093.1-	ATGACGAACAGGAGGTGATGACATTTGAGCCGCAAGTCCATACAACTGGCTGGGTGGCAT
SRR517001.36464132.1-	ATGACGAACAGGAGGTGATGACATTTGAGCCGCAAGTCCATACAACTGGCTGGGTGGCAT
SRR517001.43347006.1-	ATGACGAACAGGAGGTGATGACATTTGAGCCGCAAGTCCATACAACTGGCTGGGTGGCAT
SRR517001.8564550.1+	ATGACGAACAGGAGGTGATGACATTTGAGCCGCAAGTCCATACAACTGGCTGGGTGGCAT
SRR517001.89049728.2+	ATGACGAACAGGAGGTGATGACATTTGAGCCGCAAGTCCATACAACTGGCTGGGTGGGAT
SRR517002.29394887.2+	ATGACGAACAGGAGGTGATGACATTTGAGCCGCAAGTCCATACAACTGGCTGGGGGGCAT
SRR517002.48542308.2-	ATGACGAACAGGAGGTGATGACATTTGAGCCGCAAGTCCATACAACTGGCTGGGTGGCAT
SRR517002.51438896.1-	ATGACGAACAGGAGGTGATGACATTTGAGCCGCAAGTCCATACAACTGGCTGGGTGGCAT
SRR517002.62107602.1-	ATGACGAACAGGAGGTGATGACATTTGAGCCGCAAGTCCATACAACTGGCTGGGTGGCAT
SRR517002.68785810.2+	ATGACGAACAGGAGGTGATGACATTTGAGCCGCAAGTCCATACAACTGGCTGGGTGGCAT
SRR517002.80851214.2-	ATGACGAACAGGAGGTGATGACATTTGAGCCGCAAGTCCATACAACTGGCTGGGTGGCAT
SRR517002.8665886.2-	ATGACGAACAGGAGGTGATGACATTTGAGCCGCAAGTCCATACAACTGGCTGGGTGGCAT
SRR517003.42514893.2-	ATGACGAACAGGAGGTGATGACATTTGAGCCGCAAGTCCATACAACTGGCTGGGTGGCAT
SRR517003.48327823.1+	ATGACGAACAGGAGGTGATGACATTTGAGCCGCAAGTCCATACAACTGGCTGGGTGGCAT
SRR517003.63008308.1-	ATGACGAACAGGAGGTGATGACATTTGAGCCGCAAGTCCATACAACTGGCTGGGTGGCAT
SRR517003.70018023.2-	ATGACGAACAGGAGGTGATGACATTTGAGCCGCAAGTCCATACAACTGGCTGGGTGGCAT
SRR517003.70967985.2+	ATGACGAACAGGAGGAGATGACATTTGAGCCGCAAGTCCATACAACTGGCTGGGGGGCAT
SRR517003.73936435.2-	ATGACGAACAGGAGGTGATGACATTTGAGCCGCAAGTCCATACAACTGGCTGGGTGGCAT

```
SRR517003.87371479.1+    ATGACGAACAGGAGGTGATGACATTTGAGCCGCAAGTCCATACAACTGGCTGGGTGGCAT
SRR517004.34678058.2-    ATGACGAACAGGAGGTGATGACATTTGAGCCGCAAGTCCATACAACTGGCTGGGTGGCAT
SRR517004.40113226.2+    ATGACGAACAGGAGGTGATGACATTTGAGCCGCAAGTCCATACAACTGGCTGGGTGGCAT
SRR517004.45940969.1+    ATGACGAACAGGAGGTGATGACATTTGAGCCGCAAGTCCATACAACTGGCTGGGTGGCAT
SRR517004.53693916.2-    ATGACGAACAGGAGGTGATGACATTTGAGCCGCAAGTCCATACAACTGGCTGGGTGGCAT
SRR517004.58862282.1+    ATGACGAACAGGAGGTGATGACATTTGAGCCGCAAGTCCATACAACTGGCTGGGGGGCAT
SRR517005.17700561.1-    ATGACGAACAGGAGGTGATGACATTTGAGCCGCAAGTCCATACAACTGGCTGGGTGGCAT
SRR517005.54428488.2+    ATGACGAACAGGAGGTGATGACATTTGAGCCGCAAGTCCATACAACTGGCTGGGTGGCGT
SRR517005.66404474.1+    ATGACGAACAGGAGGTGATGACATTTGAGCCGCAAGTCCATACAACTGGCTGGGTGGCAT
SRR517005.886243.1-      ATGACGAACAGGAGGTGATGACATTTGAGCCGCAAGTCCATACAACTGGCTGGGTGGCAT
SRR517005.97095694.1-    ATGACGAACAGGAGGTGATGACATTTGAGCCGCAAGTCCATACAACTGGCTGGGTGGCAT
SRR517006.43208825.1-    ATGACGAACAGGAGGTGATGACATTTGAGCCGCAAGTCCATACAACTGGCTGGGTGGCAT
SRR517006.62045893.2-    ATGACGAACAGGAGGTGATGACATTTGAGCCGCAAGTCCATACAACTGGCTGGGTGGCAT
SRR517006.79064283.2-    ATGACGAACAGGAGGTGATGACATTTGAGCCGCAAGTCCATACAACTGGCTGGGTGGCAT
SRR517006.8352318.1-     ATGACGAACAGGAGGTGATGACATTTGAGCCGCAAGTCCATACAACTGGCTGGGTGGCAT
SRR517007.20187658.2-    ATGACGAACAGGAGGTGATGACATTTGAGCCGCAAGTCCATACAACTGGCTGGGTGGCAT
SRR517007.44465454.2-    ATGACGAACAGGAGGTGATGACATTTGAGCCGCAAGTCCATACAACTGGCTGGGTGGCAT
SRR517007.5128092.2-     ATGACGAACAGGAGGTGATGACATTTGAGCCGCAAGTCCATACAACTGGCTGGGTGGCAT
SRR517007.55411417.1+    ATGACGAACAGGAGGTGATGACATTTGAGCCGCAAGTCCATACAACTGGCTGGGTGGCAT
SRR517008.37417547.1+    ATGACGAACAGGAGGTGATGACATTTGAGCCGCAAGTCCATACAACTGGCTGGGTGGCAT
SRR517008.53563245.1-    ATGACGAACAGGAGGTGATGACATTTGAGCCGCAAGTCCATACAACTGGCTGGGTGGCAT
SRR517009.2225310.1-     ATGACGAACAGGAGGTGATGACATTTGAGCTGCAAGTCCATACAACTGGCTGGGTGGCAT
SRR517009.28589393.1-    ATGACGAACAGGAGGTGATGACATTTGAGCCGCAAGTCCATACAACTGGCTGGGTGGCAT
SRR517009.31830082.2-    ATGACGAACAGGAGGTGATGACATTTGAGCCGCAAGTCCATACAACTGGCTGGGTGGCAT
SRR517009.38612123.1+    ATGACGAACAGGAGGTGATGACATTTGAGCCGCAAGTCCATACAACTGGCTGGGTGGCAT
SRR517009.51801045.2+    ATGACGAACAGGAGGTGATGACATTTGAGCCGCAAGTCCATACAACTGGCTGGGTGGCAT
SRR517009.70794059.2+    ATGACGAACAGGAGGTGATGACATTTGAGCCGCAAGTCCATACAACTGGCTGGGTGGCAT
SRR517009.74653167.2-    ATGACGAACAGGAGGTGATGACATTTGAGCTGCAAGTCCATACAACTGGCTGGGTGGCAT
SRR517009.89416108.1+    ATGACGAACAGGAGGTGATGACATTTGAGCCGCAAGTCCATACAACTGGCTGGGTGGCAT
SRR511912.13524230.1+     TGACGAACAGGAGGTGATGACATTTGAGCCGCAAGTCCATACAACTGGCTGGGTGGCAT
SRR516969.1006309.2-      TGACGAACAGGAGGTGATGACATTTGAGCCGCAAGTCCATACAACTGGCTGGGTGGCAT
SRR516981.62232392.2+     TGACGAACAGGAGGTGATGACATTTGAGCCGCAAGTCCATACAACTGGCTGGGTGGCAT
SRR516988.35895455.2+     TGACGAACAGGAGGTGATGACATTTGAGCCGCAAGTCCATACAACTGGCTGGGTGGCAT
SRR516999.67197738.1-     TGACGAACAGGAGGTGATGACATTTGAGCCGCAAGTCCATACAACTGGCTGGGTGGCAT
SRR511916.21097229.2-      GACGAACAGGAGGTGATGACATTTGAGCCGCAAGTCCATACAACTGGCT
SRR511917.35725322.2-      GACGAACAGGAGGTGATGACATTTGAGCCGCAAGTCCATACAACTGGCT
SRR511917.62920284.2-      GACGAACAGGAGGTGATGACATTTGAGCCGCAAGTCCATACAACTGGCT
SRR516970.29220836.2-      GACGAACAGGAGGTGATGACATTTGAGCCGCAAGTCCATACAACTGGCTGGGTGGCAT
SRR516970.30280066.2-      GACGAACAGGAGGTGATGACATTTGAGCCGCAAGTCCATACAACTGGCTGGGTGGCAT
SRR516981.70866488.1-      GACGAACAGAAGGGGATGACATTTGAGCCGCAAGTCCATACAACTGGCTGGGTGGCAT
SRR516998.31606571.1+      GACGAACAGGAGGTGATGACATTTGAGCCGCAAGTCCATACAACTGGCTGGGTGGCAT
SRR517003.51023813.2-      GACGAACAGGAGGTGATGACATTTGAGCCGCAAGTCCATACAACTGGCTGGGTGGCAT
SRR511915.64989562.1-       ACGAACAGGAGGTGATGACATTTGAGCCGCAAGTCCATACAACTGGCTGGGTGGCAT
SRR511915.64989699.1-       ACGAACAGGAGGTGATGACATTGGAGCCGCAAGTCCATACAACTGGCTGGGTGGCAT
SRR516967.5677042.1-        ACGAACGGGAGGGGATGACATTTGAGCCGCAAGTCCATACAACTGGCTGGGTGGCAT
SRR516978.52566920.2-       ACGAACAGGATTTGATGACATTTGAGCCGCAAGTCCATACAACTGGCTGGGTGGCAT
SRR516992.40366624.2-       ACGAACAGGAGGTGATGACATTTGAGCCGCAAGTCCATACAACTGGCTGGGTGGCAT
SRR516992.67474202.2+       ACGAACAGGAGGTGATGACATTTGAGCCGCAAGTCCATACAACTGGCTGGGTGGCAT
SRR516992.79411570.1-       ACGAACAGGAGGTGATGACATTTGAGCCGCAAGTCCATACAACTGGCTGGGTGGCAT
SRR516998.45967480.1-       ACGAACAGGAGGTGATGACATTTGAGCCGCAAGTCCATACAACTGGCTGGGTGGCAT
SRR517001.81299431.1+       ACGAACAGGAGGTGATGACATTTGAGCCGCAAGTCCATACAACTGGCTGGGTGGCAT
SRR511892.9368196.1+         CGAACAGGAGGTGATGACATTTGAGCCGCAAGTCCATACAACTGGCTGGGTGGCAT
SRR511911.54079531.2+        CGAACAGGAGGCGATGACATTTGAGCCGCAAGTCCATACAACTGGCTGGGTGGCAT
SRR516981.21374378.2-        CGAACAGGAGGTGATGACATTTGAGCCGCAAGTCCATACAACTGGCTGGGTGGCAT
SRR516993.5077793.2+         CGAACAGGAGGTGATGACATTTGAGCTGCAAGTCCATACAACTGGCTGGGTGGCAT
SRR516996.75662898.1-        CGAACAGGAGGTGATGACATTTGAGCCGCAAGTCCATACAACTGGCTGGGTGGCAT
SRR517004.41051761.2+        CGAACAGGAGGTGATGACATTTGAGCCGCAAGTCCATACAACTGGCTGGGTGGCAT
SRR517005.46088960.1-        CGAACAGGAGGTGATGACATTTGAGCCGCAAGTCCATACAACTGGCTGGGTGGCAT
SRR517009.48171529.2-        CGAACAGGAGGTGATGACATTTGAGCTGCAAGTCCATACAACTGGCTGGGTGGCAT
SRR511916.33544803.1+         GAACAGGAGGTGATGACATTTGAGCCGCAAGTCCATACAACTGGCTGGG
SRR511916.64388886.2+         GAACAGGAGGTGATGACATTTGAGCCGCAAGTCCATACAACTGGCTGGG
SRR511916.64388890.2+         GAACAGGAGGTGATGACATTTGAGCCGCAAGTCCATACAACTGGCTGGG
SRR516970.7484751.2+          GAACAGGAGGTGATGACATTTGAGCCGCAAGTCCATACAACTGGCTGGGTGGCAT
SRR516984.53836077.1-         GAACAGGAGGTGATGACATTTGAGCCGCAAGTCCATACAACTGGCTGGGTGGCAT
SRR516988.7333166.1-          GAACAGGAGGTGATGACATTTGAGCCGCAAGTCCATACAACTGGCTGGGTGGCAT
SRR516994.20458023.1+         GAACAGGAGGTGATGACATTTGAGCCGCAAGTCCATACAACTGGCTGGGTGGCAT
SRR516994.6814432.2+          GAACAGGAGGTGATGACATTTGAGCCGCAAGTCCATACAACTGGCTGGGTGGCAT
SRR516996.49779749.1+         GAACAGGAGGTGATGACATTTGAGCCGCAAGTCCATACAACTGGCTGGGTGGCAT
SRR517000.103614309.2+        GAACAGGAGGAGATGACATTTGAGCCCCAAGGCCATACAACTGGCTGGGAGGCAT
SRR517008.68021994.2-         GAACAGGAGGTGATGACATTTGAGCCGCAAGTCCATACAACTGGCTGGGTGGCAT
SRR511916.23726709.2-          AACAGGAGGTGATGACATTTGAGCCGCAAGTCCATACAACTGGCTGGGT
SRR511916.25107412.2+          AACAGGAGGTGATGACATTTGAGCCGCAAGTCCATACAACTGGCTGGGT
SRR511916.54907786.2-          AACAGGAGGTGATGACATTTGAGCCGCAAGTCCATACAACTGGCTGGGT
SRR511917.60109382.2-          AACAGGAGGTGATGACATTTGAGCCGCAAGTCCATACAACTGGCTGGGT
SRR511917.7621459.2-           AACAGGAGGTGATGACATTTGAGCCGCAAGTCCATACAACTGGCTGGGT
SRR516968.123158278.1+         AACAGGAGGTGATGACATTTGAGCCGCAAGTCCATACAACTGGCTGGGTGGCAT
SRR516978.37914558.1+          AACAGGAGGTGATGACATTTGAGCCGCAAGTCCATACAACTGGCTGGGTGGCAT
SRR516981.46321294.2+          AACAGGAGGTGATGACATTTGAGCCGCAAGTCCATACAACTGGCTGGGTGGCAT
SRR516989.64914875.2-          AACAGGAGGTGATGACATTTGAGCCGCAAGTCCATACAACTGGCTGGGTGGCAT
SRR516996.32218127.1-          AACAGGAGGTGATGACATTTGAGCCGCAAGTCCATACAACTGGCTGGGTGGCAT
SRR516998.88636739.2-          AACAGGAGGTGATGACATTTGAGCCGCAAGTCCATACAACTGGCTGGGTGGCAT
SRR517002.99266758.2+          AACAGGAGGTGATGACATTTGAGACGCAAGTCCATACAACTGGATGGGAGGCAT
SRR517006.17938695.2+          AACAGGAGGTGATGACATTTGAGCCGCAAGTCCATACAACTGGCTGGGTGGCAT
SRR511911.1070600.2+            ACAGGAGGTGATGACATTTGAGCCGCAAGTCCATACAACTGGCTGGGTGGCAT
```

SRR511915.27615657.2+ ACAGGAGGTGATGACATTTGAGCCGCAAGTCCATACAACTGGCTGGGTGGCAT
SRR516969.9561270.1- ACAGGAGGTGATGACATTTGAGCCGCAAGTCCATACAACTGGCTGGGTGGCAT
SRR516978.14954997.2+ ACAGGAGGTGATGACATTTGAGCCGCAAGTCCATACAACTGGCTGGGTGGCAT
SRR516979.9712358.1- ACAGGAGGTGATAACATTTGAGCCGCAAGTCCATACAACTGGCTGGGTGGCAT
SRR516986.23230956.2+ ACAGGAGGTGATGACATTTGAGCCGCAAGTCCATACAACTGGCTGGGTGGCAT
SRR516987.33930642.2+ ACAGGAGGTGATGACATTTGAGCCGCAAGTCCATACAACTGGCTGGGTGGCAT
SRR516988.52138870.2+ ACAGGAGGTGATGACATTTGAGCCGCAAGTCCATACAACTGGCTGGGTGGCAT
SRR516994.75814175.2- ACAGGAGGTGATGACATTTGAGCCGCAAGTCCATACAACTGGCTGGGTGGCAT
SRR516998.62923474.1+ ACAGGAGGTGATGACATTTGAGCCGCAAGTCCATACAACTGGCTGGGGGGCAT
SRR517001.80318310.2- ACAGGAGGTGATGACATTTGAGCCGCAAGTCCATACAACTGGCTGGGTGGCAT
SRR511918.3443919.2+ CAGGAGGTGATGACATTTGAGCCGCAAGTCCATACAACTGGCTGGGGGG
SRR511913.40896479.2+ CAGGAGGTGATGACATTTGAGCCGCAAGTCCATACAACTGGCTGGGGGGCAT
SRR516972.34696103.2+ CAGGAGGTGATGACATTTGAGCCGCAAGTCCATACAACTGGCTGGGTGGCAT
SRR516975.38025761.2- CAGGAGGTGATGACATTTGAGCCGCAAGTCCATACAACTGGCTGGGTGGCAT
SRR516977.40786703.2- CAGGAGGTGATGACATTTGAGCCGCAAGTCCATACAACTGGCTGGGTGGCAT
SRR516977.56138604.2- CAGGAGGTGATGACATTTGAGCCGCAAGTCCATACAACTGGCTGGGTGGCAT
SRR516988.23560143.1+ CAGGAGGTGATGACATTTGAGCCGCAAGTCCATACAACTGGCTGGGTGGCAT
SRR517003.83174067.1+ CAGGAGGTGATGACATTTGAGCCGCAAGTCCATACAACTGGCTGGGTGGCAT
SRR517004.25798813.1+ CAGGAGGTGATGACATTTGAGCCGCAAGTCCATACAACTGGCTGGGTGGCAT
SRR511918.18378839.1+ AGGAGGTGATGACATTTGAGCCGCAAGTCCATACAACTGGCTGGGTGGC
SRR511918.21356526.1+ AGGAGGTGATGACATTTGAGCCGCAAGTCCATACAACTGGCTGGGTGGC
SRR511918.22825667.1+ AGGAGGTGATGACATTTGAGCCGCAAGTCCATACAACTGGCTGGGTGGC
SRR511914.11127656.1- AGGAGGTGATAACATTTGAGCCGCAAGTCCATACAACTGGCTGGGTGGCAT
SRR511914.34655921.2- AGGAGGTGATGACATTTGAGCCGCAAGTCCATACAACTGGCTGGGTGGCAT
SRR516972.32806802.1- AGGAGGTGATGACATTTGAGCCGCAAGTCCATACAACTGGCTGGGTGGCAT
SRR516975.13713707.1- AGGAGGTGATGACATTTGAGCCGCAAGTCCATACAACTGGCTGGGTGGCAT
SRR516975.31280279.1- AGGAGGTGATGACATTTGAGCCGCAAGTCCATACAACTGGCTGGGTGGCAT
SRR517000.18261147.1+ AGGAGGTGATGACATTTGAGCCGCAAGTCCATACAACTGGCTGGGTGGCAT
SRR517004.4165455.2+ AGGAGGTGATGACATTTGAGCCGCAAGTCCATACAACTGGCTGGGTGGCAT
SRR517007.15671468.2+ AGGAGGTGATGACATTTGAGCCGCAAGTCCATACAACTGGCTGGGGGGCAT
SRR517008.29824779.2- AGGAGGTGATGACATTTGAGCCGCAAGTCCATACAACTGGCTGGGTGGCAT
SRR516978.56562036.1+ GGAGGTGATGACATTTGAGCCGCAAGTCCATACAACTGGCTGGGTGGCAT
SRR516993.8072866.1- GGAGGTGATGACATTTGAGCCGCAAGTCCATACAACTGGCTGGGTGGCAT
SRR517009.71698028.2- GGAGGTGATGACATTTGAGCCGCAAGTCCATACAACTGGCTGGGTGGCAT
SRR516984.6644703.2- GAGGTGATGACATTTGAGCCGCAAGTCCATACAACTGGCTGGGTGGCAT
SRR516988.41673661.2- GAGGTGATGACATTTGAGCCGCAAGTCCATACAACTGGCTGGGTGGCAT
SRR516993.23735352.1- GAGGTGATGACATTTGAGCTGCAAGTCCATACAACTGGCTGGGTGGCAT
SRR516998.74208434.1- GAGGTGATGACATTTGAGCCGCAAGTCCATACACCTGGCTGGGTGGCAT
SRR517000.56225435.1- GAGGTGATGACATTTGAGCCGCAAGTCCATACAACTGGCTGGGTGGCAT
SRR517007.43638454.1- GAGGTGATGACATTTGAGCCGCAAGTCCATACAACTGGCTGGGTGGCAT
SRR517009.35713352.2- GAGGTGATGACATTTGAGCCGCAAGTCCATACAACTGGCTGGGTGGCAT
SRR511892.42564732.2- AGGTGATGACATTTGAGCCGCAAGTCCATACAACTGGCTGGGTGGCAT
SRR511892.56496978.1- AGGTGATGACATTTGAGCCGCAAGTCCATACAACTGGCTGGGTGGCAT
SRR511915.30654048.2+ AGGTGATGACATTTGAGCCGCAAGTCCATACAACTGGCTGGGTGGCAT
SRR516980.72482160.1- AGGTGATGACATTTGAGCCGCAAGTCCATACAACTGGCTGGGTGGCAT
SRR517000.54956038.1- AGGTGATGACATTTGAGCCGCAAGTCCATACAACTGGCTGGGTGGCAT
SRR511916.20302767.1+ GGTGATGACATTTGAGCCGCAAGTCCATACAACTGGCTGGGTGGCAT
SRR516972.35896448.1- GGTGATGACATTTGAGCCGCAAGTCCATACAACTGGCTGGGTGGCAT
SRR516972.36494147.2+ GGTGATGACATTTGAGCCGCAAGTCCATACAACTGGCTGGGGGGCAT
SRR516980.58810552.1- GGTGATGACATTTGAGCCGCAAGTCCATACAACTGGCTGGGTGGCAT
SRR516992.78415230.2- GGTGATGACATTTGAGCCGCAAGTCCATACAACTGGCTGGGTGGCAT
SRR516997.48515103.2- GGTGATGACATTTGAGCCGCAAGTCCATACAACTGGCTGGGTGGCAT
SRR511892.22200889.2- GTGATGACATTTGAGCCGCAAGTCCATACAACTGGCTGGGTGGCAT
SRR511915.74307710.2- GTGATGACATTTGAGCCGCAAGTCCATACAACTGGCTGGGTGGCAT
SRR516978.384904.2- GTGATGACATTTGAGCCGCAAGTCCATACAACTGGCTGGGTGGCAT
SRR516981.20078478.1- GTGATGACATTTGAGCCGCAAGTCCATACAACTGGCTGGGTGGCAT
SRR516998.86192854.2+ GTGATGACATTTGAGCCGCAAGTCCATACAACTGGCTGGGTGGCAT
SRR516999.49626087.2- GTGATGACATTTGAGCCGCAAGTCCATACAACTGGCTGGGTGGCAT
SRR516972.47863115.2- TGATGACATTTGAGCCGCAAGTCCATACAACTGGCTGGGTGGCAT
SRR516979.58015851.1+ TGATGACATTTGAGCCGCAAGTCCATACAACTGGCTGGGTGGCAT
SRR516996.92350992.2- TGATGACATTTGAGCCGCAAGTCCATACAACTGGCTGGGTGGCAT
SRR516997.86440148.2+ TGATGACATTTGAGCCGCAAGTCCATACAACTGGCTGGGGGGCAT
SRR517005.8503673.2+ TGATGACATTTGAGCCGCAAGTCCATACAACTGGCTGGGTGGCAT
SRR517008.51758338.2+ TGATGACATTTGAGCCGCAAGTCCATACAACTGGCTGGGGGGCAT
SRR517008.6483545.2- TGATGACATTTGAGCCGCAAGTCCATACAACTGGCTGGGTGGCAT
SRR517009.11972102.1+ TGATGACATTTGAGCTGCAAGTCCATACAACTGGCTGGGTGGCAT
SRR511892.6619238.1+ GATGACATTTGAGCCGCAAGTCCATACAACTGGCTGGGTGGCAT
SRR516968.147400290.1+ GATGACATTTGAGCCGCAAGTCCATACAACTGGCTAGGTGGCAT
SRR516978.72874464.2- GATGCCATTGGAGCCGCAAGTCCATACAACTGGCTGGGTGGCAT
SRR516995.23487131.1- GATGACATTTGAGCTGCAAGTCCATACAACTGGCTGGGTGGCAT
SRR516997.92391410.2- GATGACATTTGAGCCGCAAGTCCATACAACTGGCTGGGTGGCAT
SRR511914.24386047.1+ ATGACATTTGAGCCGCAAGTCCATACAACTGGCTGGGTGGGAT
SRR511914.8117859.1- ATGACATTTGAGCCGCAAGTCCATACAACTGGCTGGGTGGCAT
SRR516967.149674807.2- ATGACATTTGAGCCGCAAGTCCATACAACTGGCTGGGTGGCAT
SRR516987.44128152.2+ ATGACATTTGAGCCGCAAGACCATACAACTGGCTGGGTGGCCT
SRR516997.34496168.2- ATGACATTTGAGCCGCAAGTCCATACAACTGGCTGGGTGGCAT
SRR516998.87299695.1- ATGACATTTGAGCCGCAAGTCCATACAACTGGCTGGGTGGCAT
SRR517000.7065464.1+ ATGACATTTGAGCCGCAAGTCCATACAACTGGCTGGGAGGCAT
SRR517009.108104.1+ ATGACATTTGAGCCGCAAGTCCATACAACTGGCTGGGTGGCAT
SRR517009.87547697.2- ATGACATTTGAGCTGCAAGTCCATACAACTGGCTAAGTGGCAT
SRR511911.2865577.2+ TGACATTTGAGCCGCAAGTCCATACAACTGGCTGGGTGGCAT
SRR516991.44574167.1+ TGACATTTGAGCCGCAAGTCCATACAACTGGCTGGGTGGCAT
SRR516994.41842983.2- TGACATTTGAGCCGCAAGTCCATACAACTGGCTGGGTGGCAT
SRR517001.32246971.1- TGACATTTGAGCCGCAAGTCCATACAACTGGCTGGGTGGCAT

SRR517008.32872042.2-	TGACATTTGAGCCGCAAGTCCATACAACTGGCTGGGTGGCAT
SRR517009.48718939.2-	TGACATTTGAGCTGCAAGTCCATACAACTGGCTGGGTGGCAT
SRR517009.54064618.2-	TGACATTTGAGCTGCAAGTCCATACAACTGGCTGGGTGGCAT
SRR511892.12533835.1+	GACATTTGAGCCGCAAGTCCATACAACTGGCTGGGTGGCAT
SRR516981.35980793.1+	GACATTTGAGCCGCAAGTCCATACAACTGGCTGGGTGGCAT
SRR516984.19133805.1-	GACATTTGAGCCGCAAGTCCATACAACTGGCTGGGTGGCAT
SRR516991.47909941.1+	GACATTTGAGCCGCAAGTCCATACAACTGGCTGGGTGGCAT
SRR516993.16481734.2-	GACATTTGAGCCGCAAGTCCATACAACTGGCTGGGTGGCAT
SRR516994.49842132.2-	GACATTTGAGCCGCAAGTCCATACAACTGGCTGGGTGGCAT
SRR516996.28202039.2-	GACATTTGAGCCGCAAGTCCATACAACTGGCTGGGTGGCAT
SRR516996.72151079.1+	GACATTTGAGCCGCAAGTCCATACAACTGGCTGGGTGGCAT
SRR517000.7876007.1-	GACATTTGAGCCGCAAGTCCATACAACTGGCTGGGTGGCAT
SRR517000.81693984.2-	GACATTTGAGCCGCAAGTCCATACAACTGGCTGGGTGGCAT
SRR517007.19456209.1-	GACATTTGAGCCGCAAGTCCATACAACTGGCTGGGTGGCAT
SRR511916.62302427.1+	ACATTTGAGCCGCAAGTCCATACAACTGGCTGGGTGGCAT
SRR511916.67819900.1+	ACATTTGAGCCGCAAGTCCATACAACTGGCTGGGTGGCAT
SRR516967.103239374.2+	ACATTTGAGCCGCAAGTCCATACAACTGGCTGGGTGGCAT
SRR516967.128996446.1-	ACATTTGAGCCGCAAGTCCATACAACTGGCTGGGTGGCAT
SRR516967.62165391.1-	ACATTTGAGCCGCAAGTCCATACAACTGGCTGGGTGGCAT
SRR516978.26253784.2-	ACATTTGAGCCGCAAGTCCATACAACTGGCTGGGTGGCAT
SRR516978.39130629.2+	ACATTTGAGCCGCAAGTCCATACAACTGGCTGGGTGGCAT
SRR516979.65960194.2-	ACATTTGAGCCGCAAGTCCATACAACTGGCTGGGTGTCAT
SRR516981.13235658.1-	ACATTTGAGCCGCAAGTCCATACAACTGGCTGGGTGGCAT
SRR516995.47487143.1+	ACATTTGAGCCGCAAGTCCATACAACTGGCTGGGTGGCAT
SRR517004.20569530.2-	ACATTTGAGCCGCAAGTCCATACAACTGGCTGGGTGGCAT
SRR517001.24362772.2+	CATTTGAGCCGCAAGTCCATACAACTGGCTGGGTGGCAT
SRR517003.41478862.1+	CATTTGAGCCGCAAGTCCATACAACTGGCTGGGTGGCAT
SRR511892.53446249.1-	ATTTGAGCCGCAAGTCCATACAACTGGCTGGGTGGCAT
SRR511914.12156601.2-	ATTTGAGCCGCAAGTCCATACAACTGGCTGGGTGGCAT
SRR516971.18513477.2-	ATTTGAGCCGCAAGTCCATACAACTGGCTGGGTGGCAT
SRR516975.66584327.2-	ATTTGAGCCGCAAGTCCATACAACTGGCTGGGTGGCAT
SRR516986.29707316.1+	ATTTGAGCCGCAAGTCCATACAACTGGCTGGGGGGCAT
SRR516994.53221266.2+	ATTTGAGCCGCAAGTCCATACAACTGGCTGGGGGGCAT
SRR516996.68386100.2+	ATTTGAGCCGCAAGTCCATACAACTGGCTGGGTGGCAT
SRR516996.755157.2-	ATTTGAGCCGCAAGTCCATACAACTGGCTGGGTGGCAT
SRR516998.784538.2-	ATTTGAGCCGCAAGTCCATACAACTGGCTGGGTGGCAT
SRR517001.70847586.2-	ATTTGAGCCGCGAGTCCATACAACTGGCTGGGTGGCAT
SRR511892.4161180.1+	TTTGAGCCGCAAGTCCATACAACTGGCTGGGTGGCAT
SRR511916.29283073.1+	TTTGAGCCGCAAGTCCATACAACTGGCTGGGTGGCAT
SRR516972.46208961.2-	TTTGAGCCGCAAGTCCATACAACTGGCTGGGTGGCAT
SRR516974.27143600.1+	TTTGAGCCGCAAGTCCATACAACTGGCTGGGTGGCAT
SRR516997.7493563.2-	TTTGAGCCGCAAGTCCATACAACTGGCTGGGTGGCAT
SRR511913.46504761.2-	TTGAGCCGCAAGTCCATACAACTGGCTGGGTGGCAT
SRR511917.48425344.1-	TTGAGCCGCAAGTCCATACAACTGGCTGGGTGGCAT
SRR516974.54645088.1-	TTGAGCCGCAAGTCCATACAACTGGCTGGGTGGCAT
SRR516980.15802379.2-	TTGATCTGCAATTCCATACAACTGGCTGGGTGGCAT
SRR516983.52981551.1+	TTGAGCCGCAAGTCCATACAACTGGCTGGGTGGCAT
SRR516987.22815449.2-	TTGAGCCGCAAGTCCATACAACTGGCTGGGTGGCAT
SRR517005.91348161.2-	TTGAGCCGCAAGTCCATACAACTGGCTGGGTGGCAT
SRR511892.54818066.1+	TGAGCCGCAAGTCCATACAACTGGCTGGGTGGCAT
SRR516974.11239183.1+	TGAGCCGCAAGTCCATACAACTGGCTGGGTGGCAT
SRR516974.60730690.1-	TGAGCCGCAAGTCCATACAACTGGCTGGGTGGCAT
SRR516983.52187946.1-	TGAGCCGCAAGTCCATACAACTGGCTGGGTGGCAT
SRR516994.13378760.2+	TGAGCCGCAAGTCCATACAACTGGCTGGGTGGCAT
SRR516995.89724041.1+	TGAGCTGCAAGTCCATACAACTGGCTGGGTGGCAT
SRR517000.35171247.1-	TGAGCCGCAAGTCCATACAACTGGCTGGGTGGCAT
SRR517003.58518606.1+	TGAGCCGCAAGTCCATACAACTGGCTGGGTGGCAT
SRR511915.28490755.2-	GAGCGGCAAGTCCATACAACTGGCTGGGTGGCAT
SRR511915.2930610.2-	GAGCCGCAAGTCCATACAACTGGCTGGGTGGCAT
SRR516994.57038585.2-	GAGCCGCAAGTCCATACAACTGGCTGGGTGGCAT
SRR517009.61399791.2+	GAGCCGCAAGTCCATACAACTGGCTGGGTGGCAT
SRR511914.6072833.2-	AGCCGCAAGTCCACACAACTGGCTGGGTGGCAT
SRR516971.28756462.1+	AGCCGCAAGTCCATACAACTGGCTGGGTGGCAT
SRR516979.61219488.2-	AGCCGCAAGTCCATACAACTGGCTGGGTGGCAT
SRR516986.14380990.1-	AGCCGCAAGTCCATACAACTGGCTGGGTGGCAT
SRR516995.62719574.1-	AGCTGCAAGTCCATACAACTGGCTGGGTGGCAT
SRR516996.81711883.2-	AGCCGCAAGTCCATACGACTGGCTGGGTGGCAT
SRR517006.14315108.1-	AGCCGCAAGTCCATACAACTGGCTGGGTGGCAT
SRR511914.85321226.1+	GCCGCAAGTCCATACAACTGGCTGGGTGGCAT
SRR511914.85321569.1+	GCCGCAAGTCCATACAACTGGCTGGGTGGCAT
SRR516975.20770772.2-	GCCGCAAGTCCATACAACTGGCTGGGTGGCAT
SRR516978.64514468.1+	GCCGCAAGTCCATACAACTGGCTGGGGGGCAT
SRR516986.47797799.1+	GCCGCAAGTCCATACAACTGGCTGGGTGGCAT
SRR517002.42093965.1-	GCCGCAAGTCCATACAACTGGCTGGGTGGCAT
SRR516974.43488651.1+	CCGCAAGTCCATACAACTGGCTGGGTGGCAT
SRR516979.57710857.2-	CCGCAAGTCCATACAACTGGCTGGGTGGCAT
SRR517007.51673519.2-	CCGCAAGTCCATACAACTGGCTGGGTGGCAT
SRR517009.90312363.1-	CCGCAAGTCCATACAACTGGCTGGGTGGCAT
SRR511892.42669582.2+	CGCAAGTCCATACAACTGGCTGGGTGGCAT
SRR511911.24730437.2+	CGCAAGTCCATACAACTGGCTGGGTGGCAT
SRR511912.18837352.1+	CGCAAGTCCATACAACTGGCTGGGTGGCAT
SRR511914.6591292.2+	CGCAAGTCCATACAACTGGCTGGGTGGCAT
SRR516967.30072848.2+	CGCAAGTCCATACAACTGGCTGGGTGGCAT
SRR516974.45483276.1-	CGCAAGTCCATACAACTGGCTGGGTGGCAT

SRR516974.72219416.1-	CGCAAGTCCATACAACTGGCTGGGTGGCAT
SRR516980.61023510.2+	CGCAAGTCCATACAACTGGCTGGGTGGCAT
SRR516993.472471.2+	CGCAAGTCCATACAACTGGCTGGGTGGCAT
SRR517009.36170965.2+	TGCAAGTCCATACAACTGGCTGGGTGGCAT
SRR517009.46333.1+	CGCAAGTCCATACAACTGGCTGGGTGGCAT
SRR516967.44398778.2+	GCAAGTCCATACAACTGGCTGGGTGGCAT
SRR516994.44481978.2-	GCAAGTCCATACAACTGGCTGGGTGGCAT
SRR516994.49855553.1+	GCAAGTCCATACAACTGGCTGGGTGGCAT
SRR516994.77692468.2+	GCAAGTCCATACAACTGGCTGGGTGGCAT
SRR517004.35890882.2+	GCAAGTCCATACAACTGGCTGGGTGGCAT
SRR516967.140688955.2-	CAAGTCCATACAACTGGCTGGGTGGCAT
SRR516977.16216859.1+	CAAGTCCATACAACTGGCTGGGTGGCAT
SRR516977.5387374.2+	CAAGTCCATACAACTGGCTGGGTGGCAT
SRR516986.38251531.2-	CAAGTCCATACAACTGGCTGGGTGGCAT
SRR516998.42387076.2-	CAAGTCCATACAACTGGCTGGGTGGCAT
SRR516972.30272084.2-	AAGTCCATACAACTGGCTGGGTGGCAT
SRR516991.78056440.1-	AAGTCCATACAACTGGCTGGGTGGCAT
SRR516981.75185944.1-	AGTCCATACAACTGGCTGGGTGGCAT
SRR516983.72843190.1-	AGTCCATACAACTGGCTGGGTGGCAT
SRR516984.67730810.2-	AGTCCATACAACTGGCTGGGTGGCAT
SRR516991.46156534.1-	AGTCCATACAACTGGCTGGGTGGCAT
SRR516996.23482257.1-	AGTCCATACAACTGGCTGGGTGGCAT
SRR511915.60393449.2-	GTCCATACAACTGGCTGGGTGGCAT
SRR516971.34870911.2+	GTCCATACAACTGGCTGGGTGGCAT
SRR516987.72954228.2-	GTCCATACAACTGGCTGGGTGGCAT
SRR516994.81814726.1-	GTCCATACAACTGGCTGGGTGGCAT
SRR517002.88618213.1+	GTCCATACAACTGGCTGGGTGGCAT
SRR517003.19490054.2-	GTCCATACAACTGGCTGGGTGGCAT
SRR517008.87918252.2-	GTCCATACAACTGGCTGGGTGGCAT
SRR516978.58338306.2+	TCCATACAACTGGCTGGGTGGCAT
SRR517000.104217922.1-	TCCATACAACTGGCTGGGTGGCAT
SRR517002.77942367.2-	TCCATACAACTGGCTGGGTGGCAT
SRR517008.65933108.2-	TCCATACAACTGGCTGGGTGGCAT
SRR511913.69503215.1+	CCATACAACTGGCTGGGTGGCAT
SRR516974.4422789.2+	CCATACAACTGGCTGGGTGGCAT
SRR516978.26037079.1+	CCATACAACTGGCTGGGTGGCAT
SRR516989.82467866.2-	CCATACAACTGGCTGGGTGGCAT
SRR516992.6010329.1-	CCATACAACTGGCTGGGTGGCAT
SRR516995.52974433.1+	CCATACAACTGGCTGGGTGGCAT
SRR516968.163565941.2+	CATACAACTGGCTGGGTGGCAT
SRR516972.83556354.1+	CATACAACTGGCTGGGTGGCAT
SRR516978.39183046.2+	CATACAACTGGCTGGGTGGCAT
SRR516988.25345425.2+	CATACAACTGGCTGGGGGGCAT
SRR516995.66321276.1+	CATACAACTGGCTGGGTGGCAT
SRR517008.9761471.2+	CATACAACTGGCTGGGTGGCAT
SRR517009.35173062.1-	CATACAACTGGCTGGGGGGCAT
SRR517009.90290402.2+	CATACAACTGGCTGGGTGGCAT
SRR516968.33193255.1+	ATACAACTGGCTGGGTGGCAT
SRR516984.88862730.1-	ATACAACTGGCTGGGTGGCAT
SRR516989.23071515.2-	ATACAACTGGCTGGGTGGCAT
SRR516994.52852729.1+	TACAACTGGCTGGGTGGCAT
SRR516996.87236229.2+	TACAACTGGCTGGGTGGCAT
SRR511913.43839483.2+	ACAACTGGCTGGGTGGCAT
SRR516969.35394539.2-	ACAACTGGCTGGGTGGCAT
SRR516990.47204932.2+	ACAACTGGCTGGGTGGCAT
SRR516991.23448506.1+	ACAACTGGCTGGGTGGCAT
SRR517001.2955954.2+	ACAACTGGCTGGGTGGCAT
SRR517005.97174151.1-	ACAACTGGCTGGGTGGCAT
SRR511911.56187465.1+	CAACTGGCTGGGTGGCAT
SRR516986.5284326.1-	CAACTGGCTGGGTGGCAT
SRR516998.74633545.1+	CAACTGGCTGGGTGGCAT
SRR516980.23012010.2+	AACTGGCTGGGTGGCAT
SRR516980.51880329.2-	AACTGGCTGGGTGGCAT
SRR516990.60531713.1-	AACTGGCTGGGTGGCAT
SRR516996.92591042.1+	AACTGGCTGGGTGGCAT
SRR516998.105713546.1-	AACTGGCTGGGTGGCAT
SRR516999.51761.1+	AACTGGCTGGGTGGCAT
SRR517002.88668875.2-	AACTGTCTGGGTGGCAT
SRR517009.79220826.2-	AACTGGCTGGGTGGCAT
SRR511912.43892789.1-	ACTGGCTGGGTGGCAT
SRR511913.2846960.2+	ACTGGCTGGGGGGCAT
SRR516968.21564094.2-	ACTGGCTGGGTGGCAT
SRR516981.10390610.2-	ACTGGCTGGGTGGCAT
SRR516980.15856106.1-	CTGGCTGGGTGGCAT
SRR516986.47463977.1-	CTGGCTGGGTGGCAT
SRR516994.3610095.1+	CTGGCTGGGTGGCAT
SRR517006.19437322.1-	CTGGCTGGGTGGCAT
SRR511914.15324340.2+	TGGCTGGGTGGCAT
SRR511914.15324684.2+	TGGCTGGGTGGCAT
SRR516974.31202954.1+	TGGCTGGGTGGCAT
SRR516974.54553033.2+	TGGCTGGGTGGCAT
SRR516989.53465710.1-	TGGCTGGGTGGCAT
SRR516992.55475284.2+	TGGCTGGGTGGCAT
SRR516995.928256.2+	TGGCTGGGTGGCAT

SRR516996.34976669.1+	TGGCTGGGTGGCAT
SRR511911.74696838.2+	GGCTGGGTGGCAT
SRR516977.10847396.1+	GGCTGGGTGGCAT
SRR516992.45921129.1+	GGCTGGGTGGCAT
SRR517003.18297409.2-	GGCTGGGTGGCAT
SRR511912.1303277.1-	GCTGGGTGGCAT
SRR511915.30731583.2-	GCTGGGTGGCAT
SRR511920.29932217.1-	GCTGGGTGGCAT
SRR511920.31903781.1-	GCTGGGTGGCAT
SRR511920.34826249.1-	GCTGGGTGGCAT
SRR511920.37463379.1-	GCTGGGTGGCAT
SRR511920.43179619.1-	GCTGGGTGGCAT
SRR511920.45487406.1-	GCTGGGTGGCAT
SRR511920.5304296.1-	GCTGGGTGGCAT
SRR511920.5304424.1-	GCTGGGTGGCAT
SRR511920.9569649.1-	GCTGGGTGGCAT
SRR516996.85062640.1-	GCTGGGTGGCAT
SRR516998.105584836.2-	GCTGGGTGGCAT
SRR517007.4303113.2+	GCTGGGTGGCAT
SRR511917.16171770.1+	CTGGGTGGCAT
SRR511917.72085621.2+	CTGGGTGGCAT
SRR516968.96419618.1-	CTGGGTGGCAT
SRR516983.39104642.2+	CTGGGTGGCAT
SRR516984.43825566.1-	CTGGGTGGCAT
SRR516984.80897040.1-	CTGGGTGGCAT
SRR516986.62178078.2+	CTGGGTGGCAT
SRR516993.2915184.1+	CTGGGTGGCAT
SRR516995.56989024.1+	CTGGGTGGCAT
SRR516998.45234491.1-	CTGGGTGGCAT
SRR517000.52358860.1+	CTGGGTGGCAT
SRR517003.70211691.2-	CTGGGTGGCAT
SRR511914.59836345.2+	TGGGTGGCAT
SRR511916.27790353.2+	TGGGTGGCAT
SRR511916.51501273.2+	TGGGTGGCAT
SRR516974.33828349.1-	TGGGTGGCAT
SRR516974.38378408.2-	TGGGTGGCAT
SRR516974.49320234.1-	TGGGTGGCAT
SRR516981.62187975.2-	TGGGTGGCAT
SRR516981.72937619.1-	TGGGTGGCAT
SRR516988.49719328.1-	TGGGTGGCAT
SRR516991.91013899.2+	TGGGTGGCAT
SRR517002.45348.1+	TGGGTGGCAT
SRR517003.75889442.2+	TGGGTGGCAT
SRR517005.58912099.2+	TGGGTGGCAT
SRR517005.61088972.2+	TGGGTGGCAT
SRR517008.44606589.1-	TGGGTGGCAT
SRR517008.68337962.1-	TGGGTGGCAT
SRR517008.69459447.1+	TGGGTGGCAT
SRR511916.34471369.1-	GGGTGGCAT
SRR511916.47771346.2-	GGGTGGCAT
SRR516969.23432456.1+	GGGTGGCAT
SRR516995.50404448.2-	GGGTGGCAT
SRR516998.74008326.1+	GGGTGGCAT
SRR516998.90024593.2-	GGGTGGCAT
SRR511915.30731584.2-	GGTGGCAT
SRR516983.29420342.1-	GGTGGCAT
SRR516997.42706554.2-	GGTGGCGT
SRR516999.103891959.2-	GGTGGCAT
SRR516999.9724330.1-	GGTGGCAT
SRR517002.98887947.1-	GGTGGCAT
SRR517005.99236075.2-	GGGGGCAA
SRR517007.626055.1-	GGTGGCAT
SRR511914.86222658.1-	GTGGCAT
SRR516968.128087344.2-	GTGGCAT
SRR516981.37172809.1-	GTGGCAT
SRR516990.33601261.1-	GTGGCAT
SRR516995.33739366.2-	GTGGCAT
SRR516998.65740325.1+	GTGGCAT
SRR517000.36416800.1+	GTGGCAT
SRR511913.35897653.2+	TGGCAT
SRR516968.39167129.1+	TGGCAT
SRR516978.15412507.1+	TGGCAT
SRR516992.15393409.1-	TGGCAT
SRR516994.39206055.2-	TGGCAT
SRR516997.97876806.2-	TGGCAT
SRR516999.108053227.2-	TGGCAT
SRR517002.24413856.1-	TGGCAT
SRR517003.41980170.1-	TGGCAT
SRR516967.3860522.1-	GGCAT
SRR516967.51922488.2-	GGCAT
SRR516977.80260583.2-	GGCAT
SRR516986.43082651.2-	GGCAT
SRR516993.35093586.1+	GGCAT
SRR516993.65644538.2+	GGCAT

```
SRR516999.52117285.1+                                                           GGCAT
SRR517001.53006899.2-                                                           GGCAT
SRR517009.40589595.1-                                                           GGCAT
SRR516967.3323960.2-                                                             GCAT
SRR516973.40895713.2-                                                            GCAT
SRR516977.12873538.2-                                                            GCAT
SRR516978.31035699.2+                                                            GCAT
SRR516995.53678391.1+                                                            GCAT
SRR517001.4174199.1+                                                             GCAT
SRR511916.10289327.1+                                                             CAT
SRR516979.52325428.1-                                                             CAT
SRR516981.79962474.1+                                                             CAT
SRR517003.61889052.2+                                                             CAT
SRR517009.47953341.2+                                                             CAT
SRR517009.59440565.2-                                                             CAT
SRR511911.59626526.2+                                                              AT
SRR516978.43155707.2+                                                              AT
SRR516989.74819632.1-                                                              AT
SRR516999.38071679.1-                                                              AT
SRR511911.51765582.2-                                                               T
SRR511915.43325895.2+                                                               T
SRR511916.25675370.2+                                                               T
SRR516967.114035844.2-                                                              T
SRR516974.40379406.1-                                                               T
SRR517005.8220247.2-                                                                T

consensus                ATGACGAACAGGAGGTGATGACATTTGAGCCGCAAGTCCATACAACTGGCTGGGTGGCAT

                             .    :    .    :    .    :    .    :    .    :    .    :
SRR516967.121468181.1-   T
SRR516976.50893891.2-    T
SRR516978.60869002.2+    T
SRR516984.50030865.2+    T
SRR516988.2929307.2-     T
SRR517000.23191480.1+    T
SRR517003.42514893.2-    T
SRR517006.79064283.2-    T
SRR517007.20187658.2-    T
SRR511912.20243841.1-    TT
SRR511916.20302767.1+    TT
SRR516968.40141403.1-    TT
SRR516973.4884301.2-     TT
SRR516978.1992911.1+     TT
SRR516988.32844820.2-    TT
SRR517001.31582093.1-    TT
SRR517002.29394887.2+    TT
SRR517009.28589393.1-    TT
SRR511913.72838419.1+    TTG
SRR511914.19779621.1-    TTG
SRR516968.106610860.1-   TTG
SRR516977.75947199.2+    TTG
SRR516989.46904169.2+    TTG
SRR516996.36234607.1+    TTG
SRR517003.73936435.2-    TTG
SRR517004.53693916.2-    TTG
SRR517008.37417547.1+    TTG
SRR511915.51271900.2+    TTGG
SRR516979.10675489.2+    TTGG
SRR516991.30740799.1+    TTGG
SRR516993.49833109.2-    TTGG
SRR517001.16403167.2-    TTGG
SRR517001.89049728.2+    TTGG
SRR517007.5128092.2-     TTGG
SRR516967.115389681.2+   TTGGA
SRR516994.76691379.2-    TTGGA
SRR517002.99266758.2+    TTGGA
SRR517003.63008308.1-    TTGGA
SRR517005.54428488.2+    TTGGA
SRR516967.148303398.2-   TTGGAT
SRR516971.15050413.1-    TTGGAT
SRR516978.43755925.2-    TTGGAT
SRR517005.97095694.1-    TTGGAT
SRR517006.43208825.1-    TTGGAT
SRR517006.8352318.1-     TTGGAT
SRR511915.56868915.2-    TTGGATT
SRR516974.66440091.2-    TTGGATT
SRR516975.15347664.1-    TTGGATT
SRR516975.39714728.2+    TTGGATT
SRR516976.2015671.2+     TTGGATT
SRR516980.19111556.1-    TTGGATT
SRR516980.32208306.1-    TTGGATT
SRR516985.77238548.2-    TTGGATT
SRR517000.94486378.2-    TTGGATT
SRR517003.87371479.1+    TTGGATT
```

SRR511911.38218078.2-	TTGGATTC
SRR516967.94416127.1-	TTGGATTC
SRR516974.29282850.1+	TTGGATTC
SRR516977.10878176.2-	TTGGATTC
SRR516986.10287761.2+	TTGGATTC
SRR516991.49815040.2+	TTGGATTC
SRR517003.70967985.2+	TTGGATTC
SRR517004.40113226.2+	TTGGATTC
SRR511916.62302427.1+	TTGGATTCA
SRR511916.67819900.1+	TTGGATTCA
SRR516977.12820030.2+	TTGGATTCA
SRR516989.8534348.1-	TTGGATTCA
SRR516991.17318347.2-	TTGGATTCA
SRR516991.1931100.1-	TTGGATTCA
SRR516991.24878492.1-	TTGGATTCA
SRR516995.74299668.1+	TTGGATTCA
SRR517000.37586981.1-	TTGGATTCA
SRR517001.36464132.1-	TTGGATTCA
SRR517005.886243.1-	TTGGATTCA
SRR516968.43850003.2-	TTGGATTCAG
SRR516975.22417896.2+	TTGGATTCAG
SRR516976.36876145.1+	TTGGATTCAG
SRR516979.6140788.2+	TTGGATTCCG
SRR516989.14777251.1+	TTGGATTCAG
SRR516994.78306274.2+	TTGGATTCAG
SRR516997.5744761.2-	TTGGATTCAG
SRR517002.68785810.2+	TTGGATTCAG
SRR516967.100239201.1+	TTGGATTCAGC
SRR516968.54367312.2+	TTGGATTCAGC
SRR516992.79918999.2+	TTGGATTCAGC
SRR516996.52900642.2-	TTGGATTCAGC
SRR511915.717767.2+	TTGGATTCAGCC
SRR511916.29283073.1+	TTGGATTCAGCC
SRR516998.57969943.1-	TTGGATTCAGCC
SRR516999.61456648.1+	TTGGATTCAGCC
SRR517001.8564550.1+	TTGGATTCAGCC
SRR511917.48425344.1-	TTGGATTCAGCCC
SRR516967.135591639.1+	TTGGATTCAGCCC
SRR516967.57741754.1+	TTGGATTCAGCCC
SRR516992.10103608.1+	TTGGATTCAGCCC
SRR516993.1447601.1+	TTGGATTCAGCCC
SRR516994.82514123.2+	TTGGATTCAGCCC
SRR516999.7152611.2+	TTGGATTCAGCCC
SRR517002.8665886.2-	TTGGATTCAGCCC
SRR517005.66404474.1+	TTGGATTTAGCCC
SRR516968.108661732.1-	TTGGATTCAGCCCT
SRR516973.16904597.1-	TTGGATTCAGCCCT
SRR516973.16906873.1-	TTGGATTCAGCCCT
SRR516976.57348554.2-	TTGGATTCAGCCCT
SRR516980.63928031.1-	TTGGATTCAGCCCT
SRR517004.34678058.2-	TTGGATTCAGCCCT
SRR517008.53563245.1-	TTGGATTCAGCCCT
SRR516990.68475801.1+	TTGGATTCAGCCCTC
SRR516994.35326807.2-	TTGGATTCAGCCCTC
SRR516999.61850545.2+	TTGGATTCAGCCCCC
SRR511912.83293905.1+	TTGGATTCAGCCCTCA
SRR516985.70259889.1-	TTGGATTCAGCCCTCA
SRR516987.36431135.1-	TTGGATTCAGCCCTCA
SRR516987.44128152.2+	TTGGATTTAGCCCTCA
SRR516989.21621378.1+	TTGGATTCAGCCCTCA
SRR516991.7741780.1-	TTGGATTCAGCCCTCA
SRR516995.15254844.2-	TTGGATTCAGCCCTCA
SRR511911.60280636.2+	TTGGATTCAGCCCTCCT
SRR511913.22705507.2-	TTGGATTCAGCCCTCAT
SRR516967.138148601.1-	TTGGATTCAGCCCTCAT
SRR516968.164894710.1+	TTGGATTCAGCCCTCAT
SRR516980.37499938.1+	TTGGATTCAGCCCTCCT
SRR516983.43684823.1-	TTGGATTCAGCCCTCAT
SRR516983.94153626.2+	TTGGATTCAGCCCTCAT
SRR516995.43808656.1-	TTGGATTCAGCCCTCAT
SRR516997.25946564.1-	TTGGATTCAGCCCTCAT
SRR516997.44735578.1-	TTGGATTCAGCCCTCAT
SRR517000.49710061.1-	TTGGATTCAGCCCTCAT
SRR517003.70018023.2-	TTGGATTCAGCCCTCAT
SRR511914.41285120.1-	TTGGATTCAGCCCTCATG
SRR511914.56122848.1+	TTGGATTCAGCCCCCCTG
SRR511915.73501967.1-	TTGGATTCAGCCCTCATG
SRR516968.8958322.1+	TTGGATTCAGCCCTCATG
SRR516975.59637801.2-	TTGGATTCAGCCCTCATG
SRR516977.57408704.1+	TTGGATTCAGCCCTCATG
SRR516980.12955843.2-	TTGGATTCAGCCCTCATG
SRR516983.60674262.1-	TTGGATTCAGCCCTCATG
SRR516989.75746913.1-	TTGGATTCAGCCCTCATG
SRR516992.63008789.1-	TTGGATTCAGCCCTCATG

SRR516997.93541116.1-   TTGGATTCAGCCCTCATG
SRR517007.55411417.1+   TTGGATTCAGCCCTCATG
SRR517009.31830082.2-   TTGGATTCAGCCCTCATG
SRR517009.74653167.2-   TTGGATTCAGCCCTCATG
SRR516978.37638949.1-   TTGGATTCAGCCCTCATGG
SRR516979.1430425.1-   TTGGATTCAGCCCTCATGG
SRR516988.31050597.2+   TTGGATTCAGCCCTCATGG
SRR516994.1794759.2+   TTGGATTCAGCCCTCATGG
SRR517006.62045893.2-   TTGGATTCAGCCCTCATGG
SRR517007.44465454.2-   TTGGATTCAGCCCTCATGG
SRR511911.7460180.1-   TTGGATTCAGCCCTCATGGA
SRR511912.59893234.1+   TTGGGTTCCGCCCCCATGGA
SRR516976.29296347.1-   TTGGATTCAGCCCTCATGGA
SRR516977.13189923.1+   TTGGATTCAGCCCTCATGGA
SRR516984.56347101.1-   TTGGATTCAGCCCTCATGGA
SRR517009.38612123.1+   TTGGATTCAGCCCTCATGGA
SRR511912.63185956.1+   TTGGATTCAGCCCTCATGGAG
SRR516967.34973661.1+   TTGGATTCAGCCCTCATGGAG
SRR516973.61866208.2+   TTGGATTCAGCCCTCCTGGAG
SRR516976.10068918.1-   TTGGATTCAGCCCTCATGGAG
SRR516976.8946365.1-   TTGGATTCAGCCCTCATGGAT
SRR516992.37277386.2-   TTGGATTCAGCCCTCATGGAG
SRR516992.78662263.1-   TTGGATTCAGCCATCATGGAG
SRR516996.85884676.2-   TTGGATTCAGCCCTCATGGAG
SRR516997.66381769.1-   TTGGATTCAGCCCTCATGGAG
SRR517000.103614309.2+   TTGGATTCAGGCCTCACGGAG
SRR517000.39251131.1+   TTGGATTCAGCCCTCATGGAG
SRR517004.58862282.1+   TTGGATTCAGCCCTCATGGAG
SRR511911.51765582.1+   TTGGATTCAGCCCTCAAGGAGA
SRR516973.16005217.1-   TTGGATTCAGCCCTCATGGAGA
SRR516983.41490614.2-   TTGGATTCAGCCCTCATGGAGA
SRR516983.54306814.2-   TTGGATTCAGCCCTCATGGAGA
SRR516992.23178703.2-   TTGGATTCAGCCCTCATGGAGA
SRR516993.12312704.1-   TTGGATTCAGCCCTCATGGAGA
SRR516993.73082870.1-   TTGGATTCAGCCCTCATGGAGA
SRR516996.88424796.2-   TTTGAGTCAGCCCTCATGGAGA
SRR517000.81566989.1+   TTGGATTCAGCCCTCATGGAGA
SRR511892.6007403.1-   TTGGATTCAGCCCTCATGGAGAG
SRR516967.55179281.2+   TTGGATTCAGCCCTCATGGAGAG
SRR516967.63778390.2-   TTGGATTCNGCCCTCATGGAGAG
SRR516972.49653018.2-   TTGGATTCAGCCCTCATGGAGAG
SRR516993.83887266.1+   TTGGATTCAGGCCTCATGGAGAG
SRR516999.81087074.2+   TTGGATTCAGCCCTCATGGAGAG
SRR516968.75435688.1-   TTGGATTCAGCCCTCATGGAGAGT
SRR516996.1617304.1+   TTGGATTCAGCCCTCATGGAGAGT
SRR516998.43983662.1-   TTGGATTCAGCCCTCATGGAGAGT
SRR517001.43347006.1-   TTGGATTCAGCCCTCATGGAGAGT
SRR511912.63185812.1+   TTGGATTCAGCCCTCATGGAGAGTT
SRR516990.32569854.2-   TTGGATTCAGCCCTCATGGAGAGTT
SRR516990.55302390.2-   TTGGATTCAGCCCTCATGGAGAGTT
SRR516993.75065526.2+   TTTGATTCAGCCCTCATGGAGAGTT
SRR516996.29500035.2-   TTGGATTCAGCCCTCATGGAGAGTT
SRR516999.96819542.2-   TTGGATTCAGCCCTCATGGAGAGTT
SRR516989.36153179.1+   TTGGATTCAGCCCTCATGGAGAGTTG
SRR516999.48060851.1-   TTGGATTCAGCCCTCATGGAGAGTTG
SRR517000.9053207.1-   TTGGATTCAGCCCTCATGGAGAGTTG
SRR517001.26562166.2-   TTGGATTCAGCCCTCATGGAGAGTTG
SRR517002.51438896.1-   TTGGATTCAGCCCTCATGGAGAGTTG
SRR516990.20970250.1+   TTGGATTCAGCCCTCATGGAGAGTTGC
SRR511915.12934870.2-   TTGGATTCAGCCCTCATGGAGAGTTGCC
SRR516968.73747636.1-   TTGGATTCAGCCCTCATGGAGAGTTGCC
SRR516974.21816576.2-   TTGGATTCAGCCCTCATGGAGAGTTGCC
SRR517004.45940969.1+   TTGGATTCAGCCCTCATGGAGAGTTGCC
SRR511892.63390781.2-   TTGGATTCAGCCCTCATGGAGAGTTGCCT
SRR511892.64175574.2-   TTGGATTCAGCCCTCATGGAGAGTTGCCT
SRR516967.59477310.1-   TTGGATTCAGCCCTCATGGAGAGTTGCCT
SRR516977.49992915.2-   TTGGATTCAGCCCTCATGGAGAGTTGCCT
SRR516978.65078285.1-   TTGGATTCAGCCCTCATGGAGAGTTGCCT
SRR517009.51801045.2+   TTGGATTCAGCCCTCATGGAGAGTTGCCT
SRR517009.70794059.2+   TTGGATTCAGCCCTCATGGAGAGTTGCCT
SRR517009.89416108.1+   TTGGATTCAGCCCTCATGGAGAGTTGCCT
SRR516980.30549936.1-   TTGGATTCAGCCCTCATGGAGAGTTGCCTG
SRR516981.70866488.1-   TTGGATTCAGCCCTCATGGAGAGTTGCCTG
SRR516984.6819096.2-   TTGGATTCAGCCCTCATGGAGAGTTGCCTG
SRR516990.21735027.2-   TTGGATTCAGCCCTCATGGAGAGTTGCCTG
SRR516995.15167538.1+   TTGGATTCAGCCCTCATGGAGAGTTGCCTG
SRR516999.15040710.1-   TTGGATTCAGCCCTCATGGAGAGTTGCCTG
SRR517002.62107602.1-   TTGGATTCAGCCCTCATGGAGAGTTGCCTG
SRR517002.80851214.2-   TTGGATTCAGCCCTCATGGAGAGTTGCCTG
SRR517005.17700561.1-   TTGGATTCAGCCCTCATGGAGAGTTGCCTG
SRR517009.2225310.1-   TTGGATTCAGCCCTCATGGAGAGTTGCCTG
SRR511911.36873194.1-   TTGGATTCAGCCCTCATGGAGAGTTGCCTGG
SRR511914.80423043.1-   TTGGATTCAGCCCTCATGGAGAGTTGCCTGG
SRR516967.132651451.1-   TTGGATTCAGCCCTCATGGAGAGTTGCCTGG

SRR516967.94039320.1-   TTGGATTCAGCCCTCATGGAGAGTTGCCTGG
SRR516968.137574947.2+   TTGGATTCAGCCCTCATGGAGAGTTGCCTGG
SRR516981.62232392.2+   TTGGATTCAGCCCTCATGGAGAGTTGACTGG
SRR516988.35895455.2+   TTGGATTCAGCCCTCATGGAGAGTTGCCTGG
SRR516999.67197738.1-   TTGGATTCAGCCCTCATGGAGAGTTGCCTGG
SRR511892.9511611.2+   TTGGATTCAGCCCTCATGGCGAGTTGGCCGGA
SRR516968.58859238.1+   TTGGATTCAGCCCTCATGGAGCGTTGCCTGGA
SRR516994.53221266.2+   TTGGATTCAGCCCTCATGGAGAGTTGCCTGGA
SRR516998.31606571.1+   TTGGATTTAGCCCTCATGGAGAGTTGCCTGGA
SRR517003.51023813.2-   TTGGATTCAGCCCTCATGGAGAGTTGCCTGGA
SRR517005.8503673.2+   TTTGATTCAGCCCTCCTGGTGAGTTGCCTTGA
SRR511911.21547512.2-   TTGGATTCAGCCCTCATGGAGAGTTGCCTGGAT
SRR511913.18165035.2-   TTGGATTCAGCCCTCATGGAGAGTTGCCTGGAT
SRR511913.52308827.1-   TTGGATTCAGCCCTCATGGAGAGTTGCCTGGAT
SRR516978.52566920.2-   TTGGATTCAGCCCTCATGGAGAGTTGCCTGGAT
SRR516979.65960194.2-   TTGGATTCAGCCCTCATGGAGAGTTGCCTGGAT
SRR516992.40366624.2-   TTGGATTCAGCCCTCATGGAGAGTTGCCTGGAT
SRR516992.67474202.2+   TTGGATTCAGCCCTCATGGAGAGTTGCCTGGAT
SRR516992.79411570.1-   TTGGATTCAGCCCTCATGGAGAGTTGCCTGGAT
SRR516994.6814432.2+   TTGGATTCAGCCCTCATGGAGAGTTGCCTGGAT
SRR516998.45967480.1-   TTGGATTCAGCCCTCATGGAGAGTTGCCTGGAT
SRR517001.81299431.1+   TTGGATTCAGCCCTCATGGAGAGTTGCCTGGAT
SRR511911.19148709.2+   TTGGATTCAGCCCTCATGGAGAGGTGCCTGGATC
SRR511912.70385865.1-   TTGGATTCAGCCCTCATGGAGAGTTGCCTGGATC
SRR516968.156018396.1-   TTGGATTCAGCCCTCATGGAGAGTTGCCTGGATC
SRR516981.21374378.2-   TTGGATTCAGCCCTCATGGAGAGTTGCCTGGATC
SRR516993.5077793.2+   TTGGATTCAGCCCTCATGGAGAGTTGCCTGGATC
SRR516996.75662898.1-   TTGGATTCAGCCCTCATGGAGAGTTGCCTGGATC
SRR517004.41051761.2+   TTGGATTCAGCCCTCATGGAGAGTTGCCTGGATC
SRR517005.46088960.1-   TTGGATTCAGCCCTCATGGAGAGTTGCCTGGATC
SRR517009.48171529.2-   TTGGATTCAGCCCTCATGGAGAGTTGCCTGGATC
SRR511892.23600637.2+   TTGGATTCAGCCCTCATGGAGAGTTGCCCGGATCT
SRR511911.3389924.2+   TTGGATTCAGCCCTCATGGAGAGTTGCCTGGATCT
SRR511920.37463379.1-   TTGGATTCAGCCCTCATGGAGAGTTGCCTGGATCT
SRR516984.53836077.1-   TTGGATTCAGCCCTCATGGAGAGTTGCCTGGATCT
SRR516988.7333166.1-   TTGGATTCAGCCCTCATGGAGAGTTGCCTGGATCT
SRR516994.20458023.1+   TTGGATTCAGCCCTCATGGAGAGTTGCCTGGATCT
SRR516996.49779749.1+   TTGGATTCAGCCCTCATGGAGAGTTGCCTGGATCT
SRR517008.68021994.2-   TTGGATTCAGCCCTCATGGAGAGTTGCCTGGATCT
SRR511913.70154375.2-   TTGGATTCAGCCCTCATGGAGAGTTGCCTGGATCTG
SRR516967.96216344.1+   TTGGATTCAGCCCTCATGGAGAGTTGCCTGGATCTG
SRR516978.37914558.1+   TTGGATTCAGCCCTCATGGAGAGTTGCCTGGATCTG
SRR516981.46321294.2+   TTGGATTCAGCCCTCATGGAGAGTTGCCTGGATCTG
SRR516989.64914875.2-   TTGGATTCAGCCCTCATGGAGAGTTGCCTGGATCTG
SRR516996.32218127.1-   TTGGATTCAGCCCTCATGGAGAGTTGCCTGGATCTG
SRR516998.88636739.2-   TTGGATTCAGCCCTCATGGAGAGTTGCCTGGATCTG
SRR517006.17938695.2+   TTGGATTCAGCCCTCATGGAGAGTTGCCTGGATCTG
SRR511914.24386047.1+   TTGGATTCAGCCCTTATGGAGAGTTGCCCGGATCTGA
SRR511920.29932217.1-   TTGGATTCAGCCCTCATGGAGAGTTGCCTGGATCTGA
SRR511920.31903781.1-   TTGGATTCAGCCCTCATGGAGAGTTGCCTGGATCTGA
SRR511920.34826249.1-   TTGGATTCAGCCCTCATGGAGAGTTGCCTGGATCTGA
SRR511920.43179619.1-   TTGGATTCAGCCCTCATGGAGAGTTGCCTGGATCTGA
SRR511920.45487406.1-   TTGGATTCAGCCCTCATGGAGAGTTGCCTGGATCTGA
SRR511920.5304296.1-   TTGGATTCAGCCCTCATGGAGAGTTGCCTGGATCTGA
SRR511920.5304424.1-   TTGGATTCAGCCCTCATGGAGAGTTGCCTGGATCTGA
SRR511920.9569649.1-   TTGGATTCAGCCCTCATGGAGAGTTGCCTGGATCTGA
SRR516978.14954997.2+   TTGGATTCAGCCCTCATGGAGAGTTGCCTGGATCTGA
SRR516979.9712358.1-   TTGGATTCAGCCCTCATGGAGAGTTGCCTGGATCTGA
SRR516986.23230956.2+   TTGGATTCAGCCCTCCTGGAGAGTTGCCTGGATCTGA
SRR516987.33930642.2+   TTGGATTCAGCCCTCATGGAGAGTTGCCTGGATCTGA
SRR516988.52138870.2+   TTGGATTCAGCCCTCATGGAGAGTTGCCTGGATCTGA
SRR516994.75814175.2-   TTGGATTCAGCCCTCATGGAGAGTTGCCTGGATCTGA
SRR516998.62923474.1+   TTGGATTCAGCCCTCATGGAGAGGTGCCTGGATCTGA
SRR517001.80318310.2-   TTGGATTCAGCCCTCATGGAGAGTTGCCTGGATCTGA
SRR511917.16171770.1+   TTGGATTCAGCCCTCATGGAGAGTTGCCTGGATCTGAC
SRR511917.72085621.2+   TTGGATTCAGCCCTCATGGAGAGTTGCCTGGATCTGAC
SRR516972.34696103.2+   TTGGATTCAGCCCTCATGGAGAGTTGCCTGGATCTGAC
SRR516975.38025761.2-   TTGGATTCAGCCCTCATGGAGAGTTGCCTGGATCTGAC
SRR516977.40786703.2-   TTGGATTCAGCCCTCATGGAGAGTTGCCTGGATCTGAC
SRR516977.56138604.2-   TTGGATTCAGCCCTCATGGAGAGTTGCCTGGATCTGAC
SRR516988.23560143.1+   TTGGATTCAGCCCTCATGGAGAGTTGCCTGGATCTGAC
SRR517003.83174067.1+   TTGGATTCAGCCCTCATGGAGAGTTGCCTGGATCTGAC
SRR517004.25798813.1+   TTGGATTCAGCCCTCATGGAGAGTTGCCTGGATCTGAC
SRR511916.27790353.2+   TTGGATTCAGCCCTCATGGAGAGTTGCCTGGATCTGACA
SRR511916.51501273.2+   TTGGATTCAGCCCTCATGGAGAGTTGCCTGGATCTGACA
SRR516972.32806802.1-   TTGGATTCAGCCCTCATGGAGAGTTGCCTGGATCTGACA
SRR516975.13713707.1-   TTGGATTCAGCCCTCATGGAGAGTTGCCTGGATCTGACA
SRR516975.31280279.1-   TTGGATTCAGCCCTCATGGAGAGTTGCCTGGATCTGACA
SRR517000.18261147.1+   TTGGATTCAGCCCTCATGGAGAGTTGCCTGGATCTGACA
SRR517004.4165455.2+   TTGGATTCAGCCCTCATGGAGAGTTGCCTGGATCTGACA
SRR517007.15671468.2+   TTGGATTCAGCCCTCATGGAGAGTTGCCTGGATCTGACA
SRR517008.29824779.2-   TTGGATTCAGCCCTCATGGAGAGTTGCCTGGATCTGACA
SRR511892.45271449.2+   TTGGATTCAGCCCTCATGGAGAGTTGCCTGGATCTGACAT
SRR511911.58123630.1+   TTCGATTCAGCCCTCATGGAGAGGTGCCTGGATCTGACAT

SRR511916.34471369.1-   TTGGATTCAGCCCTCATGGAGAGTTGCCTGGATCTGACAT
SRR511916.47771346.2-   TTGGATTCAGCCCTCATGGAGAGTTGCCTGGATCTGACAT
SRR516969.1006309.2-   TTGGATTCAGCCCTCATGGAGAGTTGCCTGGATCTGACAT
SRR516978.384904.2-   TTGGATTCAGCCCTCATGGAGAGTTGCCTGGATCTGACAT
SRR516978.56562036.1+   TTGGATTCAGCCCTCATGGAGAGTTGCCTGGATCTGACAT
SRR516993.8072866.1-   TTGGATTCAGCCCTCATGGAGAGTTGCCTGGATCTGACAT
SRR517009.71698028.2-   TTGGATTCAGCCCTCATGGAGAGTTGCCTGGATCTGACAT
SRR511912.13524230.1+   TTGGATTCAGCCCTCATGGAGAGTTGCCTGGATCTGACCTT
SRR516967.81487147.2+   TTGGATTCAGCCCTCATGGAGAGTTGCCTGGATCTGACATT
SRR516970.29220836.2-   TTGGATTCAGCCCTCATGGAGAGTTGCCTGGATCTGACATT
SRR516970.30280066.2-   TTGGATTCAGCCCTCATGGAGAGTTGCCTGGATCTGACATT
SRR516984.6644703.2-   TTGGATTCAGCCCTCATGGAGAGTTGCCTGGATCTGACATT
SRR516988.41673661.2-   TTGGATTCAGCCCTCATGGAGAGTTGCCTGGATCTGACATT
SRR516993.23735352.1-   TTGGATTCAGCCCTCATGGAGAGTTGCCTGGATCTGACATT
SRR516998.74208434.1-   TTGGATTCAGCCCTCATGGAGAGTTGCCTGGATCTGACATT
SRR517000.56225435.1-   TTGGATTCAGCCCTCATGGAGAGTTGCCTGGATCTGACATT
SRR517007.43638454.1-   TTGGATTCAGCCCTCATGGAGAGTTGCCTGGATCTGACATT
SRR517009.35713352.2-   TTGGATTCAGCCCTCATGGAGAGTTGCCTGGATCTGACATT
SRR517009.87547697.2-   TTGGATTCAGCCCTCATGTAGAGTTGCCTGGATCTAACATT
SRR516980.72482160.1-   TTGGATTCAGCCCTCATGGAGAGTTGCCTGGATCTGACATTG
SRR517000.54956038.1-   TTGGATTCAGCCCTCATGGAGAGTTGCCTGGATCTGACATTG
SRR511915.64989562.1-   TTGGATTCAGCCCTCATGGAGAGTTGCCTGGATCTGACATTGT
SRR511915.64989699.1-   TTGGATTCAGCCCTCATGGAGAGTTGCCTGGATCTGACATTGT
SRR516972.35896448.1-   TTGGATTCAGCCCTCATGGAGAGTTGCCTGGATCTGACATTGT
SRR516972.36494147.2+   TTGGATTCAGCCCTCCTGGAGAGTTGCCTGGATCTGACATTGT
SRR516980.58810552.1-   TTGGATTCAGCCCTCATGGAGAGTTGCCTGGATCTGACATTGT
SRR516992.78415230.2-   TTGGATTCAGCCCTCATGGAGAGTTGCCTGGATCTGACATTGT
SRR516997.48515103.2-   TTGGATTCAGCCCTCATGGAGAGTTGCCTGGATCTGACATTGT
SRR511892.9368196.1+   TTGGATTCAGCCCTCATGGAGAGTTGCCTGGATCTGACATTGTG
SRR511911.54079531.2+   TTGGATTCAGCCCTCATGGAGGGGTGCCTGGATCTGACATTGTG
SRR516967.5677042.1-   TTGGATTCAGCCCTCATGGAGAGTTGCCTGGATCTGACATTGTG
SRR516970.7484751.2+   TTGGATTCAGCCCTCATGGAGCGTTGCCTGGATCTGCCATTGTG
SRR516981.20078478.1-   TTGGATTCAGCCCTCATGGAGAGTTGCCTGGATCTGACATTGTG
SRR516998.86192854.2+   TTGGATTCAGCCCTCATGGAGAGTTGCCTGGATCTGACATTGTG
SRR516999.49626087.2-   TTGGATTCAGCCCTCATGGAGAGTTGCCTGGATCTGACATTGTG
SRR516972.47863115.2-   TTGGATTCAGCCCTCATGGAGAGTTGCCTGGATCTGACATTGTGA
SRR516979.58015851.1+   TTGGATTCAGCCCTCATGGAGAGTTGCCTGGATCTGACATTGTGA
SRR516996.92350992.2-   TTGGATTCAGCCCTCATGGAGAGTTGCCTGGATCTGACATTGTGA
SRR516997.86440148.2+   TTGGATTCAGCCCTCATGGAGAGTTGCCTGGATCTGACATTGTGA
SRR517008.51758338.2+   TTGGATTCAGCCCTCATGGAGAGTTGCCTGGATCTGACATTGTGA
SRR517008.6483545.2-   TTGGATTCAGCCCTCATGGAGAGTTGCCTGGATCTGACATTGTGA
SRR517009.11972102.1+   TTGGATTCAGCCCTCATGGAGAGTTGCCTGGATCTGACATTGTGA
SRR511916.10289327.1+   TTGGATTCAGCCCTCATGGAGAGTTGCCTGGATCTGACATTGTGAT
SRR516969.9561270.1-   TTGGATTCAGCCCTCATGGAGAGTTGCCTGGATCTGACATTGTGAT
SRR516978.72874464.2-   TTGGATTCAGCCCTCATGGAGAGTTGCCTGGATCTGACATTGTGAT
SRR516995.23487131.1-   TTGGATTCAGCCCTCATGGAGAGTTGCCTGGATCTGACATTGTGAT
SRR516997.34496168.2-   TTGGATTCAGCCCTCATGGAGAGTTGCCTGGATCTGACATTGTGAT
SRR516997.92391410.2-   TTGGATTCAGCCCTCATGGAGAGTTGCCTGGATCTGACATTGTGAT
SRR511911.1070600.2+   TTGGATTCAGCCCTCATGGAGAGTTGCCTGGATCTGACCTTGTGATA
SRR511914.6072833.2-   TTGGATTCAGCCCTCATGGAGAGTTGCCTGGACCTGACATTGTGATA
SRR511915.27615657.2+   TTGGATTCAGCCCTCATGGAGAGTTGCCTGGATCTGACATTGTGATA
SRR516968.123158278.1+   TTGGATTCAGCCCTCATGGAGAGTTGCCTGGATCTGACATTGTGATA
SRR516998.87299695.1-   TTGGATTCAGCCCTCATGGAGAGTTGCCTGGATCTGACATTGTGATA
SRR517000.7065464.1+   TTGGATTCAGCCCTCATGGAGAGTTGCCCGGATCTGACATTGTGATA
SRR517009.108104.1+   TTGGATTCAGCCCTCATGGAGAGTTGCCTGGATCTGACATTGTGATA
SRR511913.40896479.2+   TTGGATTCAGCCCTCATGGAGAGTTGCCTGGATCTGACATTGTGATAG
SRR511916.25675370.2+   TTGGATTCAGCCCTCATGGAGAGTTGCCTGGATCTGACATTGTGATAG
SRR516991.44574167.1+   TTGGATTCAGCCCTCATGGAGAGTTGCCTGGATCTGACATTGTGATAG
SRR516994.41842983.2-   TTGGATTCAGCCCTCATGGAGAGTTGCCTGGATCTGACATTGTGATAG
SRR517001.32246971.1-   TTGGATTCAGCCCTCATGGAGAGTTGCCTGGATCTGACATTGTGATAG
SRR517008.32872042.2-   TTGGATTCAGCCCTCATGGAGAGTTGCCTGGATCTGACATTGTGATAG
SRR517009.48718939.2-   TTGGATTCAGCCCTCATGGAGAGTTGCCTGGATCTGACATTGTGATAG
SRR517009.54064618.2-   TTGGATTCAGCCCTCATGGAGAGTTGCCTGGATCTGACATTGTGATAG
SRR511914.11127656.1-   TTGGATTCAGCCCTCATGGAGAGTTGCCTGGATCTGACATTGTGATAGG
SRR511914.34655921.2-   TTGGATTCAGCCCTCATGGAGAGTTGCCTGGATCTGACATTGTGATAGG
SRR511914.59836345.2+   TTGGATTCAGCCCTCAGGGGGAGTTGCCTGGATCTGACATTGGGATAGG
SRR516981.35980793.1+   TTGGATTCAGCCCTCATGGAGAGTTGCCTGGATCTGACATTGTGATAGG
SRR516984.19133805.1-   TTGGATTCAGCCCTCATGGAGAGTTGCCTGGATCTGACATTGTGATAGG
SRR516991.47909941.1+   TTGGATTCAGCCCTCATGGAGAGTTGCCTGGATCTGACATTGTGATAGG
SRR516993.16481734.2-   TTGGATTCAGCCCTCATGGAGAGTTGCCTGGATCTGACATTGTGATAGG
SRR516994.49842132.2-   TTGGATTCAGCCCTCATGGAGAGTTGCCTGGATCTGACATTGTGATAGG
SRR516996.28202039.2-   TTGGATTCAGCCCTCATGGAGAGTTGCCTGGATCTGACATTGTGATAGG
SRR516996.72151079.1+   TTGGATTCAGCCCTCATGGAGAGTTGCCTGGATCTGACATTGTGATAGG
SRR517000.7876007.1-   TTGGATTCAGCCCTCATGGAGAGTTGCCTGGATCTGATATTGTGATAGG
SRR517000.81693984.2-   TTGGATTCAGCCCTCATGGAGAGTTGCCTGGATCTGACATTGTGATAGG
SRR517003.58518606.1+   TTGGATTCAGCCCTCATGGAGAGTTGCCTGGATCTGACATTGTGATAGG
SRR517007.19456209.1-   TTGGATTCAGCCCTCATGGAGAGTTGCCTGGATCTGACATTGTGATAGG
SRR516978.26253784.2-   TTGGATTCAGCCCTCATGGAGAGTTGCCTGGATCTGACATTGTGATAGGA
SRR516978.39130629.2+   TTGGATTCAGCCCTCATGGAGAGTTGCCTGGATCTGACATTGTGATAGGA
SRR516981.13235658.1-   TTGGATTCAGCCCTCATGGAGAGTTGCCTGGATCTGACATTGTGATAGGA
SRR516995.47487143.1+   TTGGATTCAGCCCTCATGGAGAGTTGCCTGGATCTGACATTGTGATAGGG
SRR517004.20569530.2-   TTGGATTCAGCCCTCATGGAGAGTTGCCTGGATCTGACATTGTGATAGGA
SRR517001.24362772.2+   TTGGATTCAGCCCTCATGGAGAGTTGCCTGGATCTGACATTGTGATAGGAG
SRR517003.41478862.1+   TTGGATTCAGCCCTCATGGAGAGTTGCCTGGATCTGACATTGTGATAGGAA

SRR511892.42564732.2-   TTGGATTCAGCCCTCATGGAGAGTTGCCTGGATCTGACATTGTGATAGGAAG
SRR511892.56496978.1-   TTGGATTCAGCCCTCATGGAGAGTTGCCTGGATCTGACATTGTGATAGGAAG
SRR511915.30654048.2+   TTGGATTCAGCCCTCATGGAGAGTTGCCTGGATCTGACATTGTGATAGGAAG
SRR516975.66584327.2-   TTGGATTCAGCCCTCATGGAGAGTTGCCTGGATCTGACATTGTGATAGGAAG
SRR516986.29707316.1+   TTGGATTCAGCCCTCATGGAGAGTTGCCTGGATCTGACATTGTGATAGGAGG
SRR516996.68386100.2+   TTGGATTCAGCCCTCATGGAGAGTTGCCTGGATCTGACATTGTGATAGGAAG
SRR516996.755157.2-   TTGGATTCAGCCCTCATGGAGAGTTGCCTGGATCTGACATTGTGATAGGAAG
SRR516998.784538.2-   TTGGATTCAGCCCTCATGGAGAGTTGCCTGGATCTGACATTGTGATAGGAAG
SRR517001.70847586.2-   TTGGATTCAGCCCTCATGGAGAGTTGCCTGGATCTGACATTGTGATAGGAGG
SRR511892.53446249.1-   TTGGATTCAGCCCTCATGGAGAGTTGCCTGGATCTGACATTGTGATAGGAAGT
SRR516972.46208961.2-   TTGGATTCAGCCCTCATGGAGAGTTGCCTGGATCTGACATTGTGATAGGAAGT
SRR516974.27143600.1+   TTGGATTCAGCCCTCATGGAGAGTTGCCTGGATCTGACATTGTGATAGGAAGT
SRR516980.15802379.2-   TTGGATTCAGCACTCATGGAGAGTTGCCTGGATCTGACATTGTGATAGGAGGT
SRR516983.52981551.1+   TTGGATTCAGCCCTCATGGAGAGTTGCCTGGATCTGACATTGTGATAGGAAGG
SRR516997.7493563.2-   TTGGATTCAGCCCTCATGGAGAGTTGCCTGGATCTGACATTGTGATAGGAAGT
SRR511892.22200889.2-   TTGGATTCAGCCCTCATGGAGAGTTGCCTGGATCTGACATTGTGATAGGAAGTA
SRR511915.74307710.2-   TTGGATTCAGCCCTCATGGAGAGTTGCCTGGATCTGACATTGTGATAGGAAGTA
SRR516974.54645088.1-   TTGGATTCAGCCCTCATGGAGAGTTGCCTGGATCTGACATTGTGATAGGAAGTA
SRR516987.22815449.2-   TTGGATTCAGCCCTCATGGAGAGTTGCCTGGATCTGACATTGTGATAGGAAGTA
SRR517005.91348161.2-   TTGGATTCAGCCCTCATGGAGAGTTGCCTGGATCTGACATTGTGATAGGAAGTA
SRR516974.11239183.1+   TTGGATTCAGCCCTCATGGAGAGTTGCCTGGATCTGACATTGTGATAGGAAGGGT
SRR516974.60730690.1-   TTGGATTCAGCCCTCATGGAGAGTTGCCTGGATCTGACATTGTGATAGGAAGTGT
SRR516983.52187946.1-   TTGGATTCAGCCCTCATGGAGAGTTGCCTGGATCTGACATTGTGATAGGAAGTGT
SRR516994.13378760.2+   TTGGATTCAGCCCTCATGGAGAGTTGCCTGGATCTGACATTGTGATAGGAGGTGT
SRR516995.89724041.1+   TTGGATTCAGCCCTCATGGAGAGTTGCCTGGATCTGACATTGTGATAGGAAGTGT
SRR517000.35171247.1-   TTGGATTCAGCCCTCATGGAGAGTTGCCTGGATCTGACATTGTGATAGGAGGTGT
SRR511892.6619238.1+   TTGGATTCAGCCCTCATGGAGAGTTGCCTGGATCTGACATTGTGATAGGAAGTATC
SRR516994.57038585.2-   TTGGATTCAGCCCTCATGGAGAGTTGCCTGGATCTGACATTGTGATAGGAGGTGTC
SRR517009.61399791.2+   TTGGATTCAGCCCTCATGGAGAGTTGCCTGGATCTGACATTGTGATAGGAGGGGTC
SRR511914.8117859.1-   TTGGATTCAGCCCTCATGGAGAGTTGCCTGGATCTGACATTGTGATAGGAAGTATCT
SRR516968.147400290.1+   TTGGATTCAGCCCTCATGGAGAGTTGCCTGGATCTGACATTGTGATATGAGGTGTCT
SRR516979.61219488.2-   TTGGATTCAGCCCTCATGGAGAGTTGCCTGGATCTGACATTGTGATAGGAGGTGTCT
SRR516986.14380990.1-   TTGGATTCAGCCCTCATGGAGAGTTGCCTGGATCTGACATTGTGATAGGAGGTGTCT
SRR516995.62719574.1-   TTGGATTCAGCCCTCATGGAGAGTTGCCTGGATCTGACATTGTGATAGGAAGTGTCT
SRR516996.81711883.2-   TTGGATTCAGCCCTCATGGAGAGTTGCCTGGATCTGACATTGTGATAGGAAGTGTCT
SRR517006.14315108.1-   TTGGATTCAGCCCTCATGGAGAGTTGCCTGGATCTGACATTGTGATAGGAAGTGTCT
SRR511911.2865577.2+   TTGGATTCAGCCCTCATGGAGAGTTGCCTGGATCTGACATTGTGATAGGAAGTATCTT
SRR516967.149674807.2-   TTGGATTCAGCCCTCATGGAGAGTTGACTGGATCTGACATTGTGATAGGAAGTATCTT
SRR516975.20770772.2-   TTGGATTCAGCCCTCATGGAGAGTTGCCTGGATCTGACATTGTGATAGGAAGTATCTT
SRR516978.64514468.1+   TTGGATTCAGCCCTCATGGAGAGTTGCCTGGATCTGACATTGTGATAGGAGGTGTCTT
SRR516986.47797799.1+   TTGGATTCAGCCCTCATGGAGAGTTGCCTGGATCTTGCATTGTGATAGGAAGTGTCTT
SRR517002.42093965.1-   TTGGATTCAGCCCTCATGGAGAGTTGCCTGGATCTGACATTGTGATAGGAAGTATCTT
SRR511892.12533835.1+   TTGGATTCAGCCCTCATGGAGAGTTGCCTGGATCTGACATTGTGATAGGAAGTATCTTC
SRR516974.43488651.1+   TTGGATTCAGCCCTCATGGAGAGTTGCCTGGATCTGACATTGTGATAGGAAGTATCTTC
SRR516979.57710857.2-   TTGGATTCAGCCCTCATGGAGAGTTGCCTGGATCTGACATTGTGATAGGAGGTGTCTTC
SRR517007.51673519.2-   TTGGATTCAGCCCTCATGGAGAGTTGCCTGGATCTGACATTGTGATAGGAAGTATCTTC
SRR517009.90312363.1-   TTGGATTCAGCCCTCATGGAGAGTTGCCTGGATCTGACATTGTGATAGGAGGTGTCTTC
SRR511892.4161180.1+   TTGGATTCAGCCCTCATGGAGAGTTGCCTGGATCTGACATTGTGATAGGAAGTATCTTCC
SRR511892.42669582.2+   TTGGATTCAGCCCTCATGGAGAGTTGCCTGGATCTGACATTGTGATAGGAAGTATCTTCC
SRR511892.54818066.1+   TTGGATTCAGCCCTCATGGAGAGTTGCCTGGATCTGACATTGTGATAGGAAGTATCTTCC
SRR511911.24730437.2+   TTGGATTCAGCCCTCATGGAGAGTTGCCTGGATCTGACATTGTGATAGGAAGTATCTTCC
SRR511911.51765582.2-   TTGGATTCAGCCCTAATGGAGAGTTGCCTGGATCTGACATTGTGATAGGAAGTATCTTCC
SRR511911.56187465.1+   TTGGATTCAGCCCTCATGGAGAGTTGCCTGGATCTGACATTGTGATAGGAAGTATCTTCC
SRR511911.59626526.2+   TTGGATTCAGCCCTCATGGAGAGTTGCCTGGATCTGACATTGTGATAGGAAGTATCTTCC
SRR511911.74696838.2+   TTGGATTCAGCCCTCATGGAGAGTTGCCTGGATCTGACATTGTGATAGGAAGTATCTTCC
SRR511912.1303277.1-   TTGGATTCAGCCCTCATGGAGAGTTGCCTGGATCTGACATTGTGATAGGAAGTATCTTCC
SRR511912.18837352.1+   TTGGATTCAGCCCTCATGGAGAGTTGCCTGGATCTGACATTGTGATAGGAAGTATCTTCC
SRR511912.43892789.1-   TTGGATTCAGCCCTCATGGAGAGTTGCCTGGATCTGACATTGTGATAGGAAGTATCTTCC
SRR511913.2846960.2+   TTGGATTCAGCCCTCATGGAGAGGTGCCTGGATCTGACATTGTGGTAGGAAGTATCTTCC
SRR511913.35897653.2+   TTGGATTCAGCCCTCATGGAGTGTTGCCTGGATCTGACATTGTGATAGGAAGTATCTTCC
SRR511913.43839483.2+   TTGGATTCAGCCCTCATGGAGAGTTGCCTGGATCTGACATTGTGATAGGAAGTATCTTCC
SRR511913.46504761.2-   TTGGATTCAGCCCTCATGGAGAGTTGCCTGGATCTGACATTGTGATAGGAAGTATCTTCC
SRR511913.69503215.1+   TTGGATTCAGCCCTCATGGAGAGTTGCCTGGATCTGACATTGTGATAGGAAGTATCTTCC
SRR511914.12156601.2-   TTGGATTCAGCCCTCATGGAGAGTTGCCTGGATCTGACATTGTGATAGGAAGTATCTTCC
SRR511914.15324340.2+   TTGGATTCAGCCCTCATGGAGAGTTGCCTGGATCTGACATTGTGATAGGAAGTATCTTCC
SRR511914.15324684.2+   TTGGATTCAGCCCTCATGGAGAGTTGCCTGGATCTGACATTGTGATAGGAAGTATCTTCC
SRR511914.6591292.2+   TTGGATTCAGCCCTCATGGAGAGTTGCCTGGATCTGACATTGTGATAGGAAGTATCTTTC
SRR511914.80114329.2+   TTGGATTCAGCCCTCATGGAGAGTTGCCTGGATCTGACATTGTGATAGGAAGTATCTTCC
SRR511914.85321226.1+   TTGGATTCAGCCCTCATGGAGAGTTGCCTGGATCTGACATTGTGATAGGAAGTATCTTCC
SRR511914.85321569.1+   TTGGATTCAGCCCTCATGGAGAGTTGCCTGGATCTGACATTGTGATAGGAAGTATCTTCC
SRR511914.86222658.1-   TTGGATTCAGCCCTCATGGAGAGTTGCCTGGATCTGACATTGTGATAGGAAGTATCTTCC
SRR511915.28490755.2-   TTGGATTCAGCCCTCATGGAGAGTTGCCTGGATCTGACATTGTGATAGGAAGTATCTTCC
SRR511915.2930610.2-   TTGGATTCAGCCCTCATGGAGAGTTGCCTGGATCTGACATTGTGATAGGAAGTATCTTCC
SRR511915.30731583.2-   TTGGATTCAGCCCTCATGGAGAGTTGCCTGGATCTGACATTGTGATAGGAAGTATCTTCC
SRR511915.30731584.2-   GTGGATTCAGCCCTCATGGAGAGTTGCCTGGATCTGACATTGTGATAGGAAGTATCTTCC
SRR511915.43325895.2+   TTGGATTCAGCCCTCATGGAGAGTTGCCTGGATCTGACATTGTGATAGGAAGTATCTTCC
SRR511915.60393449.2-   TTGGATTCAGCCCTCATGGAGAGTTGCCTGGATCTGACATTGTGATAGGAAGTATCTTCC
SRR516967.103239374.2+   TTGGATTCAGCCCTCATGGAGAGTTGCCTGGATCTGACATTGTGATAGGAAGTGTCTTCC
SRR516967.114035844.2-   TTGGATTCAGCCCTCATGGAGAGTTGCCTGGATCTGACATTGTGATAGGAAGTATCTTCA
SRR516967.128996446.1-   TTGGATTCAGCCCTCATGGAGAGTTGCCTGGATCTGACATTGTGATAGGAAGTGTCTTCC
SRR516967.140688955.2-   TTGGATTCAGCCCTCATGGAGAGTTGCCTGGATCTGACATTGTGATAGGAAGTATCTTCC
SRR516967.30072848.2+   TTGGATTCAGCCCTCATGGAGAGTTGCCTGGATCTGACATTGTGATAGGAAGTATCTTCC
SRR516967.3323960.2-   TTGGATTCAGCCCTCATGGAGAGTTGCCTGGATCTGACATTGTGATAGGAAGTGTCTTCC
SRR516967.3860522.1-   TTGGATTCAGCCCTCATGGAGAGTTGCCTGGATCTGACATTGTGATAGGAAGTATCTTCC

SRR516967.44398778.2+	TTGGATTCAGCCCTCATGGAGAGTTGCCTGGATCTGACATTGTGATAGGAAGTGTCTTCC
SRR516967.51922488.2-	TTGGATTCAGCCCTCATGGAGAGTTGCCTGGATCTGACATTGTGATAGGAAGTATCTTCC
SRR516967.62165391.1-	TTGGATTCAGCCNTCATGGAGAGTTGCCTGGATCTGACATTGTGATAGGAAGTATCTTCC
SRR516968.118272353.1-	TTGGATTCAGCCCTCATGGAGAGTTGCCTGGATCTGACATTGTGATAGGAGGTGTCTTCC
SRR516968.128087344.2-	TTGGATTCAGCCCTCATGGAGAGTTGCCTGGATCTGACATTGTGATATGAGGTGTCTTCC
SRR516968.163565941.2+	TTGGATTCAGCCCTCATGGAGAGTTGCCTGGATCTGACATTGTGATATGAGGTGTCTTCC
SRR516968.21564094.2-	TTGGATTCAGCCCTCATGGAGAGTTGCCTGGATCTGACATTGTGATAGGAGGTGTCTTCC
SRR516968.33193255.1+	TTGGATTCAGCCCTCATGGAGAGTTGCCTGGATCTGACATTGTGATATGAGGTGTCTTCC
SRR516968.39167129.1+	TTGGATTCAGCCCTCATGGAGAGTTGCCTGGATCTGACATTGTGATAGGAGGTGTCTTCC
SRR516968.96419618.1-	TTGGATTCAGCCCTCATGGAGAGTTGCCTGGATCTGACATTGTGATATGAGGTGTCTTCC
SRR516969.23432456.1+	TTGGATTCAGCCCTCATGGAGAGTTGCCTGGATCTGACATTGTGATAGGAGGTGTCTTCC
SRR516969.35394539.2-	GTGGATTCAGCCCACATAGAGAGTTGCCTGGATCTGACATTGTGATAGGAGGTGGCTTCC
SRR516971.18513477.2-	TTGGATTCAGCCCTCATGGAGAGTTGCCTGGATCTGACATTGTGATAGGAGGTGTCTTCC
SRR516971.28756462.1+	TTGGATTCAGCCCTCATGGAGAGTTGCCTGGATCTGACATTGTGATAGGAGGTGTCTTCC
SRR516971.34870911.2+	TTGGATTCAGCCCTCATGGAGAGTTGCCTGGATCTGACATTGTGATAGGAGGTGTCTTCC
SRR516972.30272084.2-	TTGGATTCAGCCCTCATGGAGAGTTGCCTGGATCTGACATTGTGATAGGAAGTGTCTTCC
SRR516972.83556354.1+	TTGGATTCAGCCCTCATGGAGAGTTGCCTGGATCTGACATTGTGATAGGAAGTGTCTTTC
SRR516973.40895713.2-	TTGGATTCAGCCCTCATGGAGAGTTGCCTGGATCTGACATTGTGATAGGAAGTGTCTTCC
SRR516974.31202954.1+	TTGGATTCAGCCCTCATGGAGAGTTGCCTGGATCTGACATTGTGATAGGAAGTGTCTTCC
SRR516974.33828349.1-	TTGGATTCAGCCCTCATGGAGAGTTGCCTGGATCTGACATTGTGATAGGAAGTATCTTCC
SRR516974.38378408.2-	TTGGATTCAGCCCTCATGGAGAGTTGCCTGGATCTGACATTGTGATAGGAAGTATCTTCC
SRR516974.40379406.1-	TTGGATTCAGCCCTCATGGAGAGTTGCCTGGATCTGACATTGTGATAGGAAGTGTCTTCC
SRR516974.4422789.2+	TTGGATTCAGCCCTCATGGAGAGTTGCCTGGATCTGACATTGTGATAGGAAGTATCTTCC
SRR516974.45483276.1-	TTGGATTCAGCCCTCATGGAGAGTTGCCTGGATCTGACATTGTGATAGGAAGTATCTTCC
SRR516974.49320234.1-	TTGGATTCAGCCCTCATGGAGAGTTGCCTGGATCTGACATTGTGATAGGAAGTGTCTTCC
SRR516974.54553033.2+	TTGGATTCAGCCCTCATGGAGAGTTTCCTGGATCTGACATTGTGAAAGGAAGTATCTTAC
SRR516974.72219416.1-	TTGGATTCAGCCCTCATGGAGAGTTGCCTGGATCTGACATTGTGATAGGAAGTGTCTTCC
SRR516975.56296657.1-	TTGGATTCAGCCCTCATGGAGAGTTGCCTGGATCTGACATTGTGATAGGAAGTATCTTCC
SRR516977.10847396.1+	TTGGATTCAGCCCTCATGGAGAGTTGCCTGGATCTGACATTGTGATCGGAGGTGTCTTCC
SRR516977.12873538.2-	TTGGATTCAGCCCTCATGGAGAGTTGCCTGGATCTGACATTGTGATAGGAGGTGTCTTCC
SRR516977.16216859.1+	TTGGATTCAGCCCTCATGGAGAGTTGCCTGGATCTGACATTGTGATAGGAGGTGTCTTCC
SRR516977.43459967.2+	TTGGATTCAGCCCTCATGGAGAGTTGCCTGGATCTGACATTGTGATAGGAGGTGTCTTCC
SRR516977.5387374.2+	TTGGATTCAGCCCTCATGGAGAGTTGCCTGGATCTGACATTGTGATAGGAGGTGTCTTCC
SRR516977.80260583.2-	TTGGATTCAGCCCTCATGGAGAGTTGCCTGGATCTGACATTGTGATAGGAGGTGTCTTCC
SRR516978.15412507.1+	TTGGATTCAGCCCTCATGGAGAGTTGCCTGGATCTGACATTGTGATAGGAGGTGTCTTCC
SRR516978.26037079.1+	TTGGATTCAGCCCTCATGGAGAGTTGCCTGGATCTGACATTGTGATAGGAGGTGTCTTCC
SRR516978.31035699.2+	TTGGATTCAGCCCTCATGGAGAGTTGCCTGGATCTGACATTGTGATAGGAGGTGTCTTCC
SRR516978.39183046.2+	TTGGATTCCGCCCTCATGGAGAGTTGCCTGGATCTGACATTGTGATAGGAGGTGACTTAC
SRR516978.43155707.2+	TTGGATTCAGCCCTCATGGAGAGTTGCCTGGATCTGACATTGTGATAGGAGGTGTCTTCC
SRR516978.58338306.2+	TTGGATTCAGCCCTCATGGAGAGTTGCCTGGATCTGACATTGGGATAGGAGGTGTCTTCC
SRR516979.52325428.1-	TTGGATTCAGCCCTCATGGAGAGTTGCCTGGATCTGACATTGTGATAGGAGGTGTCTTCC
SRR516980.15856106.1-	TTGGATTCAGCCCTCATGGAGAGTTGCCTGGATCTGACATTGTGATAGGAGGTGTCTTCC
SRR516980.23012010.2+	TTGGATTCAGCCCTCATGGAGAGTTGCCTGGATCTGACATTGTGATAGGAGGGGTCTTCC
SRR516980.51880329.2-	TTGGATTCAGCCCTCATGGAGAGTTGCCTGGATCTGACATTGTGATAGGAGGTGTCTTCC
SRR516980.61023510.2+	TTGGATTCAGCCCTCATGGAGAGTTGCCTGGATCTGACATTGTGATAGGAGGTGTCTTCC
SRR516981.10390610.2-	TTGGATTCAGCCCTCATGGAGAGTTGCCTGGATCTGACATTGTGATAGGAAGTGTCTTCC
SRR516981.37172809.1-	TTGGATTCAGCCCTCATGGAGAGTTGCCTGGATCTGACATTGTGATAGGAAGTGTCTTCC
SRR516981.62187975.2-	TTGGATTCAGCTCTCATGGAGAGTTGCCTGGATCTGACATTGTGATAGGAAGTGTCTTCC
SRR516981.72937619.1-	TTGGATTCAGCCCTCATGGAGAGTTGCCTGGATCTGACATTGTGATAGGAAGTGTCTTCC
SRR516981.75185944.1-	TTGGATTCAGCCCTCATGGAGAGTTGCCTGGATCTGACATTGTGATAGGAAGTGTCTTCC
SRR516981.79962474.1+	TTGGATTCAGCCCTCATGGAGAGTTGCCTGGATCTGACATTGTGATAGGAAGTATCTTCC
SRR516983.29420342.1-	TTGGATTCAGCCCTCATGGAGAGTTGCCTGGATCTGACATTGTGATAGGAAGTATCTTCC
SRR516983.39104642.2+	TTGGATTCAGCCCTCATGGAGAGTTGCCTGGATCTGACATTGTGATAGGAAGTATCTTCC
SRR516983.72843190.1-	TTGGATTCAGCCCTCATGGAGAGTTGCCTGGATCTGACATTGTGATAGGAAGTGTCTTCC
SRR516984.43825566.1-	TTGGATTCAGCCCTCATGGAGAGTTGCCTGGATCTGACATTGTGATAGGAAGTATCTTCC
SRR516984.67730810.2-	TTGGATTCAGCCCTCATGGAGAGTTGCCTGGATCTGACATTGTGATAGGAAGTATCTTCC
SRR516984.80897040.1-	TTGGATTCAGCCCTCATGGAGAGTTGCCTGGATCTGACATTGTGATAGGAAGTATCTTCC
SRR516984.88862730.1-	TTGGATTCAGCCCTCATGGAGAGTTGCCTGGATCTGACATTGTGATAGGAGGTGTCTTCC
SRR516986.38251531.2-	TTGGATTCAGCCCTCATGGAGAGTTGCCTGGATCTGACATTGTGATAGGAAGTGTCTTCC
SRR516986.43082651.2-	TTGGATTCAGCCCTCATGGAGAGTTGCCTGGATCTGACATTGTGATAGGAGGTGTCTTCC
SRR516986.47463977.1-	TTGGATTCAGCCCTCATGGAGAGTTGCCTGGATCTGACATTGTGATAGGAGGTGTCTTCC
SRR516986.5284326.1-	TTGGATTCAGCCCTCATGGAGAGTTGCCTGGATCTGACATTGTGATAGGAAGTGTCTTCC
SRR516986.62178078.2+	TTGGATTCAGCCCTCATGGAGAGTTGCCTGGATCTGACATTGTGATAGGAAGTGTCTTCC
SRR516987.72954228.2-	TTGGATTCAGCCCTCATGGAGAGTTGCCTGGATCTGACATTGTGATAGGAAGTATCTTCC
SRR516988.25345425.2+	TTGGATTCAGCCCTCATGGAGAGTTGCCTGGATCTGACATTGTGATAGGAGGGGGCTTCC
SRR516988.49719328.1-	TTGGATTCAGCCCTCATGGAGAGTTGCCTGGATCTGACATTGTGATAGGAGGTGTCTTCC
SRR516989.23071515.2-	TTGGATTCAGCCCTCATGGAGAGTTGCCTGGATCTGACATTGTGATATGAGGTGTCTTCC
SRR516989.53465710.1-	TTGGATTCAGCCCTCATGGAGAGTTGCCTGGATCTGACATTGTGATATGAGGTGTCTTCC
SRR516989.74819632.1-	TTGGATTCAGCCCTCATGGAGAGTTGCCTGGATCTGACATTGTGATATGAGGTGTCTTCC
SRR516989.82467866.2-	TTGGATTCAGCCCTCATGGAGAGTTGCCTGGATCTGACATTGTGATATGAGGTGTCTTCC
SRR516990.33601261.1-	TTGGATTCAGCCCTCATGGAGAGTTGCCTGGATCTGACATTGTGATAGGAAGTGTCTTCC
SRR516990.47204932.2+	TTGGATTCAGCCCTCATGGAGAGTTGCCTGGATCTGACATTGTGATAGGAAGTGTCTTCC
SRR516990.60531713.1-	TTGGATTCAGCCCTCATGGAGAGTTGCCTGGATCTGACATTGTGATAGGAAGTGTCTTCC
SRR516991.23448506.1+	TTGGATTCAGCCCTCATGGAGAGTTGCCTGGATCTGACATTGTGATAGGAAGTGTCTTCC
SRR516991.46156534.1-	TTGGATTCAGCCCTCATGGAGAGTTGCCTGGATCTGACATTGTGATAGGAAGTGTCTTCC
SRR516991.78056440.1-	TTGGATTCAGCCCTCATGGAGAGTTGCCTGGATCTGACATTGTGATAGGAAGTGTCTTCC
SRR516991.91013899.2+	TTGGATTCAGCCCTCATGGAGAGTTGCCTGGATCTGACATTGTGATAGGAAGTGTCTTCC
SRR516992.15393409.1-	TTGGATTCAGCCCTCATGGAGAGTTGCCTGGATCTGACATTGTGATAGGAGGTGTCTTCC
SRR516992.45921129.1+	TTGGATTCAGCCCTCATGGAGAGTTGCCTGGATCGGACATTGTGATAGGAGGTGTCTTCC
SRR516992.55475284.2+	TTGGATTCAGCCCTCATGGAGAGTTGCCTGGATCTGACATTGTGATAGGAGGTGTCTTCC
SRR516992.6010329.1-	TTGGATTCAGCCCTCATGGAGAGTTGCCTGGATCTGACATTGTGATAGGAAGTATCTTCC
SRR516993.2915184.1+	TTGGATTCAGCCCTCATGGAGAGTTGCCTGGATCTGACATTGTGATAGGAAGTGTCTTCC
SRR516993.35093586.1+	TTGGATTCAGCCCTCATGGAGAGTTGCCTGGATCTGACATTGTGATAGGAAGTGTCTTCC
SRR516993.472471.2+	TTGGATTCAGCCCTCATGGAGAGTTGCCTGGATCTGACATTGTGATAGGAGGTGTCTTCC

```
SRR516993.65644538.2+    TTGGATTCAGCCCTCATGGAGAGTTGCCTGGATCTGACATTGTGATAGGAAGTGTCTTCC
SRR516994.3610095.1+     TTGGATTCAGCCCTCATGGAGAGTTGCCTGGATCTGACATTGTGATAGGAGGTGTCTTCC
SRR516994.39206055.2-    TTGGATTCAGCCCTCATGGAGAGTTGCCTGTATCTGACATTGTGATAGGAGGTGTCTTCC
SRR516994.44481978.2-    TTGGATTCAGCCCTCATGGAGAGTTGCCTGGATCTGACATTGTGATAGGAGGTGTCTTCC
SRR516994.49855553.1+    TTGGATTCAGCCCTCATGGAGAGTTGCCTGGATCTGACATTGGGATAGGAGGTGTCTTCC
SRR516994.52852729.1+    TTGGATTCAGCCCTCATGGAGAGTTGCCTGGATCTGACATTGTGATAGGAGGTGTCTTCC
SRR516994.77692468.2+    TTGGATTCAGCCCTCATGGAGAGTTGCCTGGATCTGACATTGTGATAGGAGGTGTCTTCC
SRR516994.81814726.1-    TTGGATTCAGCCCTCATGGAGAGTTGCCTGGATCTGACATTGTGATAGGAGGTGTCTTCC
SRR516995.22467748.1+    TTGGATTCAGCCCTCATGGAGAGTTGCCTGGATCTGACATTGTGATAGGAAGTGTCTTCC
SRR516995.33739366.2-    TTGGATTCAGCCCTCATGGAGAGTTGCCTGGATCTGACATTGTGATAGGAAGTGTCTTCC
SRR516995.50404448.2-    TTGGATTCAGCCCTCATGGAGAGTTGCCTGGACCTGACATTGTGATAGGAAGTGTCTTCC
SRR516995.52974433.1+    TTGGATTCAGCCCTCATGGAGAGTTGCCTGGATCTGACATTGTGATAGGAAGTGTCTTCC
SRR516995.53678391.1+    TTGGATTCAGCCCTCATGGAGAGTTGCCTGGATCTGACATTGTGATAGGAGGTGTCTTCC
SRR516995.56989024.1+    TTGGATTCAGCCCTCATGGAGAGTTGCCTGGATCTGACATTGTGATAGGAGGTGTCTTCC
SRR516995.66321276.1+    TTGGATTCAGCCCTCATGGAGAGTTGCCTGGATCTGACATTGTGATAGGAAGTGTCTTCC
SRR516995.928256.2+      TTGGATTCAGCCCTCACGGAGAGTTGCCTGGATCTGACATTGTGATAGGAAGTGTCTTCC
SRR516996.23482257.1-    TTGGATTCAGCCCTCATGGAGAGTTGCCTGGATCTGACATTGTGATAGGAAGTGTCTTCC
SRR516996.34976669.1+    TTGGATTCAGCCCTCATGGAGAGTTGCCTGGATCTGACATTGTGATAGGAAGTGTCTTCC
SRR516996.85062640.1-    TTGGATTCAGCCCTCATGGAGAGTTGCCTGGATCTCACATTGTGATAGGAAGTGTCTTCC
SRR516996.87236229.2+    TTGGATTCAGCCCTCATGGAGAGTTGCCTGGGTCTGACATTGTGATAGGAAAAGTCTTTC
SRR516996.92591042.1+    TTGGATTCAGCCCTCATGGAGAGTTGCCTGGATCTGACATTGTGATAGGAAGTGTCTTCC
SRR516997.42706554.2-    TTGGATTCAGCCCTCATGGAGAGTTGCCTGGATCTGACATTGTGATAGGAAGTATCTTCC
SRR516997.97876806.2-    TTGGATTCAGCCCTCATGGAGAGTTGCCTGGATCGGACATTGTGATAGGAAGTATCTTCC
SRR516998.105584836.2-   TTGGATTCAGCCCTCATGGAGAGTTGCCTGGATCTGACATTGTGATAGGAGGTGTCTTCC
SRR516998.105713546.1-   TTGGATTCAGCCCTCATGGAGAGTTGCCTGGATCTGACATTGTGATAGGAAGTGTCTTCC
SRR516998.42387076.2-    TTGGATTCAGCCCTCATGGAGAGTTGCCTGGATCTGACATTGTGATAGGAAGTGTCTTCC
SRR516998.45234491.1-    TTGGATTCAGCCCTCATGGAGAGTTGCCTGGATCTGACATTGTGATAGGAAGTGTCTTCC
SRR516998.65740325.1+    TTGGATTCAGCCCTCATGGAGAGTTGCCTGGATCTGACATTGTGATAGGAAGTGTCTTCC
SRR516998.74008326.1+    TTGGATTCAGCCCTCATGGAGAGTTGCCTGGATCTGACATTGTGATCGGAGGGGTCTTCC
SRR516998.74633545.1+    TTGGATTCAGCCCTCATGGAGAGTTGCCTGGATCTGACATTGTGATAGGAAGTGTCTTCC
SRR516998.90024593.2-    TTGGATTCAGCCCTCATGGAGAGTTGCCTGGATCTGACATTGTGATAGGAAGTGTCTTCC
SRR516999.103891959.2-   TTGGATTCAGCCCTCAGGGAGAGTTGCCTTGATCTGACGTTGTGATAGGAAGTATCTTCC
SRR516999.108053227.2-   TTGGATTCAGCCCTCATGGAGAGTTGCCTGGATCTGACATTGTGATAGGAAGTATCTTCC
SRR516999.38071679.1-    TTGGATTCAGCCCTCATGGAGAGTTGCCTGGATCTGACATTGTGATAGGAAGTATCTTCC
SRR516999.51761.1+       TTGGATTCAGCCCTCATGGAGAGTTGCCTGGATCTGACATTGTGATAGGAAGTGTCTTCC
SRR516999.52117285.1+    TTGGATTCAGCCCTCATGGAGAGTTGCCTGGATCTGACATTGTGATAGGAAGTGTCTTCC
SRR516999.9724330.1-     TTGGATTCAGCCCTCATGGAGAGTTGCCTGGATCTGACATTGTGATAGGAAGTATCTTCC
SRR517000.104217922.1-   TTGGATTCAGCCCTCATGGAGAGTTGCCTGGATCTGACATTGTGATAGGAAGTGTCTTCC
SRR517000.36416800.1+    TTGGATTCAGCCCTCATGGAGAGTTGCCTGGATCTGACATTGTGATAGGAGGTGTCTTCC
SRR517000.52358860.1+    TTGGATTCAGCCCTCATGGAGAGTTGCCTGGATCTGACATTGTGATAGGAGGTGTCTTCC
SRR517001.2955954.2+     TTGGATTCAGCCCTCATGGAGAGTTGCCTGGATCTGACATTGTGATAGGAGGTGTCTTCC
SRR517001.4174199.1+     TTGGATTCAGCCCTCATGGAGAGTTGCCTGGATCTGACATTGTGATAGGAGGTGTCTTCC
SRR517001.53006899.2-    TTGGATTCAGCCCTCATGGAGAGTTGCCTGGATCTGACATTGTGATAGGAGGTGTCTTCC
SRR517002.24413856.1-    TTGGATTCAGCCCTCATGGAGAGTTGCCTGGATCTGACATTGTGATAGGAAGTATCTTCC
SRR517002.45348.1+       TTGGATTCAGCCCTCATGGAGAGTTGCCTGGATCTGACATTGTGATAGGAAGTATCTTCC
SRR517002.77942367.2-    TTGGCTTCAGCCCTCATGGAGAGTTGCCTGGATCTGACATTGTGATAGGAAGTATCTTCC
SRR517002.88618213.1+    TTGGATTCAGCCCTCCTGGAGAGTTGCCTGGATCTGACATTGTGATAGGAAGTATCTTCC
SRR517002.88668875.2-    TTGGATTCAGCCCTCATGGAGAGTTGCCTGGATCTGACATTGTGATAGGAAGTATCTTCC
SRR517002.98887947.1-    TTGGATTCAGCCCTCATGGAGAGTTGCCTGGATCTGACATTGTGATAGGAAGTATCTTCC
SRR517003.18297409.2-    TTGGATTCAGCCCTCATGGAGAGTTGCCTGGATCTGACATTGTGATAGGAGGTGTCTTCC
SRR517003.19490054.2-    TTGGATTCAGCCCTCATGGAGAGTTGCCTGGATCTGACATTGTGATAGGAGGTGTCTTCC
SRR517003.41980170.1-    TTGGATTCAGCCCTCATGGAGAGTTGCCTGGATCTGACATTGTGATAGGAAGTATCTTCC
SRR517003.61889052.2+    TTGGATTCAGCCCTCATGGAGAGTTGCCTGGATCTGACATTGTGATAGGAAGTATCTTCC
SRR517003.70211691.2-    TTGGATTCAGCCCTCATGGAGAGTTGCCTGGATCTGACATTGTGATAGGAGGTGTCTTCC
SRR517003.75889442.2+    TTGGATTCAGCCCTCATGGAGTGATGCCTGGATCTGACATTGTTATAGGAGGTGTATTCC
SRR517004.35890882.2+    TTGGATTCAGCCCTCATGGAGAGTTGCCTGGATCTGACATTGTGATAGGAGGGGTCTTCC
SRR517005.58912099.2+    TTGGATTCAGCCCTCATGGAGAGTTGCCTGGATCTGACATTGTGATAGGAAGTATCTTCC
SRR517005.61088972.2+    TTGGATTCAGCCCTCATGGAGAGTTCCCTGGATCTGACATTGTGATAGGAAGTATCTTCC
SRR517005.8220247.2-     TTGGATTCAGCCCTCATGGAGAGTTGCCTGGATCTGACATTGTGATAGGAGGTGTCTTCC
SRR517005.97174151.1-    TTGGATTCAGCCCTCATGGAGAGTTGCCTGGATCTGACATTGTGATAGGAGGTGTCTTCC
SRR517005.99236075.2-    TTGGATTCAGCCCTAATGGAGAGTTGCCTGGATCTGACATTGTGATAGGAAGTATCTTCC
SRR517006.19437322.1-    TTGGATTCAGCCCTCATGGAGAGTTGCCTGGATCTGACATTGTGATAGGAAGTGTCTTCC
SRR517007.4303113.2+     TTGGATTCAGCCCTCATGGAGAGTTGCCTGGATCTGACATTGTGATAGGAAGTATCTTCC
SRR517007.626055.1-      TTGGATTCAGCCCTCATGGAGAGTTGCCTGGATCTGACATTGTGATAGGAAGTATCTTCC
SRR517008.44606589.1-    TTGGATTCAGCCCTCATGGAGAGTTGCCTGGATCTGACATTGTGATAGGAGGTGTCTTCC
SRR517008.65933108.2-    TTGGATTCAGCCCTCATGGAGAGTTGCCTGGATCTGACATTGTGATAGGAGGTGTCTTCC
SRR517008.68337962.1-    TTGGATTCAGCCCTCATGGAGAGTTGCCTGGATCTGACATTGTGATAGGAGGTGTCTTCC
SRR517008.68826874.2-    TTGGATTCAGCCCTCATGGAGAGTTGCCTGGATCTGACATTGTGATAGGAGGTGTCTTCC
SRR517008.69459447.1+    TTGGATTCAGCCCTCATGGAGAGTTGCCTGGATCTGACATTGTGATAGGAGGTGTCTTCC
SRR517008.87918252.2-    TTGGATTCAGCCCTCATGGAGAGTTGCCTGGATCTGACATTGTGATAGGAGGTGTCTTCC
SRR517008.9761471.2+     TTGGATTCAGCCCTCATGGAGAGTTGCCTGGATCTGACATTGTGATAGGAGGGGTCTTCC
SRR517009.35173062.1-    TTGGATTCAGCCCTCATGGAGAGTTGCCTGGATCTGACATTGTGATAGGAGGTGTCTTCC
SRR517009.36170965.2+    TTGGATTCAGCCCTCATGGAGAGTTGCCTGGATCTGACATTGTGATAGGAAGTGTCTTCC
SRR517009.40589595.1-    TTGGATTCAGCCCTCATGGAGAGTTGCCTGGATCTGACATTGTGATAGGAAGTGTCTTCC
SRR517009.46333.1+       TTGGATTCAGCCCTCATGGAGAGTTGCCTGGATCTGACATTGTGATAGGAGGGGTCTTCC
SRR517009.47953341.2+    TTGGATTCAGCCCTCATGGAGAGTTGCCTGGATCTGACATTGTGATAGGAGGTGTCTTCC
SRR517009.59440565.2-    TTGGATTCAGCCCTCATGGAGAGTTGCCTGGATCTGACATTGTGATAGGAAGTGTCTTCC
SRR517009.79220826.2-    TTGGATTCAGCCCTCATGGAGAGTTGCCTGGATCTGACATTGTGATAGGAGGTGTCTTCC
SRR517009.90290402.2+    TTGGATTCAGCCCTCATGGAGAGTTGCCTGGATCTGACATTGTGATAGGAGGTGTCTTCC
SRR511892.9511611.1-      TGGATTCAGCCCTCAGGGAGAGTTGCCTGGATCTGACATTGTGATAGGAAGTATCTTCC
SRR516967.113781662.2-    TGGATTCAGCCCTCATGGAGAGTTGCCTGGATCTGACATTGTGATAGGAAGTATCTTCC
SRR516974.49170123.2-     TGGATTCAGCCCTCATGGAGAGTTGCCTGGATCTGACATTGTGATAGGAAGTATCTTCC
SRR516981.9101608.1+      TGGATTCAGCCCTCATGGAGAGTTGCCTGGATCTGACATTATGATAGGAAGTATCTTCC
SRR516988.44614705.1-     TGGATTCAGCCCTCATGGAGAGTTGCCTGGATCTGACATTGTGATAGGAGGTGTCTTCC
```

```
SRR516996.21050103.2+     TGGATTCAGCCCTCATGGAGAGTTGCCTGGATCTGACATTGTGATAGGAAGTGTCTTCC
SRR516997.65362583.2+     TGGATTCAGCCCTCATGGAGAGTTGCCTGGATCTGACATTGTGATAGGAAGTATCTTCC
SRR517002.7720294.2-      TGGATTCAGCCCTCATGGAGAGTTGCCTGGATCTGACATTGTGATAGGAAGTATCTTCC
SRR517003.32714941.1-     TGGATTCAGCCCTCATGGAGAGTTGCCTGGATCTGACATTGTGATAGGAGGTGTCTTCC
SRR517003.71024107.1-     TGGATTCAGCCCTCATGGAGAGTTGCCTGGATCTGACATTGCGATAGGAAGTATCTTCC
SRR511918.32212009.2+      GGATTCAGCCCTCATGGAGAGTTGCCTGGATCTGACATTGTGATAGGAA
SRR511918.7457548.2+       GGATTCAGCCCTCATGGAGAGTTGCCTGGATCTGACATTGTGATAGGAA
SRR511911.60280636.1-      GGATTCAGCCCTCATGGAGAGTTGCCTGGATCTGACATTGTGATAGGAAGTATCTTCC
SRR511912.78910790.2-      GGATTCAGCCCTCATGGAGAGTTGCCTGGATCTGACATTGTGATAGGAAGTATCTTCC
SRR511914.67096997.1-      GGATTCAGCCCTCATGGAGAGTTGCCTGGATCTGACATTGTGATAGGAAGTATCTTCC
SRR516992.35154680.2+      GGATTCAGCCCTCATGGAGAGTTGCCTGGATCTGACATTGTGATAGGAGGTGTCTTCC
SRR516998.54949380.1+      GGATTCAGCCCTCATGGAGAGTTGCCTGGATCTGACATTGTGATAGGAGGTGTCTTCC
SRR516999.4765606.2-       GGATTCAGCCCTCATGGAGAGTTGCCTGGATCTGACATTGTGATAGGAAGTATCTTCC
SRR517006.28181699.2-      GGATTCAGCCCTCATGGAGAGTTGCCTGGATCTGACATTGTGATAGGAAGTGTCTTCC
SRR511914.46576162.1-       GATTCAGCCCTCAGGGAGAGTTGCCTGGATCTGACATTGTGATAGGAAGTATCTTCC
SRR511915.35046284.2-       GATTCAGCCCTCATGGAGAGTTGCCTGGATCTGACATTGTGATAGGAAGTATCTTCC
SRR516968.169513645.2+      GATTCAGCCCTCATGGAGAGTTGCCTGGATCTGACATTGTGATAGGAGGTGTCTTCC
SRR516988.46830167.2-       GATTCAGCCCTCATGGAGAGTTGCCTGGATCTGACATTGTGATAGGAGGTGTCTTCC
SRR516990.37851369.1+       GATTCAGCCCTCATGGAGAGTTGCCTGGATCTGACATTGTGATAGGAAGTGTCTTCC
SRR516995.55575214.2-       GATTCAGCCCTCATGGAGAGTTGCCTGGATCTGACATTGTGATAGGAGGTGTCTTCC
SRR517003.40047671.2-       GATTCAGCCCTCATGGAGAGTTGCCTGGATCTGACATTGTGATAGGAAGTATCTTCC
SRR516969.15376651.1-        ATTCAGCCCTCATGGAGAGTTGCCTGGATCTGACATTGTGATAGGAGGTGTCTTCC
SRR516972.13628751.2+        ATTCAGCCCTCATGGAGAGTTGCCTGGATCTGACATTGTGATAGGAAGTGTCTTCC
SRR516977.42799382.2+        ATTCAGCCCTCATGGAGAGTTGCCTGGATCTGACATTGTGATAGGAGGTGTCTTCC
SRR516999.94573754.1+        ATTCAGCCCTCATGGAGAGTTGCCTGGATCTGACATTGTGATAGGAAGTGTCTTCC
SRR517001.30441251.2+        ATTCAGCCCTCATGGAGAGTTGCCTGGATCTGACATTGTGATAGGAGGTGTCTTCC
SRR511916.42515370.1+         TTCAGCCCTCATGGAGAGTTGCCTGGATCTGACATTGTGATAGGAAGTA
SRR516968.108115361.2-        TTCAGCCCTCATGGAGAGTTGCCTGGATCTGACATTGTGATAGGAGGTGTCTTCC
SRR516968.53031658.2-         TTCAGCCCTCATGGAGAGTTGCCTGGATCTGACATTGTGATAGGAGGTGTCTTCC
SRR516969.27833237.2-         TTCAGCCCTCAGGGAGAGTTGCCTGGATCTGACATTGTGATAGGAGGTGTCTTCC
SRR516996.2184957.1+          TTCAGCCCTCATGGAGAGTTGCCTGGATCTGACATTGTGATAGGAAGTGTCTTCC
SRR516996.58664910.1-         TTCAGCCCTCATGGAGAGTTGCCTGGATCTGACATTGTGATAGGAAGTGTCTTCC
SRR517004.43077788.2+         TTCAGCCCTCATGGAGAGTTGCCTGGATCTGACATTGTGATAGGAGGTGTCTTCC
SRR517009.74371807.2-         TTCAGCCCTCATGGAGAGTTGCCTGGATCTGACATTGTGATAGGAGGTGTCTTCC
SRR511892.45271449.1-          TCAGCCCTCATGGAGAGTTGCCTGGATCTGACATTGTGATAGGAAGTATCTTCC
SRR511911.58123630.2-          TCAGCCCTCATGGAGAGTTGCCTGGATCTGACATTGTGATAGGAAGTATCTTCC
SRR511911.63363441.2+          TCAGCCCTCATGGAGAGTTGCCTGGATCTGACATTGTGATAGGAAGTATCTTCC
SRR511913.39997243.2+          TCAGCCCTCATGGAGAGTTGCCTGGATCTGACATTGTGATAGGAAGTATCTTCC
SRR516996.70382886.1-          TCAGCCCTCATGGAGAGTTGCCTGGATCTGACATTGTGATAGGAAGTGTCTTCC
SRR517009.30109562.1-          TCAGCCCTCATGGAGAGTTGCCTGGATCTGACATTGTGATAGGAAGTGTCTTCC
SRR511911.1348761.1+            CAGCCCTCATGGAGAGTTGCCTGGATCTGACATTGTGATAGGAAGTATCTTCC
SRR511912.39211104.1-           CAGCCCTCATGGAGAGTTGCCTGGATCTGACATTGTGATAGGAAGTATCTTCC
SRR516977.29390676.2+           CAGCCCTCATGGAGAGTTGCCTGGATCTGACATTGTGATAGGAGGTGTCTTCC
SRR516977.47398733.2-           CAGCCCTCATGGAGAGTTGCCTGGATCTGACATTGTGATAGGAGGTGTCTTCC
SRR516977.52908323.1-           CAGCCCTCATGGAGAGTTGCCTGGATCTGACATTGTGATAGGAGGTGTCTTCC
SRR516980.67663155.2-           CAGCCCTCATGGAGAGTTGCCTGGATCTGACATTGTGATAGGAGGTGTCTTCC
SRR516981.43550688.1+           CAGCCCTCATGGAGAGTTGCCTGGATCTGACATTGTGATAGGAAGTATCTTCC
SRR516996.85687262.2-           CAGCCCTCATGGAGAGTTGCCTGGATCTGACATTGTGATAGGAAGTGTCTTCC
SRR517000.69157843.1+           CAGCCCTCATGGAGAGTTGCCTGGATCTGACATTGTGATAGGAAGTGTCTTCC
SRR517000.8876587.2+            CAGCCCTCATGGAGAGTTGCCTGGATCTGACATTGTGATAGGAAGTGTCTTCC
SRR511911.11918542.2+            AGCCCTCATGGAGAGTTGCCTGGATCTGACATTGTGATAGGAAGTATCTTCC
SRR511911.31335408.1+            AGCCCTCATGGAGAGTTGCCTGGATCTGACATTGTGATAGGAAGTATCTTCC
SRR516968.92422565.1+            AGCCCTCATGGAGAGTTGCCTGGATCTGACATTGTGATAGGAGGTGTCTTCC
SRR516977.59451244.1-            AGCCCTCATGGAGAGTTGCCTGGATCTGACATTGTGATAGGAGGTGTCTTCC
SRR516980.7478290.1-             AGCCCTCATGGAGAGTTGCCTGGATCTGACATTGTGATAGGAGGTGTCTTCC
SRR511913.35035416.2-             GCCCTCATGGAGAGTTGCCTGGATCTGACATTGTGATAGGAAGTATCTTCC
SRR516987.61502473.1-             GCCCTCATGGAGAGTTGCCTGGATCTGACATTGTGATAGGAAGTATCTTCC
SRR516997.11539523.2+             GCCCTCATGGAGAGTTGCCTGGATCTGACATTGTGATAGGAAGTATCTTCC
SRR517000.77997273.2-             GCCCTCATGGAGAGTTGCCTGGATCTGACATTGTGATAGGAAGTGTCTTCC
SRR517008.80303637.1+             GCCCTCATGGAGAGTTGCCTGGATCTGACATTGTGATAGGAGGTGTCTTCC
SRR511913.67606827.2-              CCCTCATGGAGAGTTGCCTGGATCTGACATTGTGATAGGAAGTATCTTCC
SRR516973.6568969.2-               CCCTCATGGAGAGTTGCCTGGATCTGACATTGTGATAGGAAGTGTCTTCC
SRR516990.7184572.1+               CCCTCATGGAGAGTTGCCTGGATCTGACATTGTGATAGGAAGTGTCTTCC
SRR516996.11671795.2-              CCCTCATGGAGAGTTGCCTGGATCTGACATTGTGATAGGAAGTGTCTTCC
SRR516996.95894100.2-              CCCTCATGGAGAGTTGCCTGGATCTGACATTGTGATAGGAAGTGTCTTCC
SRR517001.80549406.1+              CCCTCATGGAGAGTTGCCTGGATCTGACATTGTGATAGGAGGTGTCTTCC
SRR511916.1909951.1-                CCTCATGGAGAGTTGCCTGGATCTGACATTGTGATAGGAAGTATCTTCC
SRR516981.84093782.2-               CCTCATGGAGAGTTGCCTGGATCTGACATTGTGATAGGAAGTATCTTCC
SRR516984.12082939.1-               CCTCATGGAGAGTTGCCTGGATCTGACATTGTGATAGGAGGTGTCTTCC
SRR517006.50484772.1+               CCTCATGGAGAGTTGCCTGGATCTGACATTGTGATAGGAAGTGTCTTCC
SRR517009.48303164.2+               CCTCATGGAGAGTTGCCTGGATCTGACATTGTGATAGGAGGTGTCTTCC
SRR511892.54323619.2+                CTCATGGAGAGTTGCCTGGATCTGACATTGTGATAGGAAGTATCTTCC
SRR511913.39320808.2-                CTCATGGAGAGTTGCCTGGATCTGACATTGTGATAGGAAGTATCTTCC
SRR516974.60321821.2-                CTCATGGAGAGTTGCCTGGATCTGACATTGTGATAGGAAGTATCTTCC
SRR516986.48107818.2-                CTCATGGAGAGTTGCCTGGATCTGACATTGTGATAGGAAGTGTCTTCC
SRR516989.87718993.2+                CTCATGGAGAGTTGCCTGGATCTGACATTGTGATATGAGGTGTCTTCC
SRR517001.78342098.1-                CTCATGGAGAGTTGCCTGGATCTGACATTGTGATAGGAGGTGTCTTCC
SRR516972.40740620.1+                 TCATGGAGAGTTGCCTGGATCTGACATTGTGATAGGAAGTGTCTTCC
SRR516977.35615817.1-                 TCATGGAGAGTTGCCTGGATCTGACATTGTGATAGGAGGTGTCTTCC
SRR516980.28641220.2+                 TCATGGAGAGTTGCCTGGATCTGACATTGTGATAGGAGGTGTCTTCC
SRR516981.7950043.2+                  TCATGGAGAGTTGCCTGGATCTGACATTGTGATAGGAAGTGTCTTCC
SRR516984.39430615.2-                 TCATGGAGAGTTGCCTGGATCTGACATTGTGATAGGAGGTGTCTTCC
SRR516993.65564502.1+                 TCATGGAGAGTTGCCTGGATCTGACATTGTGATAGGAAGTGTCTTCC
SRR516996.20396000.1-                 TCATGGAGAGTTGCCTGGATCTGACATTGTGATAGGAAGTGTCTTCC
```

SRR516997.6377760.1+	TCATGGAGAGTTGCCTGGATCTGACATTGTGATAGGAAGTATCTTCC
SRR517004.25460609.1+	TCATGGAGAGTTGCCTGGATCTGACATTGTGATAGGAGGTGTCTTCC
SRR517004.25658369.2+	TCATGGAGAGTTGCCCGGATCTGACATTGTGATAGGAGGGGTCTTCC
SRR517008.70570764.1-	TCATGGAGAGTTGCCTGGATCTGACATTGTGATAGGAGGTGTCTTCC
SRR517009.74175557.1-	TCATGGAGAGTTGCCTGGATCTGACATTGTGATAGGAAGTGTCTTCC
SRR516967.123439529.1-	CATGGAGAGTTGCCTGGATCTGACATTGTGATAGGAAGTGTCTTCC
SRR516971.10078918.2-	CATGGAGAGTTGCCTGGATCTGACATTGTGATAGGAGGTGTCTTCC
SRR516972.19207856.2+	CATGGAGAGTTGCCTGGATCTGACATTGTGATAGGAAGTGTCTTCC
SRR516978.31082004.1+	CATGGAGAGTTGCCTGGATCTGACATTGTGATAGGAGGGGTCTTCC
SRR516984.29026302.1-	CATGGAGAGTTGCCTGGATCTGACATTGTGATAGGAAGTATCTTCC
SRR516986.21336065.1-	CATGGAGAGTTGCCTGGATCTGACATTGTGATAGGAGGTGTCTTCC
SRR516989.43576713.1-	CATGGAGAGTTGCCTGGATCTGACATTGTGATATGAGGTGTCTTCC
SRR511912.35663923.2+	ATGGAGAGTTGCCTGGATCTGACATTGTGATAGGAAGTATCTTCC
SRR516967.57599839.1+	ATGGAGAGTTGCCTGGATCTGACATTGTGATAGGAAGTGTCTTCC
SRR516967.66315071.2-	ATGGAGAGTTGCCTGGATCTGACATTGTGATAGGAAGTGTCTTCC
SRR516969.10517236.1+	ATGGAGAGTTGCCTGGATCTGACATTGTGATAGGAGGTGTCTTCC
SRR516981.80205710.2-	ATGGAGAGTTGCCTGGATCTGACATTGTGATAGGAAGTGTCTTCC
SRR516983.7594697.1-	ATGGAGAGTTGCCTGGATCTGACATTGTGATAGGAAGTGTCTTCC
SRR516986.54469150.2-	ATGGAGAGTTGCCTGGATCTGACATTGTGATAGGAAGTGTCTTCC
SRR516996.47351341.1-	ATGGAGAGTTGCCTGGATCTGACATTGTGATAGGAAGTGTCTTCC
SRR517000.74370214.2-	ATGGAGAGTTGCCTGGATCTGACATTGTGATAGGAAGTGTCTTCC
SRR517009.11783183.2-	AGGGAGAGTTGCCTGAATCTGACATTGTGATAGGAGGTGTCTTCC
SRR517009.22775022.2-	ATGGAGAGTTGCCTGGATCTGACATTGTGATAGGAAGTGTCTTCC
SRR517009.27203532.2+	ATGGAGAGTTGCCTGGATCTGACATTGTGATAGGAGGTGTCTTCC
SRR516984.30238767.2+	TGGAGAGTTGCCTGGATCTGACATTGTGATAGGAGGTGTCTTCC
SRR516988.44933888.1-	TGGAGAGTTGCCTGGATCTGACATTGTGATAGGAGGTGTCTTCC
SRR516996.97571998.1+	TGGAGAGTTGCCTGGATCTGACATTGTGATAGGAAGTGTCTTCC
SRR517003.82651983.2-	TGGAGAGTTGCCTGGATCTGACATTGTGATAGGAAGTATCTTCC
SRR516976.39734428.1+	GGAGAGTTGCCTGGATCTGACATTGTGATAGGAAGTGTCTTCC
SRR516977.61637699.1-	GGAGAGTTGCCTGGATCTGACATTGTGATAGGAGGTGTCTTCC
SRR516978.523901.1-	GGAGAGTTGCCTGGATCTGACATTGTGATAGGAGGTGTCTTCC
SRR516981.76700255.2-	GGAGAGTTGCCTGGATCTGACATTGTGATAGGAAGTATCTTCC
SRR516992.9778136.2+	GGAGAGTTGCCTGGATCTGACATTGTGATAGGAAGTATCTTCC
SRR516999.82809641.2-	GGAGAGTTGCCTGGATCTGACATGGTGATAGGAAGTATCTTCC
SRR511911.35430750.2+	GAGAGTTGCCTGGATCTGACATTGTGATAGGAAGTATCTTCC
SRR511913.42551667.2-	GAGAGTGGCCTGGATCTGACATTGTGATAGGAAGTATCTTCC
SRR516994.3340112.1+	GAGAGTTGCCTGGATCTGACATTGTGATAGGAGGTGTCTTCC
SRR517000.90442651.1-	GAGAGTTGCCTGGATCTGACATTGTGATAGGAAGTGTCTTCC
SRR517007.3357101.2+	GAGAGTTGCCTGGATCTGACATTGTGATAAGAAGTATCTTCC
SRR511913.32404807.2-	AGAGTTGCCTGGATCTGACATTGTGATAGGAAGTATCTTCC
SRR516967.22260303.2-	AGAGTTGCCTGGATCTGACATTGTGATAGGAAGTATCTTCC
SRR516967.30937619.2-	AGAGTTGCCTGGATCTGACATTGTGATAGGAAGTATCTTCC
SRR516977.15021422.2+	AGAGTTGCCTGGATCTGACATTGTGATAGGAGGTGTCTTCC
SRR516978.7420145.2-	AGAGTTGCCGGGATCTGACATTGTGATAGGAGGTGTCTTCC
SRR517002.57521468.1-	AGAGTTGCCTGGATCTGACATTGTGATAGGAAGTATCTTCC
SRR517004.13346376.1-	AGAGTTGCCTGGATCTGACATTGTGATAGGAGGTGTCTTCC
SRR517004.35928028.2+	AGAGTTGCCTGGATCTGACATTGTGATAGGAGGTGTCTTCC
SRR511892.23600637.1-	GAGTTGCCTGGATCTGACATTGTGATAGGAAGTATCTTCC
SRR511911.19148709.1-	GAGTTGCCTGGATCTGACATTGTGATAGGAAGTATCTTCC
SRR511913.34197254.1+	GAGTTGCCTGGATCTGACATTGTGATAGGAAGTATCTTCC
SRR511914.15089362.1+	GAGTTGCCTGGATCTGACATTGTGATAGGAAGTATCTTCC
SRR511914.3955766.2+	GAGTTGCCTGGATCTGACATTGTGATAGGAAGTATCTTCC
SRR516974.1788736.2-	GAGTTGCCTGGATCTGACATTGTGATAGGAAGTATCTTCC
SRR516974.37498085.1-	GAGTTGCCTGGATCTGACATTGTGATAGGAAGTGTCTTCC
SRR516977.16954708.1+	GAGTTGCCTGGATCTGACATTGTGATAGGAGGTGTCTTCC
SRR516977.65345009.2+	GAGTTGCCTGGATCTGACATTGTGATAGGAGGTGTCTTCC
SRR511916.25522842.2-	AGTTGCCTGGATCTGACATTGTGATAGGAAGTATCTTCC
SRR516967.106315159.1+	AGTTGCCTGGATCTGACATTGTGATAGGAAGTATCTTCC
SRR516974.20683156.2+	AGTTGCCTGGATCTGACATTGTGATAGGAAGTATCTTCC
SRR516980.47721432.1+	AGTTGCCTGGATCTGACATTGTGATAGGAGGTGTCTTCC
SRR516981.57582697.1+	AGTTGCCTGGATCTGACATTGTGATAGGAAGTGTCTTCC
SRR516984.72200753.2+	AGTTGCCTGGATCTGACATTGTGATAGGAAGTATCTTCC
SRR516984.81600199.2+	AGTTGCCTGGATCTGACATTGTGATAGGAAGTATCTTCC
SRR516986.55336979.1+	AGTTGCCTGGATCTGACATTGTGATAGGAAGTGTCTTCC
SRR516994.9392109.1-	AGTTGCCTGGATCTGACATTGTGATAGGAGGTGTCTTCC
SRR511916.16741319.1-	GTTGCCTGGATCTGACATTGTGATAGGAAGTATCTTCC
SRR511916.60079524.1-	GTTGCCTGGATCTGACATTGTGATAGGAAGTATCTTCC
SRR516979.58152913.1-	GTTGCCTGGATCTGACATTGTGATAGGAGGTGTCTTCC
SRR516986.15321637.2-	GTTGCCTGTATCTGACATTGTGATAGGAAGTGTCTTCC
SRR516986.31225117.1-	GTTGCCTGGATCTGACATTGTGATAGGAAGTGTCTTCC
SRR511912.77664739.2+	TTGCCTGGATCTGACATTGTGATAGGAAGTATCTTCC
SRR516972.37740896.2-	TTGCCTGGATCTGACATTGTGATAGGAAGTGTCTTCC
SRR516979.23680141.2-	TTGCCTGCATCTGACATTGTGATAGGAAGTGTCTTCC
SRR517002.34258022.2-	TTGCCTGGATCGGACATTGTGATAGGAAGTATCTTCC
SRR511911.1070600.1-	TGCCTGGATCTGACATTGTGATAGGAAGTATCTTCC
SRR511912.34952624.2-	TGCCTGCATCTGACATTGTGATAGGAAGTATCTTCC
SRR511913.61157542.2+	TGCCTGGATCTGACATTGTGATAGGAAGTATCTTCC
SRR516978.59370985.2-	TGCCTGGATCTGACATTGTGATAGGAGGTGTCTTCC
SRR516980.16802146.2-	TGCCTGGAGCTGACATTGTGATAGGAGGTGTCTTCC
SRR516996.75219075.1+	TGCCTGGATCTGACATTGTGATAGGAAGTGTCTTCC
SRR517004.51835356.2-	TGCCTGGATCTGACATTGTGATAGGAGGTGTCTTCC
SRR517004.60346245.1+	TGCCTGGATCTGACATTGTGATAGGAGGTGTCTTCC
SRR511892.9368196.2-	GCCTGGATCTGACATTGTGATAGGAAGTATCTTCC
SRR511911.3389924.1-	GCCTGGATCTGACATTGTGATAGGAAGTATCTTCC

SRR516980.63481299.1+	GCCTGGATCTGACATTGTGATAGGAGGTGTCTTCC
SRR516990.19507673.1-	GCCTGGATCTGACATTGTGATAGGAAGTGTCTTCC
SRR516996.81352967.2-	GCCTGGATCTGACATTGTGATAGGAAGTGTCTTCC
SRR511916.5782551.2-	CCTGGATCTGACATTGTGATAGGAAGTATCTTCC
SRR516967.40446194.2-	CCTGGATCTGACATTGTGATAGGAAGTATCTTCC
SRR516975.14442724.1+	CCTGCATCTGACATTGTGATAGGAAGTGTCTTCC
SRR516978.79924183.1-	CCTGGATCTGACATTGTGATAGGAGGTGTCTTCC
SRR516983.62802650.1-	CCTGGATCTGACATTGTGATAGGAAGTATCTTCC
SRR516993.60285752.2-	CCTGGATCTGACATTGTGATAGGAAGTGTCTTCC
SRR516993.89918039.2+	CCTGGATCTGACATTGTGATAGGAAGTGTCTTCC
SRR516995.36618898.1-	CCTGGATCTGACATTGTGATAGGAAGTGTCTTCC
SRR517002.11367864.1+	CCTGGATCTGACATTGTGATAGGAAGTATCTTCC
SRR517006.18548220.1+	CCTGGATCTGACATTGTGATAGGAAGTGTCTTCC
SRR511911.35615293.2+	CTGGATCTGACATTGTGATAGGAAGTATCTTCC
SRR516974.26972320.1-	CTGGATCTGACATTGTGATAGGAAGTATCTTCC
SRR516984.13685120.1-	CTGGATCTGACATTGTGATAGGAAGTATCTTCC
SRR516984.40512752.1-	CTGGATCTGACATTGTGATAGGAGGTGTCTTCC
SRR516986.7146752.1+	CTGGATCTGACATTGTGATAGGAGGTGTCTTCC
SRR516991.54568772.2-	CTGCATCTGACATTGTGATAGGAAGTGTCTTCC
SRR516995.44919303.2-	CTGGATCTGACATTGTGATAGGAGGTGTCTTCC
SRR517001.35552097.2-	CTGGATCTGACATTGTGATAGGAGGTGTCTTCC
SRR517001.67367146.1-	CTGGATCTGACATTGTGATAGGAGGTGTCTTCC
SRR517001.88126405.2-	CTGGATCTGACATTGTGATAGGAGGTGTCTTCC
SRR517001.96180514.1-	CTGGATCTGACATTGTGATAGGAGGTGTCTTCC
SRR511911.2865577.1-	TGGATCTGACATTGTGATAGGAAGTATCTTCC
SRR511911.54079531.1-	TGGACCTGACATTGTGATAGGAAGTATCTTCC
SRR511918.18417571.2-	TGGATCTGACATTGTGATAGGAAGTATCTTCC
SRR516967.2436532.2-	TGGATCTGACATTGTGATAGGAAGTGTCTTCC
SRR516977.71785662.2-	TGGATCTGACATTGTGATAGGAGGTGTCTTCC
SRR516978.71851870.2-	TGGATCTGACATTGTGATAGGAGGTGTCTTCC
SRR516990.55435138.2+	TGGATCTGACATTGTGATAGGAAGTGTCTTCC
SRR516990.60833908.1+	TGGATCTGACATTGTGATAGGAAGTGTCTTCC
SRR516995.92484603.2-	TGGATCTGACATTGTGATAGGAAGTGTCTTCC
SRR517007.3795099.1+	TGGATCTGACATTGTGATAGGAAGTATCTTCC
SRR517008.71700240.2-	TGGATCTGACATTGTGATAGGAGGTGTCTTCC
SRR511915.32879460.2+	GGATCTGACATTGTGATAGGAAGTATCTTCC
SRR511915.32879604.2+	GGATCTGACATTGTGATAGGAAGTATCTTCC
SRR511916.56327930.1+	GGATCTGACATTGTGATAGGAAGTATCTTCC
SRR511917.40254043.1+	GGATCTGACATTGTGATAGGAAGTATCTTCC
SRR516972.7549648.2+	GGATCTGACATTGTGATAGGAAGTGTCTTCC
SRR516977.18124717.2-	GGATCTGACATTGTGATAGGAGGTGTCTTCC
SRR516981.34060961.1+	GGATCTGACATTGTGATAGGAAGTGTCTTCC
SRR517000.14089995.1-	GGATCTGACATTGTGATAGGAAGTGTCTTCC
SRR517006.28401128.1-	GGATCTGACATTGTGATAGGAAGTGTCTTCC
SRR517000.2083309.2+	GATCTGACATTGTGATAGGAGGTGTCTTCC
SRR517003.37904939.2+	GATCTGACATTGTGATAGGAAGTATCTTCC
SRR517005.12336995.1+	GATCTGACATTGTGATAGGAAGTATCTTCC
SRR516973.51524397.1+	ATCTGACATTGTGATAGGAAGTGTCTTCC
SRR517000.43080184.1+	ATCTGACATTGTGATAGGAAGTGTCTTCC
SRR517001.89746771.1-	ATCTGACATTGTGATAGGAGGTGTCTTCC
SRR517006.17097416.1-	ATCTGTCATTGTGATAGGAATTGTCTTCC
SRR511918.17146333.2+	TCTGACATTGTGATAGGAAGTATCTTCC
SRR516976.66377853.2+	TCTGACATTGTGATCGGAAGTGTCTTCC
SRR516987.57947905.1-	TCTGACATTGTGATAGGAAGTATCTTCC
SRR516993.22248191.1-	TCTGACATTGTGATAGGAAGTGTCTTCC
SRR516995.39713292.2+	TCTGACATTGTGATAGGAGGTGTCTTCC
SRR517003.46003189.2-	TCTGACATTGTGATAGGAAGTATCTTCC
SRR516978.31371452.1+	CTGACATTGTGATAGGAGGTGTCTTCC
SRR516982.46727106.1-	CTGACATTGTGATAGGAAGTGTCTTCC
SRR516985.36645915.1+	CTGACATTGTGATAGGAAGTGTCTTCC
SRR516987.24003748.2+	CTGACATTGTGATAGGAAGTATCTTCC
SRR516994.66993668.2-	CTGACATTGTGATAGGAGGTGTCTTCC
SRR516999.28114376.1-	CTGACATTGTGATAGGAAGTATCTTCC
SRR517005.76976432.1-	CTGACATTGTGATAGGAAGTATCTTCC
SRR511916.35863368.2-	TGACATTGTGATAGGAAGTATCTTCC
SRR516980.40326797.2-	TGACATTGTGATAGGAGGTGTCTTCC
SRR516983.50031803.1+	TGACATTGTGATAGGAAGTATCTTCC
SRR516983.77412505.1-	TGACATTGTGATAGGAAGTATCTTCC
SRR516985.68798507.2+	TGACATTGTGATAGGAAGTGTCTTCC
SRR516997.94233769.2+	TGACATTGTGATAGGAAGTGTCTTCC
SRR517006.16403245.2+	TGACATTGTGATAGGAAGTGTCTTCC
SRR517007.52883726.1-	TGACATTGTGATAGAAAGTATCTTCC
SRR517008.42787980.2+	TGACATTGTGATAGGAGGTGTCTTCC
SRR517009.60480305.2+	TGACATTGTGATAGGAGGTGTCTTCC
SRR511892.54818066.2-	GACATTGTGATAGGAAGTATCTTCC
SRR511914.30434808.1-	GACATTGTGATAGGAAGTATCTTCC
SRR516973.18772931.2-	GACATTGTGATAGGAAGTGTCTTCC
SRR516978.33666848.2+	GACATTGTGATAGGAGGTGTCTTCC
SRR516980.3868284.2-	GACATGGTGATAGGAGGTGTCTTCC
SRR516980.775983.2-	GACATTGTGATAGGAGGTGTCTTCC
SRR516988.39872613.2+	GACATTGTGATAGGAGGTGTCTTCC
SRR517003.46003185.2-	GACATTGTGATAGGAAGTATCTTCC
SRR516967.71626809.1-	ACATTGTGATAGGAAGTGTCTTCC
SRR516974.22531848.2-	ACATTGTGATAGGAAGTATCTTCC

SRR516978.36274242.1+	ACATTGTGATAGGAGGTGTCTTCC
SRR516998.59445481.2-	ACATTGTGATAGGAGGTGTCTTCC
SRR516998.60968600.2-	ACATTGTGATAGGAGGTGTCTTCC
SRR516998.70570011.2-	ACATTGTGATAGGAGGTGTCTTCC
SRR516998.96733754.2-	ACATTGTGATAGGAGGTGTCTTCC
SRR517001.103363219.1+	ACATTGTGATAGGAGGTGTCTTCC
SRR517001.36815698.1+	ACATTGTGATAGGAGGTGTCTTCC
SRR517004.29851537.1+	ACATTGTGATAGGAGGTGTCTTCC
SRR517004.35999263.2-	ACATTGTGATAGGAGGTGTCTTCC
SRR517004.54443238.1+	ACATTGTGATAGGAGGTGTCTTCC
SRR517008.23794481.2-	ACATTGTGATAGGAGGTGTCTTCC
SRR517008.4270466.1-	ACATTGTGATAGGAGGTGTCTTCC
SRR516968.81828792.1-	CATTGTGATAGGAGGTGTCTTCC
SRR516990.60778082.1-	CATTGTGATAGGAAGTGTCTTCC
SRR516995.11496028.1-	CATTGTGATAGGAAGTGTCTTCC
SRR516995.88022888.2+	CATTGTGATAGGAGGTGTCTTCC
SRR516999.25368915.2+	CATTGTGATAGGAAGTATCTTCC
SRR517000.94848556.1-	CATTGTGATAGGAAGTGTCTTCC
SRR517001.52204355.2+	CATTGTGATAGGAGGTGTCTTCC
SRR511892.12533835.2-	ATTGTGATAGGAAGTATCTTCC
SRR511915.20594063.1+	ATTGTGATAGGAAGTATCTTCC
SRR516967.14759904.1-	ATTGTGATAGGAAGTATCTTCC
SRR516995.53414570.2+	ATTGTGATAGGAGGGGTCTTCC
SRR517000.38379134.1-	ATTGTGATAGGAAGTGTCTTCC
SRR517002.91348480.2-	ATTGTGATAGGAAGTATCTTCC
SRR511915.8460498.1-	TTGTGATAGGAAGTATCTTCC
SRR511915.8460499.1-	TTGTGATAGGAAGTATCTTCC
SRR516980.25761470.2-	TTGTGATAGGAGGTGTCTTCC
SRR516985.41342798.1-	TTGTGATAGGAAGTGTCTTCC
SRR516986.37734465.1-	TTGTGATAGGAAGTGTCTTCC
SRR516987.20421578.2-	TTGTGATAGGAAGTATCTTCC
SRR516996.81858289.1+	TTGTGATAGGAAGTGTCTTCC
SRR517006.68098848.2-	TTGTGATAGGAAGTGTCTTCC
SRR511892.42669582.1-	TGTGATAGGAAGTATCTTCC
SRR511911.25507918.1+	TGTGATAGGAAGTATCTTCC
SRR511914.45205869.1+	TGTGATAGGAAGTATCTTCC
SRR516970.4586121.1+	TGTGATAGGAGGTGTCTTCC
SRR516974.52405691.1+	TGTGATAGGAAGTATCTTCC
SRR516976.17282199.1-	TGTGATAGGAAGTGTCTTCC
SRR516978.34278916.1+	TGTGATAGGAGGTGTCTTCC
SRR516988.15095747.2+	TGTGATAGGAGGTGTCTTCC
SRR516988.2226181.1+	TGTGATAGGAGGTGTCTTCC
SRR516996.44208108.1+	TGTGATAGGAAGTGTCTTCC
SRR516998.28329965.2+	TGTGATAGGAAGTGTCTTCC
SRR516998.72828587.1+	TGTGATAGGAAGTGTCTTCC
SRR516998.98321200.2-	TGTGATAGGAGGTGTCTTCC
SRR516999.51370430.2+	TGTGATAGGAAGTGTCTTCC
SRR517001.74558392.2-	TGTGATAGGAGGTGTCTGCC
SRR517003.49139834.1+	TGTGATAGGAAGTATCTTCC
SRR511892.65847545.1+	GTGATAGGAAGTATCTTCC
SRR511918.33642367.2+	GTGATAGGAAGTATCTTCC
SRR516967.41713939.2+	GTGATAGGAAGTATCTTCC
SRR516968.42660308.1-	GTGATATGAGGTGTCTTCC
SRR516979.2283610.2+	GTGATAGGAAGTGTCTTCC
SRR516985.52849243.1-	GTGATAGGAAGTGTCTTCC
SRR516985.88024111.2-	GTGATAGGAAGTGTCTTCC
SRR516986.5238698.2+	GTGATAGGAAGTGTCTTCC
SRR516987.32634383.2+	GTGATAGGAAGTATCTTCC
SRR516990.10179653.1+	GTGATAGGAAGTGTCTTCC
SRR517000.17971450.2+	GTGATAGGAAGTGTCTTCC
SRR517009.81050649.2-	GTGATAGGAGGTGTCTTCC
SRR516995.18825621.1+	TGATAGGAAGTGTCTTCC
SRR517002.60341884.1+	TGATAGGAAGTATCTTCC
SRR516967.10781394.1+	GATAGGAAGTGTCTTCC
SRR516969.5105625.2+	GATAGGAGGTGTCTTCC
SRR516970.4123582.2+	GATAGGAGGCGTCTTCC
SRR516986.41476073.2-	GATAGGAGGTGTCTTCC
SRR516992.73689497.1+	GATAGGAAGTATCTTCC
SRR516995.51804784.1-	GATAGGAAGTGTCTTCC
SRR517003.37121286.2-	GATAGGAAGTATCTTCC
SRR517005.22087228.1-	GATAGGAAGTATCTTCC
SRR517009.64828053.1+	GATAGGAAGTGTCTTCC
SRR516967.124110835.1+	ATAGGAAGTATCTTCC
SRR516974.22378986.1-	ATAGGAAGTGTCTTCC
SRR516981.2688314.1+	ATAGGAAGTGTCTTCC
SRR516991.84042397.1+	ATAGGAAGTGTCTTCC
SRR516995.21670383.2+	ATAGGAAGTGTCTTCC
SRR517003.33278135.2+	ATAGGAGGTGTCTTCC
SRR517008.1945327.2+	ATAGGAGGTGTCTTCC
SRR511913.3134553.2-	TAGGAAGTATCTTCC
SRR516978.57947464.1+	TAGGAGGTGTCTTCC
SRR516988.35162539.2+	TAGGAGGTGTCTTCC
SRR516989.41185156.2+	TATGAGGTGTCTTCC
SRR516989.70274568.1-	TATGAGGTGTCTTCC

SRR516995.69712712.2+	TAGGAAGTGTCTTCC
SRR517000.105547603.2+	TAGGAGGTGTCTTCC
SRR511892.4161180.2-	AGGAAGTATCTTCC
SRR516970.31765562.2-	AGGAGGTGTCTTCC
SRR516975.1707741.1+	AGGAAGTATCTTCC
SRR516984.49729110.2-	AGGAGTTGTCTTCC
SRR517000.28657256.2-	AGGAAGTGTCTTCC
SRR517000.62667511.1+	AGGAGGTGTCTTCC
SRR517006.63297513.1+	AGGAAGTGTCTTCC
SRR511892.6619238.2-	GGAAGTATCTTCC
SRR511911.24730437.1-	GGAAGTATCTTCC
SRR516995.68978295.1+	GGAGGTGTCTTCC
SRR516968.78910843.2+	GAGGTGTCTTCC
SRR516991.17316272.1-	GAAGTGTCTTCC
SRR516999.81142078.2-	GAAGTATCTTCC
SRR517002.13637222.1-	GAAGTATCTTCC
SRR517006.40089046.1-	GAAGTGTCTTCC
SRR517008.3800488.2-	GAGGTGTCTTCC
SRR516968.56762513.1+	AGGTGTCTTCC
SRR516985.53600329.1+	AAGTGTCTTCC
SRR517000.40713109.1-	AGGTGTCTTCC
SRR517005.5761837.2+	AAGTATCTTCC
SRR517009.20742062.1-	AAGTGTCTTCC
SRR516980.2509159.1-	GGTGTCTTCC
SRR516997.49291908.1-	AGTATCTTCC
SRR517009.69208174.1-	AGTGTCTTCC
SRR516978.47252201.2-	TTGTCTGCC
SRR516990.14450506.1-	GTGTCGTCC
SRR516992.47247048.2-	GTGTCTTCC
SRR516995.37587733.2-	GTGTCTTCC
SRR516996.75812492.2+	GTGTCTTCC
SRR517001.43868988.1-	GTGTCTTCC
SRR517005.63490107.1-	GTGTCTTCC
SRR517007.28898075.1-	GTATCTGCC
SRR517007.60677870.2-	GTATCTTCC
SRR517007.65469916.1-	GTATCTTCC
SRR511914.36821194.2+	TATCTTCC
SRR516986.49636629.1-	TGTCTTCC
SRR516986.64170753.2+	TGTCTTCC
SRR516988.3021865.2-	TGTCTTCC
SRR516994.55351595.2-	TGTCTTCC
SRR516999.69510462.1+	TGTCTTCC
SRR517003.35492282.1+	TGTCTTCC
SRR516967.23066803.2-	GTCTTCC
SRR516970.28438184.1-	GTCTTCC
SRR516971.3527363.1-	GTCTTCC
SRR516984.91805446.1-	ATCTTCC
SRR516986.3185698.1+	GTCTTCC
SRR516986.43777664.2-	GTCTTCC
SRR516989.32421189.1-	GTCTTCC
SRR516995.64633284.1+	GTCTTCC
SRR517004.1229485.2+	GTCTTCC
SRR517009.8825375.1+	GTCTTCC
SRR511913.35271843.2-	TCTGTC
SRR511914.72910108.2-	TCTTCC
SRR516973.52438644.1+	TCTTCC
SRR516978.12424990.1+	TCTTCC
SRR516988.12631476.2-	TCTTCC
SRR516993.88041825.1-	TCTTCC
SRR516994.84145190.1-	TCTTCC
SRR516998.10352572.2+	TCTTCC
SRR517002.3322034.1-	TCTTCC
SRR517007.27209892.1-	TCTTCC
SRR517008.38108470.2+	TCTTCC
SRR511917.2258772.2-	CTTCC
SRR516972.79902884.2+	CTTCC
SRR516974.7795744.1+	CTTCC
SRR516994.26366517.1-	CTTCC
SRR516994.26789295.2-	CTTCC
SRR516997.74953572.2+	CTTCC
SRR517008.80424603.2-	CTTCC
SRR516967.135591639.2-	TTCC
SRR516967.57741754.2-	TTCC
SRR516968.78882370.2+	TTCC
SRR516981.5028621.1-	TTCC
SRR516989.52805078.2+	TTCC
SRR516992.55128523.1+	TTCC
SRR516998.62511336.1-	TTCC
SRR516999.12781921.1+	TTCC
SRR517006.48465992.1+	TTCC
SRR517009.45728121.1+	TTCC
SRR511911.20033258.1-	TCC
SRR516972.33835747.2+	TCC
SRR516974.68991541.2+	TCC

```
SRR516995.28796839.1-                                                             TCC
SRR516999.2638208.1-                                                              TCC
SRR517008.38355138.1-                                                             TCC
SRR517009.21340928.1+                                                             TCC
SRR511911.74696838.1-                                                              CC
SRR511915.16586476.2+                                                              CC
SRR516967.72187734.2-                                                              CC
SRR516968.39655396.1-                                                              CC
SRR516969.1886920.1+                                                               CC
SRR516971.10454062.1-                                                              CC
SRR516978.16880897.2+                                                              CC
SRR517002.33929122.2+                                                              CC
SRR516967.692820.2-                                                                 C
SRR516968.54367312.1-                                                               C
SRR516980.28746069.1-                                                               C
SRR516998.29101479.1-                                                               C
SRR516998.94356641.1+                                                               C

consensus                TTGGATTCAGCCCTCATGGAGAGTTGCCTGGATCTGACATTGTGATAGGAAGTGTCTTCC

                             .    :    .    :    .    :    .    :    .    :    .    :
SRR516967.103239374.2+   C
SRR516967.128996446.1-   C
SRR516967.62165391.1-    C
SRR516971.18513477.2-    C
SRR516994.44481978.2-    C
SRR516994.49855553.1+    C
SRR516994.77692468.2+    C
SRR517004.35890882.2+    C
SRR516977.16216859.1+    CC
SRR516977.5387374.2+     CC
SRR516986.38251531.2-    CC
SRR516996.23482257.1-    CC
SRR516998.42387076.2-    CC
SRR511892.4161180.1+     CCA
SRR511915.28490755.2-    CCA
SRR516972.30272084.2-    CCA
SRR516991.78056440.1-    CCA
SRR511913.46504761.2-    CCAA
SRR516972.83556354.1+    CCAA
SRR516981.75185944.1-    CCAA
SRR516983.72843190.1-    CCAA
SRR516984.67730810.2-    CCAA
SRR516989.82467866.2-    CCAA
SRR516991.46156534.1-    CCAA
SRR511892.54818066.1+    CCAAT
SRR516987.72954228.2-    CCAAT
SRR516994.81814726.1-    CCAAT
SRR516996.87236229.2+    CCAAT
SRR517002.88618213.1+    ACAAT
SRR517003.19490054.2-    CCAAT
SRR517008.87918252.2-    CCAAT
SRR511915.2930610.2-     CCAATG
SRR516971.28756462.1+    CCAATG
SRR516978.58338306.2+    CCAATG
SRR517000.104217922.1-   CCAATG
SRR517002.77942367.2-    CCAATG
SRR517008.65933108.2-    CCAATG
SRR511914.6591292.2+     CCCATGG
SRR516974.4422789.2+     CCAATGG
SRR516974.54553033.2+    CCAATGG
SRR516978.26037079.1+    CCCATGG
SRR516978.39183046.2+    CCAATGG
SRR516992.6010329.1-     CCAATGG
SRR516995.52974433.1+    CCAATGG
SRR511914.85321226.1+    CCAATGGC
SRR511914.85321569.1+    CCAATGGC
SRR516988.25345425.2+    CCAATGGC
SRR516995.66321276.1+    CCAATGGC
SRR517008.9761471.2+     CCAATGGC
SRR517009.35173062.1-    CCAATGGC
SRR517009.90290402.2+    CCAATGGC
SRR516969.35394539.2-    CCAATGGCA
SRR516984.88862730.1-    CCAATGGCA
SRR516989.23071515.2-    CCAATGGCA
SRR511892.42669582.2+    CCAATGGCAG
SRR511911.24730437.2+    CCAATGGCAG
SRR511912.18837352.1+    CCAATGGCAG
SRR511916.25522842.2-    CCAATGGCAG
SRR516994.52852729.1+    CCAATGGCAG
SRR511916.16741319.1-    CCAATGGCAGC
SRR511916.60079524.1-    CCAATGGCAGC
SRR516967.30072848.2+    CCAATGGCAGC
SRR516990.47204932.2+    CCAATGGCAGC
```

SRR516991.23448506.1+	CCAATGGCAGG
SRR517001.2955954.2+	CCAATGGCAGC
SRR517005.97174151.1-	CCAATGGCAGC
SRR516967.44398778.2+	CCAATGGCAGCA
SRR516986.5284326.1-	CCAATGGCAGCA
SRR516998.74633545.1+	CCAATGGCAGCA
SRR516967.140688955.2-	CCAATGGCAGCAT
SRR516980.23012010.2+	CCAATAGCAGCAT
SRR516980.51880329.2-	CCAATGGCAGCAT
SRR516990.60531713.1-	CCAATGGCAGCAT
SRR516996.92591042.1+	CCAATGGCAGCAT
SRR516998.105713546.1-	CCAATGGCAGCAT
SRR516999.51761.1+	CCAATGGCAGCAT
SRR517002.88668875.2-	CCAATGGCAGCAT
SRR517009.79220826.2-	CCAATGGCAGCAT
SRR516971.34870911.2+	CCAATGGCAGCATC
SRR516981.10390610.2-	CCAATGGCAGCATC
SRR516996.34976669.1+	CCAATGGCAGCATC
SRR511915.60393449.2-	CCAATGGCAGCATCT
SRR511916.5782551.2-	CCAATGGCAGCATCT
SRR516980.15856106.1-	CCAATGGCAGCATCT
SRR516986.47463977.1-	CCAATGGCAGCATCT
SRR516994.3610095.1+	CCAATGGCAGCATCT
SRR517006.19437322.1-	CCAATGGCAGCATCT
SRR511913.2846960.2+	CCCATGGCAGGATCTA
SRR516974.31202954.1+	CCAATGGCAGCATCTA
SRR516989.53465710.1-	CCAATGGCAGCATCTA
SRR516992.55475284.2+	CCAATGGCAGCATCTA
SRR516995.928256.2+	CCAATGGCAGCCTCTA
SRR511913.69503215.1+	CCAATGGCAGCATCTAC
SRR511918.18417571.2-	CCAATGGCAGCATCTAC
SRR516977.10847396.1+	CCAATGGCAGCCTCTAC
SRR516992.45921129.1+	CCAATGGCAGCATCTAC
SRR517003.18297409.2-	CCAATGGCAGCATCTAC
SRR517007.4303113.2+	CCAATGGCAGCATCTAC
SRR511916.56327930.1+	CCAATGGCAGCATCTACT
SRR511917.40254043.1+	CCAATGGCAGCATCTACT
SRR516996.85062640.1-	CCAATGGCAGCATCTACT
SRR516998.105584836.2-	CCAATGGCAGCATCTACT
SRR516968.163565941.2+	CCAATGGCAGCATCTACTT
SRR516983.39104642.2+	CCAATGGCAGCATCTACTT
SRR516984.43825566.1-	CCAATGGCAGCATCTACTT
SRR516984.80897040.1-	CCAATGGCAGCATCTACTT
SRR516986.62178078.2+	CCAATGGCAGCATCTACTT
SRR516993.2915184.1+	CCAATGGCAGCATCTACTT
SRR516995.56989024.1+	CCAATGGACGCATCTACTT
SRR516998.45234491.1-	CCAATGGCAGCATCTACTT
SRR517000.52358860.1+	CCAATGGCAGCATCTACTT
SRR517003.70211691.2-	CCAATGGCAGCATCTACTT
SRR516968.33193255.1+	CCAATGGCAGCATCTACTTC
SRR516974.33828349.1-	CCAATGGCAGCATCTACTTC
SRR516974.38378408.2-	CCAATGGCAGCATCTACTTC
SRR516974.49320234.1-	CCAATGGCAGCATCTACTTC
SRR516981.62187975.2-	CCAATGGCAGCATCTACTTC
SRR516981.72937619.1-	CCAATGGCAGCATCTACTTC
SRR516988.49719328.1-	CCAATGGCAGCATCTACTTC
SRR516991.91013899.2+	CCAATGGCAGCATCTACTTC
SRR517002.45348.1+	CCAATGGCAGCATCTACTTC
SRR517003.75889442.2+	TCAATGGCAGCATCTACTTC
SRR517005.58912099.2+	CCAATGGCAGCATCTACTTC
SRR517005.61088972.2+	CCAATGGCAGCATCTACTTC
SRR517008.44606589.1-	CCAATGGCAGCATCTACTTC
SRR517008.68337962.1-	CCAATGGCAGCATCTACTTC
SRR517008.69459447.1+	CCAATGGCAGCATCTACTTC
SRR511913.43839483.2+	CCAATGGCAGCATCTACTTCT
SRR511918.17146333.2+	CCAATGGCAGCATCTACTTCT
SRR516995.50404448.2-	CCAATGGCAGCATCTACTTCT
SRR516998.74008326.1+	CCAATGGCAGCATCTATTTCT
SRR516998.90024593.2-	CCAATGGCAGCATCTACTTCT
SRR511911.56187465.1+	CCAATGGCAGCATCTACTTCTC
SRR516983.29420342.1-	CCAATGGCAGCATCTACTTCTC
SRR516997.42706554.2-	CCAATGGCAGCATCTACTTCTC
SRR516999.103891959.2-	CCAATGGCAGCATCTACTTCTC
SRR516999.9724330.1-	CCAATGGCAGCATCTACTTCTC
SRR517002.98887947.1-	CCAATGGCAGCATCTACTTCTC
SRR517005.99236075.2-	CCAATGGCAGCATCTACTTCTC
SRR517007.626055.1-	CCAATGGCAGCATCTACTTCTC
SRR511914.15324684.2+	CCAATGGCAGCATCTACTTCTCC
SRR511916.35863368.2-	CCAATGGCAGCATCTACTTCTCC
SRR516981.37172809.1-	CCAATGGCAGCATCTACTTCTCC
SRR516990.33601261.1-	CCAATGGCAGCATCTACTTCTCC
SRR516995.33739366.2-	CCAATGGCAGCATCTACTTCTCC
SRR516998.65740325.1+	CCAATGGCAGCATCTACTTCTCC
SRR517000.36416800.1+	CCAATGGCAGCATCTACTTCTCC

SRR511912.43892789.1-   CCAATGGCAGCATCTACTTCTCCg
SRR516978.15412507.1+   CCAATGGCAGCATCTACTTCTCCg
SRR516992.15393409.1-   CCAATGGCAGCATCTACTTCTCCg
SRR516994.39206055.2-   CCAATGGCAGCATCTACTTCTCCg
SRR516997.97876806.2-   CCAATGGCAGCATCTACTTCTCCg
SRR516999.108053227.2-   CCAATGGCAGCATCTACTTCTCCg
SRR517002.24413856.1-   CCAATGGCAGCATCTACTTCTCCg
SRR517003.41980170.1-   CCAATGGCAGCATCTACTTCTCCg
SRR511914.15324340.2+   CCAATGGCAGCATCTACTTCTCCgt
SRR516968.21564094.2-   CCAATGGCAGCATCTACTTCTCCgt
SRR516977.12873538.2-   CCAATGGCAGCATCTACTTCTCCgt
SRR516977.80260583.2-   CCAATGGCAGCATCTACTTCTCCgt
SRR516978.43155707.2+   CCAACGGCAGCCTCTACTTCTCCgt
SRR516986.43082651.2-   CCAATGGCAGCATCTACTTCTCCgt
SRR516993.35093586.1+   CCAATGGCAGCATCTACTTCTCCgt
SRR516993.65644538.2+   CCAATGGCAGCATCTACTTCTCCgt
SRR516999.52117285.1+   CCAATGGCAGCATCTACTTCTCCgt
SRR517001.53006899.2-   CCAATGGCAGCATCTACTTCTCCgt
SRR517009.40589595.1-   CCAATGGCAGCATCTACTTCTCCgt
SRR516973.40895713.2-   CCAATGGCAGCATCTACTTCTCCgta
SRR516978.31035699.2+   CCAATGGCAGCATCTACTTCTCCgtc
SRR516995.53678391.1+   CCAATGGCAGCATCTACTTCTCCgta
SRR517001.4174199.1+   CCAATGGCAGCATCTACTTCTCCgta
SRR511911.74696838.2+   CCAATGGCAGCATCTACTTCTCCgtaa
SRR516979.52325428.1-   CCAATGGCAGCATCTACTTCTCCgtaa
SRR516981.79962474.1+   CCAATGGCAGCATCTACTTCTCCgtaa
SRR517003.61889052.2+   CCAATGGCAGCATCTACTTCTCCgtaa
SRR517009.47953341.2+   CCAATGGCAGCATCTACTTCTCCgtaa
SRR517009.59440565.2-   CCAATGGCAGCATCTACTTCTCCgtaa
SRR511912.1303277.1-   CCAATGGCAGCATCTACTTCTCCgtaag
SRR511915.30731583.2-   CCAATGGCAGCATCTACTTCTCCgtaag
SRR511915.30731584.2-   CCAATGGCAGCATCTACTTCTCCgtaag
SRR516989.74819632.1-   CCAATGGCAGCATCTACTTCTCCgtaag
SRR516999.38071679.1-   CCAATGGCAGCATCTACTTCTCCgtaag
SRR511915.35046284.2-   CCAATGGCAGCATCTACTTCTCCgtaagt
SRR516974.40379406.1-   CCAATGGCAGCATCTACTTCTCCgtaagt
SRR517005.8220247.2-   CCAATGGCAGCATCTACTTCTCCgtaagt
SRR511913.35897653.2+   CCAATGGCAGCATCTACTTCTCCgtgagtt
SRR511914.80114329.2+   CCAATGGCAGCATCTACTTCTCCgtaaggt
SRR511918.33642367.2+   CCAATGGCAGCATCTACTTCTCCgtaagtt
SRR516968.96419618.1-   CCAATGGCAGCATCTACTTCTCCgtaagtt
SRR516969.23432456.1+   CCAATGGCAGCATCTACTTCTCCgtaagtt
SRR516975.56296657.1-   CCAATGGCAGCATCTACTTCTCCgtaagtt
SRR516977.43459967.2+   CCAATGGCAGCATCTACTTCTCCgtaagtt
SRR516995.22467748.1+   CCAATGGCAGCATCTACTTCTCCgtaagtt
SRR517008.68826874.2-   CCAATGGCAGCATCTACTTCTCCgtaagtt
SRR516974.49170123.2-   CCAATGGCAGCATCTACTTCTCCgtaagttg
SRR516981.9101608.1+   CCAATGGCAGCATCTACTTCTCCgtaagttg
SRR516988.44614705.1-   CCAATGGCAGCATCTACTTCTCCgtaagttg
SRR516996.21050103.2+   CCAATGGCAGCATCTACTTCTCCgtaagttg
SRR516997.65362583.2+   CCAATGGCAGCATCTACTTCTCCgtaagttg
SRR517002.7720294.2-   CCAATGGCAGCATCTACTTCTCCgtaagttg
SRR517003.32714941.1-   CCAATGGCAGCATCTACTTCTCCgtaagttg
SRR517003.71024107.1-   CCAATGGCAGCATCTACTTCTCCgtaagttg
SRR516992.35154680.2+   CCAATGGCAGCAACTAATTCTCCgtaagttga
SRR516998.54949380.1+   CCAATGGCAGCATCTACTTCTCCgtaagttga
SRR516999.4765606.2-   CCAATGGCAGCATCTACTTCTCCgtaagttga
SRR517006.28181699.2-   CCAATGGCAGCATCTACTTCTCCgtaagttga
SRR511914.86222658.1-   CCAATGGCAGCATCTACTTCTCCgtaagttgaa
SRR516972.13628751.2+   CCAATGGCAGCATCTACTTCTCCgtaagttgaa
SRR516980.16802146.2-   CCAATGGCAGCATCTACTTCTCCgtaagttgaa
SRR516988.46830167.2-   CCAATGGTAGCATCTACTTCTCCgtaagttgaa
SRR516990.37851369.1+   CCAATGGCAGCATCTACTTCTCCgtaagttgaa
SRR516995.55575214.2-   CCAATGGCAGCATCTACTTCTCCgtaagttgaa
SRR517003.40047671.2-   CCAATGGCAGCATCTACTTCTCCgtaagttgaa
SRR516968.128087344.2-   CCAATGGCAGCATCTACTTCTCCgtaagttgaaa
SRR516977.42799382.2+   CCAATGGCAGCATCTACTTCTCCgtaagttgaaa
SRR516999.94573754.1+   CCAATGGCAGCATCTACTTCTCCgtaagttgaaa
SRR517001.30441251.2+   CCAATGGCAGCATCTAATTCTCCgtaagttgaaa
SRR516968.39167129.1+   CCAATGGCAGCATCTACTTCTCCgtaagttgaaaa
SRR516996.2184957.1+   CCAATGGCAGCATCTACTTCTCCgtaagttgaaaa
SRR516996.58664910.1-   CCAATGGCAGCATCTACTTCTCCgtaagttgaaaa
SRR517004.43077788.2+   CCAATGGCAGCATCTACTTCTCCgtaagttgaaaa
SRR517009.74371807.2-   CCAATGGCAGCATCTACTTCTCCgtaagttgaaaa
SRR516967.3860522.1-   CCAATGGCAGCATCTACTTCTCCgtaagttgaaaag
SRR516967.51922488.2-   CCAATGGCAGCATCTACTTCTCCgtaagttgaaaag
SRR516996.70382886.1-   CCAATGGCAGCATCTACTTCTCCgtaagttgaaaag
SRR517009.30109562.1-   CCAATGGCAGCATCTACTTCTCCgtaagttgaaaag
SRR516967.3323960.2-   CCAATGGCAGCATCTACTTCTCCgtaagttgaaaaga
SRR516977.29390676.2+   CCAATGGCAGCATCTACTTCTCCgtaagttgaaaaga
SRR516977.47398733.2-   CCAATGGCAGCATCTACTTCTCCgtaagttgaaaaga
SRR516977.52908323.1-   CCAATGGCAGCATCTACTTCTCCgtaagttgaaaaga
SRR516980.67663155.2-   CCAATGGCAGCATCTACTTCTCCgtaagttgaaaaga
SRR516981.43550688.1+   CCAATGGCAGCATCTACTTCTCCgtaagttgaaaaga

SRR516996.85687262.2-   CCAATGGCAGCATCTACTTCTCCgtaagttgaaaaga
SRR517000.69157843.1+   CCAATGGCAGCATCTACTTCTCCctaagttgaaaaga
SRR517000.8876587.2+   CCAATGGCAGCATCTACTTCTCCgtaagttgaaaaga
SRR511911.59626526.2+   CCAATGGCAGCAACTACTTCTCCgtcagttggaaagac
SRR516977.59451244.1-   CCAATGGCAGCATCTACTTCTCCgtaagttgaaaagac
SRR516980.7478290.1-   CCAATGGCAGCATCTACTTCTCCgtaagttgaaaagac
SRR511911.51765582.2-   CCAATGGCAGCATCTACTTCTCCgtaagttgaaaagact
SRR511915.43325895.2+   CCAATGGCAGCATCTACTTCTCCgtaaggtgaaaagaat
SRR516973.6568969.2-   CCAATGGCAGCATCTACTTCTCCgtaagttgaaaagact
SRR516987.61502473.1-   CCAATGGCAGCATCTACTTCTCCttaagttgaaaagact
SRR516997.11539523.2+   CCAATGGCAGCATCTACTTCTCCgtaagttgaaaagact
SRR517000.77997273.2-   CCAATGGCAGCATCTACTTCTCCgtaagttgaaaagact
SRR517008.80303637.1+   CCAATGGCAGCATCTACTTCTCCgtaagttgaaaagact
SRR516967.114035844.2-   CCAATGGCAGCATCTACTTCTCCgtaagttgaaaagactt
SRR516972.19207856.2+   CCAATGGCAGCATCTACTTCTCCggaagttgaaaagactt
SRR516981.7950043.2+   CCAATGGCAGGAGCTACTTCTCCgtaaattgaaaacactt
SRR516990.7184572.1+   CCAATGGCAGCATCTACTTCTCCgtaagttgaaaagactt
SRR516996.11671795.2-   CCAATGGCAGCATCTACTTCTCCgtaagttgaaaagactt
SRR516996.95894100.2-   CCAATGGCAGCATCTACTTCTCCgtaagttgaaaagactt
SRR517001.80549406.1+   CCAATGGCAGCATCTACTTCTCCgtaagttgaaaagactt
SRR511892.9511611.1-   CCAATGGCAGCATCTACTTCTCCgtaagttgaaaagacttc
SRR516968.118272353.1-   CCAATGGCAGCATCTACTTCTCCgtaagttgaaaagacttc
SRR516981.84093782.2-   CCAATGGCAGCATCTACTTCTCCgtaagttgaaaagacttc
SRR516984.12082939.1-   CCAATGGCAGCATCTACTTCTCCgtaagttgaaaagacttc
SRR517006.50484772.1+   CCAATGGCAGCATCTACTTCTCCgtaagttgaaaagacttc
SRR517009.11783183.2-   CCAATGGCAGCTTCTACTTCTCCgtaagttgaaaagacttc
SRR517009.48303164.2+   CCAATGGCAGCATCTACTTCTCCgtaagttgaaaagacttc
SRR511911.60280636.1-   CCAATGGCAGCATCTACTTCTCCgtaagttgaaaagacttcc
SRR511912.78910790.2-   CCAATGGCAGCATCTACTTCTCCgtaagttgaaaagacttcc
SRR511914.67096997.1-   CCAATGGCAGCATCTACTTCTCCgtaagttgaaaagacttcc
SRR516967.113781662.2-   CCAATGGCAGCATCTACTTCTCCgtaagttgaaaagacttcc
SRR516974.60321821.2-   CCAATGGCAGCATCTACTTCTCCgtaagttgaaaagacttcc
SRR516986.48107818.2-   CCAATGGCAGCATCTACTTCTCCgtaagttgaaaagacttcc
SRR516989.87718993.2+   CCAATGGCAGCATCTACTTCTCCgtaagttgaaaagacttcc
SRR517001.78342098.1-   CCAATGGCAGCATCTACTTCTCCgtaagttgaaaagacttcc
SRR511914.46576162.1-   CCAATGGCAGCATCTACTTCTCCgtaagttgaaaagacttcct
SRR516969.15376651.1-   CCAATGGCAGCATCTACTTCTCCgtaagttgaaaagacttcct
SRR516972.40740620.1+   CCAATGGCAGCATCTACTTCTCCgtaagttgaaaagacttcct
SRR516977.35615817.1-   CCAATGGCAGCATCTACTTCTCCgtaagttgaaaagacttcct
SRR516980.28641220.2+   CCAATGGCAGCATCTACTTCTCCgtaagttgaaaagacttcct
SRR516984.39430615.2-   CCAATGGCAGCATCTACTTCTCCgtaagttgaaaagacttcct
SRR516993.65564502.1+   CCAATGGCAGCATCTACTTCTCCgtaagttgaaaagacttcct
SRR516996.20396000.1-   CCAATGGCAGCATCTACTTCTCCgtaagttgaaaagacttcct
SRR516997.6377760.1+   CCAATGGCAGCATCTACTTCTCCgtaagttgaaaagacttcct
SRR517004.25460609.1+   CCAATGGCAGCATCTACTTCTCCgtaagttgaaaagacttcct
SRR517004.25658369.2+   CCAATGGCAGGAACTACTTCTCCgtaagttgaaaagaaatcct
SRR517008.70570764.1-   CCAATGGCAGCATCTACTTCTCCgtaagttgaaaagacttcct
SRR517009.74175557.1-   CCAATGGCAGCATCTACTTCTCCgtaagttgaaaagacttcct
SRR511917.2258772.2-   CCAATGGCAGCATCTACTTCTCCgtaagttgaaaagacttcctc
SRR516968.169513645.2+   CCAATGGCAGCATCTACTTCTCCgtaagttgaaaagacttcctc
SRR516969.27833237.2-   CCAATGGCAGCATCTACTTCTCCgtaagttgaaaagacttcctc
SRR516976.66377853.2+   CCAATGGCAGCCTCCACTTCTCCgtaagtttaaaagacttcctc
SRR516978.31082004.1+   CCAATGGCAGCATCTACTTCTCCgtaagttgaaaagacttcctc
SRR516984.29026302.1-   CCAATGGCAGCATCTACTTCTCCgtaagttgaaaagacttcctc
SRR516986.21336065.1-   CCAATGGCAGCATCTACTTCTCCgtaagttgaaaagacttcctc
SRR516989.43576713.1-   CCAATGGCAGCATCTACTTCTCCgtaagttgaaaagacttcctc
SRR516981.80205710.2-   CCAATGGCAGCATCTACTTCTCCgtaagttgaaaagacttcctct
SRR516983.7594697.1-   CCAATGGCAGCATCTACTTCTCCgtaagttgaaaagacttcctct
SRR516986.54469150.2-   CCAATGGCAGCATCTACTTCTCCgtaagttgaaaagacttcctct
SRR516996.47351341.1-   CCAATGGCAGCATCTACTTCTCCgtaagttgaaaagacttcctct
SRR517000.74370214.2-   CCAATGGCAGCATCTACTTCTCCgtaagttgaaaagacttcctct
SRR517009.22775022.2-   CCAATGGCAGCATCTACTTCTCCgtaagttgaaaagacttcctct
SRR517009.27203532.2+   CCAATGGCAGCATCTACTTCTCCgtaagttgaaaagacttcctct
SRR511892.45271449.1-   CCAATGGCAGCATCTACTTCTCCgtaagttgaaaagacttcctctg
SRR511911.58123630.2-   CCAATGGCAGCATCTACTTCTCCgtaagttgaaaagacttcctctg
SRR511911.63363441.2+   CCAATGGCAGCATCTACTTCTCCgtaagttgaaaagacttcctctg
SRR511913.39997243.2+   CCAATGGCAGCATCTACTTCTCCgtaagttgaaaagacttcctctg
SRR516968.108115361.2-   CCAATGGCAGCATCTACTTCTCCgtaagttgaaaagacttcctctg
SRR516968.53031658.2-   CCAATGGCAGCATCTACTTCTCCgtaagttgaaaagacttcctctg
SRR516984.30238767.2+   CCAATGGCAGCATCTACTTCTCCgtaagttgaaaagacttcctctg
SRR516988.44933888.1-   CCAATGGCAGCATCTACTTCTCCgtaagttgaaaagacttcctctg
SRR516996.97571998.1+   CCAATGGCAGCATCTACTTCTCCgtaagttgaaaagacttcctctg
SRR517003.82651983.2-   CCAATGGCAGCATCTACTTCTCCgtaagttgaaaagacttcctctg
SRR511911.1348761.1+   CCAATGGCAGCATCTACTTCTCCgtaagttgaaaagacttcctctgc
SRR511912.39211104.1-   CCAATGGCAGCATCTACTTCTCCgtaagttgaaaagacttcctctgc
SRR516976.39734428.1+   CCAATGGCAGCATCTACTTCTCCgtaagttgaaaagacttcctctgc
SRR516977.61637699.1-   CCAATGGCAGCATCTACTTCTCCgtaagttgaaaagacttcctctgc
SRR516978.523901.1-   CCAATGGCAGCATCTACTTCTCCgtaagttgaaaagacttcctctgc
SRR516981.76700255.2-   CCAATGGCAGCATCTACTTCTCCgtaagttgaaaagacttcctctgc
SRR516992.9778136.2+   CCAATGGCAGCCTCTACTTCTCCgtaagttgaaaagacttcctctgc
SRR516999.82809641.2-   CCAATGGCAGCATCTACTTCTCCgtaagttgaaaagacttcctctgc
SRR511911.11918542.2+   CCAATGGCAGCATCTACTTCTCCgtaagttgaaaagacttcctctgcg
SRR511911.31335408.1+   CCAATGGCAGCATCTACTTCTCCgtaagttgaaaagacttcctctgcg
SRR516994.3340112.1+   CCAATGGCAGCATCTACTTCTCCgtaaattgaaaagaattcctctgcg

SRR517000.90442651.1-   CCAATGGCAGCATCTACTTCTCCgtaagttgaaaagacttcctctgcg
SRR517007.3357101.2+   CCAATGGCAGAATCTACTTCTCCgtaagttgaaaagacttcctctgcg
SRR511913.35035416.2-   CCAATGGCAGCATCTACTTCTCCgtaagttgaaaagacttcctctgcgt
SRR516968.92422565.1+   CCAATGGCAGCATCTACTTCTCCgtaagttgaaaagacttcctctgcgt
SRR516977.15021422.2+   CCAATGGCAGCATCTACTTCTCCgtaagttgaaaagacttcctctgcgt
SRR516978.7420145.2-   CCAATGGCAGCATCTACTTCTCCgtaagttgaaaagacttcctctgcgt
SRR517002.57521468.1-   CCAATGGCAGCATCTACTTCTCCgtaagttgaaaagacttcctctgcgt
SRR517004.13346376.1-   CCAATGGCAGCATCTACTTCTCCgtaagttgaaaagacttcctctgcgt
SRR517004.35928028.2+   CCAATGGCAGCATCTACTTCTCCgtaagttgaaaagacttcctctgcgt
SRR511913.67606827.2-   CCAATGGCAGCATCTACTTCTCCgtaagttgaaaagacttcctctgcgtc
SRR516974.1788736.2-   CCAATGGCAGCATCTACTTCTCCgtaagttgaaaagacttcctctgcgtc
SRR516974.37498085.1-   CCAATGGCAGCATCTACTTCTCCgtaagttgaaaagacttcctctgcgtc
SRR516977.16954708.1+   CCAATGGCAGCATCTACTTCTCCgtaagttgaaaagacttcctctgcgtc
SRR516977.65345009.2+   CCAATGGCAGCATCTACTTCTCCgtaagttgaaaagacttcctctgcgtc
SRR516974.20683156.2+   CCAATGGCAGCATCTACTTCTCCgtaagttgaaaagacttcctctgcgtcc
SRR516980.47721432.1+   CCAATGGCAGCATCTACTTCTCCgtaagttgaaaagacttcctctgcgtcc
SRR516981.57582697.1+   CCAATGGCAGCATCTACTTCTCCgtaagttgaaaagacttcctctgcgtcc
SRR516984.72200753.2+   CCAATGGCAGCATCTACTTCTCCgtaagttgaaaagaattcctcttcgtcc
SRR516984.81600199.2+   CCAATGGCAGCATCTACTTCTCCgtaagttgaaaagacttcctctgcgtcc
SRR516986.55336979.1+   CCAATGGCAGCATCTACTTCTCCgtaagttgaaaagacttcctctgcgtcc
SRR516994.9392109.1-   CCAATGGCAGCATCTACTTCTCCgtaagttgaaaagacttcctctgcgtcc
SRR517002.91348480.2-   CCAATGGCAGCATCTACTTCCCCgtaagttgaaaagacttcctctgcgtcc
SRR511892.54323619.2+   CCAATGGCAGCATCTACTTCTCCgtaagttgaaaagacttcctctgcgtccc
SRR511912.35663923.2+   CCAATGGCAGCATCTACTTCTCCgtaagttgaaaagacttcctctgcgtccc
SRR511913.39320808.2-   CCAATGGCAGCATCTACTTCTCCgtaagttgaaaagacttcctctgcgtccc
SRR516979.58152913.1-   CCAATGGCAGCATCTACTTCTCCgtaagttgaaaagacttcctctgcgtccc
SRR516986.15321637.2-   CCAATGGCAGCATCTACTTCTCCgtaagttgaaaagacttcctctgcgtccc
SRR516986.31225117.1-   CCAATGGCAGCATCTACTTCTCCgtaagttgaaaagacttcctctgcgtccc
SRR516967.123439529.1-   CCAATGGCAGCATCTGCTTCTCCgtaaattgaaaagacttcctctgcgtccct
SRR516971.10078918.2-   CCAATGGCAGCATCTACTTCTCCgtaagttgaaaagacttcctctgcgtccct
SRR516971.3527363.1-   CCAATGGCAGCATCTACTTCTCCgtaagttgaaaagacttcctctgcgtccct
SRR516972.37740896.2-   CCAATGGCAGCATCTACTTCTCCgtaagttgaaaagacttcctctgcgtccct
SRR516978.59370985.2-   CCAATGGCAGCATCTACTTCTCCgtaagttgaaaagacttcctctgcgtccct
SRR516979.23680141.2-   CCAATGGCAGCATCTACTTCTCCgtaagttgaaaagacttcctctgcgtccct
SRR517002.34258022.2-   CCAATGGCAGCATCTACTTCTCCgtaagttgaaaagacttcctctgcgtccct
SRR516969.10517236.1+   CCAATGGCAGCATCTACTTCTCCgtaagttgaaaagacttcctctgcgtccctc
SRR516975.14442724.1+   CCAATGGCAGCATCTACTTCTCCgtaagttgaaaagacttcctctgcgtccctc
SRR516996.75219075.1+   CCAATGGCAGCATCTACTTCTCCgtaagttgaaaagacttcctctgcgtccctc
SRR517004.51835356.2-   CCAATGGCAGCATCTACTTCTCCgtaagttgaaaagacttcctctgcgtccctc
SRR517004.60346245.1+   CCAATGGCAGCATCTACTTCTCCgtaagttgaaaagacttcctctgcgtccctc
SRR516980.63481299.1+   CCAATGGCAGCATCTACTTCTCCgtaagttgaaaagacttcctctgcgtccctct
SRR516990.19507673.1-   CCAATGGCAGCATCTACTTCTCCgtaagttgaaaagacttcctctgcgtccctct
SRR516996.81352967.2-   CCAATGGCAGCATCTACTTCTCCgtaagttgaaaagacttcctctgcgtccctct
SRR516967.57599839.1+   CCAATGGCAGCATCTACTTCTCCgtaagttgaaaagacttcctctgcgtccctctt
SRR516967.66315071.2-   CCAATGGCAGCATCTACTTCTCCgtaagttgaaaagacttcntctgcgtccctctt
SRR516978.79924183.1-   CCAATGGCAGCATCTACTTCTCCgtaagttgaaaagacttcctctgcgtccctctt
SRR516983.62802650.1-   CCAATGGCAGCATCTACTTCTCCgtaagttgaaaagacttcctctgcgtccctctt
SRR516993.60285752.2-   CCAATGGCAGCATCTACTTCTCCgtaagttgaaaagacttcctctgcgtccctctt
SRR516993.89918039.2+   CCAATGGCAGCATCTACTTCTCCgtaagttgaaaagacttcctctgcgtccctctt
SRR516995.36618898.1-   CCAATGGCAGCATCTACTTCTCCgtaagttgaaaagacttcctctgcgtccctctt
SRR517002.11367864.1+   CCAATGGCAGCATCTACTTCTCCgtaagttgaaaagacttcctctgcgtccctctt
SRR517006.18548220.1+   CCAATGGCAGCATCTACTTCTCCgtaagttgaaaagacttcctctgcgtccctctt
SRR516974.26972320.1-   CCAATGGCAGCATCTACTTCTCCgtaagttgaaaagacttcctctgcgtccctcttt
SRR516984.13685120.1-   CCAATGGCAGCATCTACTTCTCCgtaagttgaaaagacttcctctgcgtccctcttt
SRR516984.40512752.1-   CCAATGGCAGCATCTACTTCTCCgtaagttgaaaagacttcctctgcgtccctcttt
SRR516986.7146752.1+   CCAATGGCAGCATCTACTTCTCCgtaagttgaaaagacttcctctgcgtccctcttt
SRR516991.54568772.2-   CCAATGGCAGCATCTACTTCTCCgtaagttgaaaagacttcctctgcgtccctcttt
SRR516995.44919303.2-   CCAATGGCAGCATCTACTTCTCCgtaagttgaaaagacttcctctgcgtccctcttt
SRR517001.35552097.2-   CCAATGGCAGCATCTACTTCTCCgtaagttgaaaagacttcctctgcgtccctcttt
SRR517001.67367146.1-   CCAATGGCAGCATCTACTTCTCCgtaagttgaaaagacttcctctgcgtccctcttt
SRR517001.88126405.2-   CCAATGGCAGCATCTACTTCTCCgtaagttgaaaagacttcctctgcgtccctcttt
SRR517001.96180514.1-   CCAATGGCAGCATCTACTTCTCCgtaagttgaaaagacttcctctgcgtccctcttt
SRR511911.35430750.2+   CCAATGGCAGCATCTACTTCTCCgtaagttgaaaagacttcctctgcgtccctctttg
SRR511913.42551667.2-   CCAATGGCAGCATCTACTTCTCCgtaagttgaaaagacttcctctgcgtccctctttg
SRR516977.71785662.2-   CCAATGGCAGCATCTACTTCTCCgtaagttgaaaagacttcctctgcgtccctctttg
SRR516978.71851870.2-   CCAATGGCAGCATCTACTTCTCCgtaagttgaaaagacttcctctgcgtccctctttg
SRR516981.34060961.1+   CCAATGGCAGCATCTAATTCTCCgtaagttgaaaagacttcctcctcgtccctctttg
SRR516990.55435138.2+   CCAATGGCAGCATCTACTTCTCCgtaagttgaaaagacttcctctgcgtccctctttg
SRR516990.60833908.1+   CCAATGGCAGCATCTACTTCTCCgtaagttgaaaagacttcctctgcgtccctctttg
SRR516995.92484603.2-   CCAATGGCAGCATCTACTTCTCCgtaagttgaaaagacttcctctgcgtccctctttg
SRR517006.28401128.1-   CCAATGGCAGCATCTACTTCTCCgtaagttgaaaagacttcctctgcgtccctctttg
SRR517007.3795099.1+   CCAATGGCAGCATCTACTTCTCCgtaagttgaaaagacttcctctgcgtccctctttg
SRR517008.71700240.2-   CCAATGGCAGCATCTACTTCTCCgtaagttgaaaagacttcctctgcgtccctctttg
SRR511913.32404807.2-   CCAATGGCAGCATCTACTTCTCCgtaagttgaaaagacttcctctgcgtccctctttgc
SRR516972.7549648.2+   CCAATGGCAGCATCTACTTCTCCgtaaattgaaaagacttcctctgcgtccctctttgc
SRR516977.18124717.2-   CCAATGGCAGCATCTACTTCTCCgtaagttgaaaagacttcctctgcgtccctctttgc
SRR517000.14089995.1-   CCAATGGCAGCATCTACTTCTCCgtaagttgaaaagacttcctctgcgtccctctttgc
SRR511892.12533835.2-   CCAATGGCAGCATCTACTTCTCCgtaagttgaaaagacttcctctgcgtccctctttgcc
SRR511892.23600637.1-   CCAATGGCAGCATCTACTTCTCCgtaagttgaaaagacttcctctgcgtccctctttgcc
SRR511892.4161180.2-   CCAATGGCAGCATCTACTTCTCCgtaagttgaaaagacttcctctgcgtccctctttgcc
SRR511892.42669582.1-   CCAATGGCAGCATCTACTTCTCCgtaagttgaaaagacttcctctgcgtccctctttgcc
SRR511892.54818066.2-   CCAATGGCAGCATCTACTTCTCCgtaagttgaaaagacttcctctgcgtccctctttgcc
SRR511892.65847545.1+   CCAATGGCAGCATCTACTTCTCCgtaagttgaaaagacttcctctgcgtccctctttgcc
SRR511892.6619238.2-   CCAATGGCAGCATCTACTTCTCCgtaagttgaaaagacttcctctgcgtccctctttgcc

SRR511892.9368196.2-   CCAATGGCAGCATCTACTTCTCCgtaagttgaaaagacttcctctgcgtccctctttgcc
SRR511911.1070600.1-   CCAATGGCAGCATCTACTTCTCCgtaagttgaaaagacttcctctgcgtccctctttgcc
SRR511911.19148709.1-   CCAATGGCAGCATCTACTTCTCCgtaagttgaaaagacttcctctgcgtccctctttgcc
SRR511911.20033258.1-   CCAATGGCAGCATCTACTTCTCCgtacgttgaaaagccttcctctgcgtccctctttgcc
SRR511911.24730437.1-   CCAATGGCAGCATCTACTTCTCCgtaagttgaaaagacttcctctgcgtccctctttgcc
SRR511911.25507918.1+   CCAATGGCAGCATCTACTTCTCCgtaagttgaaaagacttcctctgcgtccctctttgcc
SRR511911.2753996.1+   CCAATGGCAGCATCTACTTCTCCgtaagttgaaaagacttcctctgcgtccctctttgcc
SRR511911.2865577.1-   CCAATGGCAGCATCTACTTCTCCgtaagttgaaaagacttcctctgcgtccctctttgcc
SRR511911.3389924.1-   CCAATGGCAGCATCTACTTCTCCgtaagttgaaaagacttcctctgcgtccctctttgcc
SRR511911.35615293.2+   CCAATGGCAGCATCTACTTCTCCgtaagttgaaaagacttcctctgcgtccctctttgcc
SRR511911.54079531.1-   CCAATGGCAGCATCTACTTCTCCgtaagttgaaaagacttcctctgcgtccctctttgcc
SRR511911.56187465.2-   CCAATGGCAGCATCTACTTCTCCgtaagttgaaaagacttcctctgcgtccctctttgcc
SRR511911.74696838.1-   CCAATGGCAGCATCTACTTCTCCgtaagttgaaaagacttcctctgcgtccctctttgcc
SRR511912.34952624.2-   CCAATGGCAGCATCTACTTCTCCgtaagttgaaaagacttcctctgcgtccctctttgcc
SRR511912.77664739.2+   CCAATGGCAGCATCTACTTCTCCgtaagttgaaaagacttcctctgcgtcccccttttcc
SRR511913.3134553.2-   CCAATGGCAGCATCTACTTCTCCgtaagttgaaaagacttcctctgcgtccctctttgcc
SRR511913.34197254.1+   CCAATGGCAGCATCTACTTCTCCgtaagttgaaaagacttcctctgcgtccctctttgcc
SRR511913.35271843.2-   CCAAAGGCAGCATCTACTTCACCgtaagttgaaaagacttcctctgcgtccctctttgcc
SRR511913.61157542.2+   CCAATGGCAGCATCTACTTCTCCgtaagttgaaaagacttcccctgcgtccccctttgcc
SRR511914.15089362.1+   CCAATGGCAGCATCTACTTCTCCgtaagttgaaaagacttcctctgcgtccctctttgcc
SRR511914.30434808.1-   CCAATGGCAGCATCTACTTCTCCgtaagttgaaaagacttcctctgcgtccctctttgcc
SRR511914.36821194.2+   CCAATGGCAGCATCTACTTCTCCgtaagttgaaaagacttcctctgcgtccctctttgcc
SRR511914.3955766.2+   CCAATGGCAGCATCTAATTCTCCgtaagttgaaaagactttctttgcgtccctctttgcc
SRR511914.45205869.1+   CCAATGGCAGCATCTACTTCTCCgtaagttgaaaagacttcctctgcgtccctctttgcc
SRR511914.72910108.2-   CCAATGGCAGCATCTACTTCTCCgtaagttgaaaagacttcctctgcgtccctctttgcc
SRR511915.16586476.2+   CCAATGGCAGCATCTACTTCTCCgtaagttgaaaagacttcctctgcgtccctctttgcc
SRR511915.20594063.1+   CCAATGGCAGCATCTACTTCTCCgtaacttgaaaagacttcctctgcgtccctctttgcc
SRR511915.32879460.2+   CCAATGGCAGCATCTACTTCTCCgtaagttgaaaagacttcctctgcgtccctctttgcc
SRR511915.32879604.2+   CCAATGGCAGCATCTACTTCTCCgtaagttgaaaagacttcctctgcgtccctctttgcc
SRR511915.8460498.1-   CCAATGGCAGCATCTACTTCTCCgtaagttgaaaagacttcctctgcgtccctctttgcc
SRR511915.8460499.1-   CCAATGGCAGCATCTACTTCTCCgtaagttgaaaagacttcctctgcgtccctctttgcc
SRR516967.106315159.1+   CCAATGGCAGCATCTACTTCTCCgtaagttgaaaagacttcctctgcgtccctctttgcc
SRR516967.10781394.1+   CCAATGGCAGCATCTACTTCTCCgtaagttgaaaagacttcctctgcgtccctctttgcc
SRR516967.124110835.1+   CCAATGGCAGCATCTACTTCTCCgtaagttgaaaagacttcctctgcgtccctctttgcc
SRR516967.135591639.2-   CCAATGGCAGCATCTACTTCTCCgtaagttgaaaagacttcctctgcgtccctctttgcc
SRR516967.14759904.1-   CCAATGGCAGCATCTACTTCTCCgtaagttgaaaagacttcctctgcgtccctctttgcc
SRR516967.22260303.2-   CCAATGGCAGCATCTACTTCTCCgtaagttgaaaagacttcctctgcgtccctctttgcc
SRR516967.23066803.2-   CCAATGGCAGCATCTACTTCTCCgtaagttgaaaagacttcctctgcgtccctctttgcc
SRR516967.2436532.2-   CCAATGGCAGCATCTACTTCTCCgtaagttgaaaagacttcctctgcgtccctctttgcc
SRR516967.30937619.2-   CCAATGGCAGCATCTACTTCTCCgtaagttgaaaagacttcctctgcgtccctctttgcc
SRR516967.40446194.2-   CCAATGGCAGCATCTACTTCTCCgtaagttgaaaagacttcctctgcgtccctctttgcc
SRR516967.41713939.2+   CCAATGGCAGCATCTACTTCTCCgtaagttgaaaagacttcctctgcgtccctctttgcc
SRR516967.57741754.2-   CCAATGGCAGCATCTACTTCTCCgtaagttgaaaagacttcctctgcgtccctctttgcc
SRR516967.692820.2-   CCAATGGCAGCATCTACTTCTCCgtaagttgaaaagacttcctctgcgtccctctttgcc
SRR516967.71626809.1-   CCAATGGCAGCATCTACTTCTCCgtaagntgaaaagacttcctctgcgtccctctttgcc
SRR516967.72187734.2-   CCAACGGCAACATCTACTTCTCCgtaagttgaaaagacttcctctgcgtacctctttgcc
SRR516968.24302661.1+   CCAATGGCAGCATCTACTTCTCCgtaagttgaaaagacttcctctgcgtccctctttgcc
SRR516968.39655396.1-   CCAATGGCAGCATCTACTTCTCCgtaagttgaaaagacttcctctgcgtccctctttgcc
SRR516968.42660308.1-   CCAATGGCAGCATCTACTTCTCCgtaagttgaaaagacttcctctgcgtccctctttgcc
SRR516968.54367312.1-   CCAATGGCAGCATCTACTTCTCCgtaagttgaaaagacttcctctgcgtccctctttgcc
SRR516968.56762513.1+   CCAATGGCAGCATCTACTTCTCCgtaagttgaaaagacttcctctgcgtccctctttgcc
SRR516968.78882370.2+   CCAATGGCAGCATCTACTTCTCCgtaagttgaaaagacttcctctgcgtccctctttgcc
SRR516968.78910843.2+   CCAATGGCAGCATCTACTTCTCCgtaagttgaaaagacttcctctgcgtccctctttgcc
SRR516968.81828792.1-   CCAATGGCAGCATCTACTTCTCCgtaagttgaaaagacttcctctgcgtccctctttgcc
SRR516969.1886920.1+   CCAATGGCAGCATCTACTTCTCCgtaagttgaaaagacttcctctgcgtccctctttgcc
SRR516969.5105625.2+   CCAATGGCAGCATCTACTTCTCCgtaagttgaaaagacttcctctgcgtccctctttgcc
SRR516970.28438184.1-   CCAATGGCAGCATCTACTTCTCCgtaagttgaaaagacttcctctgcgtccctctttgcc
SRR516970.31765562.2-   CCAATGGCAGCATCTACTTCTCCgtaagttgaaaagacttcctctgcgtccctctttgcc
SRR516970.4123582.2+   CCAATGGCAGCATCTACTTCTCCgtaagttgaaaagacttcctctgcgtccctctttgcc
SRR516970.4586121.1+   CCAATGGCAGCATCTACTTCTCCgtaagttgaaaagacttcctctgcgtccctctttgcc
SRR516971.10454062.1-   CCAATGGCAGCATCTACTTCTCCgtaagttgaaaagacttcctctgcgtccctctttgcc
SRR516972.33835747.2+   CCAATGGCAGCATCTACTTCTCCgtaagttgaaaagacttcctctgcgtccctctttgcc
SRR516972.79902884.2+   CCAATGGCAGCATCTACTTCTCCgtaagttgaaaagacttcctctgcgtccctctttgcc
SRR516973.18772931.2-   CCAATGGCAGCATCTACTTCTCCgtaagttgaaaagacttcctctgcgtccctctttgcc
SRR516973.51524397.1+   CCAATGGCAGCATCTACTTCTCCgtaagttgaaaagacttcctctgcgtccctctttgcc
SRR516973.52438644.1+   CCAATGGCAGCATCTACTTCTCCgtaagttgaaaagacttcctctgcgtccctctttgcc
SRR516974.22378986.1-   CCAATGGCAGCATCTACTTCTCCgtaagttgaaaagacttcctctgcgtccctctttgcc
SRR516974.22531848.2-   CCAATGGCAGCATCTACTTCTCCgtaagttgaaaagacttcctctgcgtccctctttgcc
SRR516974.52405691.1+   CCAATGGCAGCATCTACTTCTCCgtaagttgaaaagacttcccctgcgtccctctttgcc
SRR516974.68991541.2+   CCAATGGCAGCATCTACTTCTCCgtaagttgaaaagccttcctctgcgtccctctttgcc
SRR516974.7795744.1+   CCAATGGCAGCATCTACTTCTCCgtaagttgaaaagacttcctctgcgtccctctttgcc
SRR516975.1707741.1+   CCAATGGCAGCATCTACTTCTCCgtaagttgaaaagacttcctctgcgtccctctttgcc
SRR516976.17282199.1-   CCAATGGCAGCATCTACTTCTCCgtaagttgaaaagacttcctctgcgtccctctttgcc
SRR516978.12424990.1+   CCAATGGCAGCATCTACTTCTCCgtaagttgaaaagacttcctctgcgtccctctttgcc
SRR516978.16880897.2+   CCAATGGCAGCATCTACTTCTCCgtaagttgaaaagacttcctctgcgtccctctttgcc
SRR516978.31371452.1+   CCAATGGCAGCATCTACTTCTCCgtaagttgaaaagacttcctctgcgtccctctttgcc
SRR516978.33666848.2+   CCAATGGCAGCATCTACTTCTCCgtaagttgaaaagacttcctctgcgtccctctttgcc
SRR516978.34278916.1+   CCAATGGCAGCATCTACTTCTCCgtaagttgaaaagacttcctctgcgtccctctttgcc
SRR516978.36274242.1+   CCAATGGCAGCATCTACTTCTCCgtaagttgaaaagacttcctctgcgtccctctttgcc
SRR516978.47252201.2-   CCAATGGCAGCATCGACTTCTCCgtaagttgaaaagacttcctctgcgtccctctttgcc
SRR516978.57947464.1+   CCAATGGCAGCATCTACTTCTCCgtaagttgaaaagacttcctctgcgtccctctttgcc
SRR516979.2283610.2+   CCAATGGCAGCATCTACTTCTCCgtaagttgaaaagacttcctctgcgtccctctttgcc
SRR516980.2509159.1-   CCAATGGCAGCATCTACTTCTCCgtaagttgaaaagacttcctctgcgtccctctttgcc
SRR516980.25761470.2-   CCAATGGCAGCATCTACTTCTCCgtaagttgaaaagacttcctctgcgtccctctttgcc

SRR516980.28746069.1-	CCAAAGGCATCATCTACTTCTCCgtaagttgaaaagacttcctctgcgtccctctttgcc
SRR516980.3868284.2-	CCAATGGCAGCATCTACTTCTCCgtaagttgaaaagacttcctctgcgtccctctttgcc
SRR516980.40326797.2-	CCAATGGCAGCATCTACTTCTCCgtaagttgaaaagacttcctctgcgtccctctttgcc
SRR516980.775983.2-	CCAATGGCAGCATCTACTTCTCCgtaagttgaaaagacttcctctgcgtccctctttgcc
SRR516981.2688314.1+	CCAATGGCAGCATCTACTTCTCCgtaagttgaaaagacttcctctgcgtccctctttgcc
SRR516981.5028621.1-	CCAATGGCAGCATCTACTTCTCCgtaagttgaaaagacttcctctgcgtccctctttgcc
SRR516982.46727106.1-	CCAATGGCAGCATCTACTTCTCCgtaagttgaaaagacttcctctgcgtccctctttgcc
SRR516983.50031803.1+	CCAATGGCAGCATCTACTTCTCCgtaagttgaaaagacttcctctgcgtccctctttgcc
SRR516983.77412505.1-	CCAATGGCAGCATCTACTTCTCCgtaagttgaaaagacttcctctgcgtccctctttgcc
SRR516984.21208613.1+	CCAATGGCAGCATCTACTTCTCCgtaagttgaaaagacttcctctgcgtccctctttgcc
SRR516984.49729110.2-	CCAATGGCAGCATCTACTTCTCCgtaagttgaaaagacttcctctgcgtccctctttgcc
SRR516984.91805446.1-	CCAATGGCAGCATCTACTTCTCCgtaagttgaaaagacttcctctgcgtccctctttgcc
SRR516985.36645915.1+	CCAATGGCAGCATCTACTTCTCCgtaagttgaaaagacttcctctgcgtccctctttgcc
SRR516985.41342798.1-	CCAATGGCAGCATCTACTTCTCCgtaagttgaaaagacttcctctgcgtccctctttgcc
SRR516985.52849243.1-	CCAATGGCAGCATCTACTTCTCCgtaagttgaaaagacttcctctgcgtccctctttgcc
SRR516985.53600329.1+	CCAATGGCAGCATCTACTTCTCCgtaagttgaaaagacttcctctgcgtccctctttgcc
SRR516985.68798507.2+	CCAATGGCAGCATCTACTTCTCCgtaagttgaaaagacttcctctgcgtccctctttgcc
SRR516985.88024111.2-	CCAATGGCAGCATCTACTTCTCCgtaagttgaaaagacttcctctgcgtccctctttgcc
SRR516986.3185698.1+	CCAATGGCAGCATCTACTTCTCCgtaagttgaaaagacttcctctgcgtccctctttgcc
SRR516986.37734465.1-	CCAATGGCAGCATCTACTTCTCCgtaagttgaaaagacttcctctgcgtccctctttgcc
SRR516986.41476073.2-	CCAATGGCAGCATCTACTTCTCCgtaagttgaaaagacttcctctgcgtccctctttgcc
SRR516986.43777664.2-	CCAATGGCAGCATCTACTTCTCCgtaagttgaaaagacttcctctgcgtccctctttgcc
SRR516986.49636629.1-	CCAATGGCAGCATCTACTTCTCCgtaagttgaaaagacttcctctgcgtccctctttgcc
SRR516986.5238698.2+	CCAATGGCAGCATCTACTTCTCCgtaagttgaaaagacttcctctgcgtccctctttgcc
SRR516986.64170753.2+	CCAATGGCAGCATCTACTTCTCCgtaagttgaaaagacttcctctgcgtccctctttgcc
SRR516987.20421578.2-	CCAATGGCAGCATCTACTTCTCCgtaagttgaaaagacttcctctgcgtccctctttgcc
SRR516987.24003748.2+	CCAATGGCAGCATCTACTTCTCCgtaagttgaaaagacttcctctgcgtccctctttgcc
SRR516987.32634383.2+	CCAATGGCAGCATCTACTTCTCCgtaagttgaaaagacttcctctgcgtccctctttgcc
SRR516987.57947905.1-	CCAATGGCAGCATCTACTTCTCCgtaagttgaaaagacttcctctgagtccctctttgcc
SRR516988.12631476.2-	CCAATGGCATCATCTACTTCTCCgtaagttgaaaagacttcctctgcgtccctctttgcc
SRR516988.15095747.2+	CCAATGGCAGCATCTACTTCTCCgtaagttgaaaagacttcctctgcgtccctctttgcc
SRR516988.2226181.1+	CCAATGGCAGCATCTACTTCTCCgtaagttgaaaagacttcctctgcgtccctctttgcc
SRR516988.3021865.2-	CCAATGGCAGCATCTACTTCTCCgtaagttgaaaagacttccgctgcgtccctctttgcc
SRR516988.35162539.2+	CCAATGGCAGCATCTACTTCTCCgtaagttgaaaagacttcctctgcgtccctctttgcc
SRR516988.39872613.2+	CCAATGGCAGCATCTACTTCTCCgtaagttgaaaagacttcctctgcgtccctctttgcc
SRR516989.32421189.1-	CCAATGGCAGCATCTACTTCTCCgtaagttgaaaagacttcctctgcgtccctctttgcc
SRR516989.41185156.2+	CCAATGGCAGCATCTACTTCTCCgtaagttgaaaagacttcctctgcgtccctctttgcc
SRR516989.52805078.2+	CCAATGGCAGCATCTACTTCTCCgtaagttgaaaagacttcctctgcgtccctctttgcc
SRR516989.70274568.1-	CCAATGGCAGCATCTACTTCTCCgtaagttgaaaagacttcctctgcgtccctctttgcc
SRR516990.10179653.1+	CCAATGGCAGCATCTACTTCTCCgtaagttgaaaagacttcctctgcgtccctctttgcc
SRR516990.14450506.1-	CCAATGGCAGCATCTACTTCTCCgtaagttgaaaagacttcctctgcgtccctctttgcc
SRR516990.60778082.1-	CCAATGGCAGCATCTACTTCTCCgtaagttgaaaagacttcctctgcgtccctctttgcc
SRR516991.17316272.1-	CCAATGGCAGCATCTACTTCTCCgtaagttgaaaagacttcctctgcgtccctctttgcc
SRR516991.84042397.1+	CCAATGGCAGCATCTACTTCTCCgtaagttgaaaagacttcctctgcgtccctctttgcc
SRR516992.47247048.2-	CCAATGGCAGCATCTACTTCTCCgtaagttgaaaagacttcctctgcgtccctctttgcc
SRR516992.55128523.1+	CCAATGGCAGCATCTACTTCTCCgtaagttgaaaagacttcctctgcgtccctctttgcc
SRR516992.73689497.1+	CCAATGGCAGCATCTACTTCTCCgtaagttgaaaagacttcctctgcgtccctctttgcc
SRR516993.22248191.1-	CCAATGGCAGCATCTACTTCTCCgtaagttgaaaagacttcctctgcgtccctctttgcc
SRR516993.6697428.2+	CCAATGGCAGCATCTACTTCTCCgtaagttgaaaagacttcctctgcgtccctctttgcc
SRR516993.88041825.1-	CCAATGGCAGCATCTACTTCTCCgtaagttgaaaagacttcctctgcgtccctctttgcc
SRR516994.26366517.1-	CCAATGGCAGCATCTACTTCTCCgtaagttgaaaagacttcctctgcgtccctctttgcc
SRR516994.26789295.2-	CCAATGGCAGCATCTACTTCTCCgtaagttgaaaagacttcctctgcgtccctctttgcc
SRR516994.55351595.2-	CCAATGGCAGCATCTACTTCTCCgtaagttgaaaagacttcctctgcgtccctctttgcc
SRR516994.66993668.2-	CCAATGGCAGCATCTACTTCTCCgtaagttgaaaagacttcctctgcgtccctctttgcc
SRR516994.84145190.1-	CCAATGGCAGCATCTACTTCTCCgtaagttgaaaagacttcctctgcgtccctctttgcc
SRR516995.11496028.1-	CCAATGGCAGCATCTACTTCTCCgtaagttgaaaagacttcctctgcgtccctctttgcc
SRR516995.18825621.1+	CCAATGGCAGCATCTACTTCTCCgtaagttgaaaagacttcctctgcgtccctctttgcc
SRR516995.21670383.2+	CCAATGGCAGCATCTACTTCTCCgtaagttgaaaagacttcctctgcgtccctctttgcc
SRR516995.28796839.1-	CCAATGGCAGCATCTACTTCTCCgtaagttgaaaagacttcctctgcgtccctctttgcc
SRR516995.37587733.2-	CCAATGGCAGCATCTACTTCTCCgtaagttgaaaagacttcctctgcgtccctctttgcc
SRR516995.39713292.2+	CCAATGGCAGCATCTACTTCTCCgtaagttgaaaagacttcctctgcgtccctctttgcc
SRR516995.51804784.1-	CCAATGGCAGCATCTACTTCTCCgtaagttgaaaagacctcctctgcgtccctctttgcc
SRR516995.53414570.2+	CCAATGGCAGCATCTACTTCTCCgtaagttgaaaagacttcctctgcgtccctctttgcc
SRR516995.64633284.1+	CCAATGGCAGCATCTACTTCTCCgtaagttgaaaagacttcctctgcgtccctctttgcc
SRR516995.68978295.1+	CCAATGGCAGCATCTACTTCTCCgtaagttgaaaagacttcctctgcgtccctctttgcc
SRR516995.69712712.2+	CCAATGGCAGCATCTACTTCTCCgtaagttgaaaagacttcctctgcgtccctctttgcc
SRR516995.88022888.2+	CCAATGGCAGCATCTACTTCTCCgtaagttgaaaagacttcctctgcgtccctctttgcc
SRR516996.44208108.1+	CCAATGGCAGCATCTACTTCTCCgtaagttgaaaagacttcctctgcgtccctctttgcc
SRR516996.75812492.2+	CCAATGGCAGCATCTACTTCTCCgtaagttgaaaagacttcctctgcgtccctctttgcc
SRR516996.81858289.1+	CCAATGGCAGCATCTACTTCTCCgtaagttgaaaagacttcctctgcgtccctctttgcc
SRR516997.49291908.1-	CCAATGGCAGCATCTACTTCTCCgtaagttgaaaagacttcctctgcgtccctctttgcc
SRR516997.74953572.2+	CCAATGGCAGCATCTACTTCTCCgtaagttgaaaagacttccactgcgtccctctttgcc
SRR516997.94233769.2+	CCAATGGCAGCATCTACTTCTCCgtaagttgaaaagacttcctctgcgtccctctttgcc
SRR516998.10352572.2+	CCAATGGCAGCATCTACTTCTCCgtaagttgaaaagacttcctctgcgtccctctttgcc
SRR516998.28329965.2+	CCAATGGCAGCATCTACTTCTCCgtaagttgaaaagacttcctctgcgtccctctttgcc
SRR516998.29101479.1-	CCAATGGCAGCATCTACTTCTCCgtaagttgaaaagacttcctctgcgtccctctttgcc
SRR516998.59445481.2-	CCAATGGCAGCATCTACTTCTCCgtaagttgaaaagacttcctctgcgtccctctttgcc
SRR516998.60968600.2-	CCAATGGCAGCATCTACTTCTCCgtaagttgaaaagacttcctctgcgtccctctttgcc
SRR516998.62511336.1-	CCAATGGCAGCATCTACTTCTCCgtaagttgaaaagacttcctctgcgtccctctttgcc
SRR516998.70570011.2-	CCAATGGCAGCATCTACTTCTCCgtaagttgaaaagacttcctctgcgtccctctttgcc
SRR516998.72828587.1+	CCAATGGCAGCATCTACTTCTCCgtaagttgaaaagacttcctctgcgtccctctttgcc
SRR516998.94356641.1+	CCAATGGCAGCATCTACTTCTCCgtaagttgaaaagacttcctctgcgtccctctttgcc
SRR516998.96733754.2-	CCAATGGCAGCATCTACTTCTCCgtaagttgaaaagacttcctctgcgtccctctttgcc
SRR516998.98321200.2-	CCAATGGCAGCATCTACTTCTCCgtaagttgaaaagacttcctctgcgtccctctttgcc

```
SRR516999.12781921.1+    CCAATGGCAGCATCTACTTCTCCgtaagttgaaaagacttcctctgcgtccctctttgcc
SRR516999.25368915.2+    CCAATGGCAGCATCTACTTCTCCgtaagttgaaaagacttcctctgcgtccctctttgcc
SRR516999.2638208.1-     CCAATGGCAGCATCTACTTCTCCgtaagttgaaaagacttcctctgcgtccctctttgcc
SRR516999.28114376.1-    CCAATGGCAGCATCTACTTCTCCgtaagttgaaaagacttcctctgcgtccctctttgcc
SRR516999.51370430.2+    CCAATGGCAGCATCTACTTCTCCgtaagttgaaaagacttcctctgcgtccctctttgcc
SRR516999.69510462.1+    CCAATGGCAGCATCTACTTCTCCgtaagttgaaaagacttcctctgcgtccctctttgcc
SRR516999.81142078.2-    CCAATGGCAGCATCTACTTCTCCgtaagttgaaaagacttcctctgcgtccctctttgcc
SRR517000.105547603.2+   CCAATGGCAGCATCTACTTCTCCgtaagttgaaaagacttcctctgcgtccctctttgcc
SRR517000.17971450.2+    CCAATGGCAGCATCTACTTCTCCgtaagttgaaaagacttcctctgcgtccctctttgcc
SRR517000.2083309.2+     CCAATGGCAGCATCTACTTCTCCgtaagttgaaaagacttcctctgcgtccctctttgcc
SRR517000.28657256.2-    CCAATGGCAGCATCTACTTCTCCgtaagttgaaaagacttcctctgcgtccctctttgcc
SRR517000.38379134.1-    CCAATGGCAGCATCTACTTCTCCgtaagttgaaaagacttcctctgcgtccctctttgcc
SRR517000.40713109.1-    CCAATGGCAGCATCTACTTCTCCgtaagttgaaaagacttcctctgcgtccctctttgcc
SRR517000.43080184.1+    CCAATGGCAGCATCTACTTCTCCgtaagttgaaaagacttcctctgcgtccctctttgcc
SRR517000.62667511.1+    CCAATGGCAGCATCTACTTCTCCgtaagttgaaaagacttcctctgcgtccctctttgcc
SRR517000.94848556.1-    CCAATGGCAGCATCTACTTCTCCgtaagttgaaaagacttcctctgcgtccctctttgcc
SRR517001.103363219.1+   CCAATGGCAGCATCTACTTCTCCgtaagttgaaaagacttcctctgcgtccctctttgcc
SRR517001.36815698.1+    CCAATGGCAGCATCTACTTCTCCgtaagttgaaaagacttcctctgcgtccctctttgcc
SRR517001.43868988.1-    CCAATGGCAGCATCTACTTCTCCgtaagttgaaaagacttcctctgcgtccctctttgcc
SRR517001.52204355.2+    CCAATGGCAGCATCTACTTCTCCgtaagttgaaaagacttcctctgcgtccctctttgcc
SRR517001.74558392.2-    CCAATGGCAGCATCTACTTCTCCgtaagttgaaaagacttcctctgcgtccctctttgcc
SRR517001.89746771.1-    CCAATGGCAGCATCTACTTCTCCgtaagttgaaaagacttcctctgcgtccctctttgcc
SRR517002.13637222.1-    CCAATGGCAGCATCTACTTCTCCgtaagttgaaaagacttcctctgcgtccctctttgcc
SRR517002.3322034.1-     CCAATGGCAGCATCTACTTCTCCgtaagttgaaaagacttcctctgcgtccctctttgcc
SRR517002.33929122.2+    CCAATGGCAGCATCTACTTCTCCgtaagttgaaaagacttcctctgcgtccctctttgcc
SRR517002.60341884.1+    CCAATGGCAGCATCTACTTCTCCgtaagttgaaaagacttcctctgcgtccctctttgcc
SRR517003.33278135.2+    CCAATGGCAGCATCTACTTCTCCgtaagttgaaaagacttcctctgcgtccctctttgcc
SRR517003.35492282.1+    CCAATGGCAGCATCTACTTCTCCgtaagttgaaaagacttcctctgcgtccctctttgcc
SRR517003.37121286.2-    CCAATGGCAGCATCTACTTCTCCgtaagttgaaaagacttcctctgcgtccctctttgcc
SRR517003.37904939.2+    CCAATGGCAGCATCTACTTCTCCgtaagttgaaaagacttcctctgcgtccctctttgcc
SRR517003.46003185.2-    CCAATGGCAGCATCTACTTCTCCgtaagttgaaaagacttcctctgcgtccctctttgcc
SRR517003.46003189.2-    CCAATGGCAGCATCTACTTCTCCgtaagttgaaaagacttcctctgcgtccctctttgcc
SRR517003.49139834.1+    CCAATGGCAGCATCTACTTCTCCgtaagttgaaaagacttcctctgcgtccctctttgcc
SRR517004.1229485.2+     CCAATGGCAGCATCTACTTCTCCgtaagttgaaaagacttcctctgcgtccctctttgcc
SRR517004.29851537.1+    CCAATGGCAGCATCTACTTCTCCgtaagttgaaaagacttcctctgcgtccctctttgcc
SRR517004.35999263.2-    CCAATGGCAGCATCTACTTCTCCgtaagttgaaaagacttcctctgcgtccctctttgcc
SRR517004.54443238.1+    CCAATGGCAGCATCTACTTCTCCgtaagttgaaaagacttcctctgcgtccctctttgcc
SRR517005.12336995.1+    CCAATGGCAGCATCTACTTCTCCgtaagttgaaaagacttcctctgcgtccctctttgcc
SRR517005.22087228.1-    CCAATGGCAGCATCTACTTCTCCgtaagttgaaaagacttcctctgcgtccctctttgcc
SRR517005.5761837.2+     CCAATGGCAGCATCTACTTCTCCgtaagttgaaaagacttcctctgcgtccctctttgcc
SRR517005.63490107.1-    CCAATGGCAGCATCTACTTCTCCgtaagttgaaaagacttcctctgcgtccctctttgcc
SRR517005.76976432.1-    CCAATGGCAGCATCTACTTCTCCgtaagttgaaaagacttcctctgcgtccctctttgcc
SRR517006.16403245.2+    CCAATGGCAGCATCTACTTCTCCgtaagttgaaaagaattcctctgcgtccctctttgcc
SRR517006.17097416.1-    CCAATGGCAGCATCTACTTCTCCgtaagttgaaaagacttcctctgcgtccctctttgcc
SRR517006.40089046.1-    CCAATGGCAGCATCTACTTCTCCgtaagttgaaaagacttcctctgcgtccctctttgcc
SRR517006.48465992.1+    CCAATGGCAGCATCTACTTCTCCgtaagttgaaaagacttcctctgcgtccctctttgcc
SRR517006.63297513.1+    CCAATGGCAGCATCTACTTCTCCgtaagttgaaaagacttcctctgcgtccctctttgcc
SRR517006.68098848.2-    CCAATGGCAGCATCTACTTCTCCgtaagttgaaaagacttcctctgcgtccctctttgcc
SRR517007.27209892.1-    CCAATGGCAGCATCTACTTCTCCgtaagttgaaaagacttcctctgcgtccctctttgcc
SRR517007.28898075.1-    CCAATGGCAGCATCTACTTCTCCgtaagttgaaaagacttcctctgcgtccctctttgcc
SRR517007.52883726.1-    CCAATGGCAGCATCTACTTCTCCgtaagttgaaaaggcttcctctgcgtccctctttgcc
SRR517007.60677870.2-    CCAATGGCAGCATCTACTTCTCCgtaagttgaaaagacttcctctgcgtccctctttgcc
SRR517007.65469916.1-    CCAATGGCAGCATCTACTTCTCCgtaagttgaaaagacttcctctgcgtccctctttgcc
SRR517008.1945327.2+     CCAATGGCAGCATCTACTTCTCCgtaagttgaaaagacttcctctgcgtccctctttgcc
SRR517008.23794481.2-    CCAATGGCAGCATCTACTTCTCCgtaagttgaaaagacttcctctgcgtccctctttgcc
SRR517008.3800488.2-     CCAATGGCAGCATCTACTTCTCCgtaagttgaaaagacttcctctgcgtccctctttgcc
SRR517008.38108470.2+    CCAATGGCAGCATCTACTTCTCCgtaagttgaaaagacttcctctgcgtccctctttgcc
SRR517008.38355138.1-    CCAATGGCAGCATCTACTTCTCCgtaagttgaaaagacttcctctgcgtccctctttgcc
SRR517008.4270466.1-     CCAATGGCAGCATCTACTTCTCCgtaagttgaaaagacttcctctgcgtccctctttgcc
SRR517008.42787980.2+    CCAATGGCAGCATCTACTTCTCCgtaagttgtaaagacttcctctgcgtccctctttgcc
SRR517008.80424603.2-    CCAATGGCAGCATCTACTTCTCCgtaagttgaaaagacttcctctgcgtccctctttgcc
SRR517009.20742062.1-    CCAATGGCAGCATCTACTTCTCCgtaagttgaaaagacttcctctgcgtccctctttgcc
SRR517009.21340928.1+    CCAATGGCAGCATCTACTTCTCCgtaagttgaaaagacttcctctgcgtccctctttgcc
SRR517009.45728121.1+    CCAATGGCAGCATCTACTTCTCCgtaagttgaaaagacttcctctgcgtccctctttgcc
SRR517009.60480305.2+    CCAATGGCAGCATCTACTTCTCCgtaagttgaaaagacttcctctgcgtccctctttgcc
SRR517009.64828053.1+    CCAATGGCAGCATCTACTTCTCCgtaagttgaaaagacttcctctgcgtccctctttgcc
SRR517009.69208174.1-    CCAATGGCAGCATCTACTTCTCCgtaagttgaaaagacttcctctgcgtccctctttgcc
SRR517009.81050649.2-    CCAATGGCAGCATCTACTTCTCCgtaagttgaaaagacttcctctgcgtccctctttgcc
SRR517009.8825375.1+     CCAATGGCAGCATCTACTTCTCCgtaagttgaaaagacttcctctgcgtccctctttgcc
SRR516968.37214890.2-     CAATGGCAACATTTACTTCTCCgtaagttgaaaagacttcctctgcgtccctctttgcc
SRR516974.56217086.2+     CAATGGCAGCATCTACTTCTCCgtaagttgaaaagacttcctctgcgtccctctttgcc
SRR516994.78490657.1+     CAATGGCAGCATCTACTTCTCCgtaagttgaaaagacttcctctgcgtccctctttgcc
SRR516999.49488894.2-     CAATGGCAGCATCTACTTCGCCgtaagttgaaaagacttcctctgcgtccctctttgcc
SRR516974.64853297.1+      AATGGCAGCATCTACTTCTCCgtaagttgaaaagacttcctctgcgtccctctttgcc
SRR516998.63498480.1+      AATGGCAGCATCTACTTCTCCgtaagttgaaaagacttcctctgcgtccctctttgcc
SRR516998.85413374.2-      AATGGCAGCATCTACTTCTCCgtaagttgaaaagacttcctctgcgtccctctttgcc
SRR517008.31261980.1+      AATGGCAGCATCTACTTCTCCgtaagttgaaaagacttcctctgcgtccctctttgcc
SRR517009.12755840.1-      AATGGCAGCATCTACTTCTCCgtaagttgaaaagacttcctctgcgtccctctttgcc
SRR516974.72399904.1+       ATGGCAGCATCTACTTCTCCgtaagttgaaaagacttcctctgcgtccctctttgcc
SRR517008.73275508.2-       ATGGCAGCATCTACTTCTCCgtaagttgaaaagacttcctctgcgtccctctttgcc
SRR516967.141008204.2+       TGGCAGCATCTACTTCTCCgtaagttgaaaagacttcctctgcgtccctctttgcc
SRR516977.13601535.2-        TGGCAGCATCTACTTCTCCgtaagttgaaaagacttcctctgcgtccctctttgcc
SRR516977.69611362.1-        TGGCAGCATCTACTTCTCCgtaagttgaaaagacttcctctgcgtccctctttgcc
SRR516986.65904528.1-        TGGCAGCATCTACTTCTCCgtaagttgaaaagacttcctctgcgtccctctttgcc
```

```
SRR517003.46053853.2+        TGGCAGCATCTACTTCTCCgtaagttgaaaagacttcctctgcgtccctctttgcc
SRR517003.53406887.1+        TGGCAGCATCTACTTCTCCgtaagttgaaaagacttcctctgcgtccctctttgcc
SRR517005.86090511.2+        TGGCAGCATCTACTTCTCCgtaagttgaaaagacttcctctgcgtccctctttgcc
SRR511911.46262638.2+         GGCAGCATCTACTTCTCCgtaagttgaaaagacttcctctgcgtccctctttgcc
SRR516970.7640225.1-          GGCAGCATCTACTTCTCCgtaagttgaaaagacttcctctgcgtccctctttgcc
SRR516972.39048989.1-         GGCAGCATCTACTTCTCCgtaagttgaaaagacttcctctgcgtccctctttgcc
SRR516975.30822760.2-         GGCAGCATCTACTTCTCCgtaagttgaaaagacttcctctgcgtccctctttgcc
SRR516977.26165189.2+         GGCAGCATCTACTTCTCCgtaagttgaaaagacttcctctgcgtccctctttgcc
SRR516979.69530139.2-         GGCAGCATCTACTTCTCCgtaagttgaaaagacttcctctgcgtccctctttgcc
SRR516981.20922821.2-         GGCAGCATCTACTTCTCCgtaagttgaaaagacttcctctgcgtccctctttgcc
SRR516985.54679927.1+         GGCAGCATCTACTTCTCCgtaagttgaaaagacttcctctgcgtccctctttgcc
SRR516992.69961004.2-         GGCAGCATCTACTTCTCCgtaagttgaaaagacttcctctgcgtccctctttgcc
SRR516995.88144119.1+         GGCAGCATCTACTTCTCCgtaagttgaaaagacttcctctgcgtccctctttgcc
SRR516996.39635165.1+         GGCAGCATCTACTTCTCCgtaagttgaaaagacttcctctgcgtccctctttgcc
SRR516975.20052745.1-          GCAGCATCTACTTCTCCgtaagttgaaaagacttcctctgcgtccctctttgcc
SRR516976.38940434.2+          GCAGCATCTACTTCTCCgtaagttgaaaagacttcctctgcgtccctctttgcc
SRR516977.41688351.2+          GCAGCATCTACTTCTCCgtaagttgaaaagacttcctctgcgtccctctttgcc
SRR516981.30629252.2-          GCAGCATCTACTTCTCCgtaagttgaaaagacttcctctgcgtccctctttgcc
SRR516996.89760632.1-          GCAGCATCTACTTCTCCgtaagttgaaaagacttcctctgcgtccctctctgcc
SRR517001.15533161.1-          GCAGCATCTACTTCTCCgtaagttgaaaagacttcctctgcgtccctctttgcc
SRR517005.68594324.2-          GCAGCATCTACTTCTCCgtaagttgaaaagacttcctctgcgtccctctttgcc
SRR516968.109900230.2-          CAGCATCTACTTCTCCgtaagttgaagagacttcctctgcgtccctctttgcc
SRR516979.16124664.1-           CAGCATCTACTTCTCCgtaagttgaaaagacttcctctgcgtccctctttgcc
SRR516983.61644045.1+           CAGCATCTACTTCTCCgtaagttgaaaagacttcctctgcgtccctctttgcc
SRR516986.7462760.1-            CAGCATCTACTTCTCCgtaagttgaaaagacttcctctgcgtccctctttgcc
SRR516987.49326432.1-           CAGCATCTACTTCTCCgtaagttgaaaagacttcctctgcgtccctctttgcc
SRR516987.64786739.2-           CAGCATCTACTTCTCCgtaagttgaaaagacttcctctgcgtccctctttgcc
SRR516997.31433076.1+           CAGCATCTACTTCTCCgtaagttgaaaagacttcctctgcgtccctctttgcc
SRR516997.88273145.1+           CAGCATCTACTTCTCCgtaagttgaaaagacttcctctgcgtccctctttgcc
SRR516998.97862365.1-           CAGCATCTACTTCTCCgtaagttgaaaagacttcctctgcgtccctctttgcc
SRR517004.53711062.2+           CAGCATCTACTTCTCCgtaagttgaaaagacttcctctgcgtccctctttgcc
SRR517009.40763089.2+           CAGCATCTACTTCTCCgtaagttgaaaagacttcctctgcgtccctctttgcc
SRR511912.11118205.2+            AGCATCTACTTCTCCgtaagttgaaaagacttcctctgcgtccctctttgcc
SRR511912.60332391.1-            AGCATCTACTTCTCCgtaagttgaaaagacttcctctgcgtccctctttgcc
SRR511912.60332545.1-            AGCATCTACTTCTCCgtaagttgaaaagacttcctctgcgtccctctttgcc
SRR511914.71956528.2-            AGCATCTACTTCTCCgtaagttgaaaagacttcctctgcgtccctctttgcc
SRR516967.88384226.1-            AGCATCTACTTCTCCgtaagttgaaaagacttcctctgcgtccctctttgcc
SRR516969.18417523.2+            AGCATCTACTTCTCCgtaagttgaaaagacttcctctgcgtccctctttgcc
SRR516969.9631131.2-             AGCATCTACTGCTCCgtaagttgaaaagacttcctctgcgtccctctttgcc
SRR516971.24445351.2-            AGCATCTACTTCTCCgtaagttgaaaagacttcctctgcgtccctctttgcc
SRR516975.25668571.1+            AGCATCTACTTCTCCgtaagttgaaaagacttcctctgcgtccctctttgcc
SRR516982.32243345.1+            AGCATCTACTTCTCCgtaagttgaaaagacttcctctgcgtccctctttgcc
SRR516983.48910894.2+            AGCATCTACTTCTCCgtaagttgaaaagacttcctctgcgtccctctttgcc
SRR516999.13449474.2+            AGCATCTACTTCTCCgtaagttgaaaagacttcctctgcgtccctctttgcc
SRR517002.68056350.1+            AGCATCTACTTCTCCgtaagttgaaaagacttcctctgcgtccctctttgcc
SRR517004.25302532.1-            AGCATCTACTTCTCCgtaagttgaaaagacttcctctgcgtccctctttgcc
SRR517005.96155006.2-            AGCATCTACTTCTCCgtaagttgaaaagacttcctctgcgtccctctttgcc
SRR517006.42670235.2-            AGCATCTACTTCTCCgtaagttgaaaagacttcctctgcgtccctctttgcc
SRR511916.518392.1-               GCATCTACTTCTCCgtaagttgaaaagacttcctctgcgtccctctttg
SRR511916.520845.1-               GCATCTACTTCTCCgtaagttgaaaagacttcctctgcgtccctctttg
SRR511916.61995804.1-             GCATCTACTTCTCCgtaagttgaaaagacttcctctgcgtccctctttg
SRR516967.83635052.1+             GCATCTACTTCTCCgtaagttgaaaagacttcctctgcgtccctctttncc
SRR516978.12749059.1-             GCATCTACTTCTCCgtaagttgaaaagacttcctctgcgtccctctttgcc
SRR516981.56307628.1-             GCATCTACTTCTCCgtaagttgaaaagacttcctctgcgtccctctttgcc
SRR516990.36019817.1-             GCATCTACTTCTCCgtaagttgaaaagacttcctctgcgtccctctttgcc
SRR516994.66665340.2-             GCATCTACTTCTCCgtaagttgaaaagacttcctctgcgtccctctttgcc
SRR516995.85354243.2+             GCATCTACTTCTCCgtaagttgaaaagacttcctctgcgtccctctttgcc
SRR516998.89372575.2-             GCATCTACTTCTCCgtaagttgaaaagacttcctctgcgtccctctttgcc
SRR516999.89579473.1+             GCATCTACTTCTCCgtaagttgaaaagacttcctctgcgtccctctttgcc
SRR511916.25407227.1+              CATCTACTTCTCCgtaagttgaaaagacttcctctgcgtccctctttgc
SRR511914.15254481.1+              CATCTACTTCTCCgtaagttgaaaagacttcctctgcgtccctctttgcc
SRR516968.7574142.2-               CATCTACTTCTCCgtaagttgaaaagacttcctctgcgtccctctttgcc
SRR516977.83265007.2+              CATCTACTTCTCCgtaagttgaaaagacttcctctgcgtccctctttgcc
SRR516978.45987045.1-              CATCTACTTCTCCgtaagttgaaaagacttcctctgcgtccctctttgcc
SRR516982.35528402.1-              CATCTACTTCTCCgtaagttgaaaagacttcctctgcgtccctctttgcc
SRR516984.30012495.2+              CATCTACTTCTCCgtaagttgaaaagacttcctctgcgtccctctttgcc
SRR516994.13341376.1-              CATCTACTTCTCCgtaagttgaaaagacttcctctgcgtccctctttgcc
SRR516994.49602276.2+              CATCTACTTCTCCgtaagttgaaaagacttcctctgcgtccctctttgcc
SRR516997.74138406.1-              CATCTACTTCTCCgtaagttgaaaagacttcctctgcgtccctctttgcc
SRR511892.40440531.1+               ATCTACTTCTCCgtaagttgaaaagacttcctctgcgtccctctttgcc
SRR511914.61275803.2-               ATCTACTTCTCCgtaagttgaaaagacttcctctgcgtccctctttgcc
SRR511914.70015434.2-               ATCTACTTCTCCgtaagttgaaaagacttcctctgcgtccctctttgcc
SRR516967.126650903.2+              ATCTACTTCTCCgtaagttgaaaagacttcctctgcgtccctctttgcc
SRR516972.50240772.2-               ATCTACTTCTCCgtaagttgaaaagacttcctctgcgtccctctttgcc
SRR516972.50240775.2-               ATCTACTTCTCCgtaagttgaaaagacttcctctgcgtccctctttgcc
SRR516974.5445980.2+                ATCTACTTCTCCgtaagttgaaaagacttcctctgcgtccctctttgcc
SRR516978.39800576.1-               ATCTACTTCTCCgtaagttgaaaagacttcctctgcgtccctctttgcc
SRR516980.2885002.2+                ATCTACTTCTCCgtaagttgaaaagacttcctctgcgtccctctttgcc
SRR516987.11233788.1+               ATCTACTTCTCCgtaagttgaaaagacttcctctgcgtccctctttgcc
SRR516994.48478756.1+               ATCTACTTCTCCgtaagttgaaaagacttcctctgcgtccctctttgcc
SRR516994.70278140.1+               ATCTACTTCTCCgtaagttgaaaagacttcctctgcgtccctctttgcc
SRR516995.70042273.1+               ATCTACTTCTCCgtaagttgaaaagacttcctctgcgtccctctttgcc
SRR516996.23796146.1+               ATCTACTTCTCCgtaagttgaaaagacttcctctgcgtccctctttgcc
SRR517000.36416800.2-               ATCTACTTCTCCgtaagttgaaaagacttcctctgcgtccctctttgcc
```

```
SRR517000.65509884.1-               ATCTACTTCTCCgtaagttgaaaagacttcctctgcgtccctctttgcc
SRR517008.54960209.1+               ATCTACTTCTCCgtaagttgaaaagacttcctctgcgtccctctttgcc
SRR517009.48627248.1+               ATCTACTTCTCCgtaagttgaaaagacttcctctgcgtccctctttgcc
SRR511911.63900105.2+                TCTACTTCTCCgtaagttgaaaagacttcctctgcgtccctctttgcc
SRR511917.71204398.1+                TCTACTTCTCCgtaagttgaaaagacttcctctgcgtccctctttgcc
SRR516987.27292211.2+                TCTACTTCTCCgtaagttgaaaagacttcctctgcgtccctctttgcc
SRR516990.2053577.2+                 TCTACTTCTCCgtaagttgaaaagacttcctctgcgtccctctttgcc
SRR516996.95332716.1-                TCTACTTCTCCgtaagttgaaaagacttcctctgcgtccctctttgcc
SRR516999.88130122.2-                TCTACTTCTCCgtaagttgaaaagacttcctctgcgtccctctttgcc
SRR511914.1807274.1-                  CTACTTCTCCgtaagttgaaaagacttcctctgcgtccctctttgcc
SRR511914.1807431.1-                  CTACTGCTCCgtaagttgaaaagacttcctctgcgtccctctttgcc
SRR511916.44041200.1+                 CTACTTCTCCgtaagttgaaaagacttcctctgcgtccctctttgcc
SRR511917.39158438.1+                 CTACTTCTCCgtaagttgaaaagacttcctctgcgtccctctttgcc
SRR516974.6531958.2-                  CTACTTCTCCgtaagttgaaaagacttcctctgcgtccctctttgcc
SRR516977.9095642.1+                  CTACTTCTCCgtaagttgaaaagacttcctctgcgtccctctttgcc
SRR516985.1018877.2-                  CTACTTCTCCgtaagttgaaaagacttcctctgcgtccctctttgcc
SRR516985.37489698.1+                 CTACTTCTCCgtaagttgaaaagacttcctctgcgtccctctttgcc
SRR516986.18961603.2-                 CTACTTCTCCgtaagttgaaaagacttcctctgcgtccctctttgcc
SRR516994.49859430.1+                 CTACTTCTCCgtaagttgaaaagacttcctctgcgtccctctttgcc
SRR517002.78309254.2-                 CTACTTCTCCgtaagttgaaaagacttcctctgcgtccctctttgcc
SRR517003.19492607.1-                 CTACTTCTCCgtaagttgaaaagacttcctctgcgtccctctttgcc
SRR511892.3895804.1+                   TACTTCTCCgtaagttgaaaagacttcctctgcgtccctctttgcc
SRR511911.38932988.2+                  TACTTCTCCgtaagttgaaaagacttcctctgcgtccctctttgcc
SRR511915.40385008.2-                  TACTTCTCCgtaagttgaaaagacttcctctgcgtccctctttgcc
SRR511917.6944734.2-                   TACTTCTCCgtaagttgaaaagacttcctctgcgtccctctttgcc
SRR511917.6944891.2-                   TACTTCTCCgtaagttgaaaagacttcctctgcgtccctctttgcc
SRR511917.6945149.2-                   TACTTCTCCgtaagttgaaaagacttcctctgcgtccctctttgcc
SRR516970.33072342.1+                  TACTTCTCCgtaagttgaaaagacttcctctgcgtccctctttgcc
SRR516972.53884420.2+                  TACTTCTCCgtaagttgaaaagacttcctctgcgtccctctttgcc
SRR516974.31677643.2+                  TACTTCTCCgtaagttgaaaagacttcctctgcgtccctctttgcc
SRR516976.57275907.1-                  TACTTCTCCgtaagttgaaaagacttcctctgcgtccctctttgcc
SRR516987.54654380.2+                  TACTTCTCCgtaagttgaaaagacttcctctgcgtccctctttgcc
SRR516999.47022405.1+                  TACTTCTCCgtaagttgaaaagacttcctctgcgtccctctttgcc
SRR516999.59041365.2+                  TACTTCTCCgtaagttgaaaagacttcctctgcgtccctctttgcc
SRR516999.75152025.1+                  TACTTCTCCgtaagttgaaaagacttcctctgcgtccctctttgcc
SRR517004.3551177.1+                   TACTTCTCCgtaagttgaaaagacttcctctgcgtccctctttgcc
SRR517008.50269025.2-                  TACTTCTCCgtaagttgaaaagacttcctctgcgtccctctttgcc
SRR517009.53765195.1+                  TACTTCTCCgtaagttgaaaagacttcctctgcgtccctctttgcc
SRR517009.59451816.1+                  TACTTCTCCgtaagttgaaaagacttcctctgcgtccctctttgcc
SRR516967.29450276.2+                   ACTTCTCCgtaagttgaaaagacttcctctgcgtccctctttgcc
SRR516968.164894710.2-                  ACTTCTCCgtaagttgaaaagacttcctctgcgtccctctttgcc
SRR516989.86009538.1-                   ACTTCTCCgtaagttgaaaagacttcctctgcgtccctctttgcc
SRR516994.46844887.1+                   ACTTCTCCgtaagttgaaaagacttcctctgcgtccctctctgcc
SRR517002.59854150.2+                   ACTTCTCCgtaagttgaaaagacttcctctgcgtccctctttgcc
SRR517002.81662769.2-                   ACTTCTCCgtaagttgaaaagacttcctctgcgtccctctttgcc
SRR517007.71163816.2-                   ACTTCTCCgtaagttgaaaagacttcctctgcgtccctctttgcc
SRR511911.59626526.1-                    CTTCTCCgtaagttgaaaagacttcctctgcgtccctctttgcc
SRR516968.100131508.1-                   CTTCTCCgtaagttgaaaagacttcctctgcgtccctctttgcc
SRR516976.566385.2+                      CTTCTCCgtaagttgaaaagacttcctctgcgtccctctttgcc
SRR516977.74407410.2+                    CTTCTCCgtaagttgaaaagacttcctctgcgtccctctttgcc
SRR516978.48433823.1-                    CTTCCCCgtaagttgaaaagtcttcctctgcggccctctttgcc
SRR516991.23511807.2-                    CTTCTCCgtaagttgaaaagacttcctctgcgtccctctttgcc
SRR516998.33244450.1+                    CTTCTCCgtaagttgaaaagacttcctctgcgtccctctttgcc
SRR516999.73622205.2-                    CTTCTCCgtaagttgaaaagacttcctctgcgtccctctttgcc
SRR517001.40482893.2-                    CTTCTCCgtaagttgaaaagacttcctctgcgtccctctttgcc
SRR517006.65871668.2-                    CTTCTCCgtaagttgaaaagacttcctctgcgtccctctttgcc
SRR511915.40385307.2-                     TTCTCCggaagtttaaaagacttcctctgcgtccctctttgcc
SRR516967.100239201.2-                    TTCTCCgtaagttgaaaagacttcctctgcgtccctctttgcc
SRR516990.13824974.1+                     TTCTCCgtaagttgaaaagacttcctctgcgtccctctttgcc
SRR516996.2981174.1+                      TTCTCCgtaagttgaaaagacttcctctgcgtccctctttgcc
SRR516997.43055747.1+                     TTCTCCgtaagttgaaaagacttcctctgcgtccctctttgcc
SRR516997.94332852.1+                     TTCTCCgtaagttgaaaagacttcctctgcgtccctctttgcc
SRR517001.27493794.1-                     TTCTCCgtaagttgaaaagacttcctctgcgtccctctttgcc
SRR517003.72383496.2-                     TTCTCCgtaagttgaaaagacttcctctgcgtccctctttgcc
SRR517007.13407817.2+                     TTCTCCgtaagttgaaaagacttcctctgcgtccctctttgcc
SRR517007.71081447.1+                     TTCTCCgtaagttgaaaagacttcctctgcgtccctctttgcc
SRR511912.44583981.1+                      TCTCCgtaagttgaaaagacttcctctgcgtccctctttgcc
SRR516967.96216344.2-                      TCTCCgtaagttgaaaagacttcctctgcgtccctctttgcc
SRR516968.106287833.2+                     TCTCCgtaagttgaaaagacttcctctgcgtccctctttgcc
SRR516968.141834453.1+                     TCTCCgtaagttgaaaagacttcctctgcgtccctctttgcc
SRR516973.21141262.2-                      TCTCCgtaagttgaaaagacttcctctgcgtccctctttgcc
SRR516992.52058169.1-                      TCTCCgtaagttgaaaagacttcctctgcgtccctctttgcc
SRR516993.80607750.2-                      TCTCCgtaagttgaaaagacttcctctgcgtccctctttgcc
SRR517002.80001098.1-                      TCTCCgtaagttgaaaagacttcctctgcgtccctctttgcc
SRR517003.32031210.1-                      TCTCCgtaagttgaaaagacttcctctgcgtccctctttgcc
SRR517005.15953074.2-                      TCTCCgtaagttgaaaagacttcctctgcgtccctctttgcc
SRR517009.41949548.2-                      TCTCCgtaagttgaaaaggcttcctctgcgtccctctttgcc
SRR511911.53566593.1+                       CTCCgtaagttgaaaagacttcctctgcgtccctctttgcc
SRR516967.56639881.2-                       CTCCgtaagttgaaaagacttcctctgcgtccctctttgcc
SRR516979.32114585.1+                       CTCCgtaagttgaaaagacttcctctgcgtccctctttgcc
SRR516980.14547445.1-                       CTCCgtaagttgaaaagacttcctctgcgtccctctttgcc
SRR516982.65045018.2+                       CTCCgtaagttgaaaagacttcctctgcgtccctctttgcc
SRR516997.20173366.1+                       CTCCgtaagttgaaaagacttcctctgcgtccctctttgcc
SRR517009.32201735.1+                       CTCCgtaagttgaaaagacttcctctgcgtccctctttgcc
```

```
SRR516977.31933289.1-                        TCCgtaagttgaaaagacttcctctgcgtccctctttgcc
SRR516979.3253001.2-                         TCCgtaagttgaaaagacatcctctgcgtccctctttgcc
SRR516994.12728631.1-                        TCCgtaagttgaaaagacttcctctgcgtccctctttgcc
SRR516995.57402881.1-                        TCCgtaagttgaaaagacttcctctgcgtccctctttgcc
SRR516995.9806597.1+                         TCCgtaagttgaaaagacttcctctgcgtccctctttgcc
SRR516998.90514942.1+                        TCCgtaagttgaaaagacttcctctgcgtccctctttgcc
SRR516999.56852286.2-                        TCCgtaagttgaaaagacttcctctgcgtccctctttgcc
SRR516999.85004778.2+                        TCCgtaagttgaaaagacttcctctgcgtccctctttgcc
SRR517006.1651318.2+                         TCCgtaagttgaaaagacttcctctgcgtccctctttgcc
SRR511914.42597042.2+                         CCgtaagttgaaaagacttcctctgcgtccctctttgcc
SRR516971.6357400.1+                          CCgtaagttgaaaagacttcctctgcgtccctctttgcc
SRR516979.20038553.2-                         CCgtaagttgaaaagacttcctctgcgtccctctttgcc
SRR516981.40380711.2-                         CCgtaagttgaaaagacttcctctgcgtccctctttgcc
SRR517000.42965244.2-                         CCgtaagttgaaaagacttcctctgcgtccctctttgcc
SRR517002.50588676.1-                         CCgtaagttgaaaagacttcctctgcgtccctctttgcc
SRR511911.11918542.1-                          Cgtaagttgaaaagacttcctctgcgtccctctttgcc
SRR511914.45816401.1-                          Cgtaagttgaaaagacttcctctgcgtccctctttgcc
SRR516976.36662272.1-                          Cgtaagttgaaaagacttcctctgcgtccctctttgcc
SRR516979.61310102.2+                          Cgtaagttgaaaagacttcctctgcgtccctctttgcc
SRR516979.79499176.2+                          Cgtaagttgaaaagacttcctctgcgtccctctttgcc
SRR516982.7524222.1-                           Cgtaagttgaaaagacttcctctgcgtccctctttgcc
SRR516989.86262903.2+                          Cgtaagttgaaaagacttcctctgcgtccctctttgcc
SRR516994.68821829.1-                          Cgtaagttgaaaagacttcctctgcgtccctctttgcc
SRR517001.29555002.2+                          Cgtaagttgaaaagacttcctctgcgtccctctttgcc

consensus                CCAATGGCAGCATCTACTTCTCCgtaagttgaaaagacttcctctgcgtccctctttgcc
```

I. Yellow-throated sandgrouse (No. 48), exon 1, WGS data

- CAP3 alignment of WGS data spanning exon 1 (uppercase letters)

```
                             .    :    .    :    .    :    .    :    .    :    .    :
SRR959570.27243122.2-    gtatcagcaagactcagaaactgccgtggtcctgttgA
SRR959570.83928202.2+    gtatcagcaagactcagaaactgacgtggtcctgttgA
SRR959570.77484672.1+    gtatcagcaagactcagaaactgccgtggtcctgttgAT
SRR959570.70261024.2-    gtatcagcaagactcagaaactgccgtggtcctgttgATGA
SRR959569.58422157.1-    gtatcagcaagactcagaaactgccgtggtcctgttgATGATG
SRR959570.81822031.2+    gtatcagcaagactcagaaactgccgtggtcctgttgATGATGGCAG
SRR959570.23954372.1-    gtatcagcaagactcagaaactgccgtggtcctgttgATGATGGCAGTTG
SRR959570.79408794.2-    gtatcagcaagactcagaaactgccgtggtcctgttgATGATGGCAGTTG
SRR959570.96342072.2-    gtatcagcaagactcagaaactgccgtggtcctgttgATGATGGCAGTTGTCTTCT
SRR959569.6612332.2+     gtatcagcaagactcagaaactgccgtggtcctgttgATGATGGCAGTTGTCTTCTCA
SRR959569.35456521.1-    gtatcagcaagactcagaaactgccgtggtcctgttgATGATGGCAGTTGTCTTCTCAA
SRR959570.40418354.1+    gtatcagcaagactcagaaactgccgtggtcctgttgATGATGGCAGTTGTCTTTTCAA
SRR959569.108977722.1-   gtatcagcaagactcagaaactgccgtggtcctgttgATGATGGCAGTTGTCTTCTCAAG
SRR959569.115141092.1-   gtatcagcaagactcagaaactgccgtggtcctgttgATGATGGCAGTTGTCTTCTCAAG
SRR959569.26703424.2-    gtatcagcaagactcagaaactgccgtggtcctgttgATGATGGCAGTTGTCTTCTCAAG
SRR959569.31567924.1+    gtatcagcaagactcagaaactgtcgtggtcctgttgATGATGGCAGTTGTCTTCTCAAG
SRR959569.46846859.1-    gtatcagcaagactcagaaactgccgtggtcctgttgATGATGGCAGTTGTCTTCTCAAG
SRR959569.70471046.2-    gtatcagcaagactcagaaactgccgtggtcctgttgATGATGGCAGTTGTCTTCTCAAG
SRR959569.72650966.1+    gtatcagcaagactcagaaactgccgtggtcctgttgATGATGGCAGTTGTCTTCTCAAG
SRR959569.88034369.1-    gtatcagcaagactcagaaactgccgtggtcctgttgATGATGGCAGTTGTCTTCTCAAG
SRR959570.30788134.1+    gtatcagcaagactcagaaactgccgtggtcctgttgATGATGGCAGTTGTCTTCTCAAG
SRR959570.31408696.1+    gtatcagcaagactcagaaactgccgtggtcctgttgATGATGGCAGTTGTCTTCTCAAG
SRR959570.41706938.1-    gtatcagcaagactcagaaactgccgtggtcctgttgATGATGGCAGTTGTCTTCTCAAG
SRR959570.62371452.2-    gtatcagcaagactcagaaactgccgtggtcctgttgATGATGGCAGTGGTCTTCTCAAG
SRR959570.77329700.1-    gtatcagcaagactcagaaactgccgtggtcctgttgATGATGGCAGTTGTCTTCTCAAG
SRR959570.78563097.1-    gtatccacaagactcagaaactgccgtggtcctgttgATGATGGCAGTTGTCTTCTCAAG
SRR959570.84201608.2+    gtatcagcaagactcagaaactgccgtggtcctgttgATGATGGCAGTTGTCTTCTCAAG
SRR959570.89081045.1+    gtatcagcaagactcagaaactgccgtggtcctgatgATGATGGCAGTTGGCTTCTCAAG
SRR959569.36617306.2-        cagcaagactcagaaactgccgtggtcctgttgATGATGGCAGTTGTCTTCTCAAG
SRR959570.23791432.2+        cagcaagactcagaaactgccgtggtcctgttgATGATGGCAGTTGTCTTCTCAAG
SRR959569.92007559.2-         agcaagactcagaaactgccgtggtcctgttgATGATGGCAGTTGTCTTCTCAAG
SRR959569.33378889.1-               actcagaaactgccgtggtcctgttgATGATGGCAGTTGTCTTCTCAAG
SRR959570.74269683.1+                      aactgccgtggtcctgttgATGATGGCAGTTGTCTTCTCAAG
SRR959569.111712080.2-                       ctgccgtggtcctgttgATGATGGCAGTTGTCTTCTCAAG
SRR959569.21591540.2-                            cgtggtcctgttgATGATGGCAGTTGTCTTCTCAAG
SRR959569.22505139.2+                                   ctgttgATGATGGCAGTTGTCTTCTCAAG
SRR959570.67053970.2+                                    tgttgATGATGGCAGTTGTCTTCTCAAG
SRR959569.66350666.2+                                       tgATGATGGCAGTTGTCTTCTCAAG
SRR959569.81857411.2+                                        gATGATGGCAGTTGTCTTCTCAAG
SRR959570.45570120.2+                                         ATGATGGCAGTTGTCTTCTCAAG
SRR959570.58913007.1+                                            ATGGCAGTTGTCTTCTCAAG
SRR959570.67297812.2+                                                  GTTGTCTTCTCAAG
SRR959569.41410579.1-                                                        TTCTCAAG
SRR959569.90980180.1+                                                         TCTCAAG
SRR959569.23996698.2+                                                             AAG
SRR959570.43058119.1-                                                              AG
SRR959570.48604510.2-                                                              AG
SRR959569.50804322.2+                                                               G
                         ____________________________________________________________
consensus                gtatcagcaagactcagaaactgccgtggtcctgttgATGATGGCAGTTGTCTTCTCAAG

                             .    :    .    :    .    :    .    :    .    :    .    :
SRR959569.88034369.1-    AACCA
SRR959569.31567924.1+    AAACAAGGGTA
SRR959570.77329700.1-    AACCAAGGGTATG
SRR959570.78563097.1-    AACCAAGGGTATGCT
SRR959570.89081045.1+    AAACAAGGGTATGCTCTTC
SRR959570.30788134.1+    AACCAAGGGTATGCTCTTCC
SRR959570.62371452.2-    AACCAAGGGTATGCTCTTCCTCTTTTC
SRR959570.31408696.1+    AACCAAGGGTATGCTCTTACTCCTTTCT
SRR959570.41706938.1-    AACCAAGGGTATGCTCTTCCTCTTTTCTCCC
SRR959569.26703424.2-    AACCAAGGGTATGCTCTCCCTCTTTTCTCCCCA
SRR959569.70471046.2-    AACCAAGGGTATGCTCTTCCTCTTTTCTCCCCA
SRR959569.46846859.1-    AACCAAGGGTATGCTCTTCCTCTTTTCTCCCCAT
SRR959569.108977722.1-   AACCAAGGGTATGCTCTTCCTCTTTTCTCCCCATG
SRR959570.84201608.2+    AACCAAGGGTATGCTCTTCCTCTTTTCTCCCCATG
SRR959569.115141092.1-   AACCAAGGGTATGCTCTTCCTCTTTTCTCCCCATGCTTTT
SRR959569.36617306.2-    AACCAAGGGTATGCTCTTCCTCTTTTCTCCCCATGCTTTTATTA
SRR959570.23791432.2+    AACCAAGGGTATGCTCTTCCTCTTTTCTCCCCATGCTTTTATTA
SRR959569.92007559.2-    AACCAAGGGTATGCTCTTCCTCTTTTCTCCCCATGCTTTTATTAT
SRR959569.33378889.1-    AACCAAGGGTATGCTCTTCCTCTTTTCTCCCCATGCTTTTATTATGATCAG
SRR959570.74269683.1+    AACCAAGGGTATGCTCTTCCTCTTTTCTCCCCATGCTTTTATTATGATCAGCCTGCAA
SRR959569.111712080.2-   AACCAAGGGTATGCTCTTCCTCTTTTCTCCCCATGCTTTTATTATGATCAGCCTGCAACT
SRR959569.21591540.2-    AACCAAGGGTATGCTCTTCCTCTTTTCTCCCCATGCTTTTATTATGATCAGCCTGCAACT
SRR959569.22505139.2+    AACCAAGGGTATGCTCTTCCTCTTTTCTCCCCATGCTTTTATTATGATCAGCCTGCAACT
```

```
SRR959569.23996698.2+    AACCAAGGGTATGCTCTTCCTCTTTTCTCCCCATGCTTTTATTATGATCAGCCTGCAACT
SRR959569.41410579.1-    AACCAAGGGTATGCTCTTCCTCTTTTCTCCCCATGCTTTTATTATGATCAGCCTGCAACT
SRR959569.50804322.2+    AACCAAGGGTATGCTCTTCCTCTTTTCTCCCCATGCTTTTATTATGATCAGCCTGCAACT
SRR959569.66350666.2+    AACCAAGGGTATGCTCTTCCTCTTTTCTCCCCATGCTTTTATTATGATCAGCCTGCAACT
SRR959569.81857411.2+    AACCAAGGGTATGCTCTTCCTCTTTTCTCCCCATGCTTTTATTATGATCAGCCTGCAACT
SRR959569.90980180.1+    AACCAAGGGTATGCTCTTCCTCTTTTCTCCCCATGCTTTTATTATGATCAGCCTGCAACT
SRR959570.43058119.1-    AACCAAGGGTATGCTCTTCCTCTTTTCTCCCCATGCTTTTATTATGATCAGCCTGCAACT
SRR959570.45570120.2+    AACCAAGGGTATGCTCTTCCTCTTTTCTCCCCATGCTTTTATTATGATCAGCCTGCAACT
SRR959570.48604510.2-    AACCAAGGGTATGCTCTTCCTCTTTTCTCCCCATGCTTTTATTATGATCAGCCTGCAACT
SRR959570.58913007.1+    AACCAAGGGTATGCTCTTCCTCTTTTCTCCCCATGCTTTTATTATGATCAGCCTGCAACT
SRR959570.67053970.2+    AACCAAGGGTATGCTCTTCCTCTTTTCTCCCCATGCTTTTATTATGATCAGCCTGCAACT
SRR959570.67297812.2+    AACCAAGGGTATGCTCTTCCTCTTTTCTCCCCATGCTTTTATTATGATCAGCCTGCAACT
SRR959569.60776346.2-     ACCAAGGGTATGCTCTTCCTCTTTTCTCCCCATGCTTTTATTATGATCAGCCTGCAACT
SRR959569.70618318.1+              ATGCTCTTCCTCTTTTCTCCCCATGCTTTTATTATGATCAGCCTGCAACT
SRR959570.81105272.1-                         CTTTTCTCCCCATGCTTTTATTAGGATCAGCCTGCAACT
SRR959569.53622134.2-                           TTTCTCCCCATGCTTTTATTATGATCAGCCTGCAACT
SRR959569.83569668.1-                               TCCCCATGCTTTTATTATGATCAGCCTGCAACT
SRR959569.83588450.2+                                CCCCATGCTTTTATTATGATCAGCCTGCAACT
SRR959569.3519785.1+                                       GCTTTTATTATGATCAGCCTGCAACT
SRR959569.78006194.2+                                      GCTTTTATTATGATCAGCCTGCAACT
SRR959569.39372777.1+                                       CTTTTATTATGATCAGCCTGCAACT
SRR959570.51090830.1+                                       CTTTTATTATGATCAGCCTGCAACT
SRR959569.57069922.1-                                           TATTATGATCAGCCTGCAACT
SRR959569.1601221.1+                                               TATGATCAGCCTGCAACT
SRR959569.71807934.1-                                                TGATCAGCCTGCAACT
SRR959570.55820506.1+                                                  ATCAGCCTGCAACT
SRR959569.54283800.1-                                                   TCAGCCTGCAACT
SRR959570.8382473.1+                                                        CCTGCAACT
SRR959569.8323748.2-                                                         CTGCAACT
SRR959569.59129649.1-                                                         TGCACCT
SRR959570.88689336.1+                                                            AACT
SRR959569.77647544.1-                                                               T

consensus                AACCAAGGGTATGCTCTTCCTCTTTTCTCCCCATGCTTTTATTATGATCAGCCTGCAACT

                             .    :    .    :    .    :    .    :    .    :    .    :
SRR959569.21591540.2-    CCAT
SRR959569.22505139.2+    CCATAGCTGCG
SRR959570.67053970.2+    CCATAGCTGCGT
SRR959569.66350666.2+    CCATAGCTGCGTTTC
SRR959569.81857411.2+    CCATAGCTGCGTTTCT
SRR959570.45570120.2+    CCATAGCTGCGTTTCTC
SRR959570.58913007.1+    CCATAGCTGCGTTTCTCCAT
SRR959570.67297812.2+    CCATAGCTGCGTTTCTCCATCTTCCT
SRR959569.41410579.1-    CCATAGCTGCGTTTCTCCATCTTCCTGGATCC
SRR959569.90980180.1+    CCATAGCTGCGTTTCTCCATCTTCCTGGATCCT
SRR959569.23996698.2+    CCATAGCTGCGTTTCTCCATCTTCCTGGATCCTTCAA
SRR959570.43058119.1-    CCATAGCTGCGTTTCTCCATCTTCCTGGATCCTTCAAA
SRR959570.48604510.2-    CCATAGCTGCGTTTCTCCATCTTCCTGGATCCTTCAAA
SRR959569.50804322.2+    CCATAGCTGCGTTTCTCCATCTTCCTGGATCCTTCAAAC
SRR959569.60776346.2-    CCATAGCTGCGTTTCTCCATCTTCCTGGATCCTTCAAACAT
SRR959569.70618318.1+    CCATAGCTGCGTTTCTCCATCTTCCTGGATCCTTCAAACATGGTCTACCT
SRR959569.1601221.1+     CCATAGCTGCGTTTCTCCATCTTCCTGGATCCTTCAAACATGGTCTACCTCCGCTGGGAC
SRR959569.3519785.1+     CCATAGCTGCGTTTCTCCATCTTCCTGGATCCTTCAAACATGGTCTACCTCCGCTGGGAC
SRR959569.39372777.1+    CCATAGCTGCGTTTCTCCATCTTCCTGGATCCTTCAAACATGGTCTACCTCCGCTGGGAC
SRR959569.53622134.2-    CCATAGCTGCGTTTCTCCATCTTCCTGGATCCTTCAAACATGGTCTACCTCCGCTGGGAC
SRR959569.54283800.1-    CCATAGCTGCGTTTCTCCATCTTCCTGGATCCTTCAAACATGGTCTACCTCCGCTGGGAC
SRR959569.57069922.1-    CCATAGCTGCGTTTCTCCATCTTCCTGGATCCTTCAAACATGGTCTACCTCCGCTGGGAC
SRR959569.59129649.1-    CCATAGCTGCGTTTCTCCATCTTCCTGGATCCTTCAAACATGGTCTACCTCCGCTGGGAC
SRR959569.71807934.1-    CCATAGCTGCGTTTCTCCATCTTCCTGGATCCTTCAAACATGGTCTACCTCCGCTGGGAC
SRR959569.77647544.1-    CCATAGCTGCGTTTCTCCATCTTCCTGGATCCTTCAAACATGGTCTACCTCCGCTGGGAC
SRR959569.78006194.2+    CCATAGCTGCGTTTCTCCATCTTCCTGGATCCTTCAAACATGGTCTACCTCCGCTGGGAC
SRR959569.8323748.2-     CCATAGCTGCGTTTCTCCATCTTCCTGGATCCTTCAAACATGGTCTACCTCCGCTGGGAC
SRR959569.83569668.1-    CCATAGCTGCGTTTCTCCATCTTCCTGGATCCTTCAAACATGGTCTACCTCCGCTGGGAC
SRR959569.83588450.2+    CCATAGCTGCGTTTCTCCATCTTCCTGGATCCTTCAAACATGGTCTACCTCCGCTGGGAC
SRR959570.51090830.1+    CCATAGCTGCGTTTCTCCATCTTCCTGGATCCTTCAAACATGGTCTACCTCCGCTGGGAC
SRR959570.55820506.1+    CCATAGCTGCGTTTCTCCATCTTCCTGGATCCTTCAAACATGGTCTACCTCCGCTGGGAC
SRR959570.81105272.1-    CCATAGCTGCGTTTCTCCATCTTCCTGGATCCTTCAAACATGGTCTACCTCCGCTGGGAC
SRR959570.8382473.1+     CCATAGCTGCGTTTCTCCATCTTCCTGGATCCTTCAAACATGGTCTACCTCCGCTGGGAC
SRR959570.88689336.1+    CCATAGCTGCGTTTCTCCATCTTCCTGGATCCTTCAAACATGGTCTACCTCCGCTGGGAC
SRR959569.64452302.2+       TAGCTGCGTTTCTCCATCTTCCTGGATCCTTCAAACATGGTCTACCTCCGCTGGGAC
SRR959570.34914624.2+        AGCTGCGTTTCTCCATCTTCCTGGATCCTTCAAACATGGTCTACCTCCGCTGGGAC
SRR959569.15507308.1+         GCTGCGTTTCTCCATCTTCCTGGATCCTTCAAACATGGTCTACCTCCGCTGGGAC
SRR959569.91265373.1-             CGTTTCTCCATCTTCCTGGATCCTTCAAACATGGTCTACCTCCGCTGGGAC
SRR959569.50076958.1-               TTTCTCCATCTTCCTGGATCCTTCAAACATGGTCTACCTCCGCTGGGAC
SRR959569.96465996.1+               TTTCTCCATCTTCCTGGATCCTTCAAACATGGTCTACCTCCGCTGGGAC
SRR959570.70417815.2-                     CATCTTCCTGGATCCTTCAAACATGGTCTACCTCCGCTGGGAC
SRR959570.86361899.2-                                      CAAACATGGTCTACCTCCGCTGGGAC
SRR959569.109876129.2+                                      AAACATGGTCTACCTCCGCTGGGAC
SRR959569.37871094.2-                                              GTCTACCTCCGCTGGGCC
SRR959569.34552194.2+                                                      CCGCTGGGAC
SRR959569.37295845.2-                                                          TGGGCC
SRR959569.111383851.2+                                                          GGGAC
SRR959569.11381534.2-                                                            GGAC
```

```
SRR959569.98266262.2-                                                               C
                         ____________________________________________________________
consensus                CCATAGCTGCGTTTCTCCATCTTCCTGGATCCTTCAAACATGGTCTACCTCCGCTGGGAC

                             .    :    .    :    .    :    .    :    .    :    .    :
SRR959570.81105272.1-    C
SRR959569.53622134.2-    CAT
SRR959569.83569668.1-    CATGACG
SRR959569.83588450.2+    CATGACGA
SRR959569.3519785.1+     CATGACGAACAGGA
SRR959569.78006194.2+    CATGACGAACAGGA
SRR959569.39372777.1+    CATGACGAACAGGAG
SRR959570.51090830.1+    CATGACGAACAGGAG
SRR959569.57069922.1-    CATGACGAACAGGAGCTGA
SRR959569.1601221.1+     CATGACGAACAGGAGCTGATGA
SRR959569.71807934.1-    CATGACGAACAGGAGCTGATGAAG
SRR959570.55820506.1+    CATGACGAACAGGAGCTGATGAAGTT
SRR959569.54283800.1-    CATGACGAACAGGAGCTGATGAAGTTT
SRR959570.8382473.1+     CATGACGAACAGGAGCTGATGAAGTTTGAGC
SRR959569.8323748.2-     CATGACGAACAGGAGCTGATGAAGTTTGAGCT
SRR959569.59129649.1-    CATGACGAACAGGAGCTGATGAAGTTTGAGCTG
SRR959570.88689336.1+    CATGACGAACAGGAGCTGATGAAGTTTGAGCTGC
SRR959569.77647544.1-    CATGACGAACAGGAGCTGATGAAGTTTGAGCTGCAGGTC
SRR959569.50076958.1-    CATGACGAACAGGAGCTGATGAAGTTTGAGCTGCAGGTCCATA
SRR959569.64452302.2+    CATGACGAACAGGAGCTGATGAAGTTTGAGCTGCAGGTCCATA
SRR959570.34914624.2+    CATGACGAACAGGAGCTGATGAAGTTTGAGCTGCAGGTCCATAC
SRR959569.15507308.1+    CATGACGAACAGGAGCTGATGAAGTTTGAGCTGCAGGTCCATACG
SRR959569.91265373.1-    CATGACGAACAGGAGCTGATGAAGTTTGAGCTGCAGGTCCATACGACTG
SRR959569.96465996.1+    CATGACGAACAGGAGCTGATGAAGTTTGAGCTGCAGGTCCATACGACTGGC
SRR959570.70417815.2-    CATGACGAACAGGAGCTGATGAAGTTTGAGCTGCAGGTCCATACGACTGGCTGGGTG
SRR959569.111383851.2+   CATGACGAACAGGAGCTGATGAAGTTTGAGCTGCAGGTCCATACGACTGGCTGGGTGGC
SRR959569.109876129.2+   CATGACGAACAGGAGCTGATGAAGTTTGAGCTGCAGGTCCATACGACTGGCTGGGGGGCA
SRR959569.11381534.2-    CCTGACGAACAGGAGCTGATGAAGTGTGAGCTGCAGGTCCATACGACTGGCTGGGCGGCA
SRR959569.34552194.2+    CATGACGAACAGGAGCTGATGAAGTTTGAGCTGCAGGTCCATACGACTGGCTGGGTGGCC
SRR959569.37295845.2-    CATGACGAACAGGAGCTGATGAAGTTTGAGCTGCAGGTCCATACGACTGGCTGGGTGGCA
SRR959569.37871094.2-    CATGACGAACAGGAGCTGATGAAGTTTGAGCTGCAGGTCCATACGACTGGCTGGGTGGCA
SRR959569.98266262.2-    CATGACGAACAGGAGCTGATGAAGTTTGAGCTGCAGGTCCATACGACTGGCTGGGTGGCA
SRR959570.86361899.2-    CATGACGAACAGGAGCTGATGAAGTTTGAGCTGCAGGTCCATACGACTGGCTGGGTGGCA
SRR959570.87431180.2+      TGACGAACAGGAGCTGATGAAGTTTGAGCTGCAGGTCCATACGACTGGCTGGGGGGCA
SRR959569.112410249.1-        CGAAAAGGAGCTGATGACGTTTGAGCTTCAGGTCCATACGACTGGCTGGGTGGCG
SRR959569.74320502.1-          GAACAGGAGCTGATGAAGTTTGAGCTGCAGGTCCATACGACTGGCTGGGTGGCA
SRR959569.23884205.2-            ACAGGAGCTGATGAAGTTTGAGCTGCAGGTCCATACGACTGGCTGGGTGGCA
SRR959570.65746827.1-                            TTTGAGCTGCAGGGCCATACGACTGGCTGGGTGGCA
SRR959570.96704518.1-                             TTGAGCTGCAGGTCCATACGACTGGCTGGGTGGCA
SRR959569.102843663.1+                             TGAGCTGCAGGTCCATACGACTGGCTGGGTGGCA
SRR959570.83592428.1-                               GAGCTGCAGGTCCATACGACTGGCTGGGTGGCA
SRR959570.55184012.1-                                AGCTGCAGGTCCATACGACTGGCTGGGTGGCA
SRR959569.62074445.2-                                     CAGGTCCATACGACTGGCTGGGTGGCA
SRR959569.103091153.1-                                                      TGGGTGGCA
SRR959569.91923741.1-                                                        GGGTGGTA
                         ____________________________________________________________
consensus                CATGACGAACAGGAGCTGATGAAGTTTGAGCTGCAGGTCCATACGACTGGCTGGGTGGCA

                             .    :    .    :    .    :    .    :    .    :    .    :
SRR959570.86361899.2-    TTTGGATTCAGCC
SRR959569.37871094.2-    TTTGGATTCAGCCCTCACGGAG
SRR959569.11381534.2-    TTTGGATTCAGCCCTCACGGAGAATT
SRR959569.34552194.2+    TTTGGATTCAGCCCTCACGGAGAATTG
SRR959569.37295845.2-    TTTGGATTCAGCCCTCACGGAGAATTGCCTGGAT
SRR959569.112410249.1-   TTTGGATTCAGCCCTCACGGAGAATTGCCTGGATCTG
SRR959570.87431180.2+    TTTGGATTTAGCCCTCACGGAGAATTGCCTGGGTCTG
SRR959569.98266262.2-    TTTGGATTCAGCCCTCACGGAGAATTGCCTGGATCTGAT
SRR959569.74320502.1-    TTTGGATTCAGCCCTCACGGAGAATTGCCTGGATCTGATACTGTGA
SRR959569.23884205.2-    TTTGGATTCAGCCCTCACGGAGAATTGCCTGGATCTGATACTGTGATA
SRR959569.102843663.1+   TTTGGATTCAGCCCTCACGGAGAATTGCCTGGATCTGATACTGTGATAGGAGGGGTCTTC
SRR959569.103091153.1-   TTTGGATTCAGCCCTCACGGAGAATTGCCTGGATCTGATACTGTGATAGGAGGTGTCTTC
SRR959569.62074445.2-    TTTGGATTCAGCCCTCACGGAGAATTGCCTGGATCTGATACTGTGATAGGAGGTGTCTTC
SRR959569.91923741.1-    TTTGGATTCAGCCCTCAGGGAAAATTGCCTGGATCTGCTACTGTGATAGGAGGTGTCTTC
SRR959570.25763169.2+    TTTGGATTCAGCCCTCACGGAGAATTGCCTGGATCTGATACTGTGATAGGAGGTGTCTTC
SRR959570.55184012.1-    TTTGGATTCAGCCCTCACGGAGAATTGCCTGGATCTGATACTGTGATAGGAGGTGTCTTC
SRR959570.65746827.1-    TTTGGATTCAGCCCTCACGGAGAATTGCCTGGATCTGATACTGTGATAGGAGGTGTCTTC
SRR959570.83592428.1-    TTTGGATTCAGCCCTCACGGAGAATTGCCTGGATCTGATACTGTGATAGGAGGTGTCTTC
SRR959570.96704518.1-    TTTGGATTCAGCCCTCACGGAGAATTGCCTGGATCTGATACTGTGATAGGAGGTGTCTTC
SRR959570.96400377.1-     TTGGATTCAGCCCTCACGGAGAATTGCCTGGATCTGATACTGTGATAGGAGGTGTCTTC
SRR959570.74431605.2+      TGGATTCAGCCCTCACGGAGAATTGCCTGGATCTGATACTGTGATAGGAGGTGTCTTC
SRR959569.81204863.2-                     CGGAGAATTGCCTGGATCTGATACTGTGATAGGAGGTGTCTTC
SRR959569.40343999.2+                      GGAGAATTGCCTGGATCTGATACTGTGATAGGAGGTGTCTTC
SRR959569.62656636.1-                            TTGCCTGGATCTGATACTGTGATAGGAGGTGTCTTC
SRR959570.41687226.1-                                      CTGATACTGTGATAGGAGGTGTCTTC
SRR959569.10170699.2+                                           ACTGTGATAGGAGGTGTCTTC
SRR959569.14787473.2-                                             TGTGATAGGAGGTGTCTTC
SRR959569.109491793.2-                                             GTGATAGGAGGTGTCTTC
SRR959569.88589827.2+                                                 ATAGGAGGTGTCTTC
SRR959569.115941289.1+                                                 TAGGAGGTGTCTTC
```

```
SRR959569.69829492.1-                                                       GGTGTCTTC
SRR959570.81175104.2+                                                           TCTTC
SRR959569.8173288.2+                                                                C

consensus                TTTGGATTCAGCCCTCACGGAGAATTGCCTGGATCTGATACTGTGATAGGAGGTGTCTTC

                             .    :    .    :    .    :    .    :    .    :    .    :
SRR959569.102843663.1+   CC
SRR959570.65746827.1-    CCAA
SRR959570.96704518.1-    CCAAA
SRR959570.55184012.1-    CCAAATA
SRR959570.83592428.1-    CCAAATA
SRR959569.62074445.2-    CCAAATAGCAGCA
SRR959569.103091153.1-   CCAAATAGCAGCATCTACTTCTCTgtaagct
SRR959569.91923741.1-    CCAAATGGCAGCGTCTACTTCTCTgtaagctg
SRR959570.25763169.2+    CCAAATAGCAGCATCTACTTCTCTgtaagctgaaaaaact
SRR959570.96400377.1-    CCAAATAGCAGCATCTACTTCTCTgtaagctgaaaaaactt
SRR959570.74431605.2+    CCAAATAGCAGCATCTACTTCTCTgtaagctgaaaaaacttc
SRR959569.81204863.2-    CCAAATAGCAGCATCTACTTCTCTgtaagctgaaaaaacttcctctgca
SRR959569.40343999.2+    CCAAATAGCAGCATCTACTTCTCTgtaagctgaaaaaacttcctctgcatcctctttt
SRR959569.10170699.2+    CCAAATAGCAGCATCTACTTCTCTgtaagctgaaaaaacttcctctgcatcctcttttac
SRR959569.109491793.2-   CCAAATAGCAGCGTCTACTTCTCTgtaagctgaaaaaacttcctctgcatcctcttttac
SRR959569.115941289.1+   CCAAATAGCAGCGTCTACTTCTCTgtaagctgaaaaaacttcctctgcatcctcttttac
SRR959569.14787473.2-    CCAAATAGCAGCGTCTACTTCTCTgtaagctgaaaaaacttcctctgcatcctcttttac
SRR959569.62656636.1-    CCAAATAGCAGCGTCTACTTCTCTgtaagctgaaaaaacttcctctgcatcctcttttac
SRR959569.69829492.1-    CCAAATAGCAGCGTCTACTTCTCTgtaagctgaaaaaacttcctctgcatcctcttttac
SRR959569.8173288.2+     CCAAATAGCAGCGTCTACTTCTCTgtaagctgaaaaaacttcctctgcatcctcttttac
SRR959569.88589827.2+    CCAAATAGCAGCGTCTACTTCTCTgtaagctgaaaaaacttcctctgcatactcttttac
SRR959570.41687226.1-    CCAAATAGCAGCATCTACTTCTCTgtaagctgaaaaaacttcctctgcatcctcttttac
SRR959570.81175104.2+    CCAAATAGCAGCGTCTACTTCTCTgtaagctgaaaaaacttcctctgcatcctcttttac
SRR959570.19393360.1+         TAGCAGCATCTACTTCTCTgtaagctgaaaaaacttcctctgcatcctcttttac
SRR959570.19212778.2-           GCAGCATCTACTTCTCTgtaagctgaaaaaacttcctctgcatcctcttttac
SRR959570.77680066.1+            CAGCATCTACTTCTCTgtaagctgaaaaaacttcctctgcatcctcttttac
SRR959569.6612332.1-                   CTACTTCTCTgtaagctgaaaaaacttcctctgcatcctcttttac
SRR959570.23791432.1-                    ACTTCTCTgtaagctgaaaaaacttcctctgcatcctcttttac
SRR959570.22341937.1-                      TTCTCTgtaagctgaaagaacttcctctgcatcctcttttac
SRR959570.81919161.1-                        CTCTgtaagctgaaaaaacttcctctgcatcctcttttac
SRR959569.20978526.2+                         TCTgtaagctgaaaaaacttcctctgcatcctcttttac
SRR959570.28579282.2-                          CTgtaacctgaaaaaacttcctctgcatcctcttttac

consensus                CCAAATAGCAGCATCTACTTCTCTgtaagctgaaaaaacttcctctgcatcctcttttac
```

J. Little egret (No. 36), exon 3, WGS data

- CAP3 alignment of WGS data spanning exon 3 (uppercase letters)

```
                             .    :    .    :    .    :    .    :    .    :    .    :
SRR1144868.67845280.1+   gaataaaggagagtttccagggaatgttcccattactgA
SRR1144872.17734476.2+   gaataaaggagagtttccagtgaatgttcccattactgA
SRR1144879.33672987.2+   gaataaaggagagtttccagtgaatgttcccattactgA
SRR1144879.5160460.1+    gaataaaggagagtttccagtgaatgttcccattactgA
SRR1144879.61692482.1+   gaataaaggagagtttccagtgaatgttcccattactgA
SRR1144872.33824599.2-   gaataaaggagagtttccagtgaatgttcccattactgAT
SRR1144871.41481488.2-   gaataaaggagagtttccagtgaatgttcccattactgATG
SRR1144870.72294457.1-   gaataaaggagagtttccagtgaatgttcccattactgATGGA
SRR1144871.8299377.2-    gaataaaggagagtttccagtgaatgttcccattactgATGGA
SRR1144877.33448080.1+   gaataaaggagagtttccagtgaatgttcccattactgATGGA
SRR1144870.9639987.1-    gaataaaggagagtttccagtgaatgttcccattactgATGGATA
SRR1144872.16363319.1-   gaataaaggagagtttccagtgaatgttcccattactgATGGATAC
SRR1144872.4813573.1+    gaataaaggagagtttccagtgaatgttcccattactgATGGATAC
SRR1144878.41746520.1-   gaataaaggagagtttccagtgaatgttcccattactgATGGATAC
SRR1144879.62324334.1-   gaataaaggagagtttccagtgaatgttcccattactgATGGATACA
SRR1144872.45335912.1+   gaataaaggagagtttccagtgaatgttcccattactgATGGATACAG
SRR1144869.30903186.1+   gaataaaggagagtttccagtgaatgttcccattactgATGGATACAGC
SRR1144870.17119836.1+   gaataaaggagagtttccagtgaatgttcccattactgATGGATACAGCAC
SRR1144870.60844810.1-   gaataaaggagagtttccagtgaatgttcccattactgATGGATACAGCACACTTC
SRR1144869.67771093.2-   gaataaaggagagtttccagtgaatgttcccattactgATGGATACAGCACACTTCA
SRR1144870.31792783.1+   gaataaaggagagtttccagtgaatgttcccattactgATGGATACAGCACACTTCA
SRR1144868.59303752.1-   gaataaaggagagtttccagtgaatgttcccattactgATGGATACAGCACACTTCATTA
SRR1144868.60702162.1+   gaataaaggagagtttccagtgaatgttcccattactgATGGATACAGGACACTTCATTA
SRR1144868.65264933.1+   gaataaaggagagtttccagtgaatgttcccattactgATGGATACAGCACACTTCATTA
SRR1144868.66155008.1-   gaataaaggagagtttccagtgaatgttcccattactgATGGATACAGCACACTTCATTA
SRR1144868.66155189.1-   gaataaaggagagtttccagtgaatgttcccattactgATGGATACAGCACACTTCATTA
SRR1144869.49970874.2+   gaataaaggagagtttccagtgaatgttcccattactgATGGATACAGCACACTTCATTA
SRR1144869.52571317.2-   gaataaaggagagtttccagtgaatgttcccattactgATGGATACAGCACACTTCATTA
SRR1144869.54065919.1+   gaataaaggagagtttccagtgaatgttcccattactgATGGATACAGCACACTTCCTTA
SRR1144869.54605399.2-   taataaaggagagtttccagtgaatgttcccattactgATGGATACAGCACACTTCATTA
SRR1144870.13423647.1-   gaataaaggagagtttccagtgaatgttcccattactgATGGATACAGCACACTTCATTA
SRR1144870.17119836.2-   gaataaaggagagtttccagtgaatgttcccattactgATGGATACAGCACACTTCATTA
SRR1144870.47046512.2-   gaataaaggagagtttccagtgaatgttcccattactgATGGATACAGCACACTTCATTA
SRR1144870.73811889.2+   gaataaaggagagtttccagtgaatgttcccattactgATGGATACAGCACACTTCATTA
SRR1144871.38837713.2-   gaataaaggagagtttccagtgaatgttcccattactgATGGATACAGCACACTTCATTA
SRR1144871.46921594.2+   gaataaaggagagtttccagtgaatgttcccattactgATGGATACAGCACACTTCATTA
SRR1144871.65469880.2+   gaataaaggagagtttccagtgaatgttcccattactgATGGATACAGCACACTTCATTA
SRR1144879.26392612.1+     ataaaggagagtttccagtgaatgttcccattactgATGGATACAGCAC
SRR1144879.30863984.1+     ataaaggagagtttccagtgaatgttcccattactgATGGATACAGCAC
SRR1144871.34797005.2+     ataaaggagagtttccagtgaatgttcccattactgATGGATACAGCACACTTCATTA
SRR1144868.49756615.2-         aggagagtttccagtgaatgttcccattactgATGGATACAGCACACTTCATTA
SRR1144877.2549599.2+           ggagagtttccagtgaatgttcccattactgATGGATACAGCACACTTC
SRR1144878.29103724.1-          gagagtttccagtgaatgttcccattactgATGGATACAGCACACTTCA
SRR1144869.41401888.1-          gagagtttccagtgaatgttcccattactgATGGATACAGCACACTTCATTA
SRR1144869.41402035.1-          gagagtttccagtgaatgttcccattactgATGGATACAGCACACTTCATTA
SRR1144870.6652156.1+             gagtttccagtgaatgttcccattactgATGGATACAGCACACTTCATTA
SRR1144870.17469659.1+             agtttccagtgaatgttcccattactgATGGATACAGCACACTTCATTA
SRR1144873.2442525.1+              agtttccagtgaatgttcccattactgATGGATACAGCACACTTCATTA
SRR1144873.38918322.1+             agtttccagtgaatgttcccattactgATGGATACAGCACACTTCATTA
SRR1144879.65691056.1+             agtttccagtgaatgttcccattactgATGGATACAGCACACTTCATTA
SRR1144879.48553894.2-               tttccagtgaatgttcccattactgATGGATACAGCACACTTCATTA
SRR1144872.50503088.1-                     gtgaatgttcccattactgATGGATACAGCACACTTCATTA
SRR1144877.27788866.2-                     gtgaatgttcccattactgATGGATACAGCACACTTCATTA
SRR1144870.31792783.2-                      tgaatgttcccattactgATGGATACAGCACACTTCATTA
SRR1144871.46921594.1-                      tgaatgttcccattactgATGGATACAGCACACTTCATTA
SRR1144877.14750144.2-                      tgaatgttcccattactgATGGATACAGCACACTTCATTA
SRR1144871.31380950.1+                        aatgttcccattactgATGGATACAGCACACTTCATTA
SRR1144870.49105108.1+                           gttcccattactgATGGATACAGCACACTTCATTA
SRR1144877.9660172.2+                              tcccattactgATGGATACAGCACACTTCATTA
SRR1144869.12737898.1+                               ccattactgATGGATACAGCACACTTCATTA
SRR1144871.65469880.1-                               ccattactgATGGATACAGCACACTTCATTA
SRR1144873.49970208.2-                               ccattactgATGGATACAGCACACTTCATTA
SRR1144879.42389030.2-                               ccattactgATGGATACAGCACACTTCATTA
SRR1144879.42389148.2-                               ccattactgATGGATACAGCACACTTCATTA
SRR1144874.2179594.1-                                 cattactgATGGATACAGCACACTTCATTA
SRR1144874.2179688.1-                                 cattactgATGGATACAGCACACTTCATTA
SRR1144868.42978057.2-                                 attactgATGGATACAGCACACTTCATTA
SRR1144874.40029454.2-                                    actgATGGATACAGCACACTTCATTA
SRR1144880.40259839.1-                                    actgATGGATACAGCACACTTCATTA
SRR1144874.36764503.1+                                       gATGGATACAGCACACTTCATTA
SRR1144878.19643713.2-                                        ATGGATACAGCACACTTCATTA
SRR1144871.3356165.2+                                           GGATACAGCACACTTCATTA
SRR1144868.458086.2-                                             GATACAGCACACTTCATTA
SRR1144878.5309551.1-                                            GATACAGCACACTTCATTA
SRR1144869.61144136.2-                                            ATACAGCACACTTCATTA
```

```
SRR1144868.10327791.1+                                                CAGCACACTTCATTA
SRR1144877.61617793.1+                                                CAGCACACTTCATTA
SRR1144869.4673946.1+                                                    CACACTTCATTA
SRR1144869.16891662.2+                                                      ACTTCATTA
SRR1144872.11063924.2+                                                      ACTTCATTA
SRR1144868.84620699.2+                                                        TTCATTA
SRR1144878.42694301.1-                                                        TTCATTA
SRR1144869.80255173.2-                                                         TCATTA
SRR1144879.15009515.2+                                                          CATTA
SRR1144879.50959999.2+                                                          CATTA
SRR1144875.16266660.2-                                                           ATTA
SRR1144875.1937562.2-                                                            ATTA
SRR1144875.35369155.2-                                                           ATTA
SRR1144875.53033606.2-                                                           ATTA
SRR1144875.54444743.2-                                                           ATTA
SRR1144880.41591542.2-                                                           ATTA
SRR1144880.50830648.2-                                                           ATTA
SRR1144880.55477883.2-                                                           ATTA
SRR1144880.70110649.2-                                                           ATTA
SRR1144880.71079675.2-                                                           ATTA
SRR1144874.11322088.2-                                                             TA
SRR1144878.5996325.2+                                                               A

consensus                gaataaaggagagtttccagtgaatgttcccattactgATGGATACAGCACACTTCATTA

                             .    :    .    :    .    :    .    :    .    :    .    :
SRR1144879.48553894.2-   CT
SRR1144868.60702162.1+   CTG
SRR1144871.38837713.2-   CTGCC
SRR1144869.49970874.2+   CTGCCT
SRR1144869.52571317.2-   CTGCCT
SRR1144869.54065919.1+   CTGCCT
SRR1144872.50503088.1-   CTGCCTTT
SRR1144877.27788866.2-   CTGCCTTT
SRR1144870.13423647.1-   CTGCCTTTG
SRR1144877.14750144.2-   CTGCCTTTG
SRR1144871.65469880.2+   CTGCCTTTGGTA
SRR1144868.65264933.1+   CTGCCTTTGGTAC
SRR1144877.9660172.2+    CTGCCTTTGGTACTTA
SRR1144874.2179594.1-    CTGCCTTTGGTACTTAT
SRR1144874.2179688.1-    CTGCCTTTGGTACTTAT
SRR1144873.49970208.2-   CTGCCTTTGGTACTTATG
SRR1144879.42389030.2-   CTGCCTTTGGTACTTATG
SRR1144879.42389148.2-   CTGCCTTTGGTACTTATG
SRR1144874.40029454.2-   CTGCCTTTGGTACTTATGACACA
SRR1144880.40259839.1-   CTGCCTTTGGTACTTATGACACA
SRR1144874.36764503.1+   CTGCCTTTGGTACTTATGACACAGTC
SRR1144870.73811889.2+   CTGCCTTTGGTACTTATGACACAGTCT
SRR1144878.19643713.2-   CTGCCTTTGGTACTTATGACACAGTCT
SRR1144870.47046512.2-   CTGCCTTTGGTACTTATGACACAGTCTA
SRR1144878.5309551.1-    CTGCCTTTGGTACTTATGACACAGTCTAAT
SRR1144869.54605399.2-   CTGCCTTTGGTACTTATGACACAGTCTAATT
SRR1144877.61617793.1+   CTGCCTTTGGTACTTATGACACAGTCTAATTCTT
SRR1144870.17119836.2-   CTGCCTTTGGTACTTATGACACAGTCTAATTCTTT
SRR1144868.59303752.1-   CTGCCTTTGGTACTTATGACACAGTCTAATTCTTTA
SRR1144868.66155008.1-   CTGCCTTTGGTACTTATGACACAGTCTAATTCTTTA
SRR1144868.66155189.1-   CTGCCTTTGGTACTTATGACACAGTCTAATTCTTTA
SRR1144872.11063924.2+   CTGCCTTTGGTACTTATGACACAGTCTAATTCTTTAAAGG
SRR1144871.34797005.2+   CTGCCTTTGGTACTTATGACACAGTCTAATTCTTTAAAGGCC
SRR1144878.42694301.1-   CTGCCTTTGGTACTTATGACACAGTCTAATTCTTTAAAGGCC
SRR1144879.15009515.2+   CTGCCTTTGGTACTTATGACACAGTCTAATTCTTTAAAGGCCAA
SRR1144879.50959999.2+   CTGCCTTTGGTACTTATGACACAGTCTAATTCTTTAAAGGCCAA
SRR1144875.16266660.2-   CTGCCTTTGGTACTTATGACACAGTCTAATTCTTTAAAGGCCAAA
SRR1144875.1937562.2-    CTGCCTTTGGTACTTATGACACAGTCTAATTCTTTAAAGGCCAAA
SRR1144875.35369155.2-   CTGCCTTTGGTACTTATGACACAGTCTAATTCTTTAAAGGCCAAA
SRR1144875.53033606.2-   CTGCCTTTGGTACTTATGACACAGTCTAATTCTTTAAAGGCCAAA
SRR1144875.54444743.2-   CTGCCTTTGGTACTTATGACACAGTCTAATTCTTTAAAGGCCAAA
SRR1144880.41591542.2-   CTGCCTTTGGTACTTATGACACAGTCTAATTCTTTAAAGGCCAAA
SRR1144880.50830648.2-   CTGCCTTTGGTACTTATGACACAGTCTAATTCTTTAAAGGCCAAA
SRR1144880.55477883.2-   CTGCCTTTGGTACTTATGACACAGTCTAATTCTTTAAAGGCCAAA
SRR1144880.70110649.2-   CTGCCTTTGGTACTTATGACACAGTCTAATTCTTTAAAGGCCAAA
SRR1144880.71079675.2-   CTGCCTTTGGTACTTATGACACAGTCTAATTCTTTAAAGGCCAAA
SRR1144868.49756615.2-   CTGCCTTTGGTACTTATGACACAGTCTAATTCTTTAAAGGCCAAAG
SRR1144874.11322088.2-   CTGCCTTTGGTACTTATGACACAGTCTAATTCTTTAAAGGCCAAAGA
SRR1144869.41401888.1-   CTGCCTTTGGTACTTATGACACAGTCTAATTCTTTAAAGGCCAAAGAT
SRR1144869.41402035.1-   CTGCCTTTGGTACTTATGACACAGTCTAATTCTTTAAAGGCCAAAGAT
SRR1144878.5996325.2+    CTGCCTTTGGTACTTATGACACAGTCTAATTCTTTAAAGGCCAAAGAT
SRR1144873.8667254.1-    CTGCCTTTGGTACTTATGACACAGTCTAATTCTTTAAAGGCCAAAGATT
SRR1144879.52722640.1-   CTGCCTTTGGTACTTATGACACAGTCTAATTCTTTAAAGGCCAAAGATT
SRR1144870.6652156.1+    CTGCCTTTGGTACTTATGACACAGTCTAATTCTTTAAAGGCCAAAGATTT
SRR1144870.17469659.1+   CTGCCTTTGGTACTTATGACACAGTCTAATTCTTTAAAGGCCAAAGATTTT
SRR1144868.10327791.1+   CTGCCTTTGGTACTTATGACACAGTCTAATTCTTTAAAGGCCAAAGATTTTCTAAATGTC
SRR1144868.42978057.2-   CTGCCTTTGGTACTTATGACACAGTCTAATTCTTTAAAGGCCAAAGATTTTCTAAATGTC
SRR1144868.458086.2-     CTGCCTTTGGTACTTATGACACAGTCTAATTCTTTAAAGGCCAAAGATTTTCTAAATGTC
```

```
SRR1144868.84620699.2+   CTGCCTTTGGTACTTATGACACAGTCTAATTCTTTAAAGGCCAAAGATTTTCTAAATGTC
SRR1144869.12737898.1+   CTGCCTTTGGTACTTATGACACAGTCTAATTCTTTAAAGGCCAAAGATTTTCTAAATGTC
SRR1144869.16891662.2+   CTGCCTTTGGTACTTATGACACAGTCTAATTCTTTAAAGGCCAAAGATTTTCTAAATGTC
SRR1144869.4673946.1+    CTGCCTTTGGTACTTATGACACAGTCTAATTCTTTAAAGGCCAAAGATTTTCTAAATGTC
SRR1144869.61144136.2-   CTGCCTTTGGTACTTATGACACAGTCTAATTCTTTAAAGGCCAAAGATTTTCTAAATGTC
SRR1144869.80255173.2-   CTGCCTTTGGTACTTATGACACAGTCTAATTCTTTAAAGGCCAAAGATTTTCTAAATGTC
SRR1144870.31792783.2-   CTGCCTTTGGTACTTATGACACAGTCTAATTCTTTAAAGGCCAAAGATTTTCTAAATGTC
SRR1144870.49105108.1+   CTGCCTTTGGTACTTATGACACAGTCTAATTCTTTAAAGGCCAAAGATTTTCTAAATGTC
SRR1144871.31380950.1+   CTGCCTTTGGTACTTATGACACAGTCTAATTCTTTAAAGGCCAAAGATTTTCTAAATGTC
SRR1144871.3356165.2+    CTGCCTTTGGTACTTATGACACAGTCTAATTCTTTAAAGGCCAAAGATTTTCTAAATGTC
SRR1144871.46921594.1-   CTGCCTTTGGTACTTATGACACAGTCTAATTCTTTAAAGGCCAAAGATTTTCTAAATGTC
SRR1144871.65469880.1-   CTGCCTTTGGTACTTATGACACAGTCTAATTCTTTAAAGGCCAAAGATTTTCTAAATGTC
SRR1144870.73811889.1-    TGCCTTTGGTACTTATGACACAGTCTAATTCTTTAAAGGCCAAAGATTTTCTAAATGTC
SRR1144877.12192571.1-     GCCTTTGGTACTTATGACACAGTCTAATTCTTTAAAGGCCAAAGATTTT
SRR1144877.21784092.1-     GCCTTTGGTACTTATGACACAGTCTAATTCTTTAAAGGCCAAAGATTTT
SRR1144877.51862308.2-     GCCTTTGGTACTTATGACACAGTCTAATTCTTTAAAGGCCAAAGATTTT
SRR1144868.48380834.2+     GCCTTTGGTACTTATGACACAGTCTAATTCTTTAAAGGCCAAAGATTTTCTAAATGTC
SRR1144869.76043902.1-        TTTGGTACTTATGACACAGTCTAATTCTTTAAAGGCCAAAGATTTTCTAAATGTC
SRR1144878.22835328.1-          TGGTACTTATGACACAGTCTAATTCTTTAAAGGCCAAAGATTTTCTAAA
SRR1144871.34797005.1-          TGGTACTTATGACACAGTCTAATTCTTTAAAGGCCAAAGATTTTCTAAATGTC
SRR1144872.55945135.2-             TACTTATGACACAGTCTAATTCTTTAAAGGCCAAAGATTTTCTAAATGT
SRR1144870.17469659.2-             TACTTATGACACAGTCTAATTCTTTAAAGGCCAAAGATTTTCTAAATGTC
SRR1144871.45966609.1+              ACTTATGACACAGTCTAATTCTTTAAAGGCCAAAGATTTTCTAAATGTC
SRR1144870.5500488.1+                CTTATGACACAGTCTAATTCTTTAAAGGCCAAAGATTTTCTAAATGTC
SRR1144868.44665655.2+                TTATGACACAGTCTAATTCTTTAAAGGCCAAAGATTTTCTAAATGTC
SRR1144868.45671413.2+                  ATGACACAGTCTAATTCTTTAAAGGCCAAAGATTTTCTAAATGTC
SRR1144869.42749510.1-                  ATGACACAGTCTAATTCTTTAAAGGCCAAAGATTTTCTAAATGTC
SRR1144871.55766386.2+                    GACACAGTCTAATTCTTTAAAGGCCAAAGATTTTCTAAATGTC
SRR1144872.43173285.1-                    GACACAGTCTAATTCTTTAAAGGCCAAAGATTTTCTAAATGTC
SRR1144868.83667876.1-                     ACACAGTCTAATTCTTTAAAGGCCAAAGATTTTCTAAATGTC
SRR1144868.54387690.1-                       ACAGTCTAATTCTTTAAAGGCCAAAGATTTTCTAAATGTC
SRR1144868.85055638.2-                       ACAGTCTAATTCTTTAAAGGCCAAAGATTTTCTAAATGTC
SRR1144868.85055836.2-                       ACAGTCTAATTCTTTAAAGGCCAAAGATTTTCTAAATGTC
SRR1144868.21254686.2+                         AGTCTAATTCTTTAAAGGCCAAAGATTTTCTAAATGTC
SRR1144869.1009297.1+                          AGTCTAATTCTTTAAAGGCCAAAGATTTTCTAAATGTC
SRR1144870.61673725.1+                         AGTCTAATTCTTTAAAGGCCAAAGATTTTCTAAATGTC
SRR1144870.49105108.2-                           TCTAATTCTTTAAAGGCCAAAGATTTTCTAAATGTC
SRR1144868.66301021.2-                            CTAATTCTTTAAAGGCCAAAGATTTTCTAAATGTC
SRR1144877.19712880.1+                            CTAATTCTTTAAAGGCCAAAGATTTTCTAAATGTC
SRR1144870.30102722.1+                             TAATTCTTTAAAGGCCAAAGATTTTCTAAATGTC
SRR1144870.6652156.2-                              TAATTCTTTTAAGGCCAAAGATTTTCTAAATGTC
SRR1144869.27597638.2-                               ATTCTTTAAAGGCCAAAGATTTTCTAAATGTC
SRR1144874.24602974.1-                               ATTCTTTAAAGGCCAAAGATTTTCTAAATGTC
SRR1144868.83942966.1-                                TTCTTTAAAGGCCAAAGATTTTCTAAATGTC
SRR1144871.31380950.2-                                TTCTTTAAAGGCCAAAGATTTTCTAAATGTC
SRR1144869.15441406.1+                                 TCTTTAAAGGCCAAAGATTTTCTAAATGTC
SRR1144874.8194603.2-                                   CTTTAAAGGCCAAAGATTTTCTAAATGTC
SRR1144874.8194698.2-                                   CTTTAAAGGCCAAAGATTTTCTAAATGTC
SRR1144877.61006425.2-                                  CTTTAAAGGCCAAAGATTTTCTAAATGTC
SRR1144868.91567265.1+                                    TTAAAGGCCAAAGATTTTCTAAATGTC
SRR1144877.26008759.1+                                    TTAAAGGCCAAAGATTTTCTAAATGTC
SRR1144869.24285646.2-                                      AAAGGCCAAAGATTTTCTAAATGTC
SRR1144871.3356165.1-                                          GGCCAAAGATTTTCTAAATGTC
SRR1144873.17359866.2+                                         GGCCAAAGATTTTCTAAATGTC
SRR1144879.28044694.2+                                         GGCCAAAGATTTTCTAAATGTC
SRR1144868.62844636.2+                                          GCCAAAGATTTTCTAAATGTC
SRR1144868.62844799.2+                                          GCCAAAGATTTTCTAAATGTC
SRR1144873.11857326.2-                                           CCAAAGATTTTCTAAATGTC
SRR1144868.86292250.2+                                            CAAAGATTTTCTAAATGTC
SRR1144869.24327444.2+                                            CAAAGATTTTCTAAATGTC
SRR1144872.6794653.2+                                             CAAAGATTTTCTAAATGTC
SRR1144870.41621984.1+                                              AAGATTTTCTAAATGTC
SRR1144877.47335452.2+                                                GATTTTCTAAATGTC
SRR1144868.21392135.1+                                                 ATTTTCTAAATGTC
SRR1144875.17272141.2+                                                 ATTTTCTAAATGTC
SRR1144875.30098371.2+                                                 ATTTTCTAAATGTC
SRR1144875.37177977.2+                                                 ATTTTCTAAATGTC
SRR1144875.50250662.2+                                                 ATTTTCTAAATGTC
SRR1144880.2237028.2+                                                  ATTTTCTAAATGTC
SRR1144880.26404853.2+                                                 ATTTTCTAAATGTC
SRR1144880.29707237.2+                                                 ATTTTCTAAATGTC
SRR1144880.29800802.2+                                                 ATTTTCTAAATGTC
SRR1144880.32757909.2+                                                 ATTTTCTAAATGTC
SRR1144880.60128894.2+                                                 ATTTTCTAAATGTC
SRR1144880.60483778.2+                                                 ATTTTCTAAATGTC
SRR1144880.66017923.2+                                                 ATTTTCTAAATGTC
SRR1144880.67814530.2+                                                 ATTTTCTAAATGTC
SRR1144880.69930905.2+                                                 ATTTTCTAAATGTC
SRR1144880.71524804.2+                                                 ATTTTCTAAATGTC
SRR1144869.70757485.2+                                                  TTTTCTAAATGTC
SRR1144877.14297928.1-                                                    TTCTAAATGTC
SRR1144872.54085187.1+                                                     TCTAAATGTC
SRR1144879.33935846.1-                                                      CTAAATGTC
SRR1144879.51331996.1-                                                      CTAAATGTC
```

```
SRR1144869.63440671.2+                                                       TAAATGTC
SRR1144869.64941349.1-                                                       TAAATGTC
SRR1144869.64941350.1-                                                       TAAATGTC
SRR1144873.9652065.1+                                                         AAATGTC
SRR1144874.20147513.2+                                                        AAATGTC
SRR1144877.57417439.2+                                                        AAATGTC
SRR1144879.56575221.1+                                                        AAATGTC
SRR1144879.56575341.1+                                                        AAATGTC
SRR1144868.45792615.1+                                                         AATGTC
SRR1144869.12072787.2-                                                            GTC
SRR1144871.71492114.2+                                                            GTC
SRR1144868.45903722.2+                                                             TC
SRR1144868.41065561.1+                                                              C
SRR1144870.53925041.1+                                                              C
                         ____________________________________________________________
consensus                CTGCCTTTGGTACTTATGACACAGTCTAATTCTTTAAAGGCCAAAGATTTTCTAAATGTC

                             .    :    .    :    .    :    .    :    .    :    .    :
SRR1144871.31380950.1+   TT
SRR1144870.49105108.1+   TTTTC
SRR1144872.43173285.1-   TTTTCT
SRR1144869.12737898.1+   TTTTCTTGA
SRR1144871.65469880.1-   TTTTCTTGA
SRR1144868.42978057.2-   TTTTCTTGATG
SRR1144877.19712880.1+   TTTTCTTGATGAGG
SRR1144874.24602974.1-   TTTTCTTGATGAGGTAC
SRR1144871.3356165.2+    TTTTCTTGATGAGGTACAGA
SRR1144874.8194603.2-    TTTTCTTGATGAGGTACAGA
SRR1144874.8194698.2-    TTTTCTTGATGAGGTACAGA
SRR1144877.61006425.2-   TTTTCTTGATGAGGTACAGA
SRR1144868.458086.2-     TTTTCTTGATGAGGTACAGAG
SRR1144869.61144136.2-   TTTTCTTGATGAGGTACAGAGG
SRR1144877.26008759.1+   TTTTCTTGATGAGGTACAGAGG
SRR1144868.10327791.1+   TTTTCTTGATGAGGTACAGAGGCCC
SRR1144873.17359866.2+   TTTTCTTGATGAGGTACAGAGGCCCAT
SRR1144879.28044694.2+   TTTTCTTGATGAGGTACAGAGGCCCAT
SRR1144869.4673946.1+    TTTTCTTGATGAGGTACAGAGGCCCATC
SRR1144873.11857326.2-   TTTTCTTGATGAGGTACAGAGGCCCATCT
SRR1144872.6794653.2+    TTTTCTTGATGAGGTACAGAGGCCCATCTG
SRR1144869.16891662.2+   TTTTCTTGATGAGGTACAGAGGCCCATCTGA
SRR1144868.84620699.2+   TTTTCTTGATGAGGTACAGAGGCCCATCTGACC
SRR1144869.80255173.2-   TTTTCTTGATGAGGTACAGAGGCCCATCTGACCC
SRR1144877.47335452.2+   TTTTCTTGATGAGGTACAGAGGCCCATCTGACCC
SRR1144875.17272141.2+   TTTTCTTGATGAGGTACAGAGGCCCATCTGACCCA
SRR1144875.30098371.2+   TTTTCTTGATGAGGTACAGAGGCCCATCTGACCCA
SRR1144875.37177977.2+   TTTTCTTGATGAGGTACAGAGGCCCATCTGACCCA
SRR1144875.50250662.2+   TTTTCTTGATGAGGTACAGAGGCCCATCTGACCCA
SRR1144880.2237028.2+    TTTTCTTGATGAGGTACAGAGGCCCATCTGACCCA
SRR1144880.26404853.2+   TTTTCTTGATGAGGTACAGAGGCCCATCTGACCCA
SRR1144880.29707237.2+   TTTTCTTGATGAGGTACAGAGGCCCATCTGACCCA
SRR1144880.29800802.2+   TTTTCTTGATGAGGTACAGAGGCCCATCTGACCCA
SRR1144880.32757909.2+   TTTTCTTGATGAGGTACAGAGGCCCATCTGACCCA
SRR1144880.60128894.2+   TTTTCTTGATGAGGTACAGAGGCCCATCTGACCCA
SRR1144880.60483778.2+   TTTTCTTGATGAGGTACAGAGGCCCATCTGACCCA
SRR1144880.66017923.2+   TTTTCTTGATGAGGTACAGAGGCCCATCTGACCCA
SRR1144880.67814530.2+   TTTTCTTGATGAGGTACAGAGGCCCATCTGACCCA
SRR1144880.69930905.2+   TTTTCTTGATGAGGTACAGAGGCCCATCTGACCCA
SRR1144880.71524804.2+   TTTTCTTGATGAGGTACAGAGGCCCATCTGACCCA
SRR1144877.14297928.1-   TTTTCTTGATGAGGTACAGAGGCCCATCTGACCCAACT
SRR1144872.54085187.1+   TTTTCTTGATGAGGTACAGAGGCCCATCTGACCCAACTG
SRR1144879.33935846.1-   TTTTCTTGATGAGGTACAGAGGCCCATCTGACCCAACTGA
SRR1144879.51331996.1-   TTTTCTTGATGAGGTACAGAGGCCCATCTGACCCAACTGA
SRR1144870.73811889.1-   TTTTCTTGATGAGGTACAGAGGCCCATCTGACCCAACTGAC
SRR1144868.48380834.2+   TTTTCTTGATGAGGTACAGAGGCCCATCTGACCCAACTGACC
SRR1144873.9652065.1+    TTTTCTTGATGAGGTACAGAGGCCCATCTGACCCAACTGACC
SRR1144874.20147513.2+   TTTTCTTGATGAGGTACAGAGGCCCATCTGACCCAACTGACC
SRR1144877.57417439.2+   TTTTCTTGATGAGGTACAGAGGCCCATCTGACCCAACTGACC
SRR1144879.56575221.1+   TTTTCTTGATGAGGTACAGAGGCCCATCTGACCCAACTGACC
SRR1144879.56575341.1+   TTTTCTTGATGAGGTACAGAGGCCCATCTGACCCAACTGACC
SRR1144869.76043902.1-   TTTTCTTGATGAGGTACAGAGGCCCATCTGACCCAACTGACCTCA
SRR1144871.34797005.1-   TTTTCTTGATGAGGTACAGAGGCCCATCTGACCCAACTGACCTCAAA
SRR1144870.17469659.2-   TTTTCTTGATGAGGTACAGAGGCCCATCTGACCCAACTGACCTCAAAATA
SRR1144871.45966609.1+   TTTTCTTGATGAGGTACAGAGGCCCATCTGACCCAACTGACCCCAAAATAA
SRR1144870.5500488.1+    TTTTCTTGATGAGGTACAGAGGCCCATCTGACCCAACTGACCTCAAAATAAT
SRR1144868.44665655.2+   TTTTCTTGATGAGGTACAGAGGCCCATCTGACCCAACTGACCTCAAAATAATC
SRR1144868.45671413.2+   TTTTCTTGATGAGGTACAGAGGCCCATCTGACCCAACTGACCCCAACATAATCTT
SRR1144869.42749510.1-   TTTTCTTGATGAGGTACAGAGGCCCATCTGACCCAACTGACCCCAAAATAATCTT
SRR1144871.55766386.2+   TTTTCTTGATGAGGTACAGAGGCCCATCTGACCCAACTGACCCCAAAATAATCTTCA
SRR1144868.83667876.1-   TTTTCTTGATGAGGTACAGAGGCCCATCTGACCCAACTGACCTCAAAATAATCTTCAC
SRR1144868.21254686.2+   TTTTCTTGATGAGGTACAGAGGCCCATCTGACCCAACTGACCCCAAAATAATCTTCACCT
SRR1144868.21392135.1+   TTTTCTTGATGAGGTACAGAGGCCCATCTGACCCAACTGACCTCAAAATAATCTTCACCT
SRR1144868.41065561.1+   TTTTCTTGATGAGGTACAGAGGCCCATCTGACCCAACTGACCCCAAAATAATCTTCACCT
SRR1144868.45792615.1+   TTTTCTTGATGAGGTACAGAGGCCCATCTGACCCAACTGACCCCAAAATAATCTTCACCT
SRR1144868.45903722.2+   TTTTCTTGATGAGGTACAGAGGCCCATCTGACCCAACTGACCTCAAAATAATCTTCACCT
```

```
SRR1144868.54387690.1-   TTTTCTTGATGAGGTACAGAGGCCCATCTGACCCAACTGACCCCAAAATAATCTTCACCT
SRR1144868.62844636.2+   TTTTCTTGATGAGGTACAGAGGCCCATCTGACCCAACTGACCCCAAAATAATCTTCACCT
SRR1144868.62844799.2+   TTTTCTTGATGAGGTACAGAGGCCCATCTGACACAACTGACCCCAAAATAATCTTCACCT
SRR1144868.66301021.2-   TTTTCTTGATGAGGTACAGAGGCCCATCTGACCCAACTGACCCCAAAATAATCTTCACCT
SRR1144868.83942966.1-   TTTTCTTGATGAGGTACAGAGGCCCATCTGACCCAACTGACCTCAAAATAATCTTCACCT
SRR1144868.85055638.2-   TTTTCTTGATGAGGTACAGAGGCCCATCTGACCCAACTGACCTCAAAATAATCTTCACCT
SRR1144868.85055836.2-   TTTTCTTGATGAGGTACAGAGGCCCATCTGACCCAACTGACCTCAAAATAATCTTCACCT
SRR1144868.86292250.2+   TTTTCTTGATGAGGTACAGAGGCCCATCTGACCCAACTGACCCCAAAATAATCTTCACCT
SRR1144868.91567265.1+   TTTTCTTGATGAGGTACAGAGGCCCATCTGACCCAACTGACCCCAAAATAATCTTCACCT
SRR1144869.1009297.1+    TTTTCTTGATGAGGTACAGAGGCCCATCTGACCCAACTGACCTCAAAATAATCTTCACCT
SRR1144869.12072787.2-   TTTTCTTGATGAGGTACAGAGGCCCATCTGACCCAACTGACCTCAAAATAATCTTCACCT
SRR1144869.15441406.1+   TTTTCTTGATGAGGTACAGAGGCCCATCTGACCCAACTGACCTCAAAATAATCTTCACCT
SRR1144869.24285646.2-   TTTTCTTGATGAGGTACAGAGGCCCATCTGACCCAACTGACCCCAAAATAATCTTCACCT
SRR1144869.24327444.2+   TTTTCTTGATGAGGTACAGAGGCCCATCTGACCCAACTGACCTCAAAATAATCTTCACCT
SRR1144869.27597638.2-   TTTTCTTGATGAGGTACAGAGGCCCATCTGACCCAACTGACCTCAAAATAATCTTCACCT
SRR1144869.63440671.2+   TTTTCTTGATGAGGTACAGAGGCCCATCTGACCCAACTGACCTCAAAATAATCTTCACCT
SRR1144869.64941349.1-   TTTTCTTGATGAGGTACAGAGGCCCATCTGACCCAACTGACCCCAAAATAATCTTCACCT
SRR1144869.64941350.1-   TTTTCTTGATGAGGTACAGAGGCCCATCTGACCCAACTGACCCCAAAATAATCTTCACCT
SRR1144869.70757485.2+   TTTTCTTGATGAGGTACAGAGGCCCATCTGACCCAACTGACCTCAAAATAATCTTCACCT
SRR1144870.30102722.1+   TTTTCTTGATGAGGTACAGAGGCCCATCTGACCCAACTGACCTCAAAATAATCTTCACCT
SRR1144870.41621984.1+   TTTTCTTGATGAGGTACAGAGGCCCATCTGACCCAACTGACCCCAAAATAATCTTCACCT
SRR1144870.49105108.2-   TTTTCTTGATGAGGTACAGAGGCCCATCTGACCCAACTGACCTCAAAATAATCTTCACCT
SRR1144870.53925041.1+   TTTTCTTGATGAGGTACAGAGGCCCATCTGACCCAACTGACCCCAAAATAATCTTCACCT
SRR1144870.61673725.1+   TTTTCTTGATGAGGTACAGAGGCCCATCTGACCCAACTGACCTCAAAATAATCTTCACCT
SRR1144870.6652156.2-    TTTTCTTGATGAGGTACAGAGGCCCATCTGACCCAACTGACCCCAAAATAATCTTCACCT
SRR1144871.31380950.2-   TTTTCTTGATGAGGTACAGAGGCCCATCTGACCCAACTGACCCCAAAATAATCTTCACCT
SRR1144871.3356165.1-    TTTTCTTGATGAGGTACAGAGGCCCATCTGACCCAACTGACCTCAAAATAATCTTCACCT
SRR1144871.71492114.2+   TTTTCTTGATGAGGTACAGAGGCCCATCTGACCCAACTGACCCCAAAATAATCTTCACCT
SRR1144874.8753005.1+     TTTCTTGATGAGGTACAGAGGCCCATCTGACCCAACTGACCCCAAAATA
SRR1144869.43766655.1+    TTTCTTGATGAGGTACAGAGGCCCATCTGACCCAACTGACCCCAAAATAATCTTCACCT
SRR1144869.63650894.1+      TCTTGATGAGGTACAGAGGCCCATCTGACCCAACTGACCCCAAAATAATCTTCACCT
SRR1144869.8345878.2+       TCTTGATGAGGTACAGAGGCCCATCTGACCCAACTGACCCCAAAATAATCTTCACCT
SRR1144878.32179812.1-       CTTGATGAGGTACAGAGGCCCATCTGACCCAACTGACCCCAAAATAATC
SRR1144874.29736650.1-         TGATGAGGTACAGAGGCCCATCTGACCCAACTGACCCCAAAATAATCTT
SRR1144877.17194435.1-         TGATGAGGTACAGAGGCCCATCTGACCCAACTGACCCCAAAATAATCTT
SRR1144879.9178158.1+          TGATGAGGTACAGAGGCCCATCTGACCCAACTGACCCCAAAATAATCTT
SRR1144869.63616507.1-         TGATGAGGTACAGAGGCCCATCTGACCCAACTGACCCCAAAATAATCTTCACCT
SRR1144879.44479633.1-           ATGAGGTACAGAGGCCCATCTGACCCAACTGACCCCAAAATAATCTTCA
SRR1144879.62232076.1-           ATGAGGTACAGAGGCCCATCTGACCCAACTGACCCCAAAATAATCTTCA
SRR1144874.35887586.1+             GAGGTACAGAGGCCCATCTGACCCAACTGACCCCAAAATAATCTTCACC
SRR1144877.10728706.1+             GAGGTACAGAGGCCCATCTGACCCAACTGACCCCAAAATAATCTTCACC
SRR1144871.45966609.2-              AGGTACAGAGGCCCATCTGACCCAACTGACCCCAAAATAATCTTCACCT
SRR1144868.38943194.1+               GGTACAGAGGCCCATCTGACCCAACTGACCCCAAAATAATCTTCACCT
SRR1144868.50956912.1+               GGTACAGAGGCCCATCTGACCCAACTGACCCCAAAATAATCTTCACCT
SRR1144869.76334149.1-                GTACAGAGGCCCATCTGACCCAACTGACCCCAAAATAATCTTCACCT
SRR1144869.76334150.1-                GTACAGAGGCCCATCTGACCCAACTGACCCCAAAATAATCTTCACCT
SRR1144875.22303585.2-                GTACAGAGGCCCATCTGACCCAACTGACCCCAAAATAATCTTCACCT
SRR1144875.9845596.2-                 GTACAGAGGCCCATCTGACCCAACTGACCCCAAAATAATCTTCACCT
SRR1144880.18641104.1+                GTACAGAGGCCCATCTGACCCAACTGACCCCAAAATAATCTTCACCT
SRR1144880.33661683.2-                GTACAGAGGCCCATCTGACCCAACTGACCCCAAAATAATCTTCACCT
SRR1144880.34810606.1+                GTACAGAGGCCCATCTGACCCAACTGACCCCAAAATAATCTTCACCT
SRR1144880.38243217.1+                GTACAGAGGCCCATCTGACCCAACTGACCCCAAAATAATCTTCACCT
SRR1144880.70816288.2-                GTACAGAGGCCCATCTGACCCAACTGACCCCAAAATAATCTTCACCT
SRR1144870.5500488.2-                   ACAGAGGCCCATCTGACCCAACTGACCTCAAAATAATCTTCACCT
SRR1144878.34264526.2-                  ACAGAGGCCCATCTGACCCAACTGACCTCAAAATAATCTTCACCT
SRR1144869.50380653.1+                    AGAGGCCCATCTGACCCAACTGACCCCAAAATAATCTTCACCT
SRR1144868.90126106.1-                       GGCCCATCTGACCCAACTGACCTCAAAATAATCTTCACCT
SRR1144871.55766386.1-                       GGCCCATCTGACCCAACTGACCCCAAAATAATCTTCACCT
SRR1144877.33497325.1-                         CCCATCTGACCCAACTGACCTCAAAATAATCTTCACCT
SRR1144869.3270077.1+                           CCATCTGACCCAACTGACCCCAAAATAATCTTCACCT
SRR1144869.3270217.1+                           CCATCTGACCCAACTGACCCCAAAATAATCTTCACCT
SRR1144869.47784350.1-                          CCATCTGACCCAACTGACCTCAAAATAATCTTCACCT
SRR1144873.13545456.1-                           CATCTGACCCAACTGACCCCAAAATAATCTTCACCT
SRR1144873.37100088.1+                           CATCTGACCCAACTGACCTCAAAATAATCTTCACCT
SRR1144879.54514274.1-                           CATCTGACCCAACTGACCCCAAAATAATCTTCACCT
SRR1144879.55281172.1-                           CATCTGACCCAACTGACCCCAAAATAATCTTCACCT
SRR1144879.55281281.1-                           CATCTGACCCAACTGACCCCAAAATAATCTTCACCT
SRR1144868.25258814.1-                            ATCTGACCCAACTGACCTCAAAATAATCTTCACCT
SRR1144868.92408075.1-                            ATCTGACCCAACTGACCCCAAAATAATCTTCACCT
SRR1144868.92408258.1-                            ATCTGACCCAACTGACCCCAAAATAATCTTCACCT
SRR1144878.1843122.1-                               CTGACCCAACTGACCCCAAAATAATCTTCACCT
SRR1144878.48127341.1-                              CTGACCCAACTGACCCCAAAATAATCTTCACCT
SRR1144879.14521809.2+                               TGACCCAACTGACCCCAAAATAATCTTCACCT
SRR1144879.59056380.2+                               TGACCCAACTGACCCCAAAATAATCTTCACCT
SRR1144874.20419677.1-                                GACCCAACTGACCCCAAAATAATCTTCACCT
SRR1144870.61673725.2-                                    CAACTGACCTCAAAATAATCTTCACCT
SRR1144873.7791113.1-                                     CAACTGACCTCAAAATAATCTTCACCT
SRR1144877.66914433.1+                                      ACTGACCCCAAAATAATCTTCACCT
SRR1144870.30102722.2-                                        TGACCTCAAAATAATCTTCACCT
SRR1144873.24496541.1-                                        TGACCCCAAAATAATCTTCACCT
SRR1144873.38386793.2-                                        TGACCCCAAAATAATCTTCACCT
SRR1144873.9300436.1-                                         TGACCCCAAAATAATCTTCACCT
SRR1144877.44248084.2+                                        TGACCTCAAAATAATCTTCACCT
SRR1144879.46848275.2-                                        TGACCCCAAAATAATCTTCACCT
```

```
SRR1144879.48882587.1-                                        TGACCCCAAAATAATCTTCACCT
SRR1144879.8450688.2-                                         TGACCCCAAAATAATCTTCACCT
SRR1144873.10372297.2+                                          ACCCCAAAATAATCTTCACCT
SRR1144868.40331692.2-                                           CCTCAAAATAATCTTCACCT
SRR1144869.7266474.2-                                             CCCAAAATAATCTTCACCT
SRR1144872.54542593.2-                                            CCCAAAATAATCTTCACCT
SRR1144878.57144785.2+                                            CTCAAAATAATCTTCACCT
SRR1144870.41621984.2-                                               AAAATAATCTTCACCT
SRR1144879.25392406.1+                                                 AATAATCTTCACCT
SRR1144879.26982715.2-                                                 AATAATCTTCACCT
SRR1144868.93193558.2+                                                   TAATCTTCACCT
SRR1144870.63521823.1+                                                    AATCTTCACCT
SRR1144870.59632527.1+                                                     ATCTTCACCT
SRR1144877.30797025.1+                                                     ATCTTCACCT
SRR1144877.34278009.1+                                                     ATCTTCACCT
SRR1144872.1082717.1+                                                       TCTTCACCT
SRR1144869.4662147.2-                                                        CTTCACCT
SRR1144869.39444143.1-                                                         TCACCT
SRR1144877.66940950.1-                                                          CACCT
SRR1144870.2992437.2+                                                            ACCT
SRR1144870.53925041.2-                                                           ACCT
SRR1144875.16313714.1-                                                           ACCT
SRR1144875.1655993.1-                                                            ACCT
SRR1144875.32651981.2-                                                           ACCT
SRR1144875.45276718.2-                                                           ACCT
SRR1144875.62005888.1-                                                           ACCT
SRR1144880.32733766.1-                                                           ACCT
SRR1144880.3898918.1-                                                            ACCT
SRR1144880.47304521.1-                                                           ACCT
SRR1144880.47486423.1-                                                           ACCT
SRR1144880.60062253.2-                                                           ACCT
SRR1144871.71492114.1-                                                            CCT
SRR1144875.21621342.2-                                                            CCT
SRR1144872.13547249.2-                                                             CT

consensus                TTTTCTTGATGAGGTACAGAGGCCCATCTGACCCAACTGACCCCAAAATAATCTTCACCT

                             .    :    .    :    .    :    .    :    .    :    .    :
SRR1144868.21254686.2+   AT
SRR1144869.1009297.1+    AT
SRR1144870.61673725.1+   AT
SRR1144875.22303585.2-   AT
SRR1144875.9845596.2-    AT
SRR1144880.18641104.1+   AT
SRR1144880.33661683.2-   AT
SRR1144880.34810606.1+   AT
SRR1144880.38243217.1+   AT
SRR1144880.70816288.2-   AT
SRR1144870.49105108.2-   ATGA
SRR1144878.34264526.2-   ATGA
SRR1144868.66301021.2-   ATGAC
SRR1144870.6652156.2-    ATGAC
SRR1144870.30102722.1+   ATGACC
SRR1144869.27597638.2-   ATGACCTG
SRR1144868.83942966.1-   ATGACCTGA
SRR1144871.31380950.2-   ATGACCTGA
SRR1144869.15441406.1+   ATGACCTGAG
SRR1144877.33497325.1-   ATGACCTGAGG
SRR1144868.91567265.1+   ATGACCTGAGGCT
SRR1144873.13545456.1-   ATGACCTGAGGCT
SRR1144873.37100088.1+   ATGACCTGAGGCT
SRR1144878.1843122.1-    ATGACCTGAGGCT
SRR1144878.48127341.1-   ATGACCTGAGGCT
SRR1144879.54514274.1-   ATGACCTGAGGCT
SRR1144879.55281172.1-   ATGACCTGAGGCT
SRR1144879.55281281.1-   ATGACCTGAGGCT
SRR1144869.24285646.2-   ATGACCTGAGGCTGG
SRR1144879.14521809.2+   ATGACCTGAGGCTGGAC
SRR1144879.59056380.2+   ATGACCTGAGGCTGGAC
SRR1144871.3356165.1-    ATGACCTGAGGCTGGACA
SRR1144874.20419677.1-   ATGACCTGAGGCTGGACA
SRR1144868.62844636.2+   ATGACCTGAGGCTGGACAA
SRR1144868.62844799.2+   ATGACCTGAGGCTGAACAA
SRR1144868.86292250.2+   ATGACCTGAGGCTGGACAACa
SRR1144869.24327444.2+   ATGACCTGAGGCTGGACAACa
SRR1144873.7791113.1-    ATGACCTGAGGCTGGACAACat
SRR1144870.41621984.1+   ATGACCTGAGGCTGGACAACata
SRR1144877.66914433.1+   ATGACCTGAGGCTGGACAACataa
SRR1144868.21392135.1+   ATGACCTGAGGCTGGACAACataagt
SRR1144873.24496541.1-   ATGACCTGAGGCTGGACAACataagt
SRR1144873.38386793.2-   ATGACCTGAGGCTGGACAACataagt
SRR1144873.9300436.1-    ATGACCTGAGGCTGGACAACataagt
SRR1144877.44248084.2+   ATGACCTGAGGCTGGACAACataagt
SRR1144879.46848275.2-   ATGACCTGAGGCTGGACAACataagt
```

```
SRR1144879.48882587.1-   ATGACCTGAGGCTGGACAACataagt
SRR1144879.8450688.2-    ATGACCTGAGGCTGGACAACataagt
SRR1144869.70757485.2+   ATGACCTGAGGCTGGACAACataagta
SRR1144872.54542593.2-   ATGACCTGAGGCTGGACAACataagta
SRR1144873.10372297.2+   ATGACCTGAGGCTGGACAACataagtat
SRR1144878.57144785.2+   ATGACCTGAGGCTGGACAACataagtattg
SRR1144869.63440671.2+   ATGACCTGAGGCTGGACAACataagtattgg
SRR1144869.64941349.1-   ATGACCTGAGGCTGGACAACataagtattgga
SRR1144869.64941350.1-   ATGACCTGAGGCTGGACAACataagtattgga
SRR1144868.45792615.1+   ATGACCTGAGGCTGGACAACataagtattggaca
SRR1144879.25392406.1+   ATGACCTGAGGCTGGACAACataagtattggacaa
SRR1144879.26982715.2-   ATGACCTGAGGCTGGACAACataagtattggacaa
SRR1144872.1082717.1+    ATGACCTGAGGCTGGACAACataagtattggacaag
SRR1144869.12072787.2-   ATGACCTGAGGCTGGACAACataagtattggacaaga
SRR1144871.71492114.2+   ATGACCTGAGGCTGGACAACataagtattggacaaga
SRR1144868.45903722.2+   ATGACCTGAGGCTGGACAACataagtattggacaagag
SRR1144868.41065561.1+   ATGACCTGAGGCTGGACAACctaagtattggacaagagc
SRR1144870.53925041.1+   ATGACCTGAGGCTGGACAACataagtattggacaagagc
SRR1144877.30797025.1+   ATGACCTGAGGCTGGACAACataagtattggacaagagc
SRR1144877.34278009.1+   ATGACCTGAGGCTGGACAACataagtattggacaagagc
SRR1144869.43766655.1+   ATGACCTGAGGCTGGACAACataagtattggacaagagcat
SRR1144869.63650894.1+   ATGACCTGAGGCTGGACAACataagtattggacaagagcatta
SRR1144869.8345878.2+    ATGACCTGAGGCTGGACAACataagtattggacaagagcatta
SRR1144877.66940950.1-   ATGACCTGAGGCTGGACAACataagtattggacaagagcattac
SRR1144875.16313714.1-   ATGACCTGAGGCTGGACAACataagtattggacaagagcattact
SRR1144875.1655993.1-    ATGACCTGAGGCTGGACAACataagtattggacaagagcattact
SRR1144875.32651981.2-   ATGACCTGAGGCTGGACAACataagtattggacaagagcattact
SRR1144875.45276718.2-   ATGACCTGAGGCTGGACAACataagtattggacaagagcattact
SRR1144875.62005888.1-   ATGACCTGAGGCTGGACAACataagtattggacaagagcattact
SRR1144880.32733766.1-   ATGACCTGAGGCTGGACAACataagtattggacaagagcattact
SRR1144880.3898918.1-    ATGACCTGAGGCTGGACAACataagtattggacaagagcattact
SRR1144880.47304521.1-   ATGACCTGAGGCTGGACAACataagtattggacaagagcattact
SRR1144880.47486423.1-   ATGACCTGAGGCTGGACAACataagtattggacaagagcattact
SRR1144880.60062253.2-   ATGACCTGAGGCTGGACAACataagtattggacaagagcattact
SRR1144869.63616507.1-   ATGACCTGAGGCTGGACAACataagtattggacaagagcattactt
SRR1144875.21621342.2-   ATGACCTGAGGCTGGACAACataagtattggacaagagcattactt
SRR1144872.13547249.2-   ATGACCTGAGGCTGGACAACataagtattggacaagagcattacttg
SRR1144871.45966609.2-   ATGACCTGAGGCTGGACAACataagtattggacaagagcattacttggcat
SRR1144868.38943194.1+   ATGACCTGAGGCTGGACAACattaggattggacaagagcattacttggcatg
SRR1144868.50956912.1+   ATGACCTGAGGCTGGACAACataagtattggacaagagcattacctggcatg
SRR1144869.76334149.1-   ATGACCTGAGGCTGGACAACataagtattggacaagagcattacttggcatgt
SRR1144869.76334150.1-   ATGACCTGAGGCTGGACAACataagtattggacaagagcattacttggcatgt
SRR1144870.5500488.2-    ATGACCTGAGGCTGGACAACataagtattggacaagagcattacttggcatgtta
SRR1144869.50380653.1+   ATGACCTGAGGCTGGACAACataagtattggacaagagcattacttggcatgttaaa
SRR1144868.25258814.1-   ATGACCTGAGGCTGGACAACataagtattggacaagagcattacttggcatgttaaaaaa
SRR1144868.40331692.2-   ATGACCTGAGGCTGGACAACataagtattggacaagagcattacttggcatgttaaaaaa
SRR1144868.60393072.2-   ATGACCTGAGGCTGGACAACataagtattggacaagagcattacttggcatgttaaaaaa
SRR1144868.90126106.1-   ATGACCTGAGGCTGGACAACataagtattggacaagagcattacttggcatgttaaaaaa
SRR1144868.92408075.1-   ATGACCTGAGGCTGGACAACataagtattggacaagagcattacttggcatgttaaaaaa
SRR1144868.92408258.1-   ATGACCTGAGGCTGGACAACataagtattggacaagagcattacttggcatgttaaaaaa
SRR1144868.93193558.2+   ATGACCTGAGGCTGGACAACataagtattggacaagagcattacttggcatgttaaaaaa
SRR1144869.3270077.1+    ATGACCTGAGGCTGGACAACataagtattggacaagagcattacttggcatgttaaaaaa
SRR1144869.3270217.1+    ATGACCTGAGGCTGGACAACataagtattggacaagagcattacttggcatgttaaaaaa
SRR1144869.39444143.1-   ATGACCTGAGGCTGGACAACataagtattggacaagagcattacttggcatgttaaaaaa
SRR1144869.4662147.2-    ATGTGCTGAGGCTGGACAACataagtattggacaagggcattgcttggcatgttaaaaaa
SRR1144869.47784350.1-   ATGACCTGAGGCTGGACAACataagtattggacaagagcattacttggcatgttaaaaaa
SRR1144869.7266474.2-    ATGACCTGAGGCTGGACAACataagtattggacaagagcattacttggcatgttaaaaaa
SRR1144870.2992437.2+    ATGACCTGAGGCTGGACAACataagtattggacaagagcattacttggcatgttaaaaaa
SRR1144870.30102722.2-   ATGACCTGAGGCTGGACAACataagtattggacaagagcattacttggcatgttaaaaaa
SRR1144870.41621984.2-   ATGACCTGAGGCTGGACAACataagtattggacaagagcattacttggcatgttaaaaaa
SRR1144870.53925041.2-   ATGACCTGAGGCTGGACAACataagtattggacaagagcattacttggcatgttaaaaaa
SRR1144870.59632527.1+   ATGACCTGAGGCTGGACAACataagtattggacaagagcattacttggcatgttaaaaaa
SRR1144870.61673725.2-   ATGACCTGAGGCTGGACAACataagtattggacaagagcattacttggcatgttaaaaaa
SRR1144870.63521823.1+   ATGACCTGAGGCTGGACAACataagtattggacaagagcattacttggcatgttaaaaaa
SRR1144871.55766386.1-   ATGACCTGAGGCTGGACAACataagtattggacaagagcattacttggcatgttaaaaaa
SRR1144871.71492114.1-   ATGACCTGAGGCTGGACAACataagtattggacaagagcattacttggcatgttaaaaaa
SRR1144868.41459039.1+    TGACCTGAGGCTGGACAACataagtattggacaagagcattacttggcatgttaaaaaa
SRR1144868.29201477.2-     GACCTGAGGCTGGACAACataagtattggacaagagcattacttggcatgttaaaaaa
SRR1144869.37171537.2-      ACCTGAGGCTGGACAACataagtattggacaagagcattacttggcatgttaaaaaa
SRR1144877.32428901.2+        CTGAGGCTGGACAACataagtattggacaagagcattacttggcatgtt
SRR1144878.29416815.2-        CTGAGGCTGGACAACataagtattggacaagagcattacttggcatgtt
SRR1144868.78420249.1+        CTGAGGCTGGACAACataagtattggacaagagcattacttggcatgttaaaaaa
SRR1144870.8228234.2+         CTGAGGCTGGACAACataagtattggacaagagcattacttggcatgttaaaaaa
SRR1144877.39321162.1-           AGGCTGGACAACataagtattggacaagagcattacttggcatgttaaa
SRR1144878.21214886.1-           AGGCTGGACAACataagtattggacaagagcattacttggcatgttaaa
SRR1144868.27877859.2+           AGGCTGGACAACataagtattggacaagagcattacttggcatgttacaaaa
SRR1144872.25108680.2+            GGCTGGACAACataagtattggacaagagcattacttggcatgttaaaa
SRR1144872.45310390.2+            GGCTGGACAACataagtattggacaagagcattacttggcatgttaaaa
SRR1144871.50082040.2+            GGCTGGACAACataagtattggacaagagcattacttggcatgttaaaaaa
SRR1144873.48276514.1+              CTGGACAACataagtattggacaagagcattacttggcatgttaaaaaa
SRR1144879.46173129.1+              CTGGACAACataagtattggacaagagcattacttggcatgttaaaaaa
SRR1144868.27151520.2-                 GACAACataagtattggacaagagcattacttggcatgttaaaaaa
SRR1144870.3613080.1+                    CAACataagtattggacaagagcattacttggcatgttaaaaaa
SRR1144873.42090231.1-                    AACataagtattggacaagagcattacttggcatgttaaaaaa
```

```
SRR1144877.51631482.1-                   AACataagtattggacaagagcattacttggcatgttaaaaaa
SRR1144877.57857415.1+                   AACataagtattggacaagagcattacttggcatgttaaaaaa
SRR1144868.81099429.1+                    ACataagtattggacaagagcattacttggcatgttaaaaaa
SRR1144873.11440383.2-                    ACataagtattggacaagagcattacttggcatgttaaaaaa
SRR1144873.16640611.2-                    ACataagtattggacaagagcattacttggcatgttaaaaaa
SRR1144873.27516368.2-                    ACataagtattggacaagagcattacttggcatgttaaaaaa
SRR1144873.51739636.1-                    ACataagtattggacaagagcattacttggcatgttaaaaaa
SRR1144879.6458933.2-                     ACataagtattggacaagagcattacttggcatgttaaaaaa
SRR1144869.12895680.2-                     Cataagtattggacaagagcattacttggcatgttaaaaaa
SRR1144869.69410263.1-                     Cataagtattggacaagagcattacttggcatgttaaaaaa
                         ____________________________________________________________
consensus                ATGACCTGAGGCTGGACAACataagtattggacaagagcattacttggcatgttaaaaaa
```

K. Kea (No. 11), exon 4, WGS data

- CAP3 alignment of WGS data spanning exon 4 (uppercase letters)

```
                              .    :    .    :    .    :    .    :    .    :    .    :
SRR959225.55927882.1-     gttattgagttgccaggggaaccctcaggtgacccatgtgtgtttctttttcaaTTTG
SRR959225.49491860.1+     gttattgagttgccaggggaaccctcaggagacacatgtgtgtttctttttcaaTTTGCC
SRR959225.59145430.2-     gttattgagttgccaggggaaccctcaggtgacccatgtgtgtttctttttcaaTTTGCC
SRR959225.59640912.1-     gttattgagttgccaggggaaccctcaggtgacccatgtgtgtttctttttcaaTTTGCC
SRR959225.63515961.1-     gttattgagttgccaggggaaccctcaggtgacccatgtgtgtttctttttcaaTTTGCC
SRR959225.7722830.1+      gttattgagttgccaggggaaccctcaggtgacccatgtgtgtttctttttcaaTTTGCC
SRR959225.90843042.1+     gttattgagttgccaggggaaccctcaggtgacccatgtgtgtttctttttcaaTTTGCC
SRR959225.94255078.1+     gttattgagttgcccggggaaccctcaggtgccccatgtgtgtttctttttcaaTTTGCC
SRR959225.9808133.1+      gttattgagttgccaggggaaccctcaggtgacccatgtgtgtttctttttcaaTTTGCC
SRR959226.14030507.2+     gttattgagttgccaggggaaccctcaggtgacccatgtgtgtttctttttcaaTTTGCC
SRR959226.4499420.2-      gttattgagttgccaggggaaccctcaggtgacccatgtgtgtttctttttcaaTTTGCC
SRR959226.51154971.1+     gttattgagttgccaggggaaccctcaggtgacccatgtgtgtttctttttcaaTTTGCC
SRR959226.55198843.1+     gttattgagttgccaggggaaccctcaggtgacccatgtgtgtttctttttcaaTTTGCC
SRR959227.18222216.1+     gttattgagttgccaggggaaccctcaggtgacccatgtgtgtttctttttcaaTTTGCC
SRR959227.22875044.2+     gttattgagttgccaggggaaccctcaggtgacccatgtgtgtttctttttcaaTTTGCC
SRR959227.22957956.1+     gttattgagttgccaggggaaccctcaggtgacccatgtgtgtttctttttcaaTTTGCC
SRR959225.4318033.1-       ttattgagttgccaggggaaccctcaggtgacccatgtgtgtttctttttcaaTTTGCC
SRR959227.75998761.2+            agttgccaggggaaccctcaggcgacccatgtgtgtttctttttcaaTTTGCC
SRR959227.36915196.2+                gccaggggaaccctcaggtgacccatgtgtgtttctttttcaaTTTGCC
SRR959225.90843042.2-                        aaccctcaggtgacccatgtgtgtttctttttcaaTTTGCC
SRR959225.27455840.2+                           cctcaggtgacccatgtgtgtttctttttcaaTTTGCC
SRR959225.33495248.2-                               aggtgacccatgtgtgtttctttttcaaTTTGCC
SRR959225.54283651.1-                               aggtgacccatgtgtgtttctttttcaaTTTGCC
SRR959225.5996382.1-                                  gtgacccatgtgtgtttctttttcaaTTTGCC
SRR959225.3355209.2+                                    gacccatgtgtgtttctttttcaaTTTGCC
SRR959225.23411114.2-                                    acccatgtgtgtttctttttcaaTTTGCC
SRR959225.81686073.1+                                    acccatgtgtgtttctttttcaaTTTGCC
SRR959226.45349599.1-                                    acccatgtgtgtttctttttcaaTTTGCC
SRR959227.55032331.2-                                     cccatgtgtgtgtctttttcaaTTTGCC
SRR959227.53810762.2+                                         tgtgtgtttctttttcaaTTTGCC
SRR959225.84786349.1+                                          gtgtgtttctttttcaaTTTGCC
SRR959226.52538067.2+                                              gtttctttttcaaTTTGCC
SRR959225.20136878.1+                                                  ctttttcaaTTTGCC
SRR959227.50673472.1+                                                   tttttcaaTTTGCC
SRR959227.4979772.2-                                                        tcaaTTTGCG
SRR959226.35159252.1+                                                          aTTTGCC
                          ____________________________________________________________
consensus                 gttattgagttgccaggggaaccctcaggtgacccatgtgtgtttctttttcaaTTTGCC

                              .    :    .    :    .    :    .    :    .    :    .    :
SRR959227.18222216.1+     ATTCCA
SRR959225.94255078.1+     ATTCCAGT
SRR959225.59145430.2-     ATTCCAGTTG
SRR959225.59640912.1-     ATTCCAGTTGAAGA
SRR959226.4499420.2-      ATTCCAGTTGAAGAAAT
SRR959225.63515961.1-     ATTCCAGTTGAAGAAATCACA
SRR959227.22957956.1+     ATTCCAGTTGAAGAAATCACAT
SRR959225.7722830.1+      ATTCCAGTTGAAGAAATCACATACATATCCC
SRR959226.51154971.1+     ATTCCAGTTGAAGAAATCACATACATATCCC
SRR959225.49491860.1+     CTTCCAGTTGAAGAAATCACATACATATCCCTGTA
SRR959227.22875044.2+     ATTCCAGTTGAAGAAATCACATACATATACCTGTA
SRR959226.14030507.2+     ATTCCAGTTGAAGAAATCACATACATATCCCTGTACC
SRR959225.90843042.1+     ATTCCAGTTGAAGAAATCACATACATATCCCTGTACCT
SRR959225.9808133.1+      ATTCCAGTTGAAGAAATCACATACATATCCCTGTACCTT
SRR959226.55198843.1+     ATTCCAGTTGAAGAAATCACATACATATCCCTGTACCTT
SRR959225.4318033.1-      ATTCCAGTTGAAGAAATCACATACATATCCCTGTACCTTTA
SRR959227.75998761.2+     ATTCCAGTTGAAGAAATCACATACATATCCCTGTACCTTTATCCCAC
SRR959227.36915196.2+     ATTCCAGTTGAAGAAATCACATACATATCACTGTACCTTTATCCTACTGCC
SRR959225.90843042.2-     ATTCCAGTTGAAGAAATCACATACATATCCCTGTACCTTTATCCCACTGCCCACTGTCA
SRR959225.20136878.1+     ATTCCAGTTGAAGAAATCACATACATATCCCTGTACCTTTATCCCACTGCCCACTGTCAA
SRR959225.23411114.2-     ATTCCAGTTGAAGAAATCACATACATATCCCTGTACCTTTATCCCACTGCCCACTGTCAA
SRR959225.27455840.2+     ATTCCAGTTGAAGAAATCACATACATATCCCTGTACCTTTATCCCACTGCCCACTGTCAA
SRR959225.33495248.2-     ATTCCAGTTGAAGAAATCACATACATATCCCTGTACCTTTATCCCACTGCCCACTGTCAA
SRR959225.3355209.2+      ATTCCAGTTGAAGAAATCACATACATATCCCTGTACCTTTATCCCACTGCCCACTGTCAA
SRR959225.54283651.1-     ATTCCAGTTGAAGAAATCACATACATATCCCTGTACCTTTATCCCACTGCCCACTGTCAA
SRR959225.5996382.1-      ATTCCAGTTGAAGAAATCACATACATATCCCTGTACCTTTATCCCACTGCCCACTGTCAA
SRR959225.81686073.1+     ATTCCAGTTGAAGAAATCACATACATATCCCTGTACCTTTATCCCACTGCCCACTGTCAA
SRR959225.84786349.1+     ATTCCAGTTGAAGAAATCACATACATATCCCTGTACCTTTATCCCACTGCCCACTGTCAA
SRR959226.35159252.1+     ATTCCAGTTGAAGAAATCACATACATATCCCTGTACCTTTATCCCACTGCCCACTGTCAA
SRR959226.45349599.1-     ATTCCAGTTGAAGAAATCACATACATATCCCTGTACCTTTATCCCACTGCCCACTGTCAA
SRR959226.52538067.2+     ATTCCAGTTGAAGAAATCACATACATATCCCTGTACCTTTATCCCACTGCCCACTGTCAA
SRR959226.69078214.1-     ATTCCAGTGGAAGAAATCACATACATATCCCTGTACCTTTATCCCACTGCCCACTGTCAA
SRR959227.4979772.2-      ATTCCAGTTGAAGAAATCACATACATATCCCTGTTCCTTTATCCCACTGCCCACTGTCAA
SRR959227.50673472.1+     ATTCCAGTTGAAGAAATCACATACATATCCCTGTACCTTTATCCCACTGCCCACTGTCAG
SRR959227.53810762.2+     ATTCCAGTTGAAGAAATCACATACATATCCCTGTACCTTTATCCCACTGCCCACTGTCAA
```

```
SRR959227.55032331.2-    ATTCCAGTTGAAGAAATCACATACATATCCCTGTACCTTTATCCCACTGCCCACTGTCAA
SRR959226.44625528.1+            TGAAGAAATCACATACATATCCCTGTACCTTTATCCCACTGCCCACTGTCAA
SRR959225.51251850.2-             GAAGAAAGCACATACATATCCCTGCACCTTTATCCCACTGCCCACTGTCAA
SRR959225.15444684.1-                  AATCACATACATATCCCTGTACCTTTATCCCACGGCCCACTGTCAA
SRR959227.50163015.2+                      ACATACATATCCCTGTACCTTTATCCCACTGCCCACTGTCAA
SRR959227.7714433.2+                                TCCCTGTACCTTTATCCCACTGCCCACTGTCAA
SRR959227.16030144.1+                                       CCTTTATCCCACTGCCCACTGTCAA
SRR959227.19019145.1+                                             TCCCACTGCCCACTGTCAA
SRR959227.75881652.1+                                               CCACTGCCCACTGTCAA
SRR959225.74795193.1+                                                CACTGCCCACTGTCAA
SRR959226.57565219.2-                                                    GCCCACTGTCAA
SRR959227.32203429.1+                                                          TGTCAA
SRR959225.74682073.1+                                                             CAA
SRR959226.4794536.2-                                                               AA
SRR959226.63286309.2+                                                              AA
SRR959226.4463198.1-                                                                A
                         ____________________________________________________________
consensus                ATTCCAGTTGAAGAAATCACATACATATCCCTGTACCTTTATCCCACTGCCCACTGTCAA

                             .    :    .    :    .    :    .    :    .    :    .    :
SRR959225.27455840.2+    GC
SRR959225.33495248.2-    GCAGAA
SRR959225.54283651.1-    GCAGAA
SRR959225.5996382.1-     GCAGAAAC
SRR959225.3355209.2+     GCAGAAACAC
SRR959225.23411114.2-    GCAGAAACACC
SRR959225.81686073.1+    GCAGAAACACC
SRR959226.45349599.1-    GCAGAAACACC
SRR959227.55032331.2-    GCAGAAACACCA
SRR959227.53810762.2+    GCAGAAACACCATTAT
SRR959225.84786349.1+    GCAGAAACACCATTATC
SRR959226.52538067.2+    GCAGAAACACCATTATCTACA
SRR959225.20136878.1+    GCAGAAACACCATTATCTACAAGgt
SRR959227.50673472.1+    GCAGAAACACCATTATCTACAAGgta
SRR959227.4979772.2-     GCAGAAACACCATTATCTACAAGgtaaatt
SRR959226.35159252.1+    GCAGAAACACCATTATCTACAAGgtaaatttgc
SRR959226.69078214.1-    GCAGAAACACCATTATCTACAAGgtaaatttgcaggtttg
SRR959226.44625528.1+    GCAGAAACACCATTATCTACAAGgtaaatctgcaggtttgtcacaact
SRR959225.51251850.2-    GCAGAAACACCATTATCTACAAGgtaaatttgcaggtttgtcacaactg
SRR959225.15444684.1-    GCAGAAACACCATTATCTACAAGgtaaatttgcaggtttgtcacaactggggag
SRR959227.50163015.2+    GCAGAAACACCATTATCTACAAGgtaaatttgcaggtttgtcccaactggggagaaac
SRR959225.74682073.1+    GCAGAAACACCATTATCTACAAGgtaaatttgcaggtttgtcacaactggggagaaacag
SRR959225.74795193.1+    GCAGAAACACCATTATCTACAAGgtaaatttgcaggtttgtcacaactggggagaaacag
SRR959226.4463198.1-     GCAGAAACACCATTATCTACAAGgtaaatttgcaggtttgtcacaactggggagaaacag
SRR959226.4794536.2-     GCAGAAACACCATTATCTACAAGgtaaatttgcaggtttgtcacaactggggagaaacag
SRR959226.57565219.2-    GCAGAAACACCATTATCTACAAGgtaaatttgcaggtttgtcacaactggggagaaacag
SRR959226.63286309.2+    GCAGAAACACCATTATCTACAAGgtaaatttgcaggtttgtcacaactggggagaaacag
SRR959227.16030144.1+    GCAGAAACACCATTATCTACAAGgtaaatttgcaggtttgtcacaactggggagaaacag
SRR959227.19019145.1+    GCAGAAACACCATTATCTACAAGgtaaatttgcaggtttgtcacaactggggagaaacag
SRR959227.32203429.1+    GCAGAAACACCATTATCTACAAGgtaaatttgcaggtttgtcacaactggggagaaacag
SRR959227.75881652.1+    GCAGAAACACCATTATCTACAAGgtaaatttgcaggtttgtcacaactggggagaaacag
SRR959227.7714433.2+     GCAGAAACACCATTATCTACAAGgtaaatttgcaggtttgtcacaactggggagaaacag
SRR959225.35932207.1+           CACCATTATCTACAAGgtaaatttgcaggtttgtcacaactggggagaaacag
SRR959225.59869922.2+              CATTATCTACAAGgtaaatttgcaggtttgtcacaactggggagaaacag
SRR959225.7861402.1-                       ACAAGgcaaatgtgcaggtttgtcacaactggggagaaacag
SRR959227.32338303.2+                      ACAAGgtaaatttgcaggtttgtcacaactggggagaaacag
SRR959227.42566408.1+                       CAAGgtaaatttgcaggtttgtcacaactggggagaaacag
SRR959227.19171617.2-                        AAGgtaaatttgcaggtttgtcacaactggggagaaacag
SRR959227.3045510.2-                         AAGgtaaatttgcaggtttgtcacaactggggagaaacag
SRR959225.96117414.2-                         AGgtaaatttgcaggtttgtcacaactggggagaaacag
SRR959226.77806910.2+                         AGgtaaatttgcaggtttgtcacaactggggagaaacag
                         ____________________________________________________________
consensus                GCAGAAACACCATTATCTACAAGgtaaatttgcaggtttgtcacaactggggagaaacag
```

L. Budgerigar (No. 12), exon 4, WGS data

- CAP3 alignment of WGS data spanning exon 4 (uppercase letters)

```
                             .    :    .    :    .    :    .    :    .    :    .    :
ERR244157.76016802.1+    tgtttcttttccatT
ERR244146.109211469.2-   t--ttcttttccatTTT
ERR244156.98634684.2+    t--ttcttttccatTTTG
ERR244146.120120261.1+   g--ttgttttcctTTTGC
ERR244157.96563184.2-    t--ttcttttccatTTTGCTG
ERR244158.36848991.1-    t--ttcttttccatTTTGCTGT
ERR244157.55245384.1-    t--ttcttttccatTTTGCTGTTCT
ERR244156.150852715.1-   t--ttcttttccatTTTGCTGTTCTG
ERR244156.35419544.1-    tgtttcttttccatTTTGCTGTTCCAG
ERR244145.89602273.2+    t--ttcttttccatTTTGCTGTTCTGGC
ERR244147.20148833.2-    t--ttcttttccatTTTGCTGTTCTGGC
ERR244156.24514958.1-    tgtttcttttccatTTTGCTGTTCCAGT
ERR244145.96068630.2+    t--ttcttttccatTTTGCTGTTCTGGCTG
ERR244156.67541015.1-    tgtttcttttccatTTTGCTGTTCCAGTTG
ERR244146.87876677.2-    t--ttcttttccatTTTGCTGTTCTGGCTGA
ERR244151.32370255.2+    t--ttcttttccatTTTGCTGTTCTGGCTGAA
ERR244156.75594749.2+    tgtttcttttccatTTTGCTGTTCCAGTTGAA
ERR244145.142298147.1-   t--ttcttttccatTTTGCTGTTCTGGCTGAAGA
ERR244153.66220651.2-    t--ttcttttccatTTTGCTGTTCTGGCTGAAGA
ERR244145.109411984.1-   t--ttcttttccatTTTGCTGTTCTGGCTGAAGAA
ERR244145.136532548.2-   t--ttcttttccatTTTGCTGTTCTGGCTGAAGAA
ERR244156.114525817.1+   tgtttcttttccatTTTGCTGTTCCAGTTGAAGAA
ERR244145.139130025.1-   t--ttcttttccatTTTGCTGTTCTGGCTGAAGAAA
ERR244156.66837716.1-    t--ttcttttccatTTTGCTGTTCTGGCTGAAGAAACC
ERR244157.63718191.1+    t--ttcttttccatTTTGCTGTTCTGGCTGAAGAAACCA
ERR244158.806159.2-      tgtttcttttccatTTTGCTGTTCCAGTTGAAGAAACCA
ERR244156.141617992.1+   tgtttcttttccatTTTGCTGTTCCAGTTGAAGAAACCAT
ERR244157.76321302.2-    tgtttcttttccatTTTGCTGTTCCAGTTGAAGAAACCAT
ERR244156.133816308.2+   tgtttcttttccatTTTGCTGTTCCAGTTGAAGAAACCATA
ERR244157.98479751.2-    t--ttcttttccatTTTGCTGTTCTGGCTGAAGAAACCATA
ERR244145.39604221.1+    t--ttcttttccatTTTGCTGTTCTGGCTGAAGAAACCATAT
ERR244148.14907354.2+    t--ttcttttccatTTTGCTGTTCTGGCTGAAGAAACCATAT
ERR244148.67652096.2+    t--ttcttttccatTTTGCTGTTCTGGCTGAAGAAACCATAT
ERR244156.31231237.1-    tgtttcttttccatTTTGCTGTTCCAGTTGAAGAAACCATATA
ERR244157.74064951.2-    t--ttcttttccatTTTGCTGTTCTGGCTGAAGAAACCATATA
ERR244145.32145537.2-    tgtttcttttccatTTTGCTGTTCCAGTTGAAGAAACCATATAT
ERR244147.93446370.2+    tgattcttttccctTTTGCTGTTACAGTTTATGAAACCAGATATG
ERR244151.48196842.1+    t--ttcttttccatTTTGCTGTTCTGGCTGAAGAAACCATATATG
ERR244156.102713578.2+   t--ttcttttccatTTTGCTGTTCTGGCTGAAGAAACCATATATG
ERR244146.39934981.1+    t--ttcttttccatTTTGCTGTTCTGGCTGAAGAAAACATATATGC
ERR244157.24600673.1+    t--ttcttttccatTTTGCTGTTCTGGCTGAAGAAACCATATATGC
ERR244158.14554932.1+    t--ttcttttccatTTTGCTGTTCTGGCTGAAGAAACCATATATGC
ERR244158.39975997.1+    t--ttcttttccatTTTGCTGTTCTGGCTGAAGAAACCATATATGC
ERR244146.153780298.2+   tgtttcttttccatTTTGCTGTTCCAGTTGTAGAAACCATATATGCC
ERR244145.138381124.1-   tgtttcttttccatTTTGCTGTTCCAGTTGAAGAAACCATATATGCCT
ERR244158.40372127.1-    t--ttcttttccatTTTGCTGTTCTGGCTGAAGAAACCATATATGCCTG
ERR244145.19972568.1+    tgtttcttttccatTTTGCTGTTCCAGTTGAAGCAAACATATATTCCTGT
ERR244156.50761240.1+    t--ttcttttccatTTTGCTGTTCTGGCTGAAGAAACCATATATGCCTGT
ERR244156.68083478.2+    tgtttcttttccatTTTGCTGTTCCAGTTGAAGAAACCATATATGCCTGT
ERR244153.3083667.2+     t--ttcttttccatTTTGCTGTTCTGGCTGAAGAAACCATATATGCCTGTA
ERR244153.74352287.1+    t--ttcttttccatTTTGCTGTTCTGGCTGAAGAAACCATATATGCCTGTA
ERR244157.103004017.1+   tgtttcttttccatTTTGCTGTTCCAGTTGAAGAAACCATATATGCCTGTAT
ERR244145.151981935.2-   t--ttcttttccatTTTGCTGTTCTGGCTGAAGAAACCATATATGCCTGTATT
ERR272382.180088.2-      t--ttcttttccatTTTGCTGTTCTGGCTGAAGAAACCATATATGCCTGTATT
ERR244146.7970188.2-     tgtttcttttccatTTTGCTGTTCCAGTTGAAGAAACCATATATGCCTGTATCT
ERR244157.73894864.1-    tgtttcttttccatTTTGCTGTTCCAGTTGAAGAAACCATATATGCCTGTATCT
ERR244146.38383027.1-    tgtttcttttccatTTTGCTGTTCCAGTTGAAGAAACCATATATGCCTGTATCTT
ERR244156.14411817.2-    tgtttcttttccatTTTGCTGTTCCAGTTGAAGAAACCATATATGCCTGTATCTT
ERR244145.150845067.1-   tgtttcttttccatTTTGCTGTTCCAGTTGAAGAAACCATATATGCCTGTATCTTT
ERR244146.131310014.1+   t--ttcttttccatTTTGCTGTTCTGGCTGAAGAAACCATATATGCGTGTATTTTT
ERR244146.146406433.1+   tgtttcttttccatTTTGCTGTTCCAGTTGAAGAAACCCTATATGACTGTATCTTT
ERR244147.33464638.1-    t--ttcttttccatTTTGCTGTTCTGGCTGAAGAAACCATATATGCCTGTATTTTT
ERR244149.5303750.2-     t--ttcttttccatTTTGCTGTTCTGGCTGAAGAAACCATATATGCCTGTATTTTT
ERR244146.133798042.1-   t--ttcttttccatTTTGCTGTTCTGGCTGAAGAAACCATATATGCCTGTATTTTTAT
ERR244147.88524223.1-    t--ttcttttccatTTTGCTGTTCTGGCTGAAGAAACCATATATGCCTGTATTTTTAT
ERR244156.98930996.1-    tgtttcttttccatTTTGCTGTTCCAGTTGAAGAAACCATATATGCCTGTATCTTTAT
ERR244156.129121093.1-   t--ttcttttccatTTTGCTGTTCTGGCTGAAGAAACCATATATGCCTGTATTTTTATC
ERR244156.54583533.2+    t--ttcttttccatTTTGCTGTTCTGGCTGAAGAAACCATATATGCCTGTATTTTTATC
ERR244157.97834118.1-    tgtttcttttccatTTTGCTGTTCCAGTTGAAGAAACCATATATGCCTGTATCTTTATC
ERR244158.3507344.2+     tgtttcttttccatTTTGCTGTTCCAGTTGAAGAAACCATATATGCCTGTATCTTTATC
ERR244145.102457280.1+   tgtttcttttccatTTTGCTGTTCCAGTTGAAGAAACCATATATGCCTGTATCTTTATCC
ERR244145.106184922.1+   t--ttcttttccatTTTGCTGTTCTGGCTGAAGAAACCATATATGCCTGTATTTTTATCC
ERR244145.110237827.2-   tgtttcttttccatTTTGCTGTTCCAGTTGAAGAAACCATATATGCCTGTATCTTTATTC
ERR244145.127347530.1-   tgtttcttttccatTTTGCTGTTCCAGTTGAAGAAACCATATATGCCTGTATCTTTATCC
ERR244145.127347530.2+   tgtttcttttccatTTTGCTGTTCCAGTTGAAGAAACCATATATGCCTGTATCTTTATCC
```

ERR244145.17810775.1-   t--ttcttttccatTTTGCTGTTCTGGCTGAAGAAACCATATATGCCTGTAATTTTATCC
ERR244145.18845138.2+   tgtttcttttccatTTTGCTGTTCCAGTTGAAGAAACCCTATATGCCTTTATCTTTATCC
ERR244145.19972568.2-   tgtttcttttccatTTTGCTGTTCCAGTTGAAGAAACCATATATGCCTGTATCTTTATCC
ERR244145.22441587.2+   tgtttcttttccatTTTGCTGTTCCAGTTGAAGAAACCATATATGCCTGTATCTTTATCC
ERR244145.30608194.2+   t--ttcttttccatTTTGCTGTTCTGGCTGAAGAAACCATATATGCCTGTATTTTTATCC
ERR244145.37775792.1-   t--ttcttttccatTTTGCTGTTCTGGCTGAAGAAACCATATATGCCTGTATTTTTATCC
ERR244145.39604221.2-   t--ttcttttccatTTTGCTGTTCTGGCTGAAGAAACCATATATGCCTGTATTTTTATCC
ERR244145.46160951.1+   t--ttcttttccatTTTGCTGTTCTGGCTGAAGAAACCATATATGCCTGTATTTTTATCA
ERR244145.46160951.2-   t--ttcttttccatTTTGCTGTTCTGGCTGAAGAAACCATATATGCCTGTATTTTTATCC
ERR244145.46643170.2+   t--ttcttttccatTTTGCTGTTCTGGCTGAAGAAACCATATATGCCTGTATTTTTATCC
ERR244145.48548398.2+   tgtttcttttccatTTTGCTGTTCTGGCTGAAGAAACCATATATGCCTGTATTTTTATCC
ERR244145.57384943.1-   tgtttcttttccatGTTGCTGTTCCAGTTGAATAAACCATATATGCCTGTATCTTTATCC
ERR244145.57384943.2+   tgtttattttccatTTTGCTGTTCCAGTTTAAGAAAACATATATGCCTGTATCTTTATCC
ERR244145.63687745.1+   t--ttcttttccatTTTGCTGTTCTGGCTGAAGAAACCATATATGCCTGTATTTTTATCC
ERR244145.80053276.2+   t--ttcttttccatTTTGATGTTCTGGCTGAAGAAACCATATATGCCTGTATTTTTATCC
ERR244145.89602273.1-   t--ttcttttccatTTTGCTGTTCTGGCTGAAGAAACCATATATGCCTGTATTTTTATCC
ERR244145.96068630.1-   t--ttcttttccatTTTGCTGTTCTGGCTGAAGAAACCATATATGCCTGTATTTTTATCC
ERR244145.98207251.2+   tgtttcttttccatTTTGCTGTTCTGGCTGAAGAAACCATATATGCCTGTATTTTTATCC
ERR244146.103365155.1-   tgtttcttttccatTTTGCTGTTCCAGTTGAAGAAACCATATATGCCTGTATCTTTATCC
ERR244146.107304693.2+   t--ttcttttccatTTTGCTGTTCTGGCTGAAGAAACCATATATGCCTGCATTTTTATCC
ERR244146.109494033.2+   t--ttcttttccatTTTGCTGTTCTGGCTGAAGAAACCATATATGCCTGTATTTTTATCC
ERR244146.116947821.2-   tgtttcttttccatTTTGCTGTTCTGGCTGAAGAAACCATATATGCCTGTATTTTTATCC
ERR244146.118142077.2+   t--ttcttttccatTTTGCTGTTCTGGCTGAAGAAACCATATATGCCTGTATTTTTATCC
ERR244146.118918090.2-   tgtttcttttccatTTTGCTGTTCCAGTTGAAGAAACCATATATGCCTGTATCTTTATCC
ERR244146.124845523.2-   tgtttcttttccatTTTGCTGTTCCAGTTGAAGAAACCATATATGCCTGTATCTTTATCC
ERR244146.140222926.2+   tgtttcttttccatTTTGCTGTTCCAGTTGAAGAAACCATATATGCCTGTATCTTTATCC
ERR244146.14354249.2-   tgtttcttttccatTTTGCTGTTCCAGTTGAAGAAACCATATATGCCTGTATCTTTATCC
ERR244146.143563595.1-   t--ttcttttccatTTTGCTGTTCTGGCTGAAGAAACCATATATGCCTGTATTTTTATCC
ERR244146.146536627.2-   tgtttcttttccatTTTGCTGTTCCAGTTGAAGAAACCATATATGCCTGTATCTTTATCC
ERR244146.150494649.2-   t--ttcttttccatTTTGCTGTTCTGGCTGAAGAAACCATATATGCCTGTATTTTTATCC
ERR244146.29560179.1-   t--ttcttttccatTTTGCTGTTCTGGCTGAAGAAACCATATATGCCTGTATTTTTATCC
ERR244146.47506220.2-   t--ttcttttccatTTTGCTGTTCTGGCTGAAGAAACCATATATGCCTGTATTTTTATCC
ERR244146.49370712.2-   tgtttcttttccatTTTGCTGTTCCAGTTGAAGAAACCATATATGCCTGTATCTTTATCC
ERR244146.52725920.1-   t--ttcttttccatTTTGCTGTTCTGGCTGAAGAAACCATATATGCCTGTATTTTTATCC
ERR244146.64167387.2+   tgtttcttttccatTTTGCTGTTCCAGTTGAAGAAACCATATATGCCTGTATCTTTATCC
ERR244146.68666943.2+   tgtttcttttccatTTTGCTGTTCCAGTTGAAGAAACCATATATGCCTGTATCTTTATCC
ERR244146.75918108.1-   t--ttctttgccatTTTGCTGTTCTGGCTGAAGATACCATATATGCCTGTATTTTTATCC
ERR244146.77596753.1+   t--ttcttttccatTTTGCTGTTCTGGCTGAAGAAACCATATATGCCTGTATTTTTATCC
ERR244146.87059851.2-   tgtttcttttccatTTTGCTGTTCCAGTTGAAGAAACCATATATGCCTGTATCTTTATCC
ERR244146.90665826.1-   t--ttcttttccatTTTGCTGTTCTGGCTGAAGAAACCATATATGCCTGTATTTTTATCC
ERR244146.91490918.1+   tgtttcttttccatTTTGCTGTTCCAGTTGAAGAAACCATATATGCCTGTATCTTTATCC
ERR244146.9290774.1+   tgtttcttttccatTTTGCTGTTCCAGTTGAAGAAACCATATATGCCTGTATCTTTATCC
ERR244146.97675664.1+   t--ttcttttccatTTTGCTGTTCTGGCTGAAGAAACCATATATGCCTGTATTTTTATCC
ERR244147.10275342.2-   t--ttcttttccatTTTGCTGTTCTGGCTGAAGAAACCATATATGCCTGTATTTTTATCC
ERR244147.121272221.1-   tgtttcttttccatTTTGCTGTTCCAGTTGAAGAAACCATATATGCCTGTATCTTTATCC
ERR244147.12202203.1+   t--ttcttttccatTTTGCTGGTCTGGCTGAAGAAACCCTATATGCCTGTAGTTTTATCC
ERR244147.132045579.1-   t--ttcttttccatTTTGCTGTTCTGGCTGAAGAAACCATATATGCCTGTATTTTTATCC
ERR244147.136713187.1+   tgtttcttttccatTTTGCTGTTCTGGCTGAAGAAACCATATATGCCTGTATTTTTATCC
ERR244147.15961227.2+   tgtttcttttccatTTTGCTGTTCTGGCTGAAGAAACCATATATGCCTGTATTTTTATCC
ERR244147.17572057.2-   tgtttcttttccatTTTGCTGTTCCAGTTGAAGAAACCATATATGCCTGTATCTTTATCC
ERR244147.22728488.1-   t--ttcttttccatTTTGCTGTTCTGGCTGAAGAAACCATATATGCCTGTATTTTTATCC
ERR244147.25647165.2-   t--ttcttttccatTTTGCTGTTCTGGCTGAAGAAACCATATATGCCTGTATTTTTATCC
ERR244147.4157084.1+   tgtttcttttccatTTTGCTGTTCCAGTTGAAGAAACCATATATGCCTGTATCTTTATCC
ERR244147.69941180.1-   t--ttcttttccatTTTGCTGTTCTGGCTGAAGAAACCATATATGCCTGTATTTTTATCC
ERR244147.77801619.2-   tgtttcttttccatTTTGCTGTTCTGGCTGAAGAAACCATATATGCCTGTATTTTTATCC
ERR244147.82193546.1-   t--ttcttttccatTTTGCTGTTCTGGCTGAAGAAACCATATATGCCTGTATTTTTATCC
ERR244148.41993600.2-   tgtttcttttccatTTTGCTGTTCTGGCTGAAGAAACCATATATGCCTGTATTTTTATCC
ERR244148.51037509.1+   t--ttcttttccatTTTGCTGTTCTGGCTGAAGAAACCATATATGCCTGTATTTTTATCC
ERR244148.6823651.1-   tgtttcttttccatTTTGCTGTTCTGGCTGAAGAAACCATATATGCCTGTATTTTTATCC
ERR244149.25775136.1+   t--ttcttttccatTTTGCTGTTCTGGCTGAAGAAACCATATATGCCTGTATTTTTATCC
ERR244150.15347188.2+   tgtttcttttccatTTTGCTGTTCCAGTTGAAGAAACCATATATGCCTGTATCTTTATCC
ERR244150.442652.1+   t--ttcttttccatTTTGCTGTTCTGGCTGAAGAAACCATATATGTCTGTATTTTTATCC
ERR244151.17502359.2-   t--ttcttttccatTTTGCTGTTCTGGCTGAAGAAACCATATATGCCTGTATTTTTATCC
ERR244151.777093.1+   t--ttcttttccatTTTGCTGTTCTGGCTGAAGAAACCATATATGCCTGTATTTTTATCC
ERR244152.2384579.1+   tgtttcttttccatTTTGCTGTTCTGGCTGAAGAAACCATATATGCCTGTATTTTTATCC
ERR244152.73646274.1+   tgtttcttttccatTTTGCTGTTCTGGCTGAAGAAACCATATATGCCTGTATTTTTATCC
ERR244153.55115941.2+   tgtttcttttccatTTTGCTGTTCTGGCTGAAGAAACCATATATGCCTGTATTTTTATCC
ERR244156.120584320.1+   tgtttcttttccatTTTGCTGTTCTGGCTGAAGAAACCATATATGCCTGTATTTTTATCC
ERR244156.123492829.2+   t--ttcttttccatTTTGCTGTTCTGGCTGAAGAAACCATATATGCCTGTATTTTTATCC
ERR244156.130034420.1+   tgtttcttttccatTTTGCTGTTCCAGTTGAAGAAACCATATATGCCTGTATCTTTATCC
ERR244156.134587516.1-   tgtttcttttccatTTTGCTGTTCCAGTTGAAGAAACCATATATGCCTGTATCTTTATCC
ERR244156.154736233.1+   tgtttcttttccatTTTGCTGTTCTGGCTGAAGAAACCATATATGCCTGTATTTTTATCC
ERR244156.16052576.1-   tgtttcttttccatTTTGCTGTTCCAGTTGAAGAAACCATATATGCCTGTATCTTTATCC
ERR244156.162215319.2+   tgtttcttttccatTTTGCTGTTCTGGCTGAAGAAACCATATATGCCTGTATTTTTATCC
ERR244156.17686513.1-   tgtttcttttccatTTTGCTGTTCCAGTTGAAGAAACCATATATGCCTGTATCTTTATCC
ERR244156.27908911.1-   tgtttcttttccatTTGGCTGTTACAGTTGAAGAATCCATATATGCCTGTATCTTTACCC
ERR244156.38707225.2+   tgtttcttttccatTTTGCTGTTCCAGTTGAAGAAACCATATATGCCTGTATCTTTATCC
ERR244156.39426149.2+   t--ttcttttccatTTTGCTGTTCTGGCTGAAGAAACCATATATGCCTGTATTTTTATCC
ERR244156.40198159.1-   t--ttcttttccatTTTGCTGTTCTGGCTGAAGAAACCATATATGCCTGTATTTTTATCC
ERR244156.47741673.1+   tgtttcttttccatTTTGCTGTTCTGGCTGAAGAAACCATATATGCCTGTATTTTTATCC
ERR244156.61862812.2+   tgtttcttttccatTTTGCTGTTCCAGTTGAAGAAACCATATATGCCTGTATCTTTATCC
ERR244156.88532666.2-   tgtttcttttccatTTTGCTGTTCCAGTTGAAGAAACCATATATGCCTGTATCTTTATCC
ERR244157.12232170.1-   t--ttcttttccatTTTGCTGTTCTGGCTGAAGAAACCATATATGCCTGTATTTTTATCC
ERR244157.16960720.2-   tgtttcttttccatTTTGCTGTTCTGGCTGAAGAAACCATATATGCCTGTATTTTTATCC

```
ERR244157.2304262.1-      t--ttctttcccatTTTGCTGTTCTGCCTGAAGAACCCATATATGCCTGTATTTTTATCC
ERR244157.24672276.1+     tgtttcttttccatTTTGCTGTTCCAGTTGAAGAAACCATATATGCCTGTATCTTTATCC
ERR244157.28744060.1+     t--ttcttttccatTTTGCTGTTCTGGCTGACGAAACCATATATGCCTGTATTTTTATCC
ERR244157.40217339.2+     tgtttcttttccatTTTGCTGTTCCAGTTGAAGAAACCATATATGCCTGTATCTTTATCC
ERR244157.68534216.2+     t--ttcttttccatTTTGCTGTTCTGGCTGAAGAAACCATATAAGCCTGTATTTTTATCC
ERR244157.71374123.1+     tgtttcttttccatTTTGCTGTTCCAGTTGAAGAAACCATATATGCCTGTATCTTTATCC
ERR244157.83398179.2-     t--ttcttttccatTTTGCTGTTCTGGCTGAAGAAACCATATATGCCTGTATTTTTATCC
ERR244157.83604973.1+     tgtttcttttccatTTTGCTGTTCCAGTTGAAGAAACCATATATGCCTGTATCTTTATCC
ERR244157.88933338.1+     tgtttcttttccatTTTGCTGTTCTGGCTGAAGAAACCATATATGCCTGTATTTTTATCC
ERR244158.12039058.1+     tgtttcttttccatTTTGCTGTTCCAGTTGAAGAAACCATATATGCCTGTATCTTTATCC
ERR244158.12904674.1-     tgtttcttttccatTTTGCTGTTCCAGTTGAAGAAACCATATATGCCTGTATCTTTATCC
ERR244158.14530390.1-     tgtttcttttccatTTTGCTGTTCCAGTTGAAGAAACCATATATGCCTGTATCTTTATCC
ERR244158.23421992.2+     tgtttcttttccatTTTGCTGTTCCAGTTGAAGAAACCATATATGCCTGTATCTTTATCC
ERR244158.26979419.2-     tgtttcttttccatTTTGCTGTTCCAGTTGAAGAAACCATATATGCCTGTATCTTTATCC
ERR244158.28053158.1+     tgtttcttttccatTTTGCTGTTCCAGTTGAAGGAACCATATATGCCTGTATCTTTATCC
ERR244158.31623411.1+     t--ttcttttccatTTTGCTGTTCTGGCTTAAGAAACCATATATGCCTGTATTTTTATCC
ERR244158.40825569.2-     tgtttcttttccatTTTGCTGTTCCAGTTGAAGAAACCATATATGCCTGTATCTTTATCC
ERR272293.162385.2-       t--ttcttttccatTTTGCTGTTCTGGCTGAAGAAACCATATATGCCTGTATTTTTATCC
ERR272293.163306.2-       t--ttcttttccatTTTGCTGTTCTGGCTGAAGAAACCATATATGCCTGTATTTTTATCC
ERR272314.115165.2+       t--ttcttttccatTTTGCTGTTCTGGCTGAAGAAACCATATATGCCTGTATTTTTATCC
ERR272317.118222.2-       t--ttcttttccatTTTGCTGTTCTGGCTGAAGAAACCATATATGCCTGTATTTTTATCC
ERR272334.112347.2-       tgtttcttttccatTTTGCTGTTCCAGTTGAAGAAACCATATATGCCTGTATCTTTATCC
ERR272354.158567.2-       tgtttcttttccatTTTGCTGTTCCAGTTGAAGAAACCATATATGCCTGTATCTTTATCC
ERR272355.315072.2-       tgtttcttttccatTTTGCTGTTCCAGTTGAAGAAACCATATATGCCTGTATCTTTATCC
ERR272362.246723.2+       tgtttcttttccatTTTGCTGTTCCAGTTGAAGAAACCATATATGCCTGTATCTTTATCC
ERR272362.295779.2+       tgtttcttttccatTTTGCTGTTCCAGTTGAAGAAACCATATATGCCTGTATCTTTATCC
ERR272369.398363.2+       tgtttcttttccatTTTGCTGTTCCAGTTGAAGAAACCATATATGCCTGTATCTTTATCC
ERR272369.424797.2+       tgtttcttttccatTTTGCTGTTCCAGTTGAAGAAACCATATATGCCTGTATCTTTATCC
ERR272370.311833.2+       t--ttcttttccatTTTGCTGTTCTGGCTGAAGAAACCATATATGCCTGTATTTTTATCC
ERR272379.96933.2+        tgtttcttttccatTTTGCTGTTCCAGTTGAAGAAACCATATATGCCTGTATCTTTATCC
ERR272380.163688.2+       tgtttcttttccatTTTGCTGTTCCAGTTGAAGAAACCATATATGCCTGTATCTTTATCC
ERR272388.192361.4-       tgtttcttttccatTTTGCTGTTCCAGTTGAAGAAACCATATATGCCTGTATCTTTATCC
ERR272410.221315.2-       t--ttcttttccatTTTGCTGTTCTGGCTGAAGAAACCATATATGCCTGTATTTTTATCC
ERR244145.102457280.2-      tttcttttccatTGTGCTGTTCCAGTTGAAGAAACCATATATGCCTGTATCTTTATCC
ERR244146.143998000.1+      tttcttttccatTTTGCTGTTCCAGTTGAAGAAACCATATATGCCTGTATCTTTATCC
ERR244147.125423016.1-      tttcttttccatGTTGCTGTTCCAGTTGAAGAAACCATATATGCCTGTATCTTTATCC
ERR244151.49224522.1-       tttcttttccatTTTGCTGTTCCAGTTGAAGAAACCATATATGCCTGTATCTTTATCC
ERR244156.123104822.1-      tttcttttccatTTTGCTGTTCCAGTTGAAGAAACCATATATGCCTGTATCTTTATCC
ERR244156.158051380.1-      tttcttttccatTTTGCTGTTCCAGTTGAAGAAACCATATATGCCTGTATCTTTATCC
ERR244145.28586638.1+        ttcttttccatTTTGCTGTTCCAGTTGAAGAAACCATATATGCCTGTATCTTTATCC
ERR244146.87320608.2-         tcttttccatTTTGCTGTTCCATTTGAAGAAACCATATATGCCTGTATCTTTATCC
ERR244153.60364740.2+            tttccatTTTGCTGTTCTGGCTGAAGAAACCATATATGCCTGTATTTTTATCC
ERR244153.61154323.2+            tttccatTTTGCTGTTCTGGCTGAAGAAACCATATATGCCTGTATTTTTATCC
ERR244153.76184448.2+            tttccatTTTGCTGTTCTGGCTGAAGAAACCATATATGCCTGTATTTTTATCC
ERR244146.17188823.2-             ttccgtTTTGCTGTTCTGGCTGAAGAAACCATATATGCCTGTATTTTTATCC
ERR244151.2144457.2+              ttccatTTTGCTGTTCTGGCTGAAGAAACCATATATGCCTGTATTTTTATCC
ERR244151.27783561.2+             ttccatTTTGCTGTTCTGGCTGAAGAAACCATATATGCCTGTATTTTTATCC
ERR244151.87450210.2+             ttccatTTTGCTGTTCTGGCTGAAGAAACCATATATGCCTGTATTTTTATCC
ERR244152.19946562.2+             ttccatTTTGCTGTTCCAGTTGAAGAAACCATATATGCCTGTATCTTTATCC
ERR244152.93570614.2+             ttccatTTTGCTGTTCCAGTTGAAGAAACCATATATGCCTGTATCTTTATCC
ERR244151.24279433.1+              tccatTTTGCTGTTCCAGTTGAAGAAACCATATATGCCTGTATCTTTATCC
ERR244151.5056026.1+               tccatTTTGCTGTTCCAGTTGAAGAAACCATATATGCCTGTATCTTTATCC
ERR244151.59631049.1+              tccatTTTGCTGTTCCAGTTGAAGAAACCATATATGCCTGTATCTTTATCC
ERR244152.29166132.2-              tccatTTTGCTGTTCCAGTTGAAGAAACCATATATGCCTGTATCTTTATCC
ERR244145.99102567.2+               ccatTTTGCTGTTCCAGTTGAAGAAACCATATATGCCTATATCTTTATCC
ERR244145.85866834.2+                 atTTTGCTGTTCCAGTTGAAGAAACCATATATGCCTGTATCTTTATCC
ERR244158.44889252.2+                 atTTTGCTGTTCCAGTTGAAGAAACCATATATGCCTGTATCTTTATCC
ERR244146.32662140.2+                  tTTTGCTGTTCTGGCTGAAGAAACCATATATGCCTGTATTTTTATCC
ERR244150.49425094.1-                   TTTGCTGTTCTGGCTGAAGAAACCATATATGCCTGTATTTTTATCC
ERR244149.74274799.1-                    TTGCTGTTCTGGCTGAAGAAACCATATATGCCTGTATTTTTATCC
ERR244156.75594749.1-                    TTGCTGTTCCAGTTGAAGAAACCATATATGCCTGTATCTTTATCC
ERR244152.58273483.2+                     TGCTGTTCCAGTTGAAGAAACCATATATGCCTGTATCTTTATCC
ERR244156.172438389.1+                    TGCTGTTCTGGCTGAAGAAACCATATATGCCTGTATTTTTATCC
ERR244145.106184922.2-                     GCTGTTCTGGCTGAAGAAACCATATATGCCTGTATTTTTATCC
ERR244156.35228088.1-                       CTGTTCTGGCTGAAGAAACCATATATGCCTGTATTTTTATCC
ERR244156.98634684.1-                       CTGTTCTGGCTGAAGAAACCATATATGCCTGTATTTTTATCC
ERR244149.86112113.1-                        TGTTCCAGTTGAAGAAACCATATATGCCTGTATCTTTATCC
ERR244156.71030092.1+                        TGTTCTGGCTGAAGAAACCATATATGCCTGTATTTTTATCC
ERR244156.8380816.1+                         TGTTCTGGCTGAAGAAACCATATATGCCTGTATTTTTATCC
ERR244157.76016802.2-                        TGTTCCAGTTGAAGAAACCATATATGCCTGTATCTTTATCC
ERR244151.4381639.2+                          GTTCCAGTTGAAGAAACCATATATGCCTGTATCTTTATCC
ERR244151.46039351.2+                         GTTCCAGTTGAAGAAACCATATATGCCTGTATCTTTATCC
ERR244147.108026216.2-                         TTCTGGCTGAAGAAACCATATATGCCTGTATTTTTATCC
ERR244147.114799832.2-                         TTCCAGTTGAAGAAACCATATATGCCTGTATCTTTATCC
ERR244147.141632726.1+                         TTCTGGCTGAAGAAACCATATATGCCTGTATTTTTATCC
ERR244147.89284177.1-                           TCCAGTTGAAGAAACCATATATGCCTGTATCTTTATCC
ERR244149.90514765.1+                            CTGGCTGAAGAAACCATATATGCCTGTATTTTTATCC
ERR244152.91031925.1+                            CTGGCTGAAGAAACCATATATGCCTGTATTTTTATCC
ERR244153.20887662.1+                            CTGGCTGAAGAAACCATATATGCCTGTATTTTTATCC
ERR244153.21581689.1+                            CTGGCTGAAGAAACCATATATGCCTGTATTTTTATCC
ERR244156.132035253.1-                           CCAGTTGAAGAAACCATATATGCCTGTATCTTTATCC
ERR244145.22441587.1-                             CAGTTCAAGAAACCATATATGCCTGTATCTTTATCC
ERR244147.127947064.1+                            TGGCTGAAGAAACCATATATGCCTGTATTTTTATCC
ERR244148.62507049.1-                             TGGCTGAAGAAACCATATATGCCTGTATTTTTATCC
```

```
ERR244149.12363331.2-                            TGGCTGAAGAAACCATATATGCCTGTATTTTTATCC
ERR244150.74716954.2-                            TGGCTGAAGAAACCATATATGCCTGTATTTTTATCC
ERR244146.41255339.1+                             AGTTGAAGAAACCATATATGCCTGTATCTTTATCC
ERR244147.2040343.1-                              AGTTCAAGAAACCATATATGCCTGTATCTTTATCC
ERR244150.64900968.2-                              GTTGAAGAAACCATATATGCCTGTATCTTTATCC
ERR244151.38435986.1-                              GCTGAAGAAACCATATATGCCTGTATTTTTATCC
ERR244145.150059830.1+                              TTGAAGAAACCATATATGCCTGTATCTTTATCC
ERR244147.113261504.2-                              CTGAAGAAACCATATATGCCTGTATTTTGATCC
ERR244148.54289957.2+                               CTGAAGAAACCATATATGCCTGTATTTTTATCC
ERR244151.22847956.1-                               TTGAAAAAACCATATATGCCTGTATCTTTATCC
ERR244156.22399241.1+                               CTGAAGAAACCATATATGCCTGTATTTTTATCC
ERR244153.31128811.2+                                TGAAGAAACCATATATGCCTGTATCTTTATCC
ERR244147.480761.1+                                   GAAGAAACCATATATGCCTGTATCTTTATCC
ERR244151.37870396.1+                                 GAAGAAACCATATATGCCTGTATCTTTATCC
ERR244151.72885524.1+                                 GAAGAAACCATATATGCCTGTATCTTTATCC
ERR244151.7519035.1+                                  GAAGAAACCATATATGCCTGTATCTTTATCC
ERR244151.84144004.1+                                 GAAGAAACCATATATGCCTGTATCTTTATCC
ERR244156.141617992.2-                                GAAGAAACCATATATGCCTGTATCTTTATCC
ERR244145.161060100.1-                                 AAGAAACCATATATGCCTGTATTTTTATCC
ERR244145.63687745.2-                                  AAGAAACCATATATGCCTGTATTTTTATCC
ERR244157.81766401.1+                                  AAGAAACCATATATGCCTGTATTTTTATCC
ERR244157.22087889.1+                                   AGAAACCATATATGCCTGTATTTTTATCC
ERR244157.38131372.1+                                   AGAAACCATATATGCCTGTATTTTTATCC
ERR244147.51409415.2-                                     AAACCATATATGCCTGTATTTTTATCC
ERR244147.81087395.1+                                     AAACCATATATGCCTGTATCTTTATCC
ERR244145.46643170.1-                                       ACCATATATGCCTGTATTTTTATCC
ERR272418.23402.2+                                          ACCATATATGCCTGTATCTTTATCC
ERR244147.15526174.2-                                        CCATATATGCCTGTATTTTGATCC
ERR244153.20163228.1-                                        CCATATATGCCTGTATCTTTATCC
ERR272417.79284.2+                                           CCATATATGCCTGTATCTTTATCC
ERR244145.30608194.1-                                         CATATATGCCTGTATTTTTATCC
ERR244146.44976138.2+                                          ATATATGCCTGTATCTTTATCC
ERR244150.13115232.2-                                          ATATATGCCTGTATCTTTATCC
ERR244151.86796295.1-                                           TATATGCCTGTATTTTTATCC
ERR244154.2336237.1-                                            TATATGCCTGTATCTTTATCC
ERR244154.49322334.1-                                           TATATGCCTGTATCTTTATCC
ERR244154.72463811.1-                                           TATATGCCTGTATCTTTATCC
ERR244154.73689221.1-                                           TATATGCCTGTATCTTTATCC
ERR244154.74065131.1-                                           TATATGCCTGTATCTTTATCC
ERR244156.61862812.1-                                           TATATGCCTGTATCTTTATCC
ERR244156.148284162.2+                                            TATGCCTGTATCTTTATCC
ERR244156.156625878.2+                                            TATGCCTGTATCTTTATCC
ERR244146.92455626.2-                                              ATGCCTGTATTTTTATCC
ERR244148.51730682.2-                                              ATGCCTGTATCTTTATCC
ERR244147.97325321.1+                                               TGCCTGTATCTTTATCC
ERR244146.59569612.1-                                                 CCTGTATCTTTATCC
ERR244156.50761240.2-                                                  CTGTATTTTTATCC
ERR244149.56907293.2+                                                   TGTATCTTTATCC
ERR244153.2875418.1-                                                     GTATCTTTATCC
ERR244153.6010620.1-                                                     GTATCTTTATCC
ERR244153.77080599.1-                                                    GTATCTTTATCC
ERR244153.84972404.1-                                                    GTATCTTTATCC
ERR244157.24600673.2-                                                    GTATTTTTATCC
ERR244158.14554932.2-                                                    GTATTTTTATCC
ERR244158.3507344.1-                                                     GTATCTTTATCC
ERR244158.39975997.2-                                                    GTATTTTTATCC
ERR244145.28586638.2-                                                     TATCTTTATCC
ERR244146.39980490.1-                                                     TATCTTTATCC
ERR244146.135918198.2+                                                     ATCTTTATCC
ERR244153.20196093.2-                                                      ATTTTTATCC
ERR244153.664339.2-                                                        ATTTTTATCC
ERR244147.72973498.2-                                                       TCTTTATGC
ERR272416.127715.2+                                                         TCTTTATCC
ERR244156.75407830.2+                                                        CTTTATCC
ERR244148.1793981.2-                                                          TTTATCC
ERR244156.114525817.2-                                                        TTTATCC
ERR244156.123492829.1-                                                         TTATCC
ERR244157.7672243.1+                                                           TTATCC
ERR244149.71739235.1+                                                             TCC
ERR244156.27153515.2+                                                             TCC

consensus                tgtttcttttccatTTTGCTGTTCTGGCTGAAGAAACCATATATGCCTGTATCTTTATCC

                             .    :    .    :    .    :    .    :    .    :    .    :
ERR244156.123492829.2+   C
ERR244156.88532666.2-    C
ERR244146.68666943.2+    CAT
ERR244151.777093.1+      CAT
ERR244146.103365155.1-   CATT
ERR244149.25775136.1+    CATT
ERR244150.442652.1+      CATT
ERR244156.130034420.1+   CATTG
ERR244157.28744060.1+    CATTG
ERR244147.12202203.1+    CATTGT
```

ERR244147.4157084.1+ CATTGT
ERR244158.40825569.2- CATTGT
ERR244147.22728488.1- CATTGTC
ERR244147.25647165.2- CATTGTC
ERR244151.17502359.2- CATTGTC
ERR244156.61862812.2+ CATTGTC
ERR244146.124845523.2- CATTGTCAA
ERR244147.17572057.2- CATTGTCAA
ERR244158.26979419.2- CATTGTCAA
ERR244146.140222926.2+ CATTGTCAAGC
ERR244157.83398179.2- CATTGTCAAGCA
ERR244146.146536627.2- CATTGTCAAGCAG
ERR244145.46160951.1+ CATTGTCACGCAGAA
ERR244145.57384943.2+ CATTGTTAAGCAGAAA
ERR244156.39426149.2+ CATTGTCAAGCAGAAA
ERR244157.2304262.1- CATTGTCAAGCAGAAA
ERR244146.91490918.1+ CATTGTCCAGCAGAAAC
ERR244156.38707225.2+ CATTGTCAAGCAGAAAC
ERR244148.51037509.1+ CATTGTCAAGCAGAAACA
ERR244145.127347530.2+ CATTGTCAAGCAGAAACACC
ERR244145.37775792.1- CATTGTCAAGCAGAAACACCAT
ERR244157.83604973.1+ CATTGTCAAGCAGAAACACCAT
ERR244148.6823651.1- CATTGTCAAGCAGAAACACCATT
ERR244146.29560179.1- CATTGTCAAGCAGAAACACCATTA
ERR244146.52725920.1- CATTGTCAAGCAGAAACACCATTA
ERR244153.55115941.2+ CATTGTCAAGCAGAAACACCATTA
ERR244158.31623411.1+ CATTGTCAAGCAGAAACACCATTA
ERR244145.102457280.1+ CATTGTCAAGCAGAAACGCCATTAT
ERR244145.110237827.2- CATTGTCAAGCAGAAACACCATTATC
ERR244145.96068630.1- CATTGTCAAGCAGAAACACCATTATC
ERR244146.90665826.1- CATTGTCAAGCAGAAACACCATTATC
ERR244148.41993600.2- CATTGTCAAGCAGAAACACCATTATC
ERR244146.87059851.2- CATTGTCAAGCAGAAACACCATTATCT
ERR244156.16052576.1- CATTGTCAAGCAGAAACACCATTATCT
ERR244157.24672276.1+ CATTGTCAAGCAGAAACACCATTATCT
ERR244157.68534216.2+ CATTGTCAAGCAGAAACACCATTATCT
ERR244145.39604221.2- CATTGTCAAGCAGAAACACCATTATCTA
ERR244150.15347188.2+ CATTGTCAAGCAGAAACACCATTATCTA
ERR244152.2384579.1+ CATTGTCAAGCAGAAACACCATTATCTA
ERR244152.73646274.1+ CATTGTCAAGCAGAAACACCATTATCTA
ERR244156.40198159.1- CATTGTCAAGCAGAAACACCATTATCTA
ERR244158.28053158.1+ CATTGTCAAGCAGAAACACCATTATCTA
ERR244156.134587516.1- CATTGTCAAGCAGAAACACCATTATCTACAA
ERR244151.49224522.1- CATTGTCAAGCAGAAACACCATTATCTACAAG
ERR244158.12039058.1+ CATTGTCAAGCAGAAACACCATTATCTACAAG
ERR244158.14530390.1- CATTGTCAAGCAGAAACACCATTATCTACAAG
ERR244146.150494649.2- CATTGTCAAGCAGAAACACCATTATCTACAAGg
ERR244156.162215319.2+ CATTGTCAAGCAGAAACACCATTATCTACAAGg
ERR244156.17686513.1- CATTGTCAAGCAGAAACACCATTATCTACAAGg
ERR244157.88933338.1+ CATTGTCAAGCAGAAACACCATTATCTACAAGg
ERR244156.154736233.1+ CATTGTCAAGCAGAAACACCATTATCTACAAGgt
ERR244157.16960720.2- CATTGTCAAGCAGAAACACCATTATCTACAAGgt
ERR244146.118918090.2- CATTGTCAAGCAGAAACACCATTATCTACAAGgta
ERR244156.27908911.1- CATTGTCAAGCAGAAACACCATTATCTACAAGgtaa
ERR244156.47741673.1+ CATTGTCAAGCAGAAACACCATTATCTACAAGgtaa
ERR244157.40217339.2+ CATTGTCAAGCAGAAACACCATTATCTACAAGgtaa
ERR244146.97675664.1+ CATTGTCAAGCAGAAACAGCATTATCTACAACgtaaa
ERR244153.60364740.2+ CATTGTCAAGCAGAAACACCATTATCTACAAGgtaaa
ERR244153.61154323.2+ CATTGTCAAGCAGAAACACCATTATCTACAAGgtaaa
ERR244153.76184448.2+ CATTGTCAAGCAGAAACACCATTATCTACAAGgtaaa
ERR244156.120584320.1+ CATTGTCAAGCAGAAACACCATTATCTACAAGgtaaa
ERR244145.19972568.2- CATTGTCAAGCAGAAACACCATTATCTACAAGgtaaat
ERR244146.107304693.2+ CATTGTCAAGCAGAAACACCATTATCTACAAGggaaat
ERR244147.82193546.1- CATTGTCAAGCAGAAACACCATTATCTACAAGgtaaat
ERR244151.2144457.2+ CATTGTCAAGCAGAAACACCATTATCTACAAGgtaaat
ERR244151.27783561.2+ CATTGTCAAGCAGAAACACCATTATCTACAAGgtaaat
ERR244151.87450210.2+ CATTGTCAAGCAGAAACACCATTATCTACAAGgtaaat
ERR244152.19946562.2+ CATTGTCAAGCAGAAACACCATTATCTACAAGgtaaat
ERR244152.93570614.2+ CATTGTCAAGCAGAAACACCATTATCTACAAGgtaaat
ERR244151.24279433.1+ CATTGTCAAGCAGAAACACCATTATCTACAAGgtaaatt
ERR244151.5056026.1+ CATTGTCAAGCAGAAACACCATTATCTACAAGgtaaatt
ERR244151.59631049.1+ CATTGTCAAGCAGAAACACCATTATCTACAAGgtaaatt
ERR244152.29166132.2- CATTGTCAAGCAGAAACACCATTATCTACAAGgtaaatt
ERR244157.71374123.1+ CATTGTCAAGCAGAAACACCATTATCTACAAGgtaaatttg
ERR244158.23421992.2+ CATTGTCAAGCAGAAACACCATTATCTACAAGgtaaatttg
ERR244145.22441587.2+ CATTGTCAAGCAGAAACACCATTATCTACAAGgtaaatttgca
ERR244145.89602273.1- CATTGTCAAGCAGAAACACCATTATCTACAAGgtaaatttgca
ERR244146.143563595.1- CATTGTCAAGCAGAAACACCATTATCTACAAGgtaaatttgca
ERR244156.123104822.1- CATTGTCAAGCAGAAACACCATTATCTACAAGgtaaatttgca
ERR244156.158051380.1- CATTGTCAAGCAGAAACACCATTATCTACAAGgtaaatttgca
ERR244146.109494033.2+ CATTGTCAAGCAGAAACACCATTATCTACAAGgtaaatttgcag
ERR244150.49425094.1- CATTGTCAAGCAGAAACACCATTATCTACAAGgtaaatttgcag
ERR244149.74274799.1- CATTGTCAAGCAGAAACACCATTATCTACAAGgtaaatttgcagg
ERR244147.69941180.1- CATTGTCAAGCAGAAACACCATTATCTACAAGgtaaatttgcaggc

ERR244152.58273483.2+   CATTGTCAAGCAGAAACACCATTATCTACAAGgtaaatttgcaggc
ERR244146.75918108.1-   CATTGTCAAGCAGAAACACCATTATCTACAAGgtaaatttgcaggctt
ERR244151.4381639.2+   CATTGTCAAGCAGAAACACCATTATCTACAAGgtaaatttgcaggctt
ERR244151.46039351.2+   CATTGTCAAGCAGAAACACCATTATCTACAAGgtaaatttgcaggctt
ERR244149.86112113.1-   CATTGTCAAGCAGAAACACCATTATCTACAAGgtaaatttgcaggcttg
ERR244145.106184922.1+   CATTGTCAAGCAGAAACACCATTATCTACACGgtaaatttgcaggcttgtc
ERR244147.121272221.1-   CATTGTCAAGCAGAAACACCATTATCTACAAGgtaaatttgcaggcttgtc
ERR244145.17810775.1-   CATTGTCAAGCAGAAACACCATTATCTACAAGgtaaatttgcaggcttgtca
ERR244149.90514765.1+   CATTGTCAAGCAGAAACACCATTATCTACAAGgtaaatttgcaggcttgtcac
ERR244152.91031925.1+   CATTGTCAAGCAGAAACACCATTATCTACAAGgtaaatttgcaggcttgtcac
ERR244153.20887662.1+   CATTGTCAAGCAGAAACACCATTATCTACAAGgtaaatttgcaggcttgtcac
ERR244153.21581689.1+   CATTGTCAAGCAGAAACACCATTATCTACAAGgtaaatttgcaggcttgtcac
ERR244158.44889252.2+   CATTGTCAAGCAGAAACACCATTATCTACAAGgtaaatttgcaggcttgtcac
ERR244148.62507049.1-   CATTGTCAAGCAGAAACACCATTATCTACAAGgtaaatttgcaggcttgtcaca
ERR244149.12363331.2-   CATTGTCAAGCAGAAACACCATTATCTACAAGgtaaatttgcaggcttgtcaca
ERR244150.74716954.2-   CATTGTCAAGCAGAAACACCATTATCTACAAGgtaaatttgcaggcttgtcaca
ERR244150.64900968.2-   CATTGTCAAGCAGAAACACCATTATCTACAAGgtaaatttgcaggcttgtcacaac
ERR244151.38435986.1-   CATTGTCAAGCAGAAACACCATTATCTACAAGgtaaatttgcaggcttgtcacaac
ERR244156.75594749.1-   CATTGTCAAGCAGAAACACCATTATCTACAAGgtaaatttgcaggcttgtcacaac
ERR244146.49370712.2-   CATTGTCAAGCAGAAACACCATTATCTACAAGgtaaatttgcaggcttgtcacaact
ERR244148.54289957.2+   CATTGTCAAGCAGAAACACCATTATCTACAAGgtaaatttgcaggcttgtcacaact
ERR244151.22847956.1-   CATTGTCAAGCAGAAACACCATTATCTACAAGgtaaatttgcaggcttgtcacaact
ERR244156.172438389.1+   CATTGTAAAGCAGAAACACCATTATCTACAAGgtaaatttgcaggcttgtcacaact
ERR244157.76016802.2-   CATTGTCAAGCAGAAACACCATTATCTACAAGgtaaatttgcaggcttgtcacaact
ERR244153.31128811.2+   CATTGTCAAGCAGAAACACCATTATCTACAAGgtaaatttgcaggcttgtcacaactg
ERR244151.37870396.1+   CATTGTCAAGCAGAAACACCATTATCTACAAGgtaaatttgcaggcttgtcacaactga
ERR244151.72885524.1+   CATTGTCAAGCAGAAACACCATTATCTACAAGgtaaatttgcaggcttgtcacaactga
ERR244151.7519035.1+   CATTGTCAAGCAGAAACACCATTATCTACAAGgtaaatttgcaggcttgtcacaactga
ERR244151.84144004.1+   CATTGTCAAGCAGAAACACCATTATCTACAAGgtaaatttgcaggcttgtcacaactga
ERR244156.35228088.1-   CATTGTCAAGCAGAAACACCATTATCTACAAGgtaaatttgcaggcttgtcacaactga
ERR244156.98634684.1-   CATTGTCAAGCAGAAACACCATTATCTACAAGgtaaatttgcaggcttgtcacaactga
ERR244145.102457280.2-   CATTGTCAAGTAGAAACGCCATTATCTACAAGgtaaatttgcaggcttgtcacaactgag
ERR244145.106184922.2-   CATTGTCAAGCAGAAACACCATTATCTACAAGgtaaatttgcaggcttgtcacaactgag
ERR244145.127347530.1-   CATTGTCAAGCAGAAACACCATTATCTACAAGgtaaatttgcaggcttgtcacaactgag
ERR244145.150059830.1+   CATTGTCAAGCAGAAACACCATTATCTACAAGgtaaatttgcaggcttgtcacaactgag
ERR244145.161060100.1-   CATTGTCAAGCAGAAACACCATTATCTACAAGgtaaatttgcaggcttgtcacaactgag
ERR244145.22441587.1-   CATTGTCAAGCAGAAACACCATTATCTACAAGgtaaatttgcaggcttgtcacaactgag
ERR244145.28586638.1+   CATTGTCAAGCAGAAACACCATTATCTACAAGgtaaatttgcaggcttgtcacaactgag
ERR244145.28586638.2-   CATTGTCAAGCAGAAACACCATTATCTACAAGgtaaatttgcaggcttgtcacaactgag
ERR244145.30608194.1-   CATTGTCAAGCAGAAACACCATTATCTACAAGgtaaatttgcaggcttgtcacaactgag
ERR244145.30608194.2+   CATTGTCAAGCAGAAACACCATTATCTACAAGgtaaatttgcaggcttgtcacaactgag
ERR244145.46160951.2-   CATTGTCAAGCAGAAACACCATTATCTACAAGgtaaatttgcaggcttgtcacaactgag
ERR244145.46643170.1-   CATTGTCAAGCAGAAACACCATTATCTACAAGgtaaatttgcaggcttgtcacaactgag
ERR244145.46643170.2+   CATTGTCAAGCAGAAACACCATTATCTACAAGgtaaatttgcaggcttgtcacaactgag
ERR244145.48548398.2+   CATTGTCAAGCAGAAACACCATTATCTACAAGgtaaatttgcaggcttgtcacaactgag
ERR244145.57384943.1-   CATTGTCAAGCAGAAACACCATTATCTACAAGgtaaatttgcaggcttgtcacaactgag
ERR244145.63687745.1+   CATTGTCAAGCAGAAACACCATTATCTACAAGgtaaatttgcaggcttgtcacaactgag
ERR244145.63687745.2-   CATTGTCAAGCAGAAACACCATTATCTACAAGgtaaatttgcaggcttgtcacaactgag
ERR244145.80053276.2+   CATTGTCAAGCAGAAACACCATTATCTACAAGgtaaatttgcaggcttgtcacaactgag
ERR244145.85866834.1-   CATTGTCAAGCAGAAACACCATTATCTACAAGgtaaatttgcaggcttgtcacaactgag
ERR244145.85866834.2+   CATTGTCAAGCAGAAACACCATTATCTACAAGgtaaatttgcaggcttgtcacaactgag
ERR244145.98207251.2+   CATTGTCAAGCAGAAACACCATTATCTACAAGgtaaatttgcaggcttgtcacaactgag
ERR244145.99102567.2+   CATTGTCAAGCAGAAACACCATTATCTACAAGgtaaatttgcaggcttgtcacaactgag
ERR244146.116947821.2-   CATTGTCAAGCAGAAACACCATTATCTACAAGgtaaatttgcaggcttgtcacaactgag
ERR244146.118142077.2+   CATTGTCAAGCAGAAACACCATTATCTACAAGgtaaatttgcaggcttgtcacaactgag
ERR244146.135918198.2+   CATTGTCAAGCAGAAACACCATTATCTACAAGgtaaatttgcaggcttgtcacaactgag
ERR244146.14354249.2-   CATTGTCAATCAGAAACCCCACTATCTACAAGgtaaatttgcaggcttgtcacaactgag
ERR244146.143998000.1+   CATTGTCAAGCAGAAACACCATTATCTACAAGgtaaatttgcaggcttgtcacaactgag
ERR244146.17188823.2-   CATTGTCAAGCAGAAACACCATTATCTACAAGgtaaatttgcaggcttgtcacaactgag
ERR244146.32662140.2+   CATTGTCAAGCAGAAACACCATTATCTACAAGgtaaatttgcaggcttgtcacaactgag
ERR244146.39980490.1-   CATTGTCAAGCAGAAACACCATTATCTACAAGgtaaatttgcaggcttgtcacaactgag
ERR244146.41255339.1+   CATTGTCAAGCAGAAACACCATTATCTACAAGgtaaatttgcaggcttgtcacaactgag
ERR244146.44976138.2+   CATTGTCAAGCAGAAACACCATTATCTACAAGgtaaatttgcaggcttgtcacaactgag
ERR244146.47506220.2-   CATTGTCAAGCAGAAACACCATTATCTACAAGgtaaatttgcaggcttgtcacaactgag
ERR244146.59569612.1-   CATTGTCAAGCAGAAACACCATTATCTACAAGgtaaatttgcaggcttgtcacaactgag
ERR244146.64167387.2+   CATTGTCAAGCAGAAACACCATTATCTACAAGgtaaatttgcaggcttgtcacaactgag
ERR244146.77596753.1+   CATTGTCAAGCAGAAACACCATTATCTACAAGgtaaatttgcaggcttgtcacaactgag
ERR244146.87320608.2-   CATTGTCAAGTAGAAACACCATTATCTACAAGgtaaagttgcaggcttgtcacaactgag
ERR244146.92455626.2-   CATTGTCAAGCAGAAACACCATTATCTACAAGgtaaatttgcaggcttgtcacaactgag
ERR244146.9290774.1+   CATTGTCAAGCAGAAACACCATTATCTACAAGgtaaatttgcaggcttgtcacaactgag
ERR244147.10275342.2-   CATTGTCAAGCAGAAACACCATTATCTACAAGgtaaatttgcaggcttgtcacaactgag
ERR244147.108026216.2-   CATTGTCAAGCAGAAACACCATTATCTACAAGgtaaatttgcaggcttgtcacaactgag
ERR244147.113261504.2-   CATTGTCAAGCAGAAACACCATTATCTACAAGgtaaatttgcaggcttgtcacaactgag
ERR244147.114799832.2-   CATTGTCAAGCAGAAACACCATTATCTACAAGgtaaatttgcaggcttgtcacaactgag
ERR244147.125423016.1-   CATTGTCAAGCAGAAACACCATTATCTACAAGgtaaatttgcaggcttgtcacaactgag
ERR244147.127947064.1+   CATTGTCAAGCAGAAACACCATTATCTACAAGgtaaatttgcaggcttgtcacaactgag
ERR244147.132045579.1-   CATTGTCAAGCAGAAACACCATTATCTACAAGgtaaatttgcaggcttgtcacaactgag
ERR244147.136713187.1+   CATTGTCAAGCAGAAACACCATTATCTACAAGgtaaatttgcaggcttgtcacaactgag
ERR244147.141632726.1+   CATTGTCAAGCAGAAACACCATTATCTACAAGgtaaatttgcaggcttgtcacaactgag
ERR244147.15526174.2-   CATTGTCAAGCAGAAACACCATTATCTACAAGgtaaatttgcaggcttgtcacaactgag
ERR244147.15961227.2+   CATTGTCAAGCAGAAACACCATTATCTACAAGgtaaatttgcaggcttgtcacaactgag
ERR244147.2040343.1-   CATTGTCAAGCAGAAACACCATTATCTACAAGgtaaatttgcaggcttgtcacaactgag
ERR244147.480761.1+   CATTGTCAAGCAGAAACACCATTATCTACAAGgtaaatttgcaggcttgtcacaactgag
ERR244147.51409415.2-   CATGTTCAAGCAGAAACACCATTATCTACAAGgtaaatttgcaggcttgtcacaactgag

```
ERR244147.72973498.2-    CATGATCAAGCAGAAACACCATTATCAACAAGgtaaattttcaggcttggcacaactgag
ERR244147.77801619.2-    CATTGTCAAGCAGAAACACCATTATCTACAAGgtaaatttgcaggcttgtcacaactgag
ERR244147.81087395.1+    CATTGTCAAGCAGAAACACCATTATCTACAAGgtaaatttgcaggcttgtcacaactgag
ERR244147.89284177.1-    CATTGTCAAGCAGAAACACCATTATCTACAAGgtaaatttgcaggcttgtcacaactgag
ERR244147.97325321.1+    CATTGTCAAGCAGAAACACCATTATCTATAAGgtaaatttgcaggcttgtcacaactgag
ERR244148.1793981.2-     CATTGTCAAGCAGAAACACCATTATCTACAAGgtaaatttgcaggcttgtcacaactgag
ERR244148.51730682.2-    CATTGTCAAGCAGAAACACCATTATCTACAAGgtaaatttgcaggcttgtcacaactgag
ERR244149.56907293.2+    CATTGTCAAGCAGAAACACCATTATCTACAAGgtaaatttgcaggcttgtcacaactgag
ERR244149.71739235.1+    CATTGTCAAGCAGAAACACCATTATCTACAAGgtaaatttgcaggcttgtcacaactgag
ERR244150.13115232.2-    CATTGTCAAGCAGAAACACCATTATCTACAAGgtaaatttgcaggcttgtcacaactgag
ERR244151.86796295.1-    CATTGTCAAGCAGAAACACCATTATCTACAAGgtaaatttgcaggcttgtcacaactgag
ERR244153.20163228.1-    CATTGTCAAGCAGAAACACCATTATCTACAAGgtaaatttgcaggcttgtcacaactgag
ERR244153.20196093.2-    CATTGTCAAGCAGAAACACCATTATCTACAAGgtaaatttgcaggcttgtcacaactgag
ERR244153.2875418.1-     CATTGTCAAGCAGAAACACCATTAGCTACAAGgtaaatttgcaggcttgtcacaactgag
ERR244153.6010620.1-     CATTGTCAAGCAGAAACACCATTATCTACAAGgtaaatttgcaggcttgtcacaactgag
ERR244153.664339.2-      CATTGTCAAGCAGAAACACCATTATCTACAAGgtaaatttgcaggcttgtcacaactgag
ERR244153.77080599.1-    CATTGTCAAGCAGAAACACCATTATCTACAAGgtaaatttgcaggcttgtcacaactgag
ERR244153.84972404.1-    CATTGTCAAGCAGAAACACCATTATCTACAAGgtaaatttgcaggcttgtcacaactgag
ERR244154.2336237.1-     CATTATCAAGCAGAAACACCATTATCTACAAGgtaaatttgcaggcttgtcacaactgag
ERR244154.49322334.1-    CATTGTCAAGCAGAAACACCATTATCTACAAGgtaaatttgcaggcttgtcacaactgag
ERR244154.72463811.1-    CATTGTCAAGCAGAAACACCATTATCTACAAGgtaaatttgcaggcttgtcacaactgag
ERR244154.73689221.1-    CATTGTCAAGCAGAAACACCATTATCTACAAGgtaaatttgcaggcttgtcacaactgag
ERR244154.74065131.1-    CATTGTCAAGCAGAAACACCATTATCTACAAGgtaaatttgcaggcttgtcacaactgag
ERR244156.114525817.2-   CATTGTCAAGGAGAAACACCATTATCTACAAGgtaaatttgcaggcttgtcacaactgag
ERR244156.123492829.1-   CATTGTCAAGCAGAAACACCATTATCTACAAGgtaaatttgcaggcttgtcacaactgag
ERR244156.132035253.1-   CATTGTCAAGCAGAAACACCATTATCTACAAGgtaaatttgcaggcttgtcacaactgag
ERR244156.141617992.2-   CATTGTCAAGCAGAAACACCATTATCTACAAGgtaaatttgcaggcttgtcacaactgag
ERR244156.148284162.2+   CATTGTCAAGCAGAAACACCATTATCTACAAGgtaaatttgcaggcttgtcacaactgag
ERR244156.156625878.2+   CATTGTCAAGCAGAAACACCATTATCTACAAGgtaaatttgcaggcttgtcacaactgag
ERR244156.22399241.1+    CATTGTCAAGCAGAAACACCATTATCTACAAGgtaaatttgcaggcttgtcacaactgag
ERR244156.27153515.2+    CATTGTCAAGCAGAAACACCATTATCTACAAGgtaaatttgcaggcttgtcacaactgag
ERR244156.39426149.1-    CATTGTCAAGCAGAAACACCATTATCTACAAGgtaaatttgcaggcttgtcacaactgag
ERR244156.50761240.2-    CATTGTCAAGCAGAAACACCATTATCTACAAGgtaaatttgcaggcttgtcacaactgag
ERR244156.61862812.1-    CATTGTCAAGCAGAAACACCATTATCTACAAGgtaaatttgcaggcttgtcacaactgag
ERR244156.71030092.1+    CATTGTCAAGCAGAAACACCATTATCTACAAGgtaaatttgcaggcttgtcacaactgag
ERR244156.75407830.2+    CATTGTCAAGCAGAAACACCATTATCTACAAGgtaaatttgcaggcttgtcacaactgag
ERR244156.8380816.1+     CATTGTCAAGCAGAAACACCATTATCTACAAGgtaaatttgcaggcttgtcacaactgag
ERR244157.22087889.1+    CATTGTCAAGCAGAAACACCATTATCTACAAGgtaaatttgcaggcttgtcacaactgag
ERR244157.24600673.2-    CATTGTCAAGCAGAAACACCATTATCTACAAGgtaaatttgcaggcttgtcacaactgag
ERR244157.38131372.1+    CATTGTCAAGCAGAAACACCATTATCTACAAGgtaaatttgcaggcttgtcacaactgag
ERR244157.7672243.1+     CATTGTCAAGCAGAAACACCATTATCTACAAGgtaaatttgcaggcttgtcacaactgag
ERR244157.81766401.1+    CATTGTCAAGCAGAAACACCATTATCTACAAGgtaaatttgcaggcttgtcacaactgag
ERR244158.14554932.2-    CATTGTCAAGCAGAAACACCATTATCTACAAGgtaaatttgcaggcttgtcacaactgag
ERR244158.3507344.1-     CATTGTCAAGCAGAAACACCATTATCTACAAGgtaaatttgcaggcttgtcacaactgag
ERR244158.39975997.2-    CATTGTCAAGCAGAAACACCATTATCTACAAGgtaaatttgcaggcttgtcacaactgag
ERR272293.162385.2-      CATTGTCAAGCAGAAACACCATTATCTACAAGgtaaatttgcaggcttgtcacaactgag
ERR272293.163306.2-      CATTGTCAAGCAGAAACACCATTATCTACAAGgtaaatttgcaggcttgtcacaactgag
ERR272314.115165.2+      CATTGTCAAGCAGAAACACCATTATCTACAAGgtaaatttgcaggcttgtcacaactgag
ERR272317.118222.2-      CATTGTCAAGCAGAAACACCATTATCTACAAGgtaaatttgcaggcttgtcacaactgag
ERR272334.112347.2-      CATTGTCAAGCAGAAACACCATTATCTACAAGgtaaatttgcaggcttgtcacaactgag
ERR272354.158567.2-      CATTGTCAAGCAGAAACACCATTATCTACAAGgtaaatttgcaggcttgtcacaactgag
ERR272355.315072.2-      CATTGTCAAGCAGAAACACCATTATCTACAAGgtaaatttgcaggcttgtcacaactgag
ERR272362.246723.2+      CATTGTCAAGCAGAAACACCATTATCTACAAGgtaaatttgcaggcttgtcacaactgag
ERR272362.295779.2+      CATTGTCAAGCAGAAACACCATTATCTACAAGgtaaatttgcaggcttgtcacaactgag
ERR272369.398363.2+      CATTGTCAAGCAGAAACACCATTATCTACAAGgtaaatttgcaggcttgtcacaactgag
ERR272369.424797.2+      CATTGTCAAGCAGAAACACCATTATCTACAAGgtaaatttgcaggcttgtcacaactgag
ERR272370.311833.2+      CATTGTCAAGCAGAAACACCATTATCTACAAGgtaaatttgcaggcttgtcacaactgag
ERR272379.96933.2+       CATTGTCAAGCAGAAACACCATTATCTACAAGgtaaatttgcaggcttgtcacaactgag
ERR272380.163688.2+      CATTGTCAAGCAGAAACACCATTATCTACAAGgtaaatttgcaggcttgtcacaactgag
ERR272388.192361.4-      CATTGTCAAGCAGAAACACCATTATCTACAAGgtaaatttgcaggcttgtcacaactgag
ERR272410.221315.2-      CATTGTCAAGCAGAAACACCATTATCTACAAGgtaaatttgcaggcttgtcacaactgag
ERR272416.127715.2+      CATTGTCAAGCAGAAACACCATTATCTACAAGgtaaatttgcaggcttgtcacaactgag
ERR272417.79284.2+       CATTGTCAAGCAGAAACACCATTATCTACAAGgtaaatttgcaggcttgtcacaactgag
ERR272418.23402.2+       CATTGTCAAGCAGAAACACCATTATCTACAAGgtaaatttgcaggcttgtcacaactgag
ERR244147.27177651.1-     ATTGGCAAGCACAAACACCATTATCTACAAGgtaaatttgcaggcttgtcacaactgag
ERR244149.14395483.2+     ATTGTCAAGCAGAAACACCATTATCTACAAGgtaaatttgcaggcttgtcacaactgag
ERR244145.7609561.2+       TTGTCAAGCAGAAACACCATTATCTACAAGgtaaatttgcaggcttgtcacaactgag
ERR244156.133816308.1-     TTGTCAAGCAGAAACACCATTATCTACAAGgtaaatttgcaggcttgtcacaactgag
ERR244148.7498070.1+        TGTCAAGCAGAAACACCATTATCTACAAGgtaaatttgcaggcttgtcacaactgag
ERR244146.90803826.1+        GTCAAGCAGAAACACCATTATCTACAAGgtaaatttgcaggcttgtcacaactgag
ERR244149.60575310.2-        GTCAAGCAGAAACACCATTATCTACAAGgtaaatttgcaggcttgtcacaactgag
ERR244154.37716924.1-        GTCAAGCAGAAACACCATTATCTACAAGgtaaatttgcaggcttgtcacaactgag
ERR244158.47537176.1-        GTCAAGCAGAAACACCATTATCTACAAGgtaaatttgcaggcttgtcacaactgag
ERR244156.102713578.1-        TCAAGCAGAAACACCATTATCTACAAGgtaaatttgcaggcttgtcacaactgag
ERR244157.116596890.1+        TCAAGCAGAAACACCATTATCTACAAGgtaaatttgcaggcttgtcacaactgag
ERR244152.17403384.1-          CAAGCAGAAACACCATTATCTACAAGgtaaatttgcaggcttgtcacaactgag
ERR244156.130034420.2-         CAAGCAGAAACACCATTATCTACAAGgtaaatttgcaggcttgtcacaactgag
ERR244156.54583533.1-           AAGCAGAAACACCATTATCTACAAGgtaaatttgcaggcttgtcacaactgag
ERR244147.87008119.2-             GCAGAAACACCATTATCTACAAGgtaaatttgcaggcttgtcacaactgag
ERR244145.59032948.1+               AGAAACACCATTATCTACAAGgtaaatttgcaggcttgtcacaactgag
ERR244148.45966745.2+               AGAAACACCATTATCTACAAGgtaaatttgcaggcttgtcacaactgag
ERR244149.24348199.2+               AGAAACACCATTATCTACAAGgtaaatttgctggcttgtcacaactgag
ERR244149.47523738.2+               AGAAACACCATTATCTACAAGgtaaatttgcaggcttgtcacaactgag
ERR244156.150973147.1+              AGAAACACCATTATCTACAAGgtaaatatgcaggcttgtcacaactgag
```

```
ERR244157.53670734.1+               AGAAACACCATTATCTACAAGgtaaatttgcaggcttgtcacaactgag
ERR244156.57001811.1+                GAAACACCATTATCTACAAGgtaaatttgcaggcttgtcacaactgag
ERR244157.28744060.2-                GAAACACCATTATCTACAAGgtaaatttgcaggcttgtcacaactgag
ERR244157.74836971.2+                GAAACACCATTATCTACAAGgtaaatttgcaggcttgtcacaactgag
ERR244157.99766491.1+                GAAACACCATTATCTACAAGgtaaatttgcaggcttgtcacaactgag
ERR244156.143885543.2+                AAACACCATTATCTACAAGgtaaatttgcaggcttgtcacaactgag
ERR244148.29532907.2-                  AACACCATTATCTACAAGgtaaatttgcaggcttgtcacaactgag
ERR244156.153471681.1+                 AACACCATTATCTACAAGgtaaatttgcaggcttgtcacaactgag
ERR244157.105557526.1+                 AACACCATTATCTACAAGgtaaatttgcaggcttgtcacaactgag
ERR244156.62598249.1-                   ACACCATTATCTACAAGgtaaatttgcaggtttctcacaactgag
ERR244145.99102567.1-                    CACCATTATCTACAAGgtaaatttgcaggcttgtcacaactgag
ERR244156.68083478.1-                    CACCATTATCTACAAGgtaaatttgcaggcttgtcacaactgag
ERR244147.13481294.1+                     ACCATTATCTACAAGgtaaatttgcaggcttgtcacaactgag
ERR244150.43157459.2-                     ACCATTATCTACAAGgtaaatttgcaggcttgtcacaactgag
ERR244150.50826763.2-                     ACCATTATCTACAAGgtaaatttgcaggcttgtcacaactgag
ERR244148.47100029.1+                       CATTATCTACAAGgtaaatttgcaggcttgtcacaactgag
ERR244148.74455538.1-                       CATTATCTACAAGgtaaatttgcaggcttgtcacaactgag
ERR244148.80697691.2+                       CATTATCTACAAGgtaaatttgcaggcttgtcacaactgag
ERR244151.23757510.1-                       CATTATCTACAAGgtaaatttgcaggcttgtcacaactgag
ERR244151.56060598.1-                       CATTATCTACAAGgtaaatttgcaggcttgtcacaactgag
ERR244157.103004017.2-                      CATTATCTACAAGgtaaatttgcaggcttgtcacaactgag
ERR244157.63718191.2-                       CATTATCTACAAGgtaaatttgcaggcttgtcacaactgag
ERR244152.17127458.2+                        ATTATCTACAAGgtaaatttgcaggcttgtcacaactgag
ERR244150.43787057.1-                         TTATCTACAAGgtaaatttgcaggcttgtcacaactgag
ERR244156.38707225.1-                          TATCTACAAGgtaaatttgcaggcttgtcacaactgag
ERR244158.23577951.1+                          TATCTACAAGgtaaatttgcaggcttgtcacaactgag
ERR244146.56067872.1+                           ATCTACAAGgtaaatttgcaggcttgtcacaactgag
ERR244147.100501284.1-                          ATCTACAACgtaaattggcaggcttgacacaactgag
ERR244146.115328676.2+                            CTACAAGgtaaatttgcaggcttgtcacaactgag
ERR244153.13214808.2-                             CTACAAGgtaaatttgcaggcttgtcacaactgag
ERR244153.26823307.1+                             CTACAAGgtaaatttgcaggcttgtcacaactgag
ERR244153.46108787.2-                             CTACAAGgtaaatttgcaggcttgtcacaactgag
ERR244153.53014680.1+                             CTACAAGgtaaatttgcaggcttgtcacaactgag
ERR244153.59606166.1+                             CTACAAGgtaaatttgcaggcttgtcacaactgag
ERR244153.62989800.1+                             CTACAAGgtaaatttgcaggcttgtcacaactgag
ERR244153.90822497.1+                             CTACAAGgtaaatttgcaggcttgtcacaactgag
ERR244158.31623411.2-                             CTACAAGgtaaatttgcaggcttgtcacaactgag
ERR244145.150059830.2-                             TACACGgtaaatttgcaggctgctcacaactgag
ERR244146.103434928.2+                             TACAAGgtaaatttgcaggcttgtcacaactgag
ERR244156.47741673.2-                              TACAAGgtaaatttgcaggcttgtcacaactgag
ERR244150.36647122.1+                               ACAAGgtaaatttgcaggcttgtcacaactgag
ERR244156.93512221.2+                               ACAAGgtaaatttgcaggcttgtcacaactgag
ERR244158.28053158.2-                                CAAGgtaaatttgcaggcttgtcacaactgac
ERR244156.44756902.2+                                 AAGgtaaatttgcaggcttgtcacaactgag
ERR244157.22087889.2-                                 AAGgtaaatttgcaggcttgtcacaactgag
ERR244157.24672276.2-                                 AAGgtaaatttgcaggcttgtcacaactgag
ERR244149.45979634.2+                                  AGgtaaatttgcaggcttgtcacaactgag
ERR244157.81766401.2-                                  AGgtaaatttgcaggcttgtcacaactgag
ERR244158.12039058.2-                                  AGgtaaatttgcaggcttgtcacaactgag

consensus                CATTGTCAAGCAGAAACACCATTATCTACAAGgtaaatttgcaggcttgtcacaactgag
```

M. Saker falcon (No. 15), exon 4, WGS data

- CAP3 alignment of WGS data spanning exon 4 (uppercase letters)

```
                               .    :    .    :    .    :    .    :    .    :    .    :
SRR516271.42383091.2+    tgtgtctttcttttccagTTTG
SRR516271.54300452.2+    tgtgtctttcttttccagTTTG
SRR516271.59598790.2+    tgtgtctttcttttccagTTTG
SRR516269.72607350.2+    tgtgtctttcttttccagTTTGCTGTT
SRR516273.51714565.2+    tgtgtctttcttttccagTTTGCTGTTC
SRR516271.51353961.1-    tgtgtctttcttttccagTTTGCTGTTCCAGTTAA
SRR516271.66205064.1-    tgtgtctttcttttccagTTTGCTGTTCCAGTTAA
SRR516272.54263740.1-    tgtgtctttcttttccagTTTGCTGTTCCAGTTAAGAAACCAAGTAT
SRR516276.64208609.1+    tgtgtctttcttttccagTTTGCTGTTCCAGTTAAGAAACCAAGTATGCT
SRR516278.25692502.1-    tgtgtctttcttttccagTTTGCTGTTCCAGTTAAGAAACCAAGTATGCTTGT
SRR516279.48286067.1-    tgtgtctttcttttccagTTTGCTGTTCCAGTTAAGAAACCAAGTATGCTTGT
SRR516278.43431721.2-    tgtgtctttcttttccagTTTGCTGTTCCAGTTAAGAAACCAAGTATGCTTGTA
SRR516280.64868542.1+    tgtgtctttcttttccagTTTGCTGTTCCAGTTAAGAAACCAAGTATGCTTGTAC
SRR516278.21204016.2+    tgtgtctttcttttccagTTTGCTGTTCCAGTTAAGAAACCAAGTATGCTTGTACTT
SRR516275.49486814.2-    tgtgtctttcttttccagTTTGCTGTTCCAGTTAAGAAACCAAGTATGCTTGTACTTT
SRR516279.60818054.2-    tgtgtctttcttttccagTTTGCTGTTCCAGTTAAGAAACCAAGTATGCTTGTACTTTT
SRR516275.33120199.2+    tgtgtctttcttttccagTTTGCTGTTCCCGTTAAGAAACCAAGTATGCTTGTACTTTTA
SRR516275.71143232.1+    tgtgtctttcttttccagTTTGCTGTTCCAGTTAAGAAACCAAGTATGCTTGTACTTTTA
SRR516276.20232587.1-    tgtgtctttcttttccagTTTGCTGTTCCAGTTAAGAAACCAAGTATGCTTGTACTTTTA
SRR516276.20690846.1+    tgtgtctttcttttccagTTTGCTGTTCCAGTTAAGAAACCAAGTATGCTTGTACTTTTA
SRR516276.6403489.2-     tgtgtctttcttttccagTTTGCTGTTCCAGTTAAGAAACCAAGTATGCTTGTACTTTTA
SRR516276.66981451.2-    tgtgtctttcttttccagTTTGCTGTTCCAGTTAAGAAACCAAGTATGCTTGTACTTTTA
SRR516277.32099413.1-    tgtgtctttcttttccagTTTGCTGTTCCAGTTAAGAAACCAAGTATGCTTGTACTTTTA
SRR516277.38484509.1-    tgtgtctttcttttccagTTTGCTGTTCCAGTTAAGAAACCAAGTATGCTTGTACTTTTA
SRR516277.83350259.2+    tgtgtctttcttttccagTTTGCTGTTCCAGTTAAGAAACCAAGTATGCTTGTACTTTTA
SRR516278.25790579.2-    tgtgtctttcttttccagTTTGCTGTTCCAGTTAAGAAACCAAGTATGCTTGTACTTTTA
SRR516279.53275687.2-    tgtgtctttcttttccagTTTGCTGTTCCAGTTAAGAAACCAAGTATGCTTGTACTTTTA
SRR516279.63728261.2-    tgtgtctttcttttccagTTTGCTGTTCCAGTTAAGAAACCAAGTATGCTTGTACTTTTA
SRR516279.66465760.1-    tgtgtctttcttttccagTTTGCTGTTCCAGTTAAGAAACCAAGTATGCTTGTACTTTTA
SRR516279.78970894.1-    tgtgtctttcttttccagTTTGCTGTTCCAGTTAAGAAACCAAGTATGCTTGTACTTTTA
SRR516280.24131918.2-    tgtgtctttcttttccagTTTGCTGTTCCAGTTAAGAAACCAAGTATGCTTGTACTTTTA
SRR516280.24137326.2+    tgtgtctttcttttccagTTTGCTGTTCCAGTTAAGAAACCAAGTATGCTTGTACTTTTA
SRR516280.64560903.1+    tgtgtctttcttttccagTTTGCTGTTCCAGTTAAGAAACCAAGTATGCTTGTACTTTTA
SRR516272.31926998.1+       gtctttcttttccagTTTGCTGTTCCAGTTAAGAAACCAAGTATGCTTG
SRR516271.56413808.2-        tctttcttttccagTTTGCTGTTCCAGTTAAGAAACCAAGTATGCTTGT
SRR516271.17809087.2+         ctttcttttccagTTTGCTGTTCCAGTTAAGAAACCAAGTATGCTTGTA
SRR516271.62803696.2+         ctttcttttccagTTTGCTGTTCCAGTTAAGAAACCAAGTATGCTTGTA
SRR516271.62803699.2+         ctttcttttccagTTTGCTGTTCCAGTTAAGAAACCAAGTATGCTTGTA
SRR516275.58094264.2+           ttcttttccagTTTGCTGTTCCAGTTAAGAAACCAAGTATGCTTGTACTTTTA
SRR516279.38161935.1+           ttcttttccagTTTGCTGTTCCAGTTAAGAAACCAAGTATGCTTGTACTTTTA
SRR516279.63770083.1-           ttcttttccagTTTGCTGTTCCAGTTAAGAAACCAAGTATGCTTGTACTTTTA
SRR516280.45814438.1-           ttcttttccagTTTGCTGTTCCAGTTAAGAAACCAAGTATGCTTGTACTTTTA
SRR516276.23832642.1+             cttttccagTTTGCTGTTCCAGTTAAGAAACCAAGTATGCTTGTACTTTTA
SRR516278.12489663.2+             cttttccagTTTGCTGTTCCAGTTAAGAAACCAAGTATGCTTGTACTTTTA
SRR516278.57787935.1-                     gTTTGCTGTTCCAGTTAAGAAACCAAGTATGCTTGTACTTTTA
SRR516279.17476392.1-                     gTTTGCTGTTCCAGTTAAGAAACCAAGTATGCTTGTACTTTTA
SRR516279.11087506.1+                       TTGCTGTTCCAGTTAAGAAACCAAGTATGCTTGTACTTTTA
SRR516277.60425397.2-                        TGCTGTTCCAGTTAAGAAACCAAGTATGCTTGTACTTTTA
SRR516279.39710289.1+                            GTTCCAGTTAAGAAACCAAGTATGCTTGTACTTTTA
SRR516270.12142505.2+                              TCCAGTTAAGAAACCAAGTATGCTTGTACTTTTA
SRR516270.39832789.1+                              TCCAGTTAAGAAACCAAGTATGCTTGTACTTTTA
SRR516275.53203846.1+                              TCCAGTTAAGAAACCAAGTATGGTTGTACTTTTA
SRR516275.5527572.1+                               TCCAGTTAAGAAACCAAGTATGCTTGTACTTTTA
SRR516282.17515890.1+                               CCAGTTAAGAAACCGAGTATGCTTGTACTTTTA
SRR516274.109686541.2+                                   TAAGAAACCAAGTATGCTTGTACTTTTA
SRR516272.10742188.1-                                              AGTATGCTTGTACTTTTA
SRR516270.80465942.1-                                                        TACTTTTA
SRR516274.18696867.2+                                                              TA
SRR516279.37218141.2+                                                              TA
                         ____________________________________________________________
consensus                tgtgtctttcttttccagTTTGCTGTTCCAGTTAAGAAACCAAGTATGCTTGTACTTTTA

                               .    :    .    :    .    :    .    :    .    :    .    :
SRR516278.25790579.2-    TCC
SRR516276.20232587.1-    TCCC
SRR516280.24131918.2-    TCCCCCTG
SRR516277.32099413.1-    TCCCCCTGC
SRR516280.64560903.1+    TCCCCCTGC
SRR516270.12142505.2+    TCCCCCTGCCCATGG
SRR516270.39832789.1+    TCCCCCTGCCCATGG
SRR516276.20690846.1+    TCCCCCTGCCCATGGCC
SRR516274.109686541.2+   TCCCCCTGCCCATGGCCAAGC
SRR516279.63728261.2-    TCCCCCTGCCCATGGCCAAGCA
SRR516279.78970894.1-    TCCCCCTGCCCATGGCCAAGCA
SRR516275.71143232.1+    TCCCCCTGCCCATGGCCAAGCAGAAA
```

```
SRR516276.66981451.2-    TCCCCCTGCCCATGGCCAAGCAGAAA
SRR516277.38484509.1-    TCCCCCTGCCCATGGCCAAGCAGAAA
SRR516280.24137326.2+    TCCCCCTGCCCATGGCCAAGCAGAAACACC
SRR516272.10742188.1-    TCCCCCTGCCCATGGCCAAGCAGAAACACCA
SRR516277.83350259.2+    TCCCCCTGCCCATGGCCAAGCAGAAACACCA
SRR516279.66465760.1-    TCCCCCTGCCCATGGCCAAGCAGAAACACCATGTCT
SRR516275.33120199.2+    TCCCCCTGCCCATGGCCAAGCAGAAACCCCATGTCTAC
SRR516279.53275687.2-    TCCCCCTGCCCATGGCCAAGCAGAAACACCATGTCTAC
SRR516276.6403489.2-     TCCCCCTGCCCATGGCCAAGCAGAAACACCATGTCTACA
SRR516270.80465942.1-    TCCCCCTGCCCATGGCCAAGCAGAAACACCATGTCTACAAG
SRR516274.18696867.2+    TCCCCCTGCCCATGGCCAAGCAGAAACACCATGTCTACAAGgtaaaa
SRR516275.58094264.2+    TCCCCCTGCCCATGGCCAAGCAGAAACACCATGTCTACAAGgtaaaa
SRR516279.38161935.1+    TCCCCCTGCCCATGGCCAAGCAGAAACACCATGTCTACAAGgtaaaa
SRR516279.63770083.1-    TCCCCCTGCCCATGGCCAAGCAGAAACACCATGTCTACAAGgtaaaa
SRR516280.45814438.1-    TCCCCCTGCCCATGGCCAAGCAGAAACACCATGTCTACAAGgtaaaa
SRR516276.23832642.1+    TCCCCCTGCCCATGGCCAAGCAGAAACACCATGTCTACAAGgtaaaatt
SRR516278.12489663.2+    TCCCCCTGCCCATGGCCAAGCAGAAACACCATGTCTACAAGgtaaaatt
SRR516278.57787935.1-    TCCCCCTGCCCATGGCCAAGCAGAAACACCATGTCTACAAGgtaaaattgcaggctg
SRR516279.17476392.1-    TCCCCCTGCCCATGGCCAAGCAGAAACACCATGTCTACAAGgtaaaattgcaggctg
SRR516279.11087506.1+    TCCCCCTGCCCATGGCCAAGCAGAAACACCATGTCTACAAGgtaaaattgcaggctgtc
SRR516275.53203846.1+    TCCCCCTGCCCATGGCCAAGCAGAAACGCCATGTCTACAAGgtaaaattgcaggctgtct
SRR516275.5527572.1+     TCCCCCTGCCCATGGCCAAGCAGAAACACCATGTCTACAAGgtaaaattgcaggctgtct
SRR516277.60425397.2-    TCCCCCTGCCCATGGCCAAGCAGAAACACCATGTCTACAAGgtaaaattgcaggctgtct
SRR516279.37218141.2+    TCCCCCTGCCCATGGCCAAGCAGAAACACCATGTCTACAAGgtaaaattgcaggctgtct
SRR516279.39710289.1+    TCCCCCTGCCCATGGCCAAGCAGAAACACCATGTCTACAAGgtaaaattgcaggctgtct
SRR516282.17515890.1+    TCCCCCTGCCCATGGCCAAGCAGAAACACCATGTCTACAAGgtaaaattgcaggctgtct

consensus                TCCCCCTGCCCATGGCCAAGCAGAAACACCATGTCTACAAGgtaaaattgcaggctgtct
```

N. Peregrine falcon (No. 15), exon 4, WGS data

- CAP3 alignment of WGS data spanning exon 4 (uppercase letters)

```
                              .    :    .    :    .    :    .    :    .    :    .    :
SRR516255.67101666.1+    gtctttcttttccagTTT
SRR516257.9097618.1-     gtctttcttttccagTTT
SRR516258.28041248.2-    gtctttcttttccagTTT
SRR516262.3518177.1-     gtctttcttttccagTTT
SRR516262.3518202.1-     gtctttcttttccagTTT
SRR516253.8124406.2+     gtctttcttttccagTTTGC
SRR516254.4490370.1+     gtctttcttttccagTTTGCT
SRR516255.65242061.1-    gtctttcttttccagTTTGCT
SRR516255.79208835.1+    gtctttcttttccagTTTGCTG
SRR516262.4923955.2-     gtctttcttttccagTTTGCTG
SRR516262.4923959.2-     gtctttcttttccagTTTGCTG
SRR516253.68324806.1-    gtctttcttttccagTTTGCTGTT
SRR516259.58094187.1-    gtctttcttttccagTTTGCTGTT
SRR516259.77523733.1-    gtctttcttttccagTTTGCTGTT
SRR516267.46992421.2-    gtctttcttttccagTTTGCTGTT
SRR516255.39372922.2-    gtttttcttttccagTTTGCTGTTC
SRR516255.68420886.2+    gtctttcttttccagTTTGCTGTTC
SRR516264.28556706.2-    gtctttcttttccagTTTGCTGTTCC
SRR516264.30488692.2-    gtctttcttttccagTTTGCTGTTCC
SRR516264.61584647.2-    gtctttcttttccagTTTGCTGTTCC
SRR516268.66108476.1-    gtctttcttttccagTTTGCTGTTCC
SRR516253.69206126.1-    gtctttcttttccagTTTGCTGTTCCA
SRR516253.80177734.1-    gtctttcttttccagTTTGCTGTTCCA
SRR516257.59601121.2+    gtctttcttttccagTTTGCTGTTCCA
SRR516262.39538147.2-    gtctttcttttccagTTTGCTGTTCCA
SRR516262.39538148.2-    gtctttcttttccagTTTGCTGTTCCA
SRR516255.69835771.2+    gtctttcttttccagTTTGCTGTTCCAG
SRR516263.21456844.2-    gtctttcttttccagTTTGCTGTTCCAGT
SRR516263.21456847.2-    gtctttcttttccagTTTGCTGTTCCAGT
SRR516253.8547748.1-     gtctttcttttccagTTTGCTGTTCCAGTTA
SRR516256.18788178.2-    gtctttcttttccagTTTGCTGTTCCAGTTA
SRR516256.18788182.2-    gtctttcttttccagTTTGCTGTTCCAGTTA
SRR516256.21930298.1-    gtctttcttttccagTTTGCTGTTCCAGTTA
SRR516258.19489566.2-    gtctttcttttccagTTTGCTGTTCCAGTTAA
SRR516266.43829109.2-    gtctttcttttccagTTTGCTGTTCCAGTTAA
SRR516254.78958164.2-    gtctttcttttccagTTTGCTGTTCCAGTTAAGAA
SRR516264.2441810.2-     gtctttcttttccagTTTGCTGTTCCAGTTAAGAAA
SRR516264.68144895.2-    gtctttcttttccagTTTGCTGTTCCAGTTAAGAAA
SRR516267.40266999.2+    gtctttcttttccagTTTGCTGTTCCAGTTAAGAAA
SRR516253.17244217.2-    gtctttcttttccagTTTGCTGTTCCAGTTAAGAAACC
SRR516257.75910207.1-    gtctttcttttccagTTTGCTGTTCCAGTTAAGAAACC
SRR516263.56739339.2+    gtctttcttttccagTTTGCTGTTCCAGTTAAGAAACC
SRR516264.73888500.1-    gtctttcttttccagTTTGCTGTTCCAGTTAAGAAACCA
SRR516264.75833814.1-    gtctttcttttccagTTTGCTGTTCCAGTTAAGAAACCA
SRR516254.48908071.1-    gtctttcttttccagTTTGCTGTTCCAGTTAAGAAACCAAG
SRR516266.35283310.2+    gtctttcttttccagTTTGCTGTTCCAGTTAAGAAACCAAG
SRR516254.223018.1-      gtctttcttttccagTTTGCTGTTCCAGTTAAGAAACCAAGT
SRR516260.16848947.2+    gtctttcttttccagTTTGCTGTTCCAGTTAAGAAACCAAGT
SRR516260.36101835.2+    gtctttcttttccagTTTGCTGTTCCAGTTAAGAAACCAAGT
SRR516260.46009159.1+    gtctttcttttccagTTTGCTGTTCCAGTTAAGAAACCAAGT
SRR516260.64033010.2+    gtctttcttttccagTTTGCTGTTCCAGTTAAGAAACCAAGT
SRR516266.32033793.1-    gtctttcttttccagTTTGCTGTTCCAGTTAAGAAACCAAGT
SRR516253.51133180.1-    gtctttcttttccagTTTGCTGTTCCAGTTAAGAAACCAAGTA
SRR516254.41602867.1+    gtctttcttttccagTTTGCTGTTCCAGTTAAGAAACCAAGTAT
SRR516258.68260524.1-    gtctttcttttccagTTTGCTGTTCCAGTTAAGAAACCAAGTAT
SRR516266.46821086.1-    gtctttcttttccagTTTGCTGTTCCAGTTAAGAAACCAAGTAT
SRR516254.16919233.2-    gtctttcttttccagTTTGCTGTTCCAGTTAAGAAACCAAGTATG
SRR516254.89325311.2-    gtctttcttttccagTTTGCTGTTCCAGTTAAGAAACCAAGTATG
SRR516255.19693586.2-    gtctttcttttccagTTTGCTGTTCCAGTTAAGAAACCAAGTATG
SRR516261.47610756.2+    gtctttcttttccagTTTGCTGTTCCAGTTAAGAAACCAAGTATG
SRR516268.51432009.1-    gtctttcttttccagTTTGCTGTTCCAGTTAAGAAACCAAGTATG
SRR516255.81054542.2+    gtctttcttttccagTTTGCTGTTCCAGTTAAGAAACCAAGTATGCTTGT
SRR516256.6485514.2+     gtctttcttttccagTTTGCTGTTCCAGTTAAGAAACCAAGTATGCTTGT
SRR516254.4135028.1-     gtctttcttttccagTTTGCTGTTCCAGTTAAGAAACCAAGTATGCTTGTA
SRR516263.4401630.1+     gtctttcttttccagTTTGCTGTTCCAGTTAAGAAACCAAGTATGCTTGTAC
SRR516257.52841837.1+    gtctttcttttccagTTTGCTGTTCCAGTTAAGAAACCAAGTATGCTTGTACT
SRR516257.80856403.2-    gtctttcttttccagTTTGCTGTTCCAGTTAAGAAACCAAGTATGCTTGTACT
SRR516253.49535897.2-    gtctttcttttccagTTTGCTGTTCCAGTTAAGAAACCAAGTATGCTTGTACTT
SRR516255.28436949.2-    gtctttcttttccagTTTGCTGTTCCAGTTAAGAAACCAAGTATGCTTGTACTT
SRR516254.59112408.1+    gtctttcttttccagTTTGCTGTTCCAGTTAAGAAACCAAGTATGCTTGTACTTT
SRR516262.26241561.2-    gtctttcttttccagTTTGCTGTTCCAGTTAAGAAACCAAGTATGCTTGTACTTT
SRR516263.41020644.1-    gtctttcttttccagTTTGCTGTTCCAGTTAAGAAACCAAGTATGCTTGTACTTTT
SRR516255.64448961.1+    gtctttcttttccagTTTGCTGTTCCAGTTAAGAAACCAAGTATGCTTGTACTTTTAT
SRR516255.31773466.1+    gtctttcttttccagTTTGCTGTTCCAGTTAAGAAACCAAGTATGCTTGTACTTTTATC
SRR516253.365320.2+      gtctttcttttccagTTTGCTGTTCCAGTTAAGAAACCAAGTATGCTTGTACTTTTATCC
```

```
SRR516253.5211449.1+     gtctttcttttccagTTTGCTGTTCCAGTTAAGAAACCAAGTATGCTTGTACTTTTATCC
SRR516253.64655527.1+    gtctttcttttccagTTTGCTGTTCCAGTTAAGAAACCAAGTATGCTTGTACTTTTATCC
SRR516253.67066187.2+    gtctttcttttccagTTTGCTGTTCCAGTTAAGAAACCAAGTATGCTTGTACTTTTATCC
SRR516253.80551265.2+    gtctttcttttccagTTTGCTGTTCCAGTTAAGAAACCAAGTATGCTTGTACTTTTATCC
SRR516254.10680655.2-    gtctttcttttccagTTTGCTGTTCCAGTTAAGAAACCAAGTATGCTTGTACTTTTATCC
SRR516254.56196602.1+    gtctttcttttccagTTTGCTGTTCCAGTTAAGAAACCAAGTATGCTTGTACTTTTATCC
SRR516254.56749609.1-    gtctttcttttccagTTTGCTGTTCCAGTTAAGAAACCAAGTATGCTTGTACTTTTATCC
SRR516254.62410953.2+    gtctttcttttccagTTTGCTGTTCCAGTTAAGAAACCAAGTATGCTTGTACTTTTATCC
SRR516254.79124957.2+    gtctttcttttccagTTTGCTGTTCCAGTTAAGAAACCAAGTATGCTTGTACTTTTATCC
SRR516254.86674766.2+    gtctttcttttccagTTTGCTGTTCCAGTTAAGAAACCAAGTATGCTTGTACTTTTATCC
SRR516255.12845150.2-    gtctttcttttccagTTTGCTGTTCCAGTTAAGAAACCAAGTATGCTTGTACTTTTATCC
SRR516255.1697326.1+     gtctttcttttccagTTTGCTGTTCCAGTTAAGAAACCAAGTATGCTTGTACTTTTATCC
SRR516255.48156284.2+    gtctttcttttccagTTTGCTGTTCCAGTTAAGAAACCAAGTATGCTTGTACTTTTATCC
SRR516255.65504789.1-    gtctttcttttccagTTTGCTGTTCCAGTTAAGAAACCAAGTATGCTTGTACTTTTATCC
SRR516255.67101666.2-    gtctttcttttccagTTTGCTGTTCCAGTTAAGAAACCAAGTATGCTTGTACTTTTATCC
SRR516255.70481761.2+    gtctttcttttccagTTTGCTGTTCCAGTTAAGAAACCAAGTATGCTTGTACTTTTATCC
SRR516255.70485066.1+    gtctttcttttccagTTTGCTGTTCCAGTTAAGAAACCAAGTATGCTTGTACTTTTATCC
SRR516256.1347814.2-     gtctttcttttccagTTTGCTGTTCCAGTTAAGAAACCAAGTATGCTTGTACTTTTATCC
SRR516256.18761086.1-    gtctttcttttccagTTTGCTGTTCCAGTTAAGAAACCAAGTATGCTTGTACTTTTATCC
SRR516256.5907042.1+     gtctttcttttccagTTTGCTGTTCCAGTTAAGAAACCAAGTATGCTTGTACTTTTATCC
SRR516257.26973823.1+    gtctttcttttccagTTTGCTGTTCCAGTTAAGAAACCAAGTATGCTTGTACTTTTATCC
SRR516257.66685789.1+    gtctttcttttccagTTTGCTGTTCCAGTTAAGAAACCAAGTATGCTTGTACTTTTATCC
SRR516257.7312118.2+     gtctttcttttccagTTTGCTGTTCCAGTTAAGAAACCAAGTATGCTTGTACTTTTATCC
SRR516258.24981535.2+    gtctttcttttccagTTTGCTGTTCCAGTTAAGAAACCAAGTATGCTTGTACTTTTATCC
SRR516258.25211817.1+    gtctttcttttccagTTTGCTGTTCCAGTTAAGAAACCAAGTATGCTTGTACTTTTATCC
SRR516258.38515055.2+    gtctttcttttccagTTTGCTGTTCCAGTTAAGAAACCAAGTATGCTTGTACTTTTATCC
SRR516258.45585051.1+    gtctttcttttccagTTTGCTGTTCCAGTTAAGAAACCAAGTATGCTTGTACTTTTATCC
SRR516258.54363655.1-    gtctttcttttccagTTTGCTGTTCCAGTTAAGAAACCAAGTATGCTTGTACTTTTATCC
SRR516258.67589391.2+    gtctttcttttccagTTTGCTGTTCCAGTTAAGAAACCAAGTATGCTTGTACTTTTATCC
SRR516261.14549337.2+    gtctttcttttccagTTTGCTGTTCCAGTTAAGAAACCAAGTATGCTTGTACTTTTATCC
SRR516262.12338739.2+    gtctttcttttccagTTTGCTGTTCCAGTTAAGAAACCAAGTATGCTTGTACTTTTATCC
SRR516262.12338742.2+    gtctttcttttccagTTTGCTGTTCCAGTTAAGAAACCAAGTATGCTTGTACTTTTATCC
SRR516263.16241967.2+    gtctttcttttccagTTTGCTGTTCCAGTTAAGAAACCAAGTATGCTTGTACTTTTATCC
SRR516263.17118136.1-    gtctttcttttccagTTTGCTGTTCCAGTTAAGAAACCAAGTATGCTTGTACTTTTATCC
SRR516263.49448208.2-    gtctttcttttccagTTTGCTGTTCCAGTTAAGAAACCAAGTATGCTTGTACTTTTATCC
SRR516263.6095503.2-     gtctttcttttccagTTTGCTGTTCCAGTTAAGAAACCAAGTATGCTTGTACTTTTATCC
SRR516263.65854490.1-    gtctttcttttccagTTTGCTGTTCCAGTTAAGAAACCAAGTATGCTTGTACTTTTATCC
SRR516263.75018518.2-    gtctttcttttccagTTTGCTGTTCCAGTTAAGAAACCAAGTATGCTTGTACTTTTATCC
SRR516263.76557724.1+    gtctttc-tttccagTTTGCTGTTCCAGTTAAGAAACCAAGTATGCTTGTACTTTTATCC
SRR516263.76557725.1+    gtctttc-tttccagTTTGCTGTTCCAGTTAAGAAACCAAGTATGCTTGTACTTTTATCC
SRR516253.8124406.1-       ctttcttttccagTTTGCTGTTCCAGTTAAGAAACCAAGTATGCTTGTACTTTTATCC
SRR516254.4490370.2-       ctttcttttccagTTTGCTGTTCCAGTTAAGAAACCAAGTATGCTTGTACTTTTATCC
SRR516255.69835771.1-      ctttcttttccagTTTGCTGTTCCAGTTAAGAAACCAAGTATGCTTGTACTTTTATCC
SRR516257.27584421.1+      ctttcttttccagTTTGCTGTTCCAGTTAAGAAACCAAGTATGCTTGTACTTTTATCC
SRR516254.18031680.2+       tttcttttccagTTTGCTGTTCCAGTTAAGAAACCAAGTATGCTTGTACTTTTATCC
SRR516255.68420886.1-       tttcttttccagTTTGCTGTTCCAGTTAAGAAACCAAGTATGCTTGTACTTTTATCC
SRR516261.82860556.1+       tttcttttccagTTTGCTGTTCCAGTTAAGAAACCAAGTATGCTTGTACTTTTATCC
SRR516255.79208835.2-        ttcttttccagTTTGCTGTTCCAGTTAAGAAACCAAGTATGCTTGTACTTTTATCC
SRR516268.44090166.1+          cttttccagTTTGCTGTTCCAGTTAAGAAACCAAGTATGCTTGTACTTT
SRR516253.84549442.2+           ttttccagTTTGCTGTTCCAGTTAAGAAACCAAGTATGCTTGTACTTTTATCC
SRR516256.24075489.2-           ttttccagTTTGCTTGTCCAGTTAAGAAACCAAGTATGCTTGTACTTTTATCC
SRR516262.21781133.1+                cagTTTGCTGTTCCAGTTAAGAAACCAAGTATGCTTGTACTTTTATCC
SRR516255.31500806.1+                 agTTTGCTGTTCCAGTTAAGAAACCAAGTATGCTTGTACTTTTATCC
SRR516268.9704517.1+                   gTTTGCTGTTCCAGTTAAGAAACCAAGTATGCTTGTACTTTTATCC
SRR516254.34872139.1+                   TTTGCTGTTCCAGTTAAGAAACCAAGTATGCTTGTACTTTTATCC
SRR516255.25565504.2+                    TTGCTGTTCCAGTTAAGAAACCAAGTATGCTTGTACTTTTATCC
SRR516259.72895312.2+                     TGCTGTTCCAGTTAAGAAACCAAGTATGCTTGTACTTTTATCC
SRR516259.8678999.2+                      TGCTGTTCCAGTTAAGAAACCAAGTATGCTTGTACTTTTATCC
SRR516267.7046461.2+                      TGCTGTTCCAGTTAAGAAACCAAGTATGCTTGTACTTTTATCC
SRR516267.7046465.2+                      TGCTGTTCCAGTTAAGAAACCAAGTATGCTTGTACTTTTATCC
SRR516267.9720099.1+                      TGCTGTTCCAGTTAAGAAACCAAGTATGCTTGTACTTTTATCC
SRR516267.9720105.1+                      TGCTGTTCCAGTTAAGAAACCAAGTATGCTTGTACTTTTATCC
SRR516256.10718488.1-                      GCTGTTCCAGTTAAGAAACCAAGTATGCTTGTACTTTTATCC
SRR516258.18487741.1-                      GCTGTTCCAGTTAAGAAACCAAGTATGCTTGTACTTTTATCC
SRR516255.3305445.1+                        CTGTTCCAGTTAAGAAACCAAGTATGCTTGTACTTTTATCC
SRR516258.75491064.2-                       CTGTTCCAGTTAAGAAACCAAGTATGCTTGTACTTTTATCC
SRR516258.75491067.2-                       CTGTTCCAGTTAAGAAACCAAGTATGCTTGTACTTTTATCC
SRR516267.15315750.2-                       CTGTTCCAGTTAAGAAACCAAGTATGCTTGTACTTTTATCC
SRR516254.59112408.2-                         GTTCCAGTTAAGAAACCAAGTATGCTTGTACTTTTATCC
SRR516267.64842649.1+                         GTTCCAGTTAAGAAACCAAGTATGCTTGTACTTTTATCC
SRR516268.30284567.2-                            CCAGTTAAGAAACCAAGTATGCTTGTACTTTTATCC
SRR516268.40554607.2+                            CCAGTTAAGAAACCAAGTATGCTTGTACTTTTATCC
SRR516268.40554611.2+                            CCAGTTAAGAAACCAAGTATGCTTGTACTTTTATCC
SRR516254.41602867.2-                             CAGTTAAGAAACCAAGTATGCTTGTACTTTTATCC
SRR516260.26188594.1+                             CAGTTAAGAAACCAAGTATGCTTGTACTTTTATCC
SRR516266.14130943.1-                              AGTTAAGAAACCAAGTATGCTTGTACTTTTATCC
SRR516267.60638311.1+                              AGTTAAGAAACCAAGTATGCTTGTACTTTTATCC
SRR516253.19465821.1+                               GTTAAGAAACCAAGTATGCTTGTACTTTTATCC
SRR516254.54211738.2+                                 TAAGAAACCAAGTATGCTTGTACTTTTATCC
SRR516256.18160700.1+                                 TAAGAAACCAAGTATGCTTGTACTTTTATCC
SRR516255.31773466.2-                                   AGAAACCAAGTATGCTTGTACTTTTATCC
SRR516255.64448961.2-                                   AGAAACCAAGTATGCTTGTACTTTTATCC
SRR516259.18755021.1-                                   AGAAACCAAGTATGCTTGTACTTTTATCC
SRR516253.72746215.2+                                    GAAACCAAGTATGCTTGTACTTTTATCC
```

```
SRR516255.81054542.1-                                     AAACCAAGTATGCTTGTACTTTTATCC
SRR516264.19787823.2+                                      AACCAAGTATGCTTGTACTTTTATCC
SRR516264.32999117.2+                                      AACCAAGTATGCTTGTACTTTTATCC
SRR516264.47987043.2+                                      AACCAAGTATGCTTGTACTTTTATCC
SRR516264.53208831.2+                                      AACCAAGTATGCTTGTACTTTTATCC
SRR516264.76289025.2+                                      AACCAAGTATGCTTGTACTTTTATCC
SRR516266.189811.1+                                        AACCAAGTATGCTTGTACTTTTATCC
SRR516266.32039919.1+                                      AACCAAGTATGCTTGTACTTTTATCC
SRR516259.25233688.1-                                       ACCAAGTATGCTTGTACTTTTATCC
SRR516267.6877951.1-                                          CAAGTATGCTTGTACTTTTATCC
SRR516257.51968353.1-                                          AAGTATGCTTGTACTTTTATCC
SRR516257.51968354.1-                                          AAGTATGCTTGTACTTTTATCC
SRR516258.43616193.2+                                          AAGTATGCTTGTACTTTTATCC
SRR516258.43616196.2+                                          AAGTATGCTTGTACTTTTATCC
SRR516264.11312838.1+                                           AGTATGCTTGTACTTTTATCC
SRR516253.80551265.1-                                            GTATGCTTGTACTTTTATCC
SRR516257.63892613.1-                                            GTATGCTTGTACTTTTATCC
SRR516255.21474988.1+                                             TATGCTTGTACTTTTATCC
SRR516258.20261841.2+                                             TATGCTTGTACTTTTATCC
SRR516267.33129504.1+                                              ATGCTTGTACTTTTATCC
SRR516258.59882969.1-                                               TGCTTGTACTTTTATCC
SRR516263.16702536.2-                                                GCTTGCACTTTTATCC
SRR516254.56196602.2-                                                 CTTGTACTTTTATCC
SRR516267.26400091.2-                                                 CTTGTACTTTTATCC
SRR516253.57322287.2+                                                  TTGTACTTTTATCC
SRR516253.64655527.2-                                                  TTGTACTTTTACCC
SRR516254.52808494.1+                                                  TTGTACTTTTATCC
SRR516258.44928883.2-                                                   TGTACTTTTATCC
SRR516254.86674766.1-                                                     TACTTTTATCC
SRR516260.4640154.1+                                                        CTTTTATCC
SRR516256.29806347.2+                                                         TTTATCC
SRR516253.57040134.2+                                                           TATCC
SRR516261.64488870.1+                                                           TATCC
SRR516261.64488872.1+                                                           TATCC
SRR516264.31833336.1+                                                           TATCC
SRR516264.45706920.1+                                                           TATCC
SRR516264.45706923.1+                                                           TATCC
SRR516264.60940391.2+                                                           TATCC
SRR516253.5211449.2-                                                             ATCC
SRR516253.67066187.1-                                                            ATCC
SRR516258.39846263.1+                                                            ATCC
SRR516258.39846269.1+                                                            ATCC
SRR516257.20295656.1+                                                             TCC
SRR516258.56007600.1-                                                             TCC
SRR516262.2173080.1-                                                               CC
SRR516268.48113730.2+                                                              CC
SRR516253.365320.1-                                                                 C
SRR516257.25331442.1+                                                               C
SRR516258.36659311.1+                                                               C

consensus                gtctttcttttccagTTTGCTGTTCCAGTTAAGAAACCAAGTATGCTTGTACTTTTATCC

                             .    :    .    :    .    :    .    :    .    :    .    :
SRR516254.56196602.1+    C
SRR516254.56749609.1-    C
SRR516255.12845150.2-    CC
SRR516255.65504789.1-    CC
SRR516268.9704517.1+     CCC
SRR516253.64655527.1+    CCCT
SRR516259.72895312.2+    CCCTGC
SRR516259.8678999.2+     CCCTGC
SRR516267.7046461.2+     CCCTGC
SRR516267.7046465.2+     CCCTGC
SRR516267.9720099.1+     CCCTGC
SRR516267.9720105.1+     CCCTGC
SRR516258.24981535.2+    CCCTGCCC
SRR516258.38515055.2+    CCCTGCCC
SRR516258.45585051.1+    CCCTGCCC
SRR516263.17118136.1-    CCCTGCCC
SRR516267.15315750.2-    CCCTGCCC
SRR516257.26973823.1+    CCCTGCCCAT
SRR516267.64842649.1+    CCCTGCCCAT
SRR516253.80551265.2+    CCCTGCCCATG
SRR516253.365320.2+      CCCTGCCCATGGC
SRR516262.12338739.2+    CCCTGCCCATGGC
SRR516262.12338742.2+    CCCTGCCCATGGC
SRR516268.30284567.2-    CCCTGCCCATGGC
SRR516268.40554607.2+    CCCTGCCCATGGC
SRR516268.40554611.2+    CCCTGCCCATGGC
SRR516256.18761086.1-    CCCTGCCCATGGCC
SRR516260.26188594.1+    CCCTGCCCATGGCC
SRR516254.86674766.2+    CCCTGCCCATGGCCA
SRR516257.7312118.2+     CCCTGCCCATGGCCA
SRR516266.14130943.1-    CCCTGCCCATGGCCA
```

SRR516267.60638311.1+ CCCTGCCCATGGCCA
SRR516263.76557724.1+ CCCTGCCCATGGCCAA
SRR516263.76557725.1+ CCCTGCCCATGGCCAA
SRR516256.1347814.2- CCCTGCCCATGGCCAAG
SRR516263.49448208.2- CCCTGCCCATGGCCAAG
SRR516261.14549337.2+ CCCTGCCCATGGCCAAGC
SRR516255.67101666.2- CCCTGCCCATGGCCAAGCAG
SRR516259.18755021.1- CCCTGCCCATGGCCAAGCAG
SRR516253.67066187.2+ CCCTGCCCATGGCCAAGCAGAAA
SRR516264.19787823.2+ CCCTGCCCATGGCCAAGCAGAAA
SRR516264.32999117.2+ CCCTGCCCATGGCCAAGCAGAAA
SRR516264.47987043.2+ CCCTGCCCATGGCCAAGCAGAAA
SRR516264.53208831.2+ CCCTGCCCATGGCCAAGCAGAAA
SRR516264.76289025.2+ CCCTGCCCATGGCCAAGCAGAAA
SRR516266.189811.1+ CCCTGCCCATGGCCAAGCAGAAA
SRR516266.32039919.1+ CCCTGCCCATGGCCAAGCAGAAA
SRR516259.25233688.1- CCCTGCCCATGGCCAAGCAGAAAC
SRR516253.5211449.1+ CCCTGCCCATGGCCAAGCAGAAACAC
SRR516267.6877951.1- CCCTGCCCATGGCCAAGCAGAAACAC
SRR516264.11312838.1+ CCCTGCCCATGGCCAAGCAGAAACACCA
SRR516258.54363655.1- CCCTGCCCATGGCCAAGCAGAAACACCATG
SRR516263.65854490.1- CCCTGCCCATGGCCAAGCAGAAACACCATG
SRR516257.66685789.1+ CCCTGCCCATGGCCAAGCAGCAACACCATGT
SRR516258.25211817.1+ CCCTGCCCATGGCCAAGCAGAAACACCATGT
SRR516267.33129504.1+ CCCTGCCCATGGCCAAGCAGAAACACCATGT
SRR516255.70485066.1+ CCCTGCCCATGGCCAAGCAGAAACACCATGTCT
SRR516258.67589391.2+ CCCTGCCCATGGCCAAGCAGAAACACCATGTCT
SRR516263.16241967.2+ CCCTGCCCATGGCCAAGCAGAAACACCATGTCT
SRR516263.6095503.2- CCCTGCCCATGGCCAAGCAGAAACACCATGTCT
SRR516255.1697326.1+ CCCTGCCCATGGCCAAGCAGAAACACCATGTCTA
SRR516263.75018518.2- CCCTGCCCATGGCCAAGCAGAAACACCATGTCTA
SRR516267.26400091.2- CCCTGCCCATGGCCAAGCAGAAACACCATGTCTA
SRR516254.62410953.2+ CCCTGCCCATGGCCAAGCAGAAACACCATGTCTACA
SRR516254.79124957.2+ CCCTGCCCATGGCCAAGCAGAAACACCATGTCTACA
SRR516254.10680655.2- CCCTGCCCATGGCCAAGCAGAAACACCATGTCTACAA
SRR516256.5907042.1+ CCCTGCCCATGGCCAAGCAGAAACACCATGTCTACAA
SRR516255.48156284.2+ CCCTGCCCATGGCCAAGCAGAAACACCATGTCTACAAGg
SRR516255.70481761.2+ CCCTGCCCATGGCCAAGCAGAAACACCATGTCTACAAGg
SRR516260.4640154.1+ CCCTGCCCATGGCCAAGCAGAAACACCATGTCTACAAGgt
SRR516253.8124406.1- CCCTGCCCATGGCCAAGCAGAAACACCATGTCTACAAGgtaa
SRR516254.4490370.2- CCCTGCCCATGGCCAAGCAGAAACACCATGTCTACAAGgtaa
SRR516255.69835771.1- CCCTGCCCATGGCCAAGCAGAAACACCATGTCTACAAGgtaa
SRR516257.27584421.1+ CCCTGCCCATGGCCAAGCAGAAACACCATGTCTACAAGgtaa
SRR516254.18031680.2+ CCCTGCCCATGGCCAAGCAGAAACACCATGTCTACAAGgtaaa
SRR516255.68420886.1- CCCTGCCCATGGCCAAGCAGAAACACCATGTCTACAAGgtaaa
SRR516261.82860556.1+ CCCTGCCCATGGCCAAGCAGAAACACCATGTCTACAAGgtaaa
SRR516255.79208835.2- CCCTGCCCATGGCCAAGCAGAAACACCATGTCTACAAGgtaaaa
SRR516264.31833336.1+ CCCTGCCCATGGCCAAGCAGAAACACCATGTCTACAAGgtaaaa
SRR516264.45706920.1+ CCCTGCCCATGGCCAAGCAGAAACACCATGTCTACAAGgtaaaa
SRR516264.45706923.1+ CCCTGCCCATGGCCAAGCAGAAACACCATGTCTACAAGgtaaaa
SRR516264.60940391.2+ CCCTGCCCATGGCCAAGCAGAAACACCATGTCTACAAGgtaaaa
SRR516253.84549442.2+ CCCTGCCCATGGCCAAGCAGAAACACCATGTCTACAAGgtaaaattg
SRR516256.24075489.2- CCCTGCCCATGGCCAAGCAGAAACACCATGTCTACAAGgtaaaattg
SRR516268.48113730.2+ CCCTGCCCATGGCCAAGCAGAAACACCATGTCTACAAGgtaaaattg
SRR516262.21781133.1+ CCCTGCCCATGGCCAAGCAGAAACACCATGTCTACAAGgtaaaattgcagtc
SRR516255.31500806.1+ CCCTGCCCATGGCCAAGCAGAAACACCATGTCTACAAGgtaaaattgcagtct
SRR516254.34872139.1+ CCCTGCCCATGGCCAAGCAGAAACACCATGTCTACAAGgtaaaattgcagtctgt
SRR516255.25565504.2+ CCCTGCCCATGGCCAAGCAGAAACACCATGTCTACAAGgtaaaattgcagtctgtc
SRR516256.10718488.1- CCCTGCCCATGGCCAAGCAGAAACACCATGTCTACAAGgtaaaattgcagtctgtctt
SRR516258.18487741.1- CCCTGCCCATGGCCAAGCAGAAACACCATGTCTACAAGgtaaaattgcagtctgtctt
SRR516255.3305445.1+ CCCTGCCCATGGCCAAGCAGAAACACCATGTCTACAAGgtaaaattgcagtctgtctta
SRR516258.75491064.2- CCCTGCCCATGGCCAAGCAGAAACACCATGTCTACAAGgtaaaattgcagtctgtctta
SRR516258.75491067.2- CCCTGCCCATGGCCAAGCAGAAACACCATGTCTACAAGgtaaaattgcagtctgtctta
SRR516253.19465821.1+ CCCTGCCCATGGCCAAGCAGAAACACCATGTCTACAAGgtaaaattgcagtctgtcttac
SRR516253.365320.1- CCCTGCCCATGGCCAAGCAGAAACACCATGTCTACAAGgtaaaattgcagtctgtcttac
SRR516253.5211449.2- CCCTGCCCATGGCCAAGCAGAAACACCATGTCTACAAGgtaaaattgcagtctgtcttac
SRR516253.57040134.2+ CCCTGCCCATGGCCAAGCAGAAACACCATGTCTACAAGgtaaaattgcagtctgtcttac
SRR516253.57322287.2+ CCCTGCCCATGGCCAAGCAGAAACACCATGTCTACAAGgtaaaattgcagtctgtcttac
SRR516253.64655527.2- CCCTGCCCATGGCCAAGCAGAAACACCATGTCTACAAGgtaaaattgcagtctgtcttac
SRR516253.67066187.1- CCCTGCCCATGGCCAAGCAGAAACACCATGTCTACAAGgtaaaattgcagtctgtcttac
SRR516253.72746215.2+ CCCTGCCCATGGCCAAGCAGAAACACCATGTCTACAAGgtaaaattgcagtctgtcttac
SRR516253.80551265.1- CCCTGCCCATGGCCAAGCAGAAACACCATGTCTACAAGgtaaaattgcagtctgtcttac
SRR516254.41602867.2- CCCTGCCCATGGCCAAGCAGAAACACCATGTCTACAAGgtaaaattgcagtctgtcttac
SRR516254.52808494.1+ CCCTGCCCATGGCCAAGCAGAAACACCATGTCTACAAGgtaaaattgcagtctgtcttac
SRR516254.54211738.2+ CCCTGCCCATGGCCAAGCAGAAACACCATGTCTACAAGgtaaaattgcagtctgtcttac
SRR516254.56196602.2- CCCTGCCCATGGCCAAGCAGAAACACCATGTCTACAAGgtaaaattgcagtctgtcttac
SRR516254.59112408.2- CCCTGCCCATGGCCAAGCAGAAACACCATGTCTACAAGgtaaaattgcagtctgtcttac
SRR516254.86674766.1- CCCTGCCCATGGCCAAGCAGAAACACCATGTCTACAAGgtaaaattgcagtctgtcttac
SRR516255.21474988.1+ CCCTGCCCATGGCCAAGCAGAAACACCATGTCTACAAGgtaaaattgcagtctgtcttac
SRR516255.31773466.2- CCCTGCCCATGGCCAAGCAGAAACACCATGTCTACAAGgtaaaattgcagtctgtcttac
SRR516255.64448961.2- CCCTGCCCATGGCCAAGCAGAAACACCATGTCTACAAGgtaaaattgcagtctgtcttac
SRR516255.81054542.1- CCCTGCCCATGGCCAAGCAGAAACACCATGTCTACAAGgtaaaattgcagtctgtcttac
SRR516256.18160700.1+ CCCTGCCCATGGCCAAGCAGAAACACCATGTCTACAAGgtaaaattgcagtctgtcttac
SRR516256.29806347.2+ CCCTGCCCATGGCCAAGCAGAAACACCATGTCTACAAGgtaaaattgcagtctgtcttac

```
SRR516257.20295656.1+    CCCTGCCCATGGCCAAGCAGAAACACCATGTCTACAAGgtaaaattgcagtctgtcttac
SRR516257.25331442.1+    CCCTGCCCATGGCCAAGCAGAAACACCATGTCTACAAGgtaaaattgcagtctgtcttac
SRR516257.51968353.1-    CCCTGCCCATGGCCAAGCAGAAACACCATGTCTACAAGgtaaaattgcagtctgtcttac
SRR516257.51968354.1-    CCCTGCCCATGGCCAAGCAGAAACACCATGTCTACAAGgtaaaattgcagtctgtcttac
SRR516257.63892613.1-    CCCTGCCCATGGCCAAGCAGAAACACCATGTCTACAAGgtaaaattgcagtctgtcttac
SRR516258.20261841.2+    CCCTGCCCATGGCCAAGCAGAAACACCATGTCTACAAGgtaaaattgcagtctgtcttac
SRR516258.36659311.1+    CCCTGCCCATGGCCAAGCAGAAACACCATGTCTACAAGgtaaaattgcagtctgtcttac
SRR516258.39846263.1+    CCCTGCCCATGGCCAAGCAGAAACACCATGTCTACAAGgtaaaattgcagtctgtcttac
SRR516258.39846269.1+    CCCTGCCCATGGCCAAGCAGAAACACCATGTCTACAAGgtaaaattgcagtctgtcttac
SRR516258.43616193.2+    CCCTGCCCATGGCCAAGCAGAAACACCATGTCTACAAGgtaaaattgcagtctgtcttac
SRR516258.43616196.2+    CCCTGCCCATGGCCAAGCAGAAACACCATGTCTACAAGgtaaaattgcagtctgtcttac
SRR516258.44928883.2-    CCCTGCCCATGGCCAAGCAGAAACACCATGTCTACAAGgtaaaattgcagtctgtcttac
SRR516258.56007600.1-    CCCTGCCCATGGCCAAGCAGAAACACCATGTCTACAAGgtaaaattgcagtctgtcttac
SRR516258.59882969.1-    CCCTGCCCATGGCCAAGCAGAAACACCATGTCTACAAGgtaaaattgcagtctgtcttac
SRR516261.64488870.1+    CCCTGCCCATGGCCAAGCAGAAACACCATGTCTACAAGgtaaaattgcagtctgtcttac
SRR516261.64488872.1+    CCCTGCCCATGGCCAAGCAGAAACACCATGTCTACAAGgtaaaattgcagtctgtcttac
SRR516262.2173080.1-     CCCTGCCCATGGCCAAGCAGAAACACCATGTCTACAAGgtaaaattgcagtctgtcttac
SRR516263.16702536.2-    CCCTGCCCATGGTCAAGCAGAATCACCATGTCTACAAGgtaaaattgctgtctttcttac
SRR516257.77010811.1-     CCTGCCCATGGCCAAGCAGAAACACCATGTCTACAAGgtaaaattgcagtctgtcttac
SRR516257.48172373.2+      CTGCCCATGGCCAAGCAGAAACACCATGTCTACAAGgtaaaattgcagtctgtcttac
SRR516258.34238218.1+      CTGCCCATGGCCAAGCAGAAACACCATGTCTACAAGgtaaaattgcagtctgtcttac
SRR516258.51540927.2-      CTGCCTATGGCCAAGCAGAAACACCATATCTACAAGgtaaaattgcagtctgtcttcc
SRR516253.4239929.1+        TGCCCATGGCCAAGCAGAAACACCATGTCTACAAGgtaaaattgcagtctgtcttac
SRR516254.79124957.1-         CCCATGGCCAAGCAGAAACACCATGTCTACAAGgtaaaattgcagtctgtcttac
SRR516258.27992696.2+          CCATGGCCAAGCAGAAACACCATGTCTACAAGgtaaaattgcagtctgtcttac
SRR516261.77778183.1+          CCATGGCCAAGCAGAAACACCATGTCTACAAGgtaaaattgcagtctgtcttac
SRR516261.77778184.1+          CCATGGCCAAGCAGAAACACCATGTCTACAAGgtaaaattgcagtctgtcttac
SRR516261.77778186.1+          CCATGGCCAAGCAGAAACACCATGTCTACAAGgtaaaattgcagtctgtcttac
SRR516259.7378581.2+            CATGGCCAAGCAGAAACACCATGTCTACAAGgtaaaattgcagtctgtc
SRR516257.17384372.1-           CATGGCCAAGCAGAAACACCATGTCTACAAGgtaaaattgcagtctgtcttac
SRR516264.62082534.2+              GGCCAAGCAGAAACACCATGTCTACAAGgtaaaattgcagtctgtctta
SRR516264.69513869.2+              GGCCAAGCAGAAACACCATGTCTACAAGgtaaaattgcagtctgtctta
SRR516255.70485066.2-              GGCCAAGCAGAAACACCATGTCTACAAGgtaaaattgcagtctgtcttac
SRR516255.1697326.2-                 CCAAGCAGAAACACCATGTCTACAAGgtaaaattgcagtctgtcttac
SRR516258.15077561.2-                 CAAGCAGAAACACCATGTCTACAAGgtaaaattgcagtctgtcttac
SRR516263.50529282.2-                 CAAGCAGAAACACCATGTCTACAAGgtaaaattgcagtctgtcttac
SRR516263.50529284.2-                 CAAGCAGAAACACCATGTCTACAAGgtaaaattgcagtctgtcttac
SRR516257.1728763.2-                   AAGCAGAAACCCCATGTCTACAAGgtaaaattgcagtctgtcttac
SRR516257.17888695.1+                  AAGCAGAAACACCATGTCTACAAGgtaaaattgcagtctgtcttac
SRR516258.16266909.1-                  AAGCAGAAACACCATGTCTACAAGgtaaaattgcagtctgtcttac
SRR516257.14316028.2-                   AGCAGAAACACCATGTCTACAAGgtaaaattgcagtctgtcttac
SRR516259.52283762.1-                   AGCAGAAACACCATGTCTACAAGgtaaaattgcagtctgtcttac
SRR516258.26021878.2-                    GCAGAAACACCATGTCTACAAGgtaaaattgcagtctgtcttac
SRR516268.28649267.2+                    GCAGAAACACCATGTCTACAAGgtaaaattgcagtctgtcttac
SRR516254.62410953.1-                     CAGAAACACCATGTCTACAAGgtaaaattgcagtctgtcttac
SRR516262.15483265.1-                     CAGAAACACCATGTCTACAAGgtaaaattgcagtctgtcttac
SRR516254.70243913.2+                      AGAAACACCATGTCTACAAGgtaaaattgcagtctgtcttac
SRR516255.70481761.1-                      AGAAACACCATGTCTACAAGgtaaaattgcagtctgtcttac
SRR516258.54033688.2+                       GAAACACCATGTCTACAAGgtaaaattgcagtctgtcttac
SRR516262.18560856.2+                       GAAACACCATGTCTACAAGgtaaaattgcagtctgtcttac
SRR516268.60041106.1+                       GAAACACCATGTCTACAAGgtaaaattgcagtctgtcttac
SRR516268.60041107.1+                       GAAACACCATGTCTACAAGgtaaaattgcagtctgtcttac
SRR516255.48156284.1-                        AAACACCATGTCTACAAGgtaaaattgcagtctgtcttac
SRR516261.32323269.1+                        AAACACCATGTCTACAAGgtaaaattgcagtctgtcttac
SRR516254.18031680.1-                         AACACCATGTCTACAAGgtaaaattgcagtctgtcttac
SRR516257.60080163.1+                         AACACCATGTCTACAAGgtaaaattgcagtctgtcttac
SRR516258.65537777.2-                          ACACCATGTCTACAAGgtaaaattgcagtctgtcttac
SRR516253.15894140.2+                           CACCATGTCTACAAGgtaaaattgcagtctgtcttac
SRR516254.34872139.2-                           CACCATGTCTACAAGgtaaaattgcagtctgtcttac
SRR516255.31500806.2-                           CACCATGTCTACAAGgtaaaattgcagtctgtcttac
SRR516257.7070288.1+                            CACCATGTCTACAAGgtaaaattgcagtctgtcttac
SRR516258.38065682.1+                           CACCATGTCTACAAGgtaaaattgcagtctgtcttac
SRR516258.70632626.2+                           CACCATGTCTACAAGgtaaaattgcagtctgtcttac
SRR516253.84549442.1-                             CCATGTCTACAAGgtaaaattgcagtctgtcttac
SRR516266.1137482.2-                              CCATGTCTACAAGgtaaaattgcagtctgtcttac
SRR516257.76140564.2+                              CATGTCTACAAGgtaaaattgcagtctgtcttac
SRR516257.76140570.2+                              CATGTCTACAAGgtaaaattgcagtctgtcttac
SRR516261.21596646.2+                              CATGTCTACAAGgtaaaattgcagtctgtcttac
SRR516253.19465821.2-                               ATGTCTACAAGgtaaaattgcagtctgtcttac
SRR516257.11274248.1-                               ATGTCTACAAGgtaaaattgcagtctgtcttac
SRR516257.4266993.1+                                 TGTCTACAAGgtaaaattgcagtctgtcttcc
SRR516258.23794530.1+                                TGTCTACAAGgtaaaattgcagtctgtcttac
SRR516263.17371779.1-                                 GTCTACAAGgtaaaattgcagtctgtcttac
SRR516267.18227146.2-                                 GTCTACAAGgtaaaattgcagtctgtcttac
SRR516258.14228804.1-                                  TCTACAAGgtaaaattgcagtctgtcttac
SRR516262.8153223.2-                                   TCTACAAGgtaaaattgcagtctgtcttac
SRR516262.8153225.2-                                   TCTACAAGgtaaaattgcagtctgtcttac
SRR516262.8155974.2-                                   TCTACAAGgtaaaattgcagtctgtcttac
SRR516266.11706985.1-                                  TCTACAAGgtaaaattgcagtctgtcttac
SRR516256.20061014.1-                                   CTACAAGgtaaaattgcagtctgtcttac
SRR516257.57533481.1+                                   CTACAAGgtaaaattgcagtctgtcttac
SRR516258.4196313.1+                                    CTACAAGgtaaaattgcagtctgtcttac
SRR516254.6572721.1+                                     TACAAGgtaaaattgcagtctgtcttac
SRR516255.3305445.2-                                     TACAAGgtaacattgcagtctgtcttac
```

```
SRR516255.42804038.1+                                     TACAAGgtaaaattgcagtctgtcttac
SRR516256.13911759.1-                                     TACAAGgtaaaattgcagtctgtcttac
SRR516255.62696371.2+                                      ACAAGgtaaaattgcagtctgtcttac
SRR516258.73534913.1-                                       CAAGgtaaaattgcagtctgtcttac
SRR516263.39307261.1-                                         AGgtaaaattgcagtctgtcttac
SRR516263.39307262.1-                                         AGgtaaaattgcagtctgtcttac
SRR516268.28354153.2+                                         AGgtaaaattgcagtctgtcttac
SRR516268.5144193.2+                                          AGgtaaaattgcagtctgtcttac
SRR516257.34678406.1+                                          Ggtaaaattgcagtctgtcttac
SRR516258.46278282.2-                                          Ggtaaaattgcagtctgtcttac
                         ____________________________________________________________
consensus                CCCTGCCCATGGCCAAGCAGAAACACCATGTCTACAAGgtaaaattgcagtctgtcttac
```

O. Cuckoo roller (No. 22), exon 4, WGS data

- CAP3 alignment of WGS data spanning exon 4 (uppercase letters)

```
                             .    :    .    :    .    :    .    :    .    :    .    :
SRR956935.33226072.2+    tccctgtgccaggggaacctcaggtgacttgtgtgtgtttattttccagTTT
SRR956934.5726880.1+     tccctgtgccaggggaacctcaggtgacttgtgtgtgtttcttttccagTTTG
SRR956933.15501326.2-    tccctgtgccaggggaacctcaggtgacttgtgtgtgtttcttttccagTTTGCCATTCC
SRR956933.47824422.2-    tccctgtgccaggggaacctcaggtgacttgtgtgtgtttcttttccagTTTGCCATTCC
SRR956933.49304017.2-    tccctgtgccaggggaacctcaggtgacttgtgtgtgtttcttttccagTTTGCCATTCC
SRR956933.5354156.1-     tccctgtgccaggggaacctcaggtgacttgtgtgtgtttcttttccagTTTGCCATTCT
SRR956933.67330544.1+    tccctgtgccaggggaacctcaggtgacttgtgtgtgtttcttttccagTTTGCCATTCT
SRR956933.79339270.2+    tccctgtgccaggggaacctcaggtgacttgtgtgtgtttcttttccagTTTGCCATTCC
SRR956934.17462198.1-    tccctgtgccaggggaacctcaggtgacttgtgtgtgtttcttttccagTTTGCCATTCT
SRR956934.49589762.1+    tccctgtgccaggggaacctcaggtgacttgtgtgtgtttcttttccagTTTGCCATTCT
SRR956934.50976425.1-    tccctgtgccaggggaacctcaggtgacttgtgtgtgtttcttttccagTTTGCCATTCT
SRR956934.73010703.1-    tccctgtgccaggggaacctcaggtgacttgtgtgtgtttcttttccagTTTGCCATTCC
SRR956935.14673017.1+    tccctgtgccaggggaacctcaggtgacttgtgtgtgtttcttttcccgTTTGCCATTCT
SRR956935.28715952.2+    tccctgtgccaggggaacctcaggtgacttgtgtgtgtttcttttcccgTTTGCCATTCT
SRR956935.36152290.1-    tccctgtgccaggggaacctcaggtgacttgtgtgtgtttcttttccagTTTGCCATTCT
SRR956935.40761063.2-    tccctgtgccaggggaacctcaggtgacttgtgtgtgtttcttttccagTTTGCCATTCT
SRR956933.86377582.2+      cctgtgccaggggaacctcaggtgacttgtgtgtgtttcttttccagTTTGCCATTCC
SRR956934.23198342.1+      cctgtgccaggggaacctcaggtgacttgtgtgtgtttcttttccagTTTGCCATTCT
SRR956933.63677473.2+           gccaggggaacctcaggtgacttgtgtgtgtttcttttccagTTTGCCATTCC
SRR956934.56341547.2-           gccaggggaacctcaggtgacttgtgtgtgtttcttttccagTTTGCCATTCT
SRR956933.75568344.2+                     cctcaggtgacttgtgtgtgtttcttttccagTTTGCCATTCT
SRR956933.28530959.1+                            tgacttgtgtgtgtttcttttccagTTTGCCATTCT
SRR956933.69159347.1+                            tgacttgtgtgtgtttcttttccagTTTGCCATTCT
SRR956935.27409973.1-                             gacttgtgtgtgtttcttttccagTTTGCCATTCT
SRR956935.14577549.1-                               cttgtgtgtgtttcttttccagTTTGCCATTCC
SRR956934.33032496.2+                                              tttccagTTTGCCATTCC
SRR956935.4217570.2+                                                          CCATTCT
SRR956934.31868316.1+                                                          CATTCC
SRR956933.79339270.1-                                                           ATTCC
SRR956933.14838491.1+                                                             TCT
SRR956934.62812362.2+                                                             TCT
SRR956934.21178397.1+                                                               T
SRR956935.35147434.2-                                                               C
                         ____________________________________________________________
consensus                tccctgtgccaggggaacctcaggtgacttgtgtgtgtttcttttccagTTTGCCATTCT

                             .    :    .    :    .    :    .    :    .    :    .    :
SRR956935.14673017.1+    AGC
SRR956934.73010703.1-    AGCTGAAGAAA
SRR956935.28715952.2+    AGCTGAAGAAAC
SRR956934.17462198.1-    AGCTGAAGAAACCAAATAT
SRR956933.67330544.1+    AGCTGAAGAAACCAAATATG
SRR956933.49304017.2-    AGCTGAAGAAACCAAATATGCAT
SRR956934.49589762.1+    AGCTGAAGAAACCAAATATGCATGTACCTT
SRR956933.15501326.2-    AGCTGAAGAAACCAAATATGCATGTACCTTTAT
SRR956935.36152290.1-    AGCTGAAGAAACCAAATATGCATGTACCTTTAT
SRR956933.5354156.1-     AGCTGAAGAAACCAAATATGCATGTACCTTTATCCC
SRR956934.50976425.1-    AGCTGAAGAAACCAAATATGCATGTACCTTTATCCC
SRR956933.79339270.2+    AGCTGAAGAAACCAAATATGCATGTACCTTTATCCCA
SRR956935.40761063.2-    AGCTGAAGAAACCAAATATGCATGTACCTTTATCCCA
SRR956933.47824422.2-    AGCTGAAGAAACCAAATATGCATGTACCTTTATCCCACT
SRR956933.86377582.2+    AGCTGAAGAAACCAAATATGCATGTACCTTTATCCCACTGCC
SRR956934.23198342.1+    AGCTGAAGAAACCAAATATGCATGTACCTTTATCCCACTGCC
SRR956933.63677473.2+    AGCTGAAGAAACCAAATATGCATGTACCTTTATCCCACTGCCCATGG
SRR956934.56341547.2-    AGCTGAAGAAACCAAATATGCATGTACCTTTATCCCACTGCCCATGG
SRR956934.21178397.1+    AGCTGAAGAAACCAAATATGCATGTACCTTTATCCCACTGCCCATGGTCAGCAG
SRR956933.75568344.2+    AGCTGAAGAAACCAAATATGCATGTACCTTTATCCCACTGCCCATGGTCAGCAGAAA
SRR956933.14838491.1+    AGCTGAAGAAACCAAATATGCATGTACCTTTATCCCACTGCCCATGGTCAGCAGAAACAC
SRR956933.28530959.1+    AGCTGAAGAAACCAAATATGCATGTACCTTTATCCCACTGCCCATGGTCAGCAGAAACAC
SRR956933.69159347.1+    AGCTGAAGAAACCAAATATGCATGTACCTTTATCCCACTGCCCATGGTCAGCAGAAACAC
SRR956933.79339270.1-    AGCTGAAGAAACCAAATATGCATGTACCTTTATCCCACTGCCCATGGTCAGCAGAAACAC
SRR956934.31868316.1+    AGCTGAAGAAACCAAATATGCATGTACCTTTATCCCACTGCCCATGGTCAGCAGAAACAC
SRR956934.33032496.2+    AGCTGAAGAAACCAAATATGCATGTACCTTTATCCCACTGCCCATGGTCAGCAGAAACAC
SRR956934.62812362.2+    AGCTGAAGAAACCAAATATGCATGTACCTTTATCCCACTGCCCATGGTCAGCAGAAACAC
SRR956935.14577549.1-    AGCTGAAGAAACCAAATATGCATGTACCTTTATCCCACTGCCCATGGTCAGCAGAAACAC
SRR956935.27409973.1-    AGCTGAAGAAACCAAATATGCATGTACCTTTATCCCACTGCCCATGGTCAGCAGAAACAC
SRR956935.35147434.2-    AGCTGAAGAAACCAAATATGCATGTACCTTTATCCCACTGCCCATGGTCAGCAGAAACAC
SRR956935.4217570.2+     AGCTGAAGAAACCAAATATGCATGTACCTTTATCCCACTGCCCATGGTCAGCAGAAACAC
SRR956935.35312808.1+      CTGAAGAAACCAAATATGCATGTACCTTTATCCCACTGCCCATGGTCAGCAGAAACAC
SRR956933.67330544.2-         AAGAAACCAAATATGCATGTACCTTTATCCCACTGCCCATGGTCAGCAGAAACAC
SRR956933.86377582.1-          AGAAACCAAATATGCATGTACCTTTATCCCACTGCCCATGGTCAGCAGAAACAC
SRR956933.47299640.2+           GAAACCAAATATGCATGTACCTTTATCCCACTGCCCATGGTCAGCAGAAACAC
SRR956935.36662711.1+                CAAATATGCATGTACCTTTATCCCACTGCCCATGGTCAGCAGAAACAC
SRR956934.63625329.1+                       GCATGTACCTTTATCCCACTGCCCATGGTCAGCAGAAACAC
SRR956934.73546604.2+                              CCTTTATCCCACTGCCCATGGTCAGCAGAAACAC
```

```
SRR956935.64705649.1-                                CTTTATCCCCTGCCCATGGTCAGCAGAAACCC
SRR956933.10080307.2+                                           GCCCATGGTCAGCAGAAACAC
SRR956933.63677473.1-                                           GCCCCTGGTCAGCAGAAACCC
SRR956934.36665069.2-                                           GCCCAAGGTCAGCAGAAACAC
SRR956935.32484767.1+                                           GCCCATGGTCAGCAGAAACAC
SRR956935.24603228.1+                                             CCATGGTCAGCAGAAACAC
SRR956934.64268426.2-                                                 GGTCAGCAGAAACAC
SRR956934.58087977.1-                                                          AAACCC
                         ____________________________________________________________
consensus                AGCTGAAGAAACCAAATATGCATGTACCTTTATCCCACTGCCCATGGTCAGCAGAAACAC

                             .    :    .    :    .    :    .    :    .    :    .    :
SRR956933.28530959.1+    CGTA
SRR956933.69159347.1+    CGTA
SRR956935.27409973.1-    CGTAT
SRR956935.14577549.1-    CGTATCT
SRR956934.33032496.2+    CGTATCTACAAGgtaaacttgc
SRR956935.4217570.2+     CGTATCTACAAGgtaaacttgcaggctgcctta
SRR956934.31868316.1+    CGTATCTACAAGgtaaacttgcaggctgccttac
SRR956933.79339270.1-    CGTATCTACAAGgtaaacttgcaggctgccttact
SRR956933.14838491.1+    CGTATCTACAAGgtaaacttgcaggctggcttactgg
SRR956934.62812362.2+    CGTATCTACAAGgtaaacttgcaggctgccttactgg
SRR956935.35147434.2-    CGTATCTACAAGgtaaacttgcaggctgccttactgggg
SRR956935.35312808.1+    CGTATCTACAAGgtaaacttgcaggctgccttactgggg
SRR956933.67330544.2-    CGTATCTACAAGgtaaacttgcaggctgccttactggggaaaaaa
SRR956933.86377582.1-    CGTATCTACAAGgtaaacttgcaggctgccttactggggaaaaaaa
SRR956933.47299640.2+    CGTATCTACAAGgtaaacttgcaggctgccttactggggaaaaaaaa
SRR956934.73546604.2+    CGTATCTACAAGgtaaacttgcaggctgccttactggggaaaaaaaaaaaaa
SRR956935.36662711.1+    CGTATCTACAAGgtaaacttgcaggctgccttactggggaaaaaaaaaaaaa
SRR956933.63677473.1-    CGTATCTACAAGggaaacttggaggctgcctttcgggggaaaaaaaaaaaaaaaaaa
SRR956933.10080307.2+    CGTATCTACAAGgtaaacttgcaggctgccttactggggaaaaaaaaaaaaaaaaaaa
SRR956935.32484767.1+    CGTATCTACAAGgtaaacttgcaggctgccttactggggaaaaaaaaaaaaaaaaaaa
SRR956934.36665069.2-    CGTATCTACAAGgtaaacttgcaggctgccttactggggaaaaaaaaaaaaaaaaaaaa
SRR956934.63625329.1+    CGTATCTACAAGgtaaacttgcaggctgccttactggggaaaaaaaaaaaaaaaaaaaa
SRR956935.24603228.1+    CGTATCTACAAGgtaaacttgcaggctgccttactggggaaaaaaaaaaaaaaaaaaag
SRR956935.64705649.1-    CGTATTTACAAGgtaaacttgcaggctgccttactggggaaaaaaaaaaaaaaaaaaaa
SRR956934.58087977.1-    CGTATCTACAAGgtaaacttggaggctgccttactggggaaaaaaaaaaaaaaaaaaagg
SRR956934.64268426.2-    CGTATCTACAAGgtaaacttgcaggctgccttactggggaaaaaaaaaaaaaaaaaa-gg
SRR956934.32598380.1-       ATCTACAAGgtaaacttgcaggctgccttacggggaaaaaaaaaaaaaaaaaaaagg
SRR956935.64634243.2-       ATCTACAAGgtaaccttggaggctgccttactggggaaaaaaaaaaaaaaaaaa-gg
                         ____________________________________________________________
consensus                CGTATCTACAAGgtaaacttgcaggctgccttactggggaaaaaaaaaaaaaaaaaaagg
```

P. Speckled mousebird (No. 23), exon 4, WGS data

- CAP3 alignment of WGS data spanning exon 4 (uppercase letters)

```
                             .    :    .    :    .    :    .    :    .    :    .    :
SRR955003.105491392.1+   tgggtgtttcttttccaaTTTGCTGCTCCAGTTGAAAAAACCTAGCATGTCTGTACCTTT
SRR955003.70259149.1-        tgtttcttttccaaTTTGCTGCTCCAGTTGAAAAAACCTAGCATGTCTGTACCTTT
SRR955003.24405349.2-              ttttccaaTTTGCTGCTCCAGTTGAAAAAACCTAGCATGTCTGTACCTTT
SRR955004.35002639.1-                  ccaaTTTGCTGCTCCAGTTGAAAAAACCTAGCATGTCTGTACCTTT
SRR955003.110630919.1+                      TTGCTGCTCCAGTTGAAAAAACCTAGCATGTCTGTACCTTT
SRR955003.33105841.1+                           TGCTCCAGTTGAAAAAACCTAGCATGTCTGTACCTTT
SRR955004.25917219.2+                              TCCAGTTGAAAAAACCTAGCATGTCTGTACCTTT
SRR955004.60388681.1+                                   TTGAAAAAACCTAGCATGTCTGTACCTTT
SRR955003.37653578.1-                                          AACCTAGCATGTCTGTACCTTT
SRR955003.28157826.2+                                                  ATGTCTGTACCTTT
SRR955003.21063446.1+                                                      CTGTACCTTT
SRR955003.98399332.1+                                                       TGTACCTTT
SRR955003.50352646.2-                                                        GTACCTTT
SRR955003.94750184.1+                                                         TACCTTT
SRR955003.58074590.2+                                                           CCTTT
SRR955003.84103845.1+                                                           CCTTT
SRR955003.110836653.1+                                                           CTTT
SRR955003.107556485.1+                                                              T

consensus                tgggtgtttcttttccaaTTTGCTGCTCCAGTTGAAAAAACCTAGCATGTCTGTACCTTT

                             .    :    .    :    .    :    .    :    .    :    .    :
SRR955003.105491392.1+   ATGCCACTGACCATGGCCAAGCAGAACCACTATTATCAAG
SRR955003.70259149.1-    ATGCCACTGACCATGGCCAAGCAGAAACACTATTATCAAGgtaa
SRR955003.24405349.2-    ATGCCACTGACCATGGCCAAGCAGAAACACTATTATCAAGgtaaatttgc
SRR955004.35002639.1-    ATGCCACTGACCATGGCCAAGCAGAAACACTATTATCAAGgtaaatttgcaggt
SRR955003.110630919.1+   ATGCCACTGACCATGGCCAAGCAGAAACACTATTATCAAGgtaaatttgcaggttgcct
SRR955003.107556485.1+   ATGCCACTGACCATGGCCAAGCAGAAACACTATTATCAAGgtaaatttgcaggttgcctt
SRR955003.110836653.1+   ATGCCACTGACCATGGCCAAGCAGAAACACTATTATCAAGgtaaatttgcaggttgcctt
SRR955003.21063446.1+    ATGCCACTGACCATGGCCAAGCAGAAACACTATTATCAAGgtaaatttgcaggttgcctt
SRR955003.28157826.2+    ATGCCACTGACCATGGCCAAGCAGAAACACTATTATCAAGgtaaatttgcaggttgcctt
SRR955003.33105841.1+    ATGCCACTGACCATGGCCAAGCAGAAACACTATTATCAAGgtaaatttgcaggttgcctt
SRR955003.37653578.1-    ATGCCACTGACCATGGCCAAGCAGAAACACTATTATCAAGgtaaatttgcaggttgcctt
SRR955003.50352646.2-    ATGCCACTGACCATGGCCAAGCAGAAACACTATTATCAAGgtaaatttgcaggttgcctt
SRR955003.58074590.2+    ATGCCACTGACCATGGCCAAGCAGAAACACTATTATCAAGgtaaatttgcaggttgcctt
SRR955003.84103845.1+    ATGCCACTGACCATGGCCAAGCAGAAACACTATTATCAAGgtaaatttgcaggttgcctt
SRR955003.94750184.1+    ATGCCACTGACCATGGCCAAGCAGAAACACTATTATCAAGgtaaatttgcaggttgcctt
SRR955003.98399332.1+    ATGCCACTGACCATGGCCAAGCAGAAACACTATTATCAAGgtaaatttgcaggttgcctt
SRR955004.25917219.2+    ATGCCACTGACCATGGCCAAGCAGAAACACTATTATCAAGgtaaatttgaaggttgcctt
SRR955004.60388681.1+    ATGCCACTGACCATGGCCAAGCAGAAACACTATTATCAAGgtaaatttgcaggttgcctt
SRR955003.94738916.1-        CACTGACCATGGCCAAGCAGAAACACTATTATCAAGgtaaatttgcaggttgcctt
SRR955003.76475839.2-             ACCATGGCCAAGCAGAAACACTATTATCAAGgtaaatttgcaggttgcctt
SRR955003.37307100.2+                         CAGAAACACTATTATCAAGgtaaatttgcaggttgcctt
SRR955003.75057278.2+                          AGAAACACTATTATCAAGgtaaatttgcaggttgcctt
SRR955004.24836517.2-                          AGAAACACTATTATCAAGgtaaatttgcaggttgcctt
SRR955004.63401237.2-                           GAAACACTATTATCAAGgtaaatttgcaggttgcctt
SRR955003.9914472.2-                             AAACACTATTATCAAGgtaaatttgcaggttgcctt
SRR955004.31681147.2+                             AACACTATTATCAAGgtaaatttgcaggttgcctt
SRR955004.42573459.2-                                 CTATTATCAAGgtaaatttgcaggttgcctt
SRR955004.4298586.2+                                  CTATTATCAAGgaaaatttgcaggttgcctt
SRR955003.86841563.1+                                        CAAGgtaaatttgcaggttgcctt
SRR955003.57069806.1-                                         AAGgtaaatttgcaggttgcctt

consensus                ATGCCACTGACCATGGCCAAGCAGAAACACTATTATCAAGgtaaatttgcaggttgcctt
```

Q. Sunbittern (No. 29), exon 4, WGS data

- CAP3 alignment of WGS data spanning exon 4 (uppercase letters)

```
                             .    :    .    :    .    :    .    :    .    :    .    :
SRR955403.36605629.1+    gttgccacagaaaccttcaggtgactcttgtgtctgtttcttttccagT
SRR955403.3947585.1+     gttgccacagaaaccttcaggtgactcttgtgtctgtttcttttccagTT
SRR955405.22537442.1-    gttgccacagaaaccttcaggtgactcttgtgtctgtttcttttccagTT
SRR955403.94847071.2-    gttgccacagaaaccttcaggtgactcttgtgtctgtttcttttccagTTT
SRR955405.46669857.1-    gttgccacagaaaccttcaggtgactcttgtgtctgtttcttttccagTTT
SRR955403.104048366.2-   gttgccacagaaaccttcaggtgactcttgtgtctgtttcttttccagTTTGTT
SRR955403.67206227.1+    gttgccacagaaaccttcaggtgactcttgtgtctgtttcttttccagTTTGTTG
SRR955405.34734344.1+    gttgccacagaaaccttcaggtgactcttgtgtctgtttcttttccagTTTGTTGT
SRR955403.20178932.2-    gttgccacagaaaccttcaggtgactcttgtgtctgtttcttttccagTTTGTTGTTCC
SRR955403.11572506.2+    gttgccacagaaaccttcaggtgactcttgtgtctgtttcttttccagTTTGTTGTTCCT
SRR955403.1418969.1-     gttgccacagaaaccttcaggtgactcttgtgtctgtttcttttccagTTTGTTGTTCCT
SRR955403.18890725.1-    gttgccacagagaccttcaggtgactcttgtgtctgtttcttttccagTTTGTTGTTCCT
SRR955403.73606013.2+    gttgccacagaaaccttcaggtgactcttgtgtctgtttcttttccagTTTGTTGTTCCT
SRR955404.29078451.1-    gttgccacagaaaccttcaggtgactcttgtgtctgtttcttttccagTTTGTTGTTCCT
SRR955404.54624084.1+    gttgccacagaaaccttcaggtgactcttgtgtctgtttcttttccagTTTGTTGTTCCT
SRR955405.19027456.2-    gttgccacagaaaccttcaggtgactcttgtgtctgtttcttttccagTTTGTTGTTCCT
SRR955405.2762221.1-     gttgccacagaaaccttcaggtgactcttgtgtctgtttcttttccagTTTGTTGTTCCT
SRR955405.48827651.1-    gttgccacagaaaccttcaggtgactcttgtgtctgtttcttttccagTTTGTTGTTCCT
SRR955405.50023141.1-    gttgccacagaaaccttcaggtgactcttgtgtctgtttcttttccagTTTGTTGTTCCT
SRR955405.58084698.1-    gttgccacagaaaccttcaggtgactcttgtgtctgtttcttttccagTTTGTTGTTCCT
SRR955403.26514073.2+       gccacagaaaccttcaggtgactcttgtgtctgtttcttttccagTTTGTTGTTCCT
SRR955404.56098309.2+       gccacagaaaccttcaggtgactcttgtgtctgtttcttttccagTTTGTTGTTCCT
SRR955405.49245645.1+       gccacagaaaccttcaggtgactcttgtgtctgtttcttttccagTTTGTTGTTCCT
SRR955405.15935601.1-                accttcaggtgactcttgtgtctgtttcttttccagTTTGTTGTTCCT
SRR955404.6929864.1+                          tgactcttgtgtctgtttcttttccagTTTGTTGTTCCT
SRR955403.74943617.2-                          gactcttgtgtctgtttcttttccagTTTGTTGTTCCT
SRR955403.83080227.1+                          gactcttgtgtctgtttcttttccagTTTGTTGTTTCT
SRR955404.32490642.1+                          gactcttgtgtctgtttcttttccagTTTGTTGTTCCT
SRR955404.34527715.1+                          gactcttgtgtctgtttcttttccagTTTGTTGTTCCT
SRR955405.18081326.1+                          gactcttgtgtctgtttcttttccagTTTGTTGTTCCT
SRR955403.48102659.2+                           actcttgtgtctgtttcttttccagTTTGTTGTTCCT
SRR955404.27558151.2-                                      tgtttcttttccagTTTGTTGTTCCT
SRR955403.105372963.2-                                      gtttcttttccagTTTGTTGTTCCT
SRR955404.26773079.1-                                       gtttcttttccagTTTGTTGTTCCT
SRR955404.21667925.2-                                                 cagTTTGTTGTTCCT
SRR955405.12019009.2-                                                 cagTTTGTTGTTCCT
SRR955405.40333455.2+                                                  agTTTGTTGTTCCT
SRR955403.18781244.2-                                                    TTTGTTGTTCCT
SRR955404.52396791.1-                                                    TTTGTTGTTCCT
SRR955404.8824301.1-                                                     TTTGTTGTTCCT
SRR955405.17028899.1-                                                    TTTGTTGTGCCT
SRR955405.1899247.2-                                                     TTTGTTGTTCCT
SRR955405.7466411.1-                                                     TTTGTTGTTCCT
SRR955405.74881191.1-                                                    TTTGTTGTTCCT
SRR955403.54489646.2+                                                       GTTGTTCCT
SRR955405.31368143.1-                                                         TGTTCCT
SRR955404.81836800.1-                                                          GTTCCT
SRR955405.59475718.1-                                                          GTTCCT
SRR955403.43269048.2-                                                            TCCT
SRR955403.93811490.1-                                                            TCCT
SRR955404.10640934.1-                                                            TCCT
SRR955404.20012032.1-                                                            TCCT
SRR955404.3517446.1-                                                             TCCT
SRR955404.40600314.1-                                                            TCCT
SRR955404.43181078.1-                                                            TCCT
SRR955404.49301553.1-                                                            TCCT
SRR955405.75901177.2+                                                             CCT
SRR955403.92666487.2-                                                               T

consensus                gttgccacagaaaccttcaggtgactcttgtgtctgtttcttttccagTTTGTTGTTCCT

                             .    :    .    :    .    :    .    :    .    :    .    :
SRR955405.58084698.1-    G
SRR955403.18890725.1-    GTT
SRR955405.50023141.1-    GTTGAAGAAAACA
SRR955405.19027456.2-    GTTGAAGAAAACAAGT
SRR955404.54624084.1+    GTTGAAGAAAACAAGTATGCCGGTG
SRR955404.29078451.1-    GTTGAAGAAAACAAGTATGCCGGTGC
SRR955403.73606013.2+    GTTGAAGAAAACAAGTATGCCGGTGCCTTTAT
SRR955405.2762221.1-     GTTGAAGAAAACAAGTATGCCGGTGCCTTTATCCCACT
SRR955403.1418969.1-     GTTGAAGAAAACAAGTATGCCGGTGCCTTTATCCCACTGC
SRR955403.26514073.2+    GTTGAAGAAAACAAGTATGCCGGTGCCTTTATCCCACTGCCTG
SRR955404.56098309.2+    GTTGAAGAAAACAAGTATGCCGGTGCCTTTATCCCACTGCCTG
SRR955405.49245645.1+    GTTGAAGAAAACAAGTATGCCGGTGCCTTTATCCCACTGCCTG
SRR955405.15935601.1-    GTTGAAGAAAACAAGTATGCCGGTGCCTTTATCCCACTGCCTGTGGTCGAGC
```

```
SRR955403.83080227.1+    GTTGAAGAAAACAAGTATGCCGGGGCCTTTATCCCACTGCCTGTGGTCGAGCAGAAAAC
SRR955403.105372963.2-   GTTGAAGAAAACAAGTATGCCGGTGCCTTTATCCCACTGCCTGTGGTCGAGCAGAAAACA
SRR955403.18781244.2-    GTTGAAGAAAACAAGTATGCCGGTGCCTTTATCCCACTGCCTGTGGTCGAGCAGAAAACA
SRR955403.43269048.2-    GTTGAAGAAAACAAGTATGCCGGTGCCTTTATCCCACTGCCTGTGGTCGAGCAGAAAACA
SRR955403.48102659.2+    GTTGAAGAAAACAAGTATGCCGGTGCCTTTATCCCACTGCCTGTGGTCGAGCAGAAAACA
SRR955403.54489646.2+    GTTGAAGAAAACAAGTATGCCGGTGCCTTTATCCCACTGCCTGTGGTCGAGCAGAAAACA
SRR955403.74943617.2-    GTTGAAGAAAACAAGTATGCCGGTGCCTTTATCCCACTGCCTGTGGTCGAGCAGAAAACA
SRR955403.92666487.2-    GTTGAAGAAAACAAGTATGCCGGTGCCTTTATCCCACTGCCTGTGGTCGAGCAGAAAACT
SRR955403.93811490.1-    GTTGAAGAAAACAAGTATGCCGGTGCCTTTATCCCACTGCCTGTGGTCGAGCAGAAAACA
SRR955404.10640934.1-    GTTGAAGAAAACAAGTATGCCGGTGCCTTTATCCCACTGCCTGTGGTCGAGCAGAAAACA
SRR955404.20012032.1-    GTTGAAGAAAACAAGTATGCCGGTGCCTTTATCCCACTGCCTGTGGTCGAGCAGAAAACA
SRR955404.21667925.2-    GTTGAAGAAAACAAGTATGCCGGTGCCTTTATCCCACTGCCTGTGGTCGAGCAGAAAACA
SRR955404.26773079.1-    GTTGAAGAAAACAAGTATGCCGGTGCCTTTATCCCACTGCCTGTGGTCGAGCAGAAAACA
SRR955404.27558151.2-    GTTGAAGAAAACAAGTATGCCGGTGCCTTTATCCCACTGCCTGTGGTCGAGCAGAAAACA
SRR955404.32490642.1+    GTTGAAGAAAACAAGTATGCCGGTGCCTTTATCCCACTGCCTGTGGTCGAGCAGAAAACA
SRR955404.34527715.1+    GTTGAAGAAAACAAGTATGCCGGTGACTTTATCCCACTGCCTGTGGTCGAGCAGAAAACA
SRR955404.3517446.1-     GTGGAAGAAAACAAGTAGGCCGGTGCCTTTATCCCACTGCCTGTGGTCGAGCAGAAAACA
SRR955404.40600314.1-    GTTGAAGAAAACAAGTATGCCGGTGCCTTTATCCCACTGCCTGTGGTCGAGCAGAAAACA
SRR955404.43181078.1-    GTTGAAGAAAACAAGTATGCCGGTGCCTTTATCCCACTGCCTGTGGTCGAGCAGAAAACA
SRR955404.49301553.1-    GTTGAAGAAAACAAGTATGCCGGTGCCTTTATCCCACTGCCTGTGGTCGAGCAGAAAACA
SRR955404.52396791.1-    GTTGAAGAAAACAAGTATGCCGGTGCCTTTATCCCACTGCCTGTGGTCGAGCAGAAAACA
SRR955404.6929864.1+     GTTGAAGAAAACAAGTATGCCGGTGCCTTTATCCCACTGCCTGTGGTCGAGCAGAAAACA
SRR955404.81836800.1-    GTTGAAGAAAACAAGTATGCCGGTGCCTTTATCCCACTGCCTGTGGTCGAGCAGAAAACA
SRR955404.8824301.1-     GTTGAAGAAAACAAGTATGCCGGTGCCTTTATCCCACTGCCTGTGGTCGAGCAGAAAACA
SRR955405.12019009.2-    GTTGAAGAAAACAAGTATGCCGGTGCCTTTATCCCACTGCCTGTGGTCGAGCAGAAAACA
SRR955405.17028899.1-    GTTGAAGAAAACAAGTATGCCGGTGCCTTTATCCCACTGCCTGTGGTCGAGCAGAAAACA
SRR955405.18081326.1+    GTTGAAGAAAACAAGTATGCCGGTGCCTTTATCCCACTGCCTGTGGTCGAGCAGAAAACA
SRR955405.1899247.2-     GTTGAAGAAAACAAGTAGGCCGGTGCCTTTATCCCACTGCCTGTGGTCGAGCAGAAAACA
SRR955405.31368143.1-    GTTGAAAAAAACAAGTATGCCGGTGCCTTTATCCCACTGCCTGTGGTCGAGCAGAAAACA
SRR955405.40333455.2+    GTTGAAGAAAACAAGTATGCCGGTGCCTTTATCCCACTGCCTGTGGTCGAGCAGAAAACA
SRR955405.59475718.1-    GTTGAAGAAAACAAGTATGCCGGTGCCTTTATCCCACTGCCTGTGGTCGAGCAGAAAACA
SRR955405.7466411.1-     GTTGAAGAAAACAAGTATGCCGGTGCCTTTATCCCACTGCCTGTGGTCGAGCAGAAAACA
SRR955405.74881191.1-    GTTGAAGAAAACAAGTATGCCGGTGCCTTTATCCCACTGCCTGTGGTCGAGCAGAAAACA
SRR955405.75901177.2+    GTTGAAGAAAACAAGTATGCCGGTGCCTTTATCCCACTGCCTGTGGTCGAGCAGAAAACA
SRR955403.21685211.1-          GAAAACAAGTATGCCGGTGCCTTTATCCCACTGCCTGTGGTCGAGCAGAAAACA
SRR955403.87209781.2-              CAAGTATGCCGGTGCCTTTATCCCACTGCCTGTGGTCGAGCAGAAAACA
SRR955405.35312982.2+              CAAGTATGCCGGTGCCTTTATCCCACTGCCTGTGGTCGAGCAGAAAACA
SRR955404.46010507.1+                  TATGCCGGTGCCTTTATCCCACTGCCTGTGGTCGAGCAGAAAACA
SRR955405.37881069.2+                  TATGCCGGTGCCTTTATCCCACTGCCTGTGGTCGAGCAGAAAACA
SRR955405.44936739.1-                    TGCCGGTGCCTTTATCCCACTGCCTGTGGTCGAGCAGAAAACA
SRR955403.8583252.1+                      GCCGGTGCCTTTATCCCACTGCCTGTGGTCGAGCAGAAAACA
SRR955404.62566799.1-                     GCCGGTGCCTTTATCCCACTGCCTGTGGTCGAGCAGAAAACA
SRR955405.11856992.2+                       CGGTGCCTTTATCCCACTGCCTGTGGTCGAGCAGAAAACA
SRR955405.65844503.2+                       CGGTGCCTTTATCCCACTGCCTGTGGTCGAGCAGAAAACA
SRR955405.35474003.2-                        GGTGCCTTTATCCCACTGCCTGTGGTCGAGCAGAAAACA
SRR955403.31834296.2-                         GTGCCTTTATCCCACTGCCTGTGGTCGAGCAGAAAACA
SRR955404.27692177.1-                           GCCTTTATCCCACTGCCTGTGGTCGAGCAGAAAACA
SRR955403.53065993.1+                            CCTTTATCCCACTGCCTGTGGTCGAGCAGAAAACA
SRR955404.10996986.1+                            CCTTTATCCCACTGCCTGTGGTCGAGCAGAAAACA
SRR955403.53504813.2+                              TTTATCCCACTGCCTGTGGTCGAGCAGAAAACA
SRR955404.76405909.2-                                TATCCCACTGCCTGTGGTCGAGCAGAAAACA
SRR955404.8635954.2-                                   TCCCACTGCCTGTGGTCGAGCAGAAAACA
SRR955403.47362371.1+                                   CCACTGCCTGTGGTCGAGCAGAAAACA
SRR955404.6799976.2-                                           CTGTGGTCGAGCAGAAAACA
SRR955403.39527254.1-                                           TGTGGTCGAGCAGAAAACA
SRR955404.33923875.2+                                              GGTCGAGCAGAAAACA
SRR955403.61722740.1-                                                TCGAGCAGAAAACA
SRR955404.4284993.2-                                                  CGAGCAGAAAACA
SRR955405.47522187.2+                                                      AGAAAACA
SRR955405.64496342.1+                                                      AGAAAACA
SRR955404.14677905.2+                                                           ACA
SRR955404.52780057.2+                                                           ACA
SRR955403.39059575.1-                                                            CA
SRR955403.59648782.2+                                                             A
SRR955405.25863746.1+                                                             A

consensus                GTTGAAGAAAACAAGTATGCCGGTGCCTTTATCCCACTGCCTGTGGTCGAGCAGAAAACA

                             .    :    .    :    .    :    .    :    .    :    .    :
SRR955404.6929864.1+     C
SRR955403.74943617.2-    CC
SRR955404.32490642.1+    CC
SRR955404.34527715.1+    CC
SRR955405.18081326.1+    CC
SRR955403.48102659.2+    CCG
SRR955404.27558151.2-    CCGTATCTACAAGg
SRR955403.105372963.2-   CCGTATCTACAAGgt
SRR955404.26773079.1-    CCGTATCTACAAGgt
SRR955404.21667925.2-    CCGTATCTACAAGgtaaacttgcag
SRR955405.12019009.2-    CCGTATCTACAAGgtaaacttgcag
SRR955405.40333455.2+    CCGTATCTACAAGgtaaacttgcagg
SRR955403.18781244.2-    CCGTATCTACAAGgtaaacttgcaggct
SRR955404.52396791.1-    CCGTATCTACAAGgtaaacttgcaggct
SRR955404.8824301.1-     CCGTATCTACAAGgtaaacttgcaggct
```

```
SRR955405.17028899.1-    CCGTATCTACAAGgtaaacttgcaggct
SRR955405.1899247.2-     CCGTATCTACAAGgtaaacttgcaggct
SRR955405.31368143.1-    CCGTATCTACAAGgtaaacttgcaggct
SRR955405.7466411.1-     CCGTATCTACAAGgtaaacttgcaggct
SRR955405.74881191.1-    CCGTATCTACAAGgtaaacttgcaggct
SRR955403.54489646.2+    CCGTATCTACAAGgtaaacttgcaggctgta
SRR955404.81836800.1-    CCGTATCTACAAGgtaaacttgcaggctgtatta
SRR955405.59475718.1-    CCGTATCTACAAGgtaaacttgcaggctgtatta
SRR955403.43269048.2-    CCGTATCTACAAGgtaaacttgcaggctgtattact
SRR955403.93811490.1-    CCGTATCTACAAGgtaaacttgcaggctgtattact
SRR955404.10640934.1-    CCGTATCTACAAGgtaaacttgcaggctgtattact
SRR955404.20012032.1-    CCGTATCTACAAGgtaaacttgcaggctgtattact
SRR955404.3517446.1-     CCGTATCTACAAGgtaaacttgcaggctgtattact
SRR955404.40600314.1-    CCGTATCTACAAGgtaaacttgcaggctgtattact
SRR955404.43181078.1-    CCGTATCTACAAGgtaaacttgcaggctgtattact
SRR955404.49301553.1-    CCGTATCTACAAGgtaaacttgcaggctgtattact
SRR955405.75901177.2+    CCGTATCTACAAGgtaaacttgcaggctgtattactg
SRR955403.92666487.2-    CCGTATCTACAAGgtaaacttgcaggctgtattactggg
SRR955403.21685211.1-    CCGTATCTACAAGgtaaacttgcaggctgtattactgggggaaaaa
SRR955403.87209781.2-    CCGTATCTACAAGgtaaacttgcaggctgtattactgggggaaaaacagga
SRR955405.35312982.2+    CCGTATCTACAAGgtaaacttgcaggctgtattactgggggaaaaacagga
SRR955405.65844503.2+    CCGTATCTACAAGgtaaacttgcaggctgtattactggggggaaaacagga
SRR955404.46010507.1+    CCGTATCTACAAGgtaaacttgcaggctgtattactgggggcaaaacaggacagg
SRR955405.11856992.2+    CCGTATCTACAAGgtaaacttgcaggctgtattactgggggcaaaacaggacagg
SRR955405.37881069.2+    CCGTATCTACAAGgtaaacttgcaggctgtattactgggggaaaaacaggacagg
SRR955405.44936739.1-    CCGTATCTACAAGgtaaacttgcaggctgtattactgggggaaaaacaggacaggag
SRR955403.8583252.1+     CCGTATCTACAAGgtaaacttgcaggctgtattactgggggaaaaacaggacaggagt
SRR955404.62566799.1-    CCGTATCTACAAGgtaaacttgcaggctgtattactgggggaaaaacaggacaggagt
SRR955403.31834296.2-    CCGTATCTACAAGgtaaacttgcaggctgtattactgggggaaaaacaggacaggagtct
SRR955403.39059575.1-    CCGTATCTACAAGgtaaacttgcaggctgtattactgggggaaaaacaggacaggagtct
SRR955403.39527254.1-    CCGTATCTACAAGgtaaacttgcaggctgtattactgggggaaaaacaggacaggagtct
SRR955403.47362371.1+    CCGTATCTACAAGgtaaacttgcaggctgtattactgggggaaaaacaggacaggagtct
SRR955403.53065993.1+    CCGTATCTACAAGgtaaacttgcaggctgtattactgggggaaaaacaggacaggagtct
SRR955403.53504813.2+    CCGTATCTACAAGgtaaacttgcaggctgtattactgggggaaaaacaggacaggagtct
SRR955403.59648782.2+    CCGTATCTACAAGgtaaacttgcaggctgtattactgggggaaaaacaggacaggagtct
SRR955403.61722740.1-    CCGTATCTACAAGgtaaacttgcaggctgtattactgggggaaaaacaggacaggagtct
SRR955404.10996986.1+    CCGTATCTACAAGgtaaacttgcaggctgtattactgggggaaaaacaggacaggagtcc
SRR955404.14677905.2+    CCGTATCTACAAGgtaaacttgcaggctgtattactgggggaaaaacaggacaggagtct
SRR955404.27692177.1-    CCGTATCTACAAGgtaaacttgcaggctgtattactgggggaaaaacaggacaggagtct
SRR955404.33923875.2+    CCGTATCTACAAGgtaaacttgcaggctgtattactgggggaaaaacaggacaggagtct
SRR955404.4284993.2-     CCGTATCTACAAGgtaaacttgcaggctgtattactgggggaaaaacaggacaggagtct
SRR955404.52780057.2+    CCGTATCTACAAGgtaaacttgcaggctgtattactgggggaaaaacaggacaggagtct
SRR955404.6799976.2-     CCGTATCTACAAGgtaaacttgcaggctgtattactgggggaaaaacaggacaggagtct
SRR955404.76405909.2-    CCGTATCTACAAGgtaaacttgcaggctgtattactgggggaaaaacaggacaggagtct
SRR955404.8635954.2-     CCGTATCTACAAGgtaaacttgcaggctgtattactgggggaaaaacaggacaggagtct
SRR955405.12381906.1-    CCGTATCTACAAGgtaaacttgcaggctgtattactgggggaaaaacaggacaggagtct
SRR955405.25863746.1+    CCGTATCTACAAGgtaaacttgcaggctgtattactgggggaaaaacaggacaggagtct
SRR955405.35474003.2-    CCGTATCTACAAGgtaaacttgcaggctgtattactgggggaaaaacaggacaggagtct
SRR955405.47522187.2+    CCGTATCTACAAGgtaaacttgcaggctgtattactgggggaaaaacaggacaggagtct
SRR955405.64496342.1+    CCGTATCTACAAGgtaaacttgcaggctgtattactgggggaaaaacaggacaggagtct
SRR955404.41810506.2+      GTATCTACAAGgtaaacttgcaggctgtattactgggggaaaaacaggacaggagtct
SRR955403.58740753.2-           TACAAGgtaaacttgcgggctgtattactgggggaaaaacaggacaggagtct
SRR955404.84628401.1+              AAGgtaaacttgcaggctgtattactgggggaaaaacaggacaggagtct
SRR955403.14283255.1-               AGgtaaacttgcaggctgtattactgggggaaaaacaggacaggagtct
SRR955404.34992271.1-               AGgtaaacttgcaggctgtattactgggggaaaaacaggacaggagtct
SRR955405.14570796.1+                Ggtaaacttgcaggctgtattactgggggaaaaacaggacaggagtct
                         ____________________________________________________________
consensus                CCGTATCTACAAGgtaaacttgcaggctgtattactgggggaaaaacaggacaggagtct
```

R. Great cormorant (No. 34), exon 4, WGS data

- CAP3 alignment of WGS data spanning exon 4 (uppercase letters)

```
                             .    :    .    :    .    :    .    :    .    :    .    :
SRR959560.90250972.1+    gaccttcaggtgccttgtgtgtgtctttttT
SRR959561.40149424.2-    gaccttcaggtgccttgtgtgtgtctttttTC
SRR959561.68727661.2-    gaccttcaggtgccttgtgtgtgtctttttTC
SRR959560.42879338.1+    gaccttcaggtgccttgtgtgtgtctttttTCCAGTTG
SRR959560.96444595.2-    gaccttcaggtgccttgtgtgtgtctttttTCCAGTTG
SRR959561.15995664.2+    gaccttcaggtgccttgtgtgtgtctttttTCCAGTTGA
SRR959561.8135332.2+     gaccttcaggtgccttgtgtgtgtctttttTCCAGTTGAAGAA
SRR959561.5891527.2-     gaccttcaggtgccttgtgtgtgtctttttTCCAGTTGAAGAAA
SRR959561.14265058.2+    gaccttcaggtgccttgtgtgtgtctttttTCCAGTTGAAGAAACC
SRR959560.32821229.2-    gaccttcaggtgccttgtgtgtgtctttttTCCAGTTGAAGAAACCAA
SRR959561.83604022.1+    gaccttcaggtgccttgtgtgtgtctttttTCCAGTTGAAGAAACCAA
SRR959561.72524179.1+    gaccttcaggtgccttgtgtgtgtctttttTCCAGTTGAAGAAACCACGTGT
SRR959561.7528594.1-     gaccttcaggtgccttgtgtgtgtctttttTCCAGTTGAAGAAACCAAGTGT
SRR959560.88560863.2-    gaccttcaggtgccttgtgtgtgtctttttTCCAGTTGAAGAAACCAAGTGTGCC
SRR959560.104143937.2-   gaccttcaggtgccttgtgtgtgtctttttTCCAGTTGAAGAAACCAAGTGTGCCTGTAC
SRR959560.104246699.2-   gaccttcaggtgccttgtgtgtgtctttttTCCAGTTGAAGAAACCAAGTGTGCCTGTAC
SRR959560.25382432.1+    gaccttcaggtgccttgtgtgtgtctttttTCCAGTTGAAGAAACCAAGTGTGCCCGTAC
SRR959560.33001725.1-    gaccttcaggtgccttgtgtgtgtctttttTCCAGTTGAAGAAACCAAGTGTGCCTGTAC
SRR959560.3935177.2+     gaccttcaggtgccttgtgtgtgtctttttTCCAGTTGAAGAAACCAAGTGTGCCTGTAC
SRR959561.17237439.2+    gaccttcaggtgccttgtgtgtgtctttttTCCAGTTGAAGAAACCAAGTGTGCCTGTAC
SRR959561.30522868.1-    gaccttcaggtgccttgtgtgtgtctttttTCCAGTTGAAGAAACCAAGTGTGCCTGTAC
SRR959561.59165143.1+    gaccttcaggtgccttgtgtgtgtctttttTCCAGTTGAAGAAACCAAGTGTGCCTGTAC
SRR959561.60700224.1+    gaccttcaggtgccttgtgtgtgtctttttTCCAGTTGAAGAAACCAAGTGTGCCTGTAC
SRR959561.71846962.1+    gaccttcaggtgccttgtgtgtgtctttttTCCAGTTGAAGAAACCAAGTGTGCCTGTAC
SRR959561.78830570.1-    gaccttcaggtgccttgtgtgtgtctttttTCCAGTTGAAGAAACCAAGTGTGCCTGTAC
SRR959561.44204103.1-      ccttcaggtgccttgtgtgtgtctttttTCCAGTTGAAGAAACCAAGTGTGCCTGTAC
SRR959561.55982612.2-         tcaggtcccttgtgtgtgtctttttTCCAGTTGAAGAAACCAAGTGCGCATGTAC
SRR959561.70848310.1-           aggtgccttgtgtgtgtctttttTCCAGTTGAAGAAACCAAGTGTGCCTGTAC
SRR959561.35272313.2-                     tgtgtgtctttttTCCAGTTGAAGAAACCAAGTGTGCCTGTAC
SRR959560.68775550.2-                       tgtgtctttttTCCAGTTGAAGAAACCAAGTGTGCCTATAC
SRR959561.30809903.2-                           tctttttTCCAGTTGAAGAAACCAAGTGTGCCTGTAC
SRR959561.44823163.2+                            ctttttTCCAGTTGAAGAAACCAAGTGTGCCTGTAC
SRR959561.13074295.2-                                 tTCCAGTTGAAGAAACCAAGTGTGCCTGTAC
SRR959560.99357060.1+                                    CAGTTGAAGAAACCAAGTGTGCCTGTAC
SRR959561.69030711.2+                                            GAAACCAAGTGTGCCTGTAC
SRR959561.83762742.2-                                            GAAACCAAGTGTGCCTGTAC
SRR959561.17174244.2-                                              AACCAAGTCTGCCTGTAC
SRR959561.59801143.2-                                              AACCAAGTGTGCCTGTAC
SRR959561.6859783.1+                                                            TGTAC
SRR959561.71523271.1+                                                           TGTAC
SRR959561.82583502.1+                                                            GTAC
SRR959561.12787236.2-                                                             TAC
                         ____________________________________________________________
consensus                gaccttcaggtgccttgtgtgtgtctttttTCCAGTTGAAGAAACCAAGTGTGCCTGTAC

                             .    :    .    :    .    :    .    :    .    :    .    :
SRR959561.30522868.1-    C
SRR959561.71846962.1+    CTTTATT
SRR959560.104246699.2-   CTTTATTCCA
SRR959561.78830570.1-    CTTTATTCCACT
SRR959561.60700224.1+    CTTTATTCCACTGCCAAGCAGAGA
SRR959560.33001725.1-    CTTTATTCCACTGCCAAGCAGAGAC
SRR959561.17237439.2+    CTTTATTCCACTGCCAAGCAGAGACAC
SRR959561.59165143.1+    CTTTATTCCACTGCCAAGCAGAGACACC
SRR959560.104143937.2-   CTTTATTCCACTGCCAAGCAGAGACACCACGTC
SRR959561.55982612.2-    CTTTATTCCCCTGCCAAGCAGAGACACCACGTCTACA
SRR959560.25382432.1+    CTTTATTCCACTGCCAAGCAGAGACACCACGTCTACAAGg
SRR959561.44204103.1-    CTTTATTCCACTGCCAAGCAGAGACACCACGTCTACAAGg
SRR959561.70848310.1-    CTTTATTCCACTGCCAAGCAGAGACACCACGTCTACAAGgtaaactt
SRR959561.35272313.2-    CTTTATTCCACTGCCAAGCAGAGACACCACGTCTACAAGgtaaacttgcaggctgta
SRR959560.68775550.2-    CTTTATTCCACTGCCAAGCAGAGACACCACGTCTACAAGgtaaacttgcaggctgtagg
SRR959560.99357060.1+    CTTTATTCCACTGCCAAGCAGAGACACCACGTCTACAAGgtaaacttgcaggctgtagga
SRR959561.12787236.2-    CTTTATTCCACTGCCAAGCAGAGACACCACGTCTACAAGgtaaacttgcaggctgtagga
SRR959561.13074295.2-    CTTTATTCCACTGCCAAGCAGAGACACCACGTCTACAAGgtaaacttgcaggctgtagga
SRR959561.17174244.2-    CTTTATTCCACTGCCAAGCAGAGACACCACGTCTACAAGgtaaacttgcaggctgtagga
SRR959561.30809903.2-    CTTTATTCCACTGCCAAGCAGAGACACCACGTCTACAAGgtaaacttgcaggctgtagga
SRR959561.44823163.2+    CTTTATTCCACTGCCAAGCAGAGACACCACGTCTACAAGgtaaacttgcaggctgtagga
SRR959561.59801143.2-    CTTTATTCCACTGCCAAGCAGAGACTCCACGTCTACAAGgtaaacttgcaggctgtagga
SRR959561.6859783.1+     CTTTATTCCACTGCCAAGCAGAGACACCACGTCTACAAGgtaaacttgcaggctgtagga
SRR959561.69030711.2+    CTTTATTCCACTGCCAAGCAGAGACACCACGTCTACAAGgtaaacttgcaggctgtagga
SRR959561.71523271.1+    CTTTATTCCACTGCCAAGCAGAGACACCACGTCTACAAGgtaaacttgcaggctgtagga
SRR959561.82583502.1+    CTTTATTCCACTGCCAAGCAGAGACACCACGTCTACAAGgtaaacttgcaggctgtagga
SRR959561.83762742.2-    CTTTATTCCACTGCCAAGCAGAGACACCACGTCTACAAGgtaaacttgcaggctgtagga
SRR959560.100142373.1-        TTCCACTGCCAAGCAGAGACACCACGTCTACAAGgtaaacttgcaggctgtagga
SRR959561.60535851.2+                GCCAAGCAGAGACACCACGTCTACAAGgtaaacttgcaggctgtagga
```

```
SRR959560.27242127.2-                       CAAGCAGAGACACCACGTCTACAAGgtaaacttgcaggctgtagga
SRR959560.78888320.2+                                 CACCACGTCTACAAGgtaaacttgcaggctgtagga
SRR959561.21849351.1+                                 CACCACGTCTACAAGgtaaacttgcaggctgtagga
SRR959560.10398746.1-                                             AAGgtaaacttgcaggctgtagga
SRR959561.2716527.1+                                                Ggtaaacttgcaggctgtagga
                         ____________________________________________________________
consensus                CTTTATTCCACTGCCAAGCAGAGACACCACGTCTACAAGgtaaacttgcaggctgtagga
```

S. Red-legged seriema (No. 17), exon 5, WGS data

- CAP3 alignment of WGS data spanning exon 5 (uppercase letters)

```
                             .    :    .    :    .    :    .    :    .    :    .    :
SRR953484.56082989.2+    acaatataattttttcctctttctctttgttacagTTTGAA
SRR953484.2670215.2+     acaatataattttttcctctttctctttgttacagTTTGAAC
SRR953484.43987329.1-    acaatataattttttcctctttctctttgttacagTTTGAACC
SRR953484.76920546.2+    acaatataattttttcctctttctctttgttacagTTTGAACCTGTAA
SRR953484.87165353.1+    acaatataattttttcctctttctctttgttacagTTTGAACCTGTAATAAC
SRR953484.63580834.2+    acaatataattttttcctctttctctttgttacagTTTGAACCTGTAATAACAT
SRR953484.103374428.1+   acaatataattttttcctctttctctttgttacagTTTGAACCTGTAATAACATGCCACA
SRR953484.103456804.1-   acaatataattttttcctctttctctttgttacagTTTGAACCTGTAATAACATGCCACA
SRR953484.13955749.1-    acaatataattttttcctctttctctttgttacagTTTGAACCTGTAATAACATGCCACA
SRR953484.31391392.1-    acaatataattttttcctctttctctttgttacagTTTGAACCTGTAATAACATGCCACA
SRR953484.35891395.2-    acaatataattttttcctctttctctttgttacagTTTGAACCTGTAATAACATGCCACA
SRR953484.5876207.1+     acaatataattttttcctctttctctttgttacagTTTGAACCTGTAATAACATGCCACA
SRR953484.62650990.1-    acaatataattttttcctctttctctttgttacagTTTGAACCTGTAATAACATGCCACA
SRR953484.63076673.1-    acaatataattttttcctctttctctttgttacagTTTGAACCTGTAATAACATGCCACA
SRR953484.743857.1-      acaatataattttttcctctttctctttgttacagTTTGAACCTGTAATAACATGCCACA
SRR953484.874395.1-      acaatataattttttcctctttctctttgttacagTTTGAACCTGTAATAACATGCCACA
SRR953484.89126903.1-    acaatataattttttcctctttctctttgttacagTTTGAACCTGTAATAACATGCCACA
SRR953484.994219.2-      acaatataattttttcctctttctctttgttacagTTTGAACCTGTAATAACATGCCACA
SRR953484.1898976.1-            aattttttcctctttctctttgttacagTTTGAACCTGTAATAACATGCCACA
SRR953484.84252095.1+              tttttcctctttctctttgttacagTTTGAACCTGTAATAACATGCCACA
SRR953484.25615613.1-                 ttcctctttctctttgttacagTTTGAACCTGTAATAACATGCCACA
SRR953484.99885232.2+                              ttgttacagTTTGAACCTGTAATAACATGCCACA
SRR953484.32861852.2+                                              CTGTAATAACATGCCACA
SRR953484.26903530.1+                                                   ATAACATGCCACA
SRR953484.24149491.2+                                                             ACA
SRR953484.51109390.1+                                                               A
SRR953484.86859914.1+                                                               A
                         ____________________________________________________________
consensus                acaatataattttttcctctttctctttgttacagTTTGAACCTGTAATAACATGCCACA

                             .    :    .    :    .    :    .    :    .    :    .    :
SRR953484.89126903.1-    AC
SRR953484.13955749.1-    ACATCACCTTGG
SRR953484.35891395.2-    ACATCACCTTGGG
SRR953484.103374428.1+   ACATCACCTTGGGTCATC
SRR953484.994219.2-      ACATCACCTTGGGTCATCATA
SRR953484.63076673.1-    ACATCACCTTGGGTCATCATATTCTTG
SRR953484.31391392.1-    ACATCACCTTGGGTCATCATATTCTTGTTTATG
SRR953484.103456804.1-   ACATCACCTTGGGTCATCATATTCTTGTTTATGC
SRR953484.5876207.1+     ACATCACCTTGGGTCATCATATTCTTGTTTATGCCT
SRR953484.874395.1-      ACATCACCTTGGGTCATCATATTCTTGTTTATGCCT
SRR953484.62650990.1-    ACATCACCTTGGGTCATCATATTCTTGTTTATGCCTGT
SRR953484.1898976.1-     ACATCACCTTGGGTCATCATATTCTTGTTTATGCCTGTGGCAACGCC
SRR953484.84252095.1+    ACATCACCTTGGGTCATCATATTCTTGTTTATGCCTGTGGCAACGCCAGC
SRR953484.25615613.1-    ACATCACCTTGGGTCATCATATTCTTGTTTATGCCTGTGGCAACGCCAGCGTG
SRR953484.24149491.2+    ACATCACCTTGGGTCATCATATTCTTGTTTATGCCTGTGGCAACGCAAGCGTGTTACCCA
SRR953484.26903530.1+    ACATCACCTTGGGTCATCATATTCTTGTTTATGCCTGTGGCAACGCCAGCGTGTTACCCA
SRR953484.32861852.2+    ACATCACCTTGGGTCATCATATTCTTGTTTATGCCTGTGGCAACGCCAGCGTGTTACCCA
SRR953484.51109390.1+    ACATCACCTTGGGTCATCATATTCTTGTTTATGCCTGTGGCAACGCCAGCGTGTTACCCA
SRR953484.86859914.1+    ACATCACCTTGGGTCATCATATTCTTGTTTATGCCTGTGGCAACGCCAGCGTGTTACCCA
SRR953484.99885232.2+    ACATCACCTTGGGTCATCATATTCTTGTTTATGCCTGTGGCAACGCCAGCGTGTTACCCA
SRR953484.93961221.1-          CCTTGGGTCATCATATTCTTTTTTATGCCTGTGGCAACGCCAGCGTGTTACCCA
SRR953484.61212684.2-                                TTATGCCTGTGGCAACGCCAGCGTGTTACCCA
SRR953484.22039022.1+                                  ATGCCTGTGGCAACGCCAGCGTGTTACCCA
SRR953484.24892395.1+                                   TGCCTGTGGCAACGCCAGCGTGTTACCCA
SRR953484.25326404.1+                                             AACGCCAGCGTGTTACCCA
SRR953484.55616818.1+                                                  CAGCGTGTTACCCA
SRR953484.716568.2+                                                      GCGTGTTACCCA
SRR953484.11216633.1+                                                      GTGTTACCCA
                         ____________________________________________________________
consensus                ACATCACCTTGGGTCATCATATTCTTGTTTATGCCTGTGGCAACGCCAGCGTGTTACCCA

                             .    :    .    :    .    :    .    :    .    :    .    :
SRR953484.99885232.2+    GTGGCA
SRR953484.32861852.2+    GTGGCATAGACGATTGCTATGG
SRR953484.26903530.1+    GCGGCATAGACGATTGCTATGGAG
SRR953484.24149491.2+    GTGGCATAGACGATTGCTACGGAGCCAAGC
SRR953484.51109390.1+    GTGGCATAGACGATTGCTATGGAGCCAAGCCAGATTTTG
SRR953484.86859914.1+    GTGGCATAGACGATTGCTATGGAGCCAAGCCAGATTTTG
SRR953484.93961221.1-    GTGGCATAGACGATTGCTATGGAGCCAAGCCAGATTTTGCCCTGT
SRR953484.11216633.1+    GTGGCATAGACGATTGCTATGGAGCCAAGCCAGATTTTGCCCTGTGCTGTCAGGTGCTTG
SRR953484.22039022.1+    GGGGCATAGACGATTGCTATGGAGCCAAGCCAGATTTTGCCCTGTGCTGTCCGGTGCTTG
SRR953484.24892395.1+    GTGGCATAGACGATTGCTATGGAGCCAAGCCAGATTTTGCCCTGTGCTGTCAGGTGCTTG
SRR953484.25326404.1+    GTGGCATAGACGATTGCTATGGAGCCAAGCCAGATTTTGCCCTGTGCTGTCAGGTGCTTG
SRR953484.55616818.1+    GTGGCATAGACGATTGCTATGGAGCCAAGCCAGATTTTGCCCTGTGCTGTCAGGTGCTTG
```

```
SRR953484.61212684.2-    GTGGCATAGACGATTGCTATGGAGCCAAGCCAGATTTTGCCCTGTGCTGTCAGGTGCTTG
SRR953484.716568.2+      GTGGCATAGACGATTGCTATGGAGCCAAGCCAGATTTTGCCCTGTGCTGTCAGGTGCTTG
SRR953484.12255125.2+       GCATAGACGATTGCTATGGAGCCAAGCCAGATTTTGCCCTGTGCTGTCAGGTGCTTG
SRR953484.7565276.1-          ATAGACGATTGCTATGGAGCCAAGCCAGATTTTGCCCTGTGCTGTCAGGTGCTTG
SRR953484.43309622.2-             ACGATTGCTATGGAGCCAAGCCAGATTTTGCCCTGTGCTGTCAGGTGCTTG
SRR953484.54550992.1-                                       AGATTTGGCCCTGTGCTGTCAGGTGCTTG
SRR953484.76925770.1+                                          TTTTGCCCTGTGCTGTCAGGTGCTTG
SRR953484.95361795.1-                                                 CTGTGCTGTCAGGGGCTTG
SRR953484.20740787.1+                                                   GTGCTGTCAGGTGCTTG
SRR953484.99666728.1+                                                                 TTG
                         ____________________________________________________________
consensus                GTGGCATAGACGATTGCTATGGAGCCAAGCCAGATTTTGCCCTGTGCTGTCAGGTGCTTG

                             .    :    .    :    .    :    .    :    .    :    .    :
SRR953484.61212684.2-    TGGGCTGA
SRR953484.22039022.1+    TGGGCTGAGG
SRR953484.24892395.1+    TGGGCTGAGGG
SRR953484.25326404.1+    TGGGCTGAGGGACTGGAGGAG
SRR953484.55616818.1+    TGGGCTGAGGGACTGGAGGCGAGgtg
SRR953484.11216633.1+    TGGGCTGAGGGACTGGAGGAGAGgtgag
SRR953484.716568.2+      TGGGCTGAGGGACTGGAGGAGAGgtgag
SRR953484.7565276.1-     TGGGCTGAGGGACTGGAGGAGAGgtgagtctgcagaac
SRR953484.12255125.2+    TGGGCTGAGGGACTGGAGGAGAGgtgagtctgcagaacccatt
SRR953484.43309622.2-    TGGGCTGAGGGACTGGAGGAGAGgtgagtctgcagaacccattcccttc
SRR953484.20740787.1+    TGGGCTGAGGGACTGGAGGAGAGgtgagtctgcagaacccctttccttccccatttcttg
SRR953484.54550992.1-    TGGGCTGAGGGACTGGAGGAGAGgtgagtctgcagaacccattcccttccccatttcttg
SRR953484.76925770.1+    TGGGCTGAGGGACTGGAGGAGAGgtgagtctgcagaactcattcccttccccatttcttg
SRR953484.95361795.1-    TGGGCTGAGGGACTGGAGGAGAGgtgagtctgcagaacccattcccttccccatttcttg
SRR953484.99666728.1+    TGGGCTGAGGGACTGGAGGAGAGgtgagtctgcagaactcattcccttccccatttcttg
SRR953484.61172402.1-              GACTGGAGGAGAGgtgagtctgcagaacccattcccttccccatttcttg
SRR953484.64526699.2-              GACTGGAGGAGAGgtgagtctgcagaacccattcccttccccatttcttg
SRR953484.19714373.2-               ACTGGAGGAGAGgtgagtctgcagaacccattcccttccccatttcttg
SRR953484.73162379.2+                      GAGAGgtgagtctgcagaacccattcccttccccatttcttg
                         ____________________________________________________________
consensus                TGGGCTGAGGGACTGGAGGAGAGgtgagtctgcagaacccattcccttccccatttcttg
```

T. Bar-tailed trogon (No. 21), exon 5, WGS data

- CAP3 alignment of WGS data spanning exon 5 (uppercase letters)

```
                             .    :    .    :    .    :    .    :    .    :    .    :
SRR952777.74223757.2-    caataactttctctgactctttctcttggtttcagTTCGA
SRR952778.45607761.1-    caataactttctctgactctttctcttggtttcagTTCGAACCTGTA
SRR952777.4814668.2-     caataactttctctgactctttgtcttggtttcagTTCGAACCTGTACT
SRR952777.61489167.2+    caataactttctctgactctttctcttggtttcagTTCGAAGCTGTACTAACACC
SRR952778.66141715.1+    caataactttctctgactctttctcttggtttcagTTCGAACCTGTACTAACACCCC
SRR952777.10318742.1+    caataactttctctgactctttctcttggtttcagTTCGAACCTGTACTAACACCCCACA
SRR952777.113115601.2+   caataactttctctgactctttctcttggtttcagTTCGAACCTGTACTAACACCCCACA
SRR952777.1457968.2+     caataactttctctgactctttctcttggtttcagTTCGAACCTGTACTAACACCCCACA
SRR952777.21098462.1+    caataactttctctgactctttctcttggtttcagTTCGAACCTGTACTAACACCCCACA
SRR952777.24895342.1+    caataactttctctgactctttctcttggtttcagTTCGAACCTGTACTAACACCCCACA
SRR952777.28307119.1+    caataactttctctgactctttctcttggtttcagTTCGAACCTGTACTAACACCCCACA
SRR952777.30497011.1+    caataactttctctgactctttctcttggtttcagTTCGAACCTGTACTAACACCCCACA
SRR952777.30627853.1-    caataactttctctgactctttctcttggtttcagTTCGAACCTGTACTAACACCCCACA
SRR952777.47084385.1-    caataactttctctgactctttctcttggtttcagTTCGAACCTGTACTAACACCCCACA
SRR952777.60916061.1+    caataactttctctgactctttctcttggtttcagTTCGAACCTGTACTAACACCCCACA
SRR952777.67888612.2-    caataactttctctgactctttctcttggtttcagTTCGAACCTGTACTAACACCCCACA
SRR952777.83112181.1-    caataactttctctgactctttctcttggtttcagTTCGAACCTGTACTAACACCCCACA
SRR952777.85488194.1+    caataactttctctgactctttctcttggtttcagTTCGAACCTGTACTAACACCCCACA
SRR952777.93243186.2-    caataactttctctgactctttctcttggtttcagTTCGAACCTGTACTAACACCCCACA
SRR952778.11621171.2+    caataactttctctgactctttctcttggtttcagTTCGAACCTGTACTAACACCCCACA
SRR952778.30775244.1-    caataactttctctgactctttctcttggtttcagTTCGAACCTGTACTAACACCCCACA
SRR952778.31779816.2+    caataactttctctgactctttctcttggtttcagTTCGAACCTGTACTAACACCCCACA
SRR952778.36886304.2-    caataactttctctgactctttctcttggtttcagTTCGAACCTGTACTAACACCCCACA
SRR952778.48112104.1-    caataactttctctgactctttctcttggtttcagTTCGAACCTGTACTAACACCCCACA
SRR952778.77469570.1+    caataactttctctgactctttctcttggtttcagTTCGAACCTGTACTAACACCCCACA
SRR952778.82883359.1+    caataactttctctgactctttctcttggtttcagTTCGAACCTGTACTAACACCCCACA
SRR952777.28908522.2+     aataactttctctgactctttctcttggtttcagTTCGAACCTGTACTAACACCCCACA
SRR952778.8546482.2+      aataactttctctgactctttctcttggtttcagTTCGAACCTGTACTAACACCCCACA
SRR952777.83634941.1+      ataactttctctgactctttctcttggtttcagTTCGAACCTGTACTAACACCCCACA
SRR952777.93359160.2+       taactttctctgactctttctcttggtttcagTTCGAACCTGTACTAACACCCCACA
SRR952777.93509890.2+           tttctctgactctttctcttggtttcagTTCGAACCTGTACTAACACCCCACA
SRR952777.27931091.2+            ttctctgactctttctcttggtttcagTTCGAACCTGTACTAACACCCCACA
SRR952778.37452001.1+                  gactctttctcttggtttcagTTCGAACCTGTACTAACACCCCACA
SRR952777.23555206.1+                    ctctttctcttggtttcagTTCGAACCTGTACTAACACCCCACA
SRR952778.15662083.1-                    ctctttctcttggtttcagTTCGAACCTGTACTAACACCCCACA
SRR952777.93624234.1+                     tctttctcttggtttcagTTCGAACCTGTACTAACACCCCACA
SRR952778.22960022.1-                           tcttggtttcagTTCGAACCTGTACTAACACCCCACA
SRR952777.60429857.1-                             ttggtttcagTTCGAACCTGTACTAACACCCCACA
SRR952778.33893944.1-                                   tcagTTCGAACCTGTACTAACACCCCACA
SRR952777.2496180.1+                                     cagTTCGAACCTGTACTAACACCCCACA
SRR952777.12672881.2-                                         CGAACCTGTACTAACACCCCACA
SRR952778.62759729.1-                                           AACCTGTACTAACACCCCACA
SRR952777.2168053.2-                                               CTGTACTAACACCCCACA
SRR952778.21008811.1+                                              CTGTACTAACACCCCACA
SRR952778.38439500.2-                                              CTGTACTAACACCCCACA
SRR952777.7410444.2+                                                 GTACTAACACCCCACA
SRR952778.67977382.1-                                                  ACTAACACCCCACA
SRR952778.59682180.1+                                                     AACACCCCACA
SRR952778.5960998.1+                                                       ACACCCCACA
SRR952777.50947988.1+                                                       CACCCCACA
SRR952778.74260928.1-                                                        ACCCCACA
SRR952777.16671753.2-                                                               A
                         ____________________________________________________________
consensus                caataactttctctgactctttctcttggtttcagTTCGAACCTGTACTAACACCCCACA

                             .    :    .    :    .    :    .    :    .    :    .    :
SRR952777.10318742.1+    ACA
SRR952777.21098462.1+    ACA
SRR952777.24895342.1+    ACAT
SRR952778.31779816.2+    ACAT
SRR952777.30627853.1-    ACATCACC
SRR952777.47084385.1-    ACATCACC
SRR952778.82883359.1+    ACATCACCTTGGTTC
SRR952778.30775244.1-    ACATCACCTTGGTTCATCAC
SRR952777.28307119.1+    ACATCACATTGGTTCATCACA
SRR952777.60916061.1+    ACATCACCTTGGTTCATCACA
SRR952778.11621171.2+    ACATCACCTTGGTTCATCACA
SRR952778.48112104.1-    ACATCACCTTGGTTCATCACATTCT
SRR952777.93243186.2-    ACATCACCTTGGTTCATCACATTCTTGTTT
SRR952777.83112181.1-    ACATCACCTTGGTTCATCACATTCTTGTTTATGCCT
SRR952777.85488194.1+    ACATCACCTTGGTTCATCACATTCTTGTTTATGCCTG
SRR952778.77469570.1+    ACATCACCTTGGTTCATCACATTCTTGTTTATGCCTG
SRR952777.113115601.2+   ACATCACCTTGGTTCATCACATTCTTGTTTATGCCTGCG
SRR952778.36886304.2-    ACATCACCTTGGTTCATCACATTCTTGTTTATGCCTGCG
SRR952777.1457968.2+     ACATCACCTTGGTTCATCACATTCTTGTTTATGCCTGCGG
```

```
SRR952777.30497011.1+    ACATCACCTTGGTTCATCACATTCTTGTTTATGCCTGCGG
SRR952777.28908522.2+    ACATCACCTTGGTTCATCACATTCTTGTTTATGCCTGCGGC
SRR952778.8546482.2+     ACATCACCTTGGTTCATCACATTCTTGTTTATGACTGCGGC
SRR952777.83634941.1+    ACATCACCTTGGTTCATCACATTCTTGTTTATGCCTGCGGCA
SRR952777.93359160.2+    ACATCACCTTGGTTCATCACATTCTTGTTTATGCCTGCGGCAG
SRR952777.93509890.2+    ACATCACCTTGGTTCATCACATTCTTGTTTATGCCTGCGGCAG
SRR952777.23555206.1+    ACATCACCTTGGTTCATCACATTCTTGTTTATGCCTGCGGCAGCT
SRR952778.37452001.1+    ACATCACCTTGGTTCATCACATTCTTGTTTATGCCTGCGGCAGCTCT
SRR952777.27931091.2+    ACATCACCTTGGTTCATCACATTCTTGTTTATGCCTGCGGCAGCTCTG
SRR952777.93624234.1+    ACATCACCTTGGTTCATCACATTCTTGTTTATGCCTGCGGCAGCTCTGG
SRR952777.2496180.1+     ACATCACCTTGGTTCATCACATTCTTGTTTATGCCTGCGGCAGGGCTGGCGTG
SRR952778.15662083.1-    ACATCACCTTGGTTCATCACATTCTTGTTTATGCCTGCGGCAGCTCTGGCGTGTTG
SRR952777.12672881.2-    ACATCACCTTGGTTCATCACATTCTTGTTTATGCCTGCGGCAGCTCTGGCGTGTTGCCCA
SRR952777.16671753.2-    ACATCTCCTTTGTTCATCACATTCTTGTTTATGCCTGCGGCAGCTCTGGCGTGTTGCCTA
SRR952777.2168053.2-     ACATCACCTTGGTTCATCACATTCTTGTTTATGCCTGCGGCAGCTCTGGCGTGTTGCCCA
SRR952777.50947988.1+    ACATCACCTTGGTTCATCACATTCTTGTTTATGCCTGCGGCAGCTCTGGCGTGTTGCCCA
SRR952777.60429857.1-    ACATCACCTTGGTTCATCACATTCTTGTTTATGCCTGCGGCAGCTCTGGCGTGTTGCCCA
SRR952777.7410444.2+     ACATCACCTTGGTTCATCACATTCTTGTTTATGCCTGCGGCAGCTCTGGCGTGTTGCCCC
SRR952778.21008811.1+    ACATCACCTTGGTTCATCACATTCTTGTTTATGCCTGCGGCAGCTCTCGCGTGTTGCCCA
SRR952778.22960022.1-    ACATCACCTTGGTTCATCACATTCTTGTTTATGCCTGCGGCAGCTCTGGCGTGTTGCCCA
SRR952778.33893944.1-    ACATCACCTTGGTTCATCACATTCTTGTTTATGCCTGCGGCAGCTCTGGCGTGTTGCCCA
SRR952778.38439500.2-    ACATCACCTTGGTTCATCACATTCTTGTTTATGCCTGCGGCAGCTCTGGCGTGTTGCCCA
SRR952778.5960998.1+     ACATCACCTTGGTTCATCACATTCTTGTTTATGCCTGCGGCAGCTCTGGCGTGTTGCCCA
SRR952778.59682180.1+    ACATCACCTTGGTTCATCACATTCTTGTTTATGCCTGCGGCAGCTCTGGCGTGTTGCCCA
SRR952778.62759729.1-    ACATCACCTTGGTTCATCACATTCTTGTTTATGCCTGCGGCAGCTCTGGCGTGTTGCCCA
SRR952778.67977382.1-    ACATCACCTTGGTTCATCACATTCTTGTTTATGCCTGCGGCAGCTCTGGCGTGTTGCCCA
SRR952778.74260928.1-    ACATCACCTTGGTTCATCACATTCTTGTTTATGCCTGCGGCAGCTCTGGCGTGTTGCCCA
SRR952777.73164985.1+               GTTCATCACATTCTTGTTTATGCCTGCGGCAGCTCTGGCGTGTTGCCCA
SRR952778.10783276.1+                     CACATTCTTGTTTATGCCTGCGGCAGCTCTGGCGTGTTGCCCA
SRR952777.67292835.2+                              GTTTATGCCTGCGGCAGCTCTGGCGGGTTGCCCA
SRR952777.11786317.1-                                 TATGCCTGCTGCCGCTCTGGCGTGTTGCCCA
SRR952778.30117750.1+                                 TATGCCTGCGGCAGCTCTGGCGGGTTGCCCA
SRR952778.38155861.2+                                 TATGCCTGCGGCAGCTCTGGCGTGTTGCCCA
SRR952778.74642814.1+                                 TATGCCTGCGGCAGCTCTGGCGTGTTGCCCA
SRR952778.18375199.2-                                        GCGGCAGCTCTGGCGTGTTGCCCA
SRR952777.112177493.1-                                           CAGCTCTGGCGTGTTGCCCA
SRR952778.80070047.2-                                            CAGCTCTGGCGGGTTGCCCA
SRR952777.56874871.2+                                                TCTGGCGGGTTGCCCA
SRR952777.68296271.2+                                                TCTGGCGTGTTGCCCA
SRR952777.7079840.2-                                                  CTGGCGTGTTGCCCA
                         ____________________________________________________________
consensus                ACATCACCTTGGTTCATCACATTCTTGTTTATGCCTGCGGCAGCTCTGGCGTGTTGCCCA

                             .    :    .    :    .    :    .    :    .    :    .    :
SRR952778.22960022.1-    GTG
SRR952777.60429857.1-    GTGGC
SRR952778.33893944.1-    GTGGCACAGAT
SRR952777.12672881.2-    GTGGCACAGATGATTGC
SRR952778.62759729.1-    GTGGCACAGATGATTGCTA
SRR952777.2168053.2-     GTGGCACAGATGATTGCTATGG
SRR952777.7410444.2+     GTGGCACAGATGATTGCTATGG
SRR952778.21008811.1+    GGGGCACAGATGATTGCTATGG
SRR952778.38439500.2-    GTGGCACAGATGATTGCTATGG
SRR952778.67977382.1-    GTGGCACAGATGATTGCTATGGAGCC
SRR952778.5960998.1+     GTGGCACAGATGATTGCTATGGAGCCAAC
SRR952778.59682180.1+    GTGGCACAGATGATTGCTATGGAGCCAAC
SRR952777.50947988.1+    GTGGCACAGATGATTGCTATGGGGCCAACTC
SRR952778.74260928.1-    GTGGCACAGATGATTGCTATGGAGCCAACTCA
SRR952777.16671753.2-    GTGGCACAGATGATTGCTATGGAGCCAACTCAGATTTTA
SRR952777.73164985.1+    GTGGCACAGATGATTGCTTTGGAGCCAACTCAGATTTTTCCCTCCGATTAG
SRR952778.10783276.1+    GTGGCACAGATGATTGCTATGGAGCCAACTCAGATTTTACCCTCAGATTAGTGCTCT
SRR952777.112177493.1-   GTGGCACAGATGATTGCTATGGAGCCAACTCAGATTTTACCCTCAGATTAGTGCTCTCAG
SRR952777.11786317.1-    GTGGCTCAGATGATTGCTATGGAGCCAACTCAGATTTTACCCTCAGATTAGTGCTCTCAG
SRR952777.56874871.2+    GTGGCACAGATGATTGCTATGGAGCCAACTCAGATTTTACCCTCAGATTAGTGCTCTCAG
SRR952777.67292835.2+    GTGGCACAGATGATTGCTATGGAGCCAACTCAGATTTTACCCTCAGATTAGTGCTCTCCG
SRR952777.68296271.2+    GTGGCACAGATGATTGCTATGGAGCCAACTCAGATTTTACCCTCAGATTAGTGCTCTCAG
SRR952777.7079840.2-     GTGGCACAGATGATTGCTATGGAGCCAACTCAGATTTTACCCTCAGATTAGTGCTCTCAG
SRR952778.18375199.2-    GTGGCACAGATGATTGCTATGGAGCCAACTCAGATTTTACCCTCAGATTAGTGCTCTCAG
SRR952778.30117750.1+    GTGGCACAGATGATTGCTATGGAGCCAACTCAGATTTTACCCTCAGATTAGTGCTCTCAG
SRR952778.38155861.2+    GTGGCACAGATGATTGCTATGGAGCCAACTCAGATTTTACCCTCAGATTAGTGCTCTCCG
SRR952778.74642814.1+    GTGGCACAGATGATTGCTATGGAGCCAACTCAGATTTTACCCTCAGATTAGTGCTCTCAG
SRR952778.80070047.2-    GTGGCACAGATGATTGCTATGGAGCCAACTCAGATTTTACCCTCAGATTAGTGCTCTCAG
SRR952777.24575501.1+         ACAGATGATTGCTATGGAGCCAACTCAGATTTTACCCTCAGATTAGTGCTCTCAG
SRR952777.89897686.1+          CAGATGATTGCTATGGAGCCAACTCAGATTTTACCCTCAGATTAGTGCTCTCAG
SRR952777.53826315.1-             ATGATTGCTATGGAGCCAACTCAGATTTTACCCTCAGATTAGTGCTCTCAG
SRR952778.13472182.2+             ATGATTGCTATGGAGCCAACTCAGATTTTACCCTCAGATTAGTGCTCTCAG
SRR952778.43025515.1-                    CTATGGAGCCAACTCAGATTTTACCCTCAGATTAGTGCTCTCAG
SRR952778.29943706.1+                           GCCAACTCAGATTTTACCCTCAGATTAGTGCTCTCAG
SRR952777.18016292.1+                                 TCAGATTTTACCCTCAGATTAGTGCTCTCAG
SRR952777.84254921.1-                                  CAGATTTTACCCTCAGATTAGTGCTCTCAG
SRR952778.71588172.1-                                     ATTTTACCCTCAGATTAGTGCTCTCAG
SRR952777.107530151.1-                                         ACCCTCAGATTAGTGCTCTCAG
SRR952777.94376434.2-                                          ACCCTCAGATTAGTGCTCTCAG
SRR952778.8146226.2+                                           ACCCTCAGATTAGTGCTCTCAG
```

```
SRR952778.42593876.2+                                                  CCCTCAGATTAGTGCTCTCAG
SRR952777.7602259.1-                                                              GTGCTCTCAG
SRR952777.15582380.1+                                                                CTCTCAG
SRR952778.5305589.1-                                                                   CTCAG
                         ____________________________________________________________
consensus                GTGGCACAGATGATTGCTATGGAGCCAACTCAGATTTTACCCTCAGATTAGTGCTCTCAG

                             .    :    .    :    .    :    .    :    .    :    .    :
SRR952777.67292835.2+    GTTCTT
SRR952777.11786317.1-    GTTCTTGTG
SRR952778.30117750.1+    GGTCTTGTG
SRR952778.38155861.2+    GTTCTTGTG
SRR952778.74642814.1+    GTTCTTGTG
SRR952778.18375199.2-    GTTCTTGTGGGCTGGG
SRR952777.112177493.1-   GTTCTTGTGGGCTGGGCTGT
SRR952778.80070047.2-    GTTCTTGTGGGCTGGGCTGT
SRR952777.7079840.2-     GTTCTTGTGGGCTGGGCTGTT
SRR952777.56874871.2+    GTTCTTGTGGGCCGGGCTGTTGGG
SRR952777.68296271.2+    GTTCTTGTGGGCTGGGCTGTTGGG
SRR952777.24575501.1+    GTTCTTGTGGGCTGGGCTGTTGGGAGAGAGgtgaatctgtggggc
SRR952777.89897686.1+    GTTCTTGTGGGCTGGGCTGTTGGGAGAGAGgtgaatctgtggggcc
SRR952777.53826315.1-    GTTCTTGTGGGCTGGGCTGTTGGGAGAGAGgtgaatctgtggggcccat
SRR952778.13472182.2+    GTTCTTGTGGGCTGGGCTGTTGGGAGAGAGgtggattcgtggggcccat
SRR952778.43025515.1-    GTTCTTGTGGGCTGGGCTGTTGGGAGAGAGgtgaatctgtggggcccattcctttc
SRR952778.29943706.1+    GTTCTTGTGGGCTGGGCTGTTGGGAGAGAGgtgaatctgtggggcccattcctttctcc
SRR952777.107530151.1-   GTTCTTGTGGGCTGGGCTGTTGGGAGAGAGgtgaatctgtggggcccattcctttctcca
SRR952777.15582380.1+    GTTCTTGTGGGCTGGGCTGTTGGGAGAGAGgtgaatctgtggggcccattcctttctcca
SRR952777.18016292.1+    GTTCTTGTGGGCTGGGCTGTTGGGAGAGAGgtgaatctgtggggcccattcctttctcca
SRR952777.7602259.1-     GTTCTTGTGGGCTGGGCTGTTGGGAGAGAGgtgaatctgtggggcccattcctttctcca
SRR952777.84254921.1-    GTTCTTGTGGGCTGGGCTGTTGGGAGAGAGgtgaatctgtggggcccattcctttctcca
SRR952777.94376434.2-    GTTCTTGTGGGCTGGGCTGTTGGGAGAGAGgtgaatctgtggggcccattcctttctcca
SRR952778.42593876.2+    GTTCTTGTGGGCTGGGCTGTTGGGAGAGAGgtgaatctgtggggcccattcctttctcca
SRR952778.5305589.1-     GTTCTTGTGGGCTGGGCTGTTGGGAGAGAGgtgaatctgtggggcccattcctttctcca
SRR952778.71588172.1-    GTTCTTGTGGGCTGGGCTGTTGGGAGAGAGgtgaatctgtggggcccattcctttctcca
SRR952778.8146226.2+     GTTCTTGTGGGCTGGGCTGTTGGGAGAGAGgtgaatctgtggggcccattcctttctcca
SRR952777.6849640.2-                       TTGGGAGAGAGgtgaatctgtggggcccattcctttctcca
SRR952777.80203344.2-                          GAGAGAGgtgaatctgtcgggaccattcctttctcca
SRR952777.45019358.2-                           AGAGAGgtgaatctgtggggcccattccttgctcca
SRR952778.71777767.2-                           AGAGAGgtgaatctgtggggcccattcctttctcca
SRR952777.55407209.1-                             AGAGgtgaatctgtggggcccattcctttctcca
SRR952777.57063418.2+                               AGgtgaatctgtggggcccattcctttctcca
                         ____________________________________________________________
consensus                GTTCTTGTGGGCTGGGCTGTTGGGAGAGAGgtgaatctgtggggcccattcctttctcca
```

U. Rhinoceros hornbill (No. 20), exon 7, WGS data

- CAP3 alignment of WGS data spanning exon 7 (uppercase letters)

```
                             .    :    .    :    .    :    .    :    .    :    .    :
SRR952907.12398064.2-    tatgtgttgttacttcagaccccagttctgatgtttctggagtcattgctgaggtaatat
SRR952906.91064646.1-        tgttgttacttcagaccccagttctgatgtttctggagtcattgctgaggtaatat
SRR952907.36862030.1+        tgttgttacttcagaccccagttctgatgtttctggagtcattgctgaggtaatat
SRR952907.4284782.2-         tgttgttacttcagaccccagttctgatgtttctggagtcattgctgaggtaatat
SRR952908.80485683.1-        tgttgttacttcagaccccagttctgatgtttctggagtcattgctgaggtaatat
SRR952908.21742923.2-              tacttcagaccccagttctgatgtttctggagtcattgctgaggtaatat
SRR952906.2764250.1-                acttcagacccccgtgctgatgtttctggagtcattgctgaggtaatat
SRR952906.41008395.1+               acttcagaccccagttctgatgtttctggagtcattgctgaggtaatat
SRR952908.37143756.2+               acttcagaccccagttctgatgtttctggagtcattgctgaggtaatat
SRR952908.61956966.2+                     gaccccagttctgatgtttctggagtcattgctgaggtaatat
SRR952906.84150441.1+                      accccagttctgatgtttctggagtcattgctgaggtaatat
SRR952907.49893402.2-                         ccagttctgatgtttctggagtcattgctgaggtaatat
SRR952908.26607298.1-                            gttctgatgtttctggagtcattgctgaggtaatat
SRR952906.80718732.1-                             ttctgatgtttctggagtcattgctgaggtaatat
SRR952908.21402763.1-                                 gatgtttctggagtcattgctgaggtaatat
SRR952906.59429530.1-                                  atgtttctggagtcattgctgaggtaatat
SRR952906.15561415.1-                                   tgtttctggagtcattgctgaggtaatat
SRR952906.42235225.1-                                   tgtttctggagtcattgctgaggtaatat
SRR952906.46344914.2-                                     tttctggagtcattgcggaggtaatat
SRR952907.52668539.1-                                        ctggagtcattgctgaggtaatat
SRR952908.44612500.2-                                        ctggagtcattgctgaggtaatat
SRR952908.7449415.1-                                         ctggagtcattgctgaggtaatat
SRR952907.35637368.2-                                               cattgctgaggtaatat
SRR952908.83232541.2-                                                 ttgctgaggtaatat
SRR952907.60638876.2+                                                  tgctgaggtaatat
SRR952907.14433590.2+                                                     tgaggtaatat
SRR952908.73514971.1-                                                      gaggtaatat
SRR952908.3285260.1+                                                          gtaatat
SRR952908.67808806.2-                                                         gtaatat
SRR952908.30780511.2+                                                           aatat
SRR952907.42027886.1-                                                               t
                         ____________________________________________________________
consensus                tatgtgttgttacttcagaccccagttctgatgtttctggagtcattgctgaggtaatat

                             .    :    .    :    .    :    .    :    .    :    .    :
SRR952907.12398064.2-    ttcttttacagCCTTGATTGACAGCTCAGGGGTACGAATC
SRR952907.36862030.1+    ttcttttacagCCTTGATTGACAGCTCAGGGGTACGAATCTAC
SRR952906.91064646.1-    ttcttttacagCCTTGATTGACAGCTCAGGGGTACGAATCTACT
SRR952907.4284782.2-     ttcttttacagCCTTGATTGACAGCTCAGGGGTACGAATCTACT
SRR952908.80485683.1-    ttcttttacagCCTTGATTGACAGCTCAGGGGTACGAATCTACT
SRR952908.21742923.2-    ttcttttacagCCTTGATTGACAGCTCAGGGGTACGAATCTGCTATACTC
SRR952906.2764250.1-     ttcttttacagCCTTGATTGACAGCTCAGGGGTACGAATCTACTATACTCC
SRR952906.41008395.1+    ttcttttacagCCTTGATTGACAGCTCAGGGGTACGAATCTACTATACTCC
SRR952906.46344914.2-    ttcttttacagCCTTGATTGACAGCTCAGGGGTACGAATCTACTATACTCC
SRR952908.37143756.2+    ttcttttacagCCTTGATTGACAGCTCAGGGGTACGAATCTACTATACTCC
SRR952908.61956966.2+    ttcttttacagCCTTGATTGACAGCTCAGGGGTACGAATCTACTATACTCCGGAGAT
SRR952906.84150441.1+    ttcttttacagCCTTGATTGACAGCTCAGGGGTACGAATCTACTATACTCCGGAGATA
SRR952906.15561415.1-    ttcttttacagCCTTGATTGACAGCTCAGGGGTACGAATCTACTATACTCCGGAGATACG
SRR952906.42235225.1-    ttcttttacagCCTTGATTGACAGCTCAGGGGTACGAATCTACTATACTCCGGAGATACG
SRR952906.59429530.1-    tgcttttacagCCTTGATTGACAGCTCAGGGGTACGAATCTACTATACTCCGGAGATACG
SRR952906.80718732.1-    ttcttttacagCCTTGATTGACAGCTCAGGGGTACGAATCTACTATACTCCGGAGATACG
SRR952907.14433590.2+    ttcttttacagCCTTGATTGACAGCTCAGGGGTACGAATCTACTATACTCCGGAGATACG
SRR952907.35637368.2-    ttcttttacagCCTTGATTGACAGCTCAGGGGTACGAATCTACTATACTCCGGAGATACG
SRR952907.42027886.1-    ttcttttacagCCTTGATTGACAGCTCAGGGGTACGAATCTACTATACTCCGGAGATACG
SRR952907.49893402.2-    ttcttttacagCCTTGATTGACAGCTCAGGGGTACGAATCTACTATACTCCGGAGATACG
SRR952907.52668539.1-    ttcttttacagCCTTGATTGACAGCTCAGGGGTACGAATCTACTATACTCCGGAGATACG
SRR952907.60638876.2+    ttcttttacagCCTTGATTGACAGCTCAGGGGTACGAATCTACTATACTCCGGAGATACG
SRR952908.21402763.1-    ttcttttacagCCTTGATTGACAGCTCAGGGGTACGAATCTACTATACTCCGGAGATACG
SRR952908.26607298.1-    ttcttttacagCCTTGATTGACAGCTCAGGGGTACGAATCTACTATACTCCGGAGATACG
SRR952908.30780511.2+    ttcttttacagCCTTGATTGACAGCTCAGGGGTACGAATCTACTATACTCCGGAGATACG
SRR952908.3285260.1+     ttcttttacagCCTTGATTGACAGCTCAGGGGTACGAATCTACTATACTCCGGAGATACG
SRR952908.44612500.2-    ttcttttacagCCTTGATTGACAGCTCAGGGGTACGAATCTACTATACTCCGGAGATACG
SRR952908.67808806.2-    ttcttttacagCCTTGATTGACAGCTCAGGGGTACGAATCTACTATACTCCGGAGATACG
SRR952908.73514971.1-    ttcttttacagCCTTGATTGACAGCTCAGGGGTACGAATCTACTATACTCCGGAGATACG
SRR952908.7449415.1-     tccttttacagCCTTGATTGACAGCTCAGGGGTACGAATCTACGATACTCCGGAGATACG
SRR952908.83232541.2-    ttcttttacagCCTTGATTGACAGCTCAGGGGTACGAATCTACTATACTCCGGAGATACG
SRR952906.64829881.2-     tcttttacagCCTTGATTGACAGCTCAGGGGTACGAATCTACTATACTCCGGAGATACG
SRR952906.54633368.2+      cttttacagCCTTGATTGACAGCTCAGGGGTACGAATCTACTATACTCCGGAGATACG
SRR952906.35313441.1+       ttttacagCCTTGATTGACAGCTCAGGGGTACGAATCTACTATACTCCGGAGATACG
SRR952906.51221028.1-       ttttacagCCTTGATTGACAGCTCAGGGGTACGAATCTACTATACTCCGGAGATACG
SRR952906.52004496.2+          tacagCCTTGATTGACAGCTCAGGGGTACGAATCTACTATACTCCGGAGATACG
SRR952908.71081809.2-          tacagCCTTGATTGACAGCTCAGGGGTACGAATCTACTATACTCCGGAGATACG
SRR952906.67447501.2+            cagCCTTGATTGACAGCTCAGGGGTACGAATCTACTATACTCCGGAGATACG
SRR952906.82311786.1-            cagCCTTGATTGACAGCTCAGGGGTACGAATCTACTATACTCCGGAGATACG
SRR952907.62662976.1+            cagCCTTGATTGACAGCTCAGGGGTACGAATCTACTATACTCCGGAGATACG
```

```
SRR952906.21497803.1-                 TTGATTGACAGCTCAGGGGTACGAATCTACTATACTCCGGAGATACG
SRR952906.84301423.1+                 TTGATTGACAGCTCAGGGGTACGAATCTACTATACTCCGGAGATACG
SRR952908.30675309.1+                         CAGCTCAGGGGTACGAATCTACTATACTCCGGAGATACG
SRR952908.32192016.1-                          AGCTCAGGGGTACGAATCTACTATACTCCGGAGATACG
SRR952906.58628084.1+                             TCAGGGGTACGAATCTACTATACTCCGGAGATACG
SRR952907.5940313.1+                              TCAGGGGTACGAATCTACTATACTCCGGAGATACG
SRR952907.97613.1+                                 CAGGGGTACGAATCTACTATACTCCGGAGATACG
SRR952906.82629823.2-                               AGGGGTACGAATCTACTATACTCCGGAGATACG
SRR952906.24443860.1+                                 GGGTACGAATCTACTATACTCCGGAGATACG
SRR952906.39078225.1+                                 GGGTACGAATCTACTATACTCCGGAGATACG
SRR952906.56780568.1-                                 GGGTACGAATCTACTATACTCCGGAGATACG
SRR952907.62126463.1-                                    TACGAATCTACTATACTCCGGAGATACG
SRR952906.48043948.2-                                     ACGAATCTACTATACTCCGGAGATACG
SRR952906.7214801.2-                                       CGAATCTACTATACTCCGGAGATACG
SRR952908.82698325.1+                                      CGAATCTACTATACTCCGGAGATACG
SRR952906.49364931.2+                                          TCTACTATACTCCGGAGATACG
SRR952908.67260975.1+                                           CTACTATACTCCGGAGATACG
SRR952906.31777324.1-                                               TATACTCCGGAGATACG
SRR952908.65411360.2+                                               TATACTCCGGAGATACG
SRR952906.66046796.1+                                                ATACTCCGGAGATACG
SRR952908.21383184.2+                                                   CTCCGGAGATACG
SRR952908.24524207.1+                                                   CTCCGGAGATACG
SRR952906.11280118.2+                                                    TCCGGAGATACG
SRR952906.85026718.2-                                                        GAGATACG
SRR952906.69372439.1-                                                          GATACG
SRR952908.74537743.1+                                                           ATACG
SRR952906.5395772.1+                                                               CG

consensus                ttcttttacagCCTTGATTGACAGCTCAGGGGTACGAATCTACTATACTCCGGAGATACG

                             .    :    .    :    .    :    .    :    .    :    .    :
SRR952907.49893402.2-    A
SRR952908.26607298.1-    AAAA
SRR952906.80718732.1-    AAAAT
SRR952908.21402763.1-    AAAATATGA
SRR952906.59429530.1-    AAAATATGAT
SRR952906.15561415.1-    AAAATATGATG
SRR952906.42235225.1-    AAAATATGATG
SRR952907.52668539.1-    AAAATATGATGTTGGG
SRR952908.44612500.2-    AAAATATGATGTTGGG
SRR952908.7449415.1-     AAAATATGATGTTGGG
SRR952907.35637368.2-    AAAATATGATGTTGGGATTCTGC
SRR952908.83232541.2-    AAAATATGATGTTGGGATTCTGCAA
SRR952907.14433590.2+    AAAATATGATGTTGGGATTCTGCAAA
SRR952907.60638876.2+    AAAATATGATGTTGGGATTCTGCAAA
SRR952908.30780511.2+    AAAGTATGATGTTGGGATCCTGCAAA
SRR952908.73514971.1-    AAAATATGATGTTGGGATTCTGCAAACAGG
SRR952908.3285260.1+     AAAATATGATGTTGGGATTCTGCAAACAGGCAT
SRR952908.67808806.2-    AAAATATGATGTTGGGATTCTGCAAACAGGCAT
SRR952907.42027886.1-    AAAATATGATGTTGGGATTCTGCAAACAGGCATCTTCAT
SRR952906.64829881.2-    AAAATATGATGTTGGGATTCTGCAAACAGGCATCTTCATTT
SRR952906.54633368.2+    AAAATATGATGTTGGGATTCTGCAAACAGGCATCTTCCTTTT
SRR952906.35313441.1+    AAAATATGATGTTGGGATTCTGCAAACAGGCATCTTCATTTTC
SRR952906.51221028.1-    AAAATATGATGTTGGGATTCTGCAAACAGGCATCTTCATTTTC
SRR952906.52004496.2+    AAAATATGATGTTGGGATTCTGCAAACAGGCATCTTCATTTTCCTG
SRR952908.71081809.2-    AAAATATGATGTTGGGATTCTGCAAACAGGCATCTTCATTTTCCTG
SRR952906.67447501.2+    AAAATATGATGTTGGGATTCTGCAAACAGGCATCTTCATTTTCCTGTG
SRR952906.82311786.1-    AAAATATGATGTTGGGATTCTGCAAACAGGCATCTTCATTTTCCTGTG
SRR952907.62662976.1+    AAAATATGATGTTGGGATTCTGCAAACAGGAATCTTCATTTTCCTGTG
SRR952906.21497803.1-    AAAATATGATGTTGGGATTCTGCAAACAGGCATCTTCATTTTCCTGTGCATTT
SRR952906.84301423.1+    AAAATATGATGTTGGGATTCTGCAAACAGGCATCTTCATTTTCCTGTGCATTT
SRR952906.11280118.2+    AAAATATGATGTTGGGATTCTGCAAACAGGCATCTTCATTTTCCTGTGCATTTCATTCCT
SRR952906.24443860.1+    AAAATATGATGTTGGGATTCTGCAAACAGGCATCTTCATTTTCCTGTGCATTTCATTCCT
SRR952906.31777324.1-    AAAATATGATGTTGGGATTCTGCAAACAGGCATCTTCATTTTCCTGTGCATTTCATTCCT
SRR952906.39078225.1+    AAAATATGATGTTGGGATTCTGCAACCAGGCATCTTCATTTTCCTGTGCATTTCATTCCT
SRR952906.48043948.2-    AAAATATGATGTTGGGATTCTGCAAACAGGCATCTTCATTTTCCTGTGCATTTCATTCCT
SRR952906.49364931.2+    AAAATATGATGTTGGGATTCTGCAAACAGGCATCTTCATTTTCCTGTGCATTTCATTCCT
SRR952906.5395772.1+     AAAATATGATGTTGGGATTCTGCAAACAGGCATCTTCATTTTCCTGTGCATTTCATTCCT
SRR952906.56780568.1-    AAAATATGATGTTGGGATTCTGCAAACAGGCATCTTCATTTTCCTGTGCATTTCATTCCT
SRR952906.58628084.1+    AAAATATGATGTTGGGATTCTGCAAACAGGCATCTTCATTTTCCTGTGCATTTCATTCCT
SRR952906.66046796.1+    AAAATATGATGTTGGGATTCTGCAAACAGGCATCTTCATTTTCCTGTGCATTTCATTCCT
SRR952906.69372439.1-    AAAATATGATGTTGGGATTCTGCAAACAGGCATCTTCATTTTCCTGTGCATTTCATTCCT
SRR952906.7214801.2-     AAAATATGATGTTGGGATTCTGCAAACAGGCATCTTCATTTTCCTGTGCATTTCATTCCT
SRR952906.82629823.2-    AAAATATGATGTTGGGATTCTGCAAACAGGCATCTTCATTTTCCTGTGCATTTCATTCCT
SRR952906.85026718.2-    AAAATATGATGTTGGGATTCTGCAAACAGGCATCTTCATTTTCCTGTGCATTTCATTCCT
SRR952907.5940313.1+     AAAATATGATGTTGGGATTCTGCAAACAGGCATCTTCATTTTCCTGTGCATTTCATTCCT
SRR952907.62126463.1-    AAAATATGATGTTGGGATTCTGCAAACAGGCATCTTCATTTTCCTGTGCATTTCATTCCT
SRR952907.97613.1+       AAAATATGATGTTGGGATTCTGCAAACAGGCATCTTCATTTTCCTGTGCATTTCATTCCT
SRR952908.21383184.2+    AAAATATGATGTTGGGATTCTGCAAACAGGCATCTTCATTTTCCTGTGCATTTCATTCCT
SRR952908.24524207.1+    AAAATATGATGTTGGGATTCTGCAAACAGGCATCTTCATTTTCCTGTGCATTTCATTCCT
SRR952908.30675309.1+    AAAATATGATGTTGGGATTCTGCAAACAGCCATCTTCTTTTTCCTGTGCATTTCATTCCT
SRR952908.32192016.1-    AAAATATGATGTTGGGATTCTGCAAACAGGCATCTTCATTTTCCTGTGCATTTCATTCCT
SRR952908.65411360.2+    AAAATATGATGTTGGGATTCTGCAAACAGGCATCTTCATTTTCCTGTGCATTTCATTCCT
SRR952908.67260975.1+    AAAATATGATGTTGGGATTCTGCAAACAGGCATCTTCATTTTCCTGTGCATTTCATTCCT
```

```
SRR952908.74537743.1+    AAAATATGATGTTGGGATTCTGCAAACAGGCATCTTCATTTTCCTGTGCATTTCATTCCT
SRR952908.80888265.1+    AAAATATGATGTTGGGATTCTGCAAACAGGCATCTTCATTTTCCTGTGCATTTCATTCCT
SRR952908.82698325.1+    AAAATATGATGTTGGGATTCTGCAAACAGGCATCTTCATTTTCCTGTGCATTTCATTCCT
SRR952907.55074013.1+       ATATGATGTTGGGATTCTGCAAACAGGCATCTTCATTTTCCTGTGCATTTCATTCCT
SRR952908.67330860.2+         ATGATGTTGGGATTCTGCAAACAGGCATCTTCATTTTCCTGTGCATTTCATTCCT
SRR952907.53257974.2+           GATGTTGGGATTCTGCAAACAGGCATCTTCATTTTCCTGTGCATTTCATTCCT
SRR952908.44698542.1+           GATGTTGGGATTCTGCAAACAGGCATCTTCATTTTCCTGTGCATTTCATTCCT
SRR952907.22004178.1+             TGTTGGGATTCTGCAAACAGGCATCTTCATTTTCCTGTGCATTTCATTCCT
SRR952908.2284886.1+              TGTTGGGATTCTGCAAACAGGCATCTTCATTTTCCTGTGCATTTCATTCCT
SRR952907.26704456.1-              GTTGGGATTCTGCAAACAGGCATCTTCATTTTCCTGTGCATTTCATTCCT
SRR952908.16586946.2+              GTTGGGATTCTGCAAACAGGCATCTTCATTTTCCTGTGCATTTCATTCCT
SRR952906.46865034.2+               TTGGGATTCTGCAAACAGGCATCTTCATTTTCCTGTGCATTTCATTCCT
SRR952906.74252791.1-               TTGGGATTCTGCAAACAGGCATCTTCATTTTCCTGTGCATTTCATTCCT
SRR952906.19415127.1-                   GATTCTGCAAACAGGCATCTTCATTTTCCTGTGCATTTCATTCCT
SRR952906.76417132.1+                    ATTCTGCAAACAGGCATCTTCATTTTCCTGTGCATTTCATTCCT
SRR952906.4505781.2+                        CTGCAAACAGGCATCTTCATTTTCCTGTGCATTTCATTCCT
SRR952908.54081938.2-                         GCAAACAGGCATCTTCATTTTCCTGTGCATTTCATTCCT
SRR952906.9507819.2-                             AACAGGCATCTTCATTTTCCTGTGCATTTCATTCCT
SRR952907.20559736.2+                              CAGGCATCTTCATTTTCCTGTGCATTTCATTCCT
SRR952907.49454300.1+                              CAGGCATCTTCATTTTCCTGTGCATTTCATTCCT
SRR952907.53326894.2+                              CAGGCATCTTCATTTTCCTGTGCATTTCATTCCT
SRR952907.47208830.1-                                      TTCATTTTCCTGTGCATTTCATTCCT
SRR952907.33609997.2+                                        CATTTTCCTGTGCATTTCATTCCT
SRR952908.6361494.2+                                         CATTTTCCTGTGCATTTCATTCCT
SRR952907.36742234.1-                                          TTTTCCTTTGCTTTTCATTCCT
SRR952908.3917553.1+                                              TCCTGTGCATTTCATTCCT
SRR952907.54885943.1-                                              CCTGTCCATTTCATTCCT
SRR952908.18114634.1-                                               CTGTGCATTTCATTCCT
SRR952906.75122442.1+                                                TGTGCATTTCATTCCT
SRR952906.8692711.1+                                                     CATTTCATTCCT
SRR952906.32757671.2-                                                          ATTCCT
SRR952908.77975511.2+                                                          ATTCCT
SRR952907.42435039.1+                                                            TCCT
SRR952906.35266711.1+                                                             CCT
SRR952908.41568795.1-                                                               T
                         ____________________________________________________________
consensus                AAAATATGATGTTGGGATTCTGCAAACAGGCATCTTCATTTTCCTGTGCATTTCATTCCT

                             .    :    .    :    .    :    .    :    .    :    .    :
SRR952908.30675309.1+    C
SRR952908.32192016.1-    CC
SRR952906.58628084.1+    CCTGG
SRR952907.5940313.1+     CCTGG
SRR952907.97613.1+       CCTGGG
SRR952906.82629823.2-    CCTGGGG
SRR952906.24443860.1+    CCTGGGGCA
SRR952906.39078225.1+    CCTGGGGCA
SRR952906.56780568.1-    CCTGGGGCA
SRR952907.62126463.1-    CCTGGGGCACAA
SRR952906.48043948.2-    CCTGGGGCACAAT
SRR952906.7214801.2-     CCTGGGGCACAATC
SRR952908.82698325.1+    CCTGGGGCACAATC
SRR952906.49364931.2+    CCTGGGGCACAATCCTAC
SRR952908.67260975.1+    CCTGGGGCACAATCCTACA
SRR952906.31777324.1-    CCTGGGGCACAATCCTACAAATC
SRR952908.65411360.2+    CCTGGGGCACAATCCTACAAATC
SRR952906.66046796.1+    CCTGGGGCACAATCCTACAAATCT
SRR952908.21383184.2+    CCTGGGGCACAATCCTACAAATCTTAC
SRR952908.24524207.1+    CCTGGGGCACAATCCTACAAATCTTAC
SRR952906.11280118.2+    CCTGGGGCACAATCCTACAAATCTTACG
SRR952906.85026718.2-    CCTGGGGCACAATCCTACAAATCTTACGGCCT
SRR952906.69372439.1-    CCTGGGGCACAATCCTACAAATCTTACGGCCTTT
SRR952908.74537743.1+    CCTGGGGCACAATCCTACAAATCTTACGGCCTTTG
SRR952906.5395772.1+     CCTGGGGCACAATCCTACAAATCTTACGGCCTTTGCAA
SRR952908.80888265.1+    CCTGGGGCACAATCCTACAAATCTTACGGCCTTTGCAATT
SRR952907.53257974.2+    CCTGGGGCACAATCCTAAAAATCTTACGGCCTTTGCAATTC
SRR952907.55074013.1+    CCTGGGGCACAATCCTACAAATCTTACGGCCTTTGCAATTCCA
SRR952908.67330860.2+    CCTGGGGCACAATCCTACAAATCTTACGGCCTATGCAATTCCAGC
SRR952908.44698542.1+    CCTGGGGCACAATCCTACAAATCTTACGGCCTTTGCAATTCCAGCCA
SRR952907.22004178.1+    CCTGGGGCACAATCCTACAAATCTTACGGCCTTTGCAATTCCAGCCAGT
SRR952908.2284886.1+     CCTGGGGCACAATCCTACAAATCTTACGGCCTTTGCAATTCCAGCCAGT
SRR952907.26704456.1-    CCTGGGGCACAATCCTACAAATCTTACGGCCTTTGCAATTCCAGCCAGTT
SRR952908.16586946.2+    CCTGGGGCACAATCCTACAAATCTTACGGCCTTTGCAATTCCAGCCAGTT
SRR952906.46865034.2+    CCTGGGGCACAATCCTACAAATCTTACGGCCTTTGCAATTCCAGCCAGTTT
SRR952906.74252791.1-    CCTGGGGCACAATCCTACAAATCTTACGGCCTTTGCAATTCCAGCCAGTTT
SRR952906.19415127.1-    CCTGGGGCACAATCCTACAAATCTTACGGCCTTTGCAATTCCAGCCAGTTTGATG
SRR952906.76417132.1+    CCTGGGGCACAATCCTACAAATCTTACGGCCTTTGCAATTCCAGCCAGTTTGATGA
SRR952906.4505781.2+     CCTGGGGCACAATCCTACAAATCTTACGGCCTTTGCAATTCCAGCCAGTTTGATGAAg
SRR952906.32757671.2-    CCTGGGGCACAATCCTACAAATCTTACGGCCTTTGCAATTCCAGCCAGTTTGATGAAgtg
SRR952906.35266711.1+    CCTGGGGCACAATCCTACAAATCTTACGGCCTTTGCAATTCCAGCCAGTTTGATGAAgtg
SRR952906.75122442.1+    CCTGGGGCACAATCCTACAAATCTTACGGCCTTTGCAATTCCAGCCAGTTTGATGAAgtg
SRR952906.8692711.1+     CCTGGGGCACAATCCTACAAATCTTACGGCCTTTGCAATTCCAGCCAGTTTGATGAAgtg
SRR952906.9507819.2-     CCTGGGGCACAATCCTACAAATCTTACGGCCTTTGCAATTCCAGCCAGTTTGATGAAgtg
SRR952907.20559736.2+    CCTGGGGCACAATCCTACAAATCTTACGGCCTTTGCAATTCCAGCCAGTTTGATGAAgtg
```

```
SRR952907.33609997.2+    CCTGGGGCACAATCCTACAAATCTTACGGCCTTTGCAATTCCAGCCAGTTTGATGAAgtg
SRR952907.36742234.1-    CCTGGGGCACAATCCTACAAATCTTACGGCCTTTGCAATTCCAGCCAGTTTGATGAAgtg
SRR952907.42435039.1+    CCTGGGGCACAATCCTACAAATCTTACGGCCTTTGCAATTCCAGCCAGTTTGATGAAgtg
SRR952907.47208830.1-    CTGGGGGCACAATCCTACAAATCTTACGGCCTTTGCAATTCCAGCCAGTTTGATGAAgtg
SRR952907.49454300.1+    CCTGGGGCACAATCCTACAAATCTTACGGCCTTTGCAATTCCAGCCAGTTTGATGAAgtg
SRR952907.53326894.2+    CCTGGGGCACAATCCTACAAATCTTACGGCCTTTGCAATTCCAGCCAGTTTGATGAAgtg
SRR952907.54885943.1-    CCTGGGGCACAATCCTACAAATCTTACGGCCTTTGCAATTCCAGCCAGTTTGATGAAgtg
SRR952908.18114634.1-    CCTGGGGCACAATCCTACAAATCTTACGGCCTTTGCAATTCCAGCCAGTTTGATGAAgtg
SRR952908.3917553.1+     CCTGGGGCACAATCCTACAAATCTTACGGCATTTGCAATTCCAGCCAGTTTGATGAAgtg
SRR952908.41568795.1-    CCTGGGGCACAATCCTACAAATCTTACGGCCTTGGCAATTCCAGCCAGTTTGATGAAgtg
SRR952908.54081938.2-    CCTGGGGCACAATCCTACAAATCTTACGGCCTTTGCAATTCCAGCCAGTTTGATGAAgtg
SRR952908.6361494.2+     CCTGGGGCACAATCCTACAAATCTTACGGCCTTTGCAATTCCAGCCAGTTTGATGAAgtg
SRR952908.77975511.2+    CCTGGGGCACAATCCTACAAATCTTACGGCCTTTGCATTTCCAGCCAGTTTGATGAAgtg
SRR952906.58506595.1+        GGGCACAATCCTACAAATCTTACGGCCTTTGCAATTCCAGCCAGTTTGATGAAgtg
SRR952908.50300589.1+          GCACAATCCTACAAATCTTACGGCCTTTGCAATTCCAGCCAGTTTGATGAAgtg
SRR952908.51680848.1+            ACAATCCTACAAATCTTACGGCCTTTGCAATTCCAGCCAGTTTGATGAAgtg
SRR952907.3574533.1+              CAATCCTACAAATCTTACGGCCTTTGCAATTCCAGCCAGTTTGATGAAgtg
SRR952908.76321323.1+             CAATCCTACAAATCTTACGGCCTTTGCAATTCCAGCCAGTTTGATGAAgtg
SRR952906.77934628.2+                  CTACAAATCTTACGGCCTTTGCAATTCCAGCCAGTTTGATGAAgtg
SRR952906.50682290.2+                     CAAATCTTACGGCCTTTGCAATTCCAGCCAGTTTGATGAAgtg
SRR952908.49037486.2+                     CAAATCTTACGGCCTTTGCAATTCCAGCCAGTTTGATGAAgtg
SRR952908.74322018.2+                      AAATCTTACGGCCTTTGCAATTCCAGCCAGTTTGATGAAgtg
SRR952907.32150617.2-                                GCCTTTGCAATTCCAGCCAGTTTGATGAAgtg
                         ____________________________________________________________
consensus                CCTGGGGCACAATCCTACAAATCTTACGGCCTTTGCAATTCCAGCCAGTTTGATGAAgtg

                             .    :    .    :    .    :    .    :    .    :    .    :
SRR952908.54081938.2-    a
SRR952906.9507819.2-     agtg
SRR952907.20559736.2+    agtgtg
SRR952907.49454300.1+    agtgtg
SRR952907.53326894.2+    agtgtg
SRR952908.3917553.1+     agtgtgggg
SRR952907.36742234.1-    agtgtgggga
SRR952907.47208830.1-    agtgtggggaaagc
SRR952907.33609997.2+    agtgtggggacagcca
SRR952908.6361494.2+     agtgtggggaaagcca
SRR952907.54885943.1-    agtgtggggaaagccat
SRR952908.18114634.1-    agtgtggggaaagccatacaaat
SRR952906.75122442.1+    agtgtggggaaagccatacaaatt
SRR952906.8692711.1+     agtgtggggaaagccatacaaattcttt
SRR952906.32757671.2-    agtgtggggaaagccatacaaattctttcttgaa
SRR952908.77975511.2+    agtgtggggaaagccatacaaattctttcttgaa
SRR952907.42435039.1+    agtgtggggaaagccatacaaattctttcttgaact
SRR952906.35266711.1+    agtgtggggaaagccatacaaattctttcttgaactt
SRR952908.41568795.1-    agtgtgggaaaagccatacaaattctttcttgaacttcc
SRR952906.58506595.1+    agtgtggggaaagccatacaaattctttcttgaacttccacact
SRR952908.50300589.1+    agtgtggggaaagccatacaaattctttcttgaacttccacacttt
SRR952908.51680848.1+    agtgtggggaaagccatacaaattctttcttgaacttccacactttgg
SRR952907.3574533.1+     agtgtggggaaagccatacaaattctttcttgaacttccacactttggg
SRR952908.76321323.1+    agtgtggggaaagccatacaaattctttcttgaacttccacactttggg
SRR952906.77934628.2+    agtgtggggaaagccatacaaattctttcttgaacttccacactttgggctttc
SRR952906.50682290.2+    agtgtggggaaagccatacaaattctttcttgaacttccacactttgggctttcatc
SRR952908.49037486.2+    agtgtggggaaagccatacaaattctttcttgaacttccacactttgggctttcatc
SRR952908.74322018.2+    agtgtggggaaagccatacaaattctttcttgaacttccacactttgggctttcatc
SRR952907.32150617.2-    agtgtggggaaagccatacaaattctttcttgaacttccacactttgggctttcatcttg
                         ____________________________________________________________
consensus                agtgtggggaaagccatacaaattctttcttgaacttccacactttgggctttcatcttg
```

V. Anna's hummingbird (No. 44), exon 7, WGS data

- CAP3 alignment of WGS data spanning exon 7 (uppercase letters)

```
                             .    :    .    :    .    :    .    :    .    :    .    :
SRR943143.71338993.2-    tggagtcattggatgaagaagtggcaattttgttttacagA
SRR943153.78307081.1-    tggagtcactggatgaagaagtggaaattttgttttacagA
SRR943153.95911344.1-    tggagtcactggatgaagaagtggaaattttgttttacagA
SRR943143.41571662.2+    tggagtcattggatgaaggagtggcaattttggtttacagAT
SRR943149.87141934.2-    tggagtcattggatgaagaagtggcaattttgttttacagATT
SRR943145.33525948.2-    tggagtcactggatgaagaagtggaaattttgttttacagATTT
SRR943144.120004190.1+   tggaggcattggatgaagaagtggcaattttgttttacagATTTG
SRR943146.59723313.2-    tggagtcattggatgaagaagtggcaattttgttttacagATTTG
SRR943143.59649683.1+    tggagtcactggatgaagaagtggaaattttgttttacagATTTGA
SRR943143.80431057.2+    tggagtcactggatgaagaagtggaaattttgttttacagATTTGA
SRR943144.10625440.1+    tggagtcattggatgaagaagtggcacttttgttttacagATTTGA
SRR943148.51374543.2-    tggagtcactggatgaagaagtggaaattttgttttacagATTTGA
SRR943144.124836672.1+   tggagtcactggatgaagaagtggaaattttgttttacagATTTGAT
SRR943146.54309627.1+    tggagtcactggatgaagaagtggaaattttgttttacagATTTGAT
SRR943146.60319297.2-    tggagtcactggatgaagaagtggaaattttgttttacagATTTGAT
SRR943148.84011717.1-    tggagtcattggatgaagaagtggcaattttgttttacagATTTGAT
SRR943143.5986688.1-     tggagtcattggatgaagaagtggcaattttgttttacagATTTGATT
SRR943146.88093154.2-    tggagtcactggatgaagaagtggaaattttgttttacagATTTGATT
SRR943147.6549173.1-     tggagtcattggatgaagaagtggcaattttgttttacagATTTGATT
SRR943146.26601761.2-    tggagtcactggatgaagaagtggaaattttgttttacagATTTGATTG
SRR943143.30328037.1-    tggagtcattggatgaagaagtggcaattttgttttacagATTTGATTGAC
SRR943146.88155671.1+    tggagtcactggatgaagaagtggaaattttgttttacagATTTGATTGAC
SRR943146.33402082.1+    tggagtcattggatgaagaagtggcaattttgttttcagATTTTATTGACA
SRR943146.63207770.2+    tggagtcattggatgaagaagtggcaattttgttttaccgATTTGATTGACA
SRR943143.7465013.2+     tggagtcactggatgaagaagtggaaattttgttttacagATTTGATTTACAGTT
SRR943144.28072035.1-    tggagtcactggatgaagaagtggaaattttgttttacagATTTGATTGACAGTTC
SRR943144.64698558.2+    tggagtcattggatgaagaagtggcaattttgttttacagATTTGATTGACAGTTC
SRR943144.66067357.1+    tggagtcactggatgaagaagtggaaattttgttttacagATTTGATTGACAGTTC
SRR943144.25427215.1-    tggagtcattggatgaagaagtggcaattttgttttacagATTTGATTGACAGTTCA
SRR943144.43123986.2-    tggagtcattggatgaagaagtggcaattttgttttacagATTTGATTGACAGTTCA
SRR943145.19843385.1-    tggagtcattggatgaagaagtggcaattttgttttacagATTTGATTGACAGTTCA
SRR943143.12418561.2+    tggagtcattggatgaagaagtggcaattttgttttacagATTTGATTGACAGTTCAGG
SRR943143.41571662.1-    tggagtcattggatgaagaagtggcaattttgttttacagATTTGATTGACAGTTCAGG
SRR943143.51827441.2+    tggagtcattggatgaagaagtggcaattttgttttacagATTTGATTGACAGTTCAGG
SRR943143.93970140.1+    tggagtcactggatgaagaagtggaaattttgttttacagATTTGATTGACAGTTCAGG
SRR943143.97171448.1-    tggagtcactggatgaagaagtggaaattttgttttacagATTTGATTGACAGTTCAGG
SRR943144.103415697.1-   tggagtcactggatgaagaagtggaaattttgttttacagATTTGATTGACAGTTCAGG
SRR943144.10625440.2-    tggagtcattggatgaagaagtggcaattttgttttacagATTTGATTGACAGTTCAGG
SRR943144.120004190.2-   tggagtcattggatgaagaagtggcaattttgttttacagATTTGATTGACAGTTCAGG
SRR943144.120268833.1-   tggagtcactggatgaagaagtggaaattttgttttacagATTTGATTGACAGTTCAGG
SRR943144.124836672.2-   tggagtcactggatgaagaagtggaaattttgttttacagATTTGATTGACAGTTCAGG
SRR943144.125836244.2-   tggagtcactggatgaagaagtggaaattttgttttacagATTTGATTGACAGTTCAGG
SRR943144.128147151.1-   tggagtcattggatgaagaagtggcaattttgttttacagATTTGATTGACAGTTCAGG
SRR943144.128147151.2+   tggagtcattggatgaagaagtggcaattttgttttacagATTTGATTGACAGTTCAGG
SRR943144.128292763.1-   tggagtcactggatgaagaagtggaaattttgttttacagATTTGATTGACAGTTCAGG
SRR943144.1487824.1+     tggagtcattggatgaagaagtggcaattttgttttacagATTTGATTGACAGTTCAGG
SRR943144.16651012.1-    tggactcattggatgaagaagttgcaattttgttttacagATTTGATTGACAGTTCAGG
SRR943144.16651012.2+    tggagtcattggatgaagaagtggcaattttgttttacagATTTGATTGACAGTTCAGG
SRR943144.25313586.1+    tggagtcattggatgaagaagtggcaattttgttttacagATTTGATTGACAGTTCAGG
SRR943144.29810028.2-    tggagtcattggatgaagaagtggcaattttgttttacagATTTGATTGACAGTTCAGG
SRR943144.33496876.1-    tggagtcactggatgaagaagtggaaattttgttttacagATTTGATTGACAGTTCAGG
SRR943144.33496876.2+    tggagtcactggatgaagaagtggaaattttgttttacagATTTGATTGACAGTTCAGG
SRR943144.36431995.1+    tggagtcattggatgaagaagtggcaattttgttttacagATTTGATTGACAGTTCAGG
SRR943144.36431995.2-    tggagtcattggatgaagaagtggcaattttgttttacagATTTGATTGACAGTTCAGG
SRR943144.38598998.2+    tggagtcattggatgaagaagtggcaattttgttttacagATTTGATTGACAGTTCAGG
SRR943144.52466857.1+    tggagtcattggatgaagaagtggcaattttgttttacagATTTGATTGACAGTTCAGG
SRR943144.52466857.2-    tggagtcattggatgaagaagtggcaattttgttttacagATTTGATTGACAGTTCAGG
SRR943144.58987988.2+    tggagtcattggatgaagaagtggcaattttgttttacagATTTGATTGACAGTTCAGG
SRR943144.63460008.2+    tggagtcactggatgaagaagtggaaattttgttttacagATTTGATTGACAGTTCAGG
SRR943144.64698558.1-    tggagtcattggatgaagaagtggcaattttgttttacagATTTGATTGACAGTTCAGG
SRR943144.65768613.1+    tggagtcattggatgaagaagtggcaattttgttttacagATTTGATTGACAGTTCAGG
SRR943144.66067357.2-    tggagtcactggatgaagaagtggaaattttgttttacagATTTGATTGACAGTTCAGG
SRR943144.85990836.1-    tggagtcattggatgaagaagtggcaattttgttttacagATTTGATTGACAGTTCAGG
SRR943144.94389262.2+    tggagtaattggatgaagaagtggcaattttgttttacagATTTGATTGACAGTTCAGG
SRR943145.1037712.1+     tggagtcactggatgaagaagtggaaattttgttttacagATTTGATTGACAGTTCAGG
SRR943145.19882519.1+    tggagtcactggatgaagaagtggaaattttgttttacagATTTGATTGACAGTTCCGG
SRR943145.2375109.1-     tggagtcattggatgaagaagtggcaattttgttttacagATTTGATTGACAGTTCAGG
SRR943145.31911556.2-    tggagtcattggatgaagaagtggcaattttgttttacagATTTGATTGACAGTTCAGG
SRR943145.40759658.2+    tggagtcactggatgaagaagtggaaattttgttttacagATTTGATTCACAGTTCAGG
SRR943145.52403324.2+    tggagtcattggatgaagaagtggcaattttgttttacagATTTGATTGACAGTTCAGG
SRR943145.53460378.1+    tggagtcactggatgaagaagtggaaattttgttttacagATTTGATTGACAGTTCAGG
SRR943145.70579934.1-    tggagtcattggatgaagaagtggcaattttgttttacagATTTGATTGACAGTTCAGG
SRR943146.107250245.1-   tggagtcactggatgaagaagtggaaattttgttttacagATTTGATTGACAGTTCAGG
SRR943146.112914742.2+   tggagtcactggatgaagaagtggaaattttgttttacagATTTGATTGACAGTTCAGG
SRR943146.11566485.1-    tggagtcattggatgaagaagtggcaattttgttttacagATTTGATTGACAGTTCAGG
```

```
SRR943146.16880733.1-    tggagtcactggatgaagaagtggaaatttttgttttacagATTTGATTGACAGTTCAGG
SRR943146.20533547.1+    tggagtcattggatgaagaagtggcaatttttgttttacagATTTGATTGACAGTTCAGG
SRR943146.24636360.2-    tggagtcattggatgaagaagtggcaatttttgttttacagATTTGATTGACAGTTCAGG
SRR943146.37708858.1-    tggagtcactggatgaagaagtggaaatttttgttttacagATTTGATTGACAGTTCAGG
SRR943146.38846969.2-    tggattcattggatgaagaagtagcaatttttgtttttcagATTTGATTGACAGTTCAGG
SRR943146.38943217.1+    tggagtcactggatgaagaagtggaaatttttgttttacagATTTGATTGACAGTTCAGG
SRR943146.56821384.2+    tggagtcattggatgaagaagtggcaatttttgttttacagATTTGATTGACAGTTCAGG
SRR943146.57346176.1-    tggagtcattggatgaagaagtggcaatttttgttttacagATTTGATTGACAGTTCAGG
SRR943146.60669571.2-    tggagtcactggatgaagaagtggaaatttttgttttacagATTTGATTGACAGTTCAGG
SRR943146.70638704.1-    tggagtcattggatgaagaagtggcaatttttgttttacagATTTGATTGACAGTTCAGG
SRR943146.88081757.1+    tggagtcactggatgaagaagtggaaatttttgttttacagATTTGATTGACAGTTCAGG
SRR943146.96096379.1+    tggagtcattggatgaagaagtggcaatttttgttttacagATTTGATTGACAGTTCAGG
SRR943146.99253409.2+    tggagtcactggatgaagaagtggaaatttttgttttacagATTTGATTGACAGTTCAGG
SRR943147.22901505.2+    tggagtcattggatgaagaagtggcaatttttgttttacagATTTGATTGACAGTTCAGG
SRR943148.12227873.1-      gagtcactggatgaagaagtggaaatttttgttttacagATTTGATTGA
SRR943143.80431057.1-        gtcactggatgaagaagtggaaatttttgttttacagATTTGATTGACAGTTCAGG
SRR943143.117003318.1+        tcattggatgaagaagtggcaatttttgttttacagATTTGATTGACAGTTCAGG
SRR943144.65768613.2-         tcattggatgaagaagtggcaatttttgttttacagATTTGATTGACAGTTCAGG
SRR943148.40077430.1-          cattggatgaagaagtggcaatttttgttttacagATTTGATTGACAGT
SRR943148.61883455.1-          cattggatgaagaagtggcaatttttgttttacagATTTGATTGACAGT
SRR943150.60834945.2+          cattggatgaagaagtggcaatttttgttttacagATTTGATTGACAGT
SRR943153.78469985.1-            ttggatgaagaagtggcaatttttgttttacagATTTGATTGACAGTTC
SRR943144.130236616.2+          ctggatgaagaagtggaaatttttgttttacagATTTGATTGACAGTTCAGG
SRR943148.45501956.2+              ggatgaagaagtggaaatttttgttttacagATTTGATTGACAGTTCAG
SRR943144.38598998.1-              ggatgaagaagtggcaatttttgttttacagATTTGATTGACAGTTCAGG
SRR943149.73275911.1-               gatgaagaagtggcaatttttgttttacagATTTGATTGACAGTTCAGG
SRR943143.104139687.2+               tgaagaagtggaaatttttgttttacagATTTGATTGACAGTTCAGG
SRR943143.7465013.1-                 tgaagaagtggaaatttttgttttacagATTTGATTGACAGTTCAGG
SRR943144.94389262.1-                tgaagaagtggcaatttttgttttacagATTTGATTGACAGTTCAGG
SRR943147.58951334.2+                tgaagaagtggaaatttttgttttacagATTTGATTGACAGTTCAGG
SRR943143.59649683.2-                   agaagtggaaatttttgttttacagATTTGATTGACAGTTCAGG
SRR943144.63460008.1-                   agaagtgcaaattcttgttttacagATTTGAGTGACAGTTCAGG
SRR943144.25313586.2-                     aagtggcaatttttgttttacagATTTGATTGACAGTTCAGG
SRR943144.58987988.1-                      agtggcaatttttgttttacagATTTGATTGACAGTTCAGG
SRR943143.12418561.1-                        tggcaatttttgttttacagATTTGATTGACAGTTCAGG
SRR943149.73739647.1+                        tggcaatttttgttttacagATTTGATTGACAGTTCAGG
SRR943151.20532827.1+                        tggaaatttttgttttacagATTTGATTGACAGTTCAGG
SRR943151.33137181.1+                        tggaaatttttgttttacagATTTGATTGACAGTTCAGG
SRR943151.49445837.1+                        tggaaatttttgttttacagATTTGATTGACAGTTCAGG
SRR943151.83316320.1+                        tggaaatttttgttttacagATTTGATTGACAGTTCAGG
SRR943151.92173533.1+                        tggaaatttttgttttacagATTTGATTGACAGTTCAGG
SRR943151.97997498.1+                        tggaaatttttgttttacagATTTGATTGACAGTTCAGG
SRR943146.113926285.2-                          aaatttttgttttacagATTTGATTGACAGTTCAGG
SRR943149.3686943.2+                            caatttttgttttacagATTTGATTGACAGTTCAGG
SRR943149.78051790.2+                           caatttttgttttacagATTTGATTGACAGTTCAGG
SRR943144.1487824.2-                             aattatcgttttacagATTTGATTGACAGTTCAGG
SRR943143.89649215.2+                             atttttggtttacagATTTGATTGACAGTTCAGG
SRR943146.106284797.2-                            atttttgttttacagATTTGATTGACAGTTCAGG
SRR943146.81925975.2+                             atttttgttttacagATTTGATTGACAGTTCAGG
SRR943151.33893871.2+                             atttttgttttacagATTTGATTGACAGTTCAGG
SRR943147.7530622.2-                                ttttgttttacagATTTGATTGACAGTTCAGG
SRR943148.5209468.2+                                 tttgttttacagATTTGATTGACAGTTCAGG
SRR943144.46004729.1+                                 ttgttttacagATTTGATTGACAGTTCAGG
SRR943146.62058440.2-                                  tgttttacagATTTGATTGACAGTTCAGG
SRR943151.89390003.1+                                  tgttttacagATTTGATTGACAGTTCAGG
SRR943143.115573669.1+                                  gttttacagATTTGATTGACAGTTCAGG
SRR943145.1810097.1-                                    gttttacagATTTGATTGACAGTTCAGG
SRR943148.4628954.1+                                    gttttacagATTTGATTGACAGTTCAGG
SRR943146.28553096.2+                                    ttttacagATTTGATTGACAGTTCAGG
SRR943145.57608674.1+                                     tttacagATTTGATTGACAGTTCAGG
SRR943149.90288787.2+                                     tttacagATTTGATTGACAGTTCAGG
SRR943153.71670252.2-                                      ttacagATTTGATTGACAGTTCAGG
SRR943145.48591543.2+                                       tacagATTTGATTGACAGTTCAGG
SRR943145.69292912.1-                                       tacagATTTGATTGACAGTTCAGG
SRR943153.42045335.2+                                       tacagATTTGATTGACAGTTCAGG
SRR943143.106713870.1+                                      acagATTTGATTGACAGTTCAGG
SRR943143.41825835.1+                                       acagATTTGATTGACAGTTCAGG
SRR943143.106665816.1+                                       cagATTTGATTGACAGTTCAGG
SRR943143.93970140.2-                                        cagATTTGATTGACAGTTCAGG
SRR943147.61967811.2-                                         agATTTGATTGACAGTTCAGG
SRR943149.6086161.1+                                          agATTTGATTGACAGTTCAGG
SRR943153.49100440.2+                                         agATTTGATTGACAGTTCAGG
SRR943144.134799716.1+                                          ATTTGATTGACAGTTCAGG
SRR943145.4137743.2+                                            ATTTGATTGACAGTTCAGG
SRR943146.13788959.2+                                           ATTTGATTGACAGTTCAGG
SRR943144.13635896.1+                                            TTTGATTGACAGTTCAGG
SRR943145.42788029.1-                                            TTTGATTGACAGTTCAGG
SRR943149.18492996.2-                                             TTGATTGACAGTTCAGG
SRR943147.3318547.1+                                               TGATTGACAGTTCAGG
SRR943148.4470618.2+                                               TGATTGACAGTTCAGG
SRR943143.51827441.1-                                               GATTGACAGTTCAGG
SRR943148.73412914.1+                                               GATTGACAGTTCAGG
SRR943153.20644198.1+                                               GATTGACAGTTCAGG
SRR943146.15632033.1+                                                ATTGACAGTTCAGG
```

```
SRR943146.75372287.2-                                                  ATTGACAGTTCAGG
SRR943145.52626318.1+                                                   TTGACAGTTCAGG
SRR943145.59637562.2-                                                   TTGACAGTTCAGG
SRR943143.78524906.2+                                                    TGACAGTTCAGG
SRR943145.41988875.1+                                                    TGACAGTTCAGG
SRR943146.25190807.1-                                                      ACAGTTCAGG
SRR943146.17538271.1+                                                       CAGTTCAGG
SRR943148.78207349.2-                                                       CAGTTCAGG
SRR943148.88063584.2-                                                       CAGTTCAGG
SRR943153.100375048.2-                                                      CAGTTCAGG
SRR943143.26110525.1+                                                        AGTTCAGG
SRR943143.113297297.2+                                                        GTTCAGG
SRR943145.11325062.1-                                                          TTCAGG
SRR943145.69188289.2-                                                          TTCAGG
SRR943152.102397740.1-                                                         TTCAAG
SRR943152.31868961.1-                                                          TTCAGG
SRR943152.65964492.1-                                                          TTCAGG
SRR943152.85082107.1-                                                          TTCAGG
SRR943152.99181241.1-                                                          TTCAGG
SRR943152.99975745.1-                                                          TTCAGG
SRR943146.110572913.2-                                                          TCAGG
SRR943146.99816648.1-                                                           TCAGG
SRR943147.14377517.2+                                                           TCAGG
SRR943153.15751132.1+                                                           TCAGG
SRR943145.38435938.2+                                                             AGG
SRR943150.20185035.2-                                                              GG
SRR943150.27211556.2-                                                              GG
SRR943146.75164931.1-                                                               G

consensus                tggagtcattggatgaagaagtggcaatttttgttttacagATTTGATTGACAGTTCAGG

                             .    :    .    :    .    :    .    :    .    :    .    :
SRR943144.103415697.1-   G
SRR943145.19882519.1+    GG
SRR943143.12418561.2+    GGT
SRR943145.2375109.1-     GGT
SRR943145.52403324.2+    GGT
SRR943146.37708858.1-    GGTA
SRR943146.38943217.1+    GGTA
SRR943143.97171448.1-    GGTAT
SRR943145.1037712.1+     GGTAT
SRR943146.107250245.1-   GGTAT
SRR943146.56821384.2+    GGTAT
SRR943146.96096379.1+    GGGAT
SRR943147.22901505.2+    GGTAT
SRR943146.60669571.2-    GGTATGA
SRR943144.10625440.2-    GGTATGAAT
SRR943146.16880733.1-    GGTATGAAT
SRR943145.40759658.2+    GGGATGAATC
SRR943149.73739647.1+    GGTATGAATC
SRR943151.20532827.1+    GGTATGAATC
SRR943151.33137181.1+    GGTATGAATC
SRR943151.49445837.1+    GGTATGAATC
SRR943151.83316320.1+    GGTATGAATC
SRR943151.92173533.1+    GGTATGAATC
SRR943151.97997498.1+    GGTATGAATC
SRR943145.31911556.2-    GGTATGAATCA
SRR943144.120004190.2-   GGTATGAATCAC
SRR943146.20533547.1+    GGTATGAATCAC
SRR943149.3686943.2+     GGTATGAATCACC
SRR943149.78051790.2+    GGTATGAATCACC
SRR943146.88081757.1+    GGTATGAATCACCA
SRR943151.33893871.2+    GGTATGAATCACCAG
SRR943146.57346176.1-    GGTATGAATCACCAGA
SRR943143.93970140.1+    GGTATGAATCACCAGAG
SRR943145.70579934.1-    GGTATGAATCACCAGAG
SRR943144.128292763.1-   GGTATGAATCACCAGAGC
SRR943146.38846969.2-    GGTATGAATCACCAGAGC
SRR943148.5209468.2+     GGTATGAATCACCAGAGC
SRR943145.53460378.1+    GGTATGAATCACCAGAGCT
SRR943151.89390003.1+    GGTATGAATCACCAGAGCTA
SRR943148.4628954.1+     GGTATGAATCACCAGAGCTAC
SRR943149.90288787.2+    GGTATGAATCACCAGAGCTACGG
SRR943144.29810028.2-    GGTATGAATCACCAGAGCTACGGA
SRR943153.71670252.2-    GGTATGAATCACCAGAGCTACGGA
SRR943153.42045335.2+    GGTATGAATCACCAGAGCTACGGAA
SRR943143.51827441.2+    GGTATGAATCACCAGAGCTACGGAAATA
SRR943144.85990836.1-    GGTATGAATCACCAGAGCTACGGAAATA
SRR943146.11566485.1-    GGTATGAATCACCAGAGCTACGGAAATA
SRR943149.6086161.1+     GGTATGAATCACCAGAGCTACGGAAATA
SRR943153.49100440.2+    GGTATGAATCACCAGAGCTACGGAAATA
SRR943144.125836244.2-   GGTATGAATCACCAGAGCTACGGAAATATG
SRR943144.33496876.2+    GGTATGAATCACCAGAGCTACGGAAATNTGA
SRR943144.66067357.2-    GGTATGAATCACCAGAGCTACGGAAATATGAT
```

SRR943146.24636360.2- GGTATGAATCACCAGAGCTACGGAAATATGAT
SRR943149.18492996.2- GGTATGAATCACCAGAGCTACGGAAATATGAT
SRR943144.52466857.1+ GGTATGAATCACCAGAGCTACGGAAATATGATG
SRR943148.4470618.2+ GGTATGAATCACCAGAGCTACGGAAATATGATG
SRR943144.36431995.1+ GGTATGAATCACCAGAGCTACGGAAATATGATGT
SRR943146.70638704.1- GGTATGAATCACCAGAGCTACGGAAATATGATGT
SRR943148.73412914.1+ GGTATGAATCACCAGAGCTACGGAAATATGATGT
SRR943153.20644198.1+ GGTATGAATCACCAGAGCTACGGAAATATGATGT
SRR943144.124836672.2- GGTATGAATCACCAGAGCTACGGAAATATGATGTGGG
SRR943143.41571662.1- GGTATGAATCACCAGAGCTACGGAAATATGATGTGGGGA
SRR943146.112914742.2+ GGTATGAATCACCAGAGCTACGGAAATATGATGTGGGGAT
SRR943146.99253409.2+ GGTATGAATCACCAGAGCTACGGAAATATGATGTGGGGAT
SRR943148.78207349.2- GGTATGAATCACCAGAGCTACGGAAATATGATGTGGGGAT
SRR943148.88063584.2- GGTATGAATCACCAGAGCTACGGAAATATGATGTGGGGAT
SRR943153.100375048.2- GGTATGAATCACCAGAGCTACGGAAATATGATGTGGGGAT
SRR943152.102397740.1- GGTATGAATCACCAGAGCTACGGAAATATGATGTGGGGATTCT
SRR943152.31868961.1- GGTATGAATCACCAGAGCTACGGAAATATGATGTGGGGATTCT
SRR943152.65964492.1- GGTATGAATCACCAGAGCTACGGAAATATGATGTGGGGATTCT
SRR943152.85082107.1- GGTATGAATCACCAGAGCTACGGAAATATGATGTGGGGATTCT
SRR943152.99181241.1- GGTATGAATCACCAGAGCTACGGAAATATGATGTGGGGATTCT
SRR943152.99975745.1- GGTATGAATCACCAGAGCTACGGAAATATGATGTGGGGATTCT
SRR943143.80431057.1- GGTATGAATCACCAGAGCTACGGAAATATGATGTGGGGATTCTG
SRR943144.16651012.2+ GGTATGAATCACCAGAGCTACGGAAATATGCTGTGGGGATTCTG
SRR943153.15751132.1+ GGTATGAATCACCAGAGCTACGGAAATATGATGTGGGGATTCTG
SRR943143.117003318.1+ GGTATGAATCACCAGAGCTACGGAAATATGATGTGGGGATTCTGC
SRR943150.20185035.2- GGTATGAATCACCAGAGCTACGGAAATATGATGTGGGGATTCTGCAA
SRR943150.27211556.2- GGTATGAATCACCAGAGCTACGGAAATATGATGTGGGGATTCTGCAA
SRR943144.128147151.2+ GGTATGAATCACCAGAGCTACGGAAATATGATGTGGGGATTCTGCAGAC
SRR943153.47365653.1- GGTATGAATCACCAGAGCTACGGAAATATGATGTGGGGATTCTGCAAAC
SRR943153.50402848.1- GGTATGAATCACCAGAGCTACGGAAATATGATGTGGGGATTCTGCAAAC
SRR943143.104139687.2+ GGTATGAATCACCAGAGCTACGGAAATATGATGTGGGGATTCTGCAAACAGGT
SRR943143.7465013.1- GGTATGAATCACCAGAGCTACGGAAATATGATGTGGGGATTCTGCAAACAGGT
SRR943144.64698558.1- GGTATGAATCACCAGAGCTACGGAAATATGATGTGGGGATTCTGCAAACAGGT
SRR943147.58951334.2+ GGTATGAATCACCAGAGCTACGGAAATATGATGTGGGGATTCTGCAAACAGGT
SRR943143.59649683.2- GGTATGAATCACCAGAGCTACGGAAATATGATGTGGGGATTCTGCAAACAGGTGTC
SRR943144.120268833.1- GGTATGAATCACCAGAGCTACGGAAATATGATGTGGGGATTCTGCAAACAGGTGTCT
SRR943144.58987988.2+ GGTATGAATCACCAGAGCTACGGAAATATGATGTGGGGATTCTGCAAACAGGTGTCT
SRR943144.38598998.2+ GGTATGAATCACCAGAGCTACGGAAATATGATGTGGGGATTCTGCAAACAGGNGNCTT
SRR943144.63460008.2+ GGTATGAATCACCAGAGCTACGGAAATATGATGTGGGGATTCTGCAAACAGCTGTGTTC
SRR943143.106665816.1+ GGTATGAATCACCAGAGCTACGGAAATATGATGTGGGGATTCTGCAAACAGGTGTCTTCA
SRR943143.106713870.1+ GGTATGAATCACCAGAGCTACGGAAATATGATGTGGGGATTCTGCAAACAGGTGTCTTCA
SRR943143.113297297.2+ GGTATGAATCACCAGAGCTACGGAAATATGATGTGGGGATTCTGCAAACAGGTGTCTTCA
SRR943143.115573669.1+ GGTATGAATCACCAGAGCTACGGAAATATGATGTGGGGATTCTGCAAACAGGTGTCTTCA
SRR943143.12418561.1- GGTATGAATCACCAGAGCTACGGAAATATGATGTGGGGATTCTGCAAACAGGTGTCTTCA
SRR943143.26110525.1+ GGTATGAATCACCAGAGCTACGGAAATATGATGTGGGGATTCTGCAAACAGGTGTCTTCA
SRR943143.41825835.1+ GGTATGAATCACCAGAGCTACGGAAATATGATGTGGGGATTCTGCAAACAGGTGTCTTCA
SRR943143.51827441.1- GGTATGAATCACCAGAGCTACGGAAATATGATGTGGGGATTCTGCAAACAGGTGTCTTCA
SRR943143.78524906.2+ GGTATGAATCACCAGAGCTACGGAAATATGATGTGGGGATTCTGCAAACAGGTGTCTTCA
SRR943143.89649215.2+ GGTATGAATCACCAGAGCTACGGAAATATGATGTGGGGATTCTGCAAACAGGTGTCTTCA
SRR943143.93970140.2- GGTATGAATCACCAGAGCTACGGAAATATGATGTGGGGATTCTGCAAACAGGTGTCTTCA
SRR943144.128147151.1- GGTATGAATCACCAGAGCTACGGAAATATGATGTGGGGATTCTGCAAACNGNNGTCTTCA
SRR943144.130236616.2+ GGTATGAATCACCAGAGCTACGGAAATATGATGTGGGGATTCTGCAAACAGGTGTCTTCA
SRR943144.134799716.1+ GGTATGAATCACCAGAGCTACGGAAATATGATGTGGGGATTCTGCAAACAGGTGTCTTCA
SRR943144.13635896.1+ GGTATGAATCACCAGAGCTACGGAAATATGATGTGGGGATTCTGCAAACAGGTGTCTTCA
SRR943144.1487824.1+ GGTATGAATCACCAGAGCTACGGAAATATGATGTGGGGATTCTGCAAACAGGTGTCTTCA
SRR943144.1487824.2- GGTATGAATCACCAGAGCTACGGAAATATGATGTGGGGATTCTGCAAACAGGTGTCTTCA
SRR943144.16651012.1- GGTATGAATCACCAGAGCTACGGAAATATGATGTGGGGATTCTGCAAACAGGTGTCTTCA
SRR943144.25313586.1+ GGTATGAATCACCAGAGCTACGGAAATATGATGTGGGGATTCTGCAAACAGGTGTCTTCA
SRR943144.25313586.2- GGTATGAATCACCAGAGCTACGGAAATATGATGTGGGGATTCTGCAAACAGGTGTCTTCA
SRR943144.33496876.1- GGTATGAATCACCAGAGCTACGGAAATATGATGTGGGGATTCTGCAAACAGGTGTCTTCA
SRR943144.36431995.2- GGTATGAATCACCAGAGCTACGGAAATATGATGTGGGGATTCTGCAAACAGGTGTCTTCA
SRR943144.38598998.1- GGTATGAATCACCAGAGCTACGGAAATATGATGTGGGGATTCTGCAAACAGGTGTCTTCA
SRR943144.46004729.1+ GGTATGAATCACCAGAGCTACGGAAATATGATGTGGGGATTCTGCAAACAGGTGTCTTCA
SRR943144.52466857.2- GGTATGAATCACCAGAGCTACGGAAATATGATGTGGGGATTCTGCAAACAGGTGTCTTCA
SRR943144.58987988.1- GGTATGAATCACCAGAGCTACGGAAATATGATGTGGGGATTCTGCAAACAGGTGTCTTCA
SRR943144.63460008.1- GGTATGAATCACCAGAGCTACGGAAATATGATGTGGGGATTCTGCAAACAGGTGTCTTCA
SRR943144.65768613.1+ GGTATGAATCACCAGAGCTACGGAAATATGATGTGGGGATTCTGCAAACAGGTGTCTTCA
SRR943144.65768613.2- GGTATGAATCACCAGAGCTACGGAAATATGATGTGGGGATTCTGCAAACAGGTGTCTTCA
SRR943144.94389262.1- GGTATGAATCACCAGAGCTACGGAAATATGATGTGGGGATTCTGCAAACAGGTGTCTTCA
SRR943144.94389262.2+ GGTATGAATCACCAGAGCTACGGAAATATGATGTGGGGATTCTGCAAACAGGTGTCTTCA
SRR943145.11325062.1- GGTATGAATCACCAGAGCTACGGAAATATGATGTGGGGATTCTGCAAACAGGTGTCTTCA
SRR943145.1810097.1- GGTATGAATCACCAGAGCTACGGAAATATGATGTGGGGATTCTGCAAACAGGTGTCTTCA
SRR943145.38435938.2+ GGTATGAATCACCAGAGCTACGGAAATATGATGTGGGGATTCTGCAAACAGGTGTCTTCA
SRR943145.4137743.2+ GGTATGAATCACCAGAGCTACGGAAATATGATGTGGGGATTCTGCAAACAGGTGTCTTCA
SRR943145.41988875.1+ GGTATGAATCACCAGAGCTACGGAAATATGATGTGGGGATTCTGCAAACAGGTGTCTTCA
SRR943145.42788029.1- GGTATGAATCACCAGAGCTACGGAAATATGATGTGGGGATTCTGCAAACAGGTGTCTTCA
SRR943145.48591543.2+ GGTATGAATCACCAGAGCTACGGAAATATGATGTGGGGATTCTGCAAACAGGTGTCTTCA
SRR943145.52626318.1+ GGTATGAATCACCAGAGCTACGGAAATATGATGTGGGGATTCTGCAAACAGGTGTCTTCA
SRR943145.57608674.1+ GGTATGAATCACCAGAGCTACGGAAATATGATGTGGGGATTCTGCAAACAGGTGTCTTCA
SRR943145.59637562.2- GGTATGAATCACCAGAGCTACGGAAATATGATGTGGGGATTCTGCAAACAGGTGTCTTCA
SRR943145.69188289.2- GGTATGAATCACCAGAGCTACGGAAATATGATGTGGGGATTTTGCAAACAGGTGTCTTCA
SRR943145.69292912.1- GGTATGAATCACCAGAGCTACGGAAATATGATGTGGGGATTCTGCAAACAGGTGTCTTCA
SRR943146.106284797.2- GGTATGAATCACCAGAGCTACGGAAATATGATGTGGGGATTCTGCAAACAGGTGTCTTCA
SRR943146.110572913.2- GGTATGAATCACCAGAGCTACGGAAATATGATGTGGGGATTCTGCAAACAGGTGTCTTCA

```
SRR943146.113926285.2-   GGTATGAATCACCAGAGCTACGGAAATATGATGTGGGGATTCTGCAAACAGGTGTCTTCA
SRR943146.13788959.2+    GGTATGAATCACCAGAGCTACGGAAATATGATGTGGGGATTCTGCAAACAGGTGTCTTCA
SRR943146.15632033.1+    GGTATGAATCACCAGAGCTACGGAAATATGATGTGGGGATTCTGCAAACAGGTGTCTTCA
SRR943146.17538271.1+    GGTATGAATCACCAGAGCTACGGAAATATGATGTGGGGATTCTGCAAACAGGGGTCTTCA
SRR943146.25190807.1-    GGTATGAATCACCAGAGCTACGGAAATATGATGTGGGGATTCTGCAAACAGGTGTCTTCA
SRR943146.28553096.2+    GGTATGAATCACCAGAGCTACGGAAATATGATGTGGGGATTCTGCAAACAGGTGTCTTCA
SRR943146.62058440.2-    GGTATGAATCACCAGAGCTACGGAAATATGATGTGGGGATTCTGCAAACAGGTGTCTTCA
SRR943146.75164931.1-    GGTATGAATCACCAGAGCTACGGAAATATGATGTGGGGATTCTGCAAACAGGTGTCTTCA
SRR943146.75372287.2-    GGTATGAATCACCAGAGCTACGGAAATATGATGTGGGGATTCTGCAAACAGGTGTCTTCA
SRR943146.81925975.2+    GGTATGAATCACCAGAGCTACGGAAATATGATGTGGGGATTCTGCAAACAGGTGTCTTCA
SRR943146.99816648.1-    GGTATGAATCACCAGAGCTACGGAAATATGATGTGGGGATTCTGCAAACAGGTGTCTTCA
SRR943147.14377517.2+    GGTATGAATCACCAGAGCTACGGAAATATGATGTGGGGATTCTGCAAACAGGTGTCTTCA
SRR943147.3318547.1+     GGTATGAATCACCAGAGCTACGGAAATATGATGTGGGGATTCTGCAAACAGGTGTCTTCA
SRR943147.61967811.2-    GGTATGAATCACCAGAGCTACGGAAATATGATGTGGGGATTCTGCAAACAGGTGTCTTCA
SRR943147.7530622.2-     GGTATGAATCACCAGAGCTACGGAAATATGATGTGGGGATTCTGCAAACAGGTGTCTTCA
SRR943153.19521073.1-      TATGAATCACCAGAGCTACGGAAATATGATGTGGGGATTCTGCAAACAG
SRR943153.91968749.1-      TATGAATCACCAGAGCTACGGAAATATGATGTGGGGATTCTGCAAACAG
SRR943149.84401868.2-       ATGAATCACCAGAGCTACGGAAATATGATGTGGGGATTCTGCAAACAGG
SRR943146.95141330.1+       ATGAATCACCAGAGCTACGGAAATATGATGTGGGGATTCTGCAAACAGGTGTCTTCA
SRR943147.51805956.1-         GAATCACCAGAGCTACGGAAATATGATGTGGGGATTCTGCAAACAGGTGTCTTCA
SRR943143.117003318.2-         AATCACCAGAGCTACGGAAATATGATGTGGGGATTCTGCAAACAGGTGTCTTCA
SRR943147.41610683.1+           ATCACCAGAGCTACGGAAATATGATGTGGGGATTCTGCAAACAGGTGTCTTCA
SRR943145.58569145.2+             CACCAGAGCTACGGAAATATGATGTGGGGATTCTGCAAACAGGTGTCTTCA
SRR943147.40896979.2+             CACCAGAGCTACGGAAATATGATGTGGGGATTCTGCAAACAGGTGTCTTCA
SRR943147.9963273.2+              CACCAGAGCTACGGAAATATGATGTGGGGATTCTGCAAACAGGTGTCTTCA
SRR943143.104139687.1-             ACCAGAGCTACGGAAATATGATGTGGGGATTCTGCAAACAGGTGTCTTCA
SRR943146.88097701.1-              ACCAGAGCTACGGAAATAGGATGTGGGGATTCTGCAAACAGGTTTCTTCA
SRR943147.12183045.2+              ACCAGAGCTACGGAAATATGATGTGGGGATTCTGCAAACAGGTGTCTTCA
SRR943143.110177944.2+              CCAGAGCTACGGAAATATGATGTGGGGATTCTGCAAACAGGTGTCTTCA
SRR943147.53191156.1+               CCAGAGCTACGGAAATATGATGTGGGGATTCTGCAAACAGGTGTCTTCA
SRR943152.16507538.1+               CCAGAGCTACGGAAATATGATGTGGGGATTCTGCAAACAGGTGTCTTCA
SRR943143.85000359.1+                CAGAGCTACGGAAATATGATGTGGGGATTCTGCAAACAGGTGTCTTCA
SRR943150.20620102.2-                  GAGCTACGGAAATATGATGTGGGGATTCTGCAAACAGGTGTCTTCA
SRR943150.49081874.2-                  GAGCTACGGAAATATGATGTGGGGATTCTGCAAACAGGTGTCTTCA
SRR943143.44324018.2+                      TACGGAAATATGATGTGGGGATTCTGCAAACAGGTGTCTTCA
SRR943143.98833310.2+                      TACGGAAATATGATGTGGGGATTCTGCAAACAGGTGTCTTCA
SRR943153.50148005.1+                      TACGGAAATATGATGTGGGGATTCTGCAAACAGGTGTCTTCA
SRR943143.89649215.1-                       ACGGAAATATGATGTGGGGATTCTGCAAACAGGTGTCTTCA
SRR943153.85576202.1-                       ACGGAAATATGATGTGGGGATTCTGCAAACAGGTGTCTTCA
SRR943145.55459185.2-                        CGGAAATATGATGTGGGGATTCTGCAAACAGGTGTCTTCA
SRR943146.106410727.1+                       CGGAAATATGATGTGGGGATTCTGCAAACAGGTGTCTTCA
SRR943151.32180301.2+                        CGGAAATATGATGTGGGGATTCTGCAAACAGGTGTCTTCA
SRR943151.35942467.2+                        CGGAAATATGATGTGGGGATTCTGCAAACAGGTGTCTTCA
SRR943151.37483907.2+                        CGGAAATATGATGTGGGGATTCTGCAAACAGGTGTCTTCA
SRR943151.42764520.2+                        CGGAAATATGATGTGGGGATTCTGCAAACAGGTGTCTTCA
SRR943151.46891216.2+                        CGGAAATATGATGTGGGGATTCTGCAAACAGGTGTCTTCA
SRR943151.48744472.2+                        CGGAAATATGATGTGGGGATTCTGCAAACAGGTGTCTTCA
SRR943151.57682362.2+                        CGGAAATATGATGTGGGGATTCTGCAAACAGGTGTCTTCA
SRR943151.70629567.2+                        CGGAAATATGATGTGGGGATTCTGCAAACAGGTGTCTTCA
SRR943151.73554194.2+                        CGGAAATATGATGTGGGGATTCTGCAAACAGGTGTCTTCA
SRR943151.96055972.2+                        CGGAAATATGATGTGGGGATTCTGCAAACAGGTGTCTTCA
SRR943151.98557232.2+                        CGGAAATATGATGTGGGGATTCTGCAAACAGGTGTCTTCA
SRR943143.107878190.1+                        GGAAATATGATGTGGGGATTCTGCAAACAGGTGTCTTCA
SRR943147.36150911.2-                         GGAAATATGATGTGGGGATTCTGCAAACAGGTGTCTTCA
SRR943153.36028462.2-                         GGAAATATGATGTGGGGATTCTGCAAACAGGTGTCTTCA
SRR943146.13936469.2-                          GAAATATGATGTGGGGATTCTGCAAACAGGTGTCTTCA
SRR943148.20800129.2-                          GAAATATGATGTGGGGATTCTGCAAACAGGTGTCTTCA
SRR943144.46004729.2-                           AAATTTGATGTGGGGAGTCTTGAAACAGGTGTCTTCA
SRR943145.13993241.1+                           AAATATGATGTGGGGATTCTGCAAACAGGTGTCTTCA
SRR943146.3316304.2+                             AATATGATGTGGGGATTCTGCAAACAGGTGTCTTCA
SRR943143.41825835.2-                             ATATGATGTGGGGATTCTGCAAACAGGTGTCTTCA
SRR943144.134799716.2-                            ATATGATGTGGGGATTTGTCAAACAGGTGTCTTCA
SRR943144.13635896.2-                             ATATGTTGTGGGGATTCTGCAAACAGGTGTCTTCA
SRR943145.6158089.1+                              ATATGATGTGGGGATTCTGCAAACAGGTGTCTTCA
SRR943146.98785799.1+                             ATATGATGTGGGGATTCTGCAAACAGGTGTCTTCA
SRR943143.115573669.2-                             TATGATGTGGGGATTCTGCAAACAGGTGTCTTCA
SRR943143.13911124.1+                              TATGATGTGGGGATTCTGCAAACAGGTGTCTTCA
SRR943145.1935921.2-                               TATGATGTGGGGATTCTGCAAACAGGTGTCTTCA
SRR943146.53723254.2+                               ATGATGTGGGGATTCTGCAAACAGGTGTCTTCA
SRR943145.48367946.2-                                TGATGTGGGGATTCTGCAAACAGGTGTCTTCA
SRR943146.68438362.2-                                TGATGTGGGGATTCTGCAAACAGGTGTCTTCA
SRR943146.13911030.2-                                 GATGTGGGGATTCTGCAAACAGGTGTCTTCA
SRR943149.32803211.1-                                  ATGTGGGGATTCTGCAAACAGGTGTCTTCA
SRR943143.78524906.1-                                   TGTGGGGATTCTGCAAACAGGTGTCTTCA
SRR943146.113655658.1+                                  TGTGGGGATTCTGCAAACAGGTGTCTTCA
SRR943148.71970949.2+                                   TGTGGGGATTCTGCAAACAGGTGTCTTCA
SRR943149.16484339.1-                                   TGTGGGGATTCTGCAAACAGGTGTCTTCA
SRR943143.106665816.2-                                     GGGGATTCTGCAAACAGGTGTCTTCA
SRR943146.93080774.2+                                      GGGGATTCTGCAAACAGGTGTCTTCA
SRR943144.139888000.1+                                       GGATTCTGCAAACAGGTGTCTTCA
SRR943153.29753373.2+                                        GGATTCTGCAAACAGGTGTCTTCA
SRR943146.42700050.1+                                          ATTCTGCAAACAGGTGTCTTCA
SRR943149.100001168.2+                                         ATTCTGCAAACAGGTGTCTTCA
SRR943145.12257330.2+                                           TTCTGCAAACAGGTGTCTTCA
```

```
SRR943145.16844422.1-                                           TTCTGCAAACAGGTGTCTTCA
SRR943145.14723638.1+                                             CTGCAAACAGGTGTCTTCA
SRR943145.20588671.1+                                             CTGCAAACAGGTGTCTTCA
SRR943144.64510980.1+                                              TGCAAACAGGTGTCTTCA
SRR943147.6502784.1+                                               TGCAAACAGGTGTCTTCA
SRR943153.18720358.2+                                              TGCAAACAGGTGTCTTCA
SRR943143.106713870.2-                                              GCAAACAGGTGTCTTCA
SRR943145.13463055.2-                                               GCAAACAGGTGTCTTCA
SRR943147.27862538.2+                                               GCAAACAGGTGTCTTCA
SRR943148.56461203.2-                                               GCAAACAGGTGTCTTCA
SRR943143.85862572.2+                                                CAAACAGGTGTCTTCA
SRR943143.33813501.2+                                                 AAACAGGTGTCTTCA
SRR943147.4719698.1+                                                  AAACAGGTGTCTTCA
SRR943143.90158177.1+                                                  AACAGGTGTCTTCA
SRR943149.92763631.2+                                                  AACAGGTGTCTTCA
SRR943148.33856345.2-                                                   ACAGGTGTCTTCA
SRR943143.60106320.1+                                                    CAGGTGTCTTCA
SRR943149.41927252.1-                                                    CAGGTGTCTTCA
SRR943149.64475155.1-                                                    CAGGTGTCTTCA
SRR943147.17163453.1-                                                     AGTTGTCTTCA
SRR943148.8321592.1+                                                       GGTGTCTTCA
SRR943145.25852643.2-                                                       GTGTCTTCA
SRR943146.91719564.1+                                                        TGTCTTCA
SRR943149.74210498.2-                                                         GTCTTCA
SRR943153.98584337.2-                                                         GTCTTCA
SRR943146.61065462.2-                                                          TCTTCA
SRR943153.65873285.2-                                                          TCTTCA
SRR943143.113297297.1-                                                          CTTCA
SRR943144.102539740.2+                                                          CTTCA
SRR943147.14001746.1-                                                           CTTCA
SRR943149.18002460.2-                                                            TTCA
SRR943143.110177944.1-                                                             CA

consensus                GGTATGAATCACCAGAGCTACGGAAATATGATGTGGGGATTCTGCAAACAGGTGTCTTCA

                             .    :    .    :    .    :    .    :    .    :    .    :
SRR943143.12418561.1-    T
SRR943144.25313586.1+    TT
SRR943150.20620102.2-    TTT
SRR943150.49081874.2-    TTT
SRR943146.113926285.2-   TTTT
SRR943143.89649215.2+    TTTTCC
SRR943146.106284797.2-   TTTTCC
SRR943146.81925975.2+    TTTTCC
SRR943153.50148005.1+    TTTTCCC
SRR943147.7530622.2-     TTTTCCCT
SRR943153.85576202.1-    TTTTCCTT
SRR943151.32180301.2+    TTTTCCCTG
SRR943151.35942467.2+    TTTTCCCTG
SRR943151.37483907.2+    TTTTCCCTG
SRR943151.42764520.2+    TTTTCCCTG
SRR943151.46891216.2+    TTTTCCCTG
SRR943151.48744472.2+    TTTTCCCTG
SRR943151.57682362.2+    TTTTCCCTG
SRR943151.70629567.2+    TTTTCCCTG
SRR943151.73554194.2+    TTTTCCCTG
SRR943151.96055972.2+    TTTTCCCTG
SRR943151.98557232.2+    TTTTCCCTG
SRR943153.36028462.2-    TTTTCCTTGT
SRR943146.62058440.2-    TTTTCCTTGTG
SRR943148.20800129.2-    TTTTCCTTGTG
SRR943143.115573669.1+   TTTTCCTTGTGC
SRR943144.1487824.1+     TTTTCCCTGTGC
SRR943144.33496876.1-    TTTTCCTTGTGC
SRR943144.65768613.1+    TTTTCCCTGTGC
SRR943145.1810097.1-     TTTTCCTTGTGC
SRR943146.28553096.2+    TTTTCCTTGTGCA
SRR943145.57608674.1+    TTTTCCCTGTGCAT
SRR943144.94389262.2+    TTTTCCCTGTGCATT
SRR943145.48591543.2+    TTTTCCCTGTGCATTT
SRR943145.69292912.1-    TTTTCCCTGTGCATTT
SRR943143.106713870.1+   TTTTCCCTGTGCATTTC
SRR943143.41825835.1+    TTTTCCTTGTGCATTTC
SRR943143.106665816.1+   TTTTCCCTGTGCATTTCA
SRR943143.93970140.2-    TTTTCCTTGTGCATTTCA
SRR943144.52466857.2-    TTTTCCCTGTGCATTTCA
SRR943147.61967811.2-    TTTTCCTTGTGCATTTCAT
SRR943149.32803211.1-    TTTTCCCTGTGCATTTCAT
SRR943144.36431995.2-    TTTTCCCTGTGCATTTCATT
SRR943148.71970949.2+    TTTTCCCTGTGCATTTCATT
SRR943149.16484339.1-    TTTTCCCTGTGCATTTCATT
SRR943145.4137743.2+     TTTTCCCTGTGCATTTCATTC
SRR943146.13788959.2+    TTTTCCTTGTGCATTTCATTC
SRR943145.42788029.1-    TTTTCCTTGTGCATTTCATTCC
```

SRR943147.3318547.1+ TTTTCCCTGTGCATTTCATTCCCC
SRR943143.51827441.1- TTTTCCCTGTGCATTTCATTCCCCC
SRR943144.16651012.1- TTTTCCCTGTGCATTTCATTCCCCC
SRR943153.29753373.2+ TTTTCCCTGTGCATTTCATTCCCCC
SRR943146.15632033.1+ TTTTCCCTGTGCATTTCATTCCCCCT
SRR943146.75372287.2- TTTTCCCTGTGCATTTCATTCCCCCT
SRR943144.128147151.1- TTTTCCCTGTGCATTTCATTCCCCCTG
SRR943145.52626318.1+ TTTTCCTTGTGCATTTCATTCCCCCTG
SRR943145.59637562.2- TTTTCCTTGTGCATTTCATTCCCCCTG
SRR943149.100001168.2+ TTTTCCTTGTGCATTTCATTCCCCCTG
SRR943143.78524906.2+ TTTTCCCTGTGCATTTCATTCCCCCTGG
SRR943145.41988875.1+ TTTTCCTTGTGCATTTCATTCCCCCTGG
SRR943146.25190807.1- TTTTCCCTGTGCATTTCATTCCCCCTGGAG
SRR943146.17538271.1+ TTTTCCTTGTGCATTTCATTCCCCCTGGAGC
SRR943153.18720358.2+ TTTTCCTTGTGCATTTCATTCCCCCTGGAGC
SRR943143.26110525.1+ TTTTCCTTGTGCATTTCATTCCCCCTGGAGCA
SRR943145.38435938.2+ TTTTCCCTGTGCATTTCATTCCCCCTGGAGCA
SRR943148.56461203.2- TTTTCCTTGTGCATTTCATTCCCCCTGGAGCA
SRR943143.113297297.2+ TTTTCCCTGTGCATTTCATTCCCCCTGGAGCAG
SRR943145.11325062.1- TTTTCCCTGTGCATTTCATTCCCCCTGGAGCAGA
SRR943145.69188289.2- TTTTCCCTGTGCATTTCATTCCCCCTGGAGCAGA
SRR943147.14377517.2+ TTTTCCCTGTGCATTTCATTCCCCCTGGAGCAGA
SRR943144.65768613.2- TTTTCCCTGTGCATTTCATTCCCCCTGGAGCAGAA
SRR943146.110572913.2- TTTTCCTTGTGCATTTCATTCCCCCTGGAGCAGAA
SRR943146.99816648.1- TTTTCCCTGTGCATTTCATTCCCCCTGGAGCAGAA
SRR943149.92763631.2+ TTTTCCCTGTGCATTTCATTCCCCCTGGAGCAGAA
SRR943148.33856345.2- TTTTCCCTGTGCATTTCATTCCCCCTGGAGCAGAAT
SRR943144.130236616.2+ TTTTCCTTGTGAATTTCATTCCCCCTGGAGCAGAATC
SRR943149.41927252.1- TTTTCCTTGTGCATTTCATTCCCCCTGGAGCAGAATC
SRR943149.64475155.1- TTTTCCTTGTGCATTTCATTCCCCCTGGAGCAGAATC
SRR943146.75164931.1- TTTTCCTTGTGCATTTCATTCCCCCTGGAGCAGAATCCT
SRR943148.8321592.1+ TTTTCCCTGTGCATTTCATTCCCCCTGGAGCAGAATCCT
SRR943144.38598998.1- TTTTCCCTGTGCATTTCATTCCCCCTGGAGCAGAATCCTA
SRR943144.25313586.2- TTTTCCCTGTGCATTTCATTCCCCCTGGAGCAGAATCCTAC
SRR943149.74210498.2- TTTTCCCTGTGCATTTCATTCCCCCTGGAGCAGAATCCTACA
SRR943153.98584337.2- TTTTCCCTGTGCATTTCATTCCCCCTGGAGCAGAATCCTACA
SRR943144.94389262.1- TTTTCCCTGTGCATTTCATTCCCCCTGGAGCAGAATCCTACAG
SRR943146.95141330.1+ TTTTCCTTGTGCATTTCATTCCCCCTGGAGCAGAATCCTACAG
SRR943153.65873285.2- TTTTCCCTGTGCATTTCATTCCCCCTGGAGCAGAATCCTACAG
SRR943147.51805956.1- TTTTCCTTGTGCATTTCATTCCCCCTGGAGCAGAATCCTACAGAT
SRR943149.18002460.2- TTTTCCTTGTGCATTTCATTCCCCCTGGAGCAGAATCCTACAGAT
SRR943143.117003318.2- TTTTCCCTGTGCATTTCATTCCCCCTGGAGCAGAATCCTACAGATC
SRR943144.63460008.1- TTTTCCTTGTGCATTTCATTCCCCCTGGAGCAGAATCCTACAGATC
SRR943147.41610683.1+ TTTTCCTTGTGCATTTCATTCCCCCTGGAGCAGAATCCTACAGATCT
SRR943144.58987988.1- TTTTCCCTGTGCATTTCATTCCCCCTGGAGCAGAATCCTACAGATCTTA
SRR943145.58569145.2+ TTTTCCCTGTGCATTTCATTCCCCCTGGAGCAGAATCCTACAGATCTTA
SRR943147.40896979.2+ TTTTCCTTGTGCATTTCATTCCCCCTGGAGCAGAATCCTACAGATCTTA
SRR943147.9963273.2+ TTTTCCTTGTGCATTTCATTCCCCCTGGAGCAGAATCCTACAGATCTTA
SRR943143.104139687.1- TTTTCCTTGTGCATTTCATTCCCCCTGGAGCAGAATCCTACAGATCTTAT
SRR943146.88097701.1- TTTTCCCTGTTCATTTCATTCCCCCTGGAGCAGAATCCTACAGATCTTAT
SRR943147.12183045.2+ TTTTCCTTGTGCATTTCATTCCCCCTGGAGCAGAATCCTACAGATCTTAT
SRR943143.110177944.2+ TTTTCCCTGTGCATTTCATTCCCCCTGGAGCAGAATCCTACAGATCTTATG
SRR943147.53191156.1+ TTTTCCTTGTGCATTTCATTCCCCCTGGAGCAGAATCCTACAGCTCTTATG
SRR943143.85000359.1+ TTTTCCTTGTGCATTTCATTCCCCCTGGAGCAGAATCCTACAGATCTTATGG
SRR943144.1487824.2- TTTTCCCTGTGCATTTCATTCCCCCTGGAGCAGAATCCTACAGATCTTATGGACT
SRR943143.44324018.2+ CTTTCCTTGTGCATTTCATTCCCCCTGGAGCAGAATCCTACAGATCTTATGGCCTTTG
SRR943143.98833310.2+ TTTTCCCTGTGCATTTCATTCCCCCTGGAGCAGAATCCTACAGATCTTATGGACTTTG
SRR943143.89649215.1- TTTTCCCTGTGCATTTCATTCCCCCTGGAGCAGAATCCTACAGATCTTATGGACTTTGC
SRR943143.106665816.2- TTTTCCCTGTGCATTTCATTCCCCCTGGAGCAGAATCCTACAGATCTTATGGACTTTGCA
SRR943143.106713870.2- TTTTCCCTGTGCATTTCATTCCCCCTGGAGCAGAATCCTACAGATCTTATGGACTTTGCA
SRR943143.107878190.1+ TTTTCCCTGTGCATTTCATTCCCCCTGGAGCAGAATCCTACAGATCTTATGGACTTTGCA
SRR943143.110177944.1- TTTCCCCTGTGCATTTCATGCCCCCTGGAGCAGAATCCTACAGATCTTATGGACTTTGCA
SRR943143.113297297.1- TTTTCCCTGTGCATTTCATTCCCCCTGGAGCAGAATCCTACAGATCTTATGGACTTTGCA
SRR943143.115573669.2- TTTTCCTTGTGCATTTCATTCCCCCTGGAGCAGAATCCTACAGATCTTATGGACTTTGCA
SRR943143.13398540.2+ TTTTCCCTGTGCATTTCATTCCCCCTGGAGCAGAATCCTACAGATCTTATGGACTTTGCA
SRR943143.13911124.1+ TTTTCCTTGTGCATTTCATTCCCCCTGGAGCAGAATCCTACAGATCTTATGGACTTTGCA
SRR943143.33813501.2+ TTTTCCCTGTGCATTTCATTCCCCCTGGAGCAGAATCCTACAGATCTTATGGACTTTGCA
SRR943143.41825835.2- TTTTCCTTGTGCATTTCATTCCCCCTGGAGCAGAATCCTACAGATCTTATGGACTTTGCA
SRR943143.60106320.1+ TTTTCCCTGTGCATTTCATTCCCCCTGGAGCAGAATCCTACAGATCTTATGGACTTTGCA
SRR943143.78524906.1- TTTTCCCTGTGCATTTCATTCCCCCTGGAGCAGAATCCTACAGATCTTATGGACTTTGCA
SRR943143.85862572.2+ TTTTCCTTGTGCATTTCATTCCCCCTGGAGCAGAATCCTACAGATCTTATGGACTTTGCA
SRR943143.90158177.1+ TTTTCCCTGTGCATTTCATTCCCCCTGGAGCAGAATCCTACAGATCTTATGGACTTTGCA
SRR943144.102539740.2+ TTTTCCTTGTGCATTTCATTCCCCCTGGAGCAGAATCCTACAGATCTTATGGACTTTGCA
SRR943144.134799716.1+ TTTTCCTTGTGCATTTCATTCCCCCTGGAGCAGAATCCTACAGATCTTATGGACTTTGCA
SRR943144.134799716.2- TTTTCCTTGTGCATTTCATTCCCCCTGGAGCACAATCCTACAGATCTTATGGACTTTGCA
SRR943144.13635896.1+ TTTTCCTTGTGCATTTCATTCCCCCTGGAGCAGAATCCTACAGATCTTATGGACTTTGCA
SRR943144.13635896.2- TTTTCCTTGTGCATTTCATTCCCCCTGGAGCAGAATCCTACAGATCTTATGGACTTTGCA
SRR943144.139888000.1+ TTTTCCCTGTGCATTTCATTCCCCCTGGAGCAGAATCCTACAGATCTTATGGACTTTGCA
SRR943144.46004729.1+ TTTTCCCTGTGCATTTCATTCCCCCTGGAGCAGAATCCTACAGATCTTCTGCACTTTGCA
SRR943144.46004729.2- TTTTCCCTGTGCATTTCATTCCCCCTGGAGCAGAATCCTACAGATCTTATGGACTTTGCA
SRR943144.64510980.1+ TTTTCCTTGTGCATTTCATTCCCCCTGGAGCAGAATCCTACAGATCTTATGGACTTTGCA
SRR943145.12257330.2+ TTTTCCTTGTGCATTTCATTCCCCCTGGAGCAGAATCCTACAGATCTTATGGACTTTGCA
SRR943145.13463055.2- TTTTCCTTGTGCATTTCATTCCCCCTGGAGCAGAATCCTACAGATCTTATGGACTTTGCA
SRR943145.13993241.1+ TTTTCCTTGTGCATTTCATTCCCCCTGGAGCAGAATCCTACAGATCTTATGGACTTTGCC

```
SRR943145.14723638.1+    TTTTCCTTGTGCATTTCATTCCCCCTGGAGCAGAATCCTACAGATCTTATGGACTTTGCA
SRR943145.16844422.1-    GTTTCCCTGTGCATTTCATTCCCCCTGGAGCAGAATCCTACAGATCTTATGGACTTTGCA
SRR943145.1935921.2-     TTTTCCCTGTGCATTTCATTCCCCCTGGAGCAGAATCCTACAGATCTTATGGACTTTGCA
SRR943145.20588671.1+    TTTTCCCTGTGCATTTCATTCCCCCTGGAGCAGAATCCTACAGATCTTATGGACTTTGCA
SRR943145.25852643.2-    TTTTCCTTGTGCATTTCATCCCCCCTGGAGCAGAATCCTACAGATCTTATGGACTTTGCA
SRR943145.48367946.2-    TTTTCCCTGTGCATTTCATTCCCCCTGGAGCAGAATCCTACAGATCTTATGGACTTTGCA
SRR943145.55459185.2-    TTTTCCCTGTGCATTTCATTCCCCCTGAAGCAGGATCCTACAGATCTTATGGACTTTGCA
SRR943145.6158089.1+     TTTTCCCTGTGCATTTCATTCCCCCTGGAGCAGAATCCTACAGATCTTATGGACTTTGCA
SRR943146.106410727.1+   TTTTCCCTGTGCATTTCATTCCCCCTGGAGCAGAATCCTACAGATCTTATGGACTTTGCA
SRR943146.113655658.1+   TTTTCCCTGTGCATTTCATTCCCCCTGGAGCAGAATCCTACAGATCTTATGGACTTTGCA
SRR943146.13911030.2-    TTTTCCTTGTGCATTTCATTCCCCCTGGAGCAGAATCCTACAGATCTTATGGACTTTGCA
SRR943146.13936469.2-    TTTTCCCTGTGCATTTCATTCCCCCTGGAGCAGAATCCTACAGATCTTATGGACTTTGCA
SRR943146.3316304.2+     TTTTCCCTGTGCATTTCATTCCCCCTGGAGCAGAATCCTACAGATCTTATGGACTTTGCA
SRR943146.42700050.1+    TTTTCCCTGTGCATTTCATTCCCCCTGGAGCAGAATCCTACAGATCTTATGGACTTTGCA
SRR943146.53723254.2+    TTTTCCTTGTGCATTTCATTCCCCCTGGAGCAGAATCCTACAGATCTTATGGACTTTGCA
SRR943146.61065462.2-    TTTTCCTTGTGCATTTCATTCCCCCTGGAGCAGAATCCTACAGATCTTATGGACTTTGCA
SRR943146.68438362.2-    TTTTCCTTGTGCATTTCATTCCCCCTGGAGCAGAATCCTACAGATCTTATGGACTTTGCA
SRR943146.91719564.1+    TTTTCCCTGTGCATTTCATTCCCCCTGGAGCAGAATCCTACAGATCTTATGGACTTTGCA
SRR943146.93080774.2+    TTTTCCCTGTGCATTTCATTCCCCCTGGAGCAGAATCCTACAGATCTTATGGACTTTGCA
SRR943146.98785799.1+    TTTTCCCTGTGCATTTCATTCCCCCTGGAGCAGAATCCTACAGATCTTATGGACTTTGCA
SRR943147.14001746.1-    TTTTCCTTGTGCATTTCATGCCCCCTGGAGCAGAATCCTACAGATCTTATGGACTTTGCA
SRR943147.17163453.1-    TTTTCCCTGTGCATTTCATTCCCCCTGGAGCAGAATCCTACAGATCTTATGGACTTTGCA
SRR943147.27862538.2+    TTTTCCTTGTGCATTTCATTCCCCCTGGAGCAGAATCCTACAGATCTTATGGACTTTGCA
SRR943147.36150911.2-    TTTTCCTTGTGCATTTCATTCCCCCTGGAGCAGAATCCTACAGATCTTATGGACTTTGCA
SRR943147.4719698.1+     TTTTCCCTGTGCATTTCATTCCCCCTGGAGCAGAATCCTACAGATCTTATGGACTTTGCA
SRR943147.6502784.1+     TTTTCCTTGTGCATTTCATTCCCCCTGGAGCAGAATCCTACAGATCTTATGGACTTTGCA
SRR943143.26110525.2-      TTCCTTGTGCATTTCATTCCCCCTGGAGCAGAATCCTACAGATCTTATGGACTTTGCA
SRR943148.97637212.2-       TCCTTGTGCATTTCATTCCCCCTGGAGCAGAATCCTACAGATCTTATGG
SRR943149.100054377.2+      TCCTTGTGCATTTCATTCCCCCTGGAGCAGAATCCTACAGATCTTATGG
SRR943149.27586442.2+       TCCTTGTGCATTTCATTCCCCCTGGAGCAGAATCCTACAGATCTTATGG
SRR943151.26860935.2-        CCTTGTGCATTTCATTCCCCCTGGAGCAGAATCCTACAGATCTTATGGA
SRR943151.28687755.1+        CCTTGTGCATTTCATTCCCCCTGGAGCAGAATCCTACAGATCTTATGGA
SRR943151.34878530.1+        CCTTGTGCATTTCATTCCCCCTGGAGCAGAATCCTACAGATCTTATGGA
SRR943151.42922701.1+        CCTTGTGCATTTCATTCCCCCTGGAGCAGAATCCTACAGATCTTATGGA
SRR943151.4303772.1+         CCTTGTGCATTTCATTCCCCCTGGAGCAGAATCCTACAGATCTTATGGA
SRR943151.57379942.1+        CCTTGTGCATTTCATTCCCCCTGGAGCAGAATCCTACAGATCTTATGGA
SRR943151.82615231.1+        CCTTGTGCATTTCATTCCCCCTGGAGCAGAATCCTACAGATCTTATGGA
SRR943146.50372746.1+        CCCTGTGCATTTCATTCCCCCTGGAGCAGAATCCTACAGATCTTATGGACTTTGCA
SRR943147.63110318.2+        CCTTGTGCATTTCATTCCCCCTGGAGCAGAATCCTACAGATCTTATGGACTTTGCA
SRR943147.22932053.2+         CCTGTGCATTTCATTCCCCCTGGAGCAGAATCCTACAGATCTTATGGACTTTGCA
SRR943143.53168784.1+          CTGTGCATTTCATTCCCCCTGGAGCAGAATCCTACAGATCTTATGGACTTTGCA
SRR943143.85000359.2-          TTGTGCATTTCATTCCCCCTGGAGCAGAATCCTACAGATCTTATGGACTTTGCA
SRR943153.51811801.1-           TGTGCATTTCATTCCCCCTGGAGCAGAATCCTACAGATCTTATGGACTT
SRR943153.74875920.2-           TGTGCATTTCATTCCCCCTGGAGCAGAATCCTACAGATCTTATGGACTT
SRR943149.9994123.1-              TGCATTTCATTCCCCCTGGAGCAGAATCCTACAGATCTTATGGACTTTG
SRR943146.99173336.1+               CATTTCATTCCCCCTGGAGCAGAATCCTACAGATCTTATGGACTTTGCA
SRR943148.28683594.1+               CATTTCATTCCCCCTGGAGCAGAATCCTACAGATCTTATGGACTTTGCA
SRR943146.4306804.2+                 ATTTCATTCCCCCTGGAGCAGAATCCTACAGATCTTATGGACTTTGCA
SRR943149.18673434.2+                ATTTCATTCCCCCTGGAGCAGAATCCTACAGATCTTATGGACTTTGCA
SRR943144.139888000.2-                  TCATTCCCCCTGGAGCAGAATCCTACAGATCTTATGGACTTTGCA
SRR943146.78904130.1+                   TCATTCCCCCTGGAGCAGAATCCTACAGATCTTATGGACTTTGCA
SRR943143.24007821.2+                    CATTCCCCCTGGAGCAGAATCCTACAGATCTTATGGACTTTGCA
SRR943153.13386580.1-                    CATTCCCCCTGGAGCAGAATCCTACAGATCTTATGGACTTTGCA
SRR943153.24651648.1+                    CATTCCCCCTGGAGCAGAATCCTACAGATCTTATGGACTTTGCA
SRR943153.27887061.1+                    CATTCCCCCTGGAGCAGAATCCTACAGATCTTATGGACTTTGCA
SRR943153.68699168.1+                    CATTCCCCCTGGAGCAGAATCCTACAGATCTTATGGACTTTGCA
SRR943143.3390929.1+                       TTCCCCCTGGAGCAGAATCCTACAGATCTTATGGACTTTGCA
SRR943153.36526551.1+                      TTCCCCCTGGAGCAGAATCCTACAGATCTTATGGACTTTGCA
SRR943153.28565111.2-                       TCCCCCTGGAGCAGAATCCTACAGATCTTATGGACTTTGCA
SRR943143.38136470.2+                        CCCCCTGGAGCAGAATCCTACAGATCTTATGGACTTTGCA
SRR943143.98833310.1-                        CCCCTGGGAGCAGAATCCTACAGATCTTATGGATTTTGCA
SRR943145.5154494.1+                         CCCCCTGGAGCAGAATCCTACAGATCTTATGGACTTTGCA
SRR943146.102623222.1+                       CCCCCTGGAGCAGAATCCTACAGATCTTATGGACTTTGCA
SRR943148.87857597.1+                        CCCCCTGGAGCAGAATCCTACAGATCTTATGGACTTTGCA
SRR943153.25243409.1+                         CCCCTGGAGCAGAATCCTACAGATCTTATGGACTTTGCA
SRR943153.76099028.1+                         CCCCTGGAGCAGAATCCTACAGATCTTATGGACTTTGCA
SRR943143.13346873.2+                          CCCTGGAGCAGAATCCTACAGATCTTATGGACTTTGCA
SRR943145.33850655.2+                          CCCTGGAGCAGAATCCTACAGATCTTATGGACTTTGCA
SRR943143.29420079.2+                           CCTGGAGCAGAATCCTACAGATCTTATGGACTTTGCA
SRR943146.74416486.2+                           CCTGGAGCAGAATCCTACAGATCTTATGGACTTTGCA
SRR943153.57703573.2+                           CCTGGAGCAGAATCCTACAGATCTTATGGACTTTGCA
SRR943143.50272004.2+                            CTGGAGCAGAATCCTACAGATCTTATGGACTTTGCA
SRR943145.33050274.1+                            CTGGAGCAGAATCCTACAGATCTTATGGACTTTGCA
SRR943143.16082287.2+                              GGAGCAGAATCCTACAGATCTTATGGACTTTGCA
SRR943149.55608470.1-                                   AGAATCCTACAGATCTTATGGACTTTGCA
SRR943143.36194330.2+                                    GAATCCTACAGATCTTATGGACTTTGCA
SRR943144.130096723.2+                                   GAATCCTACAGATCTTATGGACTTTGCA
SRR943146.27451379.1+                                    GAATCCTACAGATCTTATGGACTTTGCA
SRR943148.19805333.2+                                     AATCCTACAGATCTTATGGACTTTGCA
SRR943150.39935377.2-                                      ATCCTACAGATCTTATGGACTTTGCA
SRR943145.54221246.2-                                          TACAGTTTTTAGGGACTTTGCA
SRR943144.102539740.1-                                          ACAGATCTTATGGACTTTGCA
SRR943143.8864295.1+                                             CAGATCTTATGGACTTTGCA
SRR943144.64510980.2-                                            CAGATTTTATGGACGTTGCA
```

```
SRR943146.99892651.2-                                             AGTTTTTTTGGCCTTTGCA
SRR943153.77678041.1+                                               ATCTTATGGACTTTGCA
SRR943153.101884461.1+                                               TCTTATGGACTTTGCA
SRR943148.54804425.1+                                                    ATGGACTTTGCA
SRR943148.57817572.1+                                                    ATGGACTTTGCA
SRR943149.98757513.1+                                                    ATGGACTTTGCA
SRR943143.50272004.1-                                                     GGTCTTTTGCA
SRR943143.90158177.2-                                                     GGGCTTTTGCA
SRR943145.18692254.2-                                                     GGACTTTGACA
SRR943145.48955657.1-                                                      GGTCTTTGCA
SRR943153.24661380.1+                                                      GGACTTTGCA
SRR943153.58254396.1+                                                      GGACTTTGCA
SRR943153.59925388.1+                                                      GGACTTTGCA
SRR943153.64863673.1+                                                      GGACTTTGCA
SRR943146.8831671.2+                                                         ACTTTGCA
SRR943147.8785045.2-                                                          CTTTGCA
SRR943144.53424615.2+                                                          TTTGCA
SRR943146.111835648.2-                                                         TTTGCA
SRR943146.18857659.1+                                                          TTTGCA
SRR943153.43817899.2-                                                          TTTGCA
SRR943143.13398540.1-                                                              CA
SRR943143.31714114.2+                                                              CA
SRR943144.86236498.1+                                                              CA
SRR943152.25540084.1-                                                              CA
SRR943152.46942647.1-                                                              CA
SRR943152.54814985.1-                                                              CA
SRR943152.68904552.1-                                                              CA
SRR943152.71600879.1-                                                              CA
SRR943152.76910800.1-                                                              CA
SRR943143.53168784.2-                                                               A
                         ____________________________________________________________
consensus                TTTTCCCTGTGCATTTCATTCCCCCTGGAGCAGAATCCTACAGATCTTATGGACTTTGCA

                             .    :    .    :    .    :    .    :    .    :    .    :
SRR943143.107878190.1+   A
SRR943147.36150911.2-    A
SRR943149.18673434.2+    A
SRR943146.13936469.2-    AT
SRR943145.13993241.1+    ATT
SRR943146.3316304.2+     ATTC
SRR943143.41825835.2-    ATTCC
SRR943145.6158089.1+     ATTCC
SRR943146.98785799.1+    ATTCC
SRR943148.87857597.1+    ATTCC
SRR943153.13386580.1-    ATTCC
SRR943153.24651648.1+    ATTCC
SRR943153.27887061.1+    ATTCC
SRR943153.68699168.1+    ATTCC
SRR943143.115573669.2-   ATTCCA
SRR943143.13911124.1+    ATTCCA
SRR943145.1935921.2-     ATTCCA
SRR943146.53723254.2+    ATTCCAG
SRR943153.36526551.1+    ATTCCAG
SRR943145.48367946.2-    ATTCCAGC
SRR943146.68438362.2-    ATTCCAGC
SRR943153.28565111.2-    ATTCCAGC
SRR943146.13911030.2-    ATTCCAGCC
SRR943153.25243409.1+    ATTCCAGCCA
SRR943153.76099028.1+    ATTCCAGCCA
SRR943143.78524906.1-    ATTCCAGCCAG
SRR943144.134799716.1+   ATTCCAGCCAG
SRR943146.113655658.1+   ATTCCAGCCAG
SRR943144.13635896.1+    ATTCCAGCCAGT
SRR943153.57703573.2+    ATTCCAGCCAGT
SRR943143.106665816.2-   ATTCCAGCCAGTTT
SRR943146.93080774.2+    ATTCCAGCCAGTTT
SRR943146.42700050.1+    ATTCCAGCCAGTTTGATG
SRR943145.12257330.2+    ATTCCAGCCAGTTTGATGA
SRR943145.16844422.1-    ATTCCAGCCAGTTTGATGA
SRR943149.55608470.1-    ATTCCAGCCAGTTTGATGAA
SRR943145.14723638.1+    ATTCCAGCCAGTTTGATGAAg
SRR943145.20588671.1+    ATTCCAGCCAGTTTGATGAAg
SRR943147.6502784.1+     ATTCCAGCCAGTTTGATGAAgt
SRR943148.19805333.2+    ATTCCAGCCAGTTTGATGAAgt
SRR943143.106713870.2-   ATTCCAGCCAGTTTGATGAAgtg
SRR943145.13463055.2-    ATTCCAGCCAGTTTGATGAAgtg
SRR943147.27862538.2+    ATTCCAGCCAGTTTGATGAAgtg
SRR943150.39935377.2-    ATTCCAGCCAGTTTGATGAAgtg
SRR943143.85862572.2+    ATTCCAGCCAGTTTGATGAAgtga
SRR943143.33813501.2+    ATTCCAGCCAGTTTGATGAAgtgag
SRR943147.4719698.1+     ATTCCAGCCAGTTTGATGCAgtgag
SRR943143.90158177.1+    ATTCCAGCCAGTTTGATGAAgtgagt
SRR943143.60106320.1+    ATTCCAGCCAGTTTGATGAAgtgagtg
SRR943145.25852643.2-    ATTCCAGCCAGTTTGATGAAgtgagtgtg
```

```
SRR943147.17163453.1-    ATTCCAGCCAGTTTGATGAAgtgagtgtg
SRR943146.91719564.1+    ATTCCAGCCAGTTTGATGAAgtgagtgtgggg
SRR943153.77678041.1+    ATTCCAGCCAGTTTGATGAAgtgagtgtgggg
SRR943153.101884461.1+   ATTCCAGCCAGTTTGATGAAgtgagtgtgggga
SRR943146.61065462.2-    ATTCCAGCCAGTTTGATGAAgtgagtgtggggac
SRR943143.113297297.1-   ATTCCAGCCAGTTTGATGAAgtgagtgtggggaca
SRR943147.14001746.1-    ATTCCAGCCAGTTTGATGAAgtgagtgtggggaca
SRR943148.54804425.1+    ATTCCAGCCAGTTTGATGAAgtgagtgtggggacagc
SRR943148.57817572.1+    ATTCCAGCCAGTTTGATGAAgtgagtgtggggacagc
SRR943149.98757513.1+    ATTCCAGCCAGTTTGATGAAgtgagtgtggggacagc
SRR943143.110177944.1-   ATTCCAGCCAGTTTGATGAAgtgagtgtggggacagcc
SRR943153.24661380.1+    ATTCCAGCCAGTTTGATGAAgtgagtgtggggacagcca
SRR943153.58254396.1+    ATTCCAGCCAGTTTGATGAAgtgagtgtggggacagcca
SRR943153.59925388.1+    ATTCCAGCCAGTTTGATGAAgtgagtgtggggacagcca
SRR943153.64863673.1+    ATTCCAGCCAGTTTGATGAAgtgagtgtggggacagcca
SRR943143.13398540.2+    ATTCCAGCCAGTTTGATGAAgtgagtgtggggacagccat
SRR943143.26110525.2-    ATTCCAGCCAGTTTGATGAAgtgagtgtggggacagccatac
SRR943153.43817899.2-    ATCCCAGCCAGTTTGATGAAgtgagtgtggggccagccatacg
SRR943146.50372746.1+    ATTCCAGCCAGTTTGATGAAgtgagtgtggggacagccatacga
SRR943147.63110318.2+    ATTCCAGCCAGTTTGATGAAgtgagtgtggggacagccctacga
SRR943147.22932053.2+    ATTCCAGCCAGTTTGATGAAgtgagtgtggggcaagccatacgag
SRR943143.53168784.1+    ATTCCAGCCAGTTTGATGAAgtgagtgtggggacagccatacgaga
SRR943143.85000359.2-    ATTCCGGCCAGTTTGATGAAgtgagtgtggggacagccatacgaga
SRR943152.25540084.1-    ATCCCAGCCAGTTTGATGAAgtgagtgtggggacagccatacgagat
SRR943152.46942647.1-    ATTCCAGCCAGTTTGATGAAgtgagtgtggggacagccatacgagat
SRR943152.54814985.1-    ATCCCAGCCAGTTTGATGAAgtgagtgtggggacagccatacgagat
SRR943152.68904552.1-    ATCCCAGCCAGTTTGATGAAgtgagtgtggggacagccatacgagat
SRR943152.71600879.1-    ATCCCAGCCAGTTTGATGAAgtgagtgtggggacagccatacgagat
SRR943152.76910800.1-    ATCCCAGCCAGTTTGATGAAgtgagtgtggggacagccatacgagat
SRR943151.28175422.1+    ATTCCAGCCAGTTTGATGAAgtgagtgtggggacagccatacgagatcc
SRR943151.59977855.1+    ATTCCAGCCAGTTTGATGAAgtgagtgtggggacagccatacgagatcc
SRR943153.33066696.1-    ATTCCAGCCAGTTTGATGAAgtgagtgtggggacagccatacgagatcc
SRR943153.92497752.1-    ATTCCAGCCAGTTTGATGAAgtgagtgtggggacagccatacgagatcc
SRR943144.46004729.2-    ATTCCAGCCAGTTTGATGAAgtgagtgtggggacagccatacgagatcct
SRR943146.99173336.1+    ATTCCAGCCAGTTTGATGAAgtgagggtggggacagccatacgagaccctt
SRR943146.4306804.2+     ATTCCAGCCAGTTTGATGAAgtgagtgtggggacagccatacgagatccttc
SRR943144.134799716.2-   ATTCCAGCCAGTTTGATGAAgtgagagtggggacagccatacgagatccttctt
SRR943144.13635896.2-    ATTCCAGCCAGTTTGATGAAgtgagtgtggggacagccatacgagatccttcttg
SRR943146.78904130.1+    ATTCCAGCCAGTTTGATGGAgtgagtgtggggacagccatacgagatccttcttg
SRR943143.24007821.2+    ATTCCAGCCAGTTTGATGAAgtgagtgtggggacagccatacgagatccttcttga
SRR943143.3390929.1+     ATTCCAGCCAGTTTGATGAAgtgagtgtggggacagccatacgagatccttcttgatc
SRR943143.98833310.1-    TTTCCAGCCAGTTTGATGAAgtgagtgtggggacagccatacgagatccttcttgatc
SRR943143.13346873.2+    ATTCCAGCCAGTTTGATGAAgtgagtgtggggacagccatacgagatccttcttgatctt
SRR943143.13398540.1-    ATCCCAGCCAGTTTGATGAAgtgagtgtggggacagccatacgagatccttcttgatctt
SRR943143.16082287.2+    ATTCCAGCCAGTTTGATGAAgtgagtgtggggacagccatacgagatccttcttgatctt
SRR943143.29420079.2+    ATTCCAGCCAGTTTGATGAAgtgagtgtggggacagccatacgagatccttcttgatctt
SRR943143.31714114.2+    ATTCCAGCCAGTTTGATGAAgtgagtgtggggacagccatacgagatccttcttgatctt
SRR943143.36194330.2+    ATTCCAGCCAGTTTGATGAAgtgagtgtggggacagccatacgagatccttcttgatctt
SRR943143.38136470.2+    ATTCCAGCCAGTTTGATGAAgtgagtgtggggacagccatacgagatccttcttgatctt
SRR943143.50272004.1-    TTTCCAGCCAGTTTGATGAAgtgagtgtggggacagccatacgagatccttcttgatctt
SRR943143.50272004.2+    ATTCCAGCCAGTTTGATGAAgtgagtgtggggacagccatacgagatccttcttgatctt
SRR943143.53168784.2-    TTCCCAGCCAGTTTGATGAAgtgagtgtggggacagccatacgagatccttcttgatctt
SRR943143.60106320.2-    TTACCAGCCAGTTTGATGAAgtgagtgtggggacagccatacgagatccttcttgatctt
SRR943143.8864295.1+     ATTCCAGCCAGTTTGATGAAgtgagtgtggggacagccatacgagatccttcttgatctt
SRR943143.90158177.2-    ATCCCAGCCAGTTTGATGAAgtgagtgtggggacagccatacgagatccttcttgatctt
SRR943144.102539740.1-   ATTCCAGCCAGTTTGATGAAgtgagtgtggggacagccatacgagatccttcttgatctt
SRR943144.102539740.2+   ATTCCAGCCAGTTTGATGAAgtgagtgtggggacagccatacgagatccttcttgatctt
SRR943144.130096723.2+   ATTCCAGCCAGTTTGATGAAgtgagtgtggggacagccatacgagatccttcttgatctt
SRR943144.139888000.1+   ATTCCAGCCAGTTTGATGAAgtgagtgtggggacagccatacgagatccttcttgatctt
SRR943144.139888000.2-   ATTCCAGCCAGTTTGATGAAgtgagtgtggggacagccatacgagatccttcttgatctt
SRR943144.53424615.2+    ATTCCAGCCAGTTTGATGAAgtgagtgtggggacagccatacgagatccttcttgatctt
SRR943144.64510980.1+    ATTCCAGCCAGTTTGATGAAgtgagtgtggggacagccatacgagatccttcttgatctt
SRR943144.64510980.2-    ATTCCAGCCAGTTTGATGAAgtgagtgtggggacagccatacgagatccttcttgatctt
SRR943144.86236498.1+    ATTCCAGCCAGTTTGCTGAAgtgagtgtggggacagccatacgagatccttcttgatctt
SRR943145.18692254.2-    TTCCCAGCCAGTTTGATGAAgtgagtgtggggacagccatacgagatccttcttgatctt
SRR943145.33050274.1+    ATTCCAGCCAGTTTGATGAAgtgagtgtggggacagccatacgagatccttcttgatctt
SRR943145.33850655.2+    ATTCCAGCCAGTTTGATGAAgtgagtgtggggacagccatacgagatccttcttgatctt
SRR943145.48955657.1-    ATCCCAGCCAGTTTGATGAAgtgagtgtggggacagccatacgagatccttcttgatctt
SRR943145.5154494.1+     ATTCCAGCCAGTTTGATGAAgtgagtgtggggacagccatacgagatccttcttgatctt
SRR943145.54221246.2-    TTTCCAGCCAGTTTGATGAAgtgagtgtggggacagccatacgagatccttcttgatctt
SRR943145.60888623.2+    ATTCCAGCCAGTTTGATGAAgtgagtgtggggacagccatacgagatccttcttgatctt
SRR943146.102623222.1+   ATTCCAGCCAGTTTGATGAAgtgagtgtggggacagccatacgagatccttcttgatctt
SRR943146.111835648.2-   ATCCCAGCCAGTTTGATGAAgtgagtgtggggacagccatacgagatccttcttgatctt
SRR943146.18857659.1+    ATTCCAGCCAGTTTGATGAAgtgagtgtggggacagccatacgagatccttcttgatctt
SRR943146.27451379.1+    ATTCCAGCCAGTTTGATGAAgtgagtgtggggacagccatacgagatccttcttgatctt
SRR943146.74416486.2+    ATTCCAGCCAGTTTGATGAAgtgagtgtggggacagccatacgagatccttcttgatctt
SRR943146.8831671.2+     ATTCCAGCCAGTTTGATGAAgtgagtgtggggacagccatacgagatccttcttgatctt
SRR943146.99892651.2-    ATGCCAGCCAGTTTGATGAAgtgagtgtgggcacagccatacgagatccttcttgatctt
SRR943147.53877219.2+    ATTCCAGCCAGTTTGATGAAgtgagtgtggggacagccatacgagatccttcttgatctt
SRR943147.8785045.2-     ATCCCAGCCAGTTTGATGAAgtgagtgtggggacagccatacgagatccttcttgatctt
SRR943153.17907903.2-      TCCAGCCAGTTTGATGAAgtgagtgtggggacagccatacgagatcctt
SRR943147.38791632.1-      CCCAGCCAGTTTGATGAAgtgagtgtggggacagccatacgagatccttcttgatctt
SRR943143.12052790.1+        CAGCCAGTTTGATGAAgtgagtgtggggacagccatacgagatccttcttgatctt
SRR943143.24007821.1-          GCCAGTTTGATGAAgtgagtgtggggacagccatacgagatccttcttgatctt
```

```
SRR943145.50345691.1-          GCCATTTTGATGAAgtgagtgtggggacagccatacgagatccttcttgatctt
SRR943150.12536979.1+           CCAGTTTGATGAAgtgagtgtggggacagccatacgagatccttcttga
SRR943150.54616047.1+           CCAGTTTGATGAAgtgagtgtggggacagccatacgagatccttcttga
SRR943145.8082094.1+            CCAGTTTGATGAAgtgagtgtggggacagccatacgagatccttcttgatctt
SRR943143.38136470.1-            CAGTTTGATGAAgtgggtgtggggacagccatacgagatccttcttgatctt
SRR943147.7282744.1-              AGTTTTATGAAttgagtgtggggccagccatacgagatccttcttgatctt
SRR943149.95711274.2-              GTTTGATGAAgtgagtgtggggacagccatacgagatccttcttgatct
SRR943143.9128684.1+               GTTTGATGAAgtgagtgtggggacagccatacgagatccttcttgatctt
SRR943143.79035346.2+                TTGATGAAgtgagtgtggggacagccatacgagatccttcttgatctt
SRR943146.103192666.2+               TTGATGAAgtgagtgtggggacagccatacgagatccttcttgatctt
SRR943143.3390929.2-                  TGATGAAgtgagtggggggacagccatacgagatccttcttgatctt
SRR943143.42964168.1+                 TGATGAAgtgagtgtggggacagccatacgagatccttcttgatctt
SRR943144.130096723.1-                TGAGGAAgtgagtgtggtgacagccatacgagatccttcttgatctt
SRR943149.21814629.2+                  GATGAAgtgagtgtggggacagccatacgagatccttcttgatctt
SRR943144.64588945.2+                    TGAAgtgagtgtggggacagccatacgagatccttcttgatctt
SRR943152.101429198.2-                    GAAgtgagtgtggggacagccatacgagatccttcttgatctt
SRR943152.11237162.1-                     GAAgtgagtgtggggacagccatacgagatccttcttgatctt
SRR943152.12196632.2-                     GAAgtgagtgtggggacagccatacgagatccttcttgatctt
SRR943152.21928907.2-                     GAAgtgagtgtggggacagccatacgagatccttcttgatctt
SRR943152.48151300.2-                     GAAgtgagtgtggggacagccatacgagatccttcttgatctt
SRR943152.48806058.1-                     GAAgtgagtgtggggacagccatacgagatccttcttgatctt
SRR943152.49173807.2-                     GAAgtgagtgtggggacagccatacgagatccttcttgatctt
SRR943152.58724879.2-                     GAAgtgagtgtggggacagccatacgagatccttcttgatctt
SRR943152.61620229.1-                     GAAgtgagtgtggggacagccatacgagatccttcttgatctt
SRR943152.64769066.2-                     GAAgtgagtgtggggacagccatacgagatccttcttgatctt
SRR943152.70954658.1-                     GAAgtgagtgtggggacagccatacgagatccttcttgatctt
SRR943152.778807.2-                       GAAgtgagtgtggggacagccatacgagatccttcttgatctt
SRR943152.80098221.1-                     GAAgtgagtgtggggacagccatacgagatccttcttgatctt
SRR943152.8247684.1-                      GAAgtgagtgtggggacagccatacgagatccttcttgatctt
SRR943152.84824452.2-                     GAAgtgagtgtggggacagccatacgaggtccttcttgatctt
SRR943152.87009145.1-                     GAAgtgagtgtggggccagccatacgagatccttcttgatctt
SRR943152.9538714.1-                      GAAgtgagtgtggggacagccatacgagatccttcttgatctt
SRR943152.98870823.2-                     GAAgtgagtgtggggacagccatacgagatccttcttgatctt
SRR943148.2350091.1-                        Agtgagtgtggggacagccatacgagatccttcttgatctt
SRR943148.5569000.1-                        Agtgagtgtggggacagccatacgagatccttcttgatctt
SRR943148.58356454.1-                       Agtgagtgtggggacagccatacgagatccttcttgatctt
SRR943148.64896133.1-                       Agtgagtgtggggacagccatacgagatccttcttgatctt
SRR943149.22618660.2-                       Agtgagtgtggggccagccatacgagatccttcttgatctt
                         ____________________________________________________________
consensus                ATTCCAGCCAGTTTGATGAAgtgagtgtggggacagccatacgagatccttcttgatctt
```

W. Brown mesite (No. 49), exon 7, WGS data

- CAP3 alignment of WGS data spanning exon 1 (uppercase letters)

```
                             .    :    .    :    .    :    .    :    .    :    .    :
SRR959125.71349998.1-    tcattgctgaaggagtaggcatttttcttttacagGTTTG
SRR959125.1164371.1-     tcattgctgaaggagtaggcatttttcttttacagGTTTGAT
SRR959126.92092290.2+    tcattgctgaaggagtaggcatttttcttttacagGTTTGAT
SRR959126.64530040.1-    tcattgctgaaggagtaggcatttttcttttacagGTTTGATCG
SRR959125.92615954.2+    tcattgctgaaggagtaggcatttttcttttacagGTTTGATCGACAGCTCAG
SRR959126.95115239.2+    tcattgctgaaggagtaggcatttttcttttacagGTTTGATCGACAGCTCAGG
SRR959125.3288420.1-     tcattgctgaaggagtaggcatttttcttttacagGTTTGATCGACAGCTCAGGGG
SRR959125.6127856.1+     tcattgctgaaggagtaggcatttttcttttacagGTTTGATCGACAGCTCAGGGG
SRR959125.23356476.2-    tcattgctgaaggagtaggcatttttcttttacagGTTTGATCGACAGCTCAGGGGTACA
SRR959125.51502940.1+    tcattgctgaaggagtaggcatttttcttttacagGTTTGATCGACAGCTCAGGGGTACA
SRR959125.66142241.1+    tcattgctgaaggagtaggcatttttcttttacagGTTTGATCGACAGCTCCGGGGTACA
SRR959125.89783208.1-    tcattgctgaaggagtaggcatttttcttttacagGTTTGATCGACAGCTCAGGGGTACA
SRR959126.20383382.2+    tcattgctgaaggagtaggcatttttcttttacagGTTTGATCGACAGCTCAGGGGTACA
SRR959126.24478131.2+    tcattgctgaaggagtaggcatttttcttttacagGTTTGATCGACAGCTCAGGGGTACA
SRR959126.89114076.2-    tcattgctgaaggagtaggcatttttcttttacagGTTTGATCGACAGCTCAGGGGTACA
SRR959126.3757619.1-          gctgaaggagtaggcatttttcttttacagGTTTGATCGACAGCTCAGGGGTACA
SRR959126.55740614.2+             aaggagtaggcatttttcttttacagGTTTGATCGACAGCTCAGGGGTACA
SRR959125.65657973.1+                   taggcatttttcttttacagGTTTGATCGACAGCTCAGGGGTACA
SRR959125.14374764.2+                          ttttcttttacagGTTTGATCGACAGCTCAGGGGTACA
SRR959125.53032334.1+                          ttttcttttacagGTTTGATCGACAGCTCAGGGGGACA
SRR959125.68278836.1-                            ttcttttacagGTTTGATCGACAGCTCAGGGGTACA
SRR959126.5384006.2-                                  ttacagGTTTGATCGACAGCTCAGGGGTACA
SRR959126.36334469.2+                                   acagGTTTGATCGACAGCTCAGGGGTACA
SRR959126.8197972.1+                                    acagGTTTGATCGACAGCTCAGGGGTACA
SRR959125.69939890.2-                                         TTGATCGACAGCTCAGGGGTACA
SRR959125.87896316.1-                                             TCGACAGCTCAGGGGTACA
SRR959125.56686998.1+                                               GACAGCTCAGGGGTACA
SRR959126.31869404.1-                                               GACAGCTCAGGGGTACA
SRR959126.68690197.2+                                                ACAGCTCAGGGGTACA
SRR959126.82431856.2-                                                   GCTCAGGGGTACA
SRR959126.65885852.2+                                                      CAGGGGTACA
SRR959125.20165755.1-                                                       AGGGGTACA
SRR959126.74768132.2+                                                       AGGGGTACA
SRR959126.20088646.1+                                                        GGGGTACA
SRR959126.54438604.2+                                                          GGTACA
                         ____________________________________________________________
consensus                tcattgctgaaggagtaggcatttttcttttacagGTTTGATCGACAGCTCAGGGGTACA

                             .    :    .    :    .    :    .    :    .    :    .    :
SRR959125.51502940.1+    A
SRR959125.66142241.1+    A
SRR959126.24478131.2+    AA
SRR959126.89114076.2-    AATCTA
SRR959126.20383382.2+    AATCTAAAACACTTCAGA
SRR959125.89783208.1-    AATCTAAAACACTTCAGAGCTACGGAA
SRR959125.23356476.2-    AATCTAAAACACTTCAGAGCTACGGAAATA
SRR959126.3757619.1-     AATCTAAAACACTTCAGAGCTACGGAAATATGATGTGGGGGTTCT
SRR959126.55740614.2+    AATCTAAAACACTTCAGAGCTACGGAAATATGATGTGGGGGTTCTGCAA
SRR959125.65657973.1+    AATCTAAAACACTTCAGAGCTACGGAAATATGATGTGGGGGTTCTGCAAACAGGC
SRR959125.14374764.2+    AATCTAAAACACTTCAGAGCTACGGAAATATGATGTGGGGGTTCTGCAAACAGGCATCTT
SRR959125.20165755.1-    AATCTAAAACACTTCAGAGCTACGGAAATATGATGTGGGGGTTCTGCAAACAGGCATCTT
SRR959125.53032334.1+    AATCTAAAACACTTCAGAGCTACGGAAATATGATGTGGGGGTTCTGCAAACAGGCATCTT
SRR959125.56686998.1+    AATCTAAAACACTTCAGAGCTACGGAAATATGATGTGGGGGTTCTGCAAACAGGCATCTT
SRR959125.68278836.1-    AATCTAAAACACTTCAGAGCTACGGAAATATGATGTGGGGGTTCTGCAAACAGGCATCTT
SRR959125.69939890.2-    AATCTAAAACACTTCAGAGCTACGGAAATATGATGTGGGGGTTCTGCAAACAGGCATCTT
SRR959125.87896316.1-    AATCTAAAACACTTCAGAGCTACGGAAATATGATGTGGGGGTTCTGCAAACAGGCATCTT
SRR959126.20088646.1+    AATCTAAAACACTTCAGAGCTACGGAAATATGATGTGGGGGTTCTGCAAACGGGCATCTT
SRR959126.31869404.1-    AATCTAAAACACTTCAGAGCTACGGAAATATGATGTGGGGGTTCTGCAAACAGGCATCTT
SRR959126.36334469.2+    AATCTAAAACACTTCAGAGCTACGGAAATATGATGTGGGGGTTCTGCAAACAGGCATCTT
SRR959126.5384006.2-     AATCTAAAACACTTCAGAGCTACGGAAATATGATGTGGGGGTTCTGCAAACAGGCATCTT
SRR959126.54438604.2+    AATCTAAAACACTTCAGAGCTACGGAAATATGATGTGGGGGTTCTGCAAACAGGCATCTT
SRR959126.65885852.2+    AATCTAAAACACTTCAGAGCTACGGAAATATGATGTGGGGGTTCTGCAAACAGGCATCTT
SRR959126.68690197.2+    AATCTAAAACACTTCAGAGCTACGGAAATATGATGTGGGGGTTCTGCAAACAGGCATCTT
SRR959126.74768132.2+    AATCTAAAACACTTCAGAGCTACGGAAATATGATGTGGGGGTTCTGCAAACAGGCATCTT
SRR959126.8197972.1+     AATCTAAAACACTTCAGAGCTACGGAAATATGATGTGGGGGTTCTGCAAACAGGCATCTT
SRR959126.82431856.2-    AATCTAAAACACTTCAGAGCTACGGAAATATGATGTGGGGGTTCTGCAAACAGGCATCTT
SRR959126.22419777.2+       CTAAAACACTTCAGAGCTACGGAAATATGATGTGGGGGTTCTGCAAACAGGCATCTT
SRR959125.304875.1+          TAAAACACTTCAGAGCTACGGAAATATGATGTGGGGGTTCTGCAAACAGGCATCTT
SRR959126.49581060.2+         AAAACACTTCAGAGCTACGGAAATATGATGTGGGGGTTCTGCAAACAGGCATCTT
SRR959125.49130245.2-          AAACGCTTCAGAGCTACGGAAATATGATGTGGGGGTTCTGCAAACAGGCATCTT
SRR959126.78363057.2+            ACACTTCAGAGCTACGGAAATATGATGTGGGGGTTCTGCAAACAGGCATCTT
SRR959126.30956045.1-                   AGAGCTACGGAAATATGATGTGGGGGTTCTGCAAACAGGCATCTT
SRR959125.26644278.2+                    GAGCTACGGAAATATGATGTGGGGGTTCTGCAAACAGGCATCTT
SRR959126.16912081.1-                    GAGCTACGGAAATATGATGTGGGGGTTCTGCAAACAGGCATCTT
SRR959126.80285931.1-                    GAGCTACGGAAATATGATGTGGGGGTTCTGCAAACAGGCATCTT
```

```
SRR959125.61805220.1-                     AGCTACGGAAATATGATGTGGGGGTTCTGCAAACAGGCATCTT
SRR959125.16836203.1-                      GCTACGGAAATATGATGTGGGGGTTCTGCAAACAGGCATCTT
SRR959125.10949088.1-                       CTACGGAAATATGATGTGGGGGTTCTGCAAACAGGCATCTT
SRR959126.96154607.2+                       CTACGGAAATATGATGTGGGGGTTCTGCAAACAGGCATCTT
SRR959126.32715419.2-                              AATATGATGTGGGGGTTCTGCAAACAGGCATCTT
SRR959126.34803302.1+                                 ATGATGTGGGGGTTCTGCAAACAGGCATCTT
SRR959126.68696902.2-                                 ATGATGTGGGGGTTCTGCAAACAGGCATCTT
SRR959125.61297419.2+                                     TGTGGGGGTTCTGCAAACAGGCATCTT
SRR959126.39146429.2+                                     TGTGGGGGTTCTGCAAACAGGCATCTT
SRR959126.83495126.1-                                     TGTGGGGGTTCTGCAAACAGGCATCTT
SRR959125.557549.1+                                        GTGGGGGTTCTGCAAACAGGCATCTT
SRR959126.45296095.1+                                       TGGGGGTTCTGCAAACAGGCATCTT
SRR959125.81390318.1+                                           GGTTCTGCAAACAGGCATCTT
SRR959126.17241068.2+                                           GGTTCTGCAAACAGGCATCTT
SRR959125.82224539.1+                                             TTCTGCAAACAGGCATCTT
SRR959125.10854535.2+                                               CTGCAAACAGGCATCTT
SRR959125.35797186.2+                                                    AACAGGCATCTT
SRR959125.12611392.1+                                                     ACAGGCATCTT
SRR959125.54037503.1-                                                     ACAGGCATCTT
SRR959125.86194665.2-                                                      CAGGCATCTT
SRR959125.21450564.1-                                                             CTT
                         ____________________________________________________________
consensus                AATCTAAAACACTTCAGAGCTACGGAAATATGATGTGGGGGTTCTGCAAACAGGCATCTT

                             .    :    .    :    .    :    .    :    .    :    .    :
SRR959125.14374764.2+    CA
SRR959125.53032334.1+    CA
SRR959125.68278836.1-    CACT
SRR959126.5384006.2-     CACTCTCCC
SRR959126.36334469.2+    CACTCTCCCTG
SRR959126.8197972.1+     CACTCTCCCTG
SRR959125.69939890.2-    CACTCTCCCTGTGCATT
SRR959125.87896316.1-    CACTCTCCCTGTGCATTTCAT
SRR959125.56686998.1+    CACTCTCCCTGTGCATTTCATTC
SRR959126.31869404.1-    CACTCTCCCTGTGCATTTCATTC
SRR959126.68690197.2+    CACTCTCCCTGTGCATTTCATTCC
SRR959126.82431856.2-    CACTCTCCCTGTGCATTTCATTCCTCT
SRR959126.65885852.2+    CACTCTCCCTGTGCATTTCATTCCTCTTGG
SRR959125.20165755.1-    CACTCTCCCTGTGCATTTCATTCCTCTTGGA
SRR959126.74768132.2+    CACTCTCCCTGTGCATTTCATTCCTCTTGGA
SRR959126.20088646.1+    CACTCTCCCTGTGCATTTCATTCCTCTTGGAG
SRR959126.54438604.2+    CACTCTCCCTGTGCATTTCATTCCTCTTGGAGCA
SRR959125.49130245.2-    CACTCTCCCTGTGCATTTCAATCCTCTTGGAGCAGTATCCTA
SRR959126.22419777.2+    CACTCTCCCTGTGCATTTCATTCCTCTTGGAGCAGTATCCTAC
SRR959125.304875.1+      CACTCTCCCTGTGCATTTCATTCCTCTTGGAGCAGTATCCTACA
SRR959126.49581060.2+    CACTCTCCCTGTGCATTTCATTCCTCTTGGAGCAGTATCCTACAG
SRR959126.78363057.2+    CACTCTCCCTGTGCATTTCATTCCTCTTGGAGCAGTATCCTACAGATC
SRR959126.30956045.1-    CACTCTCCCTGTGCATTTCATTCCTCTTGGAGCAGTATCCTACAGATCTCATGGT
SRR959125.26644278.2+    CACTCTCCCTGTGCATTTCATTCCTCTTGGAGCAGTATCCTACAGATCTCATGGTC
SRR959126.16912081.1-    CACTCTCCCTGTGCATTTCATTCCTCTTGGAGCAGTATCCTACAGATCTCATGGTC
SRR959126.80285931.1-    CACTCTCCCTGTGCATTTCATTCCTCTTGGAGCAGTATCCTACAGATCTCATGGTC
SRR959125.61805220.1-    CACTCTCCCTGTGCATTTCATTCCTCTTGGAGCAGTATCCTACAGATCTCATGGTCT
SRR959125.16836203.1-    CACTCTCCCTGTGCATTTCATTCCTCTTGGAGCAGTATCCTACAGATCTCATGGTCTT
SRR959125.10949088.1-    CACTCTCCCTGTGCATTTCATTCCTCTTGGAGCAGTATCCTACAGATCTCATGGTCTTT
SRR959126.96154607.2+    CACTCTCCCTGTGCATTTCATTCCTCTTGGAGCAGTATCCTACAGATCTCATGGTCTTT
SRR959125.10854535.2+    CACTCTCCCTGTGCATTTCATTCCTCTTGGAGCAGTATCCTACAGATCTCATGGTCTTTA
SRR959125.12611392.1+    CACTCTCCCTGTGCATTTCATTCCTCTTGGAGCAGTATCCTACAGATCTCATGGTCTTTA
SRR959125.21450564.1-    CCCTCTCCCTGTGCATTTCATTCCTCTTGGACCAGTATCCTACAGATCTCATGGTCTTTA
SRR959125.35797186.2+    CACTCTCCCTGTGCATTTCATTCCTCTTGGAGCAGTATCCTACAGATCTCATGGTCTTTA
SRR959125.54037503.1-    CCCTCTCCCTGTGCATTTCATTCCTCTTGGAGCAGTATCCTACAGATCTCATGGTCTTTA
SRR959125.557549.1+      CACTCTCTCTGTGCATTTCATTCCTCTTGGAGCAGTATCCTACAGATCTCATGGTCTTTA
SRR959125.61297419.2+    CACTCTCCCTGTGCATTTCATTCCTCTTGGAGCAGTATCCTACAGATCTCATGGTCTTTA
SRR959125.81390318.1+    CACTCTCCCTGTGCATTTCATTCCTCTTGGAGCAGTATCCTACAGATCTCATGGTCTTTA
SRR959125.82224539.1+    CACTCTCCCTGTGCATTTCATTCCTCTTGGAGCAGTATCCTACAGATCTCATGGTCTTTA
SRR959125.86194665.2-    CACTCTCCGTGTGCATTTCATTCCTCTTGGAGCAGTATCCTACAGATCTCATGGTCTTTA
SRR959126.17241068.2+    CACTCTCCCTGTGCATTTCATTCCTCTTGGAGCAGTATCCTACAGATCTCATGGTCTTTA
SRR959126.32715419.2-    CACTCTCCCTGTGCATTTCATTCCTCTTGGAGCAGTATCCTACAGATCTCATGGTCTTTA
SRR959126.34803302.1+    CACTCTCCCTGTGCATTTCATTCCTCTTGGAGCAGTATCCTACAGATCTCATGGTCTTTA
SRR959126.39146429.2+    CACTCTCCCTGTGCATTTCATTCCTCTTGGAGCAGTATCCTACAGATCTCATGGTCTTTA
SRR959126.45296095.1+    CACTCTCCCTGTGCATTTCATTCCTCTTGGAGCAGTATCCTACAGATCTCATGGTCTTTA
SRR959126.60824339.2-    CCCTCTCCCTGTGCATTTCATTCCTCTTGGAGCAGTATCCTACAGATCTCATGGTCTTTA
SRR959126.68696902.2-    CACTCTCCCTGTGCATTTCATTCCTCTTGGAGCAGTATCCTACAGATCTCATGGTCTTTA
SRR959126.83495126.1-    CACTCTCCCTGTGCATTTCATTCCTCTTGGAGCAGTATCCTACAGATCTCATGGTCTTTA
SRR959125.23563924.1+     ACTCTCCCTGTGCATTTCATTCCTCTTGGAGCAGTATCCTACAGATCTCATGGTCTTTA
SRR959126.47554465.1-       TCTCCCTGGTCATTTCATTCCTCTTGGAGCAGTATCCTACAGATCTCATGGTCTTTA
SRR959126.90756317.1-        CTCCCTGTGCATTTCATTCCTCTTGGAGCAGTATCCTACAGATCTCATGGTCTTTA
SRR959126.91861988.2-                        TTCCTCTGGGAGCAGTATCCTACAGATCTCATGGTCTTTA
SRR959126.14781749.1-                         TCCTCTTGGAGCAGTATCCTACAGATCTCATGGTCTTTA
SRR959126.20228989.2+                          CCTCTTGGAGCAGTATCCTACAGATCTCATGGTCTTTA
SRR959126.97490198.2-                            TCTTGGAGCAGTATCCTACAGATCTCATGGTCTTTA
SRR959126.21560744.2-                                 GAGCAGTATCCTACAGATCTCATGGTCTTTA
SRR959126.13996252.1+                                  AGCAGTATCCTACAGATCTCATGGTCTTTA
SRR959125.68714028.2-                                            TACAGATCTCATGGCCTTTA
SRR959125.78110571.1-                                              CAGATCTCAGGGTCTTTA
```

```
SRR959125.20973158.2+                                                 ATCTCATGGTCTTTA
SRR959126.8512286.1+                                                   TCTCATGGTCTTTA
SRR959125.63671987.2-                                                   CTCATGGTCTTTA
SRR959126.85771875.1+                                                     CATGGTCTTTA
SRR959125.30512043.1-                                                       TGGTCTTTA
SRR959125.36889549.2-                                                          TCTTTA
SRR959125.13372985.2-                                                            TTTA
SRR959126.17203992.2+                                                            TTTA
SRR959126.19663196.2-                                                             TTA
SRR959126.67507656.1-                                                               A
                         ------------------------------------------------------------
consensus                CACTCTCCCTGTGCATTTCATTCCTCTTGGAGCAGTATCCTACAGATCTCATGGTCTTTA

                             .    :    .    :    .    :    .    :    .    :    .    :
SRR959126.32715419.2-    CAATTC
SRR959126.34803302.1+    CAATTCCAG
SRR959126.68696902.2-    CAATTCCAG
SRR959125.61297419.2+    CAATTCCAGCCAG
SRR959126.39146429.2+    CAATTCCAGCCAG
SRR959126.83495126.1-    CAATTCCAGCCAG
SRR959125.557549.1+      CAATTCCAGCCAGT
SRR959126.45296095.1+    CAATTCCAGCCAGTT
SRR959125.81390318.1+    CAATTCCAGCCAGTTTGAT
SRR959126.17241068.2+    CAATTCCAGCCAGTTTGAT
SRR959125.82224539.1+    CAATTCCAGCCAGTTTGATGA
SRR959125.10854535.2+    CAATTCCAGCCAGTTTGATGAAg
SRR959125.35797186.2+    CAATTCCAGCCAGTTTGATGAAgtgagt
SRR959125.12611392.1+    CAATTCCAGCCAGTTTGATGAAgtgagtg
SRR959125.54037503.1-    CAATTCCAGCCAGTTTGATGAAgtgagtg
SRR959125.86194665.2-    CAATTCCAGCCAGTTTGATGAAgtgagtgt
SRR959125.21450564.1-    CAATTCCAGCCAGTTTGATGAAgtgagtgtggggaaa
SRR959126.60824339.2-    CAATTCCAGCCAGTTTGATGAAgtgagtgtggggaaagac
SRR959125.23563924.1+    CAATTCCAGCCAGTTTGATGAAgtgagtgtggggaaagaca
SRR959126.47554465.1-    CAATTCCAGCCAGTTTGATGAAgtgagtgtggggaaagacatg
SRR959126.90756317.1-    CAATTCCAGCCAGTTTGATGAAgtgagtgtggggaaagacatgc
SRR959125.13372985.2-    CAATCCCAGCCAGTTTGATGAAgtgagtgtggggaaagacatgcaaaatccttcttgatc
SRR959125.20973158.2+    CAATTCCAGCCAGTTTGATGAAgtgagtgtggggaaagacatgcaaaatccttcttgatc
SRR959125.30512043.1-    CAATTCCAGCCAGTTTGATGAAgtgagtgtggggaaagacatgcaaaatccttcttgatc
SRR959125.36889549.2-    CAATTCCAGCCAGTTTGATGAAgtgagtgtggggaaagacatgcaaaatccttcttgatc
SRR959125.63671987.2-    CAATCCCAGCCAGTTTGATGAAgtgagtgtggggaaagacatgcaaaatccttcttgatc
SRR959125.68714028.2-    CAATTCCAGCCAGTTTGATGAAgtgagtgtggggaaagacatgcaaaatccttcttgatc
SRR959125.78110571.1-    CAATTCCAGCCAGTTTGATGAAgtgagtgtggggaaagacatgcaaaatccttcttgatc
SRR959126.13996252.1+    CAATTCCAGCCAGTTTGATGAAgtgagtgtggggaaagacatgcaaaatccttcttgatc
SRR959126.14781749.1-    CAATTCCAGCCAGTTTGATGAAgtgagtgtggggaaagacatgcaaaatccttcttgatc
SRR959126.17203992.2+    CAATTCCAGCCAGTTTGATGAAgtgagtgtggggaaagacatgcaaaatccttcttgatc
SRR959126.19663196.2-    CAATTCCAGCCAGTTTGATGAAgtgagtgtggggaaagacatgcaaaatccttcttgatc
SRR959126.20228989.2+    CAATTCCAGCCAGTTTGATGAAgtgagtgtggggaaagacatgcaaaatccttcttgatc
SRR959126.21560744.2-    CAATTCCAGCCAGTTTGATGAAgtgagtgtggggaaagacatgcaaaatccttcttgatc
SRR959126.67507656.1-    CAATTCCAGCCAGTTTGATGAAgtgagtgtggggaaagacatgcaaaatccttcttgatc
SRR959126.8512286.1+     CAATTCCAGCCAGTTTGATGAAgtgagtgtggggaaagacatgcaaaatccttcttgatc
SRR959126.85771875.1+    CAATTCCAGCCAGTTTGATGAAgtgagtgtggggaaagacatgcaaaatccttcttgatc
SRR959126.91861988.2-    CAATTCCAGCCAGTTTGATGAAgtgagtgtggggaaagacatgcaaaatccttcttgatc
SRR959126.97490198.2-    CAATTCCAGCCAGTTTGATGAAgtgagtgtggggaaagacatgcaaaatccttcttgatc
SRR959125.20520770.1-      ATTCCAGCCAGTTTGAAGAAgtgagtgtggggaaagacatgcaaaatccttcttgatc
SRR959125.66850473.1-         CCAGCCAGTTTGATGAAgtgagtgtggggaaagacatgcaaaatccttcttgatc
SRR959125.82658505.1-         CCAGCCAGTTTGATGAAgtgagtgtggggaaagacatgcaaaatccttcttgatc
SRR959125.65104421.2-          CAGCCAGTTTGATGAAgtgagtgtggggaaagacatgcaaaatccttcttgatc
SRR959125.28048419.1+               AGTTTGATGAAgtgagtgtggggaaagacatgcaaaatccttcttgatc
SRR959126.20578648.2-                  TTGATGAAgtgagtgtggggaaagacatgcaaaatccttcttgatc
SRR959126.46510313.2+                   TGATGAAgtgagtgtggggaaagacatgcaaaatccttcttgatc
SRR959125.79334094.2-                      TGAAgtgagtgtggggaaagacatgcaaaatccttcttgatc
SRR959126.25851395.1+                      TGAAgtgagtgtggggaaagacatgcaaaatccttcttgatc
SRR959126.82800339.1-                       GAAgtgagtgtggggaaagacatgcaaaatccttcttgatc
                         ------------------------------------------------------------
consensus                CAATTCCAGCCAGTTTGATGAAgtgagtgtggggaaagacatgcaaaatccttcttgatc
```

X. Emperor penguin (No. 31), exon 8, WGS data

- CAP3 alignment of WGS data spanning exon 8 (uppercase letters)

```
                             .    :    .    :    .    :    .    :    .    :    .    :
SRR1144960.13923014.1+   tgctagactctgacctacttcattttgaactgcagA
SRR1144986.2151361.1+    tgctagactctgacctacttcattttgaactgcagATG
SRR1144949.17777503.1-   tgctagactctgacctacttcattttgaactgcagATGA
SRR1144949.20751761.1+   tgctagactctgacctacttcattttgaactgcagATGA
SRR1144951.15521086.1+   tgctagactctgacctacttcattttgaactgcagATGA
SRR1144951.3224147.2-    tgctagactctgacctacttcattttgaactgcagATGAAT
SRR1144953.11953462.1+   tgctagactctgacctacttcattttgaactgcagATGAAT
SRR1144955.14093991.2+   tgctagactctgacctacttcattttgaactgcagATGAAT
SRR1144944.16983818.2-   tgctagactctgacctacttcattttgaactgcagATGAATG
SRR1144981.2791406.2-    tgctagactctgacctacttcattttgaactgcagATGAATG
SRR1144986.4276791.2+    tgctagcctctgacctacgtcattttgaactgcagATGAATG
SRR1144954.5837236.1+    tgctagactctgacctacttcattttgaactgcagATGAATGG
SRR1144959.11681842.1-   tgctagactctgacctacttcattttgaactgcagATGAATGGTTC
SRR1144961.6807750.1+    tgctagactctgacctacttcattttgaactgcagATGAATGGTTCC
SRR1144979.832416.2+     tgctagactctgacctacttcattttgaaatgcagATGAATCGTTCCAGAT
SRR1144981.10767842.2+   tgctagactctgacctacttcattttgaactgcagATGAATTGTTCCAGATCT
SRR1144955.12492792.2-   tgctagactctgacctacttcattttgaactgcagATGAATGGTTCCAGATCTG
SRR1144949.13029897.1-   tgctagactctgacctacttcattttgaactgcagATGAATGGTTCCAGATCTGCTG
SRR1144955.19250011.1-   tgctagactctgacctacttcattttgaactgcagATGAATGGTTCCAGATCTGCTG
SRR1144978.12799072.1-   tgctagactctgacctacttcattttgaactgcagATGAATGGTTCCAGATCTGCTG
SRR1144979.9347410.1-    tgctagactctgacctacttcattttgaactgcagATGAATGGTTCCAGATCTGCTGGT
SRR1144943.15592673.2+   tgctagactctgacctacttcattttgaactgcagATGAATGGTTCCAGATCTGCTGGTT
SRR1144950.10041293.2+   tgctagactctgacctacttcattttgaactgcagATGAATGGTTCCAGATCTGCTGGTT
SRR1144950.9864783.1-    tgctagactctgacctacttcattttgaactgcagATGAATGGTTCCAGATCTGCTGGTT
SRR1144953.11606207.2+   tgctagactctgacctacttcattttgaactgcagATGAATGGTTCCAGATCTGCTGGTT
SRR1144953.5876551.1-    tgctagactctgacctacttcattttgaactgcagATGAATGGTTCCAGATCTGCTGGTT
SRR1144954.19381700.1+   tgctagactctgacctacttcattttgaactgcagATGAATGGTTCCAGATCTGCTGGTT
SRR1144955.6418335.2-    tgctagactctgacctacttcattttgatctgcagATGAATGGTTCCAGATCTGCTGGTT
SRR1144960.831264.1+     tgctagactctgacctacttcattttgaactgcagATGAATGGTTCCAGATCTGCTGGTT
SRR1144979.14388614.2-   tgctagactctgacctacttcattttgaactgcagATGAATGGTTCCAGATCTGCTGGTT
SRR1144979.9285614.1-    tgctagactctgacctacttcattttgaactgcagATGAATGGTTCCAGATCTGCTGGTT
SRR1144981.8694327.1-    tgctagactctgacctacttcattttgaactgcagATGAATGGTTCCAGATCTGCTGGTT
SRR1144984.20476661.2+   tgctagactctgacctacttcattttgaactgcagATGAATGGTTCCAGATCTGCTGGGT
SRR1144985.16473753.1+   tgctagactctgacctacttcattttgaactgcagATGAATGGTTCCAGATCTGCTGGTT
SRR1144985.23093154.1+   tgctagactctgacctacttcattttgaactgcagATGAATGGTTCCAGATCTGCTGGTT
SRR1144985.24362288.1+   tgctagactctgacctacttcattttgaactgcagATGAATGGTTCCAGATCTGCTGGTT
SRR1144986.30528143.1+   tgctagactctgacctacttcattttgaactgcagATGAATGGTTCCAGATCTGCTGGTT
SRR1144955.15966097.1-    gctagactctgacctacttcattttgaactgcagATGAATGGTTCCAGATCTGCTGGTT
SRR1144959.8113281.1-     gctagactctgacctacttcattttgaactgcagATNAATGGTTCCAGATCTGCTGGTT
SRR1144962.2493699.2+      ctagactctgacctacttcattttgaactgcagATGAATGGTTCCAGATCTGCTGGTT
SRR1144944.13147362.1+      tagactctgacctacttcattttgaactgcagATGAATGGTTCCAGAT
SRR1144978.3431911.2+       tagactctgacctacttcattttgaactgcagATGAATGGTTCCAGATCTGCTGGTT
SRR1144979.7573897.1+       tagactctgacctacttcattttgaactgcagATGAATGGTTCCAGATCTGCTGGTT
SRR1144959.7597348.1+        agactctgacctacttcattttgaactgcagATGAATGGTTCCNGATCTGCTGGTT
SRR1144951.19214455.1+         actctgacntacttcattttgaactgcagATGAATGGTTCCAGATCTGCTGGTT
SRR1144960.2183918.1+          actctgacctacttcattttgaactgcagATGAATGGTTCCAGATCTGCTGGTT
SRR1144961.10901322.2-            ctgacctacttcattttgaactgcagATGAATGGTTCCAGATCTGCTGGTT
SRR1144985.9029564.2+             ctgacctacttcattttgaactgcagATGAATGGTTCCAGATCTGCTGGTT
SRR1144961.12528769.1+             tgacctacttcattttgaactgcagATGAATGGTTCCAGATCTGCTGGTT
SRR1144984.2382315.1+              tgacctacttcattttgaactgcagATGAATGGTTCCAGATCTGCTGGTT
SRR1144984.5946477.2+              tgacctacttcattttgaactgcagATGAATGGTTCCAGATCTGCTGGTT
SRR1144959.10314728.1-               acctacttcattttgaactgcagATGAATGGTTCCAGATCTGCTGGTT
SRR1144959.15225111.1+               acctacttcattttgaactgcagATGAATGGTTCCAGATCTGCTGGTT
SRR1144986.946985.1-                    tacttcattttgaactgcagATGAATGGTTCCAGATCTGCTGGTT
SRR1144944.13164233.2+                   acttcattttgaactgcagATGAATGGTTCCAGATCTGCTGGTT
SRR1144950.12535980.2-                   acttcattttgaactgcagATGAATGGTTCCAGATCTGCTGGTT
SRR1144986.14854237.1-                     ttcattttgaactgcagATGAATGGTTCCAGATCTGCTGGTT
SRR1144950.14267840.1-                       cattttgaactgcagATGAATGGTTCCAGATCTGCTGGTT
SRR1144952.808828.2-                         cattttgaactgcagATGAATGGTTCCAGATCTGCTGGTT
SRR1144944.5032759.1+                          ttttgaactgcagATGAATGGTTCCAGATCTGCTGGTT
SRR1144954.4365300.1-                          ttttgaactgcagATGAATGGTTCCAGATCTGCTGGTT
SRR1144986.7592756.1+                           tttgaactgcagATGAATGGTTCCAGATCTGCTGGTT
SRR1144942.5908603.1-                            ttgaactgcagATGAATGGTTCCAGATCTGCTGGTT
SRR1144961.14309819.1+                           ttgaactgcagATGAATGGTTCCAGATCTGCTGGTT
SRR1144950.10119445.1+                            tgaactgcagATGAATGGTTCCAGATCTGCTGGTT
SRR1144981.17909752.1-                            tgaactgcagATGAATGGTTCCAGATCTGCTGGTT
SRR1144950.7664044.1-                              gaactgcagATGAATGGTTCCAGATCTGCTGGTT
SRR1144951.17862290.2+                             gaactgcagATGAATGGTTCCAGATCTGCTGGTT
SRR1144953.2404569.2+                              gaactgcagATGAATGGTTCCAGATCTGCTGGTT
SRR1144955.17447980.1-                             gaactgcagATGAATGGTTCCAGATCTGCTGGTT
SRR1144961.5167825.1-                              gaactgcagATGAATGGTTCCAGATCTGCTGGTT
SRR1144951.13625050.1-                              aactgcagATGAATGGTTCCAGATCTGCTGGTT
SRR1144942.4348457.2-                                  tgcagATGAATGGTTCCAGATCTGCTGGTT
SRR1144952.11024811.2+                                  gcagATGAATGGTTCCAGATCTGCTGGTT
SRR1144952.294730.1-                                    gcagATGAATGGTTCCAGATCTGCTGGTT
```

```
SRR1144978.1208094.1-                                   gcagATGAATGGTTCCAGATCTGCTGGTT
SRR1144980.13735925.2+                                  gcagATGAATGGTTCCAGATCTGCTGGTT
SRR1144955.14383370.2+                                   cagATGAATGGTTCCAGATCTGCTGGTT
SRR1144944.5026633.2-                                     agATGAATGGTTCCAGATCTGCTGGTT
SRR1144953.6056274.1-                                     agATGAATGGTTCCAGATCTGCTGGTT
SRR1144986.10009311.1+                                     gATGAATGGTTCCAGATCTGCTGGTT
SRR1144953.4311184.2-                                        TGAATGGTTCCAGATCTGCTGGTT
SRR1144984.6777508.1+                                        TGAATGGTTCCAGATCTGCTGGTT
SRR1144959.11990547.1+                                        GAATGGTTCCAGATCTGCTGGTT
SRR1144986.11256060.2+                                        GAATGGTTCCAGATCTGCTGGTT
SRR1144986.30224193.1-                                        GAATGTTTCCAGATCTGCTGGTT
SRR1144951.5147936.1-                                           ATGGTTCCAGATCTGCTGGTT
SRR1144953.20392854.2-                                           TGGTTCCAGATCTGCTGGTT
SRR1144949.15295324.1+                                            GGTTCCAGATCTGCTGGTT
SRR1144953.20128286.1-                                             GTTCCAGATCTGCTGGTT
SRR1144952.1688480.2+                                               TTCCAGATCTGCTGGTT
SRR1144979.14041499.2-                                              TTCCAGATCTGCTGGTT
SRR1144952.12650292.1-                                               TCCAGATCTGCTGGTT
SRR1144952.4502570.1-                                                 CCAGATCTGCTGGTT
SRR1144985.20729273.1-                                                CCAGATCTGCTGGTT
SRR1144960.1967638.1+                                                  CAGATCTGCTGGTT
SRR1144986.24595497.2+                                                 CAGATCTGCTGGTT
SRR1144950.13172717.1+                                                  AGATCTGCTGGTT
SRR1144978.4147148.1+                                                   AGATCTGCTGGTT
SRR1144980.9088994.2+                                                    GATCTGCTGGTT
SRR1144984.20476661.1-                                                      CTGCTGGTT
SRR1144942.10008183.1-                                                       TGCTGGTT
SRR1144985.3128712.1-                                                         GCTGGTT
SRR1144942.1413627.1+                                                           TGGTT
SRR1144949.17553424.2+                                                          TGGTT
SRR1144949.12686556.2-                                                            GTT
SRR1144954.1211192.2+                                                             GTT
SRR1144954.20544852.1-                                                            GTT
SRR1144959.12345339.1-                                                             TT
SRR1144986.6788735.2-                                                              TT
                         ____________________________________________________________
consensus                tgctagactctgacctacttcattttgaactgcagATGAATGGTTCCAGATCTGCTGGTT

                             .    :    .    :    .    :    .    :    .    :    .    :
SRR1144960.831264.1+     C
SRR1144953.5876551.1-    CCA
SRR1144953.11606207.2+   CCAG
SRR1144979.9285614.1-    CCAG
SRR1144985.16473753.1+   CCAG
SRR1144954.19381700.1+   CCAGA
SRR1144986.30528143.1+   CCAGA
SRR1144979.14388614.2-   CCAGAT
SRR1144985.24362288.1+   CCAGAT
SRR1144943.15592673.2+   CCAGATC
SRR1144981.8694327.1-    CCAGATC
SRR1144944.5032759.1+    CCAGATCT
SRR1144950.10041293.2+   CCAGATCTG
SRR1144984.20476661.2+   CCAGATCTG
SRR1144950.9864783.1-    CCAGATCTGCATG
SRR1144985.23093154.1+   CCAGATCTGCATGT
SRR1144955.15966097.1-   CCAGATCTGCATGTCT
SRR1144959.8113281.1-    CCAGATCTGCATGTCT
SRR1144962.2493699.2+    CCAGATCTGCATGTCTT
SRR1144978.3431911.2+    CCAGATCTGCATGTCTTT
SRR1144979.7573897.1+    CCAGATCTGCATGTCTTT
SRR1144959.7597348.1+    CCAGATCTGCATGTCTTTG
SRR1144951.19214455.1+   CCAGATCTGCATGTCTTTGCC
SRR1144960.2183918.1+    CCAGATCTGCATGTCTTTGCC
SRR1144961.10901322.2-   CCAGATCTGCATGTCTTTGCCTAC
SRR1144985.9029564.2+    CCAGATCTGCATGTCTTTGCCTAC
SRR1144961.12528769.1+   CCAGATCTGCATGTCTTTGCCTACT
SRR1144959.10314728.1-   CCAGATCTGCATGTCTTTGCCTACTTG
SRR1144959.15225111.1+   CCAGATCTGCATGTCTTTGCCTACTTG
SRR1144986.946985.1-     CCAGATCTGCATGTCTTTGCCTACTTGCTT
SRR1144944.13164233.2+   CCAGATCTGCATGTCTTTGCCTACTTGCTTC
SRR1144950.12535980.2-   CCAGATCTGCATGTCTTTGCCTACTTGCTTC
SRR1144986.14854237.1-   CCAGATCTGCATGTCTTTGCCTACTTGCTTCAT
SRR1144950.14267840.1-   CCAGATCTGCATGTCTTTGCCTACTTGCTTCATAC
SRR1144952.808828.2-     CCAGATCTGCATGTCTTTGCCTACTTGCTTCATAC
SRR1144954.4365300.1-    CCAGATCTGCATGTCTTTGCCTACTTGCTTCATACCC
SRR1144951.17862290.2+   CCAGATCTGCATGTCTTTGCCTACTTGCTTCATACCCA
SRR1144986.7592756.1+    CCAGATCTGCATGTCTTTGCCTACTTGCTTCATACCCA
SRR1144942.5908603.1-    CCAGATCTGCATGTCTTTACCTACTTGCTTCATACCCAC
SRR1144961.14309819.1+   CCAGATCTGCATGTCTTTGCCTACTTGCTTCATACCCAC
SRR1144950.10119445.1+   CCAGATCTGCATGTCTTTGCCTACTTGCTTCATACCCACC
SRR1144981.17909752.1-   CCAGATCTGCATGTCTTTGCCTACTTGCTTCATACCCACC
SRR1144950.7664044.1-    CCAGATCTGCATGTCTTTGCCTACTTGCTTCATACCCACCT
SRR1144953.2404569.2+    CCAGATCTGCATGTCTTTGCCTACTTGCTTCATACCCACCT
SRR1144955.17447980.1-   CCAGATCTGCATGTCTTTGCCTACTTGCTTCATACCCACCT
```

```
SRR1144961.5167825.1-    CCAGATCTGCATGTCTTTGCCTACTTGCTTCATACCCACCT
SRR1144951.13625050.1-   CCAGATCTGCATGTCTTTGCCTACTTGCTTCATACCCACCTG
SRR1144942.4348457.2-    CCAGATCTGCATGTCTTTGCCTACTTGCTTCATACCCACCTGTCT
SRR1144952.11024811.2+   CCAGATCTGCATGTCTTTGCCTACTTGCTTCATACCCACCTGTCTG
SRR1144952.294730.1-     CCAGATCTGCATGTCTTTGCCTACTTGCTTCATACCCACCTGTCTG
SRR1144978.1208094.1-    CCAGATCTGCATGTCTTTGCCTACTTGCTTCATACCCNCCTGTCTG
SRR1144980.13735925.2+   CCAGATCTGCATGTCTTTNCCTACTTGCTTCATACCCACCTGTCTG
SRR1144955.14383370.2+   CCAGATCTGCATGTCTTTGCCTACTTGCTTCATACCCACCTGTCTGG
SRR1144986.11256060.2+   CCAGATCTGCATGTCTTTGCCTACTTGCTTCATACCCACCTGTCTGG
SRR1144944.5026633.2-    CCAGATCTGCATGTCTTTGCCTACTTGCTTCATACCCACCTGTCTGGC
SRR1144953.6056274.1-    CCAGATCTGCATGTCTTTGCCTACTTGCTTCATACCCACCTGTCTGGC
SRR1144986.10009311.1+   CCAGATCTGCATGTCTTTGCCTACTTGCTTCATACCCACCTGTCTGGCA
SRR1144984.2382315.1+    CCAGATCTGCATGTCTTTGCCTACTTGCTTCATACCCACCTGTCTGGCAG
SRR1144984.5946477.2+    CCAGATCTGCATGTCTTTGCCTACTTGCTTCATACCCACCTGTCTGGCAG
SRR1144953.4311184.2+    CCAGATCTGCATGTCTTTGCCTACTTGCTTCATACCCACCTGTCTGGCAGA
SRR1144959.11990547.1+   CCAGATCTGCATGTCTTTGCCTACTTGCTTCATACCCACCTGTCTGGCAGAG
SRR1144986.30224193.1-   CCAGATCTGCATGTCTTTGCCTACTTGCTTCATACCCACCTGTCTGGCAGAG
SRR1144951.5147936.1-    CCAGATCTGCATGTCTTTGCCTACTTGCTTCATACCCACCTGTCTGGCAGAGGA
SRR1144953.20392854.2-   CCAGATCTGCATGTCTTTGCCTACTTGCTTCATACCCACCTGTCTGGCAGAGGAG
SRR1144949.15295324.1+   CCAGATCTGCATGTCTTTGCCTACTTGCTTCATACCCACCTGTCTGGCAGAGGAGT
SRR1144953.20128286.1-   CCAGATCTGCATGNCTTTGCCTACTTGCTTCATACCCGCCTGTCTGGCAGAGGAGTG
SRR1144952.1688480.2+    CCAGATCTGCATGTCTTTGCCTACTTGCTTCATACCCACCTGTCTGGCAGAGGAGTGA
SRR1144979.14041499.2-   CCAGATCGGCATGTCTTTGCCTACTTGCTTCATACCCACCTGTCTGGCAGAGGAGTGA
SRR1144952.12650292.1-   CCAGATCTGCATGTCTTTGCCTACTTGCTTCATACCCACCTGTCTGGCAGAGGAGTGAA
SRR1144942.10008183.1-   CCAGATCTGCATGTCTTTGCCTACTTGCTTCATACCCACCTGTCTGGCAGAGGAGTGAAA
SRR1144942.1413627.1+    CCAGATCTGCATGTCTTTGCCTACTTGCTTCATACCCACCTGTCTGGCAGAGGAGTGAAA
SRR1144943.15756235.2-   CCAGATCTGCATGTCTTTGCCTACTTGCTTCATACCCACCTGTCTGGCAGAGGAGTGAAA
SRR1144949.12686556.2-   CCAGATCTGCATGTCTTTGCCTACTTGCTTCATACCCACCTGTCTGGCAGAGGAGTGAAA
SRR1144949.17553424.2+   CCAGATCTGCATGTCTTTGCCTACTTGCTTCATACCCACCTGTCTGGCAGAGGAGTGAAA
SRR1144950.13172717.1+   CCAGATCTGCATGTCTTTGCCTACTTGCTTCATACCCACCTGTCTGGCAGAGGAGTGAAA
SRR1144952.4502570.1-    CCAGATCTGCATGTCTTTGCCTACTTGCTTCATACCCACCTGTCTGGCAGAGGAGTGAAA
SRR1144954.1211192.2+    CCAGATCTGCATGTCTTTGCCTACTTGCTTCATACCCACCTGTCTGGCAGAGGGGTGAAA
SRR1144954.20544852.1-   CCAGATCTGCATGTCTTTGCCTACTTGCTTCATACCCACCTGTCTGGCAGAGGAGTGAAA
SRR1144959.12345339.1-   CCAGATCTGCATGTCTTTGCCTACTTGCTTCATACCCACCTGTCTGGCAGAGGAGTGAAA
SRR1144960.1967638.1+    CCAGATCTGCATGTCTTTGCCTACTTGCTTCATACCCACCTGTCTGGCAGAGGAGTGAAA
SRR1144978.4147148.1+    CCAGATCTGCATGTCTTTGCCTACTTGCTTCATACCCACCTGTCTGGCAGAGGAGTGAAA
SRR1144980.9088994.2+    CCAGATCTGCATGTCTTTGCCTACTTGCTTCATACCCACCTGTCTGGCAGAGGAGTGAAA
SRR1144984.20476661.1-   CCAGATCTGCATGTCTTTGCCTACTTGCTTCATACCCACCTGTCTGGCAGAGGAGTGAAA
SRR1144984.6777508.1+    CCAGATCTGCATGTCTTTGCCTACTTGCTTCATACCCCCCTGTCTGGCAGAGGAGTGAAA
SRR1144985.20729273.1-   CCAGATCTGCATGTCTTTGCCTACTTGCTTCATACCCACCTGTCTGGCAGAGGAGTGAAA
SRR1144985.3128712.1-    CCAGATCTGCATGTCTTTGCCTACTTGCTTCATACCCACCTGTCTGGCAGAGGAGTGAAA
SRR1144986.24595497.2+   CCAGATCTGCATGTCTTTGCCTACTTGCTTCATACCCACCTGTCTGGCAGAGGAGCGAAA
SRR1144986.6788735.2-    CCAGATCTGCATGTCTTTGCCTACTTGCTTCATACCCACCTGTCTGGCAGAGGAGTGAAA
SRR1144954.7640918.1-     CAGATCTGCATGTCTTTGCCTACTTGCTTCATACCCACCTGTCTGGCAGAGGAGTGAAA
SRR1144950.2194951.2-      AGATCTGCATGTCTTTGCCTACTTGCTTCATACCCACCTGTCTGGCAGAGGAGTGAAA
SRR1144986.21986740.2+       ATCTGCATGTCTTTGCCTACTTGCTTCATACCCACCTGTCTGGCAGAGGAGTGAAA
SRR1144943.916024.1+          TCTGCATGTCTTTGCCTACTTGCTTCATACCCACCTGTCTGGCAG
SRR1144951.9576764.1+          CTGCATGTCTTTGCCTACTTGCTTCATACCCACCTGTCTGGCAGAGGAGTGAAA
SRR1144985.12647838.1+          TGCATGTCTTTGCCTACTTGCTTCATACCCACCTGTCTGGCAGAGGAGTGAAA
SRR1144984.20427578.1+           GCATGTCTTTGCCTACTTGCTTCATACCCACCTGTCTGGCAGAGGAGTGAAA
SRR1144961.2641769.1+             CATGTCTTTGCCTACTTGCTTCATACCCACCTGTCTGGCAGAGGAGTGAAA
SRR1144962.3029055.1-                GTCTTTGCCTACTTGCTTCATACCCACCTGTCTGGCAGAGGAGTGAAA
SRR1144961.4388119.1-                 TCTTTGCCTACTTGCTTCATACCCACCTGTCTGGCAGAGGAGTGAAA
SRR1144986.2151361.2-                  CTTTGCCTACTTGCTTCATACCCACCTGTCTGGCAGAGGAGTGAAA
SRR1144960.12078624.2+                  TTTGCCTACTTGCTTCATACCCACCTGTCTGGCAGAGGAGTGAAA
SRR1144961.12963979.2-                   TTGCCTACTTGCTTCATACCCACCTGTCTGGCAGAGGAGTGAAA
SRR1144986.4901780.2+                    TTGCCTACTTGCTTCATACCCACCTGTCTGGCAGAGGAGTGAAA
SRR1144953.18905485.1+                    TGCCTACTTGCTTCATACCCACCTGTCTGGCAGAGGAGTGAAA
SRR1144984.2382315.2-                     TGCCTACTTGCTTCATACCCACCTGTCTGGCAGAGGAGTGAAA
SRR1144986.4276791.1-                      GCCTACTTGCTTCATACCCACCTGTCTGGCAGAGGAGTGAAA
SRR1144978.18364459.1-                       CTACTTGCTTCATACCCACCTGTCTGGCAGAGGAGTGAAA
SRR1144952.11028386.1-                         ACTTGCTTCATACCCACCTGTCTGGCAGAGGAGTGAAA
SRR1144955.13320235.1+                             GCTTCATACCCACCTGTCTGGCAGAGGAGTGAAA
SRR1144960.14781519.1+                             GCTTCATACCCACCTGTCTGGCAGAGGAGTGAAA
SRR1144986.4116432.2-                              GCTTCATACCCACCTGTCTGGCAGAGGAGTGAAA
SRR1144953.16539711.2+                              CTTCATACCCACCTGTCTGGCAGAGGAGTGAAA
SRR1144943.7682877.2+                                TTCATACCCACCTGTCTGGCAGAGGAGTGAAA
SRR1144985.24362288.2-                               TTCATCCCCACCTGTCTGGCAGAGGAGTGAAA
SRR1144978.11779193.1-                                TCATACCCACCTGTCTGGCAGAGGAGTGAAA
SRR1144955.5461379.2+                                  CATACCCACCTGTCTGGCAGAGGAGTGAAA
SRR1144981.9904262.2+                                    TACCCACCTGTCTGGCAGAGGAGTGAAA
SRR1144984.5946477.1-                                    TACCCACCTGTCTGGCAGAGGAGTGAAA
SRR1144985.16473753.2-                                   TACCCGCCTGTCTGGCAGAGGAGTGAAA
SRR1144953.4488829.1-                                      CCCACCTGTCTGGCAGAGGAGTGAAA
SRR1144960.14407896.1+                                           TGTCTGGCAGAGGAGTGAAA
SRR1144951.20015101.2+                                            GTCTGGCAGAGGAGTGAAA
SRR1144985.23093154.2-                                            GTCTGGCAGAGGAGTGAAA
SRR1144942.427743.2-                                                CTGGCAGAGGAGTTAAA
SRR1144954.1318563.1-                                               CTGGCAGAGGAGTGAAA
SRR1144954.18593551.1-                                              CTGGCAGAGGAGTGAAA
SRR1144955.2259421.1+                                               CTGGCAGAGGAGTGAAA
SRR1144978.1645512.1-                                               CTGGCAGAGGAGTGAAA
SRR1144952.1093801.1+                                                TGGCAGAGGAGTGAAA
SRR1144952.16594577.1-                                               TGGCAGAGGAGTGAAA
```

```
SRR1144949.2300822.1-                                                 GGCAGAGGAGTGAAA
SRR1144950.7985367.2+                                                    AGAGGAGTGAAA
SRR1144952.514173.2+                                                     AGAGGAGTGAAA
SRR1144962.13686535.2-                                                     AGGAGTGAAA
SRR1144985.9029564.1-                                                      AGGAGTGAAA
SRR1144986.30528143.2-                                                      GGAGTGAAA
SRR1144986.13072712.1+                                                       GAGTGAAA
SRR1144949.19899083.1+                                                        AGTGAAA
SRR1144950.13428587.2-                                                        AGTGAAA
SRR1144984.15024852.1+                                                        AGTGAAA
SRR1144955.10939512.1+                                                         GTGAAA
SRR1144986.12084781.1-                                                         GTGAAA
SRR1144949.2337502.1-                                                            GAAA
SRR1144986.13351284.1+                                                           GAAA
SRR1144944.632790.2+                                                               AA
SRR1144959.12415981.2-                                                              A
                         ____________________________________________________________
consensus                CCAGATCTGCATGTCTTTGCCTACTTGCTTCATACCCACCTGTCTGGCAGAGGAGTGAAA

                             .    :    .    :    .    :    .    :    .    :    .    :
SRR1144960.1967638.1+    G
SRR1144986.24595497.2+   G
SRR1144950.13172717.1+   GC
SRR1144978.4147148.1+    GC
SRR1144980.9088994.2+    GCT
SRR1144942.10008183.1-   GCTGCTC
SRR1144985.3128712.1-    GCTGCTCA
SRR1144942.1413627.1+    GCTGCTCAAT
SRR1144949.17553424.2+   GCTGCTCAAT
SRR1144954.1211192.2+    GCTGCTCAATA
SRR1144949.12686556.2-   GCTGCTCAATAC
SRR1144954.20544852.1-   GCTGCTCAATAC
SRR1144959.12345339.1-   GCTGCTCAATACC
SRR1144986.6788735.2-    GCTGCTCAATACC
SRR1144943.15756235.2-   GCTGCTCAATACCGg
SRR1144986.21986740.2+   GCTGCTCAATACCGg
SRR1144954.7640918.1-    GCTGCTCAATACCGgt
SRR1144984.6777508.1+    GCTGCTCAATAACGgt
SRR1144950.2194951.2-    GCTGCTCAATACCGgta
SRR1144951.9576764.1+    GCTGCTCAATACCGgtaagag
SRR1144985.12647838.1+   GCTGCTCAATACCGgtaagaga
SRR1144961.2641769.1+    GCTGCTCAATACCGgtaagagaag
SRR1144962.3029055.1-    GCTGCTCAATACCGgtaagagaagcaa
SRR1144961.4388119.1-    GCTGCTCAATACCGgtaagagaagcaaa
SRR1144986.2151361.2-    GCTGCTCAATACCGgtaagagaagcaaac
SRR1144960.12078624.2+   GCTGCTCAATACCGgtaagagaagcaaact
SRR1144961.12963979.2-   GCTGCTCAATACCGgtaagagaagcaaactc
SRR1144984.20476661.1-   GCTGCTCAATACCGgtaagagaagcaaactc
SRR1144986.4901780.2+    GCTGCTCAATACCGgtaagagaagcaaactc
SRR1144953.18905485.1+   GCTGCTCAATACCGgcaagagaagcaaactcg
SRR1144986.4276791.1-    GCTGCTCAATACCGgtaagagaagcaaactcgg
SRR1144978.18364459.1-   GCTGCTNAATACCGgtaagagaagcaaactcggta
SRR1144952.11028386.1-   GCTGCTCAATACCGgtaagagaagcaaactcggtatt
SRR1144955.13320235.1+   GCTGCTCAATACCGgtaagagaagcaaactcggtattg
SRR1144952.514173.2+     GCTGCTCAATACCGgtaagagaagcaaactcggtattgctg
SRR1144960.14781519.1+   GCTGCTCAATACCGgtaagagaagcaaactcggcattgctg
SRR1144986.4116432.2-    GCTGCTCAATACCGgtaagagaagcaaactcggtattgctg
SRR1144953.16539711.2+   GCTGCTCAATACCGgtaagagaagcaaactcggtattgctgc
SRR1144943.7682877.2+    GCTGCTCAATACCGgtaagagaagcaaactcggtattgctgcc
SRR1144985.24362288.2-   GCTGCTCAATACCGgtaagagaagcaaactcggtattgctgcc
SRR1144978.11779193.1-   GCTGCTCAATACCGgtaagagaagcaaactcggtattgctgcct
SRR1144955.5461379.2+    GCTGCTCAATACCGgtaagagaagcaaactcggtattgctgccta
SRR1144985.16473753.2-   GCTGCTCAATACCGgtaagagaagcaaactcggtattgctgccta
SRR1144981.9904262.2+    GCTGCTCAATAACGgtaagagaagcaaactcgggattgctgcctatg
SRR1144953.4488829.1-    GCTGCTCAATACCGgtaagagaagcaaactcggtattgctgcctatgc
SRR1144984.20427578.1+   GCTGCTCAATACCGgtaagagaagcaaactcggtattgctgcctatgc
SRR1144951.20015101.2+   GCTGCTCAATACCGgtaagagaagcaaactcggtattgctgcctatgccaca
SRR1144960.14407896.1+   GCTGCTCAATACCGgtaagagaagcaaactcggtattgctgcctatgccacagga
SRR1144985.23093154.2-   GCTGCTCAATACCGgtaagagaagcaaactcggtattgctgcctatgccacaggat
SRR1144984.2382315.2-    GCTGCTCAATACCGgtaagagaagcaaactcggtattgctgcctatgccacaggatc
SRR1144942.427743.2-     TCTGCTCAATACCGgtaagagaagcaaactcggtattgctgcctattccacaggatca
SRR1144954.1318563.1-    TCTGCTCAATACCGgtaagagaagcaaactcggtattgctgcctatgccacaggatca
SRR1144954.18593551.1-   GCTGCTCAATACCGgtaagagaagcaaactcggtattgctgcctatgccacaggatca
SRR1144955.2259421.1+    GCTGCTCAATACCGgtaagagaagcaaactcggtattgctgcctatgccacaggatca
SRR1144978.1645512.1-    GCTGCTCAATACCGgtaagagaagcaaactcggtattgctgcctatgccacaggatca
SRR1144952.1093801.1+    GCTGCTCAATACCGgtaagagaagcaaactcggtattgctgcctatgccacaggatcag
SRR1144952.16594577.1-   GCTGCTCAATACCGgtaagagaagcaaactcggtattgctgcctatgccacaggatcag
SRR1144944.632790.2+     GCTGCTCAATACCGgtaagagaagcaaactcggtattgctgcctatgccacaggatcagt
SRR1144949.19899083.1+   GCTGCTCAATACCGgtaagagaagcaaactcggtattgctgcctatgccacaggatcagt
SRR1144949.2300822.1-    GCGGCTCAATCCCGgtaagagaagcaaactcggtattgctgcctatgccacaggatcagt
SRR1144949.2337502.1-    GCTGCTCAATACCGgtaagagaagcaacctcggtattgctgcctatgccacaggatcagt
SRR1144950.13428587.2-   GCTGCTCAATACCGgtaagagaagcaaactcggtattgctgcctatgccacaggatcagt
SRR1144950.20346962.1-   GCTGCTCAATACCGgaaagagaagcaaactcggtattgctgcctatgccacaggatcagt
SRR1144950.7985367.2+    GCTGCTCAATACCGgtaagagaagcaaactcggtattgctgcctatgccacaggatcagt
```

```
SRR1144955.10939512.1+   GCTGCTCAATACCGgtaagagaagcaaactcggtattgctgcctatgccacaggatcagt
SRR1144959.12415981.2-   GCTGCTCAATACCGgtaagagaagcaaactcggtattgctgcctatgccacaggatcagt
SRR1144959.13474460.2-   GCTGCTCAATACCGgtaagagaagcaaactcggtattgctgcctatgccacaggatcagt
SRR1144962.13686535.2-   GCTGCTCAATACCGgtaagagaagcaagctcggtattgctgcctatgccacaggatcagt
SRR1144984.15024852.1+   GCTGCTCAATACCGgtaagagaagcaaactcggtattgctgcctatgccacaggatcagt
SRR1144984.5946477.1-    GCTGCTCAATACCGgtaagagaagcaaactcggtattgctgcctatgccacaggatcagt
SRR1144985.9029564.1-    GCTGCTCAATACCGgtaagagaagcaaactcggtattgctgcctatgccacaggatcagt
SRR1144986.12084781.1-   GCTGCTCAATACCGgtaagagaagcaaactcggtattgctgcctatgccacaggatcagt
SRR1144986.13072712.1+   GCTGCTCAATACCGgtaagagaagcaaactcggtattgctgcctatgccacaggatcagt
SRR1144986.13351284.1+   GCTGCTCAATACCGgtaagagaagcaaactcggtattgctgcctatggcacaggatcagt
SRR1144986.30528143.2-   GCTGCTCAATCCCGgtaagagaagcaaactcggtattgctgcctatgccacaggatcagt
SRR1144980.13646827.2+    CTGCTCAATACCGgtaagagaagcaaactcggtattgctgcctatgccacaggaacagt
SRR1144959.15046561.1+      GCTCAATACCGgtaagagaagcaaactcggtattgctgcctatgccacaggatcagt
                         ____________________________________________________________
consensus                GCTGCTCAATACCGgtaagagaagcaaactcggtattgctgcctatgccacaggatcagt
```

Y. Adelie penguin (No. 32), exon 8, WGS data

- CAP3 alignment of WGS data spanning exon 8 (uppercase letters)

```
                             .    :    .    :    .    :    .    :    .    :    .    :
SRR1144993.41032305.2-   tctgactgccagactctgacctacttcattttgaactgcagATGAATGGGATGCTGGTTC
SRR1144993.41032429.2-   tctgactgccagactctgacctacttcattttgaactgcagATGAATGGGATGCTGGTTC
SRR1144994.61072887.1-   tctgactgccagactctgacctacttcattttgaactgcagATGAATGGGATGCTGGTTC
SRR1144999.18884048.1-   cctgactgccagactctgacctacttcattttgaactgcagATGAATGGGATGCTGGTTC
SRR1145000.15292017.2+   tctgactgccagactctgacctacttcattttgaactgcagATGAATGGGATGCTGGTTC
SRR1145000.36401457.2+   tctgactgccagactctgacctacttcattttgaactgcagATGAATGGGATGCTGGTTC
SRR1145004.65320437.2-   tctgactgccagactctgacctacttcattttgaactgcagATGAATGGGATGCTGGTTC
SRR1145006.26110484.2+   tctgactgccagactctgacctacttcattttgaactgcagATGAATGGGATGCTGGTTC
SRR1145006.67318459.1-   tctgactgccagactctgacctacttcattttgaactgcagATGAATGGGATGCTGGTTC
SRR1145006.74715836.2+   tctgactgccagactctgacctacttcattttgaactgcagATGAATGGGATGCTGGTTC
SRR1145007.14404671.1+   tctgactgccagactctgacctacttcattttgaactgcagATGAATGGGATGCTGGTTC
SRR1145007.42050412.2+   tctgactgccagactctgacctacttcattttgaactgcagATGAATGGGATGCTGGTTC
SRR1145001.32505101.2+     tgactgccagactctgacctacttcattttgaactgcagATGAATGGGA
SRR1144993.9495205.2-      tgactgccagactctgacctacttcattttgaactgcagATGAATGGGATGCTGGTTC
SRR1144993.9495317.2-      tgactgccagactctgacctacttcattttgaactgcagATGAATGGGATGCTGGTTC
SRR1144996.48719847.2-     tgactgccagactctgacctacttcattttgaactgcagATGAATGGGATGCTGGTTC
SRR1145000.28065454.2+     tgactgccagactctgacctacttcattttgaactgcagATGAATGGGATGCTGGTTC
SRR1145000.44688266.2+     tgactgccagactctgacctacttcattttgaactgcagATGAATGGGATGCTGGTTC
SRR1145007.35693248.2-     tgactgccagactctgacctacttcattttgaactgcagATGAATGGGATGCTGGTTC
SRR1144994.57031222.1+      gactgccagactctgacctacttcattttgaactgcagATGAATGGGATGCTGGTTC
SRR1144993.53904505.1-        ctgccagactctgacctacttcattttgaactgcagATGAATGGGATGCTGGTTC
SRR1144993.53904608.1-        ctgccagactctgacctacttcattttgaactgcagATGAATGGGATGCTGGTTC
SRR1144995.19363583.2-         tgccagactctgacctacttcattttgaactgcagATGAATGGGATGCTGGTTC
SRR1145006.2891410.2-          tgccagactctgacctacttcattttgaactgcagATGAATGGGATGCTGGTTC
SRR1144996.11717603.1-           ccagactctgacctacttcattttgaactgcagATGAATGGGATGCTGGTTC
SRR1144996.2678411.1+            ccagactctgacctacttcattttgaactgcagATGAATGGGATGCTGGTTC
SRR1144996.2678522.1+            ccagactctgacctacttcattttgaactgcagATGAATGGGATGCTGGTTC
SRR1145001.5099178.2-             cagactctgacctacttcattttgaactgcagATGAATGGGATGCTGGT
SRR1145007.73416802.2-            cagactctgacctacttcattttgaactgcagATGAATGGGATGCTGGTTC
SRR1144993.13151284.2+             agactctgacctacttcattttgaactgcagATGAATGGGATGCTGATTC
SRR1144993.13151419.2+             agactctgacctacttcattttgaactgcagATGAATGGGATGCTGGTTC
SRR1144996.15468538.2+              gactctgacctacttcattttgaactgcagATGAATGGGATGCTGGTTG
SRR1145000.30498291.1+              gactctgacctacttcattttgaactgcagATGAATGGGATGCTGGTTC
SRR1145007.57624437.1+               actctgacctacttcattttgaactgcagATGAATGGGATGCTGGTTC
SRR1145000.65355946.2+                ctctgacctacttcattttgaactgcagATGAATGGGATGCTGGTTC
SRR1145001.24919820.2-                ctctgacctacttcattttgaactgcagATGAATGGGATGCTGGTTC
SRR1145001.6927421.2-                 ctctgacctacttcattttgaactgcagATGAATGGGATGCTGGTTC
SRR1144995.2415275.1-                   ctgacctacttcattttgaactgcagATGAATGGGATGCTGGTTC
SRR1145005.42026519.2-                  ctgacctacttcattttgaactgcagATGAATGGGATGCTGGTTC
SRR1145001.72192711.2+                        tacttcattttgaactgcagATGAATGGGATGCTGGTTC
SRR1144993.38026379.2-                           ttcattttgaactgcagATGAATGGGATGCTGGTTC
SRR1145005.56580374.2+                           ttcattttgaactgcagATGAATGGGATGCTGGTTC
SRR1145005.61551283.2-                               ttttgaactgcagATGAATGGGATGCTGGTTC
SRR1145005.8349381.2-                                  ttgaactgcagATGAATGGGATGCTGGTTC
SRR1144994.17876275.1-                                   gaactgcagATGAATGGGATGCTGGTTC
SRR1144999.66900124.2+                                   gaactgcagATGAATGGGATGCTGGTTC
SRR1145004.66225478.1+                                   gaactgcagATGAATGGGATGCTGGTTC
SRR1145006.40154970.2-                                        gcagATGAATGGGATGCTGGTTC
SRR1144995.3469204.2+                                          cagATGAATGGGATGCTGGTTC
SRR1144998.14176477.1-                                         cagATGAATGGGATGCTGGTTC
SRR1144998.25117498.1-                                         cagATGAATGGGATGCTGGTTC
SRR1144998.46615232.1-                                         cagATGAATGGGATGCTGGTTC
SRR1144998.5699235.1-                                          cagATGAATGGGATGCTGGTTC
SRR1145006.55037379.1-                                           gATGAATGGGATGCTGGTTC
SRR1145005.34981199.2+                                              GAATGGGATGCTGGTTC
SRR1144993.907700.2+                                                  ATGGGATGCTGGTTC
SRR1144993.907701.2+                                                  ATGGGATGCTGGTTC
SRR1144998.21120878.2+                                                ATGGGATGCTGGTTC
SRR1144998.27961014.2+                                                ATGGGATGCTGGTTC
SRR1144998.28625040.2+                                                ATGGGATGCTGGTTC
SRR1144998.4243211.2+                                                 ATGGGATGCTGGTTC
SRR1144998.47686489.2+                                                ATGGGATGCTGGTTC
SRR1145007.42050412.1-                                                    GATGCTGGTTC
SRR1144993.3886278.2+                                                      ATGCTGGTTC
SRR1144998.67085243.2+                                                     ATGCTGGTTC
SRR1145000.19173285.2+                                                     ATGCTGGTTC
SRR1145004.29072333.2+                                                       GCTGGTTC
SRR1144994.57031222.2-                                                        CTGGTTC
SRR1144994.13223558.2+                                                         TGGTTC
SRR1144999.13344253.2-                                                           GTTC
SRR1145006.22933790.2+                                                           GTTC
SRR1145006.24052151.2+                                                             TC
                         ____________________________________________________________
consensus                tctgactgccagactctgacctacttcattttgaactgcagATGAATGGGATGCTGGTTC
```

```
                              .    :    .    :    .    :    .    :    .    :    .    :
SRR1145001.24919820.2-        CA
SRR1145001.6927421.2-         CA
SRR1145000.28065454.2+        CAGA
SRR1145000.44688266.2+        CAGA
SRR1145001.72192711.2+        CAGATCTGCA
SRR1145000.65355946.2+        CAGATCTGCAT
SRR1144993.41032305.2-        CAGATCTGCATGTCTTTTTG
SRR1144993.41032429.2-        CAGATCTGCATGTCTTTTTG
SRR1144999.18884048.1-        CAGATCTGCATGTCTTTTTGC
SRR1145007.14404671.1+        CAGATCTGCATGTCTTTTTGC
SRR1145000.36401457.2+        CAGATCTGCATGTCTTTTTGCC
SRR1145006.74715836.2+        CAGATCTGCATGTCTTTTTGCCTAC
SRR1145006.26110484.2+        CAGATCTGCATGTCTTTTTGCCTACTT
SRR1144994.61072887.1-        CAGATCTGCATGTCTTTTTGCCTACTTG
SRR1145000.15292017.2+        CAGATCTGCATGTCTTTTTGCCTACTTGCT
SRR1145006.67318459.1-        CAGATCTGCATGTCTTTTTGCCTACTTGCTT
SRR1145004.65320437.2-        CAGATCTGCATGTCTTTTTGCCTACTTGCTTCA
SRR1145007.42050412.2+        CAGATCTGCATGTCTTTTTGCCTACTTGCTTCATAC
SRR1145000.30498291.1+        CAGATCTGCATGTCTTTTTGCCTACTTGCTTCATACCCACC
SRR1144993.9495205.2-         CAGATCTGCATGTCTTTTTGCCTACTTGCTTCATACCCACCT
SRR1144993.9495317.2-         CAGATCTGCATGTCTTTTTGCCTACTTGCTTCATACCCACCT
SRR1144996.11717603.1-        CAGATCTGCATGTCTTTTTGCCTACTTGCTTCATACCCACCT
SRR1144996.48719847.2-        CAGATCTGCATGTCTTTTTGCCTACTTGCTTCATACCCACCT
SRR1145007.35693248.2-        CAGATCTGCATGTCTTTTTGCCTACTTGCTTCATACCCACCT
SRR1144994.57031222.1+        CAGATCTGCATGTCTTTTTGCCTACTTGCTTCATACCCACCTG
SRR1144999.13344253.2-        CAGATCTGCATGTCTTTTTGCCTACTTGCTTCATACCCACCTGT
SRR1144993.53904505.1-        CAGATCTGCATGTCTTTTTGCCTACTTGCTTCATACCCACCTGTC
SRR1144993.53904608.1-        CAGATCTGCATGTCTTTTTGCCTACTTGCTTCATACCCACCTGTC
SRR1144995.19363583.2-        CAGATCTGCATGTCTTTTTGCCTACTTGCTTCATACCCACCTGTCT
SRR1145006.2891410.2-         CAGATCTGCATGTCTTTTTGCCTACTTGCTTCATACCCACCTGTCT
SRR1144996.15468538.2+        CAGATCTGCATGTCTTTTTGCCTACTTGCTTCATACCGACCTGTCTG
SRR1144996.2678411.1+         CAGATCTGCATGTCTTTTTGACTACTTGCTTCATACCCACCTGTCTGG
SRR1144996.2678522.1+         CAGATCTGCATGTCTTTTTGCATACTTGCTTCATACCCACCTGTCTGG
SRR1144993.13151284.2+        CAGATCTGCATGTCTTTTTGCTTACTTGCTTCATAACTACCTGTCTGGC
SRR1145007.73416802.2-        CAGATCTGCATGTCTTTTTGCCTACTTGCTTCATACCCACCTGTCTGGC
SRR1144993.13151419.2+        CAGATCTGCATGTCTTTTTGCCTACTTGCTTCATACCCACCTGTCTGGCA
SRR1145007.57624437.1+        CAGATCTGCATGTCTTTTTGCCTACTTGCTTCATACCCACCTGTCTGGCAGA
SRR1144995.2415275.1-         CAGATCTGCATGTCTTTTTGCCTACTTGCTTCATACCCACCTGTCTGGCAGAGGA
SRR1145005.42026519.2-        CAGATCTGCATGTCTTTTTGCCTACTTGCTTCATACCCACCTGTCTGGCAGAGGA
SRR1144993.38026379.2-        CAGATCTGCATGTCTTTNTNCCTACTTGCTTCATACCCACCTGTCTGGCAGAGGAGTGAA
SRR1144993.3886278.2+         CAGATCTGCATGTCTTTTTGCCTACTTGCTTCATACCCACCTGTCTGGCAGAGGCGTGAA
SRR1144993.907700.2+          CAGATCTGCATGTCTTTTTGCCTACTTGCTTCATACCCACCTGTCTGGCAGAGGAGTGAA
SRR1144993.907701.2+          CAGATCTGCATGTCTTTTTGCCTACTTGCTTCATACCCACCTGTCTGGCAGAGGAGTGAA
SRR1144994.13223558.2+        CAGATCTGCATGTCTTTTTGCCTACTTGCTTCATACCCACCTGTCTGGCAGAGGAGTGAA
SRR1144994.17876275.1-        CAGATCTGCATGTCTTTTTGCCTACTTGCTTCATACCCACCTGTCTGGCAGAGGAGTGAA
SRR1144994.57031222.2-        CAGATCTGCATGTCTTTTTGCCTACTTGCTTCATACCCACCTGTCTGGCAGAGGAGTGAA
SRR1144995.3469204.2+         CAGATCTGCATGTCTTTTTGCCTACTTGCTTCATACCCACCTGTCTGGCAGAGGAGTGAA
SRR1144998.14176477.1-        CAGATCTGCATGTCTTTTTGCCTACTTGCTTCATACCCACCTGTCTGGCAGAGGAGTGAA
SRR1144998.21120878.2+        CAGATCTGCATGTCTTTTTGCCTACTTGCTTCATACCCACCTGTCTGGCAGAGGAGTGAA
SRR1144998.25117498.1-        CAGATCTGCATGTCTTTTTGCCTACTTGCTTCATTCCCACCTGTCTGGCAGAGGAGTGAA
SRR1144998.27961014.2+        CAGATCTGCATGTCTTTTTGCCTACTTGCTTCATACCCACCTGTCTGGCAGAGGCGTGAA
SRR1144998.28625040.2+        CAGATCTGCATGTCTTTTTGCCTACTTGCTTCATACCCACCTGTCTGGCAGAGGAGGGAA
SRR1144998.4243211.2+         CAGATCTGCATGTCTTTTTGCCTACTTGCTTCATACCCACCTGTCTGGCAGAGGGGTGAA
SRR1144998.46615232.1-        CAGATCTGCATGTCTTTTTGCCTACTTGCTTCATACCCACCTGTCTGGCAGAGGAGTGAA
SRR1144998.47686489.2+        CAGATCTGCATGTCTTTTTGCCTACTTGCTTCATACCCACCTGTCTGGCAGAGGAGTGAA
SRR1144998.5699235.1-         CAGATCTGCATGTCTTTTTGCCTACTTGCTTCATACCCACCTGTCTGGCAGAGGAGTGAA
SRR1144998.67085243.2+        CAGATCTGCATGTCTTTTTGCCTACTTGCTTCATACCCACCTGTCTGGCAGAGGAGTGAA
SRR1144999.66900124.2+        CAGATCTGCATGTCTTTTTGCCTACTTGCTTCATACCCACCTGTCTGGCAGAGGAGTGAA
SRR1145000.19173285.2+        CAGATCTGCATGTCTTTTTGCCTACTTGCTTCATACCCACCTGTCTGGCAGAGGAGTGAA
SRR1145004.29072333.2+        CAGATCTGCATGTCTTTTTGCCTACTTGCTTCATACCCACCTGTCTGGCAGAGGAGTGAA
SRR1145004.66225478.1+        CAGATCTGCATGTCTTTTTGCCTACTTGCTTCATACCCACCTGTCTGGCAGAGGAGTGAA
SRR1145005.34981199.2+        CAGATCTGCATGTCTTTTTGCCTACTTGCTTCATACCCACCTGTCTGGCAGAGGAGGGAA
SRR1145005.56580374.2+        CAGATCTGCATGTCTTTTTGCCTACTTGCTTCATACCCACCTGTCTGGCAGAGGAGTGAA
SRR1145005.61551283.2-        CAGATCTGCATGTCTTTTTGCCTACTTGCTTCATACCCACCTGTCTGGCAGAGGAGTGAA
SRR1145005.8349381.2-         CAGATCTGCATGTCTTTTTGCCTACTTGCTTCATACCCACCTGTCTGGCAGAGGAGTGAA
SRR1145006.22933790.2+        CAGATCTGCATGTCTTTTTGCCTACTTGCTTCATACCCACCTGTCTGGCAGAGGAGTGAA
SRR1145006.24052151.2+        CAGATCTGCATGTCTTTTTGCCTACTTGCTTCATACCCACCTGTCTGGCAGAGGAGTGAA
SRR1145006.40154970.2-        CAGATCTGCATGTCTTTTTGCCTACTTGCTTCATACCCACCTGTCTGGCAGAGGAGTGAA
SRR1145006.55037379.1-        CAGATCTGCATGTCTTTTTGCCTACTTGCTTCATACCCACCTGTCTGGCAGAGGAGTGAA
SRR1145007.14404671.2-        CAGATCTGCATGTCTTTTTGCCTACTTGCTTCATACCCACCTGTCTGGCAGAGGAGTGAA
SRR1145007.42050412.1-        CAGATCTGCATGTCTTTTTGCCTACTTGCTTCATACCCACCTGTCTGGCAGAGGAGTGAA
SRR1145007.61371462.1+        CAGATCTGCATGTCTTTTTGCCTACTTGCTTCATACCCACCTGTCTGGCAGAGGAGTGAA
SRR1145007.70295917.1+        CAGATCTGCATGTCTTTTTGCCTACTTGCTTCATACCCACCTGTCTGGCAGAGGAGTGAA
SRR1144992.12221089.1-         AGATCTGCATGTCTTTTTGCCTACTTGCTTCATACCCACCTGTCTGGCAGAGGAGTGAA
SRR1144994.57368222.1+         AGATCTGCATGTCTTTTTGCCTACTTGCTTCATACCCACCTGTCTGGCAGAGGAGTGAA
SRR1145000.54741017.2-          GATCTGCATGTCTTTTTGCCTACTTGCTTCATACCCACCTGTCTGGCAGAGGAGTGAA
SRR1144992.19974964.2-            TCTGCATGTCTTTTTGCCTACTTGCTTCATACCCACCTGTCTGGCAGAGGAGTGAA
SRR1145006.31618019.2+             CTGCATGTCTTTTTGCCTACTTGCTTCATACCCACCTGTCTGGCAGAGGAGTGAA
SRR1145001.24516344.2+               GCATGTCTTTTTGCCTACTTGCTTCATACCCACCTGTCTGGCAGAGGAG
SRR1145006.74715836.1-                  TGTCTTTTTGCCTACTTGCTTCATACCCACCTGTCTGGCAGAGGAGTGAA
SRR1144992.23918808.1-                       CTTTGCCTACTTGCTTCATACCCACCTGTCTGGCAGAGGAGTGAA
SRR1145006.26110484.1-                       TTTTGCCTACTTGCTTCATACCCACCTGTCTGGCAGAGGAGTGAA
SRR1145001.23809804.1-                            CCTACTTGCTTCATACCCACCTGTCTGGCAGAGGAGTGAA
```

```
SRR1144996.61276135.1+                      CTACTTGCTTCATACCCACCTGTCTGGCAGAGGAGTGAA
SRR1144999.53460561.1+                      CTACTTGCTTCATACCCACCTGTCTGGCAGAGGAGTGAA
SRR1145005.30784500.2-                      CTACTTGCTTCATCCCCACCTGTCTGGCAGAGGAGTGAA
SRR1145000.38165410.2+                        ACTTGCTTCATACCCACCTGTCTGGCAGAGGAGTGAA
SRR1144994.59134451.1+                          TTGCTTCATACCCACCTGTCTGGCAGAGGAGTGAA
SRR1145007.7024762.1+                             GCTTCATACCCACCTGTCTGGCAGAGGAGTGAA
SRR1145001.39751305.1-                             CTTCATACCCACCTGTCTGGCAGAGGAGTGAA
SRR1145006.67445686.2+                             CTTCATACCCACCTGTCTGGCAGAGGAGTGAA
SRR1144992.46345616.1+                              TTCATACCCACCTGTCTGGCAGAGGAGTGAA
SRR1144992.46345726.1+                              TTCATACCCACCTGTCTGGCAGAGGAGTGAA
SRR1145000.11168659.2+                               TCATACCCACCTGTCTGGCAGAGGAGTGAA
SRR1145004.51056166.1+                               TCATACCCACCTGTCTGGCAGAGGAGTGAA
SRR1145000.46913317.1+                                CATACCCACCTGTCTGGCAGAGGAGTGAA
SRR1145007.57624437.2-                                  TCCCCACCTGTCTGGCAGAGGAGTGAA
SRR1144993.17373707.2-                                   ACCCACCTGTCTGGCAGAGGAGTGAA
SRR1144999.24418686.1-                                    CCCACCTGTCTGGCAGAGGAGTGAA
SRR1144999.46976359.1+                                    CCCACCTGTCTGGCAGAGGAGTGAA
SRR1144999.48121990.2+                                    CCCACCTGTCTGGCAGAGGAGTGAA
SRR1145001.47766651.2-                                     CCACCTGTCTGGCAGAGGAGTGAA
SRR1145004.72961567.1+                                     CCACCTGTCTGGCAGAGGAGTGAA
SRR1144999.60728096.2+                                      CACCTGTCTGGCAGAGGAGTGAA
SRR1144995.8791417.1+                                        ACCTGTCTGGCAGAGGAGTGAA
SRR1145001.48505232.2+                                       ACCTGTCTGGCAGAGGAGTGAA
SRR1144995.3469204.1-                                          CTGTCTGGCAGAGGAGTGAA
SRR1144994.11187747.2+                                           GTCTGGCAGAGGAGTGAA
SRR1145004.66225478.2-                                           GTCTGGCAGAGGAGTGAA
SRR1144996.47207117.1-                                               GGCAGAGGAGTGAA
SRR1144999.23114265.1-                                                 CAGAGGAGTGAA
SRR1144999.23114265.2+                                                 CAGAGGAGTGAA
SRR1145000.11328614.1+                                                 CAGAGGAGTGAA
SRR1145004.25442647.1-                                                  AGAGGAGTGAA
SRR1145005.18474681.2+                                                   GAGGAGTGAA
SRR1145001.53473888.2-                                                         TGAA
SRR1145001.53473889.2-                                                         TGAA
SRR1145004.19102511.2+                                                           AA
SRR1144996.39150053.2+                                                            A
                         ____________________________________________________________
consensus                CAGATCTGCATGTCTTTTTGCCTACTTGCTTCATACCCACCTGTCTGGCAGAGGAGTGAA

                             .    :    .    :    .    :    .    :    .    :    .    :
SRR1145005.34981199.2+   A
SRR1144998.4243211.2+    AG
SRR1144999.66900124.2+   AG
SRR1144993.38026379.2-   AGCT
SRR1145005.56580374.2+   AGCT
SRR1144998.14176477.1-   AGCTGCTC
SRR1144998.25117498.1-   AGCTGCTC
SRR1144998.46615232.1-   AGCTGCTC
SRR1144998.5699235.1-    AGCTGCTC
SRR1145005.61551283.2-   AGCTGCTC
SRR1145001.23809804.1-   AGCTGCTCA
SRR1144998.28625040.2+   AGCTGGTCAA
SRR1145005.8349381.2-    AGCTGCTCAA
SRR1144996.61276135.1+   CGCTGCTCAAT
SRR1144994.17876275.1-   AGCTGCTCAATA
SRR1144998.21120878.2+   AGCTGCTCAATA
SRR1145004.66225478.1+   AGCTGCTCAATA
SRR1144998.27961014.2+   AGCTGCTCAATAC
SRR1144998.47686489.2+   AGGTGCTCAATACC
SRR1144993.3886278.2+    AGCTGCTCAATACCGg
SRR1145001.39751305.1-   AGCTGCTCAATACCGgt
SRR1145006.40154970.2-   AGCTGCTCAATACCGgt
SRR1144995.3469204.2+    AGCTGCTCAATACCGgta
SRR1145000.54741017.2-   AGCTGCTCAATACCGgta
SRR1144998.67085243.2+   AGCTGCTCAATACCGgtaag
SRR1145000.19173285.2+   AGCTGCTCAATACCGgtaag
SRR1145006.55037379.1-   AGCTGCTCAATACCGgtaag
SRR1144993.907700.2+     AGCTGCTCAATACCGggaagag
SRR1144993.907701.2+     AGCTGCTCAATACCGgtaagagaag
SRR1145001.47766651.2-   AGCTGCTCAATACCGgtaagagaag
SRR1145001.48505232.2+   AGCTGCTCAATACCGgtaagagaagca
SRR1145007.42050412.1-   AGCTGCTCAATACCGgtaagagaagcaaa
SRR1144999.46976359.1+   AGCTGCTCAATACCGgtaagagaagcaaact
SRR1144999.48121990.2+   AGCTGCTCAATACCGgtaagagaagcaaact
SRR1145004.29072333.2+   AGCTGCTCAATACCGgtaagagaagcaaactc
SRR1144994.57031222.2-   AGCTGCTCAATACCGgtaagagaagcaaactca
SRR1145006.24052151.2+   AGCTGCTCAATACCGgtaagagaagcaaactca
SRR1144994.13223558.2+   AGCTGCTCAATACCGgtaagagaagcaaactcat
SRR1144999.23114265.2+   AGCTGCTCAATACCGgtaagagaagcaaactcatag
SRR1145006.22933790.2+   AGCTGCTCAATACCGgtaagagaagcaaactcatag
SRR1145007.14404671.2-   AGCTGCTCAATACCGgtaagagaagcaaactcatagtgtt
SRR1145007.61371462.1+   AGCTGCTCAATACCGgtaagagaagcaaactcatagtgtt
SRR1145007.70295917.1+   AGCTGCTCAATACCGgtaagagaagcaaactcatagtgtt
SRR1144992.12221089.1-   AGCTGCTCAATACCGgtaagagaagcaaactcatagtgttg
```

```
SRR1144994.57368222.1+   AGCTGCTCAATACCGgtaagagaagcaaactcatagtgttg
SRR1144992.19974964.2-   AGCTGCTCAATACCGgtaagagaagcaaactcatagtgttgctg
SRR1145001.53473888.2-   AGCTGCTCAATACCGgtaagagaagcaaactcatagtgttgctgc
SRR1145001.53473889.2-   AGCTGCTCAATACCGgtaagagaagcaaactcatagtgttgctgc
SRR1145006.31618019.2+   AGCTGCTCAATACCGgtaagagaagcaaactcttagtgttgctgc
SRR1145006.74715836.1-   AGCTGCTCAATACCGgtaagagaagcaaactcatagtgttgctgcctatg
SRR1144999.53460561.1+   AGCTGCTCAATACCGgtaagagaagcaaactcatagtattgctgcctatgc
SRR1145000.38165410.2+   AGCTGCTCAATACCGgtaagagaagcaaactcatagtattgctgcctatgcca
SRR1144992.23918808.1-   AGCTGCTCAATACCGgtaagagaagcaaactcatagtgttgctgcctatgccaca
SRR1145006.26110484.1-   AGCTGCTCAATACCGgtaagagaagcaaactcatagtgttgctgcctatgccaca
SRR1144992.46345616.1+   AGCTGCTCAATACCGgtaagagaagcaaactcatagtgttgctgcctatgccacaggatc
SRR1144992.46345726.1+   AGCTGCTCAATACCGgtaagagaagcaaactcatagtgttgctgcctatgccacaggatc
SRR1144993.17373707.2-   AGCTGCTCAATACCGgtaagagaagcaaactcatagtgttgctgcctatgccacaggatc
SRR1144994.11187747.2+   AGCTGCTCAATACCGgtaagagaagcaaactcatagtgttgctgcctatgccacaggatc
SRR1144994.59134451.1+   AGCTGCTCAATACCGgtaagagaagcaaactcatggtgttgctgcccatgccacagggtc
SRR1144995.3469204.1-    AGCTGCTCAATACCGgtaagagaagcaaactcatagtgttgctgcctatgccacaggatc
SRR1144995.8791417.1+    AGCTGCTCAATACCGgtaagagaagcaaactcatagtgttgctgcctatgccacaggatc
SRR1144996.39150053.2+   AGCTGCTCAATACCGgtaagagaagcaaactcatagtgttgctgcctatgccacaggatc
SRR1144996.47207117.1-   AGCTGCTCAATACCGgtaagagaagcaaactcatagtgttgctgcctatgccacaggatc
SRR1144999.23114265.1-   AGCTGCTCAATACCGgtaagagaagcaaactcatagtattgctgcctatgccacaggatc
SRR1144999.24418686.1-   AGCTGCTCAATACCGgtaagagaagcaaactcatagtgttgctgcctatgccacaggatc
SRR1144999.60728096.2+   AGCTGCTCAATACCGgtaagagaagcaaactcatagtgttgctgcctatgccacaggatc
SRR1145000.11168659.2+   AGCTGCTCAATACCGgtaagagaagcaaactcatagtattgctgcctatgccacaggatc
SRR1145000.11328614.1+   AGCTGCTCAATACCGgtaagagaagcaaactcatagtattgctgcctatgccacaggatc
SRR1145000.24268485.1-   AGCTGCTCAATACCGggaagagaagcaaactcatggtattgctgcctatgccacaggatc
SRR1145000.46913317.1+   AGCTGCTCAATACCGgtaagagaagcaaactcatagtattgctgcctatgccacaggatc
SRR1145004.19102511.2+   AGCTGCTCAATACCGgtaagagaagcaaactcatagtgttgctgcctatgccacaggatc
SRR1145004.25442647.1-   AGCTGCTCAATACCGgtaagagaagcaaactcatagtgttgctgcctatgccacaggatc
SRR1145004.51056166.1+   AGCTGCTCAATACCGgtaagagaagcaaacccatagtgttgctgcctatgccacaggacc
SRR1145004.66225478.2-   AGCTGCTCAATACCGgtaagagaagcaaactcatagtgttgctgcctatgccacaggatc
SRR1145004.72961567.1+   AGCTGCTCAATACCGgtaagagaagcaaactcatagtgttgctgcctatgccacaggatc
SRR1145005.18474681.2+   AGCTGCTCAATACCGgtaagagaagcaaactcatagtgttgctgcctatgccacaggatc
SRR1145005.30784500.2-   AGCTGCTCAATACCGgtaagagaagcaaactcatagtgttgctgcctatgccacaggatc
SRR1145006.43186574.1+   AGCTGCTCAATACCGgtaagagaagcaaactcatagtgttgctgcctatgccacaggatc
SRR1145006.67445686.2+   AGCTGCTCAATACCGgtaagagaagcaaactcatagtgttgctgcctatgccacaggatc
SRR1145007.57624437.2-   AGCTGCTCAATACCGgtaagagaagcaaactcatagtgttgctgcctatgccacaggatc
SRR1145007.7024762.1+    AGCTGCTCAATACCGgtaagagaagcaaactcatagtgttgctgcctatgccacaggatc
SRR1144997.44664327.2-    GCTGCTCAATACCGgtaagagaagcaaactcatagtattgctgcctatgccacaggatc
SRR1144997.5975173.2-     GCTGCTCAATACCGgtaagagaagcaaactcatagtattgctgcctatgccacaggatc
SRR1145004.20545723.1+    GCTGCTCAATACCGgtaagagaagcaaactcatagtgttgctgcctatgccacaggatc
SRR1145001.17796305.1+     CTGCTCAATACCGgtaagagaagcaaactcatagtgttgctgcctatgc
SRR1144994.7728093.1+      CTGCTCAATACCGgtaagagaagcaaactcatagtgttgctgcctatgccacaggatc
SRR1144994.13223558.1-       GCTCAATACCGgtaagagaagcaacctcatagttttgctgcctatgccacaggatc
SRR1144999.38456569.1-       GCTCAATACCGgtaagagaagcaaactcatagtgttgctgcctatgccacaggatc
SRR1145006.59125753.2+       GCTCAATACCGgtaagagaagcaaactcatagtgttgctgcctatgccacaggatc
SRR1144994.51370169.1+        CTCAATACCGgtaagagaagcaaactcatagtgttgctgcctatgccacaggatc
SRR1144997.56753447.2-        CTCAATACCGgtaagagaagcaaactcatagtgttgctgcctatgccacaggatc
SRR1144994.57368222.2-         TCAATACCGgtaagagaagcaaactcatagtgttgctgcctatgccacaggatc
SRR1145005.35137077.2+         TCAATACCGgtaagagaagcaaactcatagtgttgctgcctatgccacaggatc
SRR1145006.36863603.2+         TCAATACCGgtaagagaagcaaactcatagtgttgctgcctatgccacaggatc
SRR1145007.12693144.2+          CAATACCGgtaagagaagcaaactcatagtgttgctgcctatgccacaggatc
SRR1145000.24983742.1+            ATACCGgtaagagaagcaaactcatagtattgctgcctatgccacaggatc
SRR1145001.33021318.1+             TACCGgtaagagaagcaaactcatagtattgctgcctatgccacagga
SRR1144992.29437553.2+               CCGgtaagagaagcaaactcatagtgttgctgcctatgccacaggatc
SRR1144994.32110124.2+               CCGgtaagagaagcaaactcatagtgttgctgcctatgccacaggatc
SRR1145004.29072333.1-                GGgtaagagaagcaaactcatagtgttcctgcctatgccacaggatc
SRR1145007.63998670.2+                GGgtaagagaagcaaactcatagtgttgctgcctatgccacaggatc
SRR1145007.70295917.2-                GGgtaagagaagcaaactcatagtgttgctgcctatgccacaggatc
SRR1145001.73025495.2-                 Ggtaagagaagcaaactcatagtattgctgcctatgccacaggatc
                         ____________________________________________________________
consensus                AGCTGCTCAATACCGgtaagagaagcaaactcatagtgttgctgcctatgccacaggatc
```

Z. Northern carmine bee-eater (No. 19), exon 9, WGS data

- CAP3 alignment of WGS data spanning exon 9 (uppercase letters)

```
                             .    :    .    :    .    :    .    :    .    :    .    :
SRR958515.20550832.1+    acttcataactttcttcccttctctgtctttcccagGAAT
SRR958516.84222393.2-    acttcataactttcttcccttctctgtctttcccagGAATGG
SRR958516.7111266.1-     acttcataactttcttcccttctctgtctttcccagGAATGGTGA
SRR958514.97072629.2-    acttcataactttcttcccttctctgtctttcccagGAATGGTGAG
SRR958516.21534044.1+    acttcataactttcttcccttctctgtctttcccagGAATGGTGAGCA
SRR958514.33856931.1+    acttcataactttcttcccttctctgtctttcccagGAATGGTGAGCAG
SRR958514.78934248.2+    acttcataactttcttcccttctctgtctttcccagGAATGGTGAGCAGCT
SRR958515.42200275.1+    acttcataactttcttcccttctctgtctttcccagGAATGGTGCGCAGCT
SRR958515.30508980.1+    acttcataactttcttcccttctctgtctttcccagGAATGGTGAGCAGCTAAGGA
SRR958514.100160626.2-   acttcataactttcttcccttctctgtctttcccagGAATGGTGAGCAGCTAAGGATCAT
SRR958514.101956646.1+   acttcataactttcttcccttctctgtctttcccagGAATGGTGAGCAGCTAAGGATCAT
SRR958514.102512771.2+   acttcataactttcttcccttctctgtctttcccagGAATGGTGAGCAGCTAAGGATCAT
SRR958514.103218794.1-   acttcataactttcttcccttctctgtctttcccagGAATGGTGAGCAGCTAAGGATCAT
SRR958514.21924864.1+    acttcataactttcttcccttctctgtctttcccagGAATGGTGAGCAGCTAAGGATCAT
SRR958514.34612862.1-    acttcataactttcttcccttctctgtctttcccagGAATGGTGAGCAGCTAAGGATCAT
SRR958514.37021802.1+    acttcataactttcttcccttctctgtctttcccagGAATGGTGAGCAGCTAAGGATCAT
SRR958514.38714466.1+    acttcataactttcttcccttctctgtctttcccagGAATGGTGAGCAGCTAAGGATCAT
SRR958514.41207065.1-    acttcataactttcttcccttctctgtctttcccagGAATGGTGAGCAGCTAAGGATCAT
SRR958514.44130169.2+    acttcataactttcttcccttctctgtctttcccagGAATGGTGAGCAGCTAAGGATCAT
SRR958515.10722057.1+    acttcataactttcttcccttctctgtctttcccagGCATGGTGAGAAGCTAAGGATCAT
SRR958515.17031678.2-    acttcataactttcttcccttctctgtctttcccagGAATGGTGAGCAGCTAAGGATCAT
SRR958515.23577348.1+    acttcataactttcttcccttctctgtctttcccagGAATGGTGAGCAGCTAAGGATCAT
SRR958515.48073369.1-    acttcataacttgcttcccttctctgtctttcccagGAATGGTGAGCAGCTAAGGATCAT
SRR958515.50733784.2+    acttcataactttcttcccttctctgtctttcccagGAATGGTGAGCAGCTAAGGATCAT
SRR958516.19993891.2+    acttcataactttcttcccttctctgtctttcccagGAATGGTGAGCAGCTAAGGATCAT
SRR958516.58853563.2-    acttcataactttcttcccttctctgtctttcccagGAATGGTGAGCAGCTAAGGATCAT
SRR958516.68750980.2+    acttcataactttcttcccttctctgtctttcccagGAATGGTGAGCAGCTAAGGATCAT
SRR958516.80803481.1-    acttcataactttcttccctgctctgtctttcccagGAATGGTGAGCAGCTAAGGATCAT
SRR958516.8984891.1-     acttcataactttcttcccttctctgtctttcccagGAATGGTGAGCAGCTAAGGATCAT
SRR958516.91823061.1-    acttcataactttcttcccttctctgtctttcccagGAATGGTGAGCAGCTAAGGATCAT
SRR958516.75198625.2+     cttcataactttcttcccttctctgtctttcccagGAATGGTGAGCAGCTAAGGATCAT
SRR958514.43162277.2-      ttcataactttcttcccttctctgtctttcccagGAATGGTGAGCAGCTAAGGATCAT
SRR958514.53475084.2-      ttcataactttcttcccttctctgtctttcccagGAATGGTGAGCAGCTAAGGATCAT
SRR958516.19559192.1-      ttcataactttcttcccttctctgtctttcccagGAATGGTGAGCAGCTAAGGATCAT
SRR958516.59335252.1+        cataactttcttcccttctctgtctttcccagGAATGGTGAGCAGCTAAGGATCAT
SRR958514.68561080.2+         ataactttcttcccttctctgtctttcccagGAATGGTGAGCAGCTAAGGATCAT
SRR958514.19678943.2+          taactttcttcccttctctgtctttcccagGAATGGTGAGCAGCTAAGGATCAT
SRR958514.70551700.2-            actttcttcccttctctgtctttcccagGAATGGTGAGCAGCTAAGGATCAT
SRR958514.4761863.2-              ctttcttcccttctctgtctttcccagGAATGGTGAGCAGCTAAGGATCAT
SRR958516.8354132.2-              ctttcttcccttctctgtctttcccagGAATGGTGAGCAGCTAAGGATCAT
SRR958516.9819331.2+              ctttcttcccttctctgtctttcccagGAATGGTGAGCAGCTAAGGATCAT
SRR958516.45410459.1-                 cttcccttctctgtctttcccagGAATGGTGAGCAGCTAAGGATCAT
SRR958516.56305879.1+                 cttcccttctctgtctttcccagGAATGGTGAGCAGCTAAGGATCAT
SRR958515.46041706.2-                  ttcccttctctgtcttaccctgGAATGGTGATCAGCTAAGGATCAT
SRR958514.87697778.2+                     ccttctctgtctttcccagGAATGGTGAGCAGCTAAGGATCAT
SRR958514.13731632.2+                      cttctctgtctttcccagGAATGGTGAGCAGCTAAGGATCAT
SRR958516.78560384.2-                      cttctctgtctttcccagGAAGTGTGAGCAGCTAAGGATCAT
SRR958514.69289961.1+                        tctctgtctttcccagGAATGGTGAGCAGCTAAGGATCAT
SRR958516.83788257.2+                        tctctgtctttcccagGAATGGTGAGCAGCTAAGGATCAT
SRR958515.46791599.1+                          tctgtctttcccagGAATGGTGAGCAGCTAAGGATCAT
SRR958516.26364114.1-                           ctgtctttcccagGAATGGTGAGCAGCTAAGGATCAT
SRR958515.55934198.2-                             gtctttcccagGAATGGTGAGCAGCTAAGGATCAT
SRR958516.72519694.2-                                  tcccagGAATGGTGAGCAGCTAAGGATCAT
SRR958515.20769019.2+                                   cccagGAATGGTGAGCAGCTAAGGATCAT
SRR958515.21148416.2-                                    ccagGAATGGTGAGCAGCTAAGGATCAT
SRR958514.66386993.2-                                     cagGAATGGTGAGCAGCTAAGGATCAT
SRR958516.62012920.1-                                     cagGAATGGTGAGCAGCTAAGGATCAT
SRR958514.33902979.1+                                      agGAATGGTGAGCAGCTAAGGATCAT
SRR958515.26701002.2+                                      agGAATGGTGAGCAGCTAAGGATCAT
SRR958516.56093803.1+                                       gGAATGGTGAGCAGCTAAGGATCAT
SRR958514.22377625.1+                                        GAATGGTGAGCAGCTAAGGATCAT
SRR958516.70437229.1-                                          ATGGTGAGCAGCTAAGGATCAT
SRR958514.100735365.2+                                          TGGTGAGCAGCTAAGGATCAT
SRR958514.15141129.2-                                                  CAGCTAAGGATCAT
SRR958515.36716288.2+                                                    GCTAAGGATCAT
SRR958516.83781102.2+                                                    GCTAAGGATCAT
SRR958516.49821190.1+                                                      TAAGGATCAT
SRR958516.9605271.2-                                                         AGGATCAT
SRR958515.32552432.2-                                                         GGATCAT

consensus                acttcataactttcttcccttctctgtctttcccagGAATGGTGAGCAGCTAAGGATCAT

                             .    :    .    :    .    :    .    :    .    :    .    :
SRR958514.37021802.1+    C
SRR958514.38714466.1+    CTGTG
```

```
SRR958516.68750980.2+    CTGTG
SRR958515.10722057.1+    CTGCGAGG
SRR958515.48073369.1-    CTGTGAGG
SRR958514.21924864.1+    CTGTGAGGA
SRR958515.17031678.2-    CTGTGAGGACAGTA
SRR958515.50733784.2+    CTGTGAGGACAATA
SRR958516.91823061.1-    CTGTGAGGACAGTA
SRR958514.103218794.1-   CTGTGAGGACAATAAG
SRR958516.58853563.2-    CTGTGAGGACAATAAGTA
SRR958514.44130169.2+    CTGTGAGGACAATAAGTATG
SRR958514.19678943.2+    CTGTGAGGACAGTAAGTATGA
SRR958514.100160626.2-   CTGTGAGGACAGTAAGTATGACTTCA
SRR958514.34612862.1-    CTGTGAGGACAGTAAGTATGACTTCAGG
SRR958515.23577348.1+    CTGTGAGGACAATAAGTATGACTTCAGGACA
SRR958516.19993891.2+    CTGTGAGGACAGTAAGTATGACTTCAGGACA
SRR958514.41207065.1-    CTGTGAGGACAGTAAGTATGACTTCAGGACATT
SRR958516.8984891.1-     CTGTGAGGACAATAAGTATGACTTCAGGACATTG
SRR958516.80803481.1-    CTGTGAGGACAATAAGTATGACTTCAGGACATTGAG
SRR958514.102512771.2+   CTGTGAGGACAATAAGTATGACTTCAGGACATTGAGG
SRR958514.101956646.1+   CTGTGAGGACAGTAAGTATGACTTCAGGACATTGAGGAC
SRR958514.53475084.2-    CTGTGAGGACAGTAAGTATGACTTCAGGACATTGAGGACAT
SRR958516.75198625.2+    CTGTGAGGACAGTAAGTATGACTTCAGGACATTGAGGACAT
SRR958514.43162277.2-    CTGTGAGGACAGTAAGTATGACTTCAGGACATTGAGGACATG
SRR958516.19559192.1-    CTGTGAGGACAGTAAGTATGACTTCAGGACATTGAGGACATG
SRR958516.59335252.1+    CTGTGAGGACAATAAGTATGACTTCAGGACATTGAGGACATGAA
SRR958514.68561080.2+    CTGTGAGGACAATAAGTATGACTTCAGGACATTGAGGACATGAAG
SRR958515.46041706.2-    CTGTGAGGAGAATAAGTATGACTTCAGGACATTGAGGACATGAAG
SRR958516.8354132.2-     CTGTGAGGACAATAAGTATGACTTCAGGACATTGAGGACATGAAGGA
SRR958514.70551700.2-    CTGTGAGGACAGTAAGTATGACTTCAGGACATTGAGGACATGAAGGAA
SRR958514.4761863.2-     CTGTGAGGACAATAAGTATGACTTCAGGACATTGAGGACATGAAGGAAA
SRR958516.9819331.2+     CTGTGAGGACAGTAAGTATGACTTAAGGCCATTGAGGACATGAAGGAAA
SRR958516.45410459.1-    CTGTGAGGACAATAAGTATGACTTCAGGACATTGAGGACATGAAGGAAATTGT
SRR958516.56305879.1+    CTGTGAGGACAGTAAGTATGACTTCAGGACATTGAGGACATGAAGGAAATTGT
SRR958514.87697778.2+    CTGTGAGGACAGTAAGTATGACTTCAGGACATTGAGGACATGAAGGAAATTGTGATA
SRR958514.13731632.2+    CTGTGAGGACAGTAAGTATGACTTCAGGACATTGAGGACATGAAGGAAATTGTGATAA
SRR958516.78560384.2-    CTGTGAGGACAATAAGTATGACTTCAGGACATTGAGGACATGAAGGAAATTGTGATAA
SRR958514.100735365.2+   CTGTGAGGACAGTAAGTATGACTTCAGGACATTGAGGACATGAAGGAAATTGTGATAACC
SRR958514.15141129.2-    CTGTGAGGACAATAAGTATGACTTCAGGACATTGAGGACATGAAGGAAATTGTGATAACC
SRR958514.22377625.1+    CTGTGAGGACAGTAAGTATGACTTCAGGACATTGAGGACATGAAGGAAATTGTGATAACC
SRR958514.33902979.1+    CTGTGAGGACAATAAGTATGACTTCAGGACATTGAGGACATGAAGGAAATTGTGATAACC
SRR958514.66386993.2-    CTGTGAGGACAATAAGTATGACTTCAGGACATTGAGGACATGAAGGAAATTGTGATAACC
SRR958514.69289961.1+    CTGTGAGGACAGTAAGTATGACTTCAGGACATTGAGGACATGAAGGAAATTGTGATAACC
SRR958515.20769019.2+    CTGTGAGGACAGTAAGTATGACTTCAGGACATTGAGGACATGAAGGAAATTGTGATAACC
SRR958515.21148416.2-    CTGTGAGGACAATAAGTATGACTTCAGGACATTGAGGACATGAAGGAAATTGTGATAACC
SRR958515.26701002.2+    CTGTGAGGACAATAAGTATGACTTCAGGACATTGAGGACATGAAGGAAATTGTGATAACC
SRR958515.32552432.2-    CTGTGAGGACAATAAGTATGACTTCAGGACATTGAGGACATGAAGGAAATTGTGATAACC
SRR958515.36716288.2+    CTGTGAGGACAATAAGTATGACTTCAGGACATTGAGGACATGAAGGAACTTGTGATAACC
SRR958515.46791599.1+    CTGTGAGGACAGTAAGTATGACTTCAGGACATTGAGGACATGAAGGAAATTGTGATAACC
SRR958515.55934198.2-    CTGTGAGGACAATAAGTATGACTTCAGGACATTGAGGACATGAAGGAAATTGTGATAACC
SRR958516.26364114.1-    CTGTGAGGACAATAAGTATGACTTCAGGACATTGAGGACATGAAGGAAATTGTGATAACC
SRR958516.49821190.1+    CTGTGAGGACAGTAAGTATGACTTCAGGACATTGAGGACATGAAGGAAATTGTGATAACC
SRR958516.56093803.1+    CTGTGAGGACAATAAGTATGACTTCAGGACATTGAGGACATGAAGGAAATTGTGATAACC
SRR958516.62012920.1-    CTGTGAGGACAGTAAGTATGACTTCAGGACATTGAGGACATGAAGGAAATTGTGATAACC
SRR958516.70437229.1-    CTGTGAGGACAATAAGTATGACTTCAGGACATTGAGGACATGAAGGAAATTGTGATAACC
SRR958516.72519694.2-    CTGTGAGGACAGTAAGTATGACTTCAGGACATTGAGGACATGAAGGAAATTGTGATAACC
SRR958516.83781102.2+    CTGTGAGGACAGTAAGTATGACTTCAGGACATTGAGGACATGAAGGAAATTGTGATAACC
SRR958516.83788257.2+    CTGTGAGGACAATAAGTATGACTTCAGGACATTGAGGACATGAAGGAAATTGTGATAACC
SRR958516.9605271.2-     CTGTGAGGACAATAAGTATGACTTCAGGACATTGAGGACATGAAGGAAATTGTGATAACC
SRR958516.79663606.1+           GACAATAAGTATGACTTCAGGACATTGAGGACATGAAGGAAATTGTGATAACC
SRR958516.82469221.2-           GACAATAAGTATGACTTCAGGACATTGAGGACATGAAGGAAATTGTGATAACC
SRR958515.50726151.1-               ATAAGTATGACTTCAGGACATTGAGGACATGAAGGAAATTGTGATAACC
SRR958516.34790766.2+                         CTTCAGGACATTGAGGACATGAAGGAAATTGTGATAACC
SRR958514.23117107.1+                            CAGGACATTGAGGACATGAAGGAAATTGTGATAACC
SRR958516.62641480.2+                                      AGGACATGAAGGAAATTGTGATAACC
SRR958515.52334685.2+                                        GACATGAAGGAAATTGTGATAACC
SRR958515.26161489.1-                                           ATGAAGGAAATTGTGATAACC
SRR958514.19677880.2+                                            TGAAGGAAATTGTGATAACC
SRR958515.59157021.2-                                            TGAAGGAAATTGTGATAACC
SRR958516.72658279.1+                                             GAAGGAAATTGTGATAACC
SRR958515.46572499.2-                                              AAGGAAAGTGTGATAACA
SRR958514.28785838.2+                                                  AAATTGTGATAACC
SRR958516.90165724.1-                                                   AATTGTGATAACC
SRR958515.31117436.1-                                                      TGTGATAACC
SRR958516.38045271.1-                                                          ATAACC
SRR958514.97294212.1-                                                           TAACA

consensus                CTGTGAGGACAATAAGTATGACTTCAGGACATTGAGGACATGAAGGAAATTGTGATAACC

                             .    :    .    :    .    :    .    :    .    :    .    :
SRR958515.46791599.1+    AA
SRR958516.26364114.1-    AAA
SRR958515.55934198.2-    AAACC
SRR958516.62012920.1-    AAACCAgta
SRR958516.72519694.2-    AAACCAgtaa
```

```
SRR958515.20769019.2+    AAACCAgtaag
SRR958515.21148416.2-    AAACCAgtaaga
SRR958514.66386993.2-    AAACCAgtaagat
SRR958514.33902979.1+    AAACCAgtaagatc
SRR958515.26701002.2+    AAACCAgtaagatc
SRR958516.56093803.1+    AAACCAgtaagatcc
SRR958514.22377625.1+    AAACCAgtaagatcct
SRR958516.70437229.1-    AAACCAgtaagatccttc
SRR958514.100735365.2+   AAACCAgtaagatccttcc
SRR958514.15141129.2-    AAACCAgtaagatccttccctgtgac
SRR958515.36716288.2+    AAACCAgtaagatccttcactgtgacac
SRR958516.83781102.2+    AAACCAgtaagatccttccctgtgacac
SRR958516.49821190.1+    AAACCAgtaagatccttccctgtgacacag
SRR958516.9605271.2-     AAACCAgtaagatccttccctgtgacacagcc
SRR958515.32552432.2-    AAACCAgtaagatccttccctgtgacacagcca
SRR958516.79663606.1+    AAACCAgtaagatccttcactgtgacacagccacgggcaggcagcct
SRR958516.82469221.2-    AAACCAgtaagatccttccctgtgacacagccacgggcaggcagcct
SRR958515.50726151.1-    AAACCAgtaagatccttccctgtgacacagccacgggcaggcagcctgggg
SRR958516.34790766.2+    AAACCAgtaagatccttccctgtgacacagccacgggcaggcagcctggggagg
SRR958514.19677880.2+    AAACCAgtaagatccttccctgtgacacagccacgggcaggcagcctggggaggtggtga
SRR958514.23117107.1+    AAACCAgtaagatccttccctgtgacacagccacgggcaggcagcctggggaggtggtga
SRR958514.28785838.2+    AAACCAgtaagatccttccctgtgacacagccacgggcaggcagcctggggaggtggtga
SRR958514.70127072.1+    AAACCAgtaagatccttccctgtgacacagccacgggcaggcagcctggggaggtggtga
SRR958514.80404086.1+    AAACCAgtaagatccttccctgtgacacagccacgggcaggcagcctggggaggtggtga
SRR958514.97294212.1-    AAACCAgtaagctcgttccctgtgacacagccacgggcaggcagcctggggaggtggtga
SRR958515.26161489.1-    AAACCAttaagacccttccctgtgacccagccacgggcaggcagcctggggaggtggtga
SRR958515.31117436.1-    AAACCAgtaagatccttccctgtgacacagccacgggcaggcagcctggggaggtggtga
SRR958515.46572499.2-    AAACCAgtaagatccttccctgtgacacagccacgggcaggcagcctggggaggtggtga
SRR958515.52334685.2+    AAACCAgtaagatccttccctgtgacacagccacgggcaggcagcctggggaggtggtga
SRR958515.59157021.2-    AAACCAgtaagatccttccctgtgacacagccacgggcaggcagcctggggaggtggtga
SRR958516.1104583.2-     AAACCAgtaagatccttccctgggacacagccacgggcaggcagcctggggaggtggtga
SRR958516.38045271.1-    AAACCAgtaagatccttccctgtgacacagccacgggcaggcagcctggggaggtggtga
SRR958516.62641480.2+    AAACCAgtaagatccttccctgtgacacagccacgggcaggcagcctggggaggtggtga
SRR958516.72658279.1+    AAACCAgtaagatccttccctgtgacacagccacgggcaggcagcctggggaggtggtga
SRR958516.90165724.1-    AAACCAgtaagatccttccctgtgacacagccacgggcaggcagcctggggaggtggtga
SRR958515.38576117.1-     AACCAgtaagatcctgccctgtgacacagccacgggcaggcagcctggggaggtggtga
SRR958514.62839659.1+        CAgtaagatccttccctgtgacacagccacgggcaggcagcctggggcggtggtga
                         ____________________________________________________________
consensus                AAACCAgtaagatccttccctgtgacacagccacgggcaggcagcctggggaggtggtga
```
