# Supplementary material for: MOXD2, a Gene Possibly Associated with Olfaction, Is Frequently Inactivated in Birds
Source: PLoS One. 2016 Apr 13;11(4):e0152431. doi: 10.1371/journal.pone.0152431 (PMC4830563; doi:10.1371/journal.pone.0152431)
Supplement: S5 Fig — (PDF) [file pone.0152431.s005.pdf]

S5 Fig. Dotplot analysis of *MOXD2* genomic sequences

No. 1
Order: Passeriformes
Family: Acanthisittidae
Scientific name: *Acanthisitta chloris*
Common name: Rifleman

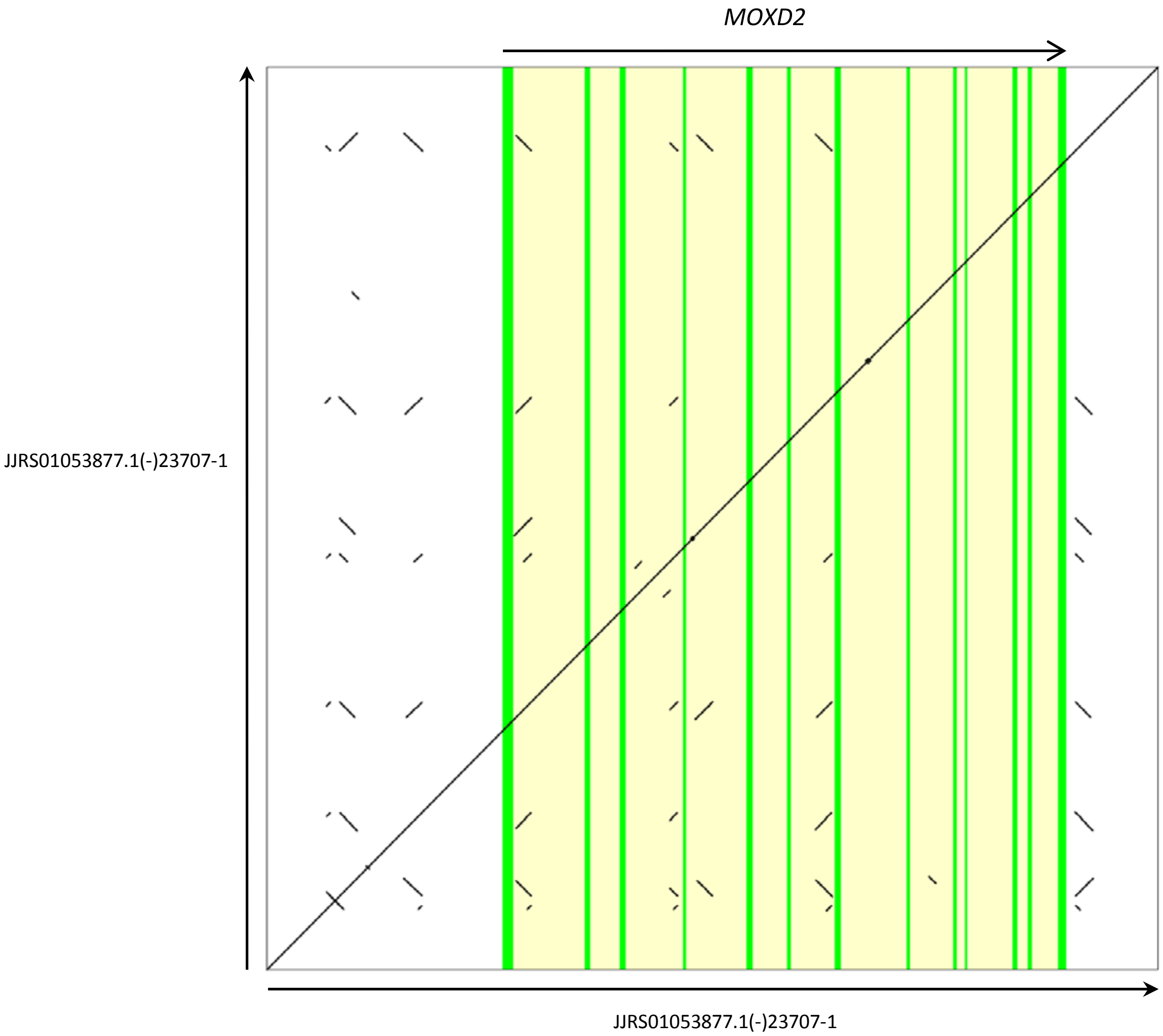

Order: Passeriformes
Family: Acanthisittidae
Scientific name: *Acanthisitta chloris*
Common name: Rifleman

No. 2
Order: Passeriformes
Family: Pipridae
Scientific name: *Manacus vitellinus*
Common name: Golden-collared manakin

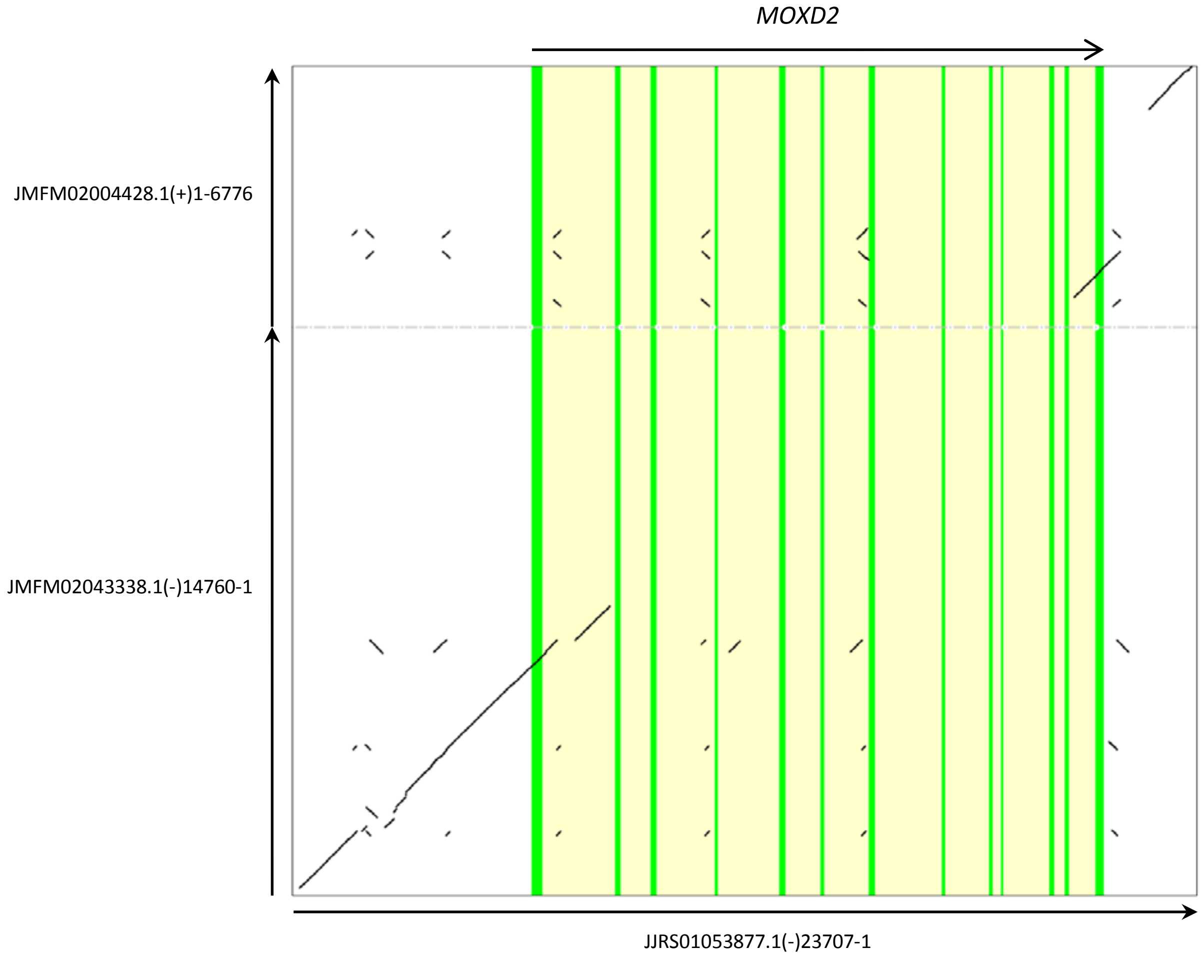

Order: Passeriformes
Family: Acanthisittidae
Scientific name: *Acanthisitta chloris*
Common name: Rifleman

No. 3
Order: Passeriformes
Family: Corvidae
Scientific name: *Corvus brachyrhynchos*
Common name: American crow

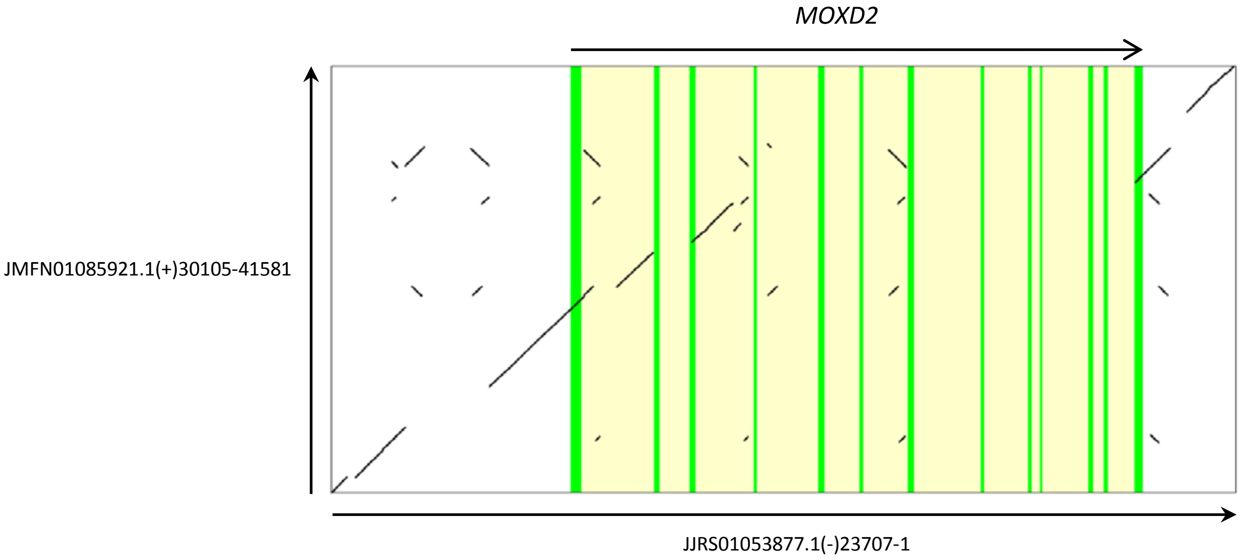

Order: Passeriformes
Family: Acanthisittidae
Scientific name: *Acanthisitta chloris*
Common name: Rifleman

No. 4
Order: Passeriformes
Family: Corvidae
Scientific name: *Corvus cornix cornix*
Common name: Hooded crow

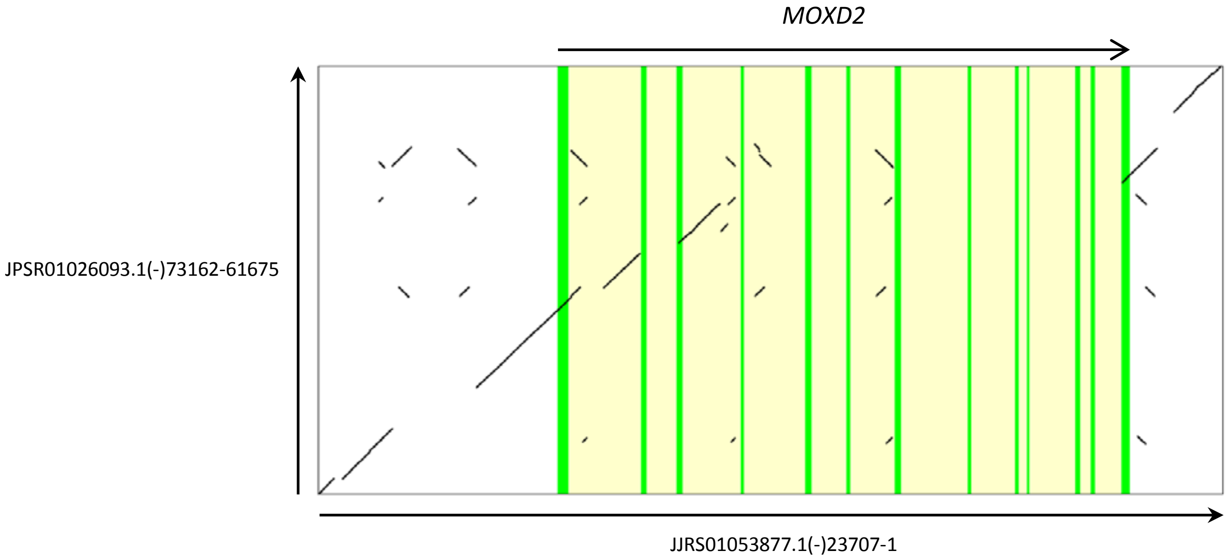

Order: Passeriformes
Family: Acanthisittidae
Scientific name: *Acanthisitta chloris*
Common name: Rifleman

No. 5
Order: Passeriformes
Family: Paridae
Scientific name: *Pseudopodoces humilis*
Common name: Ground tit

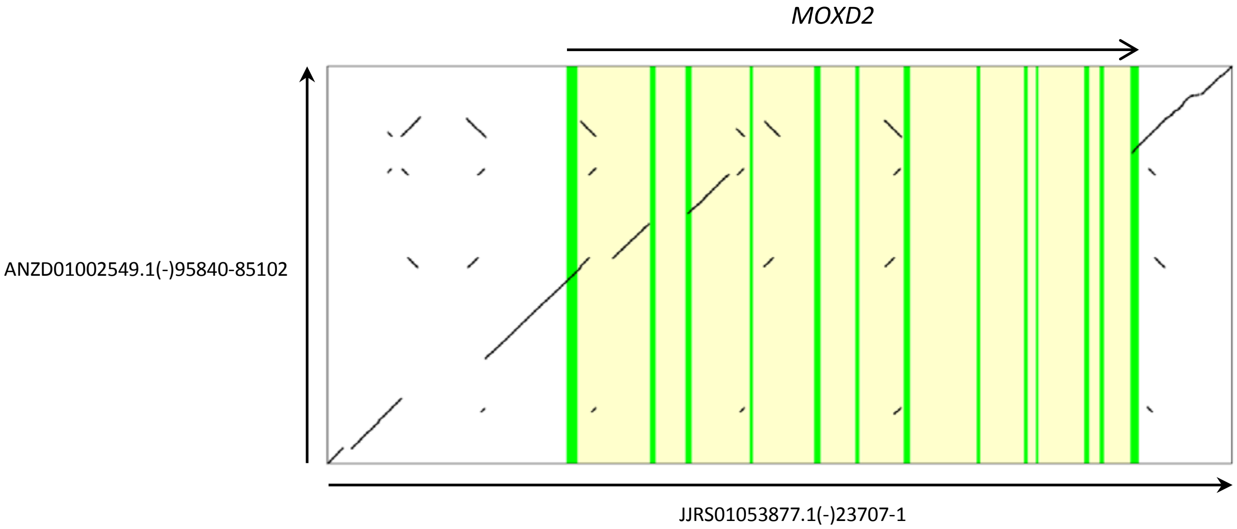

Order: Passeriformes
Family: Acanthisittidae
Scientific name: *Acanthisitta chloris*
Common name: Rifleman

No. 6
Order: Passeriformes
Family: Muscicapidae
Scientific name: *Ficedula albicollis*
Common name: Collared flycatcher

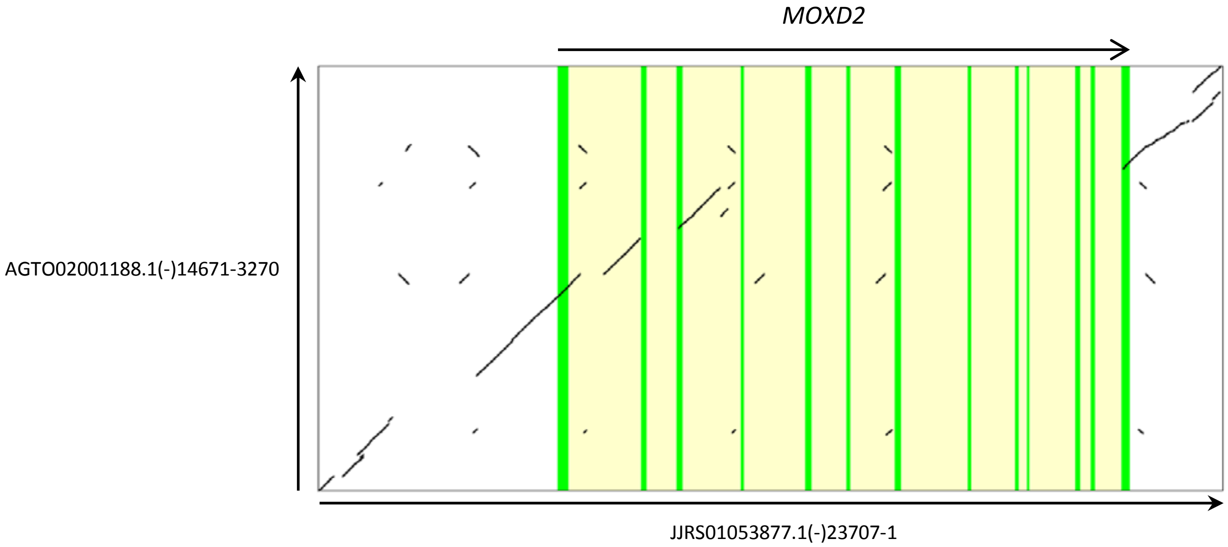

Order: Passeriformes
Family: Acanthisittidae
Scientific name: *Acanthisitta chloris*
Common name: Rifleman

No. 7
Order: Passeriformes
Family: Estrildidae
Scientific name: *Taeniopygia guttata*
Common name: Zebra finch

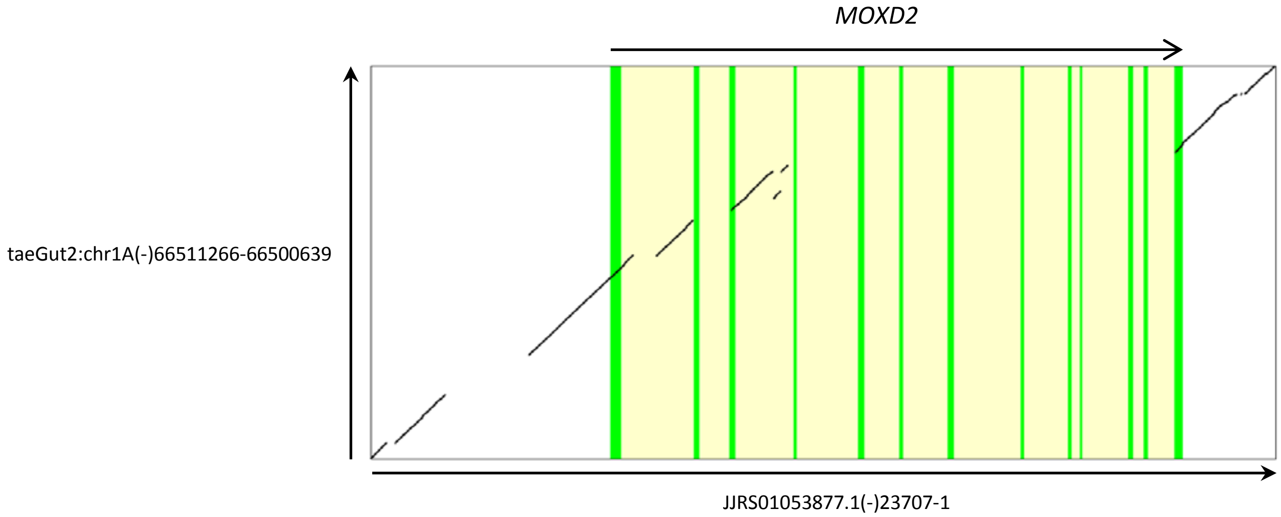

Order: Passeriformes
Family: Acanthisittidae
Scientific name: *Acanthisitta chloris*
Common name: Rifleman

No. 8
Order: Passeriformes
Family: Fringillidae
Scientific name: *Serinus canaria*
Common name: Atlantic canary

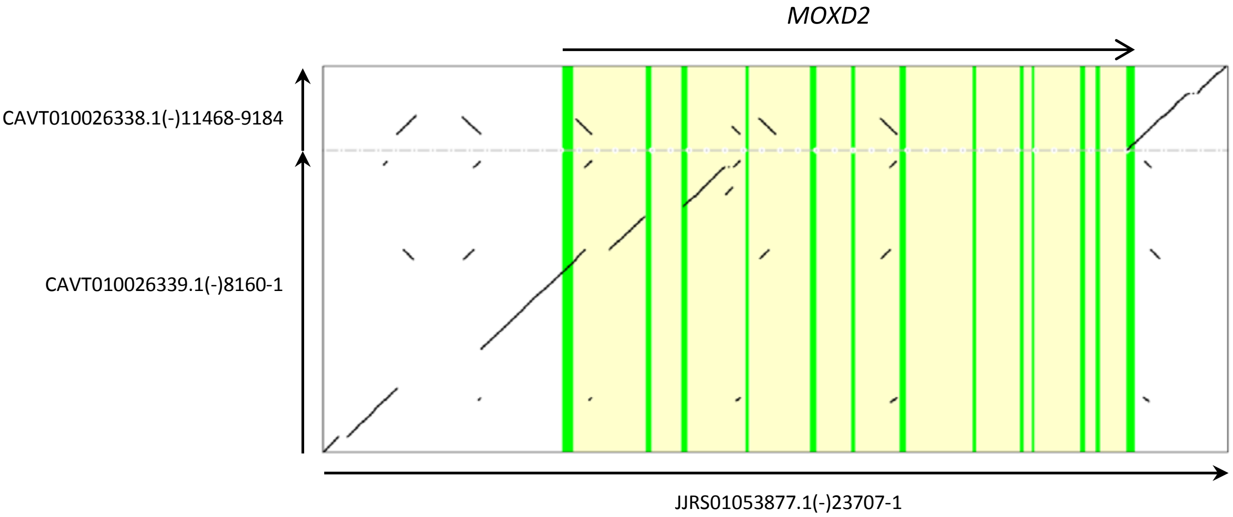

Order: Passeriformes
Family: Acanthisittidae
Scientific name: *Acanthisitta chloris*
Common name: Rifleman

No. 9
Order: Passeriformes
Family: Emberizidae
Scientific name: *Geospiza fortis*
Common name: Medium ground finch

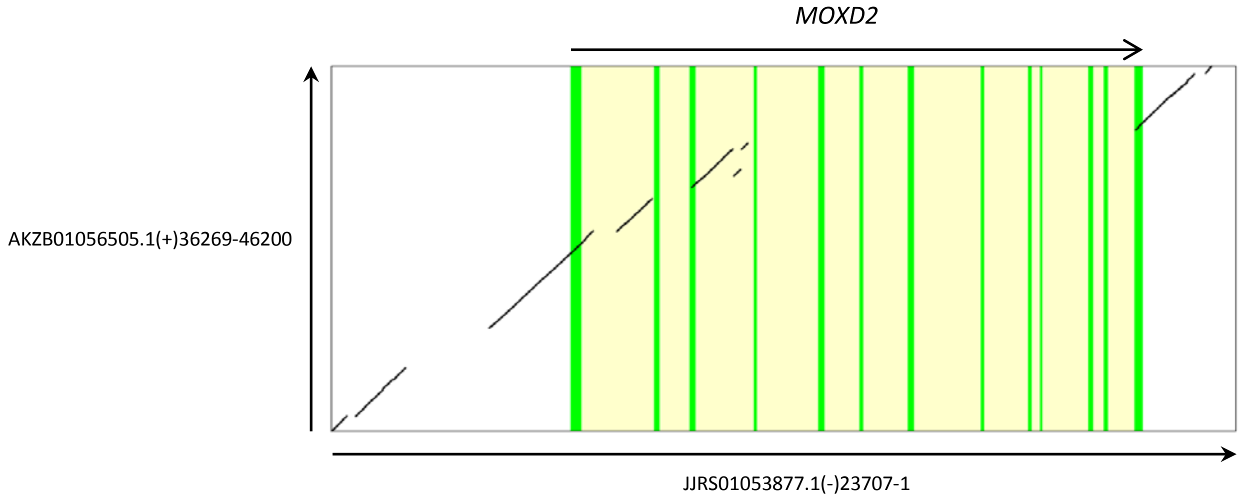

Order: Passeriformes
Family: Acanthisittidae
Scientific name: *Acanthisitta chloris*
Common name: Rifleman

No. 10
Order: Passeriformes
Family: Emberizidae
Scientific name: *Zonotrichia albicollis*
Common name: White-throated sparrow

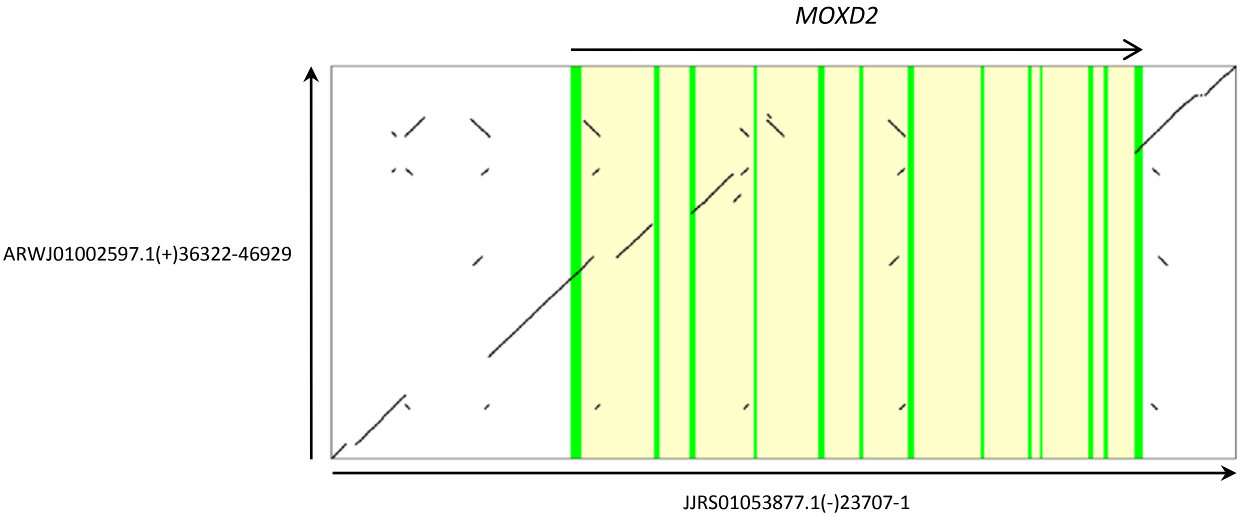

Order: Passeriformes
Family: Acanthisittidae
Scientific name: *Acanthisitta chloris*
Common name: Rifleman

No. 11
Order: Psittaciformes
Family: Psittacidae
Scientific name: *Nestor notabilis*
Common name: Kea

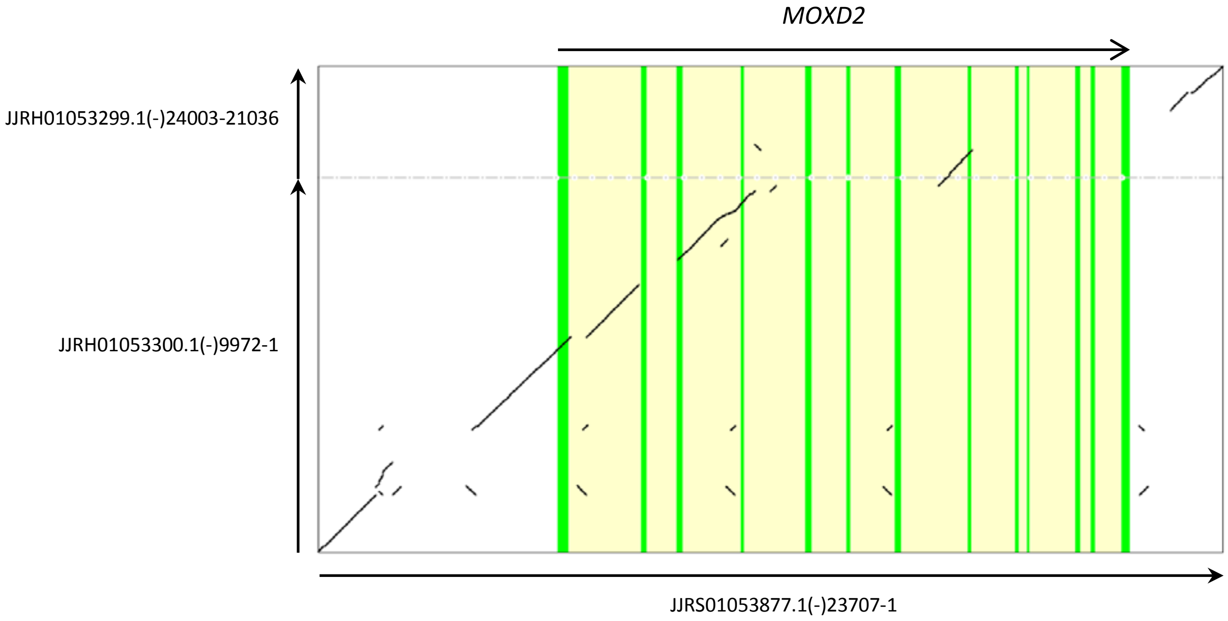

Order: Passeriformes
Family: Acanthisittidae
Scientific name: *Acanthisitta chloris*
Common name: Rifleman

No. 12
Order: Psittaciformes
Family: Psittacidae
Scientific name: *Melopsittacus undulatus*
Common name: Budgerigar

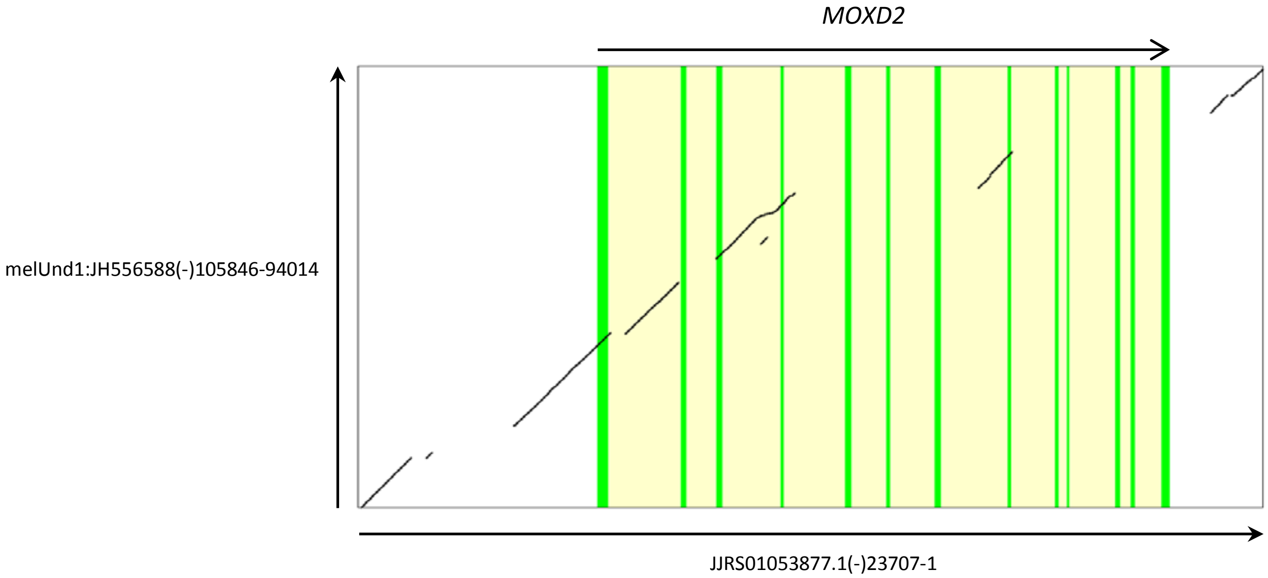

Order: Passeriformes
Family: Acanthisittidae
Scientific name: *Acanthisitta chloris*
Common name: Rifleman

No. 13
Order: Psittaciformes
Family: Psittacidae
Scientific name: *Amazona vittata*
Common name: Puerto Rican Amazon

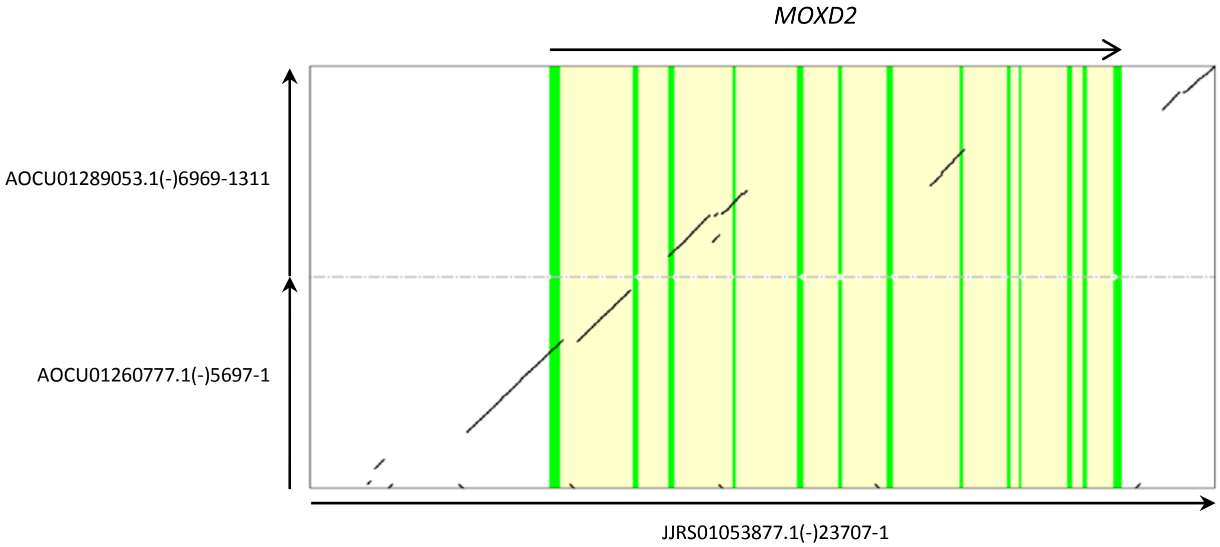

Order: Passeriformes
Family: Acanthisittidae
Scientific name: *Acanthisitta chloris*
Common name: Rifleman

No. 14
Order: Psittaciformes
Family: Psittacidae
Scientific name: *Ara macao*
Common name: Scarlet macaw

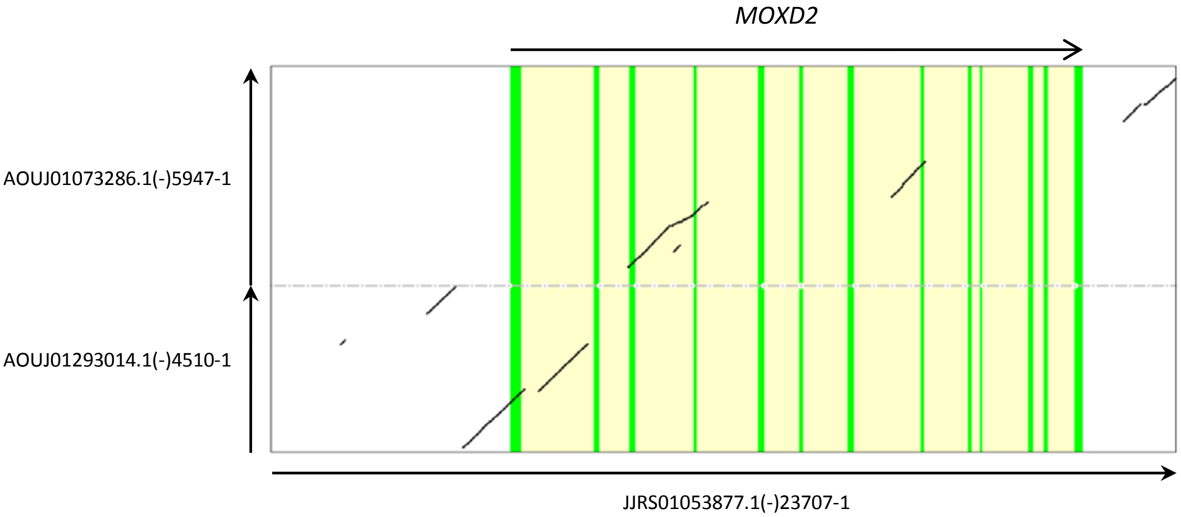

Order: Passeriformes
Family: Acanthisittidae
Scientific name: *Acanthisitta chloris*
Common name: Rifleman

No. 15
Order: Falconiformes
Family: Falconidae
Scientific name: *Falco cherrug*
Common name: Saker falcon

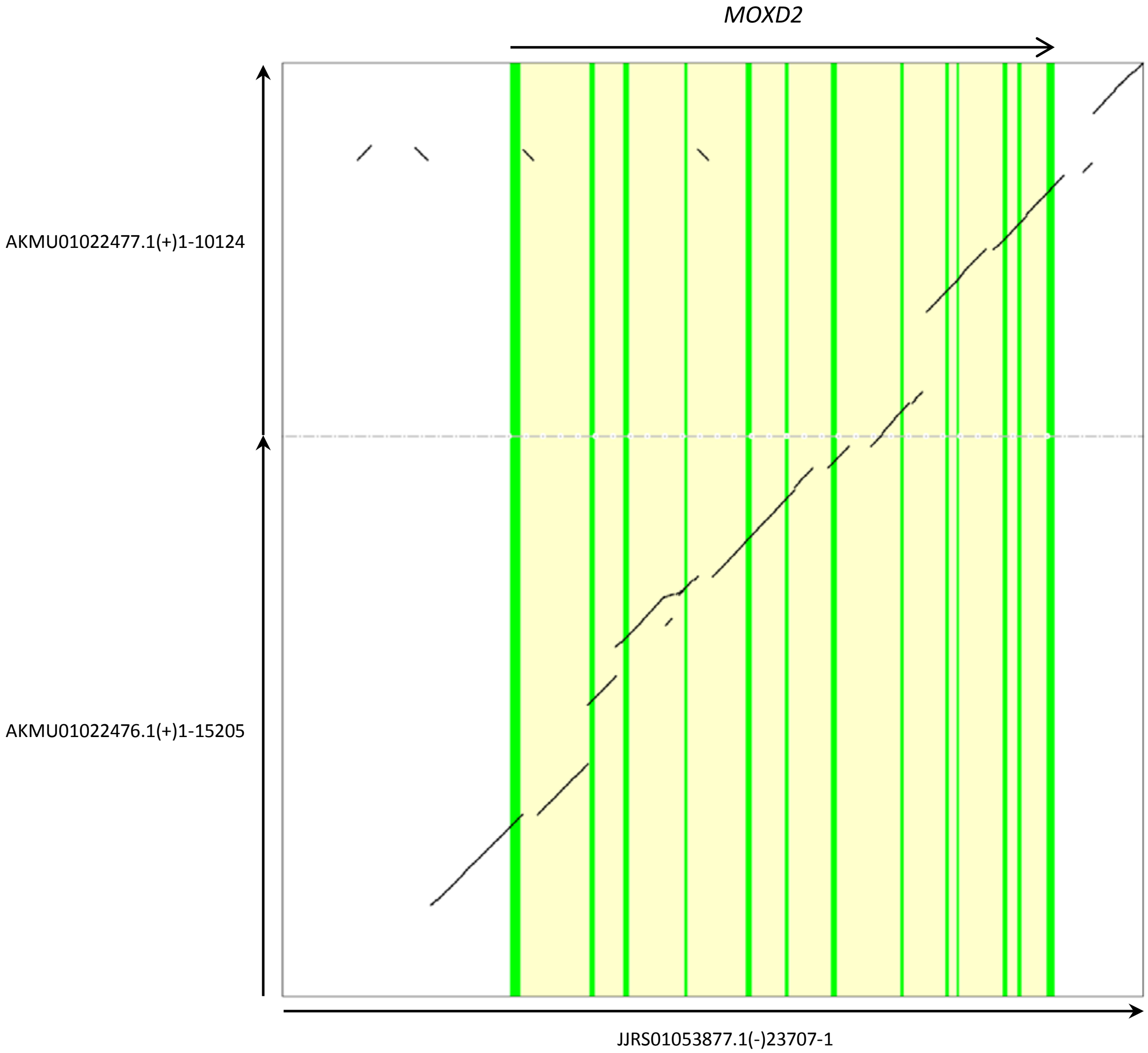

Order: Passeriformes
Family: Acanthisittidae
Scientific name: *Acanthisitta chloris*
Common name: Rifleman

No. 16
Order: Falconiformes
Family: Falconidae
Scientific name: *Falco peregrinus*
Common name: Peregrine falcon

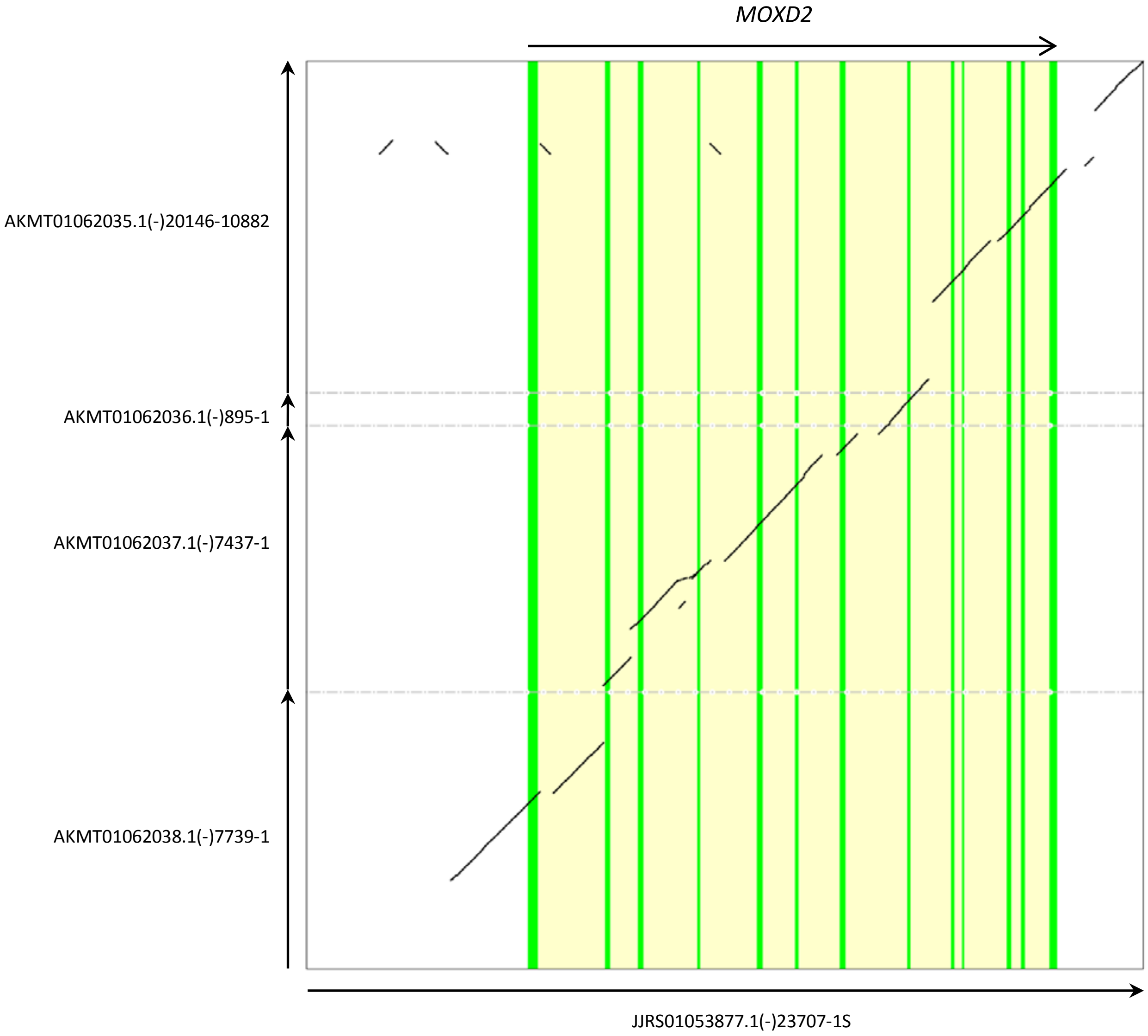

Order: Passeriformes
Family: Acanthisittidae
Scientific name: *Acanthisitta chloris*
Common name: Rifleman

No. 17
Order: Cariamiformes
Family: Cariamidae
Scientific name: *Cariama cristata*
Common name: Red-legged seriema

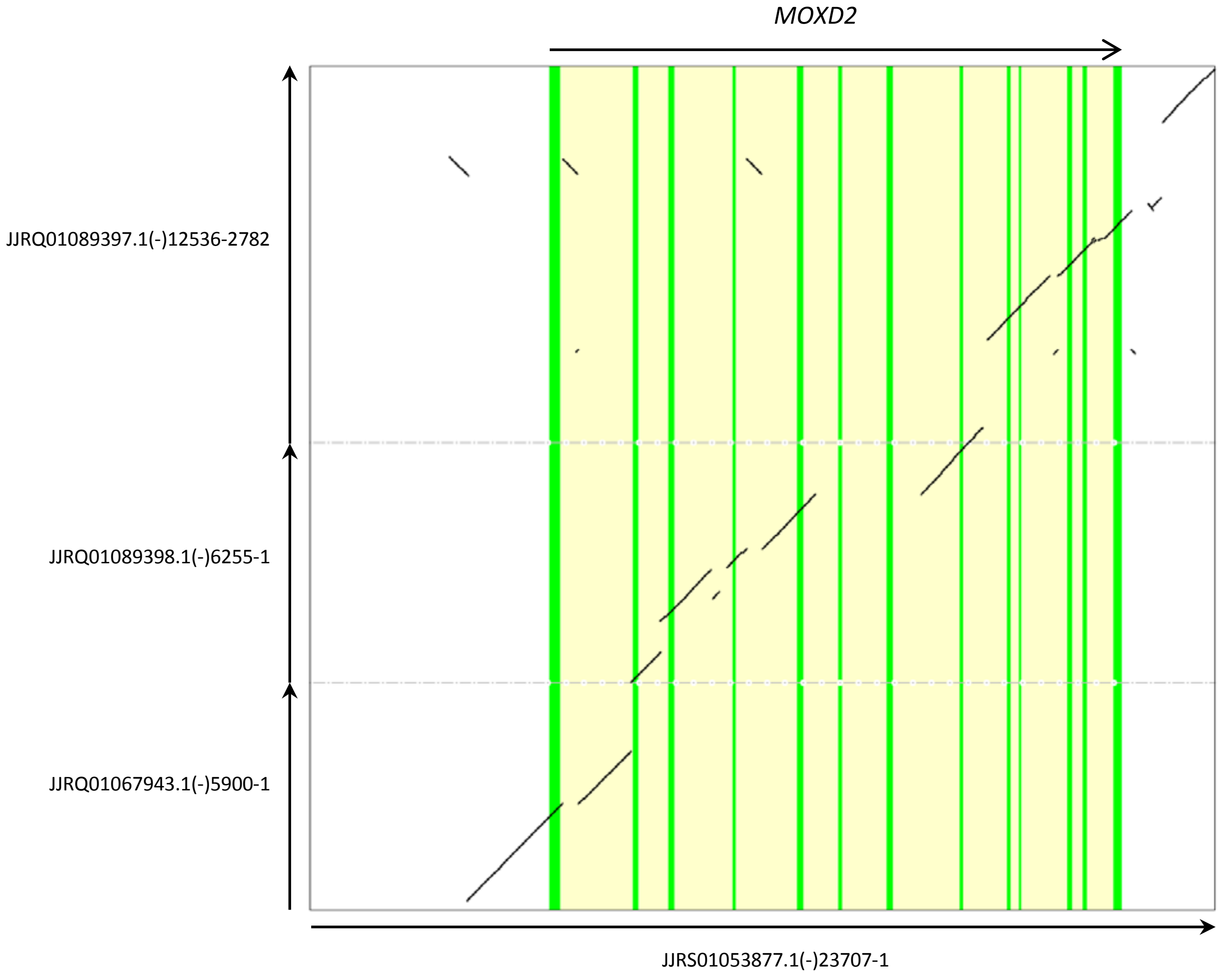

Order: Passeriformes
Family: Acanthisittidae
Scientific name: *Acanthisitta chloris*
Common name: Rifleman

No. 18
Order: Piciformes
Family: Picidae
Scientific name: *Picoides pubescens*
Common name: Downy woodpecker

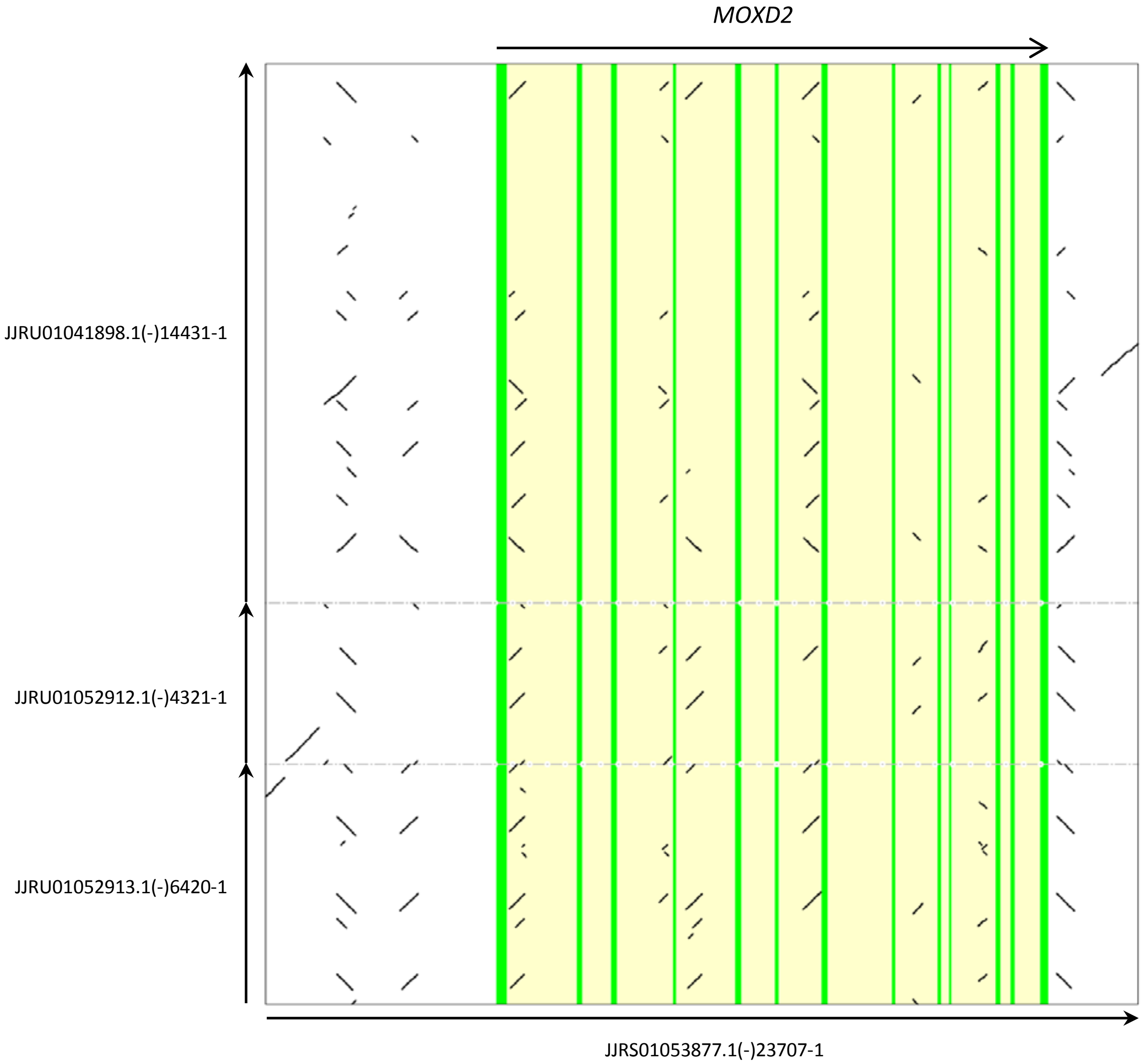

Order: Passeriformes
Family: Acanthisittidae
Scientific name: *Acanthisitta chloris*
Common name: Rifleman

No. 19
Order: Coraciiformes
Family: Meropidae
Scientific name: *Merops nubicus*
Common name: Northern carmine bee-eater

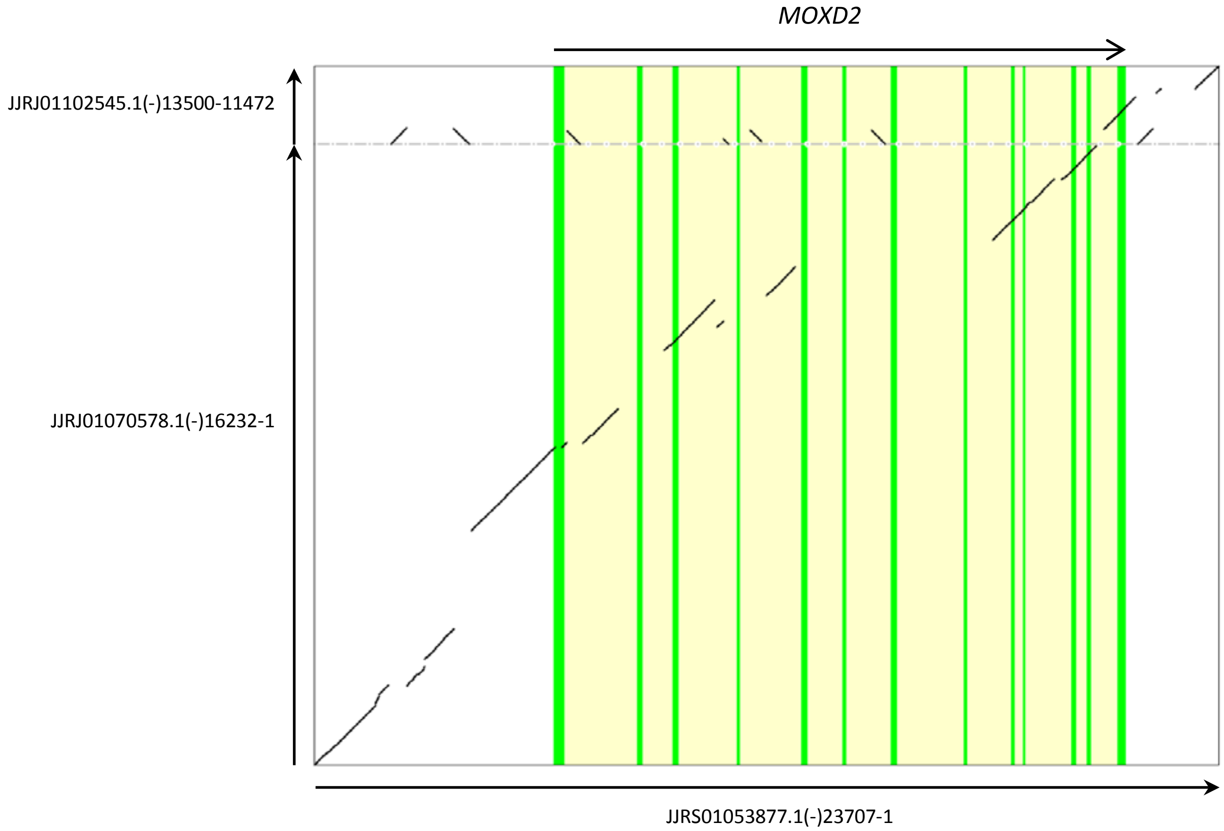

Order: Passeriformes
Family: Acanthisittidae
Scientific name: *Acanthisitta chloris*
Common name: Rifleman

No. 20
Order: Bucerotiformes
Family: Bucerotidae
Scientific name: *Buceros rhinoceros silvestris*
Common name: Rhinoceros hornbill

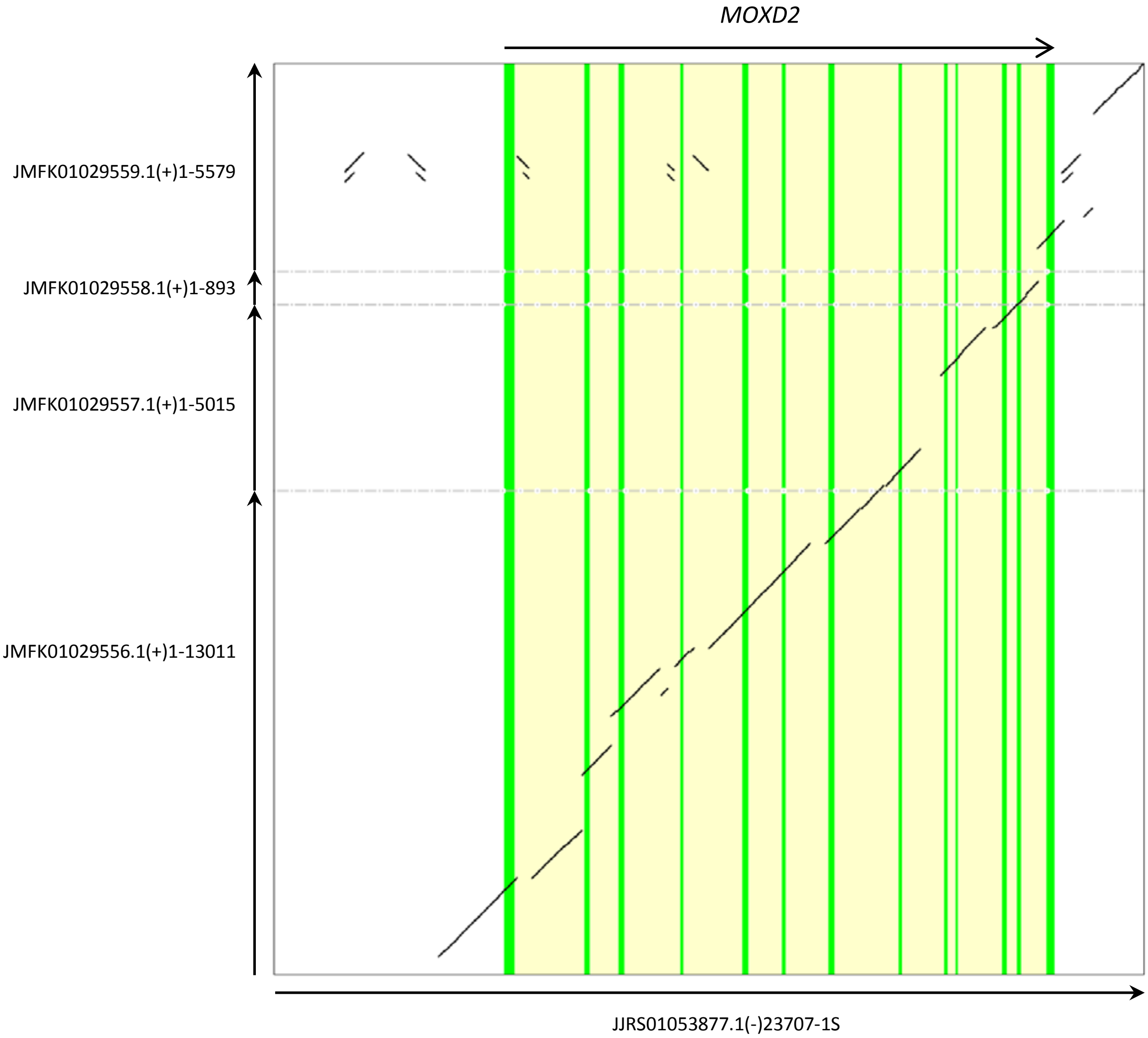

Order: Passeriformes
Family: Acanthisittidae
Scientific name: *Acanthisitta chloris*
Common name: Rifleman

No. 21
Order: Trogoniformes
Family: Trogonidae
Scientific name: *Apaloderma vittatum*
Common name: Bar-tailed trogon

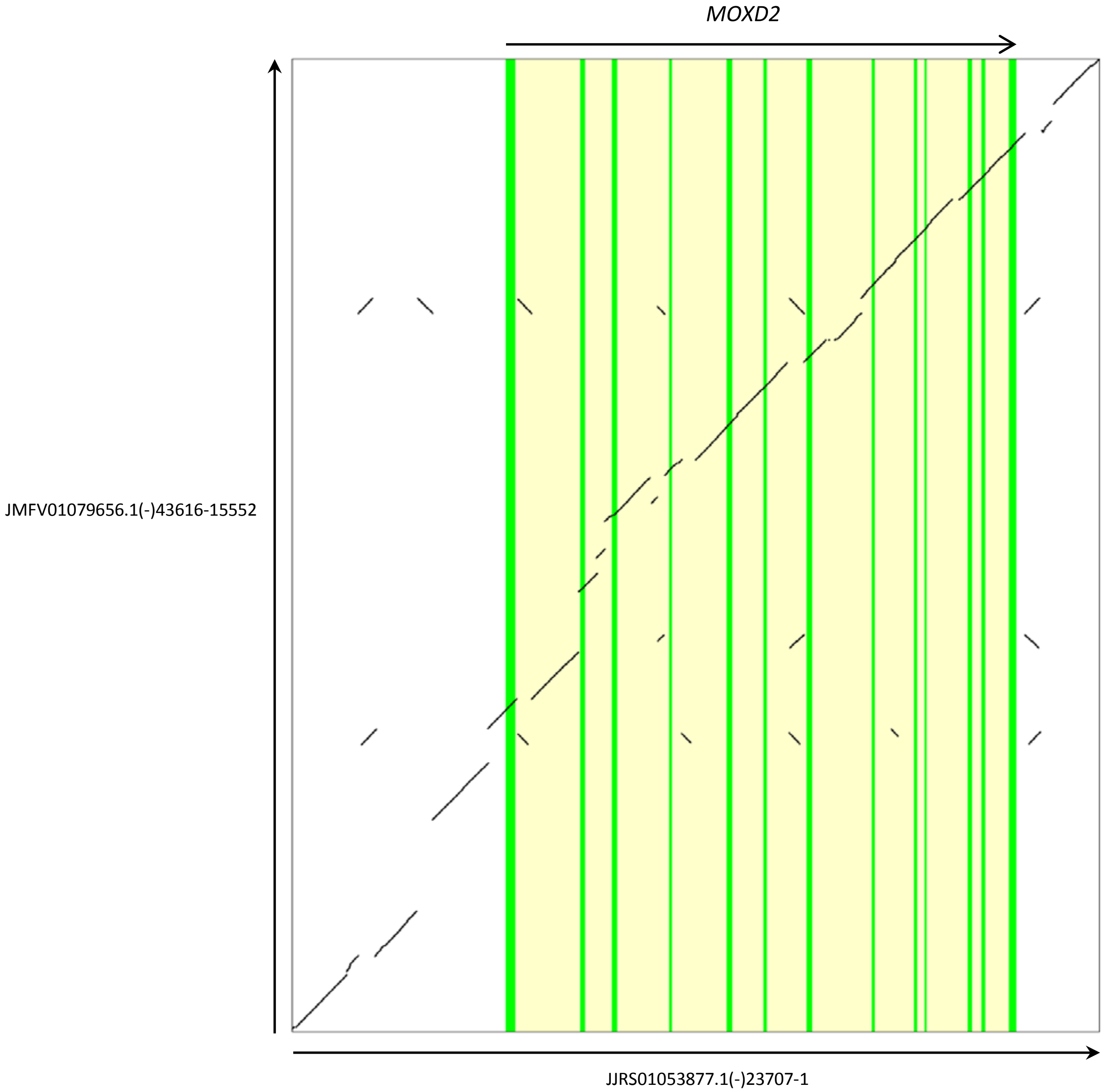

Order: Passeriformes
Family: Acanthisittidae
Scientific name: *Acanthisitta chloris*
Common name: Rifleman

No. 22
Order: Leptosomiformes
Family: Leptosomidae
Scientific name: *Leptosomus discolor*
Common name: Cuckoo roller

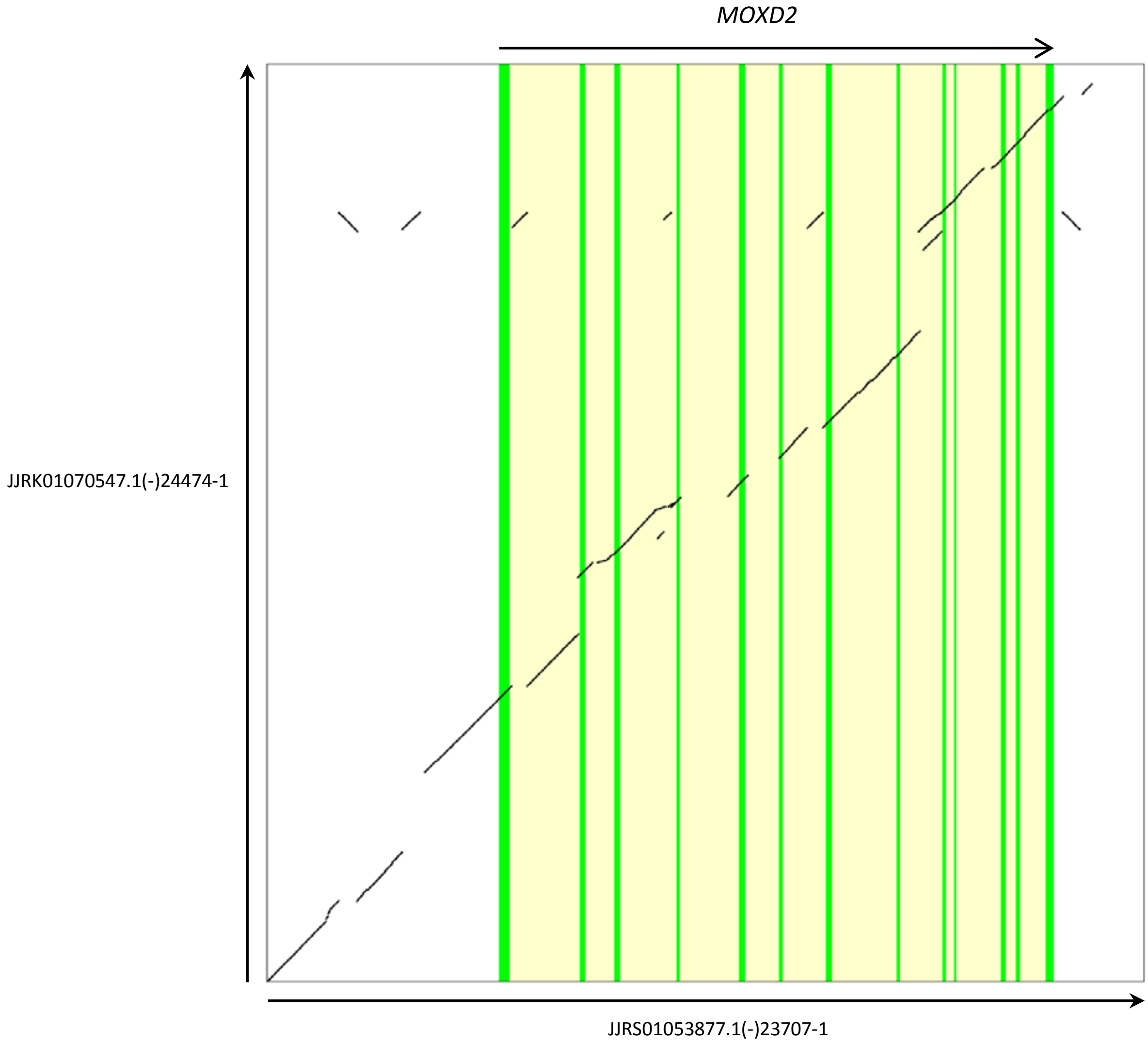

Order: Passeriformes
Family: Acanthisittidae
Scientific name: *Acanthisitta chloris*
Common name: Rifleman

No. 23
Order: Coliiformes
Family: Coliidae
Scientific name: *Colius striatus*
Common name: Speckled mousebird

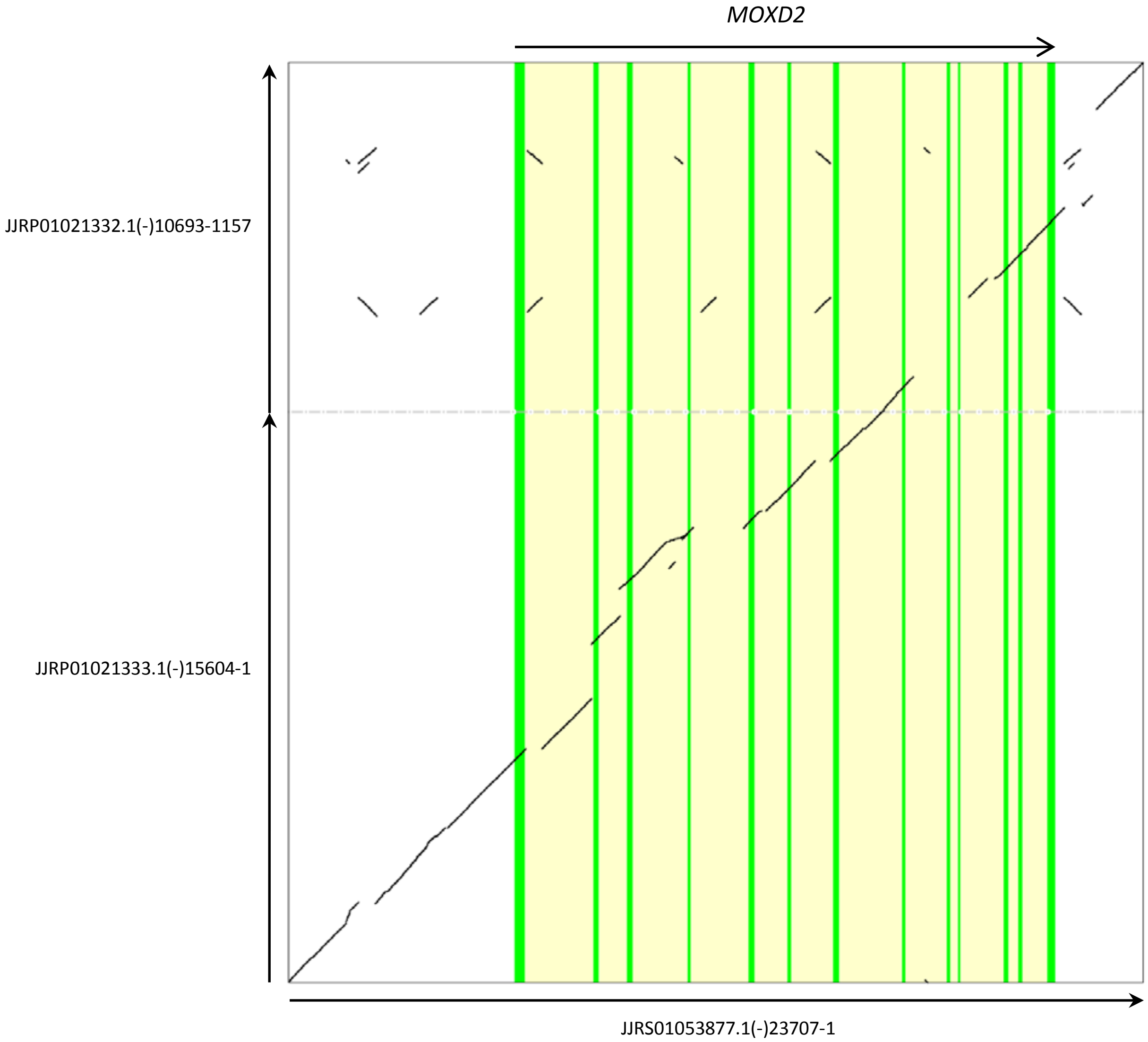

Order: Passeriformes
Family: Acanthisittidae
Scientific name: *Acanthisitta chloris*
Common name: Rifleman

No. 24
Order: Strigiformes
Family: Tytonidae
Scientific name: *Tyto alba*
Common name: Barn owl

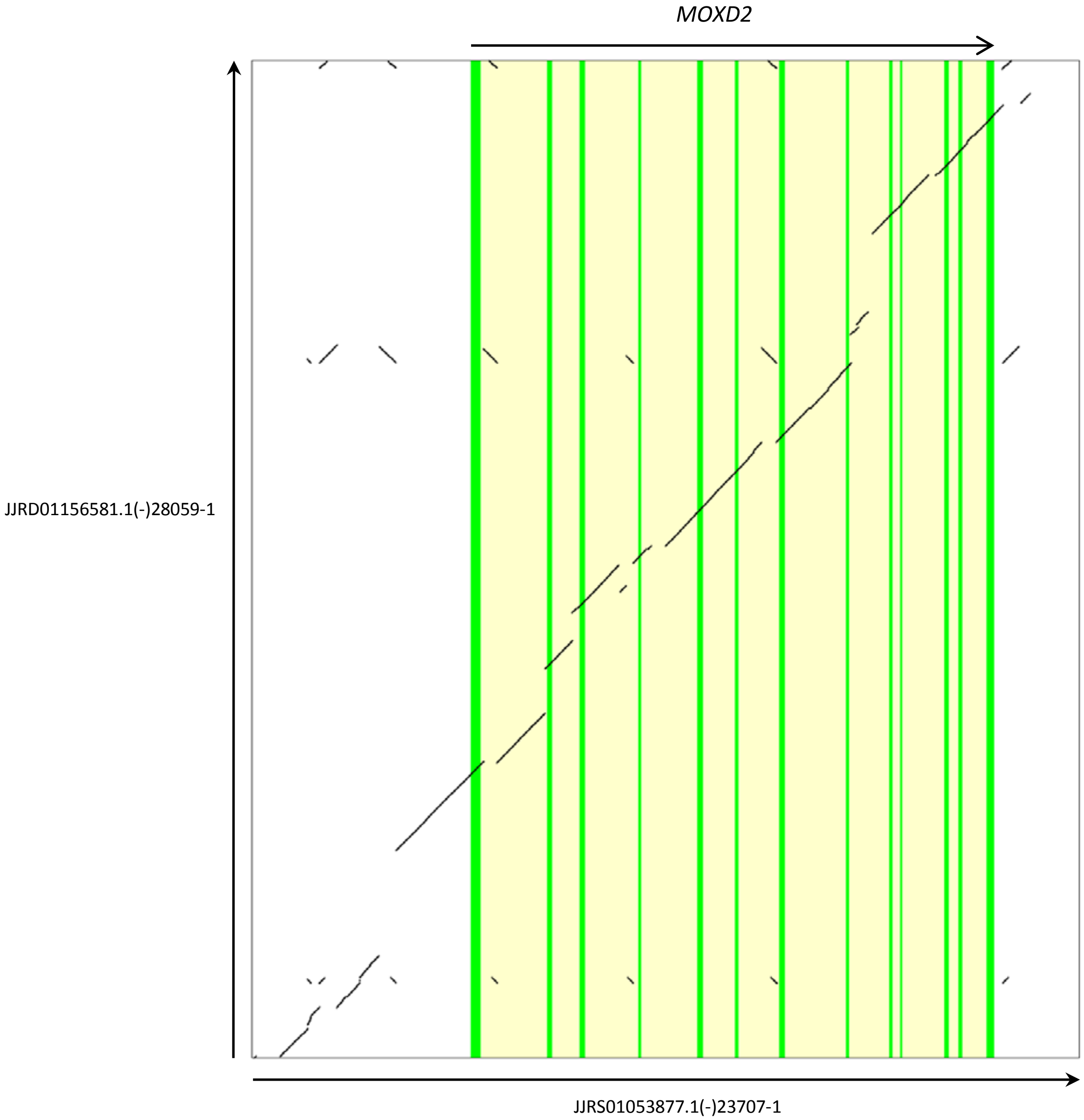

Order: Passeriformes
Family: Acanthisittidae
Scientific name: *Acanthisitta chloris*
Common name: Rifleman

No. 25
Order: Accipitriformes
Family: Cathartidae
Scientific name: *Cathartes aura*
Common name: Turkey vulture

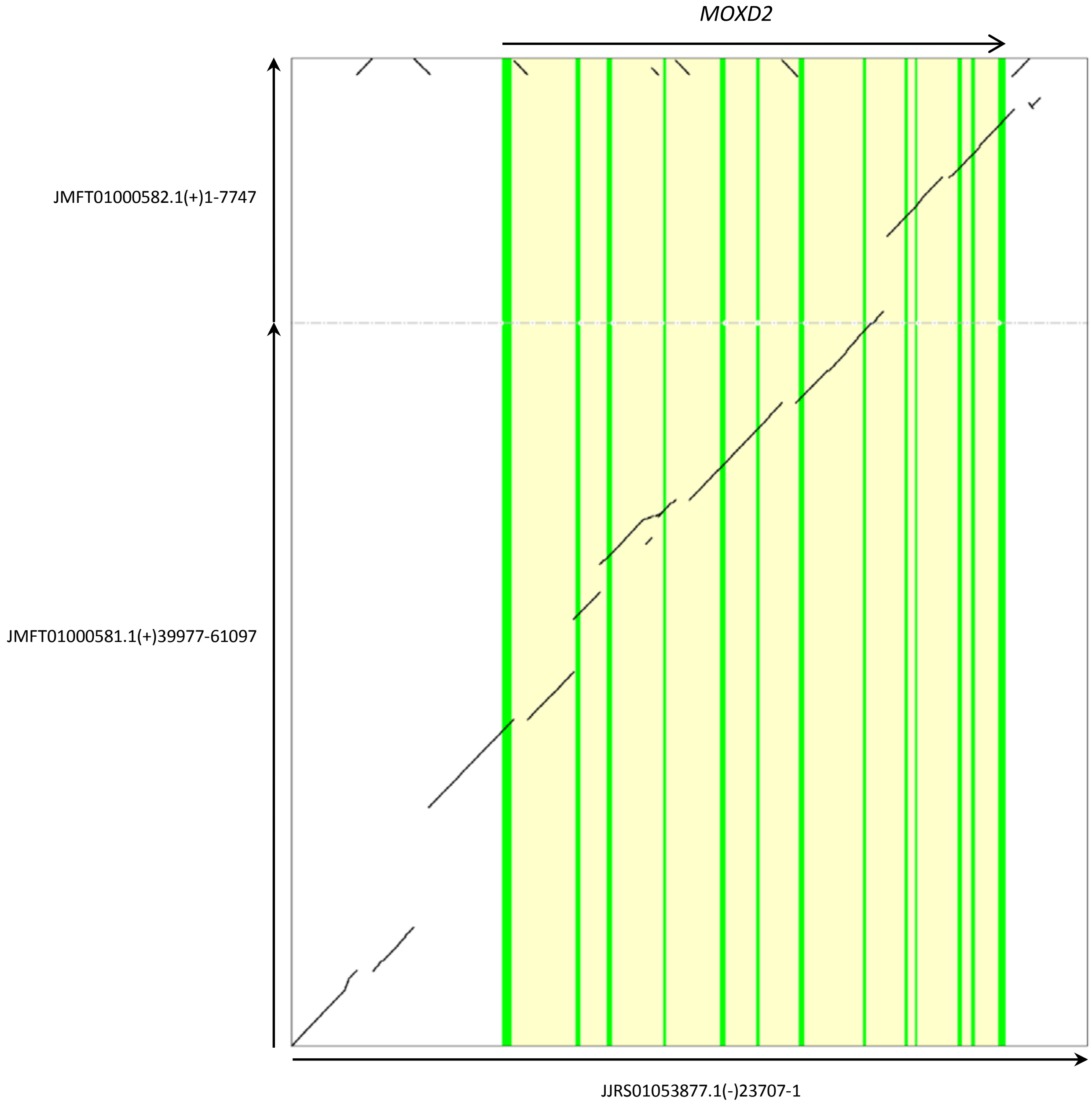

Order: Passeriformes
Family: Acanthisittidae
Scientific name: *Acanthisitta chloris*
Common name: Rifleman

No. 26
Order: Accipitriformes
Family: Accipitridae
Scientific name: *Haliaeetus albicilla*
Common name: White-tailed eagle

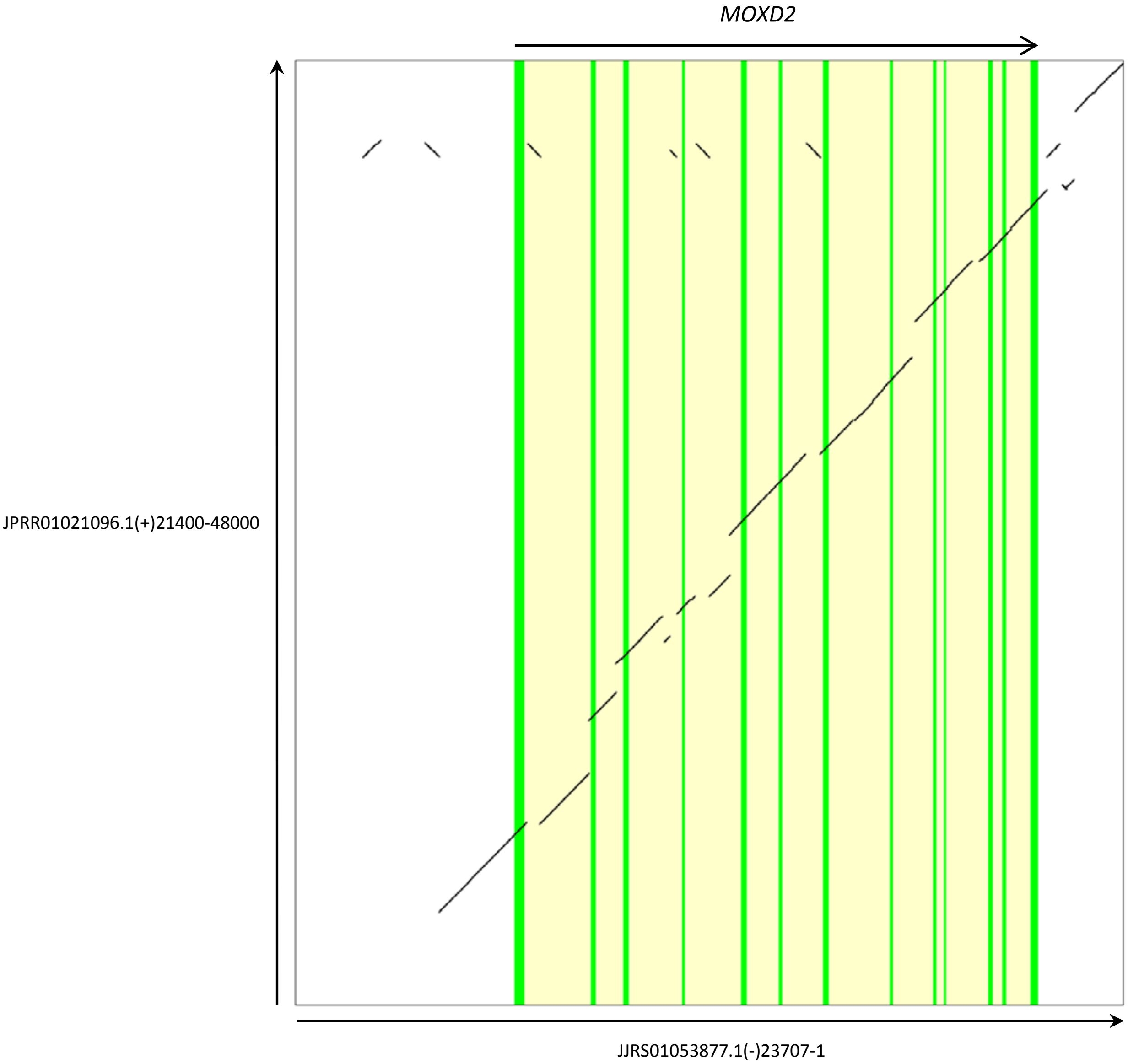

Order: Passeriformes
Family: Acanthisittidae
Scientific name: *Acanthisitta chloris*
Common name: Rifleman

No. 27
Order: Accipitriformes
Family: Accipitridae
Scientific name: *Aquila chrysaetos canadensis*
Common name: Golden eagle

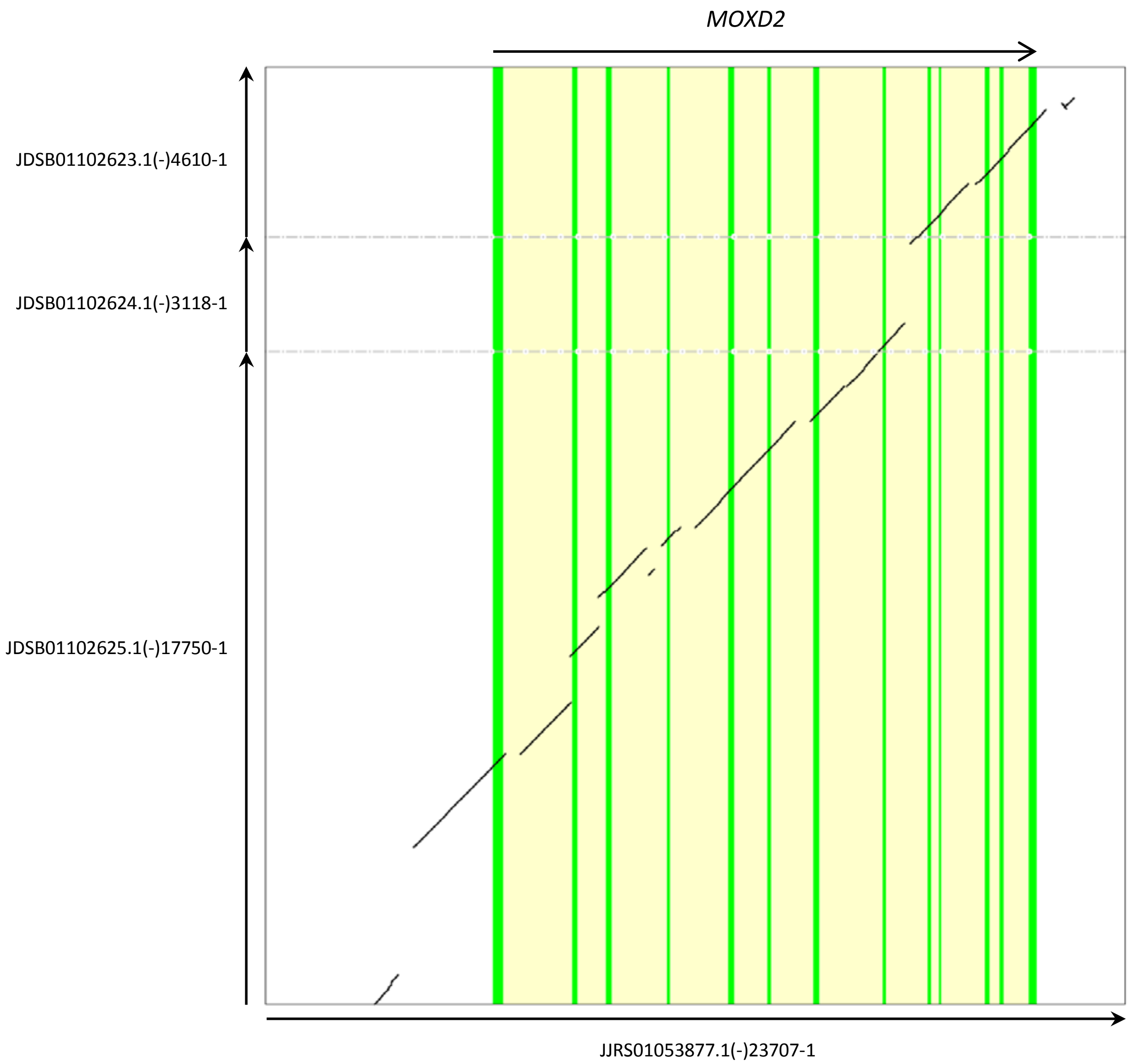

Order: Passeriformes
Family: Acanthisittidae
Scientific name: *Acanthisitta chloris*
Common name: Rifleman

No. 28
Order: Phaethontiformes
Family: Phaethontidae
Scientific name: *Phaethon lepturus*
Common name: White-tailed tropicbird

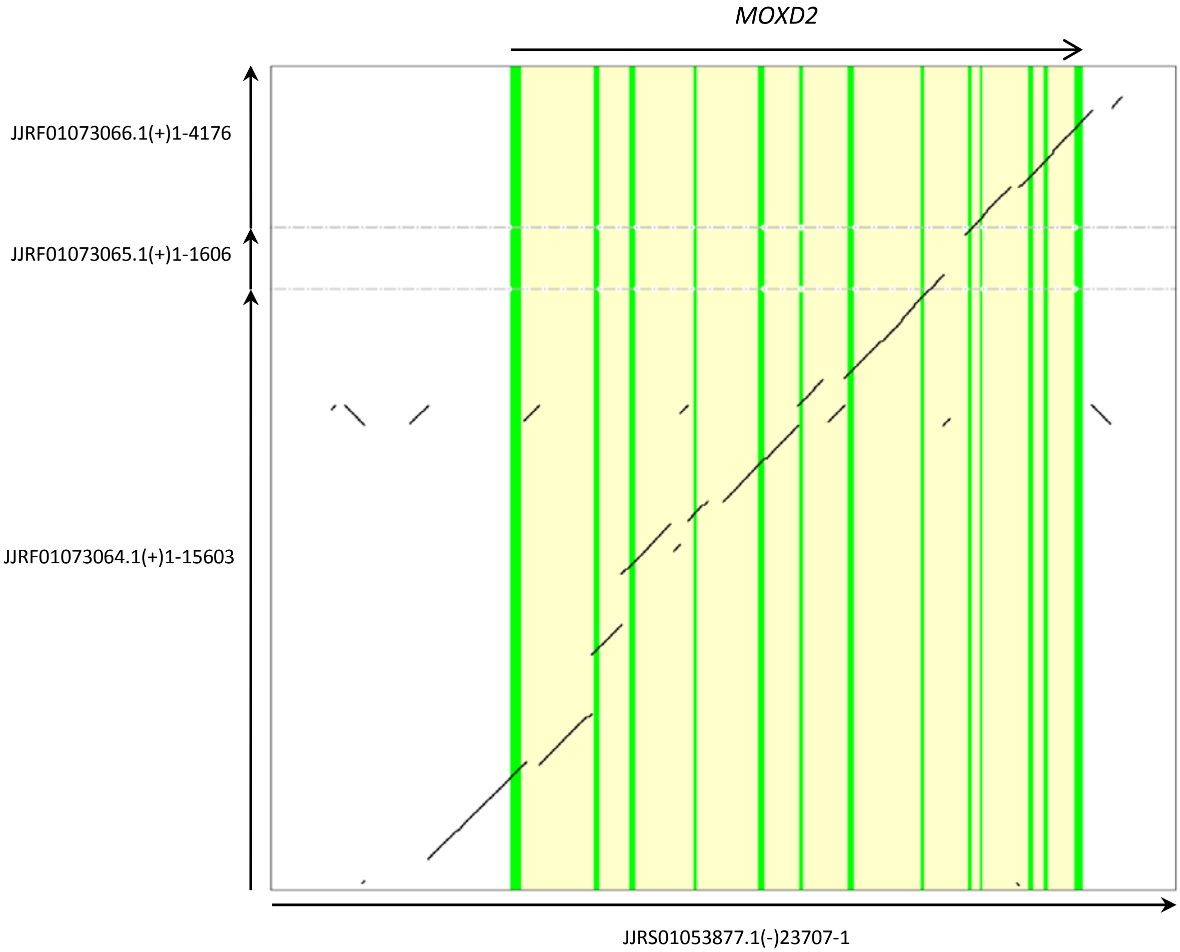

Order: Passeriformes
Family: Acanthisittidae
Scientific name: *Acanthisitta chloris*
Common name: Rifleman

No. 29
Order: Eurypygiformes
Family: Eurypygidae
Scientific name: *Eurypyga helias*
Common name: Sunbittern

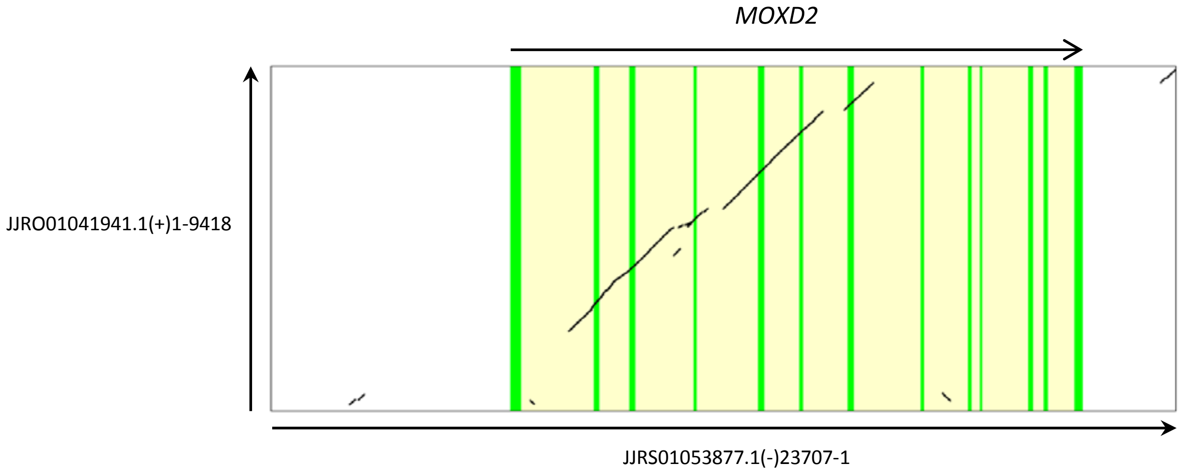

Order: Passeriformes
Family: Acanthisittidae
Scientific name: *Acanthisitta chloris*
Common name: Rifleman

No. 30
Order: Gaviiformes
Family: Gaviidae
Scientific name: *Gavia stellata*
Common name: Red-throated loon

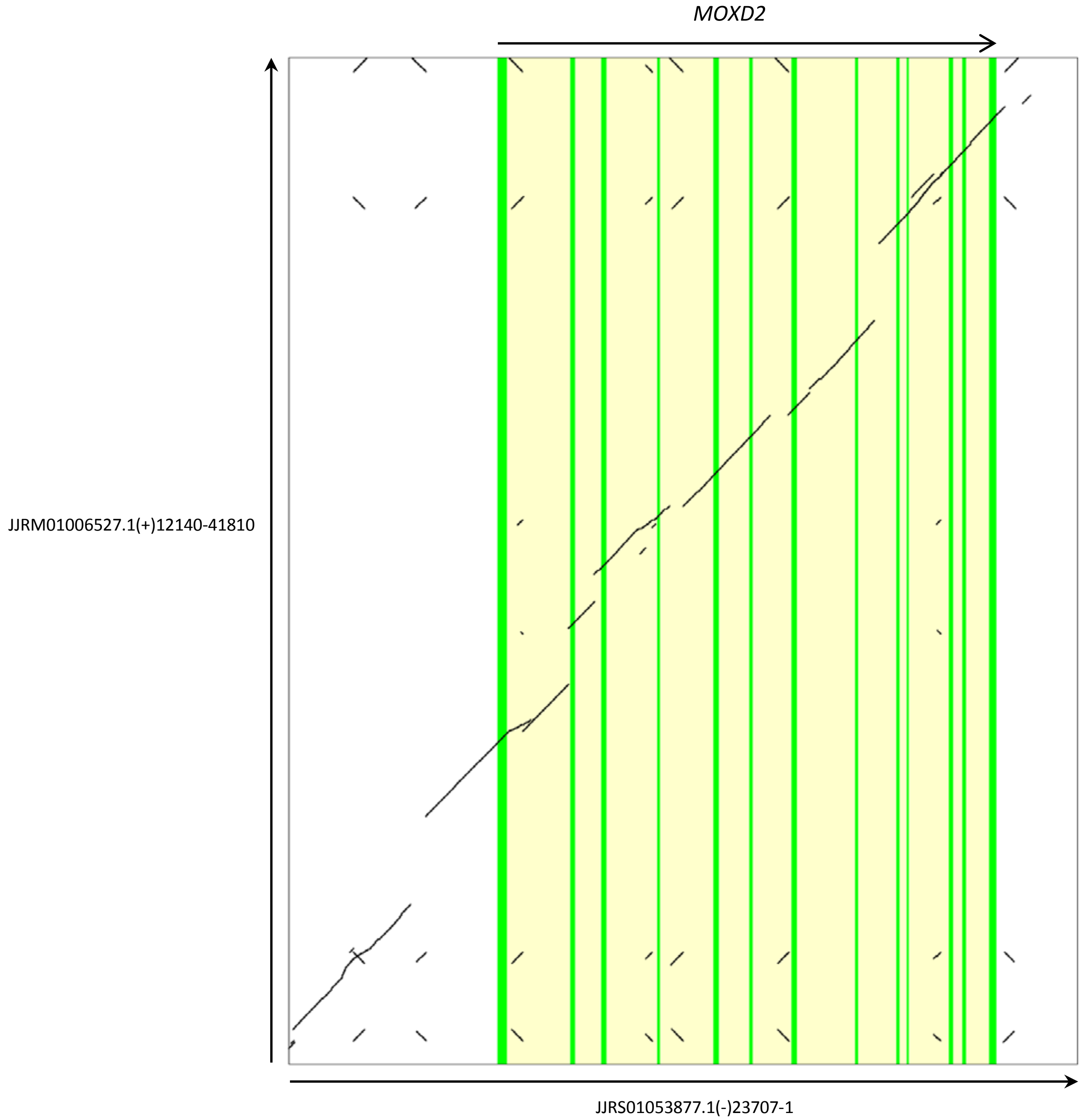

Order: Passeriformes
Family: Acanthisittidae
Scientific name: *Acanthisitta chloris*
Common name: Rifleman

No. 31
Order: Sphenisciformes
Family: Spheniscidae
Scientific name: *Aptenodytes forsteri*
Common name: Emperor penguin

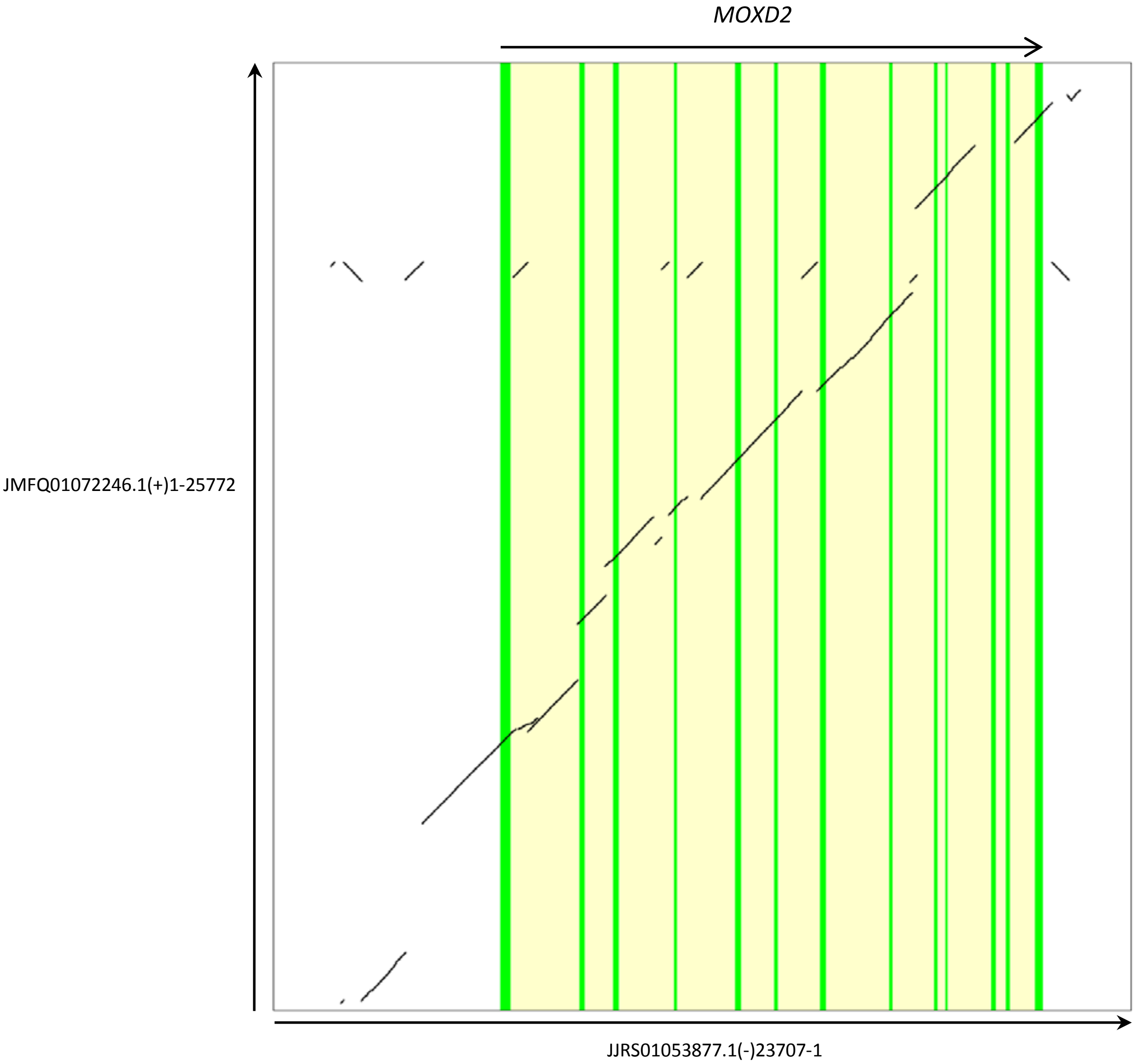

Order: Passeriformes
Family: Acanthisittidae
Scientific name: *Acanthisitta chloris*
Common name: Rifleman

No. 32
Order: Sphenisciformes
Family: Spheniscidae
Scientific name: *Pygoscelis adeliae*
Common name: Adelie Penguin

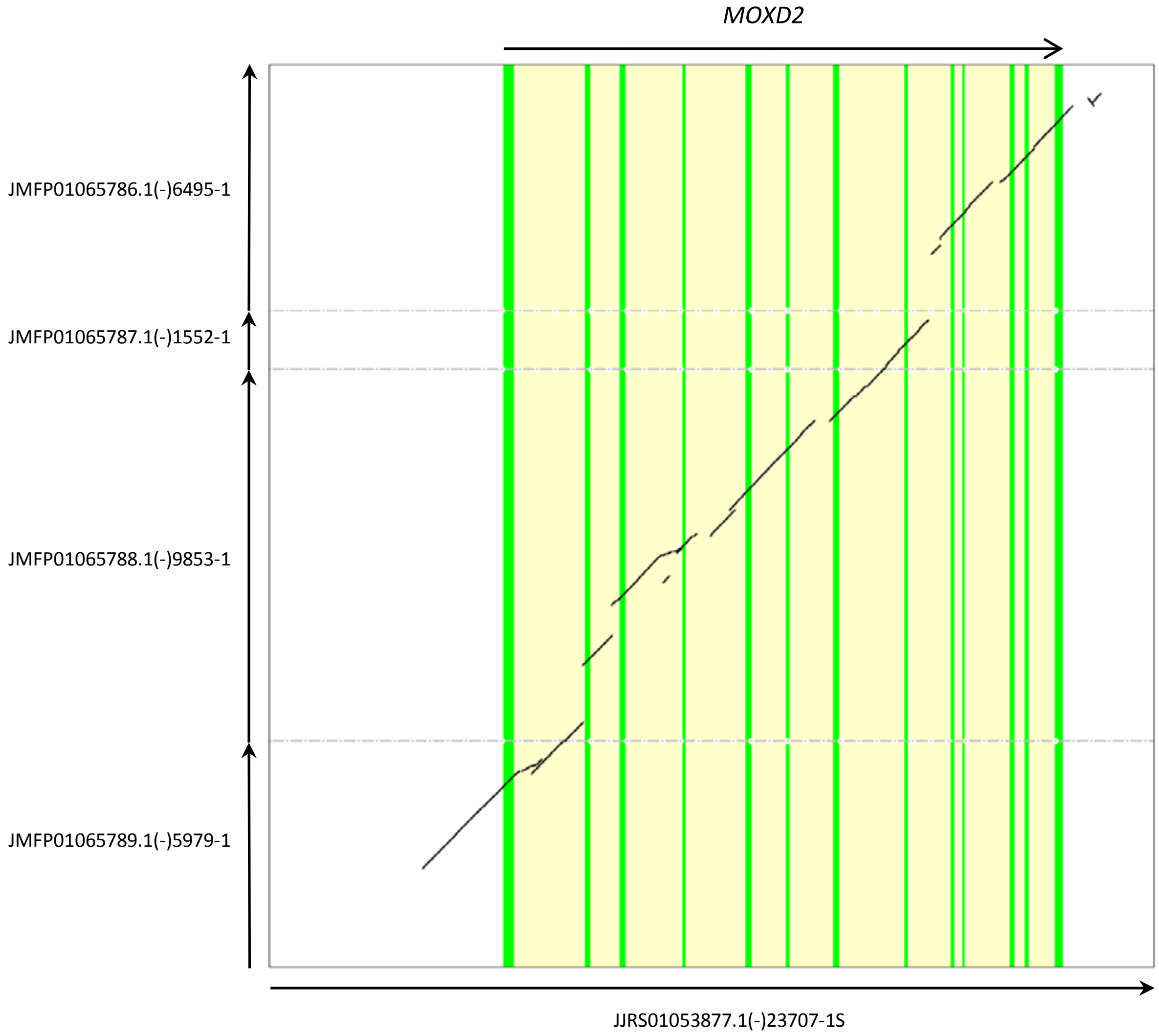

Order: Passeriformes
Family: Acanthisittidae
Scientific name: *Acanthisitta chloris*
Common name: Rifleman

No. 33
Order: Procellariiformes
Family: Procellariidae
Scientific name: *Fulmarus glacialis*
Common name: Northern fulmar

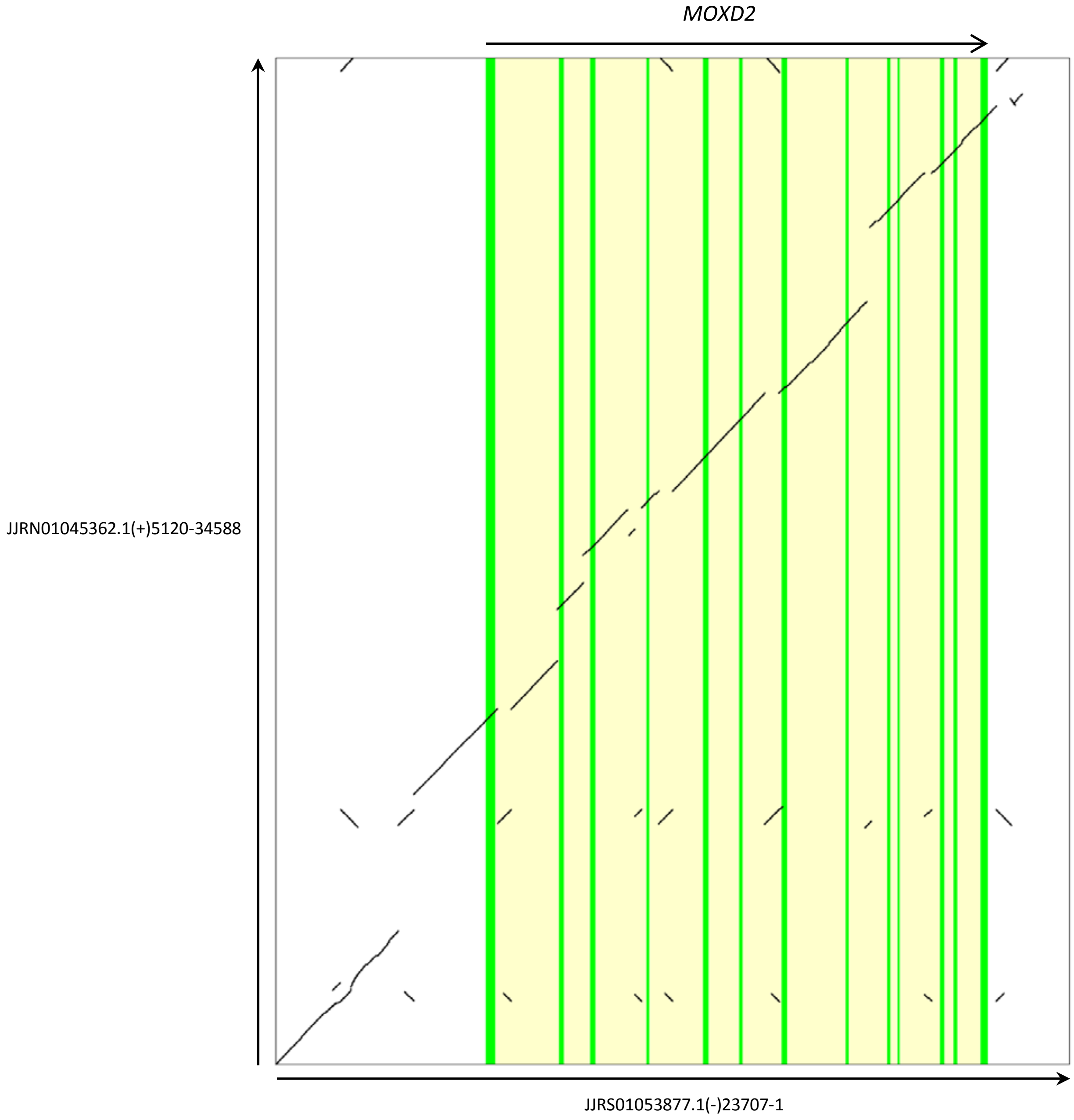

Order: Passeriformes
Family: Acanthisittidae
Scientific name: *Acanthisitta chloris*
Common name: Rifleman

No. 34
Order: Suliformes
Family: Phalacrocoracidae
Scientific name: *Phalacrocorax carbo*
Common name: Great cormorant

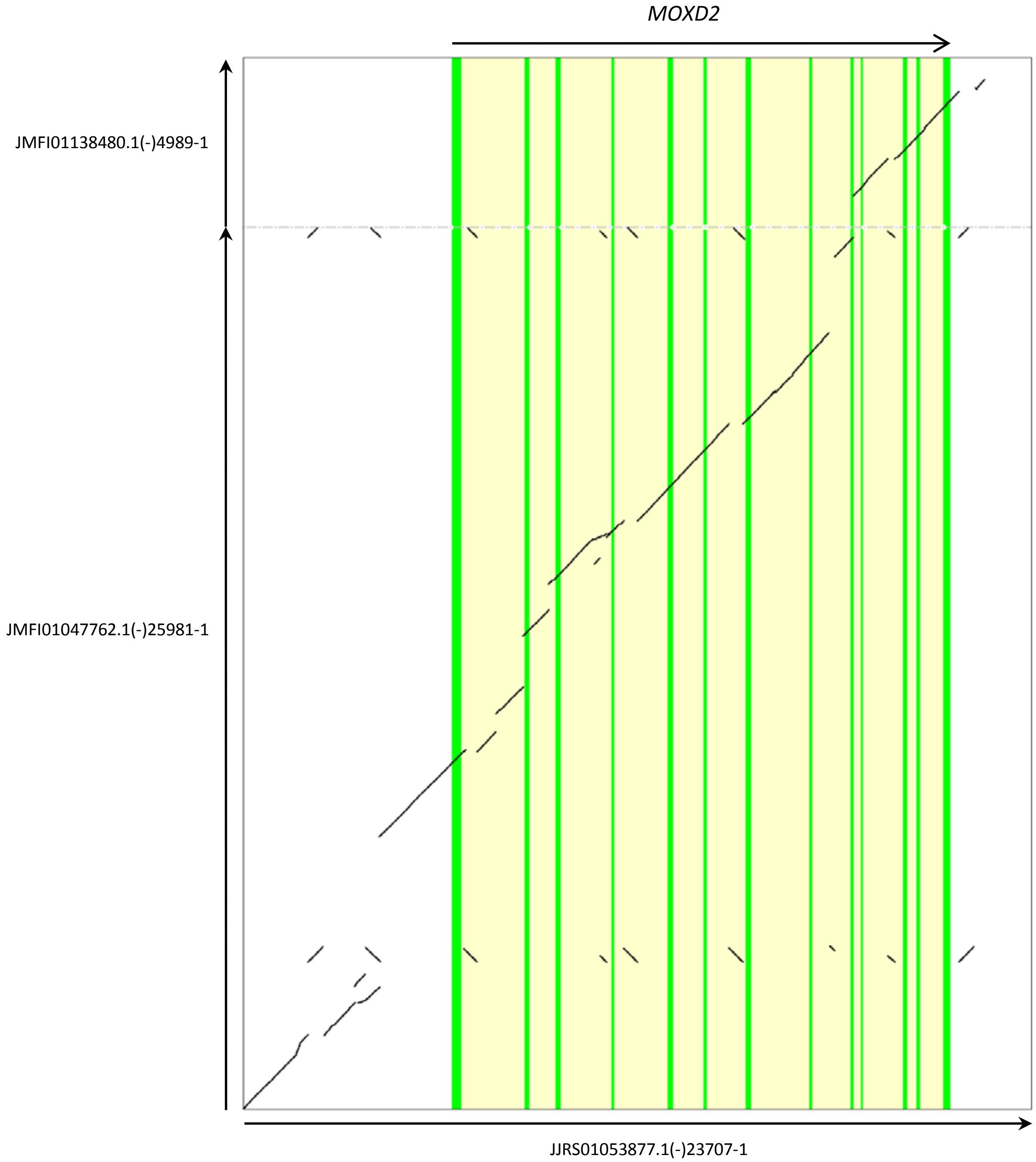

Order: Passeriformes
Family: Acanthisittidae
Scientific name: *Acanthisitta chloris*
Common name: Rifleman

No. 35
Order: Pelecaniformes
Family: Threskiornithidae
Scientific name: *Nipponia nippon*
Common name: Crested ibis

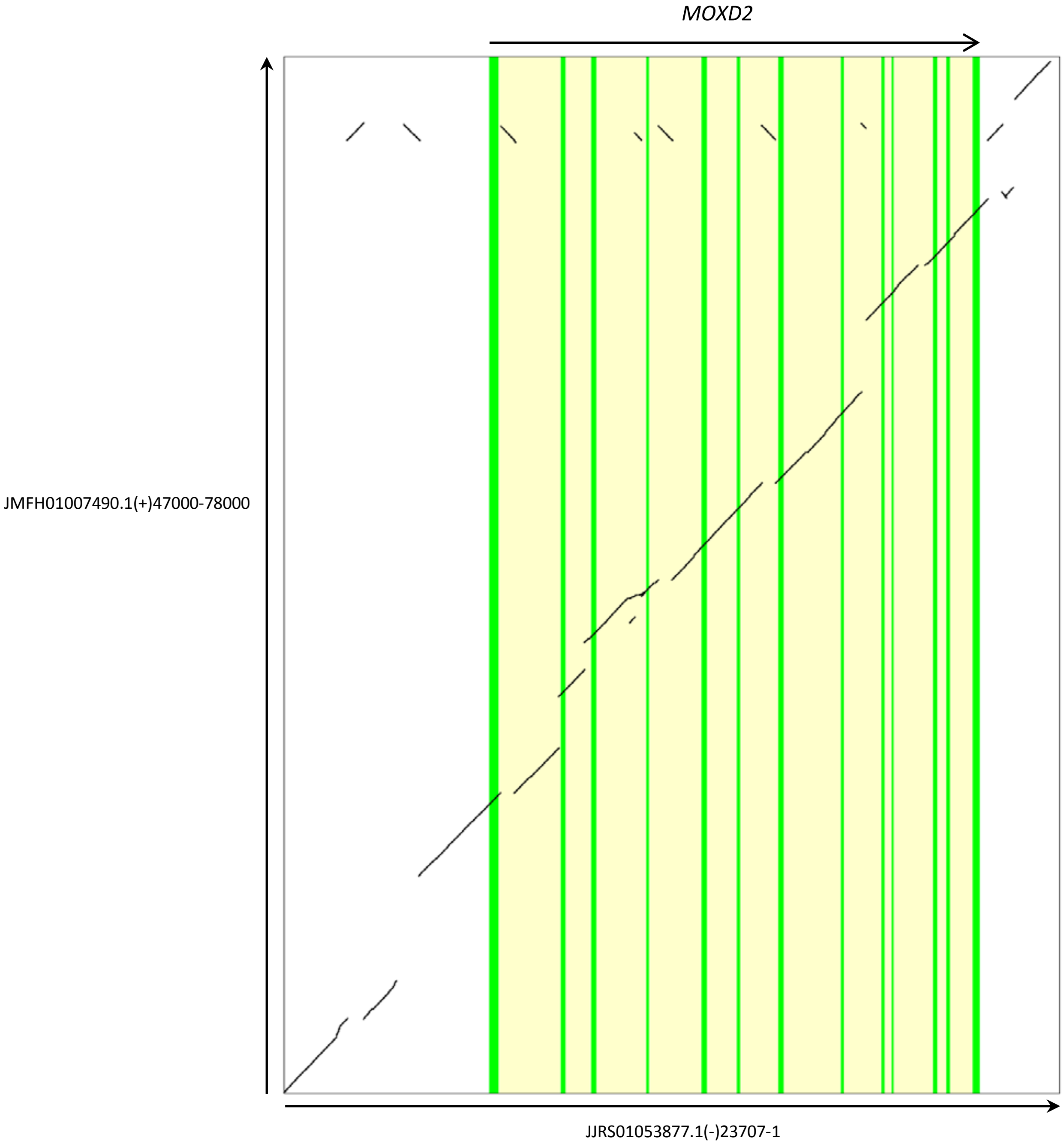

Order: Passeriformes
Family: Acanthisittidae
Scientific name: *Acanthisitta chloris*
Common name: Rifleman

No. 36
Order: Pelecaniformes
Family: Ardeidae
Scientific name: *Egretta garzetta*
Common name: Little egret

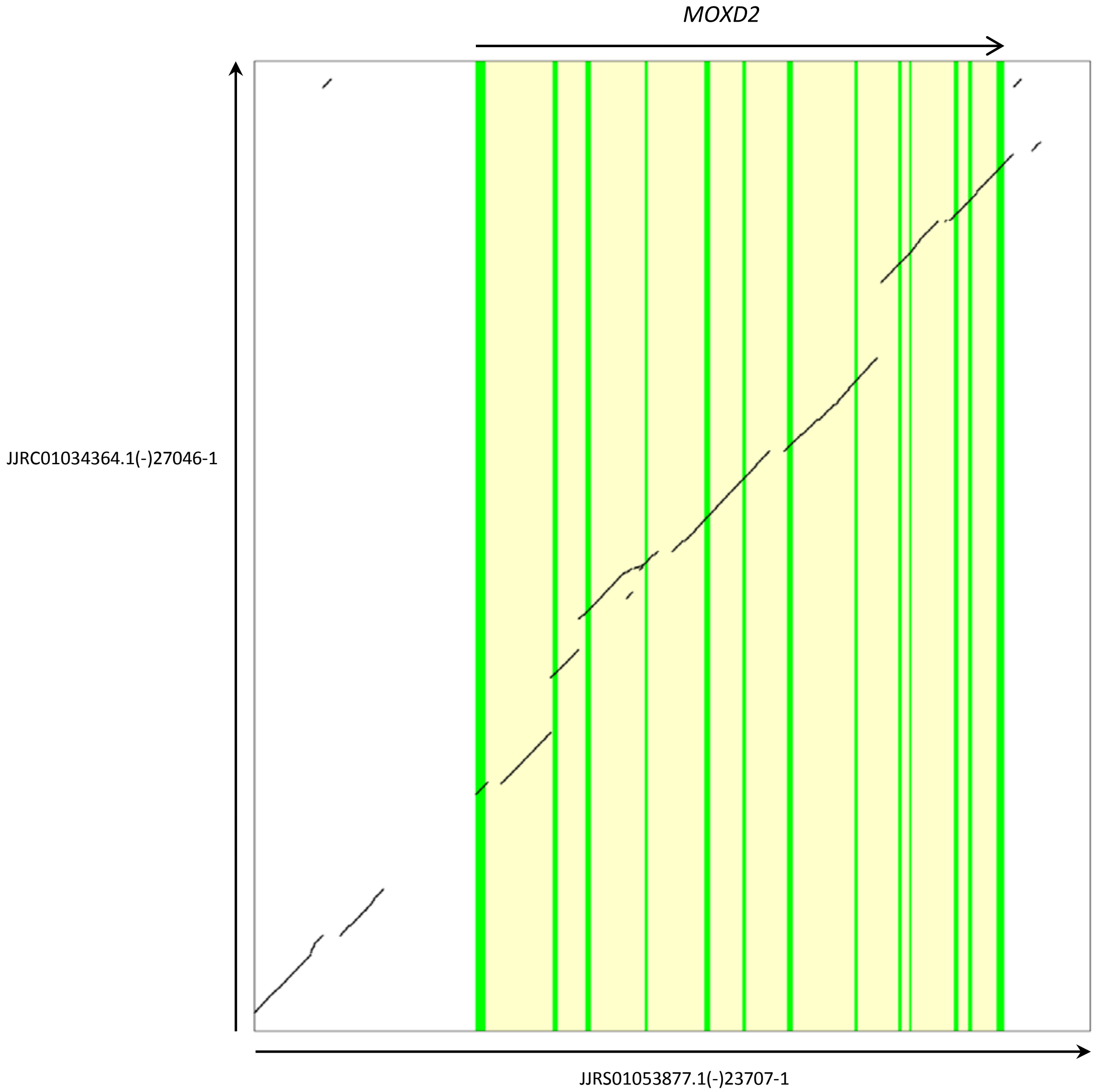

Order: Passeriformes
Family: Acanthisittidae
Scientific name: *Acanthisitta chloris*
Common name: Rifleman

No. 37
Order: Charadriiformes
Family: Charadriidae
Scientific name: *Charadrius vociferus*
Common name: Killdeer

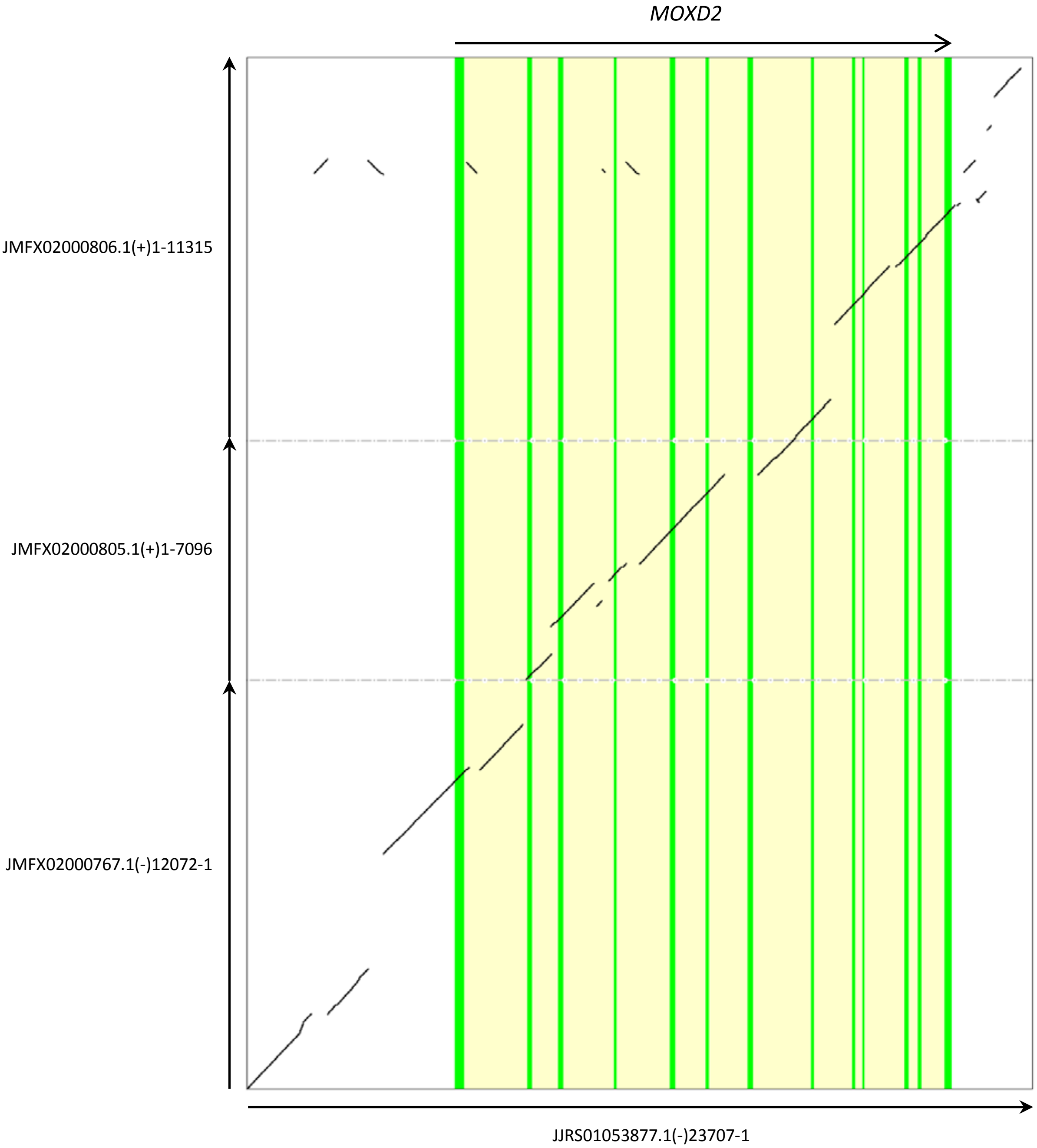

Order: Passeriformes
Family: Acanthisittidae
Scientific name: *Acanthisitta chloris*
Common name: Rifleman

No. 38
Order: Gruiformes
Family: Gruidae
Scientific name: *Balearica pavonina gibbericeps*
Common name: East African crowned crane

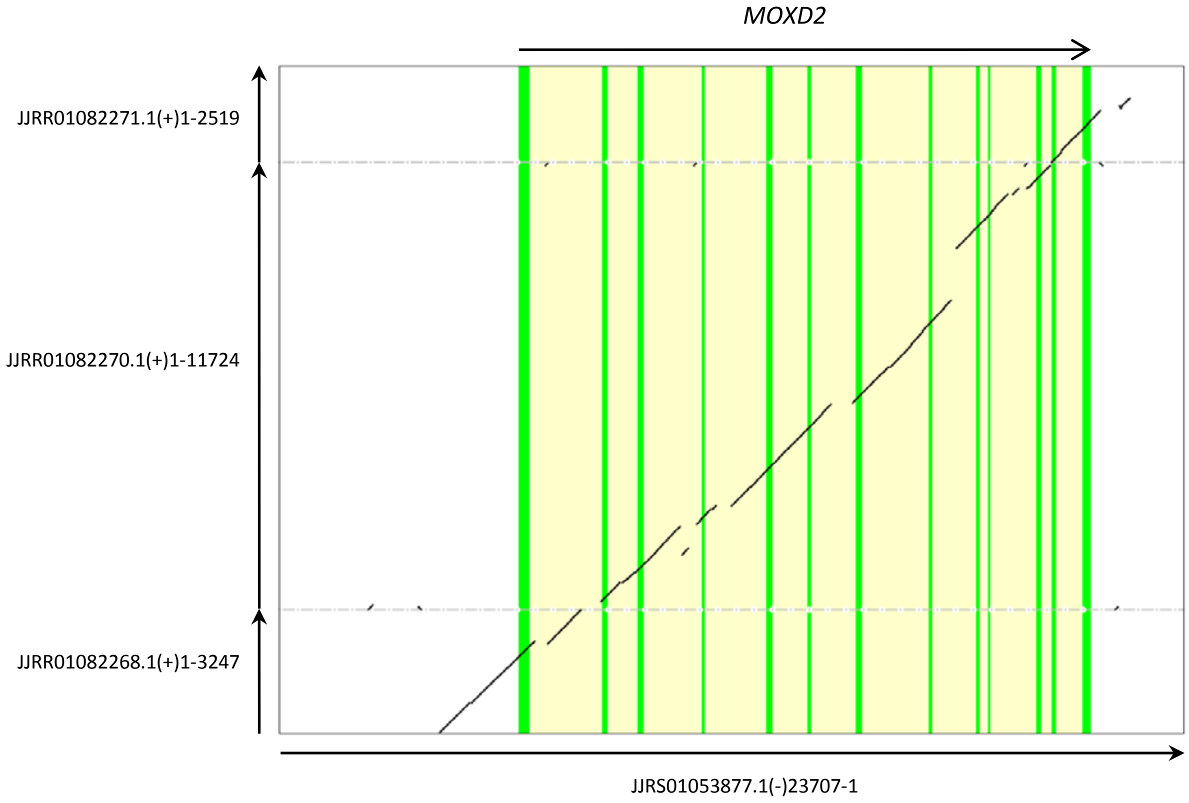

Order: Passeriformes
Family: Acanthisittidae
Scientific name: *Acanthisitta chloris*
Common name: Rifleman

No. 39
Order: Opisthocomiformes
Family: Opisthocomidae
Scientific name: *Opisthocomus hoazin*
Common name: Hoatzin

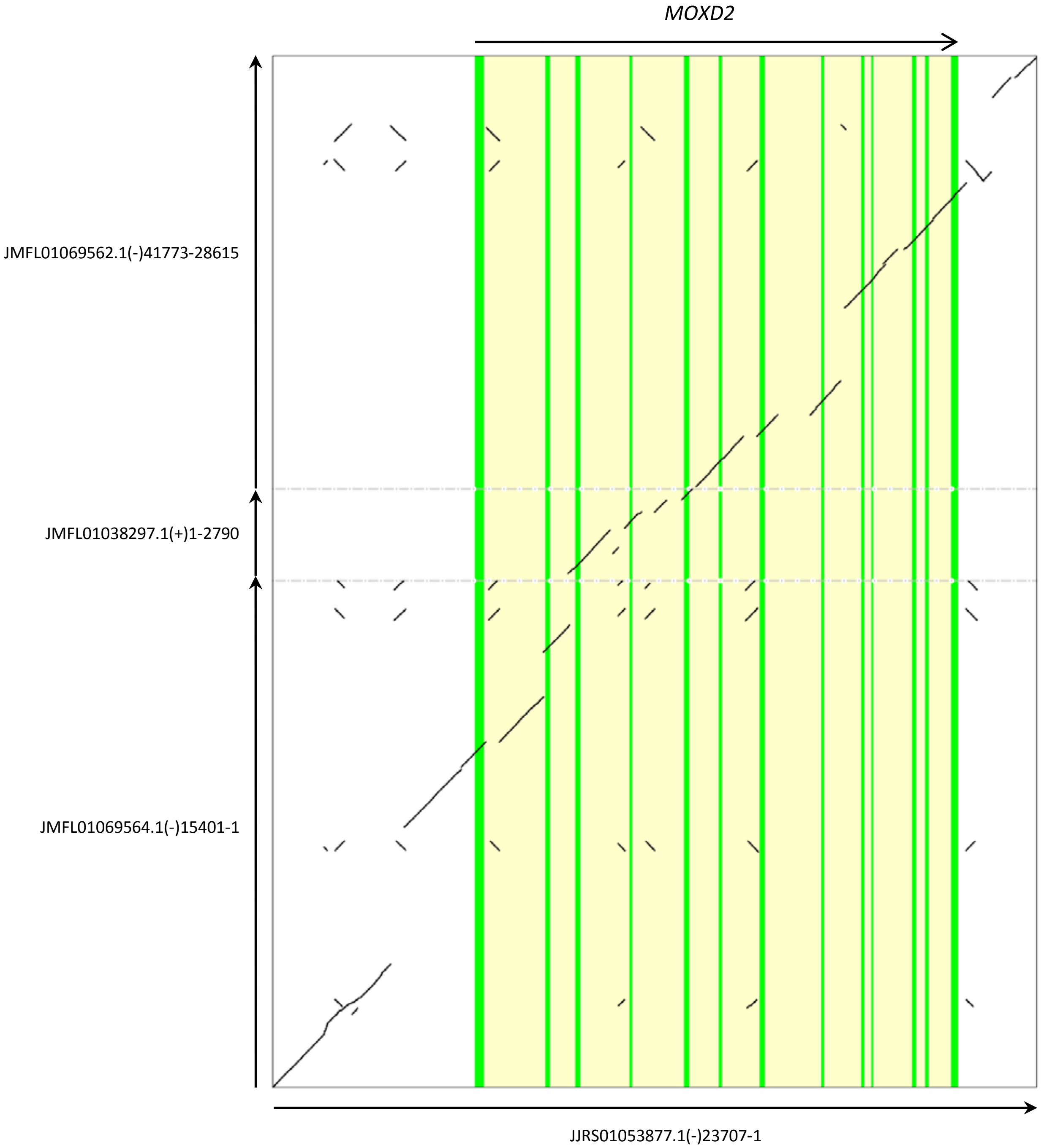

Order: Passeriformes
Family: Acanthisittidae
Scientific name: *Acanthisitta chloris*
Common name: Rifleman

No. 40
Order: Musophagiformes
Family: Musophagidae
Scientific name: *Tauraco erythrolophus*
Common name: Red-crested turaco

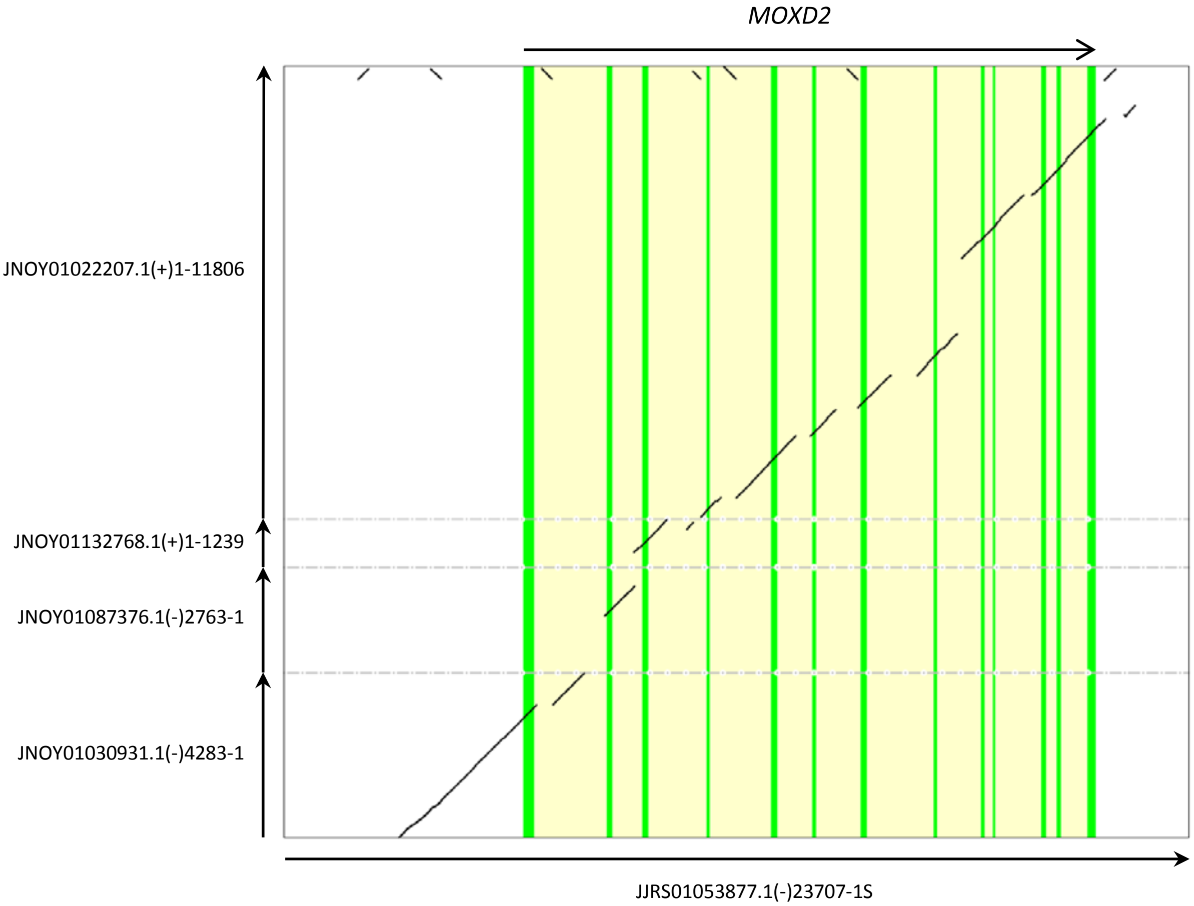

Order: Passeriformes
Family: Acanthisittidae
Scientific name: *Acanthisitta chloris*
Common name: Rifleman

No. 41
Order: Otidiformes
Family: Otididae
Scientific name: *Chlamydotis undulata macqueenii*
Common name: MacQueen's bustard

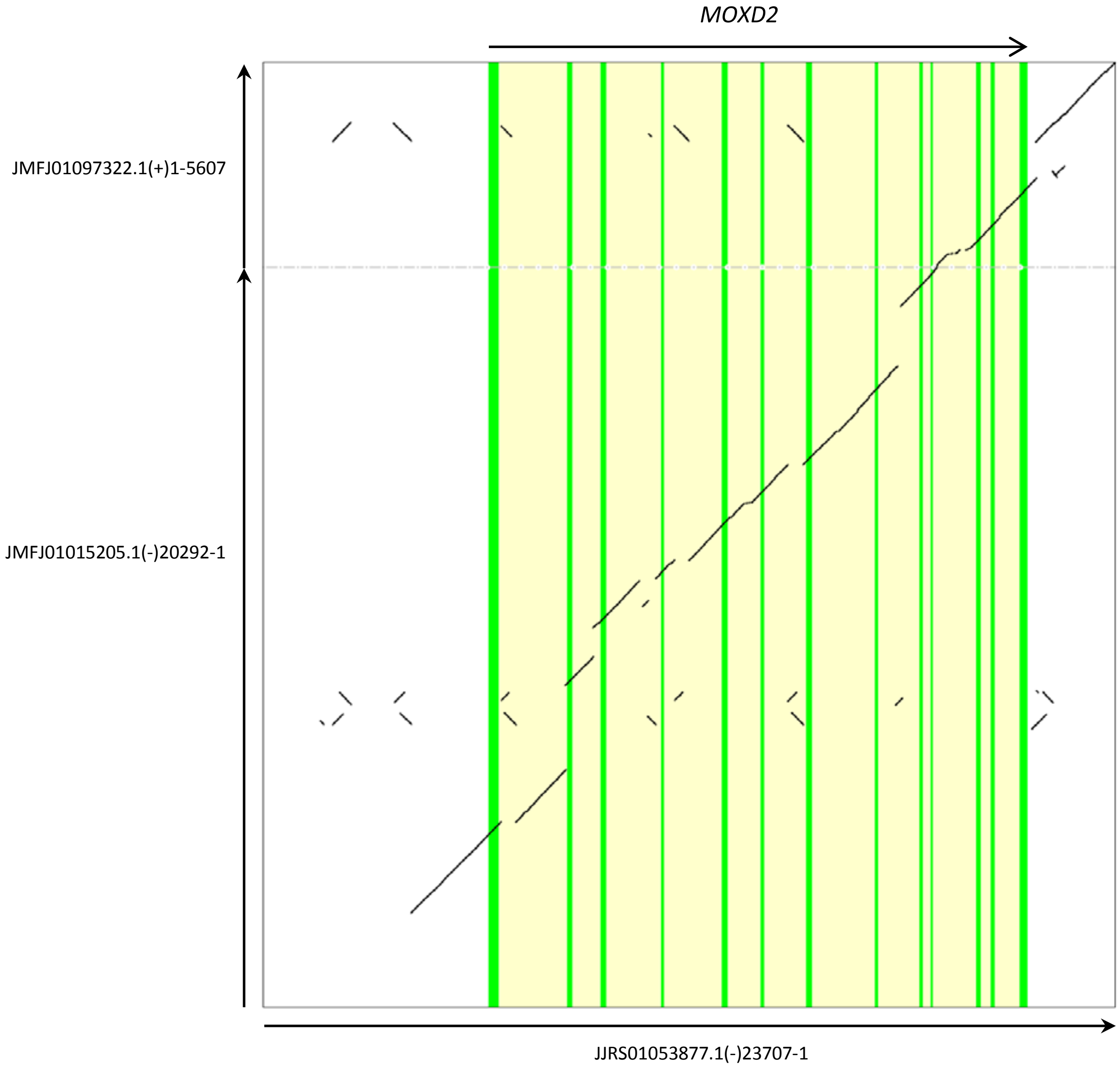

Order: Passeriformes
Family: Acanthisittidae
Scientific name: *Acanthisitta chloris*
Common name: Rifleman

No. 42
Order: Cuculiformes
Family: Cuculidae
Scientific name: *Cuculus canorus*
Common name: Common cuckoo

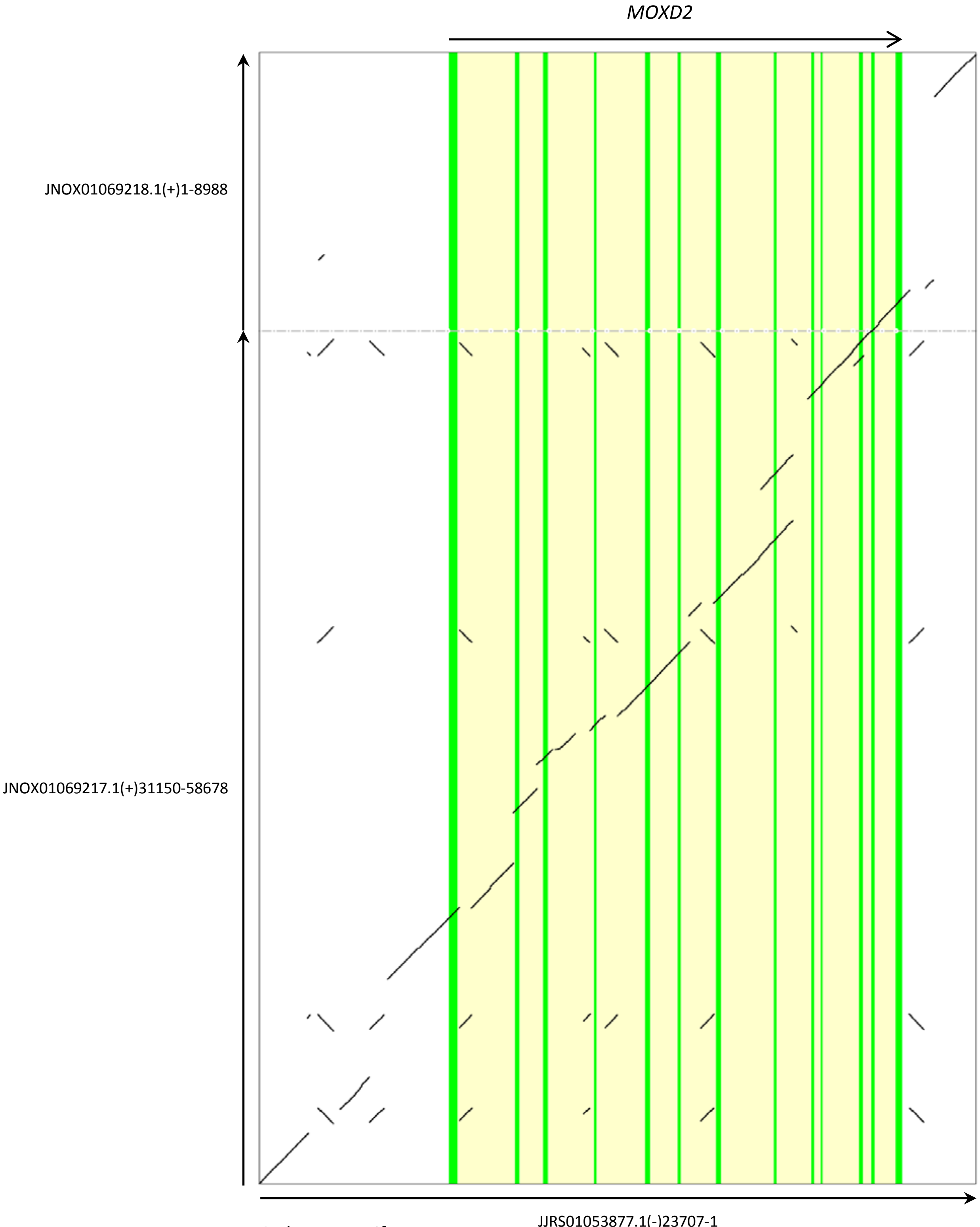

Order: Passeriformes
Family: Acanthisittidae
Scientific name: *Acanthisitta chloris*
Common name: Rifleman

No. 43
Order: Caprimulgiformes
Family: Caprimulgidae
Scientific name: *Caprimulgus carolinensis*
Common name: Chuck-will's-widow

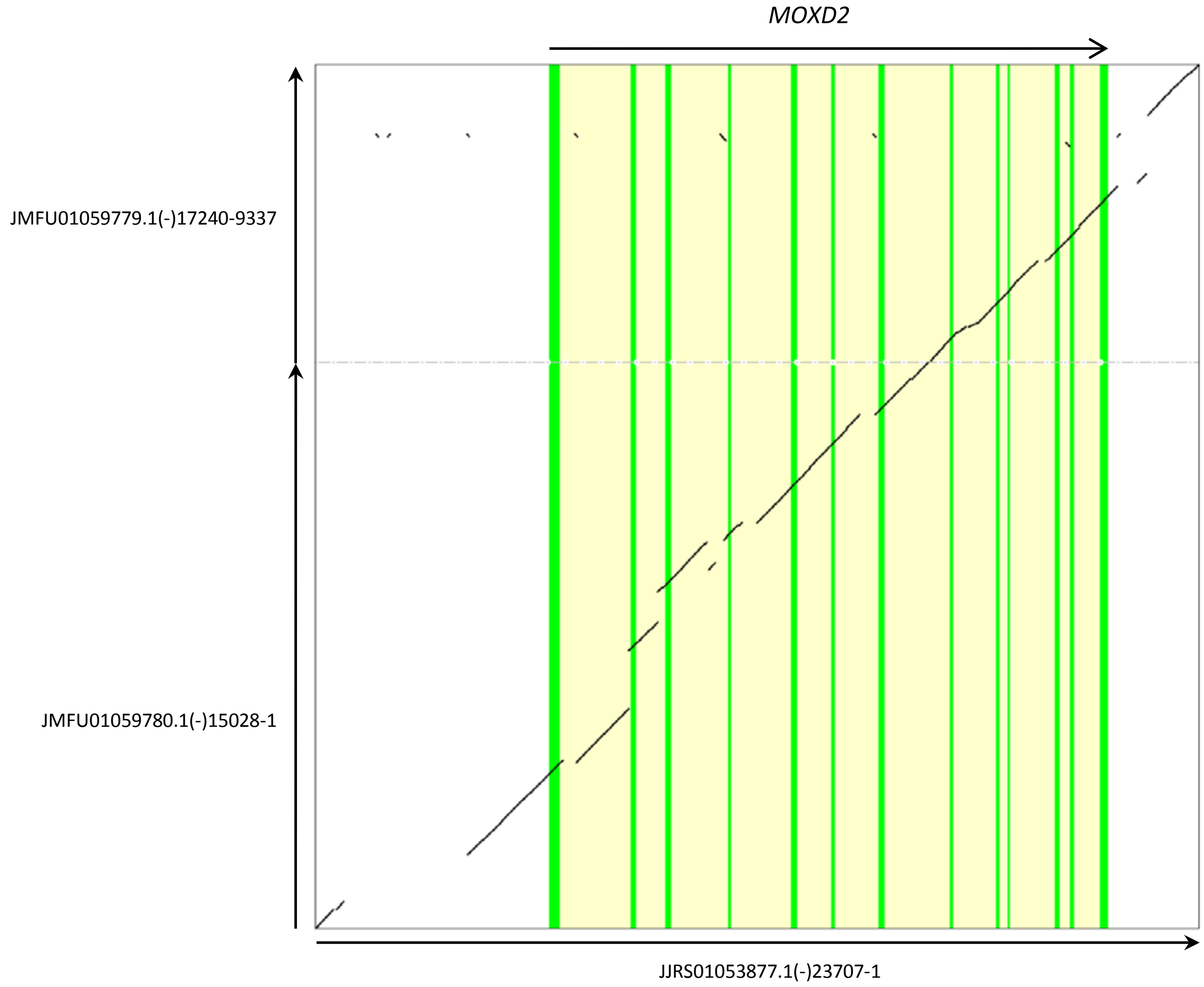

Order: Passeriformes
Family: Acanthisittidae
Scientific name: *Acanthisitta chloris*
Common name: Rifleman

No. 44
Order: Apodiformes
Family: Trochilidae
Scientific name: *Calypte anna*
Common name: Anna's hummingbird

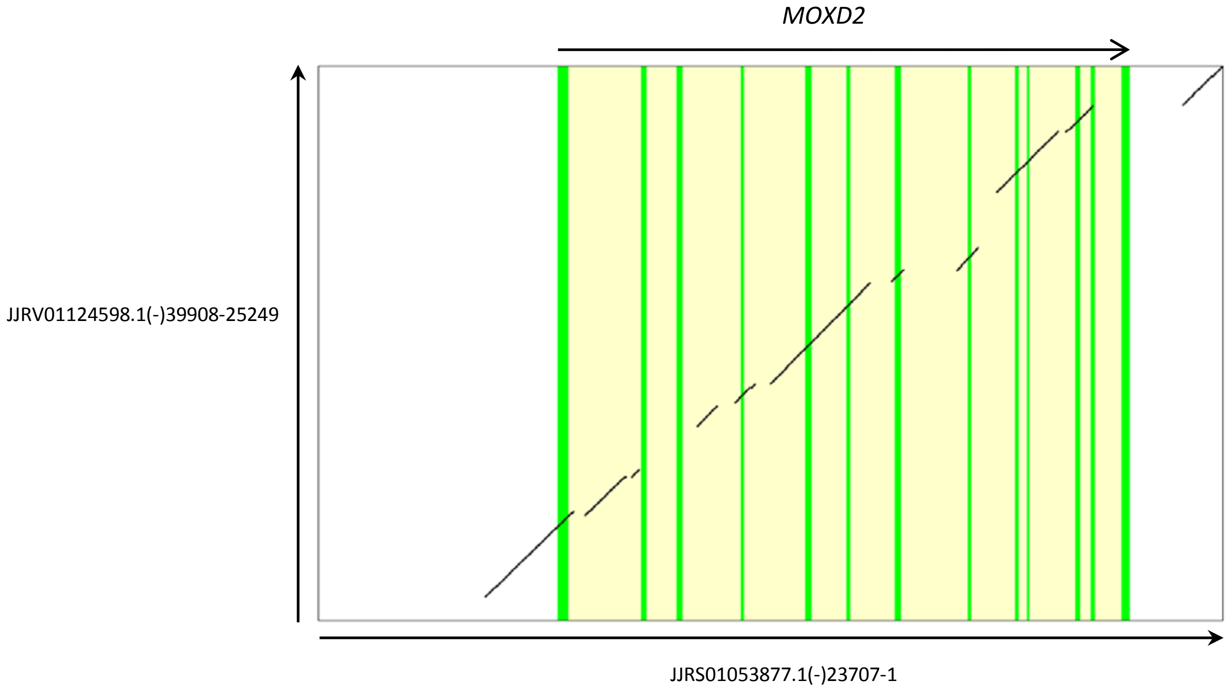

Order: Passeriformes
Family: Acanthisittidae
Scientific name: *Acanthisitta chloris*
Common name: Rifleman

No. 45
Order: Phoenicopteriformes
Family: Phoenicopteridae
Scientific name: *Phoenicopterus ruber ruber*
Common name: Flamingo

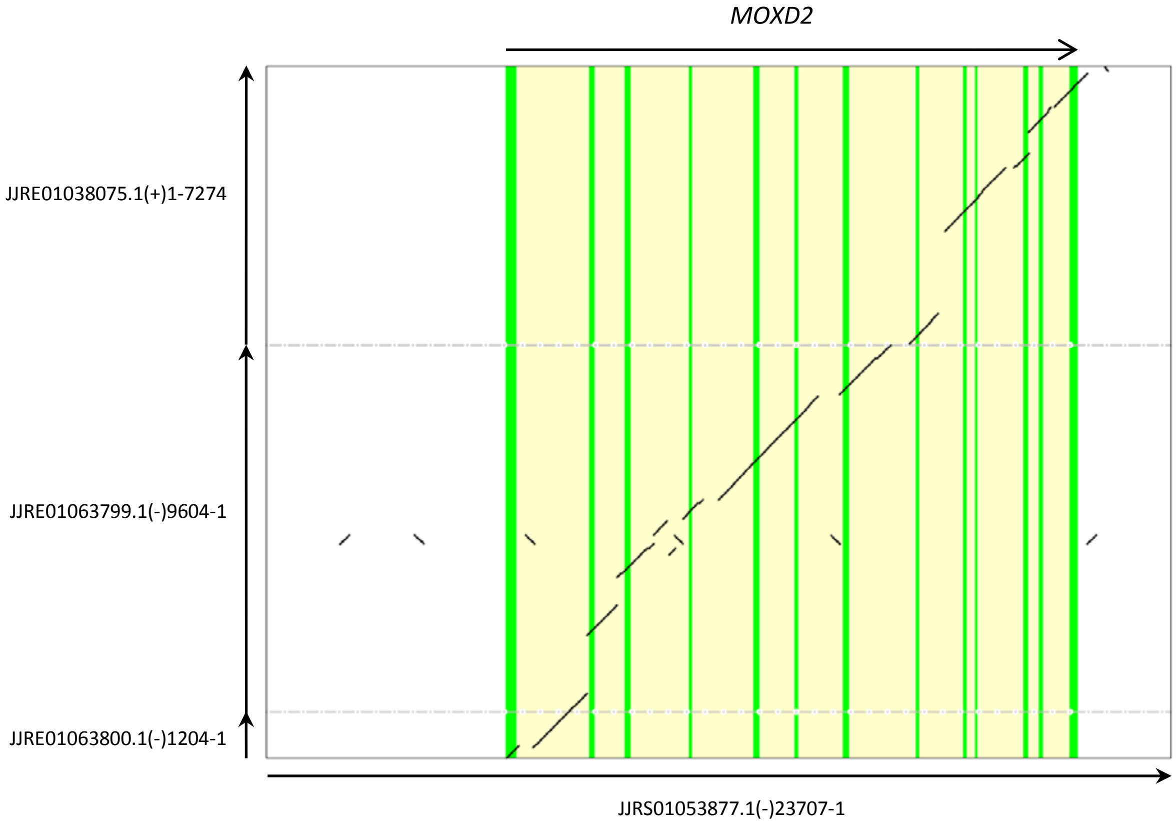

Order: Passeriformes
Family: Acanthisittidae
Scientific name: *Acanthisitta chloris*
Common name: Rifleman

No. 46
Order: Podicipediformes
Family: Podicipedidae
Scientific name: *Podiceps cristatus*
Common name: Great crested grebe

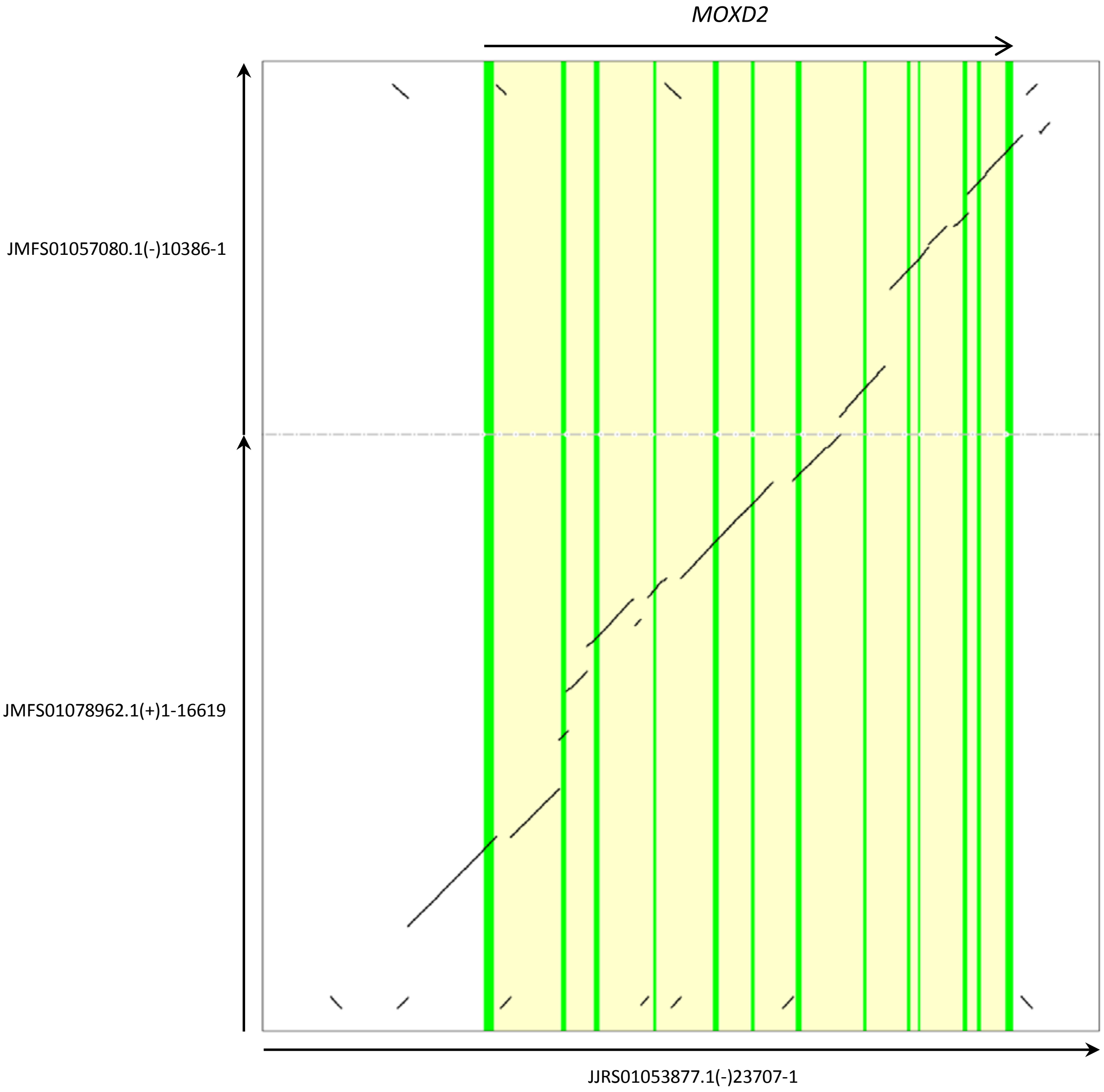

Order: Passeriformes
Family: Acanthisittidae
Scientific name: *Acanthisitta chloris*
Common name: Rifleman

No. 47
Order: Columbiformes
Family: Columbidae
Scientific name: *Columba livia*
Common name: Rock pigeon

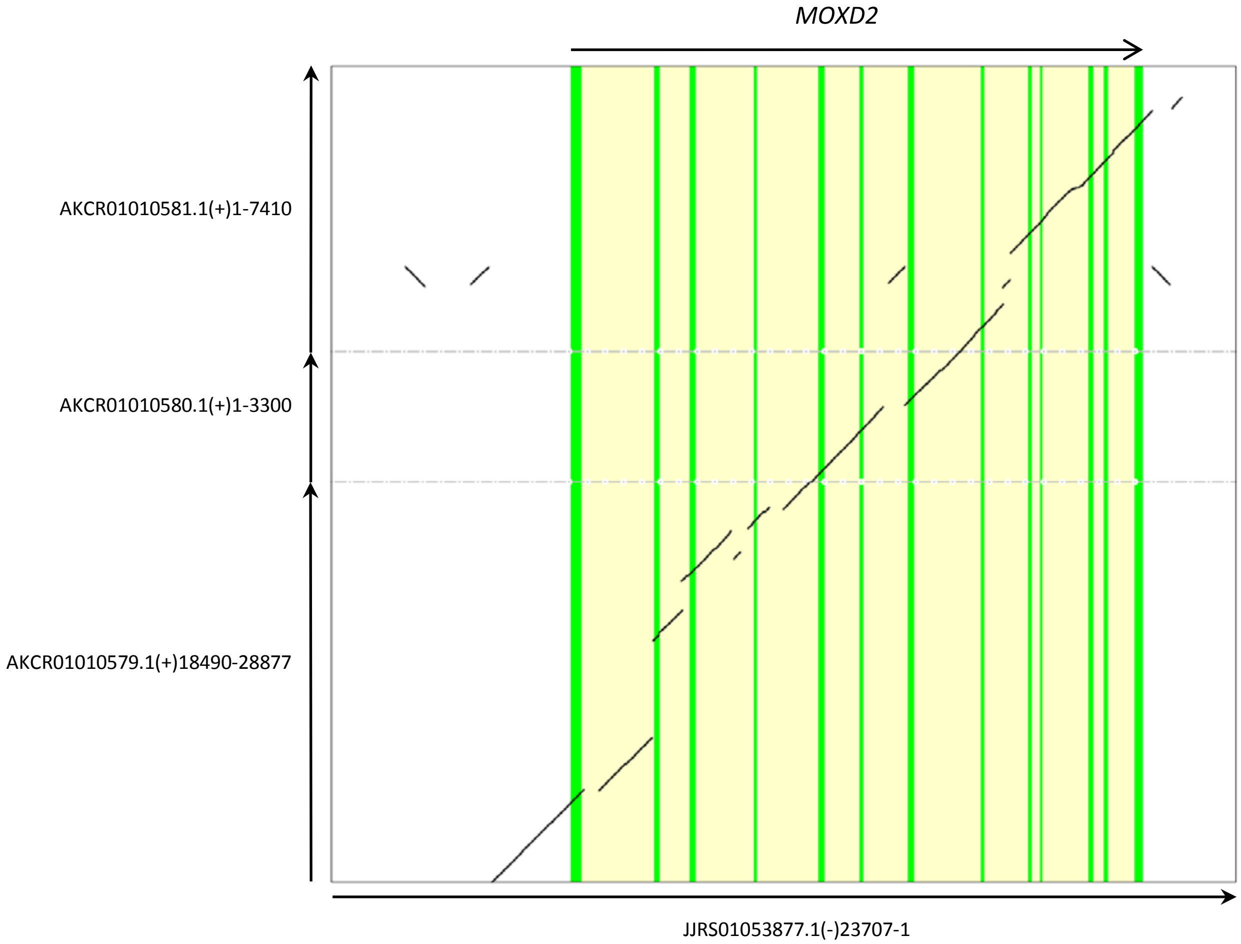

Order: Passeriformes
Family: Acanthisittidae
Scientific name: *Acanthisitta chloris*
Common name: Rifleman

No. 48
Order: Pteroclidiformes
Family: Pteroclididae
Scientific name: *Pterocles gutturalis*
Common name: Yellow-throated sandgrouse

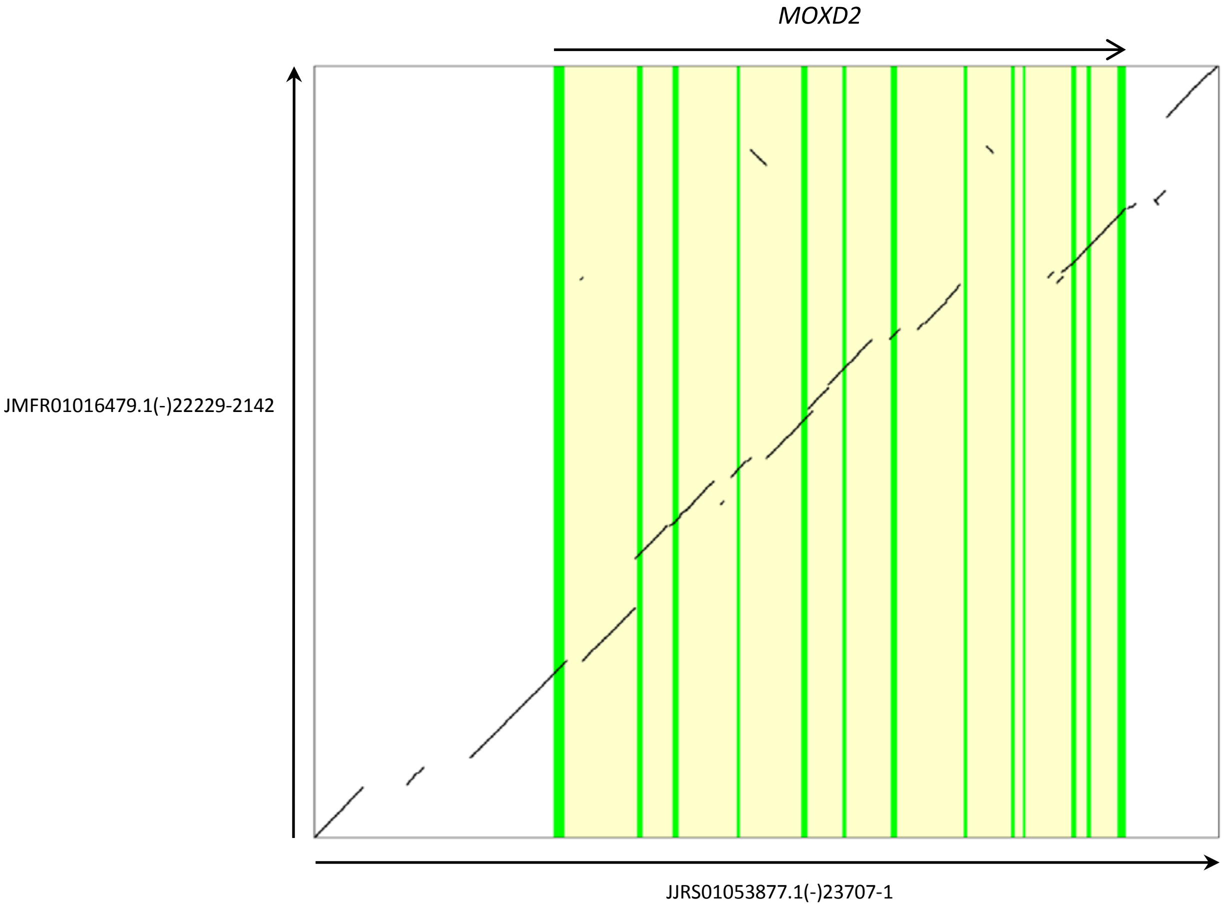

Order: Passeriformes
Family: Acanthisittidae
Scientific name: *Acanthisitta chloris*
Common name: Rifleman

No. 49
Order: Mesitornithiformes
Family: Mesitornithidae
Scientific name: *Mesitornis unicolor*
Common name: Brown mesite

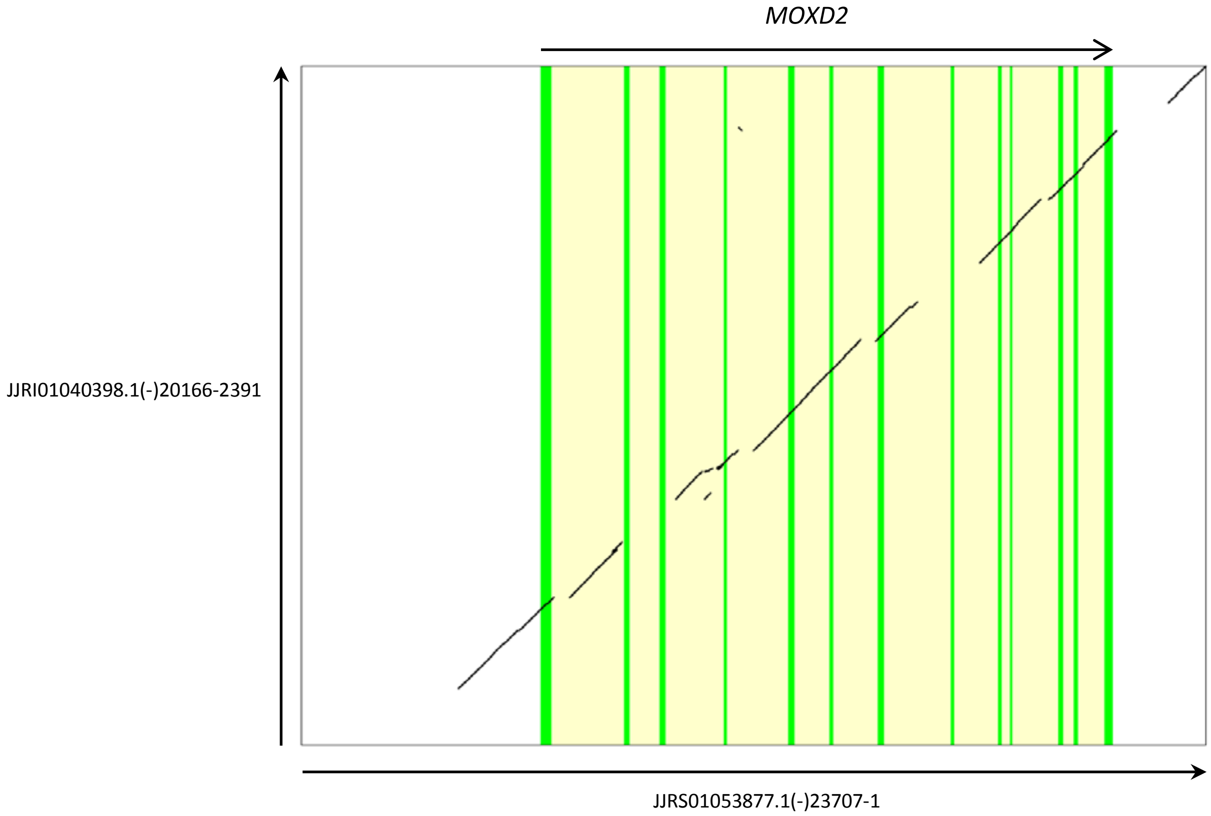

Order: Passeriformes
Family: Acanthisittidae
Scientific name: *Acanthisitta chloris*
Common name: Rifleman

No. 50
Order: Anseriformes
Family: Anatidae
Scientific name: *Anas platyrhynchos*
Common name: Mallard

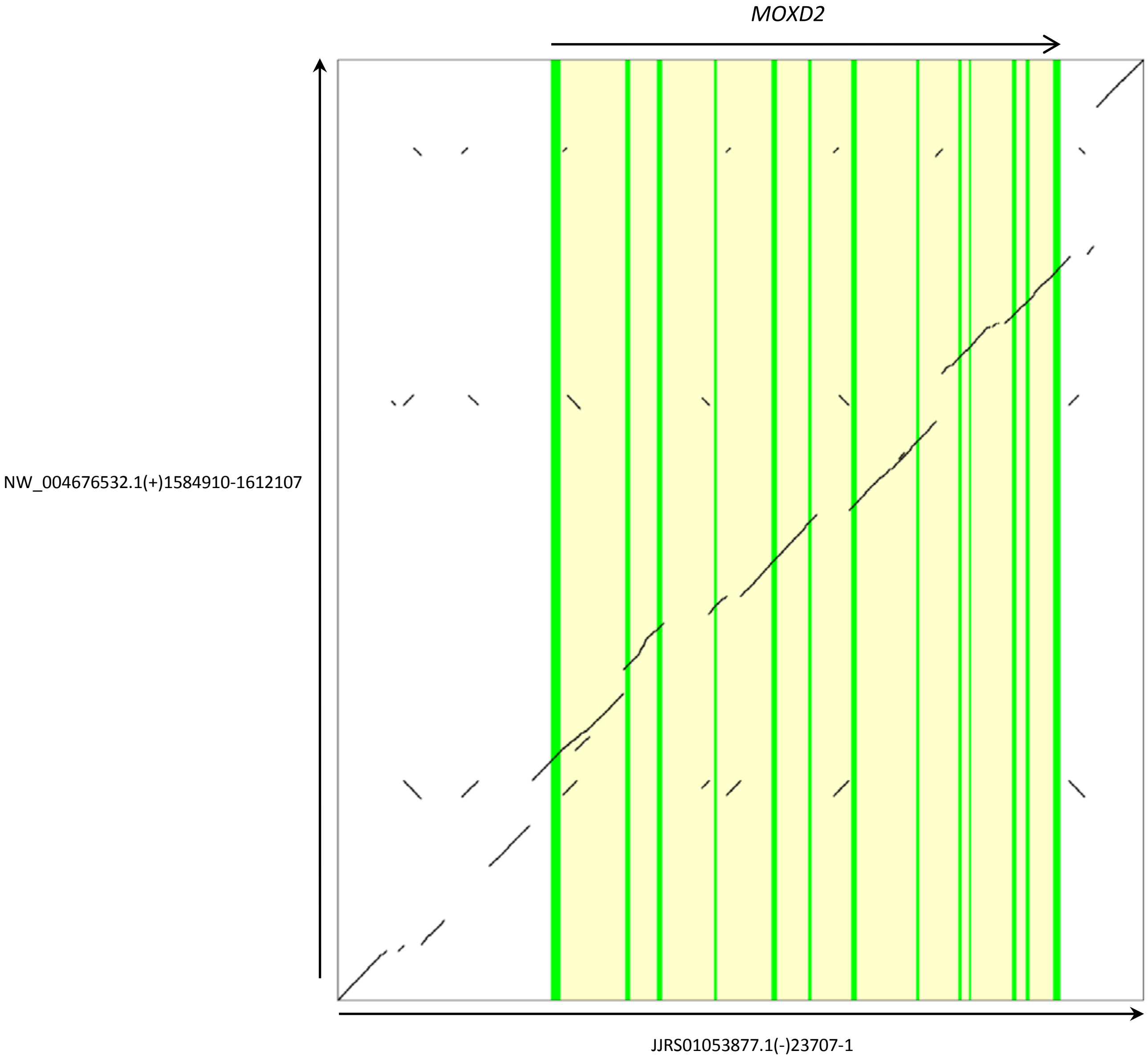

Order: Passeriformes
Family: Acanthisittidae
Scientific name: *Acanthisitta chloris*
Common name: Rifleman

No. 51
Order: Galliformes
Family: Odontophoridae
Scientific name: *Colinus virginianus*
Common name: Northern bobwhite

No *MOXD2* sequence

No. 52
Order: Galliformes
Family: Phasianidae
Scientific name: *Coturnix japonica*
Common name: Japanese quail

No *MOXD2* sequence

No. 53
Order: Galliformes
Family: Phasianidae
Scientific name: *Gallus gallus*
Common name: Chicken

No *MOXD2* sequence

No. 54
Order: Galliformes
Family: Phasianidae
Scientific name: *Meleagris gallopavo*
Common name: Wild turkey

No *MOXD2* sequence

No. 55
Order: Galliformes
Family: Phasianidae
Scientific name: *Tetrao (Lyrurus) tetrix tetrix*
Common name: Black grouse

No *MOXD2* sequence

No. 56
Order: Tinamiformes
Family: Tinamidae
Scientific name: *Tinamus guttatus*
Common name: White-throated tinamou

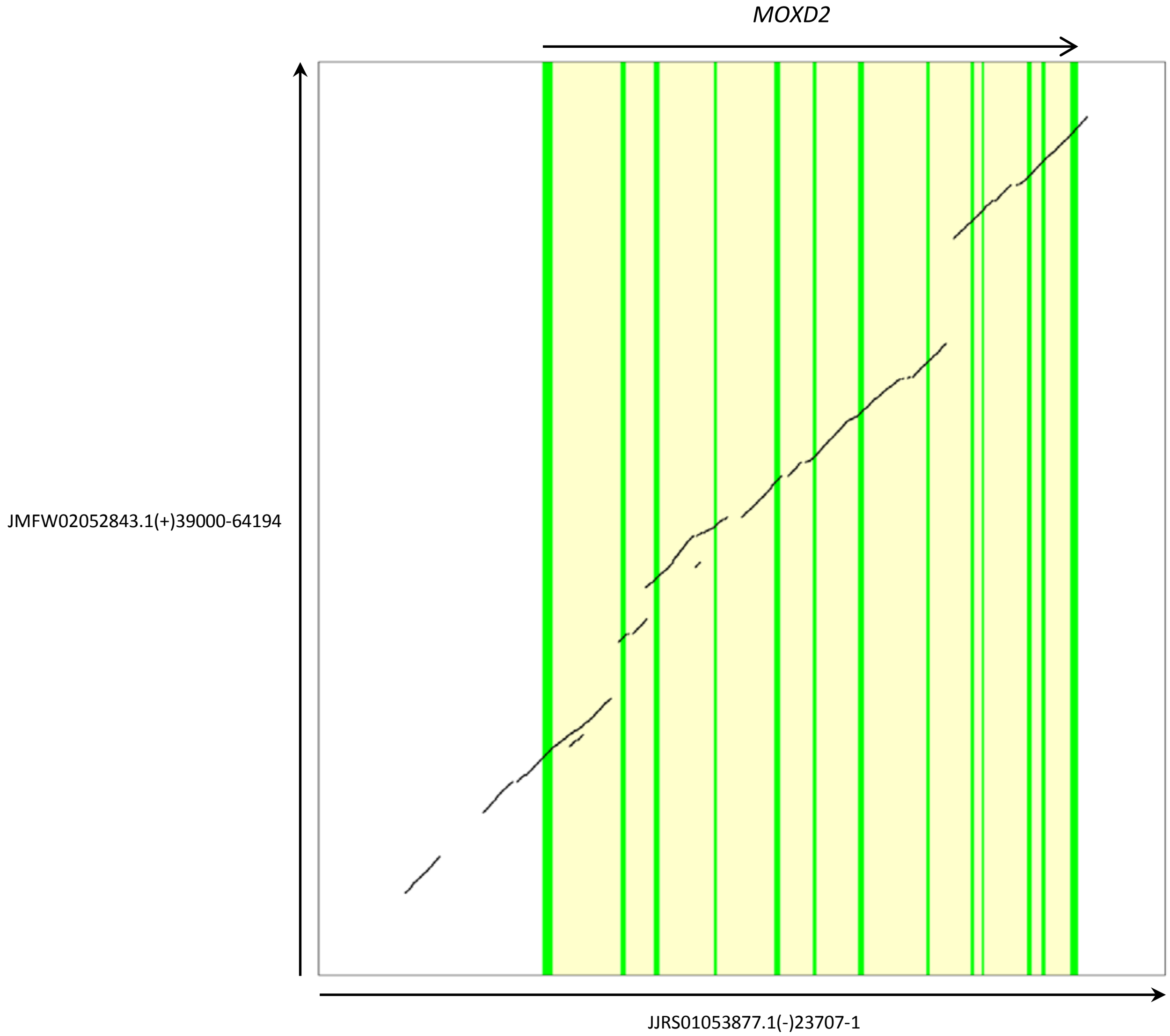

Order: Passeriformes
Family: Acanthisittidae
Scientific name: *Acanthisitta chloris*
Common name: Rifleman

No. 57
Order: Struthioniformes
Family: Struthionidae
Scientific name: *Struthio camelus australis*
Common name: African ostrich

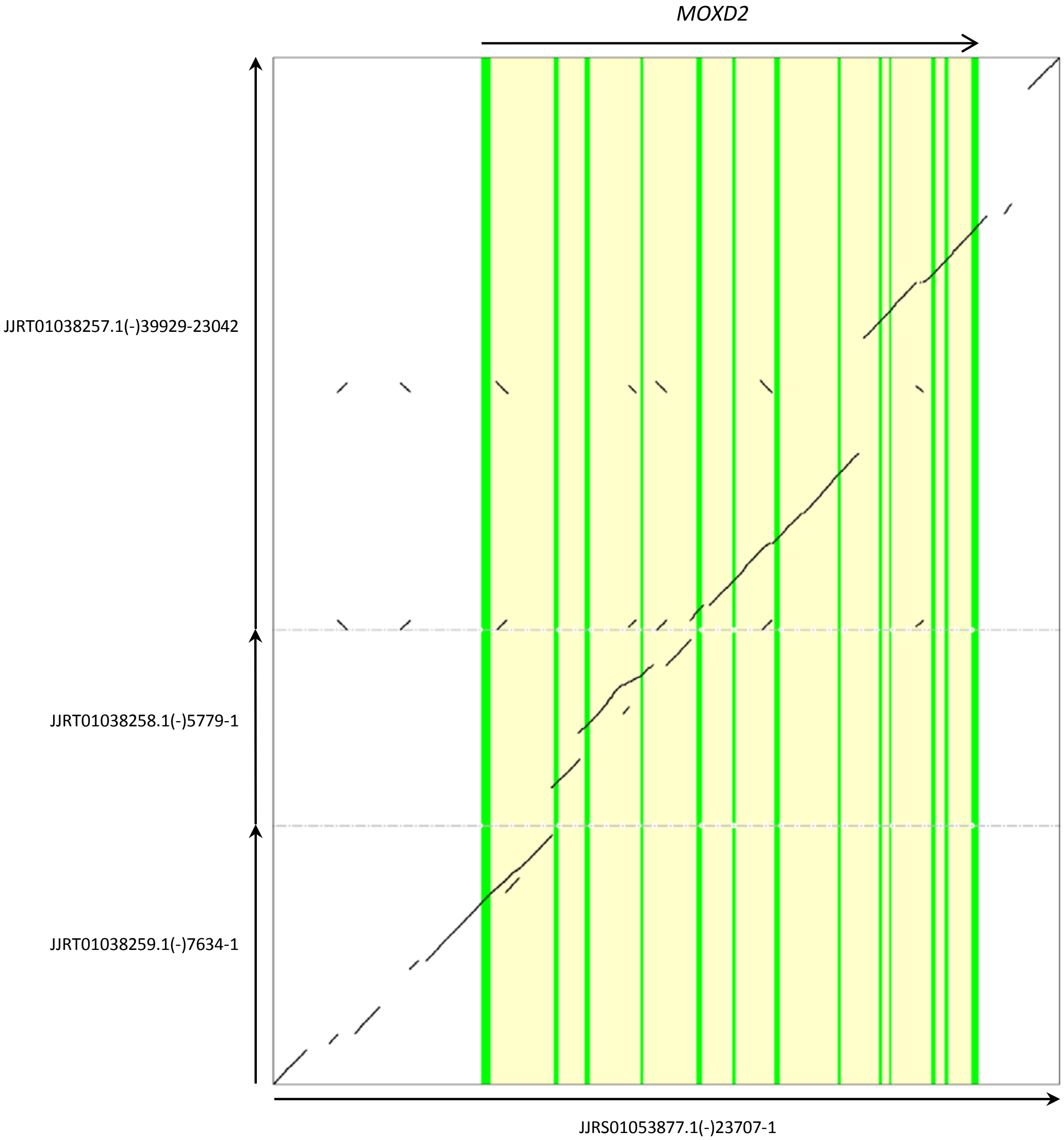

Order: Passeriformes
Family: Acanthisittidae
Scientific name: *Acanthisitta chloris*
Common name: Rifleman
