# Supplementary material for: MOXD2, a Gene Possibly Associated with Olfaction, Is Frequently Inactivated in Birds
Source: PLoS One. 2016 Apr 13;11(4):e0152431. doi: 10.1371/journal.pone.0152431 (PMC4830563; doi:10.1371/journal.pone.0152431)
Supplement: S6 Fig — (PDF) [file pone.0152431.s006.pdf]

S6 Fig. Dotplot comparison of the downy woodpecker and Northern carmine bee-eater *MOXD2* loci

No. 18
Order: Piciformes
Family: Picidae
Scientific name: *Picoides pubescens*
Common name: Downy woodpecker

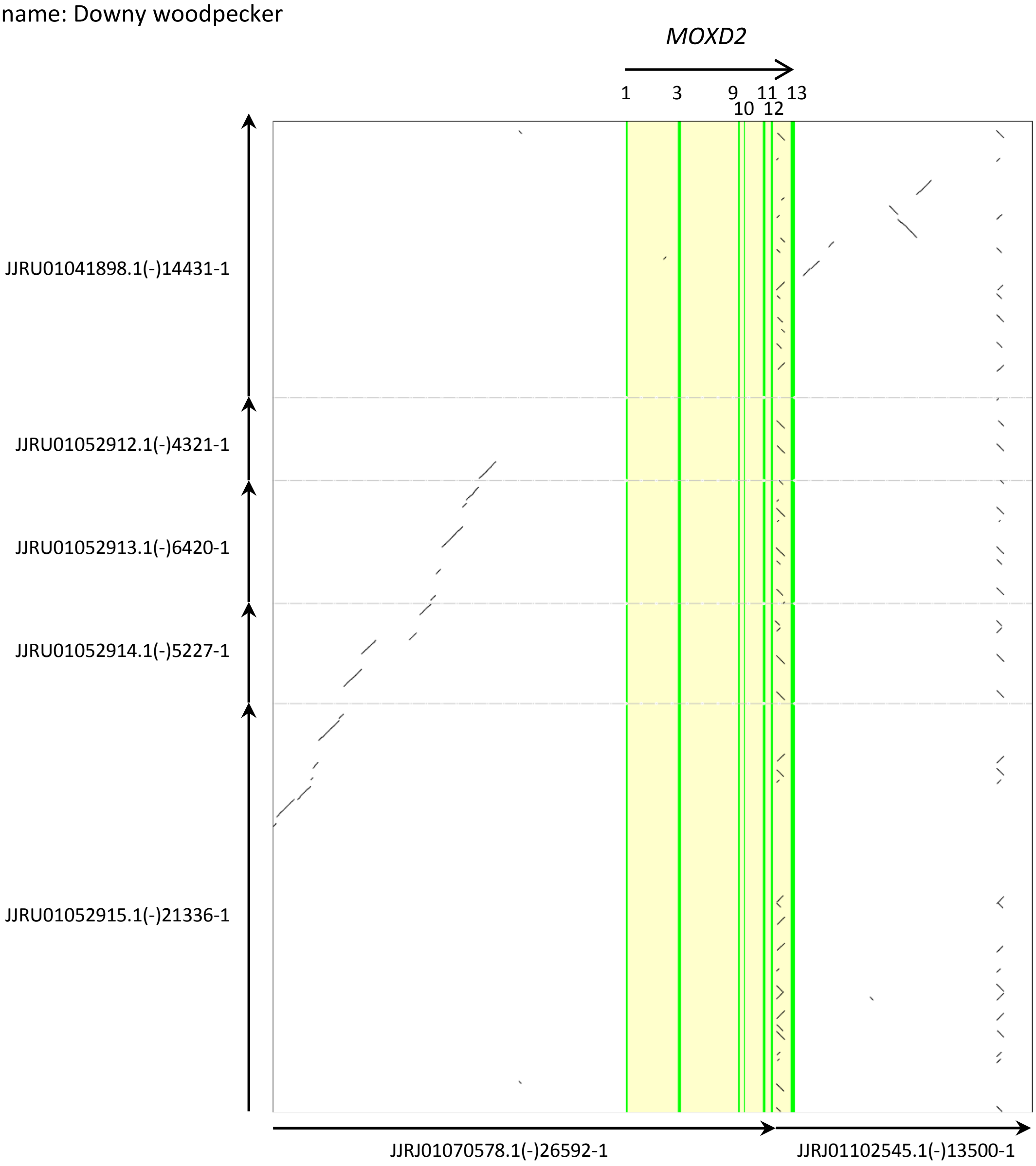

No. 19
Order: Coraciiformes
Family: Meropidae
Scientific name: *Merops nubicus*
Common name: Northern carmine bee-eater
