# Supplementary material for: MOXD2, a Gene Possibly Associated with Olfaction, Is Frequently Inactivated in Birds
Source: PLoS One. 2016 Apr 13;11(4):e0152431. doi: 10.1371/journal.pone.0152431 (PMC4830563; doi:10.1371/journal.pone.0152431)
Supplement: S7 Fig — (PDF) [file pone.0152431.s007.pdf]

S7 Fig. Dotplot comparison of the chicken and mallard *MOXD2* loci

No. 53
Order: Galliformes
Family: Phasianidae
Scientific name: *Gallus gallus*
Common name: Chicken

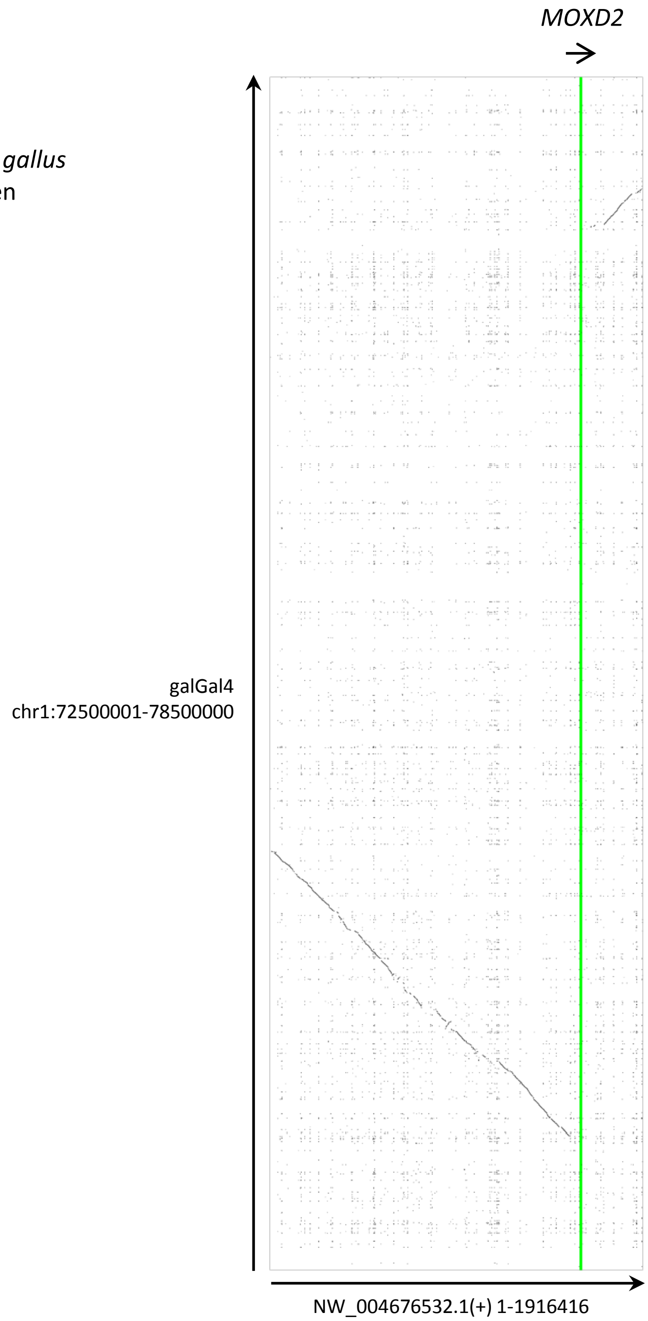

No. 50
Order: Anseriformes
Family: Anatidae
Scientific name: *Anas platyrhynchos*
Common name: Mallard
